# Hearing in Adults: A Digital Reprint of the Main Report From the MRC National Study of Hearing

**DOI:** 10.1177/2331216519887614

**Published:** 2019-12-20

**Authors:** Michael A. Akeroyd, George G. Browning, Adrian C. Davis, Mark P. Haggard

**Affiliations:** 1 Hearing Sciences, Division of Clinical Neurosciences, School of Medicine, University of Nottingham, UK; 2 Hearing Sciences (Scottish Section), Division of Clinical Neurosciences, School of Medicine, University of Nottingham, Glasgow, UK; 3 Imperial College Healthcare NHS Trust, Charing Cross Hospital, London, UK; 4 AD Cave Solutions Limited, London, UK; 5 Department of Psychology, University of Cambridge, UK

**Keywords:** audiology, prevalence, hearing loss

## Abstract

The 1011-page book, *Hearing in Adults*, published in 1995, contains the fullest report of the United Kingdom’s Medical Research Council National Study of Hearing. It was designed to determine the prevalence and distribution in Great Britain of audiometrically measured hearing loss as a function of age, gender, occupation, and noise exposure. The study’s size, quality, and breadth made it unique when it was done in the 1980s. These qualities remain, and its data are still the primary U.K. source for the prevalence of auditory problems. However, only 550 copies were printed, and the book is essentially unobtainable today. We describe here a fully searchable, open-access, digital (PDF) “reprinting” of *Hearing in Adults*, summarizing the study’s design and the book’s contents, together with a brief commentary in the light of subsequent developments.

## Introduction

The Medical Research Council’s (MRC) “National Study of Hearing” (NSH) was a group of major epidemiological studies of the prevalence and distribution of hearing problems of adults in England, Scotland, and Wales. It was conducted by the former MRC Institute of Hearing Research (MRC IHR) in the 1980s. The NSH gave the United Kingdom its first stratified and precise quantification of degrees of severity of hearing loss in a designed study and related these to demographic and pathological determinants. Its scale and type of measurement have never been repeated in the United Kingdom; its data and analysis framework are still used to determine prevalences and derive population numbers (e.g., Akeroyd, Foreman, & Holman, 2014; GBD 2016 Disease and Injury Incidence and Prevalence Collaborators, 2016; Health Survey for England, 2014). The current international standard for the statistical distribution of hearing thresholds by age and gender is in part based on its data (International Organization for Standardization [ISO] 7029: 2017). Overall, its data remain key to the case that hearing impairment matters to many people and justifies a major place within public health priorities (e.g., Action on Hearing Loss, 2015; NHS England & Department of Health, 2015).

The NSH’s design, data-acquisition methods and its key results were published in a small number of journal papers (e.g., Browning & Gatehouse, 1992; Davis, 1989; Lutman & Spencer, 1991; MRC IHR, 1981). However, the full descriptive tables from the data were only published in a 1011-page book, *Hearing in Adults* (hereafter *HiA*). This book (Davis, 1995) had a limited print run, and it is not practicably obtainable today. This article introduces and accompanies a fully searchable, open-access, digital (PDF) “reprinting” of *HiA*. To enhance access to this unique material, we give here a summary of the study’s design, the book’s contents, and a commentary in the light of subsequent developments.

## The Design of the NSH

The NSH was designed to determine the prevalence and characteristics of both measured hearing impairment and self-reported hearing disability in adults (18–80 years) as a function of severity, age, gender, occupational group, and occupational noise exposure (Davis, 1989; MRC IHR, 1981; see Online *HiA* Chapters 1 and 2). These goals were implemented strategically through six key design features. Given the number of main variables supported and the requirement for narrow confidence intervals across the wide age range, a large sample size was needed. The six design features were as follows:

1. A structured two-tier design to reconcile population reach with clinical/laboratory precision of measurement. The first tier was a postal survey; the second tier was auditory and clinical assessment at four specially equipped hospital-based research clinics with qualified otorhinolaryngolical (ORL) and audiological staff.
2. A double basis of accessing the population, via two sampling frames: individuals from the electoral register and then from households, with cross-checking of the two sets of estimates.
3. Repeated postal reminders, then domiciliary visits to ensure remarkably high response rates and hence reduce attendance bias.
4. Calibration of equipment and detailed analyses of calibration results by center, tester, and frequency to determine which effects were large enough to be worth adjusting for.
5. Initial stratification of first tier responses for efficient gearing of clinical testing, then poststratification for population projections.
6. A broad range of clinical and demographic data to be acquired, including a rigorous structured protocol for eliciting and calculating lifetime noise immission.

It is also useful to appreciate what the NSH was *not* designed to do, as no practical study can measure everything of interest. It was not intended to ascertain the numbers of severely, profoundly, or totally hearing-impaired people, nor of the prelingually deaf, because for the very low prevalences concerned it would not have provided an efficient approach. It was not directly concerned with the coverage or targeting of audiology services in Great Britain (but see Haggard, 1993; Haggard, Gatehouse, & Davis, 1981 for a discussion of those issues in light of NSH findings). Also, some of the data collected in the NSH were not reported in *HiA*, including blood samples, frequency resolution, and speech perception (see Coles, 1984; Lutman, 1990; Lutman, Gatehouse, & Worthington, 1991). Tinnitus was measured, though it is only reported briefly in *HiA* (Online Chapter 9; see Coles, 1984; Davis, 1989 for more details.)

The main NSH study had two tiers with a random sample that was stratified by age and self-report, weighted to reflect the population, and then projected back to the population by the double-sample method. Data were collected in three phases between 1980 and 1986, with the analyses subsequently collapsed over phases using fixed questions that were common between phases and a rigorous statistical procedure. It was conducted in and around four British cities: Cardiff, Glasgow, Nottingham, and Southampton. There were no important differences across phases or cities. The study design was optimized and powered to give a 95% confidence interval of about ±1% for prevalences in the region of 15% to 20% and ±0.5% for prevalences in the region of 3% to 5%. Throughout every stage of the study, considerable attention and resources were committed to investigate any biases (e.g., by domiciliary visits to document loss to follow-up; repeat testing of some audiometric thresholds) and to maintain audiometric calibration and fixed procedures.

In the first tier, a postal questionnaire on hearing, tinnitus, and demographics was sent to 48,313 adults selected at random from the electoral registers. The overall response rate was 80%. The questionnaire (reported in *HiA* Online Appendix 1) was informed by related earlier work by Schein and Delk (1974) in the United States, Noble (1978) in the United Kingdom and Australia, as well as prior U.K. studies (e.g., Hinchcliffe, 1961) and the principal investigators’ own experience. It underwent continued evolution and improvement between phases.

In the second tier, a reduced sample drawn from those who responded to Tier 1 was invited to one of the four clinics of MRC IHR for an audiological assessment, a detailed questionnaire (see *HiA* Online Appendix 4), otological examination and clinical interview. The sample was initially stratified by age, self-reported hearing, and whether they possessed a hearing aid (see later for all definitions). For efficiency and precise estimation of means and variances, the stratum with self-reported hearing disability was sampled more densely than those with no reported hearing problem. For example, all those who reported in Tier 1 that they used a hearing aid were contacted to take part in Tier 2, whereas only about 1 in 25 people who reported no hearing problem whatsoever were contacted. The final Tier B sample contained 2,578 individuals with complete audiograms at the usual octave-spaced frequencies up to 8 kHz for each ear but additionally including 3, 6, and 12 kHz. There were 2,208 otoscopic examinations. The data were poststratified for calculating prevalences, according to location, age band, gender, and self-reported hearing.

A complementary two-stage study was also conducted on 6,650 addresses to check the national representativeness of the main study sample. The addresses were chosen from the U.K. Postal Address File, which was the most complete postcode database in the United Kingdom at the time, also being stratified by health region and by the CACI Acorn housing type (https://acorn.caci.co.uk/what-is-acorn). This was a widely used surrogate residential marker for socioeconomic status. This study also had a two-tier design, with a postal questionnaire followed by a home visit for abbreviated clinical assessment.

The data were stratified according to three binary classifications. *Difficulty in noise* was taken as a “yes”/“no” answer to the Tier 1 question “Do you find it very difficult to follow a conversation if there is a background noise, eg TV, radio, children playing?” (Davis, 1989, p. 914). *Tinnitus* was defined as having prolonged sustained tinnitus, lasting more than 5 min: “Nowadays, do you get noises in your head or ears?” “Do these noises usually last longer than 5 minutes” (Davis, 1989, p. 914). *Hearing-aid possession* was “yes” to “Have you ever had a hearing aid?” Further definitions were as follows: for *audiometry*, standard across-frequency averages were calculated on audiograms (*HiA*, p. 44), with a pragmatic boundary for conductive hearing loss was taken as an air-bone gap ≥15 dB when averaged over 0.5, 1, and 2 kHz (*HiA*, p. 691). *Occupational groups* were based on head-of-household information collected at the clinical interview rather than the self-report data. They were coded in accordance with the recommended Registrar General’s Classification of Occupations at the time (Office of Population Censuses and Surveys, 1980), namely, professional (I), managerial (II), semiskilled/clerical (IIINM), skilled (IIIM), semiskilled (IV), and unskilled (V). These were also aggregated into nonmanual (I, II, and IIINM) and Manual (IIIM, IV, and V; *HiA*, p. 44). *Noise emission* was defined in terms of the equivalent continuous sound level (*L*_eq_) for a 50-year working lifetime; *no noise* was ≤80 dB(A). The 80-dB cutoff was used to identify individuals with no noise exposure in the screened data. Higher bands were also defined, but these were only used in associated papers (*HiA*, p5, Browning & Davis, 2019; Davis, 1989; Lutman & Spencer, 1991). These definitions were all current when the work was done in the 1980s.

Odds ratios for hearing impairment at cutoffs of 25 dB HL, 35 dB HL, and so on, were calculated from a logistic regression model including age band, gender, occupationally based socioeconomic status group, and noise exposure. For these, the hearing losses were calculated for the better hearing ear across the typical four-frequency average (0.5, 1, 2, and 4 kHz). This led to another major product of the study: a parsimonious four-term demographic model for predicting the probability of a hearing loss (see Table 5 in Davis, 1989). We found that logistic regression models for the probability of a hearing impairment at 25 dB HL or greater (in the better ear) and at 45 dB HL or greater gave a good fit with four highly significant factors. Age gave by far the highest odds ratio, reaching 95 for an age of 71 to 80 years; noise emission, social–economic class, and sex gave more modest odds ratios, reaching 2.3.

## The Publication of the NSH

In addition to the main publication, *HiA*, the measurement methods of the NSH and the main prevalence data on hearing impairment and reported hearing disability were reported in Davis (1983, 1989). Further articles considered the profile of hearing loss (Davis, 1991), the distribution of hearing loss (Bowater, Copas, Machado, & Davis, 1996; Lutman & Davis, 1994), effects of blood viscosity (Browning, Gatehouse, & Lowe, 1986), noise (Davis & Thornton, 1990; Lutman & Spencer, 1991), tinnitus (Coles, 1984; Coles, Davis, & Smith, 1990), middle ear disease (Browning & Gatehouse, 1992), self-reported and performance disability (Lutman, Brown, & Coles, 1987), and hearing aid use (Haggard & Gatehouse, 1993). The data have also been used in studies of diabetic retinopathy (Miller, Beck, Davis, Jones, & Thomas, 1983), blood lipid levels (Jones & Davis, 2000), hyperlipidemia (Jones & Davis, 1999, 2002), and inflammation (Verschuur, Agyemang-Prempeh, & Newman, 2014). In a separate monograph, Haggard (1993) summarized the major policy messages from the NSH for the prevalence and severity distribution of adult hearing impairment and aetiology.

## The Digital Version of *HiA*

*HiA* was published in 1995 by Whurr. The rights were later acquired by Wiley when they bought out Whurr and then transferred back to one of us (Davis) in October 2017. The British Library’s (BL) digitization department undertook the task of scanning *HiA*. Photographic reproduction was briefly considered, but pilot work showed that scanning was preferred as generally delivering more consistently high quality. The digital version was thus created by scanning each page of a copy of *HiA* and then converting to PDF. Due to an oversight on our part, the opening material (the original Foreword, Preface, and Epigraphs) was not scanned by the BL. This was therefore done by one of us (Akeroyd) at the University of Nottingham and then added to the PDFs. The BL scan is fully text searchable so that any table, section, or page can be accessed rapidly. It is easy to find key words in the text chapters, which enables a high degree of access to the data in a relatively raw form.

## The Content of *HiA*

Online Chapters 1 and 2 give an overview and describe the methods; Online Chapters 3, 4, and 5 report, respectively, the prevalence, distribution, and type of hearing impairment; Online Chapters 6, 7, and 8 report, respectively, hearing impairment by age, social classification, and regional districts current in the mid-1980s; and Online Chapters 9 and 10 cover self-reported hearing impairment.

A first-time reader may be somewhat daunted by the amount of data and the number of tables. As an orientation, it is therefore useful to give examples of some tables that are most useful for answering particular questions:

- For the overall prevalence of hearing loss (four-frequency average) in the better ear across age, gender, and occupational group, see Table B5124-1 (online p. 46).
- For the prevalence of hearing loss up to 95 dB for those aged 80+, see Table 8.1 (online p. 822).
- For medico-legal purposes, see the tables in Online Chapter 4 for decade bands giving audiometric averages and distributions for each of the eight test frequencies, stratified by gender, and manual/nonmanual occupational group. Online Chapter 6 is also useful, giving data in single-year bands of three- and four- frequency averages by gender along with their distribution in percentiles.

In more detail, Online Chapter 3 reports the cumulative prevalence of different degrees of hearing impairment (0 to 95 dB in 5- or 10-dB steps) for each ear and frequency (plus frequency average) as a function of age-group (decades), gender, and occupational group (manual vs. nonmanual). There is a table for every combination of age (18–30, 41–40, 41–50, 51–60, 71–80, or all ages), gender (male, female, or both), occupational group (manual, nonmanual, or both), audiogram (averages of 0.5, 1, 2, 4 kHz; 0.5, 1,2; 0.25, 5, 1; 1, 2 3; 1, 2, 4; 4, 6, 8 then single frequencies of 0.25, 0.5, 1, 2, 3, 4, 6, or 8 kHz), and ears (better or worse). Online Chapter 4 reports the distribution of hearing threshold levels (mean, standard deviation, confidence intervals, and percentiles in 5% steps) for the same combinations of classifications, along with new classifications of everyone or “normal” ears (i.e., with no air-bone gap or noise exposure) only, and better, worse, left, and right ears. The plots on pp. 357–358 illustrate the mean hearing losses by gender and decade for the better and worse ears, also left, right, and overall.

Online Chapter 5 reports prevalences classified by conductive loss, sensorineural loss, or normal hearing with factorial tables by age, gender, and occupational group. Online Chapter 6 provides numerical estimates of the percentiles of the distribution of hearing impairment year-by-year then models the percentiles of the hearing threshold distribution using a smoothing window within the regression, as a function of age, gender, and type of population. Note that this is the only chapter that reports modelled data; all other chapters use estimates from the data that are not smoothed or modeled in any way. The tables are structured by age, gender, otologically typical or otologically screened (defined as excluding those with significant noise exposure, any noise immission rating greater than 0, or any air-bone gap), conductive or sensorineural hearing loss, and ear.

Online Chapter 7 presents the distribution of hearing impairment as a function of a full social class coding according to head of household. The tables report combinations of age, gender, and particular ear. Online Chapter 8 projects the data to estimate the number of people at different levels of severity, for local authority and health regions and districts in Great Britain, as at 1992. The numbers were derived using census figures for the population and its age structure; they have been recalculated since (e.g., Action on Hearing Loss, 2015; Akeroyd et al., 2014; Projecting Older People Population Information, 2017). Finally, Online Chapters 9 and 10 examine self-reported disability. Online Chapter 9 reports first the distribution of replies to every question asked in the postal questionnaires for the main study as a function of age-group, gender, and occupational group. This includes all the responses that were coded for the large random sample of the population. Next, it gives the responses to the same postal questionnaire for the subset of people whose hearing was also audiometrically measured. The distribution of thresholds by the different choices of questionnaire responses is also given. Online Chapter 10 presents similar information to this for the IHR Hearing Questionnaire. Finally, there are four Online *Appendices* reproducing the questionnaires and protocols, together with a Foreword by Mark Haggard and a Preface by Adrian Davis.

## Commentary

The accompanying digital reprinting of *HiA* makes the data and conclusions as widely available as modern technology allows. In doing so, it highlights the clearest weakness in the NSH as a project, namely, its dissemination. Just 550 copies of *HiA* were printed, and so there was a general failure to secure the justified broad distribution and promotion of the study.

Timeliness is high on the list of desirable properties for science, so it may be asked why we are now republishing research prioritized and conceived in the 1970s and executed in the 1980s. We argue that the NSH’s continued relevance is rooted in the scale of the study and the quality and breadth of its data. The quality is due to the profound multidisciplinary professional care taken in its design, protocols, execution, and analysis. This level of care required substantial resources; thus, the field of hearing arguably owes a permanent debt of gratitude to the U.K. Medical Research Council, and the English, Welsh, and Scottish Health Departments. In the late 1970s and early 1980s, their funding of MRC IHR (see Appendix) enabled it to build up a team of applied scientists with the necessary multidisciplinary knowledge, ensured it had the breathing space for a profound consideration of methodological and logistical issues, and allowed piloting to work out exactly what needed to be done and how, all with the purpose of giving the requisite generalizability and precision. This marriage of research resources and hospital resources was crucial, and is usually hard to achieve. It is perhaps unlikely that such a harmonious conjunction of resource, expertise, purpose, and team structure would occur again in the foreseeable future. The NSH may remain unequaled in its experimental care.

The question of whether the data remain valid may also arise from the 30+ years elapsed since it was collected. In terms of *use*, the simple answer is yes, as evidenced by the fact that it was one of seven data sets used to determine the current ISO standard for the statistical distribution of hearing thresholds related to age and gender (ISO 7029:2017). Almost 90% of the *HiA* median thresholds for the combinations of Frequency × Age Band × Gender (e.g., Table 454) are within ±5 dB of the corresponding ISO values, though the differences reach up to 10 dB for some of the 8-kHz data or for the 71 to 80 age band. In terms of *purpose*, the answer is more complicated because a study like *HiA* is unlikely ever to be replicated at the same scale and quality as the NSH and because the population, with its health and life, has moved on. Certainly most of the empirical methods are still used; someone’s hearing is still established subjectively by self-report questionnaire and objectively by pure-tone audiometry. Stratification questions similar to “Do you find it very difficult to follow a conversation if there is a background noise, eg TV, radio, children playing?” are still used routinely in survey or cohort work (e.g., Biswas, Lugo, Gallus, Akeroyd, & Hall, 2019; NatCen Social Research, 2018; Sawyer, Armitage, Munro, Singh, & Dawes, 2019). They have a continuing history that would enable any drift over time to be studied. Large-scale audiometric surveys have not been conducted in the United Kingdom since. Instead, interest has turned to methods such as a digit triplet test of speech in noise (e.g., Biobank: *n* = 164,770; Dawes et al., 2014) or a two-tone, three-level screening test (e.g., the 2014 Health Survey for England: *n* = 3,292; Scholes, Biddulph, Davis, & Mindell, 2018). Both methods are more efficient than audiograms. However, for the purposes of population-level measurement, neither provide sufficiently precise measurements, which require specialist equipment and rooms, trained staff, and take substantial time.

Other changes with methodological implications have occurred, such as a major change in the SES marker used in the stratification. “Occupational Group,” the favored SES marker of the era and used in the NSH, was based on head-of-household information and coded by conventions of the time (OPCS, 1980). In the NSH, this was used to make a separation between manual and nonmanual, as it was a simple social descriptor, and was appropriate at the time given participants’ working lifetimes and industrial socio-economic status environments. In the United Kingdom, the OPCS system has been replaced by the National Statistics Socio-economic Classification (Rose & Pevalin, 2003), which does not perpetuate the manual/nonmanual divide.

Since the 1980s, there have been only a small number of long-term changes that could affect the overall hearing of the population as a whole and thus reduce the data’s validity (World Health Organization, 2018). First, noise exposure will always be a concern, be it from occupation, military service, or loud music. Present-day health and safety regulation ought to reduce its effects, but whether a general reduction in heavy industry (at least in the United Kingdom) balances out the higher intensity levels deliverable on affordable MP3 players and related devices is somewhat debatable. There is no clear consensus yet on the population-level effects of noise exposure, probably because of their multivariable nature and the individual differences in susceptibility. The noise immission rating, a systematic questionnaire developed by Coles and Lutman, was the basis for quantifying lifetime noise exposure retrospectively in the NSH. The question of how best to determine post hoc lifetime noise exposure remains a topic of current development (e.g., Guest et al., 2018; Lutman, Davis, & Ferguson, 2008; Moore, Zobaym, Mackinnon, Whitmer, & Akeroyd, 2017).

Second, the general improvement in health and longevity of the current older population should also affect hearing. Whether this is for the better or worse depends on the perspective adopted and whether full adjustment for the four main factors is presupposed. For an individual of a given age, lifelong improved health and nutrition plus regulation and awareness concerning noise exposure should have made the prospects slightly better (though with a plateau in recent years), but for the population in total, having many more older people will have made the population average hearing level worse. The life expectancy at age 65 years has increased from 13 and 17 years (males and females) to 19 and 21 years (Office for National Statistics, 2016). The magnitude of this increase in longevity means that the original absolute all-age prevalences should not be repeated without expressing some restrictions or qualifications, as these have become too low. Age-banded estimates should be safe, and projecting to the current population age structure would be a relatively simple piece of modeling with altered age-band weights. Making *HiA* available enables this.

Third, the growth of obesity and diabetes is a major change in population health around the world. There is now consistent evidence from large cohort studies of an association of diabetes with hearing loss (e.g., Kim et al., 2017; Loh, Hannula, Ohtonen, Sorri, & Mäki-Torkko, 2015), although the effect size is only moderate. It could be reasonably argued that extending the four-factor predictive model to include obesity or diabetes (not forgetting genetics) should be a priority for secondary use of the data. They were not included in the NSH because at the time of its design the evidence for associations with diabetes and body mass index was slender, and with a modest effect size, it is likely that major insight and specific prediction for the rarer degrees of hearing loss will only emerge when interactions between obesity/diabetes and other contributory factors are considered. Even now, the detailed information on such interactions that would be necessary is not present in the literature because of the very large sample size and judicious choices of pathological markers required to illuminate them. Perhaps the ideal “NSH 2.0” then would be as part of a general-purpose cohort study designed to explore these wider links and also allowing new measurement techniques (e.g., genetics, neuro-imaging) that were not available at the time. Unfortunately, the desired bridge to contemporary populations can thus not be found until the future, when such studies have been designed, funded, and completed.

Against this reservation about the possible incompleteness of the projection model into the indefinite future and to all populations must be placed the several favorable considerations discussed earlier, of which the main two are (a) the continuity of standards in the audiological test measures plus relevant questionnaire items and (b) the uniquely comprehensive design and sampling, otological screening, and attention to measurements. These give the NSH a continuing importance and relevance. It remains a primary source of formal control data and detailed distributions of audiological level by age, gender, occupation, and noise exposure. Thus, despite it being nearly 25 years since *HiA* was published and about 40 years since the project was started, much scientific and public comment on the prevalence of hearing loss (e.g., “one in seven adults”) is, ultimately, traceable to its data.

## Appendix: A Brief History of the MRC Institute of Hearing Research

The MRC Institute of Hearing Research was founded in 1977 following the advocacy of two members of the Wilson Government of the United Kingdom: Jack Ashley (Parliamentary Private Secretary) and Barbara Castle (Secretary of State for Health and Social Services). It was an intramural unit of the Medical Research Council, which was set up to conduct and coordinate multidisciplinary research in hearing. The NSH was one of its primary projects; the need for multicenter studies set the requirement for the multicenter structure of IHR that continued throughout its history. The Institute closed in 2018. See Ashley (1992, p. 346) for some of the political background, Haggard (1978) for its earliest history, and https://www.nottingham.ac.uk/mrcihr/index.aspx for a final overview.

## Supplemental Material

TIA887614 Supplemental Material - Supplemental material for Hearing in Adults: A Digital Reprint of the Main Report From the MRC National Study of Hearing Click here for additional data file. Supplemental material, TIA887614 Supplemental Material for Hearing in Adults: A Digital Reprint of the Main Report From the MRC National Study of Hearing by Michael A. Akeroyd, George G. Browning, Adrian C. Davis and Mark P. Haggard in Trends in Hearing
