# Supplementary material for: Hearing in Adults: A Digital Reprint of the Main Report From the MRC National Study of Hearing
Source: Trends Hear. 2019 Dec 20;23:2331216519887614. doi: 10.1177/2331216519887614 (PMC7000909; doi:10.1177/2331216519887614)
Supplement: TIA887614 Supplemental Material - Supplemental material for Hearing in Adults: A Digital Reprint of the Main Report From the MRC National Study of Hearing [file TIA887614_Supplemental_Material.pdf]

HEARING IN ADULTS

ADRIAN DAVIS

Hearing in Adults

The prevalence and distribution of hearing impairment and reported hearing disability in the MRC Institute of Hearing Research's National Study of Hearing

Adrian Davis

MRC Institute of Hearing Research

Nottingham

W

Whurr Publishers Ltd

London

List of Contents

Page number

Chapter 1
The MRC Institute of Hearing Research's National Study of Hearing - Overview **1**

1.0 Scope and introduction 1
1.1 Terms and concepts 1
1.2 The plan of the chapters 4
1.3 Uses for the data 7
1.4 Summary and conclusions 8

Chapter 2
Methodology of Study **11**

2.0 Scope and introduction 11
2.1 Aims of the study 11
2.2 Design of the main study. 12
2.3 Preliminary results pertinent to the design of the main study 15
2.4 Audiological and otological procedures 16
2.5 Bias reduction and data analysis 18
2.6 Estimation and analytical procedures 20
2.7 Comparison of main study with the domiciliary follow-up and the household study 21
2.8 Summary and conclusions 25
Figures: section i 27
Table 2.5 31
Table 2.6 34
Table 2.7 38

Chapter 3
Prevalence of hearing impairment **43**

3.0 Scope and introduction 43
3.1 Definitions of prevalence 43
3.2 The prevalence tables: summary 45
Tables: section i 46
Tables: section ii 100
Tables: section iii 172
Tables: section iv 226
Tables: section v 298

Chapter 4
Distribution of hearing impairment **323**

4.0 Scope and introduction 323
4.1 Definitions of distribution of hearing impairment 323
4.2 Summary and conclusions 326
Figures: section i 327
Figures: section ii 357
Tables: section i 375
Tables: section ii 438

Page number

Tables: section iii 501
Tables: section iv 564
Tables: section v 627

Chapter 5
Type of hearing impairment **691**

5.0 Scope and introduction 691
5.1 Arrangement of the tables 691
5.2 Summary and conclusions 691
Tables: section i 693
Tables: section ii 706

Chapter 6
Hearing impairment as a function of age **723**

6.0 Scope and introduction 723
6.1 Arrangement of the tables and figures 723
6.2 Summary and conclusions 724
Figures: section i 725
Figures: section ii 735
Tables: section i 749
Tables: section ii 779

Chapter 7
Hearing impairment as a function of social classification **811**

7.0 Scope and introduction 811
7.1 Arrangement of the table 811
7.2 Summary and conclusions 811
Table 812

Chapter 8
Hearing impairment as a function of country, region and district **821**

8.0 Scope and introduction 821
8.1 Estimating numbers of hearing-impaired people 821
8.2 The number of hearing-impaired people in each region 822
8.3 The number of hearing-impaired people in each district 823
8.4 Summary and conclusions 824
Maps 825
Tables: section i 829
Tables: section ii 833
Tables: section iii 836
Tables: section iv 837
Tables: section v 838
Tables: section vi 884

Page number

Chapter 9
Reported disability and service use **893**

9.0 Scope and introduction 893
9.1 Responses to the questionnaire 893
9.2 Hearing impairment distribution for answers to postal questionnaires 894
9.3 Hearing aid possession 896
9.4 Summary and conclusions 896
Tables: section i 898
Tables: section ii 908
Tables: section iii 953

Chapter 10
The IHR hearing disability questionnaire **957**

10.0 Scope and introduction 957
10.1 Responses to the questionnaire 957
10.2 Summary and conclusions 958
Table 959

Appendix 1 **987**
Postal Questionnaires

Appendix 2 **996**
Pure Tone audiometry

Objectives
Equipment
Instructions
Procedure
Coding and Computer Entry
Definition of presence and type of hearing impairment

Appendix 3 **1000**
Otoadmittance measurements

Objectives
Equipment
Procedure

Appendix 4 **1001**
IHR Hearing questionnaire

References **1007**

ISBN 1-897635-40-0

© 1995 Whurr Publishers Ltd
First published 1995 by
Whurr Publishers Ltd
19b Compton Terrace, London N1 2UN, England

All rights reserved. No part of this publication may be reproduced, stored in a retrieval system, or transmitted in any form or by any means, electronic, mechanical, photocopying, recording or otherwise, without the prior permission of Whurr Publishers Limited.

This publication is sold subject to the conditions that it shall not, by way of trade or otherwise, be lent, resold, hired out, or otherwise circulated without the publisher's prior consent in any form of binding or cover other than that in which it is published and without a similar condition including this condition being imposed upon any subsequent purchaser.

British Library Cataloguing in Publication Data
A catalogue record for this book is available from the British Library.

ISBN 1-897635-40-0

Singular no. 1-565932-43-9

Printed and bound in the UK by Hobbs the Printers, Southampton, UK

Foreword

In recent years there has been a shift in the way that researchers have to think through the justification for what they do and the way in which the information that they provide can or should be used. An early example of that recognition, and the style of epidemiology to which it has led, can be seen in an early paper from the National Study of Hearing (Haggard, Gatehouse and Davis, 1981). In short, the emphasis has shifted from 'medical conditions' to treatable states according to severity, and from incidence to clinical case services, to population prevalence. This volume proceeds along the same path but at a wholly contrasting level of detail. It focuses on tabulated quantitative information from the National Study of Hearing (NSH) that will be actively used to inform epidemiology, audiology and service planning. Service purchasers now have to base their plans and contracts for purchasing upon 'assessments of need' in local populations; the requirement for our version of epidemiology is thus becoming even more widely recognised. Adrian Davis' tabulations therefore represent a major applicable product of the NSH, and in terms of quantitative detail, the extreme one. Other aspects, introductions and more discursive or accessible accounts of the Study are also available in the articles cited in the bibliography.

Pursuit of a planning perspective in detail does, of course, leave other important topics uncovered. Little is said here about the pathologies or causes of the hearing impairments, because methods for such studies have not yet reached the take-off point that would make the aetiological epidemiology scientifically fruitful. Nor is much said about the health care histories of individuals, from the point of view of either need or effectiveness; in the NSH there was limited time and subject tolerance in the interviews and so those questions were excluded.

A large part of the service purchasers' recently defined function is to acquire and deploy research intelligence as a basis of negotiating contracts with service providers. However it is difficult fully to specify what you need to know, as that would imply you already half know it. The purchasers and 'end-users' of research, should ideally contribute to judging what research should be done and the way in which results of research would be used. They may not in fact be well placed to judge this, nor hence to stipulate the form that the research and its presentation should take in detail. Researchers therefore have to be assiduous and honest as well as communicative about society's requirements in arriving at the decision on precisely what should be done and how it is to be passed on. Planning of the NSH could not have involved service purchasers as research users, because it was a decade before they were even to come into existence.

Changes in mode of production are relatively straightforward to understand, if sometimes tedious to implement. At the research stage, the equipment for most of the tests in the National Study of Hearing changed only minimally in the early 1980s when data were being gathered. However, over the 15-year life of the study and analysis, there was one major change of the outstation computing systems for data gathering and entry, and three major changes in the central analysis computer. The study was conducted through and largely for the National Health Service. Since inception of the study the Health Service has undergone one major reorganisation (1989-93 reforms), plus two intermediate ones (1984 - general management; 1993-5 - combining of most Regions, combining of some districts, combining of DHAs with FHSAs and other aggregation of the purchasing function), and one minor reorganisation (1981 - abolition of Area Health Authorities). Some of these changes and their side-effects consumed much time of the senior personnel involved in the Study, but they reflect also its changing background. Amidst all these types of change, those who propose or are asked to undertake large research projects of necessarily long duration have to reconcile long-term determination with medium-term adaptability, and to cope with changes both of technology and distribution system. In respect of the NSH, a tenacity of purpose has been required, so as to bring the concept of an applicable quantitative epidemiology of need to fruition in a particular form that was going to be useful to those working in the NHS at a

particular time in a future partly unforeseeable. Although recognition of the value of our quantitative epidemiology of need has been gradual, we had to follow its imperatives. New standards had to be set for perspectives, rigour and quantitative precision, in the anticipation that the demand and the channels for implementation would indeed evolve to meet up with the research 'product' when it was ready. Such anticipation of a meeting up with new demand and new means of implementation is more characteristic of the planning of R & D in industry.

'Needs assessment' can apply to the individual or to the population. There has to be a correspondence between these levels of analysis, as the benefit from extending provision of services in the population can only be calculated from a convolution of the prevalences of various severities (or case-types) in the population, with the degrees of benefit (for example from a hearing aid) that variously representative groups of individuals have been shown to receive. This calculation may have to be done in terms of cost/benefit or cost/effectiveness, rather than in terms of benefit alone. As an extreme contrast, the benefit to quality of life (especially if computed with a ratio rather than a difference measure) for a cochlear implant in a profoundly deaf person may be greater than that for a hearing aid in a moderately affected person; considered relative to cost, the advantage might reverse. Given that over much of the range, the severity of impairment as given by hearing level is a reasonable proxy for the need for, and magnitude of benefit from, a hearing aid, the quantitative epidemiology of impairment is the first and most important step in service planning, so is the main topic of this book.

Services are mostly purchased on an incidence basis, but in the instance of a condition with which a person typically lives for a decade or more, prevalence gives a better indication of the extent to which population need is being met. The extent of shortfall, and hence the rate of provision required to meet a reasonable target in a given time (increasing say, over a five year period, provision of hearing aids from one third to one half of those over 35 dB HL in the better ear) can be calculated from prevalence data. Incidence data are less useful; the number of new 'cases' per year is not informative, because of the arbitrariness of definition of a case. Also, because of the low provision levels for existing cases and the requirement to upgrade earlier provision in the light of changes both in technology and in individuals, the number and nature of new qualifying cases relates only indirectly to issues in service planning. We have therefore refrained from generating pseudo-incidence data by differencing the prevalence curves, more for practical reasons than for reasons of difficulties with the statistical assumptions. In emphasising prevalence data, of course, one is not discussing prevalences of dichotomous conditions but the percentile points on a distribution, so that the numerical implications of a particular cut-off may be deduced. Treatment should follow not categorical diagnosis but the quantified ability to benefit. As an example of this philosophy, in clinical trials nowadays it is customary to design in enough power to determine what case-types (within a broad group of diagnoses previously receiving the treatment) or what degrees of composite risk justify the intervention suggested, in terms of demonstrable magnitudes of benefit.

In due course we may expect that it will be possible to produce a more detailed population model of the ability to benefit. So far, only hearing threshold level has been used as the predictor, but variables distinguished here (age, sex, socioeconomic group, noise and presence of a conductive component to the hearing losses) have the potential to contribute in combination. If data on the ability to benefit from the next generation of hearing aids can be obtained, stratified in this way, more refined bases for targeting according to the ability to benefit can be generated. Such a scientific basis for selectivity should not be mis-used to deny entitlement of individuals seeking services such as a hearing aid. Rather, because the approach to a long-term planning target of the above type as set out in the "Health of the Nation" cannot be instant, the resources for achieving short-term targets have to be focused on the strata where the highest benefit is shown. This serves to maintain the credibility of a more proactive approach and to ensure that any increase in cost is accompanied by a greater increase in effectiveness. The suggestion of criteria for candidature has raised some hackles in audiology, but no realistic

alternative has been put forward; *laissez faire* is likely to be both inefficient and inequitable. There is nothing intrinsically radical in having a planning target, especially if it also serves to modulate the access to limited resources across health sectors (promotion, rehabilitation, treatment) as well as across the sectors outside health care (e.g. occupational hygiene and safety).

A major consideration in the decision to produce results of the NSH in the present form has been the sustained intense demand for prevalence figures on the part of the UK Health Departments, voluntary organisations, and health authorities, for the planning of services. The economic presuppositions of the Health Service, and hence of this demand for data, have changed over the 15 years since the study began. However the forms in which the data have been requested, always diverse, have not changed systematically. Nor has the demand dipped recently, indicating no trend for the information requirement to have already been adequately met at the detailed level. Our involvement in this "reactive dissemination" (the preparation of special reports and presentations or consultations to supplement the basic work already published) has taken much time away from the science. The tables in this book are an efficient way of meeting the user requirement for the data, a requirement already abundantly documented. It is expected that the evolving requirement will be met by the book, plus other dissemination activities.

The reader coming new to this book may wonder who is going to browse through all the detailed tables. Such a reaction would be both uninformed by the point about 'reactive dissemination' made above and reflect unawareness of recent changes in modes of production. Such changes affect not only the execution of research but also its dissemination. There is available a range of media to which additions are continually being made: primary journal article; secondary (e.g. magazine) digest or popularisation, conventional book and monograph; self-published tabular report with limited circulation; dissemination bulletin; data archive; electronic publishing etc. It is necessary to choose the *combination* which meets the dissemination requirement most cost-effectively. We appreciate the partnership of Whurr publications in the present experiment of creating a 3-way hybrid between the traditional book, the data archive, and the tabular report. The obvious merit in this step is the covering of the traditional aims of each of these three media with the labour required for one alone. Another merit is the use of new technology to enable a highly specialised niche-market book to be produced, despite its substantial size, at moderate cost. The success of the experiment will depend partly upon parallel dissemination activities and on the mode of 'marketing' and accessing the product, i.e. on the efficiency of the relevant channels of access.

The more complex and sophisticated aims of archiving can only be met by collaborative working with people who understand in detail the meaning of the data items, and the structures and idiosyncrasies of the total data set, including the obstacles tackled in previous analyses. This means working with the investigators. In line with tradition, the data are maintained at IHR in such a fashion as to permit this when an application emerges which meets the criteria that would justify collaborative effort, in the opinion of those who would have to put in that effort. Amid resource constraint, the concentration of highly complex facilities is increasing the requirement for outsiders to be accommodated in this way. We have divided the archiving requirement. A second, more general, need for archiving, including certain straightforward demands for control population data in other studies is met by this book, as illustrated by the examples shown in Chapter 1. We have chosen not to follow one possible third path in archiving: documenting the data set with a data dictionary, a manual, and citations of publications that share the burden of explanation. That is much less appropriate in our case: the efficiency, the degree, and the appropriate use of that channel of access are uncertain; some datasets are popular, and others not, depending on the size of the community of secondary analysts, and the generality of the data.

Preface and acknowledgements

This book reports two major aspects of the epidemiological studies undertaken by the Medical Research Council's Institute of Hearing Research to 'further fundamental research' (Ashley, 1992) into hearing impairment in Great Britain. The major studies reported here have been collectively called the National Study of Hearing (NSH). The NSH started with pilot studies in 1979/80 and was followed by cross-sectional aspects which were accomplished predominantly in four districts around Cardiff, Glasgow, Nottingham and Southampton between 1980-86. The longitudinal aspects are still in progress and will monitor the change of hearing with age over a 12 year period. The analyses and comment in this book are intended to complement the large number of papers published (see Chapter 1 for a list of the major publications) and to distil the data into a form that is more useful to a variety of professionals than the summary data available in many publications. I have included more of these analyses at the expense of copious comment, to maximise the utility of the data to other professionals.

This book presents two major specific sets of statistics that we planned the NSH should collect: (1) concerning the extent of hearing impairment in this country, and (2) the way in which hearing varies with age. In addition an estimate is made of the number of hearing-impaired people in each health and local authority district in Great Britain, for a variety of severity criteria. The inclusion of data on hearing disability as well as on impairment and the correspondence between the two may be particularly useful to social scientists who do not have access to audiological expertise but who nevertheless wish to investigate the impact of hearing disability on people's lives. It is hoped that this book will be of substantial benefit (1) to health care purchasers who need to make local assessments of needs, (2) to providers of hearing health care when benchmarking severity and risk factors in individual cases, or when monitoring their performance and comparing their clients/patients to the national distribution of hearing impairment/disability, and (3) to those who wish to use the control data presented here to assess the impact of noise or other potentially damaging factors on auditory status.

In writing this book and compiling the tables for each chapter, I have been acutely aware of the support and encouragement of a large number of colleagues over the preceding years. A truly large number of people helped at some stage with the NSH and with this book. Mark Haggard has been influential at all stages of the NSH from its piloting to its dissemination, and has provided great encouragement, support and scientific insight throughout the study. Ross Coles supplied an endless fount of enthusiasm for the study, its audiological procedures and especially for the tinnitus research, which was much appreciated.

The NSH would not have been possible without the first rate collaboration of the major investigators; Mark Haggard, Ross Coles, Stuart Gatehouse, George Browning, Mark Lutman, Pauline Smith and Roger Thornton and their audiological staff Robert Rendell, Diana Callaghan, Kevin Thomas, Grace Hardie, Margaret McMillan, Susan Robinson, David Conrad-Armes, Vicki Parr, Daljit Mehton, Ian Bell and Stan Goodsell who carried out the experimental work meticulously and who checked and re-checked the quality of the data. I was also grateful for the support of Rich Tyler, Julian Miller, Barry McCormick, and Quentin Summerfield who provided advice and materials for the study. I would like to thank all the staff who have worked for me on this project, especially Heather Fortnum, as well as

Anne Sempik, Cheryl Haslam, Steve Holdaway, Mike Burford, and Leda Stott, and the many temporary staff who have helped over the years. Bronwen Jenkins helped compile an internal report containing some of these data which have been updated for this book. Eamonn Hughes helped at almost every stage from putting survey letters into envelopes, to visiting OPCS to find names of people for addresses that had not replied to four reminders, to programming the database for the study (which has been transferred through at least five different databases in its lifetime), to editing many books and articles. On the statistical front thanks are due over a long period to Helen Spencer who has helped provide and check most of the raw data summarised in this book and to Nick Longford. The encouragement of many people who rang or wrote letters saying they eagerly awaited this book has also provided energy when the flame flickered.

I would particularly like to single out the extended efforts of Mark Lutman, who has been responsible for the complex calibration procedures and data collection, and of Stuart Gatehouse, who has provided considerable encouragement and critical insight to the procedures and interpretations of what we have done. Over the years Pauline Smith provided considerable help especially with the protocols shown in the Appendices. Their help and contributions to the NSH have been substantial and are greatly appreciated.

Two major external sources of enthusiasm and advice that I greatly valued came from Ron Hinchcliffe and Dai Stephens.

Colin Whurr and Jane Sugarman have been extremely helpful and patient with me during the production of this book.

Such a list is not meant to be exclusive and many other people played small and large parts in the study. Our thanks are due to these people, and particularly those members of the public who volunteered their time to participate and help our research bear fruit.

Finally Eleanor Sibley provided supraliminal messages on my computer workstation entitled 'This book will be finished!' every time I took a two minute break, and she endlessly reformatted tables to make them fit the page neatly, as well as checking the whole manuscript. Many thanks.

Adrian Davis
August 1994

"The main deprivation of deafness is not, as many imagine, the loss of music and birdsong, sad though that is, but the severing of easy communication. It is the end of radio, the emptiness of television, the difficulty of small-talk, the absence of conversational nuances, and the lack of company which mark the mind. Deafness separates humans from humankind."

"The vast majority of hearing-impaired people are neither profoundly nor totally deaf, and for them a hearing aid gives access to the hearing world."

"In the longer term, of course, preventing and treating deafness by furthering fundamental research are even more important than improved hearing aids."

Jack Ashley

Acts of Defiance

Reinhardt Books, 1992

Chapter 1

The MRC Institute of Hearing Research's National Study of Hearing - Overview

1.0 Scope and introduction

This chapter serves chiefly to guide the reader through the main results reported in this book as an aid to more detailed reading and interpretation of later chapters. A series of population based epidemiological studies by the Medical Research Council's (MRC) Institute of Hearing Research (IHR) comprises the MRC IHR National Study of Hearing (NSH). The NSH was conducted to assess the prevalence and distribution of hearing impairment and disability in Great Britain (England, Scotland and Wales). Hearing disability, whose onset is tightly coupled with ageing, is one of the most prevalent of the health problems facing societies where life expectancy has greatly increased over the last century. In Great Britain where life expectancy is currently 76 years, the adult population of about 43 million contains approximately 8.58 million adults who have a significant hearing impairment in both ears (as defined later). This estimate encompasses all severities. Considering only the profoundly hearing-impaired there are probably 170 thousand adults, just over half of whom are ≥81 years of age. Mild to moderate disabilities, suffered by 5.5 million, may also present a greater handicap than has previously been recognised. This represents a serious public health problem due to the increasing role of communications in 20th century society and to the high numbers of very elderly hearing-impaired (Davis 1982).

The data presented in this book give a glimpse of the scale and nature of the need in the British population. In general, this need has not been met by the National Health Service (NHS), as there has been a shortfall of services by up to a factor of about three-fold (Haggard, Gatehouse, Davis 1981; Davis et al, 1992b; Davis, 1993; Haggard and Gatehouse, 1993). This shortfall is due partly to the role of attitudes in service uptake, but partly to issues of planning and to resourcing for the quality and accessibility of services. In the absence of similar data, in other developed countries, the data on prevalence and distribution of hearing impairment presented here would be a good starting point, provided appropriate age/sex stratification is made.

Several major IHR publications have shown different aspects of the epidemiology and aetiology of hearing disorders. The validity of the NSH and the main prevalence data on hearing impairment and reported hearing disability (Davis 1989; Davis 1987) have been complemented by articles on the effect of noise (Davis and Thornton, 1990; Lutman and Davis (Audiology, in press); Lutman and Spencer, 1991), on tinnitus (Coles et al, 1990; Davis et al, 1992a), middle ear disease (Browning and Gatehouse, 1992), self-reported and performance disability (Lutman et al, 1987), and hearing aid use (Davis 1993; Haggard and Gatehouse, 1993) to mention a few. Haggard (1993) in his monograph 'Research in development of effective services for hearing impaired people', summarises the major messages

concerning the prevalence/severity distribution of adult hearing impairment and aetiology. He takes this a practical step further to formulate an appropriate Health Service Research agenda (p.106), rooted in the evaluation of rehabilitation for the major need groups.

This book was planned as a major reference work for the NSH, and gives a detailed breakdown of some of what I regard as the most important of the results relating to hearing impairment and self-reported disability. The following chapters focus on these results; they provide (1) a detailed methodological presentation of the studies, (2) a detailed breakdown of the prevalence of hearing impairment by age group, gender and occupational group, (3) a detailed summary of the distribution of hearing threshold levels in the typical and otologically screened population which can be used as control data, (4) a graphical summary of these distributional results, (5) a descriptive statistical model of the change of hearing threshold levels with age for some percentiles of the obtained distribution, (6) an estimation from the prevalence results of the numbers of hearing-impaired people in the health regions/commissions (pre/post-April 1994) and districts (1992) in Great Britain for planning purposes, and (7) the distribution of reported hearing disability and its relationship to hearing impairment.

As mentioned in the foreword to this book, I expect that there will be several types of 'reader' who encounter this book. It is essentially a reference book and this chapter shows what is available to the reader, e.g. (1) in assessing prevalence or numbers of hearing impaired people, (2) in comparing individual or groups with standard audiological data, and (3) in contrasting reported hearing disability and impairment.

1.1 Terms and concepts

Hearing in Adults describes some aspects of the hearing of the adult population in Great Britain. There are many dimensions to hearing that might be studied, from which a limited number that reflect the public health importance of the data have been chosen. It is useful to employ the World Health Organisation's (WHO, 1980) definitions of impairment, disability, and handicap when describing hearing disorders (Davis, 1983; Stephens and Hétu, 1991; Hyde and Riko, 1994). In this framework, hearing impairment is the limitation of a function of the auditory system. The threshold of hearing for a pure-tone may be considered to measure one aspect of hearing impairment. In other words a lower threshold, having better hearing sensitivity, means less hearing impairment. Whilst the ability to detect differences in frequency, time and space for simple and complex sound stimuli are also useful measures of the performance of the auditory system, they are not reported here. On the one hand the hearing threshold level is highly correlated with these measures, and on the other hand perceiving the signal is a basic prerequisite for further processing. Temporal and frequency resolution were measured in this study and reported by Lutman et al (1990).

Perceiving a sound enables a person to process and, if necessary, respond to the sound. When this ability, for instance, to communicate with someone in quiet or in a room with, for instance, a noisy fan heater is interfered with as a result of a hearing impairment this then results in a hearing disability. Hearing disabilities may fall in to several categories e.g. recognising speech, recognising auditory signals

(e.g. doorbell, phone ringing), auditory localisation, sound identification, and environmental awareness (cf Stephens and Hétu (1991) for a fuller discussion). The nature of these disabilities may change substantially in the presence of noise, e.g. a fan. Disabilities are usually measured by questionnaire and some responses to disability questions will be presented in Chapter 10. In general the distinction between impairment and disability is an important one to make particularly in the context of rehabilitation.

The handicap experienced by someone with a hearing problem is the effect of the hearing disability on that person's social and economic life. In a way this may be reflected by a quality of life (QOL) measure. However, that measure would probably have to incorporate more than the traditional QOL-measures do, as those would not be sensitive to the need for extra effort needed in communication, for instance. In terms of rehabilitation for sensorineural hearing impairment, the aim of rehabilitation is to nominally provide as much benefit as possible by reducing the handicap and disability, usually by effective provision of a hearing aid. The underlying impairment remains the same even though it is customary to anchor the degree of benefit conveyed back into the equivalent degree of impairment, as that is widely understood. There is no hard and fast compartmentalisation of impairment and disability on the one hand, or of disability and handicap on the other. For instance an ease-of-listening measure such as the differential time to verify the sense of a spoken message when aided or not aided may convey good information about the extent to which a hearing disability is overcome. Combined with other information it may be a good indicator of the reduction in handicap. Similarly, the combination of hearing threshold levels, which individually indicate some aspects of impairment, may in combination be good indicators of hearing disability - at least in its simple sense.

The ease with which it is possible to get objective measures of impairment, disability and handicap decreases as we move from impairment to disability and then from disability to handicap. As all three concepts are usually highly correlated it is parsimonious to accept for certain general purposes that hearing impairment measures are the 'gold standard' from which one could assess the broad extent and type of needs in the population. The exact nature of the disability and handicap, taken together with biographical and psychological variables, e.g. age and personality, may be more pertinent for assessing the nature and expected benefit of the rehabilitative package provided for an individual, i.e. the need in an individual.

This book therefore concentrates particularly on the distribution of hearing impairment, as measured by hearing threshold levels in the population (Chapters 3, 4, 5, 6 and 7). We assert that these measures are probably the best measures of the extent of hearing health need in the population. By need, we mean the potential to benefit from intervention, whether that is advice, training in hearing tactics, provision of environmental aid to hearing, or provision of a hearing aid or of hearing aids.

No truly objective measures of hearing disability or handicap are provided in this book. Indeed, there is little agreement on what these would comprise and they would certainly have to be elaborate and expensive. However, we report subjective data: the answers to two of the questionnaires that were

administered to the population (Chapters 9 and 10). These serve three main purposes. Firstly, the extent of need as reflected in individually reliable hearing impairment measures can be calibrated in the regulation against the measures of self reported disability in the questionnaire. Secondly, it is possible to calibrate the individual questions, i.e. the self-reported aspects of disability, against the impairment level distributions. For example we can see that three quarters of those 2.4% of men who report 'great difficulty following the TV news when the volume is adjusted for others' have better ear hearing threshold levels in excess of 35 dB. Thirdly and conversely, it is possible to characterise the disability in specific concrete ways that may be of particular concern, e.g. access to communications. Not merely is the foregoing question a good one because of the relatively standard interpretation it involved in otherwise differing people, but it is a matter of direct particular concern to broadcasters and voluntary organisations for the hearing-impaired.

This book contains measures of hearing impairment and of self-reported disability and some aspect of handicap. It is important in the following chapters to understand the difference and relationships between these two indicators of need for hearing health services. The major problems with self-report are that it is difficult to verify and is subject to various classes of error which require large numbers and careful analytical effort to overcome.

1.2 The plan of the chapters

A previous publication (Davis, 1989) has described the NSH in some detail. The background to the NSH and its method are expanded upon in Chapter 2. It is important to understand the nature of the sampling undertaken in the study, so that the results can be used most effectively. Without that understanding it is possible to be confused or even make wrong judgements about the meaning of some of the statistics referred to in the tables that follow. For reasons of sampling efficiency the population with a high degree of self-reported hearing disability was sampled more densely than those people with a low degree of self-reported hearing disability, e.g. all the people who reported, in the initial questionnaire, that they used a hearing aid were contacted to take part in the study, whereas only about 1 in 25 people who reported no hearing problem whatsoever were contacted. The estimates of hearing impairment for the population are statistically reliable and unbiased estimates which have to take this fact into account. Finally, all estimates concerning hearing impairment have been constructed to reflect the population of adults in Great Britain.

It follows from this that with the NSH that the sample (the actual people tested) prevalence or mean is not the best estimate of the population (the people of Great Britain) prevalence or mean. The sample is weighted to reflect the population. This is a standard statistical technique that does not affect the validity of the data, only the technical procedures for making estimates of statistics such as a mean or proportion. The particular strata that are used for sampling, e.g. district, age, reported hearing disability and for reference to the Great Britain population (post-stratification by age and gender) are together known as the design matrix for the study.

The extent to which non-response or non-representativeness of the sample in some way may have distorted the results is addressed in Chapter 2, by comparing and combining three different samples. Together with the evidence presented in Chapters 9 and 10 concerning the validation of the estimation procedures, i.e. by comparing the initial questionnaire replies for the subset who underwent audiological investigations with the whole set of people who answered such questions, the overall picture is one of good reliability. Occasionally there is divergence which often occurs when using unusual subsets of the data, where large amounts of data were missing on particular variables, or when the variable of interest is not related to the stratification variables. Given an infinite number of possible analyses, it is not possible to balance the design matrix for all possibilities. However, for the main variables of interest to this book the design matrix is stable, reliable and valid (Davis, 1989).

The two main data chapters present the prevalence of hearing impairment (Chapter 3) and its distribution (Chapter 4). The first data chapter (Chapter 3), presents the cumulative prevalence of different degrees of hearing impairment for each ear and frequency (plus frequency average) as a function of age group, gender and occupational group. The hearing threshold range is split into fifteen categories ranging from ≥ 0 dB in 5 dB steps up to ≥55 dB HL and then in 10 dB steps up to ≥ 95 dB HL. Only people in the age range 18-80 are included in these chapters.

The distribution of hearing threshold levels (Chapter 4) contrasts the population (all the sample, or the typical population) with the screened subsample who did not experience any substantial noise immission (Davis, 1989) or have any air-bone gap as a function of ear, age group, gender and occupational group. The definition of substantial noise immission was <80 dB Leq 40 for a 50 year working lifetime or equivalent equal energy[1]. It is important when using these data that no biases creep into the comparison of the typical and screened populations. For instance, if there was a substantial difference in age within the 10-year age groups this might introduce an unwanted bias in to the comparison. It is therefore particularly encouraging that individual 10-year age bands show a reliable contrast between typical and screened populations in terms of the compatibility in age, with no difference between the populations exceeding 0.3 years.[2]

The extent to which the hearing impairment in the population has a conductive origin, is shown in Chapter 5. The greater the severity of the hearing impairment the greater the proportion of impairments that have a conductive component. Likewise the worse-hearing ear has more impairments with a conductive component. This apparent paradox (profound and total deafness always have a major

---

[1] The air-bone gap is the difference between the hearing threshold by air conduction and the bone conduction which reflects the degree of impairment due to pathology in the middle ear.

[2] However, for the 18-80 population the difference between the screened and typical population is slightly exaggerated as less of the older people actually have no substantial noise immission rating. The average age for the screened population is 42 years compared with 45 years for the typical population. Such a difference in age is slight in terms of the mean/median of the hearing thresholds.

sensorineural component) illustrates the value of population studies in reflecting the contribution to total prevalence, hence to population need of less dramatic pathologies, and of multiple pathologies.

Due to the design of the NSH, as will be shown in Chapter 3, the estimates in Chapters 3, 4 and 5 have been made in ten-year age bands. Chapter 6, however, provides estimates of the distribution of hearing impairment based on running averages over 7 years. These data are tabulated as a function of the centre age, for the better ear at two important frequency averages. The second part of Chapter 6 models the percentiles of the hearing threshold distribution using a smoothing regression technique as a function of age, gender and type of population, and the results shown as a number of contrastive figures for each frequency. Chapter 6 is the only chapter that reports modelled data, all other chapters use estimates from the data that are not smoothed or modelled in any way.

Occupational group is a major influence upon the prevalence of hearing impairment and its distribution, Davis (1989). The contrast between occupational groups (manual vs non-manual social class) is prominent in Chapters 3, 4 and 5. Social class was recorded and coded in a number of different ways in the NSH. Chapter 7 shows the distribution of hearing impairment as a function of a full social class coding, according to head of household (OPCS, 1980). The data emphasise the differences between the social classes, but also show the problems in achieving adequate numbers in controlled cells of the design matrix.

Whereas Chapters 3 and 5 have shown prevalence of hearing impairment as a percentage of the appropriate population, Chapter 8 projects the work in these chapters to administrative districts. The estimated number of people at different levels of severity, for health regions and districts in Great Britain and also local authority districts, in England and Wales, are tabulated and mapped. The estimated number of profoundly hearing-impaired is shown in a separate table.

Chapters 9 and 10 examine self-reported disability. In the first part of Chapter 9, the replies to the postal questionnaires for the main study are given as a function of age group, gender and occupational group. This includes all the responses that were coded for the large random sample of the population. In the second part of the chapter, the responses to the same postal questionnaire for the subset of people whose hearing was also measured are presented. This juxtaposition serves two purposes. The first is to demonstrate the extent of similarity of the prevalence estimates on the questionnaire responses, comparing the subset in whom audiological data are available with the whole of the postal sample. An important element of these data relate to hearing aid possession and consultation. The second purpose is to show the calibration between the self-reported disability and hearing impairment measured by the average hearing threshold averaged across the mid-frequencies (0.5, 1, 2, 4 kHz). Chapter 10 presents similar information to this for the IHR Hearing Questionnaire (given in full in Appendix 4).

1.3 Uses for the data

The statistical digest of the NSH data presented in this book give an excellent basis for planning services for the adult hearing-impaired. Not only are the prevalences of different degrees and types of hearing

impairment shown (Chapters 3 and 5) but the extrapolation is made to health and local authority districts (Chapter 8). These data are of interest to many of the professional groups concerned with providing direct health services for the hearing-impaired, especially ENT, audiology, hearing therapy, speech therapy health provider units, those planning the social service needs, as well as purchasers and special interest groups, e.g. RNID. These data are more valid and reliable than those provided about hearing from the OPCS Survey of Disability (Martin et al, 1987) for three reasons. Firstly in the NSH the postal sift was at the level of the person, rather than household. Secondly, as the NSH was a true population sample at all stages of the investigation there was no major uncertainty introduced of the type met in studies which rely on sifts for reasons of economy which uses some appropriate question screening level of disclosed hearing difficulty at which to proceed to a more detailed interview. This is a particularly major problem in the OPCS study, due to the inadvertent change in the sift question midway through the postal sift, and this is possibly further compounded by the high proportion of elderly people who do not admit a hearing problem and are hence underrepresented in the OPCS data. This is numerically important when estimating the characteristics of people with mild to moderate hearing impairment, as their needs may be met simply. It is less true for the less prevalent severe and profound impairments. Thirdly, the NSH actually measured impairment levels. These impairment levels are the most reliable predictors of need for hearing services in the population and they also provide a parsimonious prediction of the extent of actual 'performance disability', which is not always accurately mirrored by self-report. These three reasons are discussed more fully by Davis (1989).

The distributions of hearing threshold levels permit the reader to quantify the extent to which individuals (or perhaps groups of patients) differ from the expected values of the typical population or from sub-populations screened for noise immission and conductive hearing impairment (Chapter 4). Such comparisons may also be made in graphical format (Chapter 6); the graphs allow for annual birth cohorts, but are restricted here to male and female contrast only; no separate analysis was conducted here for the different occupational groups. As well as the possible use of these data for medicolegal purposes (cf King et al, 1992), the statistics presented in Chapters 3, 4, 5, 6, 7, 9 and 10 may be used for a primary research purpose, i.e. as a representative sample of people in Great Britain. It is also possible to use the NSH data in case-control analyses in a much more powerful way. Such analyses are possible, especially when planned prospectively but require sophisticated statistical analysis when using the 'raw data'.

Two examples of the way in which the NSH data may be used are given below. The statistics presented in this book go part way in enabling the reader to accomplish two constructive levels of general category of use of NSH as reference data, in addition to helping with the clinical management of individual patients. In the first place, it is possible to improve the external validity to findings in the literature. Many studies refer for the demonstration of an effect of some external agent (e.g. drugs, noise, disease) to hearing thresholds on inappropriate and out-of-date standards (such as ISO 7029). If sufficient data concerning the age and sex of the case or control group are available from such studies, it is possible to refer to one of the tables given here to assess whether the purported effect would stand

against the typical or screened representative population data shown here. Generally such comparisons seem to reduce the apparent claimed difference (e.g. Narula, Powell and Davis, 1989).

A second and more powerful use of the data would be to use the NSH data as controls at the outset of the analysis of data which one may have collected, or be about to collect. If the case data are collected independently, it is very easy to test a simple hypothesis with routine statistical procedures and without accessing the raw data on particular cases. For example, if we wish to test that the median threshold at 4 kHz of a particular group of patients aged 41-50 is in excess of the median (or other percentile) of the screened population, we can use the standard median test (Siegel, 1956; Siegel and Castellan, 1988). This assumes that the estimate for the reference distribution is the same as for the population distribution. This is a reasonable assumption when there is a large N contributing to the estimates of the NSH distribution, but may not be so for some smaller strata (e.g. noise-exposed females), or when the estimate is for one of the extreme percentiles. However, hypotheses are usually formulated such that comparisons are made with respect to measures of central tendency, e.g. the median values. The tables given here, in conjunction with the Figures in Chapter 6, enable appropriate contrasts with other data sets or individual patients/subjects. Whilst falling short of a proper case-control study will enable researchers/clinicians to examine the size of effect they have obtained[3].

Across most values of the demographic variables and frequencies, the distribution of hearing threshold in the population is not strictly a "normal" (Gaussian) distribution. It is therefore best to treat with caution comparisons of the means for particular hearing thresholds. The Confidence Intervals (CI) based on the standard error for those distributions need also to be treated with caution. It is probably adequate to state that if the mean of interest falls outwith the given CI then it would be worth following up the finding. At the next stage if the data fall above the mean (i.e. "worse"), but below the upper CI, there may be a more powerful statistical test procedure based on a more appropriate distribution, for which the log-normal distribution is the best candidate (Davis, 1987). The extent to which data can be seen to be skewed towards the more hearing-impaired end of the hearing impairment metric can be seen from the Chapter 4 tables and graphs and also the Chapter 6 graphs. When comparing the prevalence of hearing impairment for one of the standard demographic groups used here, where a CI is given, it represents the most appropriate CI for the data. Hence direct comparison of the NSH data with other datasets is possible with a high degree of statistical appropriateness.

1.4 Summary and conclusions

This chapter has provided the necessary brief introduction to the terms and concepts behind the formulation of the NSH. In particular the important distinction between hearing impairment, disability and handicap has been made. The types of statistical summary data to be found in this reference book for the NSH have been discussed together with their anticipated and potential uses.

---

[3] Researchers who might wish to use the data for case-control studies beyond the scope of these examples should contact the author.

The prevalence of hearing impairment data give a full description of the extent to which different types and severities of hearing impairment occur in the population. These prevalences provide a reliable, fairly objective, surrogate measure of the extent of hearing disabilities in the population. This can be used to plan services for adults with hearing disability in each health district in the UK. Provision of hearing aids over the last 15 years has not changed (Davis, 1993). Health gain targets that aimed to increase the provision of hearing aids in certain age and severity of impairment bands can be examined by using the prevalence (Chapter 3) and hearing aid provision (Chapter 9) data given in this book in a way similar to that suggested by Mark Haggard in his foreword.

The distribution of hearing impairment in the population will be useful as a guide to deviations from the expected hearing thresholds for individual patients. In addition to this clinical use there is an important role as a statistical control group in comparing data from the standard population used in the NSH to groups of patients with specific characteristics.

Chapter 2

Methodology of Study

2.0 Scope and introduction

This chapter outlines the main objectives of the National Study of Hearing, and the methodologies involved in selecting and testing the subjects in order to achieve those objectives. The study was carried out in two stages according to double-sample survey methodology. The first stage involved a simple random selection of individuals for the first stage postal screening questionnaire; the second stage involved a random drawing of individuals from a population-inclusive-set of subgroups defined by response at the first stage. These second-stage individuals were invited into our clinics. The main results reported in this book are based on 2708 of those individuals, between 18 and 80 years of age, who attended the clinics. Stratum weights used in the study minimise biases due to non-response and non-attendance in respect of a variety of hearing impairment and disability measures. Both internal and external validity of the sampling procedures and strata weights are investigated. These investigations show that there are no systematic biases in population prevalence estimates due to subject selection, non-response and non-attendance for a range of measures of hearing impairment and self-reported disability.

2.1 Aims of the study

The NSH was a multi-purpose, multi-disciplinary investigation of hearing disorders in the adult population of Great Britain. The nine main aims constraining its design were:

1) To estimate the prevalence of mild-moderate hearing impairments and disabilities among the adult population of Great Britain.

2) To provide population control data on the distribution hearing thresholds from an appropriate randomised sample of the population.

3) To quantify the extent to which demographic factors are associated with the prevalence of hearing impairments in the population.

4) To quantify the extent to which noise immission, from an individual's occupation or use of guns, is associated with the prevalence of hearing impairments and with the distribution of hearing threshold levels at individual frequencies, controlling for demographic factors.

5) To quantify the extent to which other proposed factors affect the prevalence of hearing impairments, controlling for demographic and noise immission factors.

6) To estimate the prevalence of different forms and types of tinnitus in the population of Great Britain, and to investigate their relationship to hearing impairment.

7) To estimate the prevalence of various ear pathologies and conditions in the population, and quantify the factors that may affect those prevalences.

8) To examine the relationships between the different indicators of hearing impairment, disability and handicap in a general population.

9) To document the uptake of services for hearing impairment, as a function of demographic and clinical variables.

This book offers data relevant to aims 1, 2, 3, 6, 7, 8 and 9. It would be impossible to design a single study that is near-optimal for achieving all nine aims at a given cost. The most important aim was to obtain an estimate of prevalences of differing degrees of hearing impairment in the mild-moderate region of severity (i.e. 25 to 55 dB HL), both overall in the population and then for each gender and age-group combination (using the six age bands 18-30, 31-40, 41-50, 51-60, 61-70, 71-80). The survey design was therefore optimised to give overall prevalence estimates at the second stage with 95% CI of about ± 1% for prevalences in the region of 15-20% and ± 0.5% for prevalences in the region of 3-5%.

The ability to reconcile the wide variety of further aims was achieved by phasing the study over a number of discrete time intervals, and by grafting further sub-studies onto the main study. Uniformity was retained in respect of a core content of the study (e.g. hearing threshold determinations, otological examinations). Flexibility to incorporate new methods, respond to developments in the literature, and to achieve the multiple objectives, was obtained by changing non-core material (e.g. frequency resolution measures, disability measures) and by adding specialised substudies (e.g. longitudinal studies, tinnitus natural history, psychoacoustics). This 'core-plus' approach enabled the remaining aims to be achieved more cost-effectively than with say half-a-dozen separate studies.

The study was not designed to assess (1) the numbers of severely/profoundly hearing-impaired people in Great Britain, (2) the number or prevalence of the prelingually 'deaf' in Great Britain, or (3) the efficacy or efficiency of audiology services in Great Britain. Furthermore, as the study is cross-sectional it does not necessarily shed direct light on the natural history of hearing impairment, disability or tinnitus, except where these issues were directly addressed in the data gathered. As regards those of presumed aetiological factors (e.g. noise immission, childhood ear disease) those groups who are substantially more at risk of hearing impairment than others can be usefully identified. However the types of variables documented and the nature of control exercised was not such as to permit the identification of single or even probable combinations of causes for individuals' impairments.

2.2 Design of the main study

2.2.1 Sample frame

The main considerations that influenced the choice of sample frame were the study aims outlined above, and in particular the first aim of obtaining an accurate estimation of mild-moderate adult hearing impairment prevalence. In addition to these scientific considerations, four major practical and clinical realities influenced the overall form of the study.

The first of these was consequent upon our main aim which particularly specified measurement of the impairment domain. The most important measure of impairment remains the pure tone hearing threshold. In order to execute this in line with accepted audiological practice the subjects needed to attend a well-equipped and well-staffed audiology clinic. Attendance at such clinics inevitably reduces overall response rate through lower attendance rates. Therefore, a domiciliary comparison group was planned, to monitor any bias that this might introduce.

Secondly, four audiology research sections were established, staffed by MRC and local health service personnel (e.g. audiological scientists, otologist, audiological technicians and support staff) in Cardiff (at the formerly MRC-IHR (Welsh Section), University Hospital of Wales), in Glasgow (at MRC-IHR (Scottish Section) Southern General Hospital & latterly at Glasgow Royal Infirmary), in Nottingham (at MRC-IHR, Nottingham Clinical Outstation, General Hospital) and in Southampton (at MRC-IHR, Southampton Clinical Outstation, Royal South Hants Hospital). These facilities were set up with objectives that included, but were wider than, the clinical investigations needed for this epidemiological study thereby constraining the number and location. Therefore the sample frames were chosen from areas close to these clinics. The electoral register in the constituencies for each of the four cities was taken as the sample frame, and individuals were selected at random from the most up-to-date register.

Thirdly, it was not possible to conduct the study to the desired level of accuracy with the resources available in the four clinics within a single one year period. We therefore decided to pursue the study in separate phases. A common core of tests was maintained over phases, but allowing some variation enabled a greater degree of flexibility to learn from interim results and include questions and procedures from current literature. However, this variation has added substantially to the problems of data integrity and uniformity in the data, hence to the statistical sophistication needed to analyse and comprehend the study.

Fourthly, in a simple random sample of the adult population there are inevitably few 'clinically interesting' cases from the otological point of view. The sampling procedure was tailored so that as many 'clinically interesting' subjects as possible from the tail of the impairment distribution were selected to attend the clinics, providing a diversity in, and refinements to, the other audiological and otological aims of the study.

Taking these considerations into account, it was decided to undertake two related studies (1) a main study based at the four clinical outstations, and (2) a smaller study (the 'household study'). The latter would use a sample frame that was nationally representative of Great Britain to check for biases in terms of the lack of representativeness in the main study. This latter requirement was met by using the register of postcode addresses in Great Britain as the sample frame.

2.2.2 Study designs

The essential elements of the study design have been detailed by Davis (1989). The main study is essentially a two-stage sample survey. The sample frames for the first-stage of the main study had the two dimensions of time and location as shown in Table 2.1. Each of the X's denotes an independent

sample drawn from the electoral register, and each sample was planned to have equal weight in calculating prevalences and their variances. This gives substantial initial advantages, because in principle each X could have its own specific design, or subject selection strategy. Providing it was capable of making an unbiased estimate of a particular prevalence it could be pooled with the other X's. Thus, for example, individual clinical studies could be mounted in parallel with the main study at any outstation, and specific substudies could be run in a particular phase, but not in the others.

Table 2.1 The sample frame design for the main study using individuals sampled from the electoral register

Years		1980-81	1981-82	1984-86
Phase		(1)	(2)	(3)
Place (clinical outstation)				
	Cardiff	X	X	X
	Glasgow	X	X	X
	Nottingham	X	X	X
	Southampton	X	X	X

The sample frame for the first stage of the Great Britain sample was taken from the postcode address register. A two-way stratification of the register was made in 1982 by CACI according to regional health authority and the ACORN index (Webber, 1979; CACI, 1982). One hundred and ninety postcode districts and then thirty-five addresses from each district were randomly selected. The first-stage questionnaires were addressed to households rather than individuals. In this way the overall sample frame was designed to be as representative as possible of the adult population living at private addresses in Great Britain, in order to estimate the prevalence of mild-to-moderate hearing impairment. Further details of the sample design, response rates, survey procedure and adjustment for sampling effects are given by Davis (1989). This chapter focuses upon those important aspects of the main study needed to understand the methods used to calculate the results given in following chapters.

2.2.3 Sampling stages in the main study

Questionnaires were sent to 48,313 people on the electoral registers (aged 17+) in the first stage. Clinical examination, interview and audiological assessments were carried out on 2910 people at the second stage of the study. Strategies were used to cope with non-response (first stage) and non-attendance (second stage), including sending at least two reminders, telephoning individuals, and visiting peoples' homes.

2.2.4 Second-stage sample stratification

The second stage sample were randomly selected from a fully inclusive set of natural groupings given by the response to the questionnaire. These included age-group (18-50, 51+), hearing aid possession, and other hearing-related factors (e.g. reported hearing difficulty in noise, tinnitus most of the time) specific to each phase and outstation (Davis, 1989).

Two main problems arose from this prior stratification of the second-stage sample. Firstly, the male-to-female ratio, for each age-group (in approximately ten year age-bands) was not preserved from the first to the second stage. Secondly, the proportions of people in each age band were not preserved from the first to the second stage. These problems were greatest for those over 80 years of age. The extent to which these inadequacies can be explained as differential attendance rates at the second stage is unclear. These biases in projecting the sample to the population were firstly side-stepped by (a) omitting the over 80 age-group from the analyses and (b) post-stratifying (Holt and Smith, 1979) the sample by gender and age-group within the two age-groups already used to pre-stratify the sample. This in turn led to further analytical complexities and limitation.

Maintaining the total prior design (see Table 2.1) produced a very large number of strata (144 arising from phase, outstation, gender and age-group). This required 25% of the cases to be aggregated (or allocated) across strata whilst retaining the Primary Sampling Unit (PSU) structure (phase by outstation). If a subset of the full design was selected (e.g. female non-manual occupations under 50) then the data analysis can become unstable because of the sparse design matrix. These problems were tackled by aggregating strata rather than individuals in a planned manner, and in particular by aggregating over phases of the study, such that of the original PSUs given in Table 2.1 only the four outstations gave independent estimates. Table 1 of Davis (1989) gives details of the six reply strata used in the analysis of the second stage results. That publication demonstrated that no systematic bias in prevalence estimation is introduced by this post-stratification, with its concomitant aggregation of strata and realignment of pre-stratification boundaries. This statement applies to most hearing impairment and disability measures collected at the second stage. The variances obtained for means and prevalences are probably a slight overestimate of the true variances. For other indices of hearing disorders, such as ear pathology, no firm statement can be made concerning whether the procedure introduces bias or not. However, for indices highly correlated with hearing impairment, reported hearing disability, hearing aid possession or tinnitus, there are very probably no serious biases. Other possible sources of bias are discussed below.

2.3 Preliminary results pertinent to the design of the main study

Haggard (1981) and MRC Institute of Hearing Research (1981) outlined the constraints against which the main study was planned. Initial estimates from these pilot studies suggested that a figure approaching 32% (CI 27-37%) of the adult population had a hearing impairment as measured by an average pure tone threshold in their better ear over the frequencies 0.5, 1, 2, 4 kHz (BEA) of 20 dB HL or greater (Haggard, 1980), and 20% (CI 15-25%) for a BEA of 25 dB HL or greater (MRC-IHR,

1981). Those pilot data were used to plan the initial size of the study, as were the response rates, which were in the region of 80% for the first stage and 50-60% for the second stage. The attendance rate was found to be influenced by offering a standard allowance for participation in the clinical assessment. This incentive was therefore offered in the main study (Davis, 1989; Haggard, 1981).

Whilst no large discrepancy was found between attenders and non-attenders in the pilot study, it was important to establish, and be able to allow for, any attendance bias. Thus, hearing threshold measurements were obtained from 847 non-attenders in the main study who were invited to take part in the clinical study, but either declined or did not reply to the invitation. Hearing thresholds at 0.5, 1, 2 and 4 kHz were obtained during visits to these peoples' homes.

The questionnaires used in the first stage of the main study were partly based on the experience of Schein and Delk (1974) and Noble (1978). These were modified in the light of the principal investigators' interests and experience, and underwent continued evolution and improvement during various phases of the study. An example of the postal questionnaires used in phase 3 of the main study is shown in the Appendix 1. Phase 3 had the most comprehensive set of screening questions. It was divided into five sections: section A asked about hearing ability, section B about tinnitus, section C about use of services, section D gathered biographical information, and section E sought to gather information concerning the disadvantages (or handicap) to which a hearing problem may have given rise.

The overall results for the screening questionnaires and the numbers in each cell are given in Chapter 9. Two points must be made about that chapter. Firstly, because of the limitations of the sample frame (see Davis, 1989), the responses for each question are weighted and only those replies with valid answers to particular questions are counted. Secondly, some attempt has been made here to purify the data by excluding inconsistent responses, for example if a person answers that they are "totally deaf in both ears", but "has no difficulty hearing a whisper". The tinnitus data have been extensively checked for inconsistent responses (e.g. if an individual replied they have no noises in their head or ears, but they report that they are greatly annoyed by tinnitus). Only the raw data are given here, with some imputation, as a guide to the responses given to the screening questionnaire that were used to stratify the sample for the second stage of the study. These first-stage results are used to demonstrate how the weighting scheme for the second stage is derived.

2.4 Audiological and otological procedures

Appendices 2 and 3 give the protocols and procedures used in this study for obtaining pure-tone thresholds, otoadmittance measurements, and calibrating equipment. Great attention was paid to maintaining uniform calibration and procedures over the different sites (and in addition between individuals on the same site) and between different phases of the study.

2.4.1 Audiological assessments

All auditory testing in the clinics was performed in sound-attenuating booths, conforming to ISO 8253-1 (1989) for measurement of air conduction thresholds down to 0 dB with an error not exceeding 2 dB due to penetration of extraneous noise. This standard was adequate for the majority of the study aims. More stringent criteria would have been necessary to plot accurately the lower (better) end of the distribution for young subjects with no pathology but this was not an affordable aim of the study. A rigorous calibration protocol was maintained throughout the study, based on BS 2497 (Part 2, 1969; equivalent to ISO 389, 1991) for air conduction and BS 2497 (Part 4, 1972) for bone conduction. Calibration was conducted monthly, and corrections applied to the nearest 1 dB. The method of manual audiometry, documented by previous IHR research (Tyler and Wood, 1980), recommended by the British Society of Audiology/British Association of Otolaryngologists (BSA/BAOL, 1981) was used for all threshold determinations and recorded to the nearest 5 dB. Air conduction thresholds (AC) were obtained at 0.25, 0.5, 1, 2, 3, 4, 6, 8, 12 kHz. Bone conduction thresholds (BC) were obtained in phases 1 and 2 on all subjects. Due to time constraints in phase 3, only those failing an acoustic reflex check proceeded to full BC. BC were only obtained at 0.5, 1, 2 kHz. To investigate the role of conductive pathologies in those with relatively good hearing (e.g. -10 to +15 dB HL) masked bone conduction would have had to be carried out on all subjects, for which we had no time. All home testing (non-attenders and the household study) used Kamplex TA155 portable audiometers with TDH39 earphones mounted in MX-41/AR cushions and fitted with Amplivox Audiocups.

All thresholds were obtained before any acoustically obstructing wax was removed and measurements were repeated afterwards. The data reported here are those before removal. The criteria for acceptability (see Appendix 2) were applied by the audiological technician and/or audiological scientist at the time of testing. Of the 2708 subjects who were aged 17-80 at the second stage, 29 (0.73% of the population, 1.11% of the second-stage sample) gave audiograms that were not acceptable. Acceptability did not vary with gender or age, but did vary with occupational group; the 'manual' occupational group gave more unacceptable audiograms (chi-sq = 5.3, df = 1, p <0.02).

Of the 2679 subjects with acceptable audiograms, a complete audiogram (in the range 0.25 kHz to 8 kHz) was obtained on both ears for 2578 (97.64% of the population, 95.16% of the sample). This proportion did not change with gender or occupational group, but did change with age. Up to 4% of the 61-70 age-group had incomplete audiograms, compared to about 1% for the 18-40 age-group. However, for those with acceptable audiograms, 98.0% of the population had a complete audiogram. When concentrating solely on the better ear, over the frequency range 0.5, 1, 2, 4 kHz, 99.7% of the population (99.4% of the sample) had acceptable measured thresholds, and over the range 4, 6 8 kHz, 99.5% of the population (99.2% of the sample) had acceptable measured thresholds. There were 16 people with 'acceptable' audiograms but no better ear average threshold over the range 0.5, 1, 2, 4 kHz. Of these 16 people (0.38% of the population), four had no audiometric data at any of these frequencies on either ear. For the remaining 12 individuals, from the data available, it is possible to combine the measured thresholds on the left and the right ear in the region of 0.25 to 4 kHz to produce an indicator for the better ear average threshold over the 0.5 to 4 kHz range. This shows that for the remaining 12,

four would probably be in the range up to 25 dB HL, three in the range 25 to 44 dB HL and five in the range 65 dB HL and greater. In terms of population estimates these figures would represent about 0.12% for 25 dB HL and greater, and 0.07% for 65 dB HL and greater. In other words, the biases introduced by incomplete data are about two orders of magnitude smaller than the absolute estimates of prevalence (as provided from the pilot studies and shown for the main study in Chapter 4) at 25 dB, and about one order of magnitude smaller at 65 dB.

For measurements on the worse ear, there are considerably more missing data. However, over the mid-frequency range there are only 30 missing averages, which is 0.60% of the population (1.12% of the sample with acceptable audiograms). Four (0.12% of the population) of the 30 would probably have been below 25 dB HL, three (0.03%) between 25 and 44 dB HL, six (0.08%) between 45 and 64 dB HL, 13 (0.25%) in excess of 65 dB HL, with four (0.11%) completely missing. The cumulative effect of the missing data can be seen to be quite small for most purposes.

For all the analyses reported here the better ear was defined in the following way: (1) if the average threshold over the frequencies 0.5, 1, 2, and 4 kHz was better in one ear or the other, (2) if there was no difference on (1) then a procedure was used to determined which ear was the 'better' ear as follows: the sum of all hearing thresholds was obtained for each ear and the ear with the lowest sum was assigned as the better ear. No subjects were tied after this procedure.

2.4.2 Audiological and otological assessment

When the second-stage subjects had indicated their willingness to attend one of our clinics, they were sent an appointment card, together with a clinical questionnaire which they were asked to fill in and bring to their appointment. Subjects were thus primed about the questions the otologists would be asking them by this initial questionnaire. They were also instructed to bring any medication they might be taking, together with any spectacles or hearing aids used. A careful note was made of the order (and time) for each test. At the end of each subject session, the otologist/audiological scientist conducted a normal clinical de-briefing/counselling of the subject with respect to their hearing and related matters (and if any other aspect of health was of concern the subject was encouraged to see their GP). The subject's GP was informed of the audiological outcome as a matter of course, provided the subject did not object. Appropriate ethical committee approval (as well as MRC in-house approval) was sought and support was also gained from the Community Health Councils (CHC) in the districts in which the main study was carried out. The BMA ethical committee also gave approval.

2.5 Bias reduction and data analysis

There are many ways in which bias can enter the estimation of prevalences or means in a two-stage survey; five are briefly considered here.

First, the sample frame may not be appropriate for the task in hand. For instance, it is well known that the electoral register has several flaws as a sample frame (e.g. Thomas, 1990). Our particular use of the electoral register in the four outstation cities may also be perceived as a source of bias towards a

predominantly urban population. The household study was performed to allow correction for this if necessary. Davis (1989) showed that in terms of prevalence of hearing impairment there were no gross biases due to the sample frame (in the 18-80 age-group). Deficiencies in the electoral register sample frame due to bad selection procedures in Strathclyde (which were not under our control, see Davis, 1989) were dealt with by applying a post-stratification weighting structure to the first-stage data. In any case, this only affects the sample ≥ 60 years in one cell of the study (see Table 2.1). It is important that the first-stage data be free from serious bias as the strata weights used (both *a priori* and *posteriori*) for the second-stage estimations are derived from it.

Secondly, non-response at the first-stage, might lead to bias on the screening questionnaire. An overall response rate of 80% after three mailings is a reasonable return from the electoral register between 1980 and 1986, given that some of the areas, in particular Glasgow, have a very high level of inter-mobility. Non-responders were followed up to help reduce any first-stage non-response bias. There are probably no large errors for reported mild-moderate hearing difficulties, Davis (1989). Tinnitus however may pose different problems; relative to the first-stage, it is 'under-reported' in the follow-up study and in the household study. At present no direct means of validation is available to determine whether this means that the main study somehow overestimates the prevalence of tinnitus, or whether the household study underestimates it. Davis (1989) suggests that, on evidence of the household study, it is the latter.

Thirdly, selective non-attendance at the second stage might produce biased estimates of impairment or disability prevalence. Fit young people may not want to take part in an epidemiological study and they change address more frequently. Older people are more contactable but have more difficulty in attending. To facilitate access, some clinic sessions were scheduled for weekend or evening sessions. Generally, regardless of age, it was also thought that there might be a strong tendency for those with hearing problems in the family to attend, and those without not to attend.

Some types of bias can be adequately corrected by post-stratification (Smith, 1990). The beneficial possibilities of post-stratification, to remove coverage errors and non-attendance, outlined in the previous paragraph, depend crucially on the relationship between the dependent variables of interest and the post-stratification scheme. The aim of post-stratification in this sense is to render the non-response scheme uninformative. In other words, the non-response rate needs to be independent of the outcome (the dependent variables) conditional on the post-stratification variables. Davis (1989, 1990) shows this to be true for a number of hearing impairment variables in this survey design. This important result stems primarily from the follow-up of the 857 non-attenders, which showed extremely good agreement with the prevalence estimates from the main study and did not substantiate any of the possible sources of bias outlined above. Therefore, it is possible to use the main study alone, with the weights in each stratum derived from the stratification scheme, for the estimation of most hearing impairment and disability measures, and to be fairly certain that there are no gross attendance biases. Without the use of the weights obtained by post-stratification, the sample statistics would not only be unrepresentative of the responding and attending population, but could also contain non-attendance bias as well.

Fourthly, measurement biases of two types may occur. The first lies in the patterning of missing data. We have already shown that, for most estimation purposes, missing data statistics derived from the

better and worse ear mid-frequency average are highly related to severity of impairment, but not to age-group, gender or occupational group. However, the amount of error involved is usually small. In most cases, we have ensured that the inferences drawn from the data are not just a reflection of the missing data pattern, and where there are large amounts of missing data the inferences are appropriately scaled down. The second type of measurement bias arises from sources of study variation such as the instructions given, the question wording, the particular procedure used (e.g. order effects in questions, and in audiometry where a particular ordering is experienced by a large number of people), and so on. The protocols and appendices provide the context for the questions and for the tests carried out for each subject. It is possible that an inexplicable or unexpected result has occurred because of such a sequence effect, but this is unlikely in the results reported here.

Fifthly, there are subject (self-) selection biases. This poses no problem at all where the basis of selection roughly coincides with the boundary of a stratification factor e.g. ten-year age-band, gender, outstation, reply-strata. Thus, for instance, an analysis of female hearing aid possessors aged 50-80 would in all likelihood not be biased in respect of the intended target population, provided that the appropriate stratum weights were utilised. However, great care has to be taken where the selection criteria are not orthogonal to the stratification criteria, e.g. over occupational group, over hearing impairment category, over noise exposure exclusions.

These are the five main considerations that we have taken into account in the survey design and in the analysis strategy for particular estimates and models. Broader issues, such as the cross validation of the survey methodology (Davis, 1990) and the reliability and content validity of the questionnaires and procedures are not addressed here.

2.6 Estimation and analytical procedures

Only procedures for the main study are covered in this section. Davis (1989) covers the procedures used in the Household study.

2.6.1 First-stage estimation

The prevalences and means from the first-stage screening questionnaire were calculated separately for each PSU as from a random sample, with post-stratification weights being applied to overcome inadequacies of the sampling frame. As these inadequacies only apply to two out of the twelve PSUs, and then only to the over 60 age-group, for most purposes the estimation of prevalences, means and associated variances was similar to that from a simple random sample. There are two methods by which the statistics from each PSU might then be aggregated. In the first, equal weight could be given to each PSU, and in the second, a weight proportional to the number of respondents in each PSU could be assigned. Approximately equal numbers responded from each outstation (24.6, 26.3, 22.6, 25.5%, respectively for Cardiff, Glasgow, Nottingham and Southampton), but phase 3 had more than the other two phases combined (22.6, 22.5, 52.9% respectively for phases 1, 2 and 3). A compromise solution was reached, such that the weighting structure dependent on responses to the first stage was derived

from outstation-specific prevalences. Given the decision in the design to amalgamate over phase (see section 2.2), this factor was not taken into account in deriving the weights. However, in calculating general prevalences all PSUs have been combined, giving each phase and outstation a post-stratified weight proportional to the number of respondents. There appear to be no systematic biases in this aggregation, which enables simple statistical estimation and modelling to be carried out with standard packages (e.g. P-STAT, GLIM, SPSS,).

2.6.2 Second-stage estimation

Second-stage estimation of population prevalence and means was achieved using the standard methods for stratified, and post-stratified samples (e.g. Cochran, 1977; Kish 1965; O'Muircheartaigh, 1977; Holt and Smith, 1979; Bartholomew, 1990). These methods were implemented using P-STAT macros (Buhler and Buhler, 1989) to derive appropriate means, variances and 95% CI. The estimation was carried out in three steps. In the first step the proportion of the population in each of the strata was determined from the first-stage responders over outstation, age group (18-30, 31-40, 41-50, 51-60, 61-70, 71-80), gender and reply stratum (see Table 1, Davis, 1989). The second step was to derive means and appropriate variances (Bartholomew, 1990). The third step was to combine the means and variances, maintaining an equal weight for each outstation (respective second-stage Ns are 719, 689, 710 and 590 for Cardiff, Glasgow, Nottingham and Southampton respectively). These steps can be used in a large number of situations where estimators are required. The flexibility stems from assignment of the design matrix weights at a supra-individual level. Thus, selection criteria for subjects and missing data patterns could be easily accommodated. This flexibility, however, might occasionally lead to extremely sparse and unrepresentative subsets, and therefore indices of representativeness were developed to monitor the estimators produced by the programme. The estimates presented here have all been checked to ensure that, where population estimates are required, the representativeness indices do not fall unacceptably low.

2.6.3 A note on case-weights

As most of the statistical modelling for these data (e.g. Davis, 1989; Lutman and Davis, 1994) has been conducted at the level of the individual, a weight was calculated for each individual in the second-stage sample. The approach that was taken in assigning weights was that each individual in a particular stratum had equal weight within that stratum. The overall weight for that stratum was therefore divided equally among the individuals in that stratum, and adjusted so that the overall sum of the weights was the same as the number of subjects in the whole sample (2708). These individual weights can be utilised by GLIM and other packages to provide unbiased estimates of means and prevalences in the case where there is no missing data. The results using this method are shown in Chapters 9 and 10 where the results from the hearing questionnaire and the postal questionnaire are looked at in terms of average impairment measures. Also factor effects can be quantified, and their standard error used to estimate approximate CI. The change in likelihood has been used in most cases to estimate the statistical significance of a particular factor or interaction. At this stage it is worth pointing out that if there are

missing data in particular strata then the full sample prevalence estimation and the GLIM/SPSS/P-STAT estimation sample proportion derived using with individual case-weights will be different.

2.7 Comparison of main study with the domiciliary follow-up and the household study

In this section, prior to the presentation of the main results there is a comparison of the main study data with the follow-up of the non-attenders in that study and with the people who took part in the household study. The major role of the domicilary follow-up of non-attenders was to check for non-attendance bias. Davis (1989) has shown that there is no great bias in the most important measures. In this section a more detailed descriptive comparison is presented.

Table 2.2 presents the number of people aged 18-80 in each part of the study. Overall there were 4029 people on whom we attempted to measure the audiogram. The 2708 people in the main study were contrasted with the 1321 who had their audiogram attempted in their own home. If the prevalence of hearing impairment in the non-attenders or in the household study was much less than in the main study, then a serious attendance bias would be present and prevalence over-estimated.

Table 2.2 The numbers with attempted audiometry in each part of the NSH

		Age Group						
**Gender**	**Sample**	**18-30**	**31-40**	**41-50**	**51-60**	**61-70**	**71-80**	**Overall Totals**
**Female**	**Clinic**	228	194	251	347	247	147	1414
	**Domiciliary**	57	49	74	92	62	60	394
	**Household**	61	75	46	71	46	29	328
**Male**	**Clinic**	178	143	207	348	288	130	1294
	**Domiciliary**	60	45	45	76	49	32	307
	**Household**	54	55	44	60	56	23	292
**Total N**		**638**	**561**	**667**	**994**	**748**	**421**	**4029**

Table 2.3 shows the estimates for the prevalence of hearing impairment from each study at a number of different severities. Figure 2.1 summarises these data. The domicilary non-attenders, for the sample in the age range 18-80 when corrected for age, sex and response stratum gave a higher prevalence estimate than the main study subjects. The household study gave slightly lower estimates. This was the case for both the mild and moderate degrees of hearing impairment shown in Figure 2.1. This general pattern is similar over all ages and sexes, with only a few deviations, e.g. in the male 41-50 age group where the numbers were quite small.

Table 2.3 The prevalence of hearing impairment in the better ear using the 0.5, 1, 2, 4 kHz average hearing thresholds in five severity bands, as function of the sample

Sample	Age Group	Gender	Prevalence (%) of hearing impairment levels ≥25	≥30	≥35	≥45	≥55
Clinic	18-80	Overall	16.1	11.2	8.3	3.8	2.1
Domiciliary	18-80	Overall	21.3	14.0	9.2	4.3	1.7
Household	18-80	Overall	13.5	9.0	5.5	2.7	1.6
Clinic	18-80	Female	14.6	10.3	7.8	3.7	1.9
Domiciliary	18-80	Female	18.6	12.7	8.9	4.4	1.4
Household	18-80	Female	13.6	8.8	5.7	3.3	2.3
Clinic	18-80	Male	17.9	12.2	8.8	4.0	2.3
Domiciliary	18-80	Male	24.7	15.8	9.5	4.2	2.1
Household	18-80	Male	13.4	9.1	5.2	2.1	0.9
Clinic	18-30	Female	2.9	1.2	0.6	0.3	0.0
Domiciliary	18-30	Female	1.8	0.7	0.0	0.0	0.0
Household	18-30	Female	2.3	0.3	0.2	0.2	0.2
Clinic	18-30	Male	0.6	0.1	0.1	0.1	0.1
Domiciliary	18-30	Male	1.9	1.7	1.7	1.5	0.0
Household	18-30	Male	1.7	0.3	0.3	0.0	0.0
Clinic	31-40	Female	2.9	2.0	1.2	0.8	0.4
Domiciliary	31-40	Female	6.2	0.0	0.0	0.0	0.0
Household	31-40	Female	6.4	5.0	0.6	0.6	0.0
Clinic	31-40	Male	2.5	2.5	1.6	1.3	1.1
Domiciliary	31-40	Male	10.4	5.2	5.2	0.0	0.0
Household	31-40	Male	0.0	0.0	0.0	0.0	0.0
Clinic	41-50	Female	7.1	5.2	3.8	1.7	1.0
Domiciliary	41-50	Female	11.7	9.8	6.3	5.3	0.0
Household	41-50	Female	0.3	0.3	0.3	0.3	0.3
Clinic	41-50	Male	9.6	6.9	4.4	1.8	0.5
Domiciliary	41-50	Male	26.1	13.7	9.4	1.1	1.1
Household	41-50	Male	11.0	9.2	2.7	0.0	0.0
Clinic	51-60	Female	12.9	7.9	5.3	2.6	1.5
Domiciliary	51-60	Female	17.2	11.4	5.7	3.1	2.6
Household	51-60	Female	14.5	10.2	6.7	5.0	2.5
Clinic	51-60	Male	24.6	14.1	10.3	5.4	2.8
Domiciliary	51-60	Male	23.3	14.9	7.7	5.5	3.0
Household	51-60	Male	19.4	10.9	4.7	0.8	0.4
Clinic	61-70	Female	28.6	18.6	12.9	5.5	3.3
Domiciliary	61-70	Female	34.0	21.8	16.3	2.8	2.8
Household	61-70	Female	25.2	14.8	3.5	3.5	3.5
Clinic	61-70	Male	44.8	31.3	19.6	9.2	4.8
Domiciliary	61-70	Male	55.5	37.0	15.2	7.5	5.1
Household	61-70	Male	37.0	24.5	20.0	7.7	3.1
Clinic	71-80	Female	55.5	45.5	38.7	18.7	9.1
Domiciliary	71-80	Female	63.1	50.2	39.5	23.4	4.0
Household	71-80	Female	66.2	45.3	44.4	20.0	15.0
Clinic	71-80	Male	67.7	49.2	41.5	15.4	10.2
Domiciliary	71-80	Male	58.7	40.1	31.9	14.9	5.5
Household	71-80	Male	44.5	36.1	17.4	13.4	5.9

Table 2.4 shows the prevalence of hearing impairment that would be estimated if all the studies were combined. These prevalences are very similar whether the three estimates are combined with equal weight to each study or whether weight is given for the actual numbers of people with audiograms in each study. The latter are presented in this table. Inspection of the data shows that the overall average deviates very slightly from the data of the main study. The audiometry that was attempted in people's own homes might be expected to show apparently poorer hearing at low frequencies, where ambient noise would have greatest effect. But this should only affect the people with better hearing, e.g. < 30 dB HL. For this reason, when looking at prevalence of minor hearing impairments, e.g. in the younger groups, and also when looking at estimates of the distribution of hearing threshold levels, the data from the main study should always be preferred. It is difficult to give an overall guide as to what discrepancy between studies might be considered material. This depends very much on the prevalence in each age band and the numbers in each age/study. We are looking primarily for consistency over severity and age group, which is apparent here.

Table 2.4 The combined estimate of the mean and prevalence of hearing impairment in Great Britain, from all three component studies as a function of ear and age group. Hearing impairment is derived from the averaged threshold at 0.5, 1, 2 and 4 kHz

			Prevalence of hearing impairment (%) as a function of Severity (dB HL)				
Ear	Age Group	Mean (dB HL)	≥25	≥30	≥35	≥45	≥55
**Better**	18-80	14.3	16.4	11.2	7.9	3.7	2.0
**Worse**	18-80	19.9	26.6	19.4	14.9	8.8	5.4
**Better**	18-30	6.2	1.8	0.6	0.4	0.2	0.0
**Worse**	18-30	10.2	6.2	4.1	2.2	1.2	0.4
**Better**	31-40	8.8	3.4	2.3	1.3	0.8	0.5
**Worse**	31-40	13.6	11.1	8.3	6.2	2.4	1.5
**Better**	41-50	12.2	9.0	6.4	4.0	1.7	0.6
**Worse**	41-50	18.2	20.7	14.1	10.2	6.1	4.7
**Better**	51-60	17.0	18.4	11.1	7.2	3.8	2.1
**Worse**	51-60	23.6	33.4	22.5	15.9	9.9	6.0
**Better**	61-70	23.4	36.3	24.3	15.3	6.6	3.9
**Worse**	61-70	30.6	52.1	36.9	29.7	17.3	10.3
**Better**	71-80	31.9	60.1	46.1	38.4	17.8	9.1
**Worse**	71-80	39.7	73.1	60.7	51.5	32.5	19.6

Some studies where the reader may wish to compare data with our main study may have used domiciliary audiometry, or audiometry carried out in a 'quiet room' rather than a audiometric booth. To facilitate such comparisons, but also to aid contrast between the studies and to clarify where differences may exist between the studies, a fuller breakdown of the distribution of hearing

impairment is shown for the three component studies in Tables 2.5, 2.6 and 2.7. The Figures 2.1. 2.2a and 2.2b show some of the comparisons between the studies, with Figure 2.1 showing the difference in prevalence at ≥25 and ≥45 dB HL.

Table 2.5 shows the number, mean, standard deviation and median for each age group and gender in the post-stratification design. These are presented as a function of frequency and ear. In addition to the four individual frequencies (which were the only set tested on a domicilary basis) the distribution of the average over 0.5, 1, 2 and 4 kHz (4-frequency average, 4FA) was also calculated. There are quite systematic differences between the three component studies; these occur especially at the lower frequencies as expected. Figures 2.2a and 2.2b, present the median 4FA for women (a) and for men (b). The same pattern with age is shown in all three component studies, but the main study has a substantially lower median in the younger three age groups, but less so in the older three age groups where measurement of thresholds at the respective degree of impairment are less likely to be influenced by ambient noise, again as expected.

Table 2.6 shows the five main percentiles usually quoted (10th, 25th, 50th, 75th and 90th) of the distribution of HL. Table 2.7 shows the differences between the main clinic study (C) and the domiciliary study (D), and also between the clinic study (C) and the household study (H). A negative number indicates that the clinic measurement is less impaired, i.e. better hearing. Together these show that the differences are fairly uniform across the distribution. The non-attenders (D) seem to fall between 3-5 dB worse at the 4FA, but only an average of 1.7 dB worse at 2 and 4 kHz on the median. The differences between the household study (H) and the main study were much smaller, averaging under 1 dB. There were exceptions in the highest age group 71-80 and also in the tails of the distribution (90th percentile). This would be expected with the smaller numbers in the D and H samples.

In summary, the expected differences between the three components in the study were found at the lower frequencies of 0.5 and 1 kHz. Comparative use of the present data with young subjects, or hearing in the normal range at low frequencies, should bear in mind the probable compression of differences due to the flow effect of ambient noise. In the domiciliary non-attenders group a difference was maintained for the higher frequencies, again in the direction of worse hearing. This was not seen in the household sample where the distributions were very similar. In the extremes of age and of the HL distribution there were some large differences attributable to the small size of the samples.

2.8 Summary and conclusions

The two stage sampling design for the studies has been presented. The main data presented in the following chapters derive from the second stage of the main study where 2708 people were assessed for the degree and type of their hearing impairment. The strategies for the essential stratification of the sample were outlined, and the important difference between sample and population statistics

have been clarified. All of the following chapters contain population estimates. Where necessary the number of people in a particular group are the sample N.

The strategies for assessing the bias that might arise from (a) non-response and non-attendance and (b) the sampling around four major audiometric testing facilities have been assessed and found negligible. Data was presented in Tables 2.3-2.7 and in Figures 1 and 2 that show the extent of any bias that might exist. From these data we infer that the non-attenders as a group did not have better hearing than the attenders. In fact they may be worse. There were some differences between the broader geographical sample in the household study and the main study. These differences were greatest in the 61-80 year old men, who had better hearing in the household study. As this was based on 79 men compared with 418 in the main study, and as the non-attenders, with 81 men, showed a very good agreement in the main study, this difference should not be over-interpreted, particularly as the women in the household study also showed a reasonable agreement with the main study.

We can therefore assert that the hearing threshold data from the main study do not contain any of biases of the type outlined above, and that if anything they possibly slightly underestimate the real size of the problem of hearing impairment in the population, due to the higher prevalences found in the non-attenders which persisted even up to ≥ 45 dB HL.

Fig 2.1 Overall prevalence of hearing impairment, as a function of gender, severity and study group, for the better ear hearing thresholds averaged over 0.5, 1, 2, 4kHz

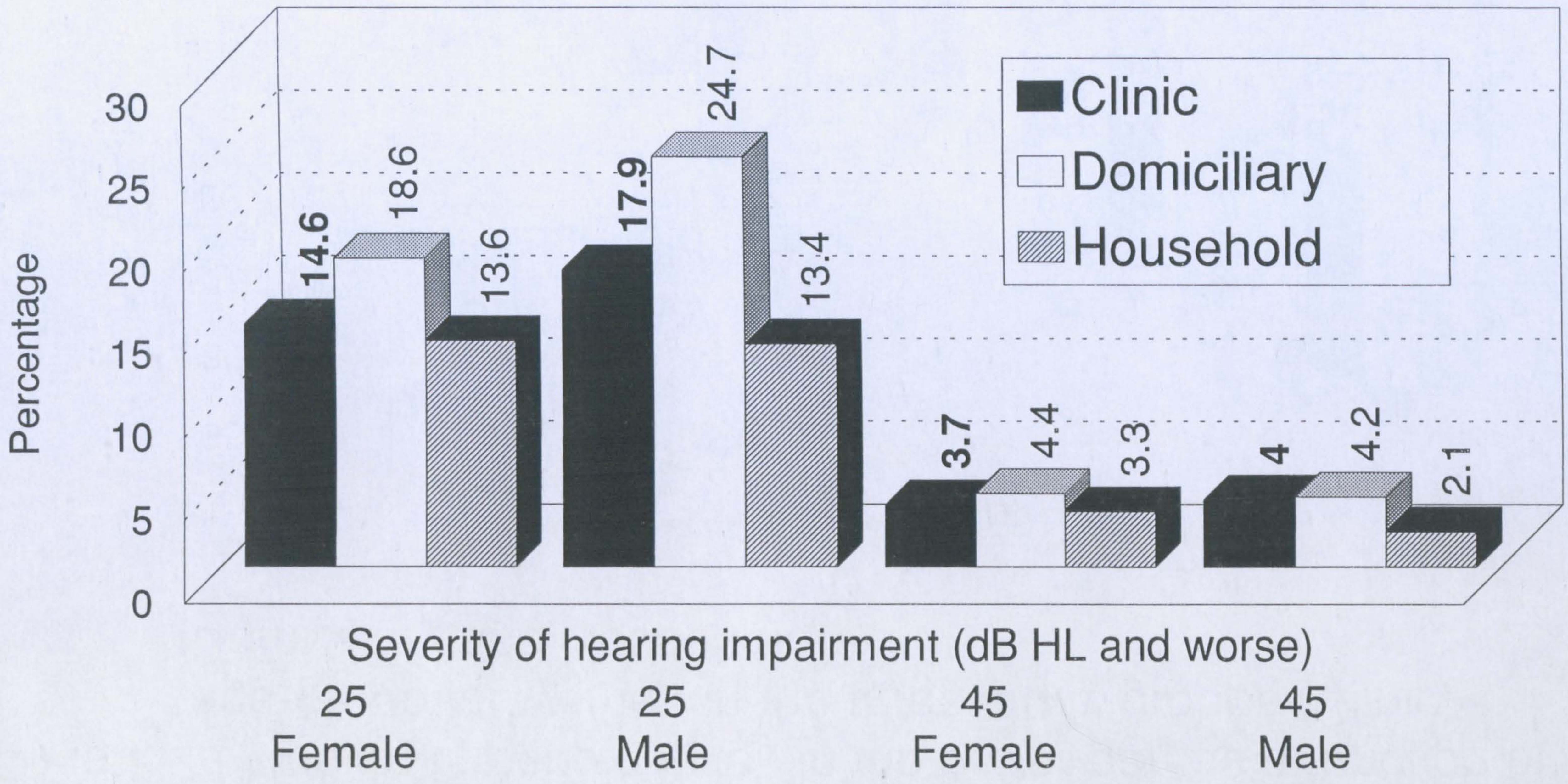

Fig 2.2a Median threshold level, in the better ear, as a function of age-group for women in the three study groups (Clinic, Domiciliary and Household)

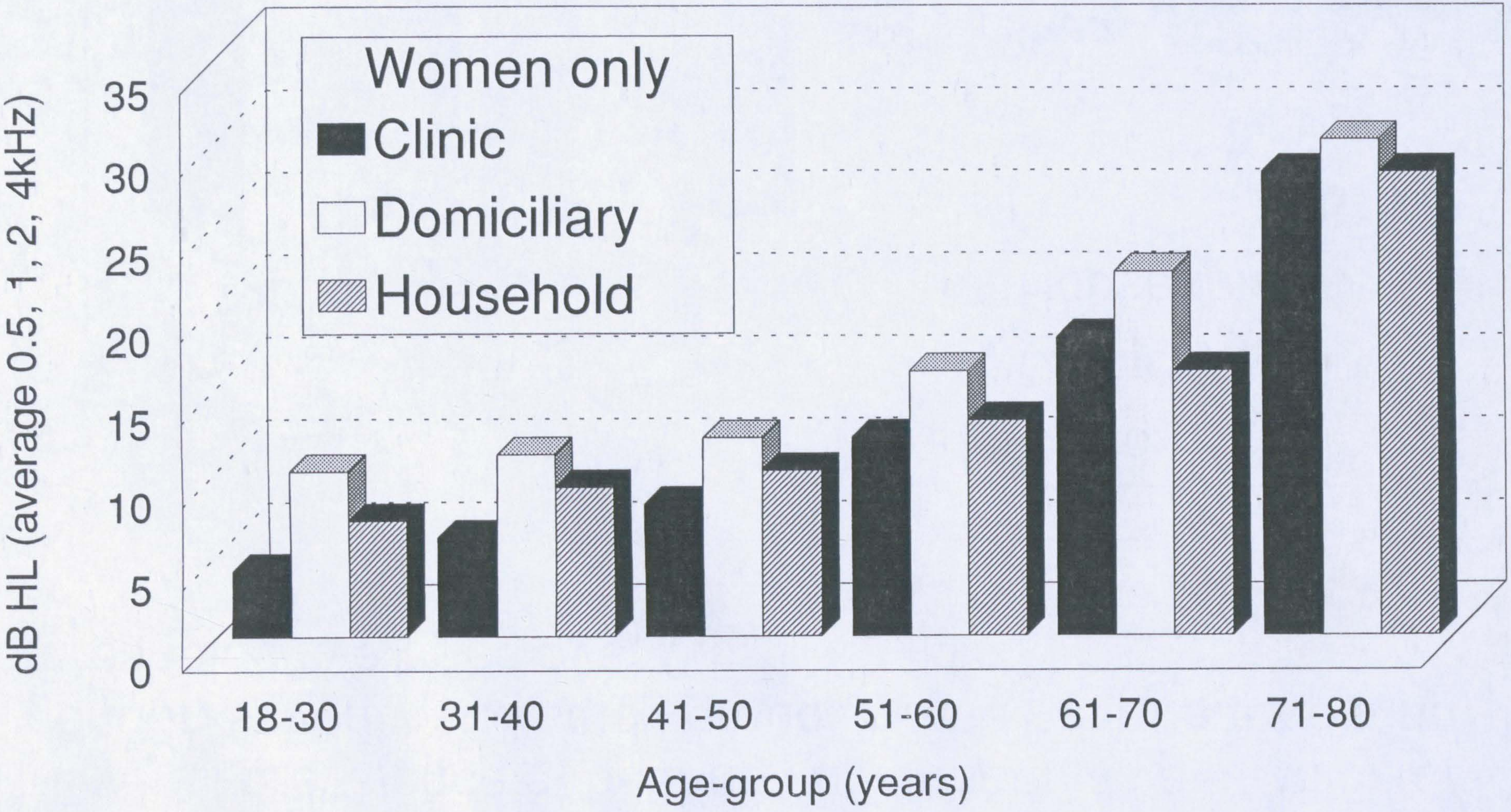

Fig 2.2b Median threshold level, in the better ear, as a function of age-group for men in the three study groups (Clinic, Domiciliary and Household)

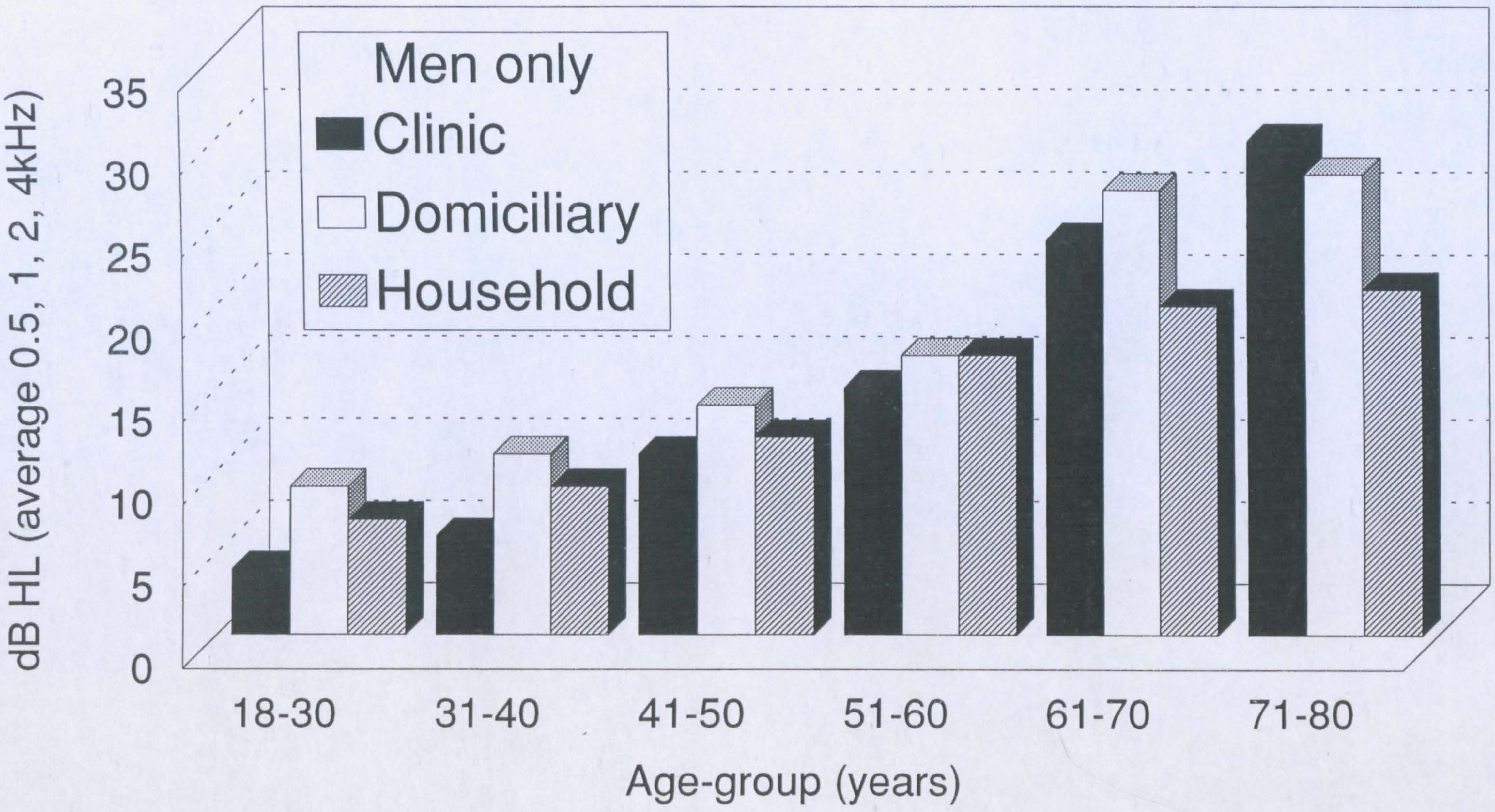

The number, means, SDs and medians for the three samples: (1) clinic (C), (2) domiciliary (D), and (3) household (H) for hearing thresholds as a function of age and gender.

Ear: better Frequency: 0.500 kHz

		NUMBER			MEANS			SD			MEDIAN		
Age Group	Gender	C	D	H	C	D	H	C	D	H	C	D	H
18–30	Female	373	74	61	6	12	10	8	7	8	5	9	9
31–40	Female	238	47	75	7	13	12	9	8	8	6	14	10
41–50	Female	201	40	46	9	16	11	11	10	7	7	14	11
51–60	Female	252	50	71	13	18	17	12	11	13	10	17	14
61–70	Female	205	40	46	18	22	20	17	10	13	15	20	19
71–80	Female	151	30	29	26	31	33	17	14	23	22	27	24
18–30	Male	363	72	54	5	15	10	7	8	7	5	14	9
31–40	Male	216	43	55	7	15	13	13	9	14	6	14	13
41–50	Male	185	36	44	10	16	13	11	12	12	8	12	13
51–60	Male	223	44	60	12	16	15	12	11	9	10	14	15
61–70	Male	175	35	56	16	19	18	14	11	11	12	20	18
71-80	Male	99	19	23	21	23	21	17	14	20	19	20	14

Ear: better Frequency: 1 kHz

		NUMBER			MEANS			SD			MEDIAN		
Age Group	Gender	C	D	H	C	D	H	C	D	H	C	D	H
18–30	Female	373	65	61	5	10	6	7	6	7	4	8	4
31–40	Female	238	47	75	6	12	9	9	8	7	4	9	8
41–50	Female	201	38	46	9	13	8	11	10	7	7	9	8
51–60	Female	252	50	71	11	16	13	12	11	13	9	13	9
61–70	Female	205	40	46	16	19	16	16	12	14	13	18	13
71–80	Female	150	30	29	26	27	32	19	14	25	21	23	24
18–30	Male	363	65	54	3	11	6	6	7	6	2	9	4
31–40	Male	216	39	55	6	12	9	13	9	14	4	9	8
41–50	Male	185	34	44	9	13	10	10	11	9	7	12	9
51–60	Male	223	43	60	12	15	11	12	12	9	9	13	10
61–70	Male	175	32	56	17	19	15	15	13	12	13	15	14
71–80	Male	99	19	23	22	23	22	17	15	22	20	18	13

Ear: better Frequency: 2 kHz

		NUMBER			MEANS			SD			MEDIAN		
Age Group	Gender	C	D	H	C	D	H	C	D	H	C	D	H
18–30	Female	373	66	61	4	9	6	8	6	8	3	9	4
31–40	Female	238	44	75	6	9	8	9	7	9	5	8	7
41–50	Female	201	36	46	10	13	9	11	12	7	7	9	8
51–60	Female	252	49	71	14	16	15	13	12	14	11	14	12
61–70	Female	205	39	46	21	22	19	16	13	18	18	19	13
71–80	Female	151	30	29	31	30	34	19	17	24	29	27	33
18–30	Male	363	55	54	4	9	5	8	8	6	3	7	4
31–40	Male	216	36	55	8	10	6	14	9	8	5	8	5
41–50	Male	185	32	44	11	14	10	13	12	11	8	9	8
51–60	Male	223	42	60	17	16	14	16	13	12	11	13	12
61–70	Male	175	32	56	25	25	25	19	15	19	20	22	20
71-80	Male	99	19	23	32	31	22	19	17	17	31	23	19

Ear: better Frequency: 4 kHz

		NUMBER			MEANS			SD			MEDIAN		
Age Group	Gender	C	D	H	C	D	H	C	D	H	C	D	H
18–30	Female	373	68	61	6	11	5	8	6	10	6	11	4
31–40	Female	236	47	75	10	11	10	11	6	13	8	12	8
41–50	Female	201	39	46	15	15	11	13	14	9	11	12	10
51–60	Female	252	50	71	20	21	21	16	15	18	17	17	17
61–70	Female	205	40	46	31	31	23	18	16	20	27	29	19
71–80	Female	150	30	29	43	43	41	20	17	21	42	40	32

The number, means, SDs and medians for the three samples: (1) clinic (C), (2) domiciliary (D), and (3) household (H) for hearing thresholds as a function of age and gender.

Ear: better Frequency: 4 kHz

		Number			Means			SD			Median		
Age Group	Gender	C	D	H	C	D	H	C	D	H	C	D	H
18–30	Male	363	62	54	8	10	8	11	7	9	7	9	7
31–40	Male	216	40	55	15	13	13	17	9	16	12	12	10
41–50	Male	185	36	44	22	24	19	18	18	16	18	18	18
51–60	Male	223	44	60	34	29	31	21	20	18	29	19	25
61–70	Male	175	35	56	46	46	38	21	19	22	44	44	33
71–80	Male	99	19	23	55	49	39	20	18	23	58	47	39

Ear: better Frequency: 0.5, 1, 2 and 4 kHz

		Number			Means			SD			Median		
Age Group	Gender	C	D	H	C	D	H	C	D	H	C	D	H
18–30	Female	373	63	61	5	11	7	7	6	7	4	10	7
31–40	Female	236	44	75	7	11	10	8	6	8	6	11	9
41–50	Female	201	35	46	11	15	10	10	10	6	8	12	10
51–60	Female	252	49	71	15	18	16	12	11	13	12	16	13
61–70	Female	205	39	46	21	24	20	15	11	15	18	22	16
71–80	Female	150	30	29	32	33	35	17	14	23	28	30	28
18–30	Male	363	51	54	5	12	7	7	7	6	4	9	7
31–40	Male	216	33	55	9	13	10	13	9	6	6	11	9
41–50	Male	185	30	44	13	18	13	11	12	9	11	14	12
51–60	Male	223	41	60	19	20	18	13	12	9	15	17	17
61–70	Male	175	32	56	26	28	24	15	12	14	24	27	20
71–80	Male	99	19	23	33	31	25	16	14	15	30	28	21

Ear: worse Frequency: 0.500 kHz

		Number			Means			SD			Median		
Age Group	Gender	C	D	H	C	D	H	C	D	H	C	D	H
18–30	Female	373	74	61	9	14	14	10	10	11	8	10	13
31–40	Female	237	47	75	11	16	18	13	9	12	9	14	16
41–50	Female	200	40	46	14	20	19	15	13	20	10	18	14
51–60	Female	251	48	71	19	25	21	18	19	15	13	20	18
61–70	Female	203	40	46	23	28	27	22	17	15	17	28	25
71–80	Female	149	29	29	33	37	40	23	21	24	25	32	33
18–30	Male	363	71	54	9	18	15	10	11	12	7	17	13
31–40	Male	216	41	55	12	18	19	14	11	16	8	15	17
41–50	Male	185	36	44	16	21	19	18	17	18	11	14	17
51–60	Male	223	44	60	18	21	20	19	20	13	13	14	18
61–70	Male	175	32	56	23	26	24	20	13	14	17	24	23
71–80	Male	99	19	23	27	27	26	23	18	21	21	20	19

Ear: worse Frequency: 1 kHz

		Number			Means			SD			Median		
Age Group	Gender	C	D	H	C	D	H	C	D	H	C	D	H
18–30	Female	373	69	61	8	12	10	10	8	8	6	13	9
31–40	Female	238	47	75	10	12	13	13	7	10	6	13	9
41–50	Female	201	38	46	14	19	14	15	13	19	10	15	10
51–60	Female	251	49	71	17	21	18	18	18	15	13	17	14
61–70	Female	205	39	46	23	25	21	22	17	15	15	20	18
71–80	Female	149	29	29	32	36	36	22	21	27	25	38	33
18–30	Male	363	72	54	7	13	11	9	10	11	5	12	8
31–40	Male	216	42	55	11	13	16	14	11	20	6	9	9
41–50	Male	185	36	44	15	18	16	17	17	17	10	13	13
51–60	Male	223	42	60	19	18	16	20	16	15	13	13	13
61–70	Male	175	35	56	24	23	23	23	18	18	18	19	18
71–80	Male	99	19	23	31	27	29	24	19	22	26	19	20

The number, means, SDs and medians for the three samples: (1) clinic (C), (2) domiciliary (D), and (3) household (H) for hearing thresholds as a function of age and gender.

Ear: worse Frequency: 2 kHz

Age Group	Gender	NUMBER C	NUMBER D	NUMBER H	MEANS C	MEANS D	MEANS H	SD C	SD D	SD H	MEDIAN C	MEDIAN D	MEDIAN H
18–30	Female	373	64	61	8	12	10	10	9	9	7	9	9
31–40	Female	238	45	75	10	13	13	13	8	11	6	9	10
41–50	Female	201	37	46	15	17	16	15	15	19	11	12	12
51–60	Female	252	49	71	20	22	21	18	18	16	16	14	15
61–70	Female	204	39	46	27	28	24	21	17	20	21	24	19
71–80	Female	151	30	29	38	45	39	23	19	25	34	43	33
18–30	Male	363	56	54	7	12	12	10	9	12	6	8	9
31–40	Male	216	39	55	12	12	16	16	12	17	9	9	9
41–50	Male	185	34	44	18	21	17	20	15	17	12	17	12
51–60	Male	221	42	60	24	23	22	23	22	19	17	17	18
61–70	Male	175	35	56	34	30	34	24	20	22	27	24	28
71–80	Male	99	19	23	43	38	31	24	19	19	40	29	28

Ear: worse Frequency: 4 kHz

Age Group	Gender	NUMBER C	NUMBER D	NUMBER H	MEANS C	MEANS D	MEANS H	SD C	SD D	SD H	MEDIAN C	MEDIAN D	MEDIAN H
18–30	Female	373	60	61	10	12	10	12	9	10	8	9	9
31–40	Female	236	47	75	15	14	15	16	7	14	12	13	12
41–50	Female	201	39	46	20	18	21	16	16	20	16	12	17
51–60	Female	251	49	71	28	29	29	20	20	19	23	23	23
61–70	Female	205	40	46	39	37	34	23	19	22	34	37	29
71–80	Female	150	30	29	53	55	50	23	26	21	54	50	43
18–30	Male	363	59	54	14	13	14	16	10	12	10	12	12
31–40	Male	216	43	55	23	18	25	20	14	24	16	14	17
41–50	Male	185	36	44	33	31	28	24	22	20	26	27	23
51–60	Male	223	44	60	44	40	44	26	25	20	40	38	43
61–70	Male	175	35	56	57	54	49	23	20	23	55	50	44
71–80	Male	99	19	23	65	63	49	23	17	21	65	64	49

Ear: worse Frequency: 0.5, 1, 2 and 4 kHz

Age Group	Gender	NUMBER C	NUMBER D	NUMBER H	MEANS C	MEANS D	MEANS H	SD C	SD D	SD H	MEDIAN C	MEDIAN D	MEDIAN H
18–30	Female	373	55	61	9	15	11	9	7	9	7	14	10
31–40	Female	235	45	75	11	14	14	12	7	11	8	12	12
41–50	Female	200	36	46	15	19	17	14	13	19	12	15	12
51–60	Female	250	47	71	21	25	21	17	18	15	16	19	17
61–70	Female	203	39	46	28	30	26	20	16	17	22	23	21
71–80	Female	149	29	29	39	43	40	21	20	23	34	38	35
18–30	Male	363	55	54	9	15	13	9	9	10	8	14	11
31–40	Male	216	38	55	14	16	17	15	11	16	10	14	12
41–50	Male	185	34	44	21	24	18	17	16	12	15	19	14
51–60	Male	221	42	60	26	25	25	20	16	14	21	21	22
61–70	Male	173	32	56	34	35	32	20	15	17	29	30	28
71–80	Male	99	19	23	42	39	32	21	16	17	38	34	28

The percentiles for the three samples: (1) clinic (C), (2) domiciliary (D), and (3) household (H) for hearing thresholds as a function of age and gender.

Ear: better Frequency: 500 Hz

		P.10			P.25			P.50			P.75			P.90		
Age Group	Gender	C	D	H	C	D	H	C	D	H	C	D	H	C	D	H
18–30	Female	–3	4	0	1	7	4	5	9	9	9	15	14	14	21	21
31–40	Female	–1	4	4	2	7	8	6	14	10	10	18	14	16	22	22
41–50	Female	0	7	3	3	9	5	7	14	11	13	22	14	21	28	20
51–60	Female	1	8	5	6	12	8	10	17	14	16	23	20	28	29	28
61–70	Female	3	9	8	7	17	13	15	20	19	22	28	25	38	33	33
71–80	Female	10	20	9	14	23	19	22	27	24	33	38	45	50	48	63
18–30	Male	–4	4	1	0	9	4	5	14	9	10	19	16	14	24	20
31–40	Male	–4	3	3	2	10	4	6	14	13	11	15	18	16	30	24
41–50	Male	0	4	–1	4	8	4	8	12	13	14	23	19	22	30	28
51–60	Male	0	4	4	5	9	9	10	14	15	17	19	20	26	33	25
61–70	Male	3	5	7	7	12	10	12	20	18	21	25	20	33	36	30
71–80	Male	4	7	3	11	12	9	19	20	14	27	28	34	41	39	34

Ear: better Frequency: 1 kHz

		P.10			P.25			P.50			P.75			P.90		
Age Group	Gender	C	D	H	C	D	H	C	D	H	C	D	H	C	D	H
18–30	Female	–2	4	–2	0	4	3	4	8	4	8	17	9	12	18	13
31–40	Female	–2	4	3	0	7	4	4	9	8	9	17	13	14	22	14
41–50	Female	0	3	0	3	7	4	7	9	8	12	15	10	20	23	18
51–60	Female	0	4	3	4	8	5	9	13	9	15	19	14	24	29	20
61–70	Female	3	5	5	7	10	9	13	18	13	20	23	18	35	33	28
71–80	Female	6	13	8	14	18	14	21	23	24	37	38	43	50	43	59
18–30	Male	–5	4	–2	–2	7	3	2	9	4	7	15	9	13	19	13
31–40	Male	–2	4	–1	0	4	3	4	9	8	9	17	14	16	20	18
41–50	Male	0	4	–1	3	4	4	7	12	9	12	18	13	19	24	19
51–60	Male	1	4	3	4	7	4	9	13	10	16	17	14	27	29	19
61–70	Male	2	4	3	7	10	8	13	15	14	23	27	19	35	37	28
71–80	Male	3	10	8	10	14	9	20	18	13	30	23	29	48	43	43

Ear: better Frequency: 2 kHz

		P.10			P.25			P.50			P.75			P.90		
Age Group	Gender	C	D	H	C	D	H	C	D	H	C	D	H	C	D	H
18–30	Female	–4	2	–2	0	4	2	3	9	4	8	15	10	13	19	14
31–40	Female	–3	3	–2	1	4	3	5	8	7	8	13	13	15	15	18
41–50	Female	–1	3	3	3	8	5	7	9	8	13	17	13	24	23	17
51–60	Female	2	3	2	5	9	7	11	14	12	19	19	18	28	34	29
61–70	Female	4	9	7	10	13	8	18	19	13	26	28	24	40	44	30
71–80	Female	9	15	13	18	19	17	29	27	33	43	40	44	55	48	63
18–30	Male	–5	3	–3	–2	4	2	3	7	4	9	10	9	15	14	14
31–40	Male	–4	3	–2	0	4	2	5	8	5	11	10	10	20	18	13
41–50	Male	–1	3	–1	3	7	2	8	9	8	15	17	13	26	29	27
51–60	Male	1	4	2	6	8	5	11	13	12	25	23	20	39	29	29
61–70	Male	6	8	8	11	16	13	20	22	20	34	29	29	52	47	47
71–80	Male	9	18	3	18	19	9	31	23	19	43	39	29	60	57	44

The percentiles for the three samples: (1) clinic (C), (2) domiciliary (D), and (3) household (H) for hearing thresholds as a function of age and gender.

Ear: better **Frequency: 4 kHz**

Age Group	Gender	P.10 C	P.10 D	P.10 H	P.25 C	P.25 D	P.25 H	P.50 C	P.50 D	P.50 H	P.75 C	P.75 D	P.75 H	P.90 C	P.90 D	P.90 H
18–30	Female	−3	2	−5	1	7	0	6	11	4	10	15	9	16	18	14
31–40	Female	−2	3	−2	4	6	3	8	12	8	13	13	12	22	17	23
41–50	Female	0	4	3	6	7	7	11	12	10	20	16	14	30	28	22
51–60	Female	5	7	4	10	12	9	17	17	17	26	27	27	37	42	42
61–70	Female	10	13	4	17	22	13	27	29	19	40	39	30	55	55	47
71–80	Female	17	22	19	25	32	23	42	40	32	58	52	53	66	68	73
18–30	Male	−3	2	−3	2	5	2	7	9	7	12	14	13	20	18	17
31–40	Male	0	4	2	6	5	5	12	12	10	22	14	17	30	27	22
41–50	Male	5	7	2	10	12	9	18	18	18	30	37	23	49	52	43
51–60	Male	10	9	14	17	14	18	29	19	25	47	42	39	60	57	57
61–70	Male	20	23	13	28	25	22	44	44	33	61	57	62	73	78	67
71–80	Male	26	24	14	44	39	19	58	47	39	68	63	54	78	69	74

Ear: better **Frequency: 0.5, 1, 2 and 4 kHz**

Age Group	Gender	P.10 C	P.10 D	P.10 H	P.25 C	P.25 D	P.25 H	P.50 C	P.50 D	P.50 H	P.75 C	P.75 D	P.75 H	P.90 C	P.90 D	P.90 H
18-30	Female	−1	4	0	2	7	3	4	10	7	8	14	10	13	21	13
31–40	Female	0	4	2	3	7	5	6	11	9	9	14	12	15	19	20
41–50	Female	2	5	5	4	9	6	8	12	10	14	15	12	20	26	16
51–60	Female	5	7	5	8	12	9	12	16	13	18	21	21	28	34	30
61–70	Female	9	13	7	12	18	13	18	22	16	26	29	25	39	38	31
71–80	Female	12	18	14	19	23	21	28	30	28	43	44	44	54	47	63
18-30	Male	-2	6	1	1	8	3	4	9	7	8	14	10	14	17	14
31–40	Male	1	6	3	4	8	5	6	11	9	13	16	13	19	26	18
41–50	Male	3	8	4	5	9	7	11	14	12	17	25	16	24	35	26
51–60	Male	6	9	8	10	12	10	15	17	17	25	24	22	36	34	30
61-70	Male	11	13	11	14	20	15	24	27	20	33	33	27	44	41	43
71-80	Male	15	19	8	23	20	13	30	28	21	40	38	34	55	49	45

Ear: worse **Frequency: 500 Hz**

Age Group	Gender	P.10 C	P.10 D	P.10 H	P.25 C	P.25 D	P.25 H	P.50 C	P.50 D	P.50 H	P.75 C	P.75 D	P.75 H	P.90 C	P.90 D	P.90 H
18–30	Female	0	4	3	4	5	4	8	10	13	11	22	22	18	29	29
31–40	Female	0	8	4	4	9	9	9	14	16	15	21	23	23	28	33
41–50	Female	1	6	5	5	9	10	10	18	14	17	27	19	31	39	30
51–60	Female	5	9	8	8	15	13	13	20	18	24	29	25	40	44	35
61–70	Female	5	13	13	10	15	19	17	28	25	30	40	32	50	53	43
71–80	Female	10	18	15	15	23	23	25	32	33	45	45	47	66	74	68
18–30	Male	−2	8	4	3	10	8	7	17	13	13	24	22	20	34	28
31–40	Male	2	5	8	5	10	10	8	15	17	15	20	22	25	33	35
41–50	Male	2	4	−1	5	9	9	11	14	17	20	30	25	40	38	38
51–60	Male	4	4	8	7	9	12	13	14	18	21	25	25	41	49	32
61–70	Male	5	13	10	10	17	14	17	24	23	30	32	28	48	49	40
71–80	Male	6	12	5	11	15	14	21	20	19	35	29	34	60	61	39

The percentiles for the three samples: (1) clinic (C), (2) domiciliary (D), and (3) household (H) for hearing thresholds as a function of age and gender.

Ear: worse Frequency: 1 kHz

		P.10			P.25			P.50			P.75			P.90		
Age Group	Gender	C	D	H	C	D	H	C	D	H	C	D	H	C	D	H
18–30	Female	–1	2	3	2	5	4	6	13	9	11	18	13	19	24	19
31–40	Female	–1	2	4	3	5	5	6	13	9	11	18	14	25	22	28
41–50	Female	0	7	4	5	9	8	10	15	10	15	24	14	29	38	23
51–60	Female	3	5	8	7	12	9	13	17	14	20	24	20	38	39	33
61–70	Female	6	9	8	10	14	13	15	20	18	28	38	24	49	50	38
71–80	Female	7	13	8	16	20	18	25	38	33	45	39	44	64	67	69
18–30	Male	–2	0	3	1	5	5	5	12	8	10	18	14	15	28	20
31–40	Male	0	0	3	3	8	8	6	9	9	14	19	15	25	28	31
41–50	Male	2	4	4	5	8	8	10	13	13	16	23	19	35	43	33
51–60	Male	2	4	4	8	8	9	13	13	13	22	22	18	40	39	25
61–70	Male	5	4	8	9	10	13	18	19	18	31	30	29	51	49	53
71–80	Male	7	12	8	14	14	18	26	19	20	42	38	39	65	59	43

Ear: worse Frequency: 2 kHz

		P.10			P.25			P.50			P.75			P.90		
Age Group	Gender	C	D	H	C	D	H	C	D	H	C	D	H	C	D	H
18–30	Female	–1	1	–1	2	4	3	7	9	9	12	14	13	18	30	22
31–40	Female	0	4	3	2	7	7	6	9	10	12	18	14	26	23	23
41–50	Female	2	3	7	6	8	9	11	12	12	19	19	17	30	39	23
51–60	Female	5	7	7	9	11	12	16	14	15	26	25	27	37	42	43
61–70	Female	8	12	8	12	14	13	21	24	19	33	39	33	55	50	42
71–80	Female	11	22	14	22	28	18	34	43	33	53	54	48	67	78	64
18–30	Male	–2	4	2	2	7	4	6	8	9	11	17	17	18	23	24
31–40	Male	–1	2	3	4	4	8	9	9	9	16	13	18	27	28	33
41–50	Male	2	7	7	6	10	8	12	17	12	22	28	18	43	42	37
51–60	Male	3	3	3	10	9	8	17	17	18	32	29	29	58	49	52
61–70	Male	10	6	12	16	13	18	27	24	28	46	38	43	67	62	64
71–80	Male	17	18	8	25	23	18	40	29	28	55	58	44	74	64	53

Ear: worse Frequency: 4 kHz

		P.10			P.25			P.50			P.75			P.90		
Age Group	Gender	C	D	H	C	D	H	C	D	H	C	D	H	C	D	H
18–30	Female	–2	3	2	3	5	5	8	9	9	15	17	14	23	23	19
31–40	Female	1	7	3	6	8	9	12	13	12	20	17	15	28	25	29
41–50	Female	5	3	7	10	8	12	16	12	17	25	22	24	36	41	32
51–60	Female	10	12	9	16	17	17	23	23	23	35	38	37	50	50	57
61–70	Female	15	14	13	21	19	22	34	37	29	51	49	42	68	67	54
71–80	Female	18	24	27	35	37	33	54	50	43	68	72	63	81	97	77
18–30	Male	0	4	2	5	7	7	10	12	12	19	17	19	36	24	29
31–40	Male	5	7	7	10	7	12	16	14	17	30	22	28	50	42	49
41–50	Male	9	7	7	15	14	17	26	27	23	45	42	37	66	63	63
51–60	Male	16	12	22	24	22	27	40	38	43	60	52	59	80	73	72
61–70	Male	27	32	20	39	39	32	55	50	44	71	63	67	89	85	77
71–80	Male	37	35	19	50	49	33	65	64	49	78	72	64	92	84	74

The percentiles for the three samples: (1) clinic (C), (2) domiciliary (D), and (3) household (H) for hearing thresholds as a function of age and gender.

Ear: worse Frequency: 0.5, 1, 2 and 4 kHz

		P.10			P.25			P.50			P.75			P.90		
Age Group	Gender	C	D	H	C	D	H	C	D	H	C	D	H	C	D	H
18–30	Female	1	7	2	4	9	6	7	14	10	11	21	14	18	24	20
31–40	Female	2	7	5	4	9	7	8	12	12	13	19	15	22	24	28
41–50	Female	5	8	7	7	10	9	12	15	12	18	25	17	30	39	27
51–60	Female	7	11	9	11	16	12	16	19	17	25	29	25	38	44	33
61–70	Female	11	16	11	16	19	16	22	23	21	34	40	33	52	50	39
71–80	Female	16	20	18	22	26	24	34	38	35	50	52	47	70	76	69
18–30	Male	–	7	4	4	9	7	8	14	11	13	19	14	21	26	22
31–40	Male	4	7	6	7	9	9	10	14	12	18	18	18	28	29	32
41–50	Male	6	7	9	9	14	9	15	19	14	26	29	21	41	45	33
51–60	Male	9	9	11	13	14	16	21	21	22	32	32	29	52	46	40
61–70	Male	16	20	14	19	26	20	29	30	28	45	37	39	59	59	47
71–80	Male	19	23	12	28	26	23	38	34	28	53	49	46	71	62	49

Differences for each threshold variable between (1) the clinic sample and domiciliary sample, and (2) the clinic sample and household sample, for selected statistics of the hearing threshold.

Ear: better Frequency: 500 Hz

		(1) CLINIC-DOMICILIARY						(2) CLINIC-HOUSEHOLD					
Age Group	Gender	Mean	P.10	P.25	P.50	P.75	P.90	Mean	P.10	P.25	P.50	P.75	P.90
18–30	Female	–6	–7	–6	–4	–6	–7	–4	–3	–3	–4	–5	–7
31–40	Female	–6	–5	–5	–8	–8	–6	–5	–5	–6	–4	–4	–6
41–50	Female	–6	–7	–6	–7	–9	–7	–2	–3	–2	–4	–1	1
51–60	Female	–5	–7	–6	–7	–7	–1	–4	–4	–2	–4	–4	0
61–70	Female	–4	–6	–10	–5	–6	5	–3	–5	–6	–4	–3	5
71–80	Female	–5	–10	–9	–5	–5	2	–7	1	–5	–2	–12	–13
18–30	Male	–10	–8	–9	–9	–9	–10	–5	–5	–4	–4	–6	–6
31–40	Male	–8	–7	–8	–8	–4	–14	–6	–7	–2	–7	–7	–8
41–50	Male	–6	–4	–4	–4	–9	–8	–3	1	0	–5	–5	–6
51–60	Male	–4	–4	–4	–4	–2	–7	–3	–4	–4	–5	–3	1
61–70	Male	–3	–2	–5	–8	–4	–3	–2	–4	–3	–6	1	3
71–80	Male	–2	–3	–1	–1	–1	2	–0	1	2	5	–7	7

Ear: better Frequency: 1 kHz

		(1) CLINIC-DOMICILIARY						(2) CLINIC-HOUSEHOLD					
Age Group	Gender	Mean	P.10	P.25	P.50	P.75	P.90	Mean	P.10	P.25	P.50	P.75	P.90
18–30	Female	–5	–6	–4	–4	–9	–6	–1	0	–3	0	–1	–1
31–40	Female	–6	–6	–7	–5	–8	–8	–3	–5	–4	–4	–4	0
41–50	Female	–4	–3	–4	–2	–3	–3	1	0	–1	–1	2	2
51–60	Female	–4	–4	–4	–4	–4	–5	–1	–3	–1	0	1	4
61–70	Female	–3	–2	–3	–5	–3	2	0	–2	–2	0	2	7
71–80	Female	–1	–7	–4	–2	–1	7	–6	–2	0	–3	–6	–9
18–30	Male	–8	–9	–9	–7	–8	–6	–3	–3	–5	–2	–2	0
31–40	Male	–5	–6	–4	–5	–8	–4	–3	–1	–3	–4	–5	–2
41–50	Male	–4	–4	–1	–5	–6	–5	–1	1	–1	–2	–1	0
51–60	Male	–3	–3	–3	–4	–1	–2	1	–2	0	–1	2	8
61–70	Male	–2	–2	–3	–2	–4	–2	1	–1	–1	–1	4	7
71–80	Male	–1	–7	–4	2	7	5	1	–5	1	7	1	5

Ear: better Frequency: 2 kHz

		(1) CLINIC-DOMICILIARY						(2) CLINIC-HOUSEHOLD					
Age Group	Gender	Mean	P.10	P.25	P.50	P.75	P.90	Mean	P.10	P.25	P.50	P.75	P.90
18–30	Female	–5	–6	–4	–6	–7	–6	–1	–2	–2	–1	–2	–1
31–40	Female	–3	–6	–3	–3	–5	0	–2	–1	–2	–2	–5	–3
41–50	Female	–3	–4	–5	–2	–4	1	1	–4	–2	–1	0	7
51–60	Female	–3	–1	–4	–3	0	–6	–1	0	–2	–1	1	–1
61–70	Female	–2	–5	–3	–1	–2	–4	2	–3	2	5	2	10
71–80	Female	1	–6	–1	2	3	7	–3	–4	1	–4	–1	–8
18–30	Male	–5	–8	–6	–4	–1	1	–1	–2	–4	–1	0	1
31–40	Male	–2	–7	–4	–3	1	2	2	–2	–2	0	1	7
41–50	Male	–3	–4	–4	–1	–2	–3	1	0	1	0	2	–1
51–60	Male	1	–3	–2	–2	2	10	3	–1	1	–1	5	10
61–70	Male	–0	–2	–5	–2	5	5	–1	–2	–2	0	5	5
71–80	Male	2	–9	–1	8	4	3	10	6	9	12	14	16

Differences for each threshold variable between (1) the clinic sample and domiciliary sample, and (2) the clinic sample and household sample, for selected statistics of the hearing threshold.

Ear: better Frequency: 4 kHz

		(1) CLINIC-DOMICILIARY						(2) CLINIC-HOUSEHOLD					
Age Group	Gender	Mean	P.10	P.25	P.50	P.75	P.90	Mean	P.10	P.25	P.50	P.75	P.90
18–30	Female	–4	–5	–6	–5	–5	–2	1	2	1	2	1	2
31–40	Female	–1	–5	–2	–4	0	5	–0	0	1	0	1	–1
41–50	Female	–0	–4	–1	–1	4	2	3	–3	–1	1	6	8
51–60	Female	–1	–2	–2	0	–1	–5	–1	1	1	0	–1	–5
61–70	Female	–1	–3	–5	–2	1	0	7	6	4	8	10	8
71–80	Female	0	–5	–7	2	6	–2	3	–2	2	10	5	–7
18–30	Male	–2	–5	–3	–2	–2	2	0	0	0	0	–1	3
31–40	Male	3	–4	1	0	8	3	2	–2	1	2	5	8
41–50	Male	–2	–2	–2	0	–7	–3	3	3	1	0	7	6
51–60	Male	5	1	3	10	5	3	3	–4	–1	4	8	3
61–70	Male	1	–3	3	0	4	–5	8	7	6	11	–1	6
71–80	Male	6	2	5	11	5	9	16	12	25	19	14	4

Ear: better Frequency: 0.5, 1, 2 and 4 kHz

		(1) CLINIC-DOMICILIARY						(2) CLINIC-HOUSEHOLD					
Age Group	Gender	Mean	P.10	P.25	P.50	P.75	P.90	Mean	P.10	P.25	P.50	P.75	P.90
18–30	Female	–5	–5	–5	–5	–6	–8	–1	–1	–2	–3	–2	–1
31–40	Female	–4	–4	–4	–5	–5	–4	–3	–2	–3	–4	–3	–5
41–50	Female	–4	–4	–4	–4	–2	–6	1	–3	–2	–2	2	4
51–60	Female	–3	–2	–4	–4	–3	–6	–2	–0	–1	–2	–3	–2
61–70	Female	–3	–5	–6	–4	–3	1	2	2	–1	2	1	8
71–80	Female	–1	–6	–4	–3	–2	7	–3	–2	–2	–0	–2	–9
18–30	Male	–7	–8	–7	–5	–6	–3	–2	–3	–2	–3	–2	0
31–40	Male	–4	–5	–4	–4	–3	–7	–0	–3	–1	–3	0	1
41–50	Male	–5	–5	–4	–4	–8	–11	0	–1	–2	–2	1	–2
51–60	Male	–1	–3	–2	–2	1	2	1	–2	0	–2	3	6
61–70	Male	–2	–2	–6	–3	–1	3	2	0	–1	4	6	1
71–80	Male	1	–4	3	2	2	6	8	7	10	9	6	10

Differences for each threshold variable between (1) the clinic sample and domiciliary sample, and (2) the clinic sample and household sample, for selected statistics of the hearing threshold.

Ear: worse Frequency: 500 Hz

		(1) CLINIC-DOMICILIARY						(2) CLINIC-HOUSEHOLD					
Age Group	Gender	Mean	P.10	P.25	P.50	P.75	P.90	Mean	P.10	P.25	P.50	P.75	P.90
18–30	Female	–5	–4	–1	–2	–11	–11	–5	–3	0	–5	–11	–11
31–40	Female	–5	–8	–5	–5	–6	–5	–6	–4	–5	–7	–8	–10
41–50	Female	–6	–5	–4	–8	–10	–8	–5	–4	–5	–4	–2	1
51–60	Female	–6	–4	–7	–7	–5	–4	–2	–3	–5	–5	–1	5
61–70	Female	–5	–8	–5	–11	–10	–3	–4	–8	–9	–8	–2	7
71–80	Female	–5	–8	–8	–7	0	–8	–7	–5	–8	–8	–2	–2
18–30	Male	–9	–10	–7	–10	–11	–14	–6	–6	–5	–6	–9	–8
31–40	Male	–5	–3	–5	–7	–5	–8	–7	–6	–5	–9	–7	–10
41–50	Male	–5	–2	–4	–3	–10	2	–4	3	–4	–6	–5	2
51–60	Male	–3	0	–2	–1	–4	–8	–2	–4	–5	–5	–4	9
61–70	Male	–3	–8	–7	–7	–2	–1	–2	–5	–4	–6	2	8
71–80	Male	0	–6	–4	1	6	–1	1	1	–3	2	1	21

Ear: worse Frequency: 1 kHz

		(1) CLINIC-DOMICILIARY						(2) CLINIC-HOUSEHOLD					
Age Group	Gender	Mean	P.10	P.25	P.50	P.75	P.90	Mean	P.10	P.25	P.50	P.75	P.90
18–30	Female	–4	–3	–3	–7	–7	–5	–2	–4	–2	–3	–2	0
31–40	Female	–3	–3	–2	–7	–7	3	–3	–5	–2	–3	–3	–3
41–50	Female	–5	–7	–4	–5	–9	–9	–1	–4	–3	0	1	6
51–60	Female	–4	–2	–5	–4	–4	–1	–1	–5	–2	–1	0	5
61–70	Female	–2	–3	–4	–5	–10	–1	2	–2	–3	–3	4	11
71–80	Female	–4	–6	–4	–13	6	–3	–4	–1	–2	–8	1	–5
18–30	Male	–6	–2	–4	–7	–8	–13	–4	–5	–4	–3	–4	–5
31–40	Male	–2	0	–5	–3	–5	–3	–5	–3	–5	–3	–1	–6
41–50	Male	–3	–2	–3	–3	–7	–8	–1	–2	–3	–3	–3	2
51–60	Male	1	–2	0	0	0	1	3	–2	–1	0	4	15
61–70	Male	1	1	–1	–1	1	2	1	–3	–4	0	2	–2
71–80	Male	4	–5	0	7	4	6	2	–1	–4	6	3	22

Ear: worse Frequency: 2 kHz

		(1) CLINIC-DOMICILIARY						(2) CLINIC-HOUSEHOLD					
Age Group	Gender	Mean	P.10	P.25	P.50	P.75	P.90	Mean	P.10	P.25	P.50	P.75	P.90
18–30	Female	–3	–2	–2	–2	–2	–12	–1	0	–1	–2	–1	–4
31–40	Female	–3	–4	–5	–3	–6	3	–3	–3	–5	–4	–2	3
41–50	Female	–2	–1	–2	–1	0	–9	–1	–5	–3	–1	2	7
51–60	Female	–2	–2	–2	2	1	–5	–1	–2	–3	1	–1	–6
61–70	Female	–1	–4	–2	–3	–6	5	2	0	–1	2	0	13
71–80	Female	–6	–11	–6	–9	–1	–11	–0	–3	4	1	5	3
18–30	Male	–5	–6	–5	–2	–6	–5	–5	–4	–2	–3	–6	–6
31–40	Male	0	–3	0	0	3	–1	–4	–4	–4	0	–2	–6
41–50	Male	–3	–5	–4	–5	–6	1	1	–5	–2	0	4	6
51–60	Male	1	0	1	0	3	9	2	0	2	–1	3	6
61–70	Male	4	4	3	3	8	5	1	–2	–2	–1	3	3
71–80	Male	6	–1	2	11	–3	10	12	9	7	12	11	21

Differences for each threshold variable between (1) the clinic sample and domiciliary sample, and (2) the clinic sample and household sample, for selected statistics of the hearing threshold.

Ear: worse **Frequency: 4 kHz**

Age Group	Gender	(1) Clinic-domiciliary Mean	P.10	P.25	P.50	P.75	P.90	(2) Clinic-household Mean	P.10	P.25	P.50	P.75	P.90
18–30	Female	−1	−5	−2	−1	−2	0	−0	−4	−2	−1	1	4
31–40	Female	2	−6	−2	−1	3	3	0	−2	−3	0	5	−1
41–50	Female	2	2	2	4	3	−5	−1	−2	−2	−1	1	4
51–60	Female	−1	−2	−1	0	−3	0	−1	1	−1	0	−2	−7
61–70	Female	2	1	2	−3	2	1	5	2	−1	5	9	14
71–80	Female	−2	−6	−2	4	−4	−16	3	−9	2	11	5	4
18–30	Male	2	−4	−2	−2	2	12	0	−2	−2	−2	0	7
31–40	Male	5	−2	3	2	8	8	−2	−2	−2	−1	2	1
41–50	Male	2	2	1	−1	3	3	5	2	−2	3	8	3
51–60	Male	4	4	2	2	8	7	0	−6	−3	−3	1	8
61–70	Male	3	−5	0	5	8	4	8	7	7	11	4	12
71–80	Male	3	2	1	1	6	8	17	18	17	16	14	18

Ear: worse **Frequency: 0.5, 1, 2 and 4 kHz**

Age Group	Gender	(1) Clinic-domiciliary Mean	P.10	P.25	P.50	P.75	P.90	(2) Clinic-household Mean	P.10	P.25	P.50	P.75	P.90
18–30	Female	−6	−6	−6	−7	−10	−6	−2	−1	−3	−3	−3	−2
31–40	Female	−3	−5	−5	−4	−6	−2	−3	−3	−3	−4	−2	−6
41–50	Female	−4	−3	−3	−3	−8	−9	−1	−2	−2	0	1	3
51–60	Female	−4	−4	−5	−3	−4	−5	−0	−2	−1	−1	0	5
61–70	Female	−2	−6	−3	−2	−6	2	2	−0	−0	1	1	13
71–80	Female	−4	−4	−4	−4	−2	−7	−1	−2	−2	−1	3	1
18–30	Male	−6	−7	−5	−6	−6	−6	−3	−4	−3	−3	−1	−2
31–40	Male	−1	−2	−2	−4	1	−1	−3	−2	−2	−2	0	−5
41–50	Male	−3	−1	−5	−5	−4	−4	3	−4	0	1	5	8
51–60	Male	1	0	−1	1	0	6	2	−2	−3	−1	3	12
61–70	Male	−0	−5	−8	−1	8	0	2	2	−2	1	6	12
71–80	Male	3	−4	2	4	4	9	10	7	5	10	7	22

Chapter 3

Prevalence of hearing impairment

3.0 Scope and introduction

The previous two chapters have dealt with the background and methodology of the NSH, and complemented the previous major publication on these aspects (Davis, 1989) including the role of the domiciliary and household studies. This chapter and the following chapters use data only from the main study of the NSH. This chapter presents five sets of tables, each set of which presents the prevalence of hearing impairments in the population. Each set of tables is a different section. The first four sections (i-iv) give prevalences in 10-year age bands for better (sections i and ii) and worse (sections iii and iv) ear. Sections ii and iv present prevalences for individual frequencies and sections i and iii for those combinations of frequencies that are of major interest. The fifth section includes the better ear only, for 20-year age bands.

3.1 Definitions of prevalence

The prevalence of hearing impairment depends on the severity of hearing impairment that is used as a criterion for discussion or action. The appropriate value for the criterion depends on the purpose for which it is going to be used. This chapter presents prevalences over a very wide range of hearing impairment to reflect the diversity of need, both for planning services (e.g. health promotion, hearing aid services etc) for the different client groups, and for research purposes. For example, in assessing the hearing health of young people the percentage with hearing thresholds $\geq$ 10 dB HL at any frequency may be of concern, because of the expected good hearing in that population. However in assessing the need for hearing aid services the percentage $\geq$ 25 or $\geq$ 30 dB HL at the 4FA would be more informative.

Prevalence is calculated in this chapter as the estimated percentage of the population whose hearing threshold (or average threshold) attain a criterion of x dB HL or any value above that criterion. This is the cumulative prevalence using that criterion. It is therefore important not to add the prevalence at two levels e.g. for $\geq$25 and $\geq$45 dB HL together, which would double the contribution of those in the range 25 to 44 dB HL. Where prevalence or numbers are given for a discrete severity range (e.g. Chapter 9) this is explicitly mentioned as a range, e.g. 25-44 dB HL and such grouping becomes permissible. The cumulated prevalence of hearing impairment used in this chapter may be interpreted as the estimated percentage of people in the British population who have at least that level of hearing impairment at the time of the study.

The prevalence tables within each section are separately numbered by a mnemonic for the ear (B-Better or W-Worse), frequency (e.g. 1k for 1 kHz), or average (see Table 3.1) followed by a sequence number (1-63). The sequence number denotes a particular combination of age group, gender and occupational group. There are 63 combinations because there are seven age groups: overall (18-80) plus the six component age-group strata, three gender groups (overall, female and male), and three occupational groups (overall, non-manual and manual). The occupational groups were based on head of household information collected at the clinical interview rather than the self-report data. The social classes were coded in accordance with the Registrar General's Classification of Occupations (OPCS, 1980), and the occupational groups were obtained from the social class by combining classes I, II, IIINM as non-manual and the remainder as 'manual'.

Section i-iv follow the same pattern of tables. Within each section the 63 tables are arranged as follows. The first three tables are overall age and occupational groups, giving the overall prevalences and then separately for each gender. The next six tables (4-9) show prevalences at each age group overall genders and occupational groups. These are followed by the marginal prevalences for each occupational group (tables 10 and 11). Twenty four tables (21-35) then give the age group by gender and age group by occupational group prevalences. The gender by occupational group prevalence tables (36-39) are then followed by the twenty four tables (40-63) for the gender by occupational group by age group prevalences. Section v is different in that it only has three age groups (18-40, 41-60, 61-80) and hence 27 tables per frequency measure. The tables in this section rotate by occupational group, gender and age group (fastest moving). In my experience this larger delineation of age group is often the compromise that has to be made in order to obtain sufficient numbers in any one age, gender and occupational cell, when comparing the NSH with smaller studies.

Table 3.1 Mnemonic used in the text for indicating averages of individual thresholds grouped in particular ways

Abbreviation Mnemonic(s) for average	Frequencies in average (kHz)
5124 (4FA)	0.5, 1, 2, 4
512 (3FA)	0.5, 1, 2
2551	0.25, 0.5, 1
123	1, 2, 3
124	1, 2, 4
468	4, 6, 8

Each page in the prevalence chapter contains seven tables, and each of these tables contains the table mnemonic, age group, gender and occupational group to which the prevalence statistics apply. The Total cases gives the number of actual people who were tested and from whom valid measurements

were obtained. Thus in section i Table B5124-1 has a total of 2663 cases who contributed valid data - i.e. all the frequencies contributing to the average had valid data.

In all sections the prevalence is shown from 0 dB HL to 55 dB HL in 5 dB steps and thereafter in 10 dB steps up to 95 dB HL. Thus for the mid-frequency 4FA in the better ear the prevalence estimate for ≥ 20 dB HL is 22.6%. By the methods outlined in Chapter 2, the 95% CI for this proportion is calculated to be 21.3 - 24.0%, for a 95% confidence region. Thus, if the non-sampling biases are small, there is a reasonably high degree of precision about this prevalence estimate, the standard errors here being about ± 5% of the estimate. The design effect (Deft) is included in section i to show the extent to which the sampling design has reduced the variance of the estimate. The design effect shown here is the square root of the ratio of the variance obtained by the sample design to that which would have been obtained if a simple random sample had been used. It essentially measures variance due to the stratification factors, hence the efficiency of the design. If the design effect is less than 1 then the design is more efficient. For this prevalence the estimate of the design effect is 0.83, which means that the study was quite efficient at this point - in general Deft depends on age and severity of impairment. Taken literally, a design effect of 0.83 implies that for a simple random sample to be as efficient, it would need 45% more people: 3861 rather than 2663. The last statistic in the table is the Number of people who have the degree of hearing impairment or worse. Thus 962 people out of the total cases of 2663 had an average hearing threshold of ≥20 dB HL. These two numbers (i.e. 962/2663) cannot be used directly to compute the prevalence because various weighting factors need to be applied to correct for the oversampling of the hearing-impaired sub-population at the second stage of the sample (see Chapter 2). But these numbers are important because indirectly they give a reassurance about the robustness of particular prevalence statistics. For instance, less reliance might be placed on the cells with a small number of total cases and a very small number of people who exceed the prevalence criterion.

3.2 The prevalence tables: summary

A gross simplification of the series of tables B5124 in section i and W5124 in section iii, suggests that if an impairment criterion level of 25 dB HL was taken, there are more than 1 in 4 of the population, aged 18-80, with at least one ear that has an impairment. For bilateral impairments there are 1 in 6. About 1 in 25 has at least a moderate bilateral hearing impairment. It is very unlikely that there have been substantial increases in prevalence (see Davis, 1991) since this study was conducted. This is also reflected in another recent study where there do not appear to be any changes in self-reported disability in the British population either, Aldridge and Davis, 1993.

The three main demographic variables - age, gender and occupational group - all show significant effects on the prevalence of hearing impairment. The effect of age is pervasive at each frequency and at all severities. The effect of gender and occupational group are similar, and they exert the greatest influence over the prevalence of slight, mild and mild-moderate hearing impairments (up to about 30 dB HL). The effect of occupational group shows up more strongly for men than for women, and is also frequency dependent.

Prevalence by age group, gender and occupational group for the better ear over 0.5, 1, 2, and 4 kHz

Table B5124-1 Age Group = 18-80 Gender = OVERALL Occupational group = OVERALL Total cases = 2663

	Threshold dB HL≥															
	0	5	10	15	20	25	30	35	40	45	50	55	65	75	85	95
**Prevalence**	**93.4**	**72.1**	**49.9**	**32.7**	**22.6**	**16.1**	**11.2**	**8.2**	**5.8**	**3.9**	**2.9**	**2.1**	**1.1**	**0.7**	**0.4**	**0.2**
**Lower CI**	91.8	70.0	47.9	31.1	21.3	15.0	10.3	7.4	5.1	3.4	2.5	1.7	0.8	0.5	0.2	0.1
**Upper CI**	94.7	74.1	51.8	34.4	24.0	17.3	12.1	9.1	6.5	4.4	3.3	2.5	1.4	1.0	0.7	0.5
**Design Effect**	1.52	1.21	1.01	0.91	0.83	0.81	0.78	0.80	0.79	0.65	0.62	0.65	0.69	0.79	0.93	1.09
**Number**	2567	2185	1701	1249	962	754	574	450	341	255	200	149	78	43	22	10

Table B5124-2 Age Group = 18-80 Gender = FEMALE Occupational group = OVERALL Total cases = 1386

	Threshold dB HL≥															
	0	5	10	15	20	25	30	35	40	45	50	55	65	75	85	95
**Prevalence**	**94.2**	**71.5**	**46.8**	**29.7**	**19.9**	**14.4**	**10.2**	**7.7**	**5.6**	**3.7**	**2.7**	**1.9**	**1.1**	**0.7**	**0.4**	**0.2**
**Lower CI**	92.3	68.7	44.4	27.7	18.2	12.9	9.0	6.6	4.7	3.1	2.3	1.6	0.8	0.4	0.2	0.1
**Upper CI**	95.6	74.0	49.2	31.7	21.8	16.0	11.5	9.0	6.8	4.4	3.3	2.4	1.4	1.0	0.7	0.4
**Design Effect**	1.32	1.12	0.92	0.85	0.85	0.86	0.79	0.85	0.85	0.63	0.58	0.56	0.57	0.63	0.68	0.69
**Number**	1333	1104	812	566	418	333	258	208	162	123	98	75	43	24	13	6

Table B5124-3 Age Group = 18-80 Gender = MALE Occupational group = OVERALL Total cases = 1277

	Threshold dB HL≥															
	0	5	10	15	20	25	30	35	40	45	50	55	65	75	85	95
**Prevalence**	**92.6**	**72.9**	**53.3**	**36.2**	**25.6**	**18.1**	**12.2**	**8.8**	**6.0**	**4.0**	**3.0**	**2.2**	**1.0**	**0.7**	**0.4**	**0.3**
**Lower CI**	89.8	69.6	50.2	33.7	23.7	16.5	10.9	7.7	5.1	3.3	2.5	1.7	0.7	0.4	0.1	0.1
**Upper CI**	94.6	76.0	56.3	38.8	27.6	19.7	13.7	10.0	6.9	4.8	3.7	2.9	1.6	1.2	1.0	1.0
**Design Effect**	1.68	1.31	1.11	0.97	0.82	0.77	0.77	0.74	0.71	0.67	0.67	0.73	0.82	0.94	1.14	1.32
**Number**	1234	1081	889	683	544	421	316	242	179	132	102	74	35	19	9	4

Table B5124-4 Age Group = 18-30 Gender = OVERALL Occupational group = OVERALL Total cases = 401

	Threshold dB HL≥															
	0	5	10	15	20	25	30	35	40	45	50	55	65	75	85	95
**Prevalence**	**82.6**	**43.0**	**18.7**	**6.9**	**2.6**	**1.8**	**0.6**	**0.4**	**0.2**	**0.2**	**0.0**	**0.0**	**0.0**	**0.0**	**0.0**	**0.0**
**Lower CI**	77.5	37.4	14.8	4.7	1.4	1.0	0.3	0.1	0.1	0.1	0.0	0.0	0.0	0.0	0.0	0.0
**Upper CI**	86.8	48.7	23.3	10.0	4.6	3.2	1.2	0.8	0.7	0.6	0.1	0.1	0.1	0.9	0.9	0.9
**Design Effect**	1.25	1.17	1.12	1.05	0.98	0.84	0.54	0.55	0.52	0.53	0.20	0.20	0.28	-	-	-
**Number**	342	196	98	41	19	15	9	6	5	4	2	2	1	0	0	0

Table B5124-5 Age Group = 31-40 Gender = OVERALL Occupational group = OVERALL Total cases = 331

	Threshold dB HL≥															
	0	5	10	15	20	25	30	35	40	45	50	55	65	75	85	95
**Prevalence**	**92.8**	**60.5**	**26.9**	**13.8**	**5.6**	**2.8**	**2.3**	**1.4**	**1.1**	**1.1**	**1.0**	**0.8**	**0.7**	**0.7**	**0.6**	**0.6**
**Lower CI**	88.4	54.0	21.6	10.1	3.5	1.6	1.2	0.6	0.4	0.4	0.3	0.2	0.1	0.1	0.1	0.1
**Upper CI**	95.6	66.6	32.9	18.6	8.8	4.9	4.5	3.3	3.1	3.1	3.0	3.0	3.0	3.0	3.1	3.1
**Design Effect**	1.28	1.21	1.19	1.14	1.05	0.91	0.96	0.98	1.04	1.07	1.11	1.19	1.27	1.27	1.36	1.36
**Number**	313	219	113	62	30	19	15	10	8	7	5	3	2	2	1	1

Table B5124-6 Age Group = 41-50 Gender = OVERALL Occupational group = OVERALL Total cases = 450

	Threshold dB HL≥															
	0	5	10	15	20	25	30	35	40	45	50	55	65	75	85	95
**Prevalence**	**97.4**	**74.6**	**48.3**	**25.2**	**13.5**	**8.2**	**6.0**	**4.0**	**2.3**	**1.7**	**1.2**	**0.7**	**0.3**	**0.3**	**0.1**	**0.1**
**Lower CI**	95.2	69.9	43.3	21.4	10.9	6.4	4.4	2.8	1.5	1.1	0.8	0.4	0.1	0.1	0.0	0.0
**Upper CI**	98.6	78.8	53.3	29.6	16.7	10.5	8.0	5.8	3.5	2.6	2.0	1.6	0.9	0.8	0.6	0.3
**Design Effect**	1.11	1.12	1.09	1.02	0.92	0.81	0.83	0.81	0.69	0.65	0.60	0.71	0.63	0.65	0.60	0.49
**Number**	440	351	244	146	92	66	48	34	23	18	14	8	4	3	2	1

Table B5124-7 Age Group = 51-60 Gender = OVERALL Occupational group = OVERALL Total cases = 682

	Threshold dB HL≥															
	0	5	10	15	20	25	30	35	40	45	50	55	65	75	85	95
**Prevalence**	**98.7**	**90.8**	**68.9**	**43.3**	**28.8**	**18.9**	**11.0**	**7.8**	**5.6**	**4.0**	**2.9**	**2.1**	**0.9**	**0.4**	**0.1**	**0.1**
**Lower CI**	97.4	88.0	64.9	39.4	25.7	16.3	9.1	6.4	4.4	3.0	2.1	1.4	0.6	0.1	0.0	0.0
**Upper CI**	99.4	93.0	72.7	47.1	32.2	21.8	13.2	9.5	7.1	5.4	4.0	3.1	1.5	0.9	0.3	0.3
**Design Effect**	1.13	1.16	1.13	1.04	0.96	0.95	0.87	0.78	0.78	0.80	0.73	0.76	0.66	0.80	0.47	0.47
**Number**	674	630	512	365	272	192	129	102	77	56	44	32	17	5	2	2

Prevalence by age group, gender and occupational group for the better ear over 0.5, 1, 2, and 4 kHz

Table B5124-8 Age Group = 61-70 Gender = OVERALL Occupational group = OVERALL Total cases = 527

	Threshold dB HL≥															
	0	5	10	15	20	25	30	35	40	45	50	55	65	75	85	95
**Prevalence**	**100.0**	**97.7**	**86.3**	**65.1**	**50.8**	**36.8**	**25.3**	**16.2**	**10.7**	**7.4**	**5.7**	**4.1**	**2.3**	**1.4**	**1.0**	**0.6**
**Lower CI**	99.3	95.3	82.4	59.9	45.9	32.5	22.2	13.6	9.0	6.1	4.7	3.2	1.6	0.9	0.5	0.3
**Upper CI**	100.0	98.9	89.4	70.0	55.8	41.2	28.7	19.2	12.6	8.9	7.0	5.2	3.4	2.3	1.8	1.4
**Design Effect**	-	1.33	1.19	1.24	1.17	1.07	0.88	0.89	0.68	0.62	0.58	0.61	0.66	0.70	0.71	0.80
**Number**	527	518	470	383	320	256	198	150	110	83	68	51	28	16	11	5

Table B5124-9 Age Group = 71-80 Gender = OVERALL Occupational group = OVERALL Total cases = 272

	Threshold dB HL≥															
	0	5	10	15	20	25	30	35	40	45	50	55	65	75	85	95
**Prevalence**	**99.6**	**99.6**	**95.5**	**87.1**	**74.0**	**60.3**	**47.0**	**40.0**	**29.7**	**17.6**	**12.4**	**9.3**	**4.0**	**2.5**	**1.0**	**0.1**
**Lower CI**	98.0	98.0	90.0	80.6	66.6	52.9	40.2	33.3	23.8	14.3	10.0	7.2	2.8	1.6	0.4	0.0
**Upper CI**	99.9	99.9	98.0	91.6	80.2	67.3	53.9	47.1	36.3	21.5	15.3	11.8	5.6	4.0	2.3	0.8
**Design Effect**	0.98	0.98	1.54	1.38	1.32	1.25	1.17	1.20	1.16	0.81	0.68	0.65	0.60	0.62	0.72	0.63
**Number**	271	271	264	252	229	206	175	148	118	87	67	53	26	17	6	1

Table B5124-10 Age Group = 18-80 Gender = OVERALL Occupational group = NON-MANUAL Total cases = 1184

	Threshold dB HL≥															
	0	5	10	15	20	25	30	35	40	45	50	55	65	75	85	95
**Prevalence**	**91.8**	**67.7**	**43.3**	**27.3**	**19.9**	**14.2**	**9.9**	**7.2**	**5.2**	**2.7**	**2.0**	**1.2**	**0.7**	**0.4**	**0.1**	**0.1**
**Lower CI**	89.3	64.6	40.8	25.1	18.1	12.8	8.9	6.3	4.6	2.3	1.6	0.9	0.5	0.2	0.1	0.0
**Upper CI**	93.7	70.6	46.0	29.7	21.8	15.7	11.0	8.1	6.0	3.2	2.4	1.6	1.1	0.7	0.3	0.2
**Design Effect**	1.42	1.14	0.92	0.90	0.80	0.74	0.61	0.59	0.53	0.50	0.52	0.55	0.60	0.64	0.60	0.63
**Number**	1127	908	656	456	349	270	206	155	115	82	66	45	26	14	6	3

Table B5124-11 Age Group = 18-80 Gender = OVERALL Occupational group = MANUAL Total cases = 1475

	Threshold dB HL≥															
	0	5	10	15	20	25	30	35	40	45	50	55	65	75	85	95
**Prevalence**	**94.7**	**77.1**	**55.7**	**37.0**	**25.2**	**17.7**	**12.1**	**8.7**	**6.1**	**4.5**	**3.3**	**2.5**	**1.2**	**0.8**	**0.6**	**0.4**
**Lower CI**	92.4	73.9	52.8	34.6	23.2	16.1	10.9	7.8	5.3	3.8	2.7	2.0	0.9	0.6	0.3	0.2
**Upper CI**	96.4	80.0	58.7	39.5	27.2	19.4	13.4	9.8	7.0	5.3	3.9	3.0	1.7	1.3	1.0	0.8
**Design Effect**	1.72	1.42	1.17	1.00	0.92	0.86	0.74	0.70	0.68	0.72	0.61	0.62	0.66	0.74	0.84	0.94
**Number**	1437	1275	1043	791	611	483	368	295	226	173	134	104	52	29	16	7

Table B5124-12 Age Group = 18-30 Gender = FEMALE Occupational group = OVERALL Total cases = 224

	Threshold dB HL≥															
	0	5	10	15	20	25	30	35	40	45	50	55	65	75	85	95
**Prevalence**	**86.3**	**43.9**	**16.0**	**5.2**	**3.1**	**2.8**	**1.1**	**0.6**	**0.4**	**0.3**	**0.0**	**0.0**	**0.0**	**0.0**	**0.0**	**0.0**
**Lower CI**	80.4	36.8	11.8	3.3	1.8	1.5	0.5	0.2	0.1	0.1	0.0	0.0	0.0	0.0	0.0	0.0
**Upper CI**	90.7	51.3	21.4	8.4	5.5	5.1	2.2	1.5	1.2	1.1	1.7	1.7	1.7	1.7	1.7	1.7
**Design Effect**	1.15	1.13	1.00	0.86	0.80	0.81	0.57	0.59	0.57	0.60	-	-	-	-	-	-
**Number**	195	108	51	22	14	12	7	4	3	2	0	0	0	0	0	0

Table B5124-13 Age Group = 31-40 Gender = FEMALE Occupational group = OVERALL Total cases = 189

	Threshold dB HL≥															
	0	5	10	15	20	25	30	35	40	45	50	55	65	75	85	95
**Prevalence**	**91.7**	**55.6**	**22.8**	**11.5**	**3.6**	**2.9**	**2.1**	**1.2**	**0.9**	**0.8**	**0.8**	**0.5**	**0.2**	**0.2**	**0.0**	**0.0**
**Lower CI**	86.0	47.2	17.0	7.6	2.0	1.5	0.9	0.5	0.4	0.3	0.3	0.1	0.0	0.0	0.0	0.0
**Upper CI**	95.2	63.7	29.9	17.0	6.2	5.5	4.6	2.7	2.2	2.2	2.2	1.7	1.2	1.2	2.0	2.0
**Design Effect**	1.15	1.18	1.09	1.04	0.76	0.79	0.84	0.65	0.63	0.67	0.67	0.66	0.63	0.63		
**Number**	177	119	58	32	15	12	8	6	5	4	4	2	1	1	0	0

Table B5124-14 Age Group = 41-50 Gender = FEMALE Occupational group = OVERALL Total cases = 247

	Threshold dB HL≥															
	0	5	10	15	20	25	30	35	40	45	50	55	65	75	85	95
**Prevalence**	**96.3**	**72.6**	**43.6**	**19.0**	**10.1**	**7.2**	**5.3**	**3.7**	**2.5**	**1.6**	**1.2**	**0.9**	**0.2**	**0.1**	**0.1**	**0.1**
**Lower CI**	92.5	66.0	37.1	14.7	7.4	5.1	3.3	2.3	1.4	0.8	0.6	0.4	0.1	0.0	0.0	0.0
**Upper CI**	98.2	78.3	50.3	24.3	13.7	10.1	8.2	6.1	4.4	3.2	2.6	2.4	0.9	0.6	0.6	0.6
**Design Effect**	1.16	1.11	1.07	0.99	0.83	0.78	0.86	0.79	0.74	0.73	0.71	0.75	0.55	0.51	0.51	0.51
**Number**	240	187	123	63	41	32	23	18	13	9	7	5	2	1	1	1

Prevalence by age group, gender and occupational group for the better ear over 0.5, 1, 2, and 4 kHz

Table B5124-15 Age Group = 51-60 Gender = FEMALE Occupational group = OVERALL Total cases = 341

Threshold dB HL≥	0	5	10	15	20	25	30	35	40	45	50	55	65	75	85	95
**Prevalence**	**98.6**	**89.7**	**62.8**	**36.1**	**20.3**	**13.2**	**8.1**	**5.3**	**3.5**	**2.6**	**2.2**	**1.5**	**1.1**	**0.5**	**0.1**	**0.1**
**Lower CI**	96.3	85.5	56.9	31.0	16.5	10.2	5.9	3.8	2.4	1.7	1.4	0.8	0.5	0.2	0.0	0.0
**Upper CI**	99.5	92.8	68.4	41.5	24.6	17.0	11.1	7.4	5.0	3.9	3.6	2.7	2.1	1.5	0.5	0.5
**Design Effect**	1.14	1.12	1.13	1.03	0.95	0.95	0.89	0.75	0.65	0.66	0.70	0.69	0.67	0.78	0.46	0.46
**Number**	337	312	238	160	105	76	51	40	30	22	19	13	10	4	2	2

Table B5124-16 Age Group = 61-70 Gender = FEMALE Occupational group = OVERALL Total cases = 242

Threshold dB HL≥	0	5	10	15	20	25	30	35	40	45	50	55	65	75	85	95
**Prevalence**	**100.0**	**96.4**	**80.8**	**59.6**	**43.8**	**29.4**	**19.6**	**13.3**	**7.4**	**5.6**	**4.5**	**3.4**	**2.1**	**1.4**	**1.1**	**0.9**
**Lower CI**	98.4	92.1	74.4	52.4	37.2	24.3	16.3	9.8	5.7	4.3	3.3	2.4	1.2	0.7	0.5	0.3
**Upper CI**	100.0	98.4	85.9	66.4	50.6	35.2	23.3	18.0	9.4	7.4	6.0	4.9	3.6	2.9	2.6	2.4
**Design Effect**	0.00	1.28	1.16	1.15	1.08	0.95	0.70	0.96	0.56	0.54	0.51	0.54	0.63	0.71	0.74	0.79
**Number**	242	236	204	160	126	94	73	60	43	35	29	24	13	7	5	3

Table B5124-17 Age Group = 71-80 Gender = FEMALE Occupational group = OVERALL Total cases = 143

Threshold dB HL≥	0	5	10	15	20	25	30	35	40	45	50	55	65	75	85	95
**Prevalence**	**99.4**	**99.4**	**95.1**	**84.4**	**70.0**	**55.5**	**45.5**	**38.8**	**32.5**	**19.0**	**12.5**	**9.2**	**4.5**	**2.9**	**1.5**	**0.0**
**Lower CI**	96.8	96.8	85.8	74.7	58.8	45.1	36.5	29.9	24.1	14.4	9.5	6.7	3.0	1.7	0.6	0.0
**Upper CI**	99.9	99.9	98.4	90.8	79.3	65.5	54.8	48.5	42.3	24.7	16.4	12.3	6.9	5.1	3.6	2.6
**Design Effect**	0.91	0.91	1.63	1.35	1.40	1.28	1.14	1.19	1.21	0.80	0.64	0.59	0.56	0.59	0.70	-
**Number**	142	142	138	129	117	107	96	80	68	51	39	31	17	11	5	0

Table B5124-18 Age Group = 18-30 Gender = MALE Occupational group = OVERALL Total cases = 177

Threshold dB HL≥	0	5	10	15	20	25	30	35	40	45	50	55	65	75	85	95
**Prevalence**	**78.8**	**42.0**	**21.3**	**8.5**	**1.8**	**0.6**	**0.1**	**0.1**	**0.1**	**0.1**	**0.1**	**0.1**	**0.0**	**0.0**	**0.0**	**0.0**
**Lower CI**	70.1	33.7	15.1	4.9	0.5	0.1	0.0	0.0	0.0	0.0	0.0	0.0	0.0	0.0	0.0	0.0
**Upper CI**	85.5	50.9	29.1	14.3	5.9	3.0	0.2	0.2	0.2	0.2	0.2	0.2	0.2	2.1	2.1	2.1
**Design Effect**	1.29	1.20	1.18	1.12	1.18	0.94	0.19	0.19	0.19	0.19	0.19	0.19	0.27	-	-	-
**Number**	147	88	47	19	5	3	2	2	2	2	2	2	1	0	0	0

Table B5124-19 Age Group = 31-40 Gender = MALE Occupational group = OVERALL Total cases = 142

Threshold dB HL≥	0	5	10	15	20	25	30	35	40	45	50	55	65	75	85	95
**Prevalence**	**94.1**	**65.6**	**31.5**	**16.6**	**7.9**	**2.6**	**2.6**	**1.7**	**1.4**	**1.4**	**1.2**	**1.2**	**1.2**	**1.2**	**1.2**	**1.2**
**Lower CI**	85.8	55.4	22.8	10.5	4.2	1.0	1.0	0.5	0.3	0.3	0.2	0.2	0.2	0.2	0.2	0.2
**Upper CI**	97.7	74.6	41.7	25.2	14.4	6.9	6.9	6.3	6.2	6.2	6.5	6.5	6.5	6.5	6.5	6.5
**Design Effect**	1.45	1.25	1.26	1.21	1.13	1.02	1.02	1.14	1.22	1.22	1.31	1.31	1.31	1.31	1.31	1.31
**Number**	136	100	55	30	15	7	7	4	3	3	1	1	1	1	1	1

Table B5124-20 Age Group = 41-50 Gender = MALE Occupational group = OVERALL Total cases = 203

Threshold dB HL≥	0	5	10	15	20	25	30	35	40	45	50	55	65	75	85	95
**Prevalence**	**98.5**	**76.8**	**53.5**	**32.0**	**17.2**	**9.3**	**6.7**	**4.3**	**2.1**	**1.7**	**1.3**	**0.5**	**0.4**	**0.4**	**0.2**	**0.0**
**Lower CI**	95.6	69.7	45.9	25.7	12.7	6.5	4.4	2.5	1.2	1.0	0.7	0.2	0.1	0.1	0.0	0.0
**Upper CI**	99.5	82.7	61.0	39.2	22.8	13.2	10.0	7.3	3.7	3.1	2.2	1.6	1.6	1.6	1.1	1.9
**Design Effect**	1.04	1.13	1.12	1.06	0.97	0.84	0.80	0.83	0.62	0.56	0.45	0.63	0.67	0.67	0.63	-
**Number**	200	164	121	83	51	34	25	16	10	9	7	3	2	2	1	0

Table B5124-21 Age Group = 51-60 Gender = MALE Occupational group = OVERALL Total cases = 341

Threshold dB HL≥	0	5	10	15	20	25	30	35	40	45	50	55	65	75	85	95
**Prevalence**	**98.9**	**92.0**	**75.6**	**51.1**	**38.4**	**25.3**	**14.2**	**10.7**	**7.9**	**5.6**	**3.7**	**2.9**	**0.8**	**0.2**	**0.0**	**0.0**
**Lower CI**	96.8	87.9	70.0	45.5	33.3	21.1	11.3	8.3	5.8	3.8	2.5	1.7	0.4	0.0	0.0	0.0
**Upper CI**	99.6	94.9	80.5	56.7	43.7	30.1	17.8	13.7	10.7	8.2	5.6	4.7	1.6	1.1	1.1	1.1
**Design Effect**	1.12	1.19	1.16	1.06	1.01	0.98	0.88	0.82	0.85	0.89	0.77	0.81	0.64	0.83	-	-
**Number**	337	318	274	205	167	116	78	62	47	34	25	19	7	1	0	0

Prevalence by age group, gender and occupational group for the better ear over 0.5, 1, 2, and 4 kHz

Table B5124-22 Age Group = 61-70 Gender = MALE Occupational group = OVERALL Total cases = 285

	Threshold dB HL≥															
	0	5	10	15	20	25	30	35	40	45	50	55	65	75	85	95
**Prevalence**	**100.0**	**99.2**	**92.6**	**71.6**	**59.1**	**45.3**	**32.1**	**19.7**	**14.7**	**9.6**	**7.2**	**4.9**	**2.7**	**1.5**	**0.9**	**0.3**
**Lower CI**	98.7	97.5	88.2	64.0	51.6	38.4	26.6	16.3	11.7	7.4	5.5	3.5	1.7	0.8	0.4	0.1
**Upper CI**	100.0	99.8	95.4	78.2	66.2	52.4	38.2	23.6	18.3	12.2	9.4	6.9	4.3	2.8	1.9	1.2
**Design Effect**	-	0.96	1.15	1.37	1.30	1.22	1.07	0.80	0.80	0.70	0.65	0.68	0.69	0.69	0.62	0.69
**Number**	285	282	266	223	194	162	125	90	67	48	39	27	15	9	6	2

Table B5124-23 Age Group = 71-80 Gender = MALE Occupational group = OVERALL Total cases = 129

	Threshold dB HL≥															
	0	5	10	15	20	25	30	35	40	45	50	55	65	75	85	95
**Prevalence**	**100.0**	**100.0**	**96.0**	**90.8**	**78.5**	**66.6**	**48.6**	**41.5**	**25.2**	**15.8**	**12.7**	**9.6**	**3.3**	**2.0**	**0.4**	**0.4**
**Lower CI**	97.1	97.1	90.0	81.9	70.8	56.8	38.8	32.1	18.9	11.7	8.9	6.4	1.8	1.0	0.1	0.1
**Upper CI**	100.0	100.0	98.5	95.6	84.6	75.1	58.4	51.5	32.9	20.9	17.7	14.2	6.0	4.3	2.0	2.0
**Design Effect**	-	-	1.16	1.35	0.98	1.14	1.16	1.17	0.95	0.73	0.76	0.76	0.66	0.65	0.67	0.67
**Number**	129	129	126	123	112	99	79	68	50	36	28	22	9	6	1	1

Table B5124-24 Age Group = 18-30 Gender = OVERALL Occupational group = NON-MANUAL Total cases = 181

	Threshold dB HL≥															
	0	5	10	15	20	25	30	35	40	45	50	55	65	75	85	95
**Prevalence**	**76.0**	**34.5**	**10.0**	**3.9**	**1.5**	**1.0**	**0.7**	**0.5**	**0.1**	**0.1**	**0.1**	**0.1**	**0.1**	**0.0**	**0.0**	**0.0**
**Lower CI**	67.6	27.2	6.2	1.5	0.7	0.3	0.2	0.1	0.0	0.0	0.0	0.0	0.0	0.0	0.0	0.0
**Upper CI**	82.8	42.7	15.8	9.4	3.3	3.0	2.4	2.4	2.2	2.2	2.2	2.2	2.2	2.1	2.1	2.1
**Design Effect**	1.24	1.13	1.09	1.29	0.68	0.79	0.79	0.85	0.00	0.00	0.00	0.00	0.00	-	-	-
**Number**	145	71	26	9	6	4	3	2	1	1	1	1	1	0	0	0

Table B5124-25 Age Group = 31-40 Gender = OVERALL Occupational group = NON-MANUAL Total cases = 148

	Threshold dB HL≥															
	0	5	10	15	20	25	30	35	40	45	50	55	65	75	85	95
**Prevalence**	**94.6**	**58.7**	**22.3**	**8.7**	**3.7**	**2.0**	**1.0**	**0.4**	**0.4**	**0.3**	**0.3**	**0.0**	**0.0**	**0.0**	**0.0**	**0.0**
**Lower CI**	90.3	48.8	15.0	4.9	1.6	0.9	0.3	0.1	0.1	0.1	0.1	0.0	0.0	0.0	0.0	0.0
**Upper CI**	97.1	67.9	31.7	15.0	8.7	4.2	3.6	1.6	1.6	1.7	1.7	2.5	2.5	2.5	2.5	2.5
**Design Effect**	0.90	1.23	1.26	1.10	1.09	0.68	0.85	0.59	0.59	0.67	0.67	-	-	-	-	-
**Number**	140	90	36	17	8	6	3	2	2	1	1	0	0	0	0	0

Table B5124-26 Age Group = 41-50 Gender = OVERALL Occupational group = NON-MANUAL Total cases = 188

	Threshold dB HL≥															
	0	5	10	15	20	25	30	35	40	45	50	55	65	75	85	95
**Prevalence**	**97.5**	**67.0**	**36.5**	**17.3**	**11.4**	**5.5**	**4.3**	**2.0**	**0.7**	**0.4**	**0.4**	**0.1**	**0.0**	**0.0**	**0.0**	**0.0**
**Lower CI**	94.3	59.4	29.8	12.5	7.6	3.4	2.4	1.5	0.3	0.1	0.1	0.0	0.0	0.0	0.0	0.0
**Upper CI**	98.9	73.8	43.7	23.4	16.7	8.8	7.7	2.7	1.4	1.3	1.3	0.7	2.0	2.0	2.0	2.0
**Design Effect**	0.97	1.08	1.02	1.01	0.99	0.81	0.88	0.28	0.44	0.60	0.60	0.48	-	-	-	-
**Number**	183	126	76	40	26	17	11	6	3	2	2	1	0	0	0	0

Table B5124-27 Age Group = 51-60 Gender = OVERALL Occupational group = NON-MANUAL Total cases = 317

	Threshold dB HL≥															
	0	5	10	15	20	25	30	35	40	45	50	55	65	75	85	95
**Prevalence**	**98.1**	**87.9**	**62.8**	**35.7**	**22.1**	**11.9**	**6.0**	**4.7**	**3.0**	**2.3**	**1.9**	**1.2**	**0.7**	**0.4**	**0.1**	**0.1**
**Lower CI**	95.9	83.7	56.7	30.6	18.3	8.4	4.5	3.6	2.1	1.6	1.2	0.6	0.3	0.1	0.0	0.0
**Upper CI**	99.2	91.2	68.5	41.2	26.4	16.6	7.9	6.2	4.2	3.4	2.9	2.3	1.6	1.3	0.4	0.4
**Design Effect**	1.02	1.04	1.12	1.01	0.89	1.14	0.64	0.57	0.56	0.53	0.55	0.67	0.68	0.74	0.46	0.46
**Number**	310	278	208	134	91	60	42	35	23	18	15	9	5	3	1	1

Table B5124-28 Age Group = 61-70 Gender = OVERALL Occupational group = NON-MANUAL Total cases = 227

	Threshold dB HL≥															
	0	5	10	15	20	25	30	35	40	45	50	55	65	75	85	95
**Prevalence**	**100.0**	**96.9**	**83.9**	**59.4**	**47.6**	**37.1**	**24.1**	**14.3**	**10.1**	**7.9**	**5.5**	**3.1**	**2.2**	**1.4**	**0.6**	**0.4**
**Lower CI**	98.3	92.7	77.9	51.0	39.9	30.1	20.4	11.1	7.8	5.9	3.9	1.9	1.1	0.5	0.2	0.1
**Upper CI**	100.0	98.7	88.6	67.3	55.5	44.7	28.2	18.2	13.1	10.5	7.6	5.1	4.2	3.5	1.8	1.6
**Design Effect**	-	1.25	1.12	1.29	1.22	1.18	0.70	0.78	0.68	0.65	0.62	0.68	0.77	0.88	0.69	0.75
**Number**	227	221	193	147	121	97	74	49	37	31	26	18	11	6	4	2

Prevalence by age group, gender and occupational group for the better ear over 0.5, 1, 2, and 4 kHz

Table B5124-29 Age Group = 71-80 Gender = OVERALL Occupational group = NON-MANUAL Total cases = 123

	Threshold dB HL≥															
	**0**	**5**	**10**	**15**	**20**	**25**	**30**	**35**	**40**	**45**	**50**	**55**	**65**	**75**	**85**	**95**
**Prevalence**	**99.1**	**99.1**	**95.0**	**84.8**	**73.3**	**60.8**	**48.7**	**41.3**	**33.9**	**11.7**	**8.4**	**5.6**	**3.3**	**1.7**	**0.4**	**0.0**
**Lower CI**	95.1	95.1	89.0	72.9	62.0	52.6	41.3	34.5	27.9	9.3	6.1	3.6	1.8	0.7	0.1	0.0
**Upper CI**	99.8	99.8	97.8	92.0	82.2	68.5	56.2	48.5	40.4	14.6	11.6	8.7	6.2	3.8	2.0	3.0
**Design Effect**	1.05	1.05	1.07	1.51	1.32	0.94	0.85	0.82	0.75	0.47	0.56	0.62	0.67	0.63	0.66	-
**Number**	122	122	117	109	97	86	73	61	49	29	21	16	9	5	1	0

Table B5124-30 Age Group = 18-30 Gender = OVERALL Occupational group = MANUAL Total cases = 218

	Threshold dB HL≥															
	**0**	**5**	**10**	**15**	**20**	**25**	**30**	**35**	**40**	**45**	**50**	**55**	**65**	**75**	**85**	**95**
**Prevalence**	**87.2**	**52.7**	**26.2**	**9.3**	**3.3**	**2.5**	**0.6**	**0.3**	**0.3**	**0.3**	**0.0**	**0.0**	**0.0**	**0.0**	**0.0**	**0.0**
**Lower CI**	79.5	43.8	19.4	5.9	1.6	1.2	0.3	0.1	0.1	0.1	0.0	0.0	0.0	0.0	0.0	0.0
**Upper CI**	92.2	61.6	34.4	14.3	6.7	5.3	1.3	0.9	0.9	0.9	0.2	0.2	1.7	1.7	1.7	1.7
**Design Effect**	1.42	1.36	1.29	1.07	1.01	0.93	0.47	0.47	0.47	0.49	0.26	0.26	-	-	-	-
**Number**	196	125	72	32	13	11	6	4	4	3	1	1	0	0	0	0

Table B5124-31 Age Group = 31-40 Gender = OVERALL Occupational group = MANUAL Total cases = 183

	Threshold dB HL≥															
	**0**	**5**	**10**	**15**	**20**	**25**	**30**	**35**	**40**	**45**	**50**	**55**	**65**	**75**	**85**	**95**
**Prevalence**	**92.2**	**63.4**	**30.3**	**18.4**	**7.9**	**3.6**	**3.4**	**2.0**	**1.5**	**1.5**	**1.3**	**1.3**	**1.1**	**1.1**	**0.9**	**0.9**
**Lower CI**	85.3	54.5	22.8	12.4	4.2	1.7	1.6	0.8	0.5	0.5	0.4	0.3	0.2	0.2	0.2	0.2
**Upper CI**	96.0	71.6	39.0	26.3	14.3	7.4	7.3	5.2	4.8	4.8	4.8	4.8	4.9	4.9	5.0	5.0
**Design Effect**	1.35	1.24	1.23	1.24	1.27	1.01	1.02	0.98	1.05	1.05	1.11	1.15	1.22	1.22	1.30	1.30
**Number**	173	129	77	45	22	13	12	8	6	6	4	3	2	2	1	1

Table B5124-32 Age Group = 41-50 Gender = OVERALL Occupational group = MANUAL Total cases = 262

	Threshold dB HL≥															
	**0**	**5**	**10**	**15**	**20**	**25**	**30**	**35**	**40**	**45**	**50**	**55**	**65**	**75**	**85**	**95**
**Prevalence**	**97.5**	**82.2**	**60.8**	**32.4**	**16.6**	**10.0**	**7.3**	**5.3**	**3.1**	**2.2**	**1.6**	**1.0**	**0.4**	**0.3**	**0.2**	**0.1**
**Lower CI**	94.1	75.6	53.6	26.2	12.3	7.3	5.1	3.5	2.0	1.3	0.9	0.4	0.2	0.1	0.1	0.0
**Upper CI**	99.0	87.4	67.5	39.3	22.0	13.7	10.4	7.9	4.6	3.6	2.8	2.4	1.1	0.9	0.7	0.5
**Design Effect**	1.19	1.28	1.19	1.16	1.07	0.88	0.82	0.80	0.61	0.64	0.63	0.73	0.52	0.52	0.52	0.48
**Number**	257	225	168	106	66	49	37	28	20	16	12	7	4	3	2	1

Table B5124-33 Age Group = 51-60 Gender = OVERALL Occupational group = MANUAL Total cases = 365

	Threshold dB HL≥															
	**0**	**5**	**10**	**15**	**20**	**25**	**30**	**35**	**40**	**45**	**50**	**55**	**65**	**75**	**85**	**95**
**Prevalence**	**99.6**	**94.6**	**75.1**	**48.9**	**34.8**	**25.0**	**14.3**	**9.7**	**6.7**	**4.8**	**3.3**	**2.5**	**1.0**	**0.2**	**0.1**	**0.1**
**Lower CI**	98.0	90.9	69.2	43.3	29.9	20.6	11.3	7.5	5.0	3.3	2.3	1.7	0.6	0.1	0.0	0.0
**Upper CI**	99.9	96.9	80.2	54.6	40.0	29.9	17.9	12.3	9.0	6.9	4.7	3.7	1.8	0.8	0.5	0.5
**Design Effect**	1.14	1.28	1.24	1.11	1.04	1.06	0.92	0.78	0.76	0.81	0.66	0.62	0.57	0.64	0.56	0.56
**Number**	364	352	304	231	181	132	87	67	54	38	29	23	12	2	1	1

Table B5124-34 Age Group = 61-70 Gender = OVERALL Occupational group = MANUAL Total cases = 299

	Threshold dB HL≥															
	**0**	**5**	**10**	**15**	**20**	**25**	**30**	**35**	**40**	**45**	**50**	**55**	**65**	**75**	**85**	**95**
**Prevalence**	**100.0**	**98.1**	**88.2**	**69.3**	**52.9**	**36.9**	**26.1**	**17.5**	**11.9**	**7.7**	**6.1**	**4.7**	**2.8**	**1.9**	**1.5**	**1.1**
**Lower CI**	98.7	93.7	82.6	61.8	45.9	31.3	21.9	14.2	9.5	6.2	4.8	3.7	2.1	1.3	1.1	0.8
**Upper CI**	100.0	99.4	92.2	75.8	59.7	42.8	30.9	21.5	14.7	9.6	7.7	6.0	3.7	2.6	2.1	1.6
**Design Effect**	0.00	1.58	1.32	1.35	1.24	1.06	0.90	0.85	0.70	0.56	0.53	0.49	0.44	0.40	0.38	0.32
**Number**	299	296	276	235	198	159	124	101	73	52	42	33	17	10	7	3

Table B5124-35 Age Group = 71-80 Gender = OVERALL Occupational group = MANUAL Total cases = 148

	Threshold dB HL≥															
	**0**	**5**	**10**	**15**	**20**	**25**	**30**	**35**	**40**	**45**	**50**	**55**	**65**	**75**	**85**	**95**
**Prevalence**	**100.0**	**100.0**	**97.3**	**91.4**	**77.2**	**59.9**	**48.1**	**38.7**	**27.8**	**21.2**	**14.4**	**11.0**	**4.4**	**3.2**	**1.5**	**0.2**
**Lower CI**	97.5	97.5	86.8	82.2	66.7	49.3	39.7	30.7	21.2	14.9	11.0	8.2	2.9	1.9	0.6	0.0
**Upper CI**	100.0	100.0	99.5	96.0	85.1	69.6	56.6	47.4	35.5	29.3	18.7	14.6	6.8	5.5	3.7	1.3
**Design Effect**	-	-	1.92	1.48	1.38	1.31	1.07	1.08	1.00	1.10	0.68	0.64	0.57	0.62	0.72	0.59
**Number**	148	148	146	142	131	119	102	87	69	58	46	37	17	12	5	1

Prevalence by age group, gender and occupational group for the better ear over 0.5, 1, 2, and 4 kHz

Table B5124-36 Age Group = 18-80 Gender = FEMALE Occupational group = NON-MANUAL Total cases = 670

	Threshold dB HL≥															
	**0**	5	**10**	15	**20**	25	**30**	35	**40**	45	**50**	55	**65**	75	**85**	95
**Prevalence**	**93.0**	**67.4**	**43.0**	**26.4**	**18.4**	**13.6**	**9.9**	**7.6**	**6.1**	**2.7**	**2.1**	**1.5**	**1.0**	**0.6**	**0.2**	**0.1**
**Lower CI**	90.4	63.7	39.8	23.7	16.1	11.9	8.6	6.5	5.3	2.1	1.6	1.1	0.7	0.3	0.1	0.0
**Upper CI**	94.9	70.9	46.1	29.4	20.9	15.5	11.3	8.7	7.0	3.3	2.7	2.1	1.6	1.0	0.6	0.5
**Design Effect**	1.14	1.02	0.84	0.85	0.81	0.69	0.60	0.55	0.48	0.48	0.49	0.55	0.61	0.62	0.64	0.66
**Number**	636	502	353	237	174	140	108	86	69	50	43	31	20	11	5	3

Table B5124-37 Age Group = 18-80 Gender = MALE Occupational group = NON-MANUAL Total cases = 514

	Threshold dB HL≥															
	**0**	5	**10**	15	**20**	25	**30**	35	**40**	45	**50**	55	**65**	75	**85**	95
**Prevalence**	**90.4**	**67.9**	**43.8**	**28.3**	**21.5**	**14.8**	**9.8**	**6.7**	**4.3**	**2.7**	**1.8**	**0.9**	**0.4**	**0.3**	**0.1**	**0.0**
**Lower CI**	85.6	62.7	39.6	24.8	18.9	12.6	8.4	5.5	3.4	2.1	1.3	0.5	0.2	0.1	0.0	0.0
**Upper CI**	93.7	72.6	48.0	32.1	24.4	17.3	11.5	8.2	5.5	3.6	2.6	1.5	1.0	0.8	0.3	0.7
**Design Effect**	1.56	1.23	0.98	0.94	0.79	0.77	0.60	0.62	0.58	0.51	0.55	0.57	0.63	0.72	0.54	-
**Number**	491	406	303	219	175	130	98	69	46	32	23	14	6	3	1	0

Table B5124-38 Age Group = 18-80 Gender = FEMALE Occupational group = MANUAL Total cases = 715

	Threshold dB HL≥															
	**0**	5	**10**	15	**20**	25	**30**	35	**40**	45	**50**	55	**65**	75	**85**	95
**Prevalence**	**95.4**	**76.4**	**51.2**	**32.3**	**21.2**	**15.2**	**10.8**	**7.8**	**5.5**	**4.2**	**2.9**	**2.2**	**1.1**	**0.8**	**0.6**	**0.3**
**Lower CI**	92.2	71.9	47.3	29.2	18.7	13.1	9.4	6.6	4.5	3.3	2.3	1.7	0.8	0.6	0.4	0.2
**Upper CI**	97.3	80.3	55.1	35.5	24.0	17.7	12.5	9.2	6.6	5.4	3.6	2.7	1.5	1.2	0.9	0.4
**Design Effect**	1.62	1.35	1.07	0.92	0.88	0.88	0.67	0.67	0.65	0.71	0.53	0.46	0.44	0.46	0.47	0.18
**Number**	696	601	458	328	243	193	150	122	93	73	55	44	23	13	8	3

Table B5124-39 Age Group = 18-80 Gender = MALE Occupational group = MANUAL Total cases = 760

	Threshold dB HL≥															
	**0**	5	**10**	15	**20**	25	**30**	35	**40**	45	**50**	55	**65**	75	**85**	95
**Prevalence**	**94.0**	**78.0**	**60.8**	**42.4**	**29.5**	**20.4**	**13.5**	**9.8**	**6.8**	**4.8**	**3.7**	**2.9**	**1.4**	**0.9**	**0.5**	**0.4**
**Lower CI**	90.1	73.3	56.2	38.7	26.5	18.1	11.7	8.3	5.7	3.8	2.9	2.1	0.8	0.4	0.2	0.1
**Upper CI**	96.4	82.1	65.2	46.3	32.7	22.9	15.6	11.4	8.1	6.0	4.8	3.9	2.2	1.8	1.6	1.6
**Design Effect**	1.83	1.50	1.30	1.09	0.96	0.85	0.80	0.72	0.70	0.71	0.70	0.74	0.83	0.97	1.16	1.29
**Number**	741	674	585	463	368	290	218	173	133	100	79	60	29	16	8	4

Table B5124-40 Age Group = 18-30 Gender = FEMALE Occupational group = NON-MANUAL Total cases = 115

	Threshold dB HL≥															
	**0**	5	**10**	15	**20**	25	**30**	35	**40**	45	**50**	55	**65**	75	**85**	95
**Prevalence**	**83.5**	**37.4**	**11.2**	**4.0**	**2.8**	**1.8**	**1.1**	**0.8**	**0.0**	**0.0**	**0.0**	**0.0**	**0.0**	**0.0**	**0.0**	**0.0**
**Lower CI**	75.5	28.7	7.0	2.1	1.2	0.6	0.3	0.1	0.0	0.0	0.0	0.0	0.0	0.0	0.0	0.0
**Upper CI**	89.2	47.0	17.4	7.7	6.1	5.5	4.5	4.5	3.2	3.2	3.2	3.2	3.2	3.2	3.2	3.2
**Design Effect**	1.01	1.05	0.90	0.76	0.77	0.90	0.89	0.98	-	-	-	-	-	-	-	-
**Number**	96	47	18	7	5	3	2	1	0	0	0	0	0	0	0	0

Table B5124-41 Age Group = 31-40 Gender = FEMALE Occupational group = NON-MANUAL Total cases = 86

	Threshold dB HL≥															
	**0**	5	**10**	15	**20**	25	**30**	35	**40**	45	**50**	55	**65**	75	**85**	95
**Prevalence**	**89.9**	**51.4**	**19.4**	**6.8**	**2.5**	**2.5**	**0.7**	**0.7**	**0.7**	**0.6**	**0.6**	**0.0**	**0.0**	**0.0**	**0.0**	**0.0**
**Lower CI**	82.1	40.0	11.9	3.0	1.3	1.3	0.2	0.2	0.2	0.1	0.1	0.0	0.0	0.0	0.0	0.0
**Upper CI**	94.5	62.6	30.1	14.5	4.7	4.7	3.0	3.0	3.0	3.2	3.2	4.3	4.3	4.3	4.3	4.3
**Design Effect**	0.96	1.10	1.10	1.04	0.50	0.50	0.62	0.62	0.62	0.71	0.71	-	-	-	-	-
**Number**	78	47	19	8	5	5	2	2	2	1	1	0	0	0	0	0

Table B5124-42 Age Group = 41-50 Gender = FEMALE Occupational group = NON-MANUAL Total cases = 112

	Threshold dB HL≥															
	**0**	5	**10**	15	**20**	25	**30**	35	**40**	45	**50**	55	**65**	75	**85**	95
**Prevalence**	**96.8**	**65.6**	**36.1**	**13.6**	**6.2**	**4.4**	**2.4**	**1.9**	**0.6**	**0.0**	**0.0**	**0.0**	**0.0**	**0.0**	**0.0**	**0.0**
**Lower CI**	91.2	55.9	28.1	9.4	4.8	3.0	1.6	1.5	0.1	0.0	0.0	0.0	0.0	0.0	0.0	0.0
**Upper CI**	98.9	74.2	44.9	19.2	8.1	6.4	3.7	2.5	4.3	3.3	3.3	3.3	3.3	3.3	3.3	3.3
**Design Effect**	1.08	1.06	0.96	0.77	0.37	0.44	0.37	0.20	0.00	-	-	-	-	-	-	-
**Number**	109	75	46	20	11	9	4	3	1	0	0	0	0	0	0	0

Prevalence by age group, gender and occupational group for the better ear over 0.5, 1, 2, and 4 kHz

Table B5124-43 Age Group = 51-60 Gender = FEMALE Occupational group = NON-MANUAL Total cases = 175

	Threshold dB HL≥															
	**0**	**5**	**10**	**15**	**20**	**25**	**30**	**35**	**40**	**45**	**50**	**55**	**65**	**75**	**85**	**95**
**Prevalence**	**98.3**	**88.9**	**59.8**	**33.2**	**16.4**	**8.7**	**5.5**	**4.1**	**3.6**	**3.0**	**2.7**	**1.7**	**1.2**	**0.8**	**0.1**	**0.1**
**Lower CI**	94.9	83.2	51.5	26.3	11.9	5.8	3.6	2.8	2.3	2.0	1.7	0.7	0.5	0.2	0.0	0.0
**Upper CI**	99.5	92.8	67.6	40.8	22.1	12.7	8.3	6.1	5.5	4.5	4.5	3.7	3.0	2.5	0.7	0.7
**Design Effect**	1.05	1.03	1.12	1.05	0.93	0.82	0.68	0.55	0.56	0.49	0.56	0.73	0.70	0.76	0.48	0.48
**Number**	172	156	111	72	43	29	21	17	14	12	11	6	5	3	1	1

Table B5124-44 Age Group = 61-70 Gender = FEMALE Occupational group = NON-MANUAL Total cases = 115

	Threshold dB HL≥															
	**0**	**5**	**10**	**15**	**20**	**25**	**30**	**35**	**40**	**45**	**50**	**55**	**65**	**75**	**85**	**95**
**Prevalence**	**100.0**	**94.8**	**79.9**	**57.6**	**43.5**	**31.1**	**21.2**	**11.5**	**6.7**	**5.5**	**4.1**	**3.1**	**2.0**	**1.2**	**0.8**	**0.7**
**Lower CI**	96.8	87.2	70.4	47.8	34.6	23.5	15.8	7.6	4.5	3.5	2.8	1.9	0.9	0.4	0.2	0.2
**Upper CI**	100.0	98.0	86.9	66.8	52.9	39.8	27.7	17.2	9.9	8.7	6.0	5.2	4.2	3.5	3.0	3.1
**Design Effect**	0.00	1.24	1.14	1.07	1.02	0.98	0.80	0.82	0.58	0.61	0.44	0.52	0.60	0.69	0.70	0.75
**Number**	115	111	95	72	57	44	36	26	20	17	15	12	7	4	3	2

Table B5124-45 Age Group = 71-80 Gender = FEMALE Occupational group = NON-MANUAL Total cases = 67

	Threshold dB HL≥															
	**0**	**5**	**10**	**15**	**20**	**25**	**30**	**35**	**40**	**45**	**50**	**55**	**65**	**75**	**85**	**95**
**Prevalence**	**98.6**	**98.6**	**95.5**	**82.3**	**72.5**	**61.6**	**50.9**	**43.9**	**40.4**	**11.6**	**8.8**	**7.3**	**5.0**	**2.3**	**0.6**	**0.0**
**Lower CI**	92.2	92.2	85.0	63.7	54.8	50.0	41.8	36.2	32.6	8.6	5.8	4.4	2.6	0.9	0.1	0.0
**Upper CI**	99.7	99.7	98.8	92.5	85.2	72.1	60.0	52.0	48.6	15.5	13.1	11.9	9.6	5.5	3.4	5.4
**Design Effect**	0.99	0.99	1.23	1.62	1.49	0.97	0.77	0.67	0.69	0.45	0.54	0.59	0.65	0.59	0.64	-
**Number**	66	66	64	58	53	50	43	37	32	20	16	13	8	4	1	0

Table B5124-46 Age Group = 18-30 Gender = MALE Occupational group = NON-MANUAL Total cases = 66

	Threshold dB HL≥															
	**0**	**5**	**10**	**15**	**20**	**25**	**30**	**35**	**40**	**45**	**50**	**55**	**65**	**75**	**85**	**95**
**Prevalence**	**68.1**	**31.5**	**8.4**	**3.6**	**0.1**	**0.1**	**0.1**	**0.1**	**0.1**	**0.1**	**0.1**	**0.1**	**0.1**	**0.0**	**0.0**	**0.0**
**Lower CI**	53.1	20.4	3.3	0.6	0.0	0.0	0.0	0.0	0.0	0.0	0.0	0.0	0.0	0.0	0.0	0.0
**Upper CI**	80.1	45.2	19.8	17.3	5.7	5.7	5.7	5.7	5.7	5.7	5.7	5.7	5.7	5.5	5.5	5.5
**Design Effect**	1.25	1.14	1.19	1.51	0.00	0.00	0.00	0.00	0.00	0.00	0.00	0.00	0.00	-	-	-
**Number**	49	24	8	2	1	1	1	1	1	1	1	1	1	0	0	0

Table B5124-47 Age Group = 31-40 Gender = MALE Occupational group = NON-MANUAL Total cases = 62

	Threshold dB HL≥															
	**0**	**5**	**10**	**15**	**20**	**25**	**30**	**35**	**40**	**45**	**50**	**55**	**65**	**75**	**85**	**95**
**Prevalence**	**100.0**	**66.3**	**25.7**	**11.1**	**5.1**	**1.3**	**1.3**	**0.0**	**0.0**	**0.0**	**0.0**	**0.0**	**0.0**	**0.0**	**0.0**	**0.0**
**Lower CI**	94.2	49.1	13.9	5.0	1.4	0.2	0.2	0.0	0.0	0.0	0.0	0.0	0.0	0.0	0.0	0.0
**Upper CI**	100.0	80.1	42.5	22.8	16.6	6.9	6.9	5.8	5.8	5.8	5.8	5.8	5.8	5.8	5.8	5.8
**Design Effect**	-	1.38	1.37	1.12	1.25	0.89	0.89	-	-	-	-	-	-	-	-	-
Number	62	43	17	9	3	1	1	0	0	0	0	0	0	0	0	0

Table B5124-48 Age Group = 41-50 Gender = MALE Occupational group = NON-MANUAL Total cases = 76

	Threshold dB HL≥															
	**0**	**5**	**10**	**15**	**20**	**25**	**30**	**35**	**40**	**45**	**50**	**55**	**65**	**75**	**85**	**95**
**Prevalence**	**98.3**	**68.5**	**37.1**	**21.4**	**17.2**	**6.8**	**6.5**	**2.1**	**0.7**	**0.7**	**0.7**	**0.3**	**0.0**	**0.0**	**0.0**	**0.0**
**Lower CI**	93.7	56.3	26.7	13.0	9.7	3.1	2.9	1.3	0.2	0.2	0.2	0.0	0.0	0.0	0.0	0.0
**Upper CI**	99.5	78.5	48.9	33.1	28.5	14.0	13.9	3.5	2.8	2.8	2.8	1.4	4.8	4.8	4.8	4.8
**Design Effect**	0.83	1.09	1.05	1.11	1.11	0.93	0.95	0.33	0.56	0.56	0.56	0.44	-	-	-	-
**Number**	74	51	30	20	15	8	7	3	2	2	2	1	0	0	0	0

Table B5124-49 Age Group = 51-60 Gender = MALE Occupational group = NON-MANUAL Total cases = 142

	Threshold dB HL≥															
	**0**	**5**	**10**	**15**	**20**	**25**	**30**	**35**	**40**	**45**	**50**	**55**	**65**	**75**	**85**	**95**
**Prevalence**	**97.9**	**86.8**	**66.0**	**38.4**	**28.4**	**15.6**	**6.5**	**5.4**	**2.3**	**1.5**	**0.9**	**0.6**	**0.0**	**0.0**	**0.0**	**0.0**
**Lower CI**	94.0	80.0	56.9	31.1	22.5	9.4	4.4	3.6	1.2	0.7	0.3	0.2	0.0	0.0	0.0	0.0
**Upper CI**	99.3	91.5	74.1	46.3	35.2	24.8	9.4	8.0	4.3	3.4	2.3	1.8	2.6	2.6	2.6	2.6
**Design Effect**	0.99	1.04	1.12	0.96	0.86	1.30	0.61	0.59	0.58	0.63	0.57	0.55	-	-	-	-
**Number**	138	122	97	62	48	31	21	18	9	6	4	3	0	0	0	0

Prevalence by age group, gender and occupational group for the better ear over 0.5, 1, 2, and 4 kHz

Table B5124-50 Age Group = 61-70 Gender = MALE Occupational group = NON-MANUAL Total cases = 112

Threshold dB HL≥	0	5	10	15	20	25	30	35	40	45	50	55	65	75	85	95
**Prevalence**	**100.0**	**99.6**	**88.7**	**62.0**	**53.1**	**44.2**	**27.1**	**17.5**	**14.0**	**10.7**	**7.0**	**3.2**	**2.4**	**1.6**	**0.4**	**0.0**
**Lower CI**	96.7	98.7	81.0	47.5	39.8	31.9	22.3	12.8	9.9	7.5	4.4	1.4	0.8	0.4	0.1	0.0
**Upper CI**	100.0	99.9	93.6	74.6	66.1	57.3	32.6	23.4	19.4	15.1	11.1	7.4	6.9	6.5	2.1	3.3
**Design Effect**		0.43	1.06	1.56	1.48	1.43	0.63	0.75	0.74	0.66	0.69	0.86	0.95	1.08	0.65	-
**Number**	112	110	98	75	64	53	38	23	17	14	11	6	4	2	1	0

Table B5124-51 Age Group = 71-80 Gender = MALE Occupational group = NON-MANUAL Total cases = 56

Threshold dB HL≥	0	5	10	15	20	25	30	35	40	45	50	55	65	75	85	95
**Prevalence**	**100.0**	**100.0**	**93.8**	**87.9**	**73.4**	**58.9**	**45.0**	**37.5**	**24.7**	**12.3**	**8.3**	**3.1**	**1.0**	**1.0**	**0.0**	**0.0**
**Lower CI**	93.6	93.6	85.9	76.9	64.1	48.4	33.8	26.4	16.9	8.5	4.9	1.2	0.2	0.2	0.0	0.0
**Upper CI**	100.0	100.0	97.4	94.1	80.9	68.7	56.7	50.2	34.6	17.5	14.0	8.1	5.3	5.3	6.4	6.4
**Design Effect**	-		0.86	1.00	0.74	0.81	0.90	0.97	0.79	0.52	0.62	0.70	0.74	0.74		
**Number**	56	56	53	51	44	36	30	24	17	9	5	3	1	1	0	0

Table B5124-52 Age Group = 18-30 Gender = FEMALE Occupational group = MANUAL Total cases = 109

Threshold dB HL≥	0	5	10	15	20	25	30	35	40	45	50	55	65	75	85	95
**Prevalence**	**88.6**	**52.9**	**22.3**	**6.9**	**3.8**	**3.8**	**1.1**	**0.6**	**0.6**	**0.5**	**0.0**	**0.0**	**0.0**	**0.0**	**0.0**	**0.0**
**Lower CI**	77.4	40.6	14.2	3.5	1.7	1.7	0.5	0.2	0.2	0.1	0.0	0.0	0.0	0.0	0.0	0.0
**Upper CI**	94.6	64.9	33.2	13.2	8.4	8.4	2.4	1.7	1.7	1.7	3.4	3.4	3.4	3.4	3.4	3.4
**Design Effect**	1.42	1.34	1.24	0.99	0.88	0.88	0.47	0.48	0.48	0.50				-		
**Number**	99	61	33	15	9	9	5	3	3	2	0	0	0	0	0	0

Table B5124-53 Age Group = 31-40 Gender = FEMALE Occupational group = MANUAL Total cases = 103

Threshold dB HL≥	0	5	10	15	20	25	30	35	40	45	50	55	65	75	85	95
**Prevalence**	**94.0**	**60.2**	**24.6**	**15.3**	**4.5**	**3.5**	**3.3**	**1.4**	**0.8**	**0.8**	**0.8**	**0.6**	**0.3**	**0.3**	**0.0**	**0.0**
**Lower CI**	85.3	47.4	16.7	9.6	2.0	1.3	1.2	0.5	0.3	0.3	0.3	0.2	0.1	0.1	0.0	0.0
**Upper CI**	97.7	71.8	34.7	23.5	9.8	9.1	8.9	3.8	2.3	2.3	2.3	2.3	1.7	1.7	3.6	3.6
**Design Effect**	1.27	1.33	1.10	1.00	0.92	1.00	1.02	0.65	0.52	0.52	0.52	0.58	0.56	0.56		
**Number**	99	72	39	24	10	7	6	4	3	3	3	2	1	1	0	0

Table B5124-54 Age Group = 41-50 Gender = FEMALE Occupational group = MANUAL Total cases = 135

Threshold dB HL≥	0	5	10	15	20	25	30	35	40	45	50	55	65	75	85	95
**Prevalence**	**96.5**	**80.8**	**53.8**	**24.3**	**13.7**	**9.1**	**7.8**	**5.5**	**3.8**	**2.6**	**2.0**	**1.6**	**0.4**	**0.2**	**0.2**	**0.2**
**Lower CI**	91.2	71.6	44.2	17.1	9.1	5.7	4.7	3.1	2.2	1.2	0.8	0.6	0.1	0.0	0.0	0.0
**Upper CI**	98.7	87.5	63.2	33.3	20.2	14.1	12.8	9.5	6.6	5.2	4.5	4.2	1.3	1.0	1.0	1.0
**Design Effect**	1.11	1.20	1.15	1.13	0.95	0.86	0.88	0.81	0.65	0.71	0.72	0.78	0.49	0.48	0.48	0.48
**Number**	131	112	77	43	30	23	19	15	12	9	7	5	2	1	1	1

Table B5124-55 Age Group = 51-60 Gender = FEMALE Occupational group = MANUAL Total cases = 166

Threshold dB HL≥	0	5	10	15	20	25	30	35	40	45	50	55	65	75	85	95
**Prevalence**	**99.3**	**92.5**	**67.9**	**38.9**	**25.0**	**18.9**	**11.0**	**6.5**	**2.8**	**1.8**	**1.5**	**1.0**	**0.7**	**0.2**	**0.2**	**0.2**
**Lower CI**	96.2	86.2	58.6	31.1	18.6	13.3	7.2	4.3	1.7	0.9	0.7	0.5	0.3	0.0	0.0	0.0
**Upper CI**	99.9	96.0	76.0	47.3	32.8	26.2	16.5	9.8	4.4	3.5	3.2	1.8	1.5	1.0	1.0	1.0
**Design Effect**	1.07	1.19	1.25	1.11	1.09	1.09	0.97	0.73	0.52	0.60	0.65	0.40	0.44	0.53	0.53	0.53
**Number**	165	156	127	88	62	47	30	23	16	10	8	7	5	1	1	1

Table B5124-56 Age Group = 61-70 Gender = FEMALE Occupational group = MANUAL Total cases = 126

Threshold dB HL≥	0	5	10	15	20	25	30	35	40	45	50	55	65	75	85	95
**Prevalence**	**100.0**	**97.6**	**82.0**	**61.1**	**42.4**	**26.5**	**17.1**	**13.6**	**8.2**	**6.2**	**5.2**	**4.2**	**2.8**	**2.1**	**1.9**	**1.6**
**Lower CI**	97.0	90.1	72.7	50.4	33.1	20.6	14.3	9.5	6.2	4.7	3.7	3.2	2.1	1.6	1.4	0.5
**Upper CI**	100.0	99.5	88.6	70.9	52.2	33.3	20.2	19.1	10.8	8.0	7.2	5.5	3.7	2.8	2.4	5.7
**Design Effect**	0.00	1.45	1.19	1.23	1.12	0.83	0.44	0.80	0.47	0.39	0.45	0.32	0.27	0.25	0.21	0.00
**Number**	126	124	108	87	68	50	37	34	23	18	14	12	6	3	2	1

Prevalence by age group, gender and occupational group for the better ear over 0.5, 1, 2, and 4 kHz

Table B5124-57 Age Group = 71-80 Gender = FEMALE Occupational group = MANUAL Total cases = 76

	Threshold dB HL≥															
	**0**	**5**	**10**	**15**	**20**	**25**	**30**	**35**	**40**	**45**	**50**	**55**	**65**	**75**	**85**	**95**
**Prevalence**	**100.0**	**100.0**	**95.4**	**87.2**	**70.8**	**53.6**	**47.8**	**36.4**	**30.2**	**23.0**	**13.2**	**9.5**	**4.3**	**3.5**	**2.1**	**0.0**
**Lower CI**	95.2	95.2	79.0	72.7	57.6	40.0	37.2	26.2	20.4	13.9	9.3	6.7	2.3	1.7	0.8	0.0
**Upper CI**	100.0	100.0	99.1	94.5	81.3	66.7	58.7	48.0	42.1	35.6	18.4	13.5	7.6	7.0	5.8	4.8
**Design Effect**	-	-	1.79	1.44	1.19	1.24	0.98	1.03	1.07	1.17	0.60	0.51	0.57	0.60	0.70	-
**Number**	76	76	74	71	64	57	53	43	36	31	23	18	9	7	4	0

Table B5124-58 Age Group = 18-30 Gender = MALE Occupational group = MANUAL Total cases = 109

	Threshold dB HL≥															
	**0**	**5**	**10**	**15**	**20**	**25**	**30**	**35**	**40**	**45**	**50**	**55**	**65**	**75**	**85**	**95**
**Prevalence**	**85.8**	**52.6**	**30.3**	**11.7**	**2.6**	**1.0**	**0.1**	**0.1**	**0.1**	**0.1**	**0.1**	**0.1**	**0.0**	**0.0**	**0.0**	**0.0**
**Lower CI**	74.0	39.8	20.1	6.5	0.8	0.2	0.0	0.0	0.0	0.0	0.0	0.0	0.0	0.0	0.0	0.0
**Upper CI**	92.7	65.0	42.9	20.2	8.7	5.1	0.4	0.4	0.4	0.4	0.4	0.4	3.4	3.4	3.4	3.4
**Design Effect**	1.43	1.39	1.36	1.13	1.14	0.99	0.27	0.27	0.27	0.27	0.27	0.27	-	-	-	-
**Number**	97	64	39	17	4	2	1	1	1	1	1	1	0	0	0	0

Table B5124-59 Age Group = 31-40 Gender = MALE Occupational group = MANUAL Total cases = 80

	Threshold dB HL≥															
	**0**	**5**	**10**	**15**	**20**	**25**	**30**	**35**	**40**	**45**	**50**	**55**	**65**	**75**	**85**	**95**
**Prevalence**	**90.2**	**66.9**	**36.7**	**21.8**	**11.6**	**3.6**	**3.6**	**2.7**	**2.3**	**2.3**	**1.9**	**1.9**	**1.9**	**1.9**	**1.9**	**1.9**
**Lower CI**	77.7	54.3	24.2	12.0	5.0	1.2	1.2	0.7	0.5	0.5	0.3	0.3	0.3	0.3	0.3	0.3
**Upper CI**	96.1	77.5	51.3	36.3	24.4	10.3	10.3	9.8	9.8	9.8	10.2	10.2	10.2	10.2	10.2	10.2
**Design Effect**	1.37	1.16	1.33	1.38	1.36	1.01	1.01	1.09	1.15	1.15	1.25	1.25	1.25	1.25	1.25	1.25
**Number**	74	57	38	21	12	6	6	4	3	3	1	1	1	1	1	1

Table B5124-60 Age Group = 41-50 Gender = MALE Occupational group = MANUAL Total cases = 127

	Threshold dB HL≥															
	**0**	**5**	**10**	**15**	**20**	**25**	**30**	**35**	**40**	**45**	**50**	**55**	**65**	**75**	**85**	**95**
**Prevalence**	**98.5**	**83.8**	**68.3**	**41.2**	**19.7**	**11.0**	**6.8**	**5.0**	**2.2**	**1.8**	**1.2**	**0.5**	**0.5**	**0.5**	**0.2**	**0.0**
**Lower CI**	92.1	73.2	57.4	31.3	12.9	7.0	4.2	2.8	1.2	0.9	0.6	0.1	0.1	0.1	0.0	0.0
**Upper CI**	99.7	90.7	77.4	51.9	28.8	16.9	10.8	8.8	4.1	3.4	2.3	1.7	1.7	1.7	1.3	2.9
**Design Effect**	1.37	1.37	1.26	1.23	1.17	0.90	0.74	0.78	0.55	0.51	0.43	0.54	0.54	0.54	0.54	-
**Number**	126	113	91	63	36	26	18	13	8	7	5	2	2	2	1	0

Table B5124-61 Age Group = 51-60 Gender = MALE Occupational group = MANUAL Total cases = 199

	Threshold dB HL≥															
	**0**	**5**	**10**	**15**	**20**	**25**	**30**	**35**	**40**	**45**	**50**	**55**	**65**	**75**	**85**	**95**
**Prevalence**	**100.0**	**97.3**	**83.0**	**59.9**	**45.7**	**31.8**	**17.9**	**13.2**	**11.1**	**8.0**	**5.3**	**4.2**	**1.3**	**0.3**	**0.0**	**0.0**
**Lower CI**	98.1	92.0	75.8	51.9	38.6	25.4	13.6	9.7	7.8	5.2	3.5	2.6	0.6	0.0	0.0	0.0
**Upper CI**	100.0	99.1	88.4	67.3	53.0	39.0	23.1	17.7	15.5	12.2	7.9	6.6	2.9	1.5	1.9	1.9
**Design Effect**	0.00	1.40	1.21	1.14	1.05	1.06	0.90	0.85	0.88	0.92	0.70	0.71	0.66	0.73	-	-
**Number**	199	196	177	143	119	85	57	44	38	28	21	16	7	1	0	0

Table B5124-62 Age Group = 61-70 Gender = MALE Occupational group = MANUAL Total cases = 173

	Threshold dB HL≥															
	**0**	**5**	**10**	**15**	**20**	**25**	**30**	**35**	**40**	**45**	**50**	**55**	**65**	**75**	**85**	**95**
**Prevalence**	**100.0**	**98.8**	**95.5**	**78.8**	**65.2**	**49.0**	**37.0**	**22.2**	**16.3**	**9.8**	**7.4**	**5.5**	**2.8**	**1.6**	**1.1**	**0.6**
**Lower CI**	97.8	93.3	88.9	68.4	54.7	39.1	28.3	17.2	12.0	7.0	5.3	3.6	1.6	0.8	0.5	0.1
**Upper CI**	100.0	99.8	98.2	86.4	74.4	59.0	46.6	28.1	21.8	13.6	10.2	8.1	4.8	3.2	2.6	2.2
**Design Effect**	-	1.47	1.41	1.50	1.41	1.36	1.29	0.88	0.89	0.75	0.62	0.66	0.63	0.60	0.63	0.74
**Number**	173	172	168	148	130	109	87	67	50	34	28	21	11	7	5	2

Table B5124-63 Age Group = 71-80 Gender = MALE Occupational group = MANUAL Total cases = 72

	Threshold dB HL≥															
	**0**	**5**	**10**	**15**	**20**	**25**	**30**	**35**	**40**	**45**	**50**	**55**	**65**	**75**	**85**	**95**
**Prevalence**	**100.0**	**100.0**	**100.0**	**97.5**	**86.0**	**69.1**	**48.4**	**42.1**	**24.1**	**18.6**	**16.3**	**13.3**	**4.7**	**2.8**	**0.6**	**0.6**
**Lower CI**	94.9	94.9	94.9	86.9	65.9	52.6	35.2	30.1	18.0	12.6	10.6	8.2	2.6	1.2	0.1	0.1
**Upper CI**	100.0	100.0	100.0	99.6	95.1	81.9	61.8	55.1	31.5	26.6	24.2	20.8	8.4	6.3	3.1	3.1
**Design Effect**	-	-	-	1.36	1.84	1.43	1.20	1.13	0.69	0.78	0.80	0.80	0.58	0.63	0.63	0.63
**Number**	72	72	72	71	67	62	49	44	33	27	23	19	8	5	1	1

Prevalence by age group, gender and occupational group for the better ear over 0.5, 1 and 2 kHz

Table B512-1 Age Group = 18-80 Gender = OVERALL Occupational group = OVERALL Total cases = 2664

Threshold dB HL≥	0	5	10	15	20	25	30	35	40	45	50	55	65	75	85	95
Prevalence	**90.1**	**65.2**	**41.0**	**26.0**	**16.6**	**11.3**	**7.9**	**5.7**	**4.2**	**2.9**	**2.1**	**1.6**	**0.9**	**0.6**	**0.3**	**0.2**
Lower CI	88.3	63.0	39.1	24.5	15.4	10.3	7.0	5.0	3.7	2.5	1.8	1.3	0.7	0.4	0.2	0.1
Upper CI	91.7	67.3	42.9	27.6	17.8	12.3	8.7	6.4	4.7	3.4	2.5	1.9	1.2	0.9	0.6	0.5
Design Effect	1.46	1.17	1.03	0.92	0.85	0.83	0.83	0.81	0.67	0.68	0.64	0.67	0.73	0.81	0.95	1.07
Number	2516	2015	1435	1022	738	551	415	327	260	197	152	115	63	37	20	11

Table B512-2 Age Group = 18-80 Gender = FEMALE Occupational group = OVERALL Total cases = 1387

Threshold dB HL≥	0	5	10	15	20	25	30	35	40	45	50	55	65	75	85	95
Prevalence	**91.9**	**66.7**	**40.4**	**25.9**	**16.6**	**11.4**	**8.5**	**6.4**	**4.7**	**3.0**	**2.2**	**1.5**	**1.0**	**0.6**	**0.4**	**0.2**
Lower CI	89.8	63.9	38.0	24.0	15.0	10.1	7.3	5.4	4.0	2.5	1.8	1.2	0.7	0.4	0.2	0.1
Upper CI	93.6	69.4	42.8	28.0	18.3	12.9	9.9	7.7	5.5	3.7	2.7	2.0	1.3	1.0	0.7	0.4
Design Effect	1.32	1.10	0.93	0.88	0.84	0.85	0.87	0.87	0.64	0.67	0.55	0.58	0.59	0.62	0.68	0.67
Number	1315	1042	713	507	364	274	219	179	146	103	85	61	36	23	13	7

Table B512-3 Age Group = 18-80 Gender = MALE Occupational group = OVERALL Total cases = 1277

Threshold dB HL≥	0	5	10	15	20	25	30	35	40	45	50	55	65	75	85	95
Prevalence	**88.2**	**63.5**	**41.7**	**26.1**	**16.6**	**11.1**	**7.0**	**4.8**	**3.6**	**2.8**	**2.0**	**1.6**	**0.8**	**0.5**	**0.3**	**0.3**
Lower CI	85.1	60.2	38.6	23.8	14.9	9.8	6.1	4.1	2.9	2.3	1.5	1.2	0.5	0.2	0.1	0.1
Upper CI	90.7	66.7	44.8	28.5	18.5	12.5	8.2	5.7	4.4	3.5	2.7	2.2	1.4	1.1	1.0	1.0
Design Effect	1.58	1.25	1.14	0.97	0.86	0.81	0.75	0.71	0.69	0.68	0.72	0.75	0.87	1.02	1.21	1.32
Number	1201	973	722	515	374	277	196	148	114	94	67	54	27	14	7	4

Table B512-4 Age Group = 18-30 Gender = OVERALL Occupational group = OVERALL Total cases = 401

Threshold dB HL≥	0	5	10	15	20	25	30	35	40	45	50	55	65	75	85	95
Prevalence	**79.6**	**41.0**	**16.8**	**5.7**	**2.2**	**1.6**	**0.8**	**0.4**	**0.4**	**0.1**	**0.0**	**0.0**	**0.0**	**0.0**	**0.0**	**0.0**
Lower CI	74.2	35.6	13.1	3.8	1.2	0.9	0.4	0.1	0.1	0.0	0.0	0.0	0.0	0.0	0.0	0.0
Upper CI	84.0	46.7	21.2	8.5	4.0	2.8	1.8	0.8	0.8	0.5	0.1	0.1	0.9	0.9	0.9	0.9
Design Effect	1.25	1.16	1.12	1.02	0.97	0.77	0.76	0.55	0.55	0.56	0.20	0.20	-	-	-	-
Number	334	186	89	37	17	15	9	6	6	3	2	2	0	0	0	0

Table B512-5 Age Group = 31-40 Gender = OVERALL Occupational group = OVERALL Total cases = 332

Threshold dB HL≥	0	5	10	15	20	25	30	35	40	45	50	55	65	75	85	95
Prevalence	**84.0**	**46.8**	**22.4**	**10.5**	**4.6**	**3.3**	**1.5**	**1.2**	**1.1**	**1.0**	**1.0**	**0.8**	**0.7**	**0.7**	**0.6**	**0.6**
Lower CI	78.4	40.6	17.6	7.2	2.8	1.8	0.7	0.5	0.4	0.3	0.3	0.2	0.1	0.1	0.1	0.1
Upper CI	88.3	53.1	28.0	15.1	7.4	5.8	3.4	3.2	3.1	3.0	3.0	3.0	3.0	3.0	3.1	3.1
Design Effect	1.26	1.18	1.17	1.18	1.02	1.02	0.97	1.02	1.06	1.13	1.13	1.20	1.27	1.27	1.36	1.36
Number	295	184	98	47	26	19	10	8	6	4	4	3	2	2	1	1

Table B512-6 Age Group = 41-50 Gender = OVERALL Occupational group = OVERALL Total cases = 450

Threshold dB HL≥	0	5	10	15	20	25	30	35	40	45	50	55	65	75	85	95
Prevalence	**93.9**	**65.9**	**35.6**	**19.1**	**9.6**	**6.4**	**4.8**	**3.4**	**2.5**	**1.4**	**0.9**	**0.6**	**0.3**	**0.3**	**0.1**	**0.1**
Lower CI	90.8	60.9	31.0	15.7	7.5	4.7	3.4	2.3	1.6	0.8	0.5	0.3	0.1	0.1	0.0	0.0
Upper CI	96.0	70.5	40.4	22.9	12.2	8.5	6.6	5.0	3.9	2.2	1.6	0.9	0.8	0.8	0.6	0.3
Design Effect	1.15	1.10	1.07	0.98	0.86	0.84	0.81	0.78	0.78	0.62	0.60	0.42	0.65	0.65	0.60	0.49
Number	428	316	187	117	72	50	39	30	23	15	10	7	3	3	2	1

Table B512-7 Age Group = 51-60 Gender = OVERALL Occupational group = OVERALL Total cases = 682

Threshold dB HL≥	0	5	10	15	20	25	30	35	40	45	50	55	65	75	85	95
Prevalence	**97.2**	**81.0**	**52.6**	**32.7**	**19.7**	**11.8**	**8.0**	**6.2**	**4.1**	**2.8**	**1.9**	**1.5**	**0.6**	**0.2**	**0.1**	**0.1**
Lower CI	95.3	77.5	48.6	29.2	16.9	9.7	6.3	4.8	3.1	2.1	1.3	1.0	0.3	0.1	0.0	0.0
Upper CI	98.3	84.1	56.7	36.4	22.8	14.3	10.0	7.9	5.4	3.9	2.8	2.3	1.2	0.4	0.3	0.3
Design Effect	1.17	1.13	1.09	1.04	0.99	0.95	0.92	0.87	0.80	0.74	0.72	0.72	0.70	0.49	0.47	0.47
Number	665	573	413	283	193	129	93	79	57	43	31	25	11	4	2	2

Prevalence by age group, gender and occupational group for the better ear over 0.5, 1 and 2 kHz

Table B512-8 Age Group = 61-70 Gender = OVERALL Occupational group = OVERALL Total cases = 527

	Threshold dB HL≥															
	0	5	10	15	20	25	30	35	40	45	50	55	65	75	85	95
**Prevalence**	**99.0**	**91.8**	**70.6**	**50.9**	**36.3**	**23.9**	**14.4**	**10.0**	**7.9**	**5.2**	**3.8**	**3.0**	**1.8**	**1.3**	**0.8**	**0.6**
**Lower CI**	97.2	88.3	65.5	45.8	31.9	20.6	11.9	8.2	6.5	4.2	2.9	2.3	1.2	0.8	0.4	0.3
**Upper CI**	99.7	94.3	75.2	55.9	40.9	27.5	17.4	12.1	9.7	6.3	4.8	4.1	2.8	2.2	1.7	1.4
**Design Effect**	1.30	1.26	1.26	1.18	1.10	0.96	0.92	0.75	0.69	0.57	0.58	0.61	0.66	0.71	0.74	0.80
**Number**	523	490	399	312	240	178	128	99	83	63	49	38	23	15	9	5

Table B512-9 Age Group = 71-80 Gender = OVERALL Occupational group = OVERALL Total cases = 272

	Threshold dB HL≥															
	0	5	10	15	20	25	30	35	40	45	50	55	65	75	85	95
**Prevalence**	**99.6**	**97.3**	**88.9**	**76.0**	**58.0**	**43.9**	**36.0**	**25.6**	**18.1**	**13.8**	**10.2**	**7.1**	**3.9**	**2.2**	**1.0**	**0.3**
**Lower CI**	98.0	93.8	83.2	69.2	50.9	37.3	29.7	20.2	14.8	10.6	8.1	5.3	2.7	1.3	0.4	0.1
**Upper CI**	99.9	98.9	92.9	81.7	64.8	50.7	42.8	31.9	21.9	17.7	12.8	9.5	5.6	3.6	2.3	1.0
**Design Effect**	0.98	1.24	1.30	1.24	1.19	1.15	1.17	1.13	0.78	0.87	0.66	0.69	0.61	0.65	0.72	0.61
**Number**	271	266	249	226	190	160	136	105	85	69	56	40	24	13	6	2

Table B512-10 Age Group = 18-80 Gender = OVERALL Occupational group = NON-MANUAL Total cases = 1184

	Threshold dB HL≥															
	0	5	10	15	20	25	30	35	40	45	50	55	65	75	85	95
**Prevalence**	**88.9**	**61.5**	**35.3**	**22.4**	**14.4**	**9.9**	**6.9**	**5.2**	**3.2**	**1.9**	**1.4**	**0.9**	**0.7**	**0.4**	**0.1**	**0.1**
**Lower CI**	86.0	58.4	32.7	20.2	13.0	8.8	6.0	4.5	2.7	1.5	1.0	0.7	0.4	0.2	0.0	0.0
**Upper CI**	91.2	64.4	37.9	24.7	16.0	11.2	7.9	6.0	3.8	2.4	1.7	1.3	1.0	0.7	0.3	0.2
**Design Effect**	1.46	1.09	0.95	0.95	0.76	0.73	0.65	0.60	0.55	0.55	0.53	0.56	0.62	0.68	0.61	0.63
**Number**	1101	836	549	377	273	195	141	111	85	64	50	36	23	12	5	3

Table B512-11 Age Group = 18-80 Gender = OVERALL Occupational group = MANUAL Total cases = 1475

	Threshold dB HL≥															
	0	5	10	15	20	25	30	35	40	45	50	55	65	75	85	95
**Prevalence**	**91.3**	**68.7**	**45.4**	**29.1**	**18.4**	**12.4**	**8.6**	**6.1**	**4.9**	**3.5**	**2.5**	**1.9**	**1.1**	**0.7**	**0.5**	**0.4**
**Lower CI**	88.9	65.5	42.5	26.8	16.7	11.0	7.6	5.2	4.1	2.8	2.0	1.5	0.8	0.5	0.3	0.2
**Upper CI**	93.3	71.8	48.4	31.5	20.3	13.8	9.7	7.0	5.7	4.2	3.0	2.3	1.5	1.2	1.0	0.8
**Design Effect**	1.54	1.35	1.16	1.01	0.91	0.83	0.73	0.74	0.73	0.76	0.61	0.63	0.69	0.76	0.85	0.92
**Number**	1412	1176	883	642	465	356	274	216	175	133	102	79	40	25	15	8

Table B512-12 Age Group = 18-30 Gender = FEMALE Occupational group = OVERALL Total cases = 224

	Threshold dB HL≥															
	0	5	10	15	20	25	30	35	40	45	50	55	65	75	85	95
**Prevalence**	**84.2**	**42.8**	**15.3**	**6.2**	**2.9**	**2.8**	**1.4**	**0.6**	**0.6**	**0.2**	**0.0**	**0.0**	**0.0**	**0.0**	**0.0**	**0.0**
**Lower CI**	77.8	35.7	11.2	3.9	1.6	1.5	0.6	0.2	0.2	0.0	0.0	0.0	0.0	0.0	0.0	0.0
**Upper CI**	89.0	50.1	20.6	9.8	5.2	5.1	3.3	1.5	1.5	1.1	1.7	1.7	1.7	1.7	1.7	1.7
**Design Effect**	1.17	1.12	0.99	0.90	0.80	0.81	0.79	0.59	0.59	0.66	-	-	-	-	-	-
**Number**	193	107	49	23	13	12	7	4	4	1	0	0	0	0	0	0

Table B512-13 Age Group = 31-40 Gender = FEMALE Occupational group = OVERALL Total cases = 190

	Threshold dB HL≥															
	0	5	10	15	20	25	30	35	40	45	50	55	65	75	85	95
**Prevalence**	**86.7**	**48.1**	**18.8**	**8.8**	**3.7**	**2.5**	**1.4**	**1.2**	**1.1**	**0.7**	**0.7**	**0.5**	**0.2**	**0.2**	**0.0**	**0.0**
**Lower CI**	80.0	40.1	13.8	5.5	2.1	1.2	0.7	0.5	0.5	0.3	0.3	0.1	0.0	0.0	0.0	0.0
**Upper CI**	91.4	56.2	25.1	13.8	6.5	5.0	3.0	2.7	2.7	2.2	2.2	1.7	1.2	1.2	2.0	2.0
**Design Effect**	1.18	1.15	1.02	1.02	0.79	0.81	0.64	0.65	0.68	0.70	0.70	0.66	0.63	0.63	-	-
**Number**	171	107	52	27	15	10	7	6	5	3	3	2	1	1	0	0

Table B512-14 Age Group = 41-50 Gender = FEMALE Occupational group = OVERALL Total cases = 247

	Threshold dB HL≥															
	0	5	10	15	20	25	30	35	40	45	50	55	65	75	85	95
**Prevalence**	**93.6**	**65.6**	**35.3**	**17.7**	**9.1**	**6.5**	**4.6**	**4.1**	**2.9**	**1.6**	**1.0**	**0.4**	**0.1**	**0.1**	**0.1**	**0.1**
**Lower CI**	89.1	58.9	29.5	13.5	6.6	4.3	2.9	2.5	1.6	0.8	0.4	0.1	0.0	0.0	0.0	0.0
**Upper CI**	96.4	71.8	41.7	22.7	12.4	9.8	7.1	6.6	5.0	3.2	2.4	1.0	0.6	0.6	0.6	0.6
**Design Effect**	1.16	1.10	1.03	0.96	0.82	0.88	0.80	0.80	0.80	0.73	0.72	0.49	0.51	0.51	0.51	0.51
**Number**	235	174	102	61	38	27	21	19	14	9	6	3	1	1	1	1

Prevalence by age group, gender and occupational group for the better ear over 0.5, 1 and 2 kHz

Table B512-15 | Age Group = 51-60 | Gender = FEMALE | Occupational group = OVERALL | Total cases = 341

Threshold dB HL≥	0	5	10	15	20	25	30	35	40	45	50	55	65	75	85	95
Prevalence	97.2	81.7	50.7	29.6	18.6	10.2	7.5	5.8	3.5	2.2	1.4	1.0	0.6	0.3	0.1	0.1
Lower CI	94.2	76.7	44.9	24.9	15.0	7.5	5.3	3.9	2.3	1.3	0.8	0.6	0.3	0.1	0.0	0.0
Upper CI	98.6	85.8	56.4	34.7	22.9	13.7	10.5	8.4	5.2	3.6	2.3	1.9	1.6	0.7	0.5	0.5
Design Effect	1.19	1.11	1.09	1.02	0.96	0.96	0.94	0.89	0.72	0.72	0.61	0.61	0.72	0.48	0.46	0.46
Number	334	288	201	135	94	58	45	38	27	18	14	11	6	4	2	2

Table B512-16 | Age Group = 61-70 | Gender = FEMALE | Occupational group = OVERALL | Total cases = 242

Threshold dB HL≥	0	5	10	15	20	25	30	35	40	45	50	55	65	75	85	95
Prevalence	98.7	92.3	68.4	50.1	32.9	21.9	14.2	8.6	7.3	4.4	3.7	3.0	2.0	1.4	1.1	0.9
Lower CI	95.2	87.4	61.4	43.3	27.2	17.8	10.5	6.4	5.3	3.4	2.7	2.0	1.1	0.7	0.5	0.3
Upper CI	99.7	95.4	74.6	56.9	39.2	26.7	18.8	11.4	9.8	5.8	5.1	4.5	3.4	2.9	2.6	2.4
Design Effect	1.31	1.17	1.14	1.09	1.03	0.86	0.95	0.71	0.69	0.44	0.51	0.55	0.65	0.71	0.74	0.79
Number	240	226	177	140	98	75	59	45	40	30	27	21	12	7	5	3

Table B512-17 | Age Group = 71-80 | Gender = FEMALE | Occupational group = OVERALL | Total cases = 143

Threshold dB HL≥	0	5	10	15	20	25	30	35	40	45	50	55	65	75	85	95
Prevalence	99.4	97.7	91.0	76.6	57.9	44.5	39.1	30.9	21.6	14.9	11.0	7.4	4.6	3.0	1.5	0.2
Lower CI	96.8	91.8	82.0	66.6	48.1	35.5	30.2	22.7	17.0	10.5	8.4	5.1	3.0	1.7	0.6	0.0
Upper CI	99.9	99.4	95.8	84.4	67.1	53.9	48.9	40.6	27.0	20.6	14.4	10.6	7.1	5.4	3.6	1.1
Design Effect	0.91	1.31	1.43	1.30	1.20	1.15	1.20	1.20	0.74	0.87	0.59	0.64	0.58	0.63	0.70	0.53
Number	142	140	132	121	106	92	80	67	56	42	35	24	16	10	5	1

Table B512-18 | Age Group = 18-30 | Gender = MALE | Occupational group = OVERALL | Total cases = 177

Threshold dB HL≥	0	5	10	15	20	25	30	35	40	45	50	55	65	75	85	95
Prevalence	74.8	39.2	18.2	5.0	1.3	0.2	0.1	0.1	0.1	0.1	0.1	0.1	0.0	0.0	0.0	0.0
Lower CI	65.8	31.1	12.4	2.4	0.3	0.1	0.0	0.0	0.0	0.0	0.0	0.0	0.0	0.0	0.0	0.0
Upper CI	82.1	47.9	25.8	9.9	5.4	0.8	0.2	0.2	0.2	0.2	0.2	0.2	2.1	2.1	2.1	2.1
Design Effect	1.28	1.18	1.19	1.12	1.24	0.42	0.19	0.19	0.19	0.19	0.19	0.19	-	-	-	-
Number	141	79	40	14	4	3	2	2	2	2	2	2	0	0	0	0

Table B512-19 | Age Group = 31-40 | Gender = MALE | Occupational group = OVERALL | Total cases = 142

Threshold dB HL≥	0	5	10	15	20	25	30	35	40	45	50	55	65	75	85	95
Prevalence	81.1	45.3	26.3	12.7	5.6	4.2	1.6	1.3	1.2	1.2	1.2	1.2	1.2	1.2	1.2	1.2
Lower CI	71.5	35.8	18.3	7.3	2.6	1.8	0.4	0.3	0.2	0.2	0.2	0.2	0.2	0.2	0.2	0.2
Upper CI	88.0	55.2	36.2	21.2	11.6	9.6	6.3	6.3	6.5	6.5	6.5	6.5	6.5	6.5	6.5	6.5
Design Effect	1.29	1.21	1.25	1.27	1.13	1.11	1.17	1.26	1.31	1.31	1.31	1.31	1.31	1.31	1.31	1.31
Number	124	77	46	20	11	9	3	2	1	1	1	1	1	1	1	1

Table B512-20 | Age Group = 41-50 | Gender = MALE | Occupational group = OVERALL | Total cases = 203

Threshold dB HL≥	0	5	10	15	20	25	30	35	40	45	50	55	65	75	85	95
Prevalence	94.2	66.1	35.8	20.6	10.2	6.2	4.9	2.7	2.2	1.1	0.8	0.8	0.4	0.4	0.2	0.0
Lower CI	89.4	58.6	28.9	15.6	7.1	4.0	3.0	1.4	1.1	0.6	0.4	0.4	0.1	0.1	0.0	0.0
Upper CI	96.9	72.9	43.3	26.6	14.5	9.4	8.0	4.9	4.3	1.8	1.4	1.4	1.6	1.6	1.1	1.9
Design Effect	1.13	1.10	1.10	1.00	0.89	0.79	0.81	0.75	0.75	0.42	0.36	0.36	0.67	0.67	0.63	-
Number	193	142	85	56	34	23	18	11	9	6	4	4	2	2	1	0

Table B512-21 | Age Group = 51-60 | Gender = MALE | Occupational group = OVERALL | Total cases = 341

Threshold dB HL≥	0	5	10	15	20	25	30	35	40	45	50	55	65	75	85	95
Prevalence	97.1	80.3	54.8	36.2	21.0	13.7	8.6	6.7	4.8	3.6	2.5	2.0	0.6	0.0	0.0	0.0
Lower CI	94.3	75.0	49.0	31.0	16.9	10.6	6.3	4.8	3.2	2.4	1.5	1.1	0.2	0.0	0.0	0.0
Upper CI	98.6	84.7	60.4	41.7	25.7	17.5	11.6	9.3	7.2	5.5	4.2	3.6	1.5	1.1	1.1	1.1
Design Effect	1.16	1.15	1.08	1.06	1.01	0.95	0.90	0.86	0.87	0.77	0.79	0.80	0.69	-	-	-
Number	331	285	212	148	99	71	48	41	30	25	17	14	5	0	0	0

Prevalence by age group, gender and occupational group for the better ear over 0.5, 1 and 2 kHz

Table B512-22 Age Group = 61-70 Gender = MALE Occupational group = OVERALL Total cases = 285

Threshold dB HL≥	0	5	10	15	20	25	30	35	40	45	50	55	65	75	85	95
**Prevalence**	**99.4**	**91.1**	**73.1**	**51.9**	**40.3**	**26.1**	**14.7**	**11.7**	**8.8**	**6.1**	**3.9**	**3.1**	**1.7**	**1.3**	**0.6**	**0.3**
**Lower CI**	97.5	85.4	65.4	44.4	33.7	21.0	11.7	9.0	6.7	4.4	2.6	2.0	1.0	0.6	0.2	0.1
**Upper CI**	99.8	94.6	79.6	59.3	47.1	32.0	18.4	15.1	11.4	8.3	5.7	4.8	3.0	2.6	1.5	1.2
**Design Effect**	1.04	1.37	1.39	1.29	1.19	1.08	0.81	0.81	0.71	0.69	0.67	0.70	0.66	0.70	0.64	0.69
**Number**	283	264	222	172	142	103	69	54	43	33	22	17	11	8	4	2

Table B512-23 Age Group = 71-80 Gender = MALE Occupational group = OVERALL Total cases = 129

Threshold dB HL≥	0	5	10	15	20	25	30	35	40	45	50	55	65	75	85	95
**Prevalence**	**100.0**	**96.7**	**84.9**	**74.2**	**57.4**	**42.1**	**30.8**	**17.8**	**13.2**	**12.3**	**9.1**	**6.8**	**2.9**	**1.0**	**0.4**	**0.4**
**Lower CI**	97.1	90.7	76.6	65.7	47.7	33.5	23.5	13.2	9.3	8.6	5.9	4.2	1.5	0.3	0.1	0.1
**Upper CI**	100.0	98.9	90.6	81.2	66.6	51.3	39.2	23.6	18.3	17.3	13.7	10.9	5.5	2.8	2.0	2.0
**Design Effect**	-	1.19	1.14	1.04	1.13	1.06	1.00	0.79	0.77	0.77	0.77	0.76	0.65	0.63	0.67	0.67
**Number**	129	126	117	105	84	68	56	38	29	27	21	16	8	3	1	1

Table B512-24 Age Group = 18-30 Gender = OVERALL Occupational group = NON-MANUAL Total cases = 181

Threshold dB HL≥	0	5	10	15	20	25	30	35	40	45	50	55	65	75	85	95
**Prevalence**	**74.8**	**33.7**	**10.4**	**3.7**	**1.3**	**1.0**	**0.7**	**0.5**	**0.5**	**0.1**	**0.1**	**0.1**	**0.0**	**0.0**	**0.0**	**0.0**
**Lower CI**	66.2	26.4	6.5	1.4	0.6	0.3	0.2	0.1	0.1	0.0	0.0	0.0	0.0	0.0	0.0	0.0
**Upper CI**	81.9	41.9	16.4	9.3	3.1	3.0	2.4	2.4	2.4	2.2	2.2	2.2	2.1	2.1	2.1	2.1
**Design Effect**	1.25	1.14	1.10	1.32	0.69	0.79	0.79	0.85	0.85	0.00	0.00	0.00	-	-	-	-
**Number**	144	68	26	9	5	4	3	2	2	1	1	1	0	0	0	0

Table B512-25 Age Group = 31-40 Gender = OVERALL Occupational group = NON-MANUAL Total cases = 148

Threshold dB HL≥	0	5	10	15	20	25	30	35	40	45	50	55	65	75	85	95
**Prevalence**	**86.8**	**44.3**	**18.2**	**8.3**	**3.7**	**1.8**	**0.4**	**0.4**	**0.3**	**0.3**	**0.3**	**0.0**	**0.0**	**0.0**	**0.0**	**0.0**
**Lower CI**	78.2	36.1	12.4	4.5	1.6	0.8	0.1	0.1	0.1	0.1	0.1	0.0	0.0	0.0	0.0	0.0
**Upper CI**	92.3	52.9	25.8	14.9	8.7	4.3	1.6	1.6	1.7	1.7	1.7	2.5	2.5	2.5	2.5	2.5
**Design Effect**	1.29	1.06	1.08	1.15	1.09	0.75	0.59	0.59	0.67	0.67	0.67	-	-	-	-	-
**Number**	132	75	33	13	8	6	2	2	1	1	1	0	0	0	0	0

Table B512-26 Age Group = 41-50 Gender = OVERALL Occupational group = NON-MANUAL Total cases = 188

Threshold dB HL≥	0	5	10	15	20	25	30	35	40	45	50	55	65	75	85	95
**Prevalence**	**92.3**	**62.0**	**24.8**	**13.9**	**6.1**	**4.2**	**3.4**	**0.8**	**0.8**	**0.1**	**0.1**	**0.1**	**0.0**	**0.0**	**0.0**	**0.0**
**Lower CI**	86.4	54.3	18.9	9.6	3.9	2.3	1.6	0.4	0.4	0.0	0.0	0.0	0.0	0.0	0.0	0.0
**Upper CI**	95.7	69.1	31.8	19.7	9.5	7.6	6.9	1.6	1.6	0.7	0.7	0.7	2.0	2.0	2.0	2.0
**Design Effect**	1.19	1.08	1.06	1.01	0.81	0.89	0.97	0.45	0.45	0.48	0.48	0.48	-	-	-	-
**Number**	175	118	55	33	17	10	8	4	4	1	1	1	0	0	0	0

Table B512-27 Age Group = 51-60 Gender = OVERALL Occupational group = NON-MANUAL Total cases = 317

Threshold dB HL≥	0	5	10	15	20	25	30	35	40	45	50	55	65	75	85	95
**Prevalence**	**96.6**	**77.7**	**46.7**	**27.8**	**13.8**	**6.1**	**3.9**	**3.7**	**2.6**	**2.0**	**1.2**	**0.8**	**0.5**	**0.3**	**0.1**	**0.1**
**Lower CI**	93.8	72.5	40.9	23.3	10.9	4.5	2.7	2.6	1.7	1.2	0.6	0.4	0.2	0.1	0.0	0.0
**Upper CI**	98.1	82.1	52.5	32.8	17.3	8.2	5.7	5.5	3.9	3.2	2.1	1.7	1.4	0.8	0.4	0.4
**Design Effect**	1.05	1.06	1.07	0.97	0.85	0.71	0.69	0.68	0.61	0.63	0.61	0.64	0.68	0.50	0.46	0.46
**Number**	304	248	162	108	71	41	27	26	20	15	10	7	4	3	1	1

Table B512-28 Age Group = 61-70 Gender = OVERALL Occupational group = NON-MANUAL Total cases = 227

Threshold dB HL≥	0	5	10	15	20	25	30	35	40	45	50	55	65	75	85	95
**Prevalence**	**98.6**	**92.4**	**70.6**	**47.2**	**36.1**	**24.8**	**13.5**	**9.8**	**8.1**	**4.7**	**3.2**	**2.5**	**2.1**	**1.4**	**0.5**	**0.4**
**Lower CI**	95.2	87.5	64.0	39.4	29.1	18.7	10.4	7.1	5.8	3.1	2.0	1.4	1.1	0.5	0.1	0.1
**Upper CI**	99.6	95.5	76.5	55.2	43.8	32.1	17.3	13.4	11.2	7.3	5.0	4.5	4.1	3.5	1.6	1.6
**Design Effect**	1.23	1.14	1.07	1.23	1.20	1.20	0.78	0.81	0.76	0.76	0.65	0.73	0.78	0.88	0.70	0.75
**Number**	224	209	164	118	92	66	47	35	30	24	19	14	11	6	3	2

Prevalence by age group, gender and occupational group for the better ear over 0.5, 1 and 2 kHz

Table B512-29 Age Group = 71-80 Gender = OVERALL Occupational group = NON-MANUAL Total cases = 123

Threshold dB HL≥	0	5	10	15	20	25	30	35	40	45	50	55	65	75	85	95
Prevalence	**99.1**	**95.7**	**85.1**	**71.3**	**56.7**	**47.1**	**38.8**	**30.4**	**13.8**	**8.3**	**6.6**	**4.5**	**3.0**	**1.4**	**0.4**	**0.0**
Lower CI	95.1	89.6	73.8	59.8	48.2	39.7	31.9	24.8	11.8	6.1	4.5	2.7	1.5	0.5	0.1	0.0
Upper CI	99.8	98.3	92.1	80.6	64.9	54.6	46.1	36.5	16.0	11.3	9.6	7.4	5.9	4.3	2.0	3.0
Design Effect	1.05	1.14	1.45	1.33	0.96	0.86	0.83	0.73	0.35	0.53	0.58	0.63	0.70	0.80	0.66	-
Number	122	118	109	96	80	68	54	42	28	22	18	13	8	3	1	0

Table B512-30 Age Group = 18-30 Gender = OVERALL Occupational group = MANUAL Total cases = 218

Threshold dB HL≥	0	5	10	15	20	25	30	35	40	45	50	55	65	75	85	95
Prevalence	**84.5**	**47.9**	**22.3**	**7.5**	**2.9**	**2.1**	**1.1**	**0.3**	**0.3**	**0.2**	**0.0**	**0.0**	**0.0**	**0.0**	**0.0**	**0.0**
Lower CI	77.4	39.4	16.2	4.6	1.3	1.0	0.3	0.1	0.1	0.0	0.0	0.0	0.0	0.0	0.0	0.0
Upper CI	89.7	56.6	29.7	11.9	6.0	4.6	3.2	0.9	0.9	0.7	0.2	0.2	1.7	1.7	1.7	1.7
Design Effect	1.29	1.31	1.23	1.02	1.01	0.89	0.92	0.47	0.47	0.51	0.26	0.26	-	-	-	-
Number	190	118	63	28	12	11	6	4	4	2	1	1	0	0	0	0

Table B512-31 Age Group = 31-40 Gender = OVERALL Occupational group = MANUAL Total cases = 183

Threshold dB HL≥	0	5	10	15	20	25	30	35	40	45	50	55	65	75	85	95
Prevalence	**81.4**	**49.5**	**24.7**	**12.0**	**5.8**	**4.8**	**2.2**	**1.7**	**1.7**	**1.3**	**1.3**	**1.3**	**1.1**	**1.1**	**0.9**	**0.9**
Lower CI	73.1	40.3	17.8	7.2	3.0	2.2	0.9	0.6	0.5	0.3	0.3	0.3	0.2	0.2	0.2	0.2
Upper CI	87.5	58.7	33.1	19.4	11.1	10.3	5.4	5.0	5.0	4.8	4.8	4.8	4.9	4.9	5.0	5.0
Design Effect	1.29	1.29	1.24	1.28	1.16	1.23	0.97	1.03	1.06	1.15	1.15	1.15	1.22	1.22	1.30	1.30
Number	162	108	64	33	18	13	8	6	5	3	3	3	2	2	1	1

Table B512-32 Age Group = 41-50 Gender = OVERALL Occupational group = MANUAL Total cases = 262

Threshold dB HL≥	0	5	10	15	20	25	30	35	40	45	50	55	65	75	85	95
Prevalence	**94.9**	**70.4**	**46.0**	**24.3**	**12.2**	**8.1**	**5.8**	**4.7**	**3.5**	**1.9**	**1.2**	**0.6**	**0.3**	**0.3**	**0.2**	**0.1**
Lower CI	90.3	63.2	38.9	19.0	9.0	5.7	3.9	3.1	2.1	1.1	0.6	0.4	0.1	0.1	0.1	0.0
Upper CI	97.3	76.7	53.2	30.6	16.4	11.4	8.4	7.2	5.8	3.3	2.4	1.1	0.9	0.9	0.7	0.5
Design Effect	1.28	1.23	1.19	1.13	0.93	0.86	0.78	0.78	0.80	0.64	0.65	0.36	0.52	0.52	0.52	0.48
Number	253	198	132	84	55	40	31	26	19	14	9	6	3	3	2	1

Table B512-33 Age Group = 51-60 Gender = OVERALL Occupational group = MANUAL Total cases = 365

Threshold dB HL≥	0	5	10	15	20	25	30	35	40	45	50	55	65	75	85	95
Prevalence	**98.0**	**84.5**	**56.9**	**36.6**	**24.0**	**16.7**	**11.1**	**7.8**	**5.0**	**3.2**	**2.1**	**1.7**	**0.7**	**0.1**	**0.1**	**0.1**
Lower CI	94.7	79.2	50.9	31.4	19.5	13.0	8.2	5.7	3.3	2.2	1.4	1.0	0.3	0.0	0.0	0.0
Upper CI	99.2	88.7	62.7	42.1	29.2	21.2	14.7	10.7	7.3	4.6	3.2	2.7	1.4	0.5	0.5	0.5
Design Effect	1.42	1.28	1.17	1.09	1.11	1.08	1.01	0.90	0.87	0.68	0.57	0.62	0.65	0.56	0.56	0.56
Number	361	325	251	175	122	88	66	53	37	28	21	18	7	1	1	1

Table B512-34 Age Group = 61-70 Gender = OVERALL Occupational group = MANUAL Total cases = 299

Threshold dB HL≥	0	5	10	15	20	25	30	35	40	45	50	55	65	75	85	95
Prevalence	**99.4**	**90.6**	**70.0**	**54.1**	**37.0**	**23.2**	**15.2**	**10.6**	**8.6**	**5.7**	**4.3**	**3.7**	**2.1**	**1.7**	**1.4**	**1.1**
Lower CI	96.9	84.9	62.4	46.7	31.1	19.2	12.8	8.5	7.0	4.5	3.4	2.8	1.5	1.2	1.0	0.8
Upper CI	99.9	94.3	76.7	61.4	43.4	27.8	17.8	13.2	10.6	7.1	5.5	4.8	2.8	2.4	2.0	1.6
Design Effect	1.30	1.40	1.39	1.31	1.13	0.90	0.62	0.67	0.57	0.50	0.45	0.46	0.40	0.39	0.38	0.32
Number	298	280	234	193	148	112	81	64	53	39	30	24	12	9	6	3

Table B512-35 Age Group = 71-80 Gender = OVERALL Occupational group = MANUAL Total cases = 148

Threshold dB HL≥	0	5	10	15	20	25	30	35	40	45	50	55	65	75	85	95
Prevalence	**100.0**	**99.5**	**93.1**	**81.9**	**60.8**	**41.8**	**34.4**	**23.7**	**21.6**	**17.5**	**11.6**	**7.9**	**4.5**	**2.7**	**1.5**	**0.5**
Lower CI	97.5	-	83.9	71.1	50.6	34.9	27.7	17.2	15.2	11.5	8.6	5.5	2.9	1.5	0.6	0.1
Upper CI	100.0	-	97.2	89.3	70.0	49.1	41.8	31.7	29.6	25.8	15.3	11.2	6.8	4.8	3.7	1.7
Design Effect	-	0.00	1.56	1.48	1.26	0.90	0.93	1.07	1.10	1.18	0.65	0.65	0.58	0.61	0.72	0.58
Number	148	147	139	129	110	92	82	63	57	47	38	27	16	10	5	2

Prevalence by age group, gender and occupational group for the better ear over 0.5, 1 and 2 kHz

Table B512-36 Age Group = 18-80 Gender = FEMALE Occupational group = NON-MANUAL Total cases = 670

Threshold dB HL≥	0	5	10	15	20	25	30	35	40	45	50	55	65	75	85	95
**Prevalence**	**90.9**	**64.0**	**36.1**	**22.2**	**15.3**	**10.1**	**7.9**	**6.8**	**4.1**	**2.2**	**1.8**	**1.3**	**1.0**	**0.5**	**0.2**	**0.1**
**Lower CI**	87.9	60.2	32.8	19.6	13.4	8.7	6.7	5.7	3.3	1.7	1.4	0.9	0.6	0.3	0.1	0.0
**Upper CI**	93.2	67.7	39.5	25.1	17.3	11.6	9.2	8.0	5.0	2.8	2.5	1.9	1.5	1.1	0.6	0.5
**Design Effect**	1.21	1.03	0.92	0.88	0.71	0.64	0.60	0.59	0.55	0.49	0.52	0.57	0.62	0.68	0.64	0.66
**Number**	625	474	306	206	153	108	86	74	58	42	37	26	19	10	5	3

Table B512-37 Age Group = 18-80 Gender = MALE Occupational group = NON-MANUAL Total cases = 514

Threshold dB HL≥	0	5	10	15	20	25	30	35	40	45	50	55	65	75	85	95
**Prevalence**	**86.6**	**58.5**	**34.4**	**22.5**	**13.4**	**9.8**	**5.8**	**3.4**	**2.2**	**1.6**	**0.8**	**0.6**	**0.3**	**0.2**	**0.0**	**0.0**
**Lower CI**	81.3	53.7	30.5	19.1	11.3	7.9	4.5	2.6	1.6	1.0	0.5	0.3	0.1	0.0	0.0	0.0
**Upper CI**	90.6	63.2	38.4	26.4	16.0	12.0	7.3	4.5	3.1	2.4	1.4	1.1	0.9	0.8	0.7	0.7
**Design Effect**	1.59	1.12	0.97	1.00	0.80	0.81	0.70	0.61	0.55	0.62	0.57	0.59	0.70	0.75	-	-
**Number**	476	362	243	171	120	87	55	37	27	22	13	10	4	2	0	0

Table B512-38 Age Group = 18-80 Gender = FEMALE Occupational group = MANUAL Total cases = 715

Threshold dB HL≥	0	5	10	15	20	25	30	35	40	45	50	55	65	75	85	95
**Prevalence**	**93.0**	**69.2**	**43.7**	**28.8**	**17.7**	**13.3**	**9.4**	**6.5**	**5.4**	**3.5**	**2.4**	**1.6**	**1.0**	**0.8**	**0.6**	**0.3**
**Lower CI**	89.6	64.8	40.1	25.8	15.4	11.4	7.9	5.3	4.3	2.6	1.9	1.3	0.7	0.6	0.4	0.3
**Upper CI**	95.4	73.3	47.3	32.0	20.3	15.5	11.0	7.9	6.6	4.6	2.9	2.1	1.4	1.2	0.9	0.5
**Design Effect**	1.55	1.26	0.98	0.94	0.88	0.82	0.73	0.71	0.71	0.77	0.46	0.45	0.46	0.46	0.47	0.24
**Number**	688	566	405	299	211	166	133	105	88	61	48	35	17	13	8	4

Table B512-39 Age Group = 18-80 Gender = MALE Occupational group = MANUAL Total cases = 760

Threshold dB HL≥	0	5	10	15	20	25	30	35	40	45	50	55	65	75	85	95
**Prevalence**	**89.5**	**68.3**	**47.3**	**29.4**	**19.2**	**11.3**	**7.7**	**5.6**	**4.4**	**3.5**	**2.6**	**2.1**	**1.1**	**0.7**	**0.5**	**0.4**
**Lower CI**	85.7	63.3	42.5	26.0	16.7	9.6	6.5	4.5	3.4	2.6	1.9	1.4	0.6	0.3	0.2	0.1
**Upper CI**	92.4	72.8	52.1	33.1	22.0	13.3	9.1	7.0	5.6	4.5	3.6	3.0	2.0	1.6	1.6	1.6
**Design Effect**	1.54	1.45	1.36	1.09	0.94	0.83	0.70	0.74	0.74	0.72	0.75	0.77	0.88	1.06	1.19	1.29
**Number**	724	610	478	343	254	190	141	111	87	72	54	44	23	12	7	4

Table B512-40 Age Group = 18-30 Gender = FEMALE Occupational group = NON-MANUAL Total cases = 115

Threshold dB HL≥	0	5	10	15	20	25	30	35	40	45	50	55	65	75	85	95
**Prevalence**	**83.1**	**37.6**	**12.5**	**3.5**	**2.4**	**1.8**	**1.1**	**0.8**	**0.8**	**0.0**	**0.0**	**0.0**	**0.0**	**0.0**	**0.0**	**0.0**
**Lower CI**	74.4	28.7	7.8	1.6	1.0	0.6	0.3	0.1	0.1	0.0	0.0	0.0	0.0	0.0	0.0	0.0
**Upper CI**	89.3	47.3	19.4	7.4	5.7	5.5	4.5	4.5	4.5	3.2	3.2	3.2	3.2	3.2	3.2	3.2
**Design Effect**	1.09	1.07	0.95	0.81	0.78	0.90	0.89	0.98	0.98	-	-	-	-	-	-	-
**Number**	97	46	19	6	4	3	2	1	1	0	0	0	0	0	0	0

Table B512-41 Age Group = 31-40 Gender = FEMALE Occupational group = NON-MANUAL Total cases = 86

Threshold dB HL≥	0	5	10	15	20	25	30	35	40	45	50	55	65	75	85	95
**Prevalence**	**84.3**	**46.8**	**14.8**	**6.1**	**2.5**	**1.7**	**0.7**	**0.7**	**0.6**	**0.6**	**0.6**	**0.0**	**0.0**	**0.0**	**0.0**	**0.0**
**Lower CI**	74.4	35.9	8.6	2.6	1.3	0.6	0.2	0.2	0.1	0.1	0.1	0.0	0.0	0.0	0.0	0.0
**Upper CI**	90.8	58.0	24.3	13.9	4.7	4.2	3.0	3.0	3.2	3.2	3.2	4.3	4.3	4.3	4.3	4.3
**Design Effect**	1.07	1.07	1.05	1.06	0.50	0.60	0.62	0.62	0.71	0.71	0.71	-	-	-	-	-
**Number**	74	43	16	7	5	4	2	2	1	1	1	0	0	0	0	0

Table B512-42 Age Group = 41-50 Gender = FEMALE Occupational group = NON-MANUAL Total cases = 112

Threshold dB HL≥	0	5	10	15	20	25	30	35	40	45	50	55	65	75	85	95
**Prevalence**	**93.4**	**61.2**	**25.9**	**13.6**	**5.8**	**2.4**	**1.9**	**0.8**	**0.8**	**0.0**	**0.0**	**0.0**	**0.0**	**0.0**	**0.0**	**0.0**
**Lower CI**	86.5	51.7	19.3	9.4	3.9	1.6	1.5	0.4	0.4	0.0	0.0	0.0	0.0	0.0	0.0	0.0
**Upper CI**	96.9	69.9	33.8	19.2	8.6	3.7	2.5	1.5	1.5	3.3	3.3	3.3	3.3	3.3	3.3	3.3
**Design Effect**	1.09	1.03	0.90	0.77	0.54	0.37	0.20	0.30	0.30	-	-	-	-	-	-	-
**Number**	105	72	35	20	10	4	3	2	2	0	0	0	0	0	0	0

Prevalence by age group, gender and occupational group for the better ear over 0.5, 1 and 2 kHz

Table B512-43 Age Group = 51-60 Gender = FEMALE Occupational group = NON-MANUAL Total cases = 175

	Threshold dB HL≥															
	**0**	**5**	**10**	**15**	**20**	**25**	**30**	**35**	**40**	**45**	**50**	**55**	**65**	**75**	**85**	**95**
**Prevalence**	**97.1**	**81.4**	**48.4**	**25.2**	**14.3**	**5.5**	**4.8**	**4.8**	**3.5**	**2.3**	**1.6**	**1.0**	**1.0**	**0.5**	**0.1**	**0.1**
**Lower CI**	93.1	74.8	40.5	19.3	10.4	3.6	3.0	3.0	2.2	1.3	0.8	0.4	0.4	0.2	0.0	0.0
**Upper CI**	98.8	86.6	56.4	32.2	19.3	8.2	7.5	7.5	5.6	4.3	3.4	2.6	2.6	1.4	0.7	0.7
**Design Effect**	1.05	1.04	1.09	1.00	0.86	0.67	0.71	0.71	0.61	0.64	0.64	0.70	0.70	0.50	0.48	0.48
**Number**	170	142	95	59	39	21	17	17	14	9	7	4	4	3	1	1

Table B512-44 Age Group = 61-70 Gender = FEMALE Occupational group = NON-MANUAL Total cases = 115

	Threshold dB HL≥															
	**0**	**5**	**10**	**15**	**20**	**25**	**30**	**35**	**40**	**45**	**50**	**55**	**65**	**75**	**85**	**95**
**Prevalence**	**97.6**	**92.5**	**67.0**	**46.5**	**35.3**	**20.9**	**11.8**	**8.6**	**6.8**	**4.1**	**3.6**	**2.7**	**2.2**	**1.2**	**0.8**	**0.7**
**Lower CI**	91.4	84.9	57.7	37.4	26.9	15.2	7.7	5.2	3.9	2.8	2.3	1.5	1.1	0.4	0.2	0.2
**Upper CI**	99.4	96.4	75.2	55.9	44.6	28.0	17.7	13.9	11.7	6.0	5.5	4.9	4.3	3.5	3.0	3.1
**Design Effect**	1.22	1.15	1.03	1.03	1.03	0.87	0.84	0.83	0.83	0.44	0.47	0.55	0.59	0.69	0.70	0.75
**Number**	113	107	81	60	47	34	27	21	18	15	14	10	8	4	3	2

Table B512-45 Age Group = 71-80 Gender = FEMALE Occupational group = NON-MANUAL Total cases = 67

	Threshold dB HL≥															
	**0**	**5**	**10**	**15**	**20**	**25**	**30**	**35**	**40**	**45**	**50**	**55**	**65**	**75**	**85**	**95**
**Prevalence**	**98.6**	**95.5**	**86.0**	**73.0**	**59.4**	**50.0**	**44.6**	**39.8**	**18.9**	**10.0**	**9.0**	**6.9**	**4.4**	**2.5**	**0.6**	**0.0**
**Lower CI**	92.2	85.0	67.2	55.2	47.9	40.7	36.1	32.0	16.6	6.9	5.9	4.0	2.1	0.8	0.1	0.0
**Upper CI**	99.7	98.8	94.8	85.5	70.0	59.3	53.4	48.2	21.4	14.3	13.5	11.6	9.1	7.3	3.4	5.4
**Design Effect**	0.99	1.23	1.67	1.49	0.97	0.79	0.74	0.70	0.26	0.51	0.55	0.61	0.68	0.78	0.64	-
**Number**	66	64	60	54	48	42	35	31	22	17	15	12	7	3	1	0

Table B512-46 Age Group = 18-30 Gender = MALE Occupational group = NON-MANUAL Total cases = 66

	Threshold dB HL≥															
	**0**	**5**	**10**	**15**	**20**	**25**	**30**	**35**	**40**	**45**	**50**	**55**	**65**	**75**	**85**	**95**
**Prevalence**	**66.1**	**29.7**	**8.0**	**3.8**	**0.1**	**0.1**	**0.1**	**0.1**	**0.1**	**0.1**	**0.1**	**0.1**	**0.0**	**0.0**	**0.0**	**0.0**
**Lower CI**	51.1	19.0	3.0	0.8	0.0	0.0	0.0	0.0	0.0	0.0	0.0	0.0	0.0	0.0	0.0	0.0
**Upper CI**	78.4	43.3	19.6	17.1	5.7	5.7	5.7	5.7	5.7	5.7	5.7	5.7	5.5	5.5	5.5	5.5
**Design Effect**	1.24	1.14	1.21	1.46	0.00	0.00	0.00	0.00	0.00	0.00	0.00	0.00	-	-	-	-
**Number**	47	22	7	3	1	1	1	1	1	1	1	1	0	0	0	0

Table B512-47 Age Group = 31-40 Gender = MALE Occupational group = NON-MANUAL Total cases = 62

	Threshold dB HL≥															
	**0**	**5**	**10**	**15**	**20**	**25**	**30**	**35**	**40**	**45**	**50**	**55**	**65**	**75**	**85**	**95**
**Prevalence**	**89.5**	**41.3**	**22.0**	**10.9**	**5.1**	**1.9**	**0.0**	**0.0**	**0.0**	**0.0**	**0.0**	**0.0**	**0.0**	**0.0**	**0.0**	**0.0**
**Lower CI**	72.1	29.4	12.8	4.7	1.4	0.5	0.0	0.0	0.0	0.0	0.0	0.0	0.0	0.0	0.0	0.0
**Upper CI**	96.6	54.3	35.0	23.5	16.6	7.1	5.8	5.8	5.8	5.8	5.8	5.8	5.8	5.8	5.8	5.8
**Design Effect**	1.56	1.05	1.09	1.19	1.25	0.82	-	-	-	-	-	-	-	-	-	-
**Number**	58	32	17	6	3	2	0	0	0	0	0	0	0	0	0	0

Table B512-48 Age Group = 41-50 Gender = MALE Occupational group = NON-MANUAL Total cases = 76

	Threshold dB HL≥															
	**0**	**5**	**10**	**15**	**20**	**25**	**30**	**35**	**40**	**45**	**50**	**55**	**65**	**75**	**85**	**95**
**Prevalence**	**90.9**	**63.0**	**23.6**	**14.4**	**6.5**	**6.2**	**5.0**	**0.7**	**0.7**	**0.3**	**0.3**	**0.3**	**0.0**	**0.0**	**0.0**	**0.0**
**Lower CI**	79.8	50.6	14.4	7.5	2.9	2.7	1.8	0.2	0.2	0.0	0.0	0.0	0.0	0.0	0.0	0.0
**Upper CI**	96.2	73.9	36.3	25.9	13.9	13.7	13.1	2.8	2.8	1.4	1.4	1.4	4.8	4.8	4.8	4.8
**Design Effect**	1.23	1.10	1.17	1.17	0.95	0.96	1.08	0.56	0.56	0.44	0.44	0.44	-	-	-	-
**Number**	70	46	20	13	7	6	5	2	2	1	1	1	0	0	0	0

Table B512-49 Age Group = 51-60 Gender = MALE Occupational group = NON-MANUAL Total cases = 142

	Threshold dB HL≥															
	**0**	**5**	**10**	**15**	**20**	**25**	**30**	**35**	**40**	**45**	**50**	**55**	**65**	**75**	**85**	**95**
**Prevalence**	**96.0**	**73.4**	**44.7**	**30.8**	**13.3**	**6.7**	**2.9**	**2.6**	**1.6**	**1.6**	**0.6**	**0.6**	**0.0**	**0.0**	**0.0**	**0.0**
**Lower CI**	91.1	64.9	36.4	24.3	9.4	4.3	1.5	1.3	0.7	0.7	0.2	0.2	0.0	0.0	0.0	0.0
**Upper CI**	98.2	80.4	53.2	38.2	18.5	10.4	5.4	4.9	3.4	3.4	1.8	1.8	2.6	2.6	2.6	2.6
**Design Effect**	1.05	1.08	1.04	0.93	0.82	0.73	0.67	0.66	0.63	0.63	0.55	0.55	-	-	-	-
**Number**	134	106	67	49	32	20	10	9	6	6	3	3	0	0	0	0

Prevalence by age group, gender and occupational group for the better ear over 0.5, 1 and 2 kHz

Table B512-50 Age Group = 61-70 Gender = MALE Occupational group = NON-MANUAL Total cases = 112

Threshold dB HL≥	0	5	10	15	20	25	30	35	40	45	50	55	65	75	85	95
Prevalence	99.8	92.4	75.6	49.0	37.6	29.1	15.5	11.5	9.6	5.4	2.8	2.4	2.1	1.6	0.0	0.0
Lower CI	98.9	85.3	65.8	35.8	26.1	18.2	11.3	7.6	6.5	2.6	1.1	0.8	0.6	0.4	0.0	0.0
Upper CI	100.0	96.2	83.2	62.3	50.7	43.1	20.9	17.1	14.0	10.9	7.0	6.9	6.8	6.5	3.3	3.3
Design Effect	0.47	1.08	1.11	1.48	1.42	1.52	0.72	0.81	0.68	0.95	0.88	0.95	1.01	1.08	-	-
Number	111	102	83	58	45	32	20	14	12	9	5	4	3	2	0	0

Table B512-51 Age Group = 71-80 Gender = MALE Occupational group = NON-MANUAL Total cases = 56

Threshold dB HL≥	0	5	10	15	20	25	30	35	40	45	50	55	65	75	85	95
Prevalence	100.0	95.8	83.2	67.8	52.1	42.4	30.2	17.0	6.7	5.9	3.1	1.0	1.0	0.0	0.0	0.0
Lower CI	93.6	87.2	72.8	56.7	40.4	31.5	20.3	11.0	3.6	3.1	1.2	0.2	0.2	0.0	0.0	0.0
Upper CI	100.0	98.7	90.1	77.2	63.5	54.2	42.4	25.4	12.2	11.0	8.1	5.3	5.3	6.4	6.4	6.4
Design Effect	-	0.99	0.89	0.85	0.90	0.90	0.94	0.74	0.64	0.63	0.70	0.74	0.74	-	-	-
Number	56	54	49	42	32	26	19	11	6	5	3	1	1	0	0	0

Table B512-52 Age Group = 18-30 Gender = FEMALE Occupational group = MANUAL Total cases = 109

Threshold dB HL≥	0	5	10	15	20	25	30	35	40	45	50	55	65	75	85	95
Prevalence	85.2	47.0	18.6	9.3	3.8	3.8	1.9	0.6	0.6	0.3	0.0	0.0	0.0	0.0	0.0	0.0
Lower CI	73.8	35.5	12.1	5.2	1.7	1.7	0.6	0.2	0.2	0.0	0.0	0.0	0.0	0.0	0.0	0.0
Upper CI	92.2	58.8	27.6	16.2	8.4	8.4	5.9	1.7	1.7	1.4	3.4	3.4	3.4	3.4	3.4	3.4
Design Effect	1.38	1.28	1.07	1.00	0.88	0.88	0.90	0.48	0.48	0.53	-	-	-	-	-	-
Number	96	61	30	17	9	9	5	3	3	1	0	0	0	0	0	0

Table B512-53 Age Group = 31-40 Gender = FEMALE Occupational group = MANUAL Total cases = 103

Threshold dB HL≥	0	5	10	15	20	25	30	35	40	45	50	55	65	75	85	95
Prevalence	89.0	48.5	19.7	9.0	4.8	3.3	1.7	1.4	1.4	0.6	0.6	0.6	0.3	0.3	0.0	0.0
Lower CI	79.1	36.8	13.6	5.1	2.2	1.2	0.7	0.5	0.5	0.2	0.2	0.2	0.1	0.1	0.0	0.0
Upper CI	94.6	60.4	27.7	15.2	10.3	8.9	4.1	3.8	3.8	2.3	2.3	2.3	1.7	1.7	3.6	3.6
Design Effect	1.26	1.26	0.93	0.90	0.93	1.02	0.63	0.65	0.65	0.58	0.58	0.58	0.56	0.56	-	-
Number	96	63	35	19	10	6	5	4	4	2	2	2	1	1	0	0

Table B512-54 Age Group = 41-50 Gender = FEMALE Occupational group = MANUAL Total cases = 135

Threshold dB HL≥	0	5	10	15	20	25	30	35	40	45	50	55	65	75	85	95
Prevalence	94.2	72.1	46.1	21.9	11.3	9.9	6.7	6.3	4.6	2.6	1.7	0.5	0.2	0.2	0.2	0.2
Lower CI	87.0	62.4	37.0	15.2	7.4	6.1	4.1	3.8	2.4	1.2	0.7	0.2	0.0	0.0	0.0	0.0
Upper CI	97.5	80.1	55.6	30.5	17.0	15.7	10.9	10.5	8.5	5.2	4.3	1.3	1.0	1.0	1.0	1.0
Design Effect	1.26	1.18	1.12	1.10	0.90	0.94	0.80	0.81	0.84	0.71	0.74	0.40	0.48	0.48	0.48	0.48
Number	130	102	67	41	28	23	18	17	12	9	6	3	1	1	1	1

Table B512-55 Age Group = 51-60 Gender = FEMALE Occupational group = MANUAL Total cases = 166

Threshold dB HL≥	0	5	10	15	20	25	30	35	40	45	50	55	65	75	85	95
Prevalence	97.9	83.8	53.2	33.6	23.5	16.4	10.8	6.8	3.3	1.6	1.0	1.0	0.3	0.2	0.2	0.2
Lower CI	92.3	75.9	44.2	26.3	17.1	11.0	6.7	4.1	1.7	0.8	0.5	0.5	0.1	0.0	0.0	0.0
Upper CI	99.5	89.5	62.0	41.8	31.4	23.6	16.9	11.2	6.4	3.4	1.8	1.8	1.1	1.0	1.0	1.0
Design Effect	1.39	1.22	1.20	1.09	1.12	1.12	1.07	0.91	0.83	0.64	0.40	0.40	0.50	0.53	0.53	0.53
Number	164	146	106	76	55	37	28	21	13	9	7	7	2	1	1	1

Table B512-56 Age Group = 61-70 Gender = FEMALE Occupational group = MANUAL Total cases = 126

Threshold dB HL≥	0	5	10	15	20	25	30	35	40	45	50	55	65	75	85	95
Prevalence	100.0	91.8	68.0	52.5	30.1	21.6	14.5	9.0	8.3	5.2	4.4	4.0	2.4	2.1	1.9	1.6
Lower CI	97.0	83.2	57.3	42.5	22.9	16.2	12.6	7.1	6.5	4.0	3.5	3.1	1.8	1.6	1.4	0.5
Upper CI	100.0	96.2	77.1	62.3	38.3	28.2	16.7	11.3	10.6	6.9	5.6	5.2	3.2	2.8	2.4	5.7
Design Effect	0.00	1.32	1.24	1.16	0.97	0.84	0.33	0.43	0.43	0.37	0.29	0.31	0.26	0.25	0.21	0.00
Number	126	118	95	79	51	41	32	24	22	15	13	11	4	3	2	1

Prevalence by age group, gender and occupational group for the better ear over 0.5, 1 and 2 kHz

Table B512-57 Age Group = 71-80 Gender = FEMALE Occupational group = MANUAL Total cases = 76

	Threshold dB HL≥															
	0	5	10	15	20	25	30	35	40	45	50	55	65	75	85	95
**Prevalence**	**100.0**	**100.0**	**95.3**	**82.0**	**58.7**	**46.6**	**38.1**	**26.6**	**25.1**	**18.6**	**10.8**	**6.3**	**4.8**	**3.5**	**2.1**	**0.4**
**Lower CI**	95.2	95.2	83.1	67.7	44.9	36.0	27.9	17.0	15.8	10.0	7.7	3.8	2.7	1.7	0.8	0.1
**Upper CI**	100.0	100.0	98.8	90.8	71.2	57.6	49.5	39.0	37.6	31.9	15.1	10.3	8.4	7.0	5.8	2.1
**Design Effect**	-	-	1.45	1.36	1.23	0.98	1.02	1.13	1.14	1.27	0.53	0.58	0.57	0.60	0.70	0.53
**Number**	76	76	72	67	58	50	45	36	34	25	20	12	9	7	4	1

Table B512-58 Age Group = 18-30 Gender = MALE Occupational group = MANUAL Total cases = 109

	Threshold dB HL≥															
	0	5	10	15	20	25	30	35	40	45	50	55	65	75	85	95
**Prevalence**	**84.2**	**48.9**	**26.1**	**5.4**	**1.7**	**0.2**	**0.1**	**0.1**	**0.1**	**0.1**	**0.1**	**0.1**	**0.0**	**0.0**	**0.0**	**0.0**
**Lower CI**	74.6	36.7	16.4	2.4	0.4	0.1	0.0	0.0	0.0	0.0	0.0	0.0	0.0	0.0	0.0	0.0
**Upper CI**	90.6	61.3	38.7	11.8	7.7	0.9	0.4	0.4	0.4	0.4	0.4	0.4	3.4	3.4	3.4	3.4
**Design Effect**	1.17	1.35	1.39	1.06	1.20	0.39	0.27	0.27	0.27	0.27	0.27	0.27	-	-	-	-
**Number**	94	57	33	11	3	2	1	1	1	1	1	1	0	0	0	0

Table B512-59 Age Group = 31-40 Gender = MALE Occupational group = MANUAL Total cases = 80

	Threshold dB HL≥															
	0	5	10	15	20	25	30	35	40	45	50	55	65	75	85	95
**Prevalence**	**73.3**	**50.6**	**29.9**	**15.5**	**7.1**	**6.7**	**2.8**	**2.1**	**1.9**	**1.9**	**1.9**	**1.9**	**1.9**	**1.9**	**1.9**	**1.9**
**Lower CI**	59.5	36.7	18.0	7.3	2.6	2.3	0.7	0.4	0.3	0.3	0.3	0.3	0.3	0.3	0.3	0.3
**Upper CI**	83.7	64.3	45.3	30.0	18.1	17.9	10.2	9.9	10.2	10.2	10.2	10.2	10.2	10.2	10.2	10.2
**Design Effect**	1.28	1.31	1.41	1.44	1.29	1.33	1.11	1.20	1.25	1.25	1.25	1.25	1.25	1.25	1.25	1.25
**Number**	66	45	29	14	8	7	3	2	1	1	1	1	1	1	1	1

Table B512-60 Age Group = 41-50 Gender = MALE Occupational group = MANUAL Total cases = 127

	Threshold dB HL≥															
	0	5	10	15	20	25	30	35	40	45	50	55	65	75	85	95
**Prevalence**	**95.6**	**68.5**	**45.6**	**26.9**	**13.1**	**6.1**	**4.7**	**2.9**	**2.3**	**1.2**	**0.7**	**0.7**	**0.5**	**0.5**	**0.2**	**0.0**
**Lower CI**	88.4	57.4	35.1	19.0	8.5	3.7	2.6	1.5	1.1	0.6	0.4	0.4	0.1	0.1	0.0	0.0
**Upper CI**	98.4	77.8	56.5	36.5	19.8	9.9	8.3	5.7	5.0	2.3	1.3	1.3	1.7	1.7	1.3	2.9
**Design Effect**	1.29	1.29	1.26	1.15	0.96	0.73	0.75	0.69	0.71	0.43	0.31	0.31	0.54	0.54	0.54	-
**Number**	123	96	65	43	27	17	13	9	7	5	3	3	2	2	1	0

Table B512-61 Age Group = 51-60 Gender = MALE Occupational group = MANUAL Total cases = 199

	Threshold dB HL≥															
	0	5	10	15	20	25	30	35	40	45	50	55	65	75	85	95
**Prevalence**	**98.0**	**85.4**	**61.0**	**39.7**	**24.8**	**17.1**	**11.5**	**9.0**	**6.9**	**4.8**	**3.5**	**2.5**	**1.1**	**0.0**	**0.0**	**0.0**
**Lower CI**	92.9	77.7	53.3	32.8	18.9	12.5	8.1	6.0	4.3	3.1	2.1	1.3	0.4	0.0	0.0	0.0
**Upper CI**	99.5	90.8	68.2	47.1	31.8	22.9	16.2	13.3	10.9	7.5	5.5	4.7	2.7	1.9	1.9	1.9
**Design Effect**	1.44	1.33	1.11	1.07	1.08	0.99	0.92	0.91	0.93	0.72	0.65	0.74	0.73	-	-	-
**Number**	197	179	145	99	67	51	38	32	24	19	14	11	5	0	0	0

Table B512-62 Age Group = 61-70 Gender = MALE Occupational group = MANUAL Total cases = 173

	Threshold dB HL≥															
	0	5	10	15	20	25	30	35	40	45	50	55	65	75	85	95
**Prevalence**	**98.8**	**89.0**	**72.1**	**55.8**	**45.1**	**24.9**	**15.8**	**12.7**	**9.2**	**6.3**	**4.2**	**3.4**	**1.8**	**1.2**	**0.9**	**0.6**
**Lower CI**	93.3	80.2	60.9	44.9	35.7	19.4	11.4	8.8	6.4	4.4	2.7	2.0	0.9	0.6	0.3	0.1
**Upper CI**	99.8	94.1	81.2	66.1	55.0	31.3	21.4	17.8	13.0	9.1	6.6	5.7	3.3	2.7	2.4	2.2
**Design Effect**	1.47	1.47	1.55	1.47	1.33	0.93	0.91	0.91	0.76	0.64	0.64	0.66	0.59	0.61	0.66	0.74
**Number**	172	162	139	114	97	71	49	40	31	24	17	13	8	6	4	2

Table B512-63 Age Group = 71-80 Gender = MALE Occupational group = MANUAL Total cases = 72

	Threshold dB HL≥															
	0	5	10	15	20	25	30	35	40	45	50	55	65	75	85	95
**Prevalence**	**100.0**	**98.7**	**89.6**	**81.5**	**63.3**	**34.5**	**28.9**	**19.3**	**16.3**	**15.9**	**12.7**	**10.2**	**4.0**	**1.5**	**0.6**	**0.6**
**Lower CI**	94.9	-	71.1	62.5	48.7	28.3	23.3	13.3	10.6	10.2	7.7	6.1	2.1	0.5	0.1	0.1
**Upper CI**	100.0	-	96.8	92.1	75.8	41.2	35.2	27.0	24.2	23.8	20.2	16.8	7.4	4.2	3.1	3.1
**Design Effect**	-	0.00	1.77	1.70	1.26	0.59	0.57	0.76	0.80	0.81	0.81	0.76	0.56	0.57	0.63	0.63
**Number**	72	71	67	62	52	42	37	27	23	22	18	15	7	3	1	1

Prevalence by age group, gender and occupational group for the better ear over 0.25, 0.5, and 1 kHz

Table B2551-1 Age Group = 18-80 Gender = OVERALL Occupational group = OVERALL Total cases = 2658

	Threshold dB HL≥															
	0	5	10	15	20	25	30	35	40	45	50	55	65	75	85	95
**Prevalence**	**94.2**	**72.1**	**43.8**	**24.8**	**15.3**	**9.9**	**6.9**	**5.1**	**3.7**	**2.7**	**1.7**	**1.4**	**0.9**	**0.5**	**0.4**	**0.2**
**Lower CI**	92.7	69.9	41.8	23.2	14.1	9.0	6.1	4.5	3.2	2.3	1.4	1.1	0.6	0.3	0.2	0.1
**Upper CI**	95.4	74.2	45.9	26.5	16.6	11.0	7.8	5.7	4.3	3.2	2.1	1.7	1.2	0.8	0.6	0.5
**Design Effect**	1.51	1.27	1.09	0.98	0.91	0.88	0.87	0.72	0.72	0.72	0.66	0.69	0.73	0.84	0.95	1.10
**Number**	2567	2131	1481	973	678	483	370	298	232	177	125	100	61	33	20	9

Table B2551-2 Age Group = 18-80 Gender = FEMALE Occupational group = OVERALL Total cases = 1383

	Threshold dB HL≥															
	0	5	10	15	20	25	30	35	40	45	50	55	65	75	85	95
**Prevalence**	**95.4**	**72.7**	**44.2**	**25.4**	**16.9**	**11.3**	**8.3**	**5.9**	**4.3**	**3.1**	**1.8**	**1.4**	**1.0**	**0.5**	**0.3**	**0.2**
**Lower CI**	93.7	69.8	41.7	23.3	15.2	9.9	7.1	5.1	3.6	2.5	1.5	1.1	0.7	0.3	0.2	0.1
**Upper CI**	96.6	75.3	46.8	27.6	18.9	12.9	9.6	6.9	5.1	3.8	2.3	1.9	1.3	0.8	0.6	0.4
**Design Effect**	1.32	1.17	0.99	0.93	0.95	0.91	0.88	0.71	0.72	0.71	0.58	0.59	0.58	0.63	0.68	0.69
**Number**	1338	1103	760	500	366	268	220	175	135	104	72	56	37	19	12	6

Table B2551-3 Age Group = 18-80 Gender = MALE Occupational group = OVERALL Total cases = 1275

	Threshold dB HL≥															
	0	5	10	15	20	25	30	35	40	45	50	55	65	75	85	95
**Prevalence**	**92.9**	**71.5**	**43.4**	**24.2**	**13.4**	**8.3**	**5.4**	**4.1**	**3.1**	**2.3**	**1.6**	**1.3**	**0.7**	**0.5**	**0.4**	**0.2**
**Lower CI**	90.2	68.0	40.2	21.8	11.9	7.2	4.4	3.3	2.5	1.8	1.1	0.9	0.4	0.2	0.1	0.1
**Upper CI**	94.9	74.8	46.7	26.7	15.1	9.7	6.5	4.9	3.9	3.0	2.2	1.9	1.3	1.1	1.0	1.0
**Design Effect**	1.66	1.38	1.20	1.04	0.86	0.83	0.83	0.73	0.71	0.73	0.75	0.78	0.90	1.03	1.16	1.37
**Number**	1229	1028	721	473	312	215	150	123	97	73	53	44	24	14	8	3

Table B2551-4 Age Group = 18-30 Gender = OVERALL Occupational group = OVERALL Total cases = 401

	Threshold dB HL≥															
	0	5	10	15	20	25	30	35	40	45	50	55	65	75	85	95
**Prevalence**	**88.5**	**53.6**	**20.9**	**6.4**	**2.2**	**1.6**	**0.9**	**0.5**	**0.3**	**0.2**	**0.0**	**0.0**	**0.0**	**0.0**	**0.0**	**0.0**
**Lower CI**	83.9	47.8	16.8	4.3	1.3	0.9	0.4	0.3	0.1	0.1	0.0	0.0	0.0	0.0	0.0	0.0
**Upper CI**	91.9	59.3	25.7	9.5	3.7	2.8	1.9	1.1	0.7	0.6	0.1	0.1	0.9	0.9	0.9	0.9
**Design Effect**	1.27	1.19	1.13	1.09	0.81	0.78	0.74	0.54	0.53	0.54	0.20	0.20	-	-	-	-
**Number**	364	231	106	43	21	15	10	8	5	4	2	2	0	0	0	0

Table B2551-5 Age Group = 31-40 Gender = OVERALL Occupational group = OVERALL Total cases = 331

	Threshold dB HL≥															
	0	5	10	15	20	25	30	35	40	45	50	55	65	75	85	95
**Prevalence**	**90.3**	**60.1**	**29.3**	**11.6**	**5.6**	**2.7**	**2.2**	**1.4**	**1.1**	**1.0**	**1.0**	**1.0**	**0.7**	**0.7**	**0.6**	**0.6**
**Lower CI**	85.4	53.5	23.8	8.2	3.5	1.4	1.0	0.6	0.4	0.3	0.3	0.3	0.1	0.1	0.1	0.1
**Upper CI**	93.7	66.3	35.4	16.0	8.9	5.1	4.6	3.4	3.1	3.0	3.0	3.0	3.0	3.0	3.1	3.1
**Design Effect**	1.29	1.23	1.19	1.12	1.08	1.02	1.07	0.98	1.08	1.13	1.13	1.13	1.27	1.27	1.36	1.36
**Number**	306	222	117	56	28	16	12	9	5	4	4	4	2	2	1	1

Table B2551-6 Age Group = 41-50 Gender = OVERALL Occupational group = OVERALL Total cases = 450

	Threshold dB HL≥															
	0	5	10	15	20	25	30	35	40	45	50	55	65	75	85	95
**Prevalence**	**96.5**	**74.1**	**39.5**	**19.3**	**10.3**	**6.2**	**4.4**	**3.4**	**2.8**	**2.1**	**1.2**	**0.7**	**0.3**	**0.3**	**0.3**	**0.1**
**Lower CI**	94.0	69.4	34.9	16.0	8.1	4.7	3.1	2.3	1.8	1.3	0.7	0.4	0.1	0.1	0.1	0.0
**Upper CI**	98.0	78.4	44.3	23.2	13.0	8.2	6.1	4.9	4.4	3.4	2.0	1.3	0.8	0.8	0.8	0.6
**Design Effect**	1.15	1.12	1.05	0.98	0.87	0.78	0.77	0.79	0.81	0.78	0.62	0.57	0.65	0.65	0.65	0.60
**Number**	437	348	202	116	72	49	38	29	24	18	13	8	3	3	3	2

Table B2551-7 Age Group = 51-60 Gender = OVERALL Occupational group = OVERALL Total cases = 680

	Threshold dB HL≥															
	0	5	10	15	20	25	30	35	40	45	50	55	65	75	85	95
**Prevalence**	**98.1**	**82.4**	**55.8**	**30.0**	**18.2**	**10.8**	**7.1**	**5.2**	**3.4**	**2.9**	**1.9**	**1.3**	**0.6**	**0.1**	**0.1**	**0.0**
**Lower CI**	96.5	78.9	51.6	26.7	15.5	8.8	5.7	4.0	2.5	2.1	1.3	0.8	0.3	0.0	0.0	0.0
**Upper CI**	99.0	85.4	59.9	33.7	21.2	13.2	8.9	6.8	4.6	4.1	2.8	2.1	1.2	0.3	0.3	0.2
**Design Effect**	1.18	1.14	1.12	1.02	0.98	0.94	0.83	0.82	0.75	0.77	0.74	0.73	0.68	0.50	0.47	0.51
**Number**	669	579	428	268	181	124	92	70	51	43	30	22	11	3	2	1

Prevalence by age group, gender and occupational group for the better ear over 0.25, 0.5, and 1 kHz

Table B2551-8 Age Group = 61-70 Gender = OVERALL Occupational group = OVERALL Total cases = 525

	Threshold dB HL≥														
	**0**	**5**	**10**	**15**	**20**	**25**	**30**	**35**	**40**	**45**	**50**	**55**	**65**	**75**	**85**	**95**
**Prevalence**	**99.5**	**90.5**	**67.9**	**47.6**	**30.7**	**19.7**	**12.4**	**10.5**	**7.4**	**4.5**	**2.9**	**2.4**	**1.7**	**1.3**	**0.8**	**0.5**
**Lower CI**	97.6	86.8	62.9	42.4	26.3	16.3	10.0	8.4	5.5	3.5	2.2	1.7	1.1	0.8	0.4	0.2
**Upper CI**	99.9	93.3	72.6	52.9	35.4	23.5	15.1	13.2	9.9	5.7	4.0	3.4	2.6	2.2	1.6	1.3
**Design Effect**	1.44	1.30	1.22	1.24	1.16	1.05	0.90	0.92	0.96	0.62	0.62	0.63	0.67	0.70	0.74	0.84
**Number**	523	487	386	280	208	146	107	91	71	51	37	31	21	15	8	3

Table B2551-9 Age Group = 71-80 Gender = OVERALL Occupational group = OVERALL Total cases = 271

	Threshold dB HL≥															
	**0**	**5**	**10**	**15**	**20**	**25**	**30**	**35**	**40**	**45**	**50**	**55**	**65**	**75**	**85**	**95**
**Prevalence**	**99.0**	**97.9**	**85.8**	**68.4**	**51.3**	**37.7**	**28.7**	**19.4**	**15.6**	**11.8**	**6.6**	**5.6**	**3.8**	**1.6**	**1.0**	**0.3**
**Lower CI**	96.8	95.3	79.9	60.6	43.7	31.0	22.7	15.9	12.3	8.7	4.9	4.0	2.7	0.9	0.4	0.1
**Upper CI**	99.7	99.1	90.2	75.2	58.9	44.9	35.6	23.6	19.5	15.8	8.8	7.6	5.4	2.9	2.2	1.0
**Design Effect**	1.06	1.04	1.24	1.33	1.29	1.22	1.21	0.82	0.83	0.92	0.64	0.65	0.59	0.65	0.72	0.61
**Number**	268	264	242	210	168	133	111	91	76	57	39	33	24	10	6	2

Table B2551-10 Age Group = 18-80 Gender = OVERALL Occupational group = NON-MANUAL Total cases = 1180

	Threshold dB HL≥															
	**0**	**5**	**10**	**15**	**20**	**25**	**30**	**35**	**40**	**45**	**50**	**55**	**65**	**75**	**85**	**95**
**Prevalence**	**94.0**	**67.4**	**38.8**	**21.3**	**13.2**	**8.3**	**5.8**	**3.3**	**2.4**	**1.7**	**1.1**	**0.9**	**0.7**	**0.2**	**0.1**	**0.1**
**Lower CI**	91.8	64.0	36.0	19.2	11.6	7.2	5.0	2.7	2.0	1.4	0.8	0.6	0.5	0.1	0.0	0.0
**Upper CI**	95.7	70.5	41.7	23.6	14.9	9.5	6.7	4.0	2.9	2.2	1.5	1.3	1.1	0.5	0.3	0.2
**Design Effect**	1.42	1.22	1.03	0.95	0.86	0.72	0.63	0.62	0.54	0.56	0.57	0.59	0.61	0.66	0.59	0.67
**Number**	1131	884	570	351	240	164	122	93	74	57	40	33	24	8	4	2

Table B2551-11 Age Group = 18-80 Gender = OVERALL Occupational group = MANUAL Total cases = 1474

	Threshold dB HL≥															
	**0**	**5**	**10**	**15**	**20**	**25**	**30**	**35**	**40**	**45**	**50**	**55**	**65**	**75**	**85**	**95**
**Prevalence**	**94.5**	**77.1**	**48.1**	**27.6**	**17.2**	**11.5**	**8.2**	**6.0**	**4.4**	**3.4**	**2.0**	**1.6**	**1.0**	**0.7**	**0.6**	**0.4**
**Lower CI**	92.1	73.9	45.0	25.2	15.5	10.2	7.1	5.2	3.7	2.7	1.6	1.2	0.7	0.5	0.3	0.2
**Upper CI**	96.1	80.1	51.1	30.1	19.1	13.0	9.4	7.0	5.3	4.2	2.5	2.0	1.4	1.2	1.0	0.8
**Design Effect**	1.69	1.44	1.20	1.06	0.93	0.87	0.81	0.73	0.77	0.80	0.61	0.64	0.71	0.77	0.84	0.94
**Number**	1432	1245	909	620	438	319	248	205	158	120	85	67	37	25	16	7

Table B2551-12 Age Group = 18-30 Gender = FEMALE Occupational group = OVERALL Total cases = 224

	Threshold dB HL≥															
	**0**	**5**	**10**	**15**	**20**	**25**	**30**	**35**	**40**	**45**	**50**	**55**	**65**	**75**	**85**	**95**
**Prevalence**	**92.3**	**54.4**	**18.3**	**6.2**	**3.9**	**2.7**	**1.6**	**0.9**	**0.4**	**0.3**	**0.0**	**0.0**	**0.0**	**0.0**	**0.0**	**0.0**
**Lower CI**	87.3	47.0	13.8	4.1	2.2	1.4	0.7	0.4	0.1	0.1	0.0	0.0	0.0	0.0	0.0	0.0
**Upper CI**	95.5	61.7	23.9	9.4	6.8	5.1	3.4	1.9	1.3	1.1	1.7	1.7	1.7	1.7	1.7	1.7
**Design Effect**	1.16	1.14	1.00	0.84	0.87	0.82	0.77	0.58	0.58	0.60	-	-	-	-	-	-
**Number**	208	127	57	27	17	12	8	6	3	2	0	0	0	0	0	0

Table B2551-13 Age Group = 31-40 Gender = FEMALE Occupational group = OVERALL Total cases = 189

	Threshold dB HL≥															
	**0**	**5**	**10**	**15**	**20**	**25**	**30**	**35**	**40**	**45**	**50**	**55**	**65**	**75**	**85**	**95**
**Prevalence**	**91.7**	**55.6**	**28.7**	**11.2**	**6.0**	**1.8**	**1.4**	**1.4**	**1.0**	**0.7**	**0.7**	**0.7**	**0.2**	**0.2**	**0.0**	**0.0**
**Lower CI**	85.6	47.3	22.1	7.2	3.3	0.9	0.7	0.7	0.4	0.3	0.3	0.3	0.0	0.0	0.0	0.0
**Upper CI**	95.3	63.5	36.4	16.8	10.6	3.4	3.0	3.0	2.6	2.2	2.2	2.2	1.2	1.2	2.0	2.0
**Design Effect**	1.22	1.16	1.12	1.06	1.05	0.63	0.64	0.64	0.69	0.69	0.69	0.69	0.63	0.63	-	-
**Number**	177	123	66	31	19	9	7	7	4	3	3	3	1	1	0	0

Table B2551-14 Age Group = 41-50 Gender = FEMALE Occupational group = OVERALL Total cases = 247

	Threshold dB HL≥															
	**0**	**5**	**10**	**15**	**20**	**25**	**30**	**35**	**40**	**45**	**50**	**55**	**65**	**75**	**85**	**95**
**Prevalence**	**95.4**	**74.6**	**39.6**	**18.5**	**10.0**	**6.5**	**5.2**	**4.1**	**3.4**	**2.8**	**1.1**	**0.5**	**0.1**	**0.1**	**0.1**	**0.1**
**Lower CI**	91.3	68.1	33.6	14.3	7.3	4.6	3.4	2.5	1.9	1.5	0.5	0.2	0.0	0.0	0.0	0.0
**Upper CI**	97.6	80.1	45.9	23.5	13.5	9.1	7.8	6.6	5.9	5.3	2.4	1.1	0.6	0.6	0.6	0.6
**Design Effect**	1.17	1.12	1.02	0.95	0.83	0.73	0.78	0.82	0.85	0.87	0.66	0.51	0.51	0.51	0.51	0.51
**Number**	238	193	113	62	40	29	24	18	15	12	7	4	1	1	1	1

Prevalence by age group, gender and occupational group for the better ear over 0.25, 0.5, and 1 kHz

Table B2551-15 Age Group = 51-60 Gender = FEMALE Occupational group = OVERALL Total cases = 340

	Threshold dB HL≥															
	0	5	10	15	20	25	30	35	40	45	50	55	65	75	85	95
Prevalence	98.1	84.5	55.4	28.7	19.3	11.5	7.8	5.7	3.0	2.5	1.7	1.0	0.6	0.2	0.1	0.1
Lower CI	95.6	79.7	49.6	24.3	15.5	8.7	5.7	3.9	1.9	1.6	1.0	0.5	0.3	0.1	0.0	0.0
Upper CI	99.2	88.3	61.2	33.7	23.7	15.2	10.5	8.2	4.5	3.9	3.0	1.9	1.6	0.6	0.5	0.4
Design Effect	1.16	1.12	1.10	0.98	0.98	0.96	0.84	0.86	0.69	0.69	0.71	0.63	0.71	0.48	0.46	0.50
Number	335	297	215	134	95	64	51	38	26	22	15	10	6	3	2	1

Table B2551-16 Age Group = 61-70 Gender = FEMALE Occupational group = OVERALL Total cases = 240

	Threshold dB HL≥															
	0	5	10	15	20	25	30	35	40	45	50	55	65	75	85	95
Prevalence	99.2	90.5	67.5	48.8	31.5	21.8	13.6	11.6	7.7	4.3	3.3	2.7	1.9	1.2	1.1	0.9
Lower CI	95.4	85.0	60.5	41.6	25.3	16.7	10.1	8.4	5.0	3.3	2.3	1.7	1.0	0.6	0.5	0.3
Upper CI	99.9	94.2	73.9	56.1	38.5	28.1	18.0	15.9	11.8	5.7	4.9	4.1	3.4	2.7	2.6	2.4
Design Effect	1.42	1.23	1.14	1.16	1.14	1.10	0.91	0.92	1.00	0.46	0.57	0.58	0.66	0.72	0.74	0.78
Number	239	223	177	131	96	72	56	48	37	28	22	18	11	6	5	3

Table B2551-17 Age Group = 71-80 Gender = FEMALE Occupational group = OVERALL Total cases = 143

	Threshold dB HL≥															
	0	5	10	15	20	25	30	35	40	45	50	55	65	75	85	95
Prevalence	99.2	99.0	90.2	69.5	54.3	41.3	34.5	21.6	18.1	13.9	7.3	6.4	5.1	2.2	1.2	0.2
Lower CI	96.9	96.9	80.7	58.2	43.6	32.0	25.6	16.9	13.5	9.5	5.1	4.3	3.4	1.1	0.4	0.0
Upper CI	99.8	99.7	95.2	78.8	64.7	51.3	44.5	27.3	23.9	19.9	10.3	9.4	7.5	4.3	3.3	1.1
Design Effect	0.83	0.76	1.46	1.40	1.32	1.22	1.23	0.77	0.82	0.92	0.61	0.64	0.56	0.63	0.72	0.53
Number	141	140	132	115	99	82	74	58	50	37	25	21	18	8	4	1

Table B2551-18 Age Group = 18-30 Gender = MALE Occupational group = OVERALL Total cases = 177

	Threshold dB HL≥															
	0	5	10	15	20	25	30	35	40	45	50	55	65	75	85	95
Prevalence	84.5	52.9	23.6	6.6	0.4	0.2	0.1	0.1	0.1	0.1	0.1	0.1	0.0	0.0	0.0	0.0
Lower CI	76.4	44.0	16.9	3.3	0.1	0.1	0.0	0.0	0.0	0.0	0.0	0.0	0.0	0.0	0.0	0.0
Upper CI	90.2	61.6	31.9	12.7	1.0	0.8	0.2	0.2	0.2	0.2	0.2	0.2	2.1	2.1	2.1	2.1
Design Effect	1.30	1.22	1.21	1.25	0.44	0.42	0.19	0.19	0.19	0.19	0.19	0.19	-	-	-	-
Number	156	104	49	16	4	3	2	2	2	2	2	2	0	0	0	0

Table B2551-19 Age Group = 31-40 Gender = MALE Occupational group = OVERALL Total cases = 142

	Threshold dB HL≥															
	0	5	10	15	20	25	30	35	40	45	50	55	65	75	85	95
Prevalence	88.8	65.0	30.1	12.1	5.3	3.8	3.2	1.5	1.2	1.2	1.2	1.2	1.2	1.2	1.2	1.2
Lower CI	80.2	54.4	21.6	7.1	2.4	1.5	1.1	0.4	0.2	0.2	0.2	0.2	0.2	0.2	0.2	0.2
Upper CI	94.0	74.3	40.2	19.9	11.3	9.2	8.8	6.4	6.5	6.5	6.5	6.5	6.5	6.5	6.5	6.5
Design Effect	1.32	1.30	1.25	1.18	1.15	1.15	1.20	1.20	1.31	1.31	1.31	1.31	1.31	1.31	1.31	1.31
Number	129	99	51	25	9	7	5	2	1	1	1	1	1	1	1	1

Table B2551-20 Age Group = 41-50 Gender = MALE Occupational group = OVERALL Total cases = 203

	Threshold dB HL≥															
	0	5	10	15	20	25	30	35	40	45	50	55	65	75	85	95
Prevalence	97.8	73.6	39.4	20.3	10.5	5.9	3.5	2.6	2.3	1.3	1.3	1.0	0.4	0.4	0.4	0.2
Lower CI	93.9	66.3	32.5	15.3	7.3	3.7	2.0	1.4	1.1	0.7	0.7	0.5	0.1	0.1	0.1	0.0
Upper CI	99.2	79.8	46.7	26.4	15.0	9.3	6.0	4.7	4.4	2.6	2.6	2.2	1.6	1.6	1.6	1.1
Design Effect	1.17	1.12	1.07	1.00	0.91	0.84	0.76	0.73	0.76	0.58	0.58	0.59	0.67	0.67	0.67	0.63
Number	199	155	89	54	32	20	14	11	9	6	6	4	2	2	2	1

Table B2551-21 Age Group = 51-60 Gender = MALE Occupational group = OVERALL Total cases = 340

	Threshold dB HL≥															
	0	5	10	15	20	25	30	35	40	45	50	55	65	75	85	95
Prevalence	98.0	80.0	56.1	31.5	17.0	10.1	6.5	4.8	3.9	3.4	2.1	1.7	0.6	0.0	0.0	0.0
Lower CI	95.3	74.7	50.1	26.5	13.5	7.6	4.7	3.3	2.6	2.1	1.2	0.9	0.2	0.0	0.0	0.0
Upper CI	99.2	84.5	61.9	37.0	21.1	13.4	8.9	6.9	5.9	5.5	3.6	3.2	1.4	1.1	1.1	1.1
Design Effect	1.21	1.16	1.13	1.07	0.96	0.90	0.81	0.77	0.80	0.84	0.79	0.81	0.65	-	-	-
Number	334	282	213	134	86	60	41	32	25	21	15	12	5	0	0	0

Prevalence by age group, gender and occupational group for the better ear over 0.25, 0.5, and 1 kHz

Table B2551-22 Age Group = 61-70 Gender = MALE Occupational group = OVERALL Total cases = 285

Threshold dB HL≥	0	5	10	15	20	25	30	35	40	45	50	55	65	75	85	95
Prevalence	**99.8**	**90.7**	**68.6**	**46.3**	**29.7**	**17.2**	**11.0**	**9.3**	**7.1**	**4.6**	**2.5**	**2.2**	**1.5**	**1.4**	**0.4**	**0.0**
Lower CI	98.8	85.1	61.3	38.9	24.1	13.7	8.3	6.8	5.0	3.0	1.5	1.3	0.8	0.7	0.2	0.0
Upper CI	100.0	94.3	75.1	53.9	36.1	21.3	14.4	12.6	10.1	7.0	4.0	3.7	2.8	2.7	1.1	1.3
Design Effect	0.77	1.34	1.29	1.31	1.14	0.86	0.83	0.86	0.83	0.81	0.67	0.70	0.67	0.69	0.57	-
Number	284	264	209	149	112	74	51	43	34	23	15	13	10	9	3	0

Table B2551-23 Age Group = 71-80 Gender = MALE Occupational group = OVERALL Total cases = 128

Threshold dB HL≥	0	5	10	15	20	25	30	35	40	45	50	55	65	75	85	95
Prevalence	**98.7**	**96.4**	**78.4**	**66.1**	**46.3**	**31.9**	**20.2**	**16.3**	**12.0**	**8.9**	**5.7**	**4.5**	**2.1**	**0.7**	**0.7**	**0.4**
Lower CI	92.7	90.2	70.3	56.1	36.6	23.6	13.9	11.7	8.5	5.9	3.4	2.7	1.0	0.2	0.2	0.1
Upper CI	99.8	98.7	84.7	74.8	56.4	41.7	28.3	22.3	16.6	13.3	9.3	7.5	4.4	2.4	2.4	2.0
Design Effect	1.32	1.21	1.02	1.16	1.17	1.14	1.05	0.83	0.72	0.74	0.72	0.65	0.65	0.66	0.66	0.67
Number	127	124	110	95	69	51	37	33	26	20	14	12	6	2	2	1

Table B2551-24 Age Group = 18-30 Gender = OVERALL Occupational group = NON-MANUAL Total cases = 181

Threshold dB HL≥	0	5	10	15	20	25	30	35	40	45	50	55	65	75	85	95
Prevalence	**87.1**	**44.1**	**15.8**	**4.2**	**1.3**	**1.0**	**0.7**	**0.7**	**0.2**	**0.1**	**0.1**	**0.1**	**0.0**	**0.0**	**0.0**	**0.0**
Lower CI	80.3	36.0	10.8	1.8	0.6	0.3	0.2	0.2	0.1	0.0	0.0	0.0	0.0	0.0	0.0	0.0
Upper CI	91.8	52.7	22.5	9.6	3.1	3.0	2.4	2.4	0.7	2.2	2.2	2.2	2.1	2.1	2.1	2.1
Design Effect	1.18	1.17	1.11	1.25	0.69	0.79	0.79	0.79	0.43	0.00	0.00	0.00	-	-	-	-
Number	162	87	36	12	5	4	3	3	2	1	1	1	0	0	0	0

Table B2551-25 Age Group = 31-40 Gender = OVERALL Occupational group = NON-MANUAL Total cases = 148

Threshold dB HL≥	0	5	10	15	20	25	30	35	40	45	50	55	65	75	85	95
Prevalence	**92.0**	**56.9**	**28.7**	**9.3**	**5.2**	**1.3**	**0.7**	**0.4**	**0.3**	**0.3**	**0.3**	**0.3**	**0.0**	**0.0**	**0.0**	**0.0**
Lower CI	84.3	47.0	21.8	5.3	2.3	0.4	0.2	0.1	0.1	0.1	0.1	0.1	0.0	0.0	0.0	0.0
Upper CI	96.1	66.3	36.7	15.7	11.2	3.8	2.1	1.6	1.7	1.7	1.7	1.7	2.5	2.5	2.5	2.5
Design Effect	1.31	1.23	1.03	1.09	1.19	0.81	0.62	0.59	0.67	0.67	0.67	0.67	-	-	-	-
Number	137	90	44	17	9	4	3	2	1	1	1	1	0	0	0	0

Table B2551-26 Age Group = 41-50 Gender = OVERALL Occupational group = NON-MANUAL Total cases = 188

Threshold dB HL≥	0	5	10	15	20	25	30	35	40	45	50	55	65	75	85	95
Prevalence	**97.7**	**72.3**	**30.9**	**13.8**	**7.3**	**4.8**	**3.7**	**0.7**	**0.4**	**0.4**	**0.1**	**0.0**	**0.0**	**0.0**	**0.0**	**0.0**
Lower CI	94.2	64.7	24.7	9.6	4.7	2.7	1.9	0.4	0.2	0.2	0.0	0.0	0.0	0.0	0.0	0.0
Upper CI	99.2	78.8	38.0	19.4	11.2	8.5	7.1	1.1	0.7	0.7	0.7	2.0	2.0	2.0	2.0	2.0
Design Effect	1.07	1.11	1.01	0.99	0.85	0.92	0.91	0.30	0.25	0.25	0.48	-	-	-	-	-
Number	183	137	63	31	16	10	8	4	2	2	1	0	0	0	0	0

Table B2551-27 Age Group = 51-60 Gender = OVERALL Occupational group = NON-MANUAL Total cases = 316

Threshold dB HL≥	0	5	10	15	20	25	30	35	40	45	50	55	65	75	85	95
Prevalence	**96.8**	**77.2**	**47.3**	**23.0**	**13.1**	**6.5**	**4.1**	**2.7**	**2.1**	**1.7**	**1.2**	**0.7**	**0.6**	**0.2**	**0.1**	**0.0**
Lower CI	94.0	71.9	40.8	18.7	10.1	4.5	2.9	1.8	1.3	1.0	0.6	0.3	0.2	0.0	0.0	0.0
Upper CI	98.4	81.8	54.0	27.8	16.8	9.1	5.7	3.9	3.3	2.9	2.3	1.6	1.6	0.7	0.4	1.2
Design Effect	1.07	1.08	1.21	0.98	0.89	0.83	0.65	0.60	0.62	0.63	0.65	0.67	0.69	0.54	0.46	-
Number	306	249	168	100	65	41	30	21	17	14	10	6	5	2	1	0

Table B2551-28 Age Group = 61-70 Gender = OVERALL Occupational group = NON-MANUAL Total cases = 225

Threshold dB HL≥	0	5	10	15	20	25	30	35	40	45	50	55	65	75	85	95
Prevalence	**99.7**	**89.4**	**68.0**	**44.3**	**29.5**	**18.4**	**10.6**	**9.1**	**7.7**	**5.1**	**2.7**	**2.5**	**1.9**	**1.2**	**0.5**	**0.4**
Lower CI	98.4	83.7	61.5	37.9	22.5	14.3	7.8	6.5	5.6	3.4	1.5	1.4	0.9	0.4	0.1	0.1
Upper CI	99.9	93.2	73.9	50.8	37.7	23.4	14.2	12.6	10.7	7.5	4.6	4.4	3.9	3.3	1.6	1.6
Design Effect	0.80	1.18	1.02	1.00	1.29	0.89	0.79	0.80	0.73	0.72	0.71	0.72	0.80	0.90	0.69	0.75
Number	224	204	156	102	78	56	37	31	28	22	15	14	9	5	3	2

Prevalence by age group, gender and occupational group for the better ear over 0.25, 0.5, and 1 kHz

Table B2551-29 Age Group = 71-80 Gender = OVERALL Occupational group = NON-MANUAL Total cases = 122

Threshold dB HL≥	0	5	10	15	20	25	30	35	40	45	50	55	65	75	85	95
Prevalence	**98.1**	**96.7**	**79.2**	**68.9**	**48.1**	**36.3**	**29.2**	**12.8**	**8.8**	**6.4**	**4.4**	**4.1**	**3.7**	**0.4**	**0.0**	**0.0**
Lower CI	94.0	91.4	68.2	56.8	39.6	29.4	23.7	9.8	6.6	4.2	2.7	2.3	2.1	0.1	0.0	0.0
Upper CI	99.4	98.8	87.2	78.8	56.7	43.9	35.4	16.6	11.7	9.6	7.3	7.1	6.7	2.0	3.1	3.1
Design Effect	0.98	1.06	1.33	1.37	0.98	0.86	0.73	0.57	0.51	0.61	0.63	0.66	0.66	0.66	-	-
Number	119	117	103	89	67	49	41	32	24	17	12	11	10	1	0	0

Table B2551-30 Age Group = 18-30 Gender = OVERALL Occupational group = MANUAL Total cases = 218

Threshold dB HL≥	0	5	10	15	20	25	30	35	40	45	50	55	65	75	85	95
Prevalence	**89.6**	**64.3**	**26.5**	**8.2**	**3.4**	**2.2**	**1.2**	**0.5**	**0.2**	**0.2**	**0.0**	**0.0**	**0.0**	**0.0**	**0.0**	**0.0**
Lower CI	82.4	55.5	20.0	4.8	1.7	1.0	0.4	0.2	0.1	0.1	0.0	0.0	0.0	0.0	0.0	0.0
Upper CI	94.1	72.3	34.2	13.8	6.7	4.7	3.2	1.1	0.8	0.8	0.2	0.2	1.7	1.7	1.7	1.7
Design Effect	1.43	1.34	1.22	1.21	1.00	0.90	0.87	0.48	0.47	0.47	0.26	0.26	-	-	-	-
Number	200	144	70	31	16	11	7	5	3	3	1	1	0	0	0	0

Table B2551-31 Age Group = 31-40 Gender = OVERALL Occupational group = MANUAL Total cases = 183

Threshold dB HL≥	0	5	10	15	20	25	30	35	40	45	50	55	65	75	85	95
Prevalence	**89.6**	**64.1**	**29.0**	**13.5**	**6.1**	**4.0**	**3.6**	**2.1**	**1.6**	**1.3**	**1.3**	**1.3**	**1.1**	**1.1**	**0.9**	**0.9**
Lower CI	81.9	54.9	21.5	8.6	3.2	1.7	1.4	0.8	0.5	0.3	0.3	0.3	0.2	0.2	0.2	0.2
Upper CI	94.2	72.4	37.9	20.4	11.3	9.1	8.8	5.4	5.0	4.8	4.8	4.8	4.9	4.9	5.0	5.0
Design Effect	1.38	1.28	1.27	1.19	1.14	1.22	1.27	0.99	1.08	1.15	1.15	1.15	1.22	1.22	1.30	1.30
Number	169	132	73	39	19	12	9	7	4	3	3	3	2	2	1	1

Table B2551-32 Age Group = 41-50 Gender = OVERALL Occupational group = MANUAL Total cases = 262

Threshold dB HL≥	0	5	10	15	20	25	30	35	40	45	50	55	65	75	85	95
Prevalence	**95.7**	**76.4**	**49.4**	**25.3**	**12.6**	**7.2**	**5.3**	**4.7**	**4.1**	**3.2**	**1.6**	**0.9**	**0.3**	**0.3**	**0.3**	**0.2**
Lower CI	91.6	69.5	42.5	19.9	9.4	5.1	3.6	3.0	2.6	1.8	1.1	0.5	0.1	0.1	0.1	0.1
Upper CI	97.9	82.1	56.3	31.5	16.8	9.9	7.7	7.1	6.6	5.4	2.5	1.7	0.9	0.9	0.9	0.7
Design Effect	1.23	1.23	1.16	1.11	0.92	0.77	0.76	0.79	0.81	0.82	0.44	0.49	0.52	0.52	0.52	0.52
Number	254	211	139	85	56	39	30	25	22	16	12	8	3	3	3	2

Table B2551-33 Age Group = 51-60 Gender = OVERALL Occupational group = MANUAL Total cases = 364

Threshold dB HL≥	0	5	10	15	20	25	30	35	40	45	50	55	65	75	85	95
Prevalence	**99.3**	**87.6**	**61.3**	**35.1**	**22.6**	**15.1**	**9.1**	**7.2**	**4.1**	**3.4**	**2.0**	**1.5**	**0.6**	**0.1**	**0.1**	**0.1**
Lower CI	96.1	82.5	55.0	29.8	18.3	11.6	7.0	5.3	2.9	2.2	1.3	0.9	0.2	0.0	0.0	0.0
Upper CI	99.9	91.3	67.2	40.8	27.7	19.5	11.8	9.8	5.9	5.0	3.3	2.5	1.3	0.5	0.5	0.5
Design Effect	1.61	1.29	1.23	1.13	1.09	1.06	0.81	0.85	0.73	0.74	0.68	0.62	0.62	0.56	0.56	0.56
Number	363	330	260	168	116	83	62	49	34	29	20	16	6	1	1	1

Table B2551-34 Age Group = 61-70 Gender = OVERALL Occupational group = MANUAL Total cases = 299

Threshold dB HL≥	0	5	10	15	20	25	30	35	40	45	50	55	65	75	85	95
Prevalence	**99.0**	**91.0**	**66.5**	**49.6**	**31.5**	**19.9**	**13.6**	**12.0**	**7.8**	**4.9**	**3.6**	**2.7**	**2.0**	**1.8**	**1.3**	**0.9**
Lower CI	94.4	84.7	58.8	42.4	26.4	16.0	11.4	10.0	5.3	3.6	2.6	2.0	1.5	1.3	0.9	0.3
Upper CI	99.8	94.8	73.4	56.9	37.2	24.6	16.2	14.4	11.4	6.7	4.9	3.7	2.7	2.4	1.7	2.7
Design Effect	1.75	1.54	1.38	1.29	1.04	0.96	0.61	0.60	0.98	0.63	0.54	0.46	0.40	0.39	0.29	0.00
Number	298	282	229	177	130	90	70	60	43	29	22	17	12	10	5	1

Table B2551-35 Age Group = 71-80 Gender = OVERALL Occupational group = MANUAL Total cases = 148

Threshold dB HL≥	0	5	10	15	20	25	30	35	40	45	50	55	65	75	85	95
Prevalence	**100.0**	**99.3**	**93.0**	**70.2**	**57.4**	**42.3**	**33.3**	**22.3**	**19.5**	**15.6**	**7.6**	**6.0**	**4.0**	**2.5**	**1.7**	**0.5**
Lower CI	97.5	98.9	84.4	60.4	46.6	34.2	25.7	15.8	13.4	9.8	5.4	4.1	2.5	1.3	0.7	0.1
Upper CI	100.0	99.6	97.0	78.4	67.6	50.9	42.0	30.3	27.5	24.0	10.6	8.6	6.3	4.6	3.8	1.7
Design Effect	-	0.25	1.46	1.24	1.35	1.07	1.09	1.09	1.12	1.22	0.61	0.58	0.60	0.62	0.70	0.58
Number	148	146	138	120	101	84	70	59	52	40	27	22	14	9	6	2

Prevalence by age group, gender and occupational group for the better ear over 0.25, 0.5, and 1 kHz

Table B2551-36 — Age Group = 18-80 — Gender = FEMALE — Occupational group = NON-MANUAL — Total cases = 668

Threshold dB HL≥	0	5	10	15	20	25	30	35	40	45	50	55	65	75	85	95
Prevalence	**94.8**	**68.1**	**39.6**	**23.3**	**15.2**	**9.8**	**7.4**	**3.8**	**2.6**	**2.1**	**1.5**	**1.3**	**1.0**	**0.3**	**0.2**	**0.1**
Lower CI	92.3	64.3	36.1	20.5	13.1	8.4	6.4	3.0	2.0	1.6	1.0	0.9	0.6	0.1	0.1	0.0
Upper CI	96.5	71.8	43.3	26.4	17.6	11.6	8.7	4.8	3.3	2.7	2.1	1.9	1.5	0.6	0.5	0.5
Design Effect	1.22	1.06	0.97	0.92	0.82	0.71	0.57	0.59	0.50	0.51	0.57	0.59	0.61	0.62	0.62	0.70
Number	641	500	325	205	145	100	84	64	49	40	29	25	18	6	4	2

Table B2551-37 — Age Group = 18-80 — Gender = MALE — Occupational group = NON-MANUAL — Total cases = 512

Threshold dB HL≥	0	5	10	15	20	25	30	35	40	45	50	55	65	75	85	95
Prevalence	**93.0**	**66.7**	**37.9**	**19.1**	**10.9**	**6.6**	**4.0**	**2.7**	**2.3**	**1.4**	**0.7**	**0.5**	**0.4**	**0.2**	**0.0**	**0.0**
Lower CI	88.8	61.0	33.5	16.0	8.7	5.2	3.0	1.9	1.6	0.9	0.4	0.2	0.2	0.0	0.0	0.0
Upper CI	95.8	71.9	42.5	22.6	13.6	8.3	5.4	3.8	3.2	2.2	1.3	1.1	1.0	0.8	0.7	0.7
Design Effect	1.55	1.34	1.08	0.97	0.90	0.72	0.71	0.66	0.59	0.64	0.61	0.64	0.68	0.75		
Number	490	384	245	146	95	64	38	29	25	17	11	8	6	2	0	0

Table B2551-38 — Age Group = 18-80 — Gender = FEMALE — Occupational group = MANUAL — Total cases = 714

Threshold dB HL≥	0	5	10	15	20	25	30	35	40	45	50	55	65	75	85	95
Prevalence	**95.9**	**78.0**	**48.2**	**27.1**	**18.8**	**13.0**	**9.1**	**7.1**	**5.2**	**3.8**	**2.1**	**1.5**	**1.1**	**0.8**	**0.6**	**0.3**
Lower CI	92.9	73.6	44.4	24.1	16.2	11.0	7.6	5.9	4.1	2.9	1.6	1.1	0.8	0.6	0.4	0.3
Upper CI	97.6	81.9	52.0	30.2	21.7	15.3	10.9	8.5	6.6	5.1	2.6	2.0	1.5	1.2	0.9	0.5
Design Effect	1.56	1.36	1.04	0.94	0.95	0.86	0.77	0.69	0.76	0.79	0.46	0.47	0.46	0.46	0.47	0.24
Number	696	602	434	294	221	168	136	111	86	64	43	31	19	13	8	4

Table B2551-39 — Age Group = 18-80 — Gender = MALE — Occupational group = MANUAL — Total cases = 760

Threshold dB HL≥	0	5	10	15	20	25	30	35	40	45	50	55	65	75	85	95
Prevalence	**92.9**	**76.2**	**47.9**	**28.2**	**15.5**	**9.9**	**7.2**	**4.8**	**3.6**	**2.9**	**2.0**	**1.7**	**0.9**	**0.7**	**0.5**	**0.4**
Lower CI	88.9	71.4	43.0	24.5	13.4	8.2	5.7	3.8	2.8	2.1	1.4	1.1	0.5	0.3	0.2	0.1
Upper CI	95.6	80.5	52.9	32.1	17.9	11.9	8.9	6.1	4.7	3.9	2.9	2.6	1.8	1.6	1.6	1.6
Design Effect	1.81	1.52	1.39	1.19	0.87	0.86	0.86	0.75	0.73	0.76	0.77	0.79	0.96	1.07	1.16	1.36
Number	736	643	475	326	217	151	112	94	72	56	42	36	18	12	8	3

Table B2551-40 — Age Group = 18-30 — Gender = FEMALE — Occupational group = NON-MANUAL — Total cases = 115

Threshold dB HL≥	0	5	10	15	20	25	30	35	40	45	50	55	65	75	85	95
Prevalence	**90.4**	**46.4**	**13.6**	**4.5**	**2.4**	**1.8**	**1.1**	**1.1**	**0.3**	**0.0**	**0.0**	**0.0**	**0.0**	**0.0**	**0.0**	**0.0**
Lower CI	82.9	37.3	8.7	2.4	1.0	0.6	0.3	0.3	0.0	0.0	0.0	0.0	0.0	0.0	0.0	0.0
Upper CI	94.8	55.8	20.5	8.3	5.7	5.5	4.5	4.5	1.5	3.2	3.2	3.2	3.2	3.2	3.2	3.2
Design Effect	1.09	1.04	0.94	0.76	0.78	0.90	0.89	0.89	0.55							
Number	105	57	21	9	4	3	2	2	1	0	0	0	0	0	0	0

Table B2551-41 — Age Group = 31-40 — Gender = FEMALE — Occupational group = NON-MANUAL — Total cases = 86

Threshold dB HL≥	0	5	10	15	20	25	30	35	40	45	50	55	65	75	85	95
Prevalence	**90.0**	**50.7**	**28.0**	**10.5**	**5.3**	**0.7**	**0.7**	**0.7**	**0.6**	**0.6**	**0.6**	**0.6**	**0.0**	**0.0**	**0.0**	**0.0**
Lower CI	81.1	39.2	19.2	5.2	2.0	0.2	0.2	0.2	0.1	0.1	0.1	0.1	0.0	0.0	0.0	0.0
Upper CI	95.0	62.1	39.0	20.1	13.5	3.0	3.0	3.0	3.2	3.2	3.2	3.2	4.3	4.3	4.3	4.3
Design Effect	1.08	1.11	1.07	1.13	1.13	0.62	0.62	0.62	0.71	0.71	0.71	0.71				
Number	78	48	24	10	6	2	2	2	1	1	1	1	0	0	0	0

Table B2551-42 — Age Group = 41-50 — Gender = FEMALE — Occupational group = NON-MANUAL — Total cases = 112

Threshold dB HL≥	0	5	10	15	20	25	30	35	40	45	50	55	65	75	85	95
Prevalence	**98.0**	**71.3**	**31.2**	**15.0**	**8.2**	**3.9**	**1.9**	**0.8**	**0.6**	**0.6**	**0.0**	**0.0**	**0.0**	**0.0**	**0.0**	**0.0**
Lower CI	93.7	61.7	23.7	10.4	5.2	2.0	1.5	0.4	0.1	0.1	0.0	0.0	0.0	0.0	0.0	0.0
Upper CI	99.4	79.4	39.9	21.2	12.5	7.5	2.5	1.5	4.3	4.3	3.3	3.3	3.3	3.3	3.3	3.3
Design Effect	0.97	1.07	0.96	0.82	0.71	0.74	0.20	0.30	0.00	0.00	-	-	-	-	-	-
Number	109	82	40	19	10	5	3	2	1	1	0	0	0	0	0	0

Prevalence by age group, gender and occupational group for the better ear over 0.25, 0.5, and 1 kHz

Table B2551-43 Age Group = 51-60 Gender = FEMALE Occupational group = NON-MANUAL Total cases = 175

	Threshold dB HL≥															
	0	5	10	15	20	25	30	35	40	45	50	55	65	75	85	95
**Prevalence**	**97.6**	**81.6**	**49.7**	**24.0**	**16.1**	**8.4**	**5.9**	**3.7**	**2.6**	**2.4**	**1.4**	**0.8**	**0.8**	**0.3**	**0.1**	**0.0**
**Lower CI**	93.9	75.0	41.9	18.5	11.5	5.3	3.9	2.3	1.5	1.3	0.6	0.2	0.2	0.1	0.0	0.0
**Upper CI**	99.1	86.7	57.7	30.6	22.0	13.1	8.8	5.7	4.5	4.4	3.2	2.5	2.5	1.2	0.7	2.1
**Design Effect**	1.05	1.03	1.08	0.97	0.96	0.94	0.68	0.60	0.62	0.66	0.69	0.76	0.76	0.55	0.48	-
**Number**	171	146	99	57	40	25	22	15	11	10	6	3	3	2	1	0

Table B2551-44 Age Group = 61-70 Gender = FEMALE Occupational group = NON-MANUAL Total cases = 113

	Threshold dB HL≥															
	0	5	10	15	20	25	30	35	40	45	50	55	65	75	85	95
**Prevalence**	**100.0**	**89.4**	**68.9**	**44.7**	**27.7**	**18.1**	**9.9**	**7.8**	**5.2**	**4.1**	**3.0**	**3.0**	**1.8**	**0.9**	**0.9**	**0.7**
**Lower CI**	96.7	81.6	60.1	35.7	19.9	12.2	6.3	4.7	3.3	2.8	1.7	1.7	0.8	0.3	0.3	0.2
**Upper CI**	100.0	94.1	76.6	54.0	37.1	25.9	15.1	12.8	7.9	6.0	5.1	5.1	3.9	3.1	3.1	3.1
**Design Effect**	0.00	1.09	0.98	1.01	1.06	0.97	0.79	0.80	0.55	0.44	0.53	0.53	0.60	0.68	0.68	0.74
**Number**	113	103	83	58	42	31	23	19	16	14	11	11	6	3	3	2

Table B2551-45 Age Group = 71-80 Gender = FEMALE Occupational group = NON-MANUAL Total cases = 67

	Threshold dB HL≥															
	0	5	10	15	20	25	30	35	40	45	50	55	65	75	85	95
**Prevalence**	**98.2**	**97.9**	**81.5**	**73.9**	**55.3**	**44.5**	**40.4**	**14.0**	**10.4**	**8.0**	**6.6**	**6.0**	**5.5**	**0.6**	**0.0**	**0.0**
**Lower CI**	92.4	92.6	63.8	55.2	43.9	35.8	32.7	11.6	7.4	5.1	3.9	3.3	3.0	0.1	0.0	0.0
**Upper CI**	99.6	99.4	91.6	86.6	66.2	53.6	48.7	16.7	14.3	12.5	11.1	10.7	10.0	3.4	5.4	5.4
**Design Effect**	0.91	0.84	1.54	1.57	0.96	0.76	0.69	0.31	0.47	0.56	0.59	0.63	0.62	0.64	-	-
**Number**	65	64	58	52	43	34	32	24	19	14	11	10	9	1	0	0

Table B2551-46 Age Group = 18-30 Gender = MALE Occupational group = NON-MANUAL Total cases = 66

	Threshold dB HL≥															
	0	5	10	15	20	25	30	35	40	45	50	55	65	75	85	95
**Prevalence**	**83.4**	**41.9**	**17.9**	**3.8**	**0.1**	**0.1**	**0.1**	**0.1**	**0.1**	**0.1**	**0.1**	**0.1**	**0.0**	**0.0**	**0.0**	**0.0**
**Lower CI**	71.1	28.8	10.0	0.8	0.0	0.0	0.0	0.0	0.0	0.0	0.0	0.0	0.0	0.0	0.0	0.0
**Upper CI**	91.2	56.2	30.1	17.1	5.7	5.7	5.7	5.7	5.7	5.7	5.7	5.7	5.5	5.5	5.5	5.5
**Design Effect**	1.13	1.20	1.10	1.46	0.00	0.00	0.00	0.00	0.00	0.00	0.00	0.00	-	-	-	-
**Number**	57	30	15	3	1	1	1	1	1	1	1	1	0	0	0	0

Table B2551-47 Age Group = 31-40 Gender = MALE Occupational group = NON-MANUAL Total cases = 62

	Threshold dB HL≥															
	0	5	10	15	20	25	30	35	40	45	50	55	65	75	85	95
**Prevalence**	**93.9**	**63.8**	**29.9**	**7.9**	**5.1**	**1.9**	**0.6**	**0.0**	**0.0**	**0.0**	**0.0**	**0.0**	**0.0**	**0.0**	**0.0**	**0.0**
**Lower CI**	76.7	46.9	19.9	3.2	1.4	0.5	0.1	0.0	0.0	0.0	0.0	0.0	0.0	0.0	0.0	0.0
**Upper CI**	98.6	77.8	42.2	18.0	16.6	7.1	3.5	5.8	5.8	5.8	5.8	5.8	5.8	5.8	5.8	5.8
**Design Effect**	1.64	1.36	1.01	1.06	1.25	0.82	0.62	-	-	-	-	-	-	-	-	-
**Number**	59	42	20	7	3	2	1	0	0	0	0	0	0	0	0	0

Table B2551-48 Age Group = 41-50 Gender = MALE Occupational group = NON-MANUAL Total cases = 76

	Threshold dB HL≥															
	0	5	10	15	20	25	30	35	40	45	50	55	65	75	85	95
**Prevalence**	**97.4**	**73.6**	**30.6**	**12.5**	**6.5**	**5.8**	**5.8**	**0.5**	**0.3**	**0.3**	**0.3**	**0.0**	**0.0**	**0.0**	**0.0**	**0.0**
**Lower CI**	90.0	61.1	21.1	6.3	2.8	2.4	2.4	0.2	0.0	0.0	0.0	0.0	0.0	0.0	0.0	0.0
**Upper CI**	99.4	83.2	42.2	23.5	14.0	13.3	13.3	1.3	1.4	1.4	1.4	4.8	4.8	4.8	4.8	4.8
**Design Effect**	1.10	1.14	1.04	1.15	0.97	0.99	0.99	0.32	0.44	0.44	0.44	-	-	-	-	-
**Number**	74	55	23	12	6	5	5	2	1	1	1	0	0	0	0	0

Table B2551-49 Age Group = 51-60 Gender = MALE Occupational group = NON-MANUAL Total cases = 141

	Threshold dB HL≥															
	0	5	10	15	20	25	30	35	40	45	50	55	65	75	85	95
**Prevalence**	**95.9**	**72.4**	**44.7**	**21.8**	**9.7**	**4.3**	**2.1**	**1.6**	**1.6**	**1.0**	**1.0**	**0.6**	**0.5**	**0.0**	**0.0**	**0.0**
**Lower CI**	90.7	63.6	34.3	15.9	6.6	2.8	1.0	0.7	0.7	0.4	0.4	0.2	0.1	0.0	0.0	0.0
**Upper CI**	98.3	79.8	55.6	29.2	14.1	6.5	4.0	3.4	3.4	2.5	2.5	1.8	1.8	2.7	2.7	2.7
**Design Effect**	1.08	1.11	1.33	0.98	0.77	0.55	0.61	0.63	0.63	0.61	0.61	0.54	0.60	-	-	-
**Number**	135	103	69	43	25	16	8	6	6	4	4	3	2	0	0	0

Prevalence by age group, gender and occupational group for the better ear over 0.25, 0.5, and 1 kHz

Table B2551-50 Age Group = 61-70 Gender = MALE Occupational group = NON-MANUAL Total cases = 112

Threshold dB HL≥	0	5	10	15	20	25	30	35	40	45	50	55	65	75	85	95
**Prevalence**	**99.4**	**89.8**	**68.2**	**45.0**	**32.2**	**19.4**	**11.8**	**10.7**	**10.7**	**6.2**	**2.4**	**2.1**	**2.1**	**1.6**	**0.0**	**0.0**
**Lower CI**	96.4	80.8	58.6	36.4	20.6	14.1	7.8	6.9	6.9	3.3	0.8	0.6	0.6	0.4	0.0	0.0
**Upper CI**	99.9	94.9	76.4	53.9	46.6	26.0	17.5	16.3	16.3	11.3	6.9	6.8	6.8	6.5	3.3	3.3
**Design Effect**	0.85	1.24	1.05	0.96	1.55	0.82	0.80	0.81	0.81	0.87	0.95	1.01	1.01	1.08	-	-
**Number**	111	101	73	44	36	25	14	12	12	8	4	3	3	2	0	0

Table B2551-51 Age Group = 71-80 Gender = MALE Occupational group = NON-MANUAL Total cases = 55

Threshold dB HL≥	0	5	10	15	20	25	30	35	40	45	50	55	65	75	85	95
**Prevalence**	**97.9**	**95.0**	**75.1**	**61.0**	**36.9**	**24.1**	**13.1**	**11.2**	**6.5**	**4.2**	**1.3**	**1.3**	**1.3**	**0.0**	**0.0**	**0.0**
**Lower CI**	89.0	82.9	64.3	48.5	25.7	14.7	7.3	5.7	3.5	1.6	0.2	0.2	0.2	0.0	0.0	0.0
**Upper CI**	99.6	98.7	83.5	72.3	49.8	37.1	22.2	20.8	11.8	10.4	7.0	7.0	7.0	6.5	6.5	6.5
**Design Effect**	1.08	1.22	0.85	0.95	0.97	1.01	0.83	0.89	0.62	0.77	0.85	0.85	0.85	-	-	-
**Number**	54	53	45	37	24	15	9	8	5	3	1	1	1	0	0	0

Table B2551-52 Age Group = 18-30 Gender = FEMALE Occupational group = MANUAL Total cases = 109

Threshold dB HL≥	0	5	10	15	20	25	30	35	40	45	50	55	65	75	85	95
**Prevalence**	**94.1**	**65.5**	**23.7**	**7.7**	**6.1**	**3.9**	**2.2**	**0.8**	**0.4**	**0.4**	**0.0**	**0.0**	**0.0**	**0.0**	**0.0**	**0.0**
**Lower CI**	83.6	53.0	16.1	4.1	2.9	1.7	0.8	0.3	0.1	0.1	0.0	0.0	0.0	0.0	0.0	0.0
**Upper CI**	98.0	76.2	33.3	14.0	12.5	8.6	6.0	2.1	1.4	1.4	3.4	3.4	3.4	3.4	3.4	3.4
**Design Effect**	1.51	1.34	1.09	0.96	1.03	0.89	0.86	0.48	0.48	0.48	-	-	-	-	-	-
**Number**	103	70	36	18	13	9	6	4	2	2	0	0	0	0	0	0

Table B2551-53 Age Group = 31-40 Gender = FEMALE Occupational group = MANUAL Total cases = 103

Threshold dB HL≥	0	5	10	15	20	25	30	35	40	45	50	55	65	75	85	95
**Prevalence**	**93.7**	**60.1**	**27.1**	**10.5**	**5.6**	**2.3**	**1.7**	**1.7**	**1.2**	**0.6**	**0.6**	**0.6**	**0.3**	**0.3**	**0.0**	**0.0**
**Lower CI**	84.7	48.0	18.8	6.1	2.8	1.1	0.7	0.7	0.4	0.2	0.2	0.2	0.1	0.1	0.0	0.0
**Upper CI**	97.6	71.0	37.3	17.3	10.9	4.8	4.1	4.1	3.8	2.3	2.3	2.3	1.7	1.7	3.6	3.6
**Design Effect**	1.29	1.25	1.10	0.94	0.88	0.61	0.63	0.63	0.69	0.58	0.58	0.58	0.56	0.56	-	-
**Number**	99	75	42	21	13	7	5	5	3	2	2	2	1	1	0	0

Table B2551-54 Age Group = 41-50 Gender = FEMALE Occupational group = MANUAL Total cases = 135

Threshold dB HL≥	0	5	10	15	20	25	30	35	40	45	50	55	65	75	85	95
**Prevalence**	**92.9**	**79.4**	**50.3**	**22.9**	**11.9**	**8.4**	**7.5**	**6.3**	**5.6**	**4.8**	**1.9**	**0.8**	**0.2**	**0.2**	**0.2**	**0.2**
**Lower CI**	85.2	70.5	41.5	16.3	7.9	5.5	4.7	3.7	3.1	2.5	1.2	0.3	0.0	0.0	0.0	0.0
**Upper CI**	96.8	86.2	59.1	31.2	17.5	12.7	11.8	10.6	9.8	9.0	3.0	1.8	1.0	1.0	1.0	1.0
**Design Effect**	1.29	1.16	1.06	1.06	0.88	0.76	0.78	0.82	0.84	0.87	0.38	0.45	0.48	0.48	0.48	0.48
**Number**	129	111	73	43	30	24	21	16	14	11	7	4	1	1	1	1

Table B2551-55 Age Group = 51-60 Gender = FEMALE Occupational group = MANUAL Total cases = 165

Threshold dB HL≥	0	5	10	15	20	25	30	35	40	45	50	55	65	75	85	95
**Prevalence**	**98.6**	**88.4**	**61.1**	**34.3**	**24.2**	**16.4**	**9.4**	**7.8**	**3.2**	**2.2**	**1.6**	**1.0**	**0.4**	**0.2**	**0.2**	**0.2**
**Lower CI**	92.5	80.8	51.7	26.7	17.7	11.2	6.4	4.9	1.7	1.2	0.8	0.5	0.1	0.0	0.0	0.0
**Upper CI**	99.8	93.3	69.8	42.9	32.2	23.4	13.8	12.4	5.7	4.0	3.3	1.8	1.2	1.0	1.0	1.0
**Design Effect**	1.52	1.27	1.23	1.13	1.11	1.08	0.83	0.90	0.71	0.62	0.62	0.39	0.47	0.53	0.53	0.53
**Number**	164	151	116	77	55	39	29	23	15	12	9	7	3	1	1	1

Table B2551-56 Age Group = 61-70 Gender = FEMALE Occupational group = MANUAL Total cases = 126

Threshold dB HL≥	0	5	10	15	20	25	30	35	40	45	50	55	65	75	85	95
**Prevalence**	**98.1**	**91.4**	**64.5**	**50.2**	**33.2**	**23.4**	**15.1**	**13.6**	**9.2**	**5.2**	**4.5**	**3.0**	**2.6**	**2.1**	**1.9**	**1.6**
**Lower CI**	90.1	82.0	53.6	40.1	25.5	17.2	12.9	11.9	5.5	3.8	3.1	2.2	1.9	1.6	1.4	0.5
**Upper CI**	99.7	96.2	74.0	60.3	41.9	30.9	17.5	15.6	15.0	7.0	6.4	4.2	3.5	2.8	2.4	5.7
**Design Effect**	1.55	1.41	1.25	1.18	1.01	0.93	0.37	0.31	0.93	0.41	0.46	0.34	0.28	0.25	0.21	0.00
**Number**	125	119	93	72	54	41	33	29	21	14	11	7	5	3	2	1

Prevalence by age group, gender and occupational group for the better ear over 0.25, 0.5, and 1 kHz

Table B2551-57 Age Group = 71-80 Gender = FEMALE Occupational group = MANUAL Total cases = 76

Threshold dB HL≥	0	5	10	15	20	25	30	35	40	45	50	55	65	75	85	95
**Prevalence**	**100.0**	**100.0**	**99.2**	**69.0**	**57.1**	**42.6**	**33.7**	**24.9**	**22.8**	**18.5**	**7.0**	**5.9**	**4.8**	**3.5**	**2.1**	**0.4**
**Lower CI**	95.2	95.2	96.9	57.0	41.9	30.6	22.2	15.5	13.7	9.8	4.5	3.5	2.7	1.7	0.8	0.1
**Upper CI**	100.0	100.0	99.8	78.8	71.1	55.6	47.4	37.4	35.4	32.1	10.9	9.7	8.4	7.0	5.8	2.1
**Design Effect**	-	-	0.59	1.08	1.37	1.16	1.23	1.15	1.18	1.29	0.55	0.58	0.57	0.60	0.70	0.53
**Number**	76	76	74	63	56	48	42	34	31	23	14	11	9	7	4	1

Table B2551-58 Age Group = 18-30 Gender = MALE Occupational group = MANUAL Total cases = 109

Threshold dB HL≥	0	5	10	15	20	25	30	35	40	45	50	55	65	75	85	95
**Prevalence**	**85.1**	**63.3**	**29.8**	**8.8**	**0.4**	**0.2**	**0.1**	**0.1**	**0.1**	**0.1**	**0.1**	**0.1**	**0.0**	**0.0**	**0.0**	**0.0**
**Lower CI**	73.3	50.8	19.6	3.7	0.1	0.1	0.0	0.0	0.0	0.0	0.0	0.0	0.0	0.0	0.0	0.0
**Upper CI**	92.2	74.3	42.5	19.5	1.2	0.9	0.4	0.4	0.4	0.4	0.4	0.4	3.4	3.4	3.4	3.4
**Design Effect**	1.41	1.34	1.37	1.45	0.39	0.39	0.27	0.27	0.27	0.27	0.27	0.27	-	-	-	-
**Number**	97	74	34	13	3	2	1	1	1	1	1	1	0	0	0	0

Table B2551-59 Age Group = 31-40 Gender = MALE Occupational group = MANUAL Total cases = 80

Threshold dB HL≥	0	5	10	15	20	25	30	35	40	45	50	55	65	75	85	95
**Prevalence**	**85.2**	**68.6**	**30.9**	**17.0**	**6.8**	**6.1**	**5.9**	**2.6**	**1.9**	**1.9**	**1.9**	**1.9**	**1.9**	**1.9**	**1.9**	**1.9**
**Lower CI**	71.5	54.5	18.9	8.8	2.4	1.9	1.8	0.6	0.3	0.3	0.3	0.3	0.3	0.3	0.3	0.3
**Upper CI**	93.0	80.0	46.2	30.4	18.0	17.6	17.6	10.2	10.2	10.2	10.2	10.2	10.2	10.2	10.2	10.2
**Design Effect**	1.39	1.30	1.40	1.33	1.33	1.38	1.40	1.14	1.25	1.25	1.25	1.25	1.25	1.25	1.25	1.25
**Number**	70	57	31	18	6	5	4	2	1	1	1	1	1	1	1	1

Table B2551-60 Age Group = 41-50 Gender = MALE Occupational group = MANUAL Total cases = 127

Threshold dB HL≥	0	5	10	15	20	25	30	35	40	45	50	55	65	75	85	95
**Prevalence**	**98.7**	**73.0**	**48.2**	**27.7**	**13.4**	**5.8**	**2.8**	**2.8**	**2.6**	**1.4**	**1.4**	**1.1**	**0.5**	**0.5**	**0.5**	**0.2**
**Lower CI**	95.7	62.1	37.6	19.6	8.7	3.4	1.4	1.4	1.2	0.7	0.7	0.5	0.1	0.1	0.1	0.0
**Upper CI**	99.6	81.7	58.9	37.4	20.0	9.8	5.5	5.5	5.3	3.0	3.0	2.5	1.7	1.7	1.7	1.3
**Design Effect**	0.85	1.30	1.25	1.16	0.96	0.77	0.68	0.68	0.70	0.53	0.53	0.51	0.54	0.54	0.54	0.54
**Number**	125	100	66	42	26	15	9	9	8	5	5	4	2	2	2	1

Table B2551-61 Age Group = 51-60 Gender = MALE Occupational group = MANUAL Total cases = 199

Threshold dB HL≥	0	5	10	15	20	25	30	35	40	45	50	55	65	75	85	95
**Prevalence**	**100.0**	**86.6**	**61.5**	**35.9**	**21.0**	**13.8**	**8.7**	**6.6**	**5.3**	**4.7**	**2.5**	**2.1**	**0.7**	**0.0**	**0.0**	**0.0**
**Lower CI**	98.1	79.2	53.2	29.0	15.8	9.7	6.2	4.6	3.4	2.8	1.3	1.0	0.2	0.0	0.0	0.0
**Upper CI**	100.0	91.7	69.1	43.5	27.3	19.3	12.2	9.5	8.1	7.8	4.7	4.2	2.2	1.9	1.9	1.9
**Design Effect**	0.00	1.31	1.20	1.10	1.02	1.00	0.76	0.71	0.76	0.84	0.74	0.75	0.74	-	-	-
**Number**	199	179	144	91	61	44	33	26	19	17	11	9	3	0	0	0

Table B2551-62 Age Group = 61-70 Gender = MALE Occupational group = MANUAL Total cases = 173

Threshold dB HL≥	0	5	10	15	20	25	30	35	40	45	50	55	65	75	85	95
**Prevalence**	**100.0**	**90.7**	**68.8**	**48.7**	**29.2**	**15.6**	**11.6**	**9.8**	**6.3**	**4.6**	**2.5**	**2.3**	**1.4**	**1.4**	**0.6**	**0.0**
**Lower CI**	97.8	81.5	57.9	38.6	23.3	11.6	7.9	6.3	3.8	2.5	1.4	1.3	0.7	0.7	0.2	0.0
**Upper CI**	100.0	95.6	78.0	58.8	36.0	20.7	16.8	15.0	10.0	8.4	4.4	4.3	2.8	2.8	1.5	2.2
**Design Effect**	-	1.60	1.48	1.39	0.94	0.84	0.94	0.97	0.84	0.92	0.62	0.64	0.59	0.59	0.51	-
**Number**	173	163	136	105	76	49	37	31	22	15	11	10	7	7	3	0

Table B2551-63 Age Group = 71-80 Gender = MALE Occupational group = MANUAL Total cases = 72

Threshold dB HL≥	0	5	10	15	20	25	30	35	40	45	50	55	65	75	85	95
**Prevalence**	**100.0**	**98.3**	**83.8**	**71.6**	**57.4**	**41.4**	**32.4**	**18.3**	**14.7**	**11.5**	**8.4**	**6.1**	**2.8**	**1.0**	**1.0**	**0.6**
**Lower CI**	94.9	97.3	64.3	54.2	43.1	33.0	26.0	12.4	9.6	7.2	4.9	3.6	1.2	0.3	0.3	0.1
**Upper CI**	100.0	99.0	93.7	84.3	70.5	50.2	39.5	26.2	21.9	17.9	14.0	10.1	6.3	3.5	3.5	3.1
**Design Effect**	-	0.27	1.76	1.51	1.25	0.77	0.63	0.78	0.76	0.72	0.70	0.57	0.63	0.60	0.60	0.63
**Number**	72	70	64	57	45	36	28	25	21	17	13	11	5	2	2	1

Prevalence by age group, gender and occupational group for the better ear over 1, 2, and 3 kHz

Table B123-1 Age Group = 18-80 Gender = OVERALL Occupational group = OVERALL Total cases = 2657

Threshold dB HL≥	0	5	10	15	20	25	30	35	40	45	50	55	65	75	85	95
**Prevalence**	**89.8**	**67.8**	**47.9**	**31.9**	**22.5**	**16.4**	**11.9**	**8.8**	**6.5**	**4.5**	**3.3**	**2.4**	**1.2**	**0.7**	**0.4**	**0.2**
Lower CI	88.0	65.7	45.9	30.3	21.1	15.3	10.9	7.9	5.8	4.0	2.9	2.0	1.0	0.5	0.2	0.1
Upper CI	91.3	69.8	49.9	33.6	23.9	17.6	12.9	9.7	7.2	5.0	3.8	2.8	1.5	1.0	0.7	0.5
Design Effect	1.45	1.17	1.04	0.92	0.89	0.83	0.81	0.80	0.73	0.68	0.67	0.66	0.69	0.79	0.93	1.06
Number	2500	2087	1621	1207	947	750	586	466	370	284	218	165	86	46	22	12

Table B123-2 Age Group = 18-80 Gender = FEMALE Occupational group = OVERALL Total cases = 1385

Threshold dB HL≥	0	5	10	15	20	25	30	35	40	45	50	55	65	75	85	95
**Prevalence**	**90.9**	**67.6**	**45.7**	**29.3**	**19.5**	**14.4**	**10.1**	**7.9**	**5.8**	**3.9**	**2.7**	**2.0**	**1.1**	**0.7**	**0.4**	**0.2**
Lower CI	88.8	64.8	43.2	27.2	17.7	12.9	8.9	6.8	5.0	3.3	2.3	1.6	0.8	0.5	0.2	0.1
Upper CI	92.7	70.3	48.2	31.4	21.4	16.0	11.5	9.2	6.7	4.6	3.3	2.5	1.5	1.0	0.7	0.4
Design Effect	1.30	1.11	0.96	0.87	0.89	0.86	0.83	0.85	0.68	0.64	0.57	0.60	0.58	0.64	0.67	0.66
Number	1300	1053	786	550	409	327	256	207	167	122	95	74	43	25	14	8

Table B123-3 Age Group = 18-80 Gender = MALE Occupational group = OVERALL Total cases = 1272

Threshold dB HL≥	0	5	10	15	20	25	30	35	40	45	50	55	65	75	85	95
**Prevalence**	**88.5**	**68.0**	**50.5**	**34.9**	**25.9**	**18.7**	**13.9**	**9.7**	**7.2**	**5.2**	**4.0**	**2.8**	**1.3**	**0.7**	**0.4**	**0.3**
Lower CI	85.5	64.8	47.3	32.4	23.8	17.0	12.5	8.6	6.2	4.4	3.2	2.2	0.9	0.4	0.1	0.1
Upper CI	91.0	71.1	53.6	37.5	28.1	20.5	15.5	11.0	8.3	6.1	4.8	3.5	1.9	1.3	1.0	1.0
Design Effect	1.57	1.24	1.13	0.96	0.90	0.81	0.80	0.75	0.75	0.71	0.75	0.71	0.78	0.93	1.16	1.32
Number	1200	1034	835	657	538	423	330	259	203	162	123	91	43	21	8	4

Table B123-4 Age Group = 18-30 Gender = OVERALL Occupational group = OVERALL Total cases = 401

Threshold dB HL≥	0	5	10	15	20	25	30	35	40	45	50	55	65	75	85	95
**Prevalence**	**78.3**	**37.9**	**19.5**	**6.7**	**3.7**	**1.8**	**0.8**	**0.5**	**0.5**	**0.1**	**0.0**	**0.0**	**0.0**	**0.0**	**0.0**	**0.0**
Lower CI	72.9	32.6	15.4	4.5	2.2	0.9	0.4	0.2	0.2	0.0	0.0	0.0	0.0	0.0	0.0	0.0
Upper CI	82.8	43.5	24.4	9.8	6.3	3.4	1.9	1.0	1.0	0.3	0.1	0.1	0.1	0.9	0.9	0.9
Design Effect	1.23	1.15	1.16	1.06	1.08	0.92	0.78	0.55	0.55	0.36	0.20	0.20	0.28	-	-	-
Number	324	177	95	39	22	14	9	7	7	3	2	2	1	0	0	0

Table B123-5 Age Group = 31-40 Gender = OVERALL Occupational group = OVERALL Total cases = 331

Threshold dB HL≥	0	5	10	15	20	25	30	35	40	45	50	55	65	75	85	95
**Prevalence**	**84.1**	**53.0**	**24.9**	**12.8**	**5.9**	**3.6**	**2.7**	**1.8**	**1.6**	**1.1**	**1.0**	**0.8**	**0.7**	**0.7**	**0.6**	**0.6**
Lower CI	78.5	46.4	19.8	9.3	3.8	2.1	1.5	0.8	0.7	0.4	0.4	0.2	0.1	0.1	0.1	0.1
Upper CI	88.4	59.5	30.9	17.5	9.1	6.3	4.8	3.9	3.8	3.1	3.1	3.0	3.0	3.0	3.1	3.1
Design Effect	1.26	1.22	1.19	1.15	1.04	1.01	0.93	1.01	1.05	1.07	1.09	1.16	1.27	1.27	1.36	1.36
Number	293	198	104	57	32	22	17	11	9	7	6	4	2	2	1	1

Table B123-6 Age Group = 41-50 Gender = OVERALL Occupational group = OVERALL Total cases = 450

Threshold dB HL≥	0	5	10	15	20	25	30	35	40	45	50	55	65	75	85	95
**Prevalence**	**92.9**	**72.0**	**45.6**	**23.9**	**14.1**	**8.1**	**6.7**	**4.4**	**2.4**	**1.8**	**1.2**	**0.8**	**0.5**	**0.3**	**0.1**	**0.1**
Lower CI	89.8	67.2	40.6	20.1	11.3	6.2	5.0	3.2	1.6	1.2	0.6	0.4	0.2	0.1	0.0	0.0
Upper CI	95.2	76.3	50.6	28.2	17.3	10.4	9.0	6.1	3.8	2.8	2.1	1.6	1.1	0.9	0.6	0.3
Design Effect	1.13	1.11	1.10	1.02	0.94	0.84	0.86	0.77	0.77	0.64	0.72	0.72	0.62	0.64	0.60	0.49
Number	424	337	234	139	94	63	53	38	22	19	12	8	6	4	2	1

Table B123-7 Age Group = 51-60 Gender = OVERALL Occupational group = OVERALL Total cases = 680

Threshold dB HL≥	0	5	10	15	20	25	30	35	40	45	50	55	65	75	85	95
**Prevalence**	**97.6**	**85.6**	**61.4**	**42.4**	**29.2**	**19.1**	**13.3**	**9.3**	**6.6**	**4.6**	**3.5**	**2.6**	**1.0**	**0.4**	**0.1**	**0.1**
Lower CI	95.8	82.3	57.3	38.5	26.0	16.4	11.1	7.5	5.2	3.4	2.5	1.8	0.6	0.2	0.0	0.0
Upper CI	98.6	88.3	65.4	46.4	32.7	22.1	15.8	11.3	8.3	6.0	4.8	3.8	1.6	1.0	0.3	0.3
Design Effect	1.18	1.14	1.11	1.06	0.98	0.96	0.91	0.87	0.84	0.81	0.84	0.82	0.69	0.75	0.50	0.50
Number	667	600	461	352	266	190	139	107	82	60	46	37	17	7	3	3

Prevalence by age group, gender and occupational group for the better ear over 1, 2, and 3 kHz

Table B123-8 Age Group = 61-70 Gender = OVERALL Occupational group = OVERALL Total cases = 524

	Threshold dB HL≥															
	0	5	10	15	20	25	30	35	40	45	50	55	65	75	85	95
**Prevalence**	**99.3**	**95.8**	**85.3**	**64.0**	**48.3**	**37.4**	**25.6**	**19.0**	**13.1**	**8.5**	**6.1**	**4.6**	**2.7**	**1.7**	**0.9**	**0.7**
**Lower CI**	97.4	93.0	81.0	58.7	43.4	33.1	21.9	16.2	10.9	7.1	5.0	3.7	1.9	1.1	0.4	0.3
**Upper CI**	99.8	97.5	88.8	69.0	53.2	42.0	29.6	22.3	15.5	10.0	7.4	5.9	3.8	2.8	1.7	1.5
**Design Effect**	1.41	1.28	1.30	1.25	1.15	1.08	1.03	0.91	0.79	0.60	0.59	0.61	0.69	0.74	0.74	0.78
**Number**	522	506	465	374	310	254	195	158	126	97	73	57	30	18	9	6

Table B123-9 Age Group = 71-80 Gender = OVERALL Occupational group = OVERALL Total cases = 271

	Threshold dB HL≥															
	0	5	10	15	20	25	30	35	40	45	50	55	65	75	85	95
**Prevalence**	**99.6**	**99.5**	**95.3**	**85.5**	**70.6**	**61.0**	**47.8**	**37.6**	**29.8**	**21.9**	**15.8**	**10.6**	**4.9**	**2.5**	**1.1**	**0.1**
**Lower CI**	98.0	98.0	89.6	78.7	62.4	53.7	41.0	31.2	24.8	17.7	12.8	8.3	3.5	1.5	0.5	0.0
**Upper CI**	99.9	99.9	98.0	90.4	77.7	67.9	54.7	44.5	35.4	26.7	19.5	13.3	6.7	4.0	2.4	0.8
**Design Effect**	0.98	0.89	1.57	1.40	1.43	1.24	1.16	1.16	0.98	0.92	0.77	0.68	0.61	0.66	0.70	0.63
**Number**	270	269	262	246	223	207	173	145	124	98	79	57	30	15	7	1

Table B123-10 Age Group = 18-80 Gender = OVERALL Occupational group = NON-MANUAL Total cases = 1181

	Threshold dB HL≥															
	0	5	10	15	20	25	30	35	40	45	50	55	65	75	85	95
**Prevalence**	**87.6**	**62.7**	**41.7**	**27.6**	**19.4**	**14.6**	**10.4**	**7.8**	**5.3**	**3.5**	**2.7**	**1.6**	**0.9**	**0.5**	**0.2**	**0.1**
**Lower CI**	84.7	59.5	38.9	25.6	17.6	13.3	9.4	7.0	4.6	3.0	2.2	1.3	0.6	0.3	0.1	0.0
**Upper CI**	90.0	65.7	44.5	29.7	21.3	16.1	11.5	8.7	6.2	4.0	3.2	2.1	1.3	0.9	0.4	0.3
**Design Effect**	1.41	1.13	0.99	0.80	0.82	0.69	0.60	0.56	0.62	0.51	0.53	0.58	0.61	0.67	0.59	0.58
**Number**	1095	859	630	457	342	273	204	163	129	90	70	52	30	17	7	5

Table B123-11 Age Group = 18-80 Gender = OVERALL Occupational group = MANUAL Total cases = 1472

	Threshold dB HL≥															
	0	5	10	15	20	25	30	35	40	45	50	55	65	75	85	95
**Prevalence**	**91.3**	**73.3**	**53.3**	**35.0**	**24.8**	**17.9**	**13.3**	**9.5**	**6.9**	**5.0**	**3.7**	**2.8**	**1.4**	**0.8**	**0.5**	**0.4**
**Lower CI**	88.8	70.1	50.2	32.5	22.7	16.1	11.9	8.4	6.0	4.3	3.1	2.3	1.0	0.6	0.3	0.2
**Upper CI**	93.3	76.3	56.3	37.6	27.1	19.7	14.8	10.6	7.9	5.9	4.3	3.5	1.8	1.3	1.0	0.8
**Design Effect**	1.56	1.36	1.22	1.04	0.99	0.92	0.82	0.73	0.73	0.70	0.62	0.66	0.66	0.76	0.85	0.94
**Number**	1402	1226	989	748	603	476	382	303	241	194	148	113	56	29	15	7

Table B123-12 Age Group = 18-30 Gender = FEMALE Occupational group = OVERALL Total cases = 224

	Threshold dB HL≥															
	0	5	10	15	20	25	30	35	40	45	50	55	65	75	85	95
**Prevalence**	**83.4**	**40.9**	**18.9**	**5.4**	**2.8**	**2.1**	**1.0**	**0.8**	**0.8**	**0.1**	**0.0**	**0.0**	**0.0**	**0.0**	**0.0**	**0.0**
**Lower CI**	77.1	33.9	14.1	3.3	1.5	1.0	0.4	0.3	0.3	0.0	0.0	0.0	0.0	0.0	0.0	0.0
**Upper CI**	88.3	48.2	24.8	8.9	5.2	4.0	2.1	1.8	1.8	0.6	1.7	1.7	1.7	1.7	1.7	1.7
**Design Effect**	1.16	1.13	1.05	0.92	0.82	0.76	0.60	0.59	0.59	0.47	-	-	-	-	-	-
**Number**	190	101	54	20	12	10	6	5	5	1	0	0	0	0	0	0

Table B123-13 Age Group = 31-40 Gender = FEMALE Occupational group = OVERALL Total cases = 189

	Threshold dB HL≥															
	0	5	10	15	20	25	30	35	40	45	50	55	65	75	85	95
**Prevalence**	**84.7**	**49.7**	**20.9**	**10.9**	**3.7**	**3.1**	**2.4**	**1.9**	**1.5**	**0.8**	**0.8**	**0.5**	**0.2**	**0.2**	**0.0**	**0.0**
**Lower CI**	77.5	41.3	15.5	7.3	2.1	1.7	1.2	0.8	0.5	0.3	0.3	0.1	0.0	0.0	0.0	0.0
**Upper CI**	89.9	58.0	27.6	16.1	6.4	5.7	5.0	4.4	4.1	2.2	2.2	1.7	1.2	1.2	2.0	2.0
**Design Effect**	1.20	1.18	1.05	0.98	0.78	0.78	0.83	0.86	0.92	0.67	0.67	0.66	0.63	0.63	-	-
**Number**	167	107	57	32	15	13	9	7	5	4	4	2	1	1	0	0

Table B123-14 Age Group = 41-50 Gender = FEMALE Occupational group = OVERALL Total cases = 247

	Threshold dB HL≥															
	0	5	10	15	20	25	30	35	40	45	50	55	65	75	85	95
**Prevalence**	**90.7**	**69.4**	**42.9**	**20.3**	**10.7**	**7.2**	**5.9**	**3.5**	**1.8**	**1.5**	**1.1**	**0.8**	**0.2**	**0.1**	**0.1**	**0.1**
**Lower CI**	85.6	62.8	36.5	15.7	7.7	5.0	3.8	2.2	0.9	0.7	0.5	0.3	0.1	0.0	0.0	0.0
**Upper CI**	94.1	75.4	49.6	25.9	14.7	10.4	9.0	5.5	3.5	3.1	2.6	2.3	0.9	0.6	0.6	0.6
**Design Effect**	1.16	1.11	1.07	1.02	0.91	0.83	0.87	0.70	0.76	0.74	0.74	0.80	0.55	0.51	0.51	0.51
**Number**	229	179	122	64	40	31	26	18	9	8	6	4	2	1	1	1

Prevalence by age group, gender and occupational group for the better ear over 1, 2, and 3 kHz

Table B123-15 Age Group = 51-60 Gender = FEMALE Occupational group = OVERALL Total cases = 340

Threshold dB HL≥	0	5	10	15	20	25	30	35	40	45	50	55	65	75	85	95
**Prevalence**	**96.7**	**82.3**	**55.4**	**35.3**	**21.1**	**12.6**	**8.5**	**5.3**	**3.9**	**2.2**	**1.9**	**1.5**	**1.0**	**0.6**	**0.2**	**0.2**
Lower CI	93.8	77.3	49.6	30.1	17.2	9.6	6.1	3.7	2.6	1.3	1.1	0.8	0.5	0.2	0.1	0.1
Upper CI	98.3	86.3	61.2	40.9	25.6	16.4	11.7	7.6	5.9	3.6	3.3	2.7	2.0	1.5	0.6	0.6
Design Effect	1.15	1.11	1.11	1.07	0.98	0.96	0.92	0.80	0.79	0.71	0.74	0.69	0.69	0.75	0.48	0.48
Number	331	293	213	153	105	71	51	38	29	18	16	13	9	5	3	3

Table B123-16 Age Group = 61-70 Gender = FEMALE Occupational group = OVERALL Total cases = 242

Threshold dB HL≥	0	5	10	15	20	25	30	35	40	45	50	55	65	75	85	95
**Prevalence**	**99.2**	**94.8**	**80.8**	**57.8**	**41.2**	**30.7**	**17.8**	**13.7**	**8.8**	**5.6**	**4.0**	**3.4**	**2.2**	**1.5**	**1.1**	**1.0**
Lower CI	95.4	90.1	74.0	50.6	34.8	25.3	13.7	10.1	6.6	4.3	2.8	2.3	1.3	0.8	0.5	0.4
Upper CI	99.9	97.3	86.2	64.7	47.8	36.7	22.9	18.2	11.5	7.4	5.7	4.9	3.7	3.1	2.6	2.5
Design Effect	1.42	1.25	1.24	1.14	1.06	0.99	0.95	0.93	0.69	0.53	0.57	0.56	0.65	0.70	0.74	0.76
Number	241	232	205	154	121	94	70	56	46	36	27	23	13	8	5	4

Table B123-17 Age Group = 71-80 Gender = FEMALE Occupational group = OVERALL Total cases = 143

Threshold dB HL≥	0	5	10	15	20	25	30	35	40	45	50	55	65	75	85	95
**Prevalence**	**99.4**	**99.2**	**93.0**	**83.2**	**67.8**	**56.3**	**45.1**	**39.5**	**30.7**	**21.9**	**14.1**	**10.2**	**4.8**	**3.0**	**1.5**	**0.0**
Lower CI	96.8	96.8	83.9	73.0	55.6	45.9	36.1	30.5	24.4	16.7	11.2	7.4	3.2	1.7	0.6	0.0
Upper CI	99.9	99.8	97.1	90.0	78.0	66.2	54.4	49.3	37.8	28.0	17.7	13.7	7.2	5.4	3.6	2.6
Design Effect	0.91	0.83	1.52	1.40	1.50	1.27	1.14	1.19	0.89	0.84	0.57	0.64	0.56	0.65	0.70	-
Number	142	141	135	127	116	108	94	83	73	55	42	32	18	10	5	0

Table B123-18 Age Group = 18-30 Gender = MALE Occupational group = OVERALL Total cases = 177

Threshold dB HL≥	0	5	10	15	20	25	30	35	40	45	50	55	65	75	85	95
**Prevalence**	**72.9**	**34.9**	**20.2**	**7.9**	**4.5**	**1.4**	**0.6**	**0.1**	**0.1**	**0.1**	**0.1**	**0.1**	**0.0**	**0.0**	**0.0**	**0.0**
Lower CI	64.0	27.2	13.9	4.5	2.1	0.4	0.1	0.0	0.0	0.0	0.0	0.0	0.0	0.0	0.0	0.0
Upper CI	80.3	43.4	28.3	13.6	9.5	4.7	3.0	0.2	0.2	0.2	0.2	0.2	0.2	2.1	2.1	2.1
Design Effect	1.26	1.17	1.23	1.11	1.16	1.06	0.94	0.19	0.19	0.19	0.19	0.19	0.27	-	-	-
Number	134	76	41	19	10	4	3	2	2	2	2	2	1	0	0	0

Table B123-19 Age Group = 31-40 Gender = MALE Occupational group = OVERALL Total cases = 142

Threshold dB HL≥	0	5	10	15	20	25	30	35	40	45	50	55	65	75	85	95
**Prevalence**	**83.2**	**56.5**	**29.2**	**15.0**	**8.3**	**4.1**	**2.9**	**1.7**	**1.7**	**1.4**	**1.3**	**1.3**	**1.2**	**1.2**	**1.2**	**1.2**
Lower CI	73.6	46.1	20.7	9.2	4.5	1.8	1.2	0.5	0.5	0.3	0.3	0.3	0.2	0.2	0.2	0.2
Upper CI	89.7	66.3	39.6	23.7	14.8	9.5	7.1	6.3	6.3	6.2	6.4	6.4	6.5	6.5	6.5	6.5
Design Effect	1.32	1.27	1.29	1.24	1.10	1.10	0.99	1.14	1.14	1.22	1.26	1.26	1.31	1.31	1.31	1.31
Number	126	91	47	25	17	9	8	4	4	3	2	2	1	1	1	1

Table B123-20 Age Group = 41-50 Gender = MALE Occupational group = OVERALL Total cases = 203

Threshold dB HL≥	0	5	10	15	20	25	30	35	40	45	50	55	65	75	85	95
**Prevalence**	**95.4**	**74.8**	**48.5**	**27.8**	**17.8**	**9.0**	**7.7**	**5.5**	**3.2**	**2.1**	**1.2**	**0.7**	**0.7**	**0.6**	**0.2**	**0.0**
Lower CI	91.0	67.6	40.9	21.9	13.3	6.2	5.1	3.4	1.8	1.3	0.5	0.3	0.3	0.2	0.0	0.0
Upper CI	97.7	80.8	56.1	34.5	23.3	12.9	11.4	8.6	5.6	3.5	2.8	1.9	1.9	1.8	1.1	1.9
Design Effect	1.11	1.12	1.12	1.03	0.95	0.85	0.85	0.81	0.76	0.56	0.70	0.63	0.63	0.65	0.63	-
Number	195	158	112	75	54	32	27	20	13	11	6	4	4	3	1	0

Table B123-21 Age Group = 51-60 Gender = MALE Occupational group = OVERALL Total cases = 340

Threshold dB HL≥	0	5	10	15	20	25	30	35	40	45	50	55	65	75	85	95
**Prevalence**	**98.6**	**89.3**	**68.0**	**50.3**	**38.4**	**26.4**	**18.6**	**13.6**	**9.6**	**7.2**	**5.2**	**4.0**	**1.0**	**0.3**	**0.0**	**0.0**
Lower CI	96.2	84.8	62.2	44.6	33.2	22.0	15.1	10.6	7.2	5.2	3.4	2.5	0.5	0.1	0.0	0.0
Upper CI	99.5	92.6	73.4	56.1	43.7	31.3	22.7	17.4	12.7	10.0	7.8	6.3	2.0	1.1	1.1	1.1
Design Effect	1.16	1.18	1.13	1.09	1.02	1.00	0.93	0.93	0.88	0.87	0.91	0.90	0.69	0.76	-	-
Number	336	307	248	199	161	119	88	69	53	42	30	24	8	2	0	0

Prevalence by age group, gender and occupational group for the better ear over 1, 2, and 3 kHz

Table B123-22 Age Group = 61-70 Gender = MALE Occupational group = OVERALL Total cases = 282

Threshold dB HL≥	0	5	10	15	20	25	30	35	40	45	50	55	65	75	85	95
**Prevalence**	**99.5**	**97.1**	**90.5**	**71.2**	**56.6**	**45.3**	**34.6**	**25.2**	**18.1**	**11.8**	**8.5**	**6.1**	**3.4**	**2.0**	**0.6**	**0.3**
**Lower CI**	97.5	93.6	84.8	63.3	49.0	38.4	28.5	20.9	14.5	9.5	6.7	4.5	2.1	1.0	0.2	0.1
**Upper CI**	99.9	98.7	94.2	78.0	63.8	52.3	41.1	30.1	22.5	14.6	10.8	8.3	5.3	3.7	1.6	1.2
**Design Effect**	1.14	1.20	1.37	1.41	1.29	1.20	1.14	0.90	0.90	0.67	0.63	0.68	0.74	0.80	0.65	0.69
**Number**	281	274	260	220	189	160	125	102	80	61	46	34	17	10	4	2

Table B123-23 Age Group = 71-80 Gender = MALE Occupational group = OVERALL Total cases = 128

Threshold dB HL≥	0	5	10	15	20	25	30	35	40	45	50	55	65	75	85	95
**Prevalence**	**100.0**	**100.0**	**98.7**	**88.5**	**73.9**	**67.1**	**51.3**	**34.5**	**28.1**	**22.0**	**18.7**	**11.4**	**5.1**	**1.8**	**0.7**	**0.4**
**Lower CI**	97.1	97.1	92.7	79.7	64.4	57.7	41.6	26.8	20.9	15.9	13.0	7.8	3.0	0.8	0.2	0.1
**Upper CI**	100.0	100.0	99.8	93.8	81.6	75.4	60.9	43.1	36.6	29.7	26.2	16.2	8.7	4.1	2.4	2.0
**Design Effect**	-	-	1.32	1.26	1.15	1.11	1.13	1.01	1.02	0.97	0.98	0.76	0.73	0.67	0.66	0.67
**Number**	128	128	127	119	107	99	79	62	51	43	37	25	12	5	2	1

Table B123-24 Age Group = 18-30 Gender = OVERALL Occupational group = NON-MANUAL Total cases = 181

Threshold dB HL≥	0	5	10	15	20	25	30	35	40	45	50	55	65	75	85	95
**Prevalence**	**70.4**	**29.8**	**12.6**	**2.2**	**1.2**	**1.0**	**0.5**	**0.5**	**0.5**	**0.1**	**0.1**	**0.1**	**0.1**	**0.0**	**0.0**	**0.0**
**Lower CI**	61.9	22.7	7.9	1.1	0.5	0.3	0.1	0.1	0.1	0.0	0.0	0.0	0.0	0.0	0.0	0.0
**Upper CI**	77.6	38.0	19.4	4.1	3.2	3.0	2.4	2.4	2.4	2.2	2.2	2.2	2.2	2.1	2.1	2.1
**Design Effect**	1.20	1.16	1.19	0.67	0.77	0.79	0.85	0.85	0.85	0.00	0.00	0.00	0.00	-	-	-
**Number**	137	60	29	8	5	4	2	2	2	1	1	1	1	0	0	0

Table B123-25 Age Group = 31-40 Gender = OVERALL Occupational group = NON-MANUAL Total cases = 148

Threshold dB HL≥	0	5	10	15	20	25	30	35	40	45	50	55	65	75	85	95
**Prevalence**	**85.3**	**45.0**	**19.3**	**8.7**	**5.0**	**2.0**	**1.8**	**0.4**	**0.3**	**0.3**	**0.3**	**0.0**	**0.0**	**0.0**	**0.0**	**0.0**
**Lower CI**	76.4	35.4	12.6	4.9	2.3	0.9	0.8	0.1	0.1	0.1	0.1	0.0	0.0	0.0	0.0	0.0
**Upper CI**	91.2	55.0	28.4	15.0	10.3	4.2	4.0	1.6	1.7	1.7	1.7	2.5	2.5	2.5	2.5	2.5
**Design Effect**	1.29	1.25	1.25	1.09	1.10	0.68	0.69	0.59	0.67	0.67	0.67	-	-	-	-	-
**Number**	131	73	32	16	10	6	5	2	1	1	1	0	0	0	0	0

Table B123-26 Age Group = 41-50 Gender = OVERALL Occupational group = NON-MANUAL Total cases = 188

Threshold dB HL≥	0	5	10	15	20	25	30	35	40	45	50	55	65	75	85	95
**Prevalence**	**91.2**	**66.9**	**36.6**	**16.8**	**10.0**	**5.4**	**4.7**	**2.2**	**1.0**	**1.0**	**0.4**	**0.4**	**0.4**	**0.2**	**0.0**	**0.0**
**Lower CI**	85.4	58.9	29.3	12.0	6.4	3.3	2.7	1.6	0.6	0.6	0.1	0.1	0.1	0.0	0.0	0.0
**Upper CI**	94.8	74.1	44.5	23.0	15.2	8.7	8.0	3.1	1.7	1.7	1.3	1.3	1.3	1.3	2.0	2.0
**Design Effect**	1.15	1.14	1.11	1.04	1.01	0.82	0.87	0.33	0.36	0.36	0.60	0.60	0.60	0.66	-	-
**Number**	172	128	78	39	23	16	13	7	3	3	2	2	2	1	0	0

Table B123-27 Age Group = 51-60 Gender = OVERALL Occupational group = NON-MANUAL Total cases = 317

Threshold dB HL≥	0	5	10	15	20	25	30	35	40	45	50	55	65	75	85	95
**Prevalence**	**98.1**	**82.8**	**54.0**	**37.1**	**23.7**	**14.8**	**9.6**	**5.3**	**4.2**	**2.4**	**1.7**	**1.3**	**0.7**	**0.5**	**0.2**	**0.2**
**Lower CI**	95.9	77.8	48.0	31.8	19.5	12.0	7.9	3.8	3.0	1.6	1.0	0.7	0.3	0.2	0.0	0.0
**Upper CI**	99.1	86.9	59.9	42.7	28.4	18.1	11.7	7.2	5.8	3.7	2.9	2.5	1.6	1.4	0.7	0.7
**Design Effect**	0.99	1.09	1.09	1.03	0.95	0.78	0.57	0.69	0.64	0.59	0.65	0.68	0.68	0.68	0.54	0.54
**Number**	310	263	184	135	93	64	45	35	28	19	13	10	5	4	2	2

Table B123-28 Age Group = 61-70 Gender = OVERALL Occupational group = NON-MANUAL Total cases = 225

Threshold dB HL≥	0	5	10	15	20	25	30	35	40	45	50	55	65	75	85	95
**Prevalence**	**99.2**	**95.0**	**80.9**	**62.2**	**45.3**	**37.3**	**22.5**	**19.3**	**11.8**	**6.9**	**5.1**	**4.3**	**2.2**	**1.6**	**0.6**	**0.5**
**Lower CI**	95.4	90.3	72.3	53.4	37.7	30.4	19.0	16.4	9.2	5.1	3.6	2.6	1.1	0.7	0.2	0.1
**Upper CI**	99.9	97.4	87.3	70.4	53.1	44.9	26.4	22.7	15.0	9.5	7.3	7.0	4.2	3.6	1.8	1.6
**Design Effect**	1.37	1.20	1.47	1.36	1.19	1.16	0.68	0.62	0.69	0.66	0.64	0.81	0.77	0.84	0.68	0.70
**Number**	224	214	189	150	117	97	68	58	44	30	24	20	11	7	4	3

Prevalence by age group, gender and occupational group for the better ear over 1, 2, and 3 kHz

Table B123-29 Age Group = 71-80 Gender = OVERALL Occupational group = NON-MANUAL Total cases = 122

Threshold dB HL≥	0	5	10	15	20	25	30	35	40	45	50	55	65	75	85	95
**Prevalence**	**99.1**	**99.1**	**96.0**	**86.4**	**69.5**	**60.8**	**49.2**	**40.3**	**28.7**	**19.9**	**16.4**	**7.9**	**4.1**	**2.3**	**0.4**	**0.0**
Lower CI	95.1	95.1	90.1	77.9	58.1	52.5	41.5	33.2	22.4	16.1	13.0	5.7	2.3	0.9	0.1	0.0
Upper CI	99.8	99.8	98.4	92.0	78.9	68.4	56.9	47.7	35.9	24.5	20.6	11.0	7.3	5.4	2.0	3.1
Design Effect	1.05	1.05	1.12	1.15	1.30	0.93	0.88	0.84	0.85	0.59	0.58	0.55	0.69	0.78	0.66	-
Number	121	121	118	109	94	86	71	59	51	36	29	19	11	5	1	0

Table B123-30 Age Group = 18-30 Gender = OVERALL Occupational group = MANUAL Total cases = 218

Threshold dB HL≥	0	5	10	15	20	25	30	35	40	45	50	55	65	75	85	95
**Prevalence**	**83.4**	**47.2**	**26.9**	**10.2**	**5.4**	**2.6**	**1.1**	**0.5**	**0.5**	**0.1**	**0.0**	**0.0**	**0.0**	**0.0**	**0.0**	**0.0**
Lower CI	75.6	38.5	19.8	6.5	3.0	1.1	0.4	0.2	0.2	0.0	0.0	0.0	0.0	0.0	0.0	0.0
Upper CI	89.1	55.9	35.3	15.7	9.8	6.0	2.8	1.1	1.1	0.6	0.2	0.2	1.7	1.7	1.7	1.7
Design Effect	1.37	1.33	1.33	1.13	1.11	1.07	0.78	0.48	0.48	0.43	0.26	0.26	-	-	-	-
Number	186	117	66	31	17	10	7	5	5	2	1	1	0	0	0	0

Table B123-31 Age Group = 31-40 Gender = OVERALL Occupational group = MANUAL Total cases = 183

Threshold dB HL≥	0	5	10	15	20	25	30	35	40	45	50	55	65	75	85	95
**Prevalence**	**83.9**	**61.9**	**29.8**	**16.6**	**6.8**	**5.4**	**3.5**	**2.9**	**2.6**	**1.5**	**1.4**	**1.4**	**1.1**	**1.1**	**0.9**	**0.9**
Lower CI	76.3	52.9	22.1	10.9	3.7	2.6	1.6	1.2	1.0	0.5	0.4	0.4	0.2	0.2	0.2	0.2
Upper CI	89.4	70.0	38.7	24.6	12.2	11.0	7.3	6.8	6.5	4.8	4.8	4.8	4.9	4.9	5.0	5.0
Design Effect	1.23	1.23	1.27	1.27	1.14	1.23	1.02	1.07	1.10	1.05	1.08	1.11	1.22	1.22	1.30	1.30
Number	162	125	72	41	22	16	12	9	8	6	5	4	2	2	1	1

Table B123-32 Age Group = 41-50 Gender = OVERALL Occupational group = MANUAL Total cases = 262

Threshold dB HL≥	0	5	10	15	20	25	30	35	40	45	50	55	65	75	85	95
**Prevalence**	**94.1**	**77.4**	**53.3**	**29.6**	**18.9**	**10.5**	**8.8**	**5.6**	**3.1**	**2.2**	**1.4**	**0.9**	**0.4**	**0.3**	**0.2**	**0.1**
Lower CI	89.3	70.8	46.1	23.8	14.3	7.4	6.0	3.9	1.9	1.3	0.7	0.4	0.2	0.1	0.1	0.0
Upper CI	96.8	82.9	60.5	36.2	24.7	14.6	12.5	7.9	5.0	3.7	2.9	2.3	1.1	0.9	0.7	0.5
Design Effect	1.28	1.20	1.20	1.14	1.10	0.97	0.94	0.71	0.73	0.64	0.70	0.76	0.52	0.52	0.52	0.48
Number	252	209	156	100	71	47	40	31	19	16	10	6	4	3	2	1

Table B123-33 Age Group = 51-60 Gender = OVERALL Occupational group = MANUAL Total cases = 363

Threshold dB HL≥	0	5	10	15	20	25	30	35	40	45	50	55	65	75	85	95
**Prevalence**	**97.6**	**89.7**	**67.9**	**46.5**	**33.6**	**23.5**	**17.1**	**11.3**	**7.6**	**5.5**	**4.4**	**3.3**	**1.1**	**0.3**	**0.1**	**0.1**
Lower CI	94.9	85.0	61.9	40.8	28.8	19.3	13.6	8.7	5.6	3.9	2.9	2.1	0.6	0.1	0.0	0.0
Upper CI	98.9	93.0	73.4	52.4	38.8	28.3	21.3	14.7	10.2	7.7	6.4	5.2	1.9	0.8	0.5	0.5
Design Effect	1.19	1.27	1.20	1.14	1.04	1.03	1.00	0.91	0.84	0.81	0.83	0.82	0.60	0.61	0.56	0.56
Number	357	337	277	217	173	126	94	72	54	41	33	27	12	3	1	1

Table B123-34 Age Group = 61-70 Gender = OVERALL Occupational group = MANUAL Total cases = 298

Threshold dB HL≥	0	5	10	15	20	25	30	35	40	45	50	55	65	75	85	95
**Prevalence**	**99.4**	**96.1**	**88.6**	**64.0**	**50.3**	**37.4**	**28.3**	**19.3**	**14.2**	**10.0**	**7.0**	**5.4**	**3.2**	**2.1**	**1.3**	**1.1**
Lower CI	96.9	91.4	83.1	56.6	43.3	31.4	22.8	15.2	11.1	8.1	5.6	4.2	2.3	1.4	0.9	0.8
Upper CI	99.9	98.2	92.4	70.7	57.4	43.7	34.4	24.2	17.9	12.2	8.7	6.9	4.3	3.0	1.9	1.6
Design Effect	1.29	1.46	1.28	1.30	1.26	1.13	1.14	1.01	0.85	0.60	0.53	0.52	0.50	0.47	0.37	0.32
Number	297	291	275	223	192	157	127	100	82	67	49	37	19	11	5	3

Table B123-35 Age Group = 71-80 Gender = OVERALL Occupational group = MANUAL Total cases = 148

Threshold dB HL≥	0	5	10	15	20	25	30	35	40	45	50	55	65	75	85	95
**Prevalence**	**100.0**	**99.7**	**94.7**	**85.9**	**70.7**	**59.7**	**47.9**	**38.6**	**29.1**	**23.0**	**15.3**	**12.2**	**5.4**	**2.8**	**1.7**	**0.2**
Lower CI	97.5	98.5	84.3	75.5	57.9	49.4	39.6	31.8	22.5	16.5	12.0	9.0	3.5	1.5	0.7	0.0
Upper CI	100.0	100.0	98.3	92.3	80.9	69.3	56.4	46.0	36.6	30.9	19.4	16.3	8.2	5.1	3.8	1.3
Design Effect	-	0.62	1.78	1.50	1.61	1.29	1.06	0.92	0.97	1.07	0.63	0.70	0.64	0.65	0.70	0.59
Number	148	147	143	136	128	120	102	86	73	62	50	38	19	10	6	1

Prevalence by age group, gender and occupational group for the better ear over 1, 2, and 3 kHz

Table B123-36 Age Group = 18-80 Gender = FEMALE Occupational group = NON-MANUAL Total cases = 670

	Threshold dB HL≥															
	0	5	10	15	20	25	30	35	40	45	50	55	65	75	85	95
**Prevalence**	**90.6**	**63.6**	**41.8**	**27.1**	**17.6**	**13.9**	**9.7**	**8.5**	**5.3**	**3.5**	**2.7**	**1.5**	**1.1**	**0.7**	**0.3**	**0.2**
**Lower CI**	87.5	59.8	38.7	24.6	15.3	12.1	8.5	7.4	4.3	2.9	2.2	1.1	0.7	0.4	0.1	0.1
**Upper CI**	92.9	67.2	45.0	29.8	20.2	15.8	11.0	9.8	6.4	4.1	3.3	2.1	1.7	1.3	0.6	0.5
**Design Effect**	1.22	1.01	0.85	0.77	0.84	0.71	0.58	0.57	0.61	0.43	0.44	0.54	0.61	0.67	0.62	0.60
**Number**	624	477	347	239	173	142	108	92	74	51	40	30	21	13	6	5

Table B123-37 Age Group = 18-80 Gender = MALE Occupational group = NON-MANUAL Total cases = 511

	Threshold dB HL≥															
	0	5	10	15	20	25	30	35	40	45	50	55	65	75	85	95
**Prevalence**	**84.1**	**61.6**	**41.5**	**28.2**	**21.3**	**15.4**	**11.2**	**7.1**	**5.4**	**3.5**	**2.7**	**1.7**	**0.7**	**0.4**	**0.1**	**0.0**
**Lower CI**	78.8	56.4	36.8	25.1	18.6	13.5	9.6	5.9	4.3	2.7	1.9	1.2	0.3	0.1	0.0	0.0
**Upper CI**	88.3	66.6	46.3	31.5	24.3	17.6	12.9	8.4	6.7	4.5	3.6	2.6	1.3	1.0	0.3	0.7
**Design Effect**	1.49	1.22	1.11	0.82	0.81	0.67	0.60	0.54	0.61	0.57	0.59	0.61	0.65	0.72	0.54	-
**Number**	471	382	283	218	169	131	96	71	55	39	30	22	9	4	1	0

Table B123-38 Age Group = 18-80 Gender = FEMALE Occupational group = MANUAL Total cases = 714

	Threshold dB HL≥															
	0	5	10	15	20	25	30	35	40	45	50	55	65	75	85	95
**Prevalence**	**91.2**	**72.7**	**49.7**	**30.9**	**20.9**	**14.9**	**11.0**	**8.0**	**5.8**	**4.2**	**2.9**	**2.3**	**1.1**	**0.8**	**0.6**	**0.3**
**Lower CI**	87.7	68.1	45.6	27.7	18.1	12.7	9.4	6.7	4.7	3.2	2.3	1.8	0.8	0.5	0.4	0.2
**Upper CI**	93.8	76.8	53.9	34.2	23.9	17.3	12.9	9.5	7.2	5.4	3.6	2.9	1.5	1.1	0.9	0.4
**Design Effect**	1.46	1.33	1.13	0.97	0.96	0.87	0.77	0.71	0.71	0.72	0.50	0.51	0.43	0.48	0.47	0.18
**Number**	675	575	438	310	235	185	148	115	93	71	55	44	22	12	8	3

Table B123-39 Age Group = 18-80 Gender = MALE Occupational group = MANUAL Total cases = 758

	Threshold dB HL≥															
	0	5	10	15	20	25	30	35	40	45	50	55	65	75	85	95
**Prevalence**	**91.5**	**74.1**	**57.2**	**39.6**	**29.3**	**21.2**	**15.9**	**11.1**	**8.0**	**6.0**	**4.5**	**3.4**	**1.7**	**0.9**	**0.5**	**0.4**
**Lower CI**	87.6	69.6	52.5	35.8	26.1	18.5	13.7	9.6	6.7	5.0	3.6	2.6	1.1	0.5	0.2	0.1
**Upper CI**	94.3	78.2	61.7	43.6	32.7	24.1	18.2	12.9	9.6	7.3	5.7	4.6	2.6	1.9	1.6	1.6
**Design Effect**	1.67	1.39	1.32	1.13	1.02	0.97	0.87	0.75	0.74	0.66	0.71	0.77	0.81	0.97	1.19	1.29
**Number**	727	651	551	438	368	291	234	188	148	123	93	69	34	17	7	4

Table B123-40 Age Group = 18-30 Gender = FEMALE Occupational group = NON-MANUAL Total cases = 115

	Threshold dB HL≥															
	0	5	10	15	20	25	30	35	40	45	50	55	65	75	85	95
**Prevalence**	**82.2**	**33.1**	**13.5**	**4.0**	**2.2**	**1.8**	**0.8**	**0.8**	**0.8**	**0.0**	**0.0**	**0.0**	**0.0**	**0.0**	**0.0**	**0.0**
**Lower CI**	73.4	24.8	8.6	2.1	0.8	0.6	0.1	0.1	0.1	0.0	0.0	0.0	0.0	0.0	0.0	0.0
**Upper CI**	88.5	42.7	20.5	7.7	5.9	5.5	4.5	4.5	4.5	3.2	3.2	3.2	3.2	3.2	3.2	3.2
**Design Effect**	1.08	1.06	0.95	0.76	0.86	0.90	0.98	0.98	0.98	-	-	-	-	-	-	-
**Number**	95	40	20	7	4	3	1	1	1	0	0	0	0	0	0	0

Table B123-41 Age Group = 31-40 Gender = FEMALE Occupational group = NON-MANUAL Total cases = 86

	Threshold dB HL≥															
	0	5	10	15	20	25	30	35	40	45	50	55	65	75	85	95
**Prevalence**	**82.9**	**43.4**	**14.5**	**7.2**	**2.5**	**2.5**	**2.1**	**0.7**	**0.6**	**0.6**	**0.6**	**0.0**	**0.0**	**0.0**	**0.0**	**0.0**
**Lower CI**	72.5	32.5	8.4	3.5	1.3	1.3	1.1	0.2	0.1	0.1	0.1	0.0	0.0	0.0	0.0	0.0
**Upper CI**	89.9	54.9	24.0	14.4	4.7	4.7	4.2	3.0	3.2	3.2	3.2	4.3	4.3	4.3	4.3	4.3
**Design Effect**	1.10	1.10	1.05	0.96	0.50	0.50	0.49	0.62	0.71	0.71	0.71	-	-	-	-	-
**Number**	74	40	16	9	5	5	4	2	1	1	1	0	0	0	0	0

Table B123-42 Age Group = 41-50 Gender = FEMALE Occupational group = NON-MANUAL Total cases = 112

	Threshold dB HL≥															
	0	5	10	15	20	25	30	35	40	45	50	55	65	75	85	95
**Prevalence**	**91.8**	**66.7**	**39.4**	**12.4**	**6.3**	**4.4**	**3.3**	**2.3**	**0.0**	**0.0**	**0.0**	**0.0**	**0.0**	**0.0**	**0.0**	**0.0**
**Lower CI**	84.3	56.7	31.1	8.5	4.3	3.0	2.1	1.6	0.0	0.0	0.0	0.0	0.0	0.0	0.0	0.0
**Upper CI**	95.9	75.3	48.5	17.8	9.3	6.4	5.1	3.5	3.3	3.3	3.3	3.3	3.3	3.3	3.3	3.3
**Design Effect**	1.10	1.08	0.98	0.76	0.55	0.44	0.45	0.33	-	-	-	-	-	-	-	-
**Number**	103	76	49	20	11	9	7	4	0	0	0	0	0	0	0	0

Prevalence by age group, gender and occupational group for the better ear over 1, 2, and 3 kHz

Table B123-43 Age Group = 51-60 Gender = FEMALE Occupational group = NON-MANUAL Total cases = 175

Threshold dB HL≥	0	5	10	15	20	25	30	35	40	45	50	55	65	75	85	95
**Prevalence**	**98.7**	**81.1**	**53.5**	**33.2**	**18.8**	**9.7**	**6.1**	**5.0**	**4.4**	**2.5**	**2.1**	**1.7**	**1.2**	**1.0**	**0.3**	**0.3**
**Lower CI**	95.8	74.2	45.5	26.3	13.9	6.5	4.0	3.2	2.7	1.5	1.0	0.7	0.5	0.4	0.1	0.1
**Upper CI**	99.6	86.6	61.4	41.0	24.9	14.2	9.0	7.8	7.1	4.4	4.1	3.7	3.0	2.6	1.2	1.2
**Design Effect**	0.96	1.08	1.10	1.06	0.95	0.87	0.70	0.70	0.72	0.60	0.68	0.73	0.70	0.70	0.55	0.55
**Number**	172	145	103	71	47	30	22	18	15	10	8	6	5	4	2	2

Table B123-44 Age Group = 61-70 Gender = FEMALE Occupational group = NON-MANUAL Total cases = 115

Threshold dB HL≥	0	5	10	15	20	25	30	35	40	45	50	55	65	75	85	95
**Prevalence**	**98.5**	**94.5**	**78.5**	**60.3**	**39.5**	**32.7**	**17.5**	**16.0**	**8.9**	**4.8**	**3.4**	**2.9**	**2.0**	**1.6**	**0.8**	**0.8**
**Lower CI**	92.0	87.0	68.1	49.7	31.1	24.9	13.1	11.8	5.9	3.1	2.1	1.6	0.9	0.7	0.2	0.2
**Upper CI**	99.7	97.8	86.3	70.1	48.5	41.7	23.0	21.4	13.0	7.4	5.4	5.0	4.2	3.8	3.0	3.0
**Design Effect**	1.32	1.22	1.23	1.16	0.99	1.00	0.71	0.72	0.68	0.54	0.49	0.54	0.60	0.63	0.70	0.70
**Number**	114	110	95	73	54	46	31	28	23	16	13	10	7	5	3	3

Table B123-45 Age Group = 71-80 Gender = FEMALE Occupational group = NON-MANUAL Total cases = 67

Threshold dB HL≥	0	5	10	15	20	25	30	35	40	45	50	55	65	75	85	95
**Prevalence**	**98.6**	**98.6**	**94.6**	**84.8**	**68.0**	**60.2**	**50.3**	**45.8**	**28.6**	**21.1**	**16.0**	**7.8**	**5.3**	**3.1**	**0.6**	**0.0**
**Lower CI**	92.2	92.2	85.0	72.3	50.4	48.5	41.0	37.2	21.3	17.0	12.7	4.9	2.8	1.1	0.1	0.0
**Upper CI**	99.7	99.7	98.2	92.3	81.6	70.8	59.5	54.6	37.2	25.8	20.0	12.2	9.9	8.0	3.4	5.4
**Design Effect**	0.99	0.99	1.12	1.17	1.47	0.98	0.79	0.74	0.74	0.45	0.42	0.56	0.64	0.76	0.64	
**Number**	66	66	64	59	52	49	43	39	34	24	18	14	9	4	1	0

Table B123-46 Age Group = 18-30 Gender = MALE Occupational group = NON-MANUAL Total cases = 66

Threshold dB HL≥	0	5	10	15	20	25	30	35	40	45	50	55	65	75	85	95
**Prevalence**	**57.7**	**26.2**	**11.5**	**0.1**	**0.1**	**0.1**	**0.1**	**0.1**	**0.1**	**0.1**	**0.1**	**0.1**	**0.1**	**0.0**	**0.0**	**0.0**
**Lower CI**	43.4	15.8	4.8	0.0	0.0	0.0	0.0	0.0	0.0	0.0	0.0	0.0	0.0	0.0	0.0	0.0
**Upper CI**	70.8	40.2	25.0	5.7	5.7	5.7	5.7	5.7	5.7	5.7	5.7	5.7	5.7	5.5	5.5	5.5
**Design Effect**	1.19	1.18	1.30	0.00	0.00	0.00	0.00	0.00	0.00	0.00	0.00	0.00	0.00	-	-	-
**Number**	42	20	9	1	1	1	1	1	1	1	1	1	1	0	0	0

Table B123-47 Age Group = 31-40 Gender = MALE Occupational group = NON-MANUAL Total cases = 62

Threshold dB HL≥	0	5	10	15	20	25	30	35	40	45	50	55	65	75	85	95
**Prevalence**	**87.4**	**46.5**	**24.6**	**10.4**	**7.7**	**1.3**	**1.3**	**0.0**	**0.0**	**0.0**	**0.0**	**0.0**	**0.0**	**0.0**	**0.0**	**0.0**
**Lower CI**	70.1	30.8	13.2	4.5	2.8	0.2	0.2	0.0	0.0	0.0	0.0	0.0	0.0	0.0	0.0	0.0
**Upper CI**	95.4	63.0	41.3	22.3	19.6	6.9	6.9	5.8	5.8	5.8	5.8	5.8	5.8	5.8	5.8	5.8
**Design Effect**	1.52	1.37	1.36	1.14	1.20	0.89	0.89	-	-	-	-	-	-	-	-	-
**Number**	57	33	16	7	5	1	1	0	0	0	0	0	0	0	0	0

Table B123-48 Age Group = 41-50 Gender = MALE Occupational group = NON-MANUAL Total cases = 76

Threshold dB HL≥	0	5	10	15	20	25	30	35	40	45	50	55	65	75	85	95
**Prevalence**	**90.4**	**67.2**	**33.5**	**21.6**	**14.1**	**6.5**	**6.2**	**2.1**	**2.1**	**2.1**	**0.7**	**0.7**	**0.7**	**0.5**	**0.0**	**0.0**
**Lower CI**	79.9	54.1	22.2	13.0	7.4	2.9	2.7	1.3	1.3	1.3	0.2	0.2	0.2	0.1	0.0	0.0
**Upper CI**	95.7	78.1	47.2	33.6	25.1	13.9	13.7	3.5	3.5	3.5	2.8	2.8	2.8	2.7	4.8	4.8
**Design Effect**	1.17	1.17	1.22	1.14	1.13	0.95	0.96	0.33	0.33	0.33	0.56	0.56	0.56	0.61	-	-
**Number**	69	52	29	19	12	7	6	3	3	3	2	2	2	1	0	0

Table B123-49 Age Group = 51-60 Gender = MALE Occupational group = NON-MANUAL Total cases = 142

Threshold dB HL≥	0	5	10	15	20	25	30	35	40	45	50	55	65	75	85	95
**Prevalence**	**97.4**	**84.5**	**54.5**	**41.4**	**29.2**	**20.4**	**13.6**	**5.6**	**3.9**	**2.3**	**1.2**	**0.9**	**0.0**	**0.0**	**0.0**	**0.0**
**Lower CI**	93.3	76.8	45.7	33.6	22.7	16.0	11.1	3.5	2.5	1.2	0.5	0.3	0.0	0.0	0.0	0.0
**Upper CI**	99.0	90.0	63.0	49.6	36.7	25.7	16.7	8.7	6.1	4.3	2.9	2.4	2.6	2.6	2.6	2.6
**Design Effect**	1.01	1.11	1.07	1.00	0.95	0.73	0.50	0.67	0.56	0.58	0.61	0.59	-	-	-	-
**Number**	138	118	81	64	46	34	23	17	13	9	5	4	0	0	0	0

Prevalence by age group, gender and occupational group for the better ear over 1, 2, and 3 kHz

Table B123-50 Age Group = 61-70 Gender = MALE Occupational group = NON-MANUAL Total cases = 110

	Threshold dB HL≥															
	0	5	10	15	20	25	30	35	40	45	50	55	65	75	85	95
**Prevalence**	**100.0**	**95.9**	**83.6**	**64.7**	**52.4**	**43.1**	**27.7**	**22.7**	**14.9**	**9.3**	**7.1**	**5.9**	**2.5**	**1.6**	**0.4**	**0.0**
**Lower CI**	96.6	90.0	67.6	49.5	39.1	31.0	22.4	18.8	11.0	6.2	4.5	2.9	0.8	0.4	0.1	0.0
**Upper CI**	100.0	98.4	92.6	77.3	65.5	56.1	33.8	27.3	19.8	13.8	11.1	11.4	7.0	6.7	2.1	3.4
**Design Effect**	-	1.05	1.83	1.62	1.47	1.41	0.69	0.55	0.66	0.70	0.68	0.93	0.96	1.08	0.65	-
**Number**	110	104	94	77	63	51	37	30	21	14	11	10	4	2	1	0

Table B123-51 Age Group = 71-80 Gender = MALE Occupational group = NON-MANUAL Total cases = 55

	Threshold dB HL≥															
	0	5	10	15	20	25	30	35	40	45	50	55	65	75	85	95
**Prevalence**	**100.0**	**100.0**	**97.9**	**88.2**	**70.5**	**60.9**	**47.6**	**32.2**	**28.7**	**18.7**	**17.7**	**8.3**	**2.5**	**1.3**	**0.0**	**0.0**
**Lower CI**	93.5	93.5	89.0	76.1	61.4	50.7	35.4	21.9	19.0	12.1	11.3	5.2	0.7	0.2	0.0	0.0
**Upper CI**	100.0	100.0	99.6	94.6	78.2	70.2	60.0	44.6	40.9	27.7	26.7	13.1	8.6	7.0	6.5	6.5
**Design Effect**	-	-	1.08	1.07	0.71	0.77	0.96	0.94	0.94	0.76	0.77	0.53	0.83	0.85	-	-
**Number**	55	55	54	50	42	37	28	20	17	12	11	5	2	1	0	0

Table B123-52 Age Group = 18-30 Gender = FEMALE Occupational group = MANUAL Total cases = 109

	Threshold dB HL≥															
	0	5	10	15	20	25	30	35	40	45	50	55	65	75	85	95
**Prevalence**	**84.0**	**51.4**	**27.1**	**7.8**	**3.5**	**2.4**	**1.1**	**0.8**	**0.8**	**0.2**	**0.0**	**0.0**	**0.0**	**0.0**	**0.0**	**0.0**
**Lower CI**	72.6	39.1	17.9	3.8	1.5	0.9	0.5	0.3	0.3	0.0	0.0	0.0	0.0	0.0	0.0	0.0
**Upper CI**	91.2	63.5	38.7	15.1	8.2	6.2	2.5	2.1	2.1	1.1	3.4	3.4	3.4	3.4	3.4	3.4
**Design Effect**	1.36	1.34	1.27	1.09	0.91	0.83	0.49	0.48	0.48	0.47	-	-	-	-	-	-
**Number**	95	61	34	13	8	7	5	4	4	1	0	0	0	0	0	0

Table B123-53 Age Group = 31-40 Gender = FEMALE Occupational group = MANUAL Total cases = 103

	Threshold dB HL≥															
	0	5	10	15	20	25	30	35	40	45	50	55	65	75	85	95
**Prevalence**	**86.7**	**55.8**	**25.9**	**14.1**	**4.8**	**3.9**	**3.0**	**3.0**	**2.4**	**0.8**	**0.8**	**0.6**	**0.3**	**0.3**	**0.0**	**0.0**
**Lower CI**	76.7	43.3	17.8	8.6	2.2	1.6	1.0	1.0	0.7	0.3	0.3	0.2	0.1	0.1	0.0	0.0
**Upper CI**	92.8	67.6	36.0	22.1	10.2	9.3	8.8	8.8	8.3	2.3	2.3	2.3	1.7	1.7	3.6	3.6
**Design Effect**	1.23	1.31	1.09	1.00	0.93	0.97	1.05	1.05	1.11	0.52	0.52	0.58	0.56	0.56	-	-
**Number**	93	67	41	23	10	8	5	5	4	3	3	2	1	1	0	0

Table B123-54 Age Group = 41-50 Gender = FEMALE Occupational group = MANUAL Total cases = 135

	Threshold dB HL≥															
	0	5	10	15	20	25	30	35	40	45	50	55	65	75	85	95
**Prevalence**	**90.1**	**74.5**	**47.4**	**27.1**	**14.7**	**9.6**	**8.3**	**4.6**	**2.8**	**2.3**	**1.8**	**1.3**	**0.4**	**0.2**	**0.2**	**0.2**
**Lower CI**	81.9	65.2	38.0	19.4	9.5	5.8	4.9	2.8	1.3	1.1	0.7	0.4	0.1	0.0	0.0	0.0
**Upper CI**	94.8	82.0	56.9	36.4	22.0	15.3	13.9	7.4	5.6	5.0	4.4	4.1	1.3	1.0	1.0	1.0
**Design Effect**	1.26	1.15	1.15	1.15	1.04	0.95	0.95	0.64	0.73	0.72	0.76	0.83	0.49	0.48	0.48	0.48
**Number**	126	103	73	44	29	22	19	14	9	8	6	4	2	1	1	1

Table B123-55 Age Group = 51-60 Gender = FEMALE Occupational group = MANUAL Total cases = 165

	Threshold dB HL≥															
	0	5	10	15	20	25	30	35	40	45	50	55	65	75	85	95
**Prevalence**	**95.5**	**85.8**	**57.4**	**37.4**	**23.4**	**16.7**	**11.6**	**6.0**	**3.4**	**1.5**	**1.5**	**1.0**	**0.5**	**0.2**	**0.2**	**0.2**
**Lower CI**	90.6	78.1	48.2	29.4	17.1	11.3	7.3	3.3	1.8	0.7	0.7	0.5	0.2	0.0	0.0	0.0
**Upper CI**	97.9	91.1	66.2	46.1	31.1	23.9	17.9	10.4	6.2	3.2	3.2	1.8	1.4	1.0	1.0	1.0
**Design Effect**	1.10	1.22	1.21	1.15	1.09	1.11	1.07	0.95	0.77	0.64	0.64	0.39	0.47	0.53	0.53	0.53
**Number**	159	148	110	82	58	41	29	20	14	8	8	7	4	1	1	1

Table B123-56 Age Group = 61-70 Gender = FEMALE Occupational group = MANUAL Total cases = 126

	Threshold dB HL≥															
	0	5	10	15	20	25	30	35	40	45	50	55	65	75	85	95
**Prevalence**	**100.0**	**94.8**	**84.3**	**54.1**	**41.2**	**27.2**	**17.6**	**11.5**	**8.2**	**7.2**	**5.2**	**4.7**	**2.8**	**2.1**	**1.9**	**1.6**
**Lower CI**	97.0	86.9	75.4	43.9	32.1	20.4	12.1	7.5	6.1	5.2	3.7	3.4	2.1	1.6	1.4	0.5
**Upper CI**	100.0	98.1	90.4	63.9	50.9	35.3	24.9	17.3	11.0	9.9	7.2	6.4	3.7	2.8	2.4	5.7
**Design Effect**	0.00	1.35	1.18	1.17	1.12	0.97	0.96	0.88	0.52	0.51	0.45	0.40	0.27	0.25	0.21	0.00
**Number**	126	121	109	80	66	48	39	28	23	20	14	13	6	3	2	1

Prevalence by age group, gender and occupational group for the better ear over 1, 2, and 3 kHz

Table B123-57 Age Group = 71-80 Gender = FEMALE Occupational group = MANUAL Total cases = 76

	Threshold dB HL≥															
	**0**	**5**	**10**	**15**	**20**	**25**	**30**	**35**	**40**	**45**	**50**	**55**	**65**	**75**	**85**	**95**
**Prevalence**	**100.0**	**99.6**	**91.0**	**82.9**	**69.6**	**56.0**	**47.0**	**41.8**	**31.4**	**23.0**	**13.6**	**10.5**	**4.3**	**3.1**	**2.1**	**0.0**
**Lower CI**	95.2	97.6	74.4	68.6	51.0	42.5	36.5	31.3	21.6	13.9	10.0	7.1	2.4	1.4	0.8	0.0
**Upper CI**	100.0	99.9	97.2	91.5	83.5	68.6	57.8	53.1	43.3	35.6	18.4	15.3	7.6	6.6	5.8	4.8
**Design Effect**	-	0.57	1.69	1.37	1.66	1.22	0.97	1.01	1.06	1.17	0.54	0.59	0.55	0.64	0.70	-
**Number**	76	75	71	68	64	59	51	44	39	31	24	18	9	6	4	0

Table B123-58 Age Group = 18-30 Gender = MALE Occupational group = MANUAL Total cases = 109

	Threshold dB HL≥															
	**0**	**5**	**10**	**15**	**20**	**25**	**30**	**35**	**40**	**45**	**50**	**55**	**65**	**75**	**85**	**95**
**Prevalence**	**83.0**	**42.7**	**26.6**	**12.7**	**7.2**	**2.6**	**1.0**	**0.1**	**0.1**	**0.1**	**0.1**	**0.1**	**0.0**	**0.0**	**0.0**	**0.0**
**Lower CI**	71.4	31.2	16.8	7.1	3.2	0.7	0.2	0.0	0.0	0.0	0.0	0.0	0.0	0.0	0.0	0.0
**Upper CI**	90.6	55.1	39.5	21.6	15.2	9.3	5.1	0.4	0.4	0.4	0.4	0.4	3.4	3.4	3.4	3.4
**Design Effect**	1.37	1.33	1.40	1.16	1.19	1.22	0.99	0.27	0.27	0.27	0.27	0.27	-	-	-	-
**Number**	91	56	32	18	9	3	2	1	1	1	1	1	0	0	0	0

Table B123-59 Age Group = 31-40 Gender = MALE Occupational group = MANUAL Total cases = 80

	Threshold dB HL≥															
	**0**	**5**	**10**	**15**	**20**	**25**	**30**	**35**	**40**	**45**	**50**	**55**	**65**	**75**	**85**	**95**
**Prevalence**	**80.9**	**68.5**	**33.9**	**19.6**	**8.9**	**7.0**	**4.0**	**2.7**	**2.7**	**2.3**	**2.1**	**2.1**	**1.9**	**1.9**	**1.9**	**1.9**
**Lower CI**	68.4	55.7	21.4	10.2	3.9	2.6	1.5	0.7	0.7	0.5	0.4	0.4	0.3	0.3	0.3	0.3
**Upper CI**	89.2	79.0	49.2	34.5	19.1	17.9	10.6	9.8	9.8	9.8	9.9	9.9	10.2	10.2	10.2	10.2
**Design Effect**	1.22	1.18	1.39	1.42	1.18	1.29	0.97	1.09	1.09	1.15	1.19	1.19	1.25	1.25	1.25	1.25
**Number**	69	58	31	18	12	8	7	4	4	3	2	2	1	1	1	1

Table B123-60 Age Group = 41-50 Gender = MALE Occupational group = MANUAL Total cases = 127

	Threshold dB HL≥															
	**0**	**5**	**10**	**15**	**20**	**25**	**30**	**35**	**40**	**45**	**50**	**55**	**65**	**75**	**85**	**95**
**Prevalence**	**98.4**	**80.5**	**59.8**	**32.3**	**23.5**	**11.4**	**9.2**	**6.7**	**3.5**	**2.1**	**1.1**	**0.5**	**0.5**	**0.5**	**0.2**	**0.0**
**Lower CI**	91.2	70.5	48.7	24.0	16.1	7.0	5.5	4.1	1.8	1.1	0.4	0.1	0.1	0.1	0.0	0.0
**Upper CI**	99.7	87.7	70.0	41.9	32.9	18.0	14.9	10.7	6.7	3.8	2.9	1.7	1.7	1.7	1.3	2.9
**Design Effect**	1.45	1.26	1.28	1.12	1.16	0.99	0.92	0.75	0.74	0.53	0.62	0.54	0.54	0.54	0.54	-
**Number**	126	106	83	56	42	25	21	17	10	8	4	2	2	2	1	0

Table B123-61 Age Group = 51-60 Gender = MALE Occupational group = MANUAL Total cases = 198

	Threshold dB HL≥															
	**0**	**5**	**10**	**15**	**20**	**25**	**30**	**35**	**40**	**45**	**50**	**55**	**65**	**75**	**85**	**95**
**Prevalence**	**100.0**	**94.1**	**79.6**	**56.7**	**45.0**	**31.2**	**23.4**	**17.3**	**12.4**	**9.9**	**7.5**	**6.0**	**1.6**	**0.4**	**0.0**	**0.0**
**Lower CI**	98.1	88.4	72.0	48.5	38.0	25.1	18.0	12.9	8.8	6.7	4.7	3.5	0.8	0.1	0.0	0.0
**Upper CI**	100.0	97.1	85.5	64.5	52.3	38.1	29.7	22.8	17.2	14.3	11.7	9.9	3.4	1.5	1.9	1.9
**Design Effect**	0.00	1.28	1.21	1.17	1.05	1.01	0.99	0.94	0.91	0.90	0.93	0.95	0.69	0.67	-	-
**Number**	198	189	167	135	115	85	65	52	40	33	25	20	8	2	0	0

Table B123-62 Age Group = 61-70 Gender = MALE Occupational group = MANUAL Total cases = 172

	Threshold dB HL≥															
	**0**	**5**	**10**	**15**	**20**	**25**	**30**	**35**	**40**	**45**	**50**	**55**	**65**	**75**	**85**	**95**
**Prevalence**	**98.8**	**97.5**	**93.3**	**75.3**	**61.0**	**49.1**	**40.9**	**28.5**	**21.5**	**13.4**	**9.3**	**6.3**	**3.7**	**2.1**	**0.7**	**0.6**
**Lower CI**	93.3	91.9	86.7	64.5	50.1	39.2	31.3	21.5	15.5	10.3	7.0	4.3	2.2	1.0	0.2	0.1
**Upper CI**	99.8	99.3	96.8	83.7	70.9	59.1	51.2	36.8	28.9	17.4	12.3	9.1	6.3	4.2	2.3	2.2
**Design Effect**	1.46	1.39	1.31	1.51	1.46	1.37	1.38	1.15	1.10	0.70	0.61	0.66	0.71	0.71	0.70	0.74
**Number**	171	170	166	143	126	109	88	72	59	47	35	24	13	8	3	2

Table B123-63 Age Group = 71-80 Gender = MALE Occupational group = MANUAL Total cases = 72

	Threshold dB HL≥															
	**0**	**5**	**10**	**15**	**20**	**25**	**30**	**35**	**40**	**45**	**50**	**55**	**65**	**75**	**85**	**95**
**Prevalence**	**100.0**	**100.0**	**100.0**	**90.0**	**72.5**	**65.3**	**49.2**	**33.7**	**25.5**	**22.8**	**17.8**	**14.7**	**6.9**	**2.4**	**1.0**	**0.6**
**Lower CI**	94.9	94.9	94.9	71.2	56.0	49.3	36.0	27.9	20.0	16.7	12.1	9.1	3.6	0.9	0.3	0.1
**Upper CI**	100.0	100.0	100.0	97.0	84.5	78.5	62.6	40.2	32.0	30.4	25.4	22.8	12.7	5.9	3.5	3.1
**Design Effect**	-	-	-	1.80	1.44	1.39	1.19	0.57	0.60	0.71	0.76	0.84	0.76	0.66	0.60	0.63
**Number**	72	72	72	68	64	61	51	42	34	31	26	20	10	4	2	1

Prevalence by age group, gender and occupational group for the better ear over 1, 2, and 4 kHz

Table B124-1 Age Group = 18-80 Gender = OVERALL Occupational group = OVERALL Total cases = 2663

Threshold dB HL≥

	0	5	10	15	20	25	30	35	40	45	50	55	65	75	85	95
Prevalence	**91.7**	**71.4**	**51.7**	**35.4**	**25.4**	**18.7**	**14.0**	**10.3**	**7.4**	**5.4**	**3.7**	**2.7**	**1.4**	**0.8**	**0.4**	**0.3**
Lower CI	89.9	69.3	49.8	33.7	23.9	17.6	13.0	9.4	6.7	4.7	3.2	2.3	1.1	0.6	0.2	0.1
Upper CI	93.1	73.4	53.7	37.0	26.9	20.0	15.0	11.3	8.3	6.1	4.2	3.1	1.7	1.1	0.7	0.5
Design Effect	1.49	1.20	1.03	0.90	0.89	0.83	0.78	0.80	0.81	0.79	0.68	0.65	0.68	0.76	0.91	1.02
Number	2543	2181	1741	1329	1035	848	676	530	414	323	241	189	98	56	24	14

Table B124-2 Age Group = 18-80 Gender = FEMALE Occupational group = OVERALL Total cases = 1386

Threshold dB HL≥

	0	5	10	15	20	25	30	35	40	45	50	55	65	75	85	95
Prevalence	**92.6**	**70.6**	**48.0**	**32.0**	**21.1**	**15.4**	**11.4**	**8.7**	**6.7**	**5.0**	**3.0**	**2.3**	**1.3**	**0.8**	**0.4**	**0.2**
Lower CI	90.5	67.9	45.5	30.0	19.3	13.8	10.1	7.6	5.7	4.1	2.5	1.8	1.0	0.6	0.2	0.1
Upper CI	94.2	73.2	50.4	34.1	23.1	17.1	12.8	10.0	7.9	6.1	3.6	2.8	1.6	1.2	0.7	0.4
Design Effect	1.33	1.12	0.94	0.84	0.87	0.85	0.79	0.80	0.86	0.87	0.62	0.61	0.55	0.62	0.67	0.66
Number	1321	1096	826	603	432	353	282	227	183	143	101	83	50	31	14	8

Table B124-3 Age Group = 18-80 Gender = MALE Occupational group = OVERALL Total cases = 1277

Threshold dB HL≥

	0	5	10	15	20	25	30	35	40	45	50	55	65	75	85	95
Prevalence	**90.6**	**72.3**	**56.0**	**39.1**	**30.2**	**22.5**	**16.8**	**12.1**	**8.2**	**5.8**	**4.4**	**3.2**	**1.5**	**0.8**	**0.4**	**0.3**
Lower CI	87.6	69.1	52.9	36.6	27.9	20.7	15.3	10.8	7.2	5.0	3.6	2.6	1.1	0.5	0.2	0.1
Upper CI	92.9	75.4	59.1	41.7	32.5	24.4	18.5	13.6	9.4	6.8	5.3	4.0	2.1	1.4	1.0	1.0
Design Effect	1.64	1.29	1.14	0.97	0.92	0.82	0.78	0.79	0.75	0.70	0.73	0.69	0.79	0.89	1.11	1.22
Number	1222	1085	915	726	603	495	394	303	231	180	140	106	48	25	10	6

Table B124-4 Age Group = 18-30 Gender = OVERALL Occupational group = OVERALL Total cases = 401

Threshold dB HL≥

	0	5	10	15	20	25	30	35	40	45	50	55	65	75	85	95
Prevalence	**78.8**	**41.2**	**19.9**	**7.5**	**4.5**	**1.8**	**0.8**	**0.7**	**0.4**	**0.1**	**0.0**	**0.0**	**0.0**	**0.0**	**0.0**	**0.0**
Lower CI	73.5	35.7	15.8	5.1	2.7	0.9	0.4	0.3	0.1	0.0	0.0	0.0	0.0	0.0	0.0	0.0
Upper CI	83.4	46.9	24.7	10.9	7.2	3.7	1.9	1.8	0.8	0.3	0.1	0.1	0.1	0.9	0.9	0.9
Design Effect	1.24	1.17	1.15	1.12	1.09	1.04	0.78	0.80	0.55	0.36	0.20	0.20	0.20	-	-	-
Number	328	191	100	43	26	13	9	8	6	3	2	2	2	0	0	0

Table B124-5 Age Group = 31-40 Gender = OVERALL Occupational group = OVERALL Total cases = 331

Threshold dB HL≥

	0	5	10	15	20	25	30	35	40	45	50	55	65	75	85	95
Prevalence	**88.5**	**59.1**	**29.4**	**14.6**	**7.0**	**3.7**	**2.5**	**1.8**	**1.6**	**1.1**	**0.9**	**0.9**	**0.7**	**0.7**	**0.6**	**0.6**
Lower CI	83.4	52.7	24.0	10.9	4.6	2.2	1.3	0.8	0.7	0.4	0.3	0.3	0.1	0.1	0.1	0.1
Upper CI	92.2	65.2	35.4	19.3	10.5	6.4	4.6	3.9	3.8	3.1	3.0	3.0	3.0	3.0	3.1	3.1
Design Effect	1.28	1.19	1.17	1.11	1.07	1.00	0.94	1.01	1.03	1.04	1.11	1.14	1.27	1.27	1.36	1.36
Number	302	216	118	68	35	23	16	11	10	8	6	5	2	2	1	1

Table B124-6 Age Group = 41-50 Gender = OVERALL Occupational group = OVERALL Total cases = 450

Threshold dB HL≥

	0	5	10	15	20	25	30	35	40	45	50	55	65	75	85	95
Prevalence	**97.1**	**74.1**	**49.5**	**27.5**	**17.1**	**10.9**	**7.3**	**4.6**	**2.8**	**1.7**	**1.2**	**1.2**	**0.5**	**0.3**	**0.1**	**0.1**
Lower CI	94.8	69.2	44.5	23.4	14.0	8.5	5.4	3.3	1.9	1.1	0.7	0.6	0.2	0.1	0.0	0.0
Upper CI	98.4	78.3	54.5	32.0	20.8	13.9	9.8	6.3	4.3	2.7	2.2	2.1	1.1	0.9	0.6	0.6
Design Effect	1.12	1.13	1.09	1.05	0.99	0.93	0.90	0.76	0.76	0.65	0.71	0.72	0.62	0.63	0.60	0.60
Number	439	350	251	153	107	76	56	41	26	18	13	12	6	4	2	2

Table B124-7 Age Group = 51-60 Gender = OVERALL Occupational group = OVERALL Total cases = 682

Threshold dB HL≥

	0	5	10	15	20	25	30	35	40	45	50	55	65	75	85	95
Prevalence	**99.1**	**91.5**	**71.6**	**47.5**	**31.1**	**23.0**	**16.0**	**10.1**	**6.6**	**5.3**	**3.8**	**3.0**	**1.4**	**0.6**	**0.1**	**0.1**
Lower CI	97.9	88.8	67.6	43.5	27.9	20.2	13.7	8.3	5.3	4.1	2.8	2.1	0.8	0.3	0.0	0.0
Upper CI	99.6	93.7	75.3	51.5	34.6	26.0	18.6	12.2	8.1	6.8	5.1	4.3	2.3	1.2	0.3	0.3
Design Effect	1.16	1.17	1.14	1.07	0.96	0.92	0.90	0.87	0.76	0.79	0.81	0.83	0.80	0.74	0.50	0.50
Number	677	635	525	394	287	225	166	115	88	69	53	42	20	9	3	3

Prevalence by age group, gender and occupational group for the better ear over 1, 2, and 4 kHz

Table B124-8 Age Group = 61-70 Gender = OVERALL Occupational group = OVERALL Total cases = 527

Threshold dB HL≥	0	5	10	15	20	25	30	35	40	45	50	55	65	75	85	95
**Prevalence**	**99.8**	**97.6**	**89.5**	**73.7**	**55.6**	**44.5**	**30.3**	**23.1**	**15.9**	**10.8**	**7.0**	**5.2**	**3.0**	**1.9**	**0.9**	**0.8**
**Lower CI**	98.8	95.1	85.9	68.9	50.5	39.9	26.8	19.7	13.3	9.3	5.8	4.2	2.1	1.2	0.5	0.4
**Upper CI**	100.0	98.8	92.2	78.1	60.6	49.2	34.2	26.8	19.0	12.5	8.5	6.4	4.1	3.0	1.7	1.6
**Design Effect**	1.04	1.36	1.21	1.23	1.19	1.11	0.95	0.98	0.92	0.61	0.62	0.57	0.66	0.72	0.73	0.76
**Number**	526	518	481	416	344	296	226	186	146	116	82	63	34	21	10	7

Table B124-9 Age Group = 71-80 Gender = OVERALL Occupational group = OVERALL Total cases = 272

Threshold dB HL≥	0	5	10	15	20	25	30	35	40	45	50	55	65	75	85	95
**Prevalence**	**99.6**	**99.6**	**95.8**	**88.3**	**77.8**	**62.9**	**57.2**	**46.0**	**35.7**	**26.6**	**17.8**	**12.0**	**5.5**	**3.2**	**1.2**	**0.1**
**Lower CI**	98.0	98.0	89.9	82.3	69.4	55.5	50.1	39.2	29.3	20.9	14.3	9.5	4.0	2.1	0.6	0.0
**Upper CI**	99.9	99.9	98.3	92.5	84.4	69.7	64.1	52.9	42.7	33.2	22.0	15.0	7.4	4.8	2.5	0.8
**Design Effect**	0.98	0.98	1.64	1.32	1.53	1.25	1.21	1.17	1.18	1.18	0.85	0.70	0.61	0.62	0.69	0.63
**Number**	271	271	266	255	236	215	203	169	138	109	85	65	34	20	8	1

Table B124-10 Age Group = 18-80 Gender = OVERALL Occupational group = NON-MANUAL Total cases = 1184

Threshold dB HL≥	0	5	10	15	20	25	30	35	40	45	50	55	65	75	85	95
**Prevalence**	**89.6**	**67.2**	**45.9**	**30.0**	**21.4**	**16.8**	**12.4**	**8.8**	**6.8**	**5.0**	**2.7**	**1.7**	**0.9**	**0.6**	**0.2**	**0.1**
**Lower CI**	86.8	64.2	43.2	28.0	19.5	15.4	11.3	7.9	6.1	4.4	2.2	1.4	0.6	0.3	0.1	0.1
**Upper CI**	91.9	70.1	48.6	32.2	23.4	18.4	13.6	9.8	7.6	5.7	3.2	2.1	1.2	0.9	0.4	0.3
**Design Effect**	1.44	1.11	0.95	0.81	0.83	0.69	0.62	0.57	0.53	0.54	0.58	0.48	0.58	0.65	0.60	0.58
**Number**	1113	916	685	504	368	309	243	185	145	108	79	59	31	18	8	6

Table B124-11 Age Group = 18-80 Gender = OVERALL Occupational group = MANUAL Total cases = 1475

Threshold dB HL≥	0	5	10	15	20	25	30	35	40	45	50	55	65	75	85	95
**Prevalence**	**93.6**	**76.1**	**57.2**	**39.3**	**28.5**	**20.4**	**15.2**	**11.3**	**7.8**	**5.5**	**4.0**	**3.2**	**1.7**	**1.0**	**0.6**	**0.4**
**Lower CI**	91.2	72.9	54.2	36.8	26.3	18.6	13.9	10.1	6.9	4.8	3.4	2.7	1.3	0.7	0.3	0.2
**Upper CI**	95.4	79.0	60.3	41.9	30.7	22.3	16.7	12.6	8.9	6.4	4.7	3.9	2.2	1.4	1.0	0.8
**Design Effect**	1.66	1.39	1.21	1.02	0.96	0.91	0.78	0.77	0.74	0.68	0.62	0.65	0.66	0.70	0.84	0.92
**Number**	1427	1263	1054	823	665	538	433	345	269	215	162	130	67	38	16	8

Table B124-12 Age Group = 18-30 Gender = FEMALE Occupational group = OVERALL Total cases = 224

Threshold dB HL≥	0	5	10	15	20	25	30	35	40	45	50	55	65	75	85	95
**Prevalence**	**82.3**	**43.1**	**17.8**	**5.7**	**3.1**	**1.8**	**1.0**	**0.8**	**0.6**	**0.1**	**0.0**	**0.0**	**0.0**	**0.0**	**0.0**	**0.0**
**Lower CI**	75.8	36.0	13.2	3.5	1.8	0.9	0.4	0.3	0.2	0.0	0.0	0.0	0.0	0.0	0.0	0.0
**Upper CI**	87.3	50.4	23.6	9.1	5.5	3.6	2.1	1.8	1.5	0.6	1.7	1.7	1.7	1.7	1.7	1.7
**Design Effect**	1.16	1.13	1.04	0.90	0.80	0.76	0.60	0.59	0.59	0.47	-	-	-	-	-	-
**Number**	187	107	53	22	14	9	6	5	4	1	0	0	0	0	0	0

Table B124-13 Age Group = 31-40 Gender = FEMALE Occupational group = OVERALL Total cases = 189

Threshold dB HL≥	0	5	10	15	20	25	30	35	40	45	50	55	65	75	85	95
**Prevalence**	**87.5**	**54.2**	**22.9**	**11.3**	**4.3**	**3.3**	**2.4**	**1.9**	**1.6**	**0.9**	**0.5**	**0.5**	**0.2**	**0.2**	**0.0**	**0.0**
**Lower CI**	80.9	45.9	17.2	7.7	2.4	1.9	1.1	0.8	0.6	0.4	0.2	0.2	0.0	0.0	0.0	0.0
**Upper CI**	92.0	62.2	29.8	16.2	7.4	5.9	4.9	4.4	4.1	2.2	1.7	1.7	1.2	1.2	2.0	2.0
**Design Effect**	1.18	1.16	1.06	0.95	0.85	0.77	0.82	0.86	0.89	0.63	0.63	0.63	0.63	0.63	-	-
**Number**	170	116	59	34	16	14	9	7	6	5	3	3	1	1	0	0

Table B124-14 Age Group = 41-50 Gender = FEMALE Occupational group = OVERALL Total cases = 247

Threshold dB HL≥	0	5	10	15	20	25	30	35	40	45	50	55	65	75	85	95
**Prevalence**	**97.1**	**71.3**	**43.3**	**22.0**	**12.0**	**7.9**	**5.3**	**3.8**	**2.5**	**1.5**	**1.1**	**1.1**	**0.2**	**0.2**	**0.1**	**0.1**
**Lower CI**	93.5	64.6	36.9	17.1	8.8	5.5	3.5	2.4	1.4	0.7	0.5	0.5	0.1	0.1	0.0	0.0
**Upper CI**	98.7	77.1	50.0	27.7	16.2	11.2	8.1	5.9	4.4	3.1	2.6	2.6	0.9	0.9	0.6	0.6
**Design Effect**	1.15	1.11	1.07	1.03	0.91	0.84	0.82	0.70	0.74	0.74	0.74	0.74	0.55	0.55	0.51	0.51
**Number**	241	185	123	68	44	33	25	20	13	8	6	6	2	2	1	1

Prevalence by age group, gender and occupational group for the better ear over 1, 2, and 4 kHz

Table B124-15 Age Group = 51-60 Gender = FEMALE Occupational group = OVERALL Total cases = 341

	Threshold dB HL≥															
	**0**	5	**10**	**15**	**20**	**25**	**30**	**35**	**40**	**45**	**50**	**55**	**65**	**75**	**85**	**95**
**Prevalence**	**99.2**	**89.2**	**65.3**	**40.3**	**19.8**	**14.4**	**9.4**	**5.6**	**3.7**	**3.0**	**2.1**	**1.6**	**1.1**	**0.7**	**0.2**	**0.2**
**Lower CI**	97.1	84.8	59.4	34.8	16.2	11.5	7.0	3.9	2.6	2.0	1.2	0.9	0.5	0.3	0.1	0.1
**Upper CI**	99.8	92.4	70.8	46.0	24.1	17.9	12.6	7.9	5.3	4.6	3.4	2.8	2.1	1.8	0.6	0.6
**Design Effect**	1.19	1.15	1.14	1.08	0.93	0.86	0.90	0.80	0.65	0.70	0.71	0.67	0.67	0.75	0.48	0.48
**Number**	339	311	245	173	105	82	58	40	31	24	18	15	10	6	3	3

Table B124-16 Age Group = 61-70 Gender = FEMALE Occupational group = OVERALL Total cases = 242

	Threshold dB HL≥															
	**0**	5	**10**	**15**	**20**	**25**	**30**	**35**	**40**	**45**	**50**	**55**	**65**	**75**	**85**	**95**
**Prevalence**	**100.0**	**96.1**	**83.7**	**67.4**	**46.3**	**35.0**	**22.0**	**15.1**	**11.4**	**7.1**	**4.3**	**3.5**	**2.4**	**1.7**	**1.1**	**1.0**
**Lower CI**	98.4	91.5	77.5	60.3	39.5	29.2	18.0	11.5	8.1	5.7	3.1	2.4	1.4	0.9	0.5	0.4
**Upper CI**	100.0	98.2	88.5	73.8	53.1	41.4	26.5	19.6	15.7	8.8	6.0	5.0	3.9	3.2	2.6	2.5
**Design Effect**	0.00	1.30	1.18	1.15	1.10	1.02	0.81	0.90	0.94	0.48	0.55	0.55	0.62	0.68	0.74	0.76
**Number**	242	235	208	175	132	107	78	63	53	42	29	24	15	9	5	4

Table B124-17 Age Group = 71-80 Gender = FEMALE Occupational group = OVERALL Total cases = 143

	Threshold dB HL≥															
	**0**	5	**10**	**15**	**20**	**25**	**30**	**35**	**40**	**45**	**50**	**55**	**65**	**75**	**85**	**95**
**Prevalence**	**99.4**	**99.4**	**93.8**	**85.1**	**74.9**	**56.1**	**52.3**	**44.6**	**35.8**	**28.8**	**16.7**	**11.3**	**5.9**	**3.7**	**1.5**	**0.0**
**Lower CI**	96.8	96.8	84.3	76.0	62.5	45.7	42.7	35.7	27.1	20.7	12.8	8.3	4.2	2.2	0.6	0.0
**Upper CI**	99.9	99.9	97.7	91.2	84.2	66.0	61.8	53.9	45.5	38.6	21.7	15.3	8.4	6.1	3.6	2.6
**Design Effect**	0.91	0.91	1.59	1.31	1.56	1.28	1.19	1.14	1.19	1.22	0.73	0.67	0.54	0.61	0.70	-
**Number**	142	142	138	131	121	108	106	92	76	63	45	35	22	13	5	0

Table B124-18 Age Group = 18-30 Gender = MALE Occupational group = OVERALL Total cases = 177

	Threshold dB HL≥															
	**0**	5	**10**	**15**	**20**	**25**	**30**	**35**	**40**	**45**	**50**	**55**	**65**	**75**	**85**	**95**
**Prevalence**	**75.3**	**39.3**	**22.1**	**9.3**	**5.7**	**1.7**	**0.6**	**0.6**	**0.1**	**0.1**	**0.1**	**0.1**	**0.1**	**0.0**	**0.0**	**0.0**
**Lower CI**	66.4	31.2	15.6	5.3	2.8	0.5	0.1	0.1	0.0	0.0	0.0	0.0	0.0	0.0	0.0	0.0
**Upper CI**	82.5	48.2	30.3	15.8	11.1	5.9	3.0	3.0	0.2	0.2	0.2	0.2	0.2	2.1	2.1	2.1
**Design Effect**	1.28	1.20	1.22	1.20	1.17	1.22	0.94	0.94	0.19	0.19	0.19	0.19	0.19	-	-	-
**Number**	141	84	47	21	12	4	3	3	2	2	2	2	2	0	0	0

Table B124-19 Age Group = 31-40 Gender = MALE Occupational group = OVERALL Total cases = 142

	Threshold dB HL≥															
	**0**	5	**10**	**15**	**20**	**25**	**30**	**35**	**40**	**45**	**50**	**55**	**65**	**75**	**85**	**95**
**Prevalence**	**89.7**	**64.3**	**36.6**	**18.5**	**10.0**	**4.1**	**2.6**	**1.7**	**1.7**	**1.4**	**1.4**	**1.3**	**1.2**	**1.2**	**1.2**	**1.2**
**Lower CI**	80.7	54.2	27.4	12.1	5.7	1.8	1.0	0.5	0.5	0.3	0.3	0.3	0.2	0.2	0.2	0.2
**Upper CI**	94.8	73.3	46.9	27.1	17.0	9.5	6.9	6.3	6.3	6.2	6.2	6.4	6.5	6.5	6.5	6.5
**Design Effect**	1.39	1.23	1.25	1.18	1.13	1.10	1.02	1.14	1.14	1.22	1.22	1.26	1.31	1.31	1.31	1.31
**Number**	132	100	59	34	19	9	7	4	4	3	3	2	1	1	1	1

Table B124-20 Age Group = 41-50 Gender = MALE Occupational group = OVERALL Total cases = 203

	Threshold dB HL≥															
	**0**	5	**10**	**15**	**20**	**25**	**30**	**35**	**40**	**45**	**50**	**55**	**65**	**75**	**85**	**95**
**Prevalence**	**97.2**	**77.1**	**56.3**	**33.5**	**22.7**	**14.2**	**9.5**	**5.5**	**3.2**	**1.9**	**1.4**	**1.2**	**0.7**	**0.4**	**0.2**	**0.2**
**Lower CI**	93.6	69.9	48.6	26.9	17.4	10.1	6.3	3.5	1.8	1.1	0.6	0.5	0.3	0.1	0.0	0.0
**Upper CI**	98.8	83.1	63.7	40.7	29.2	19.5	14.1	8.5	5.7	3.3	3.0	2.8	1.9	1.6	1.1	1.1
**Design Effect**	1.08	1.15	1.12	1.07	1.03	0.98	0.95	0.79	0.78	0.56	0.69	0.70	0.63	0.67	0.63	0.63
**Number**	198	165	128	85	63	43	31	21	13	10	7	6	4	2	1	1

Table B124-21 Age Group = 51-60 Gender = MALE Occupational group = OVERALL Total cases = 341

	Threshold dB HL≥															
	**0**	5	**10**	**15**	**20**	**25**	**30**	**35**	**40**	**45**	**50**	**55**	**65**	**75**	**85**	**95**
**Prevalence**	**99.0**	**94.1**	**78.6**	**55.5**	**43.8**	**32.6**	**23.4**	**15.1**	**9.8**	**7.8**	**5.7**	**4.6**	**1.7**	**0.4**	**0.0**	**0.0**
**Lower CI**	96.8	90.4	73.2	49.7	38.4	27.8	19.4	11.9	7.5	5.7	3.9	3.0	0.8	0.1	0.0	0.0
**Upper CI**	99.7	96.5	83.2	61.1	49.3	37.7	27.9	19.0	12.7	10.6	8.3	7.1	3.5	1.2	1.1	1.1
**Design Effect**	1.16	1.20	1.15	1.09	1.05	1.00	0.95	0.94	0.82	0.86	0.87	0.91	0.91	0.71	-	-
**Number**	338	324	280	221	182	143	108	75	57	45	35	27	10	3	0	0

Prevalence by age group, gender and occupational group for the better ear over 1, 2, and 4 kHz

Table B124-22 Age Group = 61-70 Gender = MALE Occupational group = OVERALL Total cases = 285

	Threshold dB HL≥															
	**0**	**5**	**10**	**15**	**20**	**25**	**30**	**35**	**40**	**45**	**50**	**55**	**65**	**75**	**85**	**95**
Prevalence	**99.5**	**99.4**	**96.1**	**81.0**	**66.5**	**55.6**	**40.0**	**32.4**	**21.2**	**15.1**	**10.3**	**7.3**	**3.7**	**2.2**	**0.8**	**0.5**
Lower CI	97.5	97.5	92.7	74.2	58.9	48.3	33.9	26.8	17.2	12.3	8.1	5.7	2.4	1.2	0.3	0.2
Upper CI	99.9	99.8	97.9	86.4	73.4	62.6	46.5	38.6	25.8	18.3	13.1	9.4	5.6	3.9	1.7	1.4
Design Effect	1.14	1.04	1.12	1.35	1.35	1.25	1.12	1.10	0.90	0.72	0.71	0.61	0.72	0.76	0.64	0.68
Number	284	283	273	241	212	189	148	123	93	74	53	39	19	12	5	3

Table B124-23 Age Group = 71-80 Gender = MALE Occupational group = OVERALL Total cases = 129

	Threshold dB HL≥															
	**0**	**5**	**10**	**15**	**20**	**25**	**30**	**35**	**40**	**45**	**50**	**55**	**65**	**75**	**85**	**95**
Prevalence	**100.0**	**100.0**	**98.7**	**92.7**	**81.5**	**72.0**	**63.8**	**47.4**	**35.6**	**23.2**	**19.6**	**13.4**	**4.9**	**2.5**	**0.9**	**0.4**
Lower CI	97.1	97.1	92.7	85.0	70.8	62.5	53.5	37.8	27.0	16.9	13.8	9.6	2.8	1.3	0.3	0.1
Upper CI	100.0	100.0	99.8	96.6	88.8	79.9	72.9	57.2	45.2	31.0	27.0	18.5	8.6	4.8	2.7	2.0
Design Effect	-	-	1.32	1.25	1.36	1.14	1.20	1.15	1.12	0.97	0.98	0.76	0.76	0.63	0.62	0.67
Number	129	129	128	124	115	107	97	77	62	46	40	30	12	7	3	1

Table B124-24 Age Group = 18-30 Gender = OVERALL Occupational group = NON-MANUAL Total cases = 181

	Threshold dB HL≥															
	**0**	**5**	**10**	**15**	**20**	**25**	**30**	**35**	**40**	**45**	**50**	**55**	**65**	**75**	**85**	**95**
Prevalence	**71.4**	**31.5**	**10.9**	**3.2**	**1.5**	**0.7**	**0.5**	**0.5**	**0.1**	**0.1**	**0.1**	**0.1**	**0.1**	**0.0**	**0.0**	**0.0**
Lower CI	62.7	24.4	6.8	1.7	0.7	0.2	0.1	0.1	0.0	0.0	0.0	0.0	0.0	0.0	0.0	0.0
Upper CI	78.8	39.5	17.0	5.9	3.3	2.4	2.4	2.4	2.2	2.2	2.2	2.2	2.2	2.1	2.1	2.1
Design Effect	1.24	1.13	1.12	0.78	0.68	0.79	0.85	0.85	0.00	0.00	0.00	0.00	0.00	-	-	-
Number	136	65	27	10	6	3	2	2	1	1	1	1	1	0	0	0

Table B124-25 Age Group = 31-40 Gender = OVERALL Occupational group = NON-MANUAL Total cases = 148

	Threshold dB HL≥															
	**0**	**5**	**10**	**15**	**20**	**25**	**30**	**35**	**40**	**45**	**50**	**55**	**65**	**75**	**85**	**95**
Prevalence	**89.5**	**56.4**	**25.8**	**8.9**	**4.7**	**2.0**	**1.3**	**0.4**	**0.4**	**0.4**	**0.0**	**0.0**	**0.0**	**0.0**	**0.0**	**0.0**
Lower CI	81.8	46.4	18.0	5.2	2.1	0.9	0.5	0.1	0.1	0.1	0.0	0.0	0.0	0.0	0.0	0.0
Upper CI	94.2	65.8	35.5	14.7	10.1	4.2	3.8	1.6	1.6	1.6	2.5	2.5	2.5	2.5	2.5	2.5
Design Effect	1.25	1.23	1.26	1.02	1.12	0.68	0.80	0.59	0.59	0.59	-	-	-	-	-	-
Number	134	90	40	20	9	6	4	2	2	2	0	0	0	0	0	0

Table B124-26 Age Group = 41-50 Gender = OVERALL Occupational group = NON-MANUAL Total cases = 188

	Threshold dB HL≥															
	**0**	**5**	**10**	**15**	**20**	**25**	**30**	**35**	**40**	**45**	**50**	**55**	**65**	**75**	**85**	**95**
Prevalence	**97.0**	**69.0**	**40.4**	**20.1**	**12.6**	**8.6**	**4.9**	**2.6**	**1.3**	**0.4**	**0.4**	**0.4**	**0.4**	**0.0**	**0.0**	**0.0**
Lower CI	93.6	61.6	33.3	14.7	8.4	5.8	2.9	1.9	0.9	0.1	0.1	0.1	0.1	0.0	0.0	0.0
Upper CI	98.7	75.6	47.9	26.7	18.4	12.6	8.2	3.6	1.9	1.3	1.3	1.3	1.3	2.0	2.0	2.0
Design Effect	0.98	1.06	1.05	1.05	1.05	0.84	0.84	0.37	0.31	0.60	0.60	0.60	0.60	-	-	-
Number	182	130	83	45	28	22	15	9	4	2	2	2	2	0	0	0

Table B124-27 Age Group = 51-60 Gender = OVERALL Occupational group = NON-MANUAL Total cases = 317

	Threshold dB HL≥															
	**0**	**5**	**10**	**15**	**20**	**25**	**30**	**35**	**40**	**45**	**50**	**55**	**65**	**75**	**85**	**95**
Prevalence	**98.4**	**90.7**	**65.5**	**40.6**	**24.5**	**18.0**	**11.7**	**5.2**	**3.9**	**2.9**	**2.3**	**1.7**	**0.7**	**0.6**	**0.2**	**0.2**
Lower CI	96.2	86.6	59.4	35.2	20.5	14.7	9.6	3.9	2.9	2.1	1.5	1.0	0.3	0.2	0.0	0.0
Upper CI	99.3	93.7	71.2	46.3	29.1	21.8	14.3	7.1	5.3	4.0	3.4	2.9	1.6	1.5	0.7	0.7
Design Effect	1.06	1.10	1.14	1.03	0.91	0.84	0.66	0.64	0.55	0.53	0.57	0.65	0.68	0.72	0.54	0.54
Number	312	287	217	151	98	74	54	37	29	22	18	13	5	4	2	2

Table B124-28 Age Group = 61-70 Gender = OVERALL Occupational group = NON-MANUAL Total cases = 227

	Threshold dB HL≥															
	**0**	**5**	**10**	**15**	**20**	**25**	**30**	**35**	**40**	**45**	**50**	**55**	**65**	**75**	**85**	**95**
Prevalence	**100.0**	**97.4**	**87.3**	**69.6**	**51.6**	**42.9**	**28.1**	**21.2**	**17.1**	**10.2**	**6.1**	**4.3**	**2.5**	**1.6**	**0.6**	**0.6**
Lower CI	98.3	93.2	81.7	61.0	43.4	35.5	23.7	18.0	14.2	8.0	4.3	3.3	1.4	0.7	0.2	0.2
Upper CI	100.0	99.0	91.3	76.9	59.6	50.7	33.0	24.8	20.3	13.0	8.5	5.6	4.5	3.6	1.8	1.8
Design Effect	-	1.28	1.11	1.34	1.26	1.20	0.81	0.64	0.63	0.63	0.67	0.44	0.73	0.84	0.69	0.69
Number	227	222	198	166	128	111	83	65	53	38	26	21	13	7	4	4

Prevalence by age group, gender and occupational group for the better ear over 1, 2, and 4 kHz

Table B124-29 Age Group = 71-80 Gender = OVERALL Occupational group = NON-MANUAL Total cases = 123

	Threshold dB HL≥															
	0	5	10	15	20	25	30	35	40	45	50	55	65	75	85	95
**Prevalence**	**99.1**	**99.1**	**96.7**	**87.5**	**75.3**	**65.6**	**59.5**	**47.5**	**38.1**	**31.4**	**14.9**	**9.0**	**3.5**	**2.7**	**0.7**	**0.0**
**Lower CI**	95.1	95.1	90.3	75.9	63.2	57.7	50.9	39.8	31.4	25.5	11.0	6.5	1.9	1.3	0.2	0.0
**Upper CI**	99.8	99.8	99.0	94.0	84.3	72.7	67.6	55.3	45.2	37.9	19.9	12.3	6.4	5.7	2.6	3.0
**Design Effect**	1.05	1.05	1.24	1.54	1.41	0.90	0.98	0.89	0.81	0.76	0.71	0.57	0.66	0.72	0.67	-
**Number**	122	122	120	112	99	93	85	70	56	43	32	22	10	7	2	0

Table B124-30 Age Group = 18-30 Gender = OVERALL Occupational group = MANUAL Total cases = 218

	Threshold dB HL≥															
	0	5	10	15	20	25	30	35	40	45	50	55	65	75	85	95
**Prevalence**	**85.1**	**52.7**	**28.7**	**10.5**	**6.4**	**2.5**	**1.1**	**0.9**	**0.5**	**0.1**	**0.0**	**0.0**	**0.0**	**0.0**	**0.0**	**0.0**
**Lower CI**	77.4	43.7	21.5	6.5	3.7	1.1	0.4	0.3	0.2	0.0	0.0	0.0	0.0	0.0	0.0	0.0
**Upper CI**	90.5	61.5	37.2	16.6	10.9	5.7	2.8	2.7	1.1	0.6	0.2	0.2	0.2	1.7	1.7	1.7
**Design Effect**	1.38	1.37	1.33	1.23	1.09	1.03	0.78	0.81	0.48	0.43	0.26	0.26	0.26	-	-	-
**Number**	191	126	73	33	20	10	7	6	5	2	1	1	1	0	0	0

Table B124-31 Age Group = 31-40 Gender = OVERALL Occupational group = MANUAL Total cases = 183

	Threshold dB HL≥															
	0	5	10	15	20	25	30	35	40	45	50	55	65	75	85	95
**Prevalence**	**88.9**	**61.7**	**32.1**	**20.0**	**9.3**	**5.6**	**3.4**	**2.9**	**2.6**	**1.5**	**1.5**	**1.4**	**1.1**	**1.1**	**0.9**	**0.9**
**Lower CI**	81.6	53.4	24.5	13.9	5.3	2.7	1.6	1.2	1.0	0.5	0.5	0.4	0.2	0.2	0.2	0.2
**Upper CI**	93.5	69.3	40.8	28.0	15.8	11.1	7.3	6.8	6.5	4.8	4.8	4.8	4.9	4.9	5.0	5.0
**Design Effect**	1.30	1.15	1.22	1.22	1.24	1.22	1.02	1.07	1.10	1.05	1.05	1.08	1.22	1.22	1.30	1.30
**Number**	168	126	78	48	26	17	12	9	8	6	6	5	2	2	1	1

Table B124-32 Age Group = 41-50 Gender = OVERALL Occupational group = MANUAL Total cases = 262

	Threshold dB HL≥															
	0	5	10	15	20	25	30	35	40	45	50	55	65	75	85	95
**Prevalence**	**97.5**	**80.4**	**59.5**	**33.3**	**21.6**	**13.5**	**9.5**	**5.6**	**3.7**	**2.2**	**1.6**	**1.4**	**0.4**	**0.4**	**0.2**	**0.2**
**Lower CI**	93.9	73.8	52.4	27.0	16.5	9.7	6.5	3.9	2.4	1.3	0.8	0.7	0.2	0.2	0.1	0.1
**Upper CI**	99.0	85.6	66.3	40.2	27.8	18.6	13.8	7.8	5.6	3.7	3.0	2.9	1.1	1.1	0.7	0.7
**Design Effect**	1.23	1.23	1.18	1.17	1.14	1.07	1.02	0.70	0.69	0.64	0.70	0.70	0.52	0.52	0.52	0.52
**Number**	257	220	168	108	79	54	41	32	22	16	11	10	4	4	2	2

Table B124-33 Age Group = 51-60 Gender = OVERALL Occupational group = MANUAL Total cases = 365

	Threshold dB HL≥															
	0	5	10	15	20	25	30	35	40	45	50	55	65	75	85	95
**Prevalence**	**100.0**	**93.1**	**77.0**	**52.6**	**36.9**	**28.0**	**20.0**	**12.7**	**7.8**	**6.2**	**4.5**	**3.6**	**1.7**	**0.4**	**0.1**	**0.1**
**Lower CI**	99.0	89.1	71.2	46.8	31.9	23.6	16.2	9.9	5.9	4.5	3.1	2.3	0.9	0.2	0.0	0.0
**Upper CI**	100.0	95.7	81.9	58.4	42.2	32.9	24.3	16.2	10.2	8.4	6.6	5.5	3.0	1.0	0.5	0.5
**Design Effect**	-	1.25	1.25	1.15	1.05	1.01	0.98	0.92	0.77	0.79	0.82	0.82	0.75	0.58	0.56	0.56
**Number**	365	348	308	243	189	151	112	78	59	47	35	29	15	5	1	1

Table B124-34 Age Group = 61-70 Gender = OVERALL Occupational group = MANUAL Total cases = 299

	Threshold dB HL≥															
	0	5	10	15	20	25	30	35	40	45	50	55	65	75	85	95
**Prevalence**	**99.4**	**97.4**	**92.0**	**76.3**	**57.8**	**45.9**	**31.6**	**24.7**	**16.2**	**11.8**	**8.2**	**6.1**	**3.4**	**2.4**	**1.4**	**1.1**
**Lower CI**	96.9	92.8	87.0	69.5	50.8	39.4	26.9	19.9	12.9	9.7	6.6	4.9	2.5	1.7	1.0	0.8
**Upper CI**	99.9	99.1	95.2	82.0	64.6	52.5	36.8	30.2	20.1	14.3	10.2	7.5	4.5	3.4	2.0	1.6
**Design Effect**	1.30	1.56	1.30	1.30	1.24	1.17	0.94	1.06	0.86	0.62	0.57	0.49	0.50	0.47	0.38	0.32
**Number**	298	295	282	249	215	185	143	121	93	78	56	42	21	14	6	3

Table B124-35 Age Group = 71-80 Gender = OVERALL Occupational group = MANUAL Total cases = 148

	Threshold dB HL≥															
	0	5	10	15	20	25	30	35	40	45	50	55	65	75	85	95
**Prevalence**	**100.0**	**100.0**	**95.3**	**90.4**	**81.0**	**60.7**	**57.3**	**46.8**	**35.5**	**24.0**	**16.9**	**13.7**	**7.0**	**3.6**	**1.7**	**0.2**
**Lower CI**	97.5	97.5	84.4	81.4	70.9	50.2	48.2	38.4	27.7	17.6	13.2	10.2	4.8	2.2	0.7	0.0
**Upper CI**	100.0	100.0	98.7	95.3	88.1	70.4	65.9	55.4	44.3	31.9	21.3	18.2	10.1	5.8	3.8	1.3
**Design Effect**	-	-	1.86	1.43	1.38	1.31	1.13	1.07	1.09	1.05	0.67	0.72	0.63	0.59	0.70	0.59
**Number**	148	148	145	142	136	121	118	99	82	66	53	43	24	13	6	1

Prevalence by age group, gender and occupational group for the better ear over 1, 2, and 4 kHz

Table B124-36 Age Group = 18-80 Gender = FEMALE Occupational group = NON-MANUAL Total cases = 670

	Threshold dB HL≥															
	0	5	10	15	20	25	30	35	40	45	50	55	65	75	85	95
Prevalence	**90.6**	**67.9**	**44.4**	**29.6**	**18.6**	**14.6**	**11.7**	**9.2**	**7.2**	**5.8**	**2.2**	**1.6**	**1.1**	**0.8**	**0.3**	**0.2**
Lower CI	87.6	64.2	41.3	26.8	16.3	12.9	10.2	8.0	6.3	5.1	1.7	1.2	0.7	0.5	0.1	0.1
Upper CI	92.9	71.4	47.6	32.5	21.2	16.5	13.4	10.5	8.1	6.7	2.9	2.2	1.7	1.4	0.6	0.5
Design Effect	1.19	1.02	0.85	0.82	0.82	0.69	0.65	0.57	0.46	0.47	0.53	0.53	0.60	0.66	0.62	0.60
Number	627	505	366	263	175	149	124	100	80	64	42	33	22	15	6	5

Table B124-37 Age Group = 18-80 Gender = MALE Occupational group = NON-MANUAL Total cases = 514

	Threshold dB HL≥															
	0	5	10	15	20	25	30	35	40	45	50	55	65	75	85	95
Prevalence	**88.4**	**66.4**	**47.5**	**30.6**	**24.4**	**19.3**	**13.2**	**8.4**	**6.4**	**4.0**	**3.2**	**1.9**	**0.6**	**0.3**	**0.1**	**0.1**
Lower CI	83.2	61.4	43.1	27.5	21.5	17.1	11.6	7.1	5.3	3.2	2.4	1.5	0.3	0.1	0.0	0.0
Upper CI	92.1	71.1	52.0	33.9	27.6	21.7	15.0	9.8	7.8	5.2	4.2	2.5	1.1	0.8	0.5	0.3
Design Effect	1.60	1.19	1.03	0.80	0.83	0.68	0.58	0.57	0.59	0.59	0.59	0.43	0.59	0.72	0.59	0.54
Number	486	411	319	241	193	160	119	85	65	44	37	26	9	3	2	1

Table B124-38 Age Group = 18-80 Gender = FEMALE Occupational group = MANUAL Total cases = 715

	Threshold dB HL≥															
	0	5	10	15	20	25	30	35	40	45	50	55	65	75	85	95
Prevalence	**94.9**	**74.2**	**52.0**	**33.8**	**23.3**	**16.2**	**11.3**	**8.7**	**6.4**	**4.5**	**3.2**	**2.6**	**1.4**	**0.9**	**0.6**	**0.3**
Lower CI	91.7	69.7	47.9	30.7	20.5	14.0	9.8	7.4	5.3	3.6	2.6	2.1	1.1	0.6	0.4	0.2
Upper CI	96.9	78.2	56.0	37.0	26.3	18.8	12.9	10.2	7.8	5.7	3.9	3.3	1.9	1.3	0.9	0.4
Design Effect	1.59	1.33	1.11	0.92	0.93	0.88	0.69	0.69	0.68	0.70	0.52	0.54	0.45	0.46	0.47	0.18
Number	693	590	459	339	256	204	158	127	103	79	59	50	28	16	8	3

Table B124-39 Age Group = 18-80 Gender = MALE Occupational group = MANUAL Total cases = 760

	Threshold dB HL≥															
	0	5	10	15	20	25	30	35	40	45	50	55	65	75	85	95
Prevalence	**92.2**	**78.3**	**63.1**	**45.5**	**34.2**	**25.0**	**19.7**	**14.2**	**9.4**	**6.7**	**5.0**	**3.9**	**2.0**	**1.1**	**0.5**	**0.5**
Lower CI	88.2	73.7	58.4	41.5	30.9	22.2	17.4	12.2	7.9	5.6	4.0	3.0	1.3	0.6	0.2	0.1
Upper CI	94.9	82.3	67.6	49.6	37.7	28.0	22.2	16.4	11.2	7.9	6.2	5.1	2.9	2.0	1.6	1.6
Design Effect	1.74	1.47	1.35	1.15	1.01	0.94	0.86	0.84	0.80	0.65	0.71	0.74	0.81	0.89	1.16	1.25
Number	734	673	595	484	409	334	275	218	166	136	103	80	39	22	8	5

Table B124-40 Age Group = 18-30 Gender = FEMALE Occupational group = NON-MANUAL Total cases = 115

	Threshold dB HL≥															
	0	5	10	15	20	25	30	35	40	45	50	55	65	75	85	95
Prevalence	**77.1**	**35.2**	**12.6**	**4.9**	**2.8**	**1.1**	**0.8**	**0.8**	**0.0**	**0.0**	**0.0**	**0.0**	**0.0**	**0.0**	**0.0**	**0.0**
Lower CI	68.0	26.7	7.9	2.6	1.2	0.3	0.1	0.1	0.0	0.0	0.0	0.0	0.0	0.0	0.0	0.0
Upper CI	84.1	44.7	19.5	9.2	6.1	4.5	4.5	4.5	3.2	3.2	3.2	3.2	3.2	3.2	3.2	3.2
Design Effect	1.06	1.05	0.96	0.81	0.77	0.89	0.98	0.98	-	-	-	-	-	-	-	-
Number	89	44	19	8	5	2	1	1	0	0	0	0	0	0	0	0

Table B124-41 Age Group = 31-40 Gender = FEMALE Occupational group = NON-MANUAL Total cases = 86

	Threshold dB HL≥															
	0	5	10	15	20	25	30	35	40	45	50	55	65	75	85	95
Prevalence	**84.7**	**54.9**	**20.3**	**6.8**	**2.5**	**2.5**	**1.3**	**0.7**	**0.7**	**0.7**	**0.0**	**0.0**	**0.0**	**0.0**	**0.0**	**0.0**
Lower CI	75.1	43.4	12.8	3.6	1.3	1.3	0.4	0.2	0.2	0.2	0.0	0.0	0.0	0.0	0.0	0.0
Upper CI	91.0	65.8	30.5	12.5	4.7	4.7	3.8	3.0	3.0	3.0	4.3	4.3	4.3	4.3	4.3	4.3
Design Effect	1.05	1.09	1.05	0.82	0.50	0.50	0.61	0.62	0.62	0.62	-	-	-	-	-	-
Number	74	49	21	10	5	5	3	2	2	2	0	0	0	0	0	0

Table B124-42 Age Group = 41-50 Gender = FEMALE Occupational group = NON-MANUAL Total cases = 112

	Threshold dB HL≥															
	0	5	10	15	20	25	30	35	40	45	50	55	65	75	85	95
Prevalence	**98.0**	**67.4**	**37.8**	**15.9**	**6.3**	**5.4**	**3.3**	**2.8**	**0.6**	**0.0**	**0.0**	**0.0**	**0.0**	**0.0**	**0.0**	**0.0**
Lower CI	92.7	57.7	29.7	11.0	4.3	3.9	2.1	1.8	0.1	0.0	0.0	0.0	0.0	0.0	0.0	0.0
Upper CI	99.4	75.9	46.6	22.5	9.3	7.3	5.1	4.5	4.3	3.3	3.3	3.3	3.3	3.3	3.3	3.3
Design Effect	1.09	1.07	0.96	0.85	0.55	0.40	0.45	0.43	0.00	-	-	-	-	-	-	-
Number	110	77	48	23	11	10	7	5	1	0	0	0	0	0	0	0

Prevalence by age group, gender and occupational group for the better ear over 1, 2, and 4 kHz

Table B124-43 Age Group = 51-60 Gender = FEMALE Occupational group = NON-MANUAL Total cases = 175

	Threshold dB HL≥															
	0	5	10	15	20	25	30	35	40	45	50	55	65	75	85	95
**Prevalence**	**98.7**	**90.1**	**62.4**	**37.5**	**15.7**	**11.8**	**6.9**	**5.1**	**3.8**	**3.5**	**2.5**	**2.1**	**1.2**	**1.0**	**0.3**	**0.3**
**Lower CI**	95.2	84.1	54.1	30.2	11.6	8.4	4.8	3.3	2.5	2.2	1.4	1.0	0.5	0.4	0.1	0.1
**Upper CI**	99.7	94.0	70.1	45.5	20.8	16.4	9.9	7.9	5.7	5.4	4.2	4.1	3.0	2.9	1.2	1.2
**Design Effect**	1.11	1.11	1.13	1.07	0.86	0.83	0.68	0.70	0.56	0.56	0.59	0.68	0.70	0.74	0.55	0.55
**Number**	173	158	117	80	43	34	25	19	15	13	10	8	5	4	2	2

Table B124-44 Age Group = 61-70 Gender = FEMALE Occupational group = NON-MANUAL Total cases = 115

	Threshold dB HL≥															
	0	5	10	15	20	25	30	35	40	45	50	55	65	75	85	95
**Prevalence**	**100.0**	**95.4**	**82.1**	**67.9**	**44.7**	**34.4**	**24.1**	**16.7**	**14.4**	**6.8**	**3.4**	**3.0**	**2.3**	**1.6**	**0.8**	**0.8**
**Lower CI**	96.8	88.0	72.9	57.7	35.5	26.4	17.7	12.5	11.1	4.9	2.1	1.7	1.1	0.7	0.2	0.2
**Upper CI**	100.0	98.3	88.7	76.7	54.3	43.5	32.0	21.9	18.5	9.3	5.4	5.1	4.5	3.8	3.0	3.0
**Design Effect**	0.00	1.25	1.14	1.14	1.05	1.00	0.92	0.69	0.57	0.48	0.49	0.53	0.59	0.63	0.70	0.70
**Number**	115	111	96	81	57	48	38	30	27	20	13	11	8	5	3	3

Table B124-45 Age Group = 71-80 Gender = FEMALE Occupational group = NON-MANUAL Total cases = 67

	Threshold dB HL≥															
	0	5	10	15	20	25	30	35	40	45	50	55	65	75	85	95
**Prevalence**	**98.6**	**98.6**	**95.8**	**86.3**	**74.3**	**61.6**	**61.6**	**50.9**	**41.8**	**39.1**	**12.4**	**7.8**	**5.3**	**4.0**	**0.6**	**0.0**
**Lower CI**	92.2	92.2	85.0	67.4	55.7	50.0	50.0	41.8	34.1	31.4	8.1	4.9	2.8	1.7	0.1	0.0
**Upper CI**	99.7	99.7	98.9	95.1	86.9	72.1	72.1	60.0	49.9	47.5	18.5	12.2	9.9	8.8	3.4	5.4
**Design Effect**	0.99	0.99	1.27	1.69	1.56	0.97	0.97	0.77	0.68	0.70	0.65	0.56	0.64	0.71	0.64	-
**Number**	66	66	65	61	54	50	50	43	35	29	19	14	9	6	1	0

Table B124-46 Age Group = 18-30 Gender = MALE Occupational group = NON-MANUAL Total cases = 66

	Threshold dB HL≥															
	0	5	10	15	20	25	30	35	40	45	50	55	65	75	85	95
**Prevalence**	**65.4**	**27.5**	**9.0**	**1.4**	**0.1**	**0.1**	**0.1**	**0.1**	**0.1**	**0.1**	**0.1**	**0.1**	**0.1**	**0.0**	**0.0**	**0.0**
**Lower CI**	50.4	17.2	3.6	0.3	0.0	0.0	0.0	0.0	0.0	0.0	0.0	0.0	0.0	0.0	0.0	0.0
**Upper CI**	77.9	41.0	20.8	6.7	5.7	5.7	5.7	5.7	5.7	5.7	5.7	5.7	5.7	5.5	5.5	5.5
**Design Effect**	1.24	1.14	1.21	0.88	0.00	0.00	0.00	0.00	0.00	0.00	0.00	0.00	0.00	-	-	-
**Number**	47	21	8	2	1	1	1	1	1	1	1	1	1	0	0	0

Table B124-47 Age Group = 31-40 Gender = MALE Occupational group = NON-MANUAL Total cases = 62

	Threshold dB HL≥															
	0	5	10	15	20	25	30	35	40	45	50	55	65	75	85	95
**Prevalence**	**94.8**	**57.7**	**32.1**	**11.3**	**7.1**	**1.3**	**1.3**	**0.0**	**0.0**	**0.0**	**0.0**	**0.0**	**0.0**	**0.0**	**0.0**	**0.0**
**Lower CI**	76.1	41.1	18.6	5.2	2.4	0.2	0.2	0.0	0.0	0.0	0.0	0.0	0.0	0.0	0.0	0.0
**Upper CI**	99.1	72.8	49.4	22.9	19.3	6.9	6.9	5.8	5.8	5.8	5.8	5.8	5.8	5.8	5.8	5.8
**Design Effect**	1.77	1.36	1.39	1.11	1.24	0.89	0.89	-	-	-	-	-	-	-	-	-
**Number**	60	41	19	10	4	1	1	0	0	0	0	0	0	0	0	0

Table B124-48 Age Group = 41-50 Gender = MALE Occupational group = NON-MANUAL Total cases = 76

	Threshold dB HL≥															
	0	5	10	15	20	25	30	35	40	45	50	55	65	75	85	95
**Prevalence**	**96.1**	**70.8**	**43.6**	**24.6**	**19.5**	**12.2**	**6.8**	**2.4**	**2.1**	**0.7**	**0.7**	**0.7**	**0.7**	**0.0**	**0.0**	**0.0**
**Lower CI**	90.2	59.1	32.0	15.4	11.4	6.9	3.1	1.5	1.3	0.2	0.2	0.2	0.2	0.0	0.0	0.0
**Upper CI**	98.5	80.3	55.9	37.0	31.5	20.6	14.0	3.7	3.5	2.8	2.8	2.8	2.8	4.8	4.8	4.8
**Design Effect**	0.88	1.06	1.11	1.14	1.14	0.92	0.93	0.32	0.33	0.56	0.56	0.56	0.56	-	-	-
**Number**	72	53	35	22	17	12	8	4	3	2	2	2	2	0	0	0

Table B124-49 Age Group = 51-60 Gender = MALE Occupational group = NON-MANUAL Total cases = 142

	Threshold dB HL≥															
	0	5	10	15	20	25	30	35	40	45	50	55	65	75	85	95
**Prevalence**	**98.0**	**91.3**	**69.0**	**44.0**	**34.5**	**24.9**	**17.1**	**5.4**	**4.1**	**2.2**	**2.0**	**1.2**	**0.0**	**0.0**	**0.0**	**0.0**
**Lower CI**	94.1	84.9	59.8	36.4	27.5	19.4	13.5	3.6	2.6	1.3	1.0	0.5	0.0	0.0	0.0	0.0
**Upper CI**	99.4	95.1	76.9	52.0	42.2	31.4	21.5	8.0	6.2	3.7	3.7	2.9	2.6	2.6	2.6	2.6
**Design Effect**	1.02	1.08	1.14	0.97	0.96	0.85	0.65	0.59	0.54	0.48	0.56	0.61	-	-	-	-
**Number**	139	129	100	71	55	40	29	18	14	9	8	5	0	0	0	0

Prevalence by age group, gender and occupational group for the better ear over 1, 2, and 4 kHz

Table B124-50 | Age Group = 61-70 | Gender = MALE | Occupational group = NON-MANUAL | Total cases = 112

Threshold dB HL≥	0	5	10	15	20	25	30	35	40	45	50	55	65	75	85	95
**Prevalence**	**100.0**	**99.8**	**93.4**	**71.6**	**59.6**	**53.2**	**32.4**	**25.8**	**19.6**	**14.0**	**9.1**	**6.0**	**2.8**	**1.6**	**0.4**	**0.4**
**Lower CI**	96.7	98.9	87.6	56.7	45.4	39.8	26.7	21.1	15.1	10.1	6.0	4.5	1.1	0.4	0.1	0.1
**Upper CI**	100.0	100.0	96.7	82.9	72.4	66.1	38.8	31.1	25.0	19.1	13.6	7.9	7.0	6.5	2.1	2.1
**Design Effect**	-	0.47	0.96	1.62	1.55	1.48	0.70	0.62	0.67	0.69	0.71	0.39	0.88	1.08	0.65	0.65
**Number**	112	111	102	85	71	63	45	35	26	18	13	10	5	2	1	1

Table B124-51 | Age Group = 71-80 | Gender = MALE | Occupational group = NON-MANUAL | Total cases = 56

Threshold dB HL≥	0	5	10	15	20	25	30	35	40	45	50	55	65	75	85	95
**Prevalence**	**100.0**	**100.0**	**97.9**	**88.8**	**75.6**	**70.6**	**55.8**	**42.3**	**32.8**	**20.5**	**18.7**	**11.1**	**1.0**	**1.0**	**1.0**	**0.0**
**Lower CI**	93.6	93.6	89.0	78.8	64.2	62.2	43.8	30.1	22.3	13.3	12.1	7.0	0.2	0.2	0.2	0.0
**Upper CI**	100.0	100.0	99.6	94.4	84.3	77.8	67.2	55.5	45.4	30.3	27.7	17.1	5.3	5.3	5.3	6.4
**Design Effect**	-	-	1.09	0.93	0.91	0.66	0.92	1.02	0.96	0.82	0.77	0.61	0.74	0.74	0.74	-
**Number**	56	56	55	51	45	43	35	27	21	14	13	8	1	1	1	0

Table B124-52 | Age Group = 18-30 | Gender = FEMALE | Occupational group = MANUAL | Total cases = 109

Threshold dB HL≥	0	5	10	15	20	25	30	35	40	45	50	55	65	75	85	95
**Prevalence**	**88.1**	**54.5**	**24.8**	**6.7**	**3.8**	**2.4**	**1.1**	**0.8**	**0.8**	**0.2**	**0.0**	**0.0**	**0.0**	**0.0**	**0.0**	**0.0**
**Lower CI**	77.1	42.0	16.0	3.3	1.7	0.9	0.5	0.3	0.3	0.0	0.0	0.0	0.0	0.0	0.0	0.0
**Upper CI**	94.2	66.5	36.2	13.1	8.4	6.2	2.5	2.1	2.1	1.1	3.4	3.4	3.4	3.4	3.4	3.4
**Design Effect**	1.40	1.35	1.27	1.00	0.88	0.83	0.49	0.48	0.48	0.47	-	-	-	-	-	-
**Number**	98	63	34	14	9	7	5	4	4	1	0	0	0	0	0	0

Table B124-53 | Age Group = 31-40 | Gender = FEMALE | Occupational group = MANUAL | Total cases = 103

Threshold dB HL≥	0	5	10	15	20	25	30	35	40	45	50	55	65	75	85	95
**Prevalence**	**91.3**	**52.6**	**24.3**	**15.2**	**5.8**	**4.2**	**3.3**	**3.0**	**2.4**	**0.8**	**0.8**	**0.8**	**0.3**	**0.3**	**0.0**	**0.0**
**Lower CI**	82.1	40.6	16.4	9.5	2.7	1.8	1.2	1.0	0.7	0.3	0.3	0.3	0.1	0.1	0.0	0.0
**Upper CI**	96.0	64.4	34.4	23.5	12.1	9.5	8.9	8.8	8.3	2.3	2.3	2.3	1.7	1.7	3.6	3.6
**Design Effect**	1.24	1.27	1.10	1.01	0.99	0.94	1.02	1.05	1.11	0.52	0.52	0.52	0.56	0.56	-	-
**Number**	96	67	38	24	11	9	6	5	4	3	3	3	1	1	0	0

Table B124-54 | Age Group = 41-50 | Gender = FEMALE | Occupational group = MANUAL | Total cases = 135

Threshold dB HL≥	0	5	10	15	20	25	30	35	40	45	50	55	65	75	85	95
**Prevalence**	**96.5**	**76.3**	**51.2**	**27.3**	**17.1**	**10.4**	**7.2**	**4.8**	**3.8**	**2.3**	**1.8**	**1.8**	**0.4**	**0.4**	**0.2**	**0.2**
**Lower CI**	90.7	66.9	41.7	19.7	11.4	6.4	4.2	3.0	2.2	1.1	0.7	0.7	0.1	0.1	0.0	0.0
**Upper CI**	98.7	83.7	60.6	36.7	24.9	16.4	11.9	7.7	6.6	5.0	4.4	4.4	1.3	1.3	1.0	1.0
**Design Effect**	1.17	1.19	1.14	1.15	1.06	0.96	0.87	0.64	0.65	0.72	0.76	0.76	0.49	0.49	0.48	0.48
**Number**	131	108	75	45	33	23	18	15	12	8	6	6	2	2	1	1

Table B124-55 | Age Group = 51-60 | Gender = FEMALE | Occupational group = MANUAL | Total cases = 166

Threshold dB HL≥	0	5	10	15	20	25	30	35	40	45	50	55	65	75	85	95
**Prevalence**	**100.0**	**89.7**	**69.4**	**42.1**	**24.5**	**18.2**	**12.5**	**6.3**	**3.5**	**2.1**	**1.5**	**1.0**	**0.7**	**0.3**	**0.2**	**0.2**
**Lower CI**	97.7	82.9	59.9	33.9	18.1	12.9	8.1	3.6	2.0	1.1	0.7	0.5	0.3	0.1	0.0	0.0
**Upper CI**	100.0	94.0	77.4	50.9	32.2	25.1	18.7	10.7	5.9	3.9	3.2	1.8	1.5	1.1	1.0	1.0
**Design Effect**	0.00	1.19	1.27	1.15	1.09	1.05	1.05	0.95	0.68	0.62	0.65	0.40	0.44	0.50	0.53	0.53
**Number**	166	153	128	93	62	48	33	21	16	11	8	7	5	2	1	1

Table B124-56 | Age Group = 61-70 | Gender = FEMALE | Occupational group = MANUAL | Total cases = 126

Threshold dB HL≥	0	5	10	15	20	25	30	35	40	45	50	55	65	75	85	95
**Prevalence**	**100.0**	**96.2**	**86.3**	**67.3**	**46.2**	**34.6**	**18.1**	**13.1**	**9.3**	**7.9**	**5.8**	**4.7**	**3.0**	**2.4**	**1.9**	**1.6**
**Lower CI**	97.0	88.2	77.6	57.1	36.6	26.4	15.2	8.9	7.1	5.8	4.3	3.4	2.2	1.8	1.4	0.5
**Upper CI**	100.0	98.9	91.9	76.1	56.1	43.9	21.4	18.9	11.9	10.7	7.7	6.4	4.0	3.2	2.4	5.7
**Design Effect**	0.00	1.42	1.19	1.18	1.15	1.07	0.46	0.84	0.47	0.51	0.42	0.40	0.29	0.26	0.21	0.00
**Number**	126	123	111	93	74	59	40	33	26	22	16	13	7	4	2	1

Prevalence by age group, gender and occupational group for the better ear over 1, 2, and 4 kHz

Table B124-57 Age Group = 71-80 Gender = FEMALE Occupational group = MANUAL Total cases = 76

Threshold dB HL≥	0	5	10	15	20	25	30	35	40	45	50	55	65	75	85	95
**Prevalence**	**100.0**	**100.0**	**92.0**	**83.7**	**78.6**	**54.6**	**49.3**	**45.3**	**34.1**	**24.3**	**15.7**	**12.6**	**6.8**	**3.5**	**2.1**	**0.0**
**Lower CI**	95.2	95.2	74.7	69.4	61.6	41.0	38.5	34.8	24.1	15.0	11.4	8.5	4.3	1.7	0.8	0.0
**Upper CI**	100.0	100.0	97.8	92.1	89.3	67.6	60.1	56.3	45.8	36.8	21.2	18.2	10.7	7.0	5.8	4.8
**Design Effect**	-	-	1.77	1.39	1.55	1.23	0.98	0.99	1.04	1.15	0.60	0.65	0.56	0.60	0.70	-
**Number**	76	76	73	70	67	58	56	49	41	34	26	21	13	7	4	0

Table B124-58 Age Group = 18-30 Gender = MALE Occupational group = MANUAL Total cases = 109

Threshold dB HL≥	0	5	10	15	20	25	30	35	40	45	50	55	65	75	85	95
**Prevalence**	**82.1**	**50.9**	**33.0**	**14.4**	**8.9**	**2.5**	**1.0**	**1.0**	**0.1**	**0.1**	**0.1**	**0.1**	**0.1**	**0.0**	**0.0**	**0.0**
**Lower CI**	70.3	38.2	22.1	7.6	4.4	0.7	0.2	0.2	0.0	0.0	0.0	0.0	0.0	0.0	0.0	0.0
**Upper CI**	89.9	63.5	46.1	25.7	17.2	8.7	5.1	5.1	0.4	0.4	0.4	0.4	0.4	3.4	3.4	3.4
**Design Effect**	1.38	1.39	1.40	1.37	1.17	1.17	0.99	0.99	0.27	0.27	0.27	0.27	0.27	-	-	-
**Number**	93	63	39	19	11	3	2	2	1	1	1	1	1	0	0	0

Table B124-59 Age Group = 31-40 Gender = MALE Occupational group = MANUAL Total cases = 80

Threshold dB HL≥	0	5	10	15	20	25	30	35	40	45	50	55	65	75	85	95
**Prevalence**	**86.4**	**71.6**	**40.8**	**25.4**	**13.1**	**7.0**	**3.6**	**2.7**	**2.7**	**2.3**	**2.3**	**2.1**	**1.9**	**1.9**	**1.9**	**1.9**
**Lower CI**	73.7	60.2	27.8	15.0	6.2	2.6	1.2	0.7	0.7	0.5	0.5	0.4	0.3	0.3	0.3	0.3
**Upper CI**	93.5	80.7	55.4	39.8	25.6	17.9	10.3	9.8	9.8	9.8	9.8	9.9	10.2	10.2	10.2	10.2
**Design Effect**	1.32	1.06	1.33	1.34	1.31	1.29	1.01	1.09	1.09	1.15	1.15	1.19	1.25	1.25	1.25	1.25
**Number**	72	59	40	24	15	8	6	4	4	3	3	2	1	1	1	1

Table B124-60 Age Group = 41-50 Gender = MALE Occupational group = MANUAL Total cases = 127

Threshold dB HL≥	0	5	10	15	20	25	30	35	40	45	50	55	65	75	85	95
**Prevalence**	**98.5**	**84.7**	**68.3**	**39.7**	**26.5**	**16.9**	**12.1**	**6.4**	**3.5**	**2.1**	**1.4**	**1.1**	**0.5**	**0.5**	**0.2**	**0.2**
**Lower CI**	92.1	74.8	57.4	30.1	18.4	10.7	7.1	3.9	1.8	1.1	0.6	0.4	0.1	0.1	0.0	0.0
**Upper CI**	99.7	91.2	77.5	50.2	36.6	25.7	19.9	10.4	6.9	3.8	3.3	2.9	1.7	1.7	1.3	1.3
**Design Effect**	1.37	1.32	1.26	1.20	1.21	1.15	1.13	0.75	0.75	0.53	0.62	0.62	0.54	0.54	0.54	0.54
**Number**	126	112	93	63	46	31	23	17	10	8	5	4	2	2	1	1

Table B124-61 Age Group = 51-60 Gender = MALE Occupational group = MANUAL Total cases = 199

Threshold dB HL≥	0	5	10	15	20	25	30	35	40	45	50	55	65	75	85	95
**Prevalence**	**100.0**	**96.9**	**85.5**	**64.2**	**50.8**	**39.0**	**28.4**	**19.9**	**12.6**	**10.7**	**7.9**	**6.6**	**2.7**	**0.5**	**0.0**	**0.0**
**Lower CI**	98.1	91.8	78.8	56.0	43.2	32.1	22.7	15.1	9.1	7.5	5.1	4.0	1.3	0.2	0.0	0.0
**Upper CI**	100.0	98.9	90.3	71.6	58.3	46.2	35.0	25.8	17.1	15.1	12.1	10.6	5.5	1.7	1.9	1.9
**Design Effect**	0.00	1.34	1.18	1.19	1.10	1.05	0.99	0.97	0.86	0.89	0.92	0.94	0.87	0.64	-	-
**Number**	199	195	180	150	127	103	79	57	43	36	27	22	10	3	0	0

Table B124-62 Age Group = 61-70 Gender = MALE Occupational group = MANUAL Total cases = 173

Threshold dB HL≥	0	5	10	15	20	25	30	35	40	45	50	55	65	75	85	95
**Prevalence**	**98.8**	**98.8**	**98.5**	**86.9**	**71.6**	**58.8**	**47.5**	**38.5**	**24.5**	**16.6**	**11.4**	**7.9**	**4.0**	**2.6**	**0.9**	**0.6**
**Lower CI**	93.3	93.3	93.5	78.1	61.2	49.0	37.6	29.5	18.0	12.9	8.4	5.8	2.4	1.4	0.3	0.1
**Upper CI**	99.8	99.8	99.7	92.5	80.1	68.1	57.6	48.4	32.4	21.2	15.3	10.6	6.5	4.8	2.4	2.2
**Design Effect**	1.47	1.47	1.35	1.43	1.43	1.32	1.37	1.33	1.14	0.75	0.73	0.59	0.70	0.68	0.66	0.74
**Number**	172	172	171	156	141	126	103	88	67	56	40	29	14	10	4	2

Table B124-63 Age Group = 71-80 Gender = MALE Occupational group = MANUAL Total cases = 72

Threshold dB HL≥	0	5	10	15	20	25	30	35	40	45	50	55	65	75	85	95
**Prevalence**	**100.0**	**100.0**	**100.0**	**100.0**	**84.8**	**69.8**	**69.1**	**48.8**	**37.8**	**23.5**	**18.7**	**15.5**	**7.3**	**3.6**	**1.0**	**0.6**
**Lower CI**	94.9	94.9	94.9	94.9	79.2	53.2	52.6	35.6	26.1	17.4	12.8	9.9	3.9	1.9	0.3	0.1
**Upper CI**	100.0	100.0	100.0	100.0	89.1	82.4	81.9	62.2	51.1	30.9	26.6	23.4	13.1	6.9	3.5	3.1
**Design Effect**	-	-	-	-	0.60	1.44	1.43	1.19	1.15	0.70	0.77	0.81	0.75	0.56	0.60	0.63
**Number**	72	72	72	72	69	63	62	50	41	32	27	22	11	6	2	1

Prevalence by age group, gender and occupational group for the better ear over 4, 6, and 8 kHz

Table B468-1 Age Group = 18-80 Gender = OVERALL Occupational group = OVERALL Total cases = 2658

Threshold dB HL≥	0	5	10	15	20	25	30	35	40	45	50	55	65	75	85	95
**Prevalence**	**97.5**	**91.2**	**77.3**	**63.0**	**50.1**	**40.5**	**34.5**	**28.3**	**24.5**	**20.8**	**17.2**	**14.2**	**8.7**	**4.1**	**2.0**	**0.9**
**Lower CI**	96.4	89.5	75.3	61.0	48.3	38.9	33.0	27.0	23.2	19.5	16.1	13.1	7.9	3.5	1.6	0.7
**Upper CI**	98.3	92.6	79.3	64.9	51.8	42.1	35.9	29.7	25.8	22.1	18.4	15.3	9.6	4.7	2.4	1.2
**Design Effect**	1.55	1.46	1.26	1.06	0.93	0.86	0.81	0.78	0.81	0.83	0.78	0.81	0.78	0.78	0.82	0.77
**Number**	2622	2528	2299	2002	1723	1490	1307	1130	993	874	768	667	444	240	124	62

Table B468-2 Age Group = 18-80 Gender = FEMALE Occupational group = OVERALL Total cases = 1385

Threshold dB HL≥	0	5	10	15	20	25	30	35	40	45	50	55	65	75	85	95
**Prevalence**	**97.8**	**90.8**	**75.5**	**59.0**	**45.2**	**35.6**	**29.6**	**23.9**	**19.7**	**16.1**	**13.4**	**10.5**	**5.9**	**2.6**	**1.2**	**0.5**
**Lower CI**	96.4	88.6	72.8	56.6	43.0	33.6	27.7	22.2	17.9	14.4	11.9	9.1	5.0	2.1	0.9	0.3
**Upper CI**	98.6	92.6	78.0	61.4	47.4	37.7	31.5	25.8	21.5	17.9	14.9	12.0	6.9	3.2	1.7	0.8
**Design Effect**	1.35	1.31	1.16	0.93	0.83	0.82	0.80	0.80	0.87	0.89	0.82	0.89	0.79	0.69	0.63	0.65
**Number**	1365	1303	1153	963	795	660	557	466	390	329	283	241	150	84	45	19

Table B468-3 Age Group = 18-80 Gender = MALE Occupational group = OVERALL Total cases = 1273

Threshold dB HL≥	0	5	10	15	20	25	30	35	40	45	50	55	65	75	85	95
**Prevalence**	**97.2**	**91.6**	**79.4**	**67.5**	**55.6**	**45.9**	**40.0**	**33.2**	**29.8**	**26.1**	**21.5**	**18.3**	**11.9**	**5.7**	**2.8**	**1.4**
**Lower CI**	95.2	88.8	76.2	64.3	52.7	43.4	37.7	31.3	28.0	24.2	19.8	16.7	10.6	4.8	2.1	1.0
**Upper CI**	98.4	93.8	82.3	70.6	58.4	48.3	42.2	35.3	31.8	28.0	23.3	19.9	13.4	6.8	3.7	2.0
**Design Effect**	1.73	1.63	1.38	1.22	1.04	0.91	0.83	0.77	0.76	0.79	0.76	0.75	0.78	0.82	0.90	0.82
**Number**	1257	1225	1146	1039	928	830	750	664	603	545	485	426	294	156	79	43

Table B468-4 Age Group = 18-30 Gender = OVERALL Occupational group = OVERALL Total cases = 401

Threshold dB HL≥	0	5	10	15	20	25	30	35	40	45	50	55	65	75	85	95
**Prevalence**	**93.0**	**76.0**	**47.7**	**25.2**	**12.5**	**5.8**	**3.4**	**1.3**	**0.9**	**0.8**	**0.7**	**0.4**	**0.3**	**0.3**	**0.3**	**0.0**
**Lower CI**	89.4	70.6	41.9	20.6	9.3	3.7	1.8	0.7	0.4	0.3	0.2	0.1	0.1	0.1	0.1	0.0
**Upper CI**	95.4	80.7	53.4	30.3	16.7	9.0	6.2	2.6	2.2	2.1	2.1	1.5	1.5	1.6	1.6	0.1
**Design Effect**	1.20	1.21	1.18	1.14	1.14	1.13	1.18	0.81	0.89	0.97	1.01	0.95	1.01	1.04	1.04	0.28
**Number**	375	315	213	120	65	34	20	13	8	6	5	4	3	2	2	1

Table B468-5 Age Group = 31-40 Gender = OVERALL Occupational group = OVERALL Total cases = 331

Threshold dB HL≥	0	5	10	15	20	25	30	35	40	45	50	55	65	75	85	95
**Prevalence**	**96.5**	**89.6**	**67.6**	**45.9**	**26.9**	**16.2**	**10.2**	**7.2**	**5.4**	**4.4**	**2.5**	**2.1**	**1.3**	**0.8**	**0.6**	**0.6**
**Lower CI**	92.9	84.6	61.1	39.4	21.7	12.2	7.1	4.6	3.3	2.4	1.3	1.0	0.5	0.2	0.1	0.1
**Upper CI**	98.3	93.1	73.5	52.4	32.8	21.2	14.4	11.1	8.9	7.8	4.7	4.2	3.5	2.9	3.0	3.1
**Design Effect**	1.30	1.29	1.24	1.22	1.17	1.13	1.12	1.16	1.13	1.19	0.96	1.00	1.11	1.20	1.31	1.36
**Number**	321	302	241	169	111	78	49	34	27	20	15	12	7	4	2	1

Table B468-6 Age Group = 41-50 Gender = OVERALL Occupational group = OVERALL Total cases = 450

Threshold dB HL≥	0	5	10	15	20	25	30	35	40	45	50	55	65	75	85	95
**Prevalence**	**100.0**	**96.8**	**86.3**	**66.6**	**48.2**	**32.7**	**24.5**	**16.8**	**13.7**	**10.0**	**7.2**	**4.9**	**3.2**	**1.6**	**0.6**	**0.3**
**Lower CI**	99.2	94.5	82.3	61.8	43.4	28.3	20.7	13.7	10.9	7.7	5.4	3.4	2.0	0.8	0.3	0.1
**Upper CI**	100.0	98.2	89.4	71.1	53.1	37.4	28.7	20.4	17.0	13.0	9.7	6.9	5.0	3.1	1.3	0.8
**Design Effect**	-	1.10	1.11	1.08	1.06	1.04	1.01	0.98	0.96	0.94	0.91	0.88	0.91	0.94	0.61	0.65
**Number**	450	437	396	314	240	177	144	108	89	68	54	40	26	15	8	3

Table B468-7 Age Group = 51-60 Gender = OVERALL Occupational group = OVERALL Total cases = 680

Threshold dB HL≥	0	5	10	15	20	25	30	35	40	45	50	55	65	75	85	95
**Prevalence**	**100.0**	**99.7**	**95.3**	**87.7**	**73.9**	**59.9**	**47.8**	**36.3**	**29.1**	**23.2**	**18.6**	**15.0**	**7.2**	**4.0**	**2.2**	**1.5**
**Lower CI**	99.4	98.7	93.0	84.5	70.0	55.8	43.8	32.7	25.9	20.3	16.1	12.7	5.8	2.9	1.4	0.9
**Upper CI**	100.0	99.9	96.8	90.3	77.5	63.9	51.8	40.1	32.5	26.4	21.4	17.6	9.0	5.5	3.3	2.5
**Design Effect**	0.00	1.17	1.19	1.17	1.14	1.10	1.08	1.03	0.97	0.95	0.90	0.90	0.83	0.86	0.81	0.89
**Number**	680	678	653	612	543	467	390	321	264	220	186	154	86	47	28	17

Prevalence by age group, gender and occupational group for the better ear over 4, 6, and 8 kHz

Table B468-8 Age Group = 61-70 Gender = OVERALL Occupational group = OVERALL Total cases = 526

Threshold dB HL≥	0	5	10	15	20	25	30	35	40	45	50	55	65	75	85	95
Prevalence	**100.0**	**100.0**	**100.0**	**97.7**	**91.5**	**85.9**	**80.6**	**69.9**	**61.7**	**52.5**	**42.6**	**34.9**	**20.6**	**8.9**	**3.8**	**2.0**
Lower CI	99.3	99.3	99.3	94.8	87.7	81.7	76.1	65.0	56.7	47.7	38.2	30.7	17.6	7.3	2.9	1.3
Upper CI	100.0	100.0	100.0	99.0	94.2	89.3	84.3	74.4	66.4	57.4	47.2	39.4	23.9	10.9	5.0	3.1
Design Effect	-	-	-	1.55	1.36	1.28	1.22	1.20	1.17	1.14	1.06	1.08	0.91	0.73	0.65	0.71
Number	526	526	526	519	498	473	446	405	365	331	290	251	171	91	45	23

Table B468-9 Age Group = 71-80 Gender = OVERALL Occupational group = OVERALL Total cases = 270

Threshold dB HL≥	0	5	10	15	20	25	30	35	40	45	50	55	65	75	85	95
Prevalence	**100.0**	**100.0**	**100.0**	**99.5**	**98.1**	**93.1**	**91.1**	**86.7**	**81.3**	**75.3**	**68.7**	**59.8**	**42.3**	**17.9**	**8.1**	**2.6**
Lower CI	98.6	98.6	98.6	98.1	94.6	85.7	83.8	79.3	72.8	66.9	61.0	52.1	35.9	14.0	5.5	1.6
Upper CI	100.0	100.0	100.0	99.9	99.4	96.8	95.3	91.7	87.6	82.1	75.5	67.1	48.9	22.6	11.9	4.1
Design Effect	-	-	-	0.89	1.32	1.76	1.66	1.53	1.61	1.49	1.32	1.30	1.11	0.94	0.97	0.65
Number	270	270	270	268	266	261	258	249	240	229	218	206	151	81	39	17

Table B468-10 Age Group = 18-80 Gender = OVERALL Occupational group = NON-MANUAL Total cases = 1183

Threshold dB HL≥	0	5	10	15	20	25	30	35	40	45	50	55	65	75	85	95
Prevalence	**97.8**	**90.0**	**74.0**	**58.4**	**45.3**	**36.3**	**31.7**	**25.8**	**22.4**	**19.1**	**15.3**	**13.0**	**7.3**	**2.7**	**1.4**	**0.6**
Lower CI	96.1	87.4	71.2	55.5	42.8	34.3	29.8	24.0	20.5	17.3	14.1	11.8	6.3	2.1	1.0	0.4
Upper CI	98.8	92.1	76.7	61.2	47.8	38.5	33.7	27.8	24.4	21.0	16.7	14.4	8.5	3.4	1.8	0.8
Design Effect	1.53	1.35	1.11	1.02	0.90	0.76	0.75	0.77	0.82	0.83	0.63	0.67	0.74	0.69	0.59	0.55
Number	1167	1112	990	830	691	590	521	439	383	329	282	243	155	69	42	17

Table B468-11 Age Group = 18-80 Gender = OVERALL Occupational group = MANUAL Total cases = 1471

Threshold dB HL≥	0	5	10	15	20	25	30	35	40	45	50	55	65	75	85	95
Prevalence	**97.0**	**92.1**	**80.2**	**67.2**	**54.5**	**43.6**	**36.5**	**30.1**	**25.9**	**22.2**	**18.2**	**14.9**	**9.2**	**4.7**	**2.3**	**1.2**
Lower CI	94.9	89.6	77.0	64.2	51.6	41.2	34.3	28.2	24.2	20.5	16.7	13.5	8.1	4.0	1.7	0.9
Upper CI	98.2	94.1	83.0	70.1	57.3	46.0	38.7	32.0	27.8	24.0	19.8	16.3	10.4	5.6	3.0	1.7
Design Effect	1.85	1.66	1.48	1.23	1.11	0.94	0.88	0.80	0.80	0.83	0.79	0.78	0.78	0.75	0.83	0.73
Number	1451	1412	1306	1170	1030	898	784	689	608	543	484	423	289	171	82	45

Table B468-12 Age Group = 18-30 Gender = FEMALE Occupational group = OVERALL Total cases = 224

Threshold dB HL≥	0	5	10	15	20	25	30	35	40	45	50	55	65	75	85	95
Prevalence	**93.7**	**76.7**	**48.6**	**20.7**	**11.2**	**4.5**	**2.6**	**1.6**	**1.2**	**0.8**	**0.7**	**0.1**	**0.0**	**0.0**	**0.0**	**0.0**
Lower CI	89.1	69.8	41.2	15.5	7.5	2.6	1.3	0.7	0.5	0.2	0.1	0.0	0.0	0.0	0.0	0.0
Upper CI	96.5	82.4	56.0	27.0	16.4	7.6	5.3	3.6	3.2	3.0	3.0	0.6	1.7	1.7	1.7	1.7
Design Effect	1.14	1.14	1.15	1.09	1.08	0.90	0.91	0.80	0.86	0.98	1.05	0.47	-	-	-	-
Number	211	175	117	58	35	18	11	8	5	3	2	1	0	0	0	0

Table B468-13 Age Group = 31-40 Gender = FEMALE Occupational group = OVERALL Total cases = 189

Threshold dB HL≥	0	5	10	15	20	25	30	35	40	45	50	55	65	75	85	95
Prevalence	**96.5**	**87.4**	**59.9**	**38.1**	**16.8**	**11.7**	**5.6**	**4.8**	**4.1**	**3.1**	**1.5**	**1.5**	**1.2**	**0.2**	**0.0**	**0.0**
Lower CI	92.6	80.4	51.4	30.6	12.1	7.9	3.4	2.7	2.2	1.4	0.6	0.6	0.4	0.0	0.0	0.0
Upper CI	98.4	92.2	67.8	46.3	22.8	16.9	9.3	8.4	7.7	6.7	4.0	4.0	3.8	1.2	2.0	2.0
Design Effect	1.05	1.24	1.19	1.15	1.01	0.98	0.88	0.90	0.94	1.00	0.88	0.88	0.95	0.63	-	-
Number	182	170	128	86	50	36	19	16	13	9	6	6	4	1	0	0

Table B468-14 Age Group = 41-50 Gender = FEMALE Occupational group = OVERALL Total cases = 247

Threshold dB HL≥	0	5	10	15	20	25	30	35	40	45	50	55	65	75	85	95
Prevalence	**100.0**	**94.2**	**79.5**	**56.8**	**37.4**	**23.3**	**17.8**	**10.6**	**7.9**	**5.1**	**4.6**	**2.9**	**1.2**	**0.4**	**0.3**	**0.1**
Lower CI	98.5	89.9	73.2	50.0	31.3	18.5	13.6	7.7	5.6	3.4	2.9	1.7	0.5	0.1	0.1	0.0
Upper CI	100.0	96.8	84.6	63.3	44.0	29.0	22.9	14.4	11.1	7.5	7.2	4.9	2.8	1.2	1.0	0.6
Design Effect	-	1.15	1.13	1.09	1.06	1.00	0.98	0.87	0.82	0.74	0.81	0.76	0.78	0.59	0.60	0.51
Number	247	235	203	150	107	75	61	43	34	24	21	14	6	3	2	1

Prevalence by age group, gender and occupational group for the better ear over 4, 6, and 8 kHz

Table B468-15 Age Group = 51-60 Gender = FEMALE Occupational group = OVERALL Total cases = 340

Threshold dB HL≥	0	5	10	15	20	25	30	35	40	45	50	55	65	75	85	95
**Prevalence**	**100.0**	**99.5**	**93.2**	**83.2**	**65.8**	**49.0**	**35.4**	**23.4**	**15.5**	**10.8**	**7.2**	**6.1**	**3.1**	**2.2**	**1.0**	**0.7**
**Lower CI**	98.9	97.7	89.3	78.1	59.8	43.2	30.0	19.0	12.2	8.1	5.2	4.2	2.0	1.3	0.5	0.3
**Upper CI**	100.0	99.9	95.7	87.3	71.3	54.8	41.1	28.4	19.6	14.3	9.9	8.7	4.8	3.7	2.1	1.8
**Design Effect**	-	1.12	1.17	1.17	1.14	1.10	1.10	1.05	0.97	0.93	0.85	0.87	0.74	0.74	0.68	0.75
**Number**	340	338	320	292	247	197	152	114	83	64	47	38	24	17	9	6

Table B468-16 Age Group = 61-70 Gender = FEMALE Occupational group = OVERALL Total cases = 243

Threshold dB HL≥	0	5	10	15	20	25	30	35	40	45	50	55	65	75	85	95
**Prevalence**	**100.0**	**100.0**	**100.0**	**97.1**	**86.0**	**77.6**	**69.7**	**58.5**	**48.4**	**39.0**	**32.1**	**24.9**	**10.6**	**5.2**	**2.7**	**1.0**
**Lower CI**	98.4	98.4	98.4	93.2	79.9	70.7	62.7	51.3	41.5	32.9	26.5	19.5	7.8	3.6	1.7	0.4
**Upper CI**	100.0	100.0	100.0	98.8	90.5	83.2	76.0	65.4	55.4	45.6	38.2	31.3	14.3	7.6	4.2	2.4
**Design Effect**	0.00	0.00	0.00	1.24	1.22	1.20	1.16	1.15	1.12	1.05	1.00	1.09	0.84	0.71	0.60	0.71
**Number**	243	243	243	237	217	199	180	157	133	114	98	81	47	29	16	5

Table B468-17 Age Group = 71-80 Gender = FEMALE Occupational group = OVERALL Total cases = 142

Threshold dB HL≥	0	5	10	15	20	25	30	35	40	45	50	55	65	75	85	95
**Prevalence**	**100.0**	**100.0**	**100.0**	**99.2**	**97.7**	**90.4**	**88.9**	**84.1**	**77.3**	**70.3**	**63.9**	**50.8**	**33.6**	**12.4**	**5.5**	**1.9**
**Lower CI**	97.4	97.4	97.4	96.9	91.8	78.5	77.1	72.5	64.1	57.6	53.3	40.4	26.1	8.7	3.5	0.9
**Upper CI**	100.0	100.0	100.0	99.8	99.4	96.1	95.0	91.4	86.6	80.5	73.4	61.2	42.0	17.4	8.8	4.0
**Design Effect**	0.00	0.00	0.00	0.83	1.30	1.76	1.70	1.59	1.67	1.56	1.30	1.29	1.04	0.80	0.70	0.66
**Number**	142	142	142	140	139	135	134	128	122	115	109	101	69	34	18	7

Table B468-18 Age Group = 18-30 Gender = MALE Occupational group = OVERALL Total cases = 177

Threshold dB HL≥	0	5	10	15	20	25	30	35	40	45	50	55	65	75	85	95
**Prevalence**	**92.2**	**75.3**	**46.8**	**29.8**	**13.8**	**7.1**	**4.2**	**1.0**	**0.6**	**0.6**	**0.6**	**0.6**	**0.6**	**0.6**	**0.6**	**0.0**
**Lower CI**	85.9	66.6	38.1	22.6	8.9	3.7	1.7	0.3	0.1	0.1	0.1	0.1	0.1	0.1	0.1	0.0
**Upper CI**	95.8	82.4	55.6	38.1	20.8	13.3	9.9	3.1	3.0	3.0	3.0	3.0	3.0	3.1	3.1	0.2
**Design Effect**	1.23	1.26	1.20	1.17	1.17	1.23	1.30	0.83	0.94	0.94	0.94	0.94	0.94	0.97	0.97	0.27
**Number**	164	140	96	62	30	16	9	5	3	3	3	3	3	2	2	1

Table B468-19 Age Group = 31-40 Gender = MALE Occupational group = OVERALL Total cases = 142

Threshold dB HL≥	0	5	10	15	20	25	30	35	40	45	50	55	65	75	85	95
**Prevalence**	**96.6**	**91.9**	**76.1**	**54.4**	**38.1**	**21.3**	**15.1**	**9.7**	**6.7**	**5.8**	**3.6**	**2.6**	**1.4**	**1.4**	**1.3**	**1.2**
**Lower CI**	88.9	83.4	65.6	43.7	28.6	14.4	9.4	5.2	3.2	2.6	1.6	1.0	0.3	0.3	0.3	0.2
**Upper CI**	99.0	96.3	84.2	64.7	48.6	30.4	23.2	17.3	13.5	12.6	7.9	7.0	6.2	6.2	6.4	6.5
**Design Effect**	1.49	1.39	1.35	1.31	1.27	1.20	1.17	1.22	1.21	1.25	0.96	1.03	1.22	1.22	1.26	1.31
**Number**	139	132	113	83	61	42	30	18	14	11	9	6	3	3	2	1

Table B468-20 Age Group = 41-50 Gender = MALE Occupational group = OVERALL Total cases = 203

Threshold dB HL≥	0	5	10	15	20	25	30	35	40	45	50	55	65	75	85	95
**Prevalence**	**100.0**	**99.7**	**93.7**	**77.4**	**60.1**	**43.0**	**31.8**	**23.6**	**20.0**	**15.4**	**10.2**	**7.0**	**5.3**	**2.9**	**1.0**	**0.4**
**Lower CI**	98.1	98.1	88.3	70.2	52.5	35.7	25.6	18.1	15.0	11.1	6.9	4.4	3.1	1.3	0.5	0.1
**Upper CI**	100.0	99.9	96.7	83.2	67.2	50.6	38.9	30.2	26.2	21.1	14.8	11.0	9.0	6.1	2.2	1.6
**Design Effect**	-	0.83	1.21	1.14	1.11	1.10	1.05	1.04	1.03	1.01	0.95	0.92	0.93	0.98	0.60	0.67
**Number**	203	202	193	164	133	102	83	65	55	44	33	26	20	12	6	2

Table B468-21 Age Group = 51-60 Gender = MALE Occupational group = OVERALL Total cases = 340

Threshold dB HL≥	0	5	10	15	20	25	30	35	40	45	50	55	65	75	85	95
**Prevalence**	**100.0**	**100.0**	**97.6**	**92.6**	**82.9**	**72.0**	**61.7**	**50.8**	**44.3**	**37.1**	**31.3**	**25.0**	**11.8**	**6.0**	**3.4**	**2.3**
**Lower CI**	98.9	98.9	94.8	88.6	77.7	66.2	55.7	45.0	38.7	31.9	26.6	20.8	9.1	4.0	2.1	1.2
**Upper CI**	100.0	100.0	98.9	95.3	87.1	77.2	67.3	56.6	50.0	42.6	36.5	29.7	15.2	8.8	5.6	4.3
**Design Effect**	0.00	0.00	1.20	1.20	1.18	1.16	1.13	1.10	1.08	1.05	1.00	0.98	0.90	0.94	0.88	0.95
**Number**	340	340	333	320	296	270	238	207	181	156	139	116	62	30	19	11

Prevalence by age group, gender and occupational group for the better ear over 4, 6, and 8 kHz

Table B468-22 Age Group = 61-70 Gender = MALE Occupational group = OVERALL Total cases = 283

Threshold dB HL≥	0	5	10	15	20	25	30	35	40	45	50	55	65	75	85	95
**Prevalence**	**100.0**	**100.0**	**100.0**	**98.4**	**97.7**	**95.5**	**93.0**	**83.1**	**77.0**	**68.2**	**54.8**	**46.6**	**32.5**	**13.3**	**5.2**	**3.2**
**Lower CI**	98.7	98.7	98.7	91.5	91.3	89.9	87.4	76.5	69.7	60.3	47.9	40.2	27.1	10.5	3.7	2.0
**Upper CI**	100.0	100.0	100.0	99.7	99.4	98.1	96.2	88.2	82.9	75.1	61.6	53.1	38.4	16.7	7.3	5.1
**Design Effect**	-	-	-	2.14	1.95	1.60	1.44	1.35	1.36	1.38	1.19	1.12	1.04	0.79	0.70	0.75
**Number**	283	283	283	282	281	274	266	248	232	217	192	170	124	62	29	18

Table B468-23 Age Group = 71-80 Gender = MALE Occupational group = OVERALL Total cases = 128

Threshold dB HL≥	0	5	10	15	20	25	30	35	40	45	50	55	65	75	85	95
**Prevalence**	**100.0**	**100.0**	**100.0**	**100.0**	**98.7**	**96.7**	**93.8**	**89.8**	**86.8**	**82.1**	**75.6**	**73.2**	**54.6**	**26.5**	**12.1**	**3.7**
**Lower CI**	97.1	97.1	97.1	97.1	92.7	88.4	85.3	81.2	77.8	72.9	64.4	61.9	44.0	18.8	6.8	2.1
**Upper CI**	100.0	100.0	100.0	100.0	99.8	99.1	97.6	94.7	92.5	88.7	84.1	82.1	64.8	35.8	20.7	6.6
**Design Effect**	-	-	-	-	1.32	1.50	1.39	1.26	1.26	1.19	1.35	1.34	1.23	1.13	1.23	0.67
**Number**	128	128	128	128	127	126	124	121	118	114	109	105	82	47	21	10

Table B468-24 Age Group = 18-30 Gender = OVERALL Occupational group = NON-MANUAL Total cases = 181

Threshold dB HL≥	0	5	10	15	20	25	30	35	40	45	50	55	65	75	85	95
**Prevalence**	**93.7**	**71.3**	**38.3**	**18.6**	**7.4**	**1.8**	**0.9**	**0.9**	**0.8**	**0.5**	**0.5**	**0.1**	**0.1**	**0.0**	**0.0**	**0.0**
**Lower CI**	87.8	62.9	31.1	13.1	4.0	0.8	0.4	0.4	0.3	0.1	0.1	0.0	0.0	0.0	0.0	0.0
**Upper CI**	96.9	78.5	46.1	25.8	13.3	4.0	2.3	2.3	2.2	2.3	2.3	2.2	2.2	2.1	2.1	2.1
**Design Effect**	1.24	1.20	1.08	1.13	1.19	0.75	0.64	0.64	0.66	0.83	0.83	0.00	0.00	-	-	-
**Number**	170	135	82	41	17	7	4	4	3	2	2	1	1	0	0	0

Table B468-25 Age Group = 31-40 Gender = OVERALL Occupational group = NON-MANUAL Total cases = 148

Threshold dB HL≥	0	5	10	15	20	25	30	35	40	45	50	55	65	75	85	95
**Prevalence**	**97.2**	**90.8**	**65.6**	**39.8**	**20.6**	**14.2**	**8.6**	**6.5**	**5.2**	**4.1**	**1.7**	**1.4**	**0.7**	**0.0**	**0.0**	**0.0**
**Lower CI**	93.5	85.3	56.3	30.6	13.8	8.6	4.2	2.9	2.1	1.3	0.6	0.4	0.2	0.0	0.0	0.0
**Upper CI**	98.8	94.3	73.8	49.8	29.7	22.5	16.7	14.0	12.5	12.1	4.6	4.6	3.5	2.5	2.5	2.5
**Design Effect**	0.94	0.95	1.16	1.25	1.23	1.24	1.35	1.34	1.37	1.51	0.87	0.95	0.93	-	-	-
**Number**	143	133	101	62	35	24	15	11	9	6	4	3	2	0	0	0

Table B468-26 Age Group = 41-50 Gender = OVERALL Occupational group = NON-MANUAL Total cases = 188

Threshold dB HL≥	0	5	10	15	20	25	30	35	40	45	50	55	65	75	85	95
**Prevalence**	**100.0**	**95.8**	**82.6**	**61.8**	**41.4**	**25.1**	**20.1**	**12.6**	**9.6**	**6.5**	**3.8**	**3.7**	**2.8**	**0.6**	**0.4**	**0.0**
**Lower CI**	98.0	92.2	76.0	54.6	34.3	20.2	15.6	8.9	6.3	4.0	2.7	2.6	2.3	0.3	0.1	0.0
**Upper CI**	100.0	97.8	87.7	68.6	48.9	30.7	25.4	17.7	14.4	10.6	5.3	5.2	3.5	1.3	1.3	2.0
**Design Effect**	-	0.95	1.09	1.02	1.04	0.85	0.86	0.93	0.95	0.91	0.47	0.46	0.26	0.41	0.60	-
**Number**	188	179	156	115	79	54	45	31	25	17	12	11	8	4	2	0

Table B468-27 Age Group = 51-60 Gender = OVERALL Occupational group = NON-MANUAL Total cases = 317

Threshold dB HL≥	0	5	10	15	20	25	30	35	40	45	50	55	65	75	85	95
**Prevalence**	**100.0**	**99.9**	**95.7**	**83.6**	**68.8**	**54.3**	**43.0**	**31.3**	**24.4**	**19.3**	**15.7**	**11.4**	**5.1**	**1.7**	**1.1**	**0.8**
**Lower CI**	98.8	99.3	92.6	78.3	62.9	48.5	37.4	26.5	20.2	15.6	12.7	7.8	2.7	1.0	0.5	0.3
**Upper CI**	100.0	100.0	97.5	87.7	74.3	60.1	48.8	36.6	29.1	23.6	19.3	16.3	9.6	2.8	2.1	1.8
**Design Effect**	0.00	0.62	1.06	1.16	1.13	1.06	1.05	0.99	0.95	0.93	0.83	1.20	1.38	0.64	0.65	0.70
**Number**	317	316	302	269	229	191	152	120	95	75	64	51	25	12	8	6

Table B468-28 Age Group = 61-70 Gender = OVERALL Occupational group = NON-MANUAL Total cases = 228

Threshold dB HL≥	0	5	10	15	20	25	30	35	40	45	50	55	65	75	85	95
**Prevalence**	**100.0**	**100.0**	**100.0**	**96.3**	**89.6**	**83.3**	**79.6**	**66.1**	**59.3**	**50.1**	**38.7**	**30.9**	**19.5**	**8.6**	**4.0**	**1.9**
**Lower CI**	98.3	98.3	98.3	87.2	81.3	74.9	71.3	57.6	51.1	42.5	33.1	26.3	16.0	6.2	3.0	0.9
**Upper CI**	100.0	100.0	100.0	99.0	94.5	89.2	86.1	73.6	67.1	57.7	44.7	35.9	23.4	11.8	5.3	3.8
**Design Effect**	-	-	-	2.10	1.64	1.48	1.42	1.32	1.27	1.18	0.92	0.80	0.72	0.76	0.44	0.77
**Number**	228	228	228	224	213	199	192	166	149	132	110	90	63	31	19	8

Prevalence by age group, gender and occupational group for the better ear over 4, 6, and 8 kHz

Table B468-29 Age Group = 71-80 Gender = OVERALL Occupational group = NON-MANUAL Total cases = 121

	Threshold dB HL≥															
	0	5	10	15	20	25	30	35	40	45	50	55	65	75	85	95
**Prevalence**	**100.0**	**100.0**	**100.0**	**98.9**	**98.1**	**93.2**	**91.3**	**86.2**	**80.1**	**75.8**	**68.4**	**65.3**	**35.3**	**11.7**	**6.5**	**2.0**
**Lower CI**	96.9	96.9	96.9	95.2	94.0	84.1	82.4	77.5	67.7	63.6	59.6	56.1	27.1	7.6	3.8	1.2
**Upper CI**	100.0	100.0	100.0	99.8	99.4	97.3	95.9	91.9	88.5	84.8	76.1	73.5	44.4	17.6	10.8	3.3
**Design Effect**	0.00	0.00	0.00	0.97	0.98	1.39	1.31	1.17	1.49	1.42	1.01	1.04	1.03	0.86	0.78	0.41
**Number**	121	121	121	119	118	115	113	107	102	97	90	87	56	22	13	3

Table B468-30 Age Group = 18-30 Gender = OVERALL Occupational group = MANUAL Total cases = 218

	Threshold dB HL≥															
	0	5	10	15	20	25	30	35	40	45	50	55	65	75	85	95
**Prevalence**	**91.1**	**79.8**	**56.3**	**31.9**	**17.8**	**8.5**	**5.3**	**1.3**	**0.9**	**0.7**	**0.6**	**0.6**	**0.5**	**0.5**	**0.5**	**0.0**
**Lower CI**	84.0	71.9	47.3	24.5	12.1	4.9	2.6	0.6	0.3	0.2	0.1	0.1	0.1	0.1	0.1	0.0
**Upper CI**	95.2	86.0	65.0	40.4	25.5	14.3	10.7	3.0	2.7	2.6	2.6	2.6	2.6	2.6	2.6	0.2
**Design Effect**	1.46	1.34	1.37	1.30	1.33	1.26	1.32	0.74	0.83	0.87	0.93	0.93	0.99	0.99	0.99	0.26
**Number**	203	178	130	79	48	27	16	9	5	4	3	3	2	2	2	1

Table B468-31 Age Group = 31-40 Gender = OVERALL Occupational group = MANUAL Total cases = 183

	Threshold dB HL≥															
	0	5	10	15	20	25	30	35	40	45	50	55	65	75	85	95
**Prevalence**	**96.4**	**88.4**	**67.2**	**50.7**	**33.7**	**18.6**	**11.3**	**7.8**	**5.6**	**4.6**	**2.9**	**2.2**	**1.4**	**1.3**	**1.0**	**0.9**
**Lower CI**	90.4	79.9	57.5	41.5	25.7	13.1	7.1	4.5	3.2	2.4	1.4	0.9	0.4	0.3	0.2	0.2
**Upper CI**	98.7	93.6	75.6	60.0	42.8	25.7	17.6	13.2	9.5	8.7	5.8	5.4	4.8	4.8	4.9	5.0
**Design Effect**	1.40	1.47	1.36	1.30	1.27	1.12	1.13	1.11	0.92	1.00	0.86	0.96	1.08	1.14	1.24	1.30
**Number**	178	169	140	107	76	54	34	23	18	14	11	9	5	4	2	1

Table B468-32 Age Group = 41-50 Gender = OVERALL Occupational group = MANUAL Total cases = 262

	Threshold dB HL≥															
	0	5	10	15	20	25	30	35	40	45	50	55	65	75	85	95
**Prevalence**	**100.0**	**98.2**	**90.7**	**71.7**	**55.9**	**39.2**	**27.7**	**20.0**	**16.5**	**13.1**	**9.4**	**5.4**	**3.6**	**2.2**	**0.6**	**0.3**
**Lower CI**	98.6	95.1	85.9	64.8	48.7	32.7	22.2	15.4	12.3	9.4	6.5	3.4	1.9	0.9	0.3	0.1
**Upper CI**	100.0	99.3	93.9	77.7	62.8	46.2	33.9	25.5	21.6	18.1	13.5	8.5	6.8	5.3	1.4	0.9
**Design Effect**	-	1.17	1.13	1.19	1.18	1.15	1.09	1.05	1.04	1.07	0.99	0.91	1.04	1.17	0.54	0.52
**Number**	262	258	240	199	161	123	99	77	64	51	42	29	18	11	6	3

Table B468-33 Age Group = 51-60 Gender = OVERALL Occupational group = MANUAL Total cases = 363

	Threshold dB HL≥															
	0	5	10	15	20	25	30	35	40	45	50	55	65	75	85	95
**Prevalence**	**100.0**	**99.6**	**95.6**	**92.3**	**78.3**	**64.9**	**53.0**	**41.4**	**33.7**	**26.8**	**21.0**	**17.6**	**8.6**	**4.9**	**2.5**	**1.7**
**Lower CI**	99.0	98.0	92.1	88.0	72.4	58.7	47.0	36.0	28.9	22.6	17.3	14.1	6.4	3.3	1.6	0.9
**Upper CI**	100.0	99.9	97.6	95.2	83.2	70.7	58.9	47.0	39.0	31.4	25.2	21.7	11.5	7.1	4.0	3.1
**Design Effect**	-	1.15	1.26	1.29	1.28	1.24	1.17	1.09	1.05	0.97	0.94	0.97	0.87	0.85	0.73	0.81
**Number**	363	362	351	343	314	276	238	201	169	145	122	103	61	35	20	11

Table B468-34 Age Group = 61-70 Gender = OVERALL Occupational group = MANUAL Total cases = 297

	Threshold dB HL≥															
	0	5	10	15	20	25	30	35	40	45	50	55	65	75	85	95
**Prevalence**	**100.0**	**100.0**	**100.0**	**98.3**	**92.1**	**87.0**	**80.1**	**71.6**	**62.2**	**54.2**	**44.8**	**38.7**	**20.9**	**9.6**	**3.9**	**2.5**
**Lower CI**	98.7	98.7	98.7	95.0	86.7	80.9	73.9	65.1	55.7	47.4	38.8	32.7	16.7	7.6	2.9	1.8
**Upper CI**	100.0	100.0	100.0	99.5	95.5	91.4	85.1	77.4	68.3	60.9	51.0	45.0	25.8	12.0	5.3	3.5
**Design Effect**	0.00	0.00	0.00	1.35	1.40	1.36	1.23	1.21	1.15	1.21	1.08	1.12	0.98	0.67	0.54	0.48
**Number**	297	297	297	294	284	273	253	238	215	198	179	161	108	60	26	15

Table B468-35 Age Group = 71-80 Gender = OVERALL Occupational group = MANUAL Total cases = 148

	Threshold dB HL≥															
	0	5	10	15	20	25	30	35	40	45	50	55	65	75	85	95
**Prevalence**	**100.0**	**100.0**	**100.0**	**100.0**	**98.0**	**94.8**	**92.8**	**89.2**	**85.0**	**77.8**	**69.1**	**55.4**	**43.2**	**21.0**	**9.7**	**3.7**
**Lower CI**	97.5	97.5	97.5	97.5	89.5	84.1	82.8	76.6	71.4	65.5	58.9	47.1	34.6	15.6	5.6	2.2
**Upper CI**	100.0	100.0	100.0	100.0	99.7	98.4	97.2	95.4	92.8	86.6	77.8	63.4	52.1	27.7	16.4	6.3
**Design Effect**	-	-	-	-	1.73	1.82	1.65	1.85	1.87	1.61	1.29	1.03	1.11	0.92	1.13	0.66
**Number**	148	148	148	148	147	145	144	141	137	131	127	118	95	59	26	14

Prevalence by age group, gender and occupational group for the better ear over 4, 6, and 8 kHz

Table B468-36 Age Group = 18-80 Gender = FEMALE Occupational group = NON-MANUAL Total cases = 670

	Threshold dB HL≥															
	**0**	**5**	**10**	**15**	**20**	**25**	**30**	**35**	**40**	**45**	**50**	**55**	**65**	**75**	**85**	**95**
**Prevalence**	**97.6**	**89.6**	**73.2**	**56.2**	**41.5**	**32.7**	**28.6**	**23.2**	**19.4**	**15.7**	**13.1**	**11.6**	**5.5**	**1.9**	**0.9**	**0.3**
**Lower CI**	95.8	86.5	69.7	53.0	38.8	30.2	26.3	21.1	17.1	13.5	11.5	10.0	4.2	1.3	0.6	0.2
**Upper CI**	98.6	92.0	76.5	59.4	44.2	35.2	31.1	25.5	22.0	18.1	14.9	13.3	7.1	2.8	1.4	0.7
**Design Effect**	1.18	1.19	1.03	0.86	0.73	0.70	0.69	0.69	0.82	0.83	0.68	0.70	0.83	0.70	0.50	0.59
**Number**	658	621	541	438	349	287	252	210	180	146	123	107	68	34	21	8

Table B468-37 Age Group = 18-80 Gender = MALE Occupational group = NON-MANUAL Total cases = 513

	Threshold dB HL≥															
	**0**	**5**	**10**	**15**	**20**	**25**	**30**	**35**	**40**	**45**	**50**	**55**	**65**	**75**	**85**	**95**
**Prevalence**	**98.1**	**90.5**	**74.9**	**60.8**	**49.5**	**40.4**	**35.1**	**28.7**	**25.6**	**22.8**	**17.8**	**14.7**	**9.2**	**3.5**	**1.8**	**0.8**
**Lower CI**	94.4	86.0	70.3	55.9	45.1	37.0	31.9	25.6	22.6	19.9	15.9	12.8	7.7	2.6	1.2	0.5
**Upper CI**	99.4	93.6	79.1	65.6	54.0	43.9	38.4	32.1	28.8	25.9	19.8	16.7	11.0	4.7	2.7	1.3
**Design Effect**	1.85	1.49	1.17	1.15	1.03	0.82	0.79	0.83	0.82	0.83	0.58	0.63	0.66	0.66	0.61	0.52
**Number**	509	491	449	392	342	303	269	229	203	183	159	136	87	35	21	9

Table B468-38 Age Group = 18-80 Gender = FEMALE Occupational group = MANUAL Total cases = 714

	Threshold dB HL≥															
	**0**	**5**	**10**	**15**	**20**	**25**	**30**	**35**	**40**	**45**	**50**	**55**	**65**	**75**	**85**	**95**
**Prevalence**	**97.7**	**92.0**	**78.4**	**62.3**	**49.1**	**38.3**	**30.1**	**23.8**	**19.3**	**15.8**	**12.8**	**9.2**	**5.0**	**2.8**	**1.4**	**0.7**
**Lower CI**	94.7	88.4	74.1	58.4	45.3	35.2	27.3	21.2	16.8	13.5	10.9	7.6	3.9	2.2	1.0	0.5
**Upper CI**	99.0	94.5	82.2	66.0	52.9	41.6	33.0	26.6	22.2	18.5	15.1	11.1	6.2	3.5	2.0	1.1
**Design Effect**	1.81	1.53	1.35	1.07	1.05	0.91	0.86	0.86	0.93	0.93	0.85	0.83	0.72	0.56	0.56	0.51
**Number**	706	681	611	524	445	372	304	255	209	182	159	134	82	50	24	11

Table B468-39 Age Group = 18-80 Gender = MALE Occupational group = MANUAL Total cases = 757

	Threshold dB HL≥															
	**0**	**5**	**10**	**15**	**20**	**25**	**30**	**35**	**40**	**45**	**50**	**55**	**65**	**75**	**85**	**95**
**Prevalence**	**96.2**	**92.3**	**82.2**	**72.8**	**60.5**	**49.4**	**43.7**	**37.1**	**33.3**	**29.2**	**24.2**	**21.2**	**13.9**	**6.9**	**3.3**	**1.7**
**Lower CI**	92.6	88.1	77.3	68.0	56.3	45.9	40.4	34.6	31.0	26.8	21.9	19.1	12.0	5.5	2.2	1.1
**Upper CI**	98.0	95.1	86.2	77.0	64.6	53.0	47.1	39.7	35.7	31.8	26.6	23.6	16.1	8.7	4.8	2.7
**Design Effect**	1.90	1.81	1.63	1.43	1.20	1.00	0.94	0.74	0.70	0.77	0.76	0.76	0.84	0.86	0.98	0.83
**Number**	745	731	695	646	585	526	480	434	399	361	325	289	207	121	58	34

Table B468-40 Age Group = 18-30 Gender = FEMALE Occupational group = NON-MANUAL Total cases = 115

	Threshold dB HL≥															
	**0**	**5**	**10**	**15**	**20**	**25**	**30**	**35**	**40**	**45**	**50**	**55**	**65**	**75**	**85**	**95**
**Prevalence**	**93.5**	**74.3**	**43.5**	**18.1**	**7.9**	**3.0**	**1.7**	**1.7**	**1.4**	**0.9**	**0.9**	**0.0**	**0.0**	**0.0**	**0.0**	**0.0**
**Lower CI**	87.5	64.9	34.3	12.3	4.3	1.3	0.6	0.6	0.4	0.2	0.2	0.0	0.0	0.0	0.0	0.0
**Upper CI**	96.7	81.9	53.2	25.7	13.9	7.1	4.5	4.5	4.3	4.7	4.7	3.2	3.2	3.2	3.2	3.2
**Design Effect**	0.99	1.07	1.07	0.96	0.95	0.86	0.75	0.75	0.78	1.00	1.00	-	-	-	-	-
**Number**	107	87	55	26	12	5	3	3	2	1	1	0	0	0	0	0

Table B468-41 Age Group = 31-40 Gender = FEMALE Occupational group = NON-MANUAL Total cases = 86

	Threshold dB HL≥															
	**0**	**5**	**10**	**15**	**20**	**25**	**30**	**35**	**40**	**45**	**50**	**55**	**65**	**75**	**85**	**95**
**Prevalence**	**96.0**	**85.5**	**56.3**	**34.4**	**9.7**	**5.4**	**3.4**	**3.4**	**3.4**	**1.4**	**1.4**	**1.4**	**1.4**	**0.0**	**0.0**	**0.0**
**Lower CI**	90.0	75.8	45.5	24.5	5.6	2.9	1.5	1.5	1.5	0.3	0.3	0.3	0.3	0.0	0.0	0.0
**Upper CI**	98.5	91.7	66.6	45.8	16.3	9.7	7.5	7.5	7.5	6.5	6.5	6.5	6.5	4.3	4.3	4.3
**Design Effect**	0.96	1.07	1.03	1.09	0.85	0.70	0.75	0.75	0.75	0.98	0.98	0.98	0.98	-	-	-
**Number**	82	74	51	32	13	8	5	5	5	2	2	2	2	0	0	0

Table B468-42 Age Group = 41-50 Gender = FEMALE Occupational group = NON-MANUAL Total cases = 112

	Threshold dB HL≥															
	**0**	**5**	**10**	**15**	**20**	**25**	**30**	**35**	**40**	**45**	**50**	**55**	**65**	**75**	**85**	**95**
**Prevalence**	**100.0**	**92.5**	**76.7**	**52.3**	**31.4**	**18.1**	**15.7**	**8.2**	**6.4**	**3.3**	**2.1**	**1.8**	**1.3**	**0.0**	**0.0**	**0.0**
**Lower CI**	96.7	85.8	67.2	42.8	23.5	12.9	10.6	5.7	4.0	2.1	1.1	0.9	0.8	0.0	0.0	0.0
**Upper CI**	100.0	96.2	84.1	61.6	40.5	24.9	22.6	11.9	10.1	5.1	3.9	3.5	2.4	3.3	3.3	3.3
**Design Effect**	-	1.04	1.09	1.04	1.01	0.85	0.90	0.61	0.67	0.44	0.50	0.49	0.37	-	-	-
**Number**	112	104	88	60	39	26	23	15	12	7	4	3	2	0	0	0

Prevalence by age group, gender and occupational group for the better ear over 4, 6, and 8 kHz

Table B468-43 Age Group = 51-60 Gender = FEMALE Occupational group = NON-MANUAL Total cases = 175

	Threshold dB HL≥															
	0	5	10	15	20	25	30	35	40	45	50	55	65	75	85	95
Prevalence	**100.0**	**99.8**	**94.5**	**79.9**	**62.9**	**45.2**	**32.8**	**21.4**	**14.7**	**10.5**	**7.0**	**5.7**	**3.4**	**2.1**	**1.3**	**1.0**
Lower CI	97.9	98.7	89.6	72.2	54.4	37.4	25.8	15.9	10.5	7.0	4.5	3.5	1.9	1.1	0.6	0.4
Upper CI	100.0	100.0	97.1	85.9	70.6	53.3	40.7	28.1	20.2	15.6	10.6	9.3	6.1	4.0	3.0	2.9
Design Effect	-	0.64	1.07	1.16	1.14	1.09	1.09	1.01	0.92	0.94	0.81	0.83	0.76	0.64	0.66	0.74
Number	175	174	165	143	118	92	70	53	41	30	22	17	11	8	5	4

Table B468-44 Age Group = 61-70 Gender = FEMALE Occupational group = NON-MANUAL Total cases = 116

	Threshold dB HL≥															
	0	5	10	15	20	25	30	35	40	45	50	55	65	75	85	95
Prevalence	**100.0**	**100.0**	**100.0**	**97.8**	**86.5**	**78.1**	**73.0**	**59.9**	**50.7**	**37.9**	**31.9**	**26.2**	**11.4**	**5.9**	**2.7**	**0.9**
Lower CI	96.8	96.8	96.8	92.1	77.0	67.9	62.7	49.7	41.0	30.1	24.5	19.3	7.0	3.3	1.6	0.3
Upper CI	100.0	100.0	100.0	99.4	92.5	85.8	81.3	69.4	60.4	46.4	40.5	34.5	18.0	10.5	4.7	2.9
Design Effect	0.00	0.00	0.00	1.15	1.24	1.21	1.17	1.13	1.09	0.94	0.96	0.96	0.94	0.82	0.51	0.65
Number	116	116	116	113	103	94	89	75	65	53	45	37	23	16	10	3

Table B468-45 Age Group = 71-80 Gender = FEMALE Occupational group = NON-MANUAL Total cases = 66

	Threshold dB HL≥															
	0	5	10	15	20	25	30	35	40	45	50	55	65	75	85	95
Prevalence	**100.0**	**100.0**	**100.0**	**98.2**	**98.2**	**91.9**	**91.9**	**87.5**	**78.9**	**75.6**	**67.5**	**64.0**	**29.8**	**6.8**	**3.0**	**0.3**
Lower CI	94.5	94.5	94.5	92.4	92.4	77.8	77.8	73.8	59.4	56.4	55.3	51.2	19.5	3.3	1.5	0.1
Upper CI	100.0	100.0	100.0	99.6	99.6	97.4	97.4	94.6	90.5	88.1	77.8	75.0	42.5	13.4	5.8	1.7
Design Effect	-	-	-	0.91	0.91	1.41	1.41	1.30	1.64	1.61	1.02	1.06	1.07	0.81	0.50	0.44
Number	66	66	66	64	64	62	62	59	55	53	49	48	30	10	6	1

Table B468-46 Age Group = 18-30 Gender = MALE Occupational group = NON-MANUAL Total cases = 66

	Threshold dB HL≥															
	0	5	10	15	20	25	30	35	40	45	50	55	65	75	85	95
Prevalence	**94.0**	**68.4**	**32.9**	**19.0**	**6.7**	**0.5**	**0.1**	**0.1**	**0.1**	**0.1**	**0.1**	**0.1**	**0.1**	**0.0**	**0.0**	**0.0**
Lower CI	81.7	54.1	22.5	10.5	2.2	0.1	0.0	0.0	0.0	0.0	0.0	0.0	0.0	0.0	0.0	0.0
Upper CI	98.2	79.9	45.4	32.0	18.8	2.1	5.7	5.7	5.7	5.7	5.7	5.7	5.7	5.5	5.5	5.5
Design Effect	1.32	1.19	1.04	1.15	1.29	0.45	0.00	0.00	0.00	0.00	0.00	0.00	0.00	-	-	-
Number	63	48	27	15	5	2	1	1	1	1	1	1	1	0	0	0

Table B468-47 Age Group = 31-40 Gender = MALE Occupational group = NON-MANUAL Total cases = 62

	Threshold dB HL≥															
	0	5	10	15	20	25	30	35	40	45	50	55	65	75	85	95
Prevalence	**98.5**	**96.6**	**76.2**	**46.2**	**33.0**	**24.3**	**14.4**	**9.9**	**7.3**	**7.3**	**1.9**	**1.3**	**0.0**	**0.0**	**0.0**	**0.0**
Lower CI	92.1	91.9	59.5	30.5	19.5	13.0	5.9	3.4	1.9	1.9	0.6	0.2	0.0	0.0	0.0	0.0
Upper CI	99.7	98.6	87.5	62.7	50.1	40.9	31.0	25.9	23.8	23.8	6.3	6.9	5.8	5.8	5.8	5.8
Design Effect	0.96	0.70	1.37	1.37	1.37	1.36	1.44	1.46	1.56	1.56	0.73	0.89	-	-	-	-
Number	61	59	50	30	22	16	10	6	4	4	2	1	0	0	0	0

Table B468-48 Age Group = 41-50 Gender = MALE Occupational group = NON-MANUAL Total cases = 76

	Threshold dB HL≥															
	0	5	10	15	20	25	30	35	40	45	50	55	65	75	85	95
Prevalence	**100.0**	**99.5**	**89.2**	**72.3**	**52.6**	**32.6**	**24.8**	**17.4**	**13.1**	**10.0**	**5.7**	**5.7**	**4.4**	**1.2**	**0.7**	**0.0**
Lower CI	95.2	97.2	78.5	60.8	40.4	24.6	17.9	10.5	7.2	5.2	3.8	3.8	3.6	0.6	0.2	0.0
Upper CI	100.0	99.9	95.0	81.4	64.4	41.8	33.4	27.6	22.7	18.5	8.3	8.3	5.5	2.6	2.8	4.8
Design Effect	0.00	0.62	1.16	1.04	1.10	0.83	0.81	1.01	1.02	0.97	0.43	0.43	0.21	0.38	0.56	-
Number	76	75	68	55	40	28	22	16	13	10	8	8	6	4	2	0

Table B468-49 Age Group = 51-60 Gender = MALE Occupational group = NON-MANUAL Total cases = 142

	Threshold dB HL≥															
	0	5	10	15	20	25	30	35	40	45	50	55	65	75	85	95
Prevalence	**100.0**	**100.0**	**97.0**	**87.6**	**75.4**	**64.5**	**54.5**	**42.5**	**35.3**	**29.0**	**25.5**	**17.8**	**7.1**	**1.1**	**0.8**	**0.5**
Lower CI	97.4	97.4	92.4	79.9	66.7	55.8	45.8	34.6	28.0	22.5	19.9	11.0	2.8	0.4	0.2	0.1
Upper CI	100.0	100.0	98.9	92.6	82.5	72.3	62.9	50.8	43.3	36.5	32.1	27.6	16.9	2.9	2.3	1.9
Design Effect	-	-	1.06	1.17	1.12	1.06	1.06	1.01	0.99	0.95	0.85	1.33	1.60	0.65	0.63	0.64
Number	142	142	137	126	111	99	82	67	54	45	42	34	14	4	3	2

Prevalence by age group, gender and occupational group for the better ear over 4, 6, and 8 kHz

Table B468-50 Age Group = 61-70 Gender = MALE Occupational group = NON-MANUAL Total cases = 112

Threshold dB HL≥	0	5	10	15	20	25	30	35	40	45	50	55	65	75	85	95
**Prevalence**	**100.0**	**100.0**	**100.0**	**94.4**	**93.1**	**89.1**	**87.0**	**73.3**	**69.2**	**64.3**	**46.5**	**36.2**	**28.5**	**11.7**	**5.6**	**3.1**
**Lower CI**	96.7	96.7	96.7	73.5	73.7	72.2	70.5	58.3	54.5	49.8	38.3	30.7	23.6	8.0	4.1	1.3
**Upper CI**	100.0	100.0	100.0	99.1	98.5	96.3	95.0	84.4	80.7	76.6	54.9	42.2	34.0	16.7	7.5	7.1
**Design Effect**	-	-	-	2.57	2.39	2.04	1.96	1.64	1.59	1.56	0.91	0.65	0.63	0.72	0.40	0.84
**Number**	112	112	112	111	110	105	103	91	84	79	65	53	40	15	9	5

Table B468-51 Age Group = 71-80 Gender = MALE Occupational group = NON-MANUAL Total cases = 55

Threshold dB HL≥	0	5	10	15	20	25	30	35	40	45	50	55	65	75	85	95
**Prevalence**	**100.0**	**100.0**	**100.0**	**100.0**	**97.9**	**95.0**	**89.8**	**83.2**	**81.2**	**75.2**	**69.5**	**67.4**	**42.3**	**19.0**	**11.7**	**4.9**
**Lower CI**	93.5	93.5	93.5	93.5	89.0	82.9	77.5	71.9	69.9	64.5	57.0	54.7	30.6	11.2	6.0	2.9
**Upper CI**	100.0	100.0	100.0	100.0	99.6	98.7	95.8	90.6	88.9	83.4	79.7	78.0	54.8	30.3	21.6	8.1
**Design Effect**	-	-	-	-	1.08	1.22	1.12	0.95	0.93	0.84	0.96	0.97	0.96	0.93	0.90	0.44
**Number**	55	55	55	55	54	53	51	48	47	44	41	39	26	12	7	2

Table B468-52 Age Group = 18-30 Gender = FEMALE Occupational group = MANUAL Total cases = 109

Threshold dB HL≥	0	5	10	15	20	25	30	35	40	45	50	55	65	75	85	95
**Prevalence**	**92.6**	**79.3**	**56.7**	**25.5**	**17.1**	**5.6**	**3.4**	**1.1**	**0.7**	**0.5**	**0.2**	**0.2**	**0.0**	**0.0**	**0.0**	**0.0**
**Lower CI**	81.5	68.0	44.1	16.7	9.7	2.7	1.3	0.5	0.2	0.1	0.0	0.0	0.0	0.0	0.0	0.0
**Upper CI**	97.2	87.3	68.4	36.9	28.3	11.3	8.7	2.5	2.1	1.7	1.1	1.1	3.4	3.4	3.4	3.4
**Design Effect**	1.52	1.29	1.34	1.26	1.33	0.97	1.00	0.49	0.52	0.51	0.47	0.47	-	-	-	-
**Number**	104	88	62	32	23	13	8	5	3	2	1	1	0	0	0	0

Table B468-53 Age Group = 31-40 Gender = FEMALE Occupational group = MANUAL Total cases = 103

Threshold dB HL≥	0	5	10	15	20	25	30	35	40	45	50	55	65	75	85	95
**Prevalence**	**97.7**	**88.9**	**61.5**	**41.5**	**24.0**	**17.6**	**6.9**	**5.6**	**4.6**	**4.4**	**1.1**	**1.1**	**0.6**	**0.3**	**0.0**	**0.0**
**Lower CI**	92.6	77.0	49.3	30.6	15.9	10.8	3.5	2.6	1.9	1.7	0.4	0.4	0.2	0.1	0.0	0.0
**Upper CI**	99.3	95.0	72.4	53.3	34.6	27.2	13.0	11.7	11.0	10.9	2.8	2.8	2.3	1.7	3.6	3.6
**Design Effect**	1.02	1.46	1.26	1.22	1.16	1.12	0.94	0.99	1.06	1.08	0.52	0.52	0.58	0.56	-	-
**Number**	100	96	77	54	37	28	14	11	8	7	4	4	2	1	0	0

Table B468-54 Age Group = 41-50 Gender = FEMALE Occupational group = MANUAL Total cases = 135

Threshold dB HL≥	0	5	10	15	20	25	30	35	40	45	50	55	65	75	85	95
**Prevalence**	**100.0**	**96.4**	**84.2**	**62.0**	**44.7**	**28.7**	**19.7**	**12.3**	**8.4**	**6.1**	**6.1**	**3.6**	**1.3**	**0.6**	**0.4**	**0.2**
**Lower CI**	97.2	90.6	76.1	52.2	35.3	21.0	13.6	8.0	5.4	3.6	3.6	2.1	0.4	0.2	0.1	0.0
**Upper CI**	100.0	98.7	89.9	70.9	54.4	37.9	27.6	18.3	12.9	10.1	10.1	6.2	4.1	1.7	1.4	1.0
**Design Effect**	-	1.18	1.13	1.17	1.16	1.13	1.05	0.92	0.80	0.78	0.78	0.64	0.83	0.51	0.51	0.48
**Number**	135	131	115	90	68	49	38	28	22	17	17	11	4	3	2	1

Table B468-55 Age Group = 51-60 Gender = FEMALE Occupational group = MANUAL Total cases = 165

Threshold dB HL≥	0	5	10	15	20	25	30	35	40	45	50	55	65	75	85	95
**Prevalence**	**100.0**	**99.3**	**92.6**	**88.1**	**69.0**	**53.8**	**38.5**	**25.1**	**16.8**	**10.8**	**7.1**	**5.9**	**2.4**	**1.8**	**0.6**	**0.3**
**Lower CI**	97.7	96.3	86.2	80.6	59.5	44.6	30.2	18.5	11.4	7.0	4.2	3.3	1.4	0.9	0.3	0.1
**Upper CI**	100.0	99.9	96.1	92.9	77.1	62.8	47.5	33.2	24.1	16.2	11.6	10.2	4.0	3.7	1.3	1.1
**Design Effect**	0.00	1.06	1.21	1.24	1.26	1.22	1.19	1.12	1.12	0.97	0.93	0.93	0.56	0.64	0.43	0.50
**Number**	165	164	155	149	129	105	82	61	42	34	25	21	13	9	4	2

Table B468-56 Age Group = 61-70 Gender = FEMALE Occupational group = MANUAL Total cases = 126

Threshold dB HL≥	0	5	10	15	20	25	30	35	40	45	50	55	65	75	85	95
**Prevalence**	**100.0**	**100.0**	**100.0**	**96.8**	**85.3**	**77.3**	**66.9**	**56.7**	**45.9**	**40.0**	**31.4**	**23.7**	**9.1**	**5.3**	**3.1**	**1.9**
**Lower CI**	97.0	97.0	97.0	90.6	75.7	66.8	56.5	46.3	36.3	30.9	24.2	16.9	6.4	3.6	2.1	1.4
**Upper CI**	100.0	100.0	100.0	99.0	91.5	85.2	75.8	66.5	55.8	49.7	39.7	32.3	12.7	7.7	4.4	2.4
**Design Effect**	0.00	0.00	0.00	1.23	1.28	1.27	1.20	1.19	1.14	1.12	0.97	1.04	0.63	0.51	0.38	0.21
**Number**	126	126	126	123	113	104	90	81	67	60	52	44	24	13	6	2

Prevalence by age group, gender and occupational group for the better ear over 4, 6, and 8 kHz

Table B468-57 Age Group = 71-80 Gender = FEMALE Occupational group = MANUAL Total cases = 76

	Threshold dB HL≥															
	0	5	10	15	20	25	30	35	40	45	50	55	65	75	85	95
Prevalence	**100.0**	**100.0**	**100.0**	**100.0**	**96.6**	**91.2**	**87.8**	**81.8**	**76.7**	**66.2**	**60.9**	**39.0**	**28.7**	**14.0**	**7.0**	**3.4**
Lower CI	95.2	95.2	95.2	95.2	82.6	74.2	71.8	62.3	55.9	47.6	45.1	27.1	18.9	9.7	3.9	1.5
Upper CI	100.0	100.0	100.0	100.0	99.4	97.4	95.3	92.4	89.5	80.8	74.8	52.5	41.1	19.9	12.2	7.5
Design Effect	-	-	-	-	1.64	1.73	1.58	1.79	1.87	1.65	1.42	1.20	1.12	0.66	0.71	0.70
Number	76	76	76	76	75	73	72	69	67	62	60	53	39	24	12	6

Table B468-58 Age Group = 18-30 Gender = MALE Occupational group = MANUAL Total cases = 109

	Threshold dB HL≥															
	0	5	10	15	20	25	30	35	40	45	50	55	65	75	85	95
Prevalence	**89.5**	**80.2**	**56.2**	**38.4**	**18.4**	**11.4**	**7.4**	**1.5**	**1.0**	**1.0**	**1.0**	**1.0**	**1.0**	**1.0**	**1.0**	**0.1**
Lower CI	78.5	68.0	43.3	27.1	10.8	5.4	2.8	0.4	0.2	0.2	0.2	0.2	0.2	0.2	0.2	0.0
Upper CI	95.2	88.6	68.3	51.0	29.7	22.6	18.3	5.0	5.1	5.1	5.1	5.1	5.1	5.1	5.1	0.4
Design Effect	1.43	1.40	1.39	1.35	1.31	1.43	1.51	0.87	0.99	0.99	0.99	0.99	0.99	0.99	0.99	0.27
Number	99	90	68	47	25	14	8	4	2	2	2	2	2	2	2	1

Table B468-59 Age Group = 31-40 Gender = MALE Occupational group = MANUAL Total cases = 80

	Threshold dB HL≥															
	0	5	10	15	20	25	30	35	40	45	50	55	65	75	85	95
Prevalence	**94.9**	**87.8**	**72.9**	**60.4**	**44.3**	**19.7**	**16.2**	**9.9**	**6.5**	**4.7**	**4.7**	**3.4**	**2.3**	**2.3**	**2.1**	**1.9**
Lower CI	82.3	73.5	56.8	45.0	30.5	11.8	8.8	4.8	3.4	2.0	2.0	1.1	0.5	0.5	0.4	0.3
Upper CI	98.7	94.9	84.6	74.0	59.1	31.1	27.9	19.6	12.1	10.8	10.8	10.3	9.8	9.8	9.9	10.2
Design Effect	1.51	1.48	1.48	1.41	1.37	1.12	1.19	1.11	0.78	0.90	0.90	1.03	1.15	1.15	1.19	1.25
Number	78	73	63	53	39	26	20	12	10	7	7	5	3	3	2	1

Table B468-60 Age Group = 41-50 Gender = MALE Occupational group = MANUAL Total cases = 127

	Threshold dB HL≥															
	0	5	10	15	20	25	30	35	40	45	50	55	65	75	85	95
Prevalence	**100.0**	**100.0**	**97.5**	**82.1**	**68.0**	**50.5**	**36.3**	**28.3**	**25.2**	**20.7**	**13.0**	**7.5**	**6.2**	**3.9**	**0.9**	**0.5**
Lower CI	97.1	97.1	90.9	71.8	56.9	40.0	27.4	20.2	17.5	13.6	7.8	3.9	2.9	1.4	0.4	0.1
Upper CI	100.0	100.0	99.4	89.2	77.3	60.9	46.2	38.1	34.9	30.3	20.9	13.8	12.6	10.8	2.4	1.7
Design Effect	-	-	1.33	1.32	1.28	1.23	1.14	1.16	1.17	1.19	1.11	1.06	1.12	1.28	0.55	0.54
Number	127	127	125	109	93	74	61	49	42	34	25	18	14	8	4	2

Table B468-61 Age Group = 51-60 Gender = MALE Occupational group = MANUAL Total cases = 198

	Threshold dB HL≥															
	0	5	10	15	20	25	30	35	40	45	50	55	65	75	85	95
Prevalence	**100.0**	**100.0**	**98.9**	**97.1**	**88.6**	**77.4**	**69.2**	**59.5**	**52.6**	**44.6**	**36.6**	**30.6**	**15.5**	**8.2**	**4.6**	**3.1**
Lower CI	98.1	98.1	95.4	92.1	81.6	68.9	60.8	51.1	44.5	37.1	29.7	24.0	11.1	5.2	2.7	1.6
Upper CI	100.0	100.0	99.7	99.0	93.2	84.0	76.6	67.4	60.5	52.5	44.1	38.1	21.2	12.6	7.6	6.1
Design Effect	0.00	0.00	1.19	1.33	1.31	1.31	1.25	1.21	1.17	1.13	1.09	1.10	1.00	0.96	0.82	0.90
Number	198	198	196	194	185	171	156	140	127	111	97	82	48	26	16	9

Table B468-62 Age Group = 61-70 Gender = MALE Occupational group = MANUAL Total cases = 171

	Threshold dB HL≥															
	0	5	10	15	20	25	30	35	40	45	50	55	65	75	85	95
Prevalence	**100.0**	**100.0**	**100.0**	**100.0**	**100.0**	**98.5**	**95.6**	**89.1**	**81.2**	**70.6**	**60.3**	**56.2**	**35.3**	**14.8**	**5.0**	**3.2**
Lower CI	97.8	97.8	97.8	97.8	97.8	93.5	90.5	82.2	72.9	60.1	50.4	46.2	26.8	11.0	3.2	1.9
Upper CI	100.0	100.0	100.0	100.0	100.0	99.7	98.0	93.6	87.4	79.3	69.5	65.7	44.9	19.7	7.7	5.5
Design Effect	-	-	-	-	-	1.35	1.16	1.21	1.25	1.44	1.33	1.33	1.29	0.82	0.68	0.67
Number	171	171	171	171	171	169	163	157	148	138	127	117	84	47	20	13

Table B468-63 Age Group = 71-80 Gender = MALE Occupational group = MANUAL Total cases = 72

	Threshold dB HL≥															
	0	5	10	15	20	25	30	35	40	45	50	55	65	75	85	95
Prevalence	**100.0**	**100.0**	**100.0**	**100.0**	**100.0**	**100.0**	**100.0**	**100.0**	**97.1**	**94.6**	**81.4**	**79.9**	**64.6**	**31.6**	**13.8**	**4.2**
Lower CI	94.9	94.9	94.9	94.9	94.9	94.9	94.9	94.9	87.2	85.0	74.3	72.5	50.0	20.2	5.7	2.2
Upper CI	100.0	100.0	100.0	100.0	100.0	100.0	100.0	100.0	99.4	98.2	86.9	85.7	76.9	45.7	30.0	7.7
Design Effect	-	-	-	-	-	-	-	-	1.28	1.16	0.70	0.72	1.27	1.23	1.53	0.58
Number	72	72	72	72	72	72	72	72	70	69	67	65	56	35	14	8

Prevalence by age group, gender and occupational group for the better ear over 0.25 kHz

Table B250-1 Age Group = 18-80 Gender = OVERALL Occupational group = OVERALL Total cases = 2659

	Threshold dB HL≥															
	**0**	**5**	**10**	**15**	**20**	**25**	**30**	**35**	**40**	**45**	**50**	**55**	**65**	**75**	**85**	**95**
**Prevalence**	**96.1**	**82.8**	**60.1**	**37.6**	**21.5**	**12.6**	**8.3**	**6.0**	**4.2**	**2.8**	**2.2**	**1.6**	**0.9**	**0.5**	**0.3**	**0.2**
**Lower CI**	94.9	80.8	57.8	35.6	19.9	11.5	7.4	5.3	3.7	2.4	1.8	1.3	0.7	0.4	0.2	0.1
**Upper CI**	97.0	84.7	62.4	39.7	23.2	13.8	9.3	6.8	4.8	3.3	2.6	2.0	1.2	0.8	0.6	0.3
**Number**	2581	2311	1820	1275	843	587	417	327	253	182	143	111	63	36	21	11

Table B250-2 Age Group = 18-80 Gender = FEMALE Occupational group = OVERALL Total cases = 1384

	Threshold dB HL≥															
	**0**	**5**	**10**	**15**	**20**	**25**	**30**	**35**	**40**	**45**	**50**	**55**	**65**	**75**	**85**	**95**
**Prevalence**	**96.5**	**83.9**	**60.0**	**38.6**	**22.5**	**14.1**	**9.6**	**6.7**	**5.1**	**3.2**	**2.4**	**1.7**	**1.0**	**0.6**	**0.3**	**0.2**
**Lower CI**	95.1	81.4	57.0	36.0	20.4	12.5	8.2	5.8	4.3	2.7	2.0	1.4	0.8	0.4	0.1	0.1
**Upper CI**	97.5	86.1	62.9	41.2	24.8	15.9	11.1	7.9	6.0	3.9	3.0	2.2	1.4	0.9	0.5	0.4
**Number**	1343	1204	942	666	446	324	239	188	152	108	85	63	41	22	11	6

Table B250-3 Age Group = 18-80 Gender = MALE Occupational group = OVERALL Total cases = 1275

	Threshold dB HL≥															
	**0**	**5**	**10**	**15**	**20**	**25**	**30**	**35**	**40**	**45**	**50**	**55**	**65**	**75**	**85**	**95**
**Prevalence**	**95.6**	**81.7**	**60.3**	**36.5**	**20.4**	**10.9**	**6.9**	**5.2**	**3.2**	**2.5**	**1.9**	**1.6**	**0.8**	**0.5**	**0.4**	**0.1**
**Lower CI**	93.4	78.5	56.6	33.4	18.1	9.4	5.8	4.2	2.6	1.9	1.4	1.1	0.4	0.2	0.2	0.1
**Upper CI**	97.0	84.6	63.8	39.8	23.0	12.4	8.2	6.3	4.0	3.2	2.6	2.2	1.3	1.1	1.0	0.3
**Number**	1238	1107	878	609	397	263	178	139	101	74	58	48	22	14	10	5

Table B250-4 Age Group = 18-30 Gender = OVERALL Occupational group = OVERALL Total cases = 401

	Threshold dB HL≥															
	**0**	**5**	**10**	**15**	**20**	**25**	**30**	**35**	**40**	**45**	**50**	**55**	**65**	**75**	**85**	**95**
**Prevalence**	**93.6**	**72.4**	**42.5**	**17.8**	**7.6**	**1.6**	**0.9**	**0.5**	**0.4**	**0.3**	**0.2**	**0.0**	**0.0**	**0.0**	**0.0**	**0.0**
**Lower CI**	90.1	67.0	37.0	14.0	5.0	1.0	0.5	0.2	0.2	0.1	0.1	0.0	0.0	0.0	0.0	0.0
**Upper CI**	95.9	77.2	48.3	22.3	11.3	2.8	1.6	1.0	0.9	0.8	0.6	0.9	0.9	0.9	0.9	0.9
**Number**	374	291	184	95	40	18	13	8	6	4	3	0	0	0	0	0

Table B250-5 Age Group = 31-40 Gender = OVERALL Occupational group = OVERALL Total cases = 331

	Threshold dB HL≥															
	**0**	**5**	**10**	**15**	**20**	**25**	**30**	**35**	**40**	**45**	**50**	**55**	**65**	**75**	**85**	**95**
**Prevalence**	**92.9**	**75.0**	**48.8**	**27.1**	**12.1**	**5.3**	**3.4**	**2.2**	**1.4**	**1.1**	**1.0**	**1.0**	**0.7**	**0.7**	**0.6**	**0.0**
**Lower CI**	88.7	68.7	42.1	21.6	8.7	3.2	1.8	1.0	0.6	0.4	0.3	0.3	0.1	0.1	0.1	0.0
**Upper CI**	95.6	80.4	55.4	33.3	16.5	8.5	6.3	4.6	3.3	3.1	3.0	3.0	3.0	3.0	3.1	1.1
**Number**	312	254	178	100	55	27	17	11	8	5	4	4	2	2	1	0

Table B250-6 Age Group = 41-50 Gender = OVERALL Occupational group = OVERALL Total cases = 450

	Threshold dB HL≥															
	**0**	**5**	**10**	**15**	**20**	**25**	**30**	**35**	**40**	**45**	**50**	**55**	**65**	**75**	**85**	**95**
**Prevalence**	**96.3**	**84.9**	**58.9**	**34.1**	**16.6**	**9.7**	**5.6**	**4.5**	**3.2**	**2.5**	**1.7**	**1.3**	**0.6**	**0.3**	**0.3**	**0.1**
**Lower CI**	93.7	80.9	54.0	29.7	13.5	7.6	4.2	3.3	2.2	1.6	1.1	0.8	0.3	0.1	0.1	0.0
**Upper CI**	97.8	88.2	63.7	38.8	20.1	12.3	7.5	6.2	4.8	3.9	2.8	2.3	1.2	0.8	0.8	0.6
**Number**	434	384	278	172	101	69	47	38	28	22	17	13	6	3	3	2

Table B250-7 Age Group = 51-60 Gender = OVERALL Occupational group = OVERALL Total cases = 680

	Threshold dB HL≥															
	**0**	**5**	**10**	**15**	**20**	**25**	**30**	**35**	**40**	**45**	**50**	**55**	**65**	**75**	**85**	**95**
**Prevalence**	**98.4**	**89.9**	**69.4**	**47.6**	**28.9**	**15.9**	**9.7**	**6.1**	**4.0**	**3.2**	**2.5**	**1.9**	**0.6**	**0.4**	**0.1**	**0.0**
**Lower CI**	96.9	87.1	65.5	43.5	25.4	13.4	8.0	4.8	3.0	2.3	1.8	1.2	0.3	0.2	0.0	0.0
**Upper CI**	99.1	92.2	73.1	51.7	32.7	18.7	11.8	7.7	5.2	4.4	3.6	2.8	1.1	0.7	0.3	0.6
**Number**	670	624	501	365	246	159	111	81	59	47	37	27	11	8	2	0

Prevalence by age group, gender and occupational group for the better ear over 0.25 kHz

Table B250-8 Age Group = 61-70 Gender = OVERALL Occupational group = OVERALL Total cases = 526

Threshold dB HL≥	0	5	10	15	20	25	30	35	40	45	50	55	65	75	85	95
Prevalence	**99.0**	**92.9**	**77.9**	**56.5**	**36.7**	**24.6**	**16.1**	**12.2**	**9.8**	**5.4**	**3.8**	**2.8**	**1.8**	**1.1**	**0.7**	**0.5**
Lower CI	97.3	89.7	73.1	51.1	31.9	20.8	13.2	9.7	7.7	4.1	2.8	2.1	1.2	0.7	0.4	0.2
Upper CI	99.6	95.2	82.1	61.7	41.8	28.8	19.5	15.3	12.4	7.0	5.2	3.8	2.6	1.9	1.4	1.2
Number	521	495	428	327	232	176	122	98	82	54	39	32	22	13	9	5

Table B250-9 Age Group = 71-80 Gender = OVERALL Occupational group = OVERALL Total cases = 271

Threshold dB HL≥	0	5	10	15	20	25	30	35	40	45	50	55	65	75	85	95
Prevalence	**99.9**	**96.8**	**90.2**	**73.8**	**50.7**	**37.8**	**28.7**	**22.5**	**14.3**	**9.5**	**7.7**	**5.9**	**3.6**	**1.7**	**0.8**	**0.6**
Lower CI	99.3	93.6	83.9	66.7	43.0	31.0	22.5	17.5	11.3	7.2	5.8	4.3	2.5	0.9	0.4	0.2
Upper CI	100.0	98.5	94.2	79.9	58.3	45.1	35.9	28.4	17.8	12.3	10.2	7.9	5.2	3.1	1.7	1.4
Number	270	263	251	216	169	138	107	91	70	50	43	35	22	10	6	4

Table B250-10 Age Group = 18-80 Gender = OVERALL Occupational group = NON-MANUAL Total cases = 1181

Threshold dB HL≥	0	5	10	15	20	25	30	35	40	45	50	55	65	75	85	95
Prevalence	**96.0**	**81.0**	**55.7**	**33.9**	**18.8**	**10.6**	**6.5**	**3.8**	**2.5**	**1.8**	**1.4**	**1.1**	**0.6**	**0.3**	**0.1**	**0.0**
Lower CI	94.4	78.0	52.3	30.8	16.4	9.2	5.5	3.1	2.1	1.4	1.0	0.8	0.4	0.2	0.0	0.0
Upper CI	97.2	83.6	59.0	37.1	21.3	12.1	7.6	4.6	3.1	2.3	1.9	1.6	1.0	0.5	0.2	0.2
Number	1141	991	728	475	294	205	137	101	78	55	45	37	22	12	4	2

Table B250-11 Age Group = 18-80 Gender = OVERALL Occupational group = MANUAL Total cases = 1474

Threshold dB HL≥	0	5	10	15	20	25	30	35	40	45	50	55	65	75	85	95
Prevalence	**96.2**	**85.1**	**64.8**	**40.6**	**24.5**	**14.0**	**9.9**	**7.7**	**5.1**	**3.4**	**2.5**	**1.9**	**1.1**	**0.7**	**0.5**	**0.3**
Lower CI	94.3	82.1	61.3	37.7	22.1	12.5	8.7	6.6	4.3	2.8	2.1	1.5	0.8	0.5	0.3	0.2
Upper CI	97.5	87.7	68.1	43.6	27.0	15.6	11.2	8.9	6.0	4.1	3.1	2.4	1.5	1.1	0.9	0.4
Number	1436	1318	1090	798	548	381	280	226	175	127	98	74	41	24	17	9

Table B250-12 Age Group = 18-30 Gender = FEMALE Occupational group = OVERALL Total cases = 224

Threshold dB HL≥	0	5	10	15	20	25	30	35	40	45	50	55	65	75	85	95
Prevalence	**94.5**	**72.5**	**37.6**	**14.5**	**6.3**	**2.4**	**1.5**	**0.8**	**0.6**	**0.5**	**0.3**	**0.0**	**0.0**	**0.0**	**0.0**	**0.0**
Lower CI	90.4	65.8	30.8	10.7	3.9	1.4	0.8	0.4	0.2	0.2	0.1	0.0	0.0	0.0	0.0	0.0
Upper CI	97.0	78.2	44.9	19.2	9.8	3.9	2.8	1.8	1.6	1.5	1.2	1.7	1.7	1.7	1.7	1.7
Number	210	159	96	51	24	14	10	6	4	3	2	0	0	0	0	0

Table B250-13 Age Group = 31-40 Gender = FEMALE Occupational group = OVERALL Total cases = 189

Threshold dB HL≥	0	5	10	15	20	25	30	35	40	45	50	55	65	75	85	95
Prevalence	**93.3**	**75.7**	**48.7**	**28.1**	**13.5**	**4.8**	**3.2**	**1.3**	**1.3**	**1.0**	**0.7**	**0.7**	**0.2**	**0.2**	**0.0**	**0.0**
Lower CI	88.0	67.7	40.3	21.2	8.9	2.5	1.4	0.6	0.6	0.4	0.3	0.3	0.0	0.0	0.0	0.0
Upper CI	96.3	82.3	57.1	36.1	20.1	9.0	7.3	2.9	2.9	2.6	2.2	2.2	1.2	1.2	2.0	2.0
Number	178	149	104	60	33	15	10	6	6	4	3	3	1	1	0	0

Table B250-14 Age Group = 41-50 Gender = FEMALE Occupational group = OVERALL Total cases = 247

Threshold dB HL≥	0	5	10	15	20	25	30	35	40	45	50	55	65	75	85	95
Prevalence	**95.9**	**83.9**	**60.1**	**34.7**	**14.6**	**10.4**	**5.8**	**4.7**	**3.8**	**3.2**	**1.8**	**1.2**	**0.2**	**0.1**	**0.1**	**0.1**
Lower CI	92.2	78.2	53.4	28.9	11.1	7.7	4.1	3.1	2.3	1.8	0.9	0.5	0.1	0.0	0.0	0.0
Upper CI	97.9	88.4	66.4	41.0	19.0	13.9	8.3	7.1	6.4	5.8	3.7	3.0	0.8	0.6	0.6	0.6
Number	237	210	157	96	53	41	27	21	17	14	9	6	2	1	1	1

Prevalence by age group, gender and occupational group for the better ear over 0.25 kHz

Table B250-15 Age Group = 51-60 Gender = FEMALE Occupational group = OVERALL Total cases = 340

	Threshold dB HL≥															
	0	5	10	15	20	25	30	35	40	45	50	55	65	75	85	95
**Prevalence**	**99.1**	**91.2**	**69.6**	**47.1**	**28.6**	**17.9**	**9.9**	**5.7**	**4.1**	**3.2**	**2.3**	**1.3**	**0.8**	**0.4**	**0.1**	**0.0**
**Lower CI**	97.3	87.4	64.1	41.4	24.0	14.2	7.6	4.1	2.9	2.1	1.4	0.7	0.4	0.2	0.0	0.0
**Upper CI**	99.7	93.9	74.5	52.8	33.8	22.2	12.8	7.9	5.9	4.7	3.7	2.4	1.7	0.8	0.5	1.1
**Number**	337	315	249	181	124	84	58	41	33	26	20	12	8	5	2	0

Table B250-16 Age Group = 61-70 Gender = FEMALE Occupational group = OVERALL Total cases = 241

	Threshold dB HL≥															
	0	5	10	15	20	25	30	35	40	45	50	55	65	75	85	95
**Prevalence**	**98.9**	**95.5**	**81.0**	**62.0**	**39.4**	**26.7**	**17.7**	**14.1**	**11.3**	**5.4**	**3.8**	**3.2**	**2.2**	**1.4**	**0.8**	**0.7**
**Lower CI**	95.5	91.1	73.9	54.5	32.6	21.1	13.4	10.3	8.0	3.9	2.7	2.3	1.4	0.7	0.3	0.3
**Upper CI**	99.8	97.8	86.6	69.0	46.7	33.1	23.1	19.1	15.7	7.5	5.3	4.5	3.4	2.5	2.1	2.1
**Number**	239	232	204	160	114	87	64	53	44	30	22	19	14	7	4	3

Table B250-17 Age Group = 71-80 Gender = FEMALE Occupational group = OVERALL Total cases = 143

	Threshold dB HL≥															
	0	5	10	15	20	25	30	35	40	45	50	55	65	75	85	95
**Prevalence**	**99.8**	**98.8**	**89.9**	**76.0**	**55.0**	**40.4**	**33.6**	**25.7**	**17.1**	**10.1**	**9.1**	**6.8**	**4.6**	**2.4**	**0.9**	**0.5**
**Lower CI**	98.9	96.8	80.4	65.8	43.7	30.8	24.5	19.0	12.8	7.2	6.4	4.6	3.0	1.2	0.4	0.1
**Upper CI**	100.0	99.6	95.1	83.8	65.8	50.8	44.2	33.7	22.4	14.0	12.8	9.8	7.1	4.8	2.3	1.8
**Number**	142	139	132	118	98	83	70	61	48	31	29	23	16	8	4	2

Table B250-18 Age Group = 18-30 Gender = MALE Occupational group = OVERALL Total cases = 177

	Threshold dB HL≥															
	0	5	10	15	20	25	30	35	40	45	50	55	65	75	85	95
**Prevalence**	**92.5**	**72.5**	**47.9**	**21.3**	**9.0**	**0.8**	**0.2**	**0.1**	**0.1**	**0.0**	**0.0**	**0.0**	**0.0**	**0.0**	**0.0**	**0.0**
**Lower CI**	85.8	63.7	39.0	14.8	4.8	0.2	0.1	0.0	0.0	0.0	0.0	0.0	0.0	0.0	0.0	0.0
**Upper CI**	96.1	79.9	56.8	29.5	16.3	3.0	0.8	0.2	0.2	0.2	0.2	2.1	2.1	2.1	2.1	2.1
**Number**	164	132	88	44	16	4	3	2	2	1	1	0	0	0	0	0

Table B250-19 Age Group = 31-40 Gender = MALE Occupational group = OVERALL Total cases = 142

	Threshold dB HL≥															
	0	5	10	15	20	25	30	35	40	45	50	55	65	75	85	95
**Prevalence**	**92.5**	**74.5**	**49.0**	**26.1**	**10.5**	**5.9**	**3.8**	**3.2**	**1.5**	**1.2**	**1.2**	**1.2**	**1.2**	**1.2**	**1.2**	**0.0**
**Lower CI**	84.9	64.3	38.7	18.0	6.2	2.9	1.5	1.1	0.4	0.2	0.2	0.2	0.2	0.2	0.2	0.0
**Upper CI**	96.4	82.5	59.4	36.1	17.2	11.8	9.2	8.8	6.4	6.5	6.5	6.5	6.5	6.5	6.5	2.6
**Number**	134	105	74	40	22	12	7	5	2	1	1	1	1	1	1	0

Table B250-20 Age Group = 41-50 Gender = MALE Occupational group = OVERALL Total cases = 203

	Threshold dB HL≥															
	0	5	10	15	20	25	30	35	40	45	50	55	65	75	85	95
**Prevalence**	**96.6**	**86.0**	**57.7**	**33.4**	**18.7**	**8.9**	**5.4**	**4.3**	**2.6**	**1.7**	**1.7**	**1.4**	**1.0**	**0.4**	**0.4**	**0.2**
**Lower CI**	92.2	80.1	50.4	27.1	14.0	6.0	3.4	2.6	1.4	0.9	0.9	0.8	0.5	0.1	0.1	0.0
**Upper CI**	98.6	90.4	64.7	40.5	24.5	13.1	8.4	7.1	4.7	3.1	3.1	2.7	2.2	1.6	1.6	1.1
**Number**	197	174	121	76	48	28	20	17	11	8	8	7	4	2	2	1

Table B250-21 Age Group = 51-60 Gender = MALE Occupational group = OVERALL Total cases = 340

	Threshold dB HL≥															
	0	5	10	15	20	25	30	35	40	45	50	55	65	75	85	95
**Prevalence**	**97.5**	**88.5**	**69.3**	**48.2**	**29.2**	**13.6**	**9.5**	**6.6**	**3.8**	**3.3**	**2.8**	**2.5**	**0.3**	**0.3**	**0.0**	**0.0**
**Lower CI**	94.7	83.9	63.5	42.3	24.2	10.6	7.0	4.7	2.5	2.0	1.6	1.4	0.1	0.1	0.0	0.0
**Upper CI**	98.9	92.0	74.6	54.2	34.7	17.2	12.6	9.2	5.8	5.2	4.7	4.4	1.0	1.0	1.1	1.1
**Number**	333	309	252	184	122	75	53	40	26	21	17	15	3	3	0	0

Prevalence by age group, gender and occupational group for the better ear over 0.25 kHz

Table B250-22 Age Group = 61-70 Gender = MALE Occupational group = OVERALL Total cases = 285

Threshold dB HL≥

	0	5	10	15	20	25	30	35	40	45	50	55	65	75	85	95
Prevalence	**99.1**	**90.0**	**74.6**	**50.2**	**33.9**	**22.1**	**14.2**	**10.0**	**8.1**	**5.4**	**3.9**	**2.3**	**1.3**	**0.9**	**0.6**	**0.3**
Lower CI	97.1	84.7	68.2	42.6	27.3	17.6	10.8	7.4	6.0	3.5	2.2	1.3	0.6	0.4	0.3	0.1
Upper CI	99.7	93.6	80.1	57.7	41.2	27.5	18.6	13.3	10.9	8.2	6.6	4.0	2.6	2.1	1.4	0.9
Number	282	263	224	167	118	89	58	45	38	24	17	13	8	6	5	2

Table B250-23 Age Group = 71-80 Gender = MALE Occupational group = OVERALL Total cases = 128

Threshold dB HL≥

	0	5	10	15	20	25	30	35	40	45	50	55	65	75	85	95
Prevalence	**100.0**	**93.4**	**90.3**	**70.4**	**44.1**	**33.7**	**21.4**	**17.6**	**10.4**	**8.7**	**5.9**	**4.6**	**2.3**	**0.7**	**0.7**	**0.7**
Lower CI	97.1	84.8	81.5	60.7	35.3	25.5	14.9	11.5	6.9	5.6	3.5	2.8	1.0	0.2	0.2	0.2
Upper CI	100.0	97.3	95.2	78.5	53.2	43.0	29.9	25.9	15.3	13.2	9.6	7.7	4.9	2.4	2.4	2.4
Number	128	124	119	98	71	55	37	30	22	19	14	12	6	2	2	2

Table B250-24 Age Group = 18-30 Gender = OVERALL Occupational group = NON-MANUAL Total cases = 181

Threshold dB HL≥

	0	5	10	15	20	25	30	35	40	45	50	55	65	75	85	95
Prevalence	**93.1**	**69.6**	**36.4**	**14.5**	**6.4**	**1.2**	**0.7**	**0.2**	**0.2**	**0.1**	**0.1**	**0.0**	**0.0**	**0.0**	**0.0**	**0.0**
Lower CI	87.9	61.8	28.9	9.3	3.0	0.5	0.2	0.1	0.1	0.0	0.0	0.0	0.0	0.0	0.0	0.0
Upper CI	96.2	76.4	44.7	21.9	13.4	3.2	2.4	0.7	0.7	0.8	0.8	2.1	2.1	2.1	2.1	2.1
Number	168	124	67	31	12	5	3	2	2	1	1	0	0	0	0	0

Table B250-25 Age Group = 31-40 Gender = OVERALL Occupational group = NON-MANUAL Total cases = 148

Threshold dB HL≥

	0	5	10	15	20	25	30	35	40	45	50	55	65	75	85	95
Prevalence	**94.2**	**76.1**	**51.1**	**30.4**	**12.7**	**4.8**	**2.4**	**0.7**	**0.4**	**0.3**	**0.3**	**0.3**	**0.0**	**0.0**	**0.0**	**0.0**
Lower CI	89.3	67.3	42.0	22.0	7.9	2.0	0.8	0.2	0.1	0.1	0.1	0.1	0.0	0.0	0.0	0.0
Upper CI	96.9	83.2	60.1	40.3	20.0	11.0	6.5	2.1	1.6	1.7	1.7	1.7	2.5	2.5	2.5	2.5
Number	139	112	75	40	20	8	5	3	2	1	1	1	0	0	0	0

Table B250-26 Age Group = 41-50 Gender = OVERALL Occupational group = NON-MANUAL Total cases = 188

Threshold dB HL≥

	0	5	10	15	20	25	30	35	40	45	50	55	65	75	85	95
Prevalence	**95.2**	**83.1**	**55.4**	**30.9**	**10.7**	**7.0**	**3.7**	**2.3**	**1.2**	**0.4**	**0.4**	**0.1**	**0.0**	**0.0**	**0.0**	**0.0**
Lower CI	90.9	77.2	48.1	24.0	7.0	4.5	1.9	1.9	1.0	0.2	0.2	0.0	0.0	0.0	0.0	0.0
Upper CI	97.5	87.8	62.5	38.7	16.0	10.7	7.0	2.7	1.5	0.7	0.7	0.7	2.0	2.0	2.0	2.0
Number	178	154	103	56	22	16	9	7	4	2	2	1	0	0	0	0

Table B250-27 Age Group = 51-60 Gender = OVERALL Occupational group = NON-MANUAL Total cases = 316

Threshold dB HL≥

	0	5	10	15	20	25	30	35	40	45	50	55	65	75	85	95
Prevalence	**97.8**	**86.6**	**61.2**	**38.3**	**20.5**	**11.4**	**6.4**	**3.6**	**2.4**	**1.8**	**1.6**	**1.1**	**0.6**	**0.4**	**0.1**	**0.0**
Lower CI	95.3	81.8	54.7	32.4	16.5	8.5	4.5	2.2	1.5	1.1	0.9	0.5	0.2	0.1	0.0	0.0
Upper CI	99.0	90.3	67.3	44.7	25.3	15.1	9.2	5.8	3.6	3.0	2.8	2.2	1.6	1.0	0.4	1.2
Number	309	278	206	139	85	56	36	24	19	15	13	8	5	4	1	0

Table B250-28 Age Group = 61-70 Gender = OVERALL Occupational group = NON-MANUAL Total cases = 226

Threshold dB HL≥

	0	5	10	15	20	25	30	35	40	45	50	55	65	75	85	95
Prevalence	**100.0**	**91.0**	**71.5**	**54.0**	**33.1**	**23.5**	**14.1**	**10.1**	**8.5**	**6.6**	**4.1**	**3.5**	**1.8**	**1.3**	**0.4**	**0.3**
Lower CI	98.3	85.8	65.2	47.6	25.9	18.6	10.8	7.4	6.3	4.5	2.3	2.0	0.9	0.5	0.1	0.1
Upper CI	100.0	94.5	77.2	60.2	41.2	29.2	18.2	13.6	11.5	9.6	7.2	6.0	3.6	3.1	1.3	1.2
Number	226	207	166	123	86	69	47	36	31	24	17	16	10	6	3	2

Prevalence by age group, gender and occupational group for the better ear over 0.25 kHz

Table B250-29 Age Group = 71-80 Gender = OVERALL Occupational group = NON-MANUAL Total cases = 122

Threshold dB HL≥	0	5	10	15	20	25	30	35	40	45	50	55	65	75	85	95
**Prevalence**	**99.8**	**96.0**	**88.8**	**65.4**	**53.8**	**32.5**	**24.0**	**13.7**	**7.1**	**4.3**	**4.1**	**4.1**	**2.8**	**0.6**	**0.0**	**0.0**
**Lower CI**	98.8	91.1	76.6	53.7	42.7	25.5	18.4	8.7	4.7	2.5	2.3	2.3	1.3	0.2	0.0	0.0
**Upper CI**	100.0	98.3	95.0	75.5	64.6	40.5	30.5	21.0	10.5	7.4	7.1	7.1	5.9	2.3	3.1	3.1
**Number**	121	116	111	86	69	51	37	29	20	12	11	11	7	2	0	0

Table B250-30 Age Group = 18-30 Gender = OVERALL Occupational group = MANUAL Total cases = 218

Threshold dB HL≥	0	5	10	15	20	25	30	35	40	45	50	55	65	75	85	95
**Prevalence**	**93.7**	**75.9**	**51.8**	**21.8**	**10.0**	**1.8**	**1.1**	**0.5**	**0.4**	**0.3**	**0.2**	**0.0**	**0.0**	**0.0**	**0.0**	**0.0**
**Lower CI**	87.8	67.4	43.0	15.8	5.8	0.9	0.6	0.2	0.1	0.1	0.0	0.0	0.0	0.0	0.0	0.0
**Upper CI**	96.9	82.8	60.4	29.3	16.5	3.5	2.1	1.2	1.0	0.9	0.7	1.7	1.7	1.7	1.7	1.7
**Number**	204	167	117	64	28	13	10	6	4	3	2	0	0	0	0	0

Table B250-31 Age Group = 31-40 Gender = OVERALL Occupational group = MANUAL Total cases = 183

Threshold dB HL≥	0	5	10	15	20	25	30	35	40	45	50	55	65	75	85	95
**Prevalence**	**92.5**	**74.1**	**45.0**	**23.2**	**12.2**	**5.5**	**4.2**	**3.5**	**2.0**	**1.6**	**1.3**	**1.3**	**1.1**	**1.1**	**0.9**	**0.0**
**Lower CI**	86.0	64.5	35.8	16.6	7.7	2.9	1.9	1.4	0.8	0.5	0.3	0.3	0.2	0.2	0.2	0.0
**Upper CI**	96.1	81.8	54.5	31.4	18.8	10.4	9.3	8.8	5.3	5.0	4.8	4.8	4.9	4.9	5.0	2.1
**Number**	173	142	103	60	35	19	12	8	6	4	3	3	2	2	1	0

Table B250-32 Age Group = 41-50 Gender = OVERALL Occupational group = MANUAL Total cases = 262

Threshold dB HL≥	0	5	10	15	20	25	30	35	40	45	50	55	65	75	85	95
**Prevalence**	**97.5**	**89.3**	**65.4**	**38.8**	**21.9**	**12.1**	**6.9**	**5.8**	**4.5**	**3.7**	**2.3**	**1.8**	**0.7**	**0.3**	**0.3**	**0.2**
**Lower CI**	94.3	84.8	58.5	32.3	17.0	8.9	4.9	3.9	2.9	2.3	1.4	1.1	0.4	0.1	0.1	0.1
**Upper CI**	99.0	92.7	71.8	45.8	27.8	16.3	9.5	8.4	6.9	6.0	3.6	3.0	1.5	0.9	0.9	0.7
**Number**	256	230	175	116	79	53	38	31	24	20	15	12	6	3	3	2

Table B250-33 Age Group = 51-60 Gender = OVERALL Occupational group = MANUAL Total cases = 364

Threshold dB HL≥	0	5	10	15	20	25	30	35	40	45	50	55	65	75	85	95
**Prevalence**	**99.3**	**92.8**	**76.3**	**54.7**	**35.3**	**19.2**	**12.0**	**7.9**	**4.7**	**4.0**	**2.9**	**2.1**	**0.5**	**0.3**	**0.1**	**0.0**
**Lower CI**	97.7	88.5	70.4	48.6	29.8	15.5	9.3	5.9	3.3	2.7	1.8	1.3	0.2	0.1	0.0	0.0
**Upper CI**	99.8	95.6	81.3	60.7	41.1	23.6	15.2	10.5	6.6	5.9	4.5	3.5	0.9	0.8	0.5	1.0
**Number**	361	346	295	226	161	103	75	57	40	32	24	19	6	4	1	0

Table B250-34 Age Group = 61-70 Gender = OVERALL Occupational group = MANUAL Total cases = 299

Threshold dB HL≥	0	5	10	15	20	25	30	35	40	45	50	55	65	75	85	95
**Prevalence**	**97.8**	**94.2**	**82.2**	**57.4**	**40.0**	**24.7**	**17.4**	**14.2**	**11.0**	**5.0**	**4.0**	**2.9**	**2.1**	**1.4**	**1.3**	**1.0**
**Lower CI**	93.9	89.4	75.6	49.9	33.6	20.0	13.6	11.0	8.6	3.6	2.8	2.0	1.5	1.0	1.0	0.8
**Upper CI**	99.2	96.9	87.3	64.7	46.8	30.1	22.0	18.3	14.0	6.9	5.9	4.2	2.9	1.9	1.6	1.3
**Number**	294	287	261	203	146	107	75	62	51	30	22	16	12	7	6	3

Table B250-35 Age Group = 71-80 Gender = OVERALL Occupational group = MANUAL Total cases = 148

Threshold dB HL≥	0	5	10	15	20	25	30	35	40	45	50	55	65	75	85	95
**Prevalence**	**100.0**	**97.4**	**91.1**	**80.1**	**51.4**	**44.1**	**37.4**	**30.8**	**18.5**	**12.1**	**9.6**	**6.6**	**4.2**	**2.5**	**1.4**	**0.9**
**Lower CI**	97.5	88.2	79.9	70.2	42.1	35.7	29.3	23.2	13.7	9.0	6.9	4.7	2.7	1.3	0.7	0.4
**Upper CI**	100.0	99.5	96.3	87.3	60.6	52.8	46.2	39.6	24.5	16.1	13.1	9.1	6.6	4.7	2.9	2.3
**Number**	148	146	139	129	99	86	70	62	50	38	32	24	15	8	6	4

Prevalence by age group, gender and occupational group for the better ear over 0.25 kHz

Table B250-36 Age Group = 18-80 Gender = FEMALE Occupational group = NON-MANUAL Total cases = 669

Threshold dB HL≥	0	5	10	15	20	25	30	35	40	45	50	55	65	75	85	95
**Prevalence**	**95.6**	**81.4**	**55.6**	**35.0**	**20.6**	**12.8**	**7.9**	**4.2**	**2.8**	**2.2**	**1.8**	**1.5**	**0.9**	**0.4**	**0.1**	**0.1**
Lower CI	93.5	78.0	51.6	31.5	17.7	10.9	6.5	3.2	2.2	1.6	1.3	1.0	0.5	0.2	0.0	0.0
Upper CI	97.0	84.4	59.5	38.7	23.8	15.1	9.6	5.5	3.5	2.9	2.4	2.1	1.4	0.7	0.4	0.4
Number	643	563	419	281	177	131	90	67	53	40	34	28	17	9	4	2

Table B250-37 Age Group = 18-80 Gender = MALE Occupational group = NON-MANUAL Total cases = 512

Threshold dB HL≥	0	5	10	15	20	25	30	35	40	45	50	55	65	75	85	95
**Prevalence**	**96.5**	**80.7**	**56.1**	**32.7**	**16.8**	**8.1**	**4.9**	**3.3**	**2.3**	**1.4**	**1.0**	**0.8**	**0.4**	**0.2**	**0.0**	**0.0**
Lower CI	93.5	75.6	50.5	27.7	13.2	6.3	3.7	2.5	1.6	0.9	0.5	0.4	0.1	0.1	0.0	0.0
Upper CI	98.1	84.9	61.5	38.2	21.1	10.3	6.6	4.4	3.2	2.3	1.9	1.6	1.0	0.8	0.7	0.7
Number	498	428	309	194	117	74	47	34	25	15	11	9	5	3	0	0

Table B250-38 Age Group = 18-80 Gender = FEMALE Occupational group = MANUAL Total cases = 714

Threshold dB HL≥	0	5	10	15	20	25	30	35	40	45	50	55	65	75	85	95
**Prevalence**	**97.0**	**85.8**	**64.2**	**41.3**	**24.9**	**14.7**	**10.7**	**8.1**	**6.2**	**3.7**	**2.6**	**1.8**	**1.2**	**0.8**	**0.5**	**0.4**
Lower CI	94.7	81.8	59.4	37.5	21.8	12.7	9.0	6.6	5.1	2.9	2.1	1.4	0.9	0.6	0.3	0.3
Upper CI	98.3	89.1	68.6	45.2	28.2	17.0	12.6	9.8	7.6	4.6	3.4	2.3	1.6	1.2	0.7	0.5
Number	699	640	522	384	269	193	149	121	99	68	51	35	24	13	7	4

Table B250-39 Age Group = 18-80 Gender = MALE Occupational group = MANUAL Total cases = 760

Threshold dB HL≥	0	5	10	15	20	25	30	35	40	45	50	55	65	75	85	95
**Prevalence**	**95.3**	**84.3**	**65.5**	**40.0**	**24.1**	**13.2**	**9.0**	**7.2**	**3.8**	**3.1**	**2.4**	**2.0**	**0.9**	**0.6**	**0.6**	**0.2**
Lower CI	91.9	79.6	60.4	35.4	20.6	11.2	7.3	5.7	3.0	2.3	1.7	1.3	0.5	0.2	0.2	0.1
Upper CI	97.3	88.1	70.3	44.7	28.1	15.5	10.9	9.0	5.0	4.2	3.4	2.9	1.8	1.6	1.6	0.4
Number	737	678	568	414	279	188	131	105	76	59	47	39	17	11	10	5

Table B250-40 Age Group = 18-30 Gender = FEMALE Occupational group = NON-MANUAL Total cases = 115

Threshold dB HL≥	0	5	10	15	20	25	30	35	40	45	50	55	65	75	85	95
**Prevalence**	**92.4**	**67.7**	**29.2**	**9.9**	**3.8**	**2.2**	**1.1**	**0.3**	**0.3**	**0.3**	**0.3**	**0.0**	**0.0**	**0.0**	**0.0**	**0.0**
Lower CI	86.3	59.4	21.5	6.4	1.7	0.8	0.3	0.0	0.0	0.0	0.0	0.0	0.0	0.0	0.0	0.0
Upper CI	95.9	75.1	38.4	14.9	8.1	5.9	4.5	1.5	1.5	1.5	1.5	3.2	3.2	3.2	3.2	3.2
Number	106	78	38	18	7	4	2	1	1	1	1	0	0	0	0	0

Table B250-41 Age Group = 31-40 Gender = FEMALE Occupational group = NON-MANUAL Total cases = 86

Threshold dB HL≥	0	5	10	15	20	25	30	35	40	45	50	55	65	75	85	95
**Prevalence**	**92.0**	**76.1**	**48.6**	**31.4**	**14.3**	**4.4**	**2.9**	**0.7**	**0.7**	**0.6**	**0.6**	**0.6**	**0.0**	**0.0**	**0.0**	**0.0**
Lower CI	84.6	65.0	37.5	21.6	7.8	1.4	0.7	0.2	0.2	0.1	0.1	0.1	0.0	0.0	0.0	0.0
Upper CI	96.0	84.5	59.8	43.2	24.9	13.1	11.0	3.0	3.0	3.2	3.2	3.2	4.3	4.3	4.3	4.3
Number	79	66	43	25	12	4	3	2	2	1	1	1	0	0	0	0

Table B250-42 Age Group = 41-50 Gender = FEMALE Occupational group = NON-MANUAL Total cases = 112

Threshold dB HL≥	0	5	10	15	20	25	30	35	40	45	50	55	65	75	85	95
**Prevalence**	**95.4**	**82.5**	**55.4**	**29.4**	**9.6**	**7.6**	**1.8**	**1.5**	**0.6**	**0.6**	**0.6**	**0.0**	**0.0**	**0.0**	**0.0**	**0.0**
Lower CI	89.6	73.9	46.1	21.7	6.2	4.9	1.2	1.1	0.1	0.1	0.1	0.0	0.0	0.0	0.0	0.0
Upper CI	98.0	88.7	64.4	38.5	14.5	11.5	2.6	2.1	4.3	4.3	4.3	3.3	3.3	3.3	3.3	3.3
Number	106	92	63	33	13	10	4	3	1	1	1	0	0	0	0	0

Prevalence by age group, gender and occupational group for the better ear over 0.25 kHz

Table B250-43 Age Group = 51-60 Gender = FEMALE Occupational group = NON-MANUAL Total cases = 175

Threshold dB HL≥	0	5	10	15	20	25	30	35	40	45	50	55	65	75	85	95
**Prevalence**	**98.4**	**87.4**	**64.9**	**41.3**	**23.6**	**16.0**	**8.2**	**4.8**	**3.3**	**2.3**	**2.2**	**1.3**	**0.8**	**0.3**	**0.1**	**0.0**
**Lower CI**	95.4	81.2	57.6	33.8	17.8	11.2	5.2	2.6	1.9	1.3	1.2	0.5	0.2	0.1	0.0	0.0
**Upper CI**	99.5	91.8	71.7	49.3	30.7	22.2	12.5	8.6	5.5	4.3	4.2	3.3	2.5	1.2	0.7	2.1
**Number**	172	155	119	82	52	37	23	16	13	10	9	5	3	2	1	0

Table B250-44 Age Group = 61-70 Gender = FEMALE Occupational group = NON-MANUAL Total cases = 114

Threshold dB HL≥	0	5	10	15	20	25	30	35	40	45	50	55	65	75	85	95
**Prevalence**	**100.0**	**95.1**	**79.2**	**60.1**	**32.5**	**24.6**	**14.2**	**9.4**	**7.0**	**5.5**	**3.5**	**3.5**	**2.1**	**1.3**	**0.7**	**0.6**
**Lower CI**	96.7	88.6	70.8	51.2	24.5	17.9	9.8	6.1	4.9	3.5	2.3	2.3	1.1	0.6	0.2	0.1
**Upper CI**	100.0	98.0	85.7	68.4	41.7	32.9	20.1	14.4	10.0	8.4	5.3	5.3	3.8	2.9	2.4	2.4
**Number**	114	108	95	74	48	41	30	23	20	16	12	12	8	5	3	2

Table B250-45 Age Group = 71-80 Gender = FEMALE Occupational group = NON-MANUAL Total cases = 67

Threshold dB HL≥	0	5	10	15	20	25	30	35	40	45	50	55	65	75	85	95
**Prevalence**	**99.7**	**97.9**	**88.6**	**69.3**	**66.3**	**38.8**	**32.1**	**16.0**	**8.5**	**6.4**	**6.0**	**6.0**	**4.0**	**1.1**	**0.0**	**0.0**
**Lower CI**	98.1	92.6	69.2	51.7	48.8	29.1	24.3	9.2	5.6	3.6	3.3	3.3	1.7	0.3	0.0	0.0
**Upper CI**	99.9	99.4	96.4	82.6	80.3	49.3	41.0	26.3	12.8	11.1	10.7	10.7	8.8	3.8	5.4	5.4
**Number**	66	64	61	49	45	35	28	22	16	11	10	10	6	2	0	0

Table B250-46 Age Group = 18-30 Gender = MALE Occupational group = NON-MANUAL Total cases = 66

Threshold dB HL≥	0	5	10	15	20	25	30	35	40	45	50	55	65	75	85	95
**Prevalence**	**93.7**	**71.7**	**44.0**	**19.3**	**9.2**	**0.1**	**0.1**	**0.1**	**0.1**	**0.0**	**0.0**	**0.0**	**0.0**	**0.0**	**0.0**	**0.0**
**Lower CI**	83.0	57.9	31.2	10.0	3.3	0.0	0.0	0.0	0.0	0.0	0.0	0.0	0.0	0.0	0.0	0.0
**Upper CI**	97.8	82.4	57.6	33.8	23.5	5.7	5.7	5.7	5.7	5.5	5.5	5.5	5.5	5.5	5.5	5.5
**Number**	62	46	29	13	5	1	1	1	1	0	0	0	0	0	0	0

Table B250-47 Age Group = 31-40 Gender = MALE Occupational group = NON-MANUAL Total cases = 62

Threshold dB HL≥	0	5	10	15	20	25	30	35	40	45	50	55	65	75	85	95
**Prevalence**	**96.7**	**76.7**	**54.6**	**29.6**	**11.0**	**5.3**	**1.9**	**0.6**	**0.0**	**0.0**	**0.0**	**0.0**	**0.0**	**0.0**	**0.0**	**0.0**
**Lower CI**	88.2	61.8	39.9	16.9	5.1	1.6	0.5	0.1	0.0	0.0	0.0	0.0	0.0	0.0	0.0	0.0
**Upper CI**	99.1	87.0	68.5	46.6	22.5	16.7	7.1	3.5	5.8	5.8	5.8	5.8	5.8	5.8	5.8	5.8
**Number**	60	46	32	15	8	4	2	1	0	0	0	0	0	0	0	0

Table B250-48 Age Group = 41-50 Gender = MALE Occupational group = NON-MANUAL Total cases = 76

Threshold dB HL≥	0	5	10	15	20	25	30	35	40	45	50	55	65	75	85	95
**Prevalence**	**95.1**	**84.3**	**55.9**	**32.6**	**12.1**	**6.5**	**5.8**	**3.1**	**1.8**	**0.3**	**0.3**	**0.3**	**0.0**	**0.0**	**0.0**	**0.0**
**Lower CI**	87.4	75.6	44.4	21.5	6.0	2.8	2.4	2.7	1.4	0.0	0.0	0.0	0.0	0.0	0.0	0.0
**Upper CI**	98.2	90.3	66.8	45.9	22.8	14.0	13.3	3.7	2.4	1.4	1.4	1.4	4.8	4.8	4.8	4.8
**Number**	72	62	40	23	9	6	5	4	3	1	1	1	0	0	0	0

Table B250-49 Age Group = 51-60 Gender = MALE Occupational group = NON-MANUAL Total cases = 141

Threshold dB HL≥	0	5	10	15	20	25	30	35	40	45	50	55	65	75	85	95
**Prevalence**	**97.1**	**85.7**	**57.0**	**35.1**	**17.1**	**6.2**	**4.6**	**2.3**	**1.4**	**1.2**	**1.0**	**0.8**	**0.5**	**0.5**	**0.0**	**0.0**
**Lower CI**	92.4	77.6	46.1	26.2	11.9	3.8	2.4	1.1	0.6	0.5	0.4	0.3	0.1	0.1	0.0	0.0
**Upper CI**	99.0	91.2	67.3	45.2	23.8	9.9	8.4	4.8	3.0	2.9	2.5	2.5	1.8	1.8	2.7	2.7
**Number**	137	123	87	57	33	19	13	8	6	5	4	3	2	2	0	0

Prevalence by age group, gender and occupational group for the better ear over 0.25 kHz

Table B250-50 Age Group = 61-70 Gender = MALE Occupational group = NON-MANUAL Total cases = 112

Threshold dB HL≥	0	5	10	15	20	25	30	35	40	45	50	55	65	75	85	95
Prevalence	**100.0**	**86.8**	**63.9**	**48.1**	**34.7**	**22.8**	**14.4**	**11.0**	**10.3**	**7.9**	**4.9**	**3.4**	**1.6**	**1.2**	**0.0**	**0.0**
Lower CI	96.7	77.6	54.1	39.2	22.8	16.1	9.7	7.2	6.5	4.4	1.9	1.2	0.4	0.2	0.0	0.0
Upper CI	100.0	92.6	72.6	57.2	49.0	31.2	20.9	16.6	15.8	13.7	12.1	9.5	6.5	6.6	3.3	3.3
Number	112	99	71	49	38	28	17	13	11	8	5	4	2	1	0	0

Table B250-51 Age Group = 71-80 Gender = MALE Occupational group = NON-MANUAL Total cases = 55

Threshold dB HL≥	0	5	10	15	20	25	30	35	40	45	50	55	65	75	85	95
Prevalence	**100.0**	**92.9**	**89.1**	**59.3**	**34.9**	**23.1**	**12.2**	**9.9**	**5.0**	**1.3**	**1.3**	**1.3**	**1.3**	**0.0**	**0.0**	**0.0**
Lower CI	93.5	81.2	75.6	46.8	24.5	14.5	6.4	4.7	1.9	0.2	0.2	0.2	0.2	0.0	0.0	0.0
Upper CI	100.0	97.6	95.5	70.7	47.0	34.9	21.9	19.8	12.6	7.0	7.0	7.0	7.0	6.5	6.5	6.5
Number	55	52	50	37	24	16	9	7	4	1	1	1	1	0	0	0

Table B250-52 Age Group = 18-30 Gender = FEMALE Occupational group = MANUAL Total cases = 109

Threshold dB HL≥	0	5	10	15	20	25	30	35	40	45	50	55	65	75	85	95
Prevalence	**96.1**	**76.3**	**48.6**	**20.9**	**9.5**	**2.3**	**1.9**	**1.0**	**0.6**	**0.5**	**0.3**	**0.0**	**0.0**	**0.0**	**0.0**	**0.0**
Lower CI	88.9	64.4	36.5	13.7	4.9	1.2	0.9	0.4	0.2	0.1	0.0	0.0	0.0	0.0	0.0	0.0
Upper CI	98.7	85.1	60.8	30.5	17.4	4.2	3.7	2.3	1.8	1.7	1.4	3.4	3.4	3.4	3.4	3.4
Number	104	81	58	33	17	10	8	5	3	2	1	0	0	0	0	0

Table B250-53 Age Group = 31-40 Gender = FEMALE Occupational group = MANUAL Total cases = 103

Threshold dB HL≥	0	5	10	15	20	25	30	35	40	45	50	55	65	75	85	95
Prevalence	**94.0**	**74.1**	**45.2**	**21.7**	**12.1**	**3.9**	**2.7**	**1.6**	**1.6**	**1.2**	**0.6**	**0.6**	**0.3**	**0.3**	**0.0**	**0.0**
Lower CI	85.3	61.6	33.8	14.9	7.3	2.1	1.3	0.6	0.6	0.4	0.2	0.2	0.1	0.1	0.0	0.0
Upper CI	97.7	83.7	57.0	30.5	19.4	7.0	5.8	4.1	4.1	3.8	2.3	2.3	1.7	1.7	3.6	3.6
Number	99	83	61	35	21	11	7	4	4	3	2	2	1	1	0	0

Table B250-54 Age Group = 41-50 Gender = FEMALE Occupational group = MANUAL Total cases = 135

Threshold dB HL≥	0	5	10	15	20	25	30	35	40	45	50	55	65	75	85	95
Prevalence	**96.1**	**85.9**	**65.9**	**41.9**	**20.4**	**13.1**	**8.2**	**6.8**	**6.2**	**5.4**	**2.7**	**2.0**	**0.4**	**0.2**	**0.2**	**0.2**
Lower CI	90.0	77.7	56.6	33.0	14.5	8.9	5.3	4.1	3.6	2.9	1.5	1.0	0.1	0.0	0.0	0.0
Upper CI	98.6	91.4	74.1	51.3	28.0	18.9	12.5	11.1	10.4	9.6	5.0	4.0	1.4	1.0	1.0	1.0
Number	131	118	94	63	40	31	23	18	16	13	8	6	2	1	1	1

Table B250-55 Age Group = 51-60 Gender = FEMALE Occupational group = MANUAL Total cases = 165

Threshold dB HL≥	0	5	10	15	20	25	30	35	40	45	50	55	65	75	85	95
Prevalence	**100.0**	**94.5**	**75.5**	**52.4**	**34.3**	**20.6**	**11.8**	**6.6**	**4.4**	**3.7**	**2.0**	**0.9**	**0.7**	**0.4**	**0.2**	**0.0**
Lower CI	97.7	87.8	66.6	43.7	26.7	15.0	8.2	4.3	2.7	2.1	1.1	0.5	0.3	0.1	0.0	0.0
Upper CI	100.0	97.6	82.7	60.9	42.8	27.5	16.5	10.1	7.2	6.5	3.9	1.7	1.5	1.2	1.0	2.3
Number	165	160	130	99	72	47	35	25	20	16	11	7	5	3	1	0

Table B250-56 Age Group = 61-70 Gender = FEMALE Occupational group = MANUAL Total cases = 126

Threshold dB HL≥	0	5	10	15	20	25	30	35	40	45	50	55	65	75	85	95
Prevalence	**97.6**	**96.2**	**80.5**	**60.1**	**45.3**	**27.2**	**19.5**	**17.0**	**13.5**	**5.2**	**4.3**	**3.5**	**2.8**	**1.9**	**1.6**	**1.6**
Lower CI	90.2	88.3	69.7	49.3	35.9	20.2	14.0	12.2	9.8	3.7	2.8	2.2	2.0	1.4	0.5	0.5
Upper CI	99.4	98.8	88.1	70.0	55.1	35.6	26.5	23.2	18.2	7.4	6.5	5.5	4.0	2.4	5.7	5.7
Number	124	123	108	85	66	46	34	30	24	14	10	7	6	2	1	1

Prevalence by age group, gender and occupational group for the better ear over 0.25 kHz

Table B250-57 Age Group = 71-80 Gender = FEMALE Occupational group = MANUAL Total cases = 76

	Threshold dB HL≥															
	0	5	10	15	20	25	30	35	40	45	50	55	65	75	85	95
Prevalence	**100.0**	**99.5**	**90.4**	**80.1**	**47.9**	**41.6**	**38.2**	**31.0**	**21.9**	**11.0**	**10.3**	**6.6**	**5.2**	**3.6**	**1.7**	**0.9**
Lower CI	95.2	97.0	74.0	67.1	35.0	29.6	26.3	19.8	14.8	7.4	6.9	4.2	3.1	1.7	0.7	0.3
Upper CI	100.0	99.9	96.9	88.8	61.1	54.7	51.7	45.0	31.1	16.0	15.3	10.4	8.7	7.2	4.0	3.0
Number	76	75	71	69	53	48	42	39	32	20	19	13	10	6	4	2

Table B250-58 Age Group = 18-30 Gender = MALE Occupational group = MANUAL Total cases = 109

	Threshold dB HL≥															
	0	5	10	15	20	25	30	35	40	45	50	55	65	75	85	95
Prevalence	**91.1**	**75.9**	**55.5**	**23.0**	**10.7**	**1.2**	**0.2**	**0.1**	**0.1**	**0.1**	**0.1**	**0.0**	**0.0**	**0.0**	**0.0**	**0.0**
Lower CI	80.0	63.0	43.1	14.0	4.6	0.3	0.1	0.0	0.0	0.0	0.0	0.0	0.0	0.0	0.0	0.0
Upper CI	96.3	85.4	67.3	35.4	22.9	5.0	0.9	0.4	0.4	0.4	0.4	3.4	3.4	3.4	3.4	3.4
Number	100	86	59	31	11	3	2	1	1	1	1	0	0	0	0	0

Table B250-59 Age Group = 31-40 Gender = MALE Occupational group = MANUAL Total cases = 80

	Threshold dB HL≥															
	0	5	10	15	20	25	30	35	40	45	50	55	65	75	85	95
Prevalence	**91.0**	**73.8**	**44.5**	**24.6**	**12.3**	**7.5**	**6.1**	**5.9**	**2.6**	**1.9**	**1.9**	**1.9**	**1.9**	**1.9**	**1.9**	**0.0**
Lower CI	79.5	58.4	30.4	14.1	5.6	2.9	1.9	1.8	0.6	0.3	0.3	0.3	0.3	0.3	0.3	0.0
Upper CI	96.3	85.0	59.6	39.3	24.9	18.4	17.6	17.6	10.2	10.2	10.2	10.2	10.2	10.2	10.2	4.6
Number	74	59	42	25	14	8	5	4	2	1	1	1	1	1	1	0

Table B250-60 Age Group = 41-50 Gender = MALE Occupational group = MANUAL Total cases = 127

	Threshold dB HL≥															
	0	5	10	15	20	25	30	35	40	45	50	55	65	75	85	95
Prevalence	**99.0**	**93.0**	**64.8**	**35.3**	**23.5**	**11.0**	**5.3**	**4.5**	**2.6**	**1.8**	**1.8**	**1.6**	**1.1**	**0.5**	**0.5**	**0.2**
Lower CI	96.0	88.7	54.2	26.2	16.2	6.6	3.1	2.5	1.2	0.9	0.9	0.8	0.5	0.1	0.1	0.0
Upper CI	99.8	95.7	74.1	45.7	32.8	17.6	8.9	8.0	5.3	3.5	3.5	3.2	2.5	1.7	1.7	1.3
Number	125	112	81	53	39	22	15	13	8	7	7	6	4	2	2	1

Table B250-61 Age Group = 51-60 Gender = MALE Occupational group = MANUAL Total cases = 199

	Threshold dB HL≥															
	0	5	10	15	20	25	30	35	40	45	50	55	65	75	85	95
Prevalence	**98.5**	**90.9**	**77.1**	**57.1**	**36.2**	**17.6**	**12.2**	**9.3**	**5.0**	**4.3**	**3.8**	**3.5**	**0.2**	**0.2**	**0.0**	**0.0**
Lower CI	95.2	84.0	69.0	48.7	28.9	13.3	8.6	6.3	3.1	2.5	2.1	1.8	0.0	0.0	0.0	0.0
Upper CI	99.6	95.0	83.6	65.2	44.3	22.9	16.9	13.5	8.0	7.3	6.8	6.4	0.9	0.9	1.9	1.9
Number	196	186	165	127	89	56	40	32	20	16	13	12	1	1	0	0

Table B250-62 Age Group = 61-70 Gender = MALE Occupational group = MANUAL Total cases = 173

	Threshold dB HL≥															
	0	5	10	15	20	25	30	35	40	45	50	55	65	75	85	95
Prevalence	**98.1**	**92.0**	**84.7**	**54.2**	**33.8**	**21.3**	**14.5**	**10.5**	**7.9**	**4.8**	**3.8**	**2.2**	**1.2**	**0.9**	**0.9**	**0.4**
Lower CI	93.4	84.7	77.3	43.8	25.7	16.0	10.1	6.9	5.3	2.6	1.9	1.2	0.6	0.4	0.4	0.1
Upper CI	99.5	96.0	90.0	64.3	43.1	27.8	20.4	15.7	11.7	8.7	7.5	4.1	2.7	1.9	1.9	1.3
Number	170	164	153	118	80	61	41	32	27	16	12	9	6	5	5	2

Table B250-63 Age Group = 71-80 Gender = MALE Occupational group = MANUAL Total cases = 72

	Threshold dB HL≥															
	0	5	10	15	20	25	30	35	40	45	50	55	65	75	85	95
Prevalence	**100.0**	**94.2**	**91.9**	**80.2**	**56.5**	**47.3**	**35.8**	**30.0**	**13.6**	**13.6**	**8.6**	**6.5**	**2.8**	**1.0**	**1.0**	**1.0**
Lower CI	94.9	72.4	73.2	62.8	44.4	37.9	27.7	23.4	8.6	8.6	5.0	4.0	1.2	0.3	0.3	0.3
Upper CI	100.0	99.0	97.9	90.6	67.8	56.8	44.9	37.7	20.9	20.9	14.3	10.5	6.6	3.5	3.5	3.5
Number	72	71	68	60	46	38	28	23	18	18	13	11	5	2	2	2

Prevalence by age group, gender and occupational group for the better ear over 0.5 kHz

Table B500-1 Age Group = 18-80 Gender = OVERALL Occupational group = OVERALL Total cases = 2666

	Threshold dB HL≥															
	**0**	**5**	**10**	**15**	**20**	**25**	**30**	**35**	**40**	**45**	**50**	**55**	**65**	**75**	**85**	**95**
**Prevalence**	**90.5**	**70.5**	**46.1**	**27.7**	**17.4**	**10.5**	**7.4**	**5.5**	**4.1**	**2.9**	**2.2**	**1.5**	**0.9**	**0.6**	**0.4**	**0.2**
**Lower CI**	88.8	68.2	44.0	26.1	16.1	9.5	6.6	4.8	3.5	2.4	1.8	1.2	0.7	0.4	0.3	0.1
**Upper CI**	92.0	72.6	48.2	29.5	18.9	11.6	8.3	6.2	4.7	3.4	2.6	1.8	1.2	0.9	0.7	0.5
**Number**	2487	2081	1499	1032	710	504	383	303	239	183	146	110	68	39	27	10

Table B500-2 Age Group = 18-80 Gender = FEMALE Occupational group = OVERALL Total cases = 1389

	Threshold dB HL≥															
	**0**	**5**	**10**	**15**	**20**	**25**	**30**	**35**	**40**	**45**	**50**	**55**	**65**	**75**	**85**	**95**
**Prevalence**	**91.7**	**70.6**	**46.7**	**29.5**	**19.3**	**11.9**	**8.9**	**6.6**	**5.0**	**3.3**	**2.4**	**1.6**	**1.1**	**0.7**	**0.4**	**0.2**
**Lower CI**	89.6	67.8	44.0	27.2	17.4	10.4	7.7	5.6	4.1	2.6	1.9	1.3	0.8	0.5	0.3	0.1
**Upper CI**	93.4	73.3	49.3	31.9	21.4	13.6	10.4	7.7	5.9	4.1	3.1	2.0	1.4	1.0	0.7	0.4
**Number**	1301	1082	784	549	388	283	225	183	145	109	84	64	43	25	16	6

Table B500-3 Age Group = 18-80 Gender = MALE Occupational group = OVERALL Total cases = 1277

	Threshold dB HL≥															
	**0**	**5**	**10**	**15**	**20**	**25**	**30**	**35**	**40**	**45**	**50**	**55**	**65**	**75**	**85**	**95**
**Prevalence**	**89.2**	**70.3**	**45.4**	**25.8**	**15.4**	**8.9**	**5.7**	**4.2**	**3.1**	**2.5**	**1.9**	**1.4**	**0.8**	**0.5**	**0.4**	**0.3**
**Lower CI**	86.3	66.8	42.1	23.3	13.6	7.6	4.8	3.4	2.5	1.9	1.4	1.0	0.4	0.2	0.2	0.1
**Upper CI**	91.6	73.7	48.8	28.4	17.3	10.4	6.8	5.2	3.9	3.2	2.6	2.0	1.3	1.1	1.0	1.0
**Number**	1186	999	715	483	322	221	158	120	94	74	62	46	25	14	11	4

Table B500-4 Age Group = 18-30 Gender = OVERALL Occupational group = OVERALL Total cases = 401

	Threshold dB HL≥															
	**0**	**5**	**10**	**15**	**20**	**25**	**30**	**35**	**40**	**45**	**50**	**55**	**65**	**75**	**85**	**95**
**Prevalence**	**83.8**	**53.3**	**26.0**	**8.8**	**4.1**	**1.6**	**0.9**	**0.7**	**0.6**	**0.2**	**0.1**	**0.0**	**0.0**	**0.0**	**0.0**	**0.0**
**Lower CI**	78.8	47.5	21.4	6.3	2.6	0.9	0.4	0.3	0.2	0.1	0.0	0.0	0.0	0.0	0.0	0.0
**Upper CI**	87.8	59.0	31.2	12.0	6.4	2.9	2.0	1.7	1.6	0.6	0.5	0.1	0.9	0.9	0.9	0.9
**Number**	348	235	123	56	31	16	10	8	7	4	3	1	0	0	0	0

Table B500-5 Age Group = 31-40 Gender = OVERALL Occupational group = OVERALL Total cases = 332

	Threshold dB HL≥															
	**0**	**5**	**10**	**15**	**20**	**25**	**30**	**35**	**40**	**45**	**50**	**55**	**65**	**75**	**85**	**95**
**Prevalence**	**85.9**	**61.5**	**31.6**	**15.5**	**7.3**	**2.8**	**1.7**	**1.4**	**1.1**	**1.1**	**1.0**	**1.0**	**0.7**	**0.7**	**0.7**	**0.6**
**Lower CI**	80.5	54.9	26.0	11.4	4.8	1.5	0.8	0.5	0.4	0.4	0.3	0.3	0.1	0.1	0.1	0.1
**Upper CI**	90.0	67.6	37.8	20.8	11.1	5.2	3.5	3.3	3.1	3.1	3.0	3.0	3.0	3.0	3.0	3.1
**Number**	294	225	124	68	35	17	11	8	6	5	4	4	2	2	2	1

Table B500-6 Age Group = 41-50 Gender = OVERALL Occupational group = OVERALL Total cases = 450

	Threshold dB HL≥															
	**0**	**5**	**10**	**15**	**20**	**25**	**30**	**35**	**40**	**45**	**50**	**55**	**65**	**75**	**85**	**95**
**Prevalence**	**93.2**	**71.1**	**42.7**	**23.1**	**13.1**	**6.8**	**4.4**	**3.5**	**3.2**	**2.3**	**1.5**	**0.8**	**0.3**	**0.3**	**0.3**	**0.1**
**Lower CI**	90.3	66.3	38.0	19.4	10.4	5.1	3.1	2.4	2.2	1.4	0.9	0.4	0.1	0.1	0.1	0.0
**Upper CI**	95.3	75.4	47.5	27.3	16.4	9.1	6.1	5.1	4.8	3.8	2.7	1.4	0.9	0.8	0.8	0.3
**Number**	421	334	217	132	83	52	38	31	27	19	14	8	4	3	3	1

Table B500-7 Age Group = 51-60 Gender = OVERALL Occupational group = OVERALL Total cases = 682

	Threshold dB HL≥															
	**0**	**5**	**10**	**15**	**20**	**25**	**30**	**35**	**40**	**45**	**50**	**55**	**65**	**75**	**85**	**95**
**Prevalence**	**93.9**	**80.4**	**56.5**	**34.7**	**19.3**	**12.6**	**8.3**	**5.2**	**3.7**	**2.7**	**2.3**	**1.7**	**0.7**	**0.3**	**0.2**	**0.0**
**Lower CI**	91.6	76.8	52.3	31.1	16.6	10.4	6.6	4.0	2.8	1.9	1.6	1.1	0.4	0.1	0.0	0.0
**Upper CI**	95.7	83.6	60.6	38.6	22.4	15.1	10.5	6.9	5.0	3.8	3.2	2.6	1.2	0.6	0.5	0.2
**Number**	645	563	425	290	188	138	95	68	55	42	35	28	13	6	3	1

Prevalence by age group, gender and occupational group for the better ear over 0.5 kHz

Table B500-8 Age Group = 61-70 Gender = OVERALL Occupational group = OVERALL Total cases = 528

	Threshold dB HL≥															
	0	5	10	15	20	25	30	35	40	45	50	55	65	75	85	95
Prevalence	97.2	86.0	66.7	48.9	31.1	20.0	15.1	11.9	8.2	5.9	3.9	2.7	2.0	1.3	1.0	0.5
Lower CI	94.8	82.0	61.6	44.0	26.5	16.4	12.2	9.4	6.1	4.2	3.0	2.0	1.4	0.8	0.6	0.2
Upper CI	98.5	89.2	71.5	53.9	36.1	24.1	18.5	15.0	11.0	8.3	5.1	3.7	2.8	2.1	1.6	1.2
Number	513	465	373	280	200	143	117	96	72	56	45	35	26	15	11	4

Table B500-9 Age Group = 71-80 Gender = OVERALL Occupational group = OVERALL Total cases = 273

	Threshold dB HL≥															
	0	5	10	15	20	25	30	35	40	45	50	55	65	75	85	95
Prevalence	97.5	94.2	85.8	69.7	59.2	39.3	29.3	21.2	15.5	11.3	9.0	5.6	3.6	2.1	1.1	0.4
Lower CI	94.4	89.9	80.6	61.9	51.3	32.2	23.2	16.8	12.1	8.2	6.2	4.1	2.5	1.3	0.6	0.1
Upper CI	98.9	96.7	89.8	76.5	66.7	46.9	36.1	26.4	19.7	15.2	12.9	7.6	5.2	3.5	2.0	1.1
Number	266	259	237	206	173	138	112	92	72	57	45	34	23	13	8	3

Table B500-10 Age Group = 18-80 Gender = OVERALL Occupational group = NON-MANUAL Total cases = 1185

	Threshold dB HL≥															
	0	5	10	15	20	25	30	35	40	45	50	55	65	75	85	95
Prevalence	89.0	67.2	41.6	24.8	15.5	8.7	6.1	3.3	2.6	1.7	1.4	1.0	0.7	0.3	0.2	0.0
Lower CI	86.2	63.8	38.6	22.4	13.5	7.5	5.3	2.7	2.1	1.3	1.1	0.8	0.5	0.2	0.1	0.0
Upper CI	91.2	70.4	44.6	27.4	17.6	10.0	7.1	4.1	3.3	2.2	1.8	1.4	1.1	0.6	0.3	0.2
Number	1085	869	576	383	260	173	128	94	77	56	48	37	27	12	6	2

Table B500-11 Age Group = 18-80 Gender = OVERALL Occupational group = MANUAL Total cases = 1476

	Threshold dB HL≥															
	0	5	10	15	20	25	30	35	40	45	50	55	65	75	85	95
Prevalence	91.8	73.8	49.9	30.0	19.3	12.6	9.0	7.0	4.9	3.6	2.7	1.7	1.1	0.8	0.6	0.4
Lower CI	89.3	70.4	46.6	27.6	17.4	11.1	7.9	6.0	4.0	2.9	2.1	1.3	0.7	0.5	0.4	0.2
Upper CI	93.8	77.0	53.1	32.5	21.3	14.3	10.3	8.1	5.9	4.5	3.4	2.2	1.5	1.2	1.0	0.8
Number	1397	1208	920	646	448	331	255	209	162	127	98	73	41	27	21	8

Table B500-12 Age Group = 18-30 Gender = FEMALE Occupational group = OVERALL Total cases = 224

	Threshold dB HL≥															
	0	5	10	15	20	25	30	35	40	45	50	55	65	75	85	95
Prevalence	85.9	51.4	23.3	9.1	5.4	2.7	1.7	1.2	1.1	0.3	0.2	0.0	0.0	0.0	0.0	0.0
Lower CI	79.8	44.2	18.0	6.1	3.2	1.4	0.8	0.5	0.4	0.1	0.0	0.0	0.0	0.0	0.0	0.0
Upper CI	90.5	58.6	29.6	13.4	8.9	5.1	3.6	3.1	3.0	1.1	1.1	1.7	1.7	1.7	1.7	1.7
Number	198	128	67	33	20	12	8	6	5	2	1	0	0	0	0	0

Table B500-13 Age Group = 31-40 Gender = FEMALE Occupational group = OVERALL Total cases = 190

	Threshold dB HL≥															
	0	5	10	15	20	25	30	35	40	45	50	55	65	75	85	95
Prevalence	88.2	59.5	31.4	14.2	7.4	1.8	1.4	1.1	1.1	1.0	0.7	0.7	0.2	0.2	0.2	0.0
Lower CI	81.5	51.3	24.6	9.5	4.2	0.9	0.7	0.5	0.5	0.4	0.3	0.3	0.0	0.0	0.0	0.0
Upper CI	92.7	67.2	39.1	20.6	12.6	3.4	3.0	2.7	2.7	2.6	2.2	2.2	1.2	1.2	1.2	2.0
Number	172	128	72	36	21	9	7	5	5	4	3	3	1	1	1	0

Table B500-14 Age Group = 41-50 Gender = FEMALE Occupational group = OVERALL Total cases = 247

	Threshold dB HL≥															
	0	5	10	15	20	25	30	35	40	45	50	55	65	75	85	95
Prevalence	91.4	69.3	40.8	22.7	12.5	7.0	4.9	4.6	4.1	2.7	1.7	0.5	0.2	0.1	0.1	0.1
Lower CI	87.0	62.7	34.6	18.0	9.3	4.9	3.2	2.9	2.5	1.4	0.8	0.2	0.1	0.0	0.0	0.0
Upper CI	94.4	75.2	47.2	28.2	16.5	9.8	7.5	7.1	6.7	5.0	3.8	1.4	0.8	0.6	0.6	0.6
Number	228	181	116	73	47	31	23	21	18	12	8	4	2	1	1	1

Prevalence by age group, gender and occupational group for the better ear over 0.5 kHz

Table B500-15 Age Group = 51-60 Gender = FEMALE Occupational group = OVERALL Total cases = 341

Threshold dB HL≥	0	5	10	15	20	25	30	35	40	45	50	55	65	75	85	95
Prevalence	**94.9**	**81.8**	**56.7**	**36.6**	**20.0**	**13.0**	**9.3**	**5.9**	**3.6**	**2.2**	**1.9**	**1.4**	**0.7**	**0.4**	**0.1**	**0.1**
Lower CI	91.7	76.8	50.9	31.3	16.2	10.0	6.8	4.0	2.4	1.4	1.1	0.8	0.3	0.2	0.0	0.0
Upper CI	96.8	85.9	62.4	42.1	24.4	16.8	12.7	8.5	5.3	3.5	3.2	2.4	1.6	0.8	0.5	0.4
Number	324	286	218	154	99	72	53	38	29	21	17	14	7	5	2	1

Table B500-16 Age Group = 61-70 Gender = FEMALE Occupational group = OVERALL Total cases = 243

Threshold dB HL≥	0	5	10	15	20	25	30	35	40	45	50	55	65	75	85	95
Prevalence	**96.9**	**87.0**	**69.1**	**52.5**	**32.4**	**21.6**	**16.7**	**13.9**	**9.7**	**6.7**	**4.0**	**3.1**	**2.2**	**1.4**	**1.3**	**0.7**
Lower CI	92.6	81.3	62.3	46.0	25.9	16.4	12.3	10.1	6.4	4.1	3.0	2.1	1.4	0.7	0.7	0.3
Upper CI	98.7	91.1	75.2	59.0	39.6	27.8	22.2	18.9	14.5	10.5	5.2	4.5	3.5	2.6	2.5	2.1
Number	238	219	181	136	98	72	60	53	42	34	26	21	15	7	6	3

Table B500-17 Age Group = 71-80 Gender = FEMALE Occupational group = OVERALL Total cases = 144

Threshold dB HL≥	0	5	10	15	20	25	30	35	40	45	50	55	65	75	85	95
Prevalence	**99.0**	**97.5**	**90.8**	**74.1**	**65.3**	**43.9**	**34.4**	**23.5**	**17.4**	**12.8**	**10.7**	**6.5**	**5.0**	**3.2**	**1.4**	**0.2**
Lower CI	96.9	91.8	83.7	61.8	53.7	33.9	25.7	18.0	12.8	8.6	6.6	4.4	3.3	1.8	0.7	0.0
Upper CI	99.7	99.3	95.0	83.5	75.3	54.4	44.2	30.1	23.3	18.8	16.7	9.5	7.4	5.5	2.8	1.1
Number	141	140	130	117	103	87	74	60	46	36	29	22	18	11	6	1

Table B500-18 Age Group = 18-30 Gender = MALE Occupational group = OVERALL Total cases = 177

Threshold dB HL≥	0	5	10	15	20	25	30	35	40	45	50	55	65	75	85	95
Prevalence	**81.4**	**55.3**	**28.7**	**8.3**	**2.7**	**0.4**	**0.1**	**0.1**	**0.1**	**0.1**	**0.1**	**0.0**	**0.0**	**0.0**	**0.0**	**0.0**
Lower CI	73.1	46.4	21.5	4.9	1.2	0.1	0.0	0.0	0.0	0.0	0.0	0.0	0.0	0.0	0.0	0.0
Upper CI	87.7	64.0	37.3	13.6	6.4	1.0	0.2	0.2	0.2	0.2	0.2	0.2	2.1	2.1	2.1	2.1
Number	150	107	56	23	11	4	2	2	2	2	2	1	0	0	0	0

Table B500-19 Age Group = 31-40 Gender = MALE Occupational group = OVERALL Total cases = 142

Threshold dB HL≥	0	5	10	15	20	25	30	35	40	45	50	55	65	75	85	95
Prevalence	**83.2**	**63.3**	**32.0**	**17.3**	**7.5**	**4.0**	**2.0**	**1.6**	**1.2**	**1.2**	**1.2**	**1.2**	**1.2**	**1.2**	**1.2**	**1.2**
Lower CI	73.8	52.9	23.3	10.9	3.9	1.6	0.6	0.4	0.2	0.2	0.2	0.2	0.2	0.2	0.2	0.2
Upper CI	89.6	72.6	42.1	26.4	14.0	9.4	6.4	6.3	6.5	6.5	6.5	6.5	6.5	6.5	6.5	6.5
Number	122	97	52	32	14	8	4	3	1	1	1	1	1	1	1	1

Table B500-20 Age Group = 41-50 Gender = MALE Occupational group = OVERALL Total cases = 203

Threshold dB HL≥	0	5	10	15	20	25	30	35	40	45	50	55	65	75	85	95
Prevalence	**95.1**	**72.9**	**44.8**	**23.5**	**13.8**	**6.7**	**3.7**	**2.4**	**2.3**	**1.9**	**1.3**	**1.0**	**0.4**	**0.4**	**0.4**	**0.0**
Lower CI	90.6	65.7	37.8	18.0	9.7	4.2	2.2	1.2	1.1	0.9	0.7	0.5	0.1	0.1	0.1	0.0
Upper CI	97.5	79.1	52.1	30.1	19.4	10.6	6.2	4.5	4.4	4.0	2.6	2.2	1.6	1.6	1.6	1.9
Number	193	153	101	59	36	21	15	10	9	7	6	4	2	2	2	0

Table B500-21 Age Group = 51-60 Gender = MALE Occupational group = OVERALL Total cases = 341

Threshold dB HL≥	0	5	10	15	20	25	30	35	40	45	50	55	65	75	85	95
Prevalence	**92.9**	**78.9**	**56.2**	**32.7**	**18.6**	**12.1**	**7.2**	**4.5**	**3.8**	**3.3**	**2.6**	**2.0**	**0.7**	**0.2**	**0.2**	**0.0**
Lower CI	88.9	73.4	50.2	27.6	14.8	9.2	5.1	3.1	2.5	2.0	1.6	1.1	0.3	0.0	0.0	0.0
Upper CI	95.6	83.5	62.1	38.2	23.0	15.7	10.1	6.6	5.8	5.3	4.4	3.6	1.5	1.0	1.0	1.1
Number	321	277	207	136	89	66	42	30	26	21	18	14	6	1	1	0

Prevalence by age group, gender and occupational group for the better ear over 0.5 kHz

Table B500-22 Age Group = 61-70 Gender = MALE Occupational group = OVERALL Total cases = 285

	Threshold dB HL≥															
	0	5	10	15	20	25	30	35	40	45	50	55	65	75	85	95
Prevalence	**97.6**	**85.1**	**64.1**	**44.9**	**29.7**	**18.2**	**13.3**	**9.5**	**6.6**	**5.1**	**3.9**	**2.3**	**1.7**	**1.2**	**0.6**	**0.1**
Lower CI	95.1	79.0	56.3	37.6	23.5	13.7	10.1	6.9	4.5	3.3	2.4	1.4	1.0	0.6	0.3	0.0
Upper CI	98.8	89.6	71.2	52.4	36.8	23.8	17.3	12.9	9.5	8.0	6.3	3.8	3.0	2.5	1.3	0.8
Number	275	246	192	144	102	71	57	43	30	22	19	14	11	8	5	1

Table B500-23 Age Group = 71-80 Gender = MALE Occupational group = OVERALL Total cases = 129

	Threshold dB HL≥															
	0	5	10	15	20	25	30	35	40	45	50	55	65	75	85	95
Prevalence	**95.0**	**88.8**	**77.6**	**62.1**	**49.2**	**32.6**	**21.4**	**17.7**	**12.7**	**9.1**	**6.6**	**4.3**	**1.7**	**0.6**	**0.6**	**0.6**
Lower CI	87.0	79.9	68.6	53.6	39.3	23.7	14.8	11.7	8.6	6.0	4.1	2.6	0.8	0.2	0.2	0.2
Upper CI	98.2	94.1	84.7	69.8	59.0	42.9	29.9	25.8	18.4	13.5	10.5	7.1	4.0	2.3	2.3	2.3
Number	125	119	107	89	70	51	38	32	26	21	16	12	5	2	2	2

Table B500-24 Age Group = 18-30 Gender = OVERALL Occupational group = NON-MANUAL Total cases = 181

	Threshold dB HL≥															
	0	5	10	15	20	25	30	35	40	45	50	55	65	75	85	95
Prevalence	**79.2**	**47.9**	**23.2**	**7.9**	**3.6**	**1.0**	**0.6**	**0.2**	**0.2**	**0.1**	**0.1**	**0.0**	**0.0**	**0.0**	**0.0**	**0.0**
Lower CI	71.0	39.4	16.8	4.4	1.4	0.3	0.2	0.1	0.1	0.0	0.0	0.0	0.0	0.0	0.0	0.0
Upper CI	85.6	56.4	31.2	13.8	9.2	3.0	2.0	0.7	0.7	2.2	2.2	2.1	2.1	2.1	2.1	2.1
Number	153	93	47	19	9	4	3	2	2	1	1	0	0	0	0	0

Table B500-25 Age Group = 31-40 Gender = OVERALL Occupational group = NON-MANUAL Total cases = 148

	Threshold dB HL≥															
	0	5	10	15	20	25	30	35	40	45	50	55	65	75	85	95
Prevalence	**86.9**	**59.2**	**31.0**	**12.6**	**7.0**	**1.6**	**0.7**	**0.4**	**0.4**	**0.3**	**0.3**	**0.3**	**0.0**	**0.0**	**0.0**	**0.0**
Lower CI	78.7	49.9	24.2	7.8	3.6	0.6	0.2	0.1	0.1	0.1	0.1	0.1	0.0	0.0	0.0	0.0
Upper CI	92.3	68.0	38.8	19.6	13.3	4.2	2.1	1.6	1.6	1.7	1.7	1.7	2.5	2.5	2.5	2.5
Number	132	95	48	23	12	5	3	2	2	1	1	1	0	0	0	0

Table B500-26 Age Group = 41-50 Gender = OVERALL Occupational group = NON-MANUAL Total cases = 188

	Threshold dB HL≥															
	0	5	10	15	20	25	30	35	40	45	50	55	65	75	85	95
Prevalence	**94.7**	**67.5**	**29.8**	**16.7**	**8.3**	**5.4**	**3.2**	**0.7**	**0.4**	**0.1**	**0.1**	**0.0**	**0.0**	**0.0**	**0.0**	**0.0**
Lower CI	90.9	59.8	23.6	11.9	5.5	3.2	1.5	0.4	0.2	0.0	0.0	0.0	0.0	0.0	0.0	0.0
Upper CI	96.9	74.4	36.9	22.9	12.5	9.0	6.7	1.1	0.7	0.7	0.7	2.0	2.0	2.0	2.0	2.0
Number	174	126	59	35	18	11	7	4	2	1	1	0	0	0	0	0

Table B500-27 Age Group = 51-60 Gender = OVERALL Occupational group = NON-MANUAL Total cases = 317

	Threshold dB HL≥															
	0	5	10	15	20	25	30	35	40	45	50	55	65	75	85	95
Prevalence	**91.8**	**76.3**	**47.9**	**29.3**	**15.0**	**7.4**	**4.6**	**2.5**	**2.1**	**1.4**	**1.2**	**1.1**	**0.6**	**0.3**	**0.2**	**0.0**
Lower CI	87.8	71.1	41.3	23.7	11.7	5.5	3.1	1.7	1.3	0.8	0.6	0.5	0.2	0.1	0.1	0.0
Upper CI	94.5	80.8	54.6	35.5	19.1	10.1	6.7	3.8	3.2	2.4	2.3	2.0	1.6	1.0	0.8	1.2
Number	290	240	166	112	72	45	31	20	17	12	10	9	5	3	2	0

Table B500-28 Age Group = 61-70 Gender = OVERALL Occupational group = NON-MANUAL Total cases = 228

	Threshold dB HL≥															
	0	5	10	15	20	25	30	35	40	45	50	55	65	75	85	95
Prevalence	**95.7**	**86.1**	**68.4**	**49.2**	**31.8**	**19.3**	**12.7**	**9.6**	**8.4**	**5.8**	**4.4**	**3.2**	**2.2**	**1.3**	**0.5**	**0.3**
Lower CI	91.6	80.3	61.8	43.1	26.2	13.6	9.5	6.8	5.8	3.8	2.9	2.1	1.2	0.5	0.2	0.1
Upper CI	97.9	90.4	74.3	55.4	38.0	26.7	16.7	13.5	12.2	8.8	6.8	5.0	4.0	3.3	1.4	1.2
Number	218	201	154	108	80	56	44	35	31	24	20	16	13	6	3	2

Prevalence by age group, gender and occupational group for the better ear over 0.5 kHz

Table B500-29 Age Group = 71-80 Gender = OVERALL Occupational group = NON-MANUAL Total cases = 123

	Threshold dB HL≥															
	0	5	10	15	20	25	30	35	40	45	50	55	65	75	85	95
**Prevalence**	**97.1**	**92.8**	**83.2**	**66.7**	**54.9**	**36.1**	**29.3**	**14.2**	**9.6**	**6.2**	**5.5**	**4.0**	**3.3**	**1.1**	**0.4**	**0.0**
Lower CI	93.1	85.7	75.5	55.0	43.9	29.2	23.7	9.6	6.5	4.1	3.5	2.3	1.8	0.4	0.1	0.0
Upper CI	98.8	96.5	88.8	76.7	65.5	43.6	35.6	20.6	14.0	9.4	8.7	6.8	6.2	2.9	2.0	3.0
Number	118	114	102	86	69	52	40	31	23	17	15	11	9	3	1	0

Table B500-30 Age Group = 18-30 Gender = OVERALL Occupational group = MANUAL Total cases = 218

	Threshold dB HL≥															
	0	5	10	15	20	25	30	35	40	45	50	55	65	75	85	95
**Prevalence**	**86.7**	**60.2**	**29.4**	**9.1**	**5.5**	**2.3**	**1.2**	**1.1**	**1.0**	**0.2**	**0.2**	**0.0**	**0.0**	**0.0**	**0.0**	**0.0**
Lower CI	79.1	51.1	22.2	5.9	3.1	1.1	0.4	0.3	0.3	0.1	0.0	0.0	0.0	0.0	0.0	0.0
Upper CI	91.8	68.6	37.8	13.9	9.7	4.8	3.2	3.2	3.2	0.8	0.7	0.2	1.7	1.7	1.7	1.7
Number	193	141	76	37	22	12	7	6	5	3	2	1	0	0	0	0

Table B500-31 Age Group = 31-40 Gender = OVERALL Occupational group = MANUAL Total cases = 183

	Threshold dB HL≥															
	0	5	10	15	20	25	30	35	40	45	50	55	65	75	85	95
**Prevalence**	**85.6**	**62.9**	**31.3**	**16.4**	**7.1**	**4.0**	**2.2**	**2.0**	**1.6**	**1.6**	**1.3**	**1.3**	**1.1**	**1.1**	**1.1**	**0.9**
Lower CI	77.3	53.1	23.6	11.0	4.0	1.7	0.9	0.7	0.5	0.5	0.3	0.3	0.2	0.2	0.2	0.2
Upper CI	91.2	71.7	40.3	23.8	12.5	9.1	5.4	5.3	5.0	5.0	4.8	4.8	4.9	4.9	4.9	5.0
Number	161	129	75	44	22	12	8	6	4	4	3	3	2	2	2	1

Table B500-32 Age Group = 41-50 Gender = OVERALL Occupational group = MANUAL Total cases = 262

	Threshold dB HL≥															
	0	5	10	15	20	25	30	35	40	45	50	55	65	75	85	95
**Prevalence**	**91.8**	**74.5**	**55.2**	**29.4**	**17.1**	**8.8**	**5.4**	**4.9**	**4.7**	**3.4**	**2.2**	**1.0**	**0.4**	**0.3**	**0.3**	**0.1**
Lower CI	86.6	67.5	48.1	23.5	12.8	6.2	3.7	3.2	3.0	2.0	1.1	0.5	0.2	0.1	0.1	0.0
Upper CI	95.1	80.5	62.1	36.0	22.5	12.3	7.8	7.4	7.1	5.6	4.3	1.8	1.1	0.9	0.9	0.5
Number	247	208	158	97	65	41	31	27	25	18	13	8	4	3	3	1

Table B500-33 Age Group = 51-60 Gender = OVERALL Occupational group = MANUAL Total cases = 365

	Threshold dB HL≥															
	0	5	10	15	20	25	30	35	40	45	50	55	65	75	85	95
**Prevalence**	**96.7**	**85.5**	**63.6**	**39.9**	**23.1**	**17.5**	**11.8**	**7.0**	**4.5**	**3.4**	**2.7**	**1.8**	**0.7**	**0.2**	**0.1**	**0.1**
Lower CI	93.6	80.3	57.3	34.3	18.8	13.8	8.8	5.1	3.1	2.2	1.8	1.1	0.4	0.1	0.0	0.0
Upper CI	98.4	89.5	69.4	45.8	28.1	21.9	15.7	9.6	6.4	5.0	4.1	2.9	1.4	0.6	0.5	0.5
Number	355	323	259	178	116	93	64	48	38	30	25	19	8	3	1	1

Table B500-34 Age Group = 61-70 Gender = OVERALL Occupational group = MANUAL Total cases = 299

	Threshold dB HL≥															
	0	5	10	15	20	25	30	35	40	45	50	55	65	75	85	95
**Prevalence**	**98.6**	**85.0**	**63.7**	**49.5**	**30.7**	**21.0**	**17.5**	**14.1**	**8.7**	**6.5**	**4.0**	**3.1**	**2.1**	**1.6**	**1.5**	**1.0**
Lower CI	94.7	78.3	55.9	42.5	25.0	16.8	13.6	10.8	5.7	4.2	3.0	2.2	1.5	1.2	1.1	0.8
Upper CI	99.6	89.8	70.8	56.7	37.0	25.9	22.3	18.2	12.9	10.0	5.3	4.3	2.9	2.1	2.0	1.1
Number	294	263	218	171	119	87	73	61	41	32	25	19	13	9	8	2

Table B500-35 Age Group = 71-80 Gender = OVERALL Occupational group = MANUAL Total cases = 149

	Threshold dB HL≥															
	0	5	10	15	20	25	30	35	40	45	50	55	65	75	85	95
**Prevalence**	**97.5**	**94.8**	**89.3**	**73.1**	**64.5**	**46.2**	**35.2**	**27.6**	**19.1**	**15.3**	**12.0**	**6.1**	**3.9**	**2.9**	**1.6**	**0.6**
Lower CI	88.1	86.0	79.7	62.2	53.8	35.8	28.0	20.9	12.9	9.5	6.6	4.3	2.5	1.7	0.8	0.2
Upper CI	99.5	98.2	94.7	81.8	74.0	57.1	43.0	35.5	27.3	23.7	20.6	8.7	6.2	5.1	3.1	1.8
Number	147	144	134	119	104	86	72	61	49	40	30	23	14	10	7	3

Prevalence by age group, gender and occupational group for the better ear over 0.5 kHz

Table B500-36 Age Group = 18-80 Gender = FEMALE Occupational group = NON-MANUAL Total cases = 671

	Threshold dB HL≥ 0	5	10	15	20	25	30	35	40	45	50	55	65	75	85	95
**Prevalence**	**90.3**	**67.1**	**42.1**	**27.0**	**16.6**	**10.1**	**7.5**	**4.1**	**3.0**	**2.0**	**1.6**	**1.4**	**1.1**	**0.4**	**0.2**	**0.1**
**Lower CI**	87.4	63.3	38.6	24.0	14.1	8.6	6.4	3.2	2.3	1.5	1.2	1.0	0.7	0.2	0.1	0.0
**Upper CI**	92.6	70.6	45.6	30.3	19.4	11.9	8.8	5.2	3.9	2.6	2.2	2.0	1.6	0.8	0.6	0.4
**Number**	614	489	330	228	153	107	82	67	53	39	33	29	22	9	5	2

Table B500-37 Age Group = 18-80 Gender = MALE Occupational group = NON-MANUAL Total cases = 514

	Threshold dB HL≥ 0	5	10	15	20	25	30	35	40	45	50	55	65	75	85	95
**Prevalence**	**87.5**	**67.4**	**41.0**	**22.3**	**14.2**	**7.1**	**4.6**	**2.5**	**2.3**	**1.5**	**1.2**	**0.6**	**0.4**	**0.3**	**0.1**	**0.0**
**Lower CI**	82.4	61.6	36.1	18.7	11.4	5.4	3.4	1.7	1.5	0.9	0.7	0.3	0.1	0.1	0.0	0.0
**Upper CI**	91.3	72.6	46.1	26.4	17.4	9.4	6.0	3.7	3.5	2.4	1.9	1.1	0.9	0.8	0.3	0.7
**Number**	471	380	246	155	107	66	46	27	24	17	15	8	5	3	1	0

Table B500-38 Age Group = 18-80 Gender = FEMALE Occupational group = MANUAL Total cases = 716

	Threshold dB HL≥ 0	5	10	15	20	25	30	35	40	45	50	55	65	75	85	95
**Prevalence**	**93.5**	**75.2**	**50.8**	**31.8**	**21.6**	**13.9**	**10.7**	**8.0**	**6.1**	**4.2**	**3.0**	**1.7**	**1.1**	**0.9**	**0.7**	**0.3**
**Lower CI**	90.0	70.6	46.6	28.4	18.8	11.7	9.0	6.5	4.7	3.1	2.1	1.3	0.8	0.7	0.5	0.3
**Upper CI**	95.8	79.3	55.0	35.3	24.8	16.4	12.6	9.7	7.8	5.6	4.3	2.2	1.5	1.3	0.9	0.5
**Number**	685	591	452	319	233	176	143	116	92	70	51	35	21	16	11	4

Table B500-39 Age Group = 18-80 Gender = MALE Occupational group = MANUAL Total cases = 760

	Threshold dB HL≥ 0	5	10	15	20	25	30	35	40	45	50	55	65	75	85	95
**Prevalence**	**90.0**	**72.4**	**48.7**	**28.0**	**16.7**	**11.2**	**7.1**	**5.8**	**3.6**	**3.0**	**2.2**	**1.8**	**1.0**	**0.6**	**0.6**	**0.4**
**Lower CI**	85.9	67.1	43.8	24.6	14.2	9.3	5.9	4.8	2.7	2.2	1.6	1.2	0.5	0.2	0.2	0.1
**Upper CI**	93.0	77.1	53.7	31.7	19.4	13.4	8.6	7.1	4.7	4.0	3.2	2.7	1.9	1.6	1.6	1.6
**Number**	712	617	468	327	215	155	112	93	70	57	47	38	20	11	10	4

Table B500-40 Age Group = 18-30 Gender = FEMALE Occupational group = NON-MANUAL Total cases = 115

	Threshold dB HL≥ 0	5	10	15	20	25	30	35	40	45	50	55	65	75	85	95
**Prevalence**	**84.1**	**48.1**	**21.5**	**7.3**	**3.3**	**1.8**	**1.0**	**0.3**	**0.3**	**0.0**	**0.0**	**0.0**	**0.0**	**0.0**	**0.0**	**0.0**
**Lower CI**	75.6	39.3	15.1	4.0	1.5	0.6	0.2	0.0	0.0	0.0	0.0	0.0	0.0	0.0	0.0	0.0
**Upper CI**	90.0	57.0	29.6	12.7	7.1	5.5	3.8	1.5	1.5	3.2	3.2	3.2	3.2	3.2	3.2	3.2
**Number**	99	60	30	12	6	3	2	1	1	0	0	0	0	0	0	0

Table B500-41 Age Group = 31-40 Gender = FEMALE Occupational group = NON-MANUAL Total cases = 86

	Threshold dB HL≥ 0	5	10	15	20	25	30	35	40	45	50	55	65	75	85	95
**Prevalence**	**87.0**	**53.3**	**25.1**	**12.9**	**6.9**	**0.7**	**0.7**	**0.7**	**0.7**	**0.6**	**0.6**	**0.6**	**0.0**	**0.0**	**0.0**	**0.0**
**Lower CI**	77.9	41.8	16.5	7.0	2.7	0.2	0.2	0.2	0.2	0.1	0.1	0.1	0.0	0.0	0.0	0.0
**Upper CI**	92.7	64.4	36.2	22.7	16.3	3.0	3.0	3.0	3.0	3.2	3.2	3.2	4.3	4.3	4.3	4.3
**Number**	76	50	23	12	6	2	2	2	2	1	1	1	0	0	0	0

Table B500-42 Age Group = 41-50 Gender = FEMALE Occupational group = NON-MANUAL Total cases = 112

	Threshold dB HL≥ 0	5	10	15	20	25	30	35	40	45	50	55	65	75	85	95
**Prevalence**	**90.4**	**64.2**	**29.3**	**18.2**	**9.0**	**5.1**	**0.8**	**0.8**	**0.6**	**0.0**	**0.0**	**0.0**	**0.0**	**0.0**	**0.0**	**0.0**
**Lower CI**	83.5	54.6	22.0	12.8	5.8	2.9	0.4	0.4	0.1	0.0	0.0	0.0	0.0	0.0	0.0	0.0
**Upper CI**	94.6	72.7	37.9	25.1	13.8	8.7	1.5	1.5	4.3	3.3	3.3	3.3	3.3	3.3	3.3	3.3
**Number**	100	74	36	22	11	6	2	2	1	0	0	0	0	0	0	0

Prevalence by age group, gender and occupational group for the better ear over 0.5 kHz

Table B500-43 Age Group = 51-60 Gender = FEMALE Occupational group = NON-MANUAL Total cases = 175

Threshold dB HL≥	0	5	10	15	20	25	30	35	40	45	50	55	65	75	85	95
Prevalence	**93.6**	**79.2**	**49.9**	**30.8**	**16.0**	**8.5**	**5.9**	**3.7**	**2.8**	**1.8**	**1.4**	**1.4**	**0.8**	**0.3**	**0.1**	**0.0**
Lower CI	89.1	72.7	41.9	24.1	11.8	5.6	3.5	2.3	1.7	0.9	0.6	0.6	0.2	0.1	0.0	0.0
Upper CI	96.4	84.4	57.9	38.4	21.4	12.8	9.5	5.7	4.6	3.5	3.2	3.2	2.5	1.2	0.7	2.1
Number	163	137	96	66	43	27	20	15	12	8	6	6	3	2	1	0

Table B500-44 Age Group = 61-70 Gender = FEMALE Occupational group = NON-MANUAL Total cases = 116

Threshold dB HL≥	0	5	10	15	20	25	30	35	40	45	50	55	65	75	85	95
Prevalence	**95.8**	**86.8**	**70.1**	**53.2**	**26.8**	**17.4**	**11.9**	**9.4**	**7.7**	**5.0**	**3.7**	**3.0**	**2.7**	**1.1**	**1.0**	**0.6**
Lower CI	89.1	79.3	60.9	44.5	19.0	11.5	7.6	6.0	4.6	3.3	2.5	1.8	1.6	0.5	0.4	0.1
Upper CI	98.5	91.9	77.9	61.7	36.3	25.3	18.0	14.6	12.6	7.4	5.5	4.9	4.6	2.7	2.6	2.3
Number	112	105	86	64	43	32	26	23	20	17	14	12	11	4	3	2

Table B500-45 Age Group = 71-80 Gender = FEMALE Occupational group = NON-MANUAL Total cases = 67

Threshold dB HL≥	0	5	10	15	20	25	30	35	40	45	50	55	65	75	85	95
Prevalence	**97.9**	**95.2**	**89.9**	**73.4**	**64.4**	**45.8**	**40.2**	**17.1**	**10.4**	**7.6**	**7.0**	**6.0**	**5.0**	**1.8**	**0.6**	**0.0**
Lower CI	92.6	85.0	79.0	55.0	47.8	36.8	32.4	11.0	7.4	4.8	4.2	3.3	2.6	0.7	0.1	0.0
Upper CI	99.4	98.6	95.4	86.1	78.1	55.0	48.5	25.8	14.6	11.9	11.5	10.7	9.6	4.9	3.4	5.4
Number	64	63	59	52	44	37	30	24	17	13	12	10	8	3	1	0

Table B500-46 Age Group = 18-30 Gender = MALE Occupational group = NON-MANUAL Total cases = 66

Threshold dB HL≥	0	5	10	15	20	25	30	35	40	45	50	55	65	75	85	95
Prevalence	**74.0**	**47.7**	**24.9**	**8.5**	**3.8**	**0.1**	**0.1**	**0.1**	**0.1**	**0.1**	**0.1**	**0.0**	**0.0**	**0.0**	**0.0**	**0.0**
Lower CI	59.4	33.7	14.5	3.3	0.8	0.0	0.0	0.0	0.0	0.0	0.0	0.0	0.0	0.0	0.0	0.0
Upper CI	84.7	62.1	39.3	20.3	17.1	5.7	5.7	5.7	5.7	5.7	5.7	5.5	5.5	5.5	5.5	5.5
Number	54	33	17	7	3	1	1	1	1	1	1	0	0	0	0	0

Table B500-47 Age Group = 31-40 Gender = MALE Occupational group = NON-MANUAL Total cases = 62

Threshold dB HL≥	0	5	10	15	20	25	30	35	40	45	50	55	65	75	85	95
Prevalence	**86.4**	**65.6**	**38.0**	**12.4**	**7.2**	**2.5**	**0.6**	**0.0**	**0.0**	**0.0**	**0.0**	**0.0**	**0.0**	**0.0**	**0.0**	**0.0**
Lower CI	70.5	50.2	27.8	5.9	2.8	0.8	0.1	0.0	0.0	0.0	0.0	0.0	0.0	0.0	0.0	0.0
Upper CI	94.4	78.3	49.3	24.2	17.7	7.7	3.5	5.8	5.8	5.8	5.8	5.8	5.8	5.8	5.8	5.8
Number	56	45	25	11	6	3	1	0	0	0	0	0	0	0	0	0

Table B500-48 Age Group = 41-50 Gender = MALE Occupational group = NON-MANUAL Total cases = 76

Threshold dB HL≥	0	5	10	15	20	25	30	35	40	45	50	55	65	75	85	95
Prevalence	**99.2**	**71.2**	**30.3**	**15.1**	**7.6**	**5.8**	**5.8**	**0.5**	**0.3**	**0.3**	**0.3**	**0.0**	**0.0**	**0.0**	**0.0**	**0.0**
Lower CI	96.8	58.4	20.8	7.9	3.6	2.4	2.4	0.2	0.0	0.0	0.0	0.0	0.0	0.0	0.0	0.0
Upper CI	99.8	81.3	42.0	26.7	15.6	13.3	13.3	1.3	1.4	1.4	1.4	4.8	4.8	4.8	4.8	4.8
Number	74	52	23	13	7	5	5	2	1	1	1	0	0	0	0	0

Table B500-49 Age Group = 51-60 Gender = MALE Occupational group = NON-MANUAL Total cases = 142

Threshold dB HL≥	0	5	10	15	20	25	30	35	40	45	50	55	65	75	85	95
Prevalence	**89.7**	**73.0**	**45.6**	**27.5**	**13.9**	**6.2**	**3.1**	**1.3**	**1.3**	**1.0**	**1.0**	**0.6**	**0.5**	**0.3**	**0.3**	**0.0**
Lower CI	82.3	64.5	35.1	19.1	9.2	4.0	1.7	0.5	0.5	0.4	0.4	0.2	0.1	0.1	0.1	0.0
Upper CI	94.2	80.2	56.5	37.9	20.5	9.5	5.6	3.1	3.1	2.5	2.5	1.8	1.8	1.7	1.7	2.6
Number	127	103	70	46	29	18	11	5	5	4	4	3	2	1	1	0

Prevalence by age group, gender and occupational group for the better ear over 0.5 kHz

Table B500-50 Age Group = 61-70 Gender = MALE Occupational group = NON-MANUAL Total cases = 112

	Threshold dB HL≥															
	**0**	**5**	**10**	**15**	**20**	**25**	**30**	**35**	**40**	**45**	**50**	**55**	**65**	**75**	**85**	**95**
**Prevalence**	**95.8**	**86.0**	**67.2**	**45.8**	**38.2**	**22.4**	**14.1**	**10.1**	**9.5**	**6.9**	**5.5**	**3.7**	**1.6**	**1.6**	**0.0**	**0.0**
**Lower CI**	90.1	76.5	57.6	37.4	30.7	12.7	9.6	5.9	5.4	3.4	2.7	1.8	0.4	0.4	0.0	0.0
**Upper CI**	98.3	92.0	75.5	54.5	46.3	36.5	20.3	16.8	16.1	13.3	10.9	7.3	6.5	6.5	3.3	3.3
**Number**	106	96	68	44	37	24	18	12	11	7	6	4	2	2	0	0

Table B500-51 Age Group = 71-80 Gender = MALE Occupational group = NON-MANUAL Total cases = 56

	Threshold dB HL≥															
	**0**	**5**	**10**	**15**	**20**	**25**	**30**	**35**	**40**	**45**	**50**	**55**	**65**	**75**	**85**	**95**
**Prevalence**	**95.8**	**89.1**	**72.5**	**56.0**	**39.9**	**22.2**	**13.6**	**9.7**	**8.6**	**4.5**	**3.6**	**1.0**	**1.0**	**0.0**	**0.0**	**0.0**
**Lower CI**	87.2	75.6	59.5	44.2	28.3	13.2	7.6	4.7	3.6	1.8	1.3	0.2	0.2	0.0	0.0	0.0
**Upper CI**	98.7	95.5	82.5	67.2	52.7	34.9	23.1	19.2	19.1	11.0	9.7	5.3	5.3	6.4	6.4	6.4
**Number**	54	51	43	34	25	15	10	7	6	4	3	1	1	0	0	0

Table B500-52 Age Group = 18-30 Gender = FEMALE Occupational group = MANUAL Total cases = 109

	Threshold dB HL≥															
	**0**	**5**	**10**	**15**	**20**	**25**	**30**	**35**	**40**	**45**	**50**	**55**	**65**	**75**	**85**	**95**
**Prevalence**	**87.8**	**58.3**	**25.7**	**10.8**	**8.6**	**3.9**	**2.2**	**1.9**	**1.7**	**0.4**	**0.3**	**0.0**	**0.0**	**0.0**	**0.0**	**0.0**
**Lower CI**	76.2	45.7	16.9	6.1	4.3	1.7	0.8	0.6	0.5	0.1	0.0	0.0	0.0	0.0	0.0	0.0
**Upper CI**	94.1	69.9	37.1	18.4	16.4	8.6	6.0	5.9	5.8	1.4	1.4	3.4	3.4	3.4	3.4	3.4
**Number**	99	68	37	21	14	9	6	5	4	2	1	0	0	0	0	0

Table B500-53 Age Group = 31-40 Gender = FEMALE Occupational group = MANUAL Total cases = 103

	Threshold dB HL≥															
	**0**	**5**	**10**	**15**	**20**	**25**	**30**	**35**	**40**	**45**	**50**	**55**	**65**	**75**	**85**	**95**
**Prevalence**	**89.1**	**64.7**	**35.6**	**12.4**	**4.7**	**2.3**	**1.7**	**1.2**	**1.2**	**1.2**	**0.6**	**0.6**	**0.3**	**0.3**	**0.3**	**0.0**
**Lower CI**	78.3	52.6	25.8	7.6	2.9	1.1	0.7	0.4	0.4	0.4	0.2	0.2	0.1	0.1	0.1	0.0
**Upper CI**	94.9	75.1	46.8	19.7	7.5	4.8	4.1	3.8	3.8	3.8	2.3	2.3	1.7	1.7	1.7	3.6
**Number**	95	77	48	23	14	7	5	3	3	3	2	2	1	1	1	0

Table B500-54 Age Group = 41-50 Gender = FEMALE Occupational group = MANUAL Total cases = 135

	Threshold dB HL≥															
	**0**	**5**	**10**	**15**	**20**	**25**	**30**	**35**	**40**	**45**	**50**	**55**	**65**	**75**	**85**	**95**
**Prevalence**	**93.7**	**76.2**	**53.7**	**28.5**	**16.1**	**8.6**	**7.5**	**7.0**	**6.5**	**4.5**	**3.0**	**0.9**	**0.4**	**0.2**	**0.2**	**0.2**
**Lower CI**	87.2	66.7	44.3	20.9	11.0	5.7	4.7	4.3	3.9	2.4	1.2	0.3	0.1	0.0	0.0	0.0
**Upper CI**	97.0	83.7	62.9	37.4	22.9	12.9	11.8	11.3	10.8	8.3	7.0	2.3	1.4	1.0	1.0	1.0
**Number**	128	107	80	51	36	25	21	19	17	12	8	4	2	1	1	1

Table B500-55 Age Group = 51-60 Gender = FEMALE Occupational group = MANUAL Total cases = 166

	Threshold dB HL≥															
	**0**	**5**	**10**	**15**	**20**	**25**	**30**	**35**	**40**	**45**	**50**	**55**	**65**	**75**	**85**	**95**
**Prevalence**	**98.0**	**86.3**	**63.8**	**44.5**	**25.4**	**18.9**	**13.5**	**7.5**	**3.8**	**2.3**	**2.0**	**1.1**	**0.6**	**0.4**	**0.2**	**0.2**
**Lower CI**	94.8	78.3	54.4	35.9	18.8	13.5	9.0	4.7	2.2	1.3	1.1	0.6	0.3	0.1	0.0	0.0
**Upper CI**	99.2	91.6	72.2	53.6	33.4	25.9	19.7	11.9	6.6	4.1	3.9	1.8	1.3	1.2	1.0	1.0
**Number**	161	149	122	88	56	45	33	23	17	13	11	8	4	3	1	1

Table B500-56 Age Group = 61-70 Gender = FEMALE Occupational group = MANUAL Total cases = 126

	Threshold dB HL≥															
	**0**	**5**	**10**	**15**	**20**	**25**	**30**	**35**	**40**	**45**	**50**	**55**	**65**	**75**	**85**	**95**
**Prevalence**	**98.1**	**86.6**	**66.4**	**52.0**	**34.5**	**23.8**	**20.9**	**17.2**	**11.0**	**8.0**	**4.7**	**3.9**	**2.3**	**2.1**	**2.1**	**1.6**
**Lower CI**	90.1	77.0	55.5	42.0	26.0	17.4	14.9	12.4	6.4	4.5	3.4	2.6	1.7	1.6	1.6	0.5
**Upper CI**	99.7	92.6	75.7	61.8	44.1	31.6	28.4	23.4	18.4	13.8	6.6	5.9	3.1	2.8	2.8	5.7
**Number**	125	113	94	71	54	40	34	30	22	17	12	9	4	3	3	1

Prevalence by age group, gender and occupational group for the better ear over 0.5 kHz

Table B500-57 Age Group = 71-80 Gender = FEMALE Occupational group = MANUAL Total cases = 77

Threshold dB HL≥	0	5	10	15	20	25	30	35	40	45	50	55	65	75	85	95
**Prevalence**	**100.0**	**100.0**	**93.3**	**77.1**	**69.9**	**46.0**	**35.9**	**25.5**	**22.0**	**17.9**	**14.4**	**6.1**	**5.0**	**4.3**	**2.0**	**0.4**
**Lower CI**	95.2	95.2	82.1	60.8	54.6	31.5	25.7	16.0	12.9	9.3	6.6	3.7	2.9	2.3	0.9	0.1
**Upper CI**	100.0	100.0	97.7	88.0	81.8	61.1	47.5	38.1	34.9	31.5	28.8	9.9	8.5	7.8	4.2	2.1
**Number**	77	77	71	65	59	50	44	36	29	23	17	12	10	8	5	1

Table B500-58 Age Group = 18-30 Gender = MALE Occupational group = MANUAL Total cases = 109

Threshold dB HL≥	0	5	10	15	20	25	30	35	40	45	50	55	65	75	85	95
**Prevalence**	**85.7**	**62.3**	**33.3**	**7.3**	**2.2**	**0.5**	**0.1**	**0.1**	**0.1**	**0.1**	**0.1**	**0.1**	**0.0**	**0.0**	**0.0**	**0.0**
**Lower CI**	74.5	49.3	22.6	3.7	0.9	0.1	0.0	0.0	0.0	0.0	0.0	0.0	0.0	0.0	0.0	0.0
**Upper CI**	92.5	73.7	46.0	13.9	5.6	1.6	0.4	0.4	0.4	0.4	0.4	0.4	3.4	3.4	3.4	3.4
**Number**	94	73	39	16	8	3	1	1	1	1	1	1	0	0	0	0

Table B500-59 Age Group = 31-40 Gender = MALE Occupational group = MANUAL Total cases = 80

Threshold dB HL≥	0	5	10	15	20	25	30	35	40	45	50	55	65	75	85	95
**Prevalence**	**81.8**	**60.5**	**26.4**	**21.1**	**10.2**	**6.1**	**2.8**	**2.8**	**1.9**	**1.9**	**1.9**	**1.9**	**1.9**	**1.9**	**1.9**	**1.9**
**Lower CI**	68.0	44.9	15.4	11.6	4.3	1.9	0.7	0.7	0.3	0.3	0.3	0.3	0.3	0.3	0.3	0.3
**Upper CI**	90.5	74.2	41.4	35.1	22.2	17.6	10.2	10.2	10.2	10.2	10.2	10.2	10.2	10.2	10.2	10.2
**Number**	66	52	27	21	8	5	3	3	1	1	1	1	1	1	1	1

Table B500-60 Age Group = 41-50 Gender = MALE Occupational group = MANUAL Total cases = 127

Threshold dB HL≥	0	5	10	15	20	25	30	35	40	45	50	55	65	75	85	95
**Prevalence**	**89.7**	**72.7**	**56.7**	**30.3**	**18.2**	**8.8**	**3.0**	**2.6**	**2.6**	**2.1**	**1.4**	**1.1**	**0.5**	**0.5**	**0.5**	**0.0**
**Lower CI**	80.5	61.8	46.1	21.7	11.8	5.0	1.6	1.2	1.2	0.9	0.7	0.5	0.1	0.1	0.1	0.0
**Upper CI**	94.9	81.4	66.7	40.5	27.1	15.1	5.7	5.3	5.3	4.9	3.0	2.5	1.7	1.7	1.7	2.9
**Number**	119	101	78	46	29	16	10	8	8	6	5	4	2	2	2	0

Table B500-61 Age Group = 51-60 Gender = MALE Occupational group = MANUAL Total cases = 199

Threshold dB HL≥	0	5	10	15	20	25	30	35	40	45	50	55	65	75	85	95
**Prevalence**	**95.5**	**84.6**	**63.4**	**34.8**	**20.6**	**15.9**	**10.0**	**6.5**	**5.2**	**4.5**	**3.4**	**2.6**	**0.8**	**0.0**	**0.0**	**0.0**
**Lower CI**	89.3	77.2	55.1	28.1	15.6	11.5	6.6	4.4	3.3	2.7	2.0	1.4	0.3	0.0	0.0	0.0
**Upper CI**	98.2	90.0	71.0	42.1	26.7	21.7	15.1	9.5	8.3	7.6	6.0	4.9	2.3	1.9	1.9	1.9
**Number**	194	174	137	90	60	48	31	25	21	17	14	11	4	0	0	0

Table B500-62 Age Group = 61-70 Gender = MALE Occupational group = MANUAL Total cases = 173

Threshold dB HL≥	0	5	10	15	20	25	30	35	40	45	50	55	65	75	85	95
**Prevalence**	**99.1**	**83.2**	**60.5**	**46.4**	**26.0**	**17.4**	**13.1**	**10.0**	**5.9**	**4.7**	**3.1**	**2.1**	**1.9**	**1.0**	**0.8**	**0.2**
**Lower CI**	97.7	73.3	49.2	36.6	19.3	12.9	9.0	6.5	3.5	2.6	1.9	1.2	1.0	0.5	0.4	0.0
**Upper CI**	99.7	89.9	70.7	56.4	34.0	23.0	18.7	15.3	9.7	8.6	5.3	3.6	3.5	2.0	1.8	1.1
**Number**	169	150	124	100	65	47	39	31	19	15	13	10	9	6	5	1

Table B500-63 Age Group = 71-80 Gender = MALE Occupational group = MANUAL Total cases = 72

Threshold dB HL≥	0	5	10	15	20	25	30	35	40	45	50	55	65	75	85	95
**Prevalence**	**93.8**	**87.1**	**83.2**	**66.6**	**56.0**	**46.2**	**33.5**	**30.2**	**14.8**	**11.4**	**8.3**	**6.3**	**2.4**	**1.0**	**1.0**	**1.0**
**Lower CI**	72.7	67.3	63.9	52.1	41.3	32.6	25.6	23.8	9.7	7.1	4.8	3.8	0.9	0.3	0.3	0.3
**Upper CI**	98.8	95.7	93.3	78.6	69.7	60.4	42.5	37.5	22.0	17.8	14.0	10.1	5.9	3.5	3.5	3.5
**Number**	70	67	63	54	45	36	28	25	20	17	13	11	4	2	2	2

Prevalence by age group, gender and occupational group for the better ear over 1 kHz

Table B1K-1 Age Group = 18-80 Gender = OVERALL Occupational group = OVERALL Total cases = 2664

Threshold dB HL≥	0	5	10	15	20	25	30	35	40	45	50	55	65	75	85	95
**Prevalence**	**87.2**	**62.8**	**39.1**	**24.5**	**15.4**	**10.2**	**7.6**	**5.5**	**4.1**	**3.0**	**2.4**	**1.7**	**0.9**	**0.6**	**0.4**	**0.3**
**Lower CI**	85.3	60.5	37.2	23.0	14.2	9.3	6.8	4.9	3.6	2.6	2.0	1.4	0.7	0.4	0.2	0.1
**Upper CI**	88.9	65.0	41.1	26.1	16.6	11.3	8.5	6.2	4.6	3.5	2.8	2.0	1.2	0.9	0.7	0.6
**Number**	2445	1921	1353	958	678	504	397	315	247	202	161	120	66	41	24	12

Table B1K-2 Age Group = 18-80 Gender = FEMALE Occupational group = OVERALL Total cases = 1387

Threshold dB HL≥	0	5	10	15	20	25	30	35	40	45	50	55	65	75	85	95
**Prevalence**	**89.7**	**65.4**	**40.9**	**25.2**	**16.1**	**11.0**	**8.6**	**6.4**	**4.8**	**3.4**	**2.5**	**1.8**	**1.0**	**0.6**	**0.5**	**0.3**
**Lower CI**	87.5	62.4	38.3	23.2	14.5	9.6	7.3	5.5	4.1	2.8	2.0	1.4	0.7	0.4	0.3	0.1
**Upper CI**	91.6	68.2	43.5	27.2	17.9	12.5	10.0	7.5	5.7	4.1	3.2	2.2	1.4	1.0	0.8	0.5
**Number**	1284	1001	704	494	352	266	219	179	142	113	87	68	37	23	16	8

Table B1K-3 Age Group = 18-80 Gender = MALE Occupational group = OVERALL Total cases = 1277

Threshold dB HL≥	0	5	10	15	20	25	30	35	40	45	50	55	65	75	85	95
**Prevalence**	**84.3**	**59.9**	**37.1**	**23.8**	**14.5**	**9.4**	**6.4**	**4.4**	**3.2**	**2.6**	**2.2**	**1.6**	**0.9**	**0.6**	**0.4**	**0.3**
**Lower CI**	81.1	56.4	34.2	21.5	12.9	8.2	5.4	3.7	2.6	2.1	1.7	1.1	0.5	0.3	0.1	0.1
**Upper CI**	87.1	63.3	40.1	26.3	16.1	10.8	7.6	5.3	3.9	3.3	2.8	2.2	1.5	1.2	1.0	1.0
**Number**	1161	920	649	464	326	238	178	136	105	89	74	52	29	18	8	4

Table B1K-4 Age Group = 18-30 Gender = OVERALL Occupational group = OVERALL Total cases = 401

Threshold dB HL≥	0	5	10	15	20	25	30	35	40	45	50	55	65	75	85	95
**Prevalence**	**74.9**	**43.6**	**17.0**	**5.9**	**2.2**	**1.3**	**1.0**	**0.7**	**0.3**	**0.3**	**0.1**	**0.0**	**0.0**	**0.0**	**0.0**	**0.0**
**Lower CI**	69.5	38.0	13.3	4.1	1.3	0.7	0.4	0.3	0.1	0.1	0.0	0.0	0.0	0.0	0.0	0.0
**Upper CI**	79.6	49.4	21.5	8.6	3.6	2.6	2.2	1.7	0.8	0.8	0.5	0.1	0.1	0.9	0.9	0.9
**Number**	313	191	90	42	21	12	9	8	5	5	3	2	1	0	0	0

Table B1K-5 Age Group = 31-40 Gender = OVERALL Occupational group = OVERALL Total cases = 332

Threshold dB HL≥	0	5	10	15	20	25	30	35	40	45	50	55	65	75	85	95
**Prevalence**	**81.8**	**48.0**	**23.5**	**12.2**	**5.0**	**3.0**	**2.2**	**1.4**	**1.1**	**1.0**	**1.0**	**1.0**	**0.7**	**0.7**	**0.7**	**0.6**
**Lower CI**	75.8	41.7	18.7	8.8	3.1	1.6	1.1	0.6	0.4	0.3	0.3	0.3	0.1	0.1	0.1	0.1
**Upper CI**	86.5	54.3	29.2	16.8	7.8	5.5	4.3	3.3	3.1	3.0	3.0	3.0	3.0	3.0	3.0	3.1
**Number**	283	180	96	53	29	18	13	8	6	5	4	4	2	2	2	1

Table B1K-6 Age Group = 41-50 Gender = OVERALL Occupational group = OVERALL Total cases = 450

Threshold dB HL≥	0	5	10	15	20	25	30	35	40	45	50	55	65	75	85	95
**Prevalence**	**91.6**	**65.6**	**37.1**	**19.8**	**9.5**	**5.6**	**4.4**	**3.7**	**2.9**	**1.6**	**0.9**	**0.6**	**0.3**	**0.3**	**0.1**	**0.1**
**Lower CI**	88.2	60.6	32.5	16.4	7.4	4.1	3.1	2.5	1.9	1.0	0.5	0.4	0.1	0.1	0.0	0.0
**Upper CI**	94.1	70.3	42.0	23.6	12.1	7.6	6.2	5.3	4.5	2.7	1.6	1.0	0.8	0.8	0.6	0.3
**Number**	417	310	187	116	67	45	37	31	24	15	10	8	3	3	2	1

Table B1K-7 Age Group = 51-60 Gender = OVERALL Occupational group = OVERALL Total cases = 682

Threshold dB HL≥	0	5	10	15	20	25	30	35	40	45	50	55	65	75	85	95
**Prevalence**	**93.9**	**73.3**	**49.0**	**30.5**	**17.5**	**10.6**	**7.4**	**5.4**	**3.9**	**2.7**	**2.1**	**1.4**	**0.6**	**0.3**	**0.1**	**0.1**
**Lower CI**	91.4	69.3	44.9	26.9	14.8	8.6	5.8	4.1	2.9	2.0	1.5	0.9	0.3	0.1	0.0	0.0
**Upper CI**	95.7	76.9	53.1	34.2	20.6	13.0	9.4	7.0	5.3	3.7	3.0	2.2	1.2	0.6	0.3	0.3
**Number**	649	530	384	265	171	119	91	70	54	44	34	23	11	7	2	2

Prevalence by age group, gender and occupational group for the better ear over 1 kHz

Table B1K-8 Age Group = 61-70 Gender = OVERALL Occupational group = OVERALL Total cases = 527

Threshold dB HL≥	0	5	10	15	20	25	30	35	40	45	50	55	65	75	85	95
Prevalence	**96.8**	**83.4**	**63.2**	**46.6**	**31.6**	**20.6**	**14.8**	**10.8**	**6.8**	**5.0**	**4.2**	**3.3**	**2.1**	**1.4**	**1.0**	**0.6**
Lower CI	94.3	78.9	58.0	41.7	27.4	17.3	12.1	8.5	5.4	4.0	3.3	2.5	1.4	0.8	0.5	0.3
Upper CI	98.2	87.0	68.0	51.6	36.1	24.3	18.0	13.7	8.6	6.1	5.3	4.3	3.1	2.3	1.8	1.4
Number	513	454	361	281	210	158	124	98	74	62	53	41	25	16	11	5

Table B1K-9 Age Group = 71-80 Gender = OVERALL Occupational group = OVERALL Total cases = 272

Threshold dB HL≥	0	5	10	15	20	25	30	35	40	45	50	55	65	75	85	95
Prevalence	**99.1**	**91.0**	**82.0**	**65.7**	**55.3**	**41.8**	**31.5**	**22.8**	**19.0**	**14.9**	**11.9**	**7.6**	**4.1**	**2.2**	**1.2**	**0.6**
Lower CI	96.8	84.8	75.7	59.0	48.0	35.2	25.4	18.6	15.0	11.6	8.8	5.8	2.8	1.3	0.5	0.2
Upper CI	99.8	94.8	86.9	71.9	62.4	48.7	38.3	27.6	23.8	18.9	15.8	9.9	6.0	3.6	2.4	1.9
Number	270	256	235	201	180	152	123	100	84	71	57	42	24	13	7	3

Table B1K-10 Age Group = 18-80 Gender = OVERALL Occupational group = NON-MANUAL Total cases = 1184

Threshold dB HL≥	0	5	10	15	20	25	30	35	40	45	50	55	65	75	85	95
Prevalence	**84.6**	**58.2**	**34.6**	**20.7**	**13.1**	**8.7**	**6.7**	**4.7**	**3.3**	**2.0**	**1.5**	**1.2**	**0.7**	**0.4**	**0.1**	**0.1**
Lower CI	81.7	55.0	31.9	18.8	11.6	7.7	5.8	3.9	2.7	1.6	1.2	0.9	0.4	0.2	0.1	0.0
Upper CI	87.1	61.3	37.3	22.7	14.7	9.8	7.7	5.6	3.9	2.5	1.9	1.6	1.0	0.8	0.3	0.2
Number	1062	800	524	354	242	177	138	110	85	68	56	44	24	14	6	3

Table B1K-11 Age Group = 18-80 Gender = OVERALL Occupational group = MANUAL Total cases = 1475

Threshold dB HL≥	0	5	10	15	20	25	30	35	40	45	50	55	65	75	85	95
Prevalence	**89.5**	**67.0**	**43.3**	**27.4**	**17.2**	**11.6**	**8.1**	**6.0**	**4.6**	**3.6**	**2.7**	**1.8**	**1.1**	**0.8**	**0.6**	**0.4**
Lower CI	86.9	63.6	40.2	25.1	15.6	10.2	7.1	5.1	3.8	2.9	2.2	1.4	0.8	0.5	0.4	0.2
Upper CI	91.7	70.3	46.5	29.9	18.9	13.0	9.3	7.0	5.4	4.4	3.5	2.3	1.5	1.2	1.0	0.9
Number	1380	1118	826	603	436	327	259	205	162	134	105	76	42	27	18	9

Table B1K-12 Age Group = 18-30 Gender = FEMALE Occupational group = OVERALL Total cases = 224

Threshold dB HL≥	0	5	10	15	20	25	30	35	40	45	50	55	65	75	85	95
Prevalence	**83.0**	**49.9**	**18.4**	**6.3**	**3.6**	**2.4**	**1.8**	**1.3**	**0.5**	**0.5**	**0.2**	**0.0**	**0.0**	**0.0**	**0.0**	**0.0**
Lower CI	76.6	42.5	13.7	4.0	2.1	1.2	0.8	0.5	0.2	0.2	0.0	0.0	0.0	0.0	0.0	0.0
Upper CI	87.9	57.3	24.4	9.9	6.2	4.7	4.1	3.2	1.4	1.4	1.1	1.7	1.7	1.7	1.7	1.7
Number	188	116	53	24	16	10	7	6	3	3	1	0	0	0	0	0

Table B1K-13 Age Group = 31-40 Gender = FEMALE Occupational group = OVERALL Total cases = 190

Threshold dB HL≥	0	5	10	15	20	25	30	35	40	45	50	55	65	75	85	95
Prevalence	**82.8**	**47.0**	**23.0**	**9.7**	**4.8**	**2.6**	**2.4**	**1.3**	**1.1**	**0.8**	**0.7**	**0.7**	**0.2**	**0.2**	**0.2**	**0.0**
Lower CI	75.5	38.9	17.0	6.4	2.7	1.3	1.2	0.6	0.5	0.3	0.3	0.3	0.0	0.0	0.0	0.0
Upper CI	88.3	55.3	30.3	14.5	8.5	5.2	5.0	2.9	2.7	2.2	2.2	2.2	1.2	1.2	1.2	2.0
Number	163	101	55	29	17	11	9	6	5	4	3	3	1	1	1	0

Table B1K-14 Age Group = 41-50 Gender = FEMALE Occupational group = OVERALL Total cases = 247

Threshold dB HL≥	0	5	10	15	20	25	30	35	40	45	50	55	65	75	85	95
Prevalence	**91.8**	**66.6**	**36.9**	**21.7**	**10.8**	**5.3**	**4.3**	**4.3**	**3.7**	**2.2**	**1.0**	**0.5**	**0.1**	**0.1**	**0.1**	**0.1**
Lower CI	86.9	59.8	30.9	17.0	7.7	3.3	2.7	2.7	2.1	1.1	0.4	0.2	0.0	0.0	0.0	0.0
Upper CI	95.0	72.7	43.3	27.3	14.9	8.2	6.8	6.8	6.2	4.4	2.3	1.1	0.6	0.6	0.6	0.6
Number	231	173	103	67	39	23	20	20	16	10	6	4	1	1	1	1

Prevalence by age group, gender and occupational group for the better ear over 1 kHz

Table B1K-15 Age Group = 51-60 Gender = FEMALE Occupational group = OVERALL Total cases = 341

	Threshold dB HL≥															
	**0**	**5**	**10**	**15**	**20**	**25**	**30**	**35**	**40**	**45**	**50**	**55**	**65**	**75**	**85**	**95**
**Prevalence**	**93.4**	**74.0**	**50.2**	**29.7**	**16.0**	**10.1**	**6.8**	**5.4**	**3.9**	**2.4**	**1.6**	**1.2**	**0.6**	**0.3**	**0.1**	**0.1**
**Lower CI**	89.6	68.4	44.4	25.0	12.5	7.4	4.7	3.6	2.4	1.5	0.9	0.7	0.2	0.1	0.0	0.0
**Upper CI**	95.9	78.9	56.0	34.9	20.3	13.5	9.7	8.1	6.2	3.8	2.8	2.2	1.5	0.7	0.5	0.5
**Number**	324	267	198	135	85	59	43	34	26	21	15	12	5	4	2	2

Table B1K-16 Age Group = 61-70 Gender = FEMALE Occupational group = OVERALL Total cases = 242

	Threshold dB HL≥															
	**0**	**5**	**10**	**15**	**20**	**25**	**30**	**35**	**40**	**45**	**50**	**55**	**65**	**75**	**85**	**95**
**Prevalence**	**96.6**	**83.5**	**64.4**	**45.6**	**28.9**	**21.2**	**16.3**	**11.1**	**6.8**	**4.5**	**3.7**	**3.3**	**2.0**	**1.4**	**1.2**	**0.9**
**Lower CI**	92.3	77.4	57.5	39.2	23.7	16.3	12.2	7.6	4.8	3.3	2.7	2.3	1.2	0.7	0.5	0.3
**Upper CI**	98.5	88.2	70.7	52.2	34.6	27.1	21.5	15.9	9.6	6.0	5.1	4.7	3.5	2.9	2.7	2.4
**Number**	236	207	167	126	91	75	64	49	37	31	27	23	13	7	6	3

Table B1K-17 Age Group = 71-80 Gender = FEMALE Occupational group = OVERALL Total cases = 143

	Threshold dB HL≥															
	**0**	**5**	**10**	**15**	**20**	**25**	**30**	**35**	**40**	**45**	**50**	**55**	**65**	**75**	**85**	**95**
**Prevalence**	**99.4**	**92.6**	**85.2**	**68.4**	**57.7**	**43.0**	**34.8**	**26.9**	**22.9**	**16.7**	**13.3**	**8.4**	**5.1**	**3.0**	**1.7**	**0.7**
**Lower CI**	96.8	83.1	76.0	59.3	47.0	34.1	26.4	21.0	17.1	12.2	9.0	6.0	3.3	1.6	0.8	0.2
**Upper CI**	99.9	97.0	91.3	76.3	67.6	52.4	44.4	33.8	30.0	22.5	19.1	11.8	7.9	5.3	3.8	3.0
**Number**	142	137	128	113	104	88	76	64	55	44	35	26	17	10	6	2

Table B1K-18 Age Group = 18-30 Gender = MALE Occupational group = OVERALL Total cases = 177

	Threshold dB HL≥															
	**0**	**5**	**10**	**15**	**20**	**25**	**30**	**35**	**40**	**45**	**50**	**55**	**65**	**75**	**85**	**95**
**Prevalence**	**66.3**	**37.2**	**15.5**	**5.5**	**0.6**	**0.1**	**0.1**	**0.1**	**0.1**	**0.1**	**0.1**	**0.1**	**0.0**	**0.0**	**0.0**	**0.0**
**Lower CI**	57.3	29.0	10.2	3.0	0.2	0.0	0.0	0.0	0.0	0.0	0.0	0.0	0.0	0.0	0.0	0.0
**Upper CI**	74.3	46.2	22.8	9.8	1.7	0.2	0.2	0.2	0.2	0.2	0.2	0.2	0.2	2.1	2.1	2.1
**Number**	125	75	37	18	5	2	2	2	2	2	2	2	1	0	0	0

Table B1K-19 Age Group = 31-40 Gender = MALE Occupational group = OVERALL Total cases = 142

	Threshold dB HL≥															
	**0**	**5**	**10**	**15**	**20**	**25**	**30**	**35**	**40**	**45**	**50**	**55**	**65**	**75**	**85**	**95**
**Prevalence**	**80.6**	**49.4**	**24.4**	**15.1**	**5.2**	**3.5**	**2.1**	**1.4**	**1.2**	**1.2**	**1.2**	**1.2**	**1.2**	**1.2**	**1.2**	**1.2**
**Lower CI**	70.5	39.8	17.1	9.2	2.5	1.3	0.7	0.3	0.2	0.2	0.2	0.2	0.2	0.2	0.2	0.2
**Upper CI**	87.9	59.0	33.5	23.7	10.4	9.0	6.4	6.3	6.5	6.5	6.5	6.5	6.5	6.5	6.5	6.5
**Number**	120	79	41	24	12	7	4	2	1	1	1	1	1	1	1	1

Table B1K-20 Age Group = 41-50 Gender = MALE Occupational group = OVERALL Total cases = 203

	Threshold dB HL≥															
	**0**	**5**	**10**	**15**	**20**	**25**	**30**	**35**	**40**	**45**	**50**	**55**	**65**	**75**	**85**	**95**
**Prevalence**	**91.4**	**64.5**	**37.3**	**17.6**	**8.0**	**5.9**	**4.6**	**3.0**	**2.1**	**1.0**	**0.8**	**0.8**	**0.4**	**0.4**	**0.2**	**0.0**
**Lower CI**	86.3	56.9	30.2	13.0	5.5	3.8	2.7	1.6	1.0	0.5	0.4	0.4	0.1	0.1	0.0	0.0
**Upper CI**	94.8	71.5	44.9	23.3	11.6	9.0	7.5	5.4	4.2	1.7	1.4	1.4	1.6	1.6	1.1	1.9
**Number**	186	137	84	49	28	22	17	11	8	5	4	4	2	2	1	0

Table B1K-21 Age Group = 51-60 Gender = MALE Occupational group = OVERALL Total cases = 341

	Threshold dB HL≥															
	**0**	**5**	**10**	**15**	**20**	**25**	**30**	**35**	**40**	**45**	**50**	**55**	**65**	**75**	**85**	**95**
**Prevalence**	**94.4**	**72.4**	**47.6**	**31.2**	**19.2**	**11.3**	**8.2**	**5.4**	**3.9**	**3.1**	**2.6**	**1.7**	**0.7**	**0.3**	**0.0**	**0.0**
**Lower CI**	90.8	66.5	41.9	26.1	15.2	8.5	6.0	3.8	2.7	2.0	1.6	0.8	0.3	0.1	0.0	0.0
**Upper CI**	96.6	77.6	53.5	36.9	23.9	14.9	11.2	7.5	5.7	4.7	4.2	3.2	1.6	1.0	1.1	1.1
**Number**	325	263	186	130	86	60	48	36	28	23	19	11	6	3	0	0

Prevalence by age group, gender and occupational group for the better ear over 1 kHz

Table B1K-22 Age Group = 61-70 Gender = MALE Occupational group = OVERALL Total cases = 285

Threshold dB HL≥	0	5	10	15	20	25	30	35	40	45	50	55	65	75	85	95
Prevalence	**97.0**	**83.4**	**61.9**	**47.8**	**34.9**	**19.9**	**13.0**	**10.4**	**6.9**	**5.6**	**4.7**	**3.3**	**2.2**	**1.4**	**0.8**	**0.3**
Lower CI	93.9	76.4	54.0	40.3	28.3	16.2	10.1	7.8	5.3	4.1	3.3	2.2	1.3	0.8	0.3	0.1
Upper CI	98.6	88.6	69.2	55.4	42.1	24.3	16.6	13.8	9.1	7.6	6.7	5.0	3.8	2.7	1.7	1.2
Number	277	247	194	155	119	83	60	49	37	31	26	18	12	9	5	2

Table B1K-23 Age Group = 71-80 Gender = MALE Occupational group = OVERALL Total cases = 129

Threshold dB HL≥	0	5	10	15	20	25	30	35	40	45	50	55	65	75	85	95
Prevalence	**98.7**	**88.2**	**76.3**	**61.0**	**50.7**	**39.1**	**26.3**	**16.6**	**13.2**	**12.3**	**9.7**	**6.5**	**2.7**	**1.1**	**0.4**	**0.4**
Lower CI	92.7	79.1	67.3	50.5	41.9	30.3	19.0	12.1	9.3	8.6	6.6	4.1	1.3	0.4	0.1	0.1
Upper CI	99.8	93.7	83.5	70.5	59.4	48.7	35.2	22.3	18.3	17.3	14.2	10.2	5.5	3.1	2.0	2.0
Number	128	119	107	88	76	64	47	36	29	27	22	16	7	3	1	1

Table B1K-24 Age Group = 18-30 Gender = OVERALL Occupational group = NON-MANUAL Total cases = 181

Threshold dB HL≥	0	5	10	15	20	25	30	35	40	45	50	55	65	75	85	95
Prevalence	**66.1**	**34.3**	**12.3**	**2.9**	**1.9**	**1.0**	**0.5**	**0.5**	**0.5**	**0.5**	**0.1**	**0.1**	**0.1**	**0.0**	**0.0**	**0.0**
Lower CI	57.9	27.2	7.8	1.4	0.9	0.3	0.1	0.1	0.1	0.1	0.0	0.0	0.0	0.0	0.0	0.0
Upper CI	73.5	42.2	19.0	5.6	3.9	3.0	2.4	2.4	2.4	2.4	2.2	2.2	2.2	2.1	2.1	2.1
Number	130	72	26	9	7	4	2	2	2	2	1	1	1	0	0	0

Table B1K-25 Age Group = 31-40 Gender = OVERALL Occupational group = NON-MANUAL Total cases = 148

Threshold dB HL≥	0	5	10	15	20	25	30	35	40	45	50	55	65	75	85	95
Prevalence	**82.2**	**44.2**	**19.5**	**7.6**	**2.6**	**1.2**	**1.1**	**0.3**	**0.3**	**0.3**	**0.3**	**0.3**	**0.0**	**0.0**	**0.0**	**0.0**
Lower CI	73.1	36.2	13.6	4.1	1.1	0.5	0.4	0.1	0.1	0.1	0.1	0.1	0.0	0.0	0.0	0.0
Upper CI	88.7	52.6	27.0	13.7	6.0	2.7	2.7	1.7	1.7	1.7	1.7	1.7	2.5	2.5	2.5	2.5
Number	123	74	33	13	7	5	4	1	1	1	1	1	0	0	0	0

Table B1K-26 Age Group = 41-50 Gender = OVERALL Occupational group = NON-MANUAL Total cases = 188

Threshold dB HL≥	0	5	10	15	20	25	30	35	40	45	50	55	65	75	85	95
Prevalence	**91.1**	**62.6**	**31.4**	**13.5**	**7.1**	**4.0**	**3.4**	**2.4**	**0.6**	**0.4**	**0.1**	**0.1**	**0.0**	**0.0**	**0.0**	**0.0**
Lower CI	86.0	54.5	25.6	9.2	4.5	2.1	1.6	0.9	0.3	0.2	0.0	0.0	0.0	0.0	0.0	0.0
Upper CI	94.5	70.0	37.9	19.2	11.0	7.3	6.9	6.3	1.0	0.7	0.7	0.7	2.0	2.0	2.0	2.0
Number	170	119	63	32	18	9	8	5	3	2	1	1	0	0	0	0

Table B1K-27 Age Group = 51-60 Gender = OVERALL Occupational group = NON-MANUAL Total cases = 317

Threshold dB HL≥	0	5	10	15	20	25	30	35	40	45	50	55	65	75	85	95
Prevalence	**92.2**	**71.7**	**41.0**	**25.5**	**11.2**	**5.7**	**4.4**	**3.5**	**2.4**	**2.2**	**1.5**	**0.8**	**0.5**	**0.3**	**0.1**	**0.1**
Lower CI	88.3	66.0	35.3	20.9	8.3	4.2	3.1	2.3	1.6	1.4	0.8	0.4	0.2	0.1	0.0	0.0
Upper CI	94.9	76.8	46.9	30.8	14.9	7.6	6.3	5.2	3.6	3.4	2.5	1.8	1.4	0.8	0.4	0.4
Number	296	235	151	105	58	39	30	24	20	18	12	7	4	3	1	1

Table B1K-28 Age Group = 61-70 Gender = OVERALL Occupational group = NON-MANUAL Total cases = 227

Threshold dB HL≥	0	5	10	15	20	25	30	35	40	45	50	55	65	75	85	95
Prevalence	**98.0**	**81.8**	**64.9**	**46.5**	**29.6**	**18.1**	**13.7**	**11.3**	**6.9**	**4.3**	**3.6**	**3.2**	**1.9**	**1.6**	**0.6**	**0.4**
Lower CI	94.7	75.4	58.0	38.7	22.8	14.1	10.4	8.5	4.9	3.0	2.3	2.0	0.9	0.7	0.2	0.1
Upper CI	99.3	86.8	71.2	54.6	37.5	22.9	17.9	15.0	9.7	6.1	5.5	5.1	3.9	3.7	1.8	1.6
Number	222	187	148	111	75	58	46	39	27	22	21	18	10	7	4	2

Prevalence by age group, gender and occupational group for the better ear over 1 kHz

Table B1K-29 Age Group = 71-80 Gender = OVERALL Occupational group = NON-MANUAL Total cases = 123

	Threshold dB HL≥															
	0	5	10	15	20	25	30	35	40	45	50	55	65	75	85	95
Prevalence	**98.3**	**87.2**	**78.1**	**62.6**	**55.0**	**44.4**	**33.9**	**21.4**	**17.8**	**8.6**	**7.5**	**6.0**	**3.3**	**1.7**	**0.4**	**0.0**
Lower CI	94.0	75.0	67.7	55.6	46.4	37.1	27.5	16.2	13.0	6.4	5.5	4.0	1.7	0.6	0.1	0.0
Upper CI	99.5	93.9	85.9	69.1	63.2	51.9	41.0	27.8	23.7	11.6	10.2	9.0	6.3	4.5	2.0	3.0
Number	121	113	103	84	77	62	48	39	32	23	20	16	9	4	1	0

Table B1K-30 Age Group = 18-30 Gender = OVERALL Occupational group = MANUAL Total cases = 218

	Threshold dB HL≥															
	0	5	10	15	20	25	30	35	40	45	50	55	65	75	85	95
Prevalence	**83.3**	**53.8**	**23.0**	**8.7**	**2.4**	**1.8**	**1.6**	**1.1**	**0.2**	**0.2**	**0.2**	**0.0**	**0.0**	**0.0**	**0.0**	**0.0**
Lower CI	75.9	44.9	16.5	5.6	1.2	0.7	0.6	0.3	0.1	0.1	0.0	0.0	0.0	0.0	0.0	0.0
Upper CI	88.8	62.4	31.1	13.5	4.9	4.3	4.3	3.2	0.8	0.8	0.7	0.2	1.7	1.7	1.7	1.7
Number	183	119	64	33	14	8	7	6	3	3	2	1	0	0	0	0

Table B1K-31 Age Group = 31-40 Gender = OVERALL Occupational group = MANUAL Total cases = 183

	Threshold dB HL≥															
	0	5	10	15	20	25	30	35	40	45	50	55	65	75	85	95
Prevalence	**80.9**	**51.1**	**27.5**	**15.4**	**7.0**	**4.9**	**3.2**	**2.0**	**1.7**	**1.3**	**1.3**	**1.3**	**1.1**	**1.1**	**1.1**	**0.9**
Lower CI	72.0	42.1	20.2	9.9	3.9	2.2	1.4	0.8	0.5	0.4	0.3	0.3	0.2	0.2	0.2	0.2
Upper CI	87.5	60.0	36.3	23.1	12.4	10.2	7.0	5.1	5.0	4.8	4.8	4.8	4.9	4.9	4.9	5.0
Number	159	105	62	39	22	13	9	7	5	4	3	3	2	2	2	1

Table B1K-32 Age Group = 41-50 Gender = OVERALL Occupational group = MANUAL Total cases = 262

	Threshold dB HL≥															
	0	5	10	15	20	25	30	35	40	45	50	55	65	75	85	95
Prevalence	**92.5**	**67.8**	**43.3**	**26.2**	**10.8**	**6.8**	**5.4**	**4.7**	**4.2**	**2.5**	**1.2**	**0.7**	**0.3**	**0.3**	**0.2**	**0.1**
Lower CI	87.5	60.6	36.4	20.7	7.7	4.7	3.6	3.1	2.6	1.3	0.6	0.4	0.1	0.1	0.1	0.0
Upper CI	95.6	74.2	50.5	32.5	14.9	9.8	8.0	7.1	6.7	4.5	2.4	1.1	0.9	0.9	0.7	0.5
Number	247	191	124	84	49	36	29	26	21	13	9	7	3	3	2	1

Table B1K-33 Age Group = 51-60 Gender = OVERALL Occupational group = MANUAL Total cases = 365

	Threshold dB HL≥															
	0	5	10	15	20	25	30	35	40	45	50	55	65	75	85	95
Prevalence	**95.6**	**74.8**	**53.5**	**32.5**	**23.1**	**14.8**	**9.8**	**6.5**	**4.8**	**2.7**	**2.2**	**1.5**	**0.7**	**0.4**	**0.1**	**0.1**
Lower CI	92.0	69.0	47.4	27.4	18.7	11.3	7.2	4.7	3.3	1.8	1.5	0.9	0.3	0.1	0.0	0.0
Upper CI	97.6	79.9	59.4	38.1	28.3	19.1	13.1	8.9	6.9	3.9	3.2	2.6	1.4	1.1	0.5	0.5
Number	353	295	233	160	113	80	61	46	34	26	22	16	7	4	1	1

Table B1K-34 Age Group = 61-70 Gender = OVERALL Occupational group = MANUAL Total cases = 299

	Threshold dB HL≥															
	0	5	10	15	20	25	30	35	40	45	50	55	65	75	85	95
Prevalence	**95.1**	**84.8**	**62.8**	**45.4**	**32.5**	**22.2**	**15.2**	**11.0**	**7.3**	**5.8**	**4.7**	**3.6**	**2.5**	**1.7**	**1.5**	**1.1**
Lower CI	90.2	77.9	55.5	38.5	27.2	18.2	12.8	8.0	6.0	4.6	3.7	2.8	1.8	1.2	1.1	0.8
Upper CI	97.6	89.8	69.7	52.5	38.2	26.8	17.9	14.8	8.9	7.3	5.9	4.7	3.4	2.4	2.2	1.6
Number	290	266	212	170	135	100	78	59	47	40	32	23	15	9	7	3

Table B1K-35 Age Group = 71-80 Gender = OVERALL Occupational group = MANUAL Total cases = 148

	Threshold dB HL≥															
	0	5	10	15	20	25	30	35	40	45	50	55	65	75	85	95
Prevalence	**100.0**	**94.7**	**86.5**	**72.8**	**58.1**	**40.9**	**28.4**	**22.4**	**19.6**	**18.6**	**14.4**	**7.4**	**4.2**	**2.4**	**1.8**	**1.0**
Lower CI	97.5	85.2	76.6	63.9	50.5	33.7	21.8	16.1	13.4	12.4	8.7	5.1	2.7	1.2	0.8	0.3
Upper CI	100.0	98.2	92.6	80.3	65.4	48.6	36.1	30.5	27.8	26.9	22.9	10.5	6.7	4.6	4.0	3.2
Number	148	142	131	117	103	90	75	61	52	48	37	26	15	9	6	3

Prevalence by age group, gender and occupational group for the better ear over 1 kHz

Table B1K-36 Age Group = 18-80 Gender = FEMALE Occupational group = NON-MANUAL Total cases = 670

	Threshold dB HL≥															
	0	5	10	15	20	25	30	35	40	45	50	55	65	75	85	95
**Prevalence**	**88.4**	**62.8**	**37.0**	**22.1**	**14.1**	**9.7**	**8.0**	**5.6**	**4.4**	**2.5**	**2.0**	**1.7**	**1.0**	**0.6**	**0.2**	**0.1**
Lower CI	85.3	58.6	33.6	19.8	12.2	8.3	6.8	4.5	3.5	1.9	1.5	1.3	0.6	0.3	0.1	0.0
Upper CI	91.0	66.7	40.6	24.5	16.2	11.2	9.3	6.9	5.5	3.3	2.6	2.3	1.5	1.1	0.6	0.5
Number	609	460	306	202	139	107	87	74	59	46	39	33	19	11	5	3

Table B1K-37 Age Group = 18-80 Gender = MALE Occupational group = NON-MANUAL Total cases = 514

	Threshold dB HL≥															
	0	5	10	15	20	25	30	35	40	45	50	55	65	75	85	95
**Prevalence**	**80.2**	**53.2**	**31.8**	**19.2**	**11.9**	**7.6**	**5.3**	**3.6**	**2.0**	**1.5**	**1.1**	**0.7**	**0.4**	**0.3**	**0.1**	**0.0**
Lower CI	75.0	48.3	27.9	16.2	9.8	6.2	4.1	2.6	1.5	1.1	0.7	0.4	0.1	0.1	0.0	0.0
Upper CI	84.5	58.0	36.0	22.5	14.4	9.2	6.9	5.0	2.7	2.1	1.6	1.3	0.9	0.8	0.3	0.7
Number	453	340	218	152	103	70	51	36	26	22	17	11	5	3	1	0

Table B1K-38 Age Group = 18-80 Gender = FEMALE Occupational group = MANUAL Total cases = 715

	Threshold dB HL≥															
	0	5	10	15	20	25	30	35	40	45	50	55	65	75	85	95
**Prevalence**	**91.2**	**68.1**	**44.6**	**27.5**	**18.0**	**12.6**	**9.3**	**7.0**	**5.3**	**4.0**	**2.8**	**1.7**	**1.1**	**0.8**	**0.7**	**0.4**
Lower CI	87.8	63.4	40.4	24.4	15.7	10.7	7.8	5.6	4.2	3.1	1.9	1.3	0.8	0.5	0.5	0.3
Upper CI	93.8	72.6	48.9	30.8	20.6	14.7	11.2	8.6	6.6	5.2	3.9	2.2	1.5	1.1	1.1	0.7
Number	673	539	396	291	213	159	132	105	83	67	48	35	18	12	11	5

Table B1K-39 Age Group = 18-80 Gender = MALE Occupational group = MANUAL Total cases = 760

	Threshold dB HL≥															
	0	5	10	15	20	25	30	35	40	45	50	55	65	75	85	95
**Prevalence**	**87.7**	**65.8**	**41.9**	**27.4**	**16.2**	**10.4**	**6.8**	**4.9**	**3.8**	**3.2**	**2.7**	**1.9**	**1.1**	**0.8**	**0.5**	**0.4**
Lower CI	83.4	60.8	37.4	24.0	14.1	8.7	5.6	3.9	2.9	2.4	2.0	1.3	0.6	0.4	0.2	0.1
Upper CI	91.0	70.5	46.5	31.1	18.5	12.4	8.2	6.1	4.8	4.2	3.7	2.9	2.0	1.7	1.6	1.6
Number	707	579	430	312	223	168	127	100	79	67	57	41	24	15	7	4

Table B1K-40 Age Group = 18-30 Gender = FEMALE Occupational group = NON-MANUAL Total cases = 115

	Threshold dB HL≥															
	0	5	10	15	20	25	30	35	40	45	50	55	65	75	85	95
**Prevalence**	**79.4**	**44.0**	**13.2**	**3.5**	**3.5**	**1.8**	**0.8**	**0.8**	**0.8**	**0.8**	**0.0**	**0.0**	**0.0**	**0.0**	**0.0**	**0.0**
Lower CI	70.4	35.0	8.2	1.6	1.6	0.6	0.1	0.1	0.1	0.1	0.0	0.0	0.0	0.0	0.0	0.0
Upper CI	86.2	53.4	20.5	7.4	7.4	5.5	4.5	4.5	4.5	4.5	3.2	3.2	3.2	3.2	3.2	3.2
Number	93	52	18	6	6	3	1	1	1	1	0	0	0	0	0	0

Table B1K-41 Age Group = 31-40 Gender = FEMALE Occupational group = NON-MANUAL Total cases = 86

	Threshold dB HL≥															
	0	5	10	15	20	25	30	35	40	45	50	55	65	75	85	95
**Prevalence**	**83.9**	**46.1**	**19.1**	**6.2**	**3.2**	**1.7**	**1.5**	**0.6**	**0.6**	**0.6**	**0.6**	**0.6**	**0.0**	**0.0**	**0.0**	**0.0**
Lower CI	74.0	35.4	12.1	3.0	1.1	0.6	0.5	0.1	0.1	0.1	0.1	0.1	0.0	0.0	0.0	0.0
Upper CI	90.6	57.1	28.8	12.4	8.5	4.2	4.2	3.2	3.2	3.2	3.2	3.2	4.3	4.3	4.3	4.3
Number	72	43	19	8	5	4	3	1	1	1	1	1	0	0	0	0

Table B1K-42 Age Group = 41-50 Gender = FEMALE Occupational group = NON-MANUAL Total cases = 112

	Threshold dB HL≥															
	0	5	10	15	20	25	30	35	40	45	50	55	65	75	85	95
**Prevalence**	**89.0**	**64.6**	**31.7**	**15.4**	**7.0**	**1.9**	**1.9**	**1.9**	**0.8**	**0.6**	**0.0**	**0.0**	**0.0**	**0.0**	**0.0**	**0.0**
Lower CI	80.9	54.6	24.3	10.3	4.3	1.5	1.5	1.5	0.4	0.1	0.0	0.0	0.0	0.0	0.0	0.0
Upper CI	93.9	73.4	40.1	22.3	11.3	2.5	2.5	2.5	1.5	4.3	3.3	3.3	3.3	3.3	3.3	3.3
Number	101	73	39	21	10	3	3	3	2	1	0	0	0	0	0	0

Prevalence by age group, gender and occupational group for the better ear over 1 kHz

Table B1K-43 Age Group = 51-60 Gender = FEMALE Occupational group = NON-MANUAL Total cases = 175

	Threshold dB HL≥															
	0	5	10	15	20	25	30	35	40	45	50	55	65	75	85	95
Prevalence	92.7	74.3	48.6	27.1	10.6	6.7	4.9	4.5	3.1	2.7	2.1	1.4	1.0	0.5	0.1	0.1
Lower CI	87.8	66.9	40.7	20.9	7.2	4.5	3.1	2.7	1.9	1.6	1.0	0.6	0.4	0.2	0.0	0.0
Upper CI	95.7	80.6	56.6	34.4	15.3	9.8	7.8	7.4	5.0	4.5	4.1	3.2	2.6	1.4	0.7	0.7
Number	165	133	95	60	32	24	18	16	13	11	8	6	4	3	1	1

Table B1K-44 Age Group = 61-70 Gender = FEMALE Occupational group = NON-MANUAL Total cases = 115

	Threshold dB HL≥															
	0	5	10	15	20	25	30	35	40	45	50	55	65	75	85	95
Prevalence	97.4	82.2	61.9	45.1	27.3	17.7	14.2	10.4	6.2	3.6	3.6	3.3	1.9	1.2	0.8	0.7
Lower CI	91.4	73.2	52.1	36.4	20.0	12.1	9.2	6.5	3.6	2.3	2.3	2.0	0.9	0.4	0.2	0.2
Upper CI	99.3	88.6	70.8	54.1	35.9	25.3	21.2	16.4	10.4	5.5	5.5	5.3	4.0	3.5	3.0	3.1
Number	112	95	75	56	39	34	29	24	17	14	14	12	7	4	3	2

Table B1K-45 Age Group = 71-80 Gender = FEMALE Occupational group = NON-MANUAL Total cases = 67

	Threshold dB HL≥															
	0	5	10	15	20	25	30	35	40	45	50	55	65	75	85	95
Prevalence	98.6	89.8	83.6	68.7	58.5	47.4	41.0	26.8	24.6	10.6	9.7	8.6	5.0	2.9	0.6	0.0
Lower CI	92.2	69.5	65.9	60.0	46.8	38.5	33.3	19.5	17.4	7.4	6.7	5.6	2.5	1.1	0.1	0.0
Upper CI	99.7	97.1	93.1	76.2	69.2	56.5	49.1	35.5	33.7	14.8	14.0	13.0	9.8	7.7	3.4	5.4
Number	66	64	60	51	47	39	33	29	25	18	16	14	8	4	1	0

Table B1K-46 Age Group = 18-30 Gender = MALE Occupational group = NON-MANUAL Total cases = 66

	Threshold dB HL≥															
	0	5	10	15	20	25	30	35	40	45	50	55	65	75	85	95
Prevalence	51.7	24.2	11.3	2.2	0.1	0.1	0.1	0.1	0.1	0.1	0.1	0.1	0.1	0.0	0.0	0.0
Lower CI	38.3	14.5	4.9	0.6	0.0	0.0	0.0	0.0	0.0	0.0	0.0	0.0	0.0	0.0	0.0	0.0
Upper CI	64.9	37.7	24.0	7.6	5.7	5.7	5.7	5.7	5.7	5.7	5.7	5.7	5.7	5.5	5.5	5.5
Number	37	20	8	3	1	1	1	1	1	1	1	1	1	0	0	0

Table B1K-47 Age Group = 31-40 Gender = MALE Occupational group = NON-MANUAL Total cases = 62

	Threshold dB HL≥															
	0	5	10	15	20	25	30	35	40	45	50	55	65	75	85	95
Prevalence	80.0	42.5	19.9	9.2	1.9	0.6	0.6	0.0	0.0	0.0	0.0	0.0	0.0	0.0	0.0	0.0
Lower CI	63.1	30.7	11.3	3.6	0.5	0.1	0.1	0.0	0.0	0.0	0.0	0.0	0.0	0.0	0.0	0.0
Upper CI	90.3	55.2	32.6	21.5	7.1	3.5	3.5	5.8	5.8	5.8	5.8	5.8	5.8	5.8	5.8	5.8
Number	51	31	14	5	2	1	1	0	0	0	0	0	0	0	0	0

Table B1K-48 Age Group = 41-50 Gender = MALE Occupational group = NON-MANUAL Total cases = 76

	Threshold dB HL≥															
	0	5	10	15	20	25	30	35	40	45	50	55	65	75	85	95
Prevalence	93.5	60.4	31.1	11.5	7.2	6.2	5.0	2.9	0.3	0.3	0.3	0.3	0.0	0.0	0.0	0.0
Lower CI	86.2	47.4	22.5	5.6	3.4	2.7	1.8	0.6	0.0	0.0	0.0	0.0	0.0	0.0	0.0	0.0
Upper CI	97.1	72.1	41.1	22.3	14.6	13.7	13.1	13.4	1.4	1.4	1.4	1.4	4.8	4.8	4.8	4.8
Number	69	46	24	11	8	6	5	2	1	1	1	1	0	0	0	0

Table B1K-49 Age Group = 51-60 Gender = MALE Occupational group = NON-MANUAL Total cases = 142

	Threshold dB HL≥															
	0	5	10	15	20	25	30	35	40	45	50	55	65	75	85	95
Prevalence	91.6	68.7	32.4	23.8	11.8	4.5	3.9	2.3	1.7	1.7	0.7	0.2	0.0	0.0	0.0	0.0
Lower CI	84.6	59.7	24.7	17.4	7.5	2.8	2.2	1.1	0.8	0.8	0.3	0.0	0.0	0.0	0.0	0.0
Upper CI	95.6	76.4	41.3	31.7	18.1	7.2	6.7	4.8	3.5	3.5	1.7	0.9	2.6	2.6	2.6	2.6
Number	131	102	56	45	26	15	12	8	7	7	4	1	0	0	0	0

Prevalence by age group, gender and occupational group for the better ear over 1 kHz

Table B1K-50 Age Group = 61-70 Gender = MALE Occupational group = NON-MANUAL Total cases = 112

Threshold dB HL≥	0	5	10	15	20	25	30	35	40	45	50	55	65	75	85	95
Prevalence	**98.6**	**82.1**	**69.0**	**48.6**	**32.7**	**18.7**	**13.3**	**12.4**	**7.9**	**5.3**	**3.7**	**3.2**	**2.1**	**2.1**	**0.4**	**0.0**
Lower CI	95.0	72.8	59.3	35.1	21.0	13.8	9.6	8.8	5.3	3.1	1.7	1.4	0.6	0.6	0.1	0.0
Upper CI	99.6	88.7	77.2	62.4	46.9	24.9	18.0	17.2	11.6	9.0	7.8	7.4	6.8	6.8	2.1	3.3
Number	110	92	73	55	36	24	17	15	10	8	7	6	3	3	1	0

Table B1K-51 Age Group = 71-80 Gender = MALE Occupational group = NON-MANUAL Total cases = 56

Threshold dB HL≥	0	5	10	15	20	25	30	35	40	45	50	55	65	75	85	95
Prevalence	**97.9**	**83.0**	**68.8**	**52.5**	**49.3**	**39.6**	**23.5**	**13.4**	**7.7**	**5.9**	**4.3**	**2.1**	**1.0**	**0.0**	**0.0**	**0.0**
Lower CI	89.0	68.7	59.3	41.1	37.8	28.6	14.3	7.7	4.5	3.1	2.5	0.6	0.2	0.0	0.0	0.0
Upper CI	99.6	91.6	76.9	63.7	60.8	51.9	36.0	22.2	12.8	11.0	7.0	7.5	5.3	6.4	6.4	6.4
Number	55	49	43	33	30	23	15	10	7	5	4	2	1	0	0	0

Table B1K-52 Age Group = 18-30 Gender = FEMALE Occupational group = MANUAL Total cases = 109

Threshold dB HL≥	0	5	10	15	20	25	30	35	40	45	50	55	65	75	85	95
Prevalence	**87.9**	**58.1**	**25.4**	**9.9**	**3.9**	**3.3**	**3.0**	**1.9**	**0.4**	**0.4**	**0.3**	**0.0**	**0.0**	**0.0**	**0.0**	**0.0**
Lower CI	77.5	45.6	16.6	5.3	1.7	1.3	1.1	0.6	0.1	0.1	0.0	0.0	0.0	0.0	0.0	0.0
Upper CI	93.9	69.7	36.9	17.6	8.4	8.0	7.9	5.9	1.4	1.4	1.4	3.4	3.4	3.4	3.4	3.4
Number	95	64	35	18	10	7	6	5	2	2	1	0	0	0	0	0

Table B1K-53 Age Group = 31-40 Gender = FEMALE Occupational group = MANUAL Total cases = 103

Threshold dB HL≥	0	5	10	15	20	25	30	35	40	45	50	55	65	75	85	95
Prevalence	**81.0**	**46.2**	**24.3**	**10.5**	**6.0**	**3.5**	**3.3**	**1.7**	**1.4**	**0.8**	**0.6**	**0.6**	**0.3**	**0.3**	**0.3**	**0.0**
Lower CI	69.3	34.3	15.8	6.2	2.8	1.3	1.2	0.7	0.5	0.3	0.2	0.2	0.1	0.1	0.1	0.0
Upper CI	88.9	58.6	35.4	17.3	12.1	9.1	8.9	4.1	3.8	2.3	2.3	2.3	1.7	1.7	1.7	3.6
Number	90	57	35	20	12	7	6	5	4	3	2	2	1	1	1	0

Table B1K-54 Age Group = 41-50 Gender = FEMALE Occupational group = MANUAL Total cases = 135

Threshold dB HL≥	0	5	10	15	20	25	30	35	40	45	50	55	65	75	85	95
Prevalence	**94.6**	**68.9**	**44.2**	**28.5**	**12.8**	**7.9**	**6.3**	**6.3**	**5.8**	**3.8**	**1.7**	**0.7**	**0.2**	**0.2**	**0.2**	**0.2**
Lower CI	87.6	59.2	35.3	20.8	8.2	4.8	3.8	3.8	3.2	1.9	0.7	0.4	0.0	0.0	0.0	0.0
Upper CI	97.7	77.1	53.6	37.5	19.4	12.8	10.5	10.5	10.1	7.8	4.2	1.4	1.0	1.0	1.0	1.0
Number	130	100	64	46	29	20	17	17	14	9	6	4	1	1	1	1

Table B1K-55 Age Group = 51-60 Gender = FEMALE Occupational group = MANUAL Total cases = 166

Threshold dB HL≥	0	5	10	15	20	25	30	35	40	45	50	55	65	75	85	95
Prevalence	**94.7**	**74.6**	**51.7**	**31.1**	**22.8**	**14.8**	**9.2**	**6.5**	**4.6**	**1.8**	**0.9**	**0.8**	**0.2**	**0.2**	**0.2**	**0.2**
Lower CI	88.6	65.5	42.7	23.9	16.5	9.7	5.6	3.8	2.5	0.9	0.5	0.4	0.0	0.0	0.0	0.0
Upper CI	97.7	82.0	60.6	39.2	30.6	21.9	14.8	10.9	8.6	3.5	1.8	1.6	1.0	1.0	1.0	1.0
Number	159	134	103	75	53	35	25	18	13	10	7	6	1	1	1	1

Table B1K-56 Age Group = 61-70 Gender = FEMALE Occupational group = MANUAL Total cases = 126

Threshold dB HL≥	0	5	10	15	20	25	30	35	40	45	50	55	65	75	85	95
Prevalence	**95.6**	**84.8**	**67.3**	**44.2**	**28.7**	**21.9**	**15.8**	**11.1**	**7.7**	**5.9**	**4.4**	**4.0**	**2.8**	**2.1**	**2.1**	**1.6**
Lower CI	87.4	74.8	57.1	34.9	22.0	16.6	13.6	7.2	6.0	4.5	3.5	3.1	2.1	1.6	1.6	0.5
Upper CI	98.6	91.3	76.0	53.9	36.4	28.4	18.2	16.7	9.7	7.6	5.6	5.2	3.7	2.8	2.8	5.7
Number	123	111	91	70	52	41	35	25	20	17	13	11	6	3	3	1

Prevalence by age group, gender and occupational group for the better ear over 1 kHz

Table B1K-57 Age Group = 71-80 Gender = FEMALE Occupational group = MANUAL Total cases = 76

	Threshold dB HL≥															
	0	5	10	15	20	25	30	35	40	45	50	55	65	75	85	95
Prevalence	100.0	95.0	86.3	71.9	62.4	45.5	32.5	25.5	22.1	20.5	15.5	6.5	4.8	3.1	2.6	1.3
Lower CI	95.2	79.2	70.8	58.9	50.7	34.9	22.6	16.1	13.1	11.6	7.4	4.0	2.7	1.4	1.1	0.3
Upper CI	100.0	98.9	94.2	82.0	72.8	56.5	44.2	38.0	34.9	33.6	29.4	10.6	8.4	6.6	6.2	5.2
Number	76	73	68	62	57	49	43	35	30	26	19	12	9	6	5	2

Table B1K-58 Age Group = 18-30 Gender = MALE Occupational group = MANUAL Total cases = 109

	Threshold dB HL≥															
	0	5	10	15	20	25	30	35	40	45	50	55	65	75	85	95
Prevalence	78.8	49.3	20.4	7.5	0.8	0.1	0.1	0.1	0.1	0.1	0.1	0.1	0.0	0.0	0.0	0.0
Lower CI	67.2	36.9	11.9	3.8	0.3	0.0	0.0	0.0	0.0	0.0	0.0	0.0	0.0	0.0	0.0	0.0
Upper CI	87.1	61.8	32.7	14.0	2.3	0.4	0.4	0.4	0.4	0.4	0.4	0.4	3.4	3.4	3.4	3.4
Number	88	55	29	15	4	1	1	1	1	1	1	1	0	0	0	0

Table B1K-59 Age Group = 31-40 Gender = MALE Occupational group = MANUAL Total cases = 80

	Threshold dB HL≥															
	0	5	10	15	20	25	30	35	40	45	50	55	65	75	85	95
Prevalence	81.2	56.8	31.5	20.8	8.4	6.5	3.1	2.3	1.9	1.9	1.9	1.9	1.9	1.9	1.9	1.9
Lower CI	66.6	43.7	20.0	11.1	3.5	2.2	0.9	0.5	0.3	0.3	0.3	0.3	0.3	0.3	0.3	0.3
Upper CI	90.3	69.0	45.7	35.5	18.9	17.7	10.0	9.6	10.2	10.2	10.2	10.2	10.2	10.2	10.2	10.2
Number	69	48	27	19	10	6	3	2	1	1	1	1	1	1	1	1

Table B1K-60 Age Group = 41-50 Gender = MALE Occupational group = MANUAL Total cases = 127

	Threshold dB HL≥															
	0	5	10	15	20	25	30	35	40	45	50	55	65	75	85	95
Prevalence	90.3	66.5	42.1	23.5	8.6	5.6	4.3	2.9	2.4	1.0	0.7	0.7	0.5	0.5	0.2	0.0
Lower CI	82.2	55.7	31.9	16.2	5.2	3.4	2.3	1.4	1.1	0.5	0.4	0.4	0.1	0.1	0.0	0.0
Upper CI	95.0	75.8	53.1	32.7	13.8	9.3	7.8	5.6	5.2	2.0	1.3	1.3	1.7	1.7	1.3	2.9
Number	117	91	60	38	20	16	12	9	7	4	3	3	2	2	1	0

Table B1K-61 Age Group = 51-60 Gender = MALE Occupational group = MANUAL Total cases = 199

	Threshold dB HL≥															
	0	5	10	15	20	25	30	35	40	45	50	55	65	75	85	95
Prevalence	96.6	75.0	55.4	34.0	23.5	14.9	10.5	6.6	5.0	3.7	3.6	2.4	1.2	0.7	0.0	0.0
Lower CI	91.7	67.4	47.5	27.1	17.7	10.7	7.3	4.8	3.4	2.4	2.2	1.2	0.5	0.2	0.0	0.0
Upper CI	98.7	81.4	63.0	41.6	30.6	20.4	14.9	9.2	7.3	5.8	5.6	4.6	2.8	2.2	1.9	1.9
Number	194	161	130	85	60	45	36	28	21	16	15	10	6	3	0	0

Table B1K-62 Age Group = 61-70 Gender = MALE Occupational group = MANUAL Total cases = 173

	Threshold dB HL≥															
	0	5	10	15	20	25	30	35	40	45	50	55	65	75	85	95
Prevalence	94.5	84.8	57.2	46.8	37.1	22.3	14.3	10.6	6.9	5.9	5.1	3.3	2.3	1.2	0.9	0.6
Lower CI	87.8	74.3	46.3	36.8	29.0	16.9	10.1	7.0	4.9	4.0	3.3	1.9	1.2	0.6	0.3	0.1
Upper CI	97.6	91.4	67.5	57.1	46.0	29.0	19.8	15.8	9.6	8.5	7.7	5.5	4.2	2.7	2.4	2.2
Number	167	155	121	100	83	59	43	34	27	23	19	12	9	6	4	2

Table B1K-63 Age Group = 71-80 Gender = MALE Occupational group = MANUAL Total cases = 72

	Threshold dB HL≥															
	0	5	10	15	20	25	30	35	40	45	50	55	65	75	85	95
Prevalence	100.0	94.3	86.7	74.0	51.2	33.9	22.5	17.9	15.9	15.9	12.7	8.8	3.4	1.4	0.6	0.6
Lower CI	94.9	80.9	73.8	61.8	43.0	25.7	16.4	12.1	10.2	10.2	7.7	5.1	1.5	0.5	0.1	0.1
Upper CI	100.0	98.5	93.7	83.4	59.4	43.1	30.0	25.5	23.8	23.8	20.3	14.5	7.3	4.1	3.1	3.1
Number	72	69	63	55	46	41	32	26	22	22	18	14	6	3	1	1

Prevalence by age group, gender and occupational group for the better ear over 2 kHz

Table B2K-1 Age Group = 18-80 Gender = OVERALL Occupational group = OVERALL Total cases = 2666

	Threshold dB HL≥															
	0	5	10	15	20	25	30	35	40	45	50	55	65	75	85	95
**Prevalence**	**86.4**	**65.5**	**45.7**	**31.6**	**23.1**	**16.8**	**12.5**	**9.3**	**7.2**	**4.8**	**3.5**	**2.8**	**1.5**	**0.8**	**0.4**	**0.2**
Lower CI	84.5	63.2	43.6	29.9	21.7	15.6	11.4	8.4	6.4	4.3	3.0	2.4	1.2	0.5	0.2	0.1
Upper CI	88.1	67.6	47.8	33.4	24.6	18.2	13.6	10.3	8.0	5.5	3.9	3.3	1.8	1.1	0.7	0.5
Number	2440	2010	1533	1166	939	742	596	479	393	302	230	192	103	48	25	12

Table B2K-2 Age Group = 18-80 Gender = FEMALE Occupational group = OVERALL Total cases = 1389

	Threshold dB HL≥															
	0	5	10	15	20	25	30	35	40	45	50	55	65	75	85	95
**Prevalence**	**88.3**	**66.1**	**44.7**	**30.0**	**22.1**	**15.6**	**10.9**	**8.6**	**6.5**	**3.9**	**2.7**	**2.1**	**1.3**	**0.7**	**0.3**	**0.2**
Lower CI	86.0	63.2	42.1	27.9	20.3	13.9	9.6	7.4	5.5	3.3	2.3	1.7	1.0	0.5	0.2	0.1
Upper CI	90.3	68.8	47.4	32.2	24.1	17.4	12.4	9.9	7.8	4.6	3.2	2.6	1.7	1.0	0.5	0.4
Number	1272	1029	763	558	445	340	272	222	180	128	96	78	47	27	13	8

Table B2K-3 Age Group = 18-80 Gender = MALE Occupational group = OVERALL Total cases = 1277

	Threshold dB HL≥															
	0	5	10	15	20	25	30	35	40	45	50	55	65	75	85	95
**Prevalence**	**84.3**	**64.8**	**46.7**	**33.5**	**24.1**	**18.2**	**14.2**	**10.1**	**7.8**	**5.9**	**4.3**	**3.7**	**1.7**	**0.8**	**0.5**	**0.3**
Lower CI	81.1	61.3	43.5	30.8	21.9	16.4	12.6	8.8	6.8	5.0	3.5	3.0	1.3	0.4	0.2	0.1
Upper CI	87.1	68.1	50.0	36.3	26.5	20.2	15.8	11.4	9.1	7.0	5.2	4.5	2.4	1.4	1.1	1.0
Number	1168	981	770	608	494	402	324	257	213	174	134	114	56	21	12	4

Table B2K-4 Age Group = 18-30 Gender = OVERALL Occupational group = OVERALL Total cases = 401

	Threshold dB HL≥															
	0	5	10	15	20	25	30	35	40	45	50	55	65	75	85	95
**Prevalence**	**71.9**	**40.8**	**21.9**	**9.3**	**3.1**	**2.2**	**0.9**	**0.5**	**0.5**	**0.2**	**0.0**	**0.0**	**0.0**	**0.0**	**0.0**	**0.0**
Lower CI	66.2	35.3	17.6	6.6	1.7	1.1	0.4	0.2	0.2	0.1	0.0	0.0	0.0	0.0	0.0	0.0
Upper CI	76.9	46.6	27.0	13.0	5.5	4.2	2.2	1.0	1.0	0.6	0.1	0.1	0.1	0.9	0.9	0.9
Number	304	181	100	43	21	16	9	7	7	4	2	2	1	0	0	0

Table B2K-5 Age Group = 31-40 Gender = OVERALL Occupational group = OVERALL Total cases = 332

	Threshold dB HL≥															
	0	5	10	15	20	25	30	35	40	45	50	55	65	75	85	95
**Prevalence**	**81.3**	**50.3**	**25.6**	**13.8**	**9.0**	**5.1**	**3.4**	**1.9**	**1.6**	**1.6**	**1.1**	**0.9**	**0.7**	**0.6**	**0.6**	**0.6**
Lower CI	75.6	43.7	20.4	10.0	6.0	3.1	1.9	0.9	0.7	0.7	0.4	0.3	0.1	0.1	0.1	0.1
Upper CI	86.0	56.8	31.6	18.9	13.3	8.2	6.0	4.1	3.8	3.8	3.1	3.0	3.0	3.1	3.1	3.1
Number	284	186	104	59	40	27	20	12	10	10	7	5	2	1	1	1

Table B2K-6 Age Group = 41-50 Gender = OVERALL Occupational group = OVERALL Total cases = 450

	Threshold dB HL≥															
	0	5	10	15	20	25	30	35	40	45	50	55	65	75	85	95
**Prevalence**	**88.6**	**67.7**	**41.4**	**23.2**	**14.0**	**10.1**	**6.9**	**4.5**	**3.1**	**1.9**	**1.2**	**1.1**	**0.7**	**0.3**	**0.2**	**0.1**
Lower CI	85.0	62.8	36.6	19.4	11.2	7.7	5.1	3.3	2.0	1.2	0.7	0.6	0.3	0.1	0.1	0.0
Upper CI	91.5	72.2	46.4	27.5	17.5	13.1	9.1	6.3	4.6	3.0	2.1	2.1	1.5	0.9	0.7	0.3
Number	407	322	213	130	90	66	51	39	27	18	13	12	7	4	3	1

Table B2K-7 Age Group = 51-60 Gender = OVERALL Occupational group = OVERALL Total cases = 682

	Threshold dB HL≥															
	0	5	10	15	20	25	30	35	40	45	50	55	65	75	85	95
**Prevalence**	**95.9**	**78.9**	**57.4**	**40.4**	**29.2**	**19.5**	**14.8**	**10.3**	**7.5**	**5.6**	**3.8**	**3.3**	**1.3**	**0.8**	**0.1**	**0.1**
Lower CI	93.8	75.2	53.3	36.6	25.9	16.9	12.5	8.4	5.9	4.3	2.7	2.3	0.7	0.3	0.0	0.0
Upper CI	97.2	82.2	61.4	44.4	32.8	22.5	17.5	12.7	9.4	7.3	5.2	4.6	2.3	1.8	0.3	0.3
Number	654	561	429	329	255	188	148	109	87	69	48	41	18	9	3	3

Prevalence by age group, gender and occupational group for the better ear over 2 kHz

Table B2K-8 Age Group = 61-70 Gender = OVERALL Occupational group = OVERALL Total cases = 528

Threshold dB HL≥	0	5	10	15	20	25	30	35	40	45	50	55	65	75	85	95
Prevalence	**97.9**	**90.9**	**77.3**	**61.8**	**49.0**	**36.2**	**26.4**	**19.6**	**14.3**	**9.1**	**7.0**	**6.2**	**3.3**	**1.6**	**0.8**	**0.6**
Lower CI	95.7	87.1	72.3	56.5	43.7	31.6	22.5	16.5	11.7	7.5	5.6	4.8	2.4	1.0	0.4	0.3
Upper CI	99.0	93.7	81.6	66.9	54.2	41.0	30.6	23.2	17.3	11.0	8.7	7.8	4.4	2.5	1.6	1.3
Number	520	491	434	364	310	251	194	161	131	100	81	70	38	17	10	6

Table B2K-9 Age Group = 71-80 Gender = OVERALL Occupational group = OVERALL Total cases = 273

Threshold dB HL≥	0	5	10	15	20	25	30	35	40	45	50	55	65	75	85	95
Prevalence	**99.5**	**98.4**	**89.0**	**81.5**	**72.3**	**59.2**	**48.0**	**40.0**	**32.5**	**21.3**	**15.2**	**11.4**	**6.1**	**2.7**	**1.1**	**0.1**
Lower CI	98.0	95.1	82.0	74.1	64.7	51.4	41.0	33.4	26.5	17.5	12.6	9.0	4.5	1.7	0.6	0.0
Upper CI	99.9	99.5	93.5	87.2	78.9	66.5	55.0	47.0	39.2	25.6	18.2	14.3	8.2	4.2	2.2	0.8
Number	271	269	253	241	223	194	174	151	131	101	79	62	37	17	8	1

Table B2K-10 Age Group = 18-80 Gender = OVERALL Occupational group = NON-MANUAL Total cases = 1185

Threshold dB HL≥	0	5	10	15	20	25	30	35	40	45	50	55	65	75	85	95
Prevalence	**84.1**	**61.3**	**42.0**	**28.8**	**20.6**	**15.2**	**11.7**	**8.9**	**7.0**	**3.7**	**2.4**	**1.9**	**1.0**	**0.5**	**0.2**	**0.1**
Lower CI	81.1	58.1	39.1	26.4	18.8	13.6	10.5	7.9	6.1	3.2	2.1	1.6	0.7	0.3	0.1	0.0
Upper CI	86.7	64.5	44.9	31.3	22.6	16.8	13.0	10.1	8.1	4.4	2.9	2.3	1.4	0.8	0.4	0.2
Number	1060	842	619	458	358	273	220	176	143	99	68	54	35	18	8	5

Table B2K-11 Age Group = 18-80 Gender = OVERALL Occupational group = MANUAL Total cases = 1476

Threshold dB HL≥	0	5	10	15	20	25	30	35	40	45	50	55	65	75	85	95
Prevalence	**88.4**	**69.4**	**47.9**	**33.3**	**24.7**	**18.5**	**13.2**	**9.5**	**7.4**	**5.3**	**4.2**	**3.6**	**1.8**	**0.9**	**0.6**	**0.4**
Lower CI	85.7	66.1	44.8	30.7	22.6	16.7	11.8	8.4	6.5	4.6	3.5	3.0	1.4	0.6	0.3	0.2
Upper CI	90.6	72.5	50.9	35.9	27.0	20.5	14.9	10.7	8.5	6.2	5.0	4.3	2.3	1.4	1.0	0.8
Number	1376	1165	911	705	578	468	376	303	250	203	162	138	68	30	17	7

Table B2K-12 Age Group = 18-30 Gender = FEMALE Occupational group = OVERALL Total cases = 224

Threshold dB HL≥	0	5	10	15	20	25	30	35	40	45	50	55	65	75	85	95
Prevalence	**76.9**	**43.8**	**21.2**	**7.1**	**2.6**	**2.1**	**1.0**	**0.8**	**0.8**	**0.3**	**0.0**	**0.0**	**0.0**	**0.0**	**0.0**	**0.0**
Lower CI	70.0	36.7	16.0	4.4	1.4	1.0	0.4	0.3	0.3	0.1	0.0	0.0	0.0	0.0	0.0	0.0
Upper CI	82.6	51.2	27.6	11.2	4.6	4.0	2.1	1.8	1.8	1.1	1.7	1.7	1.7	1.7	1.7	1.7
Number	177	106	55	22	12	10	6	5	5	2	0	0	0	0	0	0

Table B2K-13 Age Group = 31-40 Gender = FEMALE Occupational group = OVERALL Total cases = 190

Threshold dB HL≥	0	5	10	15	20	25	30	35	40	45	50	55	65	75	85	95
Prevalence	**83.2**	**50.2**	**20.5**	**10.9**	**7.2**	**4.8**	**3.0**	**2.2**	**1.6**	**1.6**	**0.5**	**0.5**	**0.2**	**0.0**	**0.0**	**0.0**
Lower CI	76.3	42.1	15.2	7.5	4.6	2.9	1.6	1.0	0.6	0.6	0.2	0.2	0.0	0.0	0.0	0.0
Upper CI	88.4	58.3	27.0	15.7	11.3	8.0	5.6	4.7	4.1	4.1	1.7	1.7	1.2	2.0	2.0	2.0
Number	163	108	55	34	23	17	12	8	6	6	3	3	1	0	0	0

Table B2K-14 Age Group = 41-50 Gender = FEMALE Occupational group = OVERALL Total cases = 247

Threshold dB HL≥	0	5	10	15	20	25	30	35	40	45	50	55	65	75	85	95
Prevalence	**90.1**	**65.6**	**40.9**	**21.4**	**12.6**	**9.8**	**6.1**	**3.8**	**2.0**	**1.1**	**1.1**	**1.0**	**0.6**	**0.1**	**0.1**	**0.1**
Lower CI	85.0	58.9	34.6	16.8	9.3	6.8	4.1	2.5	1.1	0.5	0.5	0.4	0.2	0.0	0.0	0.0
Upper CI	93.6	71.7	47.6	26.9	16.9	13.8	9.0	5.9	3.6	2.6	2.6	2.5	2.1	0.6	0.6	0.6
Number	227	174	115	67	46	35	26	20	11	6	6	5	3	1	1	1

Prevalence by age group, gender and occupational group for the better ear over 2 kHz

Table B2K-15 Age Group = 51-60 Gender = FEMALE Occupational group = OVERALL Total cases = 341

	Threshold dB HL≥															
	**0**	**5**	**10**	**15**	**20**	**25**	**30**	**35**	**40**	**45**	**50**	**55**	**65**	**75**	**85**	**95**
**Prevalence**	**95.0**	**78.2**	**55.8**	**36.8**	**24.9**	**13.6**	**10.2**	**6.5**	**5.1**	**3.4**	**1.5**	**1.4**	**0.7**	**0.6**	**0.2**	**0.2**
**Lower CI**	91.7	72.9	50.0	31.6	20.4	10.6	7.5	4.6	3.5	2.1	0.9	0.8	0.3	0.2	0.1	0.1
**Upper CI**	97.1	82.7	61.5	42.4	29.9	17.3	13.6	9.2	7.3	5.4	2.7	2.6	1.8	1.5	0.6	0.6
**Number**	325	278	212	151	111	73	58	41	35	25	14	12	6	5	3	3

Table B2K-16 Age Group = 61-70 Gender = FEMALE Occupational group = OVERALL Total cases = 243

	Threshold dB HL≥															
	**0**	**5**	**10**	**15**	**20**	**25**	**30**	**35**	**40**	**45**	**50**	**55**	**65**	**75**	**85**	**95**
**Prevalence**	**97.2**	**89.0**	**74.8**	**59.0**	**46.2**	**31.4**	**20.8**	**15.9**	**10.3**	**5.2**	**4.2**	**3.3**	**2.6**	**1.7**	**0.9**	**0.8**
**Lower CI**	93.4	83.1	67.7	51.7	39.0	25.2	16.0	11.9	7.2	4.0	3.1	2.4	1.7	1.0	0.4	0.3
**Upper CI**	98.9	93.1	80.9	65.9	53.5	38.3	26.6	20.9	14.5	6.6	5.6	4.7	4.0	3.0	2.2	2.1
**Number**	238	223	193	158	134	100	74	62	49	34	29	23	16	9	5	4

Table B2K-17 Age Group = 71-80 Gender = FEMALE Occupational group = OVERALL Total cases = 144

	Threshold dB HL≥															
	**0**	**5**	**10**	**15**	**20**	**25**	**30**	**35**	**40**	**45**	**50**	**55**	**65**	**75**	**85**	**95**
**Prevalence**	**99.1**	**97.3**	**89.0**	**80.5**	**72.2**	**59.0**	**45.5**	**40.6**	**33.4**	**19.4**	**14.7**	**10.6**	**5.6**	**3.3**	**0.9**	**0.0**
**Lower CI**	96.6	91.7	78.9	69.7	61.5	48.0	36.0	31.5	25.0	15.4	11.4	7.9	3.8	1.9	0.4	0.0
**Upper CI**	99.8	99.2	94.6	88.0	80.8	69.2	55.4	50.3	43.0	24.3	18.6	14.2	8.3	5.7	2.2	2.6
**Number**	142	140	133	126	119	105	96	86	74	55	44	35	21	12	4	0

Table B2K-18 Age Group = 18-30 Gender = MALE Occupational group = OVERALL Total cases = 177

	Threshold dB HL≥															
	**0**	**5**	**10**	**15**	**20**	**25**	**30**	**35**	**40**	**45**	**50**	**55**	**65**	**75**	**85**	**95**
**Prevalence**	**66.6**	**37.6**	**22.7**	**11.6**	**3.6**	**2.2**	**0.8**	**0.1**	**0.1**	**0.1**	**0.1**	**0.1**	**0.0**	**0.0**	**0.0**	**0.0**
**Lower CI**	57.4	29.3	16.2	7.3	1.5	0.7	0.2	0.0	0.0	0.0	0.0	0.0	0.0	0.0	0.0	0.0
**Upper CI**	74.6	46.7	30.8	18.1	8.3	6.5	4.0	0.2	0.2	0.2	0.2	0.2	0.2	2.1	2.1	2.1
**Number**	127	75	45	21	9	6	3	2	2	2	2	2	1	0	0	0

Table B2K-19 Age Group = 31-40 Gender = MALE Occupational group = OVERALL Total cases = 142

	Threshold dB HL≥															
	**0**	**5**	**10**	**15**	**20**	**25**	**30**	**35**	**40**	**45**	**50**	**55**	**65**	**75**	**85**	**95**
**Prevalence**	**79.1**	**50.3**	**31.0**	**17.1**	**11.0**	**5.4**	**3.8**	**1.7**	**1.7**	**1.7**	**1.7**	**1.3**	**1.2**	**1.2**	**1.2**	**1.2**
**Lower CI**	69.1	40.0	22.1	10.5	6.0	2.4	1.5	0.5	0.5	0.5	0.5	0.3	0.2	0.2	0.2	0.2
**Upper CI**	86.4	60.6	41.5	26.8	19.4	11.5	9.2	6.3	6.3	6.3	6.3	6.4	6.5	6.5	6.5	6.5
**Number**	121	78	49	25	17	10	8	4	4	4	4	2	1	1	1	1

Table B2K-20 Age Group = 41-50 Gender = MALE Occupational group = OVERALL Total cases = 203

	Threshold dB HL≥															
	**0**	**5**	**10**	**15**	**20**	**25**	**30**	**35**	**40**	**45**	**50**	**55**	**65**	**75**	**85**	**95**
**Prevalence**	**87.1**	**70.0**	**42.0**	**25.2**	**15.7**	**10.5**	**7.7**	**5.3**	**4.3**	**2.7**	**1.3**	**1.3**	**0.7**	**0.6**	**0.4**	**0.0**
**Lower CI**	81.3	62.7	34.7	19.4	11.2	7.0	5.1	3.3	2.5	1.5	0.6	0.6	0.3	0.2	0.1	0.0
**Upper CI**	91.2	76.5	49.6	32.2	21.5	15.3	11.5	8.5	7.2	4.7	2.8	2.8	1.9	1.8	1.4	1.9
**Number**	180	148	98	63	44	31	25	19	16	12	7	7	4	3	2	0

Table B2K-21 Age Group = 51-60 Gender = MALE Occupational group = OVERALL Total cases = 341

	Threshold dB HL≥															
	**0**	**5**	**10**	**15**	**20**	**25**	**30**	**35**	**40**	**45**	**50**	**55**	**65**	**75**	**85**	**95**
**Prevalence**	**96.8**	**79.7**	**59.0**	**44.5**	**34.1**	**26.2**	**20.0**	**14.5**	**10.2**	**8.1**	**6.3**	**5.4**	**2.0**	**1.0**	**0.0**	**0.0**
**Lower CI**	94.1	74.3	53.2	39.0	29.1	21.8	16.2	11.2	7.6	5.9	4.3	3.6	1.0	0.3	0.0	0.0
**Upper CI**	98.3	84.2	64.6	50.1	39.3	31.2	24.5	18.7	13.5	11.1	9.0	8.0	4.0	3.2	1.1	1.1
**Number**	329	283	217	178	144	115	90	68	52	44	34	29	12	4	0	0

Prevalence by age group, gender and occupational group for the better ear over 2 kHz

Table B2K-22 Age Group = 61-70 Gender = MALE Occupational group = OVERALL Total cases = 285

	Threshold dB HL≥															
	0	5	10	15	20	25	30	35	40	45	50	55	65	75	85	95
Prevalence	**98.6**	**93.0**	**80.1**	**65.2**	**52.2**	**41.7**	**32.8**	**23.9**	**19.0**	**13.7**	**10.2**	**9.5**	**4.0**	**1.5**	**0.7**	**0.3**
Lower CI	96.2	87.6	72.8	57.3	44.7	35.4	27.0	19.3	15.0	10.5	7.6	6.9	2.7	0.8	0.3	0.1
Upper CI	99.5	96.2	85.9	72.3	59.5	48.2	39.2	29.1	23.7	17.5	13.7	12.9	6.0	3.1	1.7	1.2
Number	282	268	241	206	176	151	120	99	82	66	52	47	22	8	5	2

Table B2K-23 Age Group = 71-80 Gender = MALE Occupational group = OVERALL Total cases = 129

	Threshold dB HL≥															
	0	5	10	15	20	25	30	35	40	45	50	55	65	75	85	95
Prevalence	**100.0**	**100.0**	**88.7**	**82.5**	**72.1**	**58.6**	**50.7**	**38.6**	**31.2**	**24.3**	**16.4**	**12.7**	**6.9**	**1.7**	**1.5**	**0.4**
Lower CI	97.1	97.1	78.0	72.5	61.6	48.6	41.4	30.0	23.8	17.7	12.2	8.7	4.2	0.8	0.6	0.1
Upper CI	100.0	100.0	94.5	89.4	80.7	67.9	60.0	47.9	39.8	32.3	21.7	18.1	10.9	4.0	3.7	2.0
Number	129	129	120	115	104	89	78	65	57	46	35	27	16	5	4	1

Table B2K-24 Age Group = 18-30 Gender = OVERALL Occupational group = NON-MANUAL Total cases = 181

	Threshold dB HL≥															
	0	5	10	15	20	25	30	35	40	45	50	55	65	75	85	95
Prevalence	**65.0**	**31.5**	**15.9**	**6.1**	**1.2**	**1.0**	**0.5**	**0.5**	**0.5**	**0.1**	**0.1**	**0.1**	**0.1**	**0.0**	**0.0**	**0.0**
Lower CI	56.4	24.5	10.6	3.1	0.5	0.3	0.1	0.1	0.1	0.0	0.0	0.0	0.0	0.0	0.0	0.0
Upper CI	72.6	39.5	23.1	11.8	3.2	3.0	2.4	2.4	2.4	2.2	2.2	2.2	2.2	2.1	2.1	2.1
Number	126	66	34	13	5	4	2	2	2	1	1	1	1	0	0	0

Table B2K-25 Age Group = 31-40 Gender = OVERALL Occupational group = NON-MANUAL Total cases = 148

	Threshold dB HL≥															
	0	5	10	15	20	25	30	35	40	45	50	55	65	75	85	95
Prevalence	**82.2**	**47.7**	**21.9**	**11.7**	**5.0**	**2.9**	**2.0**	**1.1**	**0.4**	**0.4**	**0.0**	**0.0**	**0.0**	**0.0**	**0.0**	**0.0**
Lower CI	73.1	38.0	14.8	6.4	2.4	1.3	0.9	0.6	0.1	0.1	0.0	0.0	0.0	0.0	0.0	0.0
Upper CI	88.8	57.6	31.2	20.3	10.3	6.4	4.2	2.2	1.6	1.6	2.5	2.5	2.5	2.5	2.5	2.5
Number	127	73	36	18	10	7	6	4	2	2	0	0	0	0	0	0

Table B2K-26 Age Group = 41-50 Gender = OVERALL Occupational group = NON-MANUAL Total cases = 188

	Threshold dB HL≥															
	0	5	10	15	20	25	30	35	40	45	50	55	65	75	85	95
Prevalence	**87.6**	**61.9**	**35.6**	**18.4**	**11.7**	**6.5**	**4.5**	**2.7**	**1.4**	**0.4**	**0.4**	**0.4**	**0.4**	**0.2**	**0.2**	**0.0**
Lower CI	81.4	53.5	28.3	12.9	7.6	3.9	3.0	1.9	0.8	0.1	0.1	0.1	0.1	0.0	0.0	0.0
Upper CI	91.9	69.7	43.7	25.4	17.4	10.5	6.8	3.8	2.4	1.3	1.3	1.3	1.3	1.3	1.3	2.0
Number	167	122	76	40	27	17	13	10	5	2	2	2	2	1	1	0

Table B2K-27 Age Group = 51-60 Gender = OVERALL Occupational group = NON-MANUAL Total cases = 317

	Threshold dB HL≥															
	0	5	10	15	20	25	30	35	40	45	50	55	65	75	85	95
Prevalence	**94.8**	**78.3**	**53.3**	**37.0**	**26.0**	**15.9**	**12.3**	**8.2**	**6.0**	**3.4**	**1.5**	**1.1**	**0.9**	**0.5**	**0.2**	**0.2**
Lower CI	91.8	72.9	47.4	31.9	21.6	12.9	9.9	5.2	3.4	2.3	0.9	0.6	0.4	0.2	0.0	0.0
Upper CI	96.7	82.8	59.1	42.4	31.1	19.5	15.1	12.8	10.5	5.2	2.6	2.2	2.0	1.4	0.7	0.7
Number	296	250	177	133	97	65	53	38	30	23	12	9	7	4	2	2

Table B2K-28 Age Group = 61-70 Gender = OVERALL Occupational group = NON-MANUAL Total cases = 228

	Threshold dB HL≥															
	0	5	10	15	20	25	30	35	40	45	50	55	65	75	85	95
Prevalence	**96.8**	**91.8**	**77.9**	**59.8**	**49.9**	**36.0**	**26.8**	**19.9**	**15.2**	**7.3**	**5.0**	**3.8**	**2.7**	**1.4**	**0.6**	**0.4**
Lower CI	92.5	86.7	71.1	51.5	42.0	29.2	20.7	16.1	12.0	5.3	3.3	2.5	1.5	0.8	0.2	0.1
Upper CI	98.7	95.1	83.5	67.6	57.9	43.5	33.9	24.4	18.9	9.9	7.6	5.8	4.6	2.5	1.5	1.2
Number	223	211	181	146	125	96	71	59	49	33	25	21	14	8	4	3

Prevalence by age group, gender and occupational group for the better ear over 2 kHz

Table B2K-29 Age Group = 71-80 Gender = OVERALL Occupational group = NON-MANUAL Total cases = 123

Threshold dB HL≥	0	5	10	15	20	25	30	35	40	45	50	55	65	75	85	95
Prevalence	**98.4**	**96.8**	**93.2**	**84.4**	**69.2**	**63.6**	**53.1**	**44.1**	**37.0**	**21.2**	**14.8**	**11.6**	**4.1**	**1.9**	**0.4**	**0.0**
Lower CI	95.5	91.0	86.6	75.9	57.7	52.3	45.0	36.5	31.1	16.7	12.5	9.1	2.2	0.7	0.1	0.0
Upper CI	99.4	98.9	96.7	90.3	78.8	73.6	61.1	51.9	43.3	26.4	17.5	14.8	7.3	4.8	2.0	3.0
Number	121	120	115	108	94	84	75	63	55	38	28	21	11	5	1	0

Table B2K-30 Age Group = 18-30 Gender = OVERALL Occupational group = MANUAL Total cases = 218

Threshold dB HL≥	0	5	10	15	20	25	30	35	40	45	50	55	65	75	85	95
Prevalence	**76.8**	**49.1**	**26.5**	**12.3**	**4.3**	**3.1**	**1.4**	**0.5**	**0.5**	**0.3**	**0.0**	**0.0**	**0.0**	**0.0**	**0.0**	**0.0**
Lower CI	69.0	40.4	20.2	8.1	2.2	1.4	0.5	0.2	0.2	0.1	0.0	0.0	0.0	0.0	0.0	0.0
Upper CI	83.2	57.9	34.1	18.0	8.2	6.8	4.3	1.1	1.1	0.9	0.2	0.2	1.7	1.7	1.7	1.7
Number	177	115	66	30	16	12	7	5	5	3	1	1	0	0	0	0

Table B2K-31 Age Group = 31-40 Gender = OVERALL Occupational group = MANUAL Total cases = 183

Threshold dB HL≥	0	5	10	15	20	25	30	35	40	45	50	55	65	75	85	95
Prevalence	**80.4**	**54.4**	**27.6**	**15.7**	**12.3**	**7.3**	**5.0**	**2.6**	**2.6**	**2.6**	**1.7**	**1.4**	**1.1**	**0.9**	**0.9**	**0.9**
Lower CI	72.2	45.0	20.4	10.2	7.4	4.0	2.3	1.0	1.0	1.0	0.6	0.4	0.2	0.2	0.2	0.2
Upper CI	86.7	63.5	36.1	23.4	19.8	13.0	10.6	6.5	6.5	6.5	4.9	4.8	4.9	5.0	5.0	5.0
Number	156	112	67	40	29	20	14	8	8	8	7	5	2	1	1	1

Table B2K-32 Age Group = 41-50 Gender = OVERALL Occupational group = MANUAL Total cases = 262

Threshold dB HL≥	0	5	10	15	20	25	30	35	40	45	50	55	65	75	85	95
Prevalence	**90.8**	**73.5**	**47.2**	**28.5**	**17.3**	**13.9**	**8.7**	**5.7**	**4.2**	**2.5**	**1.5**	**1.4**	**0.8**	**0.3**	**0.2**	**0.1**
Lower CI	85.8	66.7	40.0	22.6	12.9	9.8	6.0	3.8	2.5	1.4	0.8	0.7	0.3	0.1	0.1	0.0
Upper CI	94.1	79.3	54.6	35.3	22.9	19.3	12.5	8.4	6.9	4.1	2.9	2.9	2.2	0.9	0.7	0.5
Number	240	200	137	90	63	49	38	29	22	16	11	10	5	3	2	1

Table B2K-33 Age Group = 51-60 Gender = OVERALL Occupational group = MANUAL Total cases = 365

Threshold dB HL≥	0	5	10	15	20	25	30	35	40	45	50	55	65	75	85	95
Prevalence	**98.0**	**79.5**	**59.8**	**41.0**	**30.9**	**23.2**	**17.4**	**11.8**	**8.6**	**6.4**	**5.0**	**4.5**	**1.5**	**0.9**	**0.1**	**0.1**
Lower CI	95.8	73.9	53.5	35.7	26.2	19.0	13.9	8.8	6.3	4.6	3.4	3.0	0.7	0.3	0.0	0.0
Upper CI	99.1	84.1	65.7	46.6	36.0	28.0	21.7	15.5	11.7	8.8	7.3	6.7	3.4	3.1	0.5	0.5
Number	358	311	252	196	158	123	95	71	57	46	36	32	11	5	1	1

Table B2K-34 Age Group = 61-70 Gender = OVERALL Occupational group = MANUAL Total cases = 299

Threshold dB HL≥	0	5	10	15	20	25	30	35	40	45	50	55	65	75	85	95
Prevalence	**98.6**	**90.7**	**77.2**	**63.0**	**48.1**	**37.6**	**27.4**	**19.8**	**14.6**	**10.6**	**8.9**	**7.9**	**3.8**	**1.9**	**1.4**	**1.1**
Lower CI	96.0	84.7	70.3	55.8	40.8	31.2	22.0	15.6	11.4	8.0	6.5	5.7	2.8	1.3	1.0	0.8
Upper CI	99.5	94.5	83.0	69.6	55.5	44.5	33.5	24.8	18.5	13.9	12.1	11.0	5.1	2.8	2.0	1.6
Number	296	279	252	217	184	154	123	102	82	67	56	49	24	9	6	3

Table B2K-35 Age Group = 71-80 Gender = OVERALL Occupational group = MANUAL Total cases = 149

Threshold dB HL≥	0	5	10	15	20	25	30	35	40	45	50	55	65	75	85	95
Prevalence	**100.0**	**99.7**	**80.7**	**76.2**	**74.3**	**56.2**	**42.6**	**36.3**	**30.5**	**20.0**	**16.4**	**12.9**	**7.4**	**3.2**	**1.7**	**0.2**
Lower CI	97.5	98.5	68.4	64.4	62.6	45.9	34.9	29.2	23.9	16.4	12.9	9.6	5.1	1.8	0.8	0.0
Upper CI	100.0	100.0	89.0	85.1	83.3	66.1	50.6	44.0	38.0	24.2	20.8	16.9	10.8	5.4	3.4	1.3
Number	149	148	137	132	128	110	99	88	76	63	51	41	26	12	7	1

Prevalence by age group, gender and occupational group for the better ear over 2 kHz

Table B2K-36 Age Group = 18-80 Gender = FEMALE Occupational group = NON-MANUAL Total cases = 671

Threshold dB HL≥	0	5	10	15	20	25	30	35	40	45	50	55	65	75	85	95
**Prevalence**	**86.8**	**63.1**	**43.1**	**28.7**	**20.1**	**14.4**	**11.2**	**9.6**	**7.3**	**3.8**	**2.6**	**2.1**	**1.1**	**0.7**	**0.2**	**0.2**
**Lower CI**	83.5	59.2	39.7	26.1	17.7	12.3	9.7	8.3	6.3	3.2	2.2	1.7	0.7	0.4	0.1	0.1
**Upper CI**	89.5	66.8	46.5	31.5	22.8	16.8	12.8	11.0	8.3	4.5	3.1	2.7	1.7	1.3	0.6	0.4
**Number**	602	473	348	250	192	141	121	102	80	56	39	31	22	14	6	5

Table B2K-37 Age Group = 18-80 Gender = MALE Occupational group = NON-MANUAL Total cases = 514

Threshold dB HL≥	0	5	10	15	20	25	30	35	40	45	50	55	65	75	85	95
**Prevalence**	**80.8**	**59.3**	**40.8**	**28.9**	**21.2**	**16.0**	**12.3**	**8.1**	**6.7**	**3.7**	**2.2**	**1.6**	**0.9**	**0.3**	**0.1**	**0.0**
**Lower CI**	75.5	54.0	36.1	24.9	18.4	13.9	10.4	6.5	5.2	2.7	1.6	1.1	0.5	0.1	0.0	0.0
**Upper CI**	85.3	64.3	45.7	33.3	24.2	18.4	14.4	10.2	8.7	4.9	3.1	2.3	1.5	0.6	0.5	0.7
**Number**	458	369	271	208	166	132	99	74	63	43	29	23	13	4	2	0

Table B2K-38 Age Group = 18-80 Gender = FEMALE Occupational group = MANUAL Total cases = 716

Threshold dB HL≥	0	5	10	15	20	25	30	35	40	45	50	55	65	75	85	95
**Prevalence**	**89.5**	**69.5**	**45.0**	**30.0**	**23.1**	**16.8**	**10.9**	**8.1**	**6.4**	**3.9**	**3.0**	**2.5**	**1.4**	**0.8**	**0.5**	**0.3**
**Lower CI**	85.7	65.0	41.1	26.8	20.4	14.4	9.3	6.7	5.3	3.1	2.4	1.9	1.0	0.5	0.3	0.2
**Upper CI**	92.4	73.7	49.0	33.4	26.0	19.5	12.9	9.8	7.9	4.9	3.8	3.1	2.0	1.1	0.7	0.4
**Number**	668	554	413	306	251	198	151	120	100	72	57	47	25	13	7	3

Table B2K-39 Age Group = 18-80 Gender = MALE Occupational group = MANUAL Total cases = 760

Threshold dB HL≥	0	5	10	15	20	25	30	35	40	45	50	55	65	75	85	95
**Prevalence**	**87.2**	**69.3**	**51.0**	**37.1**	**26.5**	**20.4**	**15.7**	**10.9**	**8.5**	**6.9**	**5.5**	**4.9**	**2.2**	**1.1**	**0.7**	**0.4**
**Lower CI**	83.2	64.2	46.2	33.1	23.2	17.7	13.4	9.3	7.1	5.7	4.3	3.7	1.5	0.6	0.3	0.1
**Upper CI**	90.3	73.9	55.7	41.2	30.1	23.5	18.4	12.8	10.2	8.4	7.0	6.3	3.3	2.2	1.6	1.6
**Number**	708	611	498	399	327	270	225	183	150	131	105	91	43	17	10	4

Table B2K-40 Age Group = 18-30 Gender = FEMALE Occupational group = NON-MANUAL Total cases = 115

Threshold dB HL≥	0	5	10	15	20	25	30	35	40	45	50	55	65	75	85	95
**Prevalence**	**73.8**	**36.6**	**18.8**	**7.0**	**2.2**	**1.8**	**0.8**	**0.8**	**0.8**	**0.0**	**0.0**	**0.0**	**0.0**	**0.0**	**0.0**	**0.0**
**Lower CI**	64.6	27.9	12.5	3.8	0.8	0.6	0.1	0.1	0.1	0.0	0.0	0.0	0.0	0.0	0.0	0.0
**Upper CI**	81.3	46.3	27.3	12.5	5.9	5.5	4.5	4.5	4.5	3.2	3.2	3.2	3.2	3.2	3.2	3.2
**Number**	85	44	24	10	4	3	1	1	1	0	0	0	0	0	0	0

Table B2K-41 Age Group = 31-40 Gender = FEMALE Occupational group = NON-MANUAL Total cases = 86

Threshold dB HL≥	0	5	10	15	20	25	30	35	40	45	50	55	65	75	85	95
**Prevalence**	**84.0**	**46.0**	**16.8**	**8.3**	**3.2**	**2.5**	**2.5**	**2.1**	**0.7**	**0.7**	**0.0**	**0.0**	**0.0**	**0.0**	**0.0**	**0.0**
**Lower CI**	74.1	34.8	10.1	4.4	1.9	1.3	1.3	1.1	0.2	0.2	0.0	0.0	0.0	0.0	0.0	0.0
**Upper CI**	90.6	57.6	26.6	15.2	5.2	4.7	4.7	4.2	3.0	3.0	4.3	4.3	4.3	4.3	4.3	4.3
**Number**	73	43	18	11	6	5	5	4	2	2	0	0	0	0	0	0

Table B2K-42 Age Group = 41-50 Gender = FEMALE Occupational group = NON-MANUAL Total cases = 112

Threshold dB HL≥	0	5	10	15	20	25	30	35	40	45	50	55	65	75	85	95
**Prevalence**	**89.9**	**62.2**	**36.5**	**15.7**	**8.2**	**4.9**	**4.0**	**3.3**	**0.7**	**0.0**	**0.0**	**0.0**	**0.0**	**0.0**	**0.0**	**0.0**
**Lower CI**	81.7	52.5	28.2	11.0	5.9	3.0	2.7	2.1	0.2	0.0	0.0	0.0	0.0	0.0	0.0	0.0
**Upper CI**	94.7	71.0	45.6	21.8	11.3	7.8	5.7	5.1	2.8	3.3	3.3	3.3	3.3	3.3	3.3	3.3
**Number**	103	73	48	24	15	9	8	7	2	0	0	0	0	0	0	0

Prevalence by age group, gender and occupational group for the better ear over 2 kHz

Table B2K-43 Age Group = 51-60 Gender = FEMALE Occupational group = NON-MANUAL Total cases = 175

Threshold dB HL≥	0	5	10	15	20	25	30	35	40	45	50	55	65	75	85	95
Prevalence	**94.5**	**81.9**	**56.0**	**36.4**	**24.0**	**11.0**	**8.4**	**5.7**	**4.7**	**3.9**	**1.6**	**1.2**	**1.2**	**1.0**	**0.3**	**0.3**
Lower CI	90.0	75.1	48.1	29.6	18.1	7.8	5.7	3.7	2.9	2.2	0.8	0.5	0.5	0.4	0.1	0.1
Upper CI	97.1	87.2	63.7	43.9	31.0	15.2	12.2	8.7	7.5	6.9	3.4	3.0	3.0	2.6	1.2	1.2
Number	164	143	104	74	53	31	27	19	16	13	7	5	5	4	2	2

Table B2K-44 Age Group = 61-70 Gender = FEMALE Occupational group = NON-MANUAL Total cases = 116

Threshold dB HL≥	0	5	10	15	20	25	30	35	40	45	50	55	65	75	85	95
Prevalence	**94.8**	**88.9**	**75.8**	**60.3**	**47.6**	**30.9**	**22.0**	**19.4**	**13.6**	**5.2**	**3.6**	**3.0**	**2.6**	**1.9**	**0.7**	**0.7**
Lower CI	87.2	80.1	65.9	50.1	38.1	23.2	16.3	14.3	10.1	3.5	2.3	1.8	1.4	0.9	0.2	0.2
Upper CI	98.0	94.0	83.6	69.6	57.3	39.9	29.1	25.7	18.0	7.8	5.4	5.0	4.6	3.7	2.3	2.3
Number	112	106	91	73	62	45	35	32	26	17	13	11	9	6	3	3

Table B2K-45 Age Group = 71-80 Gender = FEMALE Occupational group = NON-MANUAL Total cases = 67

Threshold dB HL≥	0	5	10	15	20	25	30	35	40	45	50	55	65	75	85	95
Prevalence	**97.3**	**94.6**	**94.2**	**83.0**	**68.0**	**65.0**	**54.0**	**48.1**	**39.3**	**21.1**	**17.0**	**13.7**	**4.7**	**2.6**	**0.6**	**0.0**
Lower CI	92.8	85.0	84.8	70.0	50.4	47.9	43.4	39.0	33.6	17.0	14.4	10.5	2.3	0.9	0.1	0.0
Upper CI	99.0	98.2	97.9	91.1	81.6	79.0	64.3	57.2	45.2	25.8	20.0	17.7	9.4	7.5	3.4	5.4
Number	65	64	63	58	52	48	45	39	33	24	19	15	8	4	1	0

Table B2K-46 Age Group = 18-30 Gender = MALE Occupational group = NON-MANUAL Total cases = 66

Threshold dB HL≥	0	5	10	15	20	25	30	35	40	45	50	55	65	75	85	95
Prevalence	**55.4**	**26.0**	**12.8**	**5.0**	**0.1**	**0.1**	**0.1**	**0.1**	**0.1**	**0.1**	**0.1**	**0.1**	**0.1**	**0.0**	**0.0**	**0.0**
Lower CI	41.3	16.0	5.7	1.3	0.0	0.0	0.0	0.0	0.0	0.0	0.0	0.0	0.0	0.0	0.0	0.0
Upper CI	68.6	39.4	26.3	17.8	5.7	5.7	5.7	5.7	5.7	5.7	5.7	5.7	5.7	5.5	5.5	5.5
Number	41	22	10	3	1	1	1	1	1	1	1	1	1	0	0	0

Table B2K-47 Age Group = 31-40 Gender = MALE Occupational group = NON-MANUAL Total cases = 62

Threshold dB HL≥	0	5	10	15	20	25	30	35	40	45	50	55	65	75	85	95
Prevalence	**79.8**	**49.4**	**27.6**	**15.5**	**7.1**	**3.3**	**1.3**	**0.0**	**0.0**	**0.0**	**0.0**	**0.0**	**0.0**	**0.0**	**0.0**	**0.0**
Lower CI	62.6	33.4	15.4	6.4	2.4	0.9	0.2	0.0	0.0	0.0	0.0	0.0	0.0	0.0	0.0	0.0
Upper CI	90.3	65.6	44.4	33.0	19.3	11.8	6.9	5.8	5.8	5.8	5.8	5.8	5.8	5.8	5.8	5.8
Number	54	30	18	7	4	2	1	0	0	0	0	0	0	0	0	0

Table B2K-48 Age Group = 41-50 Gender = MALE Occupational group = NON-MANUAL Total cases = 76

Threshold dB HL≥	0	5	10	15	20	25	30	35	40	45	50	55	65	75	85	95
Prevalence	**85.1**	**61.8**	**35.0**	**21.6**	**15.6**	**8.2**	**5.1**	**2.1**	**2.1**	**0.7**	**0.7**	**0.7**	**0.7**	**0.5**	**0.5**	**0.0**
Lower CI	74.7	47.5	23.2	12.1	8.1	3.9	2.6	1.3	1.3	0.2	0.2	0.2	0.2	0.1	0.1	0.0
Upper CI	91.7	74.3	49.0	35.4	27.9	16.8	10.0	3.5	3.5	2.8	2.8	2.8	2.8	2.7	2.7	4.8
Number	64	49	28	16	12	8	5	3	3	2	2	2	2	1	1	0

Table B2K-49 Age Group = 51-60 Gender = MALE Occupational group = NON-MANUAL Total cases = 142

Threshold dB HL≥	0	5	10	15	20	25	30	35	40	45	50	55	65	75	85	95
Prevalence	**95.1**	**74.0**	**50.2**	**37.6**	**28.3**	**21.5**	**16.5**	**11.0**	**7.6**	**2.9**	**1.4**	**1.0**	**0.6**	**0.0**	**0.0**	**0.0**
Lower CI	90.5	65.3	41.6	30.3	21.8	16.3	12.9	5.5	3.1	1.6	0.6	0.4	0.2	0.0	0.0	0.0
Upper CI	97.5	81.1	58.8	45.6	35.7	27.7	21.0	20.7	17.4	5.1	3.0	2.4	2.2	2.6	2.6	2.6
Number	132	107	73	59	44	34	26	19	14	10	5	4	2	0	0	0

Prevalence by age group, gender and occupational group for the better ear over 2 kHz

Table B2K-50 Age Group = 61-70 Gender = MALE Occupational group = NON-MANUAL Total cases = 112

Threshold dB HL≥	0	5	10	15	20	25	30	35	40	45	50	55	65	75	85	95
**Prevalence**	**99.2**	**95.7**	**81.1**	**60.2**	**52.8**	**41.9**	**32.3**	**20.1**	**16.6**	**9.6**	**6.6**	**4.9**	**2.8**	**0.8**	**0.4**	**0.0**
Lower CI	95.9	90.5	71.8	46.2	39.7	30.3	21.3	14.8	11.5	6.2	3.6	2.6	1.1	0.2	0.1	0.0
Upper CI	99.9	98.1	87.9	72.6	65.5	54.6	45.7	26.8	23.2	14.5	11.9	8.9	7.2	2.3	2.1	3.3
Number	111	105	90	73	63	51	36	27	23	16	12	10	5	2	1	0

Table B2K-51 Age Group = 71-80 Gender = MALE Occupational group = NON-MANUAL Total cases = 56

Threshold dB HL≥	0	5	10	15	20	25	30	35	40	45	50	55	65	75	85	95
**Prevalence**	**100.0**	**100.0**	**91.1**	**85.3**	**69.7**	**60.2**	**51.5**	**38.2**	**34.4**	**21.6**	**12.0**	**8.9**	**3.1**	**1.0**	**0.0**	**0.0**
Lower CI	93.6	93.6	78.3	74.4	59.3	49.8	39.6	26.5	23.4	13.5	8.3	5.2	1.0	0.2	0.0	0.0
Upper CI	100.0	100.0	96.7	92.1	78.5	69.7	63.2	51.4	47.5	32.6	17.2	14.8	8.9	5.3	6.4	6.4
Number	56	56	52	50	42	36	30	24	22	14	9	6	3	1	0	0

Table B2K-52 Age Group = 18-30 Gender = FEMALE Occupational group = MANUAL Total cases = 109

Threshold dB HL≥	0	5	10	15	20	25	30	35	40	45	50	55	65	75	85	95
**Prevalence**	**78.8**	**51.5**	**23.0**	**8.4**	**2.9**	**2.4**	**1.1**	**0.8**	**0.8**	**0.5**	**0.0**	**0.0**	**0.0**	**0.0**	**0.0**	**0.0**
Lower CI	66.5	39.6	15.5	4.1	1.2	0.9	0.5	0.3	0.3	0.1	0.0	0.0	0.0	0.0	0.0	0.0
Upper CI	87.4	63.1	32.6	16.3	6.7	6.2	2.5	2.1	2.1	1.7	3.4	3.4	3.4	3.4	3.4	3.4
Number	92	62	31	12	8	7	5	4	4	2	0	0	0	0	0	0

Table B2K-53 Age Group = 31-40 Gender = FEMALE Occupational group = MANUAL Total cases = 103

Threshold dB HL≥	0	5	10	15	20	25	30	35	40	45	50	55	65	75	85	95
**Prevalence**	**82.0**	**54.5**	**21.6**	**12.1**	**10.1**	**7.4**	**3.6**	**2.4**	**2.4**	**2.4**	**0.8**	**0.8**	**0.3**	**0.0**	**0.0**	**0.0**
Lower CI	71.5	42.6	14.8	7.5	5.8	3.8	1.4	0.7	0.7	0.7	0.3	0.3	0.1	0.0	0.0	0.0
Upper CI	89.2	65.9	30.4	19.0	17.2	13.9	9.1	8.3	8.3	8.3	2.3	2.3	1.7	3.6	3.6	3.6
Number	89	64	36	22	16	12	7	4	4	4	3	3	1	0	0	0

Table B2K-54 Age Group = 41-50 Gender = FEMALE Occupational group = MANUAL Total cases = 135

Threshold dB HL≥	0	5	10	15	20	25	30	35	40	45	50	55	65	75	85	95
**Prevalence**	**91.5**	**70.6**	**45.9**	**26.3**	**16.8**	**14.2**	**8.1**	**4.4**	**2.7**	**1.8**	**1.8**	**1.6**	**1.1**	**0.2**	**0.2**	**0.2**
Lower CI	84.4	61.0	36.4	18.9	11.1	8.9	4.7	2.6	1.3	0.7	0.7	0.6	0.3	0.0	0.0	0.0
Upper CI	95.5	78.7	55.7	35.4	24.6	21.8	13.7	7.2	5.5	4.4	4.4	4.3	4.0	1.0	1.0	1.0
Number	124	101	67	43	31	26	18	13	9	6	6	5	3	1	1	1

Table B2K-55 Age Group = 51-60 Gender = FEMALE Occupational group = MANUAL Total cases = 166

Threshold dB HL≥	0	5	10	15	20	25	30	35	40	45	50	55	65	75	85	95
**Prevalence**	**97.2**	**76.7**	**54.1**	**34.1**	**24.4**	**16.7**	**12.3**	**7.7**	**5.5**	**2.6**	**1.5**	**1.5**	**0.2**	**0.2**	**0.2**	**0.2**
Lower CI	93.3	68.4	44.9	26.7	18.1	11.4	8.0	4.3	3.0	1.4	0.6	0.6	0.0	0.0	0.0	0.0
Upper CI	98.8	83.4	63.1	42.4	32.0	23.8	18.7	13.3	9.7	5.1	3.8	3.8	1.0	1.0	1.0	1.0
Number	161	135	108	77	58	42	31	22	19	12	7	7	1	1	1	1

Table B2K-56 Age Group = 61-70 Gender = FEMALE Occupational group = MANUAL Total cases = 126

Threshold dB HL≥	0	5	10	15	20	25	30	35	40	45	50	55	65	75	85	95
**Prevalence**	**99.5**	**90.0**	**73.8**	**56.1**	**43.1**	**30.8**	**19.6**	**12.9**	**8.3**	**5.9**	**5.7**	**4.4**	**3.3**	**2.1**	**1.9**	**1.6**
Lower CI	97.4	80.9	63.3	46.1	33.4	22.1	13.2	8.5	6.1	4.3	4.2	3.2	2.3	1.6	1.4	0.5
Upper CI	99.9	95.1	82.1	65.6	53.3	41.1	28.0	19.1	11.1	7.9	7.8	6.1	4.8	2.8	2.4	5.7
Number	125	116	101	84	71	54	39	30	23	17	16	12	7	3	2	1

Prevalence by age group, gender and occupational group for the better ear over 2 kHz

Table B2K-57 Age Group = 71-80 Gender = FEMALE Occupational group = MANUAL Total cases = 77

Threshold dB HL≥	0	5	10	15	20	25	30	35	40	45	50	55	65	75	85	95
**Prevalence**	**100.0**	**99.6**	**82.3**	**77.7**	**77.1**	**56.9**	**41.0**	**38.0**	**33.7**	**17.1**	**14.1**	**10.7**	**6.3**	**3.7**	**1.2**	**0.0**
Lower CI	95.2	97.6	62.8	59.5	59.0	43.0	30.7	27.8	23.7	12.7	10.0	7.4	3.8	1.9	0.4	0.0
Upper CI	100.0	99.9	92.7	89.2	88.7	69.8	52.1	49.4	45.4	22.7	19.7	15.2	10.3	7.1	3.2	4.8
Number	77	76	70	68	67	57	51	47	41	31	25	20	13	8	3	0

Table B2K-58 Age Group = 18-30 Gender = MALE Occupational group = MANUAL Total cases = 109

Threshold dB HL≥	0	5	10	15	20	25	30	35	40	45	50	55	65	75	85	95
**Prevalence**	**75.2**	**46.9**	**30.2**	**16.3**	**5.5**	**3.6**	**1.7**	**0.1**	**0.1**	**0.1**	**0.1**	**0.1**	**0.0**	**0.0**	**0.0**	**0.0**
Lower CI	65.2	34.5	20.3	9.9	2.2	1.1	0.3	0.0	0.0	0.0	0.0	0.0	0.0	0.0	0.0	0.0
Upper CI	83.1	59.6	42.2	25.6	13.0	10.8	8.6	0.4	0.4	0.4	0.4	0.4	3.4	3.4	3.4	3.4
Number	85	53	35	18	8	5	2	1	1	1	1	1	0	0	0	0

Table B2K-59 Age Group = 31-40 Gender = MALE Occupational group = MANUAL Total cases = 80

Threshold dB HL≥	0	5	10	15	20	25	30	35	40	45	50	55	65	75	85	95
**Prevalence**	**78.8**	**54.6**	**33.5**	**19.7**	**14.8**	**7.0**	**6.3**	**2.7**	**2.7**	**2.7**	**2.7**	**2.1**	**1.9**	**1.9**	**1.9**	**1.9**
Lower CI	64.9	40.1	21.3	10.2	6.7	2.6	2.1	0.7	0.7	0.7	0.7	0.4	0.3	0.3	0.3	0.3
Upper CI	88.2	68.4	48.4	34.6	29.4	17.9	17.3	9.8	9.8	9.8	9.8	9.9	10.2	10.2	10.2	10.2
Number	67	48	31	18	13	8	7	4	4	4	4	2	1	1	1	1

Table B2K-60 Age Group = 41-50 Gender = MALE Occupational group = MANUAL Total cases = 127

Threshold dB HL≥	0	5	10	15	20	25	30	35	40	45	50	55	65	75	85	95
**Prevalence**	**90.0**	**76.5**	**48.6**	**30.9**	**17.9**	**13.6**	**9.4**	**7.1**	**5.8**	**3.2**	**1.3**	**1.3**	**0.5**	**0.5**	**0.2**	**0.0**
Lower CI	82.0	66.5	37.9	22.1	11.6	8.0	5.7	4.0	2.9	1.7	0.5	0.5	0.1	0.1	0.0	0.0
Upper CI	94.7	84.1	59.5	41.3	26.5	22.1	15.1	12.4	11.0	6.0	3.0	3.0	1.7	1.7	1.3	2.9
Number	116	99	70	47	32	23	20	16	13	10	5	5	2	2	1	0

Table B2K-61 Age Group = 51-60 Gender = MALE Occupational group = MANUAL Total cases = 199

Threshold dB HL≥	0	5	10	15	20	25	30	35	40	45	50	55	65	75	85	95
**Prevalence**	**99.0**	**82.8**	**66.0**	**48.6**	**38.0**	**30.5**	**23.2**	**16.4**	**12.1**	**10.5**	**8.9**	**7.8**	**3.0**	**1.8**	**0.0**	**0.0**
Lower CI	95.5	75.1	57.6	41.2	31.4	24.3	17.9	11.9	8.5	7.2	5.9	4.9	1.3	0.5	0.0	0.0
Upper CI	99.8	88.5	73.5	56.1	45.1	37.6	29.4	22.1	17.0	15.2	13.3	12.0	7.1	6.5	1.9	1.9
Number	197	176	144	119	100	81	64	49	38	34	29	25	10	4	0	0

Table B2K-62 Age Group = 61-70 Gender = MALE Occupational group = MANUAL Total cases = 173

Threshold dB HL≥	0	5	10	15	20	25	30	35	40	45	50	55	65	75	85	95
**Prevalence**	**97.5**	**91.3**	**81.0**	**71.0**	**53.8**	**45.2**	**36.3**	**27.9**	**22.3**	**16.4**	**12.8**	**12.2**	**4.4**	**1.7**	**0.9**	**0.6**
Lower CI	91.9	82.2	71.4	60.6	43.2	36.7	28.1	21.0	16.0	11.2	8.3	7.8	2.8	0.7	0.3	0.1
Upper CI	99.3	95.9	88.0	79.7	64.2	54.0	45.3	36.1	30.1	23.3	19.3	18.8	7.0	3.9	2.4	2.2
Number	171	163	151	133	113	100	84	72	59	50	40	37	17	6	4	2

Table B2K-63 Age Group = 71-80 Gender = MALE Occupational group = MANUAL Total cases = 72

Threshold dB HL≥	0	5	10	15	20	25	30	35	40	45	50	55	65	75	85	95
**Prevalence**	**100.0**	**100.0**	**78.6**	**74.4**	**70.5**	**55.1**	**44.8**	**33.6**	**25.7**	**24.3**	**19.9**	**16.1**	**9.2**	**2.4**	**2.4**	**0.6**
Lower CI	94.9	94.9	64.0	59.9	56.2	39.9	34.1	25.8	20.1	18.7	14.0	10.3	5.2	0.9	0.9	0.1
Upper CI	100.0	100.0	88.3	85.0	81.6	69.3	55.9	42.3	32.2	31.0	27.4	24.2	15.8	5.9	5.9	3.1
Number	72	72	67	64	61	53	48	41	35	32	26	21	13	4	4	1

Prevalence by age group, gender and occupational group for the better ear over 3 kHz

Table B3K-1 Age Group = 18-80 Gender = OVERALL Occupational group = OVERALL Total cases = 2659

Threshold dB HL≥	0	5	10	15	20	25	30	35	40	45	50	55	65	75	85	95
Prevalence	**89.5**	**74.7**	**57.5**	**42.1**	**32.4**	**24.4**	**19.5**	**15.6**	**12.6**	**9.7**	**7.7**	**5.8**	**3.1**	**1.6**	**0.8**	**0.4**
Lower CI	87.8	72.6	55.4	40.3	30.8	22.9	18.2	14.5	11.6	8.9	7.0	5.1	2.6	1.2	0.5	0.2
Upper CI	91.0	76.7	59.6	43.9	34.0	26.0	20.9	16.8	13.7	10.6	8.5	6.4	3.7	2.0	1.2	0.6
Number	2477	2215	1858	1494	1232	997	846	713	606	508	425	340	195	99	50	25

Table B3K-2 Age Group = 18-80 Gender = FEMALE Occupational group = OVERALL Total cases = 1387

Threshold dB HL≥	0	5	10	15	20	25	30	35	40	45	50	55	65	75	85	95
Prevalence	**88.7**	**72.8**	**55.1**	**38.0**	**27.9**	**19.3**	**15.1**	**11.5**	**8.6**	**6.6**	**5.0**	**3.1**	**1.6**	**0.8**	**0.6**	**0.2**
Lower CI	86.4	70.0	52.3	35.6	25.8	17.4	13.4	10.0	7.5	5.7	4.2	2.6	1.3	0.6	0.4	0.1
Upper CI	90.6	75.5	57.8	40.4	30.1	21.3	16.9	13.1	10.0	7.6	5.9	3.8	2.1	1.2	0.9	0.4
Number	1272	1108	908	685	531	405	333	269	218	178	144	106	60	33	23	10

Table B3K-3 Age Group = 18-80 Gender = MALE Occupational group = OVERALL Total cases = 1272

Threshold dB HL≥	0	5	10	15	20	25	30	35	40	45	50	55	65	75	85	95
Prevalence	**90.5**	**76.9**	**60.3**	**46.8**	**37.5**	**30.2**	**24.4**	**20.2**	**17.0**	**13.2**	**10.8**	**8.7**	**4.8**	**2.4**	**1.0**	**0.5**
Lower CI	87.6	73.6	57.1	43.9	35.1	27.9	22.4	18.5	15.3	11.9	9.5	7.6	3.9	1.8	0.6	0.3
Upper CI	92.7	79.9	63.4	49.6	39.9	32.7	26.6	22.1	18.8	14.7	12.2	10.0	5.8	3.2	1.8	1.1
Number	1205	1107	950	809	701	592	513	444	388	330	281	234	135	66	27	15

Table B3K-4 Age Group = 18-30 Gender = OVERALL Occupational group = OVERALL Total cases = 401

Threshold dB HL≥	0	5	10	15	20	25	30	35	40	45	50	55	65	75	85	95
Prevalence	**78.8**	**49.6**	**25.7**	**12.0**	**6.3**	**3.6**	**2.5**	**1.4**	**1.2**	**0.5**	**0.4**	**0.3**	**0.3**	**0.0**	**0.0**	**0.0**
Lower CI	73.7	43.9	21.1	8.7	4.2	2.1	1.2	0.6	0.5	0.1	0.1	0.1	0.1	0.0	0.0	0.0
Upper CI	83.2	55.3	31.0	16.2	9.4	6.4	5.0	3.0	2.9	1.6	1.5	1.5	1.5	0.1	0.9	0.9
Number	321	218	120	60	35	19	13	10	8	5	4	3	3	1	0	0

Table B3K-5 Age Group = 31-40 Gender = OVERALL Occupational group = OVERALL Total cases = 331

Threshold dB HL≥	0	5	10	15	20	25	30	35	40	45	50	55	65	75	85	95
Prevalence	**85.8**	**65.7**	**37.5**	**21.7**	**11.9**	**7.4**	**3.8**	**3.3**	**2.6**	**1.8**	**1.8**	**1.6**	**0.7**	**0.6**	**0.6**	**0.6**
Lower CI	80.4	59.3	31.6	17.0	8.4	4.6	2.2	1.8	1.3	0.8	0.8	0.7	0.2	0.1	0.1	0.1
Upper CI	89.8	71.6	43.9	27.3	16.5	11.7	6.6	5.9	5.3	3.9	3.9	3.7	3.0	3.0	3.0	3.0
Number	291	233	145	93	53	30	21	19	13	10	10	9	3	2	2	2

Table B3K-6 Age Group = 41-50 Gender = OVERALL Occupational group = OVERALL Total cases = 450

Threshold dB HL≥	0	5	10	15	20	25	30	35	40	45	50	55	65	75	85	95
Prevalence	**90.9**	**78.3**	**62.7**	**37.1**	**23.4**	**14.6**	**10.4**	**7.5**	**5.3**	**4.3**	**3.1**	**2.6**	**1.5**	**0.6**	**0.2**	**0.1**
Lower CI	87.5	73.8	57.6	32.5	19.7	11.8	8.2	5.6	3.8	3.0	2.1	1.7	0.8	0.2	0.1	0.0
Upper CI	93.4	82.2	67.5	42.0	27.5	17.9	13.3	10.0	7.2	6.2	4.5	3.8	2.6	1.3	0.7	0.5
Number	415	367	300	200	141	95	73	55	43	36	28	25	14	6	3	2

Table B3K-7 Age Group = 51-60 Gender = OVERALL Occupational group = OVERALL Total cases = 680

Threshold dB HL≥	0	5	10	15	20	25	30	35	40	45	50	55	65	75	85	95
Prevalence	**95.3**	**88.3**	**75.3**	**56.0**	**43.5**	**31.9**	**24.9**	**18.8**	**14.4**	**11.3**	**8.9**	**6.8**	**3.8**	**1.6**	**0.9**	**0.3**
Lower CI	93.1	85.3	71.6	52.0	39.7	28.5	21.9	16.3	12.2	9.4	7.3	5.4	2.7	1.0	0.5	0.1
Upper CI	96.9	90.8	78.7	60.0	47.5	35.5	28.2	21.6	16.9	13.5	10.9	8.6	5.3	2.6	1.6	0.7
Number	656	613	538	433	357	286	232	186	147	119	99	78	47	20	12	6

Prevalence by age group, gender and occupational group for the better ear over 3 kHz

Table B3K-8 Age Group = 61-70 Gender = OVERALL Occupational group = OVERALL Total cases = 525

	Threshold dB HL≥														
	**0**	**5**	**10**	**15**	**20**	**25**	**30**	**35**	**40**	**45**	**50**	**55**	**65**	**75**	**85**	**95**
**Prevalence**	**99.0**	**97.0**	**90.5**	**82.7**	**70.6**	**53.6**	**42.1**	**34.3**	**27.7**	**21.7**	**17.9**	**13.5**	**7.0**	**3.8**	**1.5**	**0.8**
**Lower CI**	96.6	94.3	86.5	78.2	65.6	48.3	37.3	30.1	23.9	18.4	14.9	11.5	5.7	2.7	0.9	0.4
**Upper CI**	99.7	98.5	93.4	86.4	75.2	58.8	47.1	38.9	32.0	25.4	21.4	15.9	8.7	5.2	2.4	1.6
**Number**	522	513	488	453	402	334	286	246	214	181	153	127	72	37	17	8

Table B3K-9 Age Group = 71-80 Gender = OVERALL Occupational group = OVERALL Total cases = 272

	Threshold dB HL≥														
	**0**	**5**	**10**	**15**	**20**	**25**	**30**	**35**	**40**	**45**	**50**	**55**	**65**	**75**	**85**	**95**
**Prevalence**	**100.0**	**99.1**	**95.3**	**87.6**	**82.0**	**74.0**	**68.1**	**58.7**	**50.0**	**40.1**	**31.0**	**20.6**	**11.0**	**6.2**	**3.3**	**0.9**
**Lower CI**	98.6	95.1	88.6	80.2	73.4	65.1	59.5	50.8	43.0	34.6	25.8	16.7	8.2	4.1	1.6	0.5
**Upper CI**	100.0	99.8	98.2	92.5	88.3	81.3	75.6	66.1	57.0	45.9	36.6	25.0	14.6	9.4	6.5	1.9
**Number**	272	271	267	255	244	233	221	197	181	157	131	98	56	33	16	7

Table B3K-10 Age Group = 18-80 Gender = OVERALL Occupational group = NON-MANUAL Total cases = 1182

	Threshold dB HL≥														
	**0**	**5**	**10**	**15**	**20**	**25**	**30**	**35**	**40**	**45**	**50**	**55**	**65**	**75**	**85**	**95**
**Prevalence**	**87.6**	**69.9**	**52.3**	**36.4**	**28.2**	**21.1**	**17.3**	**14.1**	**10.8**	**8.0**	**6.2**	**4.5**	**2.3**	**1.2**	**0.6**	**0.2**
**Lower CI**	84.8	66.7	49.3	34.1	26.3	19.4	15.9	12.7	9.7	7.1	5.3	3.8	1.8	0.8	0.3	0.1
**Upper CI**	90.0	72.9	55.2	38.7	30.2	22.8	18.8	15.6	11.9	8.9	7.2	5.4	2.8	1.7	1.0	0.4
**Number**	1080	923	749	578	469	367	305	257	208	171	139	109	53	28	18	6

Table B3K-11 Age Group = 18-80 Gender = OVERALL Occupational group = MANUAL Total cases = 1473

	Threshold dB HL≥														
	**0**	**5**	**10**	**15**	**20**	**25**	**30**	**35**	**40**	**45**	**50**	**55**	**65**	**75**	**85**	**95**
**Prevalence**	**90.5**	**79.3**	**62.3**	**46.9**	**35.7**	**27.1**	**21.0**	**16.6**	**13.9**	**10.8**	**8.6**	**6.6**	**3.7**	**1.8**	**0.9**	**0.5**
**Lower CI**	88.0	76.2	59.1	43.9	33.3	24.8	19.1	14.9	12.4	9.7	7.5	5.7	3.1	1.4	0.6	0.3
**Upper CI**	92.6	82.1	65.4	49.9	38.3	29.4	23.1	18.4	15.5	12.1	9.7	7.6	4.5	2.4	1.3	0.9
**Number**	1393	1289	1107	914	761	628	539	455	397	337	286	231	142	71	32	19

Table B3K-12 Age Group = 18-30 Gender = FEMALE Occupational group = OVERALL Total cases = 224

	Threshold dB HL≥														
	**0**	**5**	**10**	**15**	**20**	**25**	**30**	**35**	**40**	**45**	**50**	**55**	**65**	**75**	**85**	**95**
**Prevalence**	**79.8**	**51.2**	**24.9**	**11.2**	**5.1**	**1.9**	**1.0**	**0.8**	**0.4**	**0.3**	**0.1**	**0.0**	**0.0**	**0.0**	**0.0**	**0.0**
**Lower CI**	73.5	43.8	19.2	7.6	3.1	0.9	0.4	0.3	0.1	0.1	0.0	0.0	0.0	0.0	0.0	0.0
**Upper CI**	85.0	58.5	31.6	16.1	8.5	3.8	2.1	1.8	1.2	1.1	0.6	1.7	1.7	1.7	1.7	1.7
**Number**	180	122	65	35	19	9	6	5	3	2	1	0	0	0	0	0

Table B3K-13 Age Group = 31-40 Gender = FEMALE Occupational group = OVERALL Total cases = 189

	Threshold dB HL≥														
	**0**	**5**	**10**	**15**	**20**	**25**	**30**	**35**	**40**	**45**	**50**	**55**	**65**	**75**	**85**	**95**
**Prevalence**	**83.2**	**60.2**	**35.2**	**18.5**	**9.9**	**3.6**	**3.2**	**2.5**	**1.6**	**1.5**	**1.5**	**1.2**	**0.2**	**0.0**	**0.0**	**0.0**
**Lower CI**	75.9	51.8	27.8	13.4	6.4	1.9	1.6	1.2	0.6	0.5	0.5	0.4	0.0	0.0	0.0	0.0
**Upper CI**	88.6	68.0	43.3	24.9	14.8	6.7	6.3	5.1	4.1	4.1	4.1	3.8	1.2	2.0	2.0	2.0
**Number**	162	124	79	50	28	13	11	10	6	5	5	4	1	0	0	0

Table B3K-14 Age Group = 41-50 Gender = FEMALE Occupational group = OVERALL Total cases = 247

	Threshold dB HL≥														
	**0**	**5**	**10**	**15**	**20**	**25**	**30**	**35**	**40**	**45**	**50**	**55**	**65**	**75**	**85**	**95**
**Prevalence**	**88.0**	**72.2**	**58.6**	**33.4**	**18.0**	**10.3**	**6.2**	**3.3**	**2.3**	**2.0**	**1.2**	**1.2**	**1.0**	**0.1**	**0.1**	**0.1**
**Lower CI**	82.6	65.7	51.7	27.5	13.7	7.3	4.0	2.0	1.3	1.0	0.5	0.5	0.4	0.0	0.0	0.0
**Upper CI**	91.9	78.0	65.1	39.9	23.2	14.3	9.3	5.6	4.0	3.8	2.7	2.7	2.5	0.6	0.6	0.6
**Number**	222	189	154	97	60	38	26	16	12	10	6	6	5	1	1	1

Prevalence by age group, gender and occupational group for the better ear over 3 kHz

Table B3K-15 Age Group = 51-60 Gender = FEMALE Occupational group = OVERALL Total cases = 340

	Threshold dB HL≥															
	0	5	10	15	20	25	30	35	40	45	50	55	65	75	85	95
Prevalence	**93.4**	**84.0**	**69.0**	**44.6**	**32.1**	**19.9**	**14.4**	**8.2**	**5.7**	**4.3**	**3.2**	**2.5**	**1.5**	**0.9**	**0.7**	**0.2**
Lower CI	89.7	79.1	63.3	39.0	27.1	15.9	11.1	6.0	4.0	2.9	2.1	1.5	0.8	0.4	0.2	0.1
Upper CI	95.8	87.9	74.1	50.2	37.5	24.5	18.4	11.1	8.0	6.3	4.8	3.9	2.7	1.9	1.7	0.6
Number	323	295	254	182	139	101	77	52	39	31	26	20	13	8	5	3

Table B3K-16 Age Group = 61-70 Gender = FEMALE Occupational group = OVERALL Total cases = 243

	Threshold dB HL≥															
	0	5	10	15	20	25	30	35	40	45	50	55	65	75	85	95
Prevalence	**98.3**	**95.6**	**85.8**	**74.5**	**60.3**	**42.2**	**30.3**	**23.6**	**16.3**	**11.6**	**9.5**	**5.6**	**3.2**	**1.5**	**1.2**	**0.4**
Lower CI	93.9	90.8	79.4	67.5	53.0	35.0	24.2	18.5	12.1	8.1	6.4	4.4	2.1	0.8	0.6	0.1
Upper CI	99.5	97.9	90.4	80.5	67.1	49.7	37.2	29.8	21.7	16.3	13.9	7.0	4.8	2.9	2.6	1.8
Number	241	235	216	191	160	124	99	83	65	50	42	34	19	9	7	2

Table B3K-17 Age Group = 71-80 Gender = FEMALE Occupational group = OVERALL Total cases = 144

	Threshold dB HL≥															
	0	5	10	15	20	25	30	35	40	45	50	55	65	75	85	95
Prevalence	**100.0**	**98.5**	**93.5**	**83.0**	**77.5**	**69.1**	**63.6**	**54.0**	**45.4**	**35.4**	**26.1**	**14.1**	**6.7**	**4.1**	**2.6**	**0.9**
Lower CI	97.4	91.8	83.0	71.8	64.2	56.0	51.1	42.9	36.2	28.6	20.0	10.3	4.4	2.5	1.4	0.4
Upper CI	100.0	99.7	97.7	90.4	86.8	79.7	74.6	64.6	54.9	42.8	33.3	19.0	10.1	6.7	4.7	2.3
Number	144	143	140	130	125	120	114	103	93	80	64	42	22	15	10	4

Table B3K-18 Age Group = 18-30 Gender = MALE Occupational group = OVERALL Total cases = 177

	Threshold dB HL≥															
	0	5	10	15	20	25	30	35	40	45	50	55	65	75	85	95
Prevalence	**77.5**	**48.0**	**26.6**	**12.7**	**7.4**	**5.4**	**4.0**	**2.0**	**2.0**	**0.6**	**0.6**	**0.6**	**0.6**	**0.0**	**0.0**	**0.0**
Lower CI	69.1	39.4	19.6	7.8	4.1	2.6	1.7	0.7	0.7	0.1	0.1	0.1	0.1	0.0	0.0	0.0
Upper CI	84.2	56.7	35.1	20.2	13.0	10.8	9.2	5.5	5.5	3.0	3.0	3.0	3.0	0.2	2.1	2.1
Number	141	96	55	25	16	10	7	5	5	3	3	3	3	1	0	0

Table B3K-19 Age Group = 31-40 Gender = MALE Occupational group = OVERALL Total cases = 142

	Threshold dB HL≥															
	0	5	10	15	20	25	30	35	40	45	50	55	65	75	85	95
Prevalence	**88.4**	**71.6**	**40.0**	**25.3**	**14.1**	**11.7**	**4.5**	**4.1**	**3.7**	**2.0**	**2.0**	**2.0**	**1.3**	**1.3**	**1.3**	**1.3**
Lower CI	79.5	61.3	30.7	17.6	8.4	6.4	2.0	1.7	1.4	0.6	0.6	0.6	0.3	0.3	0.3	0.3
Upper CI	93.8	79.9	50.0	35.1	22.6	20.4	9.7	9.4	9.1	6.4	6.4	6.4	6.4	6.4	6.4	6.4
Number	129	109	66	43	25	17	10	9	7	5	5	5	2	2	2	2

Table B3K-20 Age Group = 41-50 Gender = MALE Occupational group = OVERALL Total cases = 203

	Threshold dB HL≥															
	0	5	10	15	20	25	30	35	40	45	50	55	65	75	85	95
Prevalence	**94.0**	**85.0**	**67.2**	**41.2**	**29.4**	**19.2**	**15.2**	**12.1**	**8.6**	**6.9**	**5.2**	**4.1**	**2.1**	**1.1**	**0.4**	**0.2**
Lower CI	89.3	78.6	59.5	34.2	23.5	14.6	11.2	8.6	5.9	4.5	3.3	2.6	1.0	0.4	0.1	0.0
Upper CI	96.7	89.7	74.1	48.6	36.0	24.9	20.2	16.8	12.4	10.5	8.0	6.4	4.1	2.7	1.4	1.0
Number	193	178	146	103	81	57	47	39	31	26	22	19	9	5	2	1

Table B3K-21 Age Group = 51-60 Gender = MALE Occupational group = OVERALL Total cases = 340

	Threshold dB HL≥															
	0	5	10	15	20	25	30	35	40	45	50	55	65	75	85	95
Prevalence	**97.6**	**93.2**	**82.4**	**68.8**	**56.3**	**45.4**	**36.7**	**30.6**	**24.2**	**19.1**	**15.3**	**11.7**	**6.4**	**2.3**	**1.1**	**0.4**
Lower CI	94.8	89.6	77.5	63.0	50.4	39.8	31.6	26.0	20.0	15.5	12.0	8.9	4.3	1.2	0.5	0.1
Upper CI	98.9	95.7	86.3	74.1	62.0	51.1	42.1	35.7	28.9	23.4	19.2	15.3	9.4	4.3	2.3	1.3
Number	333	318	284	251	218	185	155	134	108	88	73	58	34	12	7	3

Prevalence by age group, gender and occupational group for the better ear over 3 kHz

Table B3K-22 Age Group = 61-70 Gender = MALE Occupational group = OVERALL Total cases = 282

	Threshold dB HL≥															
	**0**	**5**	**10**	**15**	**20**	**25**	**30**	**35**	**40**	**45**	**50**	**55**	**65**	**75**	**85**	**95**
**Prevalence**	**99.9**	**98.8**	**95.8**	**92.0**	**82.5**	**66.8**	**55.8**	**46.7**	**41.1**	**33.5**	**27.7**	**22.9**	**11.6**	**6.5**	**1.7**	**1.2**
**Lower CI**	99.3	96.1	90.3	86.3	75.3	58.9	48.3	40.0	34.6	28.0	22.6	18.8	9.0	4.5	0.9	0.5
**Upper CI**	100.0	99.6	98.3	95.5	87.9	73.8	63.1	53.5	47.8	39.6	33.5	27.7	14.8	9.2	3.3	2.7
**Number**	281	278	272	262	242	210	187	163	149	131	111	93	53	28	10	6

Table B3K-23 Age Group = 71-80 Gender = MALE Occupational group = OVERALL Total cases = 128

	Threshold dB HL≥															
	**0**	**5**	**10**	**15**	**20**	**25**	**30**	**35**	**40**	**45**	**50**	**55**	**65**	**75**	**85**	**95**
**Prevalence**	**100.0**	**100.0**	**98.1**	**94.8**	**89.0**	**80.9**	**74.3**	**65.2**	**56.2**	**46.3**	**37.8**	**30.2**	**17.6**	**9.5**	**4.3**	**1.0**
**Lower CI**	97.1	97.1	89.7	86.4	79.8	70.2	63.8	54.9	46.0	37.9	30.0	23.1	11.9	5.2	1.4	0.3
**Upper CI**	100.0	100.0	99.7	98.1	94.3	88.4	82.6	74.2	65.9	54.9	46.4	38.4	25.2	16.8	12.6	2.8
**Number**	128	128	127	125	119	113	107	94	88	77	67	56	34	18	6	3

Table B3K-24 Age Group = 18-30 Gender = OVERALL Occupational group = NON-MANUAL Total cases = 181

	Threshold dB HL≥															
	**0**	**5**	**10**	**15**	**20**	**25**	**30**	**35**	**40**	**45**	**50**	**55**	**65**	**75**	**85**	**95**
**Prevalence**	**74.8**	**42.5**	**18.5**	**4.2**	**2.2**	**1.0**	**0.5**	**0.5**	**0.1**	**0.1**	**0.1**	**0.1**	**0.1**	**0.0**	**0.0**	**0.0**
**Lower CI**	66.7	34.5	12.9	2.5	1.1	0.3	0.1	0.1	0.0	0.0	0.0	0.0	0.0	0.0	0.0	0.0
**Upper CI**	81.5	50.9	25.9	7.1	4.4	3.0	2.4	2.4	2.2	2.2	2.2	2.2	2.2	2.1	2.1	2.1
**Number**	139	83	41	14	8	4	2	2	1	1	1	1	1	0	0	0

Table B3K-25 Age Group = 31-40 Gender = OVERALL Occupational group = NON-MANUAL Total cases = 148

	Threshold dB HL≥															
	**0**	**5**	**10**	**15**	**20**	**25**	**30**	**35**	**40**	**45**	**50**	**55**	**65**	**75**	**85**	**95**
**Prevalence**	**85.3**	**58.1**	**32.0**	**17.9**	**7.8**	**4.5**	**1.5**	**1.5**	**1.0**	**0.3**	**0.3**	**0.0**	**0.0**	**0.0**	**0.0**	**0.0**
**Lower CI**	76.6	48.5	23.7	11.5	4.3	2.0	0.6	0.6	0.3	0.1	0.1	0.0	0.0	0.0	0.0	0.0
**Upper CI**	91.1	67.2	41.5	26.6	13.7	10.0	3.6	3.6	3.6	1.7	1.7	2.5	2.5	2.5	2.5	2.5
**Number**	129	92	52	31	16	8	4	4	3	1	1	0	0	0	0	0

Table B3K-26 Age Group = 41-50 Gender = OVERALL Occupational group = NON-MANUAL Total cases = 188

	Threshold dB HL≥															
	**0**	**5**	**10**	**15**	**20**	**25**	**30**	**35**	**40**	**45**	**50**	**55**	**65**	**75**	**85**	**95**
**Prevalence**	**87.9**	**70.3**	**53.8**	**27.3**	**17.7**	**9.4**	**7.6**	**4.5**	**1.5**	**1.5**	**1.3**	**1.3**	**1.2**	**0.4**	**0.2**	**0.2**
**Lower CI**	81.9	62.8	45.9	21.0	12.9	6.3	4.9	2.9	0.9	0.9	0.8	0.8	0.7	0.1	0.0	0.0
**Upper CI**	92.1	76.8	61.5	34.6	23.8	13.9	11.5	7.0	2.4	2.4	2.2	2.2	2.1	1.3	1.3	1.3
**Number**	165	134	104	61	42	23	19	12	6	6	5	5	4	2	1	1

Table B3K-27 Age Group = 51-60 Gender = OVERALL Occupational group = NON-MANUAL Total cases = 317

	Threshold dB HL≥															
	**0**	**5**	**10**	**15**	**20**	**25**	**30**	**35**	**40**	**45**	**50**	**55**	**65**	**75**	**85**	**95**
**Prevalence**	**94.6**	**86.2**	**71.3**	**48.8**	**36.9**	**26.7**	**20.6**	**16.2**	**11.1**	**8.7**	**5.6**	**4.8**	**1.8**	**0.7**	**0.7**	**0.2**
**Lower CI**	90.9	81.6	65.9	43.3	31.9	22.2	16.7	12.9	8.9	7.0	3.0	2.4	1.0	0.3	0.3	0.0
**Upper CI**	96.9	89.9	76.1	54.4	42.2	31.6	25.1	20.1	13.8	10.8	10.0	9.3	3.0	1.8	1.8	0.7
**Number**	302	273	226	169	135	103	79	63	46	36	28	24	13	5	5	2

Table B3K-28 Age Group = 61-70 Gender = OVERALL Occupational group = NON-MANUAL Total cases = 226

	Threshold dB HL≥															
	**0**	**5**	**10**	**15**	**20**	**25**	**30**	**35**	**40**	**45**	**50**	**55**	**65**	**75**	**85**	**95**
**Prevalence**	**98.2**	**96.9**	**88.4**	**79.9**	**65.9**	**49.0**	**39.0**	**32.6**	**26.5**	**20.8**	**17.3**	**11.4**	**4.6**	**3.6**	**1.0**	**0.3**
**Lower CI**	94.3	92.8	80.1	71.3	57.1	42.3	32.9	27.5	22.1	17.6	14.8	9.3	3.0	2.2	0.4	0.1
**Upper CI**	99.5	98.7	93.5	86.4	73.8	55.7	45.5	38.2	31.3	24.5	20.0	14.0	7.0	6.0	2.2	1.2
**Number**	223	219	207	188	160	129	107	93	77	66	55	44	17	11	6	2

Prevalence by age group, gender and occupational group for the better ear over 3 kHz

Table B3K-29 Age Group = 71-80 Gender = OVERALL Occupational group = NON-MANUAL Total cases = 122

Threshold dB HL≥	0	5	10	15	20	25	30	35	40	45	50	55	65	75	85	95
**Prevalence**	**100.0**	**100.0**	**95.8**	**91.4**	**86.8**	**78.1**	**73.0**	**61.1**	**51.4**	**36.2**	**28.8**	**19.8**	**12.0**	**5.5**	**2.9**	**0.5**
**Lower CI**	96.9	96.9	87.2	83.1	77.9	68.7	64.2	49.9	43.2	28.8	22.1	15.1	8.7	3.1	1.1	0.1
**Upper CI**	100.0	100.0	98.7	95.8	92.4	85.2	80.2	71.3	59.6	44.2	36.5	25.4	16.4	9.6	7.6	2.7
**Number**	122	122	119	115	108	100	94	83	75	61	49	35	18	10	6	1

Table B3K-30 Age Group = 18-30 Gender = OVERALL Occupational group = MANUAL Total cases = 218

Threshold dB HL≥	0	5	10	15	20	25	30	35	40	45	50	55	65	75	85	95
**Prevalence**	**80.5**	**56.9**	**32.5**	**19.3**	**9.3**	**5.4**	**3.8**	**2.2**	**2.1**	**0.7**	**0.6**	**0.5**	**0.5**	**0.0**	**0.0**	**0.0**
**Lower CI**	72.6	47.8	24.7	13.2	5.8	2.9	1.8	0.9	0.8	0.2	0.1	0.1	0.1	0.0	0.0	0.0
**Upper CI**	86.6	65.6	41.3	27.4	14.6	9.8	7.9	5.5	5.4	2.6	2.6	2.6	2.6	0.2	1.7	1.7
**Number**	180	134	79	46	27	15	11	8	7	4	3	2	2	1	0	0

Table B3K-31 Age Group = 31-40 Gender = OVERALL Occupational group = MANUAL Total cases = 183

Threshold dB HL≥	0	5	10	15	20	25	30	35	40	45	50	55	65	75	85	95
**Prevalence**	**85.9**	**73.2**	**42.4**	**25.6**	**16.0**	**10.9**	**6.3**	**5.1**	**4.3**	**2.7**	**2.7**	**2.7**	**1.2**	**1.0**	**1.0**	**1.0**
**Lower CI**	78.2	65.0	33.5	18.5	10.3	6.1	3.2	2.4	1.7	1.1	1.1	1.1	0.3	0.2	0.2	0.2
**Upper CI**	91.2	80.2	51.8	34.4	24.1	18.8	12.1	10.7	10.1	6.6	6.6	6.6	4.8	4.9	4.9	4.9
**Number**	162	141	93	62	37	22	17	15	10	9	9	9	3	2	2	2

Table B3K-32 Age Group = 41-50 Gender = OVERALL Occupational group = MANUAL Total cases = 262

Threshold dB HL≥	0	5	10	15	20	25	30	35	40	45	50	55	65	75	85	95
**Prevalence**	**93.1**	**84.7**	**70.4**	**45.1**	**28.3**	**19.3**	**12.7**	**9.7**	**7.2**	**5.9**	**3.9**	**3.2**	**1.6**	**0.5**	**0.2**	**0.1**
**Lower CI**	88.1	78.6	63.2	38.1	22.5	14.7	9.3	6.8	5.0	3.9	2.5	2.0	0.8	0.2	0.1	0.0
**Upper CI**	96.1	89.3	76.7	52.4	34.8	24.8	17.1	13.7	10.3	8.9	5.9	5.0	3.3	1.4	0.7	0.5
**Number**	250	233	196	139	99	72	54	43	37	30	23	20	10	4	2	1

Table B3K-33 Age Group = 51-60 Gender = OVERALL Occupational group = MANUAL Total cases = 363

Threshold dB HL≥	0	5	10	15	20	25	30	35	40	45	50	55	65	75	85	95
**Prevalence**	**95.9**	**91.3**	**81.5**	**63.3**	**49.5**	**36.8**	**28.6**	**21.1**	**17.1**	**13.8**	**10.9**	**8.0**	**4.6**	**1.9**	**0.8**	**0.4**
**Lower CI**	92.4	87.1	76.1	57.1	43.7	31.6	24.2	17.5	13.9	11.0	8.6	5.9	3.0	1.1	0.4	0.2
**Upper CI**	97.9	94.2	85.8	69.0	55.3	42.3	33.4	25.2	20.8	17.2	13.9	10.7	6.9	3.3	1.8	1.2
**Number**	354	340	312	264	222	183	153	123	101	83	71	54	34	15	7	4

Table B3K-34 Age Group = 61-70 Gender = OVERALL Occupational group = MANUAL Total cases = 298

Threshold dB HL≥	0	5	10	15	20	25	30	35	40	45	50	55	65	75	85	95
**Prevalence**	**100.0**	**97.3**	**91.1**	**84.2**	**73.9**	**56.5**	**43.3**	**34.8**	**28.2**	**21.8**	**18.9**	**15.3**	**8.9**	**4.6**	**2.1**	**0.8**
**Lower CI**	98.7	92.7	85.8	78.2	67.4	49.3	36.4	28.8	23.3	17.8	15.0	11.9	7.0	3.2	1.4	0.4
**Upper CI**	100.0	99.0	94.6	88.8	79.6	63.4	50.5	41.3	33.8	26.6	23.6	19.4	11.2	6.3	3.0	1.8
**Number**	298	293	280	264	241	204	178	153	137	115	98	83	55	26	11	6

Table B3K-35 Age Group = 71-80 Gender = OVERALL Occupational group = MANUAL Total cases = 149

Threshold dB HL≥	0	5	10	15	20	25	30	35	40	45	50	55	65	75	85	95
**Prevalence**	**100.0**	**98.0**	**95.4**	**83.7**	**78.7**	**70.4**	**65.3**	**56.5**	**50.9**	**43.6**	**30.9**	**20.9**	**11.7**	**6.6**	**2.7**	**1.4**
**Lower CI**	97.5	89.5	84.4	70.9	65.5	58.1	54.4	46.1	41.8	36.2	23.9	16.8	8.5	4.3	1.4	0.6
**Upper CI**	100.0	99.7	98.8	91.5	87.8	80.3	74.8	66.4	60.0	51.3	38.8	25.7	16.0	9.9	5.0	2.8
**Number**	149	148	147	139	135	132	126	113	105	96	82	63	38	23	10	6

Prevalence by age group, gender and occupational group for the better ear over 3 kHz

Table B3K-36 Age Group = 18-80 Gender = FEMALE Occupational group = NON-MANUAL Total cases = 671

Threshold dB HL≥	0	5	10	15	20	25	30	35	40	45	50	55	65	75	85	95
**Prevalence**	**88.6**	**70.1**	**52.2**	**34.8**	**27.2**	**19.2**	**15.8**	**12.2**	**9.5**	**6.4**	**5.2**	**3.3**	**1.9**	**0.7**	**0.5**	**0.1**
Lower CI	85.5	66.2	48.6	32.0	24.7	17.1	14.0	10.3	8.2	5.3	4.2	2.7	1.5	0.4	0.3	0.1
Upper CI	91.1	73.7	55.7	37.7	29.7	21.4	17.8	14.5	10.9	7.6	6.3	4.1	2.5	1.1	1.0	0.4
Number	609	512	410	301	238	179	148	124	101	82	68	49	25	14	12	4

Table B3K-37 Age Group = 18-80 Gender = MALE Occupational group = NON-MANUAL Total cases = 511

Threshold dB HL≥	0	5	10	15	20	25	30	35	40	45	50	55	65	75	85	95
**Prevalence**	**86.3**	**69.5**	**52.4**	**38.3**	**29.3**	**23.2**	**18.9**	**16.0**	**12.1**	**9.7**	**7.3**	**5.8**	**2.7**	**1.7**	**0.6**	**0.2**
Lower CI	81.2	64.2	47.5	34.6	26.3	20.7	16.7	14.1	10.5	8.4	5.9	4.5	2.0	1.1	0.2	0.0
Upper CI	90.2	74.4	57.2	42.0	32.5	25.9	21.2	18.2	14.0	11.1	9.0	7.5	3.7	2.7	1.5	0.6
Number	471	411	339	277	231	188	157	133	107	89	71	60	28	14	6	2

Table B3K-38 Age Group = 18-80 Gender = FEMALE Occupational group = MANUAL Total cases = 715

Threshold dB HL≥	0	5	10	15	20	25	30	35	40	45	50	55	65	75	85	95
**Prevalence**	**88.5**	**76.1**	**59.1**	**41.4**	**28.8**	**19.9**	**14.8**	**11.0**	**8.0**	**6.6**	**4.5**	**3.0**	**1.8**	**1.1**	**0.7**	**0.2**
Lower CI	84.8	71.5	54.6	37.4	25.5	17.2	12.6	9.2	6.7	5.4	3.5	2.3	1.3	0.7	0.5	0.1
Upper CI	91.4	80.1	63.5	45.6	32.4	22.9	17.3	13.1	9.6	8.0	5.8	3.9	2.4	1.6	1.0	0.5
Number	662	595	497	383	292	225	184	145	117	96	76	57	35	19	11	6

Table B3K-39 Age Group = 18-80 Gender = MALE Occupational group = MANUAL Total cases = 758

Threshold dB HL≥	0	5	10	15	20	25	30	35	40	45	50	55	65	75	85	95
**Prevalence**	**92.9**	**83.0**	**65.9**	**53.0**	**43.5**	**35.0**	**28.1**	**22.9**	**20.4**	**15.5**	**13.1**	**10.5**	**5.8**	**2.7**	**1.1**	**0.8**
Lower CI	88.9	78.5	61.3	48.5	39.8	31.4	25.0	20.1	17.7	13.6	11.3	8.9	4.6	1.9	0.6	0.3
Upper CI	95.5	86.8	70.3	57.5	47.2	38.8	31.4	26.0	23.4	17.7	15.1	12.5	7.3	3.7	2.1	1.7
Number	731	694	610	531	469	403	355	310	280	241	210	174	107	52	21	13

Table B3K-40 Age Group = 18-30 Gender = FEMALE Occupational group = NON-MANUAL Total cases = 115

Threshold dB HL≥	0	5	10	15	20	25	30	35	40	45	50	55	65	75	85	95
**Prevalence**	**79.9**	**48.3**	**21.2**	**7.3**	**3.5**	**1.8**	**0.8**	**0.8**	**0.0**	**0.0**	**0.0**	**0.0**	**0.0**	**0.0**	**0.0**	**0.0**
Lower CI	71.2	38.9	14.8	4.1	1.6	0.6	0.1	0.1	0.0	0.0	0.0	0.0	0.0	0.0	0.0	0.0
Upper CI	86.5	57.8	29.5	12.5	7.4	5.5	4.5	4.5	3.2	3.2	3.2	3.2	3.2	3.2	3.2	3.2
Number	91	56	28	12	6	3	1	1	0	0	0	0	0	0	0	0

Table B3K-41 Age Group = 31-40 Gender = FEMALE Occupational group = NON-MANUAL Total cases = 86

Threshold dB HL≥	0	5	10	15	20	25	30	35	40	45	50	55	65	75	85	95
**Prevalence**	**83.9**	**51.5**	**27.1**	**13.1**	**7.0**	**2.1**	**1.5**	**1.5**	**0.7**	**0.6**	**0.6**	**0.0**	**0.0**	**0.0**	**0.0**	**0.0**
Lower CI	74.2	40.2	18.2	7.6	3.4	1.1	0.8	0.8	0.2	0.1	0.1	0.0	0.0	0.0	0.0	0.0
Upper CI	90.5	62.8	38.3	21.4	14.0	4.2	3.1	3.1	3.0	3.2	3.2	4.3	4.3	4.3	4.3	4.3
Number	73	48	26	16	9	4	3	3	2	1	1	0	0	0	0	0

Table B3K-42 Age Group = 41-50 Gender = FEMALE Occupational group = NON-MANUAL Total cases = 112

Threshold dB HL≥	0	5	10	15	20	25	30	35	40	45	50	55	65	75	85	95
**Prevalence**	**88.4**	**68.4**	**54.8**	**26.9**	**12.7**	**5.3**	**4.1**	**0.9**	**0.7**	**0.7**	**0.4**	**0.4**	**0.4**	**0.0**	**0.0**	**0.0**
Lower CI	80.9	58.5	45.1	19.6	8.8	3.8	2.8	0.3	0.2	0.2	0.1	0.1	0.1	0.0	0.0	0.0
Upper CI	93.2	76.9	64.2	35.7	18.1	7.3	6.1	2.6	2.5	2.5	2.2	2.2	2.2	3.3	3.3	3.3
Number	98	79	62	35	20	10	8	3	2	2	1	1	1	0	0	0

Prevalence by age group, gender and occupational group for the better ear over 3 kHz

Table B3K-50 Age Group = 61-70 Gender = MALE Occupational group = NON-MANUAL Total cases = 110

Threshold dB HL≥	0	5	10	15	20	25	30	35	40	45	50	55	65	75	85	95
**Prevalence**	**99.6**	**97.3**	**88.4**	**83.6**	**70.6**	**54.6**	**46.6**	**38.9**	**33.0**	**27.0**	**21.6**	**17.8**	**7.1**	**6.4**	**0.8**	**0.0**
**Lower CI**	97.8	91.9	71.3	67.4	55.3	45.5	37.7	31.1	26.2	22.1	18.2	13.9	4.3	3.7	0.2	0.0
**Upper CI**	99.9	99.2	95.9	92.6	82.3	63.4	55.7	47.4	40.6	32.6	25.5	22.5	11.5	10.8	2.8	3.4
**Number**	109	106	101	94	82	69	60	50	44	40	32	26	9	7	2	0

Table B3K-51 Age Group = 71-80 Gender = MALE Occupational group = NON-MANUAL Total cases = 55

Threshold dB HL≥	0	5	10	15	20	25	30	35	40	45	50	55	65	75	85	95
**Prevalence**	**100.0**	**100.0**	**97.1**	**97.1**	**86.6**	**78.5**	**69.9**	**60.9**	**49.5**	**40.9**	**33.1**	**22.5**	**13.8**	**9.2**	**4.4**	**1.3**
**Lower CI**	93.5	93.5	84.9	84.9	72.5	65.4	57.4	48.3	37.1	29.2	22.8	14.8	7.8	4.3	1.1	0.2
**Upper CI**	100.0	100.0	99.5	99.5	94.0	87.6	80.0	72.2	62.1	53.7	45.2	32.8	23.3	18.5	16.1	7.0
**Number**	55	55	54	54	49	45	41	35	31	24	20	15	8	4	2	1

Table B3K-52 Age Group = 18-30 Gender = FEMALE Occupational group = MANUAL Total cases = 109

Threshold dB HL≥	0	5	10	15	20	25	30	35	40	45	50	55	65	75	85	95
**Prevalence**	**78.9**	**55.9**	**31.4**	**17.1**	**7.7**	**2.2**	**1.1**	**0.8**	**0.6**	**0.5**	**0.2**	**0.0**	**0.0**	**0.0**	**0.0**	**0.0**
**Lower CI**	67.8	43.2	21.1	9.9	3.7	0.8	0.5	0.3	0.2	0.1	0.0	0.0	0.0	0.0	0.0	0.0
**Upper CI**	86.9	67.9	44.0	28.0	15.1	6.0	2.5	2.2	1.7	1.7	1.1	3.4	3.4	3.4	3.4	3.4
**Number**	89	66	37	23	13	6	5	4	3	2	1	0	0	0	0	0

Table B3K-53 Age Group = 31-40 Gender = FEMALE Occupational group = MANUAL Total cases = 103

Threshold dB HL≥	0	5	10	15	20	25	30	35	40	45	50	55	65	75	85	95
**Prevalence**	**82.4**	**69.0**	**43.3**	**23.6**	**12.4**	**5.3**	**5.2**	**3.5**	**2.4**	**2.4**	**2.4**	**2.4**	**0.3**	**0.0**	**0.0**	**0.0**
**Lower CI**	70.8	57.1	32.0	15.7	7.2	2.3	2.2	1.3	0.7	0.7	0.7	0.7	0.1	0.0	0.0	0.0
**Upper CI**	90.0	78.9	55.2	34.0	20.4	11.9	11.8	9.1	8.3	8.3	8.3	8.3	1.7	3.6	3.6	3.6
**Number**	89	76	53	34	19	9	8	7	4	4	4	4	1	0	0	0

Table B3K-54 Age Group = 41-50 Gender = FEMALE Occupational group = MANUAL Total cases = 135

Threshold dB HL≥	0	5	10	15	20	25	30	35	40	45	50	55	65	75	85	95
**Prevalence**	**88.2**	**76.9**	**63.8**	**40.0**	**23.6**	**15.6**	**8.0**	**4.9**	**3.1**	**2.6**	**1.6**	**1.6**	**1.3**	**0.2**	**0.2**	**0.2**
**Lower CI**	79.7	67.7	53.9	31.0	16.5	10.1	4.7	2.6	1.6	1.2	0.6	0.6	0.4	0.0	0.0	0.0
**Upper CI**	93.5	84.1	72.6	49.8	32.5	23.4	13.4	8.9	6.1	5.5	4.3	4.3	4.1	1.0	1.0	1.0
**Number**	124	110	92	62	40	28	18	13	10	8	5	5	4	1	1	1

Table B3K-55 Age Group = 51-60 Gender = FEMALE Occupational group = MANUAL Total cases = 165

Threshold dB HL≥	0	5	10	15	20	25	30	35	40	45	50	55	65	75	85	95
**Prevalence**	**93.4**	**86.1**	**74.1**	**50.7**	**34.6**	**21.7**	**15.1**	**7.8**	**5.4**	**3.9**	**2.1**	**1.8**	**1.0**	**0.6**	**0.2**	**0.2**
**Lower CI**	87.4	78.8	65.3	41.6	26.8	15.6	10.3	4.8	3.0	2.0	1.2	0.9	0.5	0.3	0.0	0.0
**Upper CI**	96.7	91.1	81.3	59.7	43.3	29.3	21.5	12.5	9.6	7.8	3.6	3.4	1.8	1.4	1.0	1.0
**Number**	157	146	131	99	73	54	41	26	19	15	12	10	7	4	1	1

Table B3K-56 Age Group = 61-70 Gender = FEMALE Occupational group = MANUAL Total cases = 126

Threshold dB HL≥	0	5	10	15	20	25	30	35	40	45	50	55	65	75	85	95
**Prevalence**	**100.0**	**95.0**	**83.8**	**73.1**	**58.3**	**40.8**	**28.1**	**20.6**	**12.6**	**8.7**	**7.0**	**5.7**	**4.5**	**2.5**	**2.1**	**0.0**
**Lower CI**	97.0	87.0	74.4	62.8	48.1	31.1	19.8	14.0	8.8	6.4	5.0	4.1	3.1	1.9	1.6	0.0
**Upper CI**	100.0	98.2	90.2	81.4	67.9	51.2	38.2	29.3	17.8	11.8	9.8	7.9	6.5	3.4	2.8	3.0
**Number**	126	121	109	96	81	63	51	40	32	24	19	16	11	5	3	0

Prevalence by age group, gender and occupational group for the better ear over 3 kHz

Table B3K-43 Age Group = 51-60 Gender = FEMALE Occupational group = NON-MANUAL Total cases = 175

	Threshold dB HL≥															
	0	5	10	15	20	25	30	35	40	45	50	55	65	75	85	95
**Prevalence**	**93.2**	**83.1**	**66.8**	**39.4**	**30.8**	**19.2**	**14.2**	**8.8**	**6.0**	**4.9**	**4.2**	**2.9**	**1.7**	**1.0**	**1.0**	**0.3**
**Lower CI**	87.1	75.8	58.8	32.3	24.1	13.9	9.7	5.7	3.9	3.1	2.5	1.6	0.7	0.4	0.4	0.1
**Upper CI**	96.5	88.6	74.0	47.0	38.3	25.9	20.3	13.4	9.0	7.7	7.0	5.2	3.7	2.9	2.9	1.2
**Number**	166	149	123	83	66	47	36	26	20	16	14	10	6	4	4	2

Table B3K-44 Age Group = 61-70 Gender = FEMALE Occupational group = NON-MANUAL Total cases = 116

	Threshold dB HL≥															
	0	5	10	15	20	25	30	35	40	45	50	55	65	75	85	95
**Prevalence**	**97.2**	**96.8**	**88.2**	**76.4**	**61.9**	**44.0**	**32.2**	**27.0**	**20.6**	**15.1**	**12.8**	**5.7**	**2.3**	**1.1**	**1.1**	**0.6**
**Lower CI**	90.1	90.0	78.8	65.8	51.1	34.7	24.2	20.5	15.2	10.9	9.6	4.0	1.2	0.4	0.4	0.1
**Upper CI**	99.2	99.0	93.7	84.5	71.8	53.8	41.4	34.7	27.3	20.5	16.9	8.0	4.2	3.0	3.0	2.3
**Number**	114	113	106	94	78	60	47	43	33	26	23	18	8	4	4	2

Table B3K-45 Age Group = 71-80 Gender = FEMALE Occupational group = NON-MANUAL Total cases = 67

	Threshold dB HL≥															
	0	5	10	15	20	25	30	35	40	45	50	55	65	75	85	95
**Prevalence**	**100.0**	**100.0**	**95.0**	**87.9**	**87.2**	**77.5**	**74.4**	**61.0**	**52.5**	**32.7**	**25.7**	**18.0**	**11.1**	**3.2**	**2.0**	**0.0**
**Lower CI**	94.6	94.6	81.6	75.7	75.0	64.2	62.3	45.0	42.3	24.1	17.8	13.0	8.1	1.6	0.7	0.0
**Upper CI**	100.0	100.0	98.8	94.5	93.9	86.8	83.7	75.0	62.5	42.7	35.5	24.5	15.1	6.3	5.1	5.4
**Number**	67	67	65	61	59	55	53	48	44	37	29	20	10	6	4	0

Table B3K-46 Age Group = 18-30 Gender = MALE Occupational group = NON-MANUAL Total cases = 66

	Threshold dB HL≥															
	0	5	10	15	20	25	30	35	40	45	50	55	65	75	85	95
**Prevalence**	**69.0**	**36.4**	**15.7**	**0.9**	**0.9**	**0.1**	**0.1**	**0.1**	**0.1**	**0.1**	**0.1**	**0.1**	**0.1**	**0.0**	**0.0**	**0.0**
**Lower CI**	54.9	24.3	7.8	0.2	0.2	0.0	0.0	0.0	0.0	0.0	0.0	0.0	0.0	0.0	0.0	0.0
**Upper CI**	80.3	50.5	29.1	3.9	3.9	5.7	5.7	5.7	5.7	5.7	5.7	5.7	5.7	5.5	5.5	5.5
**Number**	48	27	13	2	2	1	1	1	1	1	1	1	1	0	0	0

Table B3K-47 Age Group = 31-40 Gender = MALE Occupational group = NON-MANUAL Total cases = 62

	Threshold dB HL≥															
	0	5	10	15	20	25	30	35	40	45	50	55	65	75	85	95
**Prevalence**	**86.2**	**64.9**	**37.2**	**23.7**	**8.6**	**7.1**	**1.3**	**1.3**	**1.3**	**0.0**	**0.0**	**0.0**	**0.0**	**0.0**	**0.0**	**0.0**
**Lower CI**	69.3	48.4	23.5	12.5	3.4	2.4	0.2	0.2	0.2	0.0	0.0	0.0	0.0	0.0	0.0	0.0
**Upper CI**	94.6	78.5	53.3	40.3	20.0	19.3	6.9	6.9	6.9	5.8	5.8	5.8	5.8	5.8	5.8	5.8
**Number**	56	44	26	15	7	4	1	1	1	0	0	0	0	0	0	0

Table B3K-48 Age Group = 41-50 Gender = MALE Occupational group = NON-MANUAL Total cases = 76

	Threshold dB HL≥															
	0	5	10	15	20	25	30	35	40	45	50	55	65	75	85	95
**Prevalence**	**87.4**	**72.3**	**52.7**	**27.8**	**23.1**	**13.9**	**11.3**	**8.5**	**2.4**	**2.4**	**2.4**	**2.4**	**2.1**	**0.7**	**0.5**	**0.5**
**Lower CI**	76.7	60.5	40.3	18.0	14.4	7.9	6.3	5.2	1.5	1.5	1.5	1.5	1.3	0.2	0.1	0.1
**Upper CI**	93.6	81.6	64.9	40.1	35.0	23.2	19.5	13.5	3.7	3.7	3.7	3.7	3.5	2.8	2.7	2.7
**Number**	67	55	42	26	22	13	11	9	4	4	4	4	3	2	1	1

Table B3K-49 Age Group = 51-60 Gender = MALE Occupational group = NON-MANUAL Total cases = 142

	Threshold dB HL≥															
	0	5	10	15	20	25	30	35	40	45	50	55	65	75	85	95
**Prevalence**	**96.3**	**89.8**	**76.0**	**59.4**	**43.7**	**35.0**	**27.7**	**24.4**	**16.9**	**13.0**	**7.0**	**7.0**	**1.9**	**0.3**	**0.3**	**0.0**
**Lower CI**	91.4	83.7	68.9	50.8	36.4	28.2	21.5	18.7	13.0	10.1	2.7	2.7	0.9	0.1	0.1	0.0
**Upper CI**	98.5	93.7	82.0	67.4	51.3	42.5	34.8	31.3	21.7	16.5	16.8	16.8	3.8	1.9	1.9	2.6
**Number**	136	124	103	86	69	56	43	37	26	20	14	14	7	1	1	0

Prevalence by age group, gender and occupational group for the better ear over 3 kHz

Table B3K-57 Age Group = 71-80 Gender = FEMALE Occupational group = MANUAL Total cases = 77

Threshold dB HL≥	0	5	10	15	20	25	30	35	40	45	50	55	65	75	85	95
Prevalence	**100.0**	**96.6**	**92.3**	**78.1**	**69.9**	**65.6**	**59.4**	**52.8**	**44.5**	**40.0**	**24.8**	**11.6**	**6.6**	**5.1**	**2.9**	**1.6**
Lower CI	95.2	82.6	74.8	59.9	51.2	47.6	44.1	39.4	33.9	29.4	15.4	8.0	3.7	2.6	1.3	0.7
Upper CI	100.0	99.4	98.0	89.5	83.7	80.0	73.1	65.8	55.7	51.5	37.3	16.7	11.6	9.9	6.4	3.7
Number	77	76	75	69	66	65	61	55	49	43	35	22	12	9	6	4

Table B3K-58 Age Group = 18-30 Gender = MALE Occupational group = MANUAL Total cases = 109

Threshold dB HL≥	0	5	10	15	20	25	30	35	40	45	50	55	65	75	85	95
Prevalence	**82.1**	**57.9**	**33.7**	**21.7**	**10.9**	**8.5**	**6.6**	**3.6**	**3.6**	**1.0**	**1.0**	**1.0**	**1.0**	**0.1**	**0.0**	**0.0**
Lower CI	69.7	45.1	23.0	12.7	5.9	4.1	2.8	1.2	1.2	0.2	0.2	0.2	0.2	0.0	0.0	0.0
Upper CI	90.1	69.8	46.4	34.5	19.3	16.9	14.8	10.4	10.4	5.1	5.1	5.1	5.1	0.4	3.4	3.4
Number	91	68	42	23	14	9	6	4	4	2	2	2	2	1	0	0

Table B3K-59 Age Group = 31-40 Gender = MALE Occupational group = MANUAL Total cases = 80

Threshold dB HL≥	0	5	10	15	20	25	30	35	40	45	50	55	65	75	85	95
Prevalence	**89.9**	**78.0**	**41.5**	**27.6**	**19.8**	**16.9**	**7.4**	**6.7**	**6.1**	**3.1**	**3.1**	**3.1**	**2.1**	**2.1**	**2.1**	**2.1**
Lower CI	78.2	65.9	28.1	16.5	10.3	8.1	2.8	2.4	2.0	0.9	0.9	0.9	0.4	0.4	0.4	0.4
Upper CI	95.7	86.7	56.3	42.4	34.6	32.1	18.1	17.5	17.3	9.8	9.8	9.8	9.9	9.9	9.9	9.9
Number	73	65	40	28	18	13	9	8	6	5	5	5	2	2	2	2

Table B3K-60 Age Group = 41-50 Gender = MALE Occupational group = MANUAL Total cases = 127

Threshold dB HL≥	0	5	10	15	20	25	30	35	40	45	50	55	65	75	85	95
Prevalence	**98.4**	**93.2**	**77.4**	**50.6**	**33.3**	**23.1**	**17.9**	**14.9**	**11.6**	**9.5**	**6.4**	**5.0**	**2.0**	**0.9**	**0.2**	**0.0**
Lower CI	91.2	83.5	66.5	39.9	24.7	16.5	12.2	9.7	7.5	5.8	4.0	3.1	0.8	0.3	0.0	0.0
Upper CI	99.7	97.4	85.5	61.2	43.3	31.4	25.4	22.3	17.5	15.2	10.1	8.0	4.7	2.7	1.3	2.9
Number	126	123	104	77	59	44	36	30	27	22	18	15	6	3	1	0

Table B3K-61 Age Group = 51-60 Gender = MALE Occupational group = MANUAL Total cases = 198

Threshold dB HL≥	0	5	10	15	20	25	30	35	40	45	50	55	65	75	85	95
Prevalence	**98.9**	**97.3**	**89.8**	**77.2**	**66.1**	**53.7**	**43.7**	**35.9**	**30.1**	**24.6**	**20.7**	**15.0**	**8.6**	**3.4**	**1.5**	**0.7**
Lower CI	93.7	92.5	83.5	68.9	57.5	45.4	36.3	29.4	24.2	19.4	15.8	10.7	5.4	1.8	0.7	0.2
Upper CI	99.8	99.1	93.9	83.9	73.8	61.8	51.4	43.0	36.7	30.7	26.6	20.5	13.3	6.3	3.5	2.3
Number	197	194	181	165	149	129	112	97	82	68	59	44	27	11	6	3

Table B3K-62 Age Group = 61-70 Gender = MALE Occupational group = MANUAL Total cases = 172

Threshold dB HL≥	0	5	10	15	20	25	30	35	40	45	50	55	65	75	85	95
Prevalence	**100.0**	**100.0**	**99.7**	**97.0**	**91.9**	**74.9**	**61.3**	**51.4**	**46.6**	**37.6**	**33.2**	**26.8**	**14.4**	**7.1**	**2.1**	**1.7**
Lower CI	97.8	97.8	98.3	91.4	83.3	64.2	50.2	41.4	36.8	29.0	24.8	19.8	10.7	4.5	1.0	0.8
Upper CI	100.0	100.0	99.9	99.0	96.2	83.2	71.3	61.3	56.8	47.1	42.8	35.2	19.1	11.3	4.2	3.9
Number	172	172	171	168	160	141	127	113	105	91	79	67	44	21	8	6

Table B3K-63 Age Group = 71-80 Gender = MALE Occupational group = MANUAL Total cases = 72

Threshold dB HL≥	0	5	10	15	20	25	30	35	40	45	50	55	65	75	85	95
Prevalence	**100.0**	**100.0**	**100.0**	**91.7**	**91.3**	**77.7**	**74.4**	**62.0**	**60.3**	**48.8**	**39.8**	**34.5**	**19.3**	**8.7**	**2.4**	**1.0**
Lower CI	94.9	94.9	94.9	71.3	71.3	63.3	59.7	45.8	44.1	40.6	32.1	26.2	13.1	5.2	0.9	0.3
Upper CI	100.0	100.0	100.0	98.0	97.8	87.6	85.0	75.9	74.5	57.2	48.0	44.0	27.4	14.4	5.9	3.5
Number	72	72	72	70	69	67	65	58	56	53	47	41	26	14	4	2

Prevalence by age group, gender and occupational group for the better ear over 4 kHz

Table B4K-1 Age Group = 18-80 Gender = OVERALL Occupational group = OVERALL Total cases = 2664

	Threshold dB HL≥															
	0	5	10	15	20	25	30	35	40	45	50	55	65	75	85	95
Prevalence	92.6	83.0	65.3	51.9	40.4	32.7	26.0	21.8	18.1	14.9	12.1	9.8	5.0	2.7	1.4	0.7
Lower CI	91.0	81.0	63.3	50.0	38.7	31.1	24.6	20.4	16.9	13.8	11.2	8.9	4.5	2.2	1.0	0.5
Upper CI	93.9	84.8	67.4	53.8	42.1	34.4	27.5	23.1	19.4	16.0	13.2	10.7	5.7	3.2	1.8	1.0
Number	2545	2372	2045	1745	1466	1249	1059	930	816	704	599	505	310	173	84	47

Table B4K-2 Age Group = 18-80 Gender = FEMALE Occupational group = OVERALL Total cases = 1387

	Threshold dB HL≥															
	0	5	10	15	20	25	30	35	40	45	50	55	65	75	85	95
Prevalence	92.0	82.1	60.7	45.2	33.6	26.5	19.4	15.7	12.8	9.7	7.6	6.0	2.8	1.5	0.7	0.4
Lower CI	89.8	79.5	58.0	42.9	31.6	24.5	17.7	14.1	11.3	8.5	6.5	5.0	2.3	1.2	0.5	0.2
Upper CI	93.7	84.5	63.3	47.5	35.8	28.7	21.4	17.6	14.5	11.2	8.8	7.2	3.3	1.9	1.0	0.6
Number	1311	1209	984	793	630	515	411	343	289	236	194	160	100	57	26	15

Table B4K-3 Age Group = 18-80 Gender = MALE Occupational group = OVERALL Total cases = 1277

	Threshold dB HL≥															
	0	5	10	15	20	25	30	35	40	45	50	55	65	75	85	95
Prevalence	93.3	83.9	70.6	59.5	48.0	39.7	33.4	28.5	24.0	20.7	17.3	14.0	7.6	4.1	2.2	1.1
Lower CI	90.6	80.8	67.3	56.4	45.3	37.2	31.1	26.4	22.2	19.0	15.7	12.6	6.5	3.2	1.6	0.7
Upper CI	95.3	86.7	73.7	62.5	50.8	42.2	35.7	30.7	26.0	22.4	19.0	15.5	8.9	5.1	3.1	1.7
Number	1234	1163	1061	952	836	734	648	587	527	468	405	345	210	116	58	32

Table B4K-4 Age Group = 18-30 Gender = OVERALL Occupational group = OVERALL Total cases = 401

	Threshold dB HL≥															
	0	5	10	15	20	25	30	35	40	45	50	55	65	75	85	95
Prevalence	83.3	61.3	32.7	16.2	7.6	5.2	3.5	1.8	0.7	0.5	0.4	0.4	0.3	0.3	0.3	0.0
Lower CI	78.3	55.5	27.7	12.5	5.2	3.3	2.0	0.8	0.3	0.1	0.1	0.1	0.1	0.1	0.1	0.0
Upper CI	87.3	66.8	38.1	20.7	11.0	8.0	6.1	3.7	1.7	1.6	1.5	1.5	1.5	1.6	1.6	0.9
Number	341	260	153	80	45	30	20	13	7	5	4	4	3	2	1	0

Table B4K-5 Age Group = 31-40 Gender = OVERALL Occupational group = OVERALL Total cases = 331

	Threshold dB HL≥															
	0	5	10	15	20	25	30	35	40	45	50	55	65	75	85	95
Prevalence	88.7	77.2	50.7	34.5	20.5	12.0	7.5	5.2	3.5	2.6	1.6	1.5	1.2	0.8	0.6	0.6
Lower CI	83.6	71.1	44.3	28.7	15.8	8.5	4.9	3.2	1.9	1.3	0.7	0.6	0.5	0.2	0.1	0.1
Upper CI	92.4	82.3	57.1	40.7	26.1	16.8	11.4	8.4	6.2	4.9	3.5	3.4	3.2	2.9	3.0	3.0
Number	300	263	186	129	80	51	35	26	18	14	11	10	8	5	2	2

Table B4K-6 Age Group = 41-50 Gender = OVERALL Occupational group = OVERALL Total cases = 450

	Threshold dB HL≥															
	0	5	10	15	20	25	30	35	40	45	50	55	65	75	85	95
Prevalence	95.6	88.3	69.1	50.3	35.7	24.5	17.9	13.5	10.7	7.8	5.6	3.8	2.2	1.6	0.7	0.2
Lower CI	93.0	84.6	64.3	45.4	31.2	20.7	14.6	10.7	8.3	5.8	3.9	2.5	1.2	0.8	0.3	0.1
Upper CI	97.3	91.2	73.6	55.3	40.4	28.8	21.7	17.0	13.9	10.5	7.9	5.8	3.9	3.2	1.5	0.6
Number	433	401	327	249	188	140	107	84	70	54	42	30	18	13	8	3

Table B4K-7 Age Group = 51-60 Gender = OVERALL Occupational group = OVERALL Total cases = 682

	Threshold dB HL≥															
	0	5	10	15	20	25	30	35	40	45	50	55	65	75	85	95
Prevalence	98.5	95.6	85.4	73.0	56.7	45.0	32.8	27.0	22.1	17.5	13.6	10.5	5.0	3.0	1.5	1.2
Lower CI	97.0	93.5	82.0	69.1	52.8	41.2	29.7	24.0	19.3	15.1	11.5	8.8	3.8	2.1	0.9	0.7
Upper CI	99.2	97.0	88.2	76.5	60.5	48.8	36.2	30.2	25.1	20.2	15.9	12.5	6.4	4.2	2.5	2.2
Number	672	653	601	539	448	374	302	256	214	176	142	120	65	39	20	14

Prevalence by age group, gender and occupational group for the better ear over 4 kHz

Table B4K-8 Age Group = 61-70 Gender = OVERALL Occupational group = OVERALL Total cases = 528

	Threshold dB HL≥															
	**0**	**5**	**10**	**15**	**20**	**25**	**30**	**35**	**40**	**45**	**50**	**55**	**65**	**75**	**85**	**95**
**Prevalence**	**99.6**	**98.8**	**95.1**	**89.2**	**79.7**	**70.9**	**58.0**	**50.0**	**40.8**	**34.5**	**29.0**	**22.2**	**12.2**	**5.9**	**3.2**	**1.7**
**Lower CI**	98.0	97.1	92.2	85.5	75.1	65.9	53.0	45.2	36.2	30.3	25.1	18.8	10.2	4.6	2.2	1.0
**Upper CI**	99.9	99.5	97.0	92.1	83.6	75.4	62.9	54.8	45.5	39.1	33.2	25.9	14.5	7.5	4.6	2.7
**Number**	527	523	508	484	447	404	354	319	288	252	219	183	118	62	31	18

Table B4K-9 Age Group = 71-80 Gender = OVERALL Occupational group = OVERALL Total cases = 272

	Threshold dB HL≥															
	**0**	**5**	**10**	**15**	**20**	**25**	**30**	**35**	**40**	**45**	**50**	**55**	**65**	**75**	**85**	**95**
**Prevalence**	**100.0**	**100.0**	**98.2**	**95.1**	**90.4**	**84.3**	**78.8**	**72.9**	**66.3**	**58.0**	**50.0**	**42.9**	**20.2**	**9.7**	**4.4**	**1.4**
**Lower CI**	98.6	98.6	92.2	89.4	83.4	75.8	70.5	63.9	57.7	50.9	43.0	36.1	16.4	7.0	2.5	0.8
**Upper CI**	100.0	100.0	99.6	97.9	94.6	90.2	85.2	80.3	74.0	64.7	57.1	49.9	24.6	13.4	7.6	2.6
**Number**	272	272	270	264	258	250	241	232	219	203	181	158	98	52	22	10

Table B4K-10 Age Group = 18-80 Gender = OVERALL Occupational group = NON-MANUAL Total cases = 1185

	Threshold dB HL≥															
	**0**	**5**	**10**	**15**	**20**	**25**	**30**	**35**	**40**	**45**	**50**	**55**	**65**	**75**	**85**	**95**
**Prevalence**	**92.2**	**80.0**	**60.6**	**46.9**	**36.0**	**27.8**	**21.8**	**18.5**	**15.4**	**12.6**	**10.4**	**8.2**	**3.3**	**1.5**	**1.0**	**0.5**
**Lower CI**	89.7	76.9	57.7	44.2	33.8	25.9	19.9	17.0	14.1	11.4	9.3	7.3	2.8	1.1	0.7	0.3
**Upper CI**	94.2	82.7	63.5	49.5	38.3	29.8	23.7	20.2	16.8	14.0	11.7	9.2	4.0	2.0	1.5	0.7
**Number**	1124	1021	839	689	568	464	378	334	289	250	204	169	97	45	26	16

Table B4K-11 Age Group = 18-80 Gender = OVERALL Occupational group = MANUAL Total cases = 1475

	Threshold dB HL≥															
	**0**	**5**	**10**	**15**	**20**	**25**	**30**	**35**	**40**	**45**	**50**	**55**	**65**	**75**	**85**	**95**
**Prevalence**	**92.5**	**85.9**	**70.1**	**56.9**	**44.6**	**37.2**	**29.6**	**24.2**	**20.0**	**16.4**	**13.4**	**10.9**	**6.0**	**3.4**	**1.7**	**0.9**
**Lower CI**	89.9	82.9	66.8	53.8	42.0	34.9	27.5	22.3	18.3	15.0	12.1	9.7	5.1	2.8	1.2	0.6
**Upper CI**	94.5	88.5	73.2	59.9	47.1	39.6	31.7	26.3	21.9	17.9	14.8	12.3	7.0	4.2	2.3	1.4
**Number**	1417	1348	1203	1054	896	783	679	594	526	453	394	335	213	128	58	31

Table B4K-12 Age Group = 18-30 Gender = FEMALE Occupational group = OVERALL Total cases = 224

	Threshold dB HL≥															
	**0**	**5**	**10**	**15**	**20**	**25**	**30**	**35**	**40**	**45**	**50**	**55**	**65**	**75**	**85**	**95**
**Prevalence**	**82.3**	**61.1**	**28.3**	**11.3**	**5.3**	**3.4**	**1.4**	**1.1**	**0.5**	**0.3**	**0.1**	**0.1**	**0.0**	**0.0**	**0.0**	**0.0**
**Lower CI**	75.8	53.6	22.3	7.8	3.4	2.0	0.8	0.5	0.2	0.1	0.0	0.0	0.0	0.0	0.0	0.0
**Upper CI**	87.4	68.2	35.2	16.2	8.3	5.8	2.5	2.1	1.4	1.1	0.6	0.6	1.7	1.7	1.7	1.7
**Number**	187	142	77	38	23	16	9	7	3	2	1	1	0	0	0	0

Table B4K-13 Age Group = 31-40 Gender = FEMALE Occupational group = OVERALL Total cases = 189

	Threshold dB HL≥															
	**0**	**5**	**10**	**15**	**20**	**25**	**30**	**35**	**40**	**45**	**50**	**55**	**65**	**75**	**85**	**95**
**Prevalence**	**86.8**	**74.8**	**41.6**	**21.6**	**11.5**	**8.1**	**5.1**	**3.1**	**2.3**	**2.0**	**0.6**	**0.6**	**0.6**	**0.3**	**0.0**	**0.0**
**Lower CI**	79.9	66.6	34.0	16.0	7.6	4.9	2.9	1.5	1.0	0.8	0.2	0.2	0.2	0.1	0.0	0.0
**Upper CI**	91.6	81.5	49.6	28.4	17.0	13.2	8.6	6.2	5.4	5.1	1.7	1.7	1.7	1.2	2.0	2.0
**Number**	168	148	94	55	32	22	17	11	7	6	4	4	4	2	0	0

Table B4K-14 Age Group = 41-50 Gender = FEMALE Occupational group = OVERALL Total cases = 247

	Threshold dB HL≥															
	**0**	**5**	**10**	**15**	**20**	**25**	**30**	**35**	**40**	**45**	**50**	**55**	**65**	**75**	**85**	**95**
**Prevalence**	**94.2**	**85.3**	**61.8**	**42.5**	**28.0**	**16.1**	**9.9**	**6.6**	**5.2**	**3.5**	**2.1**	**1.3**	**1.2**	**0.6**	**0.2**	**0.1**
**Lower CI**	89.9	79.6	54.9	36.0	22.5	12.1	7.0	4.4	3.2	2.0	1.1	0.6	0.5	0.2	0.1	0.0
**Upper CI**	96.7	89.6	68.2	49.2	34.3	21.1	13.8	9.8	8.2	6.3	3.8	2.8	2.7	2.1	0.9	0.6
**Number**	235	214	162	116	81	55	38	28	22	15	11	7	6	3	2	1

Prevalence by age group, gender and occupational group for the better ear over 4 kHz

Table B4K-15 Age Group = 51-60 Gender = FEMALE Occupational group = OVERALL Total cases = 341

	Threshold dB HL≥															
	0	5	10	15	20	25	30	35	40	45	50	55	65	75	85	95
Prevalence	**98.5**	**94.4**	**79.8**	**62.3**	**42.5**	**30.1**	**17.3**	**11.8**	**9.1**	**6.3**	**4.4**	**3.9**	**2.2**	**1.8**	**0.7**	**0.7**
Lower CI	96.2	91.0	74.5	56.3	37.1	25.4	14.0	9.1	6.8	4.6	3.1	2.6	1.4	1.0	0.3	0.3
Upper CI	99.4	96.6	84.3	67.9	48.1	35.3	21.2	15.2	12.2	8.6	6.3	5.7	3.4	3.0	1.8	1.8
Number	336	323	284	239	183	137	97	73	57	43	33	30	19	14	6	6

Table B4K-16 Age Group = 61-70 Gender = FEMALE Occupational group = OVERALL Total cases = 243

	Threshold dB HL≥															
	0	5	10	15	20	25	30	35	40	45	50	55	65	75	85	95
Prevalence	**99.3**	**98.0**	**91.4**	**82.5**	**68.9**	**60.4**	**44.7**	**34.7**	**26.4**	**21.0**	**16.0**	**10.6**	**5.6**	**2.5**	**1.3**	**1.0**
Lower CI	96.1	94.7	86.0	75.9	61.7	53.2	37.9	28.8	20.9	16.1	11.9	7.4	4.3	1.6	0.6	0.4
Upper CI	99.9	99.2	94.8	87.5	75.2	67.1	51.7	41.2	32.8	26.9	21.1	14.9	7.3	4.2	2.7	2.3
Number	242	239	226	208	180	159	128	106	91	74	60	48	33	16	7	5

Table B4K-17 Age Group = 71-80 Gender = FEMALE Occupational group = OVERALL Total cases = 143

	Threshold dB HL≥															
	0	5	10	15	20	25	30	35	40	45	50	55	65	75	85	95
Prevalence	**100.0**	**100.0**	**97.0**	**93.6**	**85.9**	**78.0**	**71.8**	**68.5**	**60.1**	**46.9**	**40.9**	**34.4**	**12.1**	**6.0**	**2.9**	**0.7**
Lower CI	97.4	97.4	87.6	84.2	75.0	64.7	59.0	55.4	48.0	37.8	31.8	25.5	8.9	4.0	1.6	0.2
Upper CI	100.0	100.0	99.3	97.6	92.5	87.3	81.8	79.2	71.2	56.3	50.6	44.5	16.2	8.8	5.1	2.1
Number	143	143	141	137	131	126	122	118	109	96	85	70	38	22	11	3

Table B4K-18 Age Group = 18-30 Gender = MALE Occupational group = OVERALL Total cases = 177

	Threshold dB HL≥															
	0	5	10	15	20	25	30	35	40	45	50	55	65	75	85	95
Prevalence	**84.3**	**61.7**	**37.3**	**21.2**	**10.0**	**6.9**	**5.6**	**2.4**	**0.8**	**0.6**	**0.6**	**0.6**	**0.6**	**0.6**	**0.6**	**0.0**
Lower CI	76.1	52.7	29.5	14.9	5.9	3.7	2.7	0.9	0.2	0.1	0.1	0.1	0.1	0.1	0.1	0.0
Upper CI	90.0	70.0	45.9	29.2	16.5	12.4	11.0	6.6	3.1	3.0	3.0	3.0	3.0	3.1	3.1	2.1
Number	154	118	76	42	22	14	11	6	4	3	3	3	3	2	1	0

Table B4K-19 Age Group = 31-40 Gender = MALE Occupational group = OVERALL Total cases = 142

	Threshold dB HL≥															
	0	5	10	15	20	25	30	35	40	45	50	55	65	75	85	95
Prevalence	**90.9**	**79.8**	**60.7**	**48.5**	**30.3**	**16.1**	**10.1**	**7.5**	**4.8**	**3.2**	**2.7**	**2.4**	**1.9**	**1.4**	**1.3**	**1.3**
Lower CI	82.2	70.1	49.9	38.1	21.7	10.1	5.5	3.9	2.2	1.3	1.0	0.8	0.5	0.3	0.3	0.3
Upper CI	95.6	86.9	70.5	59.0	40.5	24.8	17.9	13.6	10.2	7.4	6.9	6.7	6.4	6.2	6.4	6.4
Number	132	115	92	74	48	29	18	15	11	8	7	6	4	3	2	2

Table B4K-20 Age Group = 41-50 Gender = MALE Occupational group = OVERALL Total cases = 203

	Threshold dB HL≥															
	0	5	10	15	20	25	30	35	40	45	50	55	65	75	85	95
Prevalence	**97.2**	**91.6**	**77.2**	**59.0**	**44.1**	**33.7**	**26.7**	**21.1**	**16.9**	**12.5**	**9.5**	**6.6**	**3.3**	**2.7**	**1.2**	**0.3**
Lower CI	93.4	86.3	70.0	51.6	37.0	27.2	20.8	15.8	12.3	8.7	6.3	4.1	1.6	1.2	0.5	0.1
Upper CI	98.8	95.0	83.1	66.1	51.4	40.9	33.5	27.6	22.7	17.5	14.2	10.7	6.8	6.0	2.8	1.2
Number	198	187	165	133	107	85	69	56	48	39	31	23	12	10	6	2

Table B4K-21 Age Group = 51-60 Gender = MALE Occupational group = OVERALL Total cases = 341

	Threshold dB HL≥															
	0	5	10	15	20	25	30	35	40	45	50	55	65	75	85	95
Prevalence	**98.4**	**96.9**	**91.5**	**84.9**	**72.4**	**61.6**	**50.2**	**44.0**	**36.6**	**30.1**	**23.8**	**17.8**	**8.1**	**4.3**	**2.4**	**1.7**
Lower CI	96.1	94.1	87.4	79.8	66.6	55.7	44.6	38.5	31.5	25.4	19.7	14.5	5.9	2.8	1.4	0.8
Upper CI	99.4	98.4	94.3	88.8	77.4	67.2	55.8	49.6	42.0	35.2	28.4	21.6	11.0	6.6	4.3	3.6
Number	336	330	317	300	265	237	205	183	157	133	109	90	46	25	14	8

Prevalence by age group, gender and occupational group for the better ear over 4 kHz

Table B4K-22 Age Group = 61-70 Gender = MALE Occupational group = OVERALL Total cases = 285

	Threshold dB HL≥															
	0	5	10	15	20	25	30	35	40	45	50	55	65	75	85	95
Prevalence	100.0	99.8	99.5	97.1	92.2	82.9	73.3	67.7	57.6	50.4	44.2	35.7	19.9	9.9	5.5	2.5
Lower CI	98.7	98.8	98.4	94.0	86.0	75.8	65.7	59.9	50.2	43.3	37.5	29.5	16.0	7.5	3.5	1.4
Upper CI	100.0	100.0	99.8	98.7	95.7	88.2	79.7	74.6	64.7	57.4	51.2	42.3	24.6	12.9	8.4	4.3
Number	285	284	282	276	267	245	226	213	197	178	159	135	85	46	24	13

Table B4K-23 Age Group = 71-80 Gender = MALE Occupational group = OVERALL Total cases = 129

	Threshold dB HL≥															
	0	5	10	15	20	25	30	35	40	45	50	55	65	75	85	95
Prevalence	100.0	100.0	100.0	97.3	97.3	93.4	88.6	79.1	75.3	73.9	63.1	54.9	32.7	15.8	6.7	2.6
Lower CI	97.1	97.1	97.1	91.3	91.3	85.3	80.8	68.0	64.1	62.7	52.4	45.3	24.7	9.8	3.0	1.2
Upper CI	100.0	100.0	100.0	99.2	99.2	97.1	93.5	87.1	83.9	82.7	72.8	64.1	41.7	24.4	14.5	5.2
Number	129	129	129	127	127	124	119	114	110	107	96	88	60	30	11	7

Table B4K-24 Age Group = 18-30 Gender = OVERALL Occupational group = NON-MANUAL Total cases = 181

	Threshold dB HL≥															
	0	5	10	15	20	25	30	35	40	45	50	55	65	75	85	95
Prevalence	82.9	54.2	23.4	8.6	3.9	2.5	0.5	0.5	0.1	0.1	0.1	0.1	0.1	0.0	0.0	0.0
Lower CI	75.0	45.6	17.3	4.8	2.2	1.2	0.3	0.3	0.0	0.0	0.0	0.0	0.0	0.0	0.0	0.0
Upper CI	88.6	62.5	30.8	14.9	6.8	4.9	0.9	0.9	2.2	2.2	2.2	2.2	2.2	2.1	2.1	2.1
Number	153	104	49	18	12	8	3	3	1	1	1	1	1	0	0	0

Table B4K-25 Age Group = 31-40 Gender = OVERALL Occupational group = NON-MANUAL Total cases = 148

	Threshold dB HL≥															
	0	5	10	15	20	25	30	35	40	45	50	55	65	75	85	95
Prevalence	89.1	76.4	48.1	30.7	15.4	6.6	5.1	2.1	1.1	0.7	0.1	0.1	0.1	0.0	0.0	0.0
Lower CI	81.2	67.2	38.7	22.6	9.4	2.9	1.9	0.9	0.3	0.2	0.0	0.0	0.0	0.0	0.0	0.0
Upper CI	93.9	83.6	57.5	40.2	24.0	14.1	12.5	5.0	3.7	3.5	2.7	2.7	2.7	2.5	2.5	2.5
Number	133	115	77	46	27	13	8	5	3	2	1	1	1	0	0	0

Table B4K-26 Age Group = 41-50 Gender = OVERALL Occupational group = NON-MANUAL Total cases = 188

	Threshold dB HL≥															
	0	5	10	15	20	25	30	35	40	45	50	55	65	75	85	95
Prevalence	94.1	85.0	63.6	42.6	29.5	15.6	11.6	8.8	5.8	4.7	4.2	2.3	0.7	0.5	0.4	0.1
Lower CI	88.9	78.6	56.3	35.9	24.1	11.1	8.1	6.1	3.8	2.9	2.5	1.8	0.3	0.2	0.1	0.0
Upper CI	97.0	89.7	70.3	49.6	35.6	21.5	16.5	12.4	8.7	7.5	7.0	3.0	1.7	1.3	1.3	0.7
Number	178	159	117	81	56	37	28	22	16	13	11	7	4	3	2	1

Table B4K-27 Age Group = 51-60 Gender = OVERALL Occupational group = NON-MANUAL Total cases = 317

	Threshold dB HL≥															
	0	5	10	15	20	25	30	35	40	45	50	55	65	75	85	95
Prevalence	97.9	94.1	81.1	68.4	49.8	37.1	26.7	22.2	18.0	11.3	8.3	6.2	2.4	1.7	0.7	0.7
Lower CI	95.7	90.7	75.8	62.4	44.1	32.1	22.6	18.4	14.5	7.8	5.2	4.2	1.6	1.0	0.3	0.3
Upper CI	99.0	96.3	85.4	73.8	55.5	42.4	31.3	26.6	22.1	16.1	13.0	8.9	3.6	2.9	1.8	1.8
Number	309	295	259	226	175	136	106	89	70	54	40	36	19	12	5	5

Table B4K-28 Age Group = 61-70 Gender = OVERALL Occupational group = NON-MANUAL Total cases = 228

	Threshold dB HL≥															
	0	5	10	15	20	25	30	35	40	45	50	55	65	75	85	95
Prevalence	100.0	98.2	94.1	85.8	76.6	64.3	50.8	42.5	37.4	32.1	26.4	21.0	10.6	3.8	3.2	1.3
Lower CI	98.3	94.3	89.3	79.8	68.2	57.6	44.2	36.7	31.9	27.1	22.1	17.7	8.5	2.5	2.0	0.7
Upper CI	100.0	99.4	96.8	90.2	83.3	70.5	57.4	48.6	43.3	37.6	31.3	24.7	13.2	5.8	5.3	2.4
Number	228	225	215	199	184	160	131	116	106	95	77	62	40	15	11	7

Prevalence by age group, gender and occupational group for the better ear over 4 kHz

Table B4K-29 Age Group = 71-80 Gender = OVERALL Occupational group = NON-MANUAL Total cases = 123

	Threshold dB HL≥															
	0	5	10	15	20	25	30	35	40	45	50	55	65	75	85	95
**Prevalence**	**100.0**	**100.0**	**99.1**	**97.3**	**91.9**	**89.2**	**79.4**	**75.8**	**65.7**	**59.0**	**51.6**	**42.3**	**14.1**	**6.5**	**4.7**	**2.1**
Lower CI	97.0	97.0	95.1	93.2	83.1	80.4	67.0	63.0	56.3	50.5	43.5	34.7	10.1	4.0	2.5	1.4
Upper CI	100.0	100.0	99.8	99.0	96.3	94.3	88.0	85.1	74.0	67.0	59.7	50.4	19.4	10.4	8.6	3.1
Number	123	123	122	119	114	110	102	99	93	85	74	62	32	15	8	3

Table B4K-30 Age Group = 18-30 Gender = OVERALL Occupational group = MANUAL Total cases = 218

	Threshold dB HL≥															
	0	5	10	15	20	25	30	35	40	45	50	55	65	75	85	95
**Prevalence**	**82.7**	**68.9**	**42.5**	**24.6**	**9.8**	**6.6**	**5.2**	**2.4**	**1.0**	**0.7**	**0.6**	**0.6**	**0.5**	**0.5**	**0.5**	**0.0**
Lower CI	74.4	59.9	34.0	17.7	6.0	3.9	2.8	1.1	0.4	0.2	0.1	0.1	0.1	0.1	0.1	0.0
Upper CI	88.8	76.7	51.5	33.1	15.5	11.0	9.3	5.4	2.8	2.6	2.6	2.6	2.6	2.6	2.7	1.7
Number	186	155	103	62	33	22	17	10	6	4	3	3	2	2	1	0

Table B4K-31 Age Group = 31-40 Gender = OVERALL Occupational group = MANUAL Total cases = 183

	Threshold dB HL≥															
	0	5	10	15	20	25	30	35	40	45	50	55	65	75	85	95
**Prevalence**	**87.9**	**77.6**	**52.7**	**37.0**	**24.8**	**18.3**	**10.2**	**8.3**	**5.5**	**3.7**	**2.4**	**2.2**	**1.9**	**1.3**	**1.0**	**1.0**
Lower CI	79.8	68.6	43.2	28.6	17.7	12.3	6.1	4.6	2.7	1.8	1.0	0.9	0.7	0.4	0.2	0.2
Upper CI	93.0	84.6	62.0	46.2	33.6	26.3	16.6	14.6	10.8	7.4	5.5	5.4	5.1	4.7	4.9	4.9
Number	167	148	109	83	53	38	27	21	15	12	10	9	7	5	2	2

Table B4K-32 Age Group = 41-50 Gender = OVERALL Occupational group = MANUAL Total cases = 262

	Threshold dB HL≥															
	0	5	10	15	20	25	30	35	40	45	50	55	65	75	85	95
**Prevalence**	**96.9**	**92.2**	**75.6**	**58.7**	**45.5**	**32.7**	**23.5**	**17.5**	**14.6**	**10.2**	**6.4**	**4.6**	**3.0**	**2.3**	**0.7**	**0.2**
Lower CI	93.4	87.7	68.7	51.6	38.7	26.7	18.3	13.0	10.5	6.9	4.0	2.7	1.4	0.9	0.3	0.1
Upper CI	98.5	95.1	81.3	65.4	52.5	39.4	29.6	23.2	20.0	14.9	10.2	7.9	6.2	5.7	1.6	0.7
Number	255	242	210	168	132	103	79	62	54	41	31	23	14	10	6	2

Table B4K-33 Age Group = 51-60 Gender = OVERALL Occupational group = MANUAL Total cases = 365

	Threshold dB HL≥															
	0	5	10	15	20	25	30	35	40	45	50	55	65	75	85	95
**Prevalence**	**99.0**	**97.3**	**91.0**	**78.2**	**63.5**	**52.4**	**37.9**	**31.1**	**26.1**	**21.7**	**17.4**	**12.3**	**5.8**	**3.2**	**1.9**	**1.4**
Lower CI	96.4	94.1	86.5	72.5	57.6	46.8	33.4	26.9	22.0	18.1	14.2	9.9	4.2	2.1	1.1	0.7
Upper CI	99.7	98.8	94.1	83.0	68.9	57.9	42.7	35.7	30.5	25.9	21.2	15.2	8.0	4.7	3.2	2.8
Number	363	358	342	313	273	238	196	167	144	122	102	84	46	27	15	9

Table B4K-34 Age Group = 61-70 Gender = OVERALL Occupational group = MANUAL Total cases = 299

	Threshold dB HL≥															
	0	5	10	15	20	25	30	35	40	45	50	55	65	75	85	95
**Prevalence**	**99.3**	**99.0**	**95.3**	**92.2**	**81.8**	**75.5**	**63.4**	**55.1**	**42.1**	**35.5**	**30.5**	**25.0**	**13.6**	**7.5**	**3.8**	**2.2**
Lower CI	96.0	96.0	90.0	86.8	75.4	69.1	56.5	48.4	35.6	29.5	25.2	20.4	10.7	5.8	2.6	1.5
Upper CI	99.9	99.8	97.8	95.5	86.8	80.9	69.8	61.6	48.8	42.0	36.3	30.2	17.2	9.7	5.7	3.2
Number	298	297	292	284	262	243	222	202	182	157	142	121	78	47	20	11

Table B4K-35 Age Group = 71-80 Gender = OVERALL Occupational group = MANUAL Total cases = 148

	Threshold dB HL≥															
	0	5	10	15	20	25	30	35	40	45	50	55	65	75	85	95
**Prevalence**	**100.0**	**100.0**	**97.4**	**93.3**	**90.7**	**84.5**	**82.5**	**71.4**	**68.7**	**60.3**	**52.2**	**45.3**	**24.2**	**12.7**	**4.4**	**1.7**
Lower CI	97.5	97.5	86.6	83.2	81.7	74.0	72.7	59.9	57.3	53.1	44.2	36.2	18.3	8.2	2.5	0.9
Upper CI	100.0	100.0	99.6	97.5	95.5	91.2	89.2	80.8	78.3	67.0	60.0	54.8	31.3	19.3	7.5	3.5
Number	148	148	147	144	143	139	138	132	125	117	106	95	66	37	14	7

Prevalence by age group, gender and occupational group for the better ear over 4 kHz

Table B4K-36 Age Group = 18-80 Gender = FEMALE Occupational group = NON-MANUAL Total cases = 671

	Threshold dB HL≥															
	**0**	**5**	**10**	**15**	**20**	**25**	**30**	**35**	**40**	**45**	**50**	**55**	**65**	**75**	**85**	**95**
**Prevalence**	**92.0**	**79.8**	**57.5**	**41.4**	**31.9**	**25.2**	**18.7**	**16.1**	**13.0**	**10.4**	**8.9**	**7.1**	**2.2**	**1.1**	**0.6**	**0.3**
**Lower CI**	89.1	76.2	54.1	38.5	29.3	23.0	16.4	14.0	11.3	9.0	7.6	5.9	1.7	0.7	0.3	0.2
**Upper CI**	94.2	83.0	60.8	44.3	34.7	27.6	21.3	18.4	14.8	12.0	10.3	8.4	2.9	1.6	1.0	0.7
**Number**	633	569	442	345	275	222	175	154	131	111	92	77	45	23	12	8

Table B4K-37 Age Group = 18-80 Gender = MALE Occupational group = NON-MANUAL Total cases = 514

	Threshold dB HL≥															
	**0**	**5**	**10**	**15**	**20**	**25**	**30**	**35**	**40**	**45**	**50**	**55**	**65**	**75**	**85**	**95**
**Prevalence**	**92.4**	**80.3**	**64.1**	**53.0**	**40.6**	**30.6**	**25.1**	**21.1**	**18.1**	**15.0**	**12.1**	**9.4**	**4.6**	**1.9**	**1.5**	**0.7**
**Lower CI**	87.5	74.9	59.2	48.5	36.9	27.5	22.3	19.0	16.1	13.0	10.2	8.1	3.6	1.3	1.0	0.4
**Upper CI**	95.4	84.7	68.7	57.4	44.4	33.9	28.0	23.4	20.3	17.3	14.2	11.0	5.7	2.9	2.4	1.0
**Number**	491	452	397	344	293	242	203	180	158	139	112	92	52	22	14	8

Table B4K-38 Age Group = 18-80 Gender = FEMALE Occupational group = MANUAL Total cases = 715

	Threshold dB HL≥															
	**0**	**5**	**10**	**15**	**20**	**25**	**30**	**35**	**40**	**45**	**50**	**55**	**65**	**75**	**85**	**95**
**Prevalence**	**91.7**	**85.3**	**65.4**	**49.7**	**35.7**	**28.2**	**20.1**	**15.3**	**12.5**	**9.2**	**6.4**	**5.1**	**2.9**	**1.8**	**0.8**	**0.5**
**Lower CI**	88.0	81.1	60.8	45.7	32.6	25.1	17.6	12.9	10.3	7.6	5.2	4.1	2.3	1.4	0.6	0.3
**Upper CI**	94.4	88.6	69.7	53.8	39.0	31.5	22.8	17.9	15.1	11.0	7.7	6.3	3.7	2.4	1.3	0.7
**Number**	677	639	541	447	354	292	235	188	158	125	102	83	55	34	14	7

Table B4K-39 Age Group = 18-80 Gender = MALE Occupational group = MANUAL Total cases = 760

	Threshold dB HL≥															
	**0**	**5**	**10**	**15**	**20**	**25**	**30**	**35**	**40**	**45**	**50**	**55**	**65**	**75**	**85**	**95**
**Prevalence**	**93.5**	**86.8**	**75.4**	**64.8**	**54.4**	**47.3**	**40.2**	**34.3**	**28.5**	**24.6**	**21.3**	**17.5**	**9.4**	**5.3**	**2.6**	**1.4**
**Lower CI**	89.3	82.2	70.5	60.1	50.3	43.8	36.9	31.3	25.8	22.3	19.0	15.2	7.8	4.0	1.7	0.8
**Upper CI**	96.1	90.3	79.7	69.3	58.5	50.9	43.6	37.5	31.3	27.1	23.8	19.9	11.4	6.9	3.8	2.4
**Number**	740	709	662	607	542	491	444	406	368	328	292	252	158	94	44	24

Table B4K-40 Age Group = 18-30 Gender = FEMALE Occupational group = NON-MANUAL Total cases = 115

	Threshold dB HL≥															
	**0**	**5**	**10**	**15**	**20**	**25**	**30**	**35**	**40**	**45**	**50**	**55**	**65**	**75**	**85**	**95**
**Prevalence**	**82.1**	**54.7**	**22.3**	**7.6**	**6.2**	**3.5**	**0.8**	**0.8**	**0.0**	**0.0**	**0.0**	**0.0**	**0.0**	**0.0**	**0.0**	**0.0**
**Lower CI**	73.5	44.9	15.9	4.4	3.4	1.7	0.4	0.4	0.0	0.0	0.0	0.0	0.0	0.0	0.0	0.0
**Upper CI**	88.4	64.2	30.4	12.8	10.8	7.0	1.5	1.5	3.2	3.2	3.2	3.2	3.2	3.2	3.2	3.2
**Number**	96	65	31	12	10	6	2	2	0	0	0	0	0	0	0	0

Table B4K-41 Age Group = 31-40 Gender = FEMALE Occupational group = NON-MANUAL Total cases = 86

	Threshold dB HL≥															
	**0**	**5**	**10**	**15**	**20**	**25**	**30**	**35**	**40**	**45**	**50**	**55**	**65**	**75**	**85**	**95**
**Prevalence**	**86.4**	**73.2**	**39.6**	**15.9**	**7.8**	**4.0**	**4.0**	**2.8**	**2.0**	**1.4**	**0.2**	**0.2**	**0.2**	**0.0**	**0.0**	**0.0**
**Lower CI**	76.6	62.2	29.7	9.6	4.0	1.9	1.9	1.1	0.6	0.3	0.0	0.0	0.0	0.0	0.0	0.0
**Upper CI**	92.5	81.9	50.3	25.1	14.5	8.4	8.4	6.9	6.8	6.5	4.6	4.6	4.6	4.3	4.3	4.3
**Number**	75	64	38	17	10	6	6	4	3	2	1	1	1	0	0	0

Table B4K-42 Age Group = 41-50 Gender = FEMALE Occupational group = NON-MANUAL Total cases = 112

	Threshold dB HL≥															
	**0**	**5**	**10**	**15**	**20**	**25**	**30**	**35**	**40**	**45**	**50**	**55**	**65**	**75**	**85**	**95**
**Prevalence**	**94.8**	**84.1**	**58.9**	**36.0**	**22.5**	**8.4**	**5.8**	**4.4**	**2.6**	**1.1**	**0.9**	**0.4**	**0.4**	**0.0**	**0.0**	**0.0**
**Lower CI**	87.9	75.5	49.4	27.9	16.5	5.6	3.8	3.1	1.4	0.4	0.2	0.1	0.1	0.0	0.0	0.0
**Upper CI**	97.8	90.1	67.7	45.0	29.8	12.4	8.9	6.3	4.6	3.4	3.2	2.2	2.2	3.3	3.3	3.3
**Number**	107	95	65	43	28	15	10	8	5	3	2	1	1	0	0	0

Prevalence by age group, gender and occupational group for the better ear over 4 kHz

Table B4K-43 Age Group = 51-60 Gender = FEMALE Occupational group = NON-MANUAL Total cases = 175

Threshold dB HL≥	0	5	10	15	20	25	30	35	40	45	50	55	65	75	85	95
Prevalence	98.7	94.6	76.0	59.4	38.4	27.0	16.5	12.3	10.1	6.8	5.1	4.9	2.5	2.1	1.0	1.0
Lower CI	95.9	90.3	68.3	51.0	31.2	21.1	12.1	8.4	6.6	4.6	3.1	2.9	1.5	1.1	0.4	0.4
Upper CI	99.6	97.1	82.3	67.3	46.1	34.0	22.2	17.5	15.2	9.9	8.2	8.0	4.4	4.0	2.9	2.9
Number	172	164	136	113	81	60	43	35	28	23	17	16	10	8	4	4

Table B4K-44 Age Group = 61-70 Gender = FEMALE Occupational group = NON-MANUAL Total cases = 116

Threshold dB HL≥	0	5	10	15	20	25	30	35	40	45	50	55	65	75	85	95
Prevalence	100.0	97.0	90.2	77.6	69.5	61.1	43.6	33.6	27.0	23.3	19.5	14.9	5.7	1.5	1.1	1.1
Lower CI	96.8	89.5	81.5	67.2	59.4	51.0	34.2	26.0	20.3	17.1	14.0	10.9	4.0	0.6	0.4	0.4
Upper CI	100.0	99.2	95.1	85.4	78.0	70.3	53.4	42.2	34.8	30.9	26.5	20.1	8.2	3.4	3.0	3.0
Number	116	114	106	95	86	76	58	49	42	37	30	25	18	6	4	4

Table B4K-45 Age Group = 71-80 Gender = FEMALE Occupational group = NON-MANUAL Total cases = 67

Threshold dB HL≥	0	5	10	15	20	25	30	35	40	45	50	55	65	75	85	95
Prevalence	100.0	100.0	98.6	98.2	89.3	88.9	78.3	78.3	66.0	55.4	51.0	40.2	8.5	4.4	2.3	0.0
Lower CI	94.6	94.6	92.2	92.4	75.4	75.1	58.8	58.8	53.4	44.9	40.9	30.9	5.2	2.5	0.9	0.0
Upper CI	100.0	100.0	99.7	99.6	95.8	95.5	90.2	90.2	76.7	65.3	61.1	50.3	13.8	7.5	5.5	5.4
Number	67	67	66	65	60	59	56	56	53	46	42	34	15	9	4	0

Table B4K-46 Age Group = 18-30 Gender = MALE Occupational group = NON-MANUAL Total cases = 66

Threshold dB HL≥	0	5	10	15	20	25	30	35	40	45	50	55	65	75	85	95
Prevalence	83.5	54.0	24.4	9.4	1.4	1.4	0.1	0.1	0.1	0.1	0.1	0.1	0.1	0.0	0.0	0.0
Lower CI	69.0	40.1	14.9	3.6	0.3	0.3	0.0	0.0	0.0	0.0	0.0	0.0	0.0	0.0	0.0	0.0
Upper CI	91.9	67.3	37.3	22.3	6.7	6.7	5.7	5.7	5.7	5.7	5.7	5.7	5.7	5.5	5.5	5.5
Number	57	39	18	6	2	2	1	1	1	1	1	1	1	0	0	0

Table B4K-47 Age Group = 31-40 Gender = MALE Occupational group = NON-MANUAL Total cases = 62

Threshold dB HL≥	0	5	10	15	20	25	30	35	40	45	50	55	65	75	85	95
Prevalence	92.1	79.9	57.3	47.4	23.9	9.4	6.2	1.3	0.0	0.0	0.0	0.0	0.0	0.0	0.0	0.0
Lower CI	76.0	63.3	40.7	31.6	12.6	3.0	1.4	0.2	0.0	0.0	0.0	0.0	0.0	0.0	0.0	0.0
Upper CI	97.7	90.1	72.4	63.7	40.5	25.6	24.0	6.9	5.8	5.8	5.8	5.8	5.8	5.8	5.8	5.8
Number	58	51	39	29	17	7	2	1	0	0	0	0	0	0	0	0

Table B4K-48 Age Group = 41-50 Gender = MALE Occupational group = NON-MANUAL Total cases = 76

Threshold dB HL≥	0	5	10	15	20	25	30	35	40	45	50	55	65	75	85	95
Prevalence	93.3	86.0	68.7	49.9	37.6	23.5	17.9	13.4	9.2	8.5	7.8	4.4	1.0	1.0	0.7	0.3
Lower CI	83.6	75.6	57.2	39.1	28.4	14.9	11.1	8.3	5.5	5.0	4.5	3.6	0.4	0.4	0.2	0.0
Upper CI	97.5	92.4	78.3	60.6	47.8	35.2	27.7	20.9	15.0	14.2	13.3	5.5	2.8	2.8	2.8	1.4
Number	71	64	52	38	28	22	18	14	11	10	9	6	3	3	2	1

Table B4K-49 Age Group = 51-60 Gender = MALE Occupational group = NON-MANUAL Total cases = 142

Threshold dB HL≥	0	5	10	15	20	25	30	35	40	45	50	55	65	75	85	95
Prevalence	96.9	93.4	86.7	78.5	62.4	48.2	38.2	33.4	26.7	16.4	11.8	7.6	2.3	1.3	0.3	0.3
Lower CI	92.6	87.3	79.1	69.8	53.5	40.2	31.3	26.9	20.8	9.8	6.1	4.4	1.3	0.5	0.1	0.1
Upper CI	98.8	96.7	91.8	85.2	70.5	56.3	45.6	40.6	33.6	26.2	21.6	12.7	4.1	3.0	1.9	1.9
Number	137	131	123	113	94	76	63	54	42	31	23	20	9	4	1	1

Prevalence by age group, gender and occupational group for the better ear over 4 kHz

Table B4K-50 Age Group = 61-70 Gender = MALE Occupational group = NON-MANUAL Total cases = 112

	Threshold dB HL≥															
	0	5	10	15	20	25	30	35	40	45	50	55	65	75	85	95
Prevalence	**100.0**	**99.4**	**98.3**	**95.1**	**84.0**	**67.2**	**58.3**	**52.4**	**49.1**	**42.0**	**34.1**	**27.6**	**15.9**	**6.4**	**5.7**	**1.5**
Lower CI	96.7	96.4	95.3	90.0	67.8	58.7	49.4	43.7	40.3	34.3	27.5	22.3	12.1	4.0	3.4	0.7
Upper CI	100.0	99.9	99.4	97.6	92.9	74.7	66.7	60.9	57.9	50.0	41.3	33.6	20.6	10.1	9.4	3.0
Number	112	111	109	104	98	84	73	67	64	58	47	37	22	9	7	3

Table B4K-51 Age Group = 71-80 Gender = MALE Occupational group = NON-MANUAL Total cases = 56

	Threshold dB HL≥															
	0	5	10	15	20	25	30	35	40	45	50	55	65	75	85	95
Prevalence	**100.0**	**100.0**	**100.0**	**95.8**	**95.8**	**89.5**	**80.5**	**71.6**	**65.2**	**64.1**	**52.2**	**45.0**	**22.5**	**9.9**	**8.6**	**5.6**
Lower CI	93.6	93.6	93.6	87.2	87.2	78.2	69.5	57.3	51.3	50.1	39.7	33.5	14.6	5.0	4.1	3.7
Upper CI	100.0	100.0	100.0	98.7	98.7	95.3	88.2	82.5	76.9	76.0	64.3	57.1	33.1	18.6	17.3	8.2
Number	56	56	56	54	54	51	46	43	40	39	32	28	17	6	4	3

Table B4K-52 Age Group = 18-30 Gender = FEMALE Occupational group = MANUAL Total cases = 109

	Threshold dB HL≥															
	0	5	10	15	20	25	30	35	40	45	50	55	65	75	85	95
Prevalence	**82.1**	**70.4**	**39.1**	**18.4**	**4.1**	**3.1**	**1.5**	**1.1**	**0.7**	**0.5**	**0.2**	**0.2**	**0.0**	**0.0**	**0.0**	**0.0**
Lower CI	70.0	57.8	27.6	10.5	2.2	1.4	0.7	0.5	0.2	0.1	0.0	0.0	0.0	0.0	0.0	0.0
Upper CI	90.0	80.5	52.0	30.1	7.7	6.8	3.1	2.5	2.1	1.7	1.1	1.1	3.4	3.4	3.4	3.4
Number	91	77	46	26	13	10	7	5	3	2	1	1	0	0	0	0

Table B4K-53 Age Group = 31-40 Gender = FEMALE Occupational group = MANUAL Total cases = 103

	Threshold dB HL≥															
	0	5	10	15	20	25	30	35	40	45	50	55	65	75	85	95
Prevalence	**87.4**	**76.8**	**44.3**	**27.1**	**15.1**	**12.1**	**5.8**	**3.3**	**2.4**	**2.4**	**0.8**	**0.8**	**0.8**	**0.5**	**0.0**	**0.0**
Lower CI	76.5	64.3	32.9	18.4	8.6	6.2	2.7	1.2	0.7	0.7	0.3	0.3	0.3	0.1	0.0	0.0
Upper CI	93.7	86.0	56.4	38.0	25.3	22.3	12.0	8.6	8.3	8.3	2.3	2.3	2.3	1.6	3.6	3.6
Number	93	84	56	38	22	16	11	7	4	4	3	3	3	2	0	0

Table B4K-54 Age Group = 41-50 Gender = FEMALE Occupational group = MANUAL Total cases = 135

	Threshold dB HL≥															
	0	5	10	15	20	25	30	35	40	45	50	55	65	75	85	95
Prevalence	**93.9**	**87.5**	**66.0**	**49.8**	**36.3**	**23.6**	**13.6**	**8.7**	**7.3**	**5.2**	**2.6**	**1.8**	**1.6**	**1.1**	**0.4**	**0.2**
Lower CI	87.4	79.8	56.0	40.1	27.7	16.6	8.9	5.3	4.1	2.6	1.3	0.7	0.6	0.3	0.1	0.0
Upper CI	97.2	92.6	74.8	59.6	45.8	32.5	20.2	14.1	12.8	10.3	5.4	4.4	4.3	4.0	1.3	1.0
Number	128	119	97	73	53	40	28	20	17	12	9	6	5	3	2	1

Table B4K-55 Age Group = 51-60 Gender = FEMALE Occupational group = MANUAL Total cases = 166

	Threshold dB HL≥															
	0	5	10	15	20	25	30	35	40	45	50	55	65	75	85	95
Prevalence	**98.2**	**94.8**	**86.4**	**67.0**	**48.2**	**34.0**	**18.0**	**11.6**	**8.8**	**5.8**	**3.5**	**2.5**	**1.4**	**1.0**	**0.3**	**0.3**
Lower CI	93.2	88.9	78.7	57.6	39.4	26.5	13.2	7.7	5.4	3.2	2.0	1.5	0.8	0.4	0.1	0.1
Upper CI	99.5	97.7	91.6	75.3	57.0	42.5	24.1	17.2	14.1	10.0	6.0	4.2	2.5	2.2	1.1	1.1
Number	164	159	148	126	102	77	54	38	29	20	16	14	9	6	2	2

Table B4K-56 Age Group = 61-70 Gender = FEMALE Occupational group = MANUAL Total cases = 126

	Threshold dB HL≥															
	0	5	10	15	20	25	30	35	40	45	50	55	65	75	85	95
Prevalence	**98.6**	**98.1**	**91.3**	**86.9**	**68.4**	**59.8**	**46.0**	**34.8**	**24.9**	**18.2**	**12.0**	**8.2**	**6.0**	**4.0**	**2.1**	**1.6**
Lower CI	92.4	92.5	82.0	77.7	57.6	49.3	36.3	26.3	17.6	12.0	7.9	6.0	4.2	2.8	1.5	0.5
Upper CI	99.8	99.6	96.0	92.7	77.5	69.4	56.1	44.5	34.1	26.7	18.0	11.1	8.5	5.6	2.7	5.7
Number	125	124	119	112	93	82	69	56	49	37	30	23	15	10	3	1

Prevalence by age group, gender and occupational group for the better ear over 4 kHz

Table B4K-57 Age Group = 71-80 Gender = FEMALE Occupational group = MANUAL Total cases = 76

Threshold dB HL≥

	0	5	10	15	20	25	30	35	40	45	50	55	65	75	85	95
Prevalence	**100.0**	**100.0**	**95.7**	**88.6**	**84.3**	**73.7**	**70.3**	**62.3**	**58.0**	**44.7**	**36.0**	**31.4**	**13.1**	**7.0**	**3.8**	**1.3**
Lower CI	95.2	95.2	78.7	72.5	69.8	57.4	55.1	44.3	40.5	34.2	25.7	21.2	8.8	4.3	1.8	0.5
Upper CI	100.0	100.0	99.3	95.8	92.5	85.4	82.1	77.4	73.7	55.8	47.9	43.8	19.0	11.3	7.9	3.6
Number	76	76	75	72	71	67	66	62	56	50	43	36	23	13	7	3

Table B4K-58 Age Group = 18-30 Gender = MALE Occupational group = MANUAL Total cases = 109

Threshold dB HL≥

	0	5	10	15	20	25	30	35	40	45	50	55	65	75	85	95
Prevalence	**83.6**	**67.6**	**46.0**	**31.1**	**15.6**	**10.1**	**8.8**	**3.8**	**1.3**	**1.0**	**1.0**	**1.0**	**1.0**	**1.0**	**0.9**	**0.0**
Lower CI	71.0	54.3	34.1	20.5	8.5	5.2	4.3	1.4	0.3	0.2	0.2	0.2	0.2	0.2	0.2	0.0
Upper CI	91.4	78.5	58.4	44.0	26.9	18.5	17.1	10.1	5.0	5.1	5.1	5.1	5.1	5.1	5.2	3.4
Number	95	78	57	36	20	12	10	5	3	2	2	2	2	2	1	0

Table B4K-59 Age Group = 31-40 Gender = MALE Occupational group = MANUAL Total cases = 80

Threshold dB HL≥

	0	5	10	15	20	25	30	35	40	45	50	55	65	75	85	95
Prevalence	**88.5**	**78.6**	**61.7**	**47.4**	**35.2**	**24.8**	**14.9**	**13.7**	**9.1**	**5.1**	**4.2**	**3.8**	**3.0**	**2.3**	**2.1**	**2.1**
Lower CI	74.8	64.8	46.1	33.1	22.7	14.9	7.7	6.8	4.0	2.2	1.6	1.3	0.9	0.5	0.4	0.4
Upper CI	95.2	88.0	75.2	62.3	50.1	38.3	26.9	25.9	19.4	11.1	10.7	10.5	10.1	9.8	9.9	9.9
Number	74	64	53	45	31	22	16	14	11	8	7	6	4	3	2	2

Table B4K-60 Age Group = 41-50 Gender = MALE Occupational group = MANUAL Total cases = 127

Threshold dB HL≥

	0	5	10	15	20	25	30	35	40	45	50	55	65	75	85	95
Prevalence	**100.0**	**97.2**	**85.8**	**68.1**	**55.5**	**42.5**	**34.2**	**27.0**	**22.5**	**15.7**	**10.7**	**7.8**	**4.5**	**3.7**	**1.1**	**0.2**
Lower CI	97.1	91.4	75.7	57.7	45.0	32.9	24.9	18.7	15.0	9.8	6.0	4.1	1.7	1.2	0.4	0.0
Upper CI	100.0	99.1	92.2	77.0	65.5	52.7	44.8	37.4	32.4	24.3	18.2	14.5	11.5	10.8	2.9	1.3
Number	127	123	113	95	79	63	51	42	37	29	22	17	9	7	4	1

Table B4K-61 Age Group = 51-60 Gender = MALE Occupational group = MANUAL Total cases = 199

Threshold dB HL≥

	0	5	10	15	20	25	30	35	40	45	50	55	65	75	85	95
Prevalence	**100.0**	**100.0**	**96.1**	**90.6**	**80.3**	**72.7**	**60.1**	**52.8**	**45.3**	**39.5**	**32.8**	**23.1**	**10.7**	**5.6**	**3.6**	**2.5**
Lower CI	98.1	98.1	90.8	84.1	72.7	64.6	52.2	45.2	37.8	32.4	26.2	18.1	7.4	3.6	2.0	1.1
Upper CI	100.0	100.0	98.4	94.6	86.2	79.6	67.5	60.4	52.9	47.1	40.2	28.9	15.2	8.7	6.3	5.4
Number	199	199	194	187	171	161	142	129	115	102	86	70	37	21	13	7

Table B4K-62 Age Group = 61-70 Gender = MALE Occupational group = MANUAL Total cases = 173

Threshold dB HL≥

	0	5	10	15	20	25	30	35	40	45	50	55	65	75	85	95
Prevalence	**100.0**	**100.0**	**100.0**	**98.6**	**97.7**	**94.2**	**84.0**	**79.1**	**62.7**	**56.1**	**52.5**	**44.8**	**23.0**	**12.0**	**6.2**	**2.9**
Lower CI	97.8	97.8	97.8	92.2	92.4	88.4	73.7	68.1	51.6	45.5	42.0	35.0	17.1	8.5	3.5	1.5
Upper CI	100.0	100.0	100.0	99.7	99.4	97.2	90.8	87.1	72.6	66.1	62.7	55.1	30.3	16.6	10.7	5.3
Number	173	173	173	172	169	161	153	146	133	120	112	98	63	37	17	10

Table B4K-63 Age Group = 71-80 Gender = MALE Occupational group = MANUAL Total cases = 72

Threshold dB HL≥

	0	5	10	15	20	25	30	35	40	45	50	55	65	75	85	95
Prevalence	**100.0**	**100.0**	**100.0**	**100.0**	**100.0**	**100.0**	**100.0**	**85.2**	**84.8**	**83.4**	**76.2**	**65.9**	**40.7**	**21.3**	**5.2**	**2.4**
Lower CI	94.9	94.9	94.9	94.9	94.9	94.9	94.9	79.6	79.2	77.3	64.7	49.2	27.5	11.3	2.3	0.9
Upper CI	100.0	100.0	100.0	100.0	100.0	100.0	100.0	89.4	89.1	88.0	84.9	79.4	55.5	36.5	11.3	5.9
Number	72	72	72	72	72	72	72	70	69	67	63	59	43	24	7	4

Prevalence by age group, gender and occupational group for the better ear over 6 kHz

Table B6K-1 Age Group = 18-80 Gender = OVERALL Occupational group = OVERALL Total cases = 2660

Threshold dB HL≥	0	5	10	15	20	25	30	35	40	45	50	55	65	75	85	95
**Prevalence**	**97.7**	**95.2**	**87.8**	**77.0**	**65.8**	**53.1**	**42.2**	**35.5**	**29.7**	**25.7**	**22.5**	**18.3**	**12.3**	**7.0**	**3.6**	**1.8**
**Lower CI**	96.7	93.9	85.9	75.0	63.7	51.1	40.5	33.9	28.2	24.3	21.1	17.1	11.3	6.2	3.0	1.4
**Upper CI**	98.4	96.3	89.4	79.0	67.9	55.1	44.0	37.2	31.2	27.2	24.0	19.6	13.4	7.8	4.2	2.3
**Number**	2625	2589	2470	2262	2031	1753	1503	1313	1147	1015	908	789	573	360	199	110

Table B6K-2 Age Group = 18-80 Gender = FEMALE Occupational group = OVERALL Total cases = 1386

Threshold dB HL≥	0	5	10	15	20	25	30	35	40	45	50	55	65	75	85	95
**Prevalence**	**97.9**	**95.8**	**87.3**	**74.7**	**61.6**	**49.4**	**37.6**	**30.9**	**24.7**	**21.5**	**18.6**	**14.8**	**9.1**	**5.1**	**2.2**	**1.1**
**Lower CI**	96.6	94.2	84.9	72.0	58.9	46.8	35.3	28.7	22.8	19.6	16.8	13.3	7.9	4.3	1.7	0.7
**Upper CI**	98.7	97.0	89.4	77.2	64.2	52.0	40.1	33.1	26.7	23.4	20.6	16.4	10.6	6.1	2.9	1.5
**Number**	1367	1348	1268	1131	980	820	671	571	472	409	360	302	210	133	71	37

Table B6K-3 Age Group = 18-80 Gender = MALE Occupational group = OVERALL Total cases = 1274

Threshold dB HL≥	0	5	10	15	20	25	30	35	40	45	50	55	65	75	85	95
**Prevalence**	**97.5**	**94.6**	**88.3**	**79.7**	**70.7**	**57.3**	**47.4**	**40.7**	**35.2**	**30.5**	**26.9**	**22.3**	**15.8**	**9.1**	**5.1**	**2.6**
**Lower CI**	95.8	92.2	85.2	76.5	67.3	54.2	44.8	38.2	32.9	28.3	24.7	20.4	14.2	7.9	4.1	1.9
**Upper CI**	98.5	96.3	90.8	82.7	73.8	60.2	50.1	43.3	37.6	32.8	29.2	24.2	17.6	10.5	6.3	3.5
**Number**	1258	1241	1202	1131	1051	933	832	742	675	606	548	487	363	227	128	73

Table B6K-4 Age Group = 18-30 Gender = OVERALL Occupational group = OVERALL Total cases = 401

Threshold dB HL≥	0	5	10	15	20	25	30	35	40	45	50	55	65	75	85	95
**Prevalence**	**93.4**	**86.7**	**69.8**	**49.7**	**33.9**	**18.9**	**8.5**	**5.7**	**2.4**	**2.2**	**2.0**	**0.7**	**0.3**	**0.3**	**0.3**	**0.3**
**Lower CI**	90.3	82.3	64.1	44.0	28.6	14.9	5.8	3.5	1.1	1.0	0.8	0.2	0.1	0.1	0.1	0.1
**Upper CI**	95.6	90.1	75.0	55.4	39.5	23.6	12.2	9.2	4.8	4.7	4.6	2.1	1.5	1.5	1.6	1.6
**Number**	376	353	295	211	143	84	44	27	15	13	10	5	3	3	2	2

Table B6K-5 Age Group = 31-40 Gender = OVERALL Occupational group = OVERALL Total cases = 331

Threshold dB HL≥	0	5	10	15	20	25	30	35	40	45	50	55	65	75	85	95
**Prevalence**	**97.6**	**95.2**	**86.4**	**70.4**	**53.3**	**35.8**	**22.5**	**16.4**	**10.3**	**7.0**	**6.2**	**3.8**	**2.1**	**1.9**	**0.6**	**0.6**
**Lower CI**	94.9	91.2	81.0	64.1	46.8	29.7	17.5	12.1	7.0	4.4	3.6	2.1	1.0	0.9	0.1	0.1
**Upper CI**	98.8	97.4	90.4	76.0	59.8	42.3	28.3	21.7	14.8	11.1	10.3	6.7	4.3	4.1	3.0	3.1
**Number**	323	316	291	241	189	129	89	67	44	29	24	18	12	10	2	1

Table B6K-6 Age Group = 41-50 Gender = OVERALL Occupational group = OVERALL Total cases = 450

Threshold dB HL≥	0	5	10	15	20	25	30	35	40	45	50	55	65	75	85	95
**Prevalence**	**99.7**	**98.4**	**92.4**	**81.7**	**67.3**	**50.9**	**36.7**	**27.9**	**21.4**	**16.2**	**12.4**	**9.4**	**5.1**	**2.4**	**1.5**	**0.7**
**Lower CI**	98.1	96.2	89.1	77.4	62.7	46.0	32.2	23.8	17.8	13.1	9.7	7.1	3.6	1.5	0.7	0.4
**Upper CI**	99.9	99.3	94.7	85.4	71.7	55.9	41.4	32.3	25.6	19.9	15.7	12.3	7.3	4.0	3.0	1.3
**Number**	449	444	422	376	319	251	193	154	123	98	78	63	40	22	14	9

Table B6K-7 Age Group = 51-60 Gender = OVERALL Occupational group = OVERALL Total cases = 680

Threshold dB HL≥	0	5	10	15	20	25	30	35	40	45	50	55	65	75	85	95
**Prevalence**	**99.8**	**99.7**	**97.3**	**92.2**	**84.4**	**73.0**	**61.2**	**49.4**	**39.8**	**32.3**	**26.5**	**21.7**	**12.7**	**7.0**	**3.8**	**2.3**
**Lower CI**	98.7	98.7	95.4	89.4	81.0	69.1	57.1	45.4	36.0	28.8	23.4	18.8	10.5	5.4	2.7	1.5
**Upper CI**	100.0	99.9	98.4	94.2	87.3	76.6	65.1	53.5	43.7	36.1	30.0	24.9	15.2	8.9	5.3	3.5
**Number**	679	678	665	639	596	532	462	391	329	274	236	199	127	74	43	27

Prevalence by age group, gender and occupational group for the better ear over 6 kHz

Table B6K-8 Age Group = 61-70 Gender = OVERALL Occupational group = OVERALL Total cases = 527

Threshold dB HL≥	0	5	10	15	20	25	30	35	40	45	50	55	65	75	85	95
**Prevalence**	**100.0**	**100.0**	**99.6**	**99.6**	**97.7**	**90.1**	**83.1**	**73.7**	**65.6**	**60.5**	**53.8**	**45.2**	**30.3**	**16.2**	**8.0**	**3.9**
**Lower CI**	99.3	99.3	98.0	98.0	95.3	86.0	78.6	68.9	60.5	55.4	48.8	40.5	26.1	13.4	6.1	2.8
**Upper CI**	100.0	100.0	99.9	99.9	98.9	93.1	86.8	77.9	70.4	65.4	58.7	50.0	34.9	19.5	10.6	5.4
**Number**	527	527	526	526	517	494	459	422	391	365	334	295	217	138	74	41

Table B6K-9 Age Group = 71-80 Gender = OVERALL Occupational group = OVERALL Total cases = 271

Threshold dB HL≥	0	5	10	15	20	25	30	35	40	45	50	55	65	75	85	95
**Prevalence**	**100.0**	**100.0**	**100.0**	**99.4**	**97.4**	**95.5**	**89.5**	**87.6**	**85.5**	**79.3**	**74.9**	**66.1**	**51.3**	**30.1**	**15.1**	**6.0**
**Lower CI**	98.6	98.6	98.6	97.2	92.4	90.4	82.0	80.2	78.1	70.6	66.2	58.4	43.7	24.6	11.3	3.8
**Upper CI**	100.0	100.0	100.0	99.9	99.1	98.0	94.1	92.6	90.7	85.9	82.0	73.1	58.8	36.1	19.8	9.3
**Number**	271	271	271	269	267	263	256	252	245	236	226	209	174	113	64	30

Table B6K-10 Age Group = 18-80 Gender = OVERALL Occupational group = NON-MANUAL Total cases = 1184

Threshold dB HL≥	0	5	10	15	20	25	30	35	40	45	50	55	65	75	85	95
**Prevalence**	**97.8**	**95.1**	**85.9**	**73.9**	**61.8**	**48.8**	**39.1**	**32.7**	**27.2**	**23.7**	**20.2**	**16.3**	**11.3**	**6.1**	**2.5**	**1.4**
**Lower CI**	96.4	92.9	83.0	71.0	58.7	46.0	36.8	30.5	25.3	21.8	18.3	14.9	10.2	5.1	1.9	1.0
**Upper CI**	98.6	96.6	88.3	76.6	64.7	51.7	41.5	34.9	29.3	25.9	22.2	17.7	12.6	7.3	3.2	2.0
**Number**	1168	1149	1082	973	855	720	613	525	449	392	342	294	208	122	65	39

Table B6K-11 Age Group = 18-80 Gender = OVERALL Occupational group = MANUAL Total cases = 1472

Threshold dB HL≥	0	5	10	15	20	25	30	35	40	45	50	55	65	75	85	95
**Prevalence**	**97.7**	**95.4**	**89.8**	**79.9**	**68.9**	**56.6**	**44.7**	**37.7**	**31.5**	**27.4**	**24.8**	**19.8**	**12.6**	**7.2**	**4.0**	**1.9**
**Lower CI**	96.2	93.4	87.2	76.7	65.7	53.6	41.9	35.2	29.4	25.4	22.8	18.1	11.3	6.2	3.2	1.4
**Upper CI**	98.6	96.9	92.0	82.7	72.0	59.5	47.5	40.3	33.8	29.6	27.0	21.7	14.0	8.3	4.9	2.5
**Number**	1453	1436	1385	1286	1173	1031	888	786	696	621	564	493	363	238	134	71

Table B6K-12 Age Group = 18-30 Gender = FEMALE Occupational group = OVERALL Total cases = 224

Threshold dB HL≥	0	5	10	15	20	25	30	35	40	45	50	55	65	75	85	95
**Prevalence**	**94.8**	**88.3**	**72.4**	**48.4**	**30.3**	**19.0**	**8.2**	**5.1**	**2.1**	**1.9**	**1.7**	**0.7**	**0.0**	**0.0**	**0.0**	**0.0**
**Lower CI**	90.7	82.8	65.2	41.2	23.9	13.9	4.9	2.6	0.9	0.7	0.6	0.1	0.0	0.0	0.0	0.0
**Upper CI**	97.1	92.2	78.6	55.7	37.5	25.4	13.2	9.7	4.8	4.6	4.6	3.0	1.7	1.7	1.7	1.7
**Number**	213	200	168	113	74	47	23	13	8	6	5	2	0	0	0	0

Table B6K-13 Age Group = 31-40 Gender = FEMALE Occupational group = OVERALL Total cases = 189

Threshold dB HL≥	0	5	10	15	20	25	30	35	40	45	50	55	65	75	85	95
**Prevalence**	**96.7**	**95.4**	**82.6**	**62.2**	**45.5**	**30.7**	**18.4**	**11.9**	**6.2**	**3.4**	**3.1**	**2.2**	**1.5**	**1.4**	**0.0**	**0.0**
**Lower CI**	92.6	90.9	75.0	53.7	37.5	23.7	13.0	7.8	3.7	1.8	1.5	0.9	0.6	0.5	0.0	0.0
**Upper CI**	98.6	97.8	88.3	70.0	53.8	38.8	25.4	17.8	10.1	6.5	6.1	5.3	4.0	4.0	2.0	2.0
**Number**	183	180	161	128	96	65	45	32	20	12	10	7	6	5	0	0

Table B6K-14 Age Group = 41-50 Gender = FEMALE Occupational group = OVERALL Total cases = 247

Threshold dB HL≥	0	5	10	15	20	25	30	35	40	45	50	55	65	75	85	95
**Prevalence**	**99.4**	**98.1**	**88.3**	**76.4**	**56.1**	**40.8**	**27.4**	**20.8**	**14.7**	**12.4**	**10.1**	**7.2**	**2.5**	**1.2**	**0.5**	**0.3**
**Lower CI**	96.5	94.9	82.8	70.0	49.5	34.5	22.1	16.4	10.9	9.0	7.1	4.9	1.4	0.5	0.2	0.1
**Upper CI**	99.9	99.3	92.2	81.8	62.5	47.5	33.3	26.1	19.5	17.0	14.1	10.6	4.4	2.7	1.4	1.0
**Number**	246	243	224	193	152	116	86	69	50	42	36	28	12	6	3	2

Prevalence by age group, gender and occupational group for the better ear over 6 kHz

Table B6K-15 Age Group = 51-60 Gender = FEMALE Occupational group = OVERALL Total cases = 340

Threshold dB HL≥	0	5	10	15	20	25	30	35	40	45	50	55	65	75	85	95
**Prevalence**	**99.6**	**99.6**	**96.3**	**89.9**	**79.2**	**65.4**	**50.1**	**38.1**	**25.4**	**19.7**	**14.0**	**10.7**	**5.7**	**3.6**	**2.2**	**1.2**
**Lower CI**	97.6	97.6	93.2	85.6	73.9	59.4	44.2	32.6	20.8	15.6	10.6	7.9	3.8	2.3	1.3	0.6
**Upper CI**	99.9	99.9	98.0	93.1	83.7	70.8	56.0	43.8	30.6	24.6	18.2	14.5	8.3	5.7	3.6	2.5
**Number**	339	339	330	313	282	240	192	155	115	89	70	57	34	24	17	8

Table B6K-16 Age Group = 61-70 Gender = FEMALE Occupational group = OVERALL Total cases = 243

Threshold dB HL≥	0	5	10	15	20	25	30	35	40	45	50	55	65	75	85	95
**Prevalence**	**100.0**	**100.0**	**99.3**	**99.3**	**96.0**	**83.7**	**75.2**	**63.8**	**55.4**	**50.5**	**44.2**	**34.7**	**19.4**	**9.6**	**4.5**	**2.7**
**Lower CI**	98.4	98.4	96.1	96.1	91.6	77.1	68.2	56.5	48.2	43.5	37.5	28.8	14.5	6.7	2.8	1.4
**Upper CI**	100.0	100.0	99.9	99.9	98.1	88.7	81.1	70.4	62.3	57.5	51.2	41.2	25.6	13.5	7.0	5.2
**Number**	243	243	242	242	235	214	193	171	151	138	123	100	68	41	23	13

Table B6K-17 Age Group = 71-80 Gender = FEMALE Occupational group = OVERALL Total cases = 143

Threshold dB HL≥	0	5	10	15	20	25	30	35	40	45	50	55	65	75	85	95
**Prevalence**	**100.0**	**100.0**	**100.0**	**99.8**	**97.9**	**95.2**	**86.2**	**86.0**	**84.0**	**77.1**	**72.5**	**63.8**	**47.1**	**25.5**	**10.3**	**3.8**
**Lower CI**	97.4	97.4	97.4	98.9	89.9	87.3	74.3	74.2	72.4	64.1	59.6	53.2	37.0	19.3	6.7	2.3
**Upper CI**	100.0	100.0	100.0	100.0	99.6	98.3	93.1	93.0	91.3	86.4	82.5	73.2	57.4	32.8	15.5	6.4
**Number**	143	143	143	142	141	138	132	131	128	122	116	108	90	57	28	14

Table B6K-18 Age Group = 18-30 Gender = MALE Occupational group = OVERALL Total cases = 177

Threshold dB HL≥	0	5	10	15	20	25	30	35	40	45	50	55	65	75	85	95
**Prevalence**	**92.1**	**84.9**	**67.2**	**51.2**	**37.6**	**18.5**	**8.7**	**6.4**	**2.6**	**2.6**	**2.3**	**0.6**	**0.6**	**0.6**	**0.6**	**0.6**
**Lower CI**	86.7	77.7	58.0	42.4	29.5	12.8	5.0	3.2	0.8	0.8	0.6	0.1	0.1	0.1	0.1	0.1
**Upper CI**	95.4	90.1	75.3	60.0	46.5	25.9	14.9	12.4	7.8	7.8	7.7	3.0	3.0	3.0	3.1	3.1
**Number**	163	153	127	98	69	37	21	14	7	7	5	3	3	3	2	2

Table B6K-19 Age Group = 31-40 Gender = MALE Occupational group = OVERALL Total cases = 142

Threshold dB HL≥	0	5	10	15	20	25	30	35	40	45	50	55	65	75	85	95
**Prevalence**	**98.5**	**95.0**	**90.5**	**79.5**	**62.3**	**41.6**	**27.2**	**21.4**	**14.6**	**10.9**	**9.5**	**5.4**	**2.8**	**2.3**	**1.3**	**1.2**
**Lower CI**	93.7	87.2	81.8	69.5	51.6	31.7	19.1	14.1	8.8	6.0	4.9	2.6	1.0	0.8	0.3	0.2
**Upper CI**	99.7	98.2	95.3	86.8	71.9	52.3	37.2	31.0	23.3	19.0	17.9	10.9	7.2	6.7	6.4	6.5
**Number**	140	136	130	113	93	64	44	35	24	17	14	11	6	5	2	1

Table B6K-20 Age Group = 41-50 Gender = MALE Occupational group = OVERALL Total cases = 203

Threshold dB HL≥	0	5	10	15	20	25	30	35	40	45	50	55	65	75	85	95
**Prevalence**	**100.0**	**98.7**	**96.8**	**87.5**	**79.6**	**62.1**	**47.1**	**35.6**	**28.9**	**20.4**	**14.9**	**11.8**	**8.0**	**3.8**	**2.6**	**1.1**
**Lower CI**	98.1	94.6	92.5	81.2	72.8	54.4	39.7	28.8	22.7	15.3	10.6	8.0	5.2	2.0	1.1	0.6
**Upper CI**	100.0	99.7	98.7	91.9	85.1	69.3	54.5	43.0	35.9	26.7	20.5	16.9	12.3	7.0	5.8	2.3
**Number**	203	201	198	183	167	135	107	85	73	56	42	35	28	16	11	7

Table B6K-21 Age Group = 51-60 Gender = MALE Occupational group = OVERALL Total cases = 340

Threshold dB HL≥	0	5	10	15	20	25	30	35	40	45	50	55	65	75	85	95
**Prevalence**	**100.0**	**99.8**	**98.3**	**94.7**	**90.2**	**81.5**	**73.5**	**62.2**	**55.9**	**46.5**	**40.6**	**33.9**	**20.4**	**10.6**	**5.5**	**3.5**
**Lower CI**	98.9	98.8	95.8	91.0	85.7	76.2	67.8	56.1	49.9	40.8	35.2	28.8	16.5	7.9	3.6	2.2
**Upper CI**	100.0	100.0	99.3	96.9	93.4	85.8	78.5	67.8	61.7	52.3	46.3	39.4	25.0	14.1	8.5	5.7
**Number**	340	339	335	326	314	292	270	236	214	185	166	142	93	50	26	19

Prevalence by age group, gender and occupational group for the better ear over 6 kHz

Table B6K-22 Age Group = 61-70 Gender = MALE Occupational group = OVERALL Total cases = 284

Threshold dB HL≥	0	5	10	15	20	25	30	35	40	45	50	55	65	75	85	95
**Prevalence**	**100.0**	**100.0**	**100.0**	**100.0**	**99.7**	**97.5**	**92.1**	**85.1**	**77.5**	**72.1**	**64.8**	**57.2**	**43.2**	**24.2**	**12.3**	**5.2**
Lower CI	98.7	98.7	98.7	98.7	99.1	91.4	86.3	78.9	70.1	64.5	57.4	49.8	36.4	19.3	8.6	3.7
Upper CI	100.0	100.0	100.0	100.0	99.9	99.3	95.6	89.7	83.6	78.6	71.5	64.4	50.3	29.9	17.3	7.5
Number	284	284	284	284	282	280	266	251	240	227	211	195	149	97	51	28

Table B6K-23 Age Group = 71-80 Gender = MALE Occupational group = OVERALL Total cases = 128

Threshold dB HL≥	0	5	10	15	20	25	30	35	40	45	50	55	65	75	85	95
**Prevalence**	**100.0**	**100.0**	**100.0**	**98.7**	**96.7**	**95.9**	**94.0**	**89.4**	**87.0**	**81.8**	**78.0**	**69.5**	**57.3**	**36.9**	**22.5**	**9.2**
Lower CI	97.1	97.1	97.1	92.7	88.4	87.8	85.5	80.6	77.9	71.2	67.3	58.0	46.2	27.6	15.1	4.8
Upper CI	100.0	100.0	100.0	99.8	99.1	98.7	97.6	94.5	92.6	89.1	85.9	79.0	67.7	47.3	32.2	16.8
Number	128	128	128	127	126	125	124	121	117	114	110	101	84	56	36	16

Table B6K-24 Age Group = 18-30 Gender = OVERALL Occupational group = NON-MANUAL Total cases = 181

Threshold dB HL≥	0	5	10	15	20	25	30	35	40	45	50	55	65	75	85	95
**Prevalence**	**93.8**	**85.8**	**63.6**	**40.0**	**27.3**	**15.3**	**4.8**	**3.0**	**0.8**	**0.8**	**0.5**	**0.5**	**0.1**	**0.1**	**0.0**	**0.0**
Lower CI	89.2	78.6	54.6	32.3	20.4	10.3	2.6	1.4	0.3	0.3	0.1	0.1	0.0	0.0	0.0	0.0
Upper CI	96.5	90.9	71.6	48.3	35.4	22.2	8.4	6.2	2.2	2.2	2.3	2.3	2.2	2.2	2.1	2.1
Number	170	158	126	83	57	33	14	7	3	3	2	2	1	1	0	0

Table B6K-25 Age Group = 31-40 Gender = OVERALL Occupational group = NON-MANUAL Total cases = 148

Threshold dB HL≥	0	5	10	15	20	25	30	35	40	45	50	55	65	75	85	95
**Prevalence**	**97.6**	**96.3**	**85.4**	**69.3**	**48.2**	**31.4**	**21.5**	**14.7**	**9.8**	**7.5**	**7.5**	**1.9**	**1.4**	**1.4**	**0.0**	**0.0**
Lower CI	93.6	91.9	78.4	61.1	39.2	23.1	14.4	8.9	5.2	3.6	3.6	0.7	0.4	0.4	0.0	0.0
Upper CI	99.1	98.3	90.4	76.5	57.3	41.1	30.8	23.5	17.6	15.0	15.0	4.7	4.6	4.6	2.5	2.5
Number	144	142	126	99	71	47	31	22	14	10	10	5	3	3	0	0

Table B6K-26 Age Group = 41-50 Gender = OVERALL Occupational group = NON-MANUAL Total cases = 188

Threshold dB HL≥	0	5	10	15	20	25	30	35	40	45	50	55	65	75	85	95
**Prevalence**	**99.4**	**97.8**	**91.6**	**81.6**	**63.9**	**45.5**	**31.5**	**23.2**	**17.1**	**13.9**	**9.1**	**6.0**	**3.8**	**2.0**	**0.7**	**0.4**
Lower CI	96.7	94.4	86.8	75.0	57.0	38.7	26.0	18.4	13.0	10.3	5.8	4.1	2.7	1.5	0.3	0.1
Upper CI	99.9	99.2	94.8	86.7	70.3	52.4	37.6	28.7	22.0	18.4	13.8	8.9	5.3	2.7	1.7	1.3
Number	187	183	171	152	122	91	67	51	37	30	22	17	12	7	4	2

Table B6K-27 Age Group = 51-60 Gender = OVERALL Occupational group = NON-MANUAL Total cases = 317

Threshold dB HL≥	0	5	10	15	20	25	30	35	40	45	50	55	65	75	85	95
**Prevalence**	**100.0**	**99.7**	**97.3**	**90.2**	**79.8**	**66.5**	**55.8**	**46.0**	**34.9**	**27.4**	**21.3**	**16.5**	**11.0**	**5.0**	**1.7**	**1.1**
Lower CI	98.8	98.4	94.5	85.5	74.4	60.7	49.9	40.3	29.7	22.8	17.4	13.2	8.5	2.5	0.9	0.5
Upper CI	100.0	99.9	98.6	93.4	84.3	71.9	61.5	51.8	40.4	32.7	25.8	20.4	14.1	9.7	3.1	2.1
Number	317	316	309	291	262	222	190	158	126	98	80	65	43	21	11	8

Table B6K-28 Age Group = 61-70 Gender = OVERALL Occupational group = NON-MANUAL Total cases = 228

Threshold dB HL≥	0	5	10	15	20	25	30	35	40	45	50	55	65	75	85	95
**Prevalence**	**100.0**	**100.0**	**100.0**	**100.0**	**98.7**	**87.8**	**81.9**	**69.9**	**63.0**	**58.5**	**49.3**	**42.2**	**25.7**	**15.4**	**6.7**	**4.1**
Lower CI	98.3	98.3	98.3	98.3	95.6	79.4	73.6	61.6	54.7	50.2	42.7	36.2	21.4	11.8	4.6	2.6
Upper CI	100.0	100.0	100.0	100.0	99.6	93.1	88.1	77.0	70.6	66.4	56.0	48.5	30.6	19.8	9.5	6.4
Number	228	228	228	228	225	211	198	176	161	149	132	116	78	50	27	16

Prevalence by age group, gender and occupational group for the better ear over 6 kHz

Table B6K-29 Age Group = 71-80 Gender = OVERALL Occupational group = NON-MANUAL Total cases = 122

	Threshold dB HL≥															
	0	5	10	15	20	25	30	35	40	45	50	55	65	75	85	95
Prevalence	100.0	100.0	100.0	99.0	95.7	94.2	90.8	88.9	87.1	79.1	74.3	67.9	55.9	28.5	12.5	7.1
Lower CI	96.9	96.9	96.9	95.8	87.3	86.0	82.0	80.6	78.5	66.7	61.9	59.0	46.9	21.3	8.1	4.2
Upper CI	100.0	100.0	100.0	99.8	98.6	97.7	95.5	93.9	92.5	87.8	83.7	75.7	64.6	37.1	19.0	11.9
Number	122	122	122	120	118	116	113	111	108	102	96	89	71	40	23	13

Table B6K-30 Age Group = 18-30 Gender = OVERALL Occupational group = MANUAL Total cases = 218

	Threshold dB HL≥															
	0	5	10	15	20	25	30	35	40	45	50	55	65	75	85	95
Prevalence	93.2	87.5	76.5	58.9	38.4	22.9	11.9	7.8	3.8	3.6	3.3	0.6	0.5	0.5	0.5	0.5
Lower CI	88.3	81.1	68.4	50.1	30.3	16.5	7.1	4.1	1.6	1.4	1.2	0.1	0.1	0.1	0.1	0.1
Upper CI	96.2	92.0	83.0	67.2	47.2	30.9	19.3	14.5	9.1	9.0	8.9	2.6	2.6	2.6	2.6	2.6
Number	204	193	168	127	85	51	30	20	12	10	8	3	2	2	2	2

Table B6K-31 Age Group = 31-40 Gender = OVERALL Occupational group = MANUAL Total cases = 183

	Threshold dB HL≥															
	0	5	10	15	20	25	30	35	40	45	50	55	65	75	85	95
Prevalence	97.9	94.5	86.6	69.9	57.2	38.9	24.0	17.6	10.6	6.9	5.9	5.7	2.5	2.0	1.0	0.9
Lower CI	93.9	87.5	78.2	60.1	47.6	30.2	17.0	11.8	6.4	3.8	3.0	2.8	1.1	0.7	0.2	0.2
Upper CI	99.3	97.7	92.1	78.1	66.3	48.4	32.6	25.4	16.8	12.0	11.2	11.0	5.4	5.2	4.9	5.0
Number	179	174	165	142	118	82	58	45	30	19	14	13	9	7	2	1

Table B6K-32 Age Group = 41-50 Gender = OVERALL Occupational group = MANUAL Total cases = 262

	Threshold dB HL≥															
	0	5	10	15	20	25	30	35	40	45	50	55	65	75	85	95
Prevalence	100.0	99.0	94.1	82.6	70.4	54.7	41.0	32.1	24.8	18.3	15.4	12.0	5.7	2.9	1.9	0.7
Lower CI	98.6	94.6	89.2	76.0	63.4	47.4	34.4	26.0	19.4	13.8	11.3	8.4	3.6	1.4	0.7	0.4
Upper CI	100.0	99.8	96.9	87.7	76.5	61.7	47.9	38.8	31.1	23.8	20.7	17.0	8.9	5.8	5.2	1.4
Number	262	261	251	224	197	160	126	103	86	68	56	46	28	15	10	7

Table B6K-33 Age Group = 51-60 Gender = OVERALL Occupational group = MANUAL Total cases = 363

	Threshold dB HL≥															
	0	5	10	15	20	25	30	35	40	45	50	55	65	75	85	95
Prevalence	99.6	99.6	97.8	94.4	89.1	79.4	66.4	53.8	45.1	37.6	32.2	27.2	14.6	8.3	4.5	2.6
Lower CI	98.0	98.0	95.2	90.7	84.4	73.7	60.1	47.6	39.4	32.3	27.3	22.7	11.4	6.1	3.0	1.6
Upper CI	99.9	99.9	99.0	96.7	92.5	84.2	72.1	59.8	51.0	43.2	37.6	32.4	18.5	11.4	6.7	4.2
Number	362	362	356	348	334	310	272	233	203	176	156	134	84	53	32	19

Table B6K-34 Age Group = 61-70 Gender = OVERALL Occupational group = MANUAL Total cases = 298

	Threshold dB HL≥															
	0	5	10	15	20	25	30	35	40	45	50	55	65	75	85	95
Prevalence	100.0	100.0	99.3	99.3	96.6	90.7	82.2	75.0	66.6	61.3	56.6	46.4	32.2	16.3	8.4	3.9
Lower CI	98.7	98.7	96.0	96.0	92.1	84.9	75.9	68.6	59.7	54.6	49.9	39.7	26.4	12.9	5.9	2.8
Upper CI	100.0	100.0	99.9	99.9	98.5	94.5	87.2	80.5	72.9	67.5	63.1	53.1	38.6	20.4	11.7	5.3
Number	298	298	297	297	291	282	260	245	229	215	201	178	138	88	47	25

Table B6K-35 Age Group = 71-80 Gender = OVERALL Occupational group = MANUAL Total cases = 148

	Threshold dB HL≥															
	0	5	10	15	20	25	30	35	40	45	50	55	65	75	85	95
Prevalence	100.0	100.0	100.0	100.0	100.0	97.4	89.4	88.2	85.4	81.6	79.1	65.3	45.7	28.1	16.7	4.8
Lower CI	97.5	97.5	97.5	97.5	97.5	89.6	76.7	75.6	73.9	68.8	66.5	55.1	37.2	21.6	11.1	2.9
Upper CI	100.0	100.0	100.0	100.0	100.0	99.4	95.6	94.7	92.4	89.9	87.9	74.2	54.6	35.8	24.3	7.9
Number	148	148	148	148	148	146	142	140	136	133	129	119	102	73	41	17

Prevalence by age group, gender and occupational group for the better ear over 6 kHz

Table B6K-36 Age Group = 18-80 Gender = FEMALE Occupational group = NON-MANUAL Total cases = 671

	Threshold dB HL≥															
	**0**	**5**	**10**	**15**	**20**	**25**	**30**	**35**	**40**	**45**	**50**	**55**	**65**	**75**	**85**	**95**
**Prevalence**	**98.0**	**95.1**	**85.0**	**72.0**	**59.2**	**46.5**	**35.7**	**29.4**	**24.1**	**21.0**	**17.7**	**14.7**	**10.5**	**5.3**	**2.1**	**1.1**
**Lower CI**	96.1	92.7	81.6	68.4	55.8	43.2	32.8	26.8	21.9	18.5	15.3	12.8	8.9	4.1	1.3	0.6
**Upper CI**	98.9	96.8	87.9	75.3	62.6	49.9	38.7	32.2	26.5	23.7	20.4	16.8	12.2	6.9	3.2	1.9
**Number**	662	648	599	527	454	374	305	258	217	186	158	134	98	60	33	19

Table B6K-37 Age Group = 18-80 Gender = MALE Occupational group = NON-MANUAL Total cases = 513

	Threshold dB HL≥															
	**0**	**5**	**10**	**15**	**20**	**25**	**30**	**35**	**40**	**45**	**50**	**55**	**65**	**75**	**85**	**95**
**Prevalence**	**97.6**	**95.0**	**87.0**	**76.2**	**64.6**	**51.3**	**42.8**	**36.3**	**30.7**	**26.8**	**23.0**	**18.0**	**12.3**	**7.0**	**3.0**	**1.8**
**Lower CI**	95.1	91.0	82.0	71.4	59.5	46.6	39.1	32.8	27.4	23.7	20.1	16.1	10.7	5.5	2.2	1.2
**Upper CI**	98.8	97.3	90.7	80.4	69.4	56.1	46.6	39.9	34.2	30.2	26.1	20.0	14.1	9.0	4.1	2.7
**Number**	506	501	483	446	401	346	308	267	232	206	184	160	110	62	32	20

Table B6K-38 Age Group = 18-80 Gender = FEMALE Occupational group = MANUAL Total cases = 714

	Threshold dB HL≥															
	**0**	**5**	**10**	**15**	**20**	**25**	**30**	**35**	**40**	**45**	**50**	**55**	**65**	**75**	**85**	**95**
**Prevalence**	**97.9**	**97.0**	**90.7**	**78.5**	**64.3**	**52.6**	**39.9**	**32.1**	**24.7**	**21.1**	**19.0**	**14.2**	**7.2**	**4.3**	**2.0**	**1.1**
**Lower CI**	95.8	94.6	87.2	74.1	60.0	48.4	35.9	28.7	21.9	18.4	16.4	12.1	5.8	3.5	1.5	0.7
**Upper CI**	99.0	98.3	93.4	82.3	68.4	56.8	44.0	35.8	27.8	24.2	22.0	16.7	8.8	5.3	2.7	1.6
**Number**	704	699	668	603	525	445	365	312	254	222	201	167	111	73	38	18

Table B6K-39 Age Group = 18-80 Gender = MALE Occupational group = MANUAL Total cases = 758

	Threshold dB HL≥															
	**0**	**5**	**10**	**15**	**20**	**25**	**30**	**35**	**40**	**45**	**50**	**55**	**65**	**75**	**85**	**95**
**Prevalence**	**97.5**	**93.7**	**88.8**	**81.4**	**74.1**	**61.0**	**50.2**	**44.0**	**39.1**	**34.4**	**31.3**	**26.1**	**18.6**	**10.4**	**6.2**	**2.7**
**Lower CI**	95.1	89.9	84.4	76.5	69.3	56.7	46.4	40.3	35.9	31.4	28.3	23.4	16.4	8.6	4.7	1.9
**Upper CI**	98.7	96.1	92.1	85.4	78.5	65.1	54.0	47.7	42.5	37.6	34.5	29.0	21.1	12.5	8.1	3.9
**Number**	749	737	717	683	648	586	523	474	442	399	363	326	252	165	96	53

Table B6K-40 Age Group = 18-30 Gender = FEMALE Occupational group = NON-MANUAL Total cases = 115

	Threshold dB HL≥															
	**0**	**5**	**10**	**15**	**20**	**25**	**30**	**35**	**40**	**45**	**50**	**55**	**65**	**75**	**85**	**95**
**Prevalence**	**95.2**	**87.5**	**67.9**	**42.1**	**28.8**	**15.8**	**5.7**	**4.5**	**1.4**	**1.4**	**0.9**	**0.9**	**0.0**	**0.0**	**0.0**	**0.0**
**Lower CI**	89.2	79.7	58.2	33.3	21.3	10.4	2.9	2.0	0.4	0.4	0.2	0.2	0.0	0.0	0.0	0.0
**Upper CI**	97.9	92.5	76.2	51.4	37.7	23.4	11.0	10.0	4.3	4.3	4.7	4.7	3.2	3.2	3.2	3.2
**Number**	110	101	82	52	37	22	9	5	2	2	1	1	0	0	0	0

Table B6K-41 Age Group = 31-40 Gender = FEMALE Occupational group = NON-MANUAL Total cases = 86

	Threshold dB HL≥															
	**0**	**5**	**10**	**15**	**20**	**25**	**30**	**35**	**40**	**45**	**50**	**55**	**65**	**75**	**85**	**95**
**Prevalence**	**96.8**	**94.3**	**78.4**	**58.6**	**41.4**	**26.5**	**13.9**	**8.7**	**5.3**	**3.4**	**3.4**	**1.4**	**1.4**	**1.4**	**0.0**	**0.0**
**Lower CI**	90.2	86.8	67.7	47.3	31.8	18.2	7.9	4.5	2.5	1.5	1.5	0.3	0.3	0.3	0.0	0.0
**Upper CI**	99.0	97.6	86.3	69.0	51.7	36.9	23.4	16.3	10.5	7.5	7.5	6.5	6.5	6.5	4.3	4.3
**Number**	83	81	68	51	36	23	14	10	7	5	5	2	2	2	0	0

Table B6K-42 Age Group = 41-50 Gender = FEMALE Occupational group = NON-MANUAL Total cases = 112

	Threshold dB HL≥															
	**0**	**5**	**10**	**15**	**20**	**25**	**30**	**35**	**40**	**45**	**50**	**55**	**65**	**75**	**85**	**95**
**Prevalence**	**98.9**	**96.3**	**84.5**	**74.0**	**51.6**	**37.6**	**24.9**	**16.0**	**10.2**	**9.3**	**8.2**	**6.3**	**2.1**	**1.3**	**0.4**	**0.0**
**Lower CI**	94.0	89.9	75.7	64.8	42.2	28.9	18.1	11.0	6.3	5.4	4.6	3.3	1.1	0.8	0.1	0.0
**Upper CI**	99.8	98.7	90.5	81.5	60.8	47.2	33.2	22.6	16.3	15.5	14.1	11.6	3.9	2.4	2.2	3.3
**Number**	111	108	96	83	61	46	34	24	17	14	12	9	4	2	1	0

Prevalence by age group, gender and occupational group for the better ear over 6 kHz

Table B6K-43 Age Group = 51-60 Gender = FEMALE Occupational group = NON-MANUAL Total cases = 175

	Threshold dB HL≥															
	0	5	10	15	20	25	30	35	40	45	50	55	65	75	85	95
Prevalence	100.0	100.0	96.9	89.3	75.7	62.1	46.2	37.0	24.1	18.3	11.3	9.3	5.8	3.8	1.8	1.3
Lower CI	97.9	97.9	92.7	82.7	67.9	53.9	38.2	29.6	18.1	13.0	7.5	5.9	3.5	2.0	0.9	0.6
Upper CI	100.0	100.0	98.7	93.6	82.1	69.7	54.3	44.9	31.3	25.0	16.7	14.3	9.3	7.0	3.7	3.0
Number	175	175	170	159	140	116	90	76	57	42	30	25	17	11	7	5

Table B6K-44 Age Group = 61-70 Gender = FEMALE Occupational group = NON-MANUAL Total cases = 116

	Threshold dB HL≥															
	0	5	10	15	20	25	30	35	40	45	50	55	65	75	85	95
Prevalence	100.0	100.0	100.0	100.0	98.5	83.8	77.6	63.1	57.1	51.6	43.7	35.1	19.0	10.6	5.6	3.5
Lower CI	96.8	96.8	96.8	96.8	92.0	73.6	67.4	52.8	47.0	41.7	34.1	27.0	12.9	6.3	2.9	1.3
Upper CI	100.0	100.0	100.0	100.0	99.7	90.6	85.3	72.3	66.6	61.4	53.7	44.2	27.2	17.2	10.6	8.7
Number	116	116	116	116	115	103	96	81	73	65	57	47	32	22	14	8

Table B6K-45 Age Group = 71-80 Gender = FEMALE Occupational group = NON-MANUAL Total cases = 67

	Threshold dB HL≥															
	0	5	10	15	20	25	30	35	40	45	50	55	65	75	85	95
Prevalence	100.0	100.0	100.0	99.7	96.1	94.7	90.7	90.7	90.3	82.0	75.3	68.5	62.2	27.2	8.3	3.8
Lower CI	94.6	94.6	94.6	98.1	82.3	81.6	76.7	76.7	76.6	62.2	56.2	56.0	50.1	17.8	3.8	1.6
Upper CI	100.0	100.0	100.0	99.9	99.2	98.6	96.6	96.6	96.4	92.7	87.9	78.8	72.9	39.3	17.1	8.7
Number	67	67	67	66	65	64	62	62	61	58	53	50	43	23	11	6

Table B6K-46 Age Group = 18-30 Gender = MALE Occupational group = NON-MANUAL Total cases = 66

	Threshold dB HL≥															
	0	5	10	15	20	25	30	35	40	45	50	55	65	75	85	95
Prevalence	92.4	84.3	59.4	38.3	25.6	14.5	3.7	1.4	0.1	0.1	0.1	0.1	0.1	0.1	0.0	0.0
Lower CI	84.4	71.1	44.5	26.2	15.1	7.1	1.3	0.3	0.0	0.0	0.0	0.0	0.0	0.0	0.0	0.0
Upper CI	96.5	92.1	72.7	52.1	40.0	27.1	10.0	6.7	5.7	5.7	5.7	5.7	5.7	5.7	5.5	5.5
Number	60	57	44	31	20	11	5	2	1	1	1	1	1	1	0	0

Table B6K-47 Age Group = 31-40 Gender = MALE Occupational group = NON-MANUAL Total cases = 62

	Threshold dB HL≥															
	0	5	10	15	20	25	30	35	40	45	50	55	65	75	85	95
Prevalence	98.5	98.5	93.5	81.9	56.7	37.8	30.4	21.9	14.9	12.2	12.2	2.3	1.3	1.3	0.0	0.0
Lower CI	92.1	92.1	82.8	69.1	40.9	23.4	17.5	11.0	6.4	4.7	4.7	0.8	0.2	0.2	0.0	0.0
Upper CI	99.7	99.7	97.7	90.1	71.2	54.8	47.2	38.8	30.8	28.1	28.1	6.6	6.9	6.9	5.8	5.8
Number	61	61	58	48	35	24	17	12	7	5	5	3	1	1	0	0

Table B6K-48 Age Group = 41-50 Gender = MALE Occupational group = NON-MANUAL Total cases = 76

	Threshold dB HL≥															
	0	5	10	15	20	25	30	35	40	45	50	55	65	75	85	95
Prevalence	100.0	99.5	99.5	89.8	77.3	53.9	38.5	31.0	24.5	18.9	10.0	5.7	5.7	2.6	1.0	0.7
Lower CI	95.2	97.2	97.2	78.8	66.4	43.7	30.2	23.1	17.7	13.2	5.2	3.8	3.8	1.8	0.4	0.2
Upper CI	100.0	99.9	99.9	95.4	85.4	63.8	47.6	40.2	32.9	26.3	18.5	8.3	8.3	3.7	2.8	2.8
Number	76	75	75	69	61	45	33	27	20	16	10	8	8	5	3	2

Table B6K-49 Age Group = 51-60 Gender = MALE Occupational group = NON-MANUAL Total cases = 142

	Threshold dB HL≥															
	0	5	10	15	20	25	30	35	40	45	50	55	65	75	85	95
Prevalence	100.0	99.4	97.6	91.1	84.5	71.4	66.5	56.1	47.0	37.7	32.5	24.6	17.0	6.3	1.4	0.8
Lower CI	97.4	96.5	92.9	83.7	76.5	62.9	57.9	47.4	38.6	30.0	25.6	19.0	12.6	2.2	0.5	0.2
Upper CI	100.0	99.9	99.2	95.3	90.1	78.7	74.1	64.4	55.5	46.0	40.2	31.1	22.5	16.8	4.1	2.3
Number	142	141	139	132	122	106	100	82	69	56	50	40	26	10	4	3

Prevalence by age group, gender and occupational group for the better ear over 6 kHz

Table B6K-50 Age Group = 61-70 Gender = MALE Occupational group = NON-MANUAL Total cases = 112

	Threshold dB HL≥															
	0	5	10	15	20	25	30	35	40	45	50	55	65	75	85	95
**Prevalence**	**100.0**	**100.0**	**100.0**	**100.0**	**99.0**	**92.2**	**86.8**	**77.9**	**70.4**	**67.1**	**55.8**	**50.1**	**33.4**	**21.2**	**8.3**	**5.1**
**Lower CI**	96.7	96.7	96.7	96.7	97.1	73.8	70.4	63.3	55.9	52.6	47.0	41.5	27.9	15.8	5.6	3.7
**Upper CI**	100.0	100.0	100.0	100.0	99.7	98.0	94.8	87.9	81.7	79.0	64.3	58.7	39.4	27.9	12.0	7.0
**Number**	112	112	112	112	110	108	102	95	88	84	75	69	46	28	13	8

Table B6K-51 Age Group = 71-80 Gender = MALE Occupational group = NON-MANUAL Total cases = 55

	Threshold dB HL≥															
	0	5	10	15	20	25	30	35	40	45	50	55	65	75	85	95
**Prevalence**	**100.0**	**100.0**	**100.0**	**97.9**	**95.0**	**93.4**	**90.5**	**85.5**	**81.4**	**74.2**	**72.1**	**66.6**	**46.7**	**30.2**	**19.0**	**12.2**
**Lower CI**	93.5	93.5	93.5	89.0	82.9	81.3	78.8	75.3	69.4	61.1	59.7	54.2	33.7	20.7	11.2	6.4
**Upper CI**	100.0	100.0	100.0	99.6	98.7	97.9	96.0	91.9	89.4	84.0	81.8	77.0	60.2	41.9	30.3	22.0
**Number**	55	55	55	54	53	52	51	49	47	44	43	39	28	17	12	7

Table B6K-52 Age Group = 18-30 Gender = FEMALE Occupational group = MANUAL Total cases = 109

	Threshold dB HL≥															
	0	5	10	15	20	25	30	35	40	45	50	55	65	75	85	95
**Prevalence**	**94.1**	**90.7**	**80.4**	**59.4**	**34.0**	**24.8**	**12.5**	**6.1**	**2.9**	**2.4**	**2.4**	**0.2**	**0.0**	**0.0**	**0.0**	**0.0**
**Lower CI**	86.4	82.5	69.5	47.2	23.3	15.8	6.1	2.1	1.0	0.6	0.6	0.0	0.0	0.0	0.0	0.0
**Upper CI**	97.6	95.3	88.0	70.6	46.5	36.7	24.2	16.3	8.4	8.5	8.5	1.1	3.4	3.4	3.4	3.4
**Number**	103	99	86	61	37	25	14	8	6	4	4	1	0	0	0	0

Table B6K-53 Age Group = 31-40 Gender = FEMALE Occupational group = MANUAL Total cases = 103

	Threshold dB HL≥															
	0	5	10	15	20	25	30	35	40	45	50	55	65	75	85	95
**Prevalence**	**97.7**	**97.6**	**85.9**	**63.7**	**48.0**	**33.6**	**22.5**	**14.6**	**6.4**	**3.3**	**2.7**	**2.7**	**1.1**	**1.0**	**0.0**	**0.0**
**Lower CI**	92.6	92.6	74.3	51.1	36.4	23.3	14.2	8.4	3.1	1.2	0.9	0.9	0.4	0.3	0.0	0.0
**Upper CI**	99.3	99.2	92.8	74.7	59.8	45.8	33.9	24.2	12.6	8.7	8.4	8.4	2.8	2.7	3.6	3.6
**Number**	100	99	93	77	60	42	31	22	13	7	5	5	4	3	0	0

Table B6K-54 Age Group = 41-50 Gender = FEMALE Occupational group = MANUAL Total cases = 135

	Threshold dB HL≥															
	0	5	10	15	20	25	30	35	40	45	50	55	65	75	85	95
**Prevalence**	**100.0**	**100.0**	**93.4**	**80.5**	**62.3**	**45.3**	**30.2**	**24.5**	**17.2**	**13.8**	**10.6**	**7.1**	**2.9**	**1.2**	**0.4**	**0.4**
**Lower CI**	97.2	97.2	86.3	71.2	52.5	35.9	22.4	17.6	11.7	9.2	6.8	4.3	1.5	0.7	0.1	0.1
**Upper CI**	100.0	100.0	97.0	87.3	71.1	55.0	39.4	32.9	24.7	20.3	16.2	11.5	5.5	2.1	1.4	1.4
**Number**	135	135	128	110	91	70	52	45	33	28	24	19	8	4	2	2

Table B6K-55 Age Group = 51-60 Gender = FEMALE Occupational group = MANUAL Total cases = 165

	Threshold dB HL≥															
	0	5	10	15	20	25	30	35	40	45	50	55	65	75	85	95
**Prevalence**	**99.3**	**99.3**	**97.2**	**91.7**	**83.8**	**69.6**	**55.9**	**41.0**	**27.9**	**21.5**	**16.6**	**12.0**	**4.8**	**2.9**	**2.0**	**0.8**
**Lower CI**	96.3	96.3	93.3	85.5	76.1	60.3	46.4	32.4	20.4	15.2	11.1	7.5	2.6	1.6	1.0	0.2
**Upper CI**	99.9	99.9	98.8	95.5	89.4	77.5	65.0	50.3	36.9	29.6	24.0	18.8	8.9	5.5	3.8	2.9
**Number**	164	164	160	154	142	124	102	79	58	47	40	32	17	13	10	3

Table B6K-56 Age Group = 61-70 Gender = FEMALE Occupational group = MANUAL Total cases = 126

	Threshold dB HL≥															
	0	5	10	15	20	25	30	35	40	45	50	55	65	75	85	95
**Prevalence**	**100.0**	**100.0**	**98.6**	**98.6**	**93.6**	**83.1**	**72.3**	**64.9**	**53.6**	**49.4**	**45.3**	**33.7**	**16.6**	**8.6**	**4.1**	**2.8**
**Lower CI**	97.0	97.0	92.4	92.4	85.7	72.8	61.9	54.4	43.4	39.8	35.7	25.5	10.8	5.6	2.7	1.9
**Upper CI**	100.0	100.0	99.8	99.8	97.3	90.0	80.8	74.0	63.6	59.1	55.2	43.0	24.5	13.0	6.1	4.2
**Number**	126	126	125	125	119	110	96	89	77	72	65	52	35	19	9	5

Prevalence by age group, gender and occupational group for the better ear over 6 kHz

Table B6K-57 Age Group = 71-80 Gender = FEMALE Occupational group = MANUAL Total cases = 76

Threshold dB HL≥	0	5	10	15	20	25	30	35	40	45	50	55	65	75	85	95
**Prevalence**	**100.0**	**100.0**	**100.0**	**100.0**	**100.0**	**95.6**	**82.1**	**81.8**	**77.9**	**71.5**	**70.5**	**59.9**	**32.8**	**20.3**	**9.5**	**4.2**
**Lower CI**	95.2	95.2	95.2	95.2	95.2	82.7	62.5	62.3	60.2	52.1	51.2	44.3	22.6	15.5	6.0	2.1
**Upper CI**	100.0	100.0	100.0	100.0	100.0	99.0	92.6	92.4	89.2	85.3	84.5	73.8	44.8	26.2	14.7	8.4
**Number**	76	76	76	76	76	74	70	69	67	64	63	58	47	34	17	8

Table B6K-58 Age Group = 18-30 Gender = MALE Occupational group = MANUAL Total cases = 109

Threshold dB HL≥	0	5	10	15	20	25	30	35	40	45	50	55	65	75	85	95
**Prevalence**	**92.3**	**84.0**	**72.3**	**58.3**	**42.8**	**20.4**	**11.2**	**9.6**	**4.9**	**4.9**	**4.5**	**1.0**	**1.0**	**1.0**	**1.0**	**1.0**
**Lower CI**	84.6	72.9	59.6	45.5	31.3	12.4	5.4	4.2	1.4	1.4	1.1	0.2	0.2	0.2	0.2	0.2
**Upper CI**	96.3	91.1	82.2	70.0	55.3	31.7	21.9	20.4	16.0	16.0	16.0	5.1	5.1	5.1	5.1	5.1
**Number**	101	94	82	66	48	26	16	12	6	6	4	2	2	2	2	2

Table B6K-59 Age Group = 31-40 Gender = MALE Occupational group = MANUAL Total cases = 80

Threshold dB HL≥	0	5	10	15	20	25	30	35	40	45	50	55	65	75	85	95
**Prevalence**	**98.1**	**91.3**	**87.0**	**76.2**	**67.2**	**44.3**	**25.7**	**20.9**	**15.0**	**10.6**	**9.0**	**8.6**	**3.9**	**3.0**	**2.1**	**1.9**
**Lower CI**	89.8	77.3	73.0	60.2	51.2	30.6	15.4	11.8	7.8	5.3	4.0	3.7	1.5	0.9	0.4	0.3
**Upper CI**	99.7	97.0	94.3	87.1	79.9	58.9	39.8	34.4	26.7	20.0	18.9	18.6	10.2	10.1	9.9	10.2
**Number**	79	75	72	65	58	40	27	23	17	12	9	8	5	4	2	1

Table B6K-60 Age Group = 41-50 Gender = MALE Occupational group = MANUAL Total cases = 127

Threshold dB HL≥	0	5	10	15	20	25	30	35	40	45	50	55	65	75	85	95
**Prevalence**	**100.0**	**97.9**	**94.8**	**84.9**	**79.1**	**64.9**	**52.8**	**40.3**	**33.0**	**23.1**	**20.5**	**17.4**	**8.7**	**4.8**	**3.7**	**1.1**
**Lower CI**	97.1	88.9	86.3	74.6	68.6	53.7	42.1	30.4	24.0	15.8	13.4	10.7	4.8	1.9	1.2	0.5
**Upper CI**	100.0	99.6	98.1	91.5	86.7	74.7	63.2	51.1	43.5	32.6	30.1	26.8	15.3	11.3	10.7	2.3
**Number**	127	126	123	114	106	90	74	58	53	40	32	27	20	11	8	5

Table B6K-61 Age Group = 51-60 Gender = MALE Occupational group = MANUAL Total cases = 198

Threshold dB HL≥	0	5	10	15	20	25	30	35	40	45	50	55	65	75	85	95
**Prevalence**	**100.0**	**100.0**	**98.6**	**97.4**	**95.1**	**90.4**	**78.3**	**68.2**	**64.3**	**55.6**	**49.6**	**44.2**	**25.4**	**14.3**	**7.2**	**4.5**
**Lower CI**	98.1	98.1	93.9	92.6	88.8	83.2	70.2	59.6	55.7	47.3	41.4	36.3	19.4	9.9	4.3	2.7
**Upper CI**	100.0	100.0	99.7	99.1	97.9	94.7	84.7	75.7	72.1	63.6	57.8	52.4	32.4	20.1	11.6	7.5
**Number**	198	198	196	194	192	186	170	154	145	129	116	102	67	40	22	16

Table B6K-62 Age Group = 61-70 Gender = MALE Occupational group = MANUAL Total cases = 172

Threshold dB HL≥	0	5	10	15	20	25	30	35	40	45	50	55	65	75	85	95
**Prevalence**	**100.0**	**100.0**	**100.0**	**100.0**	**100.0**	**100.0**	**93.8**	**86.8**	**81.4**	**74.8**	**69.6**	**60.9**	**50.9**	**26.0**	**13.7**	**5.2**
**Lower CI**	97.8	97.8	97.8	97.8	97.8	97.8	86.8	80.2	72.2	65.7	60.5	50.3	40.4	19.5	8.7	3.3
**Upper CI**	100.0	100.0	100.0	100.0	100.0	100.0	97.2	91.4	88.1	82.2	77.4	70.6	61.3	33.8	21.1	8.0
**Number**	172	172	172	172	172	172	164	156	152	143	136	126	103	69	38	20

Table B6K-63 Age Group = 71-80 Gender = MALE Occupational group = MANUAL Total cases = 72

Threshold dB HL≥	0	5	10	15	20	25	30	35	40	45	50	55	65	75	85	95
**Prevalence**	**100.0**	**100.0**	**100.0**	**100.0**	**100.0**	**100.0**	**100.0**	**97.5**	**96.2**	**96.2**	**91.5**	**73.4**	**65.1**	**39.9**	**27.5**	**5.8**
**Lower CI**	94.9	94.9	94.9	94.9	94.9	94.9	94.9	86.9	86.9	86.9	81.8	64.9	50.4	25.6	15.2	2.8
**Upper CI**	100.0	100.0	100.0	100.0	100.0	100.0	100.0	99.6	99.0	99.0	96.3	80.5	77.4	56.1	44.5	11.4
**Number**	72	72	72	72	72	72	72	71	69	69	66	61	55	39	24	9

Prevalence by age group, gender and occupational group for the better ear over 8 kHz

Table B8K-1 Age Group = 18-80 Gender = OVERALL Occupational group = OVERALL Total cases = 2661

Threshold dB HL≥	0	5	10	15	20	25	30	35	40	45	50	55	65	75	85	95
**Prevalence**	**93.9**	**85.3**	**73.6**	**60.6**	**51.3**	**42.9**	**37.4**	**32.7**	**28.3**	**25.4**	**22.1**	**19.0**	**12.9**	**7.4**	**3.3**	**1.2**
Lower CI	92.4	83.4	71.5	58.6	49.4	41.2	35.9	31.4	27.0	24.1	20.8	17.8	11.8	6.5	2.8	1.0
Upper CI	95.2	87.0	75.6	62.6	53.2	44.5	38.9	34.2	29.7	26.7	23.4	20.3	14.0	8.3	4.0	1.6
Number	2571	2434	2212	1957	1733	1538	1382	1243	1111	1004	901	806	586	355	187	84

Table B8K-2 Age Group = 18-80 Gender = FEMALE Occupational group = OVERALL Total cases = 1386

Threshold dB HL≥	0	5	10	15	20	25	30	35	40	45	50	55	65	75	85	95
**Prevalence**	**94.7**	**85.7**	**72.0**	**57.9**	**47.2**	**39.2**	**33.5**	**29.5**	**24.8**	**21.8**	**18.5**	**15.4**	**10.4**	**6.0**	**2.7**	**1.1**
Lower CI	92.9	83.2	69.3	55.4	44.8	37.2	31.5	27.6	23.0	20.1	16.8	13.7	9.0	4.8	2.1	0.8
Upper CI	96.1	87.8	74.5	60.4	49.5	41.3	35.5	31.4	26.7	23.6	20.4	17.2	12.0	7.3	3.5	1.5
Number	1338	1253	1105	949	804	700	611	545	477	416	361	315	224	137	76	38

Table B8K-3 Age Group = 18-80 Gender = MALE Occupational group = OVERALL Total cases = 1275

Threshold dB HL≥	0	5	10	15	20	25	30	35	40	45	50	55	65	75	85	95
**Prevalence**	**93.0**	**84.9**	**75.3**	**63.5**	**55.9**	**46.9**	**41.7**	**36.4**	**32.3**	**29.4**	**26.1**	**23.1**	**15.7**	**8.9**	**4.0**	**1.4**
Lower CI	90.3	81.7	72.0	60.3	52.8	44.3	39.5	34.4	30.3	27.4	24.2	21.3	14.2	7.7	3.2	1.0
Upper CI	95.0	87.6	78.4	66.5	58.8	49.5	44.0	38.5	34.4	31.4	28.0	25.0	17.4	10.4	5.1	2.0
Number	1233	1181	1107	1008	929	838	771	698	634	588	540	491	362	218	111	46

Table B8K-4 Age Group = 18-30 Gender = OVERALL Occupational group = OVERALL Total cases = 401

Threshold dB HL≥	0	5	10	15	20	25	30	35	40	45	50	55	65	75	85	95
**Prevalence**	**84.8**	**65.8**	**44.1**	**25.7**	**16.4**	**9.0**	**4.7**	**3.0**	**1.5**	**1.3**	**0.7**	**0.7**	**0.6**	**0.6**	**0.3**	**0.0**
Lower CI	80.0	60.1	38.5	21.1	12.6	6.4	3.1	1.9	0.8	0.6	0.3	0.2	0.2	0.2	0.1	0.0
Upper CI	88.6	71.0	49.8	30.9	21.0	12.5	7.0	4.8	2.9	2.7	2.1	2.1	2.0	2.1	1.6	0.1
Number	346	279	198	121	82	53	32	25	13	10	6	5	4	3	2	1

Table B8K-5 Age Group = 31-40 Gender = OVERALL Occupational group = OVERALL Total cases = 331

Threshold dB HL≥	0	5	10	15	20	25	30	35	40	45	50	55	65	75	85	95
**Prevalence**	**90.5**	**77.6**	**62.7**	**41.2**	**28.3**	**16.2**	**13.4**	**8.0**	**6.4**	**5.2**	**4.4**	**3.4**	**1.6**	**1.2**	**0.6**	**0.6**
Lower CI	85.5	71.3	56.1	35.0	22.9	12.2	9.7	5.4	4.0	3.1	2.4	1.7	0.7	0.4	0.1	0.1
Upper CI	93.9	82.7	68.9	47.7	34.5	21.2	18.2	11.9	10.2	8.8	8.0	6.8	3.7	3.5	3.1	3.1
Number	306	270	227	163	115	79	65	42	31	25	19	15	10	5	1	1

Table B8K-6 Age Group = 41-50 Gender = OVERALL Occupational group = OVERALL Total cases = 450

Threshold dB HL≥	0	5	10	15	20	25	30	35	40	45	50	55	65	75	85	95
**Prevalence**	**98.5**	**91.9**	**78.5**	**62.1**	**49.7**	**38.8**	**28.6**	**22.9**	**17.4**	**13.8**	**8.9**	**7.1**	**4.5**	**1.6**	**0.7**	**0.3**
Lower CI	96.8	88.6	74.0	57.1	44.8	34.2	24.4	19.1	14.1	10.9	6.7	5.2	3.1	1.0	0.4	0.1
Upper CI	99.3	94.3	82.4	66.9	54.7	43.7	33.1	27.2	21.2	17.3	11.7	9.6	6.7	2.5	1.4	0.9
Number	444	420	361	298	242	199	155	128	106	84	62	52	35	19	9	4

Table B8K-7 Age Group = 51-60 Gender = OVERALL Occupational group = OVERALL Total cases = 682

Threshold dB HL≥	0	5	10	15	20	25	30	35	40	45	50	55	65	75	85	95
**Prevalence**	**99.2**	**97.6**	**91.3**	**84.6**	**73.6**	**61.7**	**52.7**	**44.1**	**36.7**	**30.1**	**24.8**	**20.6**	**11.8**	**5.7**	**2.5**	**1.1**
Lower CI	98.0	95.8	88.4	81.2	69.5	57.5	48.5	40.1	32.9	26.6	21.6	17.8	9.7	4.3	1.7	0.6
Upper CI	99.7	98.6	93.5	87.6	77.2	65.7	56.8	48.2	40.6	33.8	28.2	23.8	14.2	7.5	3.8	2.0
Number	678	668	634	599	537	470	412	357	306	262	225	196	121	63	33	15

Prevalence by age group, gender and occupational group for the better ear over 8 kHz

Table B8K-8 Age Group = 61-70 Gender = OVERALL Occupational group = OVERALL Total cases = 527

	Threshold dB HL≥															
	**0**	5	**10**	15	**20**	25	**30**	35	**40**	45	**50**	55	**65**	75	**85**	95
Prevalence	**100.0**	**100.0**	**98.4**	**94.7**	**90.2**	**86.2**	**82.6**	**77.9**	**69.5**	**64.1**	**58.9**	**50.2**	**32.4**	**18.2**	**7.5**	**2.7**
Lower CI	99.3	99.3	95.6	91.3	86.2	82.0	78.2	73.2	64.5	59.1	53.8	45.4	28.0	14.8	5.6	2.0
Upper CI	100.0	100.0	99.5	96.8	93.0	89.5	86.3	82.0	74.1	68.9	63.7	55.0	37.0	22.1	9.9	3.7
Number	527	527	522	510	492	475	458	434	404	378	355	316	232	141	72	34

Table B8K-9 Age Group = 71-80 Gender = OVERALL Occupational group = OVERALL Total cases = 270

	Threshold dB HL≥															
	**0**	5	**10**	15	**20**	25	**30**	35	**40**	45	**50**	55	**65**	75	**85**	95
Prevalence	**100.0**	**100.0**	**100.0**	**98.3**	**97.6**	**95.8**	**93.6**	**91.0**	**87.0**	**85.0**	**78.0**	**70.3**	**56.6**	**34.9**	**17.0**	5.2
Lower CI	98.6	98.6	98.6	95.0	93.8	91.5	87.0	83.8	79.0	77.1	69.5	61.6	48.6	28.3	12.8	3.6
Upper CI	100.0	100.0	100.0	99.4	99.1	98.0	97.0	95.2	92.2	90.5	84.7	77.8	64.2	42.1	22.2	7.4
Number	270	270	270	266	265	262	260	257	251	245	234	222	184	124	70	29

Table B8K-10 Age Group = 18-80 Gender = OVERALL Occupational group = NON-MANUAL Total cases = 1183

	Threshold dB HL≥															
	**0**	5	**10**	15	**20**	25	**30**	35	**40**	45	**50**	55	**65**	75	**85**	95
Prevalence	**93.5**	**84.4**	**70.4**	**57.0**	**47.1**	**40.0**	**35.7**	**31.4**	**27.3**	**24.0**	**20.9**	**18.2**	**12.3**	**5.9**	**2.8**	**0.9**
Lower CI	91.1	81.6	67.3	54.1	44.3	37.7	33.5	29.4	25.2	22.0	19.0	16.4	10.9	4.8	2.2	0.6
Upper CI	95.3	86.8	73.2	59.9	49.8	42.3	37.9	33.5	29.5	26.3	23.0	20.2	13.8	7.1	3.7	1.2
Number	1137	1066	942	823	706	626	564	510	454	409	365	317	223	121	67	29

Table B8K-11 Age Group = 18-80 Gender = OVERALL Occupational group = MANUAL Total cases = 1474

	Threshold dB HL≥															
	**0**	5	**10**	15	**20**	25	**30**	35	**40**	45	**50**	55	**65**	75	**85**	95
Prevalence	**94.1**	**85.8**	**76.0**	**63.3**	**55.0**	**44.8**	**38.1**	**33.3**	**28.6**	**25.9**	**22.4**	**19.2**	**12.5**	**8.0**	**3.6**	**1.5**
Lower CI	91.7	82.9	72.8	60.3	52.1	42.3	36.0	31.5	26.9	24.3	20.7	17.6	11.1	6.8	2.9	1.1
Upper CI	95.9	88.3	78.9	66.2	57.9	47.3	40.2	35.2	30.3	27.7	24.1	21.0	14.1	9.2	4.4	2.0
Number	1430	1364	1267	1132	1025	910	816	731	655	593	534	488	363	234	120	55

Table B8K-12 Age Group = 18-30 Gender = FEMALE Occupational group = OVERALL Total cases = 224

	Threshold dB HL≥															
	**0**	5	**10**	15	**20**	25	**30**	35	**40**	45	**50**	55	**65**	75	**85**	95
Prevalence	**87.1**	**69.5**	**43.6**	**25.3**	**15.5**	**9.4**	**5.8**	**3.8**	**1.2**	**1.0**	**0.7**	**0.7**	**0.6**	**0.6**	**0.0**	**0.0**
Lower CI	81.2	62.3	36.4	19.6	11.1	6.3	3.5	2.2	0.5	0.3	0.1	0.1	0.1	0.1	0.0	0.0
Upper CI	91.4	75.8	51.1	32.0	21.2	13.9	9.3	6.8	3.2	3.2	3.0	3.0	3.1	3.1	1.7	1.7
Number	197	160	107	66	45	32	21	16	5	4	2	2	1	1	0	0

Table B8K-13 Age Group = 31-40 Gender = FEMALE Occupational group = OVERALL Total cases = 189

	Threshold dB HL≥															
	**0**	5	**10**	15	**20**	25	**30**	35	**40**	45	**50**	55	**65**	75	**85**	95
Prevalence	**92.1**	**75.3**	**58.0**	**34.1**	**19.4**	**10.8**	**7.9**	**5.2**	**3.6**	**3.0**	**2.4**	**2.4**	**1.7**	**0.9**	**0.0**	**0.0**
Lower CI	86.4	67.2	49.6	27.1	14.1	7.4	5.1	3.0	1.9	1.4	1.0	1.0	0.7	0.2	0.0	0.0
Upper CI	95.5	82.0	65.9	41.8	26.2	15.5	11.9	8.8	6.8	6.1	5.6	5.6	4.2	3.7	2.0	2.0
Number	177	153	124	82	50	35	26	17	13	10	7	7	6	2	0	0

Table B8K-14 Age Group = 41-50 Gender = FEMALE Occupational group = OVERALL Total cases = 247

	Threshold dB HL≥															
	**0**	5	**10**	15	**20**	25	**30**	35	**40**	45	**50**	55	**65**	75	**85**	95
Prevalence	**97.5**	**88.2**	**72.5**	**55.1**	**39.2**	**29.1**	**20.2**	**15.9**	**12.1**	**8.1**	**6.0**	**3.6**	**2.2**	**0.8**	**0.3**	**0.1**
Lower CI	94.2	83.0	66.0	48.3	32.9	23.6	15.7	11.9	8.9	5.6	3.9	2.3	1.2	0.3	0.1	0.0
Upper CI	98.9	92.0	78.1	61.8	45.8	35.3	25.7	20.9	16.3	11.5	9.0	5.7	4.0	2.1	1.0	0.6
Number	242	223	185	148	110	88	66	54	46	33	26	19	11	4	2	1

Prevalence by age group, gender and occupational group for the better ear over 8 kHz

Table B8K-15 Age Group = 51-60 Gender = FEMALE Occupational group = OVERALL Total cases = 341

Threshold dB HL≥	0	5	10	15	20	25	30	35	40	45	50	55	65	75	85	95
Prevalence	**98.5**	**97.1**	**88.8**	**81.0**	**68.4**	**53.4**	**42.9**	**35.0**	**27.8**	**21.0**	**15.6**	**11.9**	**5.9**	**3.4**	**1.7**	**0.9**
Lower CI	96.2	94.3	84.3	75.7	62.5	47.4	37.2	29.6	23.0	16.9	12.1	8.9	4.0	2.0	0.9	0.4
Upper CI	99.4	98.6	92.1	85.4	73.8	59.3	48.8	40.7	33.2	25.9	20.0	15.7	8.7	5.6	3.0	1.9
Number	337	332	308	287	249	206	173	145	122	98	77	64	36	21	13	7

Table B8K-16 Age Group = 61-70 Gender = FEMALE Occupational group = OVERALL Total cases = 243

Threshold dB HL≥	0	5	10	15	20	25	30	35	40	45	50	55	65	75	85	95
Prevalence	**100.0**	**100.0**	**98.4**	**92.0**	**84.2**	**79.1**	**74.3**	**69.4**	**60.1**	**54.9**	**49.1**	**39.4**	**23.4**	**12.9**	**6.2**	**2.6**
Lower CI	98.4	98.4	95.4	86.8	77.9	72.4	67.4	62.3	53.1	48.1	42.5	33.6	18.2	9.1	3.8	1.6
Upper CI	100.0	100.0	99.5	95.3	88.9	84.5	80.2	75.6	66.7	61.5	55.7	45.6	29.4	17.9	10.0	4.0
Number	243	243	239	228	212	202	190	179	161	145	132	112	79	49	26	15

Table B8K-17 Age Group = 71-80 Gender = FEMALE Occupational group = OVERALL Total cases = 142

Threshold dB HL≥	0	5	10	15	20	25	30	35	40	45	50	55	65	75	85	95
Prevalence	**100.0**	**100.0**	**100.0**	**97.1**	**97.1**	**95.6**	**91.8**	**89.9**	**85.0**	**82.1**	**71.8**	**63.6**	**51.4**	**29.7**	**14.4**	**5.1**
Lower CI	97.4	97.4	97.4	91.5	91.5	89.2	80.7	78.1	72.3	69.8	58.8	50.6	40.2	21.5	9.8	3.0
Upper CI	100.0	100.0	100.0	99.1	99.1	98.3	96.8	95.7	92.5	90.1	81.9	74.8	62.4	39.5	20.7	8.6
Number	142	142	142	138	138	137	135	134	130	126	117	111	91	60	35	15

Table B8K-18 Age Group = 18-30 Gender = MALE Occupational group = OVERALL Total cases = 177

Threshold dB HL≥	0	5	10	15	20	25	30	35	40	45	50	55	65	75	85	95
Prevalence	**82.1**	**62.0**	**44.4**	**25.9**	**17.1**	**8.4**	**3.4**	**2.1**	**1.9**	**1.5**	**0.8**	**0.6**	**0.6**	**0.6**	**0.6**	**0.0**
Lower CI	74.0	53.2	35.9	19.1	11.4	4.8	1.7	0.9	0.8	0.6	0.2	0.1	0.1	0.1	0.1	0.0
Upper CI	88.1	70.1	53.2	34.1	24.7	14.4	7.0	4.7	4.4	4.2	3.0	3.0	3.0	3.1	3.1	0.2
Number	149	119	91	55	37	21	11	9	8	6	4	3	3	2	2	1

Table B8K-19 Age Group = 31-40 Gender = MALE Occupational group = OVERALL Total cases = 142

Threshold dB HL≥	0	5	10	15	20	25	30	35	40	45	50	55	65	75	85	95
Prevalence	**88.6**	**79.8**	**67.7**	**48.9**	**38.0**	**22.0**	**19.3**	**11.1**	**9.4**	**7.8**	**6.7**	**4.6**	**1.6**	**1.5**	**1.2**	**1.2**
Lower CI	79.4	69.6	57.0	38.5	28.5	14.8	12.6	6.4	5.1	3.9	3.1	1.7	0.4	0.4	0.2	0.2
Upper CI	94.0	87.2	76.8	59.4	48.6	31.3	28.5	18.5	17.0	14.8	13.9	11.6	6.2	6.2	6.5	6.5
Number	129	117	103	81	65	44	39	25	18	15	12	8	4	3	1	1

Table B8K-20 Age Group = 41-50 Gender = MALE Occupational group = OVERALL Total cases = 203

Threshold dB HL≥	0	5	10	15	20	25	30	35	40	45	50	55	65	75	85	95
Prevalence	**99.7**	**95.9**	**85.1**	**69.8**	**61.3**	**49.5**	**37.7**	**30.6**	**23.2**	**20.1**	**12.0**	**10.9**	**7.2**	**2.5**	**1.2**	**0.6**
Lower CI	98.4	91.1	78.5	62.1	53.6	41.9	30.7	24.1	17.6	14.8	8.2	7.3	4.4	1.5	0.6	0.2
Upper CI	99.9	98.2	89.9	76.5	68.5	57.1	45.3	38.0	29.9	26.7	17.1	15.9	11.4	4.0	2.5	1.8
Number	202	197	176	150	132	111	89	74	60	51	36	33	24	15	7	3

Table B8K-21 Age Group = 51-60 Gender = MALE Occupational group = OVERALL Total cases = 341

Threshold dB HL≥	0	5	10	15	20	25	30	35	40	45	50	55	65	75	85	95
Prevalence	**100.0**	**98.1**	**94.0**	**88.7**	**79.3**	**70.9**	**63.6**	**54.4**	**46.7**	**40.3**	**35.1**	**30.4**	**18.4**	**8.3**	**3.5**	**1.4**
Lower CI	98.9	95.4	90.3	84.0	73.8	65.0	57.5	48.4	40.9	34.7	29.9	25.5	14.6	5.9	2.0	0.6
Upper CI	100.0	99.2	96.4	92.1	83.9	76.2	69.2	60.3	52.6	46.1	40.7	35.8	22.8	11.5	5.9	3.1
Number	341	336	326	312	288	264	239	212	184	164	148	132	85	42	20	8

Prevalence by age group, gender and occupational group for the better ear over 8 kHz

Table B8K-22 Age Group = 61-70 Gender = MALE Occupational group = OVERALL Total cases = 284

Threshold dB HL≥	0	5	10	15	20	25	30	35	40	45	50	55	65	75	85	95
**Prevalence**	**100.0**	**100.0**	**98.4**	**97.7**	**97.0**	**94.4**	**92.2**	**87.7**	**80.3**	**74.8**	**70.3**	**62.9**	**43.0**	**24.4**	**8.9**	**3.0**
Lower CI	98.7	98.7	91.5	91.3	91.1	88.8	86.5	81.3	72.7	66.9	62.4	55.0	35.9	18.9	6.5	1.9
Upper CI	100.0	100.0	99.7	99.4	99.1	97.3	95.6	92.2	86.1	81.4	77.1	70.2	50.5	31.0	12.1	4.5
Number	284	284	283	282	280	273	268	255	243	233	223	204	153	92	46	19

Table B8K-23 Age Group = 71-80 Gender = MALE Occupational group = OVERALL Total cases = 128

Threshold dB HL≥	0	5	10	15	20	25	30	35	40	45	50	55	65	75	85	95
**Prevalence**	**100.0**	**100.0**	**100.0**	**100.0**	**98.1**	**95.8**	**95.8**	**91.9**	**89.3**	**88.4**	**87.2**	**80.5**	**64.4**	**43.4**	**20.8**	**5.3**
Lower CI	97.1	97.1	97.1	97.1	89.7	87.6	87.6	83.8	80.4	79.6	78.3	71.0	53.9	33.1	13.6	3.4
Upper CI	100.0	100.0	100.0	100.0	99.7	98.6	98.6	96.2	94.4	93.8	92.8	87.5	73.7	54.4	30.4	8.1
Number	128	128	128	128	127	125	125	123	121	119	117	111	93	64	35	14

Table B8K-24 Age Group = 18-30 Gender = OVERALL Occupational group = NON-MANUAL Total cases = 181

Threshold dB HL≥	0	5	10	15	20	25	30	35	40	45	50	55	65	75	85	95
**Prevalence**	**83.3**	**64.2**	**38.3**	**21.1**	**12.1**	**5.9**	**3.4**	**1.7**	**0.8**	**0.5**	**0.5**	**0.5**	**0.5**	**0.4**	**0.0**	**0.0**
Lower CI	75.5	56.1	30.6	15.5	7.7	3.4	1.7	0.7	0.3	0.1	0.1	0.1	0.1	0.1	0.0	0.0
Upper CI	89.0	71.6	46.6	28.2	18.4	9.9	6.7	3.8	2.2	2.3	2.3	2.3	2.3	2.4	2.1	2.1
Number	155	122	78	46	28	16	9	6	3	2	2	2	2	1	0	0

Table B8K-25 Age Group = 31-40 Gender = OVERALL Occupational group = NON-MANUAL Total cases = 148

Threshold dB HL≥	0	5	10	15	20	25	30	35	40	45	50	55	65	75	85	95
**Prevalence**	**90.9**	**76.1**	**59.9**	**35.4**	**22.8**	**14.2**	**12.2**	**9.5**	**8.3**	**6.4**	**5.9**	**4.1**	**1.0**	**0.9**	**0.0**	**0.0**
Lower CI	84.1	67.8	51.4	26.6	15.4	8.5	7.0	5.0	4.0	2.7	2.3	1.3	0.3	0.3	0.0	0.0
Upper CI	95.0	82.8	67.9	45.4	32.3	22.6	20.4	17.5	16.5	14.7	14.4	12.1	3.3	3.3	2.5	2.5
Number	135	113	90	58	37	26	22	17	14	10	8	6	3	2	0	0

Table B8K-26 Age Group = 41-50 Gender = OVERALL Occupational group = NON-MANUAL Total cases = 188

Threshold dB HL≥	0	5	10	15	20	25	30	35	40	45	50	55	65	75	85	95
**Prevalence**	**98.0**	**90.8**	**74.9**	**59.4**	**44.1**	**32.6**	**23.7**	**19.5**	**17.4**	**12.5**	**6.9**	**4.3**	**3.0**	**1.2**	**0.5**	**0.0**
Lower CI	95.0	85.5	67.7	51.4	37.0	26.8	17.9	14.3	12.5	8.3	4.5	3.2	2.0	0.8	0.2	0.0
Upper CI	99.3	94.3	81.0	66.9	51.6	39.0	30.8	26.1	23.7	18.6	10.7	5.8	4.4	1.8	1.3	2.0
Number	184	172	141	115	85	65	50	41	37	27	19	14	10	6	3	0

Table B8K-27 Age Group = 51-60 Gender = OVERALL Occupational group = NON-MANUAL Total cases = 317

Threshold dB HL≥	0	5	10	15	20	25	30	35	40	45	50	55	65	75	85	95
**Prevalence**	**99.1**	**97.5**	**89.4**	**81.9**	**69.0**	**59.5**	**50.9**	**41.7**	**33.4**	**26.8**	**22.2**	**18.0**	**9.6**	**3.4**	**1.2**	**0.9**
Lower CI	97.3	94.4	84.9	76.5	63.0	53.5	45.1	36.1	28.3	21.5	17.3	13.4	6.1	2.1	0.6	0.5
Upper CI	99.7	98.9	92.7	86.2	74.4	65.2	56.7	47.4	39.0	33.0	28.1	23.7	14.7	5.6	2.2	1.9
Number	314	310	287	267	231	200	172	149	121	105	88	72	39	18	9	7

Table B8K-28 Age Group = 61-70 Gender = OVERALL Occupational group = NON-MANUAL Total cases = 228

Threshold dB HL≥	0	5	10	15	20	25	30	35	40	45	50	55	65	75	85	95
**Prevalence**	**100.0**	**100.0**	**96.6**	**93.2**	**88.5**	**86.0**	**83.0**	**78.2**	**70.4**	**63.4**	**56.6**	**49.3**	**34.1**	**18.3**	**8.0**	**2.8**
Lower CI	98.3	98.3	87.2	84.8	80.4	77.7	74.7	69.8	61.8	55.0	48.6	41.7	27.3	12.8	5.3	1.6
Upper CI	100.0	100.0	99.1	97.1	93.5	91.5	89.0	84.7	77.8	71.0	64.3	56.9	41.7	25.5	11.9	4.8
Number	228	228	225	219	208	204	197	186	173	159	147	129	94	51	29	14

Prevalence by age group, gender and occupational group for the better ear over 8 kHz

Table B8K-29 Age Group = 71-80 Gender = OVERALL Occupational group = NON-MANUAL Total cases = 121

Threshold dB HL≥	0	5	10	15	20	25	30	35	40	45	50	55	65	75	85	95
**Prevalence**	**100.0**	**100.0**	**100.0**	**97.3**	**96.2**	**94.6**	**93.9**	**89.4**	**82.2**	**82.2**	**76.9**	**73.3**	**56.9**	**26.6**	**15.9**	**3.7**
Lower CI	96.9	96.9	96.9	90.9	89.5	87.7	86.9	81.0	69.9	69.9	64.5	60.9	47.9	19.7	10.6	2.3
Upper CI	100.0	100.0	100.0	99.2	98.7	97.7	97.2	94.4	90.2	90.2	85.9	82.9	65.4	34.9	23.1	6.0
Number	121	121	121	118	117	115	114	111	106	106	101	94	75	43	26	8

Table B8K-30 Age Group = 18-30 Gender = OVERALL Occupational group = MANUAL Total cases = 218

Threshold dB HL≥	0	5	10	15	20	25	30	35	40	45	50	55	65	75	85	95
**Prevalence**	**85.1**	**65.7**	**47.7**	**29.1**	**20.7**	**11.9**	**5.1**	**3.7**	**1.8**	**1.6**	**0.7**	**0.6**	**0.5**	**0.5**	**0.5**	**0.0**
Lower CI	77.4	56.9	39.0	21.8	14.4	7.4	3.0	2.0	0.8	0.6	0.2	0.1	0.1	0.1	0.1	0.0
Upper CI	90.6	73.6	56.6	37.6	28.8	18.8	8.6	6.7	3.9	3.7	2.5	2.6	2.6	2.6	2.6	0.2
Number	189	155	119	75	54	37	23	19	10	8	4	3	2	2	2	1

Table B8K-31 Age Group = 31-40 Gender = OVERALL Occupational group = MANUAL Total cases = 183

Threshold dB HL≥	0	5	10	15	20	25	30	35	40	45	50	55	65	75	85	95
**Prevalence**	**90.6**	**80.0**	**66.4**	**46.2**	**33.6**	**17.9**	**13.8**	**6.5**	**4.5**	**4.2**	**3.3**	**2.7**	**1.8**	**1.2**	**0.9**	**0.9**
Lower CI	83.0	71.0	56.6	37.1	25.6	12.5	9.2	4.0	2.5	2.3	1.6	1.1	0.6	0.3	0.2	0.2
Upper CI	95.0	86.7	75.1	55.5	42.7	25.0	20.2	10.6	8.0	7.7	6.7	6.3	4.9	4.8	5.0	5.0
Number	171	157	137	105	78	53	43	25	17	15	11	9	7	3	1	1

Table B8K-32 Age Group = 41-50 Gender = OVERALL Occupational group = MANUAL Total cases = 262

Threshold dB HL≥	0	5	10	15	20	25	30	35	40	45	50	55	65	75	85	95
**Prevalence**	**98.9**	**93.2**	**81.7**	**64.3**	**54.5**	**44.6**	**31.8**	**25.3**	**17.3**	**14.7**	**10.5**	**9.2**	**5.0**	**1.6**	**0.7**	**0.4**
Lower CI	96.2	88.5	75.6	57.1	47.4	37.7	26.0	20.0	13.0	10.7	7.1	6.1	3.0	1.0	0.3	0.2
Upper CI	99.7	96.1	86.6	70.9	61.5	51.8	38.3	31.5	22.6	19.9	15.2	13.8	8.3	2.7	1.4	1.1
Number	260	248	220	183	157	134	105	87	69	57	43	38	25	13	6	4

Table B8K-33 Age Group = 51-60 Gender = OVERALL Occupational group = MANUAL Total cases = 365

Threshold dB HL≥	0	5	10	15	20	25	30	35	40	45	50	55	65	75	85	95
**Prevalence**	**99.6**	**97.7**	**92.9**	**87.8**	**78.4**	**62.3**	**54.3**	**46.0**	**39.3**	**31.4**	**25.2**	**21.9**	**13.0**	**6.4**	**3.3**	**1.0**
Lower CI	98.0	94.7	88.4	82.9	72.5	56.1	48.3	40.1	33.8	26.5	20.8	17.8	10.0	4.5	2.0	0.5
Upper CI	99.9	99.0	95.7	91.5	83.4	68.2	60.2	51.9	45.1	36.9	30.1	26.5	16.7	9.0	5.5	2.3
Number	364	358	347	332	306	270	240	208	185	157	137	124	82	45	24	8

Table B8K-34 Age Group = 61-70 Gender = OVERALL Occupational group = MANUAL Total cases = 298

Threshold dB HL≥	0	5	10	15	20	25	30	35	40	45	50	55	65	75	85	95
**Prevalence**	**100.0**	**100.0**	**99.6**	**95.2**	**91.2**	**85.4**	**80.9**	**76.7**	**66.5**	**63.8**	**59.6**	**50.0**	**30.8**	**19.5**	**7.8**	**3.3**
Lower CI	98.7	98.7	98.6	90.4	85.5	79.3	74.4	69.8	59.8	57.0	52.9	43.8	25.3	15.2	6.3	2.4
Upper CI	100.0	100.0	99.9	97.7	94.8	90.0	86.1	82.4	72.7	70.1	65.9	56.2	36.8	24.5	9.7	4.5
Number	298	298	296	290	283	270	260	247	230	218	207	187	138	90	43	20

Table B8K-35 Age Group = 71-80 Gender = OVERALL Occupational group = MANUAL Total cases = 148

Threshold dB HL≥	0	5	10	15	20	25	30	35	40	45	50	55	65	75	85	95
**Prevalence**	**100.0**	**100.0**	**100.0**	**99.4**	**99.4**	**97.4**	**94.8**	**94.8**	**93.8**	**89.8**	**79.5**	**69.2**	**52.1**	**38.1**	**16.5**	**6.5**
Lower CI	97.5	97.5	97.5	96.6	96.6	89.6	84.1	84.1	83.3	80.1	66.8	57.4	41.5	29.5	10.9	4.2
Upper CI	100.0	100.0	100.0	99.9	99.9	99.4	98.4	98.4	97.9	95.1	88.2	78.9	62.6	47.6	24.0	9.7
Number	148	148	148	147	147	146	145	145	144	138	132	127	109	81	44	21

Prevalence by age group, gender and occupational group for the better ear over 8 kHz

Table B8K-36 Age Group = 18-80 Gender = FEMALE Occupational group = NON-MANUAL Total cases = 670

	Threshold dB HL≥															
	0	5	10	15	20	25	30	35	40	45	50	55	65	75	85	95
Prevalence	**94.1**	**84.3**	**68.9**	**54.7**	**43.2**	**36.8**	**32.8**	**29.0**	**24.5**	**21.5**	**18.1**	**15.6**	**10.5**	**4.8**	**2.4**	**0.8**
Lower CI	91.5	80.9	65.2	51.3	40.2	34.2	30.4	26.6	21.9	19.1	15.8	13.5	8.9	3.5	1.6	0.5
Upper CI	95.9	87.3	72.4	58.1	46.3	39.5	35.4	31.4	27.3	24.1	20.7	18.1	12.4	6.4	3.6	1.3
Number	641	595	510	432	354	309	276	252	222	195	166	141	98	56	32	18

Table B8K-37 Age Group = 18-80 Gender = MALE Occupational group = NON-MANUAL Total cases = 513

	Threshold dB HL≥															
	0	5	10	15	20	25	30	35	40	45	50	55	65	75	85	95
Prevalence	**92.8**	**84.5**	**72.0**	**59.5**	**51.3**	**43.4**	**38.8**	**34.1**	**30.4**	**26.9**	**24.1**	**21.1**	**14.3**	**7.1**	**3.3**	**1.0**
Lower CI	88.3	79.9	67.0	54.5	46.6	39.7	35.1	30.7	26.9	23.5	20.9	18.2	12.1	5.5	2.4	0.6
Upper CI	95.7	88.2	76.5	64.3	55.9	47.2	42.5	37.6	34.0	30.6	27.6	24.5	16.8	9.2	4.7	1.6
Number	496	471	432	391	352	317	288	258	232	214	199	176	125	65	35	11

Table B8K-38 Age Group = 18-80 Gender = FEMALE Occupational group = MANUAL Total cases = 715

	Threshold dB HL≥															
	0	5	10	15	20	25	30	35	40	45	50	55	65	75	85	95
Prevalence	**95.4**	**86.7**	**74.4**	**60.4**	**51.2**	**40.7**	**33.2**	**29.0**	**24.0**	**20.9**	**17.5**	**14.1**	**9.1**	**5.9**	**2.7**	**1.4**
Lower CI	92.2	82.6	70.0	56.4	47.3	37.3	30.2	26.3	21.5	18.6	15.1	11.8	7.3	4.5	2.2	0.9
Upper CI	97.3	89.9	78.3	64.2	55.0	44.1	36.2	31.9	26.6	23.4	20.3	16.7	11.2	7.6	3.4	2.0
Number	696	657	594	516	449	390	334	292	254	220	194	174	126	81	44	20

Table B8K-39 Age Group = 18-80 Gender = MALE Occupational group = MANUAL Total cases = 759

	Threshold dB HL≥															
	0	5	10	15	20	25	30	35	40	45	50	55	65	75	85	95
Prevalence	**92.7**	**84.8**	**77.7**	**66.5**	**59.3**	**49.3**	**43.5**	**38.2**	**33.7**	**31.5**	**27.8**	**25.0**	**16.4**	**10.3**	**4.5**	**1.7**
Lower CI	88.6	80.3	72.8	61.7	54.9	45.6	40.6	35.8	31.4	29.2	25.6	22.8	14.3	8.5	3.2	1.1
Upper CI	95.4	88.5	81.9	70.9	63.6	53.1	46.5	40.7	36.2	34.0	30.0	27.5	18.8	12.3	6.2	2.6
Number	734	707	673	616	576	520	482	439	401	373	340	314	237	153	76	35

Table B8K-40 Age Group = 18-30 Gender = FEMALE Occupational group = NON-MANUAL Total cases = 115

	Threshold dB HL≥															
	0	5	10	15	20	25	30	35	40	45	50	55	65	75	85	95
Prevalence	**86.8**	**68.8**	**40.5**	**22.9**	**13.0**	**7.1**	**4.9**	**3.0**	**1.4**	**0.9**	**0.9**	**0.9**	**0.9**	**0.9**	**0.0**	**0.0**
Lower CI	78.8	59.1	31.4	16.1	8.2	3.9	2.4	1.3	0.4	0.2	0.2	0.2	0.2	0.2	0.0	0.0
Upper CI	92.1	77.1	50.3	31.6	20.1	12.6	9.9	7.1	4.3	4.7	4.7	4.7	4.7	4.7	3.2	3.2
Number	100	80	48	29	19	11	7	5	2	1	1	1	1	1	0	0

Table B8K-41 Age Group = 31-40 Gender = FEMALE Occupational group = NON-MANUAL Total cases = 86

	Threshold dB HL≥															
	0	5	10	15	20	25	30	35	40	45	50	55	65	75	85	95
Prevalence	**89.9**	**70.5**	**50.7**	**26.9**	**10.5**	**6.4**	**4.0**	**3.4**	**2.8**	**2.0**	**1.4**	**1.4**	**1.4**	**1.2**	**0.0**	**0.0**
Lower CI	80.6	58.9	39.8	18.3	5.4	3.2	1.9	1.5	1.1	0.6	0.3	0.3	0.3	0.2	0.0	0.0
Upper CI	95.0	79.9	61.6	37.7	19.3	12.4	8.4	7.5	6.9	6.8	6.5	6.5	6.5	6.7	4.3	4.3
Number	78	63	47	27	12	9	6	5	4	3	2	2	2	1	0	0

Table B8K-42 Age Group = 41-50 Gender = FEMALE Occupational group = NON-MANUAL Total cases = 112

	Threshold dB HL≥															
	0	5	10	15	20	25	30	35	40	45	50	55	65	75	85	95
Prevalence	**96.9**	**86.6**	**70.1**	**53.5**	**34.9**	**24.7**	**19.6**	**16.0**	**14.9**	**7.2**	**5.6**	**3.0**	**1.7**	**0.9**	**0.0**	**0.0**
Lower CI	91.2	78.1	60.6	43.8	26.5	18.0	13.6	10.8	10.1	4.3	3.4	1.9	0.9	0.2	0.0	0.0
Upper CI	99.0	92.2	78.2	62.9	44.4	32.9	27.5	22.9	21.4	11.8	9.3	4.8	3.1	5.0	3.3	3.3
Number	109	100	81	64	42	32	27	23	22	13	10	6	3	1	0	0

Prevalence by age group, gender and occupational group for the better ear over 8 kHz

Table B8K-43 Age Group = 51-60 Gender = FEMALE Occupational group = NON-MANUAL Total cases = 175

Threshold dB HL≥	0	5	10	15	20	25	30	35	40	45	50	55	65	75	85	95
Prevalence	98.2	97.3	86.4	76.8	63.7	50.7	41.9	34.2	25.7	20.8	14.8	10.5	5.7	3.8	1.5	1.3
Lower CI	94.9	93.4	79.5	68.9	55.2	42.5	34.1	27.1	19.6	15.4	10.4	7.0	3.3	2.0	0.7	0.6
Upper CI	99.4	98.9	91.3	83.2	71.3	58.8	50.0	42.0	32.9	27.5	20.5	15.3	9.6	7.3	3.2	3.0
Number	172	170	154	140	119	97	82	72	58	49	37	29	16	10	6	5

Table B8K-44 Age Group = 61-70 Gender = FEMALE Occupational group = NON-MANUAL Total cases = 116

Threshold dB HL≥	0	5	10	15	20	25	30	35	40	45	50	55	65	75	85	95
Prevalence	100.0	100.0	98.3	93.1	85.4	82.4	78.7	72.3	65.0	59.3	50.1	40.8	24.2	12.1	5.8	2.4
Lower CI	96.8	96.8	92.7	84.8	76.3	72.7	68.9	62.2	55.1	49.8	40.9	32.6	17.2	7.3	2.8	1.3
Upper CI	100.0	100.0	99.6	97.0	91.4	89.2	86.0	80.5	73.8	68.2	59.2	49.6	32.8	19.3	11.9	4.4
Number	116	116	114	109	99	97	92	86	79	72	63	53	37	22	12	8

Table B8K-45 Age Group = 71-80 Gender = FEMALE Occupational group = NON-MANUAL Total cases = 66

Threshold dB HL≥	0	5	10	15	20	25	30	35	40	45	50	55	65	75	85	95
Prevalence	100.0	100.0	100.0	95.5	95.5	95.5	94.2	90.7	80.1	80.1	72.8	71.7	54.5	19.1	13.0	2.3
Lower CI	94.5	94.5	94.5	85.0	85.0	85.0	83.8	76.7	60.7	60.7	54.1	53.2	42.2	11.9	7.1	1.0
Upper CI	100.0	100.0	100.0	98.8	98.8	98.8	98.1	96.6	91.2	91.2	85.8	84.9	66.3	29.1	22.5	5.3
Number	66	66	66	63	63	63	62	61	57	57	53	50	39	21	14	5

Table B8K-46 Age Group = 18-30 Gender = MALE Occupational group = NON-MANUAL Total cases = 66

Threshold dB HL≥	0	5	10	15	20	25	30	35	40	45	50	55	65	75	85	95
Prevalence	79.4	59.5	36.2	19.1	11.1	4.6	1.6	0.1	0.1	0.1	0.1	0.1	0.1	0.0	0.0	0.0
Lower CI	65.1	46.5	24.5	11.1	4.9	1.7	0.3	0.0	0.0	0.0	0.0	0.0	0.0	0.0	0.0	0.0
Upper CI	88.9	71.4	49.8	31.0	23.0	11.7	7.5	5.7	5.7	5.7	5.7	5.7	5.7	5.5	5.5	5.5
Number	55	42	30	17	9	5	2	1	1	1	1	1	1	0	0	0

Table B8K-47 Age Group = 31-40 Gender = MALE Occupational group = NON-MANUAL Total cases = 62

Threshold dB HL≥	0	5	10	15	20	25	30	35	40	45	50	55	65	75	85	95
Prevalence	92.1	82.4	70.3	45.3	36.7	22.8	21.3	16.3	14.4	11.5	11.1	7.3	0.6	0.6	0.0	0.0
Lower CI	80.1	69.5	56.7	29.6	22.4	11.9	10.8	7.3	5.9	4.1	3.8	1.9	0.1	0.1	0.0	0.0
Upper CI	97.1	90.5	81.0	62.0	53.8	39.1	37.6	32.4	31.0	28.3	28.2	23.8	7.0	7.0	5.8	5.8
Number	57	50	43	31	25	17	16	12	10	7	6	4	1	1	0	0

Table B8K-48 Age Group = 41-50 Gender = MALE Occupational group = NON-MANUAL Total cases = 76

Threshold dB HL≥	0	5	10	15	20	25	30	35	40	45	50	55	65	75	85	95
Prevalence	99.3	95.4	80.2	65.9	54.1	41.1	28.1	23.4	20.1	18.4	8.3	5.7	4.3	1.5	1.0	0.0
Lower CI	96.1	88.0	68.5	52.5	42.2	31.6	18.4	14.5	12.0	10.4	4.2	3.8	2.6	0.8	0.3	0.0
Upper CI	99.9	98.3	88.4	77.2	65.6	51.2	40.5	35.4	31.9	30.4	15.7	8.3	7.1	2.8	2.8	4.8
Number	75	72	60	51	43	33	23	18	15	14	9	8	7	5	3	0

Table B8K-49 Age Group = 51-60 Gender = MALE Occupational group = NON-MANUAL Total cases = 142

Threshold dB HL≥	0	5	10	15	20	25	30	35	40	45	50	55	65	75	85	95
Prevalence	100.0	97.7	92.8	87.6	74.9	69.4	61.1	50.3	42.2	33.7	30.8	26.5	14.0	3.0	0.8	0.5
Lower CI	97.4	91.2	86.2	79.8	66.4	60.5	52.3	41.8	34.0	24.4	21.7	17.9	7.6	1.5	0.3	0.1
Upper CI	100.0	99.4	96.4	92.6	81.9	77.0	69.2	58.7	50.9	44.4	41.5	37.3	24.3	6.1	2.5	1.9
Number	142	140	133	127	112	103	90	77	63	56	51	43	23	8	3	2

Prevalence by age group, gender and occupational group for the better ear over 8 kHz

Table B8K-50 Age Group = 61-70 Gender = MALE Occupational group = NON-MANUAL Total cases = 112

Threshold dB HL≥	0	5	10	15	20	25	30	35	40	45	50	55	65	75	85	95
**Prevalence**	**100.0**	**100.0**	**94.4**	**93.1**	**92.0**	**89.9**	**87.8**	**84.6**	**76.5**	**68.3**	**64.4**	**59.5**	**46.1**	**25.8**	**10.4**	**3.3**
**Lower CI**	96.7	96.7	73.5	73.7	73.4	72.5	70.9	68.6	60.5	53.2	50.0	45.4	33.5	15.5	6.4	1.4
**Upper CI**	100.0	100.0	99.1	98.5	97.9	96.8	95.5	93.2	87.3	80.4	76.6	72.1	59.3	39.6	16.3	7.6
**Number**	112	112	111	110	109	107	105	100	94	87	84	76	57	29	17	6

Table B8K-51 Age Group = 71-80 Gender = MALE Occupational group = NON-MANUAL Total cases = 55

Threshold dB HL≥	0	5	10	15	20	25	30	35	40	45	50	55	65	75	85	95
**Prevalence**	**100.0**	**100.0**	**100.0**	**100.0**	**97.1**	**92.8**	**92.8**	**86.9**	**84.9**	**84.9**	**83.0**	**75.7**	**60.1**	**37.6**	**19.8**	**6.1**
**Lower CI**	93.5	93.5	93.5	93.5	84.9	80.0	80.0	76.5	74.0	74.0	71.5	62.4	47.9	25.1	11.5	3.5
**Upper CI**	100.0	100.0	100.0	100.0	99.5	97.6	97.6	93.1	91.7	91.7	90.5	85.4	71.2	52.0	32.1	10.4
**Number**	55	55	55	55	54	52	52	50	49	49	48	44	36	22	12	3

Table B8K-52 Age Group = 18-30 Gender = FEMALE Occupational group = MANUAL Total cases = 109

Threshold dB HL≥	0	5	10	15	20	25	30	35	40	45	50	55	65	75	85	95
**Prevalence**	**87.2**	**69.1**	**44.9**	**26.7**	**19.8**	**12.2**	**5.9**	**4.2**	**0.7**	**0.7**	**0.2**	**0.2**	**0.0**	**0.0**	**0.0**	**0.0**
**Lower CI**	76.0	56.8	33.1	17.6	11.9	6.4	2.9	1.8	0.2	0.2	0.0	0.0	0.0	0.0	0.0	0.0
**Upper CI**	93.6	79.2	57.2	38.3	31.1	21.9	11.7	9.3	2.1	2.1	1.1	1.1	3.4	3.4	3.4	3.4
**Number**	97	80	59	37	26	21	14	11	3	3	1	1	0	0	0	0

Table B8K-53 Age Group = 31-40 Gender = FEMALE Occupational group = MANUAL Total cases = 103

Threshold dB HL≥	0	5	10	15	20	25	30	35	40	45	50	55	65	75	85	95
**Prevalence**	**95.1**	**80.7**	**66.2**	**40.5**	**27.2**	**14.2**	**10.6**	**6.2**	**3.6**	**3.1**	**2.6**	**2.6**	**1.3**	**0.3**	**0.0**	**0.0**
**Lower CI**	87.4	68.0	53.2	30.2	18.7	9.5	6.4	3.0	1.6	1.2	0.9	0.9	0.5	0.1	0.0	0.0
**Upper CI**	98.2	89.1	77.1	51.6	37.7	20.6	17.1	12.4	8.0	7.5	7.3	7.3	3.3	1.9	3.6	3.6
**Number**	99	90	77	55	38	26	20	12	9	7	5	5	4	1	0	0

Table B8K-54 Age Group = 41-50 Gender = FEMALE Occupational group = MANUAL Total cases = 135

Threshold dB HL≥	0	5	10	15	20	25	30	35	40	45	50	55	65	75	85	95
**Prevalence**	**97.9**	**90.1**	**74.7**	**56.7**	**43.1**	**34.2**	**20.5**	**15.5**	**9.7**	**7.9**	**5.5**	**3.7**	**2.5**	**0.8**	**0.4**	**0.2**
**Lower CI**	92.7	83.2	65.7	47.0	33.8	25.7	14.2	10.3	6.1	4.8	3.1	2.1	1.1	0.2	0.1	0.0
**Upper CI**	99.4	94.4	82.0	65.8	52.9	43.9	28.6	22.6	15.1	12.8	9.5	6.5	5.2	2.4	1.4	1.0
**Number**	133	123	104	84	68	56	39	31	24	20	16	13	8	3	2	1

Table B8K-55 Age Group = 51-60 Gender = FEMALE Occupational group = MANUAL Total cases = 166

Threshold dB HL≥	0	5	10	15	20	25	30	35	40	45	50	55	65	75	85	95
**Prevalence**	**99.3**	**97.1**	**90.7**	**85.2**	**74.3**	**53.7**	**42.6**	**33.8**	**28.4**	**19.7**	**14.7**	**11.8**	**5.4**	**2.1**	**1.6**	**0.3**
**Lower CI**	96.3	91.7	83.3	77.1	65.2	44.6	34.2	25.9	21.2	13.8	9.9	7.7	3.0	1.1	0.7	0.1
**Upper CI**	99.9	99.1	95.1	90.7	81.6	62.6	51.4	42.7	36.9	27.3	21.3	17.7	9.6	3.9	3.5	1.0
**Number**	165	162	154	147	130	109	91	73	64	49	40	35	20	11	7	2

Table B8K-56 Age Group = 61-70 Gender = FEMALE Occupational group = MANUAL Total cases = 126

Threshold dB HL≥	0	5	10	15	20	25	30	35	40	45	50	55	65	75	85	95
**Prevalence**	**100.0**	**100.0**	**99.3**	**91.1**	**84.1**	**76.1**	**70.4**	**66.5**	**53.3**	**49.8**	**46.0**	**36.2**	**21.5**	**14.2**	**8.1**	**3.6**
**Lower CI**	97.0	97.0	97.4	82.6	74.0	65.7	59.6	55.7	43.1	39.7	36.5	28.6	15.7	9.9	6.2	2.4
**Upper CI**	100.0	100.0	99.8	95.6	90.7	84.2	79.4	75.8	63.3	59.9	55.9	44.5	28.6	19.9	10.6	5.5
**Number**	126	126	124	118	112	104	97	92	81	72	68	59	42	27	14	7

Prevalence by age group, gender and occupational group for the better ear over 8 kHz

Table B8K-57 Age Group = 71-80 Gender = FEMALE Occupational group = MANUAL Total cases = 76

Threshold dB HL≥	0	5	10	15	20	25	30	35	40	45	50	55	65	75	85	95
**Prevalence**	**100.0**	**100.0**	**100.0**	**99.0**	**99.0**	**95.6**	**91.2**	**91.2**	**91.2**	**85.1**	**68.6**	**54.8**	**43.2**	**31.0**	**11.7**	**6.8**
**Lower CI**	95.2	95.2	95.2	94.4	94.4	82.7	74.2	74.2	74.2	69.6	49.3	37.4	28.8	20.5	8.0	3.6
**Upper CI**	100.0	100.0	100.0	99.8	99.8	99.0	97.4	97.4	97.4	93.4	83.0	71.1	58.7	44.0	17.0	12.2
**Number**	76	76	76	75	75	74	73	73	73	69	64	61	52	39	21	10

Table B8K-58 Age Group = 18-30 Gender = MALE Occupational group = MANUAL Total cases = 109

Threshold dB HL≥	0	5	10	15	20	25	30	35	40	45	50	55	65	75	85	95
**Prevalence**	**82.8**	**62.0**	**50.4**	**31.1**	**21.3**	**11.5**	**4.3**	**3.3**	**2.9**	**2.4**	**1.2**	**1.0**	**1.0**	**1.0**	**1.0**	**0.1**
**Lower CI**	70.6	49.2	37.9	20.7	12.7	5.5	1.9	1.4	1.1	0.8	0.3	0.2	0.2	0.2	0.2	0.0
**Upper CI**	90.6	73.3	62.8	43.8	33.6	22.4	9.3	7.7	7.3	7.0	5.0	5.1	5.1	5.1	5.1	0.4
**Number**	92	75	60	38	28	16	9	8	7	5	3	2	2	2	2	1

Table B8K-59 Age Group = 31-40 Gender = MALE Occupational group = MANUAL Total cases = 80

Threshold dB HL≥	0	5	10	15	20	25	30	35	40	45	50	55	65	75	85	95
**Prevalence**	**85.4**	**78.8**	**66.3**	**52.0**	**40.5**	**21.9**	**17.0**	**6.9**	**5.5**	**5.5**	**4.0**	**2.7**	**2.3**	**2.1**	**1.9**	**1.9**
**Lower CI**	71.0	64.8	50.8	37.0	27.1	12.6	9.4	3.6	2.5	2.5	1.5	0.7	0.5	0.4	0.3	0.3
**Upper CI**	93.3	88.2	78.9	66.6	55.5	35.3	28.7	12.9	11.7	11.7	10.0	9.8	9.8	9.9	10.2	10.2
**Number**	72	67	60	50	40	27	23	13	8	8	6	4	3	2	1	1

Table B8K-60 Age Group = 41-50 Gender = MALE Occupational group = MANUAL Total cases = 127

Threshold dB HL≥	0	5	10	15	20	25	30	35	40	45	50	55	65	75	85	95
**Prevalence**	**100.0**	**96.4**	**89.2**	**72.5**	**66.9**	**55.9**	**44.0**	**36.0**	**25.4**	**22.0**	**15.9**	**15.2**	**7.9**	**2.6**	**1.0**	**0.7**
**Lower CI**	97.1	87.5	79.9	61.3	55.9	45.0	34.2	26.8	17.7	14.8	9.8	9.2	4.1	1.5	0.4	0.3
**Upper CI**	100.0	99.1	94.5	81.4	76.3	66.3	54.4	46.4	35.0	31.6	24.9	24.3	14.5	4.5	2.4	2.2
**Number**	127	125	116	99	89	78	66	56	45	37	27	25	17	10	4	3

Table B8K-61 Age Group = 51-60 Gender = MALE Occupational group = MANUAL Total cases = 199

Threshold dB HL≥	0	5	10	15	20	25	30	35	40	45	50	55	65	75	85	95
**Prevalence**	**100.0**	**98.2**	**95.2**	**90.7**	**83.2**	**71.8**	**67.3**	**59.6**	**51.6**	**44.6**	**36.9**	**33.0**	**21.4**	**11.1**	**5.2**	**1.8**
**Lower CI**	98.1	94.5	89.3	84.2	75.3	63.3	58.7	51.2	43.5	36.7	29.8	26.2	16.0	7.4	2.8	0.7
**Upper CI**	100.0	99.4	97.9	94.6	88.9	79.0	74.9	67.5	59.6	52.7	44.6	40.6	28.1	16.3	9.5	4.4
**Number**	199	196	193	185	176	161	149	135	121	108	97	89	62	34	17	6

Table B8K-62 Age Group = 61-70 Gender = MALE Occupational group = MANUAL Total cases = 172

Threshold dB HL≥	0	5	10	15	20	25	30	35	40	45	50	55	65	75	85	95
**Prevalence**	**100.0**	**100.0**	**100.0**	**100.0**	**99.6**	**96.3**	**93.2**	**88.5**	**81.9**	**80.0**	**75.3**	**66.3**	**41.9**	**25.6**	**7.3**	**2.9**
**Lower CI**	97.8	97.8	97.8	97.8	97.9	91.1	87.6	80.3	73.4	71.2	66.3	55.9	32.3	18.3	5.2	1.8
**Upper CI**	100.0	100.0	100.0	100.0	99.9	98.5	96.4	93.6	88.1	86.6	82.6	75.4	52.1	34.4	10.2	4.8
**Number**	172	172	172	172	171	166	163	155	149	146	139	128	96	63	29	13

Table B8K-63 Age Group = 71-80 Gender = MALE Occupational group = MANUAL Total cases = 72

Threshold dB HL≥	0	5	10	15	20	25	30	35	40	45	50	55	65	75	85	95
**Prevalence**	**100.0**	**100.0**	**100.0**	**100.0**	**100.0**	**100.0**	**100.0**	**100.0**	**97.5**	**96.5**	**95.6**	**90.6**	**65.8**	**49.1**	**23.5**	**6.0**
**Lower CI**	94.9	94.9	94.9	94.9	94.9	94.9	94.9	94.9	86.9	87.2	86.6	82.1	51.4	35.2	11.9	3.8
**Upper CI**	100.0	100.0	100.0	100.0	100.0	100.0	100.0	100.0	99.6	99.1	98.6	95.2	77.8	63.1	41.0	9.3
**Number**	72	72	72	72	72	72	72	72	71	69	68	66	57	42	23	11

Prevalence by age group, gender and occupational group for the worse ear over 0.5, 1, 2 and 4 kHz

Table W5124-1 Age Group = 18-80 Gender = OVERALL Occupational group = OVERALL Total cases = 2646

	Threshold dB HL≥															
	**0**	**5**	**10**	**15**	**20**	**25**	**30**	**35**	**40**	**45**	**50**	**55**	**65**	**75**	**85**	**95**
**Prevalence**	**97.7**	**84.8**	**63.1**	**47.2**	**34.3**	**26.0**	**19.3**	**15.1**	**12.0**	**9.3**	**7.1**	**5.6**	**3.5**	**2.1**	**1.2**	**0.9**
**Lower CI**	96.5	82.8	61.0	45.3	32.7	24.6	18.0	14.0	11.1	8.4	6.4	4.9	3.0	1.8	1.0	0.6
**Upper CI**	98.4	86.5	65.1	49.1	36.0	27.6	20.6	16.2	13.1	10.2	7.9	6.3	4.1	2.5	1.6	1.2
**Number**	2616	2417	2002	1624	1307	1079	863	724	605	499	408	330	220	147	85	60

Table W5124-2 Age Group = 18-80 Gender = FEMALE Occupational group = OVERALL Total cases = 1377

	Threshold dB HL≥															
	**0**	**5**	**10**	**15**	**20**	**25**	**30**	**35**	**40**	**45**	**50**	**55**	**65**	**75**	**85**	**95**
**Prevalence**	**98.3**	**84.2**	**60.4**	**43.2**	**30.7**	**22.4**	**16.3**	**13.2**	**10.8**	**8.1**	**6.1**	**4.9**	**3.2**	**1.8**	**1.1**	**0.8**
**Lower CI**	97.1	81.6	57.8	40.9	28.7	20.5	14.7	11.8	9.4	7.0	5.3	4.2	2.6	1.5	0.8	0.5
**Upper CI**	99.0	86.5	63.0	45.6	32.8	24.5	18.1	14.8	12.3	9.2	7.1	5.8	4.0	2.3	1.5	1.1
**Number**	1362	1240	983	765	587	468	371	316	267	222	182	154	106	71	39	27

Table W5124-3 Age Group = 18-80 Gender = MALE Occupational group = OVERALL Total cases = 1269

	Threshold dB HL≥															
	**0**	**5**	**10**	**15**	**20**	**25**	**30**	**35**	**40**	**45**	**50**	**55**	**65**	**75**	**85**	**95**
**Prevalence**	**96.9**	**85.3**	**66.2**	**51.6**	**38.4**	**30.1**	**22.5**	**17.2**	**13.4**	**10.6**	**8.3**	**6.3**	**3.8**	**2.4**	**1.4**	**1.0**
**Lower CI**	94.7	82.3	62.9	48.5	35.8	27.9	20.6	15.6	12.0	9.4	7.2	5.3	3.0	1.8	1.0	0.7
**Upper CI**	98.2	88.0	69.3	54.6	41.1	32.4	24.5	18.9	14.9	12.0	9.6	7.4	4.7	3.1	2.1	1.7
**Number**	1254	1177	1019	859	720	611	492	408	338	277	226	176	114	76	46	33

Table W5124-4 Age Group = 18-30 Gender = OVERALL Occupational group = OVERALL Total cases = 401

	Threshold dB HL≥															
	**0**	**5**	**10**	**15**	**20**	**25**	**30**	**35**	**40**	**45**	**50**	**55**	**65**	**75**	**85**	**95**
**Prevalence**	**93.3**	**64.9**	**31.5**	**18.1**	**8.5**	**5.6**	**4.3**	**2.2**	**1.5**	**1.3**	**0.5**	**0.3**	**0.0**	**0.0**	**0.0**	**0.0**
**Lower CI**	89.2	59.2	26.5	14.3	6.0	3.9	2.9	1.4	0.9	0.7	0.2	0.1	0.0	0.0	0.0	0.0
**Upper CI**	95.9	70.3	36.8	22.6	11.8	8.0	6.4	3.5	2.6	2.4	1.1	0.8	0.1	0.1	0.1	0.1
**Number**	382	283	156	94	56	44	35	23	17	14	7	5	2	1	1	1

Table W5124-5 Age Group = 31-40 Gender = OVERALL Occupational group = OVERALL Total cases = 330

	Threshold dB HL≥															
	**0**	**5**	**10**	**15**	**20**	**25**	**30**	**35**	**40**	**45**	**50**	**55**	**65**	**75**	**85**	**95**
**Prevalence**	**97.7**	**78.5**	**43.8**	**25.8**	**17.0**	**10.4**	**8.1**	**6.1**	**3.0**	**2.5**	**2.1**	**1.9**	**1.2**	**0.9**	**0.9**	**0.7**
**Lower CI**	95.2	72.7	37.6	20.8	13.0	7.4	5.5	3.9	1.7	1.4	1.1	0.9	0.4	0.2	0.2	0.2
**Upper CI**	98.9	83.4	50.3	31.6	21.9	14.2	11.7	9.2	5.1	4.4	4.0	3.7	3.2	3.0	3.0	3.0
**Number**	323	274	172	111	81	55	43	33	21	18	14	13	6	4	4	2

Table W5124-6 Age Group = 41-50 Gender = OVERALL Occupational group = OVERALL Total cases = 448

	Threshold dB HL≥															
	**0**	**5**	**10**	**15**	**20**	**25**	**30**	**35**	**40**	**45**	**50**	**55**	**65**	**75**	**85**	**95**
**Prevalence**	**99.7**	**90.8**	**65.2**	**43.2**	**27.4**	**20.0**	**13.7**	**10.1**	**7.1**	**5.9**	**5.6**	**4.6**	**2.0**	**1.3**	**0.7**	**0.4**
**Lower CI**	98.4	87.3	60.2	38.4	23.6	16.8	11.1	8.0	5.3	4.4	4.1	3.2	1.2	0.7	0.3	0.2
**Upper CI**	99.9	93.5	69.9	48.0	31.5	23.7	16.8	12.8	9.5	8.0	7.7	6.6	3.2	2.4	1.4	1.0
**Number**	447	415	314	228	160	124	94	74	53	47	43	36	20	13	8	5

Table W5124-7 Age Group = 51-60 Gender = OVERALL Occupational group = OVERALL Total cases = 675

	Threshold dB HL≥															
	**0**	**5**	**10**	**15**	**20**	**25**	**30**	**35**	**40**	**45**	**50**	**55**	**65**	**75**	**85**	**95**
**Prevalence**	**99.7**	**97.2**	**84.6**	**61.7**	**45.1**	**33.8**	**21.9**	**16.5**	**13.6**	**10.7**	**7.8**	**6.1**	**4.4**	**2.9**	**1.7**	**1.5**
**Lower CI**	98.7	95.3	81.3	57.5	41.2	30.3	19.2	14.1	11.4	8.9	6.2	4.8	3.2	2.0	1.0	0.8
**Upper CI**	99.9	98.3	87.4	65.7	49.0	37.5	25.0	19.2	16.0	12.9	9.6	7.8	5.9	4.2	2.8	2.6
**Number**	673	658	590	472	378	298	220	178	150	122	97	76	56	40	23	19

Prevalence by age group, gender and occupational group for the worse ear over 0.5, 1, 2 and 4 kHz

Table W5124-8 Age Group = 61-70 Gender = OVERALL Occupational group = OVERALL Total cases = 522

Threshold dB HL≥	0	5	10	15	20	25	30	35	40	45	50	55	65	75	85	95
**Prevalence**	**100.0**	**99.0**	**95.5**	**84.9**	**65.0**	**51.2**	**36.9**	**30.0**	**25.2**	**19.0**	**13.9**	**10.6**	**7.5**	**4.2**	**2.1**	**1.7**
**Lower CI**	99.3	97.2	92.8	80.9	59.8	46.3	32.9	26.7	21.6	16.1	11.5	8.5	5.6	3.3	1.5	1.1
**Upper CI**	100.0	99.6	97.3	88.2	69.9	56.2	41.1	33.5	29.1	22.3	16.7	13.2	10.0	5.4	3.1	2.5
Number	522	518	503	458	383	328	264	229	202	164	132	104	72	49	26	20

Table W5124-9 Age Group = 71-80 Gender = OVERALL Occupational group = OVERALL Total cases = 270

Threshold dB HL≥	0	5	10	15	20	25	30	35	40	45	50	55	65	75	85	95
**Prevalence**	**99.6**	**99.6**	**97.6**	**93.2**	**86.8**	**71.6**	**61.4**	**53.0**	**45.2**	**33.2**	**27.6**	**20.6**	**12.6**	**6.8**	**3.9**	**2.1**
**Lower CI**	98.0	98.0	92.0	87.2	80.2	63.4	53.9	46.0	38.3	27.7	22.6	16.6	9.7	5.0	2.6	1.2
**Upper CI**	99.9	99.9	99.3	96.6	91.5	78.6	68.3	60.0	52.4	39.2	33.2	25.2	16.1	9.1	5.9	3.8
Number	269	269	267	261	249	230	207	187	162	134	115	96	64	40	23	13

Table W5124-10 Age Group = 18-80 Gender = OVERALL Occupational group = NON-MANUAL Total cases = 1179

Threshold dB HL≥	0	5	10	15	20	25	30	35	40	45	50	55	65	75	85	95
**Prevalence**	**96.4**	**81.4**	**58.6**	**41.6**	**29.9**	**24.0**	**16.6**	**13.5**	**10.7**	**7.2**	**5.1**	**3.7**	**2.5**	**1.7**	**0.9**	**0.7**
**Lower CI**	94.3	78.4	55.6	39.0	27.5	21.8	15.0	12.1	9.5	6.3	4.3	3.1	2.0	1.3	0.6	0.5
**Upper CI**	97.8	84.1	61.5	44.3	32.4	26.3	18.4	15.1	11.9	8.3	6.0	4.5	3.0	2.1	1.4	1.1
Number	1158	1044	824	626	485	400	309	261	214	171	134	104	77	55	32	25

Table W5124-11 Age Group = 18-80 Gender = OVERALL Occupational group = MANUAL Total cases = 1463

Threshold dB HL≥	0	5	10	15	20	25	30	35	40	45	50	55	65	75	85	95
**Prevalence**	**98.4**	**88.3**	**67.8**	**52.1**	**38.0**	**28.0**	**21.3**	**16.5**	**12.9**	**10.5**	**8.3**	**6.5**	**3.9**	**2.3**	**1.4**	**1.0**
**Lower CI**	96.6	85.5	64.6	49.1	35.5	25.9	19.4	15.0	11.6	9.3	7.2	5.6	3.2	1.8	1.0	0.7
**Upper CI**	99.3	90.6	70.9	55.1	40.5	30.2	23.3	18.1	14.3	11.9	9.5	7.5	4.8	2.9	1.9	1.4
Number	1454	1371	1176	996	820	677	553	462	391	328	274	226	143	92	53	35

Table W5124-12 Age Group = 18-30 Gender = FEMALE Occupational group = OVERALL Total cases = 224

Threshold dB HL≥	0	5	10	15	20	25	30	35	40	45	50	55	65	75	85	95
**Prevalence**	**96.7**	**65.6**	**29.0**	**13.7**	**6.5**	**5.0**	**4.6**	**2.9**	**2.2**	**2.0**	**0.5**	**0.3**	**0.0**	**0.0**	**0.0**	**0.0**
**Lower CI**	92.7	58.1	23.1	9.9	4.4	3.2	2.8	1.6	1.1	1.0	0.2	0.1	0.0	0.0	0.0	0.0
**Upper CI**	98.5	72.3	35.7	18.6	9.5	7.7	7.3	5.3	4.2	4.0	1.6	1.3	1.7	1.7	1.7	1.7
Number	217	158	83	47	28	22	20	13	11	9	3	2	0	0	0	0

Table W5124-13 Age Group = 31-40 Gender = FEMALE Occupational group = OVERALL Total cases = 188

Threshold dB HL≥	0	5	10	15	20	25	30	35	40	45	50	55	65	75	85	95
**Prevalence**	**96.0**	**73.0**	**37.9**	**20.0**	**14.4**	**8.3**	**6.4**	**5.2**	**2.9**	**2.2**	**2.1**	**1.7**	**0.8**	**0.6**	**0.6**	**0.3**
**Lower CI**	91.4	64.7	30.5	14.7	10.1	5.4	3.9	2.9	1.5	1.2	1.1	0.9	0.3	0.2	0.2	0.1
**Upper CI**	98.2	79.9	45.9	26.7	20.0	12.5	10.3	9.1	5.7	4.2	4.1	3.5	2.3	1.9	1.9	1.7
Number	182	149	91	55	42	28	21	16	11	10	9	8	4	3	3	1

Table W5124-14 Age Group = 41-50 Gender = FEMALE Occupational group = OVERALL Total cases = 245

Threshold dB HL≥	0	5	10	15	20	25	30	35	40	45	50	55	65	75	85	95
**Prevalence**	**100.0**	**90.0**	**58.8**	**37.5**	**21.0**	**14.9**	**10.0**	**6.0**	**4.7**	**3.5**	**3.4**	**2.9**	**1.6**	**0.9**	**0.6**	**0.4**
**Lower CI**	98.5	84.8	51.9	31.4	16.5	11.3	7.2	4.1	3.0	2.2	2.1	1.8	0.8	0.4	0.2	0.1
**Upper CI**	100.0	93.5	65.3	44.0	26.2	19.5	13.9	8.7	7.3	5.5	5.4	4.8	3.0	2.0	1.5	1.1
Number	245	226	157	108	69	52	39	28	21	18	17	15	9	6	4	3

Prevalence by age group, gender and occupational group for the worse ear over 0.5, 1, 2 and 4 kHz

Table W5124-15 Age Group = 51-60 Gender = FEMALE Occupational group = OVERALL Total cases = 338

	Threshold dB HL≥															
	0	5	10	15	20	25	30	35	40	45	50	55	65	75	85	95
**Prevalence**	**99.7**	**97.4**	**82.7**	**57.9**	**37.0**	**26.2**	**15.5**	**12.5**	**9.2**	**6.8**	**5.0**	**4.1**	**3.5**	**2.0**	**1.4**	**1.3**
**Lower CI**	98.5	94.8	77.9	52.0	31.9	21.8	12.2	9.6	6.9	4.9	3.4	2.6	2.2	1.1	0.6	0.5
**Upper CI**	100.0	98.7	86.6	63.6	42.4	31.1	19.4	16.2	12.2	9.4	7.2	6.2	5.6	3.7	3.2	3.1
**Number**	337	329	288	223	162	120	87	73	57	45	37	30	26	18	10	8

Table W5124-16 Age Group = 61-70 Gender = FEMALE Occupational group = OVERALL Total cases = 241

	Threshold dB HL≥															
	0	5	10	15	20	25	30	35	40	45	50	55	65	75	85	95
**Prevalence**	**100.0**	**98.5**	**92.3**	**78.1**	**58.4**	**42.4**	**29.1**	**23.8**	**19.0**	**13.6**	**10.5**	**9.1**	**6.9**	**3.8**	**1.9**	**1.6**
**Lower CI**	98.4	95.3	87.3	71.5	51.1	36.0	24.0	19.7	14.4	10.1	7.5	6.3	4.4	2.7	1.1	0.9
**Upper CI**	100.0	99.5	95.5	83.6	65.3	49.1	34.7	28.4	24.5	18.0	14.4	13.0	10.6	5.4	3.1	2.9
**Number**	241	238	225	197	157	127	97	87	77	63	52	45	32	22	11	9

Table W5124-17 Age Group = 71-80 Gender = FEMALE Occupational group = OVERALL Total cases = 141

	Threshold dB HL≥															
	0	5	10	15	20	25	30	35	40	45	50	55	65	75	85	95
**Prevalence**	**99.4**	**99.4**	**97.0**	**91.3**	**84.6**	**66.6**	**56.2**	**50.2**	**45.9**	**33.5**	**26.6**	**20.0**	**12.2**	**6.4**	**3.3**	**1.7**
**Lower CI**	96.8	96.8	87.6	81.5	74.4	54.0	45.8	40.7	36.3	26.8	20.7	15.4	8.5	4.3	1.9	0.7
**Upper CI**	99.9	99.9	99.3	96.2	91.2	77.2	66.1	59.8	55.7	41.1	33.4	25.7	17.1	9.4	5.8	3.9
**Number**	140	140	139	135	129	119	107	99	90	77	64	54	35	22	11	6

Table W5124-18 Age Group = 18-30 Gender = MALE Occupational group = OVERALL Total cases = 177

	Threshold dB HL≥															
	0	5	10	15	20	25	30	35	40	45	50	55	65	75	85	95
**Prevalence**	**89.6**	**64.2**	**33.9**	**22.5**	**10.4**	**6.0**	**3.9**	**1.4**	**0.7**	**0.6**	**0.4**	**0.3**	**0.1**	**0.0**	**0.0**	**0.0**
**Lower CI**	82.0	55.3	26.2	16.2	6.3	3.4	2.0	0.8	0.3	0.2	0.1	0.1	0.0	0.0	0.0	0.0
**Upper CI**	94.3	72.2	42.4	30.5	16.7	10.2	7.4	2.6	1.7	1.5	1.2	1.0	0.2	0.2	0.2	0.2
**Number**	165	125	73	47	28	22	15	10	6	5	4	3	2	1	1	1

Table W5124-19 Age Group = 31-40 Gender = MALE Occupational group = OVERALL Total cases = 142

	Threshold dB HL≥															
	0	5	10	15	20	25	30	35	40	45	50	55	65	75	85	95
**Prevalence**	**99.7**	**84.3**	**50.3**	**32.5**	**20.0**	**12.7**	**9.9**	**7.1**	**3.1**	**2.8**	**2.0**	**2.0**	**1.5**	**1.2**	**1.2**	**1.2**
**Lower CI**	98.1	75.3	40.1	23.9	13.4	7.9	5.7	3.8	1.3	1.1	0.6	0.6	0.3	0.2	0.2	0.2
**Upper CI**	99.9	90.4	60.5	42.4	28.7	19.9	16.8	12.8	7.0	6.8	6.4	6.4	6.3	6.5	6.5	6.5
**Number**	141	125	81	56	39	27	22	17	10	8	5	5	2	1	1	1

Table W5124-20 Age Group = 41-50 Gender = MALE Occupational group = OVERALL Total cases = 203

	Threshold dB HL≥															
	0	5	10	15	20	25	30	35	40	45	50	55	65	75	85	95
**Prevalence**	**99.4**	**91.7**	**72.2**	**49.4**	**34.4**	**25.6**	**17.8**	**14.7**	**9.7**	**8.5**	**8.0**	**6.5**	**2.4**	**1.7**	**0.8**	**0.4**
**Lower CI**	96.6	86.2	64.6	42.1	28.3	20.3	13.6	10.9	6.7	5.7	5.3	4.0	1.2	0.7	0.3	0.1
**Upper CI**	99.9	95.2	78.7	56.6	41.0	31.8	22.9	19.5	14.0	12.4	12.0	10.4	4.8	4.0	2.2	1.6
**Number**	202	189	157	120	91	72	55	46	32	29	26	21	11	7	4	2

Table W5124-21 Age Group = 51-60 Gender = MALE Occupational group = OVERALL Total cases = 337

	Threshold dB HL≥															
	0	5	10	15	20	25	30	35	40	45	50	55	65	75	85	95
**Prevalence**	**99.6**	**97.0**	**86.7**	**65.9**	**54.0**	**42.4**	**29.2**	**20.8**	**18.4**	**15.1**	**10.9**	**8.3**	**5.3**	**3.9**	**2.0**	**1.7**
**Lower CI**	97.6	93.7	81.8	59.9	48.3	37.0	24.7	17.2	14.9	12.0	8.3	6.2	3.6	2.4	1.1	0.9
**Upper CI**	99.9	98.6	90.5	71.5	59.7	48.0	34.1	25.0	22.5	18.9	14.1	11.1	7.9	6.2	3.8	3.4
**Number**	336	329	302	249	216	178	133	105	93	77	60	46	30	22	13	11

Prevalence by age group, gender and occupational group for the worse ear over 0.5, 1, 2 and 4 kHz

Table W5124-22 Age Group = 61-70 Gender = MALE Occupational group = OVERALL Total cases = 281

Threshold dB HL≥	0	5	10	15	20	25	30	35	40	45	50	55	65	75	85	95
**Prevalence**	**100.0**	**99.5**	**99.3**	**92.7**	**72.6**	**61.5**	**45.9**	**37.2**	**32.5**	**25.4**	**17.9**	**12.4**	**8.2**	**4.7**	**2.5**	**1.7**
**Lower CI**	98.7	97.5	97.5	88.6	64.8	53.8	39.6	32.1	27.3	20.9	14.3	9.5	5.7	3.3	1.5	0.9
**Upper CI**	100.0	99.9	99.8	95.4	79.2	68.6	52.4	42.6	38.1	30.5	22.1	16.1	11.6	6.7	4.1	3.1
**Number**	281	280	278	261	226	201	167	142	125	101	80	59	40	27	15	11

Table W5124-23 Age Group = 71-80 Gender = MALE Occupational group = OVERALL Total cases = 129

Threshold dB HL≥	0	5	10	15	20	25	30	35	40	45	50	55	65	75	85	95
**Prevalence**	**100.0**	**100.0**	**98.7**	**96.0**	**90.1**	**78.1**	**68.4**	**56.5**	**43.7**	**32.5**	**29.2**	**21.7**	**13.2**	**7.4**	**5.1**	**2.9**
**Lower CI**	97.1	97.1	92.7	90.0	82.1	70.4	58.6	46.6	34.5	24.2	21.1	15.3	9.2	4.7	2.8	1.3
**Upper CI**	100.0	100.0	99.8	98.5	94.8	84.3	76.9	66.0	53.3	42.2	38.8	29.9	18.6	11.6	8.9	6.1
**Number**	129	129	128	126	120	111	100	88	72	57	51	42	29	18	12	7

Table W5124-24 Age Group = 18-30 Gender = OVERALL Occupational group = NON-MANUAL Total cases = 181

Threshold dB HL≥	0	5	10	15	20	25	30	35	40	45	50	55	65	75	85	95
**Prevalence**	**90.0**	**55.6**	**23.2**	**11.3**	**5.6**	**4.0**	**2.1**	**1.4**	**1.2**	**1.1**	**0.3**	**0.3**	**0.1**	**0.0**	**0.0**	**0.0**
**Lower CI**	82.4	47.0	16.8	7.0	2.7	1.7	1.2	0.6	0.5	0.5	0.1	0.1	0.0	0.0	0.0	0.0
**Upper CI**	94.5	63.9	31.2	17.7	11.1	9.4	3.8	2.9	2.7	2.6	1.0	1.0	2.2	2.1	2.1	2.1
**Number**	169	110	49	23	15	12	10	7	6	5	2	2	1	0	0	0

Table W5124-25 Age Group = 31-40 Gender = OVERALL Occupational group = NON-MANUAL Total cases = 148

Threshold dB HL≥	0	5	10	15	20	25	30	35	40	45	50	55	65	75	85	95
**Prevalence**	**96.5**	**77.9**	**39.4**	**19.9**	**13.7**	**10.3**	**7.1**	**5.6**	**2.1**	**1.3**	**0.6**	**0.6**	**0.3**	**0.3**	**0.3**	**0.3**
**Lower CI**	92.6	69.0	30.5	13.2	8.8	6.2	3.8	2.7	0.9	0.6	0.2	0.2	0.1	0.1	0.1	0.1
**Upper CI**	98.4	84.8	49.2	29.0	20.7	16.6	13.1	11.2	5.1	2.9	2.3	2.3	1.7	1.7	1.7	1.7
**Number**	143	120	66	35	26	20	14	11	6	5	2	2	1	1	1	1

Table W5124-26 Age Group = 41-50 Gender = OVERALL Occupational group = NON-MANUAL Total cases = 187

Threshold dB HL≥	0	5	10	15	20	25	30	35	40	45	50	55	65	75	85	95
**Prevalence**	**99.5**	**88.9**	**59.3**	**34.8**	**23.3**	**18.9**	**13.0**	**9.9**	**5.1**	**4.3**	**4.3**	**3.1**	**1.4**	**1.2**	**0.5**	**0.5**
**Lower CI**	97.2	82.9	51.5	28.3	18.2	14.3	8.4	5.9	2.7	2.2	2.2	1.4	0.8	0.7	0.1	0.1
**Upper CI**	99.9	93.0	66.6	42.0	29.3	24.5	19.4	16.1	9.5	8.1	8.1	6.9	2.3	2.2	1.9	1.9
**Number**	186	168	115	74	47	38	26	20	11	10	10	8	5	4	2	2

Table W5124-27 Age Group = 51-60 Gender = OVERALL Occupational group = NON-MANUAL Total cases = 315

Threshold dB HL≥	0	5	10	15	20	25	30	35	40	45	50	55	65	75	85	95
**Prevalence**	**99.4**	**95.8**	**80.9**	**53.6**	**38.3**	**27.7**	**14.6**	**11.2**	**8.9**	**7.2**	**5.3**	**4.3**	**3.8**	**2.3**	**1.6**	**1.5**
**Lower CI**	97.9	92.8	75.7	47.7	33.3	23.6	10.9	8.9	6.9	5.4	3.8	2.9	2.4	1.3	0.8	0.7
**Upper CI**	99.8	97.6	85.2	59.3	43.5	32.3	19.1	13.9	11.3	9.5	7.4	6.4	5.9	4.1	3.2	3.1
**Number**	313	301	257	186	145	111	77	66	55	44	35	28	23	16	11	10

Table W5124-28 Age Group = 61-70 Gender = OVERALL Occupational group = NON-MANUAL Total cases = 225

Threshold dB HL≥	0	5	10	15	20	25	30	35	40	45	50	55	65	75	85	95
**Prevalence**	**100.0**	**98.4**	**95.6**	**85.7**	**59.8**	**49.6**	**35.3**	**28.4**	**23.3**	**16.1**	**11.4**	**7.3**	**5.2**	**3.8**	**2.5**	**1.6**
**Lower CI**	98.3	94.3	91.1	79.9	51.2	41.6	30.0	24.1	19.1	12.7	8.7	6.0	4.3	2.6	1.4	0.9
**Upper CI**	100.0	99.6	97.9	90.0	67.8	57.7	41.0	33.1	28.1	20.4	14.8	8.7	6.3	5.7	4.5	2.9
**Number**	225	223	216	192	145	123	98	83	72	58	46	34	26	20	13	10

Prevalence by age group, gender and occupational group for the worse ear over 0.5, 1, 2 and 4 kHz

Table W5124-29 Age Group = 71-80 Gender = OVERALL Occupational group = NON-MANUAL Total cases = 123

	Threshold dB HL≥															
	0	5	10	15	20	25	30	35	40	45	50	55	65	75	85	95
**Prevalence**	**99.1**	**99.1**	**98.3**	**93.4**	**82.7**	**70.7**	**58.7**	**51.8**	**46.5**	**26.7**	**19.0**	**14.1**	**8.8**	**5.1**	**2.1**	**1.1**
**Lower CI**	95.1	95.1	94.0	87.0	70.6	59.2	51.0	43.9	38.9	21.9	14.9	10.3	5.9	3.1	0.8	0.3
**Upper CI**	99.8	99.8	99.5	96.7	90.5	80.1	66.0	59.7	54.3	32.0	24.0	19.0	13.0	8.2	5.1	4.2
**Number**	122	122	121	116	107	96	84	74	64	49	39	30	21	14	5	2

Table W5124-30 Age Group = 18-30 Gender = OVERALL Occupational group = MANUAL Total cases = 218

	Threshold dB HL≥															
	0	5	10	15	20	25	30	35	40	45	50	55	65	75	85	95
**Prevalence**	**94.8**	**75.4**	**40.8**	**25.2**	**10.5**	**6.7**	**5.7**	**2.8**	**1.7**	**1.6**	**0.5**	**0.3**	**0.0**	**0.0**	**0.0**	**0.0**
**Lower CI**	87.8	67.0	32.5	18.5	6.8	4.4	3.6	1.5	0.8	0.7	0.2	0.1	0.0	0.0	0.0	0.0
**Upper CI**	97.8	82.3	49.7	33.3	15.8	10.1	9.2	5.2	3.7	3.6	1.2	0.9	0.2	0.2	0.2	0.2
**Number**	211	173	107	71	41	32	25	16	11	9	5	3	1	1	1	1

Table W5124-31 Age Group = 31-40 Gender = OVERALL Occupational group = MANUAL Total cases = 182

	Threshold dB HL≥															
	0	5	10	15	20	25	30	35	40	45	50	55	65	75	85	95
**Prevalence**	**98.9**	**79.9**	**48.4**	**30.5**	**19.4**	**10.4**	**9.2**	**6.9**	**3.4**	**3.1**	**2.9**	**2.6**	**1.5**	**1.2**	**1.2**	**0.9**
**Lower CI**	95.9	71.3	39.3	23.3	13.4	6.6	5.5	3.8	1.7	1.5	1.4	1.1	0.5	0.3	0.3	0.2
**Upper CI**	99.7	86.3	57.7	38.7	27.1	16.1	14.8	12.1	6.4	6.2	6.0	5.6	4.8	4.8	4.8	5.0
**Number**	180	154	106	76	55	35	29	22	15	13	12	11	5	3	3	1

Table W5124-32 Age Group = 41-50 Gender = OVERALL Occupational group = MANUAL Total cases = 261

	Threshold dB HL≥															
	0	5	10	15	20	25	30	35	40	45	50	55	65	75	85	95
**Prevalence**	**100.0**	**92.2**	**71.7**	**51.1**	**32.3**	**23.1**	**16.0**	**12.0**	**9.2**	**7.4**	**7.0**	**6.0**	**2.7**	**1.7**	**0.8**	**0.3**
**Lower CI**	98.6	86.9	64.9	43.9	26.4	18.1	12.2	8.9	6.6	5.2	4.7	3.9	1.4	0.7	0.3	0.1
**Upper CI**	100.0	95.5	77.7	58.2	38.8	29.0	20.7	16.1	12.7	10.6	10.1	9.2	4.9	4.1	1.7	0.9
**Number**	261	247	199	154	113	86	68	54	42	37	33	28	15	9	6	3

Table W5124-33 Age Group = 51-60 Gender = OVERALL Occupational group = MANUAL Total cases = 360

	Threshold dB HL≥															
	0	5	10	15	20	25	30	35	40	45	50	55	65	75	85	95
**Prevalence**	**100.0**	**98.9**	**88.5**	**68.9**	**50.7**	**38.5**	**26.8**	**19.0**	**15.8**	**11.9**	**8.9**	**6.6**	**4.2**	**2.8**	**1.4**	**1.2**
**Lower CI**	98.9	96.4	83.5	62.7	44.9	33.3	22.5	15.5	12.8	9.3	6.8	4.9	2.9	1.8	0.7	0.6
**Upper CI**	100.0	99.7	92.1	74.5	56.5	44.0	31.7	23.0	19.4	15.1	11.7	8.8	6.0	4.3	2.7	2.4
**Number**	360	357	333	286	233	187	143	112	95	78	62	48	33	24	12	9

Table W5124-34 Age Group = 61-70 Gender = OVERALL Occupational group = MANUAL Total cases = 296

	Threshold dB HL≥															
	0	5	10	15	20	25	30	35	40	45	50	55	65	75	85	95
**Prevalence**	**100.0**	**99.2**	**95.0**	**83.3**	**69.5**	**52.0**	**38.0**	**31.5**	**26.4**	**21.0**	**15.4**	**12.5**	**8.8**	**4.3**	**2.2**	**1.9**
**Lower CI**	98.7	96.8	90.3	76.7	61.8	45.1	32.7	27.0	21.6	17.0	11.8	9.1	5.8	3.3	1.5	1.3
**Upper CI**	100.0	99.8	97.5	88.3	76.2	58.8	43.6	36.3	31.9	25.7	19.9	16.9	13.1	5.7	3.1	2.7
**Number**	296	294	286	265	237	204	166	146	130	106	86	70	46	29	13	10

Table W5124-35 Age Group = 71-80 Gender = OVERALL Occupational group = MANUAL Total cases = 146

	Threshold dB HL≥															
	0	5	10	15	20	25	30	35	40	45	50	55	65	75	85	95
**Prevalence**	**100.0**	**100.0**	**97.4**	**94.9**	**91.9**	**75.7**	**64.2**	**57.5**	**45.7**	**39.2**	**32.6**	**23.6**	**14.0**	**7.9**	**5.8**	**3.3**
**Lower CI**	97.4	97.4	86.6	87.2	82.4	62.6	52.9	47.8	36.7	30.2	23.9	18.3	10.4	5.5	3.8	2.0
**Upper CI**	100.0	100.0	99.6	98.0	96.5	85.3	74.1	66.7	55.0	49.1	42.7	29.8	18.7	11.3	8.9	5.5
**Number**	146	146	145	144	141	133	122	112	98	85	76	66	43	26	18	11

Prevalence by age group, gender and occupational group for the worse ear over 0.5, 1, 2 and 4 kHz

Table W5124-36 Age Group = 18-80 Gender = FEMALE Occupational group = NON-MANUAL Total cases = 668

Threshold dB HL≥	0	5	10	15	20	25	30	35	40	45	50	55	65	75	85	95
**Prevalence**	**97.6**	**81.4**	**57.6**	**39.9**	**27.6**	**22.1**	**15.8**	**12.9**	**10.7**	**6.8**	**4.3**	**3.7**	**2.7**	**1.8**	**1.1**	**0.8**
**Lower CI**	95.9	77.9	54.2	36.9	24.7	19.4	13.9	11.2	9.2	5.8	3.5	3.0	2.1	1.3	0.7	0.5
**Upper CI**	98.6	84.5	60.8	43.0	30.7	24.9	17.9	14.8	12.4	8.1	5.3	4.6	3.6	2.5	1.8	1.5
**Number**	656	583	449	333	246	205	163	137	115	94	72	63	48	35	20	15

Table W5124-37 Age Group = 18-80 Gender = MALE Occupational group = NON-MANUAL Total cases = 511

Threshold dB HL≥	0	5	10	15	20	25	30	35	40	45	50	55	65	75	85	95
**Prevalence**	**95.1**	**81.3**	**59.8**	**43.5**	**32.5**	**26.0**	**17.4**	**14.2**	**10.7**	**7.8**	**6.1**	**3.8**	**2.2**	**1.5**	**0.8**	**0.6**
**Lower CI**	90.7	76.0	54.6	39.2	28.8	22.6	14.8	11.9	9.0	6.3	4.7	2.7	1.7	1.0	0.4	0.3
**Upper CI**	97.5	85.7	64.7	47.9	36.4	29.8	20.3	16.9	12.6	9.6	7.7	5.2	2.8	2.2	1.4	1.1
**Number**	502	461	375	293	239	195	146	124	99	77	62	41	29	20	12	10

Table W5124-38 Age Group = 18-80 Gender = FEMALE Occupational group = MANUAL Total cases = 708

Threshold dB HL≥	0	5	10	15	20	25	30	35	40	45	50	55	65	75	85	95
**Prevalence**	**98.7**	**87.9**	**64.4**	**46.9**	**33.6**	**22.8**	**17.0**	**13.7**	**10.9**	**8.9**	**7.0**	**5.3**	**3.1**	**1.7**	**1.1**	**0.7**
**Lower CI**	95.8	83.9	59.9	43.0	30.6	19.9	14.7	11.9	9.2	7.4	5.7	4.3	2.4	1.3	0.7	0.5
**Upper CI**	99.6	91.0	68.6	50.8	36.7	25.9	19.6	15.8	12.8	10.6	8.6	6.6	4.0	2.2	1.5	1.0
**Number**	705	656	533	431	340	262	208	179	152	128	110	91	58	36	19	12

Table W5124-39 Age Group = 18-80 Gender = MALE Occupational group = MANUAL Total cases = 755

Threshold dB HL≥	0	5	10	15	20	25	30	35	40	45	50	55	65	75	85	95
**Prevalence**	**98.1**	**88.7**	**71.7**	**57.9**	**42.9**	**33.8**	**26.1**	**19.5**	**15.1**	**12.3**	**9.7**	**7.8**	**4.8**	**3.0**	**1.8**	**1.3**
**Lower CI**	94.9	84.4	66.9	53.2	39.0	30.7	23.2	17.1	13.2	10.5	8.0	6.4	3.6	2.1	1.2	0.8
**Upper CI**	99.3	91.9	76.1	62.5	47.0	37.0	29.2	22.1	17.3	14.4	11.7	9.6	6.3	4.1	2.8	2.3
**Number**	749	715	643	565	480	415	345	283	239	200	164	135	85	56	34	23

Table W5124-40 Age Group = 18-30 Gender = FEMALE Occupational group = NON-MANUAL Total cases = 115

Threshold dB HL≥	0	5	10	15	20	25	30	35	40	45	50	55	65	75	85	95
**Prevalence**	**96.3**	**57.7**	**20.8**	**9.2**	**5.8**	**4.1**	**3.7**	**2.2**	**2.2**	**2.0**	**0.4**	**0.4**	**0.0**	**0.0**	**0.0**	**0.0**
**Lower CI**	91.2	47.9	14.4	5.6	3.2	2.3	2.0	1.0	1.0	0.8	0.1	0.1	0.0	0.0	0.0	0.0
**Upper CI**	98.5	66.9	29.0	14.5	10.1	7.4	6.9	5.1	5.1	4.9	2.2	2.2	3.2	3.2	3.2	3.2
**Number**	110	71	30	16	11	9	8	5	5	4	1	1	0	0	0	0

Table W5124-41 Age Group = 31-40 Gender = FEMALE Occupational group = NON-MANUAL Total cases = 86

Threshold dB HL≥	0	5	10	15	20	25	30	35	40	45	50	55	65	75	85	95
**Prevalence**	**93.4**	**70.8**	**35.1**	**17.4**	**12.1**	**8.1**	**6.2**	**5.4**	**2.9**	**1.4**	**1.2**	**1.2**	**0.6**	**0.6**	**0.6**	**0.6**
**Lower CI**	86.2	59.2	25.4	10.4	6.7	4.1	2.9	2.3	1.0	0.4	0.3	0.3	0.1	0.1	0.1	0.1
**Upper CI**	96.9	80.1	46.3	27.6	21.1	15.1	12.6	12.2	8.7	4.4	4.5	4.5	3.2	3.2	3.2	3.2
**Number**	81	64	35	18	13	10	8	7	4	3	2	2	1	1	1	1

Table W5124-42 Age Group = 41-50 Gender = FEMALE Occupational group = NON-MANUAL Total cases = 111

Threshold dB HL≥	0	5	10	15	20	25	30	35	40	45	50	55	65	75	85	95
**Prevalence**	**100.0**	**90.4**	**58.0**	**32.0**	**17.6**	**14.4**	**7.9**	**4.7**	**1.6**	**1.6**	**1.6**	**0.6**	**0.0**	**0.0**	**0.0**	**0.0**
**Lower CI**	96.7	83.6	48.5	24.2	12.5	9.9	4.5	2.7	0.5	0.5	0.5	0.1	0.0	0.0	0.0	0.0
**Upper CI**	100.0	94.6	67.0	41.0	24.0	20.5	13.5	8.0	5.3	5.3	5.3	4.4	3.3	3.3	3.3	3.3
**Number**	111	101	67	42	23	19	11	7	2	2	2	1	0	0	0	0

Prevalence by age group, gender and occupational group for the worse ear over 0.5, 1, 2 and 4 kHz

Table W5124-43 Age Group = 51-60 Gender = FEMALE Occupational group = NON-MANUAL Total cases = 173

	Threshold dB HL≥															
	0	5	10	15	20	25	30	35	40	45	50	55	65	75	85	95
Prevalence	99.5	97.2	82.2	51.6	33.7	24.3	11.9	10.6	8.1	7.0	5.3	4.6	4.2	2.7	2.1	1.9
Lower CI	97.5	93.8	75.4	43.5	27.2	18.7	8.7	7.5	5.7	4.6	3.3	2.7	2.4	1.3	0.8	0.7
Upper CI	99.9	98.8	87.4	59.7	40.9	30.9	16.1	14.7	11.5	10.3	8.4	7.7	7.4	5.6	5.2	5.0
Number	172	167	142	99	73	56	39	34	28	23	19	16	14	10	7	6

Table W5124-44 Age Group = 61-70 Gender = FEMALE Occupational group = NON-MANUAL Total cases = 116

	Threshold dB HL≥															
	0	5	10	15	20	25	30	35	40	45	50	55	65	75	85	95
Prevalence	100.0	97.1	92.4	80.6	53.6	42.7	33.3	23.1	17.2	11.2	7.5	6.7	4.9	3.9	2.2	1.9
Lower CI	96.8	89.9	84.1	71.2	43.5	33.5	25.4	17.2	11.7	7.8	5.6	5.0	3.7	2.6	1.1	0.9
Upper CI	100.0	99.2	96.6	87.5	63.4	52.4	42.2	30.1	24.5	15.9	10.1	8.9	6.4	5.8	4.2	3.9
Number	116	114	109	95	69	58	49	40	35	29	23	21	16	13	8	7

Table W5124-45 Age Group = 71-80 Gender = FEMALE Occupational group = NON-MANUAL Total cases = 67

	Threshold dB HL≥															
	0	5	10	15	20	25	30	35	40	45	50	55	65	75	85	95
Prevalence	98.6	98.6	98.6	92.8	79.2	70.7	58.0	54.8	52.3	27.4	16.6	14.5	11.8	6.4	2.9	1.3
Lower CI	92.2	92.2	92.2	82.1	60.5	53.2	47.9	44.5	42.7	22.4	12.4	9.9	7.3	3.6	1.0	0.2
Upper CI	99.7	99.7	99.7	97.3	90.5	83.7	67.5	64.7	61.8	33.1	21.8	20.6	18.5	11.2	7.9	6.9
Number	66	66	66	63	57	53	48	44	41	33	25	22	17	11	4	1

Table W5124-46 Age Group = 18-30 Gender = MALE Occupational group = NON-MANUAL Total cases = 66

	Threshold dB HL≥															
	0	5	10	15	20	25	30	35	40	45	50	55	65	75	85	95
Prevalence	83.4	53.3	25.6	13.4	5.2	3.8	0.4	0.4	0.1	0.1	0.1	0.1	0.1	0.0	0.0	0.0
Lower CI	68.8	39.4	15.2	6.4	1.4	0.8	0.1	0.1	0.0	0.0	0.0	0.0	0.0	0.0	0.0	0.0
Upper CI	92.0	66.8	39.8	26.0	17.8	17.1	1.3	1.3	5.7	5.7	5.7	5.7	5.7	5.5	5.5	5.5
Number	59	39	19	7	4	3	2	2	1	1	1	1	1	0	0	0

Table W5124-47 Age Group = 31-40 Gender = MALE Occupational group = NON-MANUAL Total cases = 62

	Threshold dB HL≥															
	0	5	10	15	20	25	30	35	40	45	50	55	65	75	85	95
Prevalence	100.0	85.5	44.1	23.1	15.4	12.5	7.9	5.7	1.3	1.3	0.0	0.0	0.0	0.0	0.0	0.0
Lower CI	94.2	69.3	29.2	12.2	8.1	6.0	3.0	1.8	0.5	0.5	0.0	0.0	0.0	0.0	0.0	0.0
Upper CI	100.0	93.9	60.2	39.5	27.5	24.2	19.5	16.7	3.3	3.3	5.8	5.8	5.8	5.8	5.8	5.8
Number	62	56	31	17	13	10	6	4	2	2	0	0	0	0	0	0

Table W5124-48 Age Group = 41-50 Gender = MALE Occupational group = NON-MANUAL Total cases = 76

	Threshold dB HL≥															
	0	5	10	15	20	25	30	35	40	45	50	55	65	75	85	95
Prevalence	99.0	87.0	60.6	37.9	29.8	24.0	18.7	15.7	9.1	7.3	7.3	6.0	2.8	2.5	0.9	0.9
Lower CI	94.4	75.7	47.9	27.7	21.0	16.2	10.3	8.0	4.4	3.4	3.4	2.4	1.7	1.4	0.2	0.2
Upper CI	99.8	93.5	72.1	49.3	40.5	34.0	31.3	28.6	17.8	15.0	15.0	14.3	4.7	4.4	3.8	3.8
Number	75	67	48	32	24	19	15	13	9	8	8	7	5	4	2	2

Table W5124-49 Age Group = 51-60 Gender = MALE Occupational group = NON-MANUAL Total cases = 142

	Threshold dB HL≥															
	0	5	10	15	20	25	30	35	40	45	50	55	65	75	85	95
Prevalence	99.3	94.3	79.4	55.7	43.4	31.5	17.5	11.8	9.7	7.4	5.3	4.0	3.3	1.9	1.0	1.0
Lower CI	96.3	88.4	71.1	47.3	36.0	25.8	11.2	8.7	6.8	4.9	3.2	2.2	1.6	0.8	0.4	0.4
Upper CI	99.9	97.3	85.9	63.8	51.2	37.9	26.3	15.8	13.7	11.0	8.4	7.1	6.7	4.6	2.6	2.6
Number	141	134	115	87	72	55	38	32	27	21	16	12	9	6	4	4

Prevalence by age group, gender and occupational group for the worse ear over 0.5, 1, 2 and 4 kHz

Table W5124-50 Age Group = 61-70 Gender = MALE Occupational group = NON-MANUAL Total cases = 109

Threshold dB HL≥	0	5	10	15	20	25	30	35	40	45	50	55	65	75	85	95
**Prevalence**	**100.0**	**100.0**	**99.4**	**91.5**	**66.9**	**57.7**	**37.4**	**33.9**	**30.0**	**21.5**	**16.0**	**8.2**	**5.7**	**3.8**	**2.9**	**1.3**
**Lower CI**	96.6	96.6	97.8	84.5	52.1	43.7	31.0	27.9	23.9	15.7	11.1	6.5	4.5	1.8	1.2	0.5
**Upper CI**	100.0	100.0	99.9	95.5	79.0	70.5	44.3	40.5	36.8	28.7	22.6	10.3	7.3	7.7	7.3	3.5
**Number**	109	109	107	97	76	65	49	43	37	29	23	13	10	7	5	3

Table W5124-51 Age Group = 71-80 Gender = MALE Occupational group = NON-MANUAL Total cases = 56

Threshold dB HL≥	0	5	10	15	20	25	30	35	40	45	50	55	65	75	85	95
**Prevalence**	**100.0**	**100.0**	**97.9**	**93.8**	**87.6**	**69.9**	**59.1**	**46.8**	**37.3**	**25.4**	**22.6**	**13.5**	**4.3**	**3.1**	**1.0**	**1.0**
**Lower CI**	93.6	93.6	89.0	85.9	77.9	59.1	47.9	35.5	26.7	17.3	15.3	8.0	2.5	1.2	0.2	0.2
**Upper CI**	100.0	100.0	99.6	97.4	93.4	78.8	69.5	58.4	49.4	35.7	32.1	22.1	7.0	8.1	5.3	5.3
**Number**	56	56	55	53	50	43	36	30	23	16	14	8	4	3	1	1

Table W5124-52 Age Group = 18-30 Gender = FEMALE Occupational group = MANUAL Total cases = 109

Threshold dB HL≥	0	5	10	15	20	25	30	35	40	45	50	55	65	75	85	95
**Prevalence**	**96.0**	**77.2**	**40.8**	**20.1**	**7.4**	**6.3**	**5.9**	**3.7**	**2.4**	**2.3**	**0.5**	**0.2**	**0.0**	**0.0**	**0.0**	**0.0**
**Lower CI**	84.9	65.3	29.5	12.4	4.2	3.3	3.0	1.6	0.9	0.8	0.1	0.0	0.0	0.0	0.0	0.0
**Upper CI**	99.0	85.9	53.2	31.0	12.7	11.6	11.1	8.5	6.3	6.3	1.7	1.1	3.4	3.4	3.4	3.4
**Number**	107	87	53	31	17	13	12	8	6	5	2	1	0	0	0	0

Table W5124-53 Age Group = 31-40 Gender = FEMALE Occupational group = MANUAL Total cases = 102

Threshold dB HL≥	0	5	10	15	20	25	30	35	40	45	50	55	65	75	85	95
**Prevalence**	**98.7**	**75.7**	**41.2**	**21.7**	**15.6**	**8.0**	**6.3**	**5.0**	**2.7**	**2.7**	**2.7**	**2.1**	**0.8**	**0.5**	**0.5**	**0.0**
**Lower CI**	92.8	62.8	30.2	14.9	10.0	4.6	3.2	2.3	1.2	1.2	1.2	0.9	0.3	0.1	0.1	0.0
**Upper CI**	99.8	85.2	53.1	30.6	23.5	13.4	11.7	10.6	5.9	5.9	5.9	4.8	2.3	1.6	1.6	3.6
**Number**	101	85	56	37	29	18	13	9	7	7	7	6	3	2	2	0

Table W5124-54 Age Group = 41-50 Gender = FEMALE Occupational group = MANUAL Total cases = 134

Threshold dB HL≥	0	5	10	15	20	25	30	35	40	45	50	55	65	75	85	95
**Prevalence**	**100.0**	**90.1**	**62.2**	**43.7**	**25.5**	**16.4**	**12.6**	**7.6**	**7.1**	**5.0**	**4.8**	**4.5**	**2.6**	**1.5**	**1.0**	**0.6**
**Lower CI**	97.2	81.8	52.3	34.4	18.4	11.0	8.1	4.8	4.4	3.1	2.9	2.7	1.5	0.7	0.4	0.2
**Upper CI**	100.0	94.9	71.1	53.5	34.3	23.9	19.1	11.9	11.5	7.8	7.6	7.3	4.3	3.2	2.6	1.7
**Number**	134	125	90	66	46	33	28	21	19	16	15	14	9	6	4	3

Table W5124-55 Age Group = 51-60 Gender = FEMALE Occupational group = MANUAL Total cases = 165

Threshold dB HL≥	0	5	10	15	20	25	30	35	40	45	50	55	65	75	85	95
**Prevalence**	**100.0**	**97.9**	**84.0**	**65.6**	**39.9**	**27.7**	**18.5**	**13.4**	**9.8**	**5.9**	**4.5**	**3.1**	**2.4**	**1.2**	**0.5**	**0.4**
**Lower CI**	97.7	93.2	76.0	56.4	32.0	21.0	13.1	9.2	6.5	3.6	2.6	1.7	1.3	0.7	0.2	0.1
**Upper CI**	100.0	99.4	89.7	73.9	48.4	35.7	25.5	19.1	14.5	9.6	7.8	5.4	4.3	2.1	1.5	1.3
**Number**	165	162	146	124	89	64	48	39	29	22	18	14	12	8	3	2

Table W5124-56 Age Group = 61-70 Gender = FEMALE Occupational group = MANUAL Total cases = 124

Threshold dB HL≥	0	5	10	15	20	25	30	35	40	45	50	55	65	75	85	95
**Prevalence**	**100.0**	**99.5**	**91.8**	**75.2**	**63.5**	**40.2**	**24.4**	**23.4**	**19.1**	**14.3**	**11.8**	**10.1**	**7.8**	**3.7**	**2.1**	**1.9**
**Lower CI**	97.0	97.4	83.7	64.4	52.6	31.5	19.3	18.5	13.4	10.1	7.7	6.2	4.3	2.7	1.6	1.4
**Upper CI**	100.0	99.9	96.1	83.6	73.2	49.6	30.2	29.1	26.4	20.0	17.6	15.9	13.6	5.1	2.8	2.4
**Number**	124	123	115	101	87	68	48	47	42	34	29	24	16	9	3	2

Prevalence by age group, gender and occupational group for the worse ear over 0.5, 1, 2 and 4 kHz

Table W5124-57 Age Group = 71-80 Gender = FEMALE Occupational group = MANUAL Total cases = 74

	Threshold dB HL≥															
	0	5	10	15	20	25	30	35	40	45	50	55	65	75	85	95
**Prevalence**	**100.0**	**100.0**	**95.7**	**91.4**	**91.4**	**67.3**	**59.7**	**53.3**	**44.0**	**40.1**	**31.7**	**21.5**	**10.4**	**5.8**	**3.8**	**2.3**
**Lower CI**	95.1	95.1	78.7	79.2	79.2	49.1	44.4	40.9	32.2	28.3	20.6	14.8	6.6	3.3	1.9	1.0
**Upper CI**	100.0	100.0	99.3	96.7	96.7	81.4	73.3	65.3	56.6	53.3	45.5	30.1	16.0	10.0	7.5	5.0
**Number**	74	74	73	72	72	66	59	55	49	44	39	32	18	11	7	5

Table W5124-58 Age Group = 18-30 Gender = MALE Occupational group = MANUAL Total cases = 109

	Threshold dB HL≥															
	0	5	10	15	20	25	30	35	40	45	50	55	65	75	85	95
**Prevalence**	**93.5**	**73.5**	**40.5**	**30.3**	**13.6**	**6.7**	**5.3**	**1.6**	**0.9**	**0.8**	**0.5**	**0.3**	**0.1**	**0.1**	**0.1**	**0.1**
**Lower CI**	82.2	60.8	29.1	20.0	7.3	3.9	2.7	0.8	0.4	0.3	0.2	0.1	0.0	0.0	0.0	0.0
**Upper CI**	97.8	83.3	53.1	43.0	23.7	11.5	10.2	3.1	2.2	2.0	1.6	1.5	0.4	0.4	0.4	0.4
**Number**	104	86	54	40	24	19	13	8	5	4	3	2	1	1	1	1

Table W5124-59 Age Group = 31-40 Gender = MALE Occupational group = MANUAL Total cases = 80

	Threshold dB HL≥															
	0	5	10	15	20	25	30	35	40	45	50	55	65	75	85	95
**Prevalence**	**99.3**	**84.0**	**56.5**	**40.4**	**23.7**	**13.5**	**12.8**	**9.2**	**4.1**	**3.5**	**3.1**	**3.1**	**2.3**	**1.9**	**1.9**	**1.9**
**Lower CI**	96.0	71.8	41.6	27.9	13.7	6.9	6.3	4.1	1.5	1.2	0.9	0.9	0.5	0.3	0.3	0.3
**Upper CI**	99.9	91.6	70.3	54.4	38.0	24.8	24.1	19.4	10.2	10.0	9.8	9.8	9.9	10.2	10.2	10.2
**Number**	79	69	50	39	26	17	16	13	8	6	5	5	2	1	1	1

Table W5124-60 Age Group = 41-50 Gender = MALE Occupational group = MANUAL Total cases = 127

	Threshold dB HL≥															
	0	5	10	15	20	25	30	35	40	45	50	55	65	75	85	95
**Prevalence**	**100.0**	**94.5**	**82.0**	**58.9**	**39.5**	**30.2**	**19.6**	**16.7**	**11.4**	**10.0**	**9.3**	**7.7**	**2.7**	**1.9**	**0.5**	**0.0**
**Lower CI**	97.1	86.2	72.1	48.0	30.4	22.1	13.8	11.3	7.3	6.1	5.4	4.1	0.9	0.5	0.1	0.0
**Upper CI**	100.0	97.9	88.9	69.0	49.3	39.8	26.9	24.0	17.3	16.1	15.4	13.9	7.8	7.6	1.7	2.9
**Number**	127	122	109	88	67	53	40	33	23	21	18	14	6	3	2	0

Table W5124-61 Age Group = 51-60 Gender = MALE Occupational group = MANUAL Total cases = 195

	Threshold dB HL≥															
	0	5	10	15	20	25	30	35	40	45	50	55	65	75	85	95
**Prevalence**	**100.0**	**100.0**	**93.4**	**72.6**	**62.6**	**50.4**	**36.2**	**25.1**	**22.4**	**18.4**	**13.8**	**10.5**	**6.0**	**4.5**	**2.4**	**2.0**
**Lower CI**	98.1	98.1	86.9	64.1	54.1	42.6	29.6	19.9	17.5	13.9	10.0	7.4	3.8	2.6	1.1	0.9
**Upper CI**	100.0	100.0	96.8	79.8	70.4	58.2	43.3	31.1	28.3	24.0	18.8	14.5	9.5	7.7	5.0	4.6
**Number**	195	195	187	162	144	123	95	73	66	56	44	34	21	16	9	7

Table W5124-62 Age Group = 61-70 Gender = MALE Occupational group = MANUAL Total cases = 172

	Threshold dB HL≥															
	0	5	10	15	20	25	30	35	40	45	50	55	65	75	85	95
**Prevalence**	**100.0**	**98.8**	**98.8**	**92.6**	**76.2**	**65.5**	**53.9**	**40.8**	**35.1**	**29.0**	**19.8**	**15.4**	**9.9**	**5.1**	**2.3**	**1.9**
**Lower CI**	97.8	93.3	93.3	86.0	65.0	54.8	43.9	33.3	27.7	22.3	14.1	10.4	5.6	3.4	1.2	0.9
**Upper CI**	100.0	99.8	99.8	96.3	84.6	74.8	63.5	48.8	43.3	36.9	26.9	22.4	16.7	7.7	4.2	3.9
**Number**	172	171	171	164	150	136	118	99	88	72	57	46	30	20	10	8

Table W5124-63 Age Group = 71-80 Gender = MALE Occupational group = MANUAL Total cases = 72

	Threshold dB HL≥															
	0	5	10	15	20	25	30	35	40	45	50	55	65	75	85	95
**Prevalence**	**100.0**	**100.0**	**100.0**	**100.0**	**92.5**	**87.7**	**71.1**	**63.9**	**48.3**	**38.0**	**34.2**	**26.9**	**19.5**	**11.1**	**8.8**	**5.0**
**Lower CI**	94.9	94.9	94.9	94.9	73.3	67.8	54.6	48.0	35.0	25.2	21.7	19.0	12.9	6.8	5.0	2.6
**Upper CI**	100.0	100.0	100.0	100.0	98.2	96.0	83.5	77.3	61.9	52.8	49.3	36.6	28.3	17.6	14.9	9.1
**Number**	72	72	72	72	69	67	63	57	49	41	37	34	25	15	11	6

Prevalence by age group, gender and occupational group for the worse ear over 0.5, 1 and 2 kHz

Table W512-1 Age Group = 18-80 Gender = OVERALL Occupational group = OVERALL Total cases = 2649

Threshold dB HL≥	0	5	10	15	20	25	30	35	40	45	50	55	65	75	85	95
**Prevalence**	**96.1**	**79.0**	**55.3**	**37.3**	**26.1**	**19.1**	**14.9**	**12.3**	**9.1**	**7.0**	**5.7**	**4.6**	**3.1**	**1.7**	**1.1**	**0.8**
**Lower CI**	94.8	76.9	53.1	35.5	24.6	17.8	13.7	11.3	8.2	6.3	5.1	4.0	2.6	1.4	0.8	0.5
**Upper CI**	97.1	81.0	57.4	39.1	27.7	20.5	16.1	13.4	10.1	7.8	6.4	5.2	3.6	2.1	1.4	1.1
**Number**	2594	2286	1785	1353	1063	850	710	605	482	399	334	282	194	118	73	53

Table W512-2 Age Group = 18-80 Gender = FEMALE Occupational group = OVERALL Total cases = 1379

Threshold dB HL≥	0	5	10	15	20	25	30	35	40	45	50	55	65	75	85	95
**Prevalence**	**96.1**	**79.9**	**55.6**	**36.6**	**24.8**	**18.0**	**14.0**	**12.3**	**9.2**	**6.9**	**5.6**	**4.5**	**3.0**	**1.6**	**0.9**	**0.7**
**Lower CI**	94.4	77.2	52.8	34.3	22.8	16.3	12.4	10.8	7.9	6.0	4.8	3.8	2.4	1.2	0.6	0.5
**Upper CI**	97.3	82.3	58.4	38.9	27.0	19.9	15.7	13.9	10.6	8.0	6.5	5.4	3.7	2.0	1.3	1.1
**Number**	1348	1185	908	668	507	398	332	294	237	200	171	145	99	58	33	26

Table W512-3 Age Group = 18-80 Gender = MALE Occupational group = OVERALL Total cases = 1270

Threshold dB HL≥	0	5	10	15	20	25	30	35	40	45	50	55	65	75	85	95
**Prevalence**	**96.1**	**77.9**	**54.9**	**38.1**	**27.6**	**20.3**	**15.9**	**12.3**	**9.1**	**7.0**	**5.9**	**4.7**	**3.2**	**1.9**	**1.3**	**0.9**
**Lower CI**	93.8	74.5	51.5	35.2	25.3	18.4	14.3	10.9	7.9	6.0	4.9	3.9	2.5	1.4	0.8	0.5
**Upper CI**	97.6	81.0	58.2	41.0	30.1	22.3	17.7	13.8	10.4	8.2	7.0	5.7	4.1	2.6	1.9	1.5
**Number**	1246	1101	877	685	556	452	378	311	245	199	163	137	95	60	40	27

Table W512-4 Age Group = 18-30 Gender = OVERALL Occupational group = OVERALL Total cases = 401

Threshold dB HL≥	0	5	10	15	20	25	30	35	40	45	50	55	65	75	85	95
**Prevalence**	**89.8**	**60.5**	**30.8**	**12.6**	**6.7**	**4.2**	**3.4**	**2.5**	**1.4**	**1.2**	**0.7**	**0.4**	**0.1**	**0.0**	**0.0**	**0.0**
**Lower CI**	85.2	54.7	25.9	9.6	4.7	2.8	2.3	1.6	0.8	0.7	0.3	0.2	0.0	0.0	0.0	0.0
**Upper CI**	93.0	66.1	36.3	16.4	9.5	6.3	5.1	3.8	2.5	2.3	1.3	1.0	0.5	0.1	0.1	0.1
**Number**	372	264	147	78	51	36	32	26	16	13	9	6	3	1	1	1

Table W512-5 Age Group = 31-40 Gender = OVERALL Occupational group = OVERALL Total cases = 331

Threshold dB HL≥	0	5	10	15	20	25	30	35	40	45	50	55	65	75	85	95
**Prevalence**	**94.8**	**69.1**	**35.9**	**21.6**	**14.7**	**8.9**	**6.8**	**4.8**	**2.5**	**2.2**	**2.1**	**2.0**	**1.0**	**0.9**	**0.7**	**0.6**
**Lower CI**	91.5	62.7	30.0	16.9	11.0	6.3	4.6	3.0	1.4	1.1	1.1	1.0	0.3	0.2	0.2	0.1
**Upper CI**	96.8	74.8	42.2	27.2	19.5	12.5	10.1	7.6	4.4	4.1	4.0	3.9	3.1	3.0	3.0	3.1
**Number**	313	248	145	94	72	49	38	27	18	14	13	12	5	4	2	1

Table W512-6 Age Group = 41-50 Gender = OVERALL Occupational group = OVERALL Total cases = 448

Threshold dB HL≥	0	5	10	15	20	25	30	35	40	45	50	55	65	75	85	95
**Prevalence**	**98.7**	**81.2**	**54.5**	**32.5**	**20.0**	**15.6**	**10.9**	**8.4**	**7.1**	**5.5**	**4.7**	**3.6**	**1.9**	**1.0**	**0.7**	**0.4**
**Lower CI**	96.9	76.9	49.5	28.2	16.9	12.7	8.6	6.5	5.3	3.9	3.3	2.5	1.1	0.5	0.3	0.2
**Upper CI**	99.4	85.0	59.5	37.1	23.6	18.8	13.7	10.9	9.4	7.5	6.6	5.2	3.2	1.9	1.4	1.0
**Number**	443	377	271	180	128	101	77	63	54	43	38	32	18	11	8	5

Table W512-7 Age Group = 51-60 Gender = OVERALL Occupational group = OVERALL Total cases = 676

Threshold dB HL≥	0	5	10	15	20	25	30	35	40	45	50	55	65	75	85	95
**Prevalence**	**99.8**	**90.6**	**70.2**	**47.8**	**32.4**	**21.9**	**17.2**	**14.0**	**10.4**	**7.7**	**6.2**	**5.1**	**3.5**	**2.3**	**1.5**	**1.2**
**Lower CI**	99.1	87.8	66.2	43.8	28.9	19.1	14.7	11.8	8.5	6.2	4.8	3.8	2.5	1.5	0.9	0.7
**Upper CI**	99.9	92.9	74.0	51.8	36.1	25.0	19.9	16.7	12.7	9.5	7.9	6.7	5.0	3.5	2.6	2.2
**Number**	674	623	511	382	289	221	183	148	117	98	78	66	47	31	21	17

Prevalence by age group, gender and occupational group for the worse ear over 0.5, 1 and 2 kHz

Table W512-15 Age Group = 51-60 Gender = FEMALE Occupational group = OVERALL Total cases = 339

	Threshold dB HL≥															
	**0**	**5**	**10**	**15**	**20**	**25**	**30**	**35**	**40**	**45**	**50**	**55**	**65**	**75**	**85**	**95**
**Prevalence**	**99.7**	**92.4**	**72.2**	**45.8**	**29.3**	**19.5**	**14.9**	**12.0**	**8.6**	**6.4**	**4.8**	**3.5**	**2.9**	**1.6**	**1.4**	**1.2**
**Lower CI**	98.5	88.8	66.6	40.3	24.6	15.8	11.6	9.0	6.3	4.7	3.2	2.2	1.7	0.8	0.6	0.4
**Upper CI**	100.0	94.9	77.1	51.3	34.4	23.7	19.0	15.7	11.7	8.8	7.1	5.6	4.9	3.3	3.1	3.0
Number	338	316	261	186	135	102	82	66	53	46	34	28	22	13	10	7

Table W512-16 Age Group = 61-70 Gender = FEMALE Occupational group = OVERALL Total cases = 241

	Threshold dB HL≥															
	**0**	**5**	**10**	**15**	**20**	**25**	**30**	**35**	**40**	**45**	**50**	**55**	**65**	**75**	**85**	**95**
**Prevalence**	**100.0**	**96.0**	**82.4**	**63.1**	**42.3**	**30.2**	**22.2**	**20.2**	**15.0**	**10.8**	**9.8**	**8.6**	**6.2**	**3.4**	**1.6**	**1.6**
**Lower CI**	98.4	92.1	75.9	55.9	36.1	25.0	17.5	15.8	11.3	7.9	7.0	5.9	3.8	2.3	0.9	0.9
**Upper CI**	100.0	98.0	87.4	69.8	48.9	35.9	27.6	25.4	19.8	14.8	13.7	12.3	10.1	4.8	2.9	2.9
Number	241	232	206	167	127	99	85	79	66	54	50	44	29	19	9	9

Table W512-17 Age Group = 71-80 Gender = FEMALE Occupational group = OVERALL Total cases = 141

	Threshold dB HL≥															
	**0**	**5**	**10**	**15**	**20**	**25**	**30**	**35**	**40**	**45**	**50**	**55**	**65**	**75**	**85**	**95**
**Prevalence**	**99.4**	**99.4**	**92.0**	**85.3**	**69.4**	**55.7**	**45.9**	**44.7**	**36.8**	**27.1**	**21.0**	**16.4**	**11.9**	**5.5**	**2.5**	**1.9**
**Lower CI**	96.8	96.8	82.8	76.1	57.7	45.4	36.4	35.2	27.7	20.8	16.4	11.8	8.3	3.5	1.3	0.9
**Upper CI**	99.9	99.9	96.5	91.3	79.1	65.5	55.8	54.7	46.9	34.5	26.5	22.3	16.6	8.5	4.7	4.1
Number	140	140	134	130	118	105	94	91	74	63	55	45	35	18	9	7

Table W512-18 Age Group = 18-30 Gender = MALE Occupational group = OVERALL Total cases = 177

	Threshold dB HL≥															
	**0**	**5**	**10**	**15**	**20**	**25**	**30**	**35**	**40**	**45**	**50**	**55**	**65**	**75**	**85**	**95**
**Prevalence**	**87.4**	**58.7**	**30.8**	**13.6**	**6.8**	**3.5**	**2.3**	**1.4**	**0.6**	**0.4**	**0.4**	**0.3**	**0.3**	**0.0**	**0.0**	**0.0**
**Lower CI**	79.5	49.7	23.4	8.8	3.7	1.7	1.2	0.8	0.2	0.1	0.1	0.1	0.1	0.0	0.0	0.0
**Upper CI**	92.6	67.2	39.3	20.3	12.1	6.9	4.3	2.5	1.5	1.2	1.2	1.0	1.0	0.2	0.2	0.2
Number	161	114	65	36	23	15	13	10	5	4	4	3	3	1	1	1

Table W512-19 Age Group = 31-40 Gender = MALE Occupational group = OVERALL Total cases = 142

	Threshold dB HL≥															
	**0**	**5**	**10**	**15**	**20**	**25**	**30**	**35**	**40**	**45**	**50**	**55**	**65**	**75**	**85**	**95**
**Prevalence**	**98.3**	**71.0**	**36.3**	**25.5**	**16.7**	**9.8**	**7.1**	**4.3**	**2.8**	**2.2**	**2.1**	**1.8**	**1.2**	**1.2**	**1.2**	**1.2**
**Lower CI**	96.1	60.8	27.3	17.8	10.6	5.8	3.8	2.0	1.1	0.8	0.7	0.5	0.2	0.2	0.2	0.2
**Upper CI**	99.2	79.5	46.4	35.1	25.2	16.0	12.8	9.2	6.8	6.5	6.4	6.3	6.5	6.5	6.5	6.5
Number	136	108	62	44	32	24	18	11	8	5	4	3	1	1	1	1

Table W512-20 Age Group = 41-50 Gender = MALE Occupational group = OVERALL Total cases = 203

	Threshold dB HL≥															
	**0**	**5**	**10**	**15**	**20**	**25**	**30**	**35**	**40**	**45**	**50**	**55**	**65**	**75**	**85**	**95**
**Prevalence**	**99.5**	**81.0**	**58.0**	**34.5**	**23.5**	**18.1**	**14.1**	**11.1**	**9.4**	**7.1**	**5.9**	**4.2**	**2.2**	**1.3**	**0.8**	**0.4**
**Lower CI**	97.3	74.2	50.2	28.0	18.6	13.8	10.4	7.9	6.4	4.5	3.6	2.4	1.1	0.6	0.3	0.1
**Upper CI**	99.9	86.4	65.4	41.7	29.1	23.3	18.9	15.4	13.5	10.9	9.5	7.1	4.6	3.0	2.2	1.6
Number	202	170	129	87	69	55	45	37	31	24	20	16	9	6	4	2

Table W512-21 Age Group = 51-60 Gender = MALE Occupational group = OVERALL Total cases = 337

	Threshold dB HL≥															
	**0**	**5**	**10**	**15**	**20**	**25**	**30**	**35**	**40**	**45**	**50**	**55**	**65**	**75**	**85**	**95**
**Prevalence**	**99.8**	**88.6**	**68.0**	**49.8**	**35.9**	**24.6**	**19.6**	**16.4**	**12.4**	**9.1**	**7.7**	**6.8**	**4.2**	**3.0**	**1.7**	**1.3**
**Lower CI**	98.7	83.8	62.1	44.1	30.8	20.5	16.2	13.1	9.6	6.8	5.6	4.8	2.7	1.7	0.9	0.7
**Upper CI**	100.0	92.1	73.4	55.6	41.4	29.2	23.7	20.3	16.0	12.0	10.5	9.6	6.5	5.0	3.4	2.7
Number	336	307	250	196	154	119	101	82	64	52	44	38	25	18	11	10

Prevalence by age group, gender and occupational group for the worse ear over 0.5, 1 and 2 kHz

Table W512-8 Age Group = 61-70 Gender = OVERALL Occupational group = OVERALL Total cases = 523

Threshold dB HL≥	0	5	10	15	20	25	30	35	40	45	50	55	65	75	85	95
**Prevalence**	**100.0**	**96.4**	**83.1**	**64.5**	**46.8**	**35.9**	**27.5**	**23.0**	**17.1**	**12.8**	**10.8**	**8.9**	**6.5**	**3.3**	**2.1**	**1.3**
**Lower CI**	99.3	94.0	78.8	59.2	42.0	31.8	23.7	19.7	14.3	10.4	8.6	6.9	4.6	2.5	1.4	0.9
**Upper CI**	100.0	97.9	86.8	69.4	51.6	40.2	31.7	26.6	20.3	15.5	13.5	11.4	9.0	4.4	3.1	2.1
**Number**	523	506	452	375	302	251	210	186	152	124	104	88	61	39	24	16

Table W512-9 Age Group = 71-80 Gender = OVERALL Occupational group = OVERALL Total cases = 270

Threshold dB HL≥	0	5	10	15	20	25	30	35	40	45	50	55	65	75	85	95
**Prevalence**	**99.6**	**99.1**	**93.0**	**84.8**	**70.9**	**57.1**	**46.7**	**41.8**	**33.1**	**25.4**	**20.3**	**16.0**	**11.6**	**5.7**	**2.7**	**2.2**
**Lower CI**	98.0	96.8	87.1	78.6	63.2	49.7	39.7	35.1	26.7	20.5	16.4	12.6	8.8	4.0	1.7	1.2
**Upper CI**	99.9	99.8	96.3	89.5	77.5	64.2	53.8	49.0	40.2	30.9	24.9	20.1	15.0	7.9	4.4	3.8
**Number**	269	268	259	244	221	192	170	155	125	107	92	78	60	32	17	13

Table W512-10 Age Group = 18-80 Gender = OVERALL Occupational group = NON-MANUAL Total cases = 1179

Threshold dB HL≥	0	5	10	15	20	25	30	35	40	45	50	55	65	75	85	95
**Prevalence**	**94.1**	**75.9**	**49.4**	**32.1**	**23.1**	**16.8**	**13.2**	**10.8**	**7.7**	**5.0**	**4.1**	**2.9**	**2.2**	**1.3**	**0.9**	**0.7**
**Lower CI**	91.8	72.7	46.4	29.6	21.1	15.2	11.7	9.5	6.8	4.2	3.3	2.4	1.8	1.0	0.6	0.4
**Upper CI**	95.8	78.9	52.5	34.7	25.3	18.5	15.0	12.3	8.9	5.9	5.0	3.4	2.8	1.8	1.4	1.0
**Number**	1142	979	713	504	393	310	254	213	166	133	108	90	68	45	31	23

Table W512-11 Age Group = 18-80 Gender = OVERALL Occupational group = MANUAL Total cases = 1465

Threshold dB HL≥	0	5	10	15	20	25	30	35	40	45	50	55	65	75	85	95
**Prevalence**	**98.0**	**82.0**	**60.1**	**41.5**	**28.3**	**20.9**	**16.2**	**13.0**	**10.2**	**8.2**	**6.6**	**5.4**	**3.5**	**1.9**	**1.1**	**0.9**
**Lower CI**	96.4	78.9	56.7	38.7	26.0	19.0	14.6	11.7	9.0	7.1	5.7	4.6	2.8	1.4	0.8	0.6
**Upper CI**	98.9	84.7	63.4	44.3	30.7	22.9	17.9	14.5	11.5	9.4	7.7	6.5	4.3	2.4	1.6	1.3
**Number**	1447	1304	1069	846	667	539	455	391	316	266	226	192	126	73	42	30

Table W512-12 Age Group = 18-30 Gender = FEMALE Occupational group = OVERALL Total cases = 224

Threshold dB HL≥	0	5	10	15	20	25	30	35	40	45	50	55	65	75	85	95
**Prevalence**	**92.0**	**62.1**	**30.7**	**11.6**	**6.6**	**4.8**	**4.4**	**3.5**	**2.2**	**2.0**	**0.9**	**0.5**	**0.0**	**0.0**	**0.0**	**0.0**
**Lower CI**	86.4	54.7	24.5	8.3	4.5	3.0	2.7	2.1	1.1	1.0	0.4	0.2	0.0	0.0	0.0	0.0
**Upper CI**	95.4	69.1	37.7	16.0	9.6	7.5	7.1	5.9	4.2	4.0	2.1	1.6	1.7	1.7	1.7	1.7
**Number**	211	150	82	42	28	21	19	16	11	9	5	3	0	0	0	0

Table W512-13 Age Group = 31-40 Gender = FEMALE Occupational group = OVERALL Total cases = 189

Threshold dB HL≥	0	5	10	15	20	25	30	35	40	45	50	55	65	75	85	95
**Prevalence**	**91.7**	**67.1**	**35.5**	**18.3**	**13.2**	**8.3**	**6.6**	**5.3**	**2.2**	**2.1**	**2.1**	**2.1**	**0.8**	**0.6**	**0.3**	**0.0**
**Lower CI**	85.6	58.8	28.2	13.2	9.1	5.3	4.0	3.0	1.2	1.1	1.1	1.1	0.3	0.2	0.1	0.0
**Upper CI**	95.3	74.5	43.6	24.8	18.8	12.7	10.9	9.2	4.2	4.1	4.1	4.1	2.3	1.9	1.7	2.0
**Number**	177	140	83	50	40	25	20	16	10	9	9	9	4	3	1	0

Table W512-14 Age Group = 41-50 Gender = FEMALE Occupational group = OVERALL Total cases = 245

Threshold dB HL≥	0	5	10	15	20	25	30	35	40	45	50	55	65	75	85	95
**Prevalence**	**97.9**	**81.4**	**51.3**	**30.7**	**16.9**	**13.2**	**7.9**	**6.0**	**4.9**	**4.0**	**3.5**	**3.1**	**1.6**	**0.7**	**0.6**	**0.4**
**Lower CI**	94.6	75.4	44.7	25.2	13.1	9.8	5.5	4.0	3.2	2.5	2.2	1.9	0.8	0.3	0.2	0.1
**Upper CI**	99.2	86.2	57.9	36.8	21.6	17.6	11.3	9.0	7.5	6.2	5.5	5.0	3.0	1.7	1.5	1.1
**Number**	241	207	142	93	59	46	32	26	23	19	18	16	9	5	4	3

Prevalence by age group, gender and occupational group for the worse ear over 0.5, 1 and 2 kHz

Table W512-22 Age Group = 61-70 Gender = MALE Occupational group = OVERALL Total cases = 282

Threshold dB HL≥	0	5	10	15	20	25	30	35	40	45	50	55	65	75	85	95
**Prevalence**	**100.0**	**97.0**	**84.0**	**66.0**	**52.0**	**42.4**	**33.8**	**26.2**	**19.6**	**15.0**	**12.0**	**9.2**	**6.7**	**3.3**	**2.6**	**1.0**
**Lower CI**	98.7	93.7	77.9	58.2	44.7	36.1	27.8	21.7	15.8	11.8	8.9	6.6	4.3	2.2	1.6	0.5
**Upper CI**	100.0	98.6	88.6	73.1	59.2	49.0	40.4	31.3	24.0	19.0	15.9	12.6	10.1	5.0	4.4	2.1
**Number**	282	274	246	208	175	152	125	107	86	70	54	44	32	20	15	7

Table W512-23 Age Group = 71-80 Gender = MALE Occupational group = OVERALL Total cases = 129

Threshold dB HL≥	0	5	10	15	20	25	30	35	40	45	50	55	65	75	85	95
**Prevalence**	**100.0**	**98.7**	**94.0**	**83.3**	**72.2**	**58.5**	**47.3**	**37.3**	**27.7**	**22.6**	**19.6**	**15.5**	**11.2**	**6.0**	**3.2**	**2.6**
**Lower CI**	97.1	92.7	86.0	74.2	64.0	48.5	37.9	28.8	20.1	16.2	13.4	11.1	7.5	3.5	1.6	1.1
**Upper CI**	100.0	99.8	97.6	89.7	79.2	67.9	56.8	46.7	36.8	30.7	27.8	21.3	16.5	10.0	6.3	5.8
**Number**	129	128	125	114	103	87	76	64	51	44	37	33	25	14	8	6

Table W512-24 Age Group = 18-30 Gender = OVERALL Occupational group = NON-MANUAL Total cases = 181

Threshold dB HL≥	0	5	10	15	20	25	30	35	40	45	50	55	65	75	85	95
**Prevalence**	**83.4**	**52.1**	**20.9**	**5.8**	**3.4**	**2.1**	**2.1**	**1.7**	**1.2**	**1.1**	**0.7**	**0.3**	**0.1**	**0.0**	**0.0**	**0.0**
**Lower CI**	75.7	43.6	14.7	3.0	2.0	1.2	1.2	0.8	0.5	0.5	0.2	0.1	0.0	0.0	0.0	0.0
**Upper CI**	89.1	60.5	28.7	11.1	5.6	3.8	3.8	3.5	2.7	2.6	2.5	1.0	2.2	2.1	2.1	2.1
**Number**	159	102	46	17	14	10	10	8	6	5	3	2	1	0	0	0

Table W512-25 Age Group = 31-40 Gender = OVERALL Occupational group = NON-MANUAL Total cases = 148

Threshold dB HL≥	0	5	10	15	20	25	30	35	40	45	50	55	65	75	85	95
**Prevalence**	**93.2**	**69.3**	**30.0**	**17.1**	**12.3**	**8.0**	**5.6**	**4.9**	**1.0**	**0.6**	**0.6**	**0.6**	**0.3**	**0.3**	**0.3**	**0.0**
**Lower CI**	87.9	59.5	22.7	11.4	7.6	4.6	2.7	2.3	0.4	0.2	0.2	0.2	0.1	0.1	0.1	0.0
**Upper CI**	96.3	77.7	38.4	24.8	19.3	13.6	11.2	10.4	2.7	2.3	2.3	2.3	1.7	1.7	1.7	2.5
**Number**	137	108	52	30	22	17	11	10	4	2	2	2	1	1	1	0

Table W512-26 Age Group = 41-50 Gender = OVERALL Occupational group = NON-MANUAL Total cases = 187

Threshold dB HL≥	0	5	10	15	20	25	30	35	40	45	50	55	65	75	85	95
**Prevalence**	**99.0**	**78.1**	**46.3**	**28.4**	**19.4**	**15.7**	**10.9**	**7.0**	**5.8**	**4.3**	**3.7**	**1.7**	**1.4**	**0.6**	**0.5**	**0.5**
**Lower CI**	96.4	70.7	38.9	22.5	14.8	11.5	6.7	4.2	3.3	2.2	1.8	1.1	0.8	0.2	0.1	0.1
**Upper CI**	99.7	84.1	53.9	35.2	25.0	21.2	17.4	11.4	10.1	8.1	7.7	2.5	2.3	1.8	1.9	1.9
**Number**	185	150	94	60	40	31	21	15	13	10	9	6	5	3	2	2

Table W512-27 Age Group = 51-60 Gender = OVERALL Occupational group = NON-MANUAL Total cases = 315

Threshold dB HL≥	0	5	10	15	20	25	30	35	40	45	50	55	65	75	85	95
**Prevalence**	**99.8**	**88.1**	**66.9**	**42.7**	**26.3**	**14.7**	**11.9**	**9.1**	**7.0**	**5.9**	**4.8**	**3.8**	**3.2**	**2.2**	**1.6**	**1.4**
**Lower CI**	98.7	83.7	61.1	37.1	21.9	12.0	9.5	7.0	5.2	4.3	3.2	2.4	1.9	1.2	0.8	0.6
**Upper CI**	100.0	91.4	72.1	48.4	31.3	17.8	14.8	11.8	9.4	8.2	7.0	5.9	5.3	4.0	3.2	3.0
**Number**	314	279	216	149	112	82	69	53	44	37	29	23	19	15	11	9

Table W512-28 Age Group = 61-70 Gender = OVERALL Occupational group = NON-MANUAL Total cases = 225

Threshold dB HL≥	0	5	10	15	20	25	30	35	40	45	50	55	65	75	85	95
**Prevalence**	**100.0**	**97.4**	**85.5**	**61.3**	**45.1**	**34.8**	**25.0**	**20.7**	**14.8**	**10.2**	**7.9**	**5.9**	**3.9**	**3.1**	**2.4**	**1.6**
**Lower CI**	98.3	93.5	79.2	52.7	37.4	27.7	18.7	16.3	11.2	7.8	5.7	4.9	2.6	2.0	1.3	0.9
**Upper CI**	100.0	99.0	90.2	69.3	53.0	42.6	32.6	26.1	19.3	13.2	11.0	6.9	5.7	5.0	4.4	2.9
**Number**	225	219	192	144	113	92	75	67	54	44	36	31	20	16	12	10

Prevalence by age group, gender and occupational group for the worse ear over 0.5, 1 and 2 kHz

Table W512-29 Age Group = 71-80 Gender = OVERALL Occupational group = NON-MANUAL Total cases = 123

Threshold dB HL≥	0	5	10	15	20	25	30	35	40	45	50	55	65	75	85	95
**Prevalence**	**99.1**	**98.3**	**87.8**	**79.8**	**68.9**	**55.5**	**47.9**	**41.7**	**32.1**	**16.0**	**13.1**	**10.7**	**9.0**	**4.0**	**2.1**	**1.0**
Lower CI	95.1	94.0	76.5	68.8	57.7	47.7	40.6	34.8	26.3	12.3	9.5	7.6	6.0	2.2	0.8	0.2
Upper CI	99.8	99.5	94.1	87.6	78.2	63.1	55.4	49.1	38.5	20.5	17.8	14.8	13.2	7.2	5.1	4.0
Number	122	121	113	104	92	78	68	60	45	35	29	26	22	10	5	2

Table W512-30 Age Group = 18-30 Gender = OVERALL Occupational group = MANUAL Total cases = 218

Threshold dB HL≥	0	5	10	15	20	25	30	35	40	45	50	55	65	75	85	95
**Prevalence**	**95.4**	**70.5**	**40.3**	**18.4**	**8.9**	**5.6**	**4.4**	**3.0**	**1.6**	**1.4**	**0.6**	**0.4**	**0.2**	**0.0**	**0.0**	**0.0**
Lower CI	89.4	61.9	32.0	13.1	5.5	3.4	2.6	1.6	0.7	0.6	0.3	0.1	0.0	0.0	0.0	0.0
Upper CI	98.1	77.8	49.1	25.3	14.2	9.1	7.4	5.4	3.7	3.5	1.4	1.1	0.7	0.2	0.2	0.2
Number	211	162	101	61	37	26	22	18	10	8	6	4	2	1	1	1

Table W512-31 Age Group = 31-40 Gender = OVERALL Occupational group = MANUAL Total cases = 182

Threshold dB HL≥	0	5	10	15	20	25	30	35	40	45	50	55	65	75	85	95
**Prevalence**	**96.9**	**68.9**	**39.8**	**24.2**	**15.6**	**9.5**	**7.4**	**3.7**	**3.3**	**3.0**	**2.9**	**2.7**	**1.3**	**1.2**	**0.9**	**0.9**
Lower CI	93.0	59.3	31.1	17.5	10.5	5.8	4.3	2.0	1.6	1.4	1.4	1.2	0.4	0.3	0.2	0.2
Upper CI	98.7	77.1	49.2	32.4	22.5	15.1	12.6	6.8	6.3	6.1	6.0	5.9	4.8	4.8	5.0	5.0
Number	175	139	92	63	49	31	26	16	14	12	11	10	4	3	1	1

Table W512-32 Age Group = 41-50 Gender = OVERALL Occupational group = MANUAL Total cases = 261

Threshold dB HL≥	0	5	10	15	20	25	30	35	40	45	50	55	65	75	85	95
**Prevalence**	**98.6**	**82.5**	**62.2**	**37.6**	**22.9**	**18.0**	**12.9**	**10.1**	**8.6**	**6.8**	**5.9**	**5.2**	**2.5**	**1.1**	**0.8**	**0.3**
Lower CI	95.9	76.0	54.9	31.0	18.1	13.7	9.5	7.5	6.1	4.6	3.9	3.3	1.3	0.5	0.3	0.1
Upper CI	99.5	87.5	69.0	44.7	28.5	23.2	17.2	13.6	12.1	10.1	8.9	8.2	4.7	2.1	1.7	0.9
Number	258	227	177	120	88	70	56	48	41	33	29	26	13	8	6	3

Table W512-33 Age Group = 51-60 Gender = OVERALL Occupational group = MANUAL Total cases = 361

Threshold dB HL≥	0	5	10	15	20	25	30	35	40	45	50	55	65	75	85	95
**Prevalence**	**99.8**	**93.1**	**73.5**	**51.7**	**35.2**	**26.0**	**20.1**	**16.5**	**12.0**	**8.4**	**6.7**	**5.3**	**3.3**	**1.8**	**1.2**	**0.9**
Lower CI	99.1	88.8	67.3	45.9	30.2	21.6	16.3	13.1	9.3	6.4	4.9	3.8	2.1	1.0	0.6	0.4
Upper CI	100.0	95.9	78.9	57.4	40.6	30.9	24.5	20.6	15.5	10.9	9.0	7.4	5.0	3.1	2.4	2.0
Number	360	344	295	233	177	139	114	95	73	61	49	43	28	16	10	8

Table W512-34 Age Group = 61-70 Gender = OVERALL Occupational group = MANUAL Total cases = 297

Threshold dB HL≥	0	5	10	15	20	25	30	35	40	45	50	55	65	75	85	95
**Prevalence**	**100.0**	**94.9**	**80.0**	**66.8**	**47.7**	**37.1**	**28.8**	**23.5**	**18.2**	**14.7**	**13.1**	**10.4**	**8.3**	**3.5**	**2.1**	**1.5**
Lower CI	98.7	90.4	72.8	59.0	40.9	31.7	24.4	20.0	14.4	11.1	9.5	7.3	5.3	2.6	1.5	1.0
Upper CI	100.0	97.4	85.7	73.8	54.6	42.8	33.6	27.4	22.8	19.3	17.7	14.7	12.6	4.7	3.0	2.1
Number	297	286	259	230	188	159	135	119	98	80	68	57	41	23	12	6

Table W512-35 Age Group = 71-80 Gender = OVERALL Occupational group = MANUAL Total cases = 146

Threshold dB HL≥	0	5	10	15	20	25	30	35	40	45	50	55	65	75	85	95
**Prevalence**	**100.0**	**100.0**	**97.7**	**89.9**	**75.3**	**59.7**	**47.8**	**44.3**	**37.3**	**31.3**	**22.8**	**18.5**	**12.4**	**7.2**	**3.5**	**3.3**
Lower CI	97.4	97.4	87.9	79.9	63.1	49.2	38.7	35.3	28.3	23.6	17.6	13.4	8.9	4.8	2.2	2.0
Upper CI	100.0	100.0	99.6	95.2	84.5	69.4	57.1	53.7	47.2	40.1	29.0	24.9	16.9	10.6	5.7	5.5
Number	146	146	145	139	128	114	102	95	80	72	63	52	38	22	12	11

Prevalence by age group, gender and occupational group for the worse ear over 0.5, 1 and 2 kHz

Table W512-36 Age Group = 18-80 Gender = FEMALE Occupational group = NON-MANUAL Total cases = 668

Threshold dB HL≥	0	5	10	15	20	25	30	35	40	45	50	55	65	75	85	95
**Prevalence**	**94.4**	**77.5**	**51.4**	**33.0**	**23.6**	**16.9**	**12.8**	**11.9**	**8.9**	**5.1**	**4.3**	**3.4**	**2.6**	**1.4**	**1.1**	**0.8**
Lower CI	91.7	73.8	47.6	29.8	20.8	14.9	11.1	10.2	7.6	4.2	3.5	2.7	1.9	1.0	0.6	0.4
Upper CI	96.3	80.8	55.2	36.3	26.6	19.1	14.8	13.8	10.4	6.2	5.4	4.3	3.4	2.2	1.8	1.4
Number	644	554	405	282	215	170	139	126	100	81	68	59	44	27	19	14

Table W512-37 Age Group = 18-80 Gender = MALE Occupational group = NON-MANUAL Total cases = 511

Threshold dB HL≥	0	5	10	15	20	25	30	35	40	45	50	55	65	75	85	95
**Prevalence**	**93.7**	**74.0**	**47.2**	**31.0**	**22.6**	**16.7**	**13.7**	**9.6**	**6.5**	**4.9**	**3.8**	**2.3**	**1.8**	**1.2**	**0.8**	**0.5**
Lower CI	89.6	68.3	42.5	27.2	19.7	14.2	11.1	7.7	5.0	3.7	2.6	1.8	1.2	0.8	0.4	0.3
Upper CI	96.3	78.9	51.9	35.0	25.8	19.5	16.8	11.9	8.3	6.6	5.5	3.0	2.6	2.0	1.4	1.0
Number	498	425	308	222	178	140	115	87	66	52	40	31	24	18	12	9

Table W512-38 Age Group = 18-80 Gender = FEMALE Occupational group = MANUAL Total cases = 709

Threshold dB HL≥	0	5	10	15	20	25	30	35	40	45	50	55	65	75	85	95
**Prevalence**	**98.2**	**82.9**	**59.7**	**39.7**	**25.5**	**18.9**	**14.9**	**12.4**	**9.7**	**8.0**	**6.1**	**4.8**	**3.0**	**1.5**	**0.8**	**0.7**
Lower CI	95.6	78.7	55.1	36.2	22.5	16.4	12.8	10.6	8.1	6.5	5.0	3.8	2.3	1.2	0.5	0.5
Upper CI	99.3	86.5	64.1	43.3	28.6	21.6	17.3	14.4	11.6	9.8	7.4	6.1	3.9	2.0	1.1	1.0
Number	702	629	501	384	290	227	192	167	137	119	103	86	55	31	14	12

Table W512-39 Age Group = 18-80 Gender = MALE Occupational group = MANUAL Total cases = 756

Threshold dB HL≥	0	5	10	15	20	25	30	35	40	45	50	55	65	75	85	95
**Prevalence**	**97.8**	**80.9**	**60.5**	**43.5**	**31.5**	**23.1**	**17.6**	**13.7**	**10.7**	**8.4**	**7.2**	**6.1**	**4.1**	**2.2**	**1.5**	**1.1**
Lower CI	95.4	76.0	55.5	39.0	27.9	20.4	15.3	11.9	9.0	6.9	5.8	4.8	2.9	1.5	0.9	0.6
Upper CI	99.0	84.9	65.4	48.1	35.3	26.1	20.1	15.8	12.8	10.2	8.9	7.8	5.6	3.2	2.5	2.1
Number	745	675	568	462	377	312	263	224	179	147	123	106	71	42	28	18

Table W512-40 Age Group = 18-30 Gender = FEMALE Occupational group = NON-MANUAL Total cases = 115

Threshold dB HL≥	0	5	10	15	20	25	30	35	40	45	50	55	65	75	85	95
**Prevalence**	**88.2**	**55.1**	**23.2**	**7.7**	**6.2**	**3.7**	**3.7**	**3.1**	**2.2**	**2.0**	**1.2**	**0.4**	**0.0**	**0.0**	**0.0**	**0.0**
Lower CI	80.0	45.2	16.3	4.5	3.5	2.0	2.0	1.5	1.0	0.8	0.3	0.1	0.0	0.0	0.0	0.0
Upper CI	93.4	64.5	31.9	12.8	10.5	6.9	6.9	6.5	5.1	4.9	4.7	2.2	3.2	3.2	3.2	3.2
Number	104	67	32	14	12	8	8	7	5	4	2	1	0	0	0	0

Table W512-41 Age Group = 31-40 Gender = FEMALE Occupational group = NON-MANUAL Total cases = 86

Threshold dB HL≥	0	5	10	15	20	25	30	35	40	45	50	55	65	75	85	95
**Prevalence**	**88.4**	**66.1**	**31.7**	**16.1**	**10.9**	**8.1**	**5.4**	**5.4**	**1.4**	**1.2**	**1.2**	**1.2**	**0.6**	**0.6**	**0.6**	**0.0**
Lower CI	78.3	54.5	22.2	9.5	5.8	4.1	2.3	2.3	0.4	0.3	0.3	0.3	0.1	0.1	0.1	0.0
Upper CI	94.1	76.0	43.1	26.0	19.6	15.1	12.2	12.2	4.4	4.5	4.5	4.5	3.2	3.2	3.2	4.3
Number	77	60	30	17	12	10	7	7	3	2	2	2	1	1	1	0

Table W512-42 Age Group = 41-50 Gender = FEMALE Occupational group = NON-MANUAL Total cases = 111

Threshold dB HL≥	0	5	10	15	20	25	30	35	40	45	50	55	65	75	85	95
**Prevalence**	**98.1**	**80.0**	**43.8**	**27.9**	**14.9**	**12.1**	**6.7**	**5.2**	**2.9**	**1.6**	**1.6**	**0.6**	**0.0**	**0.0**	**0.0**	**0.0**
Lower CI	93.1	71.6	35.2	20.6	10.3	7.8	3.6	2.8	1.3	0.5	0.5	0.1	0.0	0.0	0.0	0.0
Upper CI	99.5	86.4	52.9	36.6	21.0	18.3	12.2	9.5	6.5	5.3	5.3	4.4	3.3	3.3	3.3	3.3
Number	109	91	54	36	20	15	8	6	4	2	2	1	0	0	0	0

Prevalence by age group, gender and occupational group for the worse ear over 0.5, 1 and 2 kHz

Table W512-43 Age Group = 51-60 Gender = FEMALE Occupational group = NON-MANUAL Total cases = 173

Threshold dB HL≥	0	5	10	15	20	25	30	35	40	45	50	55	65	75	85	95
**Prevalence**	**99.5**	**92.5**	**73.0**	**42.5**	**26.7**	**15.0**	**11.2**	**9.7**	**7.7**	**6.5**	**4.9**	**4.0**	**3.3**	**2.5**	**2.1**	**1.8**
Lower CI	97.5	88.1	65.5	35.0	20.8	11.3	8.1	6.7	5.3	4.2	2.9	2.1	1.6	1.1	0.8	0.6
Upper CI	99.9	95.3	79.4	50.3	33.5	19.6	15.3	13.7	11.2	9.8	8.2	7.2	6.4	5.4	5.2	5.0
Number	172	158	128	82	60	44	36	30	26	22	16	13	11	9	7	5

Table W512-44 Age Group = 61-70 Gender = FEMALE Occupational group = NON-MANUAL Total cases = 116

Threshold dB HL≥	0	5	10	15	20	25	30	35	40	45	50	55	65	75	85	95
**Prevalence**	**100.0**	**95.9**	**85.4**	**60.5**	**41.9**	**31.3**	**18.5**	**17.9**	**13.3**	**8.1**	**7.0**	**6.1**	**4.0**	**3.0**	**1.9**	**1.9**
Lower CI	96.8	88.9	75.8	50.3	33.1	23.7	12.9	12.4	9.0	6.2	5.3	4.9	2.7	1.9	0.9	0.9
Upper CI	100.0	98.6	91.6	69.8	51.3	40.0	25.8	25.2	19.4	10.6	9.2	7.6	6.0	4.8	3.9	3.9
Number	116	112	101	75	58	46	38	36	30	25	23	21	13	10	7	7

Table W512-45 Age Group = 71-80 Gender = FEMALE Occupational group = NON-MANUAL Total cases = 67

Threshold dB HL≥	0	5	10	15	20	25	30	35	40	45	50	55	65	75	85	95
**Prevalence**	**98.6**	**98.6**	**83.6**	**80.5**	**72.5**	**57.5**	**50.9**	**48.8**	**41.0**	**17.1**	**15.7**	**13.8**	**12.9**	**4.6**	**2.9**	**1.7**
Lower CI	92.2	92.2	65.9	64.4	54.7	47.5	41.6	40.1	33.0	12.9	11.6	9.3	8.3	2.2	1.0	0.4
Upper CI	99.7	99.7	93.1	90.4	85.1	67.0	60.2	57.5	49.5	22.2	21.0	20.1	19.3	9.5	7.9	6.8
Number	66	66	60	58	53	47	42	40	32	26	23	21	19	7	4	2

Table W512-46 Age Group = 18-30 Gender = MALE Occupational group = NON-MANUAL Total cases = 66

Threshold dB HL≥	0	5	10	15	20	25	30	35	40	45	50	55	65	75	85	95
**Prevalence**	**78.3**	**48.7**	**18.3**	**3.8**	**0.4**	**0.4**	**0.4**	**0.1**	**0.1**	**0.1**	**0.1**	**0.1**	**0.1**	**0.0**	**0.0**	**0.0**
Lower CI	64.6	35.2	9.4	0.8	0.1	0.1	0.1	0.0	0.0	0.0	0.0	0.0	0.0	0.0	0.0	0.0
Upper CI	87.7	62.5	32.5	17.1	1.3	1.3	1.3	5.7	5.7	5.7	5.7	5.7	5.7	5.5	5.5	5.5
Number	55	35	14	3	2	2	2	1	1	1	1	1	1	0	0	0

Table W512-47 Age Group = 31-40 Gender = MALE Occupational group = NON-MANUAL Total cases = 62

Threshold dB HL≥	0	5	10	15	20	25	30	35	40	45	50	55	65	75	85	95
**Prevalence**	**98.6**	**72.8**	**28.0**	**18.4**	**13.9**	**7.8**	**5.7**	**4.5**	**0.6**	**0.0**	**0.0**	**0.0**	**0.0**	**0.0**	**0.0**	**0.0**
Lower CI	-	55.6	17.8	9.9	6.9	3.2	1.8	1.2	0.1	0.0	0.0	0.0	0.0	0.0	0.0	0.0
Upper CI	-	85.1	41.2	31.4	26.3	18.0	16.7	15.6	3.6	5.8	5.8	5.8	5.8	5.8	5.8	5.8
Number	60	48	22	13	10	7	4	3	1	0	0	0	0	0	0	0

Table W512-48 Age Group = 41-50 Gender = MALE Occupational group = NON-MANUAL Total cases = 76

Threshold dB HL≥	0	5	10	15	20	25	30	35	40	45	50	55	65	75	85	95
**Prevalence**	**100.0**	**75.7**	**49.0**	**29.1**	**24.6**	**20.0**	**15.7**	**9.1**	**9.1**	**7.3**	**6.0**	**2.8**	**2.8**	**1.2**	**0.9**	**0.9**
Lower CI	95.2	62.5	36.9	20.2	16.7	13.0	8.0	4.4	4.4	3.4	2.4	1.7	1.7	0.4	0.2	0.2
Upper CI	100.0	85.4	61.2	40.0	34.6	29.7	28.6	17.8	17.8	15.0	14.3	4.7	4.7	3.7	3.8	3.8
Number	76	59	40	24	20	16	13	9	9	8	7	5	5	3	2	2

Table W512-49 Age Group = 51-60 Gender = MALE Occupational group = NON-MANUAL Total cases = 142

Threshold dB HL≥	0	5	10	15	20	25	30	35	40	45	50	55	65	75	85	95
**Prevalence**	**100.0**	**83.1**	**59.8**	**42.7**	**25.8**	**14.4**	**12.7**	**8.5**	**6.3**	**5.3**	**4.6**	**3.6**	**3.2**	**1.9**	**1.0**	**1.0**
Lower CI	97.4	74.8	50.9	34.8	19.6	10.8	9.2	5.7	3.9	3.1	2.6	1.8	1.5	0.8	0.4	0.4
Upper CI	100.0	89.0	68.1	51.0	33.3	18.8	17.2	12.4	9.9	8.8	8.2	6.9	6.6	4.6	2.6	2.6
Number	142	121	88	67	52	38	33	23	18	15	13	10	8	6	4	4

Prevalence by age group, gender and occupational group for the worse ear over 0.5, 1 and 2 kHz

Table W512-50 Age Group = 61-70 Gender = MALE Occupational group = NON-MANUAL Total cases = 109

	Threshold dB HL≥															
	0	5	10	15	20	25	30	35	40	45	50	55	65	75	85	95
Prevalence	**100.0**	**99.2**	**85.7**	**62.6**	**48.8**	**39.0**	**32.8**	**23.9**	**16.8**	**12.6**	**8.9**	**5.7**	**3.8**	**3.4**	**2.9**	**1.3**
Lower CI	96.6	96.5	76.7	48.0	35.9	26.8	21.2	17.4	11.5	8.6	5.2	4.5	1.8	1.5	1.2	0.5
Upper CI	100.0	99.8	91.5	75.2	62.0	52.7	47.1	32.0	23.9	18.1	14.9	7.3	7.7	7.4	7.3	3.5
Number	109	107	91	69	55	46	37	31	24	19	13	10	7	6	5	3

Table W512-51 Age Group = 71-80 Gender = MALE Occupational group = NON-MANUAL Total cases = 56

	Threshold dB HL≥															
	0	5	10	15	20	25	30	35	40	45	50	55	65	75	85	95
Prevalence	**100.0**	**97.9**	**93.8**	**77.8**	**62.2**	**51.7**	**43.0**	**31.2**	**19.5**	**14.4**	**8.9**	**5.9**	**3.1**	**3.1**	**1.0**	**0.0**
Lower CI	93.6	89.0	85.9	63.7	52.7	40.3	32.4	21.3	13.0	8.6	4.0	3.1	1.2	1.2	0.2	0.0
Upper CI	100.0	99.6	97.4	87.5	71.0	63.0	54.3	43.1	28.3	23.1	18.7	11.0	8.1	8.1	5.3	6.4
Number	56	55	53	46	39	31	26	20	13	9	6	5	3	3	1	0

Table W512-52 Age Group = 18-30 Gender = FEMALE Occupational group = MANUAL Total cases = 109

	Threshold dB HL≥															
	0	5	10	15	20	25	30	35	40	45	50	55	65	75	85	95
Prevalence	**97.0**	**72.1**	**40.5**	**16.6**	**7.2**	**6.3**	**5.6**	**4.0**	**2.4**	**2.3**	**0.7**	**0.5**	**0.0**	**0.0**	**0.0**	**0.0**
Lower CI	85.3	60.3	29.1	10.4	4.0	3.3	2.8	1.8	0.9	0.8	0.2	0.1	0.0	0.0	0.0	0.0
Upper CI	99.4	81.5	53.0	25.4	12.5	11.6	10.9	8.6	6.3	6.3	2.0	1.7	3.4	3.4	3.4	3.4
Number	107	83	50	28	16	13	11	9	6	5	3	2	0	0	0	0

Table W512-53 Age Group = 31-40 Gender = FEMALE Occupational group = MANUAL Total cases = 102

	Threshold dB HL≥															
	0	5	10	15	20	25	30	35	40	45	50	55	65	75	85	95
Prevalence	**95.8**	**67.8**	**37.0**	**17.8**	**13.2**	**6.6**	**5.9**	**3.4**	**2.7**	**2.7**	**2.7**	**2.7**	**0.8**	**0.5**	**0.0**	**0.0**
Lower CI	88.2	55.0	26.8	11.7	8.2	3.5	3.0	1.6	1.2	1.2	1.2	1.2	0.3	0.1	0.0	0.0
Upper CI	98.6	78.4	48.5	26.1	20.6	12.0	11.4	6.7	5.9	5.9	5.9	5.9	2.3	1.6	3.6	3.6
Number	99	79	52	32	27	14	12	8	7	7	7	7	3	2	0	0

Table W512-54 Age Group = 41-50 Gender = FEMALE Occupational group = MANUAL Total cases = 134

	Threshold dB HL≥															
	0	5	10	15	20	25	30	35	40	45	50	55	65	75	85	95
Prevalence	**97.9**	**83.0**	**60.0**	**34.0**	**20.2**	**15.0**	**9.6**	**7.3**	**6.9**	**5.6**	**5.0**	**4.8**	**2.6**	**1.2**	**1.0**	**0.6**
Lower CI	92.7	73.9	50.4	25.8	14.0	9.8	6.0	4.5	4.2	3.5	3.1	2.9	1.5	0.5	0.4	0.2
Upper CI	99.4	89.3	69.0	43.4	28.3	22.2	15.2	11.6	11.1	8.9	7.8	7.6	4.3	2.8	2.6	1.7
Number	132	116	88	57	39	31	24	20	19	17	16	15	9	5	4	3

Table W512-55 Age Group = 51-60 Gender = FEMALE Occupational group = MANUAL Total cases = 166

	Threshold dB HL≥															
	0	5	10	15	20	25	30	35	40	45	50	55	65	75	85	95
Prevalence	**100.0**	**93.6**	**71.5**	**49.3**	**31.1**	**22.7**	**17.8**	**13.7**	**9.4**	**6.1**	**4.4**	**2.7**	**2.1**	**0.6**	**0.5**	**0.3**
Lower CI	97.7	86.8	62.1	40.9	24.2	16.7	12.5	9.2	5.9	3.8	2.5	1.5	1.0	0.3	0.2	0.1
Upper CI	100.0	97.0	79.3	57.8	39.1	30.1	24.9	19.9	14.7	9.6	7.7	4.5	4.1	1.4	1.2	1.1
Number	166	158	133	104	75	58	46	36	27	24	18	15	11	4	3	2

Table W512-56 Age Group = 61-70 Gender = FEMALE Occupational group = MANUAL Total cases = 124

	Threshold dB HL≥															
	0	5	10	15	20	25	30	35	40	45	50	55	65	75	85	95
Prevalence	**100.0**	**95.7**	**78.7**	**64.9**	**40.2**	**28.2**	**23.3**	**19.5**	**15.2**	**12.1**	**11.5**	**9.6**	**7.8**	**3.7**	**1.9**	**1.9**
Lower CI	97.0	88.8	67.8	53.9	31.5	21.8	18.6	16.5	10.8	8.0	7.3	5.9	4.4	2.7	1.4	1.4
Upper CI	100.0	98.4	86.6	74.5	49.5	35.6	28.6	22.9	21.0	18.1	17.5	15.4	13.5	5.1	2.4	2.4
Number	124	119	104	91	68	53	47	43	36	29	27	23	16	9	2	2

Prevalence by age group, gender and occupational group for the worse ear over 0.5, 1 and 2 kHz

Table W512-57 Age Group = 71-80 Gender = FEMALE Occupational group = MANUAL Total cases = 74

	Threshold dB HL≥ 0	5	10	15	20	25	30	35	40	45	50	55	65	75	85	95
Prevalence	**100.0**	**100.0**	**100.0**	**91.4**	**70.0**	**59.7**	**45.9**	**45.5**	**38.4**	**33.9**	**21.7**	**16.2**	**9.4**	**5.8**	**2.3**	**2.3**
Lower CI	95.1	95.1	95.1	79.2	53.3	44.5	33.9	33.5	26.6	22.7	15.0	9.6	5.8	3.3	1.0	1.0
Upper CI	100.0	100.0	100.0	96.7	82.7	73.2	58.3	58.0	51.7	47.2	30.4	25.9	14.9	10.0	5.0	5.0
Number	74	74	74	72	65	58	52	51	42	37	32	24	16	11	5	5

Table W512-58 Age Group = 18-30 Gender = MALE Occupational group = MANUAL Total cases = 109

	Threshold dB HL≥ 0	5	10	15	20	25	30	35	40	45	50	55	65	75	85	95
Prevalence	**93.9**	**68.6**	**39.6**	**20.2**	**10.8**	**4.5**	**2.9**	**1.7**	**0.8**	**0.5**	**0.5**	**0.3**	**0.3**	**0.1**	**0.1**	**0.1**
Lower CI	85.7	55.7	28.4	12.2	5.2	2.0	1.4	0.9	0.3	0.2	0.2	0.1	0.1	0.0	0.0	0.0
Upper CI	97.5	79.1	52.0	31.6	20.9	9.7	6.0	3.2	2.0	1.6	1.6	1.5	1.5	0.4	0.4	0.4
Number	104	79	51	33	21	13	11	9	4	3	3	2	2	1	1	1

Table W512-59 Age Group = 31-40 Gender = MALE Occupational group = MANUAL Total cases = 80

	Threshold dB HL≥ 0	5	10	15	20	25	30	35	40	45	50	55	65	75	85	95
Prevalence	**98.3**	**69.8**	**42.8**	**31.6**	**18.6**	**13.1**	**9.3**	**4.1**	**3.8**	**3.3**	**3.1**	**2.7**	**1.9**	**1.9**	**1.9**	**1.9**
Lower CI	95.3	55.0	29.0	19.9	10.1	6.6	4.2	1.5	1.4	1.0	0.9	0.7	0.3	0.3	0.3	0.3
Upper CI	99.4	81.3	57.8	46.3	31.7	24.4	19.5	10.2	10.2	9.9	9.9	9.8	10.2	10.2	10.2	10.2
Number	76	60	40	31	22	17	14	8	7	5	4	3	1	1	1	1

Table W512-60 Age Group = 41-50 Gender = MALE Occupational group = MANUAL Total cases = 127

	Threshold dB HL≥ 0	5	10	15	20	25	30	35	40	45	50	55	65	75	85	95
Prevalence	**99.3**	**82.0**	**64.4**	**41.3**	**25.5**	**21.0**	**16.2**	**13.1**	**10.4**	**8.1**	**6.8**	**5.7**	**2.3**	**0.9**	**0.5**	**0.0**
Lower CI	96.4	72.1	53.3	31.4	18.7	14.7	10.9	8.8	6.4	4.4	3.5	2.7	0.7	0.3	0.1	0.0
Upper CI	99.9	89.0	74.2	52.0	33.8	29.1	23.4	19.0	16.5	14.3	12.8	11.6	7.7	2.7	1.7	2.9
Number	126	111	89	63	49	39	32	28	22	16	13	11	4	3	2	0

Table W512-61 Age Group = 51-60 Gender = MALE Occupational group = MANUAL Total cases = 195

	Threshold dB HL≥ 0	5	10	15	20	25	30	35	40	45	50	55	65	75	85	95
Prevalence	**99.7**	**92.5**	**75.7**	**54.1**	**39.7**	**29.5**	**22.5**	**19.6**	**14.9**	**10.9**	**9.1**	**8.3**	**4.5**	**3.2**	**2.0**	**1.6**
Lower CI	98.1	85.9	67.1	46.3	32.8	23.5	17.7	15.0	11.1	7.8	6.3	5.5	2.6	1.7	0.9	0.6
Upper CI	99.9	96.2	82.6	61.6	47.1	36.2	28.2	25.3	19.8	15.0	13.0	12.3	7.6	5.9	4.6	3.9
Number	194	186	162	129	102	81	68	59	46	37	31	28	17	12	7	6

Table W512-62 Age Group = 61-70 Gender = MALE Occupational group = MANUAL Total cases = 173

	Threshold dB HL≥ 0	5	10	15	20	25	30	35	40	45	50	55	65	75	85	95
Prevalence	**100.0**	**94.1**	**81.7**	**68.6**	**56.6**	**47.0**	**35.1**	**28.1**	**21.9**	**17.8**	**15.0**	**11.3**	**8.6**	**3.3**	**2.4**	**1.0**
Lower CI	97.8	87.0	71.8	57.4	45.9	38.7	27.6	21.5	16.0	12.3	9.8	6.8	4.6	2.0	1.3	0.4
Upper CI	100.0	97.5	88.7	78.1	66.6	55.6	43.4	35.8	29.2	25.0	22.4	18.0	15.6	5.5	4.5	2.8
Number	173	167	155	139	120	106	88	76	62	51	41	34	25	14	10	4

Table W512-63 Age Group = 71-80 Gender = MALE Occupational group = MANUAL Total cases = 72

	Threshold dB HL≥ 0	5	10	15	20	25	30	35	40	45	50	55	65	75	85	95
Prevalence	**100.0**	**100.0**	**94.2**	**87.7**	**83.0**	**59.9**	**50.8**	**42.8**	**35.8**	**27.4**	**24.6**	**22.1**	**16.9**	**9.3**	**5.4**	**5.0**
Lower CI	94.9	94.9	72.4	67.8	63.7	46.6	37.3	29.9	23.1	19.5	16.9	15.0	10.8	5.3	3.0	2.6
Upper CI	100.0	100.0	99.0	96.0	93.2	72.0	64.3	56.8	50.9	37.1	34.3	31.3	25.6	15.8	9.6	9.1
Number	72	72	71	67	63	56	50	44	38	35	31	28	22	11	7	6

Prevalence by age group, gender and occupational group for the worse ear over 0.25, 0.5 and 1 kHz

Table W2551-1 Age Group = 18-80 Gender = OVERALL Occupational group = OVERALL Total cases = 2618

Threshold dB HL≥	0	5	10	15	20	25	30	35	40	45	50	55	65	75	85	95
**Prevalence**	**96.6**	**82.4**	**56.3**	**35.8**	**23.9**	**17.8**	**13.2**	**10.0**	**7.8**	**6.3**	**4.9**	**4.0**	**2.4**	**1.5**	**0.8**	**0.4**
Lower CI	95.3	80.4	54.0	33.9	22.3	16.4	12.1	9.1	7.0	5.5	4.3	3.5	2.0	1.2	0.6	0.3
Upper CI	97.5	84.3	58.5	37.7	25.5	19.3	14.4	11.0	8.7	7.1	5.6	4.6	2.9	1.8	1.2	0.7
Number	2568	2301	1749	1266	944	752	597	488	409	340	287	239	156	94	56	29

Table W2551-2 Age Group = 18-80 Gender = FEMALE Occupational group = OVERALL Total cases = 1364

Threshold dB HL≥	0	5	10	15	20	25	30	35	40	45	50	55	65	75	85	95
**Prevalence**	**96.7**	**82.5**	**57.2**	**35.2**	**24.7**	**18.1**	**13.9**	**10.7**	**8.5**	**6.8**	**5.3**	**4.2**	**2.5**	**1.5**	**0.8**	**0.4**
Lower CI	95.1	79.9	54.4	32.8	22.6	16.3	12.3	9.4	7.4	5.8	4.5	3.5	2.0	1.2	0.6	0.2
Upper CI	97.8	84.9	60.1	37.7	26.9	20.0	15.6	12.2	9.8	7.9	6.2	5.0	3.2	2.1	1.2	0.7
Number	1336	1195	910	640	484	383	311	260	226	190	161	132	89	53	30	16

Table W2551-3 Age Group = 18-80 Gender = MALE Occupational group = OVERALL Total cases = 1254

Threshold dB HL≥	0	5	10	15	20	25	30	35	40	45	50	55	65	75	85	95
**Prevalence**	**96.5**	**82.3**	**55.2**	**36.4**	**22.9**	**17.5**	**12.4**	**9.2**	**7.0**	**5.6**	**4.5**	**3.8**	**2.2**	**1.4**	**0.9**	**0.5**
Lower CI	94.3	79.1	51.7	33.5	20.6	15.4	10.9	7.9	5.9	4.7	3.7	3.0	1.7	0.9	0.5	0.2
Upper CI	97.8	85.2	58.6	39.5	25.5	19.7	14.1	10.6	8.2	6.8	5.5	4.6	2.9	2.0	1.5	1.1
Number	1232	1106	839	626	460	369	286	228	183	150	126	107	67	41	26	13

Table W2551-4 Age Group = 18-30 Gender = OVERALL Occupational group = OVERALL Total cases = 401

Threshold dB HL≥	0	5	10	15	20	25	30	35	40	45	50	55	65	75	85	95
**Prevalence**	**91.3**	**68.1**	**35.3**	**14.9**	**8.4**	**6.4**	**3.4**	**2.5**	**1.5**	**1.5**	**0.9**	**0.2**	**0.1**	**0.0**	**0.0**	**0.0**
Lower CI	87.0	62.4	30.0	11.4	6.0	4.3	2.3	1.6	0.9	0.8	0.5	0.1	0.0	0.0	0.0	0.0
Upper CI	94.3	73.4	40.8	19.2	11.7	9.6	5.1	3.9	2.6	2.6	1.7	0.6	0.5	0.1	0.1	0.1
Number	376	295	168	83	58	43	32	26	17	16	12	5	2	1	1	1

Table W2551-5 Age Group = 31-40 Gender = OVERALL Occupational group = OVERALL Total cases = 327

Threshold dB HL≥	0	5	10	15	20	25	30	35	40	45	50	55	65	75	85	95
**Prevalence**	**96.2**	**75.2**	**42.4**	**23.0**	**12.3**	**8.4**	**5.8**	**4.2**	**3.9**	**2.6**	**1.8**	**1.8**	**1.0**	**0.7**	**0.7**	**0.6**
Lower CI	93.1	69.0	36.2	18.2	8.8	5.8	3.7	2.5	2.3	1.3	0.8	0.8	0.3	0.2	0.1	0.1
Upper CI	97.9	80.5	48.8	28.7	16.8	12.0	9.1	6.9	6.6	5.0	3.7	3.7	3.0	3.0	3.0	3.1
Number	314	255	160	95	62	45	29	22	20	14	10	10	5	3	2	1

Table W2551-6 Age Group = 41-50 Gender = OVERALL Occupational group = OVERALL Total cases = 444

Threshold dB HL≥	0	5	10	15	20	25	30	35	40	45	50	55	65	75	85	95
**Prevalence**	**99.2**	**84.4**	**55.6**	**35.2**	**18.8**	**13.7**	**9.8**	**8.4**	**6.2**	**5.4**	**4.6**	**3.6**	**1.4**	**0.6**	**0.4**	**0.1**
Lower CI	97.7	80.3	50.7	30.8	15.6	11.0	7.6	6.4	4.5	3.8	3.2	2.4	0.9	0.3	0.2	0.0
Upper CI	99.7	87.8	60.4	39.9	22.5	16.9	12.6	11.0	8.4	7.5	6.5	5.3	2.2	1.4	1.0	0.4
Number	440	381	270	184	115	88	67	59	46	40	36	30	16	6	5	2

Table W2551-7 Age Group = 51-60 Gender = OVERALL Occupational group = OVERALL Total cases = 666

Threshold dB HL≥	0	5	10	15	20	25	30	35	40	45	50	55	65	75	85	95
**Prevalence**	**99.5**	**91.5**	**68.1**	**42.7**	**28.4**	**20.2**	**14.8**	**11.6**	**9.0**	**6.3**	**5.4**	**4.0**	**2.4**	**1.7**	**1.0**	**0.6**
Lower CI	98.5	88.8	64.0	38.8	25.1	17.5	12.5	9.6	7.3	5.0	4.1	3.0	1.7	1.0	0.6	0.3
Upper CI	99.8	93.6	71.9	46.7	31.9	23.2	17.4	13.9	11.1	8.0	7.0	5.4	3.4	2.6	1.8	1.2
Number	662	616	486	346	255	197	149	123	104	79	66	53	36	23	15	10

Prevalence by age group, gender and occupational group for the worse ear over 0.25, 0.5 and 1 kHz

Table W2551-8 Age Group = 61-70 Gender = OVERALL Occupational group = OVERALL Total cases = 515

	Threshold dB HL≥															
	**0**	**5**	**10**	**15**	**20**	**25**	**30**	**35**	**40**	**45**	**50**	**55**	**65**	**75**	**85**	**95**
Prevalence	**99.1**	**94.6**	**78.2**	**57.8**	**41.8**	**30.4**	**24.3**	**16.7**	**13.2**	**11.1**	**8.9**	**7.5**	**5.4**	**2.6**	**1.6**	**0.9**
Lower CI	96.9	91.6	73.4	52.6	37.2	26.3	20.7	14.1	10.8	9.0	7.0	5.7	3.8	1.9	1.0	0.5
Upper CI	99.7	96.6	82.4	62.9	46.6	34.8	28.2	19.8	16.1	13.7	11.3	9.8	7.5	3.7	2.3	1.6
Number	513	492	421	338	260	214	180	140	121	105	87	71	55	31	19	10

Table W2551-9 Age Group = 71-80 Gender = OVERALL Occupational group = OVERALL Total cases = 265

	Threshold dB HL≥															
	**0**	**5**	**10**	**15**	**20**	**25**	**30**	**35**	**40**	**45**	**50**	**55**	**65**	**75**	**85**	**95**
Prevalence	**99.1**	**99.0**	**88.7**	**74.3**	**63.5**	**50.9**	**40.8**	**32.2**	**25.4**	**21.3**	**15.9**	**14.6**	**8.9**	**6.4**	**2.8**	**0.9**
Lower CI	96.8	96.8	82.4	67.2	55.5	43.5	33.8	26.0	20.4	16.4	12.5	11.3	6.4	4.3	1.6	0.3
Upper CI	99.8	99.7	92.9	80.2	70.7	58.2	48.1	39.2	31.1	27.0	19.9	18.6	12.3	9.4	4.7	2.1
Number	263	262	244	220	194	165	140	118	101	86	76	70	42	30	14	5

Table W2551-10 Age Group = 18-80 Gender = OVERALL Occupational group = NON-MANUAL Total cases = 1166

	Threshold dB HL≥															
	**0**	**5**	**10**	**15**	**20**	**25**	**30**	**35**	**40**	**45**	**50**	**55**	**65**	**75**	**85**	**95**
Prevalence	**95.2**	**78.9**	**49.3**	**30.4**	**20.7**	**15.2**	**11.5**	**8.8**	**6.1**	**5.1**	**3.6**	**2.7**	**1.6**	**1.1**	**0.5**	**0.4**
Lower CI	93.0	75.8	46.0	27.7	18.5	13.4	10.1	7.6	5.0	4.1	2.9	2.1	1.2	0.8	0.3	0.2
Upper CI	96.7	81.7	52.6	33.3	23.0	17.1	13.0	10.1	7.3	6.3	4.4	3.5	2.0	1.6	0.8	0.7
Number	1136	985	684	463	344	268	210	170	139	118	95	77	52	35	20	14

Table W2551-11 Age Group = 18-80 Gender = OVERALL Occupational group = MANUAL Total cases = 1448

	Threshold dB HL≥															
	**0**	**5**	**10**	**15**	**20**	**25**	**30**	**35**	**40**	**45**	**50**	**55**	**65**	**75**	**85**	**95**
Prevalence	**97.7**	**86.3**	**62.4**	**40.6**	**27.3**	**19.8**	**14.8**	**10.9**	**8.8**	**6.9**	**5.8**	**4.8**	**2.8**	**1.6**	**1.0**	**0.5**
Lower CI	95.8	83.5	58.9	37.7	24.9	17.8	13.2	9.6	7.6	5.9	4.9	4.0	2.3	1.2	0.7	0.3
Upper CI	98.7	88.7	65.7	43.6	29.8	22.1	16.6	12.3	10.1	8.1	6.8	5.7	3.5	2.1	1.5	0.9
Number	1428	1314	1063	802	600	484	387	318	270	222	192	162	104	59	36	15

Table W2551-12 Age Group = 18-30 Gender = FEMALE Occupational group = OVERALL Total cases = 224

	Threshold dB HL≥															
	**0**	**5**	**10**	**15**	**20**	**25**	**30**	**35**	**40**	**45**	**50**	**55**	**65**	**75**	**85**	**95**
Prevalence	**93.3**	**68.0**	**34.4**	**13.6**	**8.4**	**5.2**	**4.5**	**3.0**	**2.4**	**2.3**	**1.3**	**0.1**	**0.0**	**0.0**	**0.0**	**0.0**
Lower CI	88.2	60.5	28.0	9.8	5.8	3.3	2.8	1.8	1.3	1.2	0.6	0.0	0.0	0.0	0.0	0.0
Upper CI	96.3	74.6	41.4	18.6	12.1	7.9	7.2	5.1	4.5	4.3	2.7	0.6	1.7	1.7	1.7	1.7
Number	212	162	93	46	34	23	20	15	12	11	7	1	0	0	0	0

Table W2551-13 Age Group = 31-40 Gender = FEMALE Occupational group = OVERALL Total cases = 185

	Threshold dB HL≥															
	**0**	**5**	**10**	**15**	**20**	**25**	**30**	**35**	**40**	**45**	**50**	**55**	**65**	**75**	**85**	**95**
Prevalence	**93.8**	**71.1**	**43.0**	**21.3**	**11.1**	**7.7**	**4.1**	**3.6**	**3.6**	**1.5**	**1.5**	**1.5**	**0.6**	**0.3**	**0.2**	**0.0**
Lower CI	88.0	62.9	35.2	15.6	7.4	4.6	2.1	1.8	1.8	0.7	0.7	0.7	0.2	0.1	0.0	0.0
Upper CI	96.9	78.1	51.1	28.3	16.3	12.5	7.6	7.1	7.1	3.4	3.4	3.4	1.7	1.2	1.2	2.0
Number	176	141	94	52	34	23	13	11	11	6	6	6	3	2	1	0

Table W2551-14 Age Group = 41-50 Gender = FEMALE Occupational group = OVERALL Total cases = 243

	Threshold dB HL≥															
	**0**	**5**	**10**	**15**	**20**	**25**	**30**	**35**	**40**	**45**	**50**	**55**	**65**	**75**	**85**	**95**
Prevalence	**99.3**	**84.5**	**54.4**	**32.5**	**17.2**	**11.9**	**8.4**	**7.3**	**5.0**	**4.5**	**3.7**	**3.3**	**1.4**	**0.6**	**0.3**	**0.1**
Lower CI	97.2	78.8	47.8	26.8	13.2	8.8	5.9	5.0	3.2	2.8	2.3	2.0	0.7	0.2	0.1	0.0
Upper CI	99.8	88.8	60.8	38.7	22.1	15.9	11.9	10.8	7.8	7.1	6.0	5.4	2.7	2.0	1.2	0.7
Number	241	209	144	93	56	41	32	28	21	19	17	16	8	3	2	1

Prevalence by age group, gender and occupational group for the worse ear over 0.25, 0.5 and 1 kHz

Table W2551-22 Age Group = 61-70 Gender = MALE Occupational group = OVERALL Total cases = 278

	Threshold dB HL≥															
	0	5	10	15	20	25	30	35	40	45	50	55	65	75	85	95
Prevalence	**100.0**	**95.1**	**77.8**	**57.9**	**40.7**	**31.3**	**26.0**	**17.4**	**13.1**	**9.8**	**8.3**	**6.9**	**3.7**	**2.3**	**1.2**	**0.7**
Lower CI	98.6	90.7	70.5	50.1	34.0	26.0	21.3	13.6	9.9	7.5	6.1	4.9	2.5	1.4	0.7	0.3
Upper CI	100.0	97.5	83.6	65.2	47.7	37.2	31.4	21.9	17.2	12.7	11.3	9.7	5.4	3.6	2.2	1.7
Number	278	266	226	183	141	116	99	73	60	48	41	33	22	15	9	5

Table W2551-23 Age Group = 71-80 Gender = MALE Occupational group = OVERALL Total cases = 126

	Threshold dB HL≥															
	0	5	10	15	20	25	30	35	40	45	50	55	65	75	85	95
Prevalence	**98.7**	**98.7**	**85.0**	**73.4**	**56.6**	**43.6**	**33.3**	**23.1**	**19.3**	**17.1**	**13.4**	**13.4**	**8.6**	**5.6**	**2.4**	**0.7**
Lower CI	92.7	92.7	75.9	63.8	46.8	34.4	24.6	16.5	13.2	11.3	9.4	9.4	5.2	3.2	1.0	0.2
Upper CI	99.8	99.8	91.1	81.3	65.9	53.3	43.2	31.3	27.4	25.0	18.9	18.9	14.0	9.7	5.7	2.6
Number	125	125	113	101	84	68	53	42	35	31	28	28	17	12	5	2

Table W2551-24 Age Group = 18-30 Gender = OVERALL Occupational group = NON-MANUAL Total cases = 181

	Threshold dB HL≥															
	0	5	10	15	20	25	30	35	40	45	50	55	65	75	85	95
Prevalence	**88.0**	**61.0**	**26.5**	**10.6**	**5.4**	**4.0**	**2.1**	**1.8**	**1.2**	**1.1**	**0.7**	**0.1**	**0.0**	**0.0**	**0.0**	**0.0**
Lower CI	80.4	52.8	19.5	6.1	2.7	1.7	1.2	0.9	0.6	0.5	0.3	0.0	0.0	0.0	0.0	0.0
Upper CI	92.9	68.7	34.9	17.8	10.5	9.4	3.8	3.6	2.8	2.6	1.4	2.2	2.1	2.1	2.1	2.1
Number	166	117	57	23	16	12	10	9	6	5	4	1	0	0	0	0

Table W2551-25 Age Group = 31-40 Gender = OVERALL Occupational group = NON-MANUAL Total cases = 146

	Threshold dB HL≥															
	0	5	10	15	20	25	30	35	40	45	50	55	65	75	85	95
Prevalence	**94.4**	**71.4**	**37.9**	**19.1**	**9.0**	**7.0**	**4.9**	**4.6**	**4.0**	**1.8**	**0.0**	**0.0**	**0.0**	**0.0**	**0.0**	**0.0**
Lower CI	89.2	62.2	30.0	13.1	5.1	3.6	2.2	2.0	1.6	0.4	0.0	0.0	0.0	0.0	0.0	0.0
Upper CI	97.2	79.1	46.4	27.1	15.4	13.0	10.6	10.5	9.8	7.8	2.6	2.6	2.6	2.6	2.6	2.6
Number	138	107	60	30	17	12	9	8	7	2	0	0	0	0	0	0

Table W2551-26 Age Group = 41-50 Gender = OVERALL Occupational group = NON-MANUAL Total cases = 186

	Threshold dB HL≥															
	0	5	10	15	20	25	30	35	40	45	50	55	65	75	85	95
Prevalence	**98.5**	**81.3**	**46.7**	**30.0**	**17.0**	**11.9**	**9.5**	**6.9**	**5.8**	**5.1**	**4.6**	**2.5**	**0.7**	**0.1**	**0.1**	**0.1**
Lower CI	93.4	74.1	39.2	23.8	12.6	7.9	5.9	4.0	3.2	2.8	2.3	0.9	0.5	0.0	0.0	0.0
Upper CI	99.7	86.8	54.4	37.0	22.5	17.5	14.9	11.7	10.4	9.4	8.9	6.9	0.9	0.7	0.7	0.7
Number	184	153	91	60	34	25	17	14	11	10	9	6	4	1	1	1

Table W2551-27 Age Group = 51-60 Gender = OVERALL Occupational group = NON-MANUAL Total cases = 309

	Threshold dB HL≥															
	0	5	10	15	20	25	30	35	40	45	50	55	65	75	85	95
Prevalence	**99.4**	**90.3**	**60.8**	**32.6**	**21.0**	**15.6**	**10.4**	**7.7**	**5.6**	**4.9**	**4.1**	**3.1**	**1.7**	**1.5**	**0.9**	**0.7**
Lower CI	97.6	86.3	54.2	27.5	17.4	12.7	8.1	5.9	4.1	3.4	2.7	2.0	1.0	0.8	0.4	0.3
Upper CI	99.8	93.3	67.1	38.1	25.1	18.9	13.4	10.0	7.7	7.0	6.1	4.8	3.0	2.8	1.9	1.7
Number	307	279	199	127	97	77	55	44	37	30	25	20	13	11	7	6

Table W2551-28 Age Group = 61-70 Gender = OVERALL Occupational group = NON-MANUAL Total cases = 222

	Threshold dB HL≥															
	0	5	10	15	20	25	30	35	40	45	50	55	65	75	85	95
Prevalence	**99.2**	**93.7**	**74.8**	**54.3**	**40.6**	**26.5**	**21.7**	**15.1**	**11.8**	**10.1**	**7.0**	**6.0**	**3.7**	**2.5**	**1.4**	**1.1**
Lower CI	95.4	88.5	66.3	45.9	32.9	21.5	17.4	11.8	9.0	7.5	5.1	4.3	2.5	1.4	0.8	0.5
Upper CI	99.9	96.7	81.7	62.5	48.9	32.1	26.7	19.2	15.2	13.4	9.5	8.3	5.4	4.5	2.6	2.3
Number	221	210	172	130	98	77	65	51	43	39	31	26	19	12	8	6

Prevalence by age group, gender and occupational group for the worse ear over 0.25, 0.5 and 1 kHz

Table W2551-15 Age Group = 51-60 Gender = FEMALE Occupational group = OVERALL Total cases = 336

	Threshold dB HL≥															
	0	5	10	15	20	25	30	35	40	45	50	55	65	75	85	95
Prevalence	**99.4**	**94.4**	**69.9**	**41.2**	**28.7**	**20.5**	**15.2**	**11.0**	**8.6**	**5.9**	**5.2**	**3.8**	**2.0**	**1.5**	**0.9**	**0.8**
Lower CI	98.0	90.9	64.2	35.9	24.1	16.6	12.0	8.5	6.3	4.2	3.6	2.5	1.3	0.9	0.4	0.3
Upper CI	99.8	96.6	75.0	46.7	33.8	24.9	19.2	14.3	11.6	8.2	7.5	5.7	3.1	2.7	1.8	1.8
Number	334	320	253	175	131	101	78	63	55	42	37	29	20	14	8	7

Table W2551-16 Age Group = 61-70 Gender = FEMALE Occupational group = OVERALL Total cases = 237

	Threshold dB HL≥															
	0	5	10	15	20	25	30	35	40	45	50	55	65	75	85	95
Prevalence	**98.3**	**94.2**	**78.7**	**57.8**	**42.7**	**29.6**	**22.8**	**16.2**	**13.3**	**12.4**	**9.5**	**7.9**	**6.8**	**3.0**	**1.8**	**1.0**
Lower CI	94.3	89.5	72.1	50.7	36.3	23.8	17.9	12.7	10.0	9.1	6.6	5.3	4.3	1.9	1.1	0.4
Upper CI	99.5	96.8	84.1	64.7	49.3	36.1	28.6	20.5	17.6	16.6	13.3	11.7	10.7	4.7	3.1	2.4
Number	235	226	195	155	119	98	81	67	61	57	46	38	33	16	10	5

Table W2551-17 Age Group = 71-80 Gender = FEMALE Occupational group = OVERALL Total cases = 139

	Threshold dB HL≥															
	0	5	10	15	20	25	30	35	40	45	50	55	65	75	85	95
Prevalence	**99.4**	**99.2**	**90.7**	**74.5**	**67.7**	**55.6**	**45.8**	**38.3**	**29.5**	**23.9**	**17.7**	**15.5**	**9.0**	**6.8**	**3.1**	**0.9**
Lower CI	96.8	96.9	81.1	64.7	56.0	45.0	35.9	29.1	22.8	17.4	13.0	11.0	5.8	4.0	1.6	0.3
Upper CI	99.9	99.8	95.7	82.4	77.5	65.7	56.0	48.3	37.2	31.9	23.6	21.4	13.8	11.3	5.9	3.1
Number	138	137	131	119	110	97	87	76	66	55	48	42	25	18	9	3

Table W2551-18 Age Group = 18-30 Gender = MALE Occupational group = OVERALL Total cases = 177

	Threshold dB HL≥															
	0	5	10	15	20	25	30	35	40	45	50	55	65	75	85	95
Prevalence	**89.2**	**68.5**	**35.9**	**16.1**	**8.3**	**7.6**	**2.1**	**1.9**	**0.6**	**0.6**	**0.6**	**0.4**	**0.2**	**0.0**	**0.0**	**0.0**
Lower CI	81.4	59.5	28.0	10.7	4.7	4.1	1.1	0.9	0.2	0.2	0.2	0.1	0.1	0.0	0.0	0.0
Upper CI	93.9	76.2	44.7	23.5	14.3	13.8	4.1	3.8	1.4	1.4	1.4	1.2	1.0	0.2	0.2	0.2
Number	164	133	75	37	24	20	12	11	5	5	5	4	2	1	1	1

Table W2551-19 Age Group = 31-40 Gender = MALE Occupational group = OVERALL Total cases = 142

	Threshold dB HL≥															
	0	5	10	15	20	25	30	35	40	45	50	55	65	75	85	95
Prevalence	**98.8**	**79.6**	**41.7**	**25.2**	**13.7**	**9.4**	**7.9**	**4.8**	**4.2**	**3.9**	**2.1**	**2.1**	**1.5**	**1.2**	**1.2**	**1.2**
Lower CI	96.8	69.8	32.2	17.7	8.2	5.5	4.2	2.3	1.9	1.6	0.7	0.7	0.3	0.2	0.2	0.2
Upper CI	99.6	86.8	51.9	34.7	21.8	15.7	14.3	9.8	9.2	8.9	6.4	6.4	6.3	6.5	6.5	6.5
Number	138	114	66	43	28	22	16	11	9	8	4	4	2	1	1	1

Table W2551-20 Age Group = 41-50 Gender = MALE Occupational group = OVERALL Total cases = 201

	Threshold dB HL≥															
	0	5	10	15	20	25	30	35	40	45	50	55	65	75	85	95
Prevalence	**99.0**	**84.2**	**57.0**	**38.2**	**20.5**	**15.6**	**11.2**	**9.5**	**7.3**	**6.3**	**5.5**	**3.9**	**1.5**	**0.5**	**0.5**	**0.1**
Lower CI	96.3	77.8	49.7	31.6	15.7	11.4	7.8	6.5	4.7	3.9	3.3	2.1	0.8	0.2	0.2	0.0
Upper CI	99.8	89.1	64.0	45.4	26.4	20.9	16.0	13.8	11.3	10.1	9.1	7.0	2.6	1.6	1.6	0.6
Number	199	172	126	91	59	47	35	31	25	21	19	14	8	3	3	1

Table W2551-21 Age Group = 51-60 Gender = MALE Occupational group = OVERALL Total cases = 330

	Threshold dB HL≥															
	0	5	10	15	20	25	30	35	40	45	50	55	65	75	85	95
Prevalence	**99.5**	**88.3**	**66.1**	**44.2**	**27.9**	**19.9**	**14.2**	**12.3**	**9.4**	**6.8**	**5.5**	**4.2**	**2.8**	**1.8**	**1.3**	**0.5**
Lower CI	97.6	83.7	60.1	38.5	23.4	16.3	11.3	9.5	7.0	4.9	3.7	2.7	1.6	0.9	0.6	0.2
Upper CI	99.9	91.7	71.6	50.1	32.9	24.0	17.9	15.9	12.5	9.4	8.1	6.4	4.8	3.7	2.8	1.5
Number	328	296	233	171	124	96	71	60	49	37	29	24	16	9	7	3

Prevalence by age group, gender and occupational group for the worse ear over 0.25, 0.5 and 1 kHz

Table W2551-29 Age Group = 71-80 Gender = OVERALL Occupational group = NON-MANUAL Total cases = 122

	Threshold dB HL≥															
	0	5	10	15	20	25	30	35	40	45	50	55	65	75	85	95
Prevalence	**98.3**	**98.1**	**81.0**	**70.2**	**62.4**	**49.4**	**39.7**	**31.2**	**17.0**	**15.6**	**10.5**	**10.0**	**7.2**	**5.3**	**1.7**	**0.7**
Lower CI	94.0	94.0	69.4	58.4	50.9	42.0	32.6	25.4	11.8	10.3	7.6	7.1	4.3	2.8	0.6	0.1
Upper CI	99.5	99.4	88.9	79.8	72.6	56.9	47.4	37.8	23.8	22.8	14.4	14.0	11.7	9.7	4.6	4.0
Number	120	119	105	93	82	65	54	44	35	32	26	24	16	11	4	1

Table W2551-30 Age Group = 18-30 Gender = OVERALL Occupational group = MANUAL Total cases = 218

	Threshold dB HL≥															
	0	5	10	15	20	25	30	35	40	45	50	55	65	75	85	95
Prevalence	**93.5**	**77.0**	**44.7**	**19.7**	**11.1**	**8.4**	**4.4**	**2.9**	**1.7**	**1.7**	**0.9**	**0.3**	**0.2**	**0.0**	**0.0**	**0.0**
Lower CI	86.4	69.0	36.2	13.8	7.1	5.0	2.6	1.6	0.8	0.8	0.4	0.1	0.0	0.0	0.0	0.0
Upper CI	97.0	83.5	53.6	27.4	17.1	13.8	7.5	5.3	3.7	3.7	2.0	0.9	0.7	0.2	0.2	0.2
Number	208	178	111	60	42	31	22	17	11	11	8	4	2	1	1	1

Table W2551-31 Age Group = 31-40 Gender = OVERALL Occupational group = MANUAL Total cases = 181

	Threshold dB HL≥															
	0	5	10	15	20	25	30	35	40	45	50	55	65	75	85	95
Prevalence	**98.6**	**80.7**	**45.9**	**25.5**	**15.0**	**9.9**	**7.0**	**3.5**	**3.4**	**3.0**	**2.8**	**2.8**	**1.5**	**1.2**	**1.1**	**0.9**
Lower CI	96.0	71.9	36.6	18.9	10.0	6.1	3.9	1.8	1.7	1.5	1.3	1.3	0.5	0.3	0.2	0.2
Upper CI	99.5	87.2	55.6	33.4	22.0	15.5	12.5	6.6	6.5	6.2	5.9	5.9	4.8	4.8	4.9	5.0
Number	176	148	100	65	45	33	20	14	13	12	10	10	5	3	2	1

Table W2551-32 Age Group = 41-50 Gender = OVERALL Occupational group = MANUAL Total cases = 258

	Threshold dB HL≥															
	0	5	10	15	20	25	30	35	40	45	50	55	65	75	85	95
Prevalence	**99.2**	**85.8**	**64.0**	**41.2**	**23.3**	**16.1**	**11.7**	**10.4**	**7.5**	**6.8**	**5.7**	**4.5**	**1.8**	**0.9**	**0.6**	**0.1**
Lower CI	96.7	79.9	56.9	34.7	18.1	12.1	8.4	7.4	5.1	4.4	3.6	2.8	1.2	0.5	0.2	0.0
Upper CI	99.8	90.3	70.5	48.1	29.4	21.2	16.0	14.3	11.0	10.3	8.8	7.0	2.8	1.7	1.5	0.8
Number	256	228	179	124	81	63	50	45	35	30	27	24	12	5	4	1

Table W2551-33 Age Group = 51-60 Gender = OVERALL Occupational group = MANUAL Total cases = 357

	Threshold dB HL≥															
	0	5	10	15	20	25	30	35	40	45	50	55	65	75	85	95
Prevalence	**99.6**	**93.0**	**74.3**	**49.6**	**33.7**	**23.1**	**17.7**	**14.0**	**11.5**	**7.3**	**6.4**	**4.7**	**2.7**	**1.6**	**0.9**	**0.4**
Lower CI	98.1	88.7	68.2	43.8	28.6	19.0	14.1	10.9	8.8	5.3	4.4	3.2	1.7	0.8	0.4	0.1
Upper CI	99.9	95.7	79.6	55.5	39.3	27.8	22.0	17.8	14.9	10.0	9.1	6.8	4.2	3.0	1.9	1.1
Number	355	337	287	219	158	120	94	79	67	49	41	33	23	12	8	4

Table W2551-34 Age Group = 61-70 Gender = OVERALL Occupational group = MANUAL Total cases = 292

	Threshold dB HL≥															
	0	5	10	15	20	25	30	35	40	45	50	55	65	75	85	95
Prevalence	**99.0**	**95.0**	**78.8**	**61.3**	**43.9**	**32.6**	**25.7**	**17.8**	**14.6**	**11.7**	**10.2**	**8.7**	**6.5**	**2.9**	**1.9**	**1.1**
Lower CI	94.4	90.6	71.5	53.5	37.4	27.1	21.0	14.8	11.0	8.9	7.4	6.1	4.3	2.1	1.3	0.9
Upper CI	99.8	97.4	84.7	68.6	50.6	38.7	30.9	21.3	19.1	15.3	13.9	12.4	9.8	3.8	2.6	1.5
Number	291	281	248	207	162	137	115	89	78	66	56	45	36	19	11	4

Table W2551-35 Age Group = 71-80 Gender = OVERALL Occupational group = MANUAL Total cases = 142

	Threshold dB HL≥															
	0	5	10	15	20	25	30	35	40	45	50	55	65	75	85	95
Prevalence	**100.0**	**100.0**	**95.4**	**80.8**	**68.5**	**53.1**	**44.7**	**34.3**	**28.5**	**22.9**	**18.6**	**17.3**	**9.8**	**6.9**	**3.6**	**0.9**
Lower CI	97.4	97.4	86.4	70.0	57.1	42.3	35.1	26.3	20.9	15.6	13.6	12.3	6.6	4.5	2.1	0.4
Upper CI	100.0	100.0	98.5	88.4	78.0	63.7	54.6	43.2	37.7	32.2	25.1	23.8	14.4	10.4	6.1	2.3
Number	142	142	138	127	112	100	86	74	66	54	50	46	26	19	10	4

Prevalence by age group, gender and occupational group for the worse ear over 0.25, 0.5 and 1 kHz

Table W2551-36 Age Group = 18-80 Gender = FEMALE Occupational group = NON-MANUAL Total cases = 662

Threshold dB HL≥	0	5	10	15	20	25	30	35	40	45	50	55	65	75	85	95
Prevalence	**95.4**	**79.2**	**51.6**	**31.5**	**22.3**	**16.6**	**12.8**	**10.3**	**6.6**	**5.4**	**3.8**	**3.1**	**2.1**	**1.4**	**0.8**	**0.5**
Lower CI	93.0	75.5	47.7	28.2	19.7	14.6	11.1	8.8	5.3	4.3	3.1	2.4	1.6	0.9	0.4	0.2
Upper CI	97.1	82.5	55.6	35.0	25.3	18.9	14.8	12.0	8.2	6.7	4.8	3.9	2.9	2.1	1.3	1.0
Number	643	560	399	270	202	160	129	111	92	79	64	53	39	24	15	10

Table W2551-37 Age Group = 18-80 Gender = MALE Occupational group = NON-MANUAL Total cases = 504

Threshold dB HL≥	0	5	10	15	20	25	30	35	40	45	50	55	65	75	85	95
Prevalence	**94.9**	**78.6**	**46.6**	**29.2**	**18.7**	**13.5**	**9.9**	**7.0**	**5.5**	**4.8**	**3.3**	**2.3**	**0.9**	**0.9**	**0.3**	**0.2**
Lower CI	90.5	73.3	41.2	24.8	15.5	10.7	7.9	5.3	4.0	3.3	2.3	1.4	0.6	0.5	0.1	0.1
Upper CI	97.3	83.1	52.0	34.0	22.5	16.8	12.4	9.2	7.6	6.8	4.9	3.7	1.5	1.5	0.7	0.5
Number	493	425	285	193	142	108	81	59	47	39	31	24	13	11	5	4

Table W2551-38 Age Group = 18-80 Gender = FEMALE Occupational group = MANUAL Total cases = 701

Threshold dB HL≥	0	5	10	15	20	25	30	35	40	45	50	55	65	75	85	95
Prevalence	**97.8**	**86.9**	**63.2**	**39.5**	**27.6**	**19.3**	**15.3**	**11.3**	**9.5**	**7.4**	**6.0**	**4.8**	**2.7**	**1.6**	**0.9**	**0.4**
Lower CI	95.0	82.9	58.6	35.6	24.3	16.7	13.1	9.5	7.8	5.9	4.8	3.7	2.0	1.2	0.6	0.3
Upper CI	99.1	90.1	67.6	43.7	31.0	22.2	17.8	13.3	11.4	9.3	7.5	6.2	3.5	2.1	1.3	0.6
Number	692	634	510	369	282	223	182	149	134	111	97	79	50	29	15	6

Table W2551-39 Age Group = 18-80 Gender = MALE Occupational group = MANUAL Total cases = 747

Threshold dB HL≥	0	5	10	15	20	25	30	35	40	45	50	55	65	75	85	95
Prevalence	**97.5**	**85.7**	**61.3**	**41.8**	**26.9**	**20.4**	**14.3**	**10.4**	**8.0**	**6.3**	**5.6**	**4.7**	**3.0**	**1.7**	**1.2**	**0.6**
Lower CI	94.3	81.4	56.1	37.6	23.4	17.3	12.0	8.7	6.5	5.1	4.4	3.7	2.2	1.1	0.7	0.2
Upper CI	98.9	89.2	66.3	46.1	30.8	24.0	17.0	12.4	9.8	7.9	7.0	6.1	4.2	2.7	2.1	1.6
Number	736	680	553	433	318	261	205	169	136	111	95	83	54	30	21	9

Table W2551-40 Age Group = 18-30 Gender = FEMALE Occupational group = NON-MANUAL Total cases = 115

Threshold dB HL≥	0	5	10	15	20	25	30	35	40	45	50	55	65	75	85	95
Prevalence	**90.9**	**60.8**	**27.8**	**9.1**	**6.8**	**4.1**	**3.7**	**3.1**	**2.2**	**2.0**	**1.2**	**0.0**	**0.0**	**0.0**	**0.0**	**0.0**
Lower CI	83.7	51.0	20.4	5.6	4.1	2.3	2.0	1.5	1.0	0.8	0.6	0.0	0.0	0.0	0.0	0.0
Upper CI	95.1	69.8	36.7	14.3	11.0	7.4	6.9	6.5	5.1	4.9	2.6	3.2	3.2	3.2	3.2	3.2
Number	106	74	39	16	13	9	8	7	5	4	3	0	0	0	0	0

Table W2551-41 Age Group = 31-40 Gender = FEMALE Occupational group = NON-MANUAL Total cases = 84

Threshold dB HL≥	0	5	10	15	20	25	30	35	40	45	50	55	65	75	85	95
Prevalence	**90.6**	**66.1**	**38.9**	**16.8**	**8.2**	**6.4**	**3.5**	**3.5**	**3.5**	**0.0**	**0.0**	**0.0**	**0.0**	**0.0**	**0.0**	**0.0**
Lower CI	80.7	54.4	28.5	9.8	3.8	2.5	1.1	1.1	1.1	0.0	0.0	0.0	0.0	0.0	0.0	0.0
Upper CI	95.7	76.1	50.3	27.3	16.7	15.1	10.6	10.6	10.6	4.4	4.4	4.4	4.4	4.4	4.4	4.4
Number	78	59	36	16	9	6	4	4	4	0	0	0	0	0	0	0

Table W2551-42 Age Group = 41-50 Gender = FEMALE Occupational group = NON-MANUAL Total cases = 111

Threshold dB HL≥	0	5	10	15	20	25	30	35	40	45	50	55	65	75	85	95
Prevalence	**99.5**	**81.8**	**46.3**	**31.6**	**14.2**	**10.9**	**7.8**	**5.4**	**4.0**	**2.7**	**1.6**	**1.6**	**0.6**	**0.0**	**0.0**	**0.0**
Lower CI	97.4	73.2	37.0	23.7	9.7	7.0	4.6	2.5	1.7	1.1	0.5	0.5	0.1	0.0	0.0	0.0
Upper CI	99.9	88.1	55.8	40.7	20.2	16.6	13.1	11.2	9.2	6.3	5.3	5.3	4.4	3.3	3.3	3.3
Number	110	92	55	38	18	13	8	6	4	3	2	2	1	0	0	0

Prevalence by age group, gender and occupational group for the worse ear over 0.25, 0.5 and 1 kHz

Table W2551-43 Age Group = 51-60 Gender = FEMALE Occupational group = NON-MANUAL Total cases = 172

	Threshold dB HL≥															
	0	5	10	15	20	25	30	35	40	45	50	55	65	75	85	95
Prevalence	99.5	95.2	66.6	35.7	23.8	17.1	12.0	8.9	6.6	5.8	4.7	3.8	2.6	2.2	1.1	1.1
Lower CI	97.5	91.0	58.9	28.6	18.7	13.0	8.6	6.5	4.5	3.8	2.9	2.3	1.4	1.1	0.4	0.4
Upper CI	99.9	97.5	73.6	43.4	29.9	22.1	16.4	12.0	9.4	8.7	7.4	6.4	4.8	4.5	2.8	2.8
Number	171	163	119	75	58	45	35	29	25	21	17	14	11	9	5	5

Table W2551-44 Age Group = 61-70 Gender = FEMALE Occupational group = NON-MANUAL Total cases = 113

	Threshold dB HL≥															
	0	5	10	15	20	25	30	35	40	45	50	55	65	75	85	95
Prevalence	98.5	92.8	76.7	56.0	39.9	25.8	19.9	13.2	10.3	10.0	6.7	5.5	4.4	2.3	1.9	1.3
Lower CI	92.0	84.4	67.2	46.1	31.3	19.0	14.3	9.5	7.1	6.7	4.9	4.2	3.0	1.1	0.9	0.5
Upper CI	99.7	96.9	84.1	65.4	49.2	34.2	27.0	18.0	14.8	14.5	9.0	7.1	6.3	4.5	3.9	3.3
Number	112	107	91	72	53	43	36	30	26	25	20	17	14	7	6	4

Table W2551-45 Age Group = 71-80 Gender = FEMALE Occupational group = NON-MANUAL Total cases = 67

	Threshold dB HL≥															
	0	5	10	15	20	25	30	35	40	45	50	55	65	75	85	95
Prevalence	98.6	98.2	81.8	72.5	71.2	59.1	48.8	43.5	21.1	19.2	14.2	13.3	9.4	6.3	2.9	1.3
Lower CI	92.2	92.4	64.0	54.8	53.5	49.6	39.4	35.2	14.0	12.0	9.7	8.8	5.2	2.9	1.0	0.2
Upper CI	99.7	99.6	91.9	85.2	84.1	68.0	58.2	52.3	30.6	29.4	20.4	19.6	16.4	13.3	7.8	6.9
Number	66	65	59	53	51	44	38	35	28	26	22	20	13	8	4	1

Table W2551-46 Age Group = 18-30 Gender = MALE Occupational group = NON-MANUAL Total cases = 66

	Threshold dB HL≥															
	0	5	10	15	20	25	30	35	40	45	50	55	65	75	85	95
Prevalence	84.8	61.3	24.8	12.0	3.8	3.8	0.4	0.4	0.1	0.1	0.1	0.1	0.0	0.0	0.0	0.0
Lower CI	70.4	47.9	14.1	4.9	0.8	0.8	0.1	0.1	0.0	0.0	0.0	0.0	0.0	0.0	0.0	0.0
Upper CI	92.9	73.2	39.9	26.7	17.1	17.1	1.3	1.3	5.7	5.7	5.7	5.7	5.5	5.5	5.5	5.5
Number	60	43	18	7	3	3	2	2	1	1	1	1	0	0	0	0

Table W2551-47 Age Group = 31-40 Gender = MALE Occupational group = NON-MANUAL Total cases = 62

	Threshold dB HL≥															
	0	5	10	15	20	25	30	35	40	45	50	55	65	75	85	95
Prevalence	98.6	77.4	36.8	21.8	9.8	7.6	6.3	5.7	4.4	3.8	0.0	0.0	0.0	0.0	0.0	0.0
Lower CI	-	61.8	25.7	12.7	4.3	3.0	2.2	1.8	1.1	0.8	0.0	0.0	0.0	0.0	0.0	0.0
Upper CI	-	87.8	49.6	35.0	20.9	17.8	17.0	16.9	15.9	16.0	5.8	5.8	5.8	5.8	5.8	5.8
Number	60	48	24	14	8	6	5	4	3	2	0	0	0	0	0	0

Table W2551-48 Age Group = 41-50 Gender = MALE Occupational group = NON-MANUAL Total cases = 75

	Threshold dB HL≥															
	0	5	10	15	20	25	30	35	40	45	50	55	65	75	85	95
Prevalence	97.4	80.3	47.3	28.4	20.4	13.1	11.3	8.7	7.9	7.9	7.9	3.5	0.7	0.3	0.3	0.3
Lower CI	86.3	67.8	35.5	19.4	13.1	6.7	5.4	4.1	3.5	3.5	3.5	0.8	0.4	0.0	0.0	0.0
Upper CI	99.5	88.8	59.4	39.5	30.3	24.0	22.3	17.5	16.7	16.7	16.7	13.5	1.4	1.4	1.4	1.4
Number	74	61	36	22	16	12	9	8	7	7	7	4	3	1	1	1

Table W2551-49 Age Group = 51-60 Gender = MALE Occupational group = NON-MANUAL Total cases = 137

	Threshold dB HL≥															
	0	5	10	15	20	25	30	35	40	45	50	55	65	75	85	95
Prevalence	99.1	84.8	54.3	29.0	17.8	13.8	8.7	6.3	4.6	3.9	3.5	2.2	0.6	0.6	0.6	0.3
Lower CI	95.0	77.1	43.4	22.2	13.3	10.2	5.8	3.9	2.5	2.0	1.7	0.9	0.2	0.2	0.2	0.1
Upper CI	99.8	90.2	64.8	36.9	23.5	18.4	12.9	10.2	8.1	7.5	7.0	5.0	2.3	2.3	2.3	1.7
Number	136	116	80	52	39	32	20	15	12	9	8	6	2	2	2	1

Prevalence by age group, gender and occupational group for the worse ear over 0.25, 0.5 and 1 kHz

Table W2551-50 Age Group = 61-70 Gender = MALE Occupational group = NON-MANUAL Total cases = 109

Threshold dB HL≥

	0	5	10	15	20	25	30	35	40	45	50	55	65	75	85	95
**Prevalence**	**100.0**	**94.9**	**72.7**	**53.0**	**41.5**	**27.2**	**23.7**	**17.5**	**13.5**	**10.2**	**7.5**	**6.7**	**3.0**	**3.0**	**0.9**	**0.9**
**Lower CI**	96.6	87.2	57.3	39.0	28.6	20.6	17.6	12.2	9.3	6.6	4.5	3.8	1.2	1.2	0.3	0.3
**Upper CI**	100.0	98.0	84.1	66.5	55.7	34.9	31.1	24.6	19.1	15.5	12.2	11.3	6.9	6.9	2.7	2.7
**Number**	109	103	81	58	45	34	29	21	17	14	11	9	5	5	2	2

Table W2551-51 Age Group = 71-80 Gender = MALE Occupational group = NON-MANUAL Total cases = 55

Threshold dB HL≥

	0	5	10	15	20	25	30	35	40	45	50	55	65	75	85	95
**Prevalence**	**97.9**	**97.9**	**79.2**	**65.9**	**48.5**	**34.6**	**26.6**	**13.2**	**10.4**	**9.6**	**4.9**	**4.9**	**3.7**	**3.7**	**0.0**	**0.0**
**Lower CI**	89.0	89.0	65.6	53.3	36.8	23.9	17.3	7.3	5.1	4.5	2.9	2.9	1.5	1.5	0.0	0.0
**Upper CI**	99.6	99.6	88.4	76.5	60.4	47.0	38.7	22.7	20.2	19.3	8.1	8.1	8.8	8.8	6.5	6.5
**Number**	54	54	46	40	31	21	16	9	7	6	4	4	3	3	0	0

Table W2551-52 Age Group = 18-30 Gender = FEMALE Occupational group = MANUAL Total cases = 109

Threshold dB HL≥

	0	5	10	15	20	25	30	35	40	45	50	55	65	75	85	95
**Prevalence**	**95.2**	**78.6**	**44.2**	**21.2**	**11.1**	**6.5**	**5.9**	**3.2**	**2.6**	**2.6**	**1.2**	**0.2**	**0.0**	**0.0**	**0.0**	**0.0**
**Lower CI**	84.0	67.0	32.6	12.8	6.3	3.5	3.0	1.4	1.0	1.0	0.4	0.0	0.0	0.0	0.0	0.0
**Upper CI**	98.7	86.9	56.4	32.9	18.7	11.7	11.2	7.2	6.5	6.5	3.1	1.1	3.4	3.4	3.4	3.4
**Number**	106	88	54	30	21	14	12	8	7	7	4	1	0	0	0	0

Table W2551-53 Age Group = 31-40 Gender = FEMALE Occupational group = MANUAL Total cases = 101

Threshold dB HL≥

	0	5	10	15	20	25	30	35	40	45	50	55	65	75	85	95
**Prevalence**	**98.0**	**77.2**	**46.5**	**24.2**	**13.0**	**7.5**	**3.8**	**3.1**	**3.1**	**2.5**	**2.5**	**2.5**	**0.8**	**0.5**	**0.3**	**0.0**
**Lower CI**	93.0	64.7	35.0	16.7	8.0	4.2	2.0	1.5	1.5	1.1	1.1	1.1	0.3	0.1	0.1	0.0
**Upper CI**	99.4	86.2	58.5	33.6	20.4	13.1	7.2	6.5	6.5	5.7	5.7	5.7	2.3	1.6	1.7	3.7
**Number**	98	82	58	36	25	17	9	7	7	6	6	6	3	2	1	0

Table W2551-54 Age Group = 41-50 Gender = FEMALE Occupational group = MANUAL Total cases = 132

Threshold dB HL≥

	0	5	10	15	20	25	30	35	40	45	50	55	65	75	85	95
**Prevalence**	**98.9**	**87.7**	**62.9**	**35.8**	**21.9**	**13.4**	**10.0**	**9.4**	**6.6**	**6.4**	**5.3**	**4.5**	**2.3**	**1.3**	**0.6**	**0.3**
**Lower CI**	93.8	79.8	53.5	27.6	15.3	8.7	6.3	5.8	4.0	3.7	3.2	2.8	1.3	0.6	0.2	0.0
**Upper CI**	99.8	92.9	71.4	44.9	30.3	20.2	15.5	15.0	10.9	10.7	8.5	7.2	3.9	2.6	2.4	1.5
**Number**	131	117	89	55	38	28	24	22	17	16	15	14	7	3	2	1

Table W2551-55 Age Group = 51-60 Gender = FEMALE Occupational group = MANUAL Total cases = 164

Threshold dB HL≥

	0	5	10	15	20	25	30	35	40	45	50	55	65	75	85	95
**Prevalence**	**99.3**	**94.1**	**74.7**	**46.2**	**34.2**	**24.2**	**18.5**	**13.1**	**11.0**	**5.9**	**5.8**	**3.8**	**1.4**	**0.8**	**0.5**	**0.4**
**Lower CI**	96.2	87.5	65.6	38.1	26.7	17.8	13.1	8.7	7.2	3.4	3.3	2.1	0.8	0.3	0.2	0.1
**Upper CI**	99.9	97.3	82.1	54.6	42.6	32.0	25.7	19.3	16.5	10.2	10.1	6.9	2.3	1.8	1.5	1.3
**Number**	163	157	134	100	73	56	43	34	30	21	20	15	9	5	3	2

Table W2551-56 Age Group = 61-70 Gender = FEMALE Occupational group = MANUAL Total cases = 123

Threshold dB HL≥

	0	5	10	15	20	25	30	35	40	45	50	55	65	75	85	95
**Prevalence**	**98.1**	**95.2**	**78.3**	**59.8**	**45.3**	**31.1**	**24.3**	**17.0**	**14.4**	**12.8**	**11.0**	**9.6**	**8.4**	**3.8**	**2.4**	**1.6**
**Lower CI**	90.1	88.0	67.4	49.0	36.0	23.9	18.3	14.7	10.1	8.8	6.9	5.8	4.9	2.7	1.6	0.4
**Upper CI**	99.7	98.1	86.3	69.7	54.9	39.5	31.4	19.7	20.1	18.4	16.9	15.5	14.1	5.3	3.3	5.7
**Number**	122	118	103	82	66	55	45	37	35	32	26	21	19	9	4	1

Prevalence by age group, gender and occupational group for the worse ear over 0.25, 0.5 and 1 kHz

Table W2551-57 Age Group = 71-80 Gender = FEMALE Occupational group = MANUAL Total cases = 72

Threshold dB HL≥	0	5	10	15	20	25	30	35	40	45	50	55	65	75	85	95
Prevalence	100.0	100.0	100.0	78.1	68.0	54.5	48.6	38.6	32.6	24.8	18.1	15.8	7.5	5.8	2.9	0.7
Lower CI	94.9	94.9	94.9	63.9	51.4	39.2	35.9	26.8	21.2	14.3	11.3	9.3	4.3	3.3	1.2	0.2
Upper CI	100.0	100.0	100.0	87.7	81.0	69.1	61.6	52.0	46.4	39.5	27.6	25.6	13.0	10.1	6.8	2.5
Number	72	72	72	66	59	53	49	41	38	29	26	22	12	10	5	2

Table W2551-58 Age Group = 18-30 Gender = MALE Occupational group = MANUAL Total cases = 109

Threshold dB HL≥	0	5	10	15	20	25	30	35	40	45	50	55	65	75	85	95
Prevalence	91.7	75.7	44.9	18.0	11.2	10.3	2.7	2.5	0.7	0.7	0.7	0.5	0.3	0.1	0.1	0.1
Lower CI	80.4	64.0	32.9	10.7	5.5	4.8	1.2	1.1	0.2	0.2	0.2	0.2	0.1	0.0	0.0	0.0
Upper CI	96.7	84.5	57.6	28.5	21.6	20.9	5.7	5.6	1.8	1.8	1.8	1.6	1.5	0.4	0.4	0.4
Number	102	90	57	30	21	17	10	9	4	4	4	3	2	1	1	1

Table W2551-59 Age Group = 31-40 Gender = MALE Occupational group = MANUAL Total cases = 80

Threshold dB HL≥	0	5	10	15	20	25	30	35	40	45	50	55	65	75	85	95
Prevalence	99.3	84.5	45.1	27.2	17.8	12.8	10.9	3.8	3.6	3.6	3.1	3.1	2.3	1.9	1.9	1.9
Lower CI	97.3	70.8	30.9	16.9	9.4	6.3	4.9	1.4	1.3	1.3	0.9	0.9	0.5	0.3	0.3	0.3
Upper CI	99.8	92.4	60.2	40.8	31.0	24.1	22.7	10.1	10.1	10.1	9.9	9.9	9.9	10.2	10.2	10.2
Number	78	66	42	29	20	16	11	7	6	6	4	4	2	1	1	1

Table W2551-60 Age Group = 41-50 Gender = MALE Occupational group = MANUAL Total cases = 126

Threshold dB HL≥	0	5	10	15	20	25	30	35	40	45	50	55	65	75	85	95
Prevalence	99.5	83.8	65.1	46.9	24.6	18.9	13.4	11.3	8.4	7.1	6.1	4.4	1.3	0.5	0.5	0.0
Lower CI	97.3	74.1	54.3	36.8	17.2	12.8	8.5	7.1	4.7	3.6	2.9	2.0	0.7	0.1	0.1	0.0
Upper CI	99.9	90.4	74.6	57.2	34.0	27.0	20.6	17.4	14.6	13.3	12.1	9.5	2.7	1.7	1.7	3.0
Number	125	111	90	69	43	35	26	23	18	14	12	10	5	2	2	0

Table W2551-61 Age Group = 51-60 Gender = MALE Occupational group = MANUAL Total cases = 193

Threshold dB HL≥	0	5	10	15	20	25	30	35	40	45	50	55	65	75	85	95
Prevalence	99.8	91.6	73.9	53.2	32.9	21.8	16.7	15.0	12.0	8.8	7.0	5.6	4.2	2.5	1.4	0.5
Lower CI	99.1	85.0	65.5	44.9	26.4	17.2	12.6	11.1	8.6	6.0	4.4	3.4	2.4	1.1	0.6	0.1
Upper CI	100.0	95.5	80.9	61.3	40.2	27.4	21.7	19.9	16.6	12.7	10.9	8.9	7.3	5.4	3.5	1.8
Number	192	180	153	119	85	64	51	45	37	28	21	18	14	7	5	2

Table W2551-62 Age Group = 61-70 Gender = MALE Occupational group = MANUAL Total cases = 169

Threshold dB HL≥	0	5	10	15	20	25	30	35	40	45	50	55	65	75	85	95
Prevalence	100.0	94.9	79.6	62.8	41.6	34.2	27.2	18.6	14.8	10.4	9.3	7.8	4.2	1.8	1.3	0.6
Lower CI	97.8	88.3	69.6	51.5	33.4	26.3	20.5	13.0	9.6	7.2	6.2	4.9	2.6	1.0	0.6	0.2
Upper CI	100.0	97.9	86.9	72.8	50.4	42.9	35.1	26.0	22.2	14.9	13.8	12.2	6.6	3.1	2.7	1.8
Number	169	163	145	125	96	82	70	52	43	34	30	24	17	10	7	3

Table W2551-63 Age Group = 71-80 Gender = MALE Occupational group = MANUAL Total cases = 70

Threshold dB HL≥	0	5	10	15	20	25	30	35	40	45	50	55	65	75	85	95
Prevalence	100.0	100.0	88.5	84.8	69.0	51.5	39.2	27.9	22.9	20.2	19.7	19.7	13.4	8.6	4.6	1.1
Lower CI	94.8	94.8	68.2	65.9	54.3	37.6	25.8	19.8	15.4	13.4	12.9	12.9	7.6	4.7	2.3	0.3
Upper CI	100.0	100.0	96.5	94.2	80.6	65.2	54.6	37.7	32.5	29.2	28.8	28.8	22.3	15.2	8.9	4.0
Number	70	70	66	61	53	47	37	33	28	25	24	24	14	9	5	2

Prevalence by age group, gender and occupational group for the worse ear over 1, 2 and 3 kHz

Table W123-1 Age Group = 18-80 Gender = OVERALL Occupational group = OVERALL Total cases = 2615

Threshold dB HL≥	0	5	10	15	20	25	30	35	40	45	50	55	65	75	85	95
**Prevalence**	**95.6**	**80.3**	**60.4**	**43.6**	**32.6**	**24.7**	**19.5**	**15.6**	**12.0**	**9.5**	**7.5**	**5.8**	**3.4**	**2.0**	**1.1**	**0.7**
Lower CI	94.3	78.2	58.3	41.8	31.0	23.3	18.2	14.4	11.1	8.7	6.7	5.2	2.9	1.7	0.9	0.5
Upper CI	96.6	82.2	62.4	45.5	34.4	26.3	20.9	16.8	13.1	10.4	8.3	6.6	3.9	2.4	1.5	1.0
Number	2551	2289	1903	1508	1230	1014	852	722	598	496	408	329	210	135	77	44

Table W123-2 Age Group = 18-80 Gender = FEMALE Occupational group = OVERALL Total cases = 1366

Threshold dB HL≥	0	5	10	15	20	25	30	35	40	45	50	55	65	75	85	95
**Prevalence**	**95.3**	**79.5**	**58.6**	**39.8**	**28.4**	**21.1**	**16.4**	**13.3**	**10.5**	**8.0**	**5.8**	**4.7**	**3.0**	**1.5**	**0.9**	**0.5**
Lower CI	93.5	76.8	56.0	37.4	26.2	19.2	14.6	11.8	9.1	7.0	5.0	3.9	2.4	1.2	0.7	0.3
Upper CI	96.6	82.0	61.2	42.2	30.6	23.2	18.3	15.0	12.0	9.2	6.8	5.7	3.7	1.9	1.3	0.8
Number	1328	1168	943	708	548	440	362	309	256	212	166	138	98	55	34	18

Table W123-3 Age Group = 18-80 Gender = MALE Occupational group = OVERALL Total cases = 1249

Threshold dB HL≥	0	5	10	15	20	25	30	35	40	45	50	55	65	75	85	95
**Prevalence**	**95.9**	**81.2**	**62.4**	**47.9**	**37.5**	**28.8**	**23.0**	**18.0**	**13.8**	**11.2**	**9.3**	**7.1**	**3.8**	**2.6**	**1.4**	**0.9**
Lower CI	93.7	77.9	59.1	45.0	34.9	26.7	21.1	16.3	12.4	9.9	8.1	6.1	3.1	2.0	0.9	0.5
Upper CI	97.4	84.1	65.5	50.9	40.1	31.0	24.9	19.9	15.3	12.6	10.6	8.3	4.7	3.4	2.0	1.4
Number	1223	1121	960	800	682	574	490	413	342	284	242	191	112	80	43	26

Table W123-4 Age Group = 18-30 Gender = OVERALL Occupational group = OVERALL Total cases = 401

Threshold dB HL≥	0	5	10	15	20	25	30	35	40	45	50	55	65	75	85	95
**Prevalence**	**89.0**	**59.7**	**30.7**	**14.9**	**8.1**	**5.5**	**3.9**	**2.8**	**1.5**	**1.1**	**0.5**	**0.3**	**0.0**	**0.0**	**0.0**	**0.0**
Lower CI	84.7	53.8	25.8	11.5	5.9	3.8	2.6	1.6	0.9	0.6	0.2	0.1	0.0	0.0	0.0	0.0
Upper CI	92.2	65.2	36.1	19.1	11.1	7.8	6.0	4.6	2.6	2.2	1.1	0.8	0.1	0.1	0.1	0.1
Number	364	260	150	84	56	42	33	24	16	12	7	5	2	2	1	1

Table W123-5 Age Group = 31-40 Gender = OVERALL Occupational group = OVERALL Total cases = 327

Threshold dB HL≥	0	5	10	15	20	25	30	35	40	45	50	55	65	75	85	95
**Prevalence**	**94.1**	**70.4**	**39.9**	**25.0**	**16.5**	**9.1**	**7.2**	**5.4**	**3.4**	**2.4**	**1.6**	**1.3**	**0.9**	**0.7**	**0.6**	**0.6**
Lower CI	90.3	63.9	33.8	19.9	12.4	6.4	4.9	3.5	2.0	1.3	0.7	0.5	0.3	0.2	0.1	0.1
Upper CI	96.4	76.1	46.3	30.9	21.7	12.8	10.5	8.3	5.8	4.5	3.5	3.2	3.0	3.0	3.0	3.1
Number	310	243	158	104	72	48	41	31	22	16	11	8	5	3	2	1

Table W123-6 Age Group = 41-50 Gender = OVERALL Occupational group = OVERALL Total cases = 443

Threshold dB HL≥	0	5	10	15	20	25	30	35	40	45	50	55	65	75	85	95
**Prevalence**	**98.3**	**86.2**	**60.6**	**40.7**	**26.8**	**18.0**	**13.4**	**9.1**	**6.1**	**5.1**	**4.3**	**3.1**	**1.5**	**1.1**	**0.6**	**0.2**
Lower CI	96.3	82.2	55.4	36.0	22.9	15.0	10.8	7.1	4.5	3.6	3.0	2.0	0.9	0.6	0.3	0.1
Upper CI	99.2	89.5	65.5	45.6	31.2	21.4	16.4	11.7	8.1	7.0	6.2	4.7	2.5	1.9	1.3	0.5
Number	437	391	293	209	151	116	91	69	51	41	36	27	17	12	8	3

Table W123-7 Age Group = 51-60 Gender = OVERALL Occupational group = OVERALL Total cases = 665

Threshold dB HL≥	0	5	10	15	20	25	30	35	40	45	50	55	65	75	85	95
**Prevalence**	**99.4**	**91.7**	**79.7**	**55.5**	**42.7**	**31.9**	**22.3**	**16.5**	**12.4**	**9.9**	**8.1**	**6.5**	**3.8**	**2.0**	**1.2**	**0.8**
Lower CI	98.4	88.9	76.0	51.3	38.8	28.5	19.5	14.2	10.4	8.2	6.5	5.1	2.7	1.3	0.7	0.4
Upper CI	99.8	93.9	82.9	59.7	46.7	35.6	25.3	19.0	14.6	12.0	9.9	8.2	5.2	2.9	2.0	1.6
Number	662	620	554	426	347	278	216	174	139	116	95	78	46	29	16	11

Prevalence by age group, gender and occupational group for the worse ear over 1, 2 and 3 kHz

Table W123-8 Age Group = 61-70 Gender = OVERALL Occupational group = OVERALL Total cases = 513

Threshold dB HL≥	0	5	10	15	20	25	30	35	40	45	50	55	65	75	85	95
**Prevalence**	**99.5**	**99.2**	**93.6**	**79.1**	**61.9**	**48.6**	**38.2**	**32.6**	**26.3**	**21.0**	**16.9**	**12.3**	**8.0**	**4.7**	**2.4**	**1.3**
**Lower CI**	97.5	97.3	90.4	74.4	56.7	43.7	33.6	28.4	22.6	17.8	14.1	9.9	6.0	3.4	1.7	0.7
**Upper CI**	99.9	99.7	95.7	83.2	66.8	53.5	43.0	37.0	30.3	24.6	20.1	15.3	10.4	6.5	3.6	2.1
**Number**	512	510	486	431	366	308	262	237	205	172	143	109	73	46	26	15

Table W123-9 Age Group = 71-80 Gender = OVERALL Occupational group = OVERALL Total cases = 266

Threshold dB HL≥	0	5	10	15	20	25	30	35	40	45	50	55	65	75	85	95
**Prevalence**	**100.0**	**99.6**	**97.5**	**90.7**	**79.6**	**71.0**	**64.4**	**55.5**	**46.2**	**36.1**	**28.2**	**24.4**	**13.2**	**7.6**	**3.8**	**2.2**
**Lower CI**	98.6	98.0	93.5	84.7	71.3	62.8	56.7	47.9	39.1	30.3	23.2	19.4	10.3	5.8	2.6	1.3
**Upper CI**	100.0	99.9	99.1	94.5	85.9	78.0	71.5	62.8	53.4	42.2	33.8	30.1	16.8	10.0	5.5	3.9
**Number**	266	265	262	254	238	222	209	187	165	139	116	102	67	43	24	13

Table W123-10 Age Group = 18-80 Gender = OVERALL Occupational group = NON-MANUAL Total cases = 1164

Threshold dB HL≥	0	5	10	15	20	25	30	35	40	45	50	55	65	75	85	95
**Prevalence**	**94.8**	**76.9**	**55.5**	**39.0**	**28.6**	**22.5**	**17.7**	**14.0**	**10.9**	**7.4**	**5.5**	**3.8**	**2.3**	**1.5**	**0.9**	**0.5**
**Lower CI**	92.6	73.7	52.5	36.2	26.4	20.6	16.1	12.6	9.7	6.5	4.7	3.2	1.9	1.2	0.6	0.3
**Upper CI**	96.4	79.8	58.5	41.8	30.9	24.6	19.4	15.6	12.3	8.3	6.4	4.4	2.8	1.9	1.3	0.9
**Number**	1128	978	775	582	462	376	312	257	213	163	128	102	66	46	27	16

Table W123-11 Age Group = 18-80 Gender = OVERALL Occupational group = MANUAL Total cases = 1447

Threshold dB HL≥	0	5	10	15	20	25	30	35	40	45	50	55	65	75	85	95
**Prevalence**	**96.0**	**84.1**	**64.9**	**47.4**	**35.8**	**27.0**	**21.4**	**16.9**	**12.7**	**11.0**	**8.7**	**6.9**	**3.9**	**2.3**	**1.3**	**0.8**
**Lower CI**	93.9	81.0	61.7	44.5	33.2	24.9	19.5	15.3	11.4	9.8	7.6	5.9	3.2	1.8	1.0	0.6
**Upper CI**	97.4	86.7	68.0	50.3	38.4	29.3	23.4	18.7	14.1	12.5	9.9	8.1	4.8	3.0	1.8	1.3
**Number**	1419	1309	1126	924	766	636	539	464	384	333	280	227	144	89	50	28

Table W123-12 Age Group = 18-30 Gender = FEMALE Occupational group = OVERALL Total cases = 224

Threshold dB HL≥	0	5	10	15	20	25	30	35	40	45	50	55	65	75	85	95
**Prevalence**	**89.5**	**62.1**	**30.6**	**13.2**	**7.1**	**5.0**	**3.9**	**2.6**	**2.2**	**1.7**	**0.5**	**0.3**	**0.0**	**0.0**	**0.0**	**0.0**
**Lower CI**	83.9	54.7	24.5	9.5	4.7	3.2	2.3	1.4	1.1	0.8	0.2	0.1	0.0	0.0	0.0	0.0
**Upper CI**	93.3	69.0	37.4	18.1	10.7	7.7	6.3	4.5	4.2	3.6	1.6	1.3	1.7	1.7	1.7	1.7
**Number**	205	148	84	45	28	22	18	13	10	8	3	2	0	0	0	0

Table W123-13 Age Group = 31-40 Gender = FEMALE Occupational group = OVERALL Total cases = 185

Threshold dB HL≥	0	5	10	15	20	25	30	35	40	45	50	55	65	75	85	95
**Prevalence**	**91.7**	**65.2**	**34.3**	**19.5**	**11.9**	**8.6**	**6.6**	**4.2**	**3.4**	**2.3**	**1.1**	**0.8**	**0.6**	**0.3**	**0.1**	**0.0**
**Lower CI**	85.7	56.8	27.4	14.3	8.3	5.5	3.9	2.2	1.6	1.0	0.5	0.3	0.2	0.1	0.0	0.0
**Upper CI**	95.4	72.8	41.9	26.1	17.0	13.2	10.8	8.1	6.9	5.0	2.7	2.1	1.7	1.2	0.5	2.0
**Number**	173	132	83	53	35	25	20	12	10	8	5	4	3	2	1	0

Table W123-14 Age Group = 41-50 Gender = FEMALE Occupational group = OVERALL Total cases = 245

Threshold dB HL≥	0	5	10	15	20	25	30	35	40	45	50	55	65	75	85	95
**Prevalence**	**97.3**	**82.6**	**55.8**	**33.5**	**20.8**	**14.2**	**10.0**	**6.9**	**3.9**	**3.3**	**2.7**	**2.3**	**1.5**	**0.6**	**0.5**	**0.1**
**Lower CI**	93.8	76.5	49.0	27.7	16.3	10.8	7.0	4.7	2.5	2.1	1.6	1.2	0.7	0.2	0.2	0.0
**Upper CI**	98.8	87.3	62.4	39.9	26.2	18.5	14.0	10.0	5.9	5.3	4.6	4.1	2.9	1.7	1.4	0.7
**Number**	240	209	151	98	67	51	38	30	20	16	13	11	8	4	3	1

Prevalence by age group, gender and occupational group for the worse ear over 1, 2 and 3 kHz

Table W123-15 Age Group = 51-60 Gender = FEMALE Occupational group = OVERALL Total cases = 334

Threshold dB HL≥	0	5	10	15	20	25	30	35	40	45	50	55	65	75	85	95
**Prevalence**	**99.7**	**89.4**	**77.4**	**50.5**	**35.4**	**22.5**	**14.9**	**10.9**	**7.5**	**5.3**	**4.4**	**3.5**	**2.7**	**1.4**	**0.9**	**0.7**
**Lower CI**	98.4	85.0	72.2	44.6	30.2	18.3	11.8	8.4	5.6	3.8	3.1	2.3	1.7	0.8	0.4	0.3
**Upper CI**	99.9	92.6	81.8	56.4	40.9	27.3	18.8	14.0	9.9	7.3	6.4	5.2	4.5	2.4	2.0	1.7
**Number**	333	304	269	198	150	109	83	67	50	40	34	28	21	13	7	5

Table W123-16 Age Group = 61-70 Gender = FEMALE Occupational group = OVERALL Total cases = 238

Threshold dB HL≥	0	5	10	15	20	25	30	35	40	45	50	55	65	75	85	95
**Prevalence**	**99.2**	**98.8**	**90.3**	**71.2**	**52.9**	**40.5**	**28.9**	**25.0**	**19.8**	**15.6**	**11.3**	**9.6**	**6.9**	**2.8**	**2.4**	**1.2**
**Lower CI**	95.4	95.4	84.8	63.9	45.7	34.2	23.1	19.6	15.0	11.5	8.0	6.5	4.4	1.8	1.4	0.6
**Upper CI**	99.9	99.7	93.9	77.6	59.9	47.1	35.6	31.3	25.7	20.8	15.8	13.9	10.6	4.4	4.0	2.5
**Number**	237	236	218	183	146	119	94	86	75	64	50	41	31	15	12	7

Table W123-17 Age Group = 71-80 Gender = FEMALE Occupational group = OVERALL Total cases = 140

Threshold dB HL≥	0	5	10	15	20	25	30	35	40	45	50	55	65	75	85	95
**Prevalence**	**100.0**	**99.4**	**97.9**	**87.0**	**73.5**	**63.6**	**59.1**	**53.7**	**44.4**	**34.2**	**25.6**	**20.9**	**12.0**	**6.5**	**3.0**	**1.5**
**Lower CI**	97.3	96.8	91.8	77.8	60.9	51.5	47.6	43.3	34.9	27.3	19.8	15.2	8.4	4.4	1.7	0.6
**Upper CI**	100.0	99.9	99.5	92.8	83.2	74.3	69.8	63.8	54.3	41.8	32.3	28.0	17.0	9.5	5.3	3.7
**Number**	140	139	138	131	122	114	109	101	91	76	61	52	35	21	11	5

Table W123-18 Age Group = 18-30 Gender = MALE Occupational group = OVERALL Total cases = 177

Threshold dB HL≥	0	5	10	15	20	25	30	35	40	45	50	55	65	75	85	95
**Prevalence**	**88.4**	**56.9**	**30.7**	**16.4**	**8.9**	**5.8**	**3.9**	**2.9**	**0.8**	**0.5**	**0.5**	**0.3**	**0.1**	**0.1**	**0.0**	**0.0**
**Lower CI**	81.2	47.8	23.3	11.2	5.6	3.3	2.0	1.3	0.3	0.2	0.2	0.1	0.0	0.0	0.0	0.0
**Upper CI**	93.1	65.6	39.1	23.5	14.0	9.9	7.4	6.5	1.8	1.3	1.3	1.0	0.2	0.2	0.2	0.2
**Number**	159	112	66	39	28	20	15	11	6	4	4	3	2	2	1	1

Table W123-19 Age Group = 31-40 Gender = MALE Occupational group = OVERALL Total cases = 142

Threshold dB HL≥	0	5	10	15	20	25	30	35	40	45	50	55	65	75	85	95
**Prevalence**	**96.7**	**76.0**	**46.1**	**31.1**	**21.6**	**9.6**	**8.0**	**6.6**	**3.5**	**2.7**	**2.1**	**1.9**	**1.3**	**1.2**	**1.2**	**1.2**
**Lower CI**	91.1	65.6	35.9	22.5	14.3	5.6	4.5	3.7	1.7	1.0	0.7	0.6	0.3	0.2	0.2	0.2
**Upper CI**	98.8	84.0	56.5	41.3	31.3	16.0	13.7	11.5	7.3	6.7	6.4	6.3	6.4	6.5	6.5	6.5
**Number**	137	111	75	51	37	23	21	19	12	8	6	4	2	1	1	1

Table W123-20 Age Group = 41-50 Gender = MALE Occupational group = OVERALL Total cases = 198

Threshold dB HL≥	0	5	10	15	20	25	30	35	40	45	50	55	65	75	85	95
**Prevalence**	**99.4**	**90.3**	**65.8**	**48.6**	**33.5**	**22.1**	**17.1**	**11.6**	**8.5**	**6.9**	**6.2**	**4.1**	**1.6**	**1.5**	**0.8**	**0.2**
**Lower CI**	96.5	84.3	57.9	41.1	27.0	17.3	13.0	8.2	5.7	4.4	3.8	2.3	0.8	0.7	0.4	0.1
**Upper CI**	99.9	94.1	72.9	56.0	40.6	27.8	22.1	16.2	12.4	10.7	9.7	7.1	3.2	3.2	1.9	0.7
**Number**	197	182	142	111	84	65	53	39	31	25	23	16	9	8	5	2

Table W123-21 Age Group = 51-60 Gender = MALE Occupational group = OVERALL Total cases = 331

Threshold dB HL≥	0	5	10	15	20	25	30	35	40	45	50	55	65	75	85	95
**Prevalence**	**99.1**	**94.4**	**82.3**	**61.0**	**50.9**	**42.5**	**30.5**	**22.7**	**17.8**	**15.1**	**12.1**	**9.9**	**4.9**	**2.7**	**1.5**	**1.0**
**Lower CI**	96.9	90.5	76.9	55.0	45.1	36.9	25.9	18.9	14.4	11.9	9.3	7.4	3.2	1.6	0.7	0.4
**Upper CI**	99.8	96.7	86.6	66.7	56.7	48.2	35.5	26.9	21.9	18.9	15.6	13.1	7.6	4.5	3.0	2.6
**Number**	329	316	285	228	197	169	133	107	89	76	61	50	25	16	9	6

Prevalence by age group, gender and occupational group for the worse ear over 1, 2 and 3 kHz

Table W123-29 Age Group = 71-80 Gender = OVERALL Occupational group = NON-MANUAL Total cases = 121

Threshold dB HL≥

	0	5	10	15	20	25	30	35	40	45	50	55	65	75	85	95
Prevalence	**100.0**	**99.1**	**96.7**	**88.6**	**77.2**	**70.0**	**58.6**	**52.1**	**46.8**	**30.0**	**23.5**	**17.4**	**11.4**	**5.7**	**2.9**	**1.2**
Lower CI	96.9	95.1	90.3	77.0	65.3	58.7	51.0	44.3	39.6	24.7	19.3	13.1	8.2	3.6	1.4	0.3
Upper CI	100.0	99.8	99.0	94.7	85.9	79.3	65.8	59.8	54.2	35.8	28.3	22.6	15.6	8.8	5.8	4.5
Number	121	120	118	112	102	93	82	73	66	52	41	35	24	14	7	2

Table W123-30 Age Group = 18-30 Gender = OVERALL Occupational group = MANUAL Total cases = 218

Threshold dB HL≥

	0	5	10	15	20	25	30	35	40	45	50	55	65	75	85	95
Prevalence	**89.7**	**69.7**	**38.1**	**19.5**	**11.2**	**7.5**	**4.8**	**3.5**	**1.9**	**1.5**	**0.5**	**0.3**	**0.0**	**0.0**	**0.0**	**0.0**
Lower CI	82.9	61.1	30.1	13.7	7.6	4.8	2.8	1.9	0.9	0.6	0.2	0.1	0.0	0.0	0.0	0.0
Upper CI	94.0	77.1	46.8	27.0	16.0	11.6	7.9	6.6	3.9	3.4	1.3	0.9	0.2	0.2	0.2	0.2
Number	202	160	103	61	43	31	22	18	12	9	5	3	1	1	1	1

Table W123-31 Age Group = 31-40 Gender = OVERALL Occupational group = MANUAL Total cases = 181

Threshold dB HL≥

	0	5	10	15	20	25	30	35	40	45	50	55	65	75	85	95
Prevalence	**94.5**	**72.8**	**43.6**	**29.4**	**19.8**	**10.8**	**8.1**	**5.5**	**4.4**	**3.9**	**2.5**	**2.0**	**1.4**	**1.2**	**1.0**	**0.9**
Lower CI	88.5	63.3	34.7	22.0	13.6	6.5	4.8	3.2	2.3	2.0	1.1	0.8	0.4	0.3	0.2	0.2
Upper CI	97.4	80.6	52.8	38.0	27.9	17.3	13.2	9.4	8.1	7.7	5.6	5.1	4.8	4.8	4.9	5.0
Number	172	140	99	73	50	33	29	21	17	14	11	8	5	3	2	1

Table W123-32 Age Group = 41-50 Gender = OVERALL Occupational group = MANUAL Total cases = 256

Threshold dB HL≥

	0	5	10	15	20	25	30	35	40	45	50	55	65	75	85	95
Prevalence	**98.9**	**88.2**	**69.0**	**45.9**	**29.8**	**19.6**	**15.3**	**11.1**	**7.0**	**6.1**	**5.6**	**4.4**	**1.9**	**1.1**	**0.8**	**0.2**
Lower CI	96.2	82.7	62.0	38.9	23.9	15.2	11.5	8.0	4.8	4.0	3.6	2.7	1.2	0.5	0.3	0.1
Upper CI	99.7	92.2	75.2	53.0	36.6	24.8	20.0	15.2	10.0	9.1	8.5	7.2	3.0	2.2	1.7	0.8
Number	254	233	188	137	100	78	63	49	37	31	29	22	13	8	6	2

Table W123-33 Age Group = 51-60 Gender = OVERALL Occupational group = MANUAL Total cases = 356

Threshold dB HL≥

	0	5	10	15	20	25	30	35	40	45	50	55	65	75	85	95
Prevalence	**99.6**	**93.6**	**84.5**	**60.3**	**47.5**	**35.3**	**26.1**	**19.1**	**13.8**	**11.8**	**9.9**	**8.2**	**4.5**	**1.9**	**1.0**	**0.7**
Lower CI	98.0	89.5	79.1	54.1	41.8	30.3	21.9	15.8	11.1	9.3	7.6	6.2	3.0	1.2	0.5	0.3
Upper CI	99.9	96.2	88.6	66.2	53.2	40.7	30.8	23.0	17.1	14.9	12.8	10.9	6.9	3.0	2.0	1.6
Number	355	341	316	259	216	174	137	111	88	77	65	54	32	18	9	6

Table W123-34 Age Group = 61-70 Gender = OVERALL Occupational group = MANUAL Total cases = 292

Threshold dB HL≥

	0	5	10	15	20	25	30	35	40	45	50	55	65	75	85	95
Prevalence	**100.0**	**99.4**	**92.3**	**80.1**	**62.7**	**49.9**	**37.2**	**33.2**	**27.8**	**24.0**	**18.5**	**13.5**	**9.6**	**5.6**	**2.5**	**1.5**
Lower CI	98.7	96.9	86.9	73.5	55.5	43.5	31.3	27.7	22.8	19.2	14.5	10.0	6.5	3.5	1.8	1.1
Upper CI	100.0	99.9	95.6	85.4	69.4	56.2	43.6	39.3	33.4	29.7	23.3	18.0	13.9	8.7	3.6	2.1
Number	292	291	277	253	222	192	162	152	132	115	95	73	50	30	15	7

Table W123-35 Age Group = 71-80 Gender = OVERALL Occupational group = MANUAL Total cases = 144

Threshold dB HL≥

	0	5	10	15	20	25	30	35	40	45	50	55	65	75	85	95
Prevalence	**100.0**	**100.0**	**97.7**	**92.6**	**83.9**	**75.5**	**73.5**	**60.7**	**46.1**	**41.2**	**33.0**	**29.0**	**14.0**	**8.7**	**4.9**	**3.4**
Lower CI	97.4	97.4	87.9	82.8	70.0	62.7	60.4	49.3	37.0	32.2	24.4	20.5	10.5	6.2	3.3	2.1
Upper CI	100.0	100.0	99.6	97.0	92.1	85.0	83.5	71.0	55.5	50.8	43.1	39.3	18.5	12.0	7.1	5.5
Number	144	144	143	141	135	128	126	113	98	87	75	67	43	29	17	11

Prevalence by age group, gender and occupational group for the worse ear over 1, 2 and 3 kHz

Table W123-22 Age Group = 61-70 Gender = MALE Occupational group = OVERALL Total cases = 275

	Threshold dB HL≥															
	**0**	5	**10**	15	**20**	25	**30**	35	**40**	45	**50**	55	**65**	75	**85**	95
Prevalence	**100.0**	**99.5**	**97.4**	**88.3**	**72.2**	**57.9**	**49.0**	**41.4**	**33.9**	**27.2**	**23.5**	**15.7**	**9.2**	**7.0**	**2.5**	**1.3**
Lower CI	98.6	97.5	94.2	82.1	64.7	50.3	42.0	35.4	28.6	22.5	19.1	12.2	6.6	4.6	1.5	0.6
Upper CI	100.0	99.9	98.8	92.6	78.6	65.1	56.0	47.8	39.6	32.5	28.5	19.9	12.7	10.6	4.3	2.7
Number	275	274	268	248	220	189	168	151	130	108	93	68	42	31	14	8

Table W123-23 Age Group = 71-80 Gender = MALE Occupational group = OVERALL Total cases = 126

	Threshold dB HL≥															
	**0**	5	**10**	15	**20**	25	**30**	35	**40**	45	**50**	55	**65**	75	**85**	95
Prevalence	**100.0**	**100.0**	**96.7**	**96.0**	**88.3**	**81.2**	**71.3**	**57.6**	**48.3**	**38.6**	**32.5**	**29.5**	**15.1**	**9.5**	**5.0**	**3.3**
Lower CI	97.0	97.0	88.4	87.9	79.7	72.1	63.1	47.2	38.5	29.6	24.1	21.4	10.8	6.4	3.0	1.6
Upper CI	100.0	100.0	99.1	98.7	93.5	87.8	78.2	67.4	58.2	48.5	42.1	39.1	20.6	13.9	8.3	6.5
Number	126	126	124	123	116	108	100	86	74	63	55	50	32	22	13	8

Table W123-24 Age Group = 18-30 Gender = OVERALL Occupational group = NON-MANUAL Total cases = 181

	Threshold dB HL≥															
	**0**	5	**10**	15	**20**	25	**30**	35	**40**	45	**50**	55	**65**	75	**85**	95
Prevalence	**86.4**	**51.8**	**22.9**	**9.9**	**3.4**	**2.3**	**2.3**	**1.2**	**1.0**	**0.7**	**0.3**	**0.3**	**0.1**	**0.1**	**0.0**	**0.0**
Lower CI	78.8	43.4	16.5	5.8	1.9	1.3	1.3	0.5	0.4	0.2	0.1	0.1	0.0	0.0	0.0	0.0
Upper CI	91.6	60.1	30.9	16.3	6.2	4.1	4.1	2.7	2.5	2.5	1.0	1.0	2.2	2.2	2.1	2.1
Number	160	100	47	23	13	11	11	6	4	3	2	2	1	1	0	0

Table W123-25 Age Group = 31-40 Gender = OVERALL Occupational group = NON-MANUAL Total cases = 146

	Threshold dB HL≥															
	**0**	5	**10**	15	**20**	25	**30**	35	**40**	45	**50**	55	**65**	75	**85**	95
Prevalence	**94.4**	**67.8**	**36.2**	**19.7**	**12.3**	**7.9**	**6.2**	**5.1**	**1.8**	**0.4**	**0.0**	**0.0**	**0.0**	**0.0**	**0.0**	**0.0**
Lower CI	89.4	57.9	27.4	12.8	7.7	4.4	3.1	2.3	0.7	0.1	0.0	0.0	0.0	0.0	0.0	0.0
Upper CI	97.2	76.3	45.9	29.0	19.2	13.6	11.9	10.8	4.7	3.9	2.6	2.6	2.6	2.6	2.6	2.6
Number	138	103	59	31	22	15	12	10	5	2	0	0	0	0	0	0

Table W123-26 Age Group = 41-50 Gender = OVERALL Occupational group = NON-MANUAL Total cases = 187

	Threshold dB HL≥															
	**0**	5	**10**	15	**20**	25	**30**	35	**40**	45	**50**	55	**65**	75	**85**	95
Prevalence	**97.9**	**84.3**	**51.7**	**35.7**	**25.4**	**18.5**	**13.8**	**8.2**	**5.9**	**4.2**	**2.8**	**1.2**	**1.1**	**1.1**	**0.8**	**0.1**
Lower CI	94.8	77.7	43.9	28.9	19.8	14.0	9.9	4.9	3.1	2.2	1.2	0.8	0.7	0.7	0.6	0.0
Upper CI	99.2	89.2	59.4	43.1	31.8	24.0	18.8	13.4	11.2	7.9	6.5	1.9	1.8	1.8	1.1	0.7
Number	183	158	105	72	51	38	28	20	14	10	7	5	4	4	2	1

Table W123-27 Age Group = 51-60 Gender = OVERALL Occupational group = NON-MANUAL Total cases = 309

	Threshold dB HL≥															
	**0**	5	**10**	15	**20**	25	**30**	35	**40**	45	**50**	55	**65**	75	**85**	95
Prevalence	**99.3**	**90.0**	**75.9**	**49.4**	**35.7**	**27.7**	**18.1**	**13.8**	**8.8**	**6.2**	**4.6**	**3.6**	**2.3**	**1.7**	**1.0**	**0.8**
Lower CI	97.3	85.6	70.3	43.5	30.8	23.4	15.2	11.7	6.8	4.7	3.3	2.5	1.3	0.9	0.5	0.3
Upper CI	99.8	93.2	80.8	55.3	41.0	32.5	21.3	16.3	11.3	8.1	6.3	5.1	4.0	3.0	2.2	1.9
Number	307	279	238	167	131	104	79	63	51	39	30	24	14	11	7	5

Table W123-28 Age Group = 61-70 Gender = OVERALL Occupational group = NON-MANUAL Total cases = 220

	Threshold dB HL≥															
	**0**	5	**10**	15	**20**	25	**30**	35	**40**	45	**50**	55	**65**	75	**85**	95
Prevalence	**99.2**	**98.4**	**94.4**	**77.2**	**60.4**	**47.1**	**39.4**	**31.3**	**24.3**	**18.1**	**14.2**	**9.6**	**5.0**	**3.7**	**2.3**	**1.7**
Lower CI	95.4	94.3	89.7	68.6	51.8	39.1	32.2	26.3	19.8	14.9	11.0	7.3	4.0	2.8	1.2	0.7
Upper CI	99.9	99.6	97.0	84.0	68.5	55.2	47.0	36.8	29.4	21.8	18.2	12.4	6.2	4.9	4.3	3.8
Number	219	218	208	177	143	115	100	85	73	57	48	36	23	16	11	8

Prevalence by age group, gender and occupational group for the worse ear over 1, 2 and 3 kHz

Table W123-36 Age Group = 18-80 Gender = FEMALE Occupational group = NON-MANUAL Total cases = 662

Threshold dB HL≥	0	5	10	15	20	25	30	35	40	45	50	55	65	75	85	95
**Prevalence**	**95.4**	**77.2**	**54.9**	**36.9**	**26.2**	**21.0**	**15.8**	**13.3**	**10.3**	**6.2**	**4.8**	**3.4**	**2.4**	**1.4**	**0.9**	**0.5**
**Lower CI**	93.1	73.5	51.4	33.6	23.4	18.4	13.9	11.6	8.8	5.2	4.1	2.6	1.8	1.0	0.5	0.2
**Upper CI**	97.0	80.6	58.4	40.4	29.3	23.8	18.0	15.2	12.0	7.4	5.7	4.3	3.1	2.0	1.4	1.0
**Number**	641	548	426	308	237	195	160	137	111	84	67	55	41	26	17	9

Table W123-37 Age Group = 18-80 Gender = MALE Occupational group = NON-MANUAL Total cases = 502

Threshold dB HL≥	0	5	10	15	20	25	30	35	40	45	50	55	65	75	85	95
**Prevalence**	**94.1**	**76.4**	**56.1**	**41.2**	**31.2**	**24.2**	**19.8**	**14.7**	**11.6**	**8.6**	**6.3**	**4.3**	**2.3**	**1.7**	**0.9**	**0.6**
**Lower CI**	89.7	71.0	51.0	36.7	28.0	21.5	17.3	12.6	9.7	7.2	5.0	3.4	1.8	1.2	0.6	0.3
**Upper CI**	96.6	81.1	61.1	45.9	34.7	27.3	22.5	17.2	13.8	10.3	8.0	5.3	2.9	2.3	1.5	1.2
**Number**	487	430	349	274	225	181	152	120	102	79	61	47	25	20	10	7

Table W123-38 Age Group = 18-80 Gender = FEMALE Occupational group = MANUAL Total cases = 703

Threshold dB HL≥	0	5	10	15	20	25	30	35	40	45	50	55	65	75	85	95
**Prevalence**	**94.8**	**82.7**	**63.1**	**42.6**	**30.1**	**21.4**	**16.8**	**13.4**	**10.4**	**9.2**	**6.3**	**5.3**	**3.2**	**1.5**	**1.0**	**0.6**
**Lower CI**	91.3	78.5	58.8	38.9	26.9	18.6	14.4	11.4	8.8	7.6	5.0	4.1	2.4	1.1	0.7	0.4
**Upper CI**	97.0	86.2	67.3	46.5	33.6	24.5	19.6	15.7	12.4	11.1	7.9	6.9	4.1	1.9	1.3	0.9
**Number**	686	619	516	399	310	244	202	172	145	128	99	83	57	29	17	9

Table W123-39 Age Group = 18-80 Gender = MALE Occupational group = MANUAL Total cases = 744

Threshold dB HL≥	0	5	10	15	20	25	30	35	40	45	50	55	65	75	85	95
**Prevalence**	**97.3**	**85.6**	**66.9**	**52.7**	**42.1**	**33.2**	**26.5**	**20.8**	**15.2**	**13.1**	**11.4**	**8.8**	**4.8**	**3.3**	**1.7**	**1.1**
**Lower CI**	94.8	80.9	62.1	48.3	38.2	30.0	23.8	18.4	13.3	11.2	9.6	7.2	3.7	2.3	1.1	0.6
**Upper CI**	98.6	89.3	71.4	57.1	46.0	36.6	29.5	23.5	17.4	15.2	13.4	10.7	6.3	4.6	2.6	2.0
**Number**	733	690	610	525	456	392	337	292	239	205	181	144	87	60	33	19

Table W123-40 Age Group = 18-30 Gender = FEMALE Occupational group = NON-MANUAL Total cases = 115

Threshold dB HL≥	0	5	10	15	20	25	30	35	40	45	50	55	65	75	85	95
**Prevalence**	**90.7**	**54.8**	**23.4**	**9.7**	**4.9**	**4.1**	**4.1**	**2.2**	**1.8**	**1.2**	**0.4**	**0.4**	**0.0**	**0.0**	**0.0**	**0.0**
**Lower CI**	83.4	45.3	16.5	5.9	2.7	2.3	2.3	1.0	0.7	0.3	0.1	0.1	0.0	0.0	0.0	0.0
**Upper CI**	95.0	64.1	32.0	15.5	8.6	7.4	7.4	5.1	4.6	4.7	2.2	2.2	3.2	3.2	3.2	3.2
**Number**	105	67	32	17	10	9	9	5	3	2	1	1	0	0	0	0

Table W123-41 Age Group = 31-40 Gender = FEMALE Occupational group = NON-MANUAL Total cases = 84

Threshold dB HL≥	0	5	10	15	20	25	30	35	40	45	50	55	65	75	85	95
**Prevalence**	**91.4**	**63.8**	**31.3**	**16.3**	**10.1**	**8.3**	**6.2**	**4.2**	**2.3**	**0.2**	**0.0**	**0.0**	**0.0**	**0.0**	**0.0**	**0.0**
**Lower CI**	83.2	52.1	22.3	9.5	5.3	4.1	2.8	1.5	0.6	0.0	0.0	0.0	0.0	0.0	0.0	0.0
**Upper CI**	95.8	74.0	41.9	26.6	18.3	15.9	13.3	11.2	8.1	4.7	4.4	4.4	4.4	4.4	4.4	4.4
**Number**	77	55	31	16	11	9	7	5	3	1	0	0	0	0	0	0

Table W123-42 Age Group = 41-50 Gender = FEMALE Occupational group = NON-MANUAL Total cases = 112

Threshold dB HL≥	0	5	10	15	20	25	30	35	40	45	50	55	65	75	85	95
**Prevalence**	**97.0**	**84.3**	**52.8**	**30.5**	**19.9**	**13.1**	**7.9**	**6.7**	**2.4**	**2.0**	**0.6**	**0.0**	**0.0**	**0.0**	**0.0**	**0.0**
**Lower CI**	91.4	75.4	43.1	22.7	14.1	9.0	4.8	3.9	1.0	0.7	0.1	0.0	0.0	0.0	0.0	0.0
**Upper CI**	99.0	90.4	62.2	39.6	27.3	18.6	12.9	11.2	5.9	5.7	4.3	3.3	3.3	3.3	3.3	3.3
**Number**	109	95	64	39	27	19	13	11	5	3	1	0	0	0	0	0

Prevalence by age group, gender and occupational group for the worse ear over 1, 2 and 3 kHz

Table W123-43 Age Group = 51-60 Gender = FEMALE Occupational group = NON-MANUAL Total cases = 170

	Threshold dB HL≥															
	0	5	10	15	20	25	30	35	40	45	50	55	65	75	85	95
**Prevalence**	**100.0**	**90.0**	**75.4**	**47.3**	**31.7**	**21.2**	**11.5**	**9.0**	**7.6**	**5.5**	**4.5**	**3.1**	**2.5**	**1.8**	**1.4**	**0.9**
Lower CI	97.8	84.0	68.0	39.3	25.2	16.1	8.5	6.5	5.2	3.7	2.8	1.8	1.3	0.8	0.5	0.3
Upper CI	100.0	93.9	81.6	55.5	39.0	27.5	15.4	12.3	11.0	8.1	7.1	5.3	4.8	3.6	3.4	2.9
Number	170	153	129	89	67	50	36	30	24	19	16	12	9	7	5	3

Table W123-44 Age Group = 61-70 Gender = FEMALE Occupational group = NON-MANUAL Total cases = 114

	Threshold dB HL≥															
	0	5	10	15	20	25	30	35	40	45	50	55	65	75	85	95
**Prevalence**	**98.5**	**97.1**	**90.6**	**72.7**	**53.1**	**43.3**	**34.2**	**28.2**	**18.8**	**12.6**	**9.6**	**7.9**	**4.5**	**2.4**	**2.0**	**1.2**
Lower CI	92.0	89.9	81.8	62.3	43.0	33.9	26.0	20.8	12.7	8.4	6.5	5.0	3.3	1.4	1.0	0.5
Upper CI	99.7	99.2	95.4	81.1	63.0	53.2	43.5	37.0	26.7	18.4	14.1	12.2	6.3	4.0	3.8	3.1
Number	113	112	105	87	67	56	47	41	35	27	23	19	14	8	7	5

Table W123-45 Age Group = 71-80 Gender = FEMALE Occupational group = NON-MANUAL Total cases = 67

	Threshold dB HL≥															
	0	5	10	15	20	25	30	35	40	45	50	55	65	75	85	95
**Prevalence**	**100.0**	**98.6**	**95.8**	**83.6**	**71.5**	**66.5**	**56.9**	**54.3**	**48.2**	**26.6**	**22.8**	**15.0**	**12.4**	**7.1**	**3.2**	**1.3**
Lower CI	94.6	92.2	85.0	65.8	53.8	50.1	46.7	44.9	39.6	22.3	18.6	10.4	7.9	4.2	1.2	0.2
Upper CI	100.0	99.7	98.9	93.1	84.3	79.8	66.6	63.4	56.9	31.4	27.7	21.1	19.0	11.7	8.1	6.9
Number	67	66	65	60	55	52	48	45	41	32	26	23	18	11	5	1

Table W123-46 Age Group = 18-30 Gender = MALE Occupational group = NON-MANUAL Total cases = 66

	Threshold dB HL≥															
	0	5	10	15	20	25	30	35	40	45	50	55	65	75	85	95
**Prevalence**	**81.7**	**48.0**	**22.2**	**10.0**	**1.8**	**0.4**	**0.4**	**0.1**	**0.1**	**0.1**	**0.1**	**0.1**	**0.1**	**0.1**	**0.0**	**0.0**
Lower CI	67.6	34.6	12.4	3.9	0.4	0.1	0.1	0.0	0.0	0.0	0.0	0.0	0.0	0.0	0.0	0.0
Upper CI	90.5	61.7	36.4	23.1	7.4	1.3	1.3	5.7	5.7	5.7	5.7	5.7	5.7	5.7	5.5	5.5
Number	55	33	15	6	3	2	2	1	1	1	1	1	1	1	0	0

Table W123-47 Age Group = 31-40 Gender = MALE Occupational group = NON-MANUAL Total cases = 62

	Threshold dB HL≥															
	0	5	10	15	20	25	30	35	40	45	50	55	65	75	85	95
**Prevalence**	**97.9**	**72.1**	**41.4**	**23.6**	**14.5**	**7.2**	**5.9**	**5.9**	**1.3**	**0.6**	**0.0**	**0.0**	**0.0**	**0.0**	**0.0**	**0.0**
Lower CI	89.1	54.9	26.5	12.3	7.4	2.8	1.9	1.9	0.5	0.1	0.0	0.0	0.0	0.0	0.0	0.0
Upper CI	99.6	84.6	58.1	40.5	26.7	17.5	16.7	16.7	3.3	7.0	5.8	5.8	5.8	5.8	5.8	5.8
Number	61	48	28	15	11	6	5	5	2	1	0	0	0	0	0	0

Table W123-48 Age Group = 41-50 Gender = MALE Occupational group = NON-MANUAL Total cases = 75

	Threshold dB HL≥															
	0	5	10	15	20	25	30	35	40	45	50	55	65	75	85	95
**Prevalence**	**99.0**	**84.3**	**50.5**	**41.5**	**31.6**	**24.7**	**20.5**	**10.0**	**10.0**	**6.6**	**5.3**	**2.6**	**2.3**	**2.3**	**1.6**	**0.3**
Lower CI	94.4	73.5	38.4	30.6	22.3	16.7	13.4	4.4	4.4	2.9	2.0	1.7	1.5	1.5	1.2	0.0
Upper CI	99.8	91.2	62.7	53.3	42.7	34.9	30.0	21.1	21.1	14.3	13.7	3.9	3.7	3.7	2.2	1.4
Number	74	63	41	33	24	19	15	9	9	7	6	5	4	4	2	1

Table W123-49 Age Group = 51-60 Gender = MALE Occupational group = NON-MANUAL Total cases = 139

	Threshold dB HL≥															
	0	5	10	15	20	25	30	35	40	45	50	55	65	75	85	95
**Prevalence**	**98.4**	**90.0**	**76.4**	**51.6**	**40.3**	**34.9**	**25.4**	**19.2**	**10.2**	**7.0**	**4.6**	**4.1**	**2.1**	**1.6**	**0.6**	**0.6**
Lower CI	94.2	82.6	67.6	43.2	33.0	28.0	20.5	15.8	7.2	4.7	2.9	2.5	0.9	0.6	0.2	0.2
Upper CI	99.6	94.5	83.4	59.9	48.1	42.6	30.9	23.2	14.2	10.1	7.2	6.7	5.2	4.2	2.3	2.3
Number	137	126	109	78	64	54	43	33	27	20	14	12	5	4	2	2

Prevalence by age group, gender and occupational group for the worse ear over 1, 2 and 3 kHz

Table W123-50 Age Group = 61-70 Gender = MALE Occupational group = NON-MANUAL Total cases = 106

	Threshold dB HL≥															
	0	5	10	15	20	25	30	35	40	45	50	55	65	75	85	95
Prevalence	**100.0**	**100.0**	**98.7**	**82.5**	**68.9**	**51.5**	**45.3**	**34.4**	**30.3**	**24.0**	**19.3**	**11.7**	**5.6**	**5.2**	**2.6**	**2.3**
Lower CI	96.5	96.5	95.6	66.6	53.8	38.1	33.1	28.4	24.3	19.5	13.9	8.5	4.2	3.7	0.9	0.7
Upper CI	100.0	100.0	99.6	91.8	80.9	64.7	58.2	41.0	37.1	29.1	26.1	15.9	7.5	7.2	7.3	7.1
Number	106	106	103	90	76	59	53	44	38	30	25	17	9	8	4	3

Table W123-51 Age Group = 71-80 Gender = MALE Occupational group = NON-MANUAL Total cases = 54

	Threshold dB HL≥															
	0	5	10	15	20	25	30	35	40	45	50	55	65	75	85	95
Prevalence	**100.0**	**100.0**	**97.9**	**96.1**	**85.3**	**74.3**	**60.1**	**48.3**	**44.3**	**34.5**	**24.5**	**20.8**	**10.1**	**3.7**	**2.6**	**1.3**
Lower CI	93.4	93.4	89.0	86.5	74.1	62.0	49.7	36.2	32.9	24.1	17.1	13.6	6.8	1.5	1.0	0.2
Upper CI	100.0	100.0	99.6	98.9	92.2	83.7	69.7	60.6	56.3	46.7	33.9	30.4	14.9	8.8	6.6	7.0
Number	54	54	53	52	47	41	34	28	25	20	15	12	6	3	2	1

Table W123-52 Age Group = 18-30 Gender = FEMALE Occupational group = MANUAL Total cases = 109

	Threshold dB HL≥															
	0	5	10	15	20	25	30	35	40	45	50	55	65	75	85	95
Prevalence	**87.0**	**73.2**	**39.7**	**18.0**	**9.9**	**6.2**	**4.0**	**2.9**	**2.6**	**2.2**	**0.5**	**0.2**	**0.0**	**0.0**	**0.0**	**0.0**
Lower CI	75.4	61.4	28.5	10.7	5.6	3.3	1.8	1.2	1.0	0.8	0.1	0.0	0.0	0.0	0.0	0.0
Upper CI	93.6	82.4	52.1	28.5	16.8	11.4	8.7	6.7	6.5	6.0	1.7	1.1	3.4	3.4	3.4	3.4
Number	100	81	52	28	18	13	9	8	7	6	2	1	0	0	0	0

Table W123-53 Age Group = 31-40 Gender = FEMALE Occupational group = MANUAL Total cases = 101

	Threshold dB HL≥															
	0	5	10	15	20	25	30	35	40	45	50	55	65	75	85	95
Prevalence	**92.5**	**67.4**	**38.3**	**21.3**	**13.2**	**8.4**	**6.2**	**4.1**	**4.1**	**4.1**	**1.8**	**1.2**	**0.8**	**0.5**	**0.2**	**0.0**
Lower CI	81.7	54.6	27.8	14.7	8.4	4.6	3.2	1.6	1.6	1.6	0.7	0.5	0.3	0.1	0.0	0.0
Upper CI	97.1	78.0	50.1	29.8	20.1	14.9	11.6	9.9	9.9	9.9	4.6	2.9	2.3	1.6	4.0	3.7
Number	96	77	52	37	24	16	13	7	7	7	5	4	3	2	1	0

Table W123-54 Age Group = 41-50 Gender = FEMALE Occupational group = MANUAL Total cases = 133

	Threshold dB HL≥															
	0	5	10	15	20	25	30	35	40	45	50	55	65	75	85	95
Prevalence	**97.9**	**81.1**	**60.5**	**37.5**	**23.0**	**16.3**	**12.2**	**7.7**	**4.8**	**4.3**	**4.1**	**3.7**	**2.4**	**1.1**	**0.8**	**0.3**
Lower CI	92.7	71.8	50.7	28.8	16.1	10.9	7.7	4.6	3.0	2.6	2.4	2.1	1.4	0.4	0.3	0.0
Upper CI	99.4	87.8	69.6	47.1	31.7	23.8	18.8	12.7	7.6	7.2	7.0	6.4	4.1	2.8	2.5	1.5
Number	131	114	87	59	40	32	25	19	15	13	12	11	8	4	3	1

Table W123-55 Age Group = 51-60 Gender = FEMALE Occupational group = MANUAL Total cases = 164

	Threshold dB HL≥															
	0	5	10	15	20	25	30	35	40	45	50	55	65	75	85	95
Prevalence	**99.3**	**89.3**	**81.1**	**53.9**	**37.3**	**23.6**	**18.4**	**12.5**	**6.9**	**4.7**	**4.2**	**3.8**	**2.7**	**1.0**	**0.4**	**0.4**
Lower CI	96.2	82.0	72.8	44.8	29.7	17.5	13.1	8.7	4.5	2.9	2.5	2.2	1.4	0.5	0.1	0.1
Upper CI	99.9	93.9	87.3	62.8	45.7	31.1	25.4	17.5	10.5	7.4	6.9	6.5	5.2	1.8	1.3	1.3
Number	163	151	140	109	83	59	47	37	26	21	18	16	12	6	2	2

Table W123-56 Age Group = 61-70 Gender = FEMALE Occupational group = MANUAL Total cases = 123

	Threshold dB HL≥															
	0	5	10	15	20	25	30	35	40	45	50	55	65	75	85	95
Prevalence	**100.0**	**100.0**	**89.5**	**70.8**	**52.2**	**36.4**	**22.8**	**20.9**	**19.0**	**17.4**	**10.8**	**9.7**	**8.0**	**3.2**	**2.7**	**1.9**
Lower CI	97.0	97.0	80.7	60.0	41.9	28.2	16.3	14.9	13.0	11.8	6.9	5.9	4.5	2.4	1.9	1.4
Upper CI	100.0	100.0	94.6	79.7	62.2	45.5	31.0	28.5	26.9	25.0	16.6	15.5	13.8	4.4	4.0	2.4
Number	123	123	112	95	78	62	47	45	40	37	27	22	17	7	5	2

Prevalence by age group, gender and occupational group for the worse ear over 1, 2 and 3 kHz

Table W123-57 Age Group = 71-80 Gender = FEMALE Occupational group = MANUAL Total cases = 73

	Threshold dB HL≥															
	**0**	**5**	**10**	**15**	**20**	**25**	**30**	**35**	**40**	**45**	**50**	**55**	**65**	**75**	**85**	**95**
**Prevalence**	**100.0**	**100.0**	**100.0**	**91.4**	**78.0**	**66.1**	**65.4**	**58.2**	**44.7**	**40.9**	**29.2**	**24.6**	**10.1**	**5.1**	**2.9**	**2.0**
**Lower CI**	95.0	95.0	95.0	79.2	57.6	48.0	47.4	42.9	32.8	29.0	18.3	14.2	6.5	2.9	1.5	0.8
**Upper CI**	100.0	100.0	100.0	96.7	90.3	80.4	79.9	72.0	57.3	54.0	43.1	39.2	15.2	8.9	5.6	4.7
**Number**	73	73	73	71	67	62	61	56	50	44	35	29	17	10	6	4

Table W123-58 Age Group = 18-30 Gender = MALE Occupational group = MANUAL Total cases = 109

	Threshold dB HL≥															
	**0**	**5**	**10**	**15**	**20**	**25**	**30**	**35**	**40**	**45**	**50**	**55**	**65**	**75**	**85**	**95**
**Prevalence**	**92.7**	**66.1**	**36.2**	**20.7**	**12.2**	**8.6**	**5.3**	**4.0**	**1.0**	**0.6**	**0.6**	**0.3**	**0.1**	**0.1**	**0.1**	**0.1**
**Lower CI**	84.5	53.1	25.5	12.7	7.3	4.7	2.7	1.7	0.4	0.2	0.2	0.1	0.0	0.0	0.0	0.0
**Upper CI**	96.7	77.0	48.6	32.0	19.7	15.4	10.2	9.4	2.4	1.8	1.8	1.5	0.4	0.4	0.4	0.4
**Number**	102	79	51	33	25	18	13	10	5	3	3	2	1	1	1	1

Table W123-59 Age Group = 31-40 Gender = MALE Occupational group = MANUAL Total cases = 80

	Threshold dB HL≥															
	**0**	**5**	**10**	**15**	**20**	**25**	**30**	**35**	**40**	**45**	**50**	**55**	**65**	**75**	**85**	**95**
**Prevalence**	**96.6**	**78.9**	**49.7**	**38.4**	**27.4**	**13.4**	**10.4**	**7.2**	**4.6**	**3.7**	**3.3**	**2.9**	**2.1**	**1.9**	**1.9**	**1.9**
**Lower CI**	89.6	63.8	35.4	25.2	16.1	6.5	5.0	3.7	2.0	1.3	1.1	0.8	0.4	0.3	0.3	0.3
**Upper CI**	98.9	88.8	64.0	53.5	42.7	25.8	20.5	13.4	10.6	10.0	9.8	9.8	9.9	10.2	10.2	10.2
**Number**	76	63	47	36	26	17	16	14	10	7	6	4	2	1	1	1

Table W123-60 Age Group = 41-50 Gender = MALE Occupational group = MANUAL Total cases = 123

	Threshold dB HL≥															
	**0**	**5**	**10**	**15**	**20**	**25**	**30**	**35**	**40**	**45**	**50**	**55**	**65**	**75**	**85**	**95**
**Prevalence**	**100.0**	**95.9**	**78.2**	**54.9**	**37.3**	**23.0**	**18.5**	**14.7**	**9.3**	**8.0**	**7.2**	**5.1**	**1.3**	**1.1**	**0.7**	**0.2**
**Lower CI**	97.0	88.3	67.8	44.0	27.8	16.7	13.0	9.7	5.6	4.5	3.9	2.4	0.5	0.4	0.2	0.0
**Upper CI**	100.0	98.7	86.0	65.3	47.8	30.8	25.7	21.8	15.3	13.9	12.8	10.8	3.1	3.0	1.9	1.1
**Number**	123	119	101	78	60	46	38	30	22	18	17	11	5	4	3	1

Table W123-61 Age Group = 51-60 Gender = MALE Occupational group = MANUAL Total cases = 192

	Threshold dB HL≥															
	**0**	**5**	**10**	**15**	**20**	**25**	**30**	**35**	**40**	**45**	**50**	**55**	**65**	**75**	**85**	**95**
**Prevalence**	**100.0**	**98.4**	**88.2**	**67.3**	**58.6**	**48.4**	**34.7**	**26.4**	**21.5**	**19.7**	**16.3**	**13.2**	**6.6**	**3.0**	**1.8**	**1.1**
**Lower CI**	98.0	94.4	80.9	58.9	50.3	40.6	28.4	21.1	16.6	14.9	12.0	9.4	3.8	1.6	0.8	0.4
**Upper CI**	100.0	99.6	92.9	74.7	66.4	56.3	41.7	32.5	27.4	25.6	21.7	18.2	11.1	5.3	3.7	2.9
**Number**	192	190	176	150	133	115	90	74	62	56	47	38	20	12	7	4

Table W123-62 Age Group = 61-70 Gender = MALE Occupational group = MANUAL Total cases = 169

	Threshold dB HL≥															
	**0**	**5**	**10**	**15**	**20**	**25**	**30**	**35**	**40**	**45**	**50**	**55**	**65**	**75**	**85**	**95**
**Prevalence**	**100.0**	**98.8**	**95.8**	**90.9**	**74.7**	**65.4**	**54.1**	**47.8**	**38.2**	**31.8**	**27.7**	**18.1**	**11.4**	**8.4**	**2.3**	**1.1**
**Lower CI**	97.8	93.3	89.0	84.5	64.6	55.5	44.1	38.4	30.7	24.3	20.8	12.6	7.0	4.4	1.2	0.5
**Upper CI**	100.0	99.8	98.4	94.8	82.6	74.1	63.8	57.5	46.3	40.3	35.9	25.4	18.2	15.5	4.4	2.7
**Number**	169	168	165	158	144	130	115	107	92	78	68	51	33	23	10	5

Table W123-63 Age Group = 71-80 Gender = MALE Occupational group = MANUAL Total cases = 71

	Threshold dB HL≥															
	**0**	**5**	**10**	**15**	**20**	**25**	**30**	**35**	**40**	**45**	**50**	**55**	**65**	**75**	**85**	**95**
**Prevalence**	**100.0**	**100.0**	**94.2**	**94.2**	**92.5**	**89.2**	**85.2**	**64.6**	**48.4**	**41.7**	**38.9**	**35.7**	**20.0**	**14.0**	**7.7**	**5.5**
**Lower CI**	94.9	94.9	72.4	72.4	73.3	70.8	65.3	47.9	35.0	29.0	26.1	23.1	13.3	9.2	4.8	3.1
**Upper CI**	100.0	100.0	99.0	99.0	98.2	96.6	94.6	78.3	62.1	55.7	53.5	50.6	28.8	20.8	12.2	9.7
**Number**	71	71	70	70	68	66	65	57	48	43	40	38	26	19	11	7

Prevalence by age group, gender and occupational group for the worse ear over 1, 2 and 4 kHz

Table W124-1 Age Group = 18-80 Gender = OVERALL Occupational group = OVERALL Total cases = 2651

	Threshold dB HL≥															
	**0**	**5**	**10**	**15**	**20**	**25**	**30**	**35**	**40**	**45**	**50**	**55**	**65**	**75**	**85**	**95**
**Prevalence**	**97.2**	**84.3**	**64.7**	**49.0**	**37.5**	**28.8**	**22.6**	**17.5**	**13.7**	**11.0**	**8.7**	**7.0**	**4.2**	**2.5**	**1.5**	**1.0**
**Lower CI**	96.0	82.3	62.6	47.1	35.8	27.2	21.2	16.3	12.6	10.1	7.9	6.3	3.6	2.1	1.2	0.7
**Upper CI**	98.0	86.1	66.7	50.9	39.3	30.4	24.0	18.7	14.8	12.0	9.5	7.8	4.8	2.9	1.9	1.3
**Number**	2613	2415	2045	1683	1388	1164	976	809	679	574	468	392	257	168	101	67

Table W124-2 Age Group = 18-80 Gender = FEMALE Occupational group = OVERALL Total cases = 1381

	Threshold dB HL≥															
	**0**	**5**	**10**	**15**	**20**	**25**	**30**	**35**	**40**	**45**	**50**	**55**	**65**	**75**	**85**	**95**
**Prevalence**	**96.9**	**83.0**	**62.2**	**45.1**	**32.3**	**23.5**	**18.6**	**14.3**	**11.5**	**9.0**	**7.0**	**5.6**	**3.5**	**1.9**	**1.2**	**0.9**
**Lower CI**	95.3	80.5	59.6	42.7	30.2	21.5	16.7	12.7	10.1	7.9	6.0	4.8	2.9	1.5	0.9	0.6
**Upper CI**	97.9	85.3	64.8	47.6	34.5	25.6	20.5	16.1	13.0	10.3	8.1	6.6	4.3	2.4	1.6	1.3
**Number**	1357	1231	1007	797	607	489	409	335	286	240	193	163	116	74	43	31

Table W124-3 Age Group = 18-80 Gender = MALE Occupational group = OVERALL Total cases = 1270

	Threshold dB HL≥															
	**0**	**5**	**10**	**15**	**20**	**25**	**30**	**35**	**40**	**45**	**50**	**55**	**65**	**75**	**85**	**95**
**Prevalence**	**97.5**	**85.7**	**67.5**	**53.3**	**43.4**	**34.7**	**27.0**	**21.0**	**16.1**	**13.2**	**10.5**	**8.5**	**5.0**	**3.1**	**1.8**	**1.1**
**Lower CI**	95.6	82.5	64.3	50.3	40.6	32.4	24.9	19.3	14.6	11.8	9.3	7.4	4.1	2.4	1.3	0.7
**Upper CI**	98.6	88.4	70.6	56.4	46.2	37.1	29.2	22.8	17.7	14.7	12.0	9.8	6.0	3.9	2.5	1.7
**Number**	1256	1184	1038	886	781	675	567	474	393	334	275	229	141	94	58	36

Table W124-4 Age Group = 18-30 Gender = OVERALL Occupational group = OVERALL Total cases = 401

	Threshold dB HL≥															
	**0**	**5**	**10**	**15**	**20**	**25**	**30**	**35**	**40**	**45**	**50**	**55**	**65**	**75**	**85**	**95**
**Prevalence**	**92.5**	**62.9**	**33.6**	**17.6**	**10.0**	**6.1**	**4.4**	**2.5**	**1.7**	**1.1**	**0.4**	**0.3**	**0.2**	**0.0**	**0.0**	**0.0**
**Lower CI**	88.6	57.1	28.5	13.7	7.3	4.2	3.0	1.6	1.0	0.6	0.2	0.1	0.0	0.0	0.0	0.0
**Upper CI**	95.2	68.4	39.0	22.2	13.5	8.8	6.5	4.0	2.8	2.2	1.0	0.8	0.6	0.1	0.1	0.1
**Number**	377	273	165	92	62	43	36	24	19	12	6	5	3	2	1	1

Table W124-5 Age Group = 31-40 Gender = OVERALL Occupational group = OVERALL Total cases = 331

	Threshold dB HL≥															
	**0**	**5**	**10**	**15**	**20**	**25**	**30**	**35**	**40**	**45**	**50**	**55**	**65**	**75**	**85**	**95**
**Prevalence**	**96.5**	**78.1**	**46.2**	**28.9**	**20.0**	**11.5**	**9.2**	**7.4**	**4.7**	**3.2**	**2.3**	**2.0**	**1.1**	**0.9**	**0.8**	**0.7**
**Lower CI**	93.5	72.1	39.9	23.5	15.5	8.4	6.4	5.1	3.0	1.9	1.2	1.0	0.4	0.2	0.2	0.2
**Upper CI**	98.2	83.1	52.6	34.8	25.3	15.6	12.9	10.7	7.5	5.4	4.2	3.9	3.1	3.0	3.0	3.0
**Number**	322	274	183	124	90	59	50	42	28	21	16	14	6	4	3	2

Table W124-6 Age Group = 41-50 Gender = OVERALL Occupational group = OVERALL Total cases = 450

	Threshold dB HL≥															
	**0**	**5**	**10**	**15**	**20**	**25**	**30**	**35**	**40**	**45**	**50**	**55**	**65**	**75**	**85**	**95**
**Prevalence**	**98.8**	**91.4**	**66.8**	**45.8**	**31.4**	**22.4**	**16.9**	**11.3**	**7.8**	**6.5**	**5.6**	**4.6**	**2.8**	**1.6**	**1.3**	**0.4**
**Lower CI**	96.9	87.8	61.8	41.0	27.2	19.0	13.9	9.0	5.9	4.8	4.1	3.2	1.8	1.0	0.7	0.2
**Upper CI**	99.5	94.0	71.4	50.7	35.9	26.3	20.4	14.2	10.1	8.7	7.7	6.6	4.4	2.8	2.4	1.0
**Number**	446	420	324	240	175	138	108	82	61	50	43	35	24	17	13	6

Table W124-7 Age Group = 51-60 Gender = OVERALL Occupational group = OVERALL Total cases = 677

	Threshold dB HL≥															
	**0**	**5**	**10**	**15**	**20**	**25**	**30**	**35**	**40**	**45**	**50**	**55**	**65**	**75**	**85**	**95**
**Prevalence**	**99.8**	**97.5**	**86.7**	**66.4**	**49.3**	**38.0**	**27.6**	**19.1**	**14.2**	**11.9**	**9.8**	**7.8**	**5.2**	**3.2**	**1.9**	**1.5**
**Lower CI**	98.9	95.8	83.5	62.2	45.3	34.4	24.5	16.6	12.1	10.0	8.0	6.3	3.9	2.2	1.2	0.9
**Upper CI**	100.0	98.5	89.4	70.3	53.3	41.8	30.9	21.9	16.6	14.2	11.9	9.7	6.9	4.5	3.1	2.6
**Number**	676	661	602	498	402	329	261	201	162	138	115	93	63	44	24	20

Prevalence by age group, gender and occupational group for the worse ear over 1, 2 and 4 kHz

Table W124-8 Age Group = 61-70 Gender = OVERALL Occupational group = OVERALL Total cases = 522

	Threshold dB HL≥															
	0	5	10	15	20	25	30	35	40	45	50	55	65	75	85	95
Prevalence	**100.0**	**99.0**	**95.6**	**86.5**	**70.1**	**57.4**	**44.9**	**33.8**	**28.9**	**23.4**	**18.8**	**14.7**	**8.4**	**5.1**	**2.7**	**1.7**
Lower CI	99.3	97.2	92.8	82.5	64.9	52.3	40.2	29.7	25.2	20.0	15.8	12.1	6.5	3.8	1.9	1.2
Upper CI	100.0	99.6	97.3	89.7	74.8	62.3	49.6	38.2	33.0	27.1	22.2	17.7	10.9	6.9	3.8	2.6
Number	522	518	504	467	405	358	299	253	228	192	157	133	84	53	31	21

Table W124-9 Age Group = 71-80 Gender = OVERALL Occupational group = OVERALL Total cases = 270

	Threshold dB HL≥															
	0	5	10	15	20	25	30	35	40	45	50	55	65	75	85	95
Prevalence	**100.0**	**99.6**	**97.6**	**93.8**	**89.3**	**75.9**	**66.8**	**62.1**	**50.9**	**40.9**	**32.4**	**26.7**	**15.8**	**8.2**	**4.8**	**2.8**
Lower CI	98.6	98.0	92.0	87.6	82.9	67.6	58.9	54.1	43.7	35.0	27.0	21.6	12.3	6.3	3.3	1.8
Upper CI	100.0	99.9	99.3	97.0	93.5	82.6	73.9	69.5	58.0	47.0	38.3	32.4	20.1	10.6	6.8	4.6
Number	270	269	267	262	254	237	222	207	181	161	131	112	77	48	29	17

Table W124-10 Age Group = 18-80 Gender = OVERALL Occupational group = NON-MANUAL Total cases = 1181

	Threshold dB HL≥															
	0	5	10	15	20	25	30	35	40	45	50	55	65	75	85	95
Prevalence	**96.4**	**81.6**	**60.3**	**43.9**	**33.4**	**26.4**	**19.8**	**15.2**	**12.4**	**8.9**	**6.8**	**4.9**	**2.9**	**1.9**	**1.1**	**0.8**
Lower CI	94.3	78.6	57.2	41.2	30.8	24.2	18.1	13.7	11.0	7.9	6.0	4.1	2.4	1.5	0.8	0.5
Upper CI	97.7	84.3	63.2	46.6	36.0	28.8	21.6	16.9	13.9	10.1	7.7	5.8	3.5	2.4	1.6	1.2
Number	1158	1044	843	661	530	439	358	289	241	200	158	128	87	61	37	28

Table W124-11 Age Group = 18-80 Gender = OVERALL Occupational group = MANUAL Total cases = 1466

	Threshold dB HL≥															
	0	5	10	15	20	25	30	35	40	45	50	55	65	75	85	95
Prevalence	**97.6**	**87.4**	**69.3**	**53.9**	**40.7**	**30.9**	**24.6**	**19.2**	**14.8**	**12.3**	**9.9**	**8.0**	**4.8**	**2.8**	**1.7**	**1.1**
Lower CI	95.8	84.4	66.0	50.9	38.2	28.6	22.5	17.5	13.3	11.0	8.8	6.9	4.0	2.2	1.3	0.8
Upper CI	98.6	89.9	72.3	56.9	43.3	33.2	26.7	21.1	16.3	13.8	11.2	9.2	5.7	3.5	2.2	1.5
Number	1451	1369	1200	1020	856	723	617	519	437	374	310	264	170	107	64	39

Table W124-12 Age Group = 18-30 Gender = FEMALE Occupational group = OVERALL Total cases = 224

	Threshold dB HL≥															
	0	5	10	15	20	25	30	35	40	45	50	55	65	75	85	95
Prevalence	**93.2**	**62.2**	**32.2**	**15.0**	**7.4**	**4.6**	**4.1**	**3.1**	**2.4**	**1.6**	**0.5**	**0.3**	**0.2**	**0.0**	**0.0**	**0.0**
Lower CI	88.2	54.6	25.9	10.9	4.9	2.9	2.5	1.7	1.3	0.7	0.2	0.1	0.0	0.0	0.0	0.0
Upper CI	96.1	69.2	39.2	20.2	10.8	7.3	6.8	5.4	4.4	3.5	1.6	1.3	1.3	1.7	1.7	1.7
Number	211	150	88	49	29	20	18	14	12	7	3	2	1	0	0	0

Table W124-13 Age Group = 31-40 Gender = FEMALE Occupational group = OVERALL Total cases = 189

	Threshold dB HL≥															
	0	5	10	15	20	25	30	35	40	45	50	55	65	75	85	95
Prevalence	**93.7**	**72.3**	**40.5**	**22.1**	**14.6**	**9.2**	**7.7**	**6.7**	**4.1**	**3.3**	**2.4**	**2.0**	**1.1**	**0.6**	**0.4**	**0.3**
Lower CI	87.9	64.1	33.0	16.6	10.4	6.1	4.9	4.1	2.3	1.8	1.3	1.0	0.5	0.2	0.1	0.1
Upper CI	96.8	79.1	48.4	28.8	20.2	13.6	11.9	10.9	7.4	6.1	4.4	3.8	2.7	1.9	1.6	1.6
Number	181	147	98	61	44	30	26	20	14	12	10	9	5	3	2	1

Table W124-14 Age Group = 41-50 Gender = FEMALE Occupational group = OVERALL Total cases = 247

	Threshold dB HL≥															
	0	5	10	15	20	25	30	35	40	45	50	55	65	75	85	95
Prevalence	**98.2**	**89.7**	**60.6**	**39.6**	**22.3**	**15.0**	**11.9**	**6.5**	**4.6**	**3.7**	**3.2**	**2.5**	**1.6**	**1.2**	**0.7**	**0.4**
Lower CI	95.0	84.4	53.8	33.4	17.6	11.4	8.7	4.6	3.1	2.4	2.0	1.4	0.8	0.6	0.3	0.1
Upper CI	99.4	93.4	67.1	46.1	27.8	19.5	16.1	8.9	6.7	5.7	5.2	4.4	3.0	2.4	1.7	1.1
Number	244	228	164	116	72	54	45	33	24	19	16	13	9	8	5	3

Prevalence by age group, gender and occupational group for the worse ear over 1, 2 and 4 kHz

Table W124-15 Age Group = 51-60 Gender = FEMALE Occupational group = OVERALL Total cases = 339

	Threshold dB HL≥															
	**0**	**5**	**10**	**15**	**20**	**25**	**30**	**35**	**40**	**45**	**50**	**55**	**65**	**75**	**85**	**95**
**Prevalence**	**100.0**	**96.8**	**83.6**	**62.5**	**40.3**	**26.7**	**17.7**	**12.2**	**9.0**	**6.9**	**4.9**	**4.3**	**3.5**	**2.3**	**1.4**	**1.4**
**Lower CI**	98.9	94.0	78.9	56.6	34.8	22.3	14.1	9.4	6.7	5.0	3.4	2.8	2.2	1.4	0.6	0.6
**Upper CI**	100.0	98.3	87.5	68.1	46.0	31.6	21.9	15.8	11.8	9.4	7.1	6.4	5.6	4.0	3.2	3.2
**Number**	339	328	291	236	170	126	97	75	59	48	38	32	26	20	9	9

Table W124-16 Age Group = 61-70 Gender = FEMALE Occupational group = OVERALL Total cases = 241

	Threshold dB HL≥															
	**0**	**5**	**10**	**15**	**20**	**25**	**30**	**35**	**40**	**45**	**50**	**55**	**65**	**75**	**85**	**95**
**Prevalence**	**100.0**	**98.5**	**93.1**	**78.7**	**61.3**	**47.2**	**35.8**	**24.0**	**21.9**	**16.8**	**13.1**	**10.7**	**7.2**	**3.0**	**2.4**	**1.7**
**Lower CI**	98.4	95.3	88.1	71.8	54.0	40.4	29.9	18.9	17.0	12.4	9.4	7.4	4.6	2.0	1.4	1.0
**Upper CI**	100.0	99.5	96.0	84.3	68.1	54.0	42.1	30.1	27.7	22.3	18.0	15.1	10.9	4.6	4.0	3.0
**Number**	241	238	227	200	162	138	110	88	82	68	55	48	35	18	13	10

Table W124-17 Age Group = 71-80 Gender = FEMALE Occupational group = OVERALL Total cases = 141

	Threshold dB HL≥															
	**0**	**5**	**10**	**15**	**20**	**25**	**30**	**35**	**40**	**45**	**50**	**55**	**65**	**75**	**85**	**95**
**Prevalence**	**100.0**	**99.4**	**97.0**	**91.3**	**86.0**	**68.6**	**60.8**	**57.1**	**47.0**	**37.7**	**31.2**	**24.4**	**13.7**	**7.3**	**4.0**	**2.3**
**Lower CI**	97.3	96.8	87.6	81.5	75.9	56.1	49.2	45.6	37.4	30.8	24.6	18.5	9.9	5.1	2.4	1.2
**Upper CI**	100.0	99.9	99.3	96.2	92.2	78.9	71.3	67.9	56.8	45.1	38.6	31.6	18.6	10.3	6.5	4.5
**Number**	141	140	139	135	130	121	113	105	95	86	71	59	40	25	14	8

Table W124-18 Age Group = 18-30 Gender = MALE Occupational group = OVERALL Total cases = 177

	Threshold dB HL≥															
	**0**	**5**	**10**	**15**	**20**	**25**	**30**	**35**	**40**	**45**	**50**	**55**	**65**	**75**	**85**	**95**
**Prevalence**	**91.9**	**63.6**	**34.8**	**20.1**	**12.6**	**7.6**	**4.5**	**1.9**	**0.9**	**0.6**	**0.3**	**0.3**	**0.1**	**0.1**	**0.0**	**0.0**
**Lower CI**	85.1	54.5	27.2	14.0	8.1	4.5	2.5	0.9	0.4	0.2	0.1	0.1	0.0	0.0	0.0	0.0
**Upper CI**	95.8	71.8	43.3	28.0	19.2	12.5	8.0	3.9	2.0	1.5	1.0	1.0	0.2	0.2	0.2	0.2
**Number**	166	123	77	43	33	23	18	10	7	5	3	3	2	2	1	1

Table W124-19 Age Group = 31-40 Gender = MALE Occupational group = OVERALL Total cases = 142

	Threshold dB HL≥															
	**0**	**5**	**10**	**15**	**20**	**25**	**30**	**35**	**40**	**45**	**50**	**55**	**65**	**75**	**85**	**95**
**Prevalence**	**99.7**	**84.4**	**52.4**	**36.5**	**26.1**	**14.1**	**10.7**	**8.3**	**5.4**	**3.1**	**2.2**	**2.0**	**1.2**	**1.2**	**1.2**	**1.2**
**Lower CI**	98.1	74.7	42.0	27.4	18.3	8.8	6.3	4.8	2.7	1.3	0.8	0.6	0.2	0.2	0.2	0.2
**Upper CI**	99.9	90.9	62.5	46.8	35.7	21.6	17.5	14.0	10.6	7.2	6.4	6.4	6.5	6.5	6.5	6.5
**Number**	141	127	85	63	46	29	24	22	14	9	6	5	1	1	1	1

Table W124-20 Age Group = 41-50 Gender = MALE Occupational group = OVERALL Total cases = 203

	Threshold dB HL≥															
	**0**	**5**	**10**	**15**	**20**	**25**	**30**	**35**	**40**	**45**	**50**	**55**	**65**	**75**	**85**	**95**
**Prevalence**	**99.4**	**93.2**	**73.5**	**52.6**	**41.4**	**30.6**	**22.3**	**16.7**	**11.2**	**9.6**	**8.2**	**6.9**	**4.2**	**2.0**	**1.8**	**0.5**
**Lower CI**	96.6	87.9	66.0	45.1	34.4	24.8	17.3	12.4	7.9	6.6	5.5	4.4	2.3	1.0	0.8	0.2
**Upper CI**	99.9	96.3	79.9	60.0	48.7	37.0	28.4	22.1	15.6	13.9	12.2	10.8	7.4	4.3	4.1	1.6
**Number**	202	192	160	124	103	84	63	49	37	31	27	22	15	9	8	3

Table W124-21 Age Group = 51-60 Gender = MALE Occupational group = OVERALL Total cases = 338

	Threshold dB HL≥															
	**0**	**5**	**10**	**15**	**20**	**25**	**30**	**35**	**40**	**45**	**50**	**55**	**65**	**75**	**85**	**95**
**Prevalence**	**99.6**	**98.3**	**90.1**	**70.7**	**59.3**	**50.7**	**38.7**	**26.7**	**20.1**	**17.5**	**15.2**	**11.7**	**7.0**	**4.2**	**2.5**	**1.7**
**Lower CI**	97.6	95.6	85.6	64.8	53.4	45.0	33.5	22.6	16.5	14.1	11.9	9.0	4.9	2.6	1.4	0.9
**Upper CI**	99.9	99.3	93.3	76.0	65.0	56.5	44.1	31.3	24.2	21.6	19.1	15.1	10.0	6.6	4.5	3.4
**Number**	337	333	311	262	232	203	164	126	103	90	77	61	37	24	15	11

Prevalence by age group, gender and occupational group for the worse ear over 1, 2 and 4 kHz

Table W124-22 Age Group = 61-70 Gender = MALE Occupational group = OVERALL Total cases = 281

	Threshold dB HL≥															
	0	5	10	15	20	25	30	35	40	45	50	55	65	75	85	95
Prevalence	100.0	99.5	98.6	95.5	80.1	69.3	55.4	45.2	37.1	31.0	25.4	19.5	9.9	7.5	3.0	1.7
Lower CI	98.7	97.5	95.7	92.0	72.6	61.6	48.2	38.9	31.7	26.2	21.0	15.8	7.2	5.1	1.9	0.9
Upper CI	100.0	99.9	99.5	97.5	86.0	76.1	62.4	51.6	42.8	36.3	30.5	23.9	13.3	11.1	4.8	3.1
Number	281	280	277	267	243	220	189	165	146	124	102	85	49	35	18	11

Table W124-23 Age Group = 71-80 Gender = MALE Occupational group = OVERALL Total cases = 129

	Threshold dB HL≥															
	0	5	10	15	20	25	30	35	40	45	50	55	65	75	85	95
Prevalence	100.0	100.0	98.7	97.3	94.3	86.1	74.8	68.5	56.1	45.4	34.3	30.2	19.1	9.6	6.1	3.7
Lower CI	97.1	97.1	92.7	91.3	88.6	77.3	65.4	58.9	46.0	35.8	25.8	22.1	13.0	6.5	3.6	1.9
Upper CI	100.0	100.0	99.8	99.2	97.3	91.8	82.4	76.7	65.8	55.4	43.9	39.7	27.2	14.1	9.9	7.0
Number	129	129	128	127	124	116	109	102	86	75	60	53	37	23	15	9

Table W124-24 Age Group = 18-30 Gender = OVERALL Occupational group = NON-MANUAL Total cases = 181

	Threshold dB HL≥															
	0	5	10	15	20	25	30	35	40	45	50	55	65	75	85	95
Prevalence	89.5	55.5	25.9	11.9	6.3	4.8	2.1	1.2	1.2	0.7	0.3	0.3	0.3	0.1	0.0	0.0
Lower CI	81.9	47.0	19.1	7.5	3.2	2.1	1.2	0.5	0.5	0.2	0.1	0.1	0.1	0.0	0.0	0.0
Upper CI	94.1	63.7	34.0	18.4	11.9	10.3	3.8	2.7	2.7	2.5	1.0	1.0	1.0	2.2	2.1	2.1
Number	166	107	54	25	16	13	10	6	6	3	2	2	2	1	0	0

Table W124-25 Age Group = 31-40 Gender = OVERALL Occupational group = NON-MANUAL Total cases = 148

	Threshold dB HL≥															
	0	5	10	15	20	25	30	35	40	45	50	55	65	75	85	95
Prevalence	96.6	77.3	42.0	22.9	17.1	10.1	8.4	6.9	3.1	1.9	0.9	0.6	0.3	0.3	0.3	0.3
Lower CI	92.6	68.4	33.1	15.6	10.9	6.0	4.8	3.7	1.5	0.8	0.4	0.2	0.1	0.1	0.1	0.1
Upper CI	98.5	84.3	51.5	32.2	26.0	16.6	14.4	12.5	6.5	4.3	2.3	2.3	1.7	1.7	1.7	1.7
Number	143	119	72	39	30	20	17	14	8	5	3	2	1	1	1	1

Table W124-26 Age Group = 41-50 Gender = OVERALL Occupational group = NON-MANUAL Total cases = 188

	Threshold dB HL≥															
	0	5	10	15	20	25	30	35	40	45	50	55	65	75	85	95
Prevalence	98.9	90.0	60.7	37.6	26.2	20.6	13.7	10.2	6.5	4.5	4.3	2.8	1.6	1.5	1.2	0.5
Lower CI	96.1	84.2	53.0	31.0	20.7	16.0	9.2	6.1	3.5	2.4	2.2	1.2	0.9	0.8	0.7	0.1
Upper CI	99.7	93.8	67.9	44.7	32.5	26.2	20.0	16.3	11.8	8.3	8.1	6.7	2.7	2.6	2.2	1.9
Number	186	170	119	81	53	43	30	23	16	11	10	7	6	5	4	2

Table W124-27 Age Group = 51-60 Gender = OVERALL Occupational group = NON-MANUAL Total cases = 316

	Threshold dB HL≥															
	0	5	10	15	20	25	30	35	40	45	50	55	65	75	85	95
Prevalence	99.7	96.4	83.0	58.2	42.6	32.9	21.8	13.7	11.2	7.8	6.1	5.0	3.6	2.9	1.6	1.6
Lower CI	98.2	93.6	78.0	52.2	37.3	28.2	18.3	10.2	8.0	6.0	4.5	3.6	2.2	1.7	0.8	0.8
Upper CI	99.9	98.0	87.0	64.0	48.1	37.9	25.7	18.2	15.5	10.2	8.2	7.1	5.7	4.8	3.2	3.2
Number	315	303	262	200	157	126	94	72	60	50	40	33	23	19	11	11

Table W124-28 Age Group = 61-70 Gender = OVERALL Occupational group = NON-MANUAL Total cases = 225

	Threshold dB HL≥															
	0	5	10	15	20	25	30	35	40	45	50	55	65	75	85	95
Prevalence	100.0	98.4	95.1	88.2	67.7	53.5	43.5	31.9	27.8	21.3	17.3	12.5	5.5	3.9	2.7	1.8
Lower CI	98.3	94.3	90.3	82.5	59.1	45.1	37.2	27.0	23.3	17.5	14.2	9.4	4.6	3.0	1.6	1.0
Upper CI	100.0	99.6	97.6	92.2	75.2	61.6	50.1	37.3	32.8	25.7	21.0	16.4	6.7	5.1	4.7	3.1
Number	225	223	215	199	163	134	115	92	81	68	55	47	28	19	14	11

Prevalence by age group, gender and occupational group for the worse ear over 1, 2 and 4 kHz

Table W124-29 Age Group = 71-80 Gender = OVERALL Occupational group = NON-MANUAL Total cases = 123

	Threshold dB HL≥															
	**0**	**5**	**10**	**15**	**20**	**25**	**30**	**35**	**40**	**45**	**50**	**55**	**65**	**75**	**85**	**95**
**Prevalence**	**100.0**	**99.1**	**98.3**	**94.2**	**86.1**	**77.8**	**63.7**	**57.4**	**50.0**	**35.4**	**25.6**	**18.1**	**13.1**	**6.1**	**2.6**	**1.5**
**Lower CI**	97.0	95.1	94.0	87.7	73.6	66.0	56.1	49.8	42.3	29.7	21.1	13.8	9.3	4.0	1.2	0.5
**Upper CI**	100.0	99.8	99.5	97.3	93.2	86.4	70.7	64.6	57.7	41.6	30.6	23.4	18.1	9.1	5.6	4.5
**Number**	123	122	121	117	111	103	92	82	70	63	48	37	27	16	7	3

Table W124-30 Age Group = 18-30 Gender = OVERALL Occupational group = MANUAL Total cases = 218

	Threshold dB HL≥															
	**0**	**5**	**10**	**15**	**20**	**25**	**30**	**35**	**40**	**45**	**50**	**55**	**65**	**75**	**85**	**95**
**Prevalence**	**94.0**	**72.0**	**42.0**	**24.8**	**12.5**	**7.0**	**5.7**	**3.4**	**2.0**	**1.5**	**0.4**	**0.3**	**0.0**	**0.0**	**0.0**	**0.0**
**Lower CI**	87.9	63.0	33.7	17.9	8.4	4.6	3.6	1.9	1.0	0.6	0.1	0.1	0.0	0.0	0.0	0.0
**Upper CI**	97.1	79.4	50.9	33.2	18.3	10.8	9.1	6.1	4.0	3.5	1.1	0.9	0.2	0.2	0.2	0.2
**Number**	209	166	111	67	46	30	26	18	13	9	4	3	1	1	1	1

Table W124-31 Age Group = 31-40 Gender = OVERALL Occupational group = MANUAL Total cases = 183

	Threshold dB HL≥															
	**0**	**5**	**10**	**15**	**20**	**25**	**30**	**35**	**40**	**45**	**50**	**55**	**65**	**75**	**85**	**95**
**Prevalence**	**96.7**	**79.1**	**50.0**	**33.9**	**21.9**	**12.9**	**10.1**	**8.1**	**6.4**	**4.3**	**3.2**	**2.8**	**1.7**	**1.2**	**1.0**	**0.9**
**Lower CI**	90.9	70.0	40.7	26.4	15.8	8.3	6.1	4.8	3.4	2.3	1.6	1.3	0.5	0.3	0.2	0.2
**Upper CI**	98.8	86.0	59.3	42.3	29.5	19.5	16.4	13.2	11.6	8.0	6.3	5.9	5.0	4.8	4.9	5.0
**Number**	179	155	111	85	60	39	33	28	20	16	13	12	5	3	2	1

Table W124-32 Age Group = 41-50 Gender = OVERALL Occupational group = MANUAL Total cases = 262

	Threshold dB HL≥															
	**0**	**5**	**10**	**15**	**20**	**25**	**30**	**35**	**40**	**45**	**50**	**55**	**65**	**75**	**85**	**95**
**Prevalence**	**98.9**	**93.3**	**73.4**	**54.7**	**37.3**	**25.8**	**21.2**	**13.9**	**9.3**	**8.1**	**7.0**	**6.0**	**3.9**	**2.0**	**1.7**	**0.4**
**Lower CI**	96.2	88.4	66.6	47.5	30.8	20.6	16.4	10.3	6.8	5.7	4.8	3.9	2.2	0.9	0.7	0.2
**Upper CI**	99.7	96.2	79.3	61.6	44.3	31.9	26.9	18.5	12.6	11.4	10.1	9.1	6.8	4.3	4.0	1.0
**Number**	260	250	205	159	122	95	78	59	45	39	33	28	18	12	9	4

Table W124-33 Age Group = 51-60 Gender = OVERALL Occupational group = MANUAL Total cases = 361

	Threshold dB HL≥															
	**0**	**5**	**10**	**15**	**20**	**25**	**30**	**35**	**40**	**45**	**50**	**55**	**65**	**75**	**85**	**95**
**Prevalence**	**100.0**	**98.8**	**91.0**	**73.1**	**53.9**	**41.9**	**31.6**	**22.3**	**16.1**	**13.5**	**11.4**	**8.7**	**5.8**	**2.9**	**1.7**	**1.2**
**Lower CI**	98.9	95.9	86.5	67.0	47.9	36.6	27.1	18.6	13.0	10.8	9.0	6.7	4.0	1.9	0.9	0.6
**Upper CI**	100.0	99.6	94.1	78.5	59.8	47.5	36.4	26.4	19.7	16.8	14.5	11.4	8.2	4.4	3.0	2.4
**Number**	361	358	340	298	245	203	167	129	102	88	75	60	40	25	13	9

Table W124-34 Age Group = 61-70 Gender = OVERALL Occupational group = MANUAL Total cases = 296

	Threshold dB HL≥															
	**0**	**5**	**10**	**15**	**20**	**25**	**30**	**35**	**40**	**45**	**50**	**55**	**65**	**75**	**85**	**95**
**Prevalence**	**100.0**	**99.2**	**95.7**	**84.4**	**71.3**	**60.0**	**45.2**	**35.4**	**30.3**	**26.1**	**20.2**	**15.5**	**10.0**	**6.1**	**2.7**	**1.9**
**Lower CI**	98.7	96.8	91.0	78.1	64.0	52.8	39.1	29.7	25.3	21.2	16.1	11.9	6.9	4.0	1.9	1.3
**Upper CI**	100.0	99.8	98.0	89.2	77.7	66.8	51.5	41.5	35.8	31.8	25.1	20.0	14.3	9.3	3.8	2.7
**Number**	296	294	288	267	241	223	184	161	147	124	102	86	56	34	17	10

Table W124-35 Age Group = 71-80 Gender = OVERALL Occupational group = MANUAL Total cases = 146

	Threshold dB HL≥															
	**0**	**5**	**10**	**15**	**20**	**25**	**30**	**35**	**40**	**45**	**50**	**55**	**65**	**75**	**85**	**95**
**Prevalence**	**100.0**	**100.0**	**97.4**	**94.9**	**94.4**	**77.0**	**69.4**	**66.9**	**52.8**	**45.1**	**38.8**	**32.2**	**16.2**	**9.4**	**6.6**	**4.3**
**Lower CI**	97.4	97.4	86.6	87.2	87.0	64.3	56.9	54.2	43.2	35.9	29.8	23.4	12.4	6.8	4.5	2.8
**Upper CI**	100.0	100.0	99.6	98.0	97.7	86.2	79.7	77.5	62.2	54.6	48.5	42.3	21.0	12.9	9.6	6.6
**Number**	146	146	145	144	142	133	129	124	110	98	83	75	50	32	22	14

Prevalence by age group, gender and occupational group for the worse ear over 1, 2 and 4 kHz

Table W124-36 Age Group = 18-80 Gender = FEMALE Occupational group = NON-MANUAL Total cases = 670

Threshold dB HL≥	0	5	10	15	20	25	30	35	40	45	50	55	65	75	85	95
**Prevalence**	**97.0**	**80.5**	**59.4**	**42.2**	**30.0**	**23.4**	**17.9**	**13.8**	**11.7**	**7.5**	**5.8**	**4.1**	**2.9**	**1.9**	**1.2**	**1.0**
Lower CI	94.9	77.0	55.9	39.1	27.0	20.7	15.8	12.0	10.1	6.3	4.8	3.2	2.3	1.4	0.8	0.6
Upper CI	98.2	83.6	62.8	45.3	33.3	26.3	20.2	15.8	13.5	8.9	6.9	5.1	3.8	2.7	2.0	1.7
Number	656	579	458	353	264	218	181	147	125	101	81	67	51	37	23	18

Table W124-37 Age Group = 18-80 Gender = MALE Occupational group = NON-MANUAL Total cases = 511

Threshold dB HL≥	0	5	10	15	20	25	30	35	40	45	50	55	65	75	85	95
**Prevalence**	**95.7**	**82.7**	**61.2**	**45.8**	**37.1**	**29.9**	**21.9**	**16.7**	**13.1**	**10.6**	**8.0**	**5.9**	**3.0**	**1.9**	**1.0**	**0.6**
Lower CI	91.3	77.4	56.1	41.4	33.0	26.3	19.2	14.2	10.9	8.9	6.6	4.6	2.3	1.4	0.7	0.3
Upper CI	98.0	86.9	66.1	50.3	41.4	33.7	24.8	19.5	15.6	12.5	9.5	7.6	3.9	2.6	1.6	1.1
Number	502	465	385	308	266	221	177	142	116	99	77	61	36	24	14	10

Table W124-38 Age Group = 18-80 Gender = FEMALE Occupational group = MANUAL Total cases = 710

Threshold dB HL≥	0	5	10	15	20	25	30	35	40	45	50	55	65	75	85	95
**Prevalence**	**96.6**	**86.4**	**66.0**	**48.6**	**34.1**	**23.7**	**19.1**	**14.8**	**11.3**	**9.9**	**7.6**	**6.1**	**3.6**	**1.7**	**1.1**	**0.8**
Lower CI	93.5	82.2	61.5	44.6	31.1	20.8	16.5	12.5	9.6	8.3	6.3	4.9	2.8	1.3	0.8	0.5
Upper CI	98.3	89.8	70.2	52.5	37.2	26.8	22.0	17.3	13.3	11.9	9.2	7.7	4.5	2.2	1.5	1.1
Number	700	651	548	443	342	270	228	188	161	139	112	96	65	37	20	13

Table W124-39 Age Group = 18-80 Gender = MALE Occupational group = MANUAL Total cases = 756

Threshold dB HL≥	0	5	10	15	20	25	30	35	40	45	50	55	65	75	85	95
**Prevalence**	**98.7**	**88.5**	**73.0**	**59.9**	**48.2**	**39.0**	**30.7**	**24.2**	**18.6**	**15.0**	**12.5**	**10.1**	**6.1**	**3.9**	**2.4**	**1.5**
Lower CI	96.7	84.0	68.1	55.3	44.1	35.6	27.6	21.6	16.4	13.1	10.6	8.4	4.8	2.8	1.6	0.9
Upper CI	99.5	91.9	77.3	64.4	52.4	42.4	34.0	27.1	21.1	17.2	14.6	12.0	7.8	5.4	3.5	2.4
Number	751	718	652	577	514	453	389	331	276	235	198	168	105	70	44	26

Table W124-40 Age Group = 18-30 Gender = FEMALE Occupational group = NON-MANUAL Total cases = 115

Threshold dB HL≥	0	5	10	15	20	25	30	35	40	45	50	55	65	75	85	95
**Prevalence**	**93.2**	**55.0**	**25.9**	**9.9**	**6.0**	**4.1**	**3.7**	**2.2**	**2.2**	**1.2**	**0.4**	**0.4**	**0.4**	**0.0**	**0.0**	**0.0**
Lower CI	86.7	45.3	18.6	6.1	3.3	2.3	2.0	1.0	1.0	0.3	0.1	0.1	0.1	0.0	0.0	0.0
Upper CI	96.6	64.4	34.9	15.7	10.7	7.4	6.9	5.1	5.1	4.7	2.2	2.2	2.2	3.2	3.2	3.2
Number	107	68	35	17	11	9	8	5	5	2	1	1	1	0	0	0

Table W124-41 Age Group = 31-40 Gender = FEMALE Occupational group = NON-MANUAL Total cases = 86

Threshold dB HL≥	0	5	10	15	20	25	30	35	40	45	50	55	65	75	85	95
**Prevalence**	**93.6**	**71.1**	**38.2**	**18.8**	**13.9**	**9.3**	**8.1**	**7.0**	**3.5**	**1.8**	**1.2**	**1.2**	**0.6**	**0.6**	**0.6**	**0.6**
Lower CI	86.3	59.9	28.3	11.5	7.9	5.0	4.1	3.4	1.3	0.6	0.3	0.3	0.1	0.1	0.1	0.1
Upper CI	97.1	80.2	49.2	29.4	23.3	16.8	15.1	14.2	9.2	5.1	4.5	4.5	3.2	3.2	3.2	3.2
Number	81	63	37	19	15	11	10	8	5	3	2	2	1	1	1	1

Table W124-42 Age Group = 41-50 Gender = FEMALE Occupational group = NON-MANUAL Total cases = 112

Threshold dB HL≥	0	5	10	15	20	25	30	35	40	45	50	55	65	75	85	95
**Prevalence**	**98.9**	**90.3**	**59.0**	**35.8**	**17.7**	**14.2**	**7.7**	**5.9**	**2.9**	**2.0**	**1.6**	**0.0**	**0.0**	**0.0**	**0.0**	**0.0**
Lower CI	93.9	82.9	49.4	27.9	12.7	9.7	4.8	3.6	1.3	0.7	0.5	0.0	0.0	0.0	0.0	0.0
Upper CI	99.8	94.7	68.0	44.5	24.2	20.2	12.0	9.4	6.5	5.7	5.3	3.3	3.3	3.3	3.3	3.3
Number	111	102	70	47	24	20	13	11	6	3	2	0	0	0	0	0

Prevalence by age group, gender and occupational group for the worse ear over 1, 2 and 4 kHz

Table W124-43 Age Group = 51-60 Gender = FEMALE Occupational group = NON-MANUAL Total cases = 174

	Threshold dB HL≥															
	0	5	10	15	20	25	30	35	40	45	50	55	65	75	85	95
Prevalence	**100.0**	**96.2**	**81.2**	**58.1**	**37.4**	**25.7**	**15.9**	**10.2**	**8.1**	**7.0**	**5.5**	**5.0**	**3.7**	**3.2**	**2.1**	**2.1**
Lower CI	97.8	92.2	74.1	49.9	30.4	20.0	11.7	7.3	5.6	4.7	3.5	3.1	1.9	1.6	0.8	0.8
Upper CI	100.0	98.2	86.6	65.9	45.1	32.4	21.1	14.1	11.5	10.1	8.4	8.1	6.8	6.1	5.2	5.2
Number	174	166	141	110	79	60	46	35	28	25	20	18	13	12	7	7

Table W124-44 Age Group = 61-70 Gender = FEMALE Occupational group = NON-MANUAL Total cases = 116

	Threshold dB HL≥															
	0	5	10	15	20	25	30	35	40	45	50	55	65	75	85	95
Prevalence	**100.0**	**97.1**	**92.6**	**81.4**	**61.1**	**46.1**	**39.6**	**26.1**	**23.1**	**14.7**	**11.6**	**8.9**	**5.2**	**3.1**	**2.6**	**2.2**
Lower CI	96.8	89.9	84.2	71.4	50.7	36.4	30.3	19.2	16.9	9.8	7.7	5.8	4.0	1.9	1.4	1.1
Upper CI	100.0	99.2	96.7	88.4	70.5	56.1	49.7	34.4	30.6	21.4	17.3	13.2	6.7	4.9	4.6	4.2
Number	116	114	109	97	76	62	54	42	38	31	26	23	17	11	9	8

Table W124-45 Age Group = 71-80 Gender = FEMALE Occupational group = NON-MANUAL Total cases = 67

	Threshold dB HL≥															
	0	5	10	15	20	25	30	35	40	45	50	55	65	75	85	95
Prevalence	**100.0**	**98.6**	**98.6**	**92.8**	**82.3**	**75.0**	**60.2**	**56.1**	**53.0**	**30.8**	**24.3**	**15.0**	**12.9**	**8.1**	**3.8**	**1.9**
Lower CI	94.6	92.2	92.2	82.1	62.6	57.2	50.0	46.0	43.4	25.9	20.1	10.4	8.3	5.1	1.6	0.5
Upper CI	100.0	99.7	99.7	97.3	92.8	87.0	69.5	65.7	62.4	36.2	29.1	21.1	19.3	12.7	8.7	7.1
Number	67	66	66	63	59	56	50	46	43	37	30	23	19	13	6	2

Table W124-46 Age Group = 18-30 Gender = MALE Occupational group = NON-MANUAL Total cases = 66

	Threshold dB HL≥															
	0	5	10	15	20	25	30	35	40	45	50	55	65	75	85	95
Prevalence	**85.7**	**55.8**	**25.6**	**13.7**	**6.5**	**5.2**	**0.4**	**0.1**	**0.1**	**0.1**	**0.1**	**0.1**	**0.1**	**0.1**	**0.0**	**0.0**
Lower CI	71.1	41.9	15.2	6.7	2.1	1.4	0.1	0.0	0.0	0.0	0.0	0.0	0.0	0.0	0.0	0.0
Upper CI	93.6	68.9	39.9	26.3	18.7	17.8	1.3	5.7	5.7	5.7	5.7	5.7	5.7	5.7	5.5	5.5
Number	59	39	19	8	5	4	2	1	1	1	1	1	1	1	0	0

Table W124-47 Age Group = 31-40 Gender = MALE Occupational group = NON-MANUAL Total cases = 62

	Threshold dB HL≥															
	0	5	10	15	20	25	30	35	40	45	50	55	65	75	85	95
Prevalence	**100.0**	**83.9**	**46.2**	**27.7**	**20.8**	**10.9**	**8.6**	**6.5**	**2.5**	**1.9**	**0.6**	**0.0**	**0.0**	**0.0**	**0.0**	**0.0**
Lower CI	94.2	67.5	31.3	15.6	10.4	4.8	3.4	2.4	0.9	0.6	0.1	0.0	0.0	0.0	0.0	0.0
Upper CI	100.0	92.9	61.8	44.2	37.2	22.8	19.8	16.9	7.1	6.3	7.0	5.8	5.8	5.8	5.8	5.8
Number	62	56	35	20	15	9	7	6	3	2	1	0	0	0	0	0

Table W124-48 Age Group = 41-50 Gender = MALE Occupational group = NON-MANUAL Total cases = 76

	Threshold dB HL≥															
	0	5	10	15	20	25	30	35	40	45	50	55	65	75	85	95
Prevalence	**99.0**	**89.7**	**62.6**	**39.6**	**35.5**	**28.0**	**20.5**	**15.0**	**10.7**	**7.3**	**7.3**	**6.0**	**3.3**	**3.0**	**2.5**	**0.9**
Lower CI	94.4	79.3	50.1	29.2	25.6	19.8	11.8	7.5	4.9	3.4	3.4	2.4	1.9	1.7	1.4	0.2
Upper CI	99.8	95.2	73.6	51.0	46.8	37.9	33.2	28.0	21.7	15.0	15.0	14.3	5.5	5.3	4.4	3.8
Number	75	68	49	34	29	23	17	12	10	8	8	7	6	5	4	2

Table W124-49 Age Group = 51-60 Gender = MALE Occupational group = NON-MANUAL Total cases = 142

	Threshold dB HL≥															
	0	5	10	15	20	25	30	35	40	45	50	55	65	75	85	95
Prevalence	**99.3**	**96.7**	**85.0**	**58.4**	**48.4**	**40.8**	**28.4**	**17.5**	**14.7**	**8.8**	**6.7**	**5.0**	**3.5**	**2.6**	**1.0**	**1.0**
Lower CI	96.3	91.7	77.4	49.6	40.5	33.6	22.9	11.2	8.9	6.0	4.4	3.1	1.7	1.1	0.4	0.4
Upper CI	99.9	98.7	90.3	66.6	56.4	48.5	34.7	26.4	23.5	12.6	10.1	8.1	6.9	5.7	2.6	2.6
Number	141	137	121	90	78	66	48	37	32	25	20	15	10	7	4	4

Prevalence by age group, gender and occupational group for the worse ear over 1, 2 and 4 kHz

Table W124-50 Age Group = 61-70 Gender = MALE Occupational group = NON-MANUAL Total cases = 109

	Threshold dB HL≥															
	0	5	10	15	20	25	30	35	40	45	50	55	65	75	85	95
**Prevalence**	**100.0**	**100.0**	**98.1**	**96.1**	**75.6**	**62.0**	**47.7**	**37.9**	**32.7**	**28.6**	**23.5**	**16.8**	**6.1**	**5.0**	**2.9**	**1.3**
Lower CI	96.6	96.6	93.1	91.2	60.3	47.6	39.7	31.5	26.4	23.1	19.1	11.6	4.7	3.7	1.2	0.5
Upper CI	100.0	100.0	99.5	98.4	86.3	74.6	55.9	44.7	39.7	34.7	28.6	23.5	8.0	6.7	7.3	3.5
Number	109	109	106	102	87	72	61	50	43	37	29	24	11	8	5	3

Table W124-51 Age Group = 71-80 Gender = MALE Occupational group = NON-MANUAL Total cases = 56

	Threshold dB HL≥															
	0	5	10	15	20	25	30	35	40	45	50	55	65	75	85	95
**Prevalence**	**100.0**	**100.0**	**97.9**	**95.8**	**91.7**	**81.4**	**68.2**	**58.3**	**44.8**	**41.7**	**27.4**	**22.6**	**13.5**	**3.1**	**1.0**	**1.0**
Lower CI	93.6	93.6	89.0	87.2	85.0	69.3	57.4	48.1	33.6	30.2	19.1	15.3	8.0	1.2	0.2	0.2
Upper CI	100.0	100.0	99.6	98.7	95.5	89.5	77.4	67.8	56.6	54.2	37.6	32.1	22.1	8.1	5.3	5.3
Number	56	56	55	54	52	47	42	36	27	26	18	14	8	3	1	1

Table W124-52 Age Group = 18-30 Gender = FEMALE Occupational group = MANUAL Total cases = 109

	Threshold dB HL≥															
	0	5	10	15	20	25	30	35	40	45	50	55	65	75	85	95
**Prevalence**	**92.2**	**72.6**	**41.4**	**22.6**	**8.6**	**5.6**	**5.1**	**4.0**	**2.6**	**2.1**	**0.5**	**0.2**	**0.0**	**0.0**	**0.0**	**0.0**
Lower CI	81.5	60.0	30.1	14.1	5.0	2.8	2.4	1.8	1.0	0.7	0.1	0.0	0.0	0.0	0.0	0.0
Upper CI	96.9	82.5	53.8	34.2	14.4	10.9	10.3	8.7	6.5	5.9	1.7	1.1	3.4	3.4	3.4	3.4
Number	104	82	53	32	18	11	10	9	7	5	2	1	0	0	0	0

Table W124-53 Age Group = 31-40 Gender = FEMALE Occupational group = MANUAL Total cases = 103

	Threshold dB HL≥															
	0	5	10	15	20	25	30	35	40	45	50	55	65	75	85	95
**Prevalence**	**94.2**	**73.8**	**43.0**	**23.6**	**14.4**	**8.4**	**7.1**	**6.1**	**4.8**	**4.8**	**3.2**	**2.6**	**1.4**	**0.5**	**0.2**	**0.0**
Lower CI	83.1	61.0	31.8	16.7	9.5	5.0	4.0	3.1	2.2	2.2	1.6	1.2	0.5	0.1	0.0	0.0
Upper CI	98.1	83.6	55.0	32.2	21.1	13.7	12.5	11.6	10.4	10.4	6.5	5.4	3.8	1.6	3.9	3.6
Number	100	84	61	42	29	19	16	12	9	9	8	7	4	2	1	0

Table W124-54 Age Group = 41-50 Gender = FEMALE Occupational group = MANUAL Total cases = 135

	Threshold dB HL≥															
	0	5	10	15	20	25	30	35	40	45	50	55	65	75	85	95
**Prevalence**	**97.9**	**90.3**	**64.3**	**45.3**	**27.8**	**16.6**	**15.9**	**7.5**	**5.5**	**4.9**	**4.5**	**4.1**	**2.6**	**1.9**	**1.2**	**0.6**
Lower CI	92.7	82.6	54.3	35.9	20.1	11.1	10.5	4.9	3.6	3.1	2.7	2.4	1.5	1.0	0.5	0.2
Upper CI	99.4	94.8	73.1	54.9	36.9	24.1	23.4	11.3	8.4	7.7	7.3	6.7	4.3	3.8	2.8	1.7
Number	133	126	94	69	48	34	32	22	18	16	14	13	9	8	5	3

Table W124-55 Age Group = 51-60 Gender = FEMALE Occupational group = MANUAL Total cases = 165

	Threshold dB HL≥															
	0	5	10	15	20	25	30	35	40	45	50	55	65	75	85	95
**Prevalence**	**100.0**	**97.6**	**87.2**	**67.7**	**42.2**	**27.4**	**18.6**	**13.4**	**9.3**	**6.2**	**4.2**	**3.1**	**2.9**	**1.2**	**0.4**	**0.4**
Lower CI	97.7	92.2	79.4	58.4	34.0	20.7	13.4	9.3	6.0	3.9	2.5	1.7	1.6	0.7	0.1	0.1
Upper CI	100.0	99.3	92.3	75.7	51.0	35.2	25.2	19.1	14.1	9.9	6.9	5.4	5.3	2.1	1.3	1.3
Number	165	162	150	126	91	66	51	40	31	23	18	14	13	8	2	2

Table W124-56 Age Group = 61-70 Gender = FEMALE Occupational group = MANUAL Total cases = 124

	Threshold dB HL≥															
	0	5	10	15	20	25	30	35	40	45	50	55	65	75	85	95
**Prevalence**	**100.0**	**99.5**	**93.1**	**76.2**	**61.7**	**47.0**	**30.7**	**21.3**	**20.4**	**17.8**	**12.6**	**10.5**	**8.2**	**3.2**	**2.5**	**1.9**
Lower CI	97.0	97.4	85.0	65.7	51.2	37.4	23.8	15.3	14.4	11.9	8.3	6.6	4.7	2.4	1.7	1.4
Upper CI	100.0	99.9	97.0	84.2	71.2	56.8	38.7	28.8	28.0	25.7	18.7	16.5	13.9	4.4	3.7	2.4
Number	124	123	117	102	85	75	56	46	44	37	29	25	18	7	4	2

Prevalence by age group, gender and occupational group for the worse ear over 1, 2 and 4 kHz

Table W124-57 Age Group = 71-80 Gender = FEMALE Occupational group = MANUAL Total cases = 74

	Threshold dB HL≥															
	0	5	10	15	20	25	30	35	40	45	50	55	65	75	85	95
**Prevalence**	**100.0**	**100.0**	**95.7**	**91.4**	**91.0**	**66.9**	**65.6**	**63.0**	**45.3**	**43.0**	**38.3**	**29.7**	**12.2**	**6.1**	**4.2**	**2.9**
**Lower CI**	95.1	95.1	78.7	79.2	78.9	48.7	47.5	45.1	33.4	31.0	26.6	18.6	8.4	3.5	2.2	1.5
**Upper CI**	100.0	100.0	99.3	96.7	96.4	81.1	80.0	77.9	57.7	55.8	51.4	43.8	17.4	10.2	7.9	5.5
**Number**	74	74	73	72	71	65	63	59	52	49	41	36	21	12	8	6

Table W124-58 Age Group = 18-30 Gender = MALE Occupational group = MANUAL Total cases = 109

	Threshold dB HL≥															
	0	5	10	15	20	25	30	35	40	45	50	55	65	75	85	95
**Prevalence**	**96.1**	**71.3**	**42.3**	**26.7**	**16.6**	**8.3**	**6.1**	**2.6**	**1.3**	**0.8**	**0.3**	**0.3**	**0.1**	**0.1**	**0.1**	**0.1**
**Lower CI**	89.4	58.3	30.7	16.9	9.6	4.8	3.3	1.1	0.6	0.3	0.1	0.1	0.0	0.0	0.0	0.0
**Upper CI**	98.6	81.6	54.8	39.4	27.3	14.2	10.9	5.7	2.7	2.0	1.5	1.5	0.4	0.4	0.4	0.4
**Number**	105	84	58	35	28	19	16	9	6	4	2	2	1	1	1	1

Table W124-59 Age Group = 31-40 Gender = MALE Occupational group = MANUAL Total cases = 80

	Threshold dB HL≥															
	0	5	10	15	20	25	30	35	40	45	50	55	65	75	85	95
**Prevalence**	**99.3**	**84.9**	**57.7**	**45.3**	**30.5**	**18.0**	**13.4**	**10.4**	**8.3**	**3.7**	**3.1**	**3.1**	**1.9**	**1.9**	**1.9**	**1.9**
**Lower CI**	96.0	70.6	42.7	31.8	19.2	9.7	6.5	5.0	3.4	1.3	0.9	0.9	0.3	0.3	0.3	0.3
**Upper CI**	99.9	92.9	71.5	59.7	44.8	31.0	25.8	20.5	18.8	10.0	9.8	9.8	10.2	10.2	10.2	10.2
**Number**	79	71	50	43	31	20	17	16	11	7	5	5	1	1	1	1

Table W124-60 Age Group = 41-50 Gender = MALE Occupational group = MANUAL Total cases = 127

	Threshold dB HL≥															
	0	5	10	15	20	25	30	35	40	45	50	55	65	75	85	95
**Prevalence**	**100.0**	**96.4**	**83.3**	**64.7**	**47.7**	**35.7**	**26.7**	**20.9**	**13.4**	**11.5**	**9.6**	**8.0**	**5.4**	**2.1**	**2.1**	**0.2**
**Lower CI**	97.1	88.5	73.4	53.7	37.2	27.0	19.1	14.2	8.9	7.2	5.7	4.4	2.4	0.6	0.6	0.0
**Upper CI**	100.0	99.0	90.0	74.3	58.3	45.5	35.9	29.6	19.6	17.9	15.8	14.2	11.5	7.5	7.5	1.1
**Number**	127	124	111	90	74	61	46	37	27	23	19	15	9	4	4	1

Table W124-61 Age Group = 51-60 Gender = MALE Occupational group = MANUAL Total cases = 196

	Threshold dB HL≥															
	0	5	10	15	20	25	30	35	40	45	50	55	65	75	85	95
**Prevalence**	**100.0**	**100.0**	**95.2**	**79.2**	**66.8**	**58.1**	**46.0**	**32.1**	**23.4**	**21.6**	**19.4**	**15.0**	**8.8**	**4.6**	**3.1**	**2.0**
**Lower CI**	98.1	98.1	89.8	71.0	58.2	49.7	38.7	26.3	18.5	16.7	14.7	11.0	5.7	2.7	1.6	0.9
**Upper CI**	100.0	100.0	97.8	85.6	74.3	66.1	53.5	38.6	29.3	27.4	25.2	20.1	13.5	7.8	5.9	4.6
**Number**	196	196	190	172	154	137	116	89	71	65	57	46	27	17	11	7

Table W124-62 Age Group = 61-70 Gender = MALE Occupational group = MANUAL Total cases = 172

	Threshold dB HL≥															
	0	5	10	15	20	25	30	35	40	45	50	55	65	75	85	95
**Prevalence**	**100.0**	**98.8**	**98.8**	**94.1**	**82.2**	**75.4**	**62.3**	**51.9**	**42.0**	**36.1**	**29.4**	**21.6**	**12.1**	**9.5**	**3.1**	**1.9**
**Lower CI**	97.8	93.3	93.3	87.4	71.5	64.3	51.6	42.0	34.4	28.6	22.6	15.8	7.6	5.3	1.8	0.9
**Upper CI**	100.0	99.8	99.8	97.3	89.5	83.9	71.9	61.7	49.9	44.2	37.4	28.8	18.8	16.5	5.3	3.9
**Number**	172	171	171	165	156	148	128	115	103	87	73	61	38	27	13	8

Table W124-63 Age Group = 71-80 Gender = MALE Occupational group = MANUAL Total cases = 72

	Threshold dB HL≥															
	0	5	10	15	20	25	30	35	40	45	50	55	65	75	85	95
**Prevalence**	**100.0**	**100.0**	**100.0**	**100.0**	**99.6**	**91.7**	**75.4**	**72.9**	**64.3**	**48.4**	**39.7**	**36.0**	**22.3**	**14.4**	**10.2**	**6.3**
**Lower CI**	94.9	94.9	94.9	94.9	97.8	73.1	59.4	56.3	48.4	34.9	26.7	23.4	15.1	9.6	6.2	3.6
**Upper CI**	100.0	100.0	100.0	100.0	99.9	97.8	86.5	84.8	77.6	62.1	54.2	50.9	31.8	21.2	16.2	11.0
**Number**	72	72	72	72	71	68	66	65	58	49	42	39	29	20	14	8

Prevalence by age group, gender and occupational group for the worse ear over 4, 6 and 8 kHz

Table W468-1 Age Group = 18-80 Gender = OVERALL Occupational group = OVERALL Total cases = 2615

	Threshold dB HL≥															
	**0**	**5**	**10**	**15**	**20**	**25**	**30**	**35**	**40**	**45**	**50**	**55**	**65**	**75**	**85**	**95**
**Prevalence**	**98.9**	**94.5**	**83.7**	**70.2**	**58.7**	**50.3**	**43.1**	**36.6**	**32.0**	**28.2**	**23.7**	**20.0**	**13.8**	**8.2**	**4.8**	**2.5**
**Lower CI**	98.1	93.1	81.8	68.3	56.7	48.5	41.4	35.0	30.5	26.7	22.4	18.7	12.7	7.4	4.1	2.1
**Upper CI**	99.3	95.7	85.5	72.2	60.6	52.2	44.9	38.2	33.6	29.7	25.1	21.5	15.1	9.2	5.5	3.0
**Number**	2597	2537	2365	2133	1885	1694	1498	1336	1199	1082	950	834	630	411	255	150

Table W468-2 Age Group = 18-80 Gender = FEMALE Occupational group = OVERALL Total cases = 1366

	Threshold dB HL≥															
	**0**	**5**	**10**	**15**	**20**	**25**	**30**	**35**	**40**	**45**	**50**	**55**	**65**	**75**	**85**	**95**
**Prevalence**	**98.9**	**93.9**	**82.2**	**67.0**	**54.1**	**46.5**	**37.8**	**31.8**	**26.5**	**22.9**	**18.3**	**14.9**	**9.7**	**5.2**	**3.0**	**1.5**
**Lower CI**	97.8	92.0	79.7	64.4	51.5	44.0	35.7	29.7	24.5	21.1	16.7	13.2	8.3	4.3	2.4	1.1
**Upper CI**	99.4	95.4	84.5	69.5	56.6	48.9	40.0	33.9	28.6	24.8	20.1	16.6	11.4	6.2	3.8	1.9
**Number**	1355	1316	1202	1051	895	789	666	574	492	435	366	310	224	141	91	51

Table W468-3 Age Group = 18-80 Gender = MALE Occupational group = OVERALL Total cases = 1249

	Threshold dB HL≥															
	**0**	**5**	**10**	**15**	**20**	**25**	**30**	**35**	**40**	**45**	**50**	**55**	**65**	**75**	**85**	**95**
**Prevalence**	**98.9**	**95.2**	**85.4**	**73.8**	**63.9**	**54.7**	**49.1**	**42.0**	**38.2**	**34.1**	**29.8**	**25.8**	**18.4**	**11.6**	**6.7**	**3.6**
**Lower CI**	97.4	92.7	82.3	70.7	60.8	51.8	46.3	39.5	35.8	31.8	27.6	23.7	16.6	10.2	5.6	2.9
**Upper CI**	99.5	96.8	88.1	76.7	66.8	57.6	52.0	44.5	40.6	36.5	32.0	28.1	20.4	13.2	8.1	4.6
**Number**	1242	1221	1163	1082	990	905	832	762	707	647	584	524	406	270	164	99

Table W468-4 Age Group = 18-30 Gender = OVERALL Occupational group = OVERALL Total cases = 399

	Threshold dB HL≥															
	**0**	**5**	**10**	**15**	**20**	**25**	**30**	**35**	**40**	**45**	**50**	**55**	**65**	**75**	**85**	**95**
**Prevalence**	**96.1**	**84.3**	**59.0**	**33.4**	**22.0**	**15.9**	**11.6**	**7.5**	**5.8**	**4.4**	**3.4**	**2.5**	**0.9**	**0.7**	**0.7**	**0.0**
**Lower CI**	93.2	79.5	53.1	28.4	17.8	12.3	8.4	5.1	3.7	2.7	1.9	1.3	0.3	0.2	0.2	0.0
**Upper CI**	97.8	88.2	64.7	38.7	26.8	20.4	15.7	10.7	8.9	7.3	6.0	5.0	2.5	2.4	2.4	0.1
**Number**	382	344	255	157	110	83	58	42	31	23	18	13	6	4	3	1

Table W468-5 Age Group = 31-40 Gender = OVERALL Occupational group = OVERALL Total cases = 329

	Threshold dB HL≥															
	**0**	**5**	**10**	**15**	**20**	**25**	**30**	**35**	**40**	**45**	**50**	**55**	**65**	**75**	**85**	**95**
**Prevalence**	**99.6**	**93.9**	**80.6**	**60.0**	**39.7**	**28.9**	**21.0**	**14.1**	**11.6**	**9.6**	**6.0**	**4.9**	**3.3**	**1.4**	**1.0**	**0.8**
**Lower CI**	97.8	89.9	75.1	53.4	33.5	23.4	16.2	10.4	8.2	6.5	3.7	2.8	1.6	0.6	0.3	0.2
**Upper CI**	99.9	96.4	85.1	66.3	46.3	35.1	26.7	18.8	16.1	13.9	9.6	8.5	6.6	3.4	3.0	3.0
**Number**	328	312	272	220	155	120	87	70	57	47	32	24	16	9	6	4

Table W468-6 Age Group = 41-50 Gender = OVERALL Occupational group = OVERALL Total cases = 443

	Threshold dB HL≥															
	**0**	**5**	**10**	**15**	**20**	**25**	**30**	**35**	**40**	**45**	**50**	**55**	**65**	**75**	**85**	**95**
**Prevalence**	**100.0**	**98.8**	**90.7**	**79.5**	**63.8**	**46.7**	**35.8**	**27.3**	**20.7**	**16.6**	**13.7**	**10.6**	**7.1**	**3.5**	**1.7**	**1.0**
**Lower CI**	99.1	97.1	87.3	75.2	58.8	41.8	31.3	23.3	17.3	13.5	10.9	8.1	5.2	2.3	0.9	0.5
**Upper CI**	100.0	99.5	93.3	83.3	68.5	51.7	40.5	31.7	24.7	20.3	17.0	13.6	9.7	5.3	3.0	2.0
**Number**	443	438	406	364	298	236	190	154	124	100	84	68	49	27	16	10

Table W468-7 Age Group = 51-60 Gender = OVERALL Occupational group = OVERALL Total cases = 662

	Threshold dB HL≥															
	**0**	**5**	**10**	**15**	**20**	**25**	**30**	**35**	**40**	**45**	**50**	**55**	**65**	**75**	**85**	**95**
**Prevalence**	**100.0**	**99.9**	**97.9**	**90.8**	**80.6**	**71.4**	**59.0**	**48.5**	**41.2**	**34.6**	**26.8**	**22.2**	**14.0**	**9.0**	**4.9**	**3.3**
**Lower CI**	99.4	99.2	96.2	88.0	76.9	67.3	54.8	44.4	37.4	31.1	23.7	19.4	11.9	7.2	3.6	2.3
**Upper CI**	100.0	100.0	98.8	93.0	83.8	75.1	63.0	52.6	45.2	38.4	30.1	25.3	16.6	11.1	6.5	4.7
**Number**	662	661	651	614	563	512	442	380	333	291	245	207	148	94	54	36

Prevalence by age group, gender and occupational group for the worse ear over 4, 6 and 8 kHz

Table W468-8 Age Group = 61-70 Gender = OVERALL Occupational group = OVERALL Total cases = 518

Threshold dB HL≥	0	5	10	15	20	25	30	35	40	45	50	55	65	75	85	95
**Prevalence**	**100.0**	**100.0**	**99.8**	**99.1**	**94.7**	**90.4**	**85.3**	**79.3**	**71.9**	**64.4**	**55.0**	**46.2**	**31.3**	**17.8**	**9.9**	**5.3**
**Lower CI**	99.3	99.3	99.0	97.3	91.7	86.6	81.0	74.6	67.0	59.4	49.9	41.2	27.2	14.7	8.1	4.1
**Upper CI**	100.0	100.0	100.0	99.7	96.7	93.1	88.8	83.3	76.3	69.1	60.0	51.3	35.7	21.3	12.2	6.9
**Number**	518	518	517	514	499	485	467	440	407	379	339	301	229	147	95	54

Table W468-9 Age Group = 71-80 Gender = OVERALL Occupational group = OVERALL Total cases = 264

Threshold dB HL≥	0	5	10	15	20	25	30	35	40	45	50	55	65	75	85	95
**Prevalence**	**100.0**	**100.0**	**100.0**	**100.0**	**97.0**	**95.2**	**93.8**	**90.8**	**87.5**	**84.7**	**79.6**	**71.3**	**55.3**	**35.2**	**20.8**	**9.3**
**Lower CI**	98.6	98.6	98.6	98.6	92.9	89.9	88.6	84.2	80.7	77.5	72.3	62.6	46.8	29.3	15.9	6.6
**Upper CI**	100.0	100.0	100.0	100.0	98.7	97.8	96.7	94.9	92.1	89.9	85.4	78.6	63.4	41.6	26.6	13.1
**Number**	264	264	264	264	260	258	254	250	247	242	232	221	182	130	81	45

Table W468-10 Age Group = 18-80 Gender = OVERALL Occupational group = NON-MANUAL Total cases = 1166

Threshold dB HL≥	0	5	10	15	20	25	30	35	40	45	50	55	65	75	85	95
**Prevalence**	**98.8**	**93.6**	**81.0**	**65.3**	**54.1**	**46.0**	**39.1**	**34.1**	**29.8**	**26.8**	**22.8**	**19.6**	**12.1**	**6.2**	**3.6**	**1.8**
**Lower CI**	97.4	91.5	78.1	62.4	51.2	43.4	36.5	31.6	27.3	24.3	20.4	17.3	10.4	5.2	2.9	1.3
**Upper CI**	99.5	95.2	83.5	68.1	56.9	48.7	41.8	36.7	32.5	29.4	25.3	22.0	13.9	7.5	4.4	2.4
**Number**	1159	1120	1025	891	771	686	597	528	468	426	366	319	218	136	83	49

Table W468-11 Age Group = 18-80 Gender = OVERALL Occupational group = MANUAL Total cases = 1445

Threshold dB HL≥	0	5	10	15	20	25	30	35	40	45	50	55	65	75	85	95
**Prevalence**	**99.1**	**95.9**	**87.0**	**75.1**	**62.7**	**54.2**	**46.7**	**38.6**	**33.7**	**29.4**	**24.7**	**20.5**	**14.6**	**9.2**	**5.5**	**3.0**
**Lower CI**	98.2	93.8	84.1	72.1	59.7	51.3	44.0	36.3	31.6	27.4	22.9	18.9	13.2	8.0	4.6	2.4
**Upper CI**	99.5	97.2	89.5	77.9	65.6	57.1	49.4	40.9	35.9	31.4	26.4	22.3	16.2	10.5	6.7	3.7
**Number**	1434	1413	1338	1240	1112	1006	899	806	729	655	583	514	411	274	172	101

Table W468-12 Age Group = 18-30 Gender = FEMALE Occupational group = OVERALL Total cases = 223

Threshold dB HL≥	0	5	10	15	20	25	30	35	40	45	50	55	65	75	85	95
**Prevalence**	**96.1**	**83.0**	**59.9**	**31.3**	**19.5**	**14.6**	**9.3**	**5.6**	**4.7**	**2.9**	**2.0**	**1.2**	**0.1**	**0.0**	**0.0**	**0.0**
**Lower CI**	92.2	76.5	52.4	25.1	14.6	10.4	6.1	3.3	2.5	1.4	0.9	0.4	0.0	0.0	0.0	0.0
**Upper CI**	98.1	88.0	66.9	38.2	25.5	20.2	13.8	9.5	8.4	5.7	4.5	3.1	0.6	1.7	1.7	1.7
**Number**	213	190	141	81	58	44	30	19	15	10	7	5	1	0	0	0

Table W468-13 Age Group = 31-40 Gender = FEMALE Occupational group = OVERALL Total cases = 187

Threshold dB HL≥	0	5	10	15	20	25	30	35	40	45	50	55	65	75	85	95
**Prevalence**	**99.3**	**92.5**	**74.3**	**54.3**	**32.9**	**25.1**	**15.2**	**12.1**	**9.7**	**7.4**	**4.0**	**2.8**	**1.5**	**0.2**	**0.2**	**0.0**
**Lower CI**	96.1	87.1	66.3	46.1	25.7	18.8	10.5	8.0	6.1	4.3	2.2	1.4	0.6	0.0	0.0	0.0
**Upper CI**	99.9	95.8	81.0	62.4	41.0	32.6	21.4	17.9	14.9	12.3	7.2	5.5	4.0	1.2	1.2	2.0
**Number**	186	176	148	119	78	61	40	33	28	21	15	11	6	1	1	0

Table W468-14 Age Group = 41-50 Gender = FEMALE Occupational group = OVERALL Total cases = 245

Threshold dB HL≥	0	5	10	15	20	25	30	35	40	45	50	55	65	75	85	95
**Prevalence**	**100.0**	**97.7**	**85.6**	**70.0**	**52.0**	**37.0**	**25.4**	**17.3**	**11.7**	**9.0**	**5.9**	**4.3**	**2.6**	**1.2**	**0.6**	**0.3**
**Lower CI**	98.5	94.4	80.0	63.4	45.1	30.9	20.4	13.3	8.7	6.3	4.1	2.8	1.5	0.6	0.2	0.1
**Upper CI**	100.0	99.0	89.9	76.0	58.7	43.6	31.2	22.2	15.7	12.6	8.4	6.7	4.6	2.4	1.6	1.1
**Number**	245	240	214	183	139	108	80	60	45	35	27	20	12	7	4	2

Prevalence by age group, gender and occupational group for the worse ear over 4, 6 and 8 kHz

Table W468-15 Age Group = 51-60 Gender = FEMALE Occupational group = OVERALL Total cases = 334

Threshold dB HL≥	0	5	10	15	20	25	30	35	40	45	50	55	65	75	85	95
**Prevalence**	**100.0**	**99.7**	**96.0**	**85.7**	**74.0**	**64.5**	**48.6**	**37.1**	**29.0**	**23.1**	**14.5**	**9.6**	**6.1**	**3.6**	**2.7**	**1.9**
**Lower CI**	98.9	98.5	92.9	80.8	68.3	58.6	42.8	31.6	24.0	18.7	11.3	7.2	4.5	2.5	1.8	1.1
**Upper CI**	100.0	100.0	97.8	89.5	79.0	70.0	54.5	42.8	34.4	28.1	18.4	12.7	8.3	5.3	4.2	3.2
**Number**	334	333	323	295	264	233	189	153	124	105	80	59	44	29	22	14

Table W468-16 Age Group = 61-70 Gender = FEMALE Occupational group = OVERALL Total cases = 239

Threshold dB HL≥	0	5	10	15	20	25	30	35	40	45	50	55	65	75	85	95
**Prevalence**	**100.0**	**100.0**	**99.7**	**98.3**	**90.7**	**83.8**	**76.4**	**70.2**	**58.6**	**51.1**	**42.1**	**33.7**	**19.4**	**11.7**	**5.0**	**2.7**
**Lower CI**	98.4	98.4	98.2	95.1	85.1	77.2	69.5	63.0	51.3	44.1	35.5	27.3	14.6	8.1	3.5	1.7
**Upper CI**	100.0	100.0	99.9	99.4	94.3	88.7	82.2	76.5	65.6	58.0	49.0	40.7	25.4	16.8	7.0	4.2
**Number**	239	239	238	235	222	210	196	180	154	139	118	102	71	45	26	15

Table W468-17 Age Group = 71-80 Gender = FEMALE Occupational group = OVERALL Total cases = 138

Threshold dB HL≥	0	5	10	15	20	25	30	35	40	45	50	55	65	75	85	95
**Prevalence**	**100.0**	**100.0**	**100.0**	**100.0**	**94.8**	**92.8**	**92.3**	**88.5**	**83.0**	**81.1**	**75.4**	**67.4**	**50.0**	**26.4**	**16.3**	**6.5**
**Lower CI**	97.3	97.3	97.3	97.3	88.0	84.4	83.9	77.9	72.5	70.1	64.5	55.0	38.4	19.8	11.1	4.1
**Upper CI**	100.0	100.0	100.0	100.0	97.8	96.9	96.5	94.4	90.1	88.7	83.8	77.8	61.6	34.2	23.3	10.0
**Number**	138	138	138	138	134	133	131	129	126	125	119	113	90	59	38	20

Table W468-18 Age Group = 18-30 Gender = MALE Occupational group = OVERALL Total cases = 176

Threshold dB HL≥	0	5	10	15	20	25	30	35	40	45	50	55	65	75	85	95
**Prevalence**	**96.0**	**85.7**	**58.1**	**35.3**	**24.3**	**17.1**	**13.8**	**9.2**	**6.8**	**5.9**	**4.7**	**3.8**	**1.6**	**1.4**	**1.4**	**0.0**
**Lower CI**	90.8	77.8	48.9	27.8	17.9	11.5	8.7	5.6	3.7	3.0	2.2	1.6	0.5	0.4	0.4	0.0
**Upper CI**	98.3	91.1	66.8	43.6	32.2	24.6	21.2	14.9	12.2	11.3	9.7	8.8	4.8	4.7	4.7	0.2
**Number**	169	154	114	76	52	39	28	23	16	13	11	8	5	4	3	1

Table W468-19 Age Group = 31-40 Gender = MALE Occupational group = OVERALL Total cases = 142

Threshold dB HL≥	0	5	10	15	20	25	30	35	40	45	50	55	65	75	85	95
**Prevalence**	**100.0**	**95.4**	**87.4**	**66.0**	**47.3**	**33.3**	**27.4**	**16.5**	**13.8**	**12.1**	**8.1**	**7.1**	**5.2**	**2.8**	**1.9**	**1.7**
**Lower CI**	97.4	88.1	79.0	55.2	37.1	24.5	19.2	10.6	8.4	7.1	4.2	3.4	2.1	1.1	0.6	0.5
**Upper CI**	100.0	98.3	92.8	75.3	57.8	43.5	37.5	24.6	22.0	20.0	15.3	14.5	12.1	7.0	6.3	6.3
**Number**	142	136	124	101	77	59	47	37	29	26	17	13	10	8	5	4

Table W468-20 Age Group = 41-50 Gender = MALE Occupational group = OVERALL Total cases = 198

Threshold dB HL≥	0	5	10	15	20	25	30	35	40	45	50	55	65	75	85	95
**Prevalence**	**100.0**	**100.0**	**96.3**	**90.0**	**76.8**	**57.5**	**47.1**	**38.4**	**30.7**	**25.0**	**22.2**	**17.4**	**12.1**	**6.0**	**2.9**	**1.8**
**Lower CI**	98.1	98.1	91.8	83.9	69.4	49.7	39.7	31.4	24.3	19.3	16.7	12.7	8.4	3.7	1.5	0.8
**Upper CI**	100.0	100.0	98.4	93.9	82.9	65.0	54.7	45.9	37.8	31.8	28.8	23.5	17.2	9.8	5.5	3.8
**Number**	198	198	192	181	159	128	110	94	79	65	57	48	37	20	12	8

Table W468-21 Age Group = 51-60 Gender = MALE Occupational group = OVERALL Total cases = 328

Threshold dB HL≥	0	5	10	15	20	25	30	35	40	45	50	55	65	75	85	95
**Prevalence**	**100.0**	**100.0**	**100.0**	**96.6**	**88.0**	**79.1**	**70.5**	**61.2**	**54.9**	**47.7**	**40.6**	**36.3**	**22.8**	**14.8**	**7.3**	**4.8**
**Lower CI**	98.8	98.8	98.8	93.4	83.2	73.5	64.6	55.1	49.0	41.9	35.3	31.1	18.7	11.4	5.0	3.0
**Upper CI**	100.0	100.0	100.0	98.3	91.6	83.8	75.9	66.9	60.8	53.5	46.2	41.8	27.6	19.1	10.5	7.6
**Number**	328	328	328	319	299	279	253	227	209	186	165	148	104	65	32	22

Prevalence by age group, gender and occupational group for the worse ear over 4, 6 and 8 kHz

Table W468-22 Age Group = 61-70 Gender = MALE Occupational group = OVERALL Total cases = 279

	Threshold dB HL≥															
	**0**	**5**	**10**	**15**	**20**	**25**	**30**	**35**	**40**	**45**	**50**	**55**	**65**	**75**	**85**	**95**
**Prevalence**	**100.0**	**100.0**	**100.0**	**100.0**	**99.4**	**98.1**	**95.6**	**89.9**	**87.2**	**79.9**	**70.0**	**60.8**	**45.3**	**24.8**	**15.7**	**8.4**
**Lower CI**	98.6	98.6	98.6	98.6	97.5	94.8	89.9	83.5	80.7	72.6	62.0	53.0	38.7	20.3	12.1	6.1
**Upper CI**	100.0	100.0	100.0	100.0	99.8	99.3	98.2	94.0	91.8	85.6	76.9	68.0	52.0	30.0	20.1	11.5
**Number**	279	279	279	279	277	275	271	260	253	240	221	199	158	102	69	39

Table W468-23 Age Group = 71-80 Gender = MALE Occupational group = OVERALL Total cases = 126

	Threshold dB HL≥															
	**0**	**5**	**10**	**15**	**20**	**25**	**30**	**35**	**40**	**45**	**50**	**55**	**65**	**75**	**85**	**95**
**Prevalence**	**100.0**	**100.0**	**100.0**	**100.0**	**100.0**	**98.7**	**96.0**	**94.2**	**94.2**	**90.2**	**86.0**	**77.2**	**62.9**	**48.0**	**27.1**	**13.6**
**Lower CI**	97.0	97.0	97.0	97.0	97.0	92.7	90.0	87.7	87.7	82.2	77.6	65.9	51.7	37.7	19.0	8.3
**Upper CI**	100.0	100.0	100.0	100.0	100.0	99.8	98.5	97.4	97.4	94.8	91.6	85.5	72.8	58.4	37.2	21.6
**Number**	126	126	126	126	126	125	123	121	121	117	113	108	92	71	43	25

Table W468-24 Age Group = 18-30 Gender = OVERALL Occupational group = NON-MANUAL Total cases = 180

	Threshold dB HL≥															
	**0**	**5**	**10**	**15**	**20**	**25**	**30**	**35**	**40**	**45**	**50**	**55**	**65**	**75**	**85**	**95**
**Prevalence**	**95.7**	**82.1**	**54.1**	**25.1**	**17.3**	**11.2**	**8.0**	**6.1**	**5.7**	**5.0**	**4.6**	**4.2**	**0.8**	**0.8**	**0.7**	**0.0**
**Lower CI**	90.3	74.9	45.7	18.6	11.9	6.8	4.2	2.8	2.5	2.0	1.8	1.5	0.2	0.2	0.1	0.0
**Upper CI**	98.1	87.5	62.3	32.9	24.6	18.0	14.6	12.7	12.4	11.7	11.5	11.2	3.9	3.9	4.0	2.1
**Number**	173	150	104	49	35	23	16	11	9	7	5	4	2	2	1	0

Table W468-25 Age Group = 31-40 Gender = OVERALL Occupational group = NON-MANUAL Total cases = 147

	Threshold dB HL≥															
	**0**	**5**	**10**	**15**	**20**	**25**	**30**	**35**	**40**	**45**	**50**	**55**	**65**	**75**	**85**	**95**
**Prevalence**	**100.0**	**91.2**	**75.8**	**51.8**	**30.2**	**20.7**	**16.4**	**13.9**	**12.4**	**9.4**	**5.6**	**5.3**	**4.3**	**0.6**	**0.3**	**0.0**
**Lower CI**	97.5	86.1	68.7	42.6	21.9	13.9	10.2	8.2	7.0	4.8	2.2	2.0	1.4	0.3	0.0	0.0
**Upper CI**	100.0	94.5	81.7	60.9	40.1	29.7	25.2	22.6	21.2	17.5	13.4	13.2	12.1	4.6	3.1	2.5
**Number**	147	133	112	83	50	37	28	24	19	15	8	7	6	2	1	0

Table W468-26 Age Group = 41-50 Gender = OVERALL Occupational group = NON-MANUAL Total cases = 187

	Threshold dB HL≥															
	**0**	**5**	**10**	**15**	**20**	**25**	**30**	**35**	**40**	**45**	**50**	**55**	**65**	**75**	**85**	**95**
**Prevalence**	**100.0**	**99.3**	**88.6**	**76.7**	**59.5**	**42.4**	**30.7**	**24.5**	**19.6**	**15.9**	**15.2**	**10.8**	**3.9**	**2.4**	**1.2**	**0.9**
**Lower CI**	98.0	96.9	83.1	69.6	51.9	35.4	24.1	18.7	14.1	10.9	10.2	6.8	2.5	1.2	0.8	0.7
**Upper CI**	100.0	99.8	92.5	82.6	66.7	49.7	38.1	31.4	26.7	22.7	21.9	16.7	6.0	4.9	1.9	1.2
**Number**	187	185	166	145	113	84	62	52	40	32	30	22	10	8	5	3

Table W468-27 Age Group = 51-60 Gender = OVERALL Occupational group = NON-MANUAL Total cases = 305

	Threshold dB HL≥															
	**0**	**5**	**10**	**15**	**20**	**25**	**30**	**35**	**40**	**45**	**50**	**55**	**65**	**75**	**85**	**95**
**Prevalence**	**100.0**	**100.0**	**97.0**	**87.7**	**75.3**	**67.2**	**52.0**	**40.6**	**33.0**	**29.6**	**23.1**	**19.4**	**11.3**	**6.9**	**2.2**	**1.7**
**Lower CI**	98.8	98.8	93.9	83.1	69.6	61.3	46.1	35.1	28.2	25.0	19.3	16.1	8.0	4.1	1.4	0.9
**Upper CI**	100.0	100.0	98.5	91.2	80.2	72.6	57.9	46.3	38.2	34.7	27.3	23.3	15.8	11.4	3.6	3.0
**Number**	305	305	297	270	237	216	176	141	120	108	89	77	52	32	15	11

Table W468-28 Age Group = 61-70 Gender = OVERALL Occupational group = NON-MANUAL Total cases = 226

	Threshold dB HL≥															
	**0**	**5**	**10**	**15**	**20**	**25**	**30**	**35**	**40**	**45**	**50**	**55**	**65**	**75**	**85**	**95**
**Prevalence**	**100.0**	**100.0**	**99.2**	**98.2**	**95.8**	**91.0**	**85.8**	**79.8**	**69.9**	**62.3**	**53.1**	**44.4**	**26.1**	**14.8**	**10.5**	**5.9**
**Lower CI**	98.3	98.3	95.7	94.2	91.3	85.4	77.6	71.4	61.5	54.0	44.8	36.7	21.3	11.3	8.0	3.8
**Upper CI**	100.0	100.0	99.9	99.4	98.1	94.6	91.3	86.2	77.2	70.0	61.3	52.4	31.6	19.0	13.6	9.0
**Number**	226	226	225	223	218	210	203	191	172	158	136	116	78	49	37	23

Prevalence by age group, gender and occupational group for the worse ear over 4, 6 and 8 kHz

Table W468-29 Age Group = 71-80 Gender = OVERALL Occupational group = NON-MANUAL Total cases = 121

Threshold dB HL≥	0	5	10	15	20	25	30	35	40	45	50	55	65	75	85	95
**Prevalence**	**100.0**	**100.0**	**100.0**	**100.0**	**95.9**	**92.9**	**90.9**	**88.3**	**84.5**	**82.7**	**74.6**	**69.4**	**53.2**	**25.9**	**14.4**	**5.7**
**Lower CI**	96.9	96.9	96.9	96.9	89.4	84.0	82.4	79.3	71.8	70.0	62.8	57.1	41.8	19.3	10.1	3.0
**Upper CI**	100.0	100.0	100.0	100.0	98.5	97.0	95.5	93.7	92.2	90.7	83.6	79.4	64.4	33.9	20.0	10.5
**Number**	121	121	121	121	118	116	112	109	108	106	98	93	70	43	24	12

Table W468-30 Age Group = 18-30 Gender = OVERALL Occupational group = MANUAL Total cases = 217

Threshold dB HL≥	0	5	10	15	20	25	30	35	40	45	50	55	65	75	85	95
**Prevalence**	**97.1**	**87.6**	**65.3**	**41.1**	**25.6**	**19.9**	**15.1**	**9.2**	**6.8**	**4.2**	**2.8**	**1.8**	**0.8**	**0.5**	**0.5**	**0.0**
**Lower CI**	94.2	80.8	56.2	33.0	19.0	14.0	9.9	5.7	3.7	2.3	1.4	0.8	0.2	0.1	0.1	0.0
**Upper CI**	98.5	92.3	73.4	49.7	33.5	27.5	22.2	14.7	12.2	7.8	5.5	4.3	2.6	2.6	2.6	0.2
**Number**	207	192	151	108	75	60	42	31	22	16	13	9	4	2	2	1

Table W468-31 Age Group = 31-40 Gender = OVERALL Occupational group = MANUAL Total cases = 182

Threshold dB HL≥	0	5	10	15	20	25	30	35	40	45	50	55	65	75	85	95
**Prevalence**	**99.3**	**97.6**	**86.6**	**69.5**	**49.5**	**36.8**	**26.1**	**14.1**	**10.5**	**9.1**	**5.9**	**4.1**	**2.4**	**1.9**	**1.4**	**1.3**
**Lower CI**	96.0	91.7	78.5	60.0	40.2	28.3	19.1	9.7	6.9	5.7	3.6	2.3	1.0	0.7	0.4	0.3
**Upper CI**	99.9	99.3	92.0	77.6	58.9	46.2	34.6	20.1	15.6	14.4	9.7	7.2	5.4	5.1	4.8	4.8
**Number**	181	179	160	137	105	83	59	46	38	32	24	17	10	7	5	4

Table W468-32 Age Group = 41-50 Gender = OVERALL Occupational group = MANUAL Total cases = 256

Threshold dB HL≥	0	5	10	15	20	25	30	35	40	45	50	55	65	75	85	95
**Prevalence**	**100.0**	**98.3**	**93.3**	**82.7**	**67.6**	**50.9**	**41.7**	**30.7**	**22.1**	**18.5**	**14.2**	**11.6**	**9.5**	**4.4**	**2.1**	**1.4**
**Lower CI**	98.5	95.1	88.8	76.8	60.6	43.6	34.9	24.7	17.3	14.1	10.4	8.2	6.5	2.5	1.0	0.5
**Upper CI**	100.0	99.4	96.1	87.3	74.0	58.1	48.7	37.4	27.7	24.0	19.0	16.2	13.7	7.6	4.6	3.8
**Number**	256	253	240	219	185	152	128	102	84	68	54	46	39	19	11	7

Table W468-33 Age Group = 51-60 Gender = OVERALL Occupational group = MANUAL Total cases = 357

Threshold dB HL≥	0	5	10	15	20	25	30	35	40	45	50	55	65	75	85	95
**Prevalence**	**100.0**	**99.6**	**98.9**	**94.5**	**86.7**	**76.5**	**65.5**	**55.7**	**48.4**	**39.5**	**29.4**	**24.4**	**15.1**	**10.1**	**6.4**	**4.1**
**Lower CI**	98.9	98.0	96.9	90.9	81.6	70.6	59.4	49.5	42.3	33.9	25.0	20.4	12.1	7.6	4.5	2.6
**Upper CI**	100.0	99.9	99.6	96.7	90.5	81.6	71.2	61.7	54.5	45.3	34.3	29.0	18.8	13.3	9.1	6.3
**Number**	357	356	354	344	326	296	266	239	213	183	156	130	96	62	39	25

Table W468-34 Age Group = 61-70 Gender = OVERALL Occupational group = MANUAL Total cases = 291

Threshold dB HL≥	0	5	10	15	20	25	30	35	40	45	50	55	65	75	85	95
**Prevalence**	**100.0**	**100.0**	**100.0**	**99.8**	**93.1**	**89.3**	**82.2**	**76.1**	**71.6**	**64.3**	**55.6**	**47.3**	**35.1**	**19.9**	**10.1**	**5.3**
**Lower CI**	98.7	98.7	98.7	99.0	87.6	83.2	76.0	69.7	65.2	57.7	49.1	41.1	29.3	15.4	7.3	3.9
**Upper CI**	100.0	100.0	100.0	100.0	96.3	93.4	87.1	81.5	77.3	70.3	61.9	53.6	41.3	25.4	13.7	7.3
**Number**	291	291	291	290	280	274	263	248	234	221	203	185	151	98	58	31

Table W468-35 Age Group = 71-80 Gender = OVERALL Occupational group = MANUAL Total cases = 142

Threshold dB HL≥	0	5	10	15	20	25	30	35	40	45	50	55	65	75	85	95
**Prevalence**	**100.0**	**100.0**	**100.0**	**100.0**	**98.0**	**98.0**	**98.0**	**95.4**	**92.1**	**88.5**	**87.2**	**73.8**	**55.4**	**39.9**	**26.0**	**11.7**
**Lower CI**	97.4	97.4	97.4	97.4	89.5	89.5	89.5	84.4	83.0	76.5	75.2	61.9	44.8	30.9	17.7	7.9
**Upper CI**	100.0	100.0	100.0	100.0	99.7	99.7	99.7	98.8	96.6	94.8	93.8	83.0	65.5	49.6	36.3	17.1
**Number**	142	142	142	142	141	141	141	140	138	135	133	127	111	86	57	33

Prevalence by age group, gender and occupational group for the worse ear over 4, 6 and 8 kHz

Table W468-36 Age Group = 18-80 Gender = FEMALE Occupational group = NON-MANUAL Total cases = 662

	Threshold dB HL≥															
	**0**	**5**	**10**	**15**	**20**	**25**	**30**	**35**	**40**	**45**	**50**	**55**	**65**	**75**	**85**	**95**
**Prevalence**	**98.8**	**93.1**	**79.3**	**62.1**	**50.3**	**43.0**	**35.3**	**29.7**	**24.4**	**21.9**	**17.7**	**15.1**	**9.1**	**3.7**	**2.0**	**1.1**
**Lower CI**	97.3	90.4	75.8	58.6	46.9	39.8	32.5	27.2	21.7	19.3	15.4	13.0	7.3	2.8	1.5	0.7
**Upper CI**	99.5	95.0	82.4	65.5	53.7	46.3	38.2	32.4	27.3	24.7	20.3	17.6	11.4	5.0	2.7	1.6
**Number**	657	631	565	473	400	351	296	255	217	196	165	143	90	57	37	22

Table W468-37 Age Group = 18-80 Gender = MALE Occupational group = NON-MANUAL Total cases = 504

	Threshold dB HL≥															
	**0**	**5**	**10**	**15**	**20**	**25**	**30**	**35**	**40**	**45**	**50**	**55**	**65**	**75**	**85**	**95**
**Prevalence**	**98.8**	**94.1**	**82.9**	**68.8**	**58.3**	**49.3**	**43.4**	**39.0**	**36.0**	**32.2**	**28.4**	**24.5**	**15.4**	**9.0**	**5.3**	**2.6**
**Lower CI**	95.4	90.4	78.2	64.0	53.5	45.1	38.8	34.5	31.6	28.0	24.3	20.5	12.7	7.2	4.1	1.8
**Upper CI**	99.7	96.4	86.7	73.2	62.9	53.6	48.0	43.6	40.7	36.8	32.9	29.0	18.4	11.2	6.9	3.8
**Number**	502	489	460	418	371	335	301	273	251	230	201	176	128	79	46	27

Table W468-38 Age Group = 18-80 Gender = FEMALE Occupational group = MANUAL Total cases = 703

	Threshold dB HL≥															
	**0**	**5**	**10**	**15**	**20**	**25**	**30**	**35**	**40**	**45**	**50**	**55**	**65**	**75**	**85**	**95**
**Prevalence**	**99.1**	**95.3**	**85.4**	**72.5**	**57.4**	**49.8**	**39.8**	**33.6**	**28.3**	**23.3**	**18.3**	**14.1**	**9.4**	**5.4**	**3.5**	**1.7**
**Lower CI**	97.9	92.1	81.1	68.3	53.4	45.9	36.4	30.2	25.2	20.6	16.1	11.9	7.7	4.3	2.5	1.2
**Upper CI**	99.7	97.3	88.8	76.3	61.4	53.7	43.4	37.1	31.6	26.2	20.8	16.6	11.4	6.9	4.7	2.3
**Number**	697	684	636	577	494	437	369	318	274	239	201	167	134	84	54	29

Table W468-39 Age Group = 18-80 Gender = MALE Occupational group = MANUAL Total cases = 742

	Threshold dB HL≥															
	**0**	**5**	**10**	**15**	**20**	**25**	**30**	**35**	**40**	**45**	**50**	**55**	**65**	**75**	**85**	**95**
**Prevalence**	**99.0**	**96.5**	**88.8**	**78.0**	**68.7**	**59.2**	**54.4**	**44.1**	**39.9**	**36.2**	**31.8**	**27.7**	**20.6**	**13.4**	**7.9**	**4.4**
**Lower CI**	97.5	93.5	84.5	73.5	64.3	54.9	50.2	41.2	37.1	33.4	29.3	25.3	18.3	11.4	6.2	3.3
**Upper CI**	99.6	98.1	92.1	82.0	72.7	63.4	58.5	47.1	42.7	39.1	34.4	30.2	23.1	15.8	10.0	5.9
**Number**	737	729	702	663	618	569	530	488	455	416	382	347	277	190	118	72

Table W468-40 Age Group = 18-30 Gender = FEMALE Occupational group = NON-MANUAL Total cases = 114

	Threshold dB HL≥															
	**0**	**5**	**10**	**15**	**20**	**25**	**30**	**35**	**40**	**45**	**50**	**55**	**65**	**75**	**85**	**95**
**Prevalence**	**95.6**	**82.1**	**57.2**	**25.3**	**16.1**	**12.2**	**5.9**	**2.3**	**1.8**	**1.5**	**0.9**	**0.0**	**0.0**	**0.0**	**0.0**	**0.0**
**Lower CI**	89.9	73.5	47.8	17.9	10.4	7.3	3.0	1.0	0.6	0.4	0.2	0.0	0.0	0.0	0.0	0.0
**Upper CI**	98.2	88.4	66.1	34.5	24.1	19.6	11.3	5.3	5.1	5.0	4.7	3.3	3.3	3.3	3.3	3.3
**Number**	109	95	68	31	22	17	10	5	4	3	1	0	0	0	0	0

Table W468-41 Age Group = 31-40 Gender = FEMALE Occupational group = NON-MANUAL Total cases = 85

	Threshold dB HL≥															
	**0**	**5**	**10**	**15**	**20**	**25**	**30**	**35**	**40**	**45**	**50**	**55**	**65**	**75**	**85**	**95**
**Prevalence**	**100.0**	**87.9**	**66.5**	**42.2**	**23.9**	**18.2**	**11.6**	**9.4**	**9.4**	**6.4**	**1.4**	**1.4**	**1.4**	**0.0**	**0.0**	**0.0**
**Lower CI**	95.7	79.6	55.9	32.0	15.5	11.1	6.1	4.5	4.5	2.5	0.3	0.3	0.3	0.0	0.0	0.0
**Upper CI**	100.0	93.1	75.7	53.2	34.8	28.5	20.8	18.3	18.3	15.2	6.5	6.5	6.5	4.3	4.3	4.3
**Number**	85	75	59	41	23	17	11	9	9	6	2	2	2	0	0	0

Table W468-42 Age Group = 41-50 Gender = FEMALE Occupational group = NON-MANUAL Total cases = 112

	Threshold dB HL≥															
	**0**	**5**	**10**	**15**	**20**	**25**	**30**	**35**	**40**	**45**	**50**	**55**	**65**	**75**	**85**	**95**
**Prevalence**	**100.0**	**98.6**	**83.9**	**69.8**	**50.4**	**32.3**	**22.3**	**14.8**	**10.4**	**8.3**	**7.4**	**4.9**	**0.4**	**0.4**	**0.0**	**0.0**
**Lower CI**	96.7	94.1	75.3	60.2	40.6	24.4	16.3	10.1	6.7	4.8	4.2	2.6	0.1	0.1	0.0	0.0
**Upper CI**	100.0	99.7	90.0	77.9	60.2	41.4	29.7	21.1	15.7	13.8	12.5	9.0	2.2	2.2	3.3	3.3
**Number**	112	110	96	81	60	43	30	23	17	13	12	8	1	1	0	0

Prevalence by age group, gender and occupational group for the worse ear over 4, 6 and 8 kHz

Table W468-43 Age Group = 51-60 Gender = FEMALE Occupational group = NON-MANUAL Total cases = 170

	Threshold dB HL≥															
	**0**	**5**	**10**	**15**	**20**	**25**	**30**	**35**	**40**	**45**	**50**	**55**	**65**	**75**	**85**	**95**
**Prevalence**	**100.0**	**100.0**	**94.3**	**81.5**	**69.4**	**60.9**	**45.4**	**32.7**	**23.7**	**20.0**	**15.4**	**10.4**	**6.2**	**3.3**	**2.6**	**1.6**
**Lower CI**	97.8	97.8	88.7	73.9	61.1	52.6	37.6	25.9	18.3	15.2	11.5	7.4	4.3	2.2	1.5	0.7
**Upper CI**	100.0	100.0	97.2	87.3	76.6	68.5	53.4	40.3	30.2	25.9	20.3	14.3	8.7	4.9	4.6	3.4
**Number**	170	170	162	142	124	110	88	68	54	48	40	31	21	13	10	6

Table W468-44 Age Group = 61-70 Gender = FEMALE Occupational group = NON-MANUAL Total cases = 115

	Threshold dB HL≥															
	**0**	**5**	**10**	**15**	**20**	**25**	**30**	**35**	**40**	**45**	**50**	**55**	**65**	**75**	**85**	**95**
**Prevalence**	**100.0**	**100.0**	**98.6**	**96.7**	**92.4**	**86.0**	**81.1**	**76.4**	**60.6**	**53.9**	**44.7**	**38.0**	**15.6**	**9.0**	**4.3**	**2.8**
**Lower CI**	96.8	96.8	92.4	89.8	84.1	76.6	71.4	66.7	50.5	44.5	35.0	29.3	10.1	5.3	3.0	1.6
**Upper CI**	100.0	100.0	99.8	99.0	96.6	92.0	88.1	84.0	69.8	63.1	54.8	47.6	23.4	14.7	5.9	4.8
**Number**	115	115	114	112	108	102	97	91	75	68	57	50	28	20	14	10

Table W468-45 Age Group = 71-80 Gender = FEMALE Occupational group = NON-MANUAL Total cases = 66

	Threshold dB HL≥															
	**0**	**5**	**10**	**15**	**20**	**25**	**30**	**35**	**40**	**45**	**50**	**55**	**65**	**75**	**85**	**95**
**Prevalence**	**100.0**	**100.0**	**100.0**	**100.0**	**93.1**	**89.5**	**88.8**	**87.0**	**80.9**	**80.9**	**71.1**	**69.3**	**53.5**	**19.0**	**9.7**	**3.8**
**Lower CI**	94.5	94.5	94.5	94.5	82.3	75.5	75.0	72.9	61.1	61.1	53.4	51.5	37.3	11.4	5.8	1.6
**Upper CI**	100.0	100.0	100.0	100.0	97.5	96.0	95.4	94.3	92.0	92.0	84.0	82.7	69.0	29.8	16.0	8.6
**Number**	66	66	66	66	63	62	60	59	58	58	53	52	38	23	13	6

Table W468-46 Age Group = 18-30 Gender = MALE Occupational group = NON-MANUAL Total cases = 66

	Threshold dB HL≥															
	**0**	**5**	**10**	**15**	**20**	**25**	**30**	**35**	**40**	**45**	**50**	**55**	**65**	**75**	**85**	**95**
**Prevalence**	**95.7**	**81.9**	**51.1**	**24.6**	**18.4**	**10.0**	**10.0**	**10.0**	**9.7**	**8.4**	**8.4**	**8.4**	**1.6**	**1.6**	**1.4**	**0.0**
**Lower CI**	84.0	69.6	37.4	14.9	10.1	3.9	3.9	3.9	3.7	3.0	3.0	3.0	0.3	0.3	0.3	0.0
**Upper CI**	98.9	90.0	64.5	37.7	31.1	23.1	23.1	23.1	22.9	21.7	21.7	21.7	7.5	7.5	7.8	5.5
**Number**	64	55	36	18	13	6	6	6	5	4	4	4	2	2	1	0

Table W468-47 Age Group = 31-40 Gender = MALE Occupational group = NON-MANUAL Total cases = 62

	Threshold dB HL≥															
	**0**	**5**	**10**	**15**	**20**	**25**	**30**	**35**	**40**	**45**	**50**	**55**	**65**	**75**	**85**	**95**
**Prevalence**	**100.0**	**94.8**	**86.2**	**62.2**	**37.6**	**23.5**	**21.7**	**19.1**	**16.0**	**12.8**	**10.1**	**9.5**	**7.5**	**1.2**	**0.6**	**0.0**
**Lower CI**	94.2	87.3	76.3	46.1	23.4	12.7	11.2	9.3	6.9	5.1	3.5	3.1	2.1	0.6	0.1	0.0
**Upper CI**	100.0	98.0	92.4	76.0	54.4	39.5	38.0	35.3	32.9	28.5	25.9	25.5	23.7	9.9	7.0	5.8
**Number**	62	58	53	42	27	20	17	15	10	9	6	5	4	2	1	0

Table W468-48 Age Group = 41-50 Gender = MALE Occupational group = NON-MANUAL Total cases = 75

	Threshold dB HL≥															
	**0**	**5**	**10**	**15**	**20**	**25**	**30**	**35**	**40**	**45**	**50**	**55**	**65**	**75**	**85**	**95**
**Prevalence**	**100.0**	**100.0**	**93.9**	**84.4**	**69.5**	**53.5**	**39.8**	**35.1**	**29.9**	**24.3**	**23.8**	**17.4**	**7.7**	**4.6**	**2.6**	**1.8**
**Lower CI**	95.1	95.1	86.0	72.5	57.2	41.8	27.9	24.2	19.2	14.7	14.3	9.6	4.8	2.1	1.7	1.4
**Upper CI**	100.0	100.0	97.4	91.7	79.5	64.9	53.0	47.8	43.4	37.4	36.9	29.4	12.0	9.6	3.9	2.4
**Number**	75	75	70	64	53	41	32	29	23	19	18	14	9	7	5	3

Table W468-49 Age Group = 51-60 Gender = MALE Occupational group = NON-MANUAL Total cases = 135

	Threshold dB HL≥															
	**0**	**5**	**10**	**15**	**20**	**25**	**30**	**35**	**40**	**45**	**50**	**55**	**65**	**75**	**85**	**95**
**Prevalence**	**100.0**	**100.0**	**100.0**	**94.7**	**81.9**	**74.4**	**59.5**	**49.3**	**43.5**	**40.4**	**31.6**	**29.5**	**17.1**	**11.0**	**1.8**	**1.8**
**Lower CI**	97.2	97.2	97.2	88.9	73.7	65.8	50.4	40.7	35.4	32.5	25.2	23.4	10.6	5.5	0.7	0.7
**Upper CI**	100.0	100.0	100.0	97.5	88.0	81.4	68.0	58.0	51.9	48.9	38.9	36.5	26.3	20.6	4.2	4.2
**Number**	135	135	135	128	113	106	88	73	66	60	49	46	31	19	5	5

Prevalence by age group, gender and occupational group for the worse ear over 4, 6 and 8 kHz

Table W468-50 Age Group = 61-70 Gender = MALE Occupational group = NON-MANUAL Total cases = 111

	Threshold dB HL≥															
	**0**	5	**10**	15	**20**	25	**30**	35	**40**	45	**50**	55	**65**	75	**85**	95
**Prevalence**	**100.0**	**100.0**	**100.0**	**100.0**	**99.8**	**97.0**	**91.1**	**84.1**	**80.8**	**72.0**	**62.8**	**51.6**	**37.8**	**21.3**	**17.4**	**9.2**
**Lower CI**	96.7	96.7	96.7	96.7	98.9	90.2	73.1	67.7	65.0	56.7	48.2	38.3	30.3	15.7	12.6	5.3
**Upper CI**	100.0	100.0	100.0	100.0	100.0	99.1	97.5	93.0	90.6	83.5	75.3	64.8	45.9	28.2	23.4	15.5
**Number**	111	111	111	111	110	108	106	100	97	90	79	66	50	29	23	13

Table W468-51 Age Group = 71-80 Gender = MALE Occupational group = NON-MANUAL Total cases = 55

	Threshold dB HL≥															
	**0**	5	**10**	15	**20**	25	**30**	35	**40**	45	**50**	55	**65**	75	**85**	95
**Prevalence**	**100.0**	**100.0**	**100.0**	**100.0**	**100.0**	**97.9**	**93.8**	**89.9**	**89.9**	**84.9**	**79.4**	**69.1**	**52.2**	**35.3**	**20.7**	**8.4**
**Lower CI**	93.5	93.5	93.5	93.5	93.5	89.0	85.9	79.6	79.6	73.3	68.1	54.6	39.0	25.0	13.0	3.5
**Upper CI**	100.0	100.0	100.0	100.0	100.0	99.6	97.4	95.3	95.3	92.0	87.4	80.6	65.1	47.2	31.2	18.9
**Number**	55	55	55	55	55	54	52	50	50	48	45	41	32	20	11	6

Table W468-52 Age Group = 18-30 Gender = FEMALE Occupational group = MANUAL Total cases = 109

	Threshold dB HL≥															
	**0**	5	**10**	15	**20**	25	**30**	35	**40**	45	**50**	55	**65**	75	**85**	95
**Prevalence**	**97.7**	**85.4**	**63.8**	**38.4**	**22.7**	**17.4**	**12.6**	**9.6**	**8.0**	**3.6**	**2.5**	**2.1**	**0.2**	**0.0**	**0.0**	**0.0**
**Lower CI**	93.6	74.0	50.9	27.8	14.5	10.3	6.9	4.5	3.4	1.5	1.0	0.7	0.0	0.0	0.0	0.0
**Upper CI**	99.2	92.4	75.0	50.3	33.6	27.9	21.9	19.1	17.5	8.4	6.5	6.0	1.1	3.4	3.4	3.4
**Number**	104	95	73	50	36	27	20	14	11	7	6	5	1	0	0	0

Table W468-53 Age Group = 31-40 Gender = FEMALE Occupational group = MANUAL Total cases = 102

	Threshold dB HL≥															
	**0**	5	**10**	15	**20**	25	**30**	35	**40**	45	**50**	55	**65**	75	**85**	95
**Prevalence**	**98.7**	**98.7**	**83.0**	**67.6**	**42.0**	**31.6**	**17.9**	**13.6**	**8.9**	**7.3**	**5.7**	**3.3**	**1.2**	**0.3**	**0.3**	**0.0**
**Lower CI**	92.8	92.8	70.9	54.6	30.8	21.6	11.1	7.9	5.1	3.8	2.9	1.7	0.5	0.1	0.1	0.0
**Upper CI**	99.8	99.8	90.8	78.4	54.1	43.7	27.6	22.6	14.9	13.6	11.1	6.5	2.9	1.7	1.7	3.6
**Number**	101	101	89	78	55	44	29	24	19	15	13	9	4	1	1	0

Table W468-54 Age Group = 41-50 Gender = FEMALE Occupational group = MANUAL Total cases = 133

	Threshold dB HL≥															
	**0**	5	**10**	15	**20**	25	**30**	35	**40**	45	**50**	55	**65**	75	**85**	95
**Prevalence**	**100.0**	**96.7**	**87.5**	**72.0**	**54.3**	**42.5**	**29.9**	**20.2**	**13.0**	**9.6**	**5.0**	**4.1**	**3.9**	**1.6**	**0.9**	**0.5**
**Lower CI**	97.2	90.8	79.0	62.7	44.4	33.3	22.1	13.9	8.6	6.0	3.1	2.4	2.2	0.7	0.3	0.1
**Upper CI**	100.0	98.9	92.8	79.8	63.9	52.3	39.0	28.4	19.2	14.9	7.9	6.9	6.8	3.5	2.2	1.7
**Number**	133	130	118	102	79	65	50	37	28	22	15	12	11	6	4	2

Table W468-55 Age Group = 51-60 Gender = FEMALE Occupational group = MANUAL Total cases = 164

	Threshold dB HL≥															
	**0**	5	**10**	15	**20**	25	**30**	35	**40**	45	**50**	55	**65**	75	**85**	95
**Prevalence**	**100.0**	**99.3**	**98.0**	**91.2**	**79.7**	**69.6**	**53.6**	**43.1**	**34.8**	**26.9**	**13.2**	**8.1**	**5.2**	**3.4**	**2.4**	**1.9**
**Lower CI**	97.7	96.2	94.1	84.9	71.0	60.4	44.5	34.3	26.6	19.8	8.9	5.0	3.2	2.0	1.4	1.0
**Upper CI**	100.0	99.9	99.3	95.0	86.2	77.4	62.4	52.3	44.0	35.5	19.1	12.7	8.2	5.9	4.1	3.6
**Number**	164	163	161	153	140	123	101	85	70	57	40	28	23	16	12	8

Table W468-56 Age Group = 61-70 Gender = FEMALE Occupational group = MANUAL Total cases = 123

	Threshold dB HL≥															
	**0**	5	**10**	15	**20**	25	**30**	35	**40**	45	**50**	55	**65**	75	**85**	95
**Prevalence**	**100.0**	**100.0**	**100.0**	**99.7**	**88.3**	**81.3**	**69.7**	**62.7**	**55.8**	**47.4**	**38.6**	**28.8**	**21.0**	**12.9**	**5.4**	**2.8**
**Lower CI**	97.0	97.0	97.0	98.1	78.5	70.9	59.3	52.3	45.5	37.7	30.1	21.4	14.6	7.8	3.3	1.8
**Upper CI**	100.0	100.0	100.0	99.9	93.9	88.6	78.4	72.0	65.7	57.3	47.9	37.6	29.3	20.6	8.7	4.2
**Number**	123	123	123	122	113	107	98	88	78	71	61	52	43	25	12	5

Prevalence by age group, gender and occupational group for the worse ear over 4, 6 and 8 kHz

Table W468-57 Age Group = 71-80 Gender = FEMALE Occupational group = MANUAL Total cases = 72

	Threshold dB HL≥															
	0	5	10	15	20	25	30	35	40	45	50	55	65	75	85	95
Prevalence	100.0	100.0	100.0	100.0	96.6	96.6	96.6	92.3	86.8	81.6	81.0	67.3	44.7	27.5	20.5	8.1
Lower CI	94.9	94.9	94.9	94.9	82.6	82.6	82.6	74.8	72.2	62.8	62.4	48.4	30.6	17.7	11.5	4.8
Upper CI	100.0	100.0	100.0	100.0	99.4	99.4	99.4	98.0	94.3	92.0	91.6	81.9	59.8	40.0	33.8	13.3
Number	72	72	72	72	71	71	71	70	68	67	66	61	52	36	25	14

Table W468-58 Age Group = 18-30 Gender = MALE Occupational group = MANUAL Total cases = 108

	Threshold dB HL≥															
	0	5	10	15	20	25	30	35	40	45	50	55	65	75	85	95
Prevalence	96.4	90.0	66.9	43.5	28.4	22.2	17.5	8.6	5.2	4.7	2.9	1.5	1.3	1.0	1.0	0.1
Lower CI	91.3	81.6	54.0	31.9	18.9	13.7	9.8	4.8	2.4	2.0	1.1	0.4	0.3	0.2	0.2	0.0
Upper CI	98.5	94.8	77.6	55.9	40.2	34.0	29.4	14.8	11.1	10.7	7.4	5.0	5.0	5.1	5.1	0.4
Number	103	97	78	58	39	33	22	17	11	9	7	4	3	2	2	1

Table W468-59 Age Group = 31-40 Gender = MALE Occupational group = MANUAL Total cases = 80

	Threshold dB HL≥															
	0	5	10	15	20	25	30	35	40	45	50	55	65	75	85	95
Prevalence	100.0	96.4	90.6	71.3	57.8	42.9	35.5	14.8	12.4	11.3	6.3	4.9	3.6	3.6	2.7	2.7
Lower CI	95.4	84.0	77.9	56.8	42.7	29.5	23.4	8.7	6.7	5.7	3.1	2.2	1.2	1.2	0.7	0.7
Upper CI	100.0	99.3	96.3	82.5	71.5	57.5	49.8	24.2	22.1	21.2	12.3	11.0	10.3	10.3	9.8	9.8
Number	80	78	71	59	50	39	30	22	19	17	11	8	6	6	4	4

Table W468-60 Age Group = 41-50 Gender = MALE Occupational group = MANUAL Total cases = 123

	Threshold dB HL≥															
	0	5	10	15	20	25	30	35	40	45	50	55	65	75	85	95
Prevalence	100.0	100.0	99.7	94.0	81.9	59.8	54.3	42.0	32.0	28.2	24.1	19.7	15.5	7.5	3.4	2.3
Lower CI	97.0	97.0	98.4	85.4	71.2	48.6	43.3	31.8	23.6	20.0	16.6	13.0	9.7	3.9	1.3	0.7
Upper CI	100.0	100.0	99.9	97.6	89.3	70.1	64.9	52.8	41.8	38.2	33.6	28.7	23.8	13.9	8.7	7.5
Number	123	123	122	117	106	87	78	65	56	46	39	34	28	13	7	5

Table W468-61 Age Group = 51-60 Gender = MALE Occupational group = MANUAL Total cases = 193

	Threshold dB HL≥															
	0	5	10	15	20	25	30	35	40	45	50	55	65	75	85	95
Prevalence	100.0	100.0	100.0	98.4	94.4	84.4	78.9	69.9	63.5	53.6	47.4	42.5	26.2	17.5	10.8	6.5
Lower CI	98.0	98.0	98.0	94.4	88.6	76.5	70.6	61.2	54.8	45.3	39.6	34.8	20.2	12.6	7.0	3.7
Upper CI	100.0	100.0	100.0	99.6	97.4	90.0	85.4	77.3	71.4	61.7	55.5	50.6	33.1	23.6	16.3	11.1
Number	193	193	193	191	186	173	165	154	143	126	116	102	73	46	27	17

Table W468-62 Age Group = 61-70 Gender = MALE Occupational group = MANUAL Total cases = 168

	Threshold dB HL≥															
	0	5	10	15	20	25	30	35	40	45	50	55	65	75	85	95
Prevalence	100.0	100.0	100.0	100.0	98.8	98.8	97.2	91.8	90.2	84.2	75.5	69.1	52.0	28.2	15.6	8.4
Lower CI	97.8	97.8	97.8	97.8	93.3	93.3	91.7	85.4	83.7	76.0	65.2	58.8	42.5	21.3	10.4	5.5
Upper CI	100.0	100.0	100.0	100.0	99.8	99.8	99.1	95.6	94.3	90.0	83.6	77.8	61.4	36.4	22.7	12.6
Number	168	168	168	168	167	167	165	160	156	150	142	133	108	73	46	26

Table W468-63 Age Group = 71-80 Gender = MALE Occupational group = MANUAL Total cases = 70

	Threshold dB HL≥															
	0	5	10	15	20	25	30	35	40	45	50	55	65	75	85	95
Prevalence	100.0	100.0	100.0	100.0	100.0	100.0	100.0	100.0	100.0	98.8	96.3	84.0	71.5	58.4	34.2	17.2
Lower CI	94.8	94.8	94.8	94.8	94.8	94.8	94.8	94.8	94.8	95.3	87.0	78.1	56.8	41.9	20.4	9.6
Upper CI	100.0	100.0	100.0	100.0	100.0	100.0	100.0	100.0	100.0	99.7	99.0	88.5	82.7	73.2	51.2	28.8
Number	70	70	70	70	70	70	70	70	70	68	67	66	59	50	32	19

Prevalence by age group, gender and occupational group for the worse ear over 0.25 kHz

Table W250-1 Age Group = 18-80 Gender = OVERALL Occupational group = OVERALL Total cases = 2627

	Threshold dB HL≥															
	0	5	10	15	20	25	30	35	40	45	50	55	65	75	85	95
**Prevalence**	**96.1**	**85.7**	**66.6**	**46.4**	**30.9**	**20.9**	**15.1**	**11.6**	**8.7**	**6.6**	**5.1**	**4.2**	**2.7**	**1.6**	**0.8**	**0.4**
**Lower CI**	94.9	83.8	64.2	44.2	29.0	19.3	13.7	10.5	7.7	5.8	4.5	3.7	2.2	1.3	0.5	0.3
**Upper CI**	97.0	87.4	68.8	48.6	32.8	22.6	16.6	12.9	9.7	7.4	5.8	4.9	3.2	2.1	1.1	0.7
**Number**	2552	2351	1956	1494	1098	816	642	526	423	344	291	249	162	94	48	29

Table W250-2 Age Group = 18-80 Gender = FEMALE Occupational group = OVERALL Total cases = 1370

	Threshold dB HL≥															
	0	5	10	15	20	25	30	35	40	45	50	55	65	75	85	95
**Prevalence**	**95.5**	**85.1**	**67.2**	**46.7**	**32.2**	**22.1**	**15.2**	**11.9**	**9.6**	**7.2**	**5.8**	**4.7**	**2.8**	**1.8**	**0.7**	**0.4**
**Lower CI**	93.8	82.5	64.2	43.9	29.8	20.0	13.5	10.5	8.3	6.1	4.8	3.9	2.2	1.3	0.5	0.2
**Upper CI**	96.8	87.3	70.0	49.6	34.7	24.3	17.2	13.6	11.2	8.4	6.8	5.6	3.6	2.5	1.1	0.7
**Number**	1326	1218	1025	784	575	432	333	282	235	195	169	144	92	53	27	17

Table W250-3 Age Group = 18-80 Gender = MALE Occupational group = OVERALL Total cases = 1257

	Threshold dB HL≥															
	0	5	10	15	20	25	30	35	40	45	50	55	65	75	85	95
**Prevalence**	**96.7**	**86.4**	**65.9**	**46.0**	**29.5**	**19.6**	**15.0**	**11.3**	**7.5**	**5.8**	**4.3**	**3.8**	**2.6**	**1.5**	**0.8**	**0.5**
**Lower CI**	94.9	83.4	62.3	42.6	26.6	17.3	12.9	9.6	6.3	4.8	3.5	3.0	1.9	1.0	0.4	0.2
**Upper CI**	97.9	88.9	69.3	49.5	32.5	22.2	17.2	13.3	9.0	7.0	5.3	4.7	3.4	2.2	1.4	1.1
**Number**	1226	1133	931	710	523	384	309	244	188	149	122	105	70	41	21	12

Table W250-4 Age Group = 18-30 Gender = OVERALL Occupational group = OVERALL Total cases = 401

	Threshold dB HL≥															
	0	5	10	15	20	25	30	35	40	45	50	55	65	75	85	95
**Prevalence**	**91.9**	**76.1**	**50.4**	**26.5**	**15.2**	**9.8**	**5.7**	**4.6**	**2.6**	**1.7**	**0.9**	**0.5**	**0.1**	**0.0**	**0.0**	**0.0**
**Lower CI**	88.2	70.7	44.7	21.9	11.6	6.9	3.6	2.8	1.5	0.9	0.5	0.2	0.0	0.0	0.0	0.0
**Upper CI**	94.5	80.8	56.1	31.7	19.5	13.7	8.9	7.5	4.5	3.0	1.7	1.1	0.5	0.1	0.1	0.1
**Number**	369	318	226	136	81	55	37	31	23	16	11	7	2	1	1	1

Table W250-5 Age Group = 31-40 Gender = OVERALL Occupational group = OVERALL Total cases = 327

	Threshold dB HL≥															
	0	5	10	15	20	25	30	35	40	45	50	55	65	75	85	95
**Prevalence**	**95.8**	**81.5**	**57.0**	**36.3**	**20.5**	**10.9**	**7.9**	**6.0**	**4.6**	**4.1**	**3.2**	**2.3**	**1.0**	**0.8**	**0.7**	**0.6**
**Lower CI**	92.8	76.1	50.3	30.2	15.9	7.8	5.2	3.8	2.8	2.4	1.8	1.1	0.3	0.2	0.1	0.1
**Upper CI**	97.6	85.9	63.4	42.9	26.0	15.1	11.8	9.4	7.5	6.8	5.7	4.6	3.0	3.0	3.0	3.1
**Number**	312	269	200	136	87	53	37	29	23	20	17	11	5	3	2	1

Table W250-6 Age Group = 41-50 Gender = OVERALL Occupational group = OVERALL Total cases = 446

	Threshold dB HL≥															
	0	5	10	15	20	25	30	35	40	45	50	55	65	75	85	95
**Prevalence**	**97.4**	**86.7**	**66.3**	**47.9**	**27.4**	**16.9**	**12.5**	**9.2**	**6.5**	**5.3**	**4.0**	**3.3**	**2.3**	**1.0**	**0.3**	**0.2**
**Lower CI**	95.2	82.8	61.4	43.1	23.4	13.8	9.8	7.1	4.8	3.8	2.7	2.2	1.4	0.5	0.1	0.1
**Upper CI**	98.6	89.9	70.8	52.8	31.9	20.6	15.7	11.7	8.8	7.4	5.8	4.9	3.7	2.0	0.9	0.6
**Number**	436	393	307	231	149	101	81	66	50	41	33	29	19	10	4	3

Table W250-7 Age Group = 51-60 Gender = OVERALL Occupational group = OVERALL Total cases = 669

	Threshold dB HL≥															
	0	5	10	15	20	25	30	35	40	45	50	55	65	75	85	95
**Prevalence**	**98.7**	**91.7**	**75.6**	**53.1**	**36.8**	**22.9**	**17.0**	**12.9**	**10.2**	**7.0**	**5.6**	**4.6**	**2.6**	**1.6**	**1.1**	**0.7**
**Lower CI**	97.5	88.9	71.8	49.0	33.1	20.0	14.5	10.8	8.3	5.5	4.3	3.4	1.9	1.0	0.6	0.3
**Upper CI**	99.4	93.8	79.1	57.2	40.6	26.1	19.9	15.5	12.4	8.9	7.2	6.0	3.7	2.5	2.0	1.5
**Number**	660	621	531	403	304	215	169	133	110	85	73	60	38	22	13	10

Prevalence by age group, gender and occupational group for the worse ear over 0.25 kHz

Table W250-8 Age Group = 61-70 Gender = OVERALL Occupational group = OVERALL Total cases = 518

	Threshold dB HL≥															
	0	5	10	15	20	25	30	35	40	45	50	55	65	75	85	95
Prevalence	**98.0**	**92.8**	**84.7**	**65.6**	**46.6**	**33.8**	**23.7**	**19.4**	**14.3**	**10.7**	**8.9**	**8.2**	**5.2**	**3.3**	**1.1**	**0.8**
Lower CI	95.4	89.2	80.2	60.3	41.5	29.7	20.4	16.4	11.8	8.6	6.9	6.2	3.6	1.9	0.6	0.4
Upper CI	99.2	95.3	88.2	70.6	51.8	38.2	27.3	22.9	17.2	13.4	11.4	10.7	7.4	5.6	1.8	1.6
Number	512	489	451	366	287	232	180	152	124	100	84	75	51	31	13	9

Table W250-9 Age Group = 71-80 Gender = OVERALL Occupational group = OVERALL Total cases = 266

	Threshold dB HL≥															
	0	5	10	15	20	25	30	35	40	45	50	55	65	75	85	95
Prevalence	**99.0**	**96.9**	**86.4**	**79.1**	**66.3**	**54.1**	**43.0**	**32.1**	**25.1**	**20.4**	**16.3**	**13.9**	**10.7**	**6.8**	**2.7**	**0.9**
Lower CI	96.8	91.9	79.1	71.8	59.2	46.1	35.1	25.5	19.2	15.6	12.3	10.6	7.6	4.3	1.6	0.3
Upper CI	99.7	98.9	91.5	85.0	72.7	61.9	51.2	39.5	32.2	26.1	21.2	17.9	14.7	10.7	4.5	2.2
Number	263	261	241	222	190	160	138	115	93	82	73	67	47	27	15	5

Table W250-10 Age Group = 18-80 Gender = OVERALL Occupational group = NON-MANUAL Total cases = 1169

	Threshold dB HL≥															
	0	5	10	15	20	25	30	35	40	45	50	55	65	75	85	95
Prevalence	**94.6**	**83.6**	**60.8**	**39.9**	**25.9**	**18.0**	**13.4**	**10.1**	**7.2**	**4.8**	**3.9**	**3.2**	**2.1**	**1.3**	**0.4**	**0.2**
Lower CI	92.7	80.7	57.3	36.8	23.3	15.8	11.5	8.5	6.1	3.9	3.1	2.5	1.5	0.8	0.3	0.1
Upper CI	96.0	86.0	64.1	43.1	28.7	20.5	15.7	12.1	8.5	6.0	4.9	4.1	2.8	2.0	0.7	0.4
Number	1121	1013	789	570	405	285	229	183	142	112	98	82	55	37	16	10

Table W250-11 Age Group = 18-80 Gender = OVERALL Occupational group = MANUAL Total cases = 1454

	Threshold dB HL≥															
	0	5	10	15	20	25	30	35	40	45	50	55	65	75	85	95
Prevalence	**97.8**	**88.1**	**73.3**	**52.8**	**35.8**	**23.9**	**16.2**	**12.7**	**9.5**	**7.4**	**5.9**	**4.9**	**3.2**	**1.9**	**0.9**	**0.6**
Lower CI	96.4	85.3	69.9	49.3	32.9	21.6	14.3	11.2	8.2	6.3	4.9	4.1	2.5	1.3	0.6	0.3
Upper CI	98.7	90.4	76.4	56.3	38.9	26.5	18.3	14.5	11.0	8.7	7.0	5.9	4.0	2.6	1.4	1.0
Number	1428	1335	1166	923	693	531	413	343	281	232	193	167	107	57	32	19

Table W250-12 Age Group = 18-30 Gender = FEMALE Occupational group = OVERALL Total cases = 224

	Threshold dB HL≥															
	0	5	10	15	20	25	30	35	40	45	50	55	65	75	85	95
Prevalence	**91.3**	**75.2**	**46.9**	**25.3**	**15.0**	**8.8**	**4.0**	**3.4**	**2.8**	**2.0**	**1.2**	**0.5**	**0.0**	**0.0**	**0.0**	**0.0**
Lower CI	86.2	68.3	39.6	19.8	10.9	5.8	2.5	2.0	1.6	1.0	0.6	0.2	0.0	0.0	0.0	0.0
Upper CI	94.6	81.1	54.2	31.8	20.4	13.2	6.2	5.6	4.8	4.1	2.6	1.6	1.7	1.7	1.7	1.7
Number	205	174	118	75	47	32	20	17	14	10	7	3	0	0	0	0

Table W250-13 Age Group = 31-40 Gender = FEMALE Occupational group = OVERALL Total cases = 185

	Threshold dB HL≥															
	0	5	10	15	20	25	30	35	40	45	50	55	65	75	85	95
Prevalence	**92.9**	**76.8**	**57.7**	**32.6**	**17.3**	**9.9**	**6.9**	**4.9**	**4.3**	**3.5**	**2.3**	**1.5**	**0.5**	**0.2**	**0.2**	**0.0**
Lower CI	87.2	69.0	49.2	25.5	12.3	6.3	3.9	2.7	2.2	1.7	1.0	0.7	0.2	0.0	0.0	0.0
Upper CI	96.2	83.1	65.7	40.7	23.7	15.2	11.8	8.7	8.3	7.0	5.1	3.4	1.7	1.2	1.2	2.0
Number	173	148	115	76	44	28	19	14	11	10	8	6	3	1	1	0

Table W250-14 Age Group = 41-50 Gender = FEMALE Occupational group = OVERALL Total cases = 245

	Threshold dB HL≥															
	0	5	10	15	20	25	30	35	40	45	50	55	65	75	85	95
Prevalence	**98.1**	**86.9**	**68.3**	**47.4**	**27.3**	**15.4**	**10.9**	**8.1**	**5.7**	**4.3**	**3.1**	**2.6**	**1.2**	**0.5**	**0.1**	**0.1**
Lower CI	94.9	81.4	61.8	40.9	22.1	11.6	8.0	5.8	3.8	2.7	1.9	1.5	0.6	0.2	0.0	0.0
Upper CI	99.3	90.9	74.1	54.0	33.3	20.1	14.8	11.1	8.5	6.7	5.2	4.4	2.6	1.5	0.7	0.7
Number	241	216	172	125	79	51	41	33	25	20	15	13	7	3	1	1

Prevalence by age group, gender and occupational group for the worse ear over 0.25 kHz

Table W250-15 Age Group = 51-60 Gender = FEMALE Occupational group = OVERALL Total cases = 337

Threshold dB HL≥	0	5	10	15	20	25	30	35	40	45	50	55	65	75	85	95
**Prevalence**	**98.7**	**93.0**	**78.8**	**55.1**	**40.0**	**23.9**	**16.7**	**13.9**	**11.1**	**7.2**	**6.3**	**5.0**	**2.7**	**1.5**	**0.8**	**0.8**
**Lower CI**	96.4	89.2	73.6	49.4	34.7	19.8	13.3	10.8	8.5	5.2	4.5	3.4	1.8	0.9	0.3	0.3
**Upper CI**	99.5	95.6	83.2	60.7	45.5	28.5	20.8	17.7	14.3	9.9	8.9	7.3	4.1	2.6	1.7	1.7
**Number**	333	317	280	213	164	113	83	72	62	48	43	35	24	14	7	7

Table W250-16 Age Group = 61-70 Gender = FEMALE Occupational group = OVERALL Total cases = 239

Threshold dB HL≥	0	5	10	15	20	25	30	35	40	45	50	55	65	75	85	95
**Prevalence**	**97.6**	**93.4**	**86.9**	**67.1**	**47.6**	**35.5**	**23.8**	**19.4**	**15.1**	**12.4**	**10.4**	**9.7**	**6.5**	**4.5**	**1.3**	**1.1**
**Lower CI**	93.4	88.3	81.1	59.9	40.3	29.3	19.2	15.1	11.4	9.1	7.4	6.7	4.0	2.3	0.6	0.5
**Upper CI**	99.2	96.3	91.1	73.6	54.9	42.1	29.1	24.4	19.8	16.7	14.5	13.8	10.5	8.8	2.6	2.5
**Number**	236	227	213	172	133	111	86	72	62	55	48	44	30	18	7	6

Table W250-17 Age Group = 71-80 Gender = FEMALE Occupational group = OVERALL Total cases = 140

Threshold dB HL≥	0	5	10	15	20	25	30	35	40	45	50	55	65	75	85	95
**Prevalence**	**99.2**	**96.0**	**85.1**	**80.5**	**70.9**	**62.1**	**48.1**	**36.4**	**30.1**	**22.8**	**19.0**	**15.6**	**11.5**	**7.9**	**3.5**	**0.9**
**Lower CI**	96.9	88.1	73.9	69.6	61.1	50.3	36.6	27.1	21.3	16.4	13.3	11.0	7.3	4.4	1.9	0.3
**Upper CI**	99.8	98.7	92.1	88.2	79.2	72.6	59.8	46.9	40.7	30.8	26.4	21.6	17.6	13.7	6.2	3.0
**Number**	138	136	127	123	108	97	84	74	61	52	48	43	28	17	11	3

Table W250-18 Age Group = 18-30 Gender = MALE Occupational group = OVERALL Total cases = 177

Threshold dB HL≥	0	5	10	15	20	25	30	35	40	45	50	55	65	75	85	95
**Prevalence**	**92.5**	**77.0**	**54.2**	**27.5**	**15.1**	**10.6**	**7.5**	**5.8**	**2.4**	**1.2**	**0.5**	**0.5**	**0.2**	**0.0**	**0.0**	**0.0**
**Lower CI**	86.2	68.3	45.4	20.4	9.8	6.2	3.9	2.8	0.9	0.5	0.2	0.2	0.1	0.0	0.0	0.0
**Upper CI**	96.0	83.9	62.7	35.9	22.5	17.7	14.2	11.8	6.2	3.2	1.4	1.4	1.0	0.2	0.2	0.2
**Number**	164	144	108	61	34	23	17	14	9	6	4	4	2	1	1	1

Table W250-19 Age Group = 31-40 Gender = MALE Occupational group = OVERALL Total cases = 142

Threshold dB HL≥	0	5	10	15	20	25	30	35	40	45	50	55	65	75	85	95
**Prevalence**	**99.0**	**87.0**	**56.1**	**40.6**	**24.2**	**12.2**	**9.1**	**7.5**	**4.9**	**4.7**	**4.2**	**3.1**	**1.5**	**1.5**	**1.2**	**1.2**
**Lower CI**	97.0	79.0	45.6	30.7	16.6	7.4	5.0	3.9	2.4	2.2	1.9	1.1	0.3	0.3	0.2	0.2
**Upper CI**	99.7	92.3	66.1	51.3	33.8	19.4	16.0	13.9	9.8	9.7	9.2	8.4	6.3	6.3	6.5	6.5
**Number**	139	121	85	60	43	25	18	15	12	10	9	5	2	2	1	1

Table W250-20 Age Group = 41-50 Gender = MALE Occupational group = OVERALL Total cases = 201

Threshold dB HL≥	0	5	10	15	20	25	30	35	40	45	50	55	65	75	85	95
**Prevalence**	**96.7**	**86.6**	**64.1**	**48.5**	**27.4**	**18.6**	**14.1**	**10.3**	**7.4**	**6.3**	**4.9**	**4.1**	**3.4**	**1.5**	**0.5**	**0.3**
**Lower CI**	92.8	80.3	56.6	41.4	21.5	13.8	10.0	7.1	4.7	3.9	2.9	2.4	1.8	0.6	0.2	0.1
**Upper CI**	98.5	91.1	70.9	55.7	34.2	24.6	19.6	14.8	11.3	10.1	8.2	7.1	6.4	3.6	1.6	1.2
**Number**	195	177	135	106	70	50	40	33	25	21	18	16	12	7	3	2

Table W250-21 Age Group = 51-60 Gender = MALE Occupational group = OVERALL Total cases = 332

Threshold dB HL≥	0	5	10	15	20	25	30	35	40	45	50	55	65	75	85	95
**Prevalence**	**98.8**	**90.1**	**72.0**	**50.7**	**33.2**	**21.7**	**17.3**	**11.9**	**9.1**	**6.7**	**4.7**	**4.0**	**2.6**	**1.7**	**1.4**	**0.7**
**Lower CI**	96.9	85.7	66.2	44.8	28.2	17.8	13.9	9.1	6.7	4.7	3.2	2.6	1.5	0.8	0.6	0.2
**Upper CI**	99.5	93.3	77.2	56.6	38.5	26.2	21.4	15.4	12.3	9.6	6.8	6.1	4.5	3.5	3.3	2.3
**Number**	327	304	251	190	140	102	86	61	48	37	30	25	14	8	6	3

Prevalence by age group, gender and occupational group for the worse ear over 0.25 kHz

Table W250-22 Age Group = 61-70 Gender = MALE Occupational group = OVERALL Total cases = 279

Threshold dB HL≥	0	5	10	15	20	25	30	35	40	45	50	55	65	75	85	95
**Prevalence**	**98.5**	**92.2**	**82.0**	**63.9**	**45.5**	**32.0**	**23.6**	**19.6**	**13.5**	**8.9**	**7.3**	**6.5**	**3.5**	**1.8**	**0.8**	**0.4**
Lower CI	94.1	86.0	74.7	55.8	38.7	26.9	19.4	15.6	10.5	6.7	5.2	4.5	2.3	1.1	0.4	0.1
Upper CI	99.7	95.8	87.5	71.3	52.5	37.6	28.5	24.3	17.0	11.9	10.1	9.4	5.3	3.0	1.7	1.3
Number	276	262	238	194	154	121	94	80	62	45	36	31	21	13	6	3

Table W250-23 Age Group = 71-80 Gender = MALE Occupational group = OVERALL Total cases = 126

Threshold dB HL≥	0	5	10	15	20	25	30	35	40	45	50	55	65	75	85	95
**Prevalence**	**98.7**	**98.7**	**88.5**	**77.3**	**59.1**	**42.1**	**35.4**	**25.9**	**17.7**	**16.4**	**12.1**	**11.4**	**9.5**	**5.3**	**1.6**	**0.7**
Lower CI	92.7	92.7	79.7	68.0	49.5	32.4	26.6	18.1	11.8	10.7	8.1	7.6	6.0	2.7	0.6	0.2
Upper CI	99.8	99.8	93.8	84.6	68.1	52.5	45.3	35.7	25.7	24.3	17.5	16.6	14.7	10.2	4.2	2.6
Number	125	125	114	99	82	63	54	41	32	30	25	24	19	10	4	2

Table W250-24 Age Group = 18-30 Gender = OVERALL Occupational group = NON-MANUAL Total cases = 181

Threshold dB HL≥	0	5	10	15	20	25	30	35	40	45	50	55	65	75	85	95
**Prevalence**	**90.1**	**73.0**	**44.9**	**17.8**	**9.8**	**8.4**	**5.5**	**5.2**	**1.3**	**0.7**	**0.5**	**0.2**	**0.0**	**0.0**	**0.0**	**0.0**
Lower CI	84.4	64.9	37.1	12.1	5.5	4.4	2.4	2.2	0.6	0.4	0.2	0.0	0.0	0.0	0.0	0.0
Upper CI	93.9	79.8	52.9	25.4	16.9	15.4	12.1	11.9	2.9	1.5	1.4	1.1	2.1	2.1	2.1	2.1
Number	163	137	84	40	21	18	13	11	7	4	3	1	0	0	0	0

Table W250-25 Age Group = 31-40 Gender = OVERALL Occupational group = NON-MANUAL Total cases = 146

Threshold dB HL≥	0	5	10	15	20	25	30	35	40	45	50	55	65	75	85	95
**Prevalence**	**93.5**	**78.4**	**48.4**	**31.1**	**16.6**	**10.2**	**7.4**	**5.0**	**4.4**	**4.4**	**3.0**	**1.5**	**0.0**	**0.0**	**0.0**	**0.0**
Lower CI	88.3	71.1	39.0	23.0	11.1	5.8	3.8	2.3	1.8	1.8	1.0	0.3	0.0	0.0	0.0	0.0
Upper CI	96.5	84.4	58.0	40.6	24.2	17.1	13.9	10.7	10.3	10.3	8.7	8.0	2.6	2.6	2.6	2.6
Number	137	113	77	48	27	16	12	9	7	7	5	1	0	0	0	0

Table W250-26 Age Group = 41-50 Gender = OVERALL Occupational group = NON-MANUAL Total cases = 187

Threshold dB HL≥	0	5	10	15	20	25	30	35	40	45	50	55	65	75	85	95
**Prevalence**	**95.3**	**85.6**	**61.4**	**43.1**	**22.4**	**11.4**	**10.9**	**8.7**	**6.5**	**5.3**	**4.6**	**4.0**	**3.3**	**1.6**	**0.1**	**0.1**
Lower CI	90.4	79.4	53.7	35.8	16.9	7.4	7.0	5.6	3.7	2.9	2.3	1.9	1.4	0.4	0.0	0.0
Upper CI	97.7	90.1	68.6	50.8	29.0	17.0	16.6	13.5	11.1	9.5	8.9	8.1	7.7	6.4	0.7	0.7
Number	179	158	113	81	49	24	23	19	14	11	9	8	7	4	1	1

Table W250-27 Age Group = 51-60 Gender = OVERALL Occupational group = NON-MANUAL Total cases = 310

Threshold dB HL≥	0	5	10	15	20	25	30	35	40	45	50	55	65	75	85	95
**Prevalence**	**98.3**	**89.5**	**71.1**	**43.7**	**28.9**	**17.3**	**11.8**	**8.5**	**6.8**	**4.4**	**4.3**	**3.4**	**2.4**	**1.5**	**0.6**	**0.5**
Lower CI	96.1	85.2	64.7	38.1	24.3	14.0	9.2	6.4	4.9	3.1	2.9	2.2	1.4	0.8	0.2	0.2
Upper CI	99.3	92.6	76.8	49.5	34.0	21.0	15.0	11.2	9.3	6.4	6.2	5.2	4.0	2.7	1.5	1.4
Number	304	279	228	157	118	80	62	48	38	29	28	23	16	11	5	4

Table W250-28 Age Group = 61-70 Gender = OVERALL Occupational group = NON-MANUAL Total cases = 223

Threshold dB HL≥	0	5	10	15	20	25	30	35	40	45	50	55	65	75	85	95
**Prevalence**	**97.1**	**91.9**	**79.5**	**62.4**	**43.2**	**30.9**	**21.5**	**17.8**	**12.6**	**8.1**	**6.6**	**6.1**	**3.5**	**1.8**	**0.7**	**0.7**
Lower CI	92.3	86.2	71.2	55.3	36.5	25.4	17.6	14.0	9.8	6.2	4.9	4.5	2.1	1.0	0.3	0.3
Upper CI	98.9	95.4	85.9	69.1	50.2	37.0	26.1	22.3	16.1	10.3	8.7	8.3	5.6	3.1	1.8	1.8
Number	219	208	183	148	111	85	66	55	44	35	29	27	17	10	4	4

Prevalence by age group, gender and occupational group for the worse ear over 0.25 kHz

Table W250-29 Age Group = 71-80 Gender = OVERALL Occupational group = NON-MANUAL Total cases = 122

Threshold dB HL≥	0	5	10	15	20	25	30	35	40	45	50	55	65	75	85	95
**Prevalence**	**98.1**	**97.0**	**81.1**	**74.7**	**62.7**	**51.9**	**41.9**	**27.1**	**22.3**	**12.9**	**9.3**	**8.8**	**6.6**	**5.6**	**2.3**	**0.3**
**Lower CI**	94.0	91.7	68.2	62.3	51.1	40.8	32.0	20.9	16.6	7.9	6.4	5.9	4.0	3.1	1.0	0.1
**Upper CI**	99.4	98.9	89.5	84.0	73.0	62.8	52.5	34.4	29.1	20.2	13.4	13.0	10.7	10.0	5.3	1.9
**Number**	119	118	104	96	79	62	53	41	32	26	24	22	15	12	6	1

Table W250-30 Age Group = 18-30 Gender = OVERALL Occupational group = MANUAL Total cases = 218

Threshold dB HL≥	0	5	10	15	20	25	30	35	40	45	50	55	65	75	85	95
**Prevalence**	**94.8**	**78.1**	**58.3**	**36.3**	**22.0**	**12.8**	**6.1**	**4.3**	**3.3**	**2.1**	**0.9**	**0.6**	**0.2**	**0.0**	**0.0**	**0.0**
**Lower CI**	90.2	69.5	49.3	28.3	15.7	8.0	3.2	2.4	1.7	1.0	0.4	0.2	0.0	0.0	0.0	0.0
**Upper CI**	97.3	84.7	66.8	45.2	30.1	20.0	11.2	7.6	6.4	4.5	2.0	1.3	0.7	0.2	0.2	0.2
**Number**	205	180	142	96	60	37	24	20	16	12	8	6	2	1	1	1

Table W250-31 Age Group = 31-40 Gender = OVERALL Occupational group = MANUAL Total cases = 181

Threshold dB HL≥	0	5	10	15	20	25	30	35	40	45	50	55	65	75	85	95
**Prevalence**	**98.1**	**85.9**	**67.0**	**41.5**	**23.4**	**11.5**	**8.3**	**7.6**	**4.5**	**3.4**	**3.0**	**2.7**	**1.5**	**1.3**	**1.1**	**0.9**
**Lower CI**	94.8	78.5	57.9	32.5	17.0	7.4	4.8	4.1	2.4	1.7	1.5	1.2	0.5	0.3	0.2	0.2
**Upper CI**	99.3	91.1	75.1	51.0	31.3	17.3	14.1	13.4	8.4	6.5	6.2	5.9	4.8	4.8	4.9	5.0
**Number**	175	156	123	88	60	37	25	20	16	13	12	10	5	3	2	1

Table W250-32 Age Group = 41-50 Gender = OVERALL Occupational group = MANUAL Total cases = 259

Threshold dB HL≥	0	5	10	15	20	25	30	35	40	45	50	55	65	75	85	95
**Prevalence**	**99.2**	**89.6**	**73.6**	**54.5**	**33.1**	**22.9**	**14.8**	**9.9**	**7.6**	**6.5**	**4.4**	**3.4**	**2.1**	**0.8**	**0.4**	**0.2**
**Lower CI**	96.7	84.6	67.1	47.6	26.9	17.8	10.8	7.2	5.2	4.2	2.8	2.2	1.2	0.4	0.1	0.1
**Upper CI**	99.8	93.1	79.2	61.3	39.9	29.1	19.9	13.5	11.1	9.9	7.0	5.2	3.6	1.8	1.0	0.9
**Number**	257	235	194	150	100	77	58	47	36	30	24	21	12	6	3	2

Table W250-33 Age Group = 51-60 Gender = OVERALL Occupational group = MANUAL Total cases = 359

Threshold dB HL≥	0	5	10	15	20	25	30	35	40	45	50	55	65	75	85	95
**Prevalence**	**99.4**	**94.4**	**79.7**	**61.2**	**43.4**	**27.0**	**20.4**	**16.0**	**12.1**	**8.6**	**6.3**	**5.3**	**2.7**	**1.5**	**1.1**	**0.7**
**Lower CI**	98.0	90.6	74.0	54.9	37.8	22.6	16.5	12.5	9.3	6.3	4.4	3.5	1.7	0.8	0.5	0.3
**Upper CI**	99.8	96.8	84.5	67.2	49.2	31.9	24.9	20.2	15.6	11.7	9.0	7.8	4.3	2.8	2.4	1.6
**Number**	356	342	303	246	186	135	107	85	72	56	45	37	22	11	8	6

Table W250-34 Age Group = 61-70 Gender = OVERALL Occupational group = MANUAL Total cases = 294

Threshold dB HL≥	0	5	10	15	20	25	30	35	40	45	50	55	65	75	85	95
**Prevalence**	**98.9**	**94.2**	**88.1**	**67.1**	**49.7**	**34.8**	**24.9**	**20.1**	**15.2**	**12.1**	**10.9**	**9.7**	**6.5**	**4.3**	**1.6**	**1.2**
**Lower CI**	94.6	89.3	82.1	59.3	42.6	29.7	20.7	16.6	11.8	9.0	7.9	6.8	4.2	2.4	1.2	0.9
**Upper CI**	99.8	96.9	92.3	74.0	56.7	40.4	29.6	24.1	19.4	16.0	14.8	13.8	9.9	7.7	2.3	1.7
**Number**	292	280	267	217	176	147	114	97	80	65	55	48	34	21	9	5

Table W250-35 Age Group = 71-80 Gender = OVERALL Occupational group = MANUAL Total cases = 143

Threshold dB HL≥	0	5	10	15	20	25	30	35	40	45	50	55	65	75	85	95
**Prevalence**	**100.0**	**96.9**	**93.0**	**83.5**	**70.1**	**61.0**	**42.4**	**35.6**	**26.5**	**23.8**	**21.2**	**16.8**	**13.4**	**7.7**	**2.9**	**0.9**
**Lower CI**	97.4	83.9	80.6	71.3	59.3	49.6	31.8	26.4	18.9	16.5	14.0	11.9	8.8	4.0	1.7	0.4
**Upper CI**	100.0	99.5	97.7	91.2	79.1	71.2	53.7	46.0	35.8	33.1	30.7	23.2	19.9	14.1	5.0	2.3
**Number**	143	142	137	126	111	98	85	74	61	56	49	45	32	15	9	4

Prevalence by age group, gender and occupational group for the worse ear over 0.25 kHz

Table W250-36 Age Group = 18-80 Gender = FEMALE Occupational group = NON-MANUAL Total cases = 664

Threshold dB HL≥	0	5	10	15	20	25	30	35	40	45	50	55	65	75	85	95
Prevalence	**93.4**	**83.4**	**61.8**	**42.6**	**28.8**	**19.7**	**14.0**	**10.6**	**8.3**	**5.2**	**4.0**	**3.1**	**1.9**	**1.5**	**0.7**	**0.4**
Lower CI	90.7	79.9	57.6	38.8	25.5	17.1	11.7	9.0	6.8	4.1	3.1	2.4	1.4	1.0	0.4	0.2
Upper CI	95.4	86.4	65.9	46.5	32.2	22.6	16.5	12.4	10.0	6.7	5.1	3.9	2.6	2.2	1.3	0.8
Number	633	576	457	343	246	179	141	117	93	77	67	56	35	26	14	9

Table W250-37 Age Group = 18-80 Gender = MALE Occupational group = NON-MANUAL Total cases = 505

Threshold dB HL≥	0	5	10	15	20	25	30	35	40	45	50	55	65	75	85	95
Prevalence	**95.9**	**83.8**	**59.6**	**36.9**	**22.6**	**16.1**	**12.8**	**9.6**	**5.9**	**4.4**	**3.8**	**3.3**	**2.3**	**1.1**	**0.1**	**0.0**
Lower CI	92.8	79.1	53.9	32.0	18.7	12.5	9.7	6.8	4.3	2.9	2.5	2.1	1.3	0.5	0.0	0.0
Upper CI	97.7	87.6	65.0	42.2	27.1	20.4	16.6	13.3	8.0	6.4	5.7	5.2	3.7	2.6	0.4	0.2
Number	488	437	332	227	159	106	88	66	49	35	31	26	20	11	2	1

Table W250-38 Age Group = 18-80 Gender = FEMALE Occupational group = MANUAL Total cases = 705

Threshold dB HL≥	0	5	10	15	20	25	30	35	40	45	50	55	65	75	85	95
Prevalence	**97.9**	**87.2**	**73.8**	**52.2**	**36.7**	**25.1**	**15.9**	**13.2**	**10.5**	**8.1**	**6.8**	**5.5**	**3.4**	**1.9**	**0.8**	**0.5**
Lower CI	95.9	83.1	69.0	47.5	32.7	21.7	13.6	11.1	8.6	6.5	5.3	4.3	2.5	1.2	0.5	0.3
Upper CI	99.0	90.3	78.0	56.8	40.9	28.8	18.7	15.5	12.7	10.0	8.6	7.0	4.6	3.1	1.2	0.7
Number	692	641	567	440	329	253	192	165	142	118	102	88	57	27	13	8

Table W250-39 Age Group = 18-80 Gender = MALE Occupational group = MANUAL Total cases = 749

Threshold dB HL≥	0	5	10	15	20	25	30	35	40	45	50	55	65	75	85	95
Prevalence	**97.7**	**89.1**	**72.8**	**53.5**	**34.9**	**22.7**	**16.6**	**12.3**	**8.4**	**6.7**	**4.9**	**4.2**	**2.9**	**1.8**	**1.1**	**0.7**
Lower CI	95.4	85.0	67.9	48.3	30.7	19.5	13.7	10.1	6.8	5.4	3.8	3.2	2.1	1.1	0.6	0.3
Upper CI	98.9	92.2	77.2	58.6	39.4	26.2	19.9	15.0	10.3	8.4	6.3	5.5	4.1	2.9	2.0	1.7
Number	736	694	599	483	364	278	221	178	139	114	91	79	50	30	19	11

Table W250-40 Age Group = 18-30 Gender = FEMALE Occupational group = NON-MANUAL Total cases = 115

Threshold dB HL≥	0	5	10	15	20	25	30	35	40	45	50	55	65	75	85	95
Prevalence	**87.8**	**71.0**	**40.1**	**18.2**	**8.9**	**6.1**	**4.1**	**3.7**	**2.4**	**1.4**	**0.9**	**0.4**	**0.0**	**0.0**	**0.0**	**0.0**
Lower CI	80.3	61.9	31.2	12.6	5.4	3.6	2.3	2.0	1.1	0.7	0.3	0.1	0.0	0.0	0.0	0.0
Upper CI	92.8	78.7	49.7	25.7	14.4	10.0	7.4	6.9	5.3	2.9	2.7	2.2	3.2	3.2	3.2	3.2
Number	102	84	51	28	15	12	9	8	6	4	3	1	0	0	0	0

Table W250-41 Age Group = 31-40 Gender = FEMALE Occupational group = NON-MANUAL Total cases = 84

Threshold dB HL≥	0	5	10	15	20	25	30	35	40	45	50	55	65	75	85	95
Prevalence	**88.5**	**75.5**	**48.7**	**26.7**	**13.8**	**8.7**	**6.4**	**4.2**	**3.2**	**3.2**	**1.7**	**0.0**	**0.0**	**0.0**	**0.0**	**0.0**
Lower CI	78.8	64.4	37.3	17.9	7.6	4.0	2.5	1.6	0.9	0.9	0.3	0.0	0.0	0.0	0.0	0.0
Upper CI	94.1	84.0	60.2	37.8	23.7	17.9	15.1	10.8	10.5	10.5	8.0	4.4	4.4	4.4	4.4	4.4
Number	76	65	44	26	13	8	6	5	3	3	2	0	0	0	0	0

Table W250-42 Age Group = 41-50 Gender = FEMALE Occupational group = NON-MANUAL Total cases = 112

Threshold dB HL≥	0	5	10	15	20	25	30	35	40	45	50	55	65	75	85	95
Prevalence	**97.3**	**87.8**	**65.7**	**46.0**	**27.8**	**10.5**	**9.7**	**7.9**	**4.5**	**2.9**	**1.6**	**0.6**	**0.6**	**0.0**	**0.0**	**0.0**
Lower CI	91.9	79.9	56.1	36.4	20.5	6.6	5.8	4.6	2.1	1.3	0.5	0.1	0.1	0.0	0.0	0.0
Upper CI	99.1	92.9	74.3	55.8	36.5	16.4	15.6	13.2	9.6	6.5	5.3	4.3	4.3	3.3	3.3	3.3
Number	109	97	72	52	33	14	13	10	6	4	2	1	1	0	0	0

Prevalence by age group, gender and occupational group for the worse ear over 0.25 kHz

Table W250-43 Age Group = 51-60 Gender = FEMALE Occupational group = NON-MANUAL Total cases = 173

Threshold dB HL≥	0	5	10	15	20	25	30	35	40	45	50	55	65	75	85	95
**Prevalence**	**98.3**	**92.9**	**77.3**	**50.8**	**34.3**	**21.1**	**13.2**	**10.9**	**9.5**	**5.6**	**5.4**	**4.5**	**3.0**	**2.2**	**0.9**	**0.9**
**Lower CI**	94.5	88.0	70.2	43.0	27.5	16.2	9.6	7.8	6.5	3.7	3.5	2.8	1.6	1.1	0.3	0.3
**Upper CI**	99.5	95.9	83.1	58.5	41.9	27.1	18.0	15.1	13.6	8.4	8.2	7.3	5.3	4.4	2.6	2.6
**Number**	170	161	137	99	73	52	38	33	28	21	20	17	12	9	4	4

Table W250-44 Age Group = 61-70 Gender = FEMALE Occupational group = NON-MANUAL Total cases = 113

Threshold dB HL≥	0	5	10	15	20	25	30	35	40	45	50	55	65	75	85	95
**Prevalence**	**96.9**	**90.9**	**83.0**	**69.6**	**45.2**	**31.2**	**19.6**	**14.6**	**10.5**	**8.9**	**6.8**	**6.4**	**3.3**	**2.5**	**1.3**	**1.3**
**Lower CI**	89.3	82.0	73.9	59.1	35.6	23.8	14.5	10.4	7.2	6.7	5.0	4.6	2.1	1.3	0.5	0.5
**Upper CI**	99.2	95.6	89.4	78.3	55.2	39.9	25.8	20.0	15.1	11.6	9.0	8.7	5.3	4.8	3.3	3.3
**Number**	111	105	97	84	64	50	38	30	25	24	20	19	11	8	4	4

Table W250-45 Age Group = 71-80 Gender = FEMALE Occupational group = NON-MANUAL Total cases = 67

Threshold dB HL≥	0	5	10	15	20	25	30	35	40	45	50	55	65	75	85	95
**Prevalence**	**98.2**	**96.4**	**79.2**	**76.1**	**71.6**	**65.8**	**50.7**	**35.2**	**30.2**	**15.7**	**12.1**	**11.3**	**7.7**	**6.9**	**3.9**	**0.5**
**Lower CI**	92.4	88.5	59.8	57.4	53.4	48.4	36.3	26.7	22.1	8.8	7.6	6.9	3.9	3.3	1.7	0.1
**Upper CI**	99.6	98.9	90.7	88.2	84.8	79.8	65.0	44.7	39.6	26.5	18.7	18.0	14.5	13.8	8.9	3.0
**Number**	65	64	56	54	48	43	37	31	25	21	20	18	11	9	6	1

Table W250-46 Age Group = 18-30 Gender = MALE Occupational group = NON-MANUAL Total cases = 66

Threshold dB HL≥	0	5	10	15	20	25	30	35	40	45	50	55	65	75	85	95
**Prevalence**	**92.4**	**74.8**	**49.7**	**17.1**	**10.6**	**10.6**	**6.9**	**6.6**	**0.1**	**0.0**	**0.0**	**0.0**	**0.0**	**0.0**	**0.0**	**0.0**
**Lower CI**	81.6	60.7	37.1	8.4	4.0	4.0	1.9	1.8	0.0	0.0	0.0	0.0	0.0	0.0	0.0	0.0
**Upper CI**	97.1	85.1	62.4	31.6	25.3	25.3	21.5	21.5	5.7	5.5	5.5	5.5	5.5	5.5	5.5	5.5
**Number**	61	53	33	12	6	6	4	3	1	0	0	0	0	0	0	0

Table W250-47 Age Group = 31-40 Gender = MALE Occupational group = NON-MANUAL Total cases = 62

Threshold dB HL≥	0	5	10	15	20	25	30	35	40	45	50	55	65	75	85	95
**Prevalence**	**99.1**	**82.3**	**48.4**	**36.6**	**19.7**	**11.7**	**8.4**	**5.7**	**5.7**	**5.7**	**4.4**	**3.2**	**0.0**	**0.0**	**0.0**	**0.0**
**Lower CI**	-	72.3	33.5	23.1	11.2	5.3	3.2	1.8	1.8	1.8	1.1	0.6	0.0	0.0	0.0	0.0
**Upper CI**	-	89.3	63.6	52.5	32.3	23.6	19.8	16.9	16.9	16.9	15.9	16.3	5.8	5.8	5.8	5.8
**Number**	61	48	33	22	14	8	6	4	4	4	3	1	0	0	0	0

Table W250-48 Age Group = 41-50 Gender = MALE Occupational group = NON-MANUAL Total cases = 75

Threshold dB HL≥	0	5	10	15	20	25	30	35	40	45	50	55	65	75	85	95
**Prevalence**	**93.1**	**83.3**	**56.8**	**40.2**	**16.3**	**12.3**	**12.3**	**9.7**	**8.7**	**7.9**	**7.9**	**7.9**	**6.5**	**3.5**	**0.3**	**0.3**
**Lower CI**	83.6	72.9	44.7	29.2	9.2	6.1	6.1	4.8	4.1	3.5	3.5	3.5	2.5	0.8	0.0	0.0
**Upper CI**	97.2	90.3	68.2	52.2	27.4	23.4	23.4	18.8	17.5	16.7	16.7	16.7	16.0	13.5	1.4	1.4
**Number**	70	61	41	29	16	10	10	9	8	7	7	7	6	4	1	1

Table W250-49 Age Group = 51-60 Gender = MALE Occupational group = NON-MANUAL Total cases = 137

Threshold dB HL≥	0	5	10	15	20	25	30	35	40	45	50	55	65	75	85	95
**Prevalence**	**98.3**	**85.6**	**64.1**	**35.7**	**22.8**	**12.9**	**10.3**	**5.8**	**3.7**	**3.1**	**3.1**	**2.2**	**1.7**	**0.6**	**0.3**	**0.0**
**Lower CI**	94.7	77.9	53.0	27.9	17.2	9.3	7.0	3.5	1.9	1.5	1.5	0.9	0.6	0.2	0.1	0.0
**Upper CI**	99.5	90.9	73.9	44.4	29.7	17.5	14.8	9.5	6.9	6.2	6.2	5.0	4.4	2.3	1.7	2.7
**Number**	134	118	91	58	45	28	24	15	10	8	8	6	4	2	1	0

Prevalence by age group, gender and occupational group for the worse ear over 0.25 kHz

Table W250-50 Age Group = 61-70 Gender = MALE Occupational group = NON-MANUAL Total cases = 110

Threshold dB HL≥	0	5	10	15	20	25	30	35	40	45	50	55	65	75	85	95
**Prevalence**	**97.3**	**93.1**	**75.1**	**54.1**	**41.1**	**30.8**	**23.8**	**21.5**	**14.9**	**7.2**	**6.4**	**5.9**	**3.5**	**0.9**	**0.0**	**0.0**
**Lower CI**	89.1	84.6	59.7	44.5	32.2	23.1	17.9	15.4	10.6	4.6	3.9	3.6	1.5	0.3	0.0	0.0
**Upper CI**	99.4	97.1	85.9	63.4	50.6	39.7	31.0	29.2	20.4	11.1	10.4	9.7	7.9	2.7	3.4	3.4
**Number**	108	103	86	64	47	35	28	25	19	11	9	8	6	2	0	0

Table W250-51 Age Group = 71-80 Gender = MALE Occupational group = NON-MANUAL Total cases = 55

Threshold dB HL≥	0	5	10	15	20	25	30	35	40	45	50	55	65	75	85	95
**Prevalence**	**97.9**	**97.9**	**83.9**	**72.9**	**48.5**	**30.5**	**27.5**	**15.3**	**10.5**	**8.0**	**4.9**	**4.9**	**4.9**	**3.7**	**0.0**	**0.0**
**Lower CI**	89.0	89.0	69.9	59.0	37.0	20.2	17.8	8.2	5.1	3.5	2.9	2.9	2.9	1.5	0.0	0.0
**Upper CI**	99.6	99.6	92.1	83.4	60.1	43.3	40.0	26.9	20.4	17.1	8.1	8.1	8.1	8.8	6.5	6.5
**Number**	54	54	48	42	31	19	16	10	7	5	4	4	4	3	0	0

Table W250-52 Age Group = 18-30 Gender = FEMALE Occupational group = MANUAL Total cases = 109

Threshold dB HL≥	0	5	10	15	20	25	30	35	40	45	50	55	65	75	85	95
**Prevalence**	**96.2**	**79.5**	**57.3**	**36.5**	**25.0**	**14.2**	**4.0**	**3.6**	**3.2**	**2.4**	**1.2**	**0.5**	**0.0**	**0.0**	**0.0**	**0.0**
**Lower CI**	89.9	67.5	44.9	25.6	15.9	7.5	2.0	1.6	1.4	0.9	0.4	0.1	0.0	0.0	0.0	0.0
**Upper CI**	98.7	87.9	68.9	49.1	37.0	25.2	8.0	7.7	7.2	6.3	3.1	1.7	3.4	3.4	3.4	3.4
**Number**	103	90	67	47	32	20	11	9	8	6	4	2	0	0	0	0

Table W250-53 Age Group = 31-40 Gender = FEMALE Occupational group = MANUAL Total cases = 101

Threshold dB HL≥	0	5	10	15	20	25	30	35	40	45	50	55	65	75	85	95
**Prevalence**	**97.2**	**78.7**	**68.3**	**37.7**	**18.6**	**9.9**	**6.4**	**5.2**	**4.9**	**3.1**	**2.5**	**2.5**	**0.8**	**0.3**	**0.3**	**0.0**
**Lower CI**	90.9	66.8	55.7	27.4	12.1	5.8	3.3	2.4	2.1	1.5	1.1	1.1	0.3	0.1	0.1	0.0
**Upper CI**	99.2	87.2	78.7	49.2	27.6	16.3	12.1	11.1	11.0	6.5	5.7	5.7	2.3	1.7	1.7	3.7
**Number**	97	83	71	50	31	20	13	9	8	7	6	6	3	1	1	0

Table W250-54 Age Group = 41-50 Gender = FEMALE Occupational group = MANUAL Total cases = 133

Threshold dB HL≥	0	5	10	15	20	25	30	35	40	45	50	55	65	75	85	95
**Prevalence**	**98.9**	**87.3**	**73.5**	**50.1**	**29.6**	**21.3**	**12.3**	**8.4**	**7.2**	**5.8**	**4.2**	**3.9**	**2.0**	**1.0**	**0.3**	**0.3**
**Lower CI**	93.8	79.8	64.9	40.7	22.1	15.0	8.0	5.5	4.4	3.4	2.6	2.3	1.1	0.3	0.0	0.0
**Upper CI**	99.8	92.3	80.6	59.5	38.3	29.3	18.4	12.7	11.4	9.7	6.9	6.4	3.7	2.8	1.5	1.5
**Number**	132	119	100	73	46	37	28	23	19	16	13	12	6	3	1	1

Table W250-55 Age Group = 51-60 Gender = FEMALE Occupational group = MANUAL Total cases = 164

Threshold dB HL≥	0	5	10	15	20	25	30	35	40	45	50	55	65	75	85	95
**Prevalence**	**99.3**	**94.1**	**81.1**	**61.6**	**47.0**	**27.0**	**20.5**	**17.3**	**12.4**	**8.3**	**7.1**	**5.4**	**2.3**	**0.9**	**0.5**	**0.5**
**Lower CI**	96.2	87.8	72.5	52.5	38.9	20.8	14.7	11.9	8.3	5.2	4.2	2.9	1.2	0.4	0.2	0.2
**Upper CI**	99.9	97.2	87.5	70.0	55.3	34.3	27.8	24.4	17.9	13.1	11.7	9.7	4.5	1.8	1.5	1.5
**Number**	163	156	143	114	91	61	45	39	34	27	23	18	12	5	3	3

Table W250-56 Age Group = 61-70 Gender = FEMALE Occupational group = MANUAL Total cases = 125

Threshold dB HL≥	0	5	10	15	20	25	30	35	40	45	50	55	65	75	85	95
**Prevalence**	**98.1**	**96.1**	**88.8**	**64.6**	**49.6**	**37.7**	**26.2**	**22.5**	**17.0**	**13.9**	**13.0**	**11.8**	**8.9**	**5.9**	**2.1**	**1.8**
**Lower CI**	90.1	88.9	79.4	53.7	39.8	29.7	20.2	17.2	11.8	9.2	8.4	7.3	5.2	2.8	1.5	1.5
**Upper CI**	99.7	98.7	94.2	74.2	59.5	46.5	33.3	28.8	23.9	20.3	19.4	18.6	14.7	12.0	2.9	2.2
**Number**	124	121	115	87	69	61	48	42	37	31	28	25	19	10	3	2

Prevalence by age group, gender and occupational group for the worse ear over 0.25 kHz

Table W250-57 Age Group = 71-80 Gender = FEMALE Occupational group = MANUAL Total cases = 73

Threshold dB HL≥	0	5	10	15	20	25	30	35	40	45	50	55	65	75	85	95
**Prevalence**	**100.0**	**94.8**	**90.5**	**85.8**	**71.3**	**63.4**	**44.1**	**37.9**	**30.4**	**25.8**	**23.4**	**17.1**	**13.2**	**7.5**	**2.7**	**0.7**
**Lower CI**	95.0	74.9	69.8	68.1	56.9	47.0	29.3	25.6	19.0	15.2	13.0	10.4	7.0	2.9	1.1	0.2
**Upper CI**	100.0	99.1	97.5	94.5	82.3	77.2	59.9	51.9	44.7	40.4	38.4	26.7	23.2	18.1	6.5	2.5
**Number**	73	72	71	69	60	54	47	43	36	31	28	25	17	8	5	2

Table W250-58 Age Group = 18-30 Gender = MALE Occupational group = MANUAL Total cases = 109

Threshold dB HL≥	0	5	10	15	20	25	30	35	40	45	50	55	65	75	85	95
**Prevalence**	**93.3**	**76.8**	**59.4**	**35.7**	**18.5**	**11.1**	**8.3**	**4.9**	**3.4**	**1.8**	**0.7**	**0.7**	**0.3**	**0.1**	**0.1**	**0.1**
**Lower CI**	85.5	63.9	46.5	24.8	10.9	5.4	3.4	2.2	1.2	0.6	0.2	0.2	0.1	0.0	0.0	0.0
**Upper CI**	97.1	86.1	71.1	48.4	29.8	21.4	18.8	10.8	8.9	5.1	1.8	1.8	1.5	0.4	0.4	0.4
**Number**	102	90	75	49	28	17	13	11	8	6	4	4	2	1	1	1

Table W250-59 Age Group = 31-40 Gender = MALE Occupational group = MANUAL Total cases = 80

Threshold dB HL≥	0	5	10	15	20	25	30	35	40	45	50	55	65	75	85	95
**Prevalence**	**98.9**	**93.9**	**65.3**	**45.4**	**29.2**	**13.5**	**10.7**	**10.4**	**4.0**	**3.6**	**3.6**	**2.9**	**2.3**	**2.3**	**1.9**	**1.9**
**Lower CI**	95.9	83.5	51.4	31.0	18.3	6.9	4.8	4.5	1.5	1.3	1.3	0.8	0.5	0.5	0.3	0.3
**Upper CI**	99.7	97.9	77.0	60.7	43.0	24.7	22.4	22.1	10.2	10.1	10.1	9.8	9.9	9.9	10.2	10.2
**Number**	78	73	52	38	29	17	12	11	8	6	6	4	2	2	1	1

Table W250-60 Age Group = 41-50 Gender = MALE Occupational group = MANUAL Total cases = 126

Threshold dB HL≥	0	5	10	15	20	25	30	35	40	45	50	55	65	75	85	95
**Prevalence**	**99.5**	**92.0**	**73.6**	**59.4**	**36.6**	**24.5**	**17.3**	**11.5**	**8.0**	**7.1**	**4.6**	**2.9**	**2.2**	**0.7**	**0.5**	**0.2**
**Lower CI**	97.3	84.0	63.6	49.1	27.1	16.9	11.0	7.3	4.4	3.6	2.1	1.4	0.9	0.3	0.1	0.0
**Upper CI**	99.9	96.2	81.6	68.9	47.2	34.2	26.2	17.6	14.2	13.3	9.6	5.9	5.1	1.7	1.7	1.3
**Number**	125	116	94	77	54	40	30	24	17	14	11	9	6	3	2	1

Table W250-61 Age Group = 51-60 Gender = MALE Occupational group = MANUAL Total cases = 195

Threshold dB HL≥	0	5	10	15	20	25	30	35	40	45	50	55	65	75	85	95
**Prevalence**	**99.5**	**94.9**	**78.2**	**60.7**	**39.3**	**26.9**	**20.2**	**14.6**	**11.7**	**8.9**	**5.5**	**5.1**	**3.2**	**2.1**	**1.8**	**0.9**
**Lower CI**	98.2	89.6	70.1	52.0	31.9	21.1	15.7	10.8	8.4	6.0	3.5	3.1	1.6	0.9	0.7	0.3
**Upper CI**	99.9	97.5	84.6	68.8	47.2	33.6	25.6	19.3	16.1	12.9	8.6	8.2	6.0	4.9	4.6	2.8
**Number**	193	186	160	132	95	74	62	46	38	29	22	19	10	6	5	3

Table W250-62 Age Group = 61-70 Gender = MALE Occupational group = MANUAL Total cases = 169

Threshold dB HL≥	0	5	10	15	20	25	30	35	40	45	50	55	65	75	85	95
**Prevalence**	**99.8**	**91.7**	**87.2**	**70.1**	**49.5**	**31.3**	**23.3**	**17.4**	**13.3**	**10.1**	**8.4**	**7.4**	**3.5**	**2.3**	**1.1**	**0.6**
**Lower CI**	99.0	83.0	78.8	59.1	39.7	25.7	17.9	13.3	9.6	6.9	5.4	4.5	2.1	1.2	0.5	0.2
**Upper CI**	100.0	96.2	92.6	79.2	59.2	37.5	29.6	22.3	18.0	14.5	12.9	11.9	5.9	4.2	2.3	1.8
**Number**	168	159	152	130	107	86	66	55	43	34	27	23	15	11	6	3

Table W250-63 Age Group = 71-80 Gender = MALE Occupational group = MANUAL Total cases = 70

Threshold dB HL≥	0	5	10	15	20	25	30	35	40	45	50	55	65	75	85	95
**Prevalence**	**100.0**	**100.0**	**96.8**	**80.2**	**68.3**	**57.2**	**40.4**	**32.8**	**20.9**	**20.9**	**18.0**	**16.6**	**14.0**	**8.2**	**3.3**	**1.1**
**Lower CI**	94.8	94.8	94.2	61.6	51.1	42.6	27.1	20.1	14.0	14.0	11.4	10.4	8.2	3.8	1.9	0.3
**Upper CI**	100.0	100.0	98.3	91.1	81.7	70.5	55.4	48.5	30.2	30.2	27.3	25.5	23.0	16.8	5.5	4.0
**Number**	70	70	66	57	51	44	38	31	25	25	21	20	15	7	4	2

Prevalence by age group, gender and occupational group for the worse ear over 0.5 kHz

Table W500-1 Age Group = 18-80 Gender = OVERALL Occupational group = OVERALL Total cases = 2655

	Threshold dB HL≥															
	0	5	10	15	20	25	30	35	40	45	50	55	65	75	85	95
Prevalence	94.5	81.7	58.1	38.4	26.2	18.9	15.3	11.4	9.2	6.9	5.5	4.7	3.0	1.9	1.1	0.7
Lower CI	93.1	79.6	55.8	36.4	24.5	17.4	14.0	10.3	8.2	6.2	4.9	4.1	2.6	1.5	0.9	0.5
Upper CI	95.7	83.6	60.3	40.4	27.9	20.4	16.6	12.5	10.2	7.7	6.2	5.3	3.6	2.3	1.5	1.0
Number	2550	2304	1805	1334	1008	799	675	543	465	383	328	283	192	119	78	51

Table W500-2 Age Group = 18-80 Gender = FEMALE Occupational group = OVERALL Total cases = 1380

	Threshold dB HL≥															
	0	5	10	15	20	25	30	35	40	45	50	55	65	75	85	95
Prevalence	95.1	81.7	59.7	38.8	26.7	19.2	16.2	12.3	10.1	7.5	5.8	5.1	3.3	2.0	1.0	0.7
Lower CI	93.4	79.1	56.9	36.3	24.6	17.3	14.5	10.8	8.7	6.5	5.0	4.3	2.6	1.5	0.7	0.5
Upper CI	96.4	84.1	62.5	41.4	29.0	21.2	18.2	14.0	11.7	8.7	6.8	6.0	4.1	2.7	1.5	1.1
Number	1329	1191	942	684	519	411	355	293	250	209	181	159	107	62	37	27

Table W500-3 Age Group = 18-80 Gender = MALE Occupational group = OVERALL Total cases = 1275

	Threshold dB HL≥															
	0	5	10	15	20	25	30	35	40	45	50	55	65	75	85	95
Prevalence	93.9	81.7	56.2	37.8	25.5	18.5	14.2	10.3	8.1	6.2	5.1	4.3	2.7	1.8	1.2	0.7
Lower CI	91.5	78.4	52.7	34.7	23.0	16.4	12.4	8.9	6.9	5.2	4.3	3.5	2.1	1.3	0.8	0.4
Upper CI	95.7	84.6	59.7	41.1	28.3	20.8	16.2	12.0	9.4	7.3	6.1	5.2	3.5	2.5	1.8	1.3
Number	1221	1113	863	650	489	388	320	250	215	174	147	124	85	57	41	24

Table W500-4 Age Group = 18-30 Gender = OVERALL Occupational group = OVERALL Total cases = 401

	Threshold dB HL≥															
	0	5	10	15	20	25	30	35	40	45	50	55	65	75	85	95
Prevalence	88.9	70.0	38.5	17.9	9.6	6.2	4.9	3.1	2.1	1.6	0.9	0.6	0.2	0.0	0.0	0.0
Lower CI	84.3	64.4	33.1	14.0	7.0	4.1	3.2	2.0	1.3	0.9	0.5	0.3	0.1	0.0	0.0	0.0
Upper CI	92.3	75.1	44.1	22.5	13.1	9.2	7.4	4.7	3.4	2.7	1.7	1.2	0.6	0.1	0.1	0.1
Number	366	298	179	97	62	43	38	28	22	17	11	8	3	1	1	1

Table W500-5 Age Group = 31-40 Gender = OVERALL Occupational group = OVERALL Total cases = 331

	Threshold dB HL≥															
	0	5	10	15	20	25	30	35	40	45	50	55	65	75	85	95
Prevalence	94.4	75.2	46.5	27.6	15.0	9.5	7.7	5.8	3.8	3.0	2.3	2.2	1.5	1.0	0.8	0.7
Lower CI	90.8	69.2	40.3	22.3	11.2	6.7	5.2	3.7	2.3	1.6	1.2	1.2	0.6	0.3	0.2	0.2
Upper CI	96.6	80.5	52.7	33.5	19.9	13.3	11.3	9.1	6.4	5.3	4.2	4.1	3.4	3.1	3.0	3.0
Number	311	258	185	114	69	49	39	28	22	18	15	14	9	4	3	2

Table W500-6 Age Group = 41-50 Gender = OVERALL Occupational group = OVERALL Total cases = 448

	Threshold dB HL≥															
	0	5	10	15	20	25	30	35	40	45	50	55	65	75	85	95
Prevalence	95.5	81.7	56.7	36.6	24.0	15.9	12.0	9.7	8.1	5.9	5.7	4.6	2.5	0.8	0.7	0.6
Lower CI	93.0	77.5	51.8	32.1	20.2	12.9	9.4	7.4	6.1	4.3	4.1	3.2	1.6	0.4	0.3	0.3
Upper CI	97.2	85.4	61.6	41.3	28.1	19.5	15.1	12.5	10.6	8.1	7.8	6.5	3.8	1.5	1.4	1.3
Number	429	375	276	191	133	98	78	66	58	45	43	36	23	9	8	7

Table W500-7 Age Group = 51-60 Gender = OVERALL Occupational group = OVERALL Total cases = 679

	Threshold dB HL≥															
	0	5	10	15	20	25	30	35	40	45	50	55	65	75	85	95
Prevalence	98.3	89.6	68.1	44.5	31.5	22.7	17.3	12.9	10.9	8.1	6.6	5.4	3.5	2.3	1.4	0.9
Lower CI	96.8	86.6	64.0	40.5	28.0	19.7	14.8	10.8	8.9	6.5	5.2	4.2	2.5	1.5	0.8	0.4
Upper CI	99.1	92.0	71.9	48.5	35.2	25.9	20.2	15.5	13.3	10.1	8.4	7.1	4.9	3.5	2.4	1.8
Number	666	622	496	360	278	219	176	142	120	96	83	70	47	31	20	12

Prevalence by age group, gender and occupational group for the worse ear over 0.5 kHz

Table W500-8 Age Group = 61-70 Gender = OVERALL Occupational group = OVERALL Total cases = 526

	Threshold dB HL≥															
	0	5	10	15	20	25	30	35	40	45	50	55	65	75	85	95
Prevalence	97.3	92.4	78.0	61.0	44.1	33.1	27.8	19.7	15.5	12.0	9.6	8.0	5.9	4.3	2.1	1.5
Lower CI	95.2	89.1	73.5	55.8	39.5	28.6	23.8	16.6	12.9	9.8	7.6	6.2	4.3	2.8	1.4	1.0
Upper CI	98.5	94.8	81.9	65.9	48.9	37.9	32.2	23.3	18.4	14.6	11.9	10.3	8.2	6.5	2.9	2.2
Number	512	490	423	351	274	226	199	157	134	116	97	82	62	42	26	18

Table W500-9 Age Group = 71-80 Gender = OVERALL Occupational group = OVERALL Total cases = 270

	Threshold dB HL≥															
	0	5	10	15	20	25	30	35	40	45	50	55	65	75	85	95
Prevalence	98.5	96.8	91.1	75.8	61.8	49.0	42.1	33.3	28.8	22.0	16.2	15.1	10.0	6.7	3.5	1.6
Lower CI	96.1	93.6	86.2	68.9	53.8	41.7	35.2	26.8	22.7	17.4	12.9	11.8	7.3	4.6	2.2	0.9
Upper CI	99.4	98.4	94.3	81.6	69.2	56.4	49.4	40.4	35.9	27.4	20.3	19.1	13.6	9.9	5.5	2.9
Number	266	261	246	221	192	164	145	122	109	91	79	73	48	32	20	11

Table W500-10 Age Group = 18-80 Gender = OVERALL Occupational group = NON-MANUAL Total cases = 1183

	Threshold dB HL≥															
	0	5	10	15	20	25	30	35	40	45	50	55	65	75	85	95
Prevalence	92.3	76.7	52.0	33.6	22.5	15.7	13.3	9.6	8.0	5.4	4.0	3.4	2.0	1.4	0.9	0.6
Lower CI	89.9	73.4	48.8	30.8	20.2	14.0	11.7	8.3	6.8	4.5	3.3	2.8	1.6	1.1	0.6	0.4
Upper CI	94.2	79.6	55.2	36.5	24.9	17.6	15.1	10.9	9.3	6.6	4.9	4.2	2.6	2.0	1.3	1.0
Number	1116	974	714	504	368	286	242	194	164	132	111	99	64	46	30	21

Table W500-11 Age Group = 18-80 Gender = OVERALL Occupational group = MANUAL Total cases = 1467

	Threshold dB HL≥															
	0	5	10	15	20	25	30	35	40	45	50	55	65	75	85	95
Prevalence	96.2	86.1	63.5	42.3	29.7	21.8	16.9	12.8	10.0	7.8	6.3	5.4	3.5	2.0	1.2	0.8
Lower CI	94.1	83.2	60.1	39.2	27.2	19.6	15.0	11.2	8.8	6.8	5.4	4.5	2.9	1.5	0.9	0.5
Upper CI	97.6	88.5	66.8	45.4	32.3	24.1	19.0	14.5	11.3	9.1	7.4	6.3	4.3	2.6	1.7	1.2
Number	1429	1326	1088	827	638	512	432	348	301	251	217	184	128	73	48	30

Table W500-12 Age Group = 18-30 Gender = FEMALE Occupational group = OVERALL Total cases = 224

	Threshold dB HL≥															
	0	5	10	15	20	25	30	35	40	45	50	55	65	75	85	95
Prevalence	91.4	69.3	37.4	16.6	9.2	5.6	5.3	4.3	3.3	2.3	1.2	0.7	0.0	0.0	0.0	0.0
Lower CI	85.9	62.0	30.7	12.2	6.3	3.6	3.3	2.6	1.9	1.2	0.6	0.2	0.0	0.0	0.0	0.0
Upper CI	94.8	75.7	44.6	22.3	13.1	8.7	8.4	7.0	5.7	4.3	2.6	1.8	1.7	1.7	1.7	1.7
Number	208	166	96	52	34	23	22	18	15	11	7	4	0	0	0	0

Table W500-13 Age Group = 31-40 Gender = FEMALE Occupational group = OVERALL Total cases = 189

	Threshold dB HL≥															
	0	5	10	15	20	25	30	35	40	45	50	55	65	75	85	95
Prevalence	93.1	71.7	49.2	26.9	13.1	8.7	7.3	4.4	3.0	2.2	2.2	2.1	1.5	0.5	0.5	0.3
Lower CI	87.2	63.4	41.2	20.5	9.0	5.4	4.2	2.5	1.6	1.2	1.2	1.1	0.7	0.1	0.1	0.1
Upper CI	96.4	78.7	57.2	34.4	18.7	13.7	12.1	7.9	5.8	4.2	4.2	4.1	3.2	1.9	1.9	1.7
Number	178	144	109	66	39	26	21	15	12	10	10	9	7	2	2	1

Table W500-14 Age Group = 41-50 Gender = FEMALE Occupational group = OVERALL Total cases = 245

	Threshold dB HL≥															
	0	5	10	15	20	25	30	35	40	45	50	55	65	75	85	95
Prevalence	95.1	80.9	54.6	34.7	21.8	14.1	10.1	8.4	6.3	4.5	4.5	4.5	2.4	0.8	0.6	0.6
Lower CI	91.3	74.9	48.0	29.0	17.1	10.5	7.1	5.8	4.2	2.8	2.8	2.8	1.3	0.3	0.2	0.2
Upper CI	97.2	85.7	61.0	41.0	27.4	18.7	14.0	12.2	9.4	7.0	7.0	7.0	4.3	1.8	1.5	1.5
Number	234	203	146	98	66	48	37	32	26	20	20	20	12	5	4	4

Prevalence by age group, gender and occupational group for the worse ear over 0.5 kHz

Table W500-15 Age Group = 51-60 Gender = FEMALE Occupational group = OVERALL Total cases = 340

Threshold dB HL≥

	0	5	10	15	20	25	30	35	40	45	50	55	65	75	85	95
**Prevalence**	**98.9**	**90.5**	**70.7**	**44.3**	**32.4**	**23.7**	**18.3**	**13.4**	**10.5**	**7.5**	**6.3**	**4.9**	**3.1**	**1.8**	**1.3**	**1.1**
**Lower CI**	97.1	86.2	65.1	38.9	27.6	19.6	14.7	10.3	7.7	5.4	4.4	3.3	1.9	1.0	0.5	0.4
**Upper CI**	99.6	93.5	75.8	49.8	37.6	28.5	22.5	17.3	14.0	10.4	8.9	7.1	5.2	3.5	3.0	3.0
**Number**	335	313	258	183	144	114	91	73	59	47	42	35	23	15	9	7

Table W500-16 Age Group = 61-70 Gender = FEMALE Occupational group = OVERALL Total cases = 241

Threshold dB HL≥

	0	5	10	15	20	25	30	35	40	45	50	55	65	75	85	95
**Prevalence**	**96.9**	**93.7**	**78.3**	**61.0**	**44.7**	**32.7**	**28.2**	**19.7**	**16.9**	**13.0**	**10.1**	**8.8**	**7.1**	**5.1**	**1.9**	**1.7**
**Lower CI**	93.0	89.2	71.7	54.1	38.5	26.5	22.5	15.4	12.9	9.6	7.3	6.1	4.5	2.8	1.1	0.9
**Upper CI**	98.7	96.3	83.7	67.4	51.1	39.7	34.6	24.9	21.7	17.2	13.9	12.5	11.1	9.2	3.2	2.9
**Number**	235	227	198	162	126	104	95	77	68	62	52	45	34	21	11	9

Table W500-17 Age Group = 71-80 Gender = FEMALE Occupational group = OVERALL Total cases = 141

Threshold dB HL≥

	0	5	10	15	20	25	30	35	40	45	50	55	65	75	85	95
**Prevalence**	**99.2**	**99.0**	**97.0**	**81.0**	**66.5**	**50.9**	**46.3**	**38.4**	**34.3**	**26.7**	**18.1**	**16.9**	**11.0**	**7.5**	**3.5**	**1.6**
**Lower CI**	96.9	96.9	91.7	71.7	54.9	40.9	36.7	29.3	25.2	19.9	13.5	12.3	7.5	4.5	1.9	0.7
**Upper CI**	99.8	99.7	99.0	87.8	76.4	60.8	56.2	48.5	44.6	34.8	24.0	22.8	16.0	12.2	6.3	3.7
**Number**	139	138	135	123	110	96	89	78	70	59	50	46	31	19	11	6

Table W500-18 Age Group = 18-30 Gender = MALE Occupational group = OVERALL Total cases = 177

Threshold dB HL≥

	0	5	10	15	20	25	30	35	40	45	50	55	65	75	85	95
**Prevalence**	**86.2**	**70.9**	**39.5**	**19.1**	**10.0**	**6.7**	**4.3**	**1.7**	**0.8**	**0.7**	**0.5**	**0.5**	**0.4**	**0.0**	**0.0**	**0.0**
**Lower CI**	78.2	62.0	31.2	13.3	6.0	3.5	2.0	0.8	0.4	0.3	0.2	0.2	0.1	0.0	0.0	0.0
**Upper CI**	91.6	78.4	48.4	26.8	16.3	12.5	9.0	3.6	1.8	1.6	1.4	1.4	1.2	0.2	0.2	0.2
**Number**	158	132	83	45	28	20	16	10	7	6	4	4	3	1	1	1

Table W500-19 Age Group = 31-40 Gender = MALE Occupational group = OVERALL Total cases = 142

Threshold dB HL≥

	0	5	10	15	20	25	30	35	40	45	50	55	65	75	85	95
**Prevalence**	**95.6**	**78.9**	**43.4**	**28.5**	**17.4**	**10.7**	**8.4**	**7.5**	**4.7**	**3.9**	**2.4**	**2.4**	**1.5**	**1.5**	**1.2**	**1.2**
**Lower CI**	90.4	69.3	34.0	20.3	11.1	6.4	4.7	3.9	2.2	1.6	0.8	0.8	0.3	0.3	0.2	0.2
**Upper CI**	98.1	86.0	53.3	38.3	26.0	17.3	14.7	13.9	9.7	8.9	6.5	6.5	6.3	6.3	6.5	6.5
**Number**	133	114	76	48	30	23	18	13	10	8	5	5	2	2	1	1

Table W500-20 Age Group = 41-50 Gender = MALE Occupational group = OVERALL Total cases = 203

Threshold dB HL≥

	0	5	10	15	20	25	30	35	40	45	50	55	65	75	85	95
**Prevalence**	**96.0**	**82.6**	**59.0**	**38.6**	**26.3**	**17.9**	**14.1**	**11.0**	**9.9**	**7.5**	**7.0**	**4.7**	**2.5**	**0.8**	**0.8**	**0.6**
**Lower CI**	92.1	76.0	51.5	31.9	20.7	13.2	10.1	7.6	6.8	4.9	4.4	2.7	1.4	0.3	0.3	0.2
**Upper CI**	98.0	87.7	66.1	45.8	32.7	23.7	19.3	15.6	14.3	11.4	10.8	8.0	4.7	2.2	2.2	1.8
**Number**	195	172	130	93	67	50	41	34	32	25	23	16	11	4	4	3

Table W500-21 Age Group = 51-60 Gender = MALE Occupational group = OVERALL Total cases = 339

Threshold dB HL≥

	0	5	10	15	20	25	30	35	40	45	50	55	65	75	85	95
**Prevalence**	**97.6**	**88.5**	**65.1**	**44.6**	**30.5**	**21.4**	**16.3**	**12.5**	**11.4**	**8.8**	**7.0**	**6.1**	**3.9**	**2.7**	**1.5**	**0.6**
**Lower CI**	94.8	83.9	59.0	38.9	25.6	17.5	13.1	9.7	8.7	6.5	5.0	4.2	2.5	1.5	0.7	0.2
**Upper CI**	98.9	92.0	70.7	50.4	35.8	25.9	20.1	15.9	14.8	11.9	9.8	8.8	6.1	4.8	2.9	1.4
**Number**	331	309	238	177	134	105	85	69	61	49	41	35	24	16	11	5

Prevalence by age group, gender and occupational group for the worse ear over 0.5 kHz

Table W500-22 Age Group = 61-70 Gender = MALE Occupational group = OVERALL Total cases = 285

Threshold dB HL≥	0	5	10	15	20	25	30	35	40	45	50	55	65	75	85	95
Prevalence	**97.9**	**91.0**	**77.7**	**60.9**	**43.4**	**33.5**	**27.5**	**19.7**	**13.9**	**10.8**	**8.9**	**7.0**	**4.5**	**3.3**	**2.2**	**1.2**
Lower CI	95.7	85.4	71.4	53.0	36.4	27.4	22.1	15.5	11.0	8.4	6.6	5.1	3.2	2.2	1.4	0.7
Upper CI	99.0	94.6	83.0	68.2	50.6	40.2	33.7	24.7	17.3	13.9	11.8	9.6	6.3	5.0	3.6	2.3
Number	277	263	225	189	148	122	104	80	66	54	45	37	28	21	15	9

Table W500-23 Age Group = 71-80 Gender = MALE Occupational group = OVERALL Total cases = 129

Threshold dB HL≥	0	5	10	15	20	25	30	35	40	45	50	55	65	75	85	95
Prevalence	**97.3**	**93.2**	**81.8**	**67.5**	**54.3**	**46.0**	**35.7**	**25.6**	**20.6**	**14.7**	**13.6**	**12.5**	**8.4**	**5.6**	**3.6**	**1.6**
Lower CI	91.3	85.1	71.2	56.8	44.2	36.0	26.8	17.9	14.3	10.5	9.5	8.6	5.0	3.2	1.9	0.7
Upper CI	99.2	97.0	89.1	76.7	64.1	56.4	45.7	35.2	28.7	20.3	19.3	18.0	13.8	9.6	6.9	3.5
Number	127	123	111	98	82	68	56	44	39	32	29	27	17	13	9	5

Table W500-24 Age Group = 18-30 Gender = OVERALL Occupational group = NON-MANUAL Total cases = 181

Threshold dB HL≥	0	5	10	15	20	25	30	35	40	45	50	55	65	75	85	95
Prevalence	**84.4**	**60.0**	**31.4**	**12.8**	**6.3**	**2.8**	**2.3**	**1.8**	**1.8**	**1.2**	**0.4**	**0.4**	**0.0**	**0.0**	**0.0**	**0.0**
Lower CI	76.6	51.4	23.9	8.1	3.3	1.6	1.3	0.9	0.9	0.6	0.2	0.2	0.0	0.0	0.0	0.0
Upper CI	90.0	68.0	40.0	19.5	11.8	4.9	4.1	3.6	3.6	2.8	0.8	0.8	2.1	2.1	2.1	2.1
Number	159	118	67	29	17	12	11	9	9	6	2	2	0	0	0	0

Table W500-25 Age Group = 31-40 Gender = OVERALL Occupational group = NON-MANUAL Total cases = 148

Threshold dB HL≥	0	5	10	15	20	25	30	35	40	45	50	55	65	75	85	95
Prevalence	**91.7**	**71.9**	**42.8**	**25.4**	**12.3**	**8.1**	**7.0**	**4.5**	**4.5**	**2.5**	**1.0**	**0.9**	**0.6**	**0.3**	**0.3**	**0.3**
Lower CI	85.9	62.6	35.1	18.6	7.6	4.4	3.6	1.9	1.9	0.8	0.4	0.3	0.2	0.1	0.1	0.1
Upper CI	95.3	79.6	50.9	33.5	19.1	14.5	13.1	10.0	10.0	7.8	2.7	2.7	2.3	1.7	1.7	1.7
Number	136	110	74	42	22	15	13	9	9	5	4	3	2	1	1	1

Table W500-26 Age Group = 41-50 Gender = OVERALL Occupational group = NON-MANUAL Total cases = 187

Threshold dB HL≥	0	5	10	15	20	25	30	35	40	45	50	55	65	75	85	95
Prevalence	**94.7**	**79.2**	**49.7**	**31.9**	**20.3**	**12.4**	**11.3**	**7.8**	**7.1**	**4.9**	**4.2**	**2.8**	**1.0**	**0.5**	**0.5**	**0.5**
Lower CI	90.0	72.1	42.2	25.6	15.4	8.4	7.4	4.7	4.2	2.6	2.0	1.1	0.5	0.1	0.1	0.1
Upper CI	97.3	84.9	57.2	38.9	26.2	18.0	17.0	12.7	11.8	9.2	8.8	7.1	2.0	1.9	1.9	1.9
Number	176	148	95	63	38	25	22	16	14	10	9	7	5	2	2	2

Table W500-27 Age Group = 51-60 Gender = OVERALL Occupational group = NON-MANUAL Total cases = 316

Threshold dB HL≥	0	5	10	15	20	25	30	35	40	45	50	55	65	75	85	95
Prevalence	**97.5**	**85.1**	**58.8**	**37.7**	**24.0**	**17.5**	**13.1**	**9.0**	**7.1**	**5.5**	**5.3**	**4.7**	**3.0**	**2.2**	**1.4**	**1.1**
Lower CI	95.0	79.3	53.0	32.3	19.9	14.3	10.4	7.0	5.2	3.8	3.7	3.1	1.8	1.2	0.7	0.4
Upper CI	98.8	89.4	64.4	43.4	28.6	21.3	16.3	11.6	9.5	7.9	7.7	7.0	5.0	4.0	3.0	2.7
Number	307	275	201	144	106	85	67	55	44	34	32	29	19	15	10	7

Table W500-28 Age Group = 61-70 Gender = OVERALL Occupational group = NON-MANUAL Total cases = 228

Threshold dB HL≥	0	5	10	15	20	25	30	35	40	45	50	55	65	75	85	95
Prevalence	**96.1**	**90.0**	**75.7**	**56.8**	**42.3**	**29.8**	**26.7**	**17.9**	**14.2**	**11.4**	**7.6**	**6.5**	**4.1**	**3.2**	**1.9**	**1.6**
Lower CI	92.2	84.4	69.1	48.5	34.7	23.1	20.3	14.2	11.2	8.7	5.6	4.6	2.7	2.0	1.1	0.9
Upper CI	98.1	93.7	81.3	64.7	50.2	37.5	34.2	22.1	17.7	14.6	10.3	9.0	6.0	5.1	3.3	2.9
Number	219	207	172	134	106	84	75	61	51	45	37	33	22	16	11	9

Prevalence by age group, gender and occupational group for the worse ear over 0.5 kHz

Table W500-29 Age Group = 71-80 Gender = OVERALL Occupational group = NON-MANUAL Total cases = 123

Threshold dB HL≥	0	5	10	15	20	25	30	35	40	45	50	55	65	75	85	95
Prevalence	97.3	95.4	86.8	70.8	59.9	47.9	40.1	31.8	25.4	14.8	10.9	10.1	6.9	5.4	2.4	1.0
Lower CI	93.2	91.2	79.2	60.0	48.7	39.4	32.7	25.8	19.9	10.1	7.8	7.2	4.1	2.9	1.1	0.2
Upper CI	99.0	97.7	91.9	79.6	70.2	56.6	48.0	38.5	31.9	21.1	15.0	14.0	11.4	9.8	5.5	4.0
Number	119	116	105	92	79	65	54	44	37	32	27	25	16	12	6	2

Table W500-30 Age Group = 18-30 Gender = OVERALL Occupational group = MANUAL Total cases = 218

Threshold dB HL≥	0	5	10	15	20	25	30	35	40	45	50	55	65	75	85	95
Prevalence	92.2	79.8	46.3	22.7	12.4	9.1	7.4	4.1	2.4	1.7	1.1	0.7	0.2	0.0	0.0	0.0
Lower CI	85.0	72.0	37.9	16.2	8.1	5.4	4.2	2.4	1.1	0.8	0.5	0.3	0.1	0.0	0.0	0.0
Upper CI	96.1	85.9	54.8	30.9	18.4	14.8	12.8	7.2	4.9	3.7	2.1	1.6	0.8	0.2	0.2	0.2
Number	205	179	112	68	45	31	27	19	13	11	9	6	3	1	1	1

Table W500-31 Age Group = 31-40 Gender = OVERALL Occupational group = MANUAL Total cases = 182

Threshold dB HL≥	0	5	10	15	20	25	30	35	40	45	50	55	65	75	85	95
Prevalence	96.7	78.9	49.8	28.3	17.0	10.0	7.8	6.8	3.2	3.2	2.9	2.9	2.0	1.3	1.1	0.9
Lower CI	91.4	69.9	40.7	21.1	11.6	6.2	4.5	3.7	1.6	1.6	1.4	1.4	0.8	0.3	0.2	0.2
Upper CI	98.8	85.8	59.0	36.7	24.2	15.7	13.2	12.3	6.3	6.3	6.0	6.0	5.2	4.8	4.9	5.0
Number	174	147	110	71	46	33	25	18	13	13	11	11	7	3	2	1

Table W500-32 Age Group = 41-50 Gender = OVERALL Occupational group = MANUAL Total cases = 261

Threshold dB HL≥	0	5	10	15	20	25	30	35	40	45	50	55	65	75	85	95
Prevalence	95.7	83.0	63.7	42.0	29.2	20.2	14.1	11.8	9.5	7.5	7.3	6.0	3.1	0.9	0.8	0.6
Lower CI	91.5	76.4	56.5	35.3	23.4	15.3	10.3	8.5	6.8	5.1	4.9	3.9	2.0	0.4	0.3	0.3
Upper CI	97.9	88.0	70.3	49.0	35.7	26.2	19.1	16.2	13.1	11.0	10.8	9.0	4.8	1.9	1.7	1.5
Number	253	227	181	128	95	73	56	50	44	35	34	29	18	7	6	5

Table W500-33 Age Group = 51-60 Gender = OVERALL Occupational group = MANUAL Total cases = 363

Threshold dB HL≥	0	5	10	15	20	25	30	35	40	45	50	55	65	75	85	95
Prevalence	99.1	93.4	75.8	49.3	37.3	26.4	20.0	15.0	13.2	9.6	7.4	5.5	3.3	1.8	0.9	0.4
Lower CI	97.0	89.0	69.8	43.3	31.9	21.8	16.1	11.7	10.2	7.1	5.3	3.8	2.1	1.0	0.5	0.2
Upper CI	99.7	96.1	81.0	55.3	43.1	31.5	24.6	19.0	17.0	12.9	10.2	7.7	5.0	3.1	1.8	1.1
Number	359	347	295	216	172	134	109	87	76	62	51	41	28	16	10	5

Table W500-34 Age Group = 61-70 Gender = OVERALL Occupational group = MANUAL Total cases = 297

Threshold dB HL≥	0	5	10	15	20	25	30	35	40	45	50	55	65	75	85	95
Prevalence	97.6	93.3	78.4	62.8	45.9	35.4	28.4	20.9	16.6	12.6	10.8	9.1	7.5	5.1	2.4	1.7
Lower CI	93.5	87.9	71.0	55.0	39.3	29.5	23.3	16.8	13.8	9.7	8.0	6.4	5.0	3.0	1.7	1.2
Upper CI	99.2	96.4	84.3	70.0	52.6	41.8	34.1	25.6	19.9	16.3	14.6	12.8	11.0	8.5	3.4	2.5
Number	292	282	250	216	167	142	124	96	83	71	60	49	40	26	15	9

Table W500-35 Age Group = 71-80 Gender = OVERALL Occupational group = MANUAL Total cases = 146

Threshold dB HL≥	0	5	10	15	20	25	30	35	40	45	50	55	65	75	85	95
Prevalence	100.0	97.5	95.0	83.4	68.3	56.5	44.6	36.6	31.7	26.6	18.7	17.7	11.6	7.1	4.4	2.0
Lower CI	97.4	88.1	86.2	72.9	57.0	47.0	35.4	27.7	23.9	19.1	13.6	12.7	8.0	4.7	2.7	1.0
Upper CI	100.0	99.5	98.3	90.3	77.8	65.6	54.2	46.7	40.8	35.6	25.1	24.2	16.4	10.6	7.1	3.6
Number	146	144	140	128	113	99	91	78	72	59	52	48	32	20	14	9

Prevalence by age group, gender and occupational group for the worse ear over 0.5 kHz

Table W500-36 Age Group = 18-80 Gender = FEMALE Occupational group = NON-MANUAL Total cases = 669

Threshold dB HL≥	0	5	10	15	20	25	30	35	40	45	50	55	65	75	85	95
**Prevalence**	**92.8**	**77.6**	**54.7**	**36.0**	**24.1**	**17.8**	**15.7**	**11.1**	**9.2**	**5.7**	**4.3**	**4.0**	**2.5**	**1.6**	**1.0**	**0.8**
**Lower CI**	90.1	73.9	50.8	32.5	21.2	15.6	13.6	9.6	7.8	4.6	3.5	3.2	1.8	1.1	0.6	0.5
**Upper CI**	94.9	81.0	58.5	39.7	27.2	20.3	18.0	12.9	10.9	7.1	5.2	4.9	3.4	2.4	1.8	1.5
**Number**	630	550	412	293	214	171	150	122	104	86	73	66	42	28	18	14

Table W500-37 Age Group = 18-80 Gender = MALE Occupational group = NON-MANUAL Total cases = 514

Threshold dB HL≥	0	5	10	15	20	25	30	35	40	45	50	55	65	75	85	95
**Prevalence**	**91.8**	**75.7**	**49.1**	**30.8**	**20.6**	**13.4**	**10.6**	**7.8**	**6.6**	**5.1**	**3.7**	**2.8**	**1.4**	**1.3**	**0.7**	**0.4**
**Lower CI**	87.3	70.0	44.0	26.5	17.1	10.9	8.3	6.0	5.0	3.6	2.6	1.8	1.0	0.8	0.4	0.2
**Upper CI**	94.7	80.5	54.3	35.4	24.6	16.5	13.4	10.0	8.7	7.0	5.3	4.4	2.2	2.0	1.3	0.8
**Number**	486	424	302	211	154	115	92	72	60	46	38	33	22	18	12	7

Table W500-38 Age Group = 18-80 Gender = FEMALE Occupational group = MANUAL Total cases = 709

Threshold dB HL≥	0	5	10	15	20	25	30	35	40	45	50	55	65	75	85	95
**Prevalence**	**97.2**	**86.7**	**65.2**	**41.3**	**29.5**	**21.1**	**16.9**	**13.4**	**11.0**	**8.5**	**6.6**	**5.5**	**3.6**	**1.9**	**1.0**	**0.7**
**Lower CI**	94.2	82.8	60.8	37.4	26.2	18.4	14.5	11.3	9.1	6.9	5.3	4.4	2.8	1.4	0.7	0.5
**Upper CI**	98.6	89.8	69.5	45.4	33.0	24.0	19.7	15.7	13.1	10.5	8.1	7.0	4.6	2.8	1.4	1.0
**Number**	697	639	528	389	303	239	204	170	146	123	108	93	65	34	19	13

Table W500-39 Age Group = 18-80 Gender = MALE Occupational group = MANUAL Total cases = 758

Threshold dB HL≥	0	5	10	15	20	25	30	35	40	45	50	55	65	75	85	95
**Prevalence**	**95.1**	**85.4**	**61.6**	**43.3**	**29.9**	**22.5**	**16.9**	**12.1**	**8.9**	**7.1**	**6.1**	**5.2**	**3.4**	**2.1**	**1.5**	**0.9**
**Lower CI**	91.7	80.8	56.4	38.6	26.2	19.2	14.1	9.9	7.5	5.8	4.9	4.0	2.5	1.4	0.9	0.4
**Upper CI**	97.2	89.0	66.5	48.2	33.9	26.2	20.2	14.7	10.5	8.6	7.7	6.6	4.6	3.1	2.4	1.8
**Number**	732	687	560	438	335	273	228	178	155	128	109	91	63	39	29	17

Table W500-40 Age Group = 18-30 Gender = FEMALE Occupational group = NON-MANUAL Total cases = 115

Threshold dB HL≥	0	5	10	15	20	25	30	35	40	45	50	55	65	75	85	95
**Prevalence**	**88.7**	**63.2**	**32.5**	**13.9**	**7.1**	**5.0**	**4.1**	**3.1**	**3.1**	**2.2**	**0.8**	**0.8**	**0.0**	**0.0**	**0.0**	**0.0**
**Lower CI**	81.0	53.5	24.3	8.8	4.1	2.7	2.3	1.5	1.5	1.0	0.4	0.4	0.0	0.0	0.0	0.0
**Upper CI**	93.5	71.8	41.9	21.3	12.0	9.0	7.4	6.5	6.5	5.1	1.5	1.5	3.2	3.2	3.2	3.2
**Number**	103	77	43	20	13	10	9	7	7	5	2	2	0	0	0	0

Table W500-41 Age Group = 31-40 Gender = FEMALE Occupational group = NON-MANUAL Total cases = 86

Threshold dB HL≥	0	5	10	15	20	25	30	35	40	45	50	55	65	75	85	95
**Prevalence**	**88.2**	**64.4**	**43.0**	**25.6**	**10.9**	**8.0**	**7.6**	**3.3**	**3.3**	**1.4**	**1.4**	**1.2**	**1.2**	**0.6**	**0.6**	**0.6**
**Lower CI**	78.3	52.5	32.3	17.1	5.9	3.7	3.4	1.2	1.2	0.4	0.4	0.3	0.3	0.1	0.1	0.1
**Upper CI**	93.9	74.7	54.3	36.4	19.3	16.4	16.3	9.0	9.0	4.4	4.4	4.5	4.5	3.2	3.2	3.2
**Number**	78	59	42	25	13	9	8	5	5	3	3	2	2	1	1	1

Table W500-42 Age Group = 41-50 Gender = FEMALE Occupational group = NON-MANUAL Total cases = 111

Threshold dB HL≥	0	5	10	15	20	25	30	35	40	45	50	55	65	75	85	95
**Prevalence**	**92.8**	**77.9**	**46.4**	**33.2**	**17.1**	**11.4**	**9.4**	**6.5**	**5.1**	**1.6**	**1.6**	**1.6**	**0.6**	**0.0**	**0.0**	**0.0**
**Lower CI**	86.5	69.2	37.6	25.2	11.8	7.5	5.6	3.1	2.3	0.5	0.5	0.5	0.1	0.0	0.0	0.0
**Upper CI**	96.3	84.6	55.5	42.2	24.2	17.1	15.3	12.9	11.0	5.3	5.3	5.3	4.4	3.3	3.3	3.3
**Number**	102	87	55	38	20	13	10	7	5	2	2	2	1	0	0	0

Prevalence by age group, gender and occupational group for the worse ear over 0.5 kHz

Table W500-43 Age Group = 51-60 Gender = FEMALE Occupational group = NON-MANUAL Total cases = 174

	Threshold dB HL≥															
	**0**	**5**	**10**	**15**	**20**	**25**	**30**	**35**	**40**	**45**	**50**	**55**	**65**	**75**	**85**	**95**
**Prevalence**	**98.2**	**88.6**	**65.5**	**40.1**	**27.4**	**19.7**	**16.0**	**10.5**	**8.0**	**6.2**	**6.0**	**5.3**	**3.7**	**2.5**	**1.9**	**1.7**
**Lower CI**	95.4	82.5	57.6	32.8	21.4	15.1	11.9	7.5	5.5	3.9	3.8	3.2	1.9	1.1	0.7	0.6
**Upper CI**	99.3	92.7	72.6	47.9	34.3	25.3	21.2	14.3	11.4	9.7	9.5	8.8	6.9	5.5	4.9	4.9
**Number**	170	154	118	81	62	49	41	34	27	21	20	18	12	9	6	5

Table W500-44 Age Group = 61-70 Gender = FEMALE Occupational group = NON-MANUAL Total cases = 116

	Threshold dB HL≥															
	**0**	**5**	**10**	**15**	**20**	**25**	**30**	**35**	**40**	**45**	**50**	**55**	**65**	**75**	**85**	**95**
**Prevalence**	**95.9**	**92.0**	**77.1**	**58.4**	**41.5**	**27.7**	**25.3**	**15.8**	**14.0**	**10.4**	**7.0**	**6.2**	**4.1**	**2.7**	**1.8**	**1.8**
**Lower CI**	88.9	84.5	67.9	49.0	33.0	20.5	18.6	11.4	9.8	7.2	5.7	4.8	2.7	1.6	0.8	0.8
**Upper CI**	98.6	96.1	84.4	67.2	50.5	36.2	33.5	21.5	19.4	14.9	8.7	7.9	6.1	4.7	3.8	3.8
**Number**	112	109	93	75	56	46	43	34	31	28	24	21	14	9	6	6

Table W500-45 Age Group = 71-80 Gender = FEMALE Occupational group = NON-MANUAL Total cases = 67

	Threshold dB HL≥															
	**0**	**5**	**10**	**15**	**20**	**25**	**30**	**35**	**40**	**45**	**50**	**55**	**65**	**75**	**85**	**95**
**Prevalence**	**98.2**	**97.9**	**94.4**	**77.5**	**69.9**	**57.2**	**52.0**	**44.2**	**34.7**	**20.5**	**14.2**	**13.9**	**9.4**	**6.9**	**3.5**	**1.7**
**Lower CI**	92.4	92.6	84.8	61.3	52.6	45.5	41.9	35.8	26.9	13.4	9.7	9.4	5.2	3.3	1.4	0.4
**Upper CI**	99.6	99.4	98.0	88.2	83.0	68.1	61.9	53.0	43.6	30.1	20.4	20.1	16.4	13.8	8.5	6.8
**Number**	65	64	61	54	50	44	39	35	29	27	22	21	13	9	5	2

Table W500-46 Age Group = 18-30 Gender = MALE Occupational group = NON-MANUAL Total cases = 66

	Threshold dB HL≥															
	**0**	**5**	**10**	**15**	**20**	**25**	**30**	**35**	**40**	**45**	**50**	**55**	**65**	**75**	**85**	**95**
**Prevalence**	**79.9**	**56.8**	**30.2**	**11.4**	**5.2**	**0.4**	**0.4**	**0.4**	**0.4**	**0.1**	**0.0**	**0.0**	**0.0**	**0.0**	**0.0**	**0.0**
**Lower CI**	65.4	42.6	18.6	5.0	1.4	0.1	0.1	0.1	0.1	0.0	0.0	0.0	0.0	0.0	0.0	0.0
**Upper CI**	89.3	70.0	45.1	24.2	17.8	1.3	1.3	1.3	1.3	5.7	5.5	5.5	5.5	5.5	5.5	5.5
**Number**	56	41	24	9	4	2	2	2	2	1	0	0	0	0	0	0

Table W500-47 Age Group = 31-40 Gender = MALE Occupational group = NON-MANUAL Total cases = 62

	Threshold dB HL≥															
	**0**	**5**	**10**	**15**	**20**	**25**	**30**	**35**	**40**	**45**	**50**	**55**	**65**	**75**	**85**	**95**
**Prevalence**	**95.4**	**80.0**	**42.8**	**25.5**	**13.7**	**8.4**	**6.3**	**5.7**	**5.7**	**3.8**	**0.6**	**0.6**	**0.0**	**0.0**	**0.0**	**0.0**
**Lower CI**	87.3	64.1	32.1	15.7	6.7	3.3	2.2	1.8	1.8	0.8	0.1	0.1	0.0	0.0	0.0	0.0
**Upper CI**	98.4	90.0	54.2	38.7	26.1	19.9	17.0	16.9	16.9	16.0	3.6	3.6	5.8	5.8	5.8	5.8
**Number**	58	51	32	17	9	6	5	4	4	2	1	1	0	0	0	0

Table W500-48 Age Group = 41-50 Gender = MALE Occupational group = NON-MANUAL Total cases = 76

	Threshold dB HL≥															
	**0**	**5**	**10**	**15**	**20**	**25**	**30**	**35**	**40**	**45**	**50**	**55**	**65**	**75**	**85**	**95**
**Prevalence**	**96.7**	**80.4**	**53.3**	**30.4**	**23.9**	**13.5**	**13.5**	**9.3**	**9.3**	**8.6**	**7.2**	**4.2**	**1.4**	**0.9**	**0.9**	**0.9**
**Lower CI**	86.6	68.0	40.9	21.2	16.0	7.0	7.0	4.5	4.5	4.0	2.9	1.2	0.5	0.2	0.2	0.2
**Upper CI**	99.2	88.8	65.2	41.4	34.1	24.4	24.4	18.0	18.0	17.4	16.6	13.7	3.8	3.8	3.8	3.8
**Number**	74	61	40	25	18	12	12	9	9	8	7	5	4	2	2	2

Table W500-49 Age Group = 51-60 Gender = MALE Occupational group = NON-MANUAL Total cases = 142

	Threshold dB HL≥															
	**0**	**5**	**10**	**15**	**20**	**25**	**30**	**35**	**40**	**45**	**50**	**55**	**65**	**75**	**85**	**95**
**Prevalence**	**96.8**	**81.0**	**51.3**	**35.0**	**20.0**	**15.1**	**9.7**	**7.4**	**6.1**	**4.7**	**4.6**	**4.0**	**2.3**	**1.9**	**1.0**	**0.3**
**Lower CI**	91.6	70.3	42.5	27.4	15.1	11.1	6.7	4.8	3.7	2.6	2.5	2.1	1.0	0.8	0.4	0.1
**Upper CI**	98.8	88.5	59.9	43.4	26.1	20.3	13.9	11.1	9.9	8.4	8.3	7.5	5.0	4.6	2.6	1.2
**Number**	137	121	83	63	44	36	26	21	17	13	12	11	7	6	4	2

Prevalence by age group, gender and occupational group for the worse ear over 0.5 kHz

Table W500-50 Age Group = 61-70 Gender = MALE Occupational group = NON-MANUAL Total cases = 112

Threshold dB HL≥	0	5	10	15	20	25	30	35	40	45	50	55	65	75	85	95
**Prevalence**	**96.5**	**87.7**	**74.3**	**55.0**	**43.3**	**32.7**	**28.9**	**20.3**	**14.6**	**12.3**	**8.1**	**6.7**	**4.1**	**3.7**	**2.1**	**1.3**
**Lower CI**	91.9	78.6	64.7	40.8	30.5	21.3	18.0	14.8	10.6	8.5	4.6	3.6	2.0	1.8	0.9	0.5
**Upper CI**	98.5	93.3	82.0	68.4	57.1	46.7	42.9	27.1	19.6	17.5	13.8	12.1	8.2	7.8	4.5	3.5
**Number**	107	98	79	59	50	38	32	27	20	17	13	12	8	7	5	3

Table W500-51 Age Group = 71-80 Gender = MALE Occupational group = NON-MANUAL Total cases = 56

Threshold dB HL≥	0	5	10	15	20	25	30	35	40	45	50	55	65	75	85	95
**Prevalence**	**95.8**	**91.3**	**75.0**	**60.0**	**44.3**	**34.5**	**22.5**	**13.8**	**11.7**	**5.9**	**5.9**	**4.3**	**3.1**	**3.1**	**1.0**	**0.0**
**Lower CI**	87.2	82.1	60.4	47.0	32.6	23.5	13.5	7.4	6.2	3.1	3.1	2.5	1.2	1.2	0.2	0.0
**Upper CI**	98.7	96.0	85.6	71.7	56.6	47.4	35.2	24.3	21.1	11.0	11.0	7.0	8.1	8.1	5.3	6.4
**Number**	54	52	44	38	29	21	15	9	8	5	5	4	3	3	1	0

Table W500-52 Age Group = 18-30 Gender = FEMALE Occupational group = MANUAL Total cases = 109

Threshold dB HL≥	0	5	10	15	20	25	30	35	40	45	50	55	65	75	85	95
**Prevalence**	**93.7**	**78.7**	**46.1**	**20.4**	**12.0**	**7.5**	**7.5**	**5.8**	**3.7**	**2.5**	**1.4**	**0.7**	**0.0**	**0.0**	**0.0**	**0.0**
**Lower CI**	82.5	67.5	35.0	12.4	7.0	3.8	3.8	2.9	1.6	1.0	0.6	0.2	0.0	0.0	0.0	0.0
**Upper CI**	97.9	86.9	57.5	31.7	19.9	14.3	14.3	11.2	8.5	6.4	3.3	2.6	3.4	3.4	3.4	3.4
**Number**	105	89	53	32	21	13	13	11	8	6	5	2	0	0	0	0

Table W500-53 Age Group = 31-40 Gender = FEMALE Occupational group = MANUAL Total cases = 102

Threshold dB HL≥	0	5	10	15	20	25	30	35	40	45	50	55	65	75	85	95
**Prevalence**	**98.0**	**79.5**	**53.3**	**25.4**	**13.8**	**7.1**	**4.6**	**3.7**	**2.7**	**2.7**	**2.7**	**2.7**	**1.7**	**0.3**	**0.3**	**0.0**
**Lower CI**	93.0	67.6	41.5	18.0	8.4	3.9	2.6	1.9	1.2	1.2	1.2	1.2	0.7	0.1	0.1	0.0
**Upper CI**	99.4	87.8	64.8	34.6	21.9	12.7	7.9	7.1	5.9	5.9	5.9	5.9	4.3	1.7	1.7	3.6
**Number**	99	84	66	40	25	16	12	9	7	7	7	7	5	1	1	0

Table W500-54 Age Group = 41-50 Gender = FEMALE Occupational group = MANUAL Total cases = 134

Threshold dB HL≥	0	5	10	15	20	25	30	35	40	45	50	55	65	75	85	95
**Prevalence**	**97.6**	**84.7**	**63.5**	**37.9**	**27.5**	**18.1**	**11.7**	**10.0**	**7.6**	**6.7**	**6.7**	**6.7**	**3.7**	**1.3**	**1.0**	**1.0**
**Lower CI**	92.0	75.8	54.0	29.6	20.1	12.2	7.4	6.3	4.8	4.0	4.0	4.0	2.1	0.6	0.4	0.4
**Upper CI**	99.3	90.7	72.1	46.9	36.4	26.1	18.0	15.6	11.9	11.0	11.0	11.0	6.3	3.0	2.6	2.6
**Number**	132	116	91	60	46	35	27	25	21	18	18	18	11	5	4	4

Table W500-55 Age Group = 51-60 Gender = FEMALE Occupational group = MANUAL Total cases = 166

Threshold dB HL≥	0	5	10	15	20	25	30	35	40	45	50	55	65	75	85	95
**Prevalence**	**99.8**	**93.7**	**77.7**	**49.6**	**37.8**	**28.1**	**20.6**	**15.5**	**12.7**	**8.6**	**6.5**	**3.8**	**2.1**	**0.9**	**0.4**	**0.3**
**Lower CI**	99.1	86.9	68.6	41.0	30.1	21.2	14.8	10.6	8.3	5.2	3.8	2.2	1.0	0.4	0.1	0.1
**Upper CI**	100.0	97.1	84.7	58.1	46.2	36.1	28.0	22.1	18.8	14.0	10.9	6.6	4.1	1.8	1.2	1.1
**Number**	165	159	140	102	82	65	50	39	32	26	22	17	11	6	3	2

Table W500-56 Age Group = 61-70 Gender = FEMALE Occupational group = MANUAL Total cases = 124

Threshold dB HL≥	0	5	10	15	20	25	30	35	40	45	50	55	65	75	85	95
**Prevalence**	**97.1**	**93.8**	**77.2**	**60.5**	**46.5**	**36.0**	**29.4**	**22.2**	**18.2**	**13.8**	**11.6**	**10.4**	**9.5**	**6.7**	**2.6**	**2.1**
**Lower CI**	89.4	86.6	66.1	49.8	37.7	27.8	22.2	16.9	14.2	9.6	7.5	6.5	5.6	3.4	1.8	1.5
**Upper CI**	99.2	97.3	85.4	70.3	55.6	45.0	37.8	28.5	23.1	19.5	17.7	16.4	15.6	12.7	3.7	2.9
**Number**	122	117	104	86	69	58	52	43	37	34	28	24	20	12	5	3

Prevalence by age group, gender and occupational group for the worse ear over 0.5 kHz

Table W500-57 Age Group = 71-80 Gender = FEMALE Occupational group = MANUAL Total cases = 74

Threshold dB HL≥	0	5	10	15	20	25	30	35	40	45	50	55	65	75	85	95
**Prevalence**	**100.0**	**100.0**	**100.0**	**87.2**	**67.6**	**52.5**	**45.4**	**38.8**	**36.5**	**30.6**	**18.5**	**17.1**	**10.2**	**6.0**	**3.2**	**1.6**
**Lower CI**	95.1	95.1	95.1	72.7	51.2	40.1	33.4	27.0	24.8	19.5	11.8	10.4	6.5	3.3	1.4	0.6
**Upper CI**	100.0	100.0	100.0	94.5	80.6	64.5	57.9	52.1	50.0	44.6	27.9	26.7	15.9	10.7	7.0	4.2
**Number**	74	74	74	69	60	52	50	43	41	32	28	25	18	10	6	4

Table W500-58 Age Group = 18-30 Gender = MALE Occupational group = MANUAL Total cases = 109

Threshold dB HL≥	0	5	10	15	20	25	30	35	40	45	50	55	65	75	85	95
**Prevalence**	**90.6**	**81.1**	**46.1**	**25.2**	**12.8**	**10.6**	**7.3**	**2.3**	**0.8**	**0.8**	**0.7**	**0.7**	**0.5**	**0.1**	**0.1**	**0.1**
**Lower CI**	79.3	69.6	34.0	15.9	6.7	5.0	2.9	0.9	0.3	0.3	0.2	0.2	0.2	0.0	0.0	0.0
**Upper CI**	96.1	89.0	58.7	37.5	23.1	21.1	17.3	5.7	2.0	2.0	1.8	1.8	1.6	0.4	0.4	0.4
**Number**	100	90	59	36	24	18	14	8	5	5	4	4	3	1	1	1

Table W500-59 Age Group = 31-40 Gender = MALE Occupational group = MANUAL Total cases = 80

Threshold dB HL≥	0	5	10	15	20	25	30	35	40	45	50	55	65	75	85	95
**Prevalence**	**95.5**	**77.8**	**45.8**	**31.3**	**20.9**	**13.5**	**11.7**	**10.5**	**3.6**	**3.6**	**3.1**	**3.1**	**2.3**	**2.3**	**1.9**	**1.9**
**Lower CI**	85.1	63.0	32.2	19.6	11.9	6.9	5.4	4.6	1.3	1.3	0.9	0.9	0.5	0.5	0.3	0.3
**Upper CI**	98.8	87.8	60.1	46.0	34.0	24.8	23.2	22.4	10.1	10.1	9.9	9.9	9.9	9.9	10.2	10.2
**Number**	75	63	44	31	21	17	13	9	6	6	4	4	2	2	1	1

Table W500-60 Age Group = 41-50 Gender = MALE Occupational group = MANUAL Total cases = 127

Threshold dB HL≥	0	5	10	15	20	25	30	35	40	45	50	55	65	75	85	95
**Prevalence**	**93.6**	**81.2**	**63.8**	**46.3**	**30.8**	**22.4**	**16.6**	**13.6**	**11.5**	**8.3**	**7.8**	**5.0**	**2.4**	**0.5**	**0.5**	**0.2**
**Lower CI**	86.0	70.7	52.8	36.0	22.5	15.1	10.8	8.6	7.3	4.6	4.3	2.4	1.1	0.1	0.1	0.0
**Upper CI**	97.2	88.6	73.4	56.9	40.6	31.8	24.7	20.8	17.6	14.3	13.9	10.0	5.0	1.7	1.7	1.3
**Number**	121	111	90	68	49	38	29	25	23	17	16	11	7	2	2	1

Table W500-61 Age Group = 51-60 Gender = MALE Occupational group = MANUAL Total cases = 197

Threshold dB HL≥	0	5	10	15	20	25	30	35	40	45	50	55	65	75	85	95
**Prevalence**	**98.4**	**93.1**	**73.8**	**48.8**	**36.7**	**24.3**	**19.3**	**14.5**	**13.8**	**10.7**	**8.4**	**7.2**	**4.5**	**2.8**	**1.5**	**0.6**
**Lower CI**	93.9	86.6	65.5	40.8	29.5	18.8	14.7	10.8	10.1	7.5	5.6	4.6	2.6	1.4	0.7	0.2
**Upper CI**	99.6	96.6	80.7	57.0	44.6	30.8	24.8	19.2	18.5	15.0	12.4	11.2	7.6	5.5	3.2	1.9
**Number**	194	188	155	114	90	69	59	48	44	36	29	24	17	10	7	3

Table W500-62 Age Group = 61-70 Gender = MALE Occupational group = MANUAL Total cases = 173

Threshold dB HL≥	0	5	10	15	20	25	30	35	40	45	50	55	65	75	85	95
**Prevalence**	**98.3**	**92.7**	**80.1**	**65.4**	**44.4**	**34.2**	**27.1**	**19.2**	**14.6**	**11.2**	**9.9**	**7.5**	**4.8**	**3.1**	**2.2**	**1.3**
**Lower CI**	93.5	83.3	70.3	54.0	34.9	26.4	20.6	13.6	10.9	7.9	6.7	4.8	3.2	1.8	1.2	0.6
**Upper CI**	99.6	97.0	87.2	75.3	54.3	42.9	34.8	26.4	19.4	15.7	14.4	11.4	7.1	5.1	4.2	3.0
**Number**	170	165	146	130	98	84	72	53	46	37	32	25	20	14	10	6

Table W500-63 Age Group = 71-80 Gender = MALE Occupational group = MANUAL Total cases = 72

Threshold dB HL≥	0	5	10	15	20	25	30	35	40	45	50	55	65	75	85	95
**Prevalence**	**100.0**	**93.8**	**87.5**	**77.6**	**69.2**	**62.3**	**43.8**	**33.7**	**24.8**	**20.6**	**19.2**	**18.8**	**13.6**	**8.7**	**6.3**	**2.4**
**Lower CI**	94.9	72.7	67.8	59.8	54.6	47.4	30.0	21.1	17.1	13.8	12.5	12.1	7.7	4.8	3.5	1.1
**Upper CI**	100.0	98.8	95.9	89.0	80.7	75.1	58.5	49.1	34.4	29.6	28.4	28.0	23.0	15.3	11.0	5.0
**Number**	72	70	66	59	53	47	41	35	31	27	24	23	14	10	8	5

Prevalence by age group, gender and occupational group for the worse ear over 1 kHz

Table W1K-1 Age Group = 18-80 Gender = OVERALL Occupational group = OVERALL Total cases = 2660

Threshold dB HL≥	0	5	10	15	20	25	30	35	40	45	50	55	65	75	85	95
Prevalence	**93.5**	**74.3**	**53.3**	**35.7**	**25.3**	**18.5**	**14.4**	**11.8**	**9.1**	**7.2**	**5.8**	**4.8**	**3.1**	**2.2**	**1.2**	**0.8**
Lower CI	92.0	72.1	51.2	33.9	23.7	17.2	13.2	10.8	8.2	6.5	5.1	4.2	2.7	1.8	0.9	0.6
Upper CI	94.7	76.4	55.5	37.6	27.0	19.9	15.6	13.0	10.0	8.0	6.5	5.4	3.6	2.6	1.5	1.1
Number	2551	2193	1721	1299	1008	809	675	582	477	400	341	296	197	138	84	55

Table W1K-2 Age Group = 18-80 Gender = FEMALE Occupational group = OVERALL Total cases = 1384

Threshold dB HL≥	0	5	10	15	20	25	30	35	40	45	50	55	65	75	85	95
Prevalence	**93.5**	**75.0**	**55.3**	**36.0**	**24.4**	**18.5**	**14.5**	**12.5**	**9.5**	**7.3**	**5.6**	**4.7**	**3.1**	**2.1**	**1.1**	**0.8**
Lower CI	91.6	72.2	52.4	33.6	22.4	16.7	12.9	11.0	8.3	6.3	4.8	4.0	2.5	1.6	0.8	0.5
Upper CI	95.0	77.7	58.2	38.4	26.6	20.5	16.3	14.1	10.8	8.4	6.5	5.6	3.7	2.7	1.5	1.1
Number	1324	1132	898	653	488	403	339	295	243	203	175	154	104	69	42	28

Table W1K-3 Age Group = 18-80 Gender = MALE Occupational group = OVERALL Total cases = 1276

Threshold dB HL≥	0	5	10	15	20	25	30	35	40	45	50	55	65	75	85	95
Prevalence	**93.4**	**73.4**	**51.1**	**35.5**	**26.3**	**18.3**	**14.2**	**11.1**	**8.7**	**7.1**	**6.0**	**4.8**	**3.2**	**2.3**	**1.3**	**0.9**
Lower CI	91.0	69.9	47.8	32.7	23.9	16.5	12.6	9.8	7.5	6.1	5.0	4.0	2.5	1.7	0.9	0.5
Upper CI	95.3	76.6	54.4	38.4	28.8	20.4	15.9	12.6	10.0	8.3	7.1	5.8	4.0	3.1	1.9	1.5
Number	1227	1061	823	646	520	406	336	287	234	197	166	142	93	69	42	27

Table W1K-4 Age Group = 18-30 Gender = OVERALL Occupational group = OVERALL Total cases = 401

Threshold dB HL≥	0	5	10	15	20	25	30	35	40	45	50	55	65	75	85	95
Prevalence	**86.4**	**56.6**	**31.6**	**15.0**	**8.3**	**4.7**	**3.3**	**2.8**	**2.1**	**1.4**	**1.1**	**0.6**	**0.1**	**0.0**	**0.0**	**0.0**
Lower CI	81.7	50.8	26.8	11.7	5.9	3.2	2.2	1.8	1.3	0.8	0.5	0.3	0.0	0.0	0.0	0.0
Upper CI	90.0	62.2	36.8	19.0	11.4	6.8	4.9	4.3	3.4	2.4	2.1	1.2	0.5	0.1	0.1	0.1
Number	357	247	155	90	55	39	31	27	21	15	11	8	3	2	1	1

Table W1K-5 Age Group = 31-40 Gender = OVERALL Occupational group = OVERALL Total cases = 332

Threshold dB HL≥	0	5	10	15	20	25	30	35	40	45	50	55	65	75	85	95
Prevalence	**91.4**	**62.7**	**37.0**	**21.6**	**15.5**	**10.2**	**6.9**	**5.6**	**3.2**	**2.4**	**2.2**	**1.8**	**1.3**	**1.0**	**0.8**	**0.6**
Lower CI	87.2	56.2	31.2	17.0	11.6	7.2	4.6	3.6	1.8	1.3	1.2	0.9	0.5	0.3	0.2	0.1
Upper CI	94.4	68.8	43.3	27.0	20.3	14.1	10.1	8.5	5.5	4.3	4.1	3.7	3.3	3.0	3.0	3.1
Number	307	230	144	96	73	51	37	30	19	16	14	11	7	5	3	1

Table W1K-6 Age Group = 41-50 Gender = OVERALL Occupational group = OVERALL Total cases = 450

Threshold dB HL≥	0	5	10	15	20	25	30	35	40	45	50	55	65	75	85	95
Prevalence	**95.3**	**77.1**	**56.0**	**32.7**	**19.2**	**15.3**	**11.3**	**9.1**	**6.8**	**5.3**	**4.8**	**4.0**	**1.8**	**1.1**	**0.7**	**0.5**
Lower CI	92.4	72.4	51.0	28.4	16.1	12.5	9.0	7.1	5.0	3.8	3.4	2.7	1.1	0.6	0.3	0.2
Upper CI	97.2	81.3	61.0	37.4	22.8	18.6	14.1	11.6	9.0	7.4	6.8	5.7	2.7	2.1	1.5	1.1
Number	434	364	271	178	119	100	79	67	52	40	37	33	18	11	7	6

Table W1K-7 Age Group = 51-60 Gender = OVERALL Occupational group = OVERALL Total cases = 681

Threshold dB HL≥	0	5	10	15	20	25	30	35	40	45	50	55	65	75	85	95
Prevalence	**96.9**	**85.2**	**64.9**	**44.2**	**31.1**	**21.0**	**16.1**	**12.5**	**9.9**	**8.0**	**6.6**	**5.7**	**3.7**	**2.6**	**1.7**	**1.3**
Lower CI	95.1	81.9	60.8	40.2	27.6	18.2	13.8	10.4	8.1	6.4	5.2	4.4	2.6	1.7	1.0	0.7
Upper CI	98.0	88.0	68.8	48.3	34.8	24.1	18.8	15.0	12.2	9.9	8.3	7.3	5.2	3.8	2.8	2.3
Number	662	597	478	361	278	209	171	139	114	98	86	76	48	36	24	18

Prevalence by age group, gender and occupational group for the worse ear over 1 kHz

Table W1K-8 Age Group = 61-70 Gender = OVERALL Occupational group = OVERALL Total cases = 526

Threshold dB HL≥	0	5	10	15	20	25	30	35	40	45	50	55	65	75	85	95
Prevalence	99.1	92.7	76.8	58.4	42.3	33.3	25.7	22.5	16.6	13.2	10.6	9.4	6.7	5.1	2.4	1.5
Lower CI	97.3	89.1	71.9	53.2	37.7	29.2	22.0	19.1	13.7	10.8	8.4	7.3	5.2	3.7	1.7	0.9
Upper CI	99.7	95.2	81.1	63.4	47.1	37.6	29.8	26.3	19.8	16.2	13.4	12.0	8.6	7.0	3.4	2.3
Number	522	493	427	346	277	229	195	179	145	123	101	90	67	49	29	17

Table W1K-9 Age Group = 71-80 Gender = OVERALL Occupational group = OVERALL Total cases = 270

Threshold dB HL≥	0	5	10	15	20	25	30	35	40	45	50	55	65	75	85	95
Prevalence	99.6	94.2	85.6	77.1	67.1	52.1	44.6	37.2	31.5	25.9	19.1	15.0	10.3	7.0	3.3	2.0
Lower CI	98.0	88.4	78.7	70.5	60.1	44.8	37.5	30.7	26.1	21.0	15.5	12.1	7.7	4.8	2.1	1.1
Upper CI	99.9	97.2	90.6	82.6	73.5	59.3	52.0	44.1	37.4	31.6	23.3	18.5	13.6	10.0	5.2	3.5
Number	269	262	246	228	206	181	162	140	126	108	92	78	54	35	20	12

Table W1K-10 Age Group = 18-80 Gender = OVERALL Occupational group = NON-MANUAL Total cases = 1183

Threshold dB HL≥	0	5	10	15	20	25	30	35	40	45	50	55	65	75	85	95
Prevalence	93.5	70.7	49.3	30.4	22.1	16.6	12.5	10.3	7.5	5.4	4.2	3.4	2.4	1.9	1.0	0.7
Lower CI	91.4	67.4	46.2	27.8	19.9	15.0	11.0	9.0	6.4	4.5	3.4	2.8	1.9	1.4	0.7	0.4
Upper CI	95.2	73.8	52.5	33.0	24.5	18.4	14.1	11.7	8.6	6.4	5.1	4.1	3.0	2.5	1.5	1.1
Number	1126	926	699	486	370	297	238	208	169	139	115	98	73	57	35	24

Table W1K-11 Age Group = 18-80 Gender = OVERALL Occupational group = MANUAL Total cases = 1472

Threshold dB HL≥	0	5	10	15	20	25	30	35	40	45	50	55	65	75	85	95
Prevalence	93.3	77.3	56.6	39.7	28.3	19.9	15.9	13.0	10.3	8.1	6.6	5.5	3.4	2.2	1.2	0.9
Lower CI	90.9	74.0	53.4	36.9	26.0	18.1	14.3	11.6	9.0	7.0	5.7	4.7	2.8	1.7	0.9	0.6
Upper CI	95.2	80.2	59.8	42.5	30.8	22.0	17.6	14.6	11.7	9.4	7.7	6.5	4.2	2.9	1.7	1.3
Number	1421	1264	1019	810	637	511	436	373	308	261	226	198	124	81	49	31

Table W1K-12 Age Group = 18-30 Gender = FEMALE Occupational group = OVERALL Total cases = 224

Threshold dB HL≥	0	5	10	15	20	25	30	35	40	45	50	55	65	75	85	95
Prevalence	88.6	58.3	35.1	15.2	9.0	5.2	3.6	3.5	2.8	1.9	1.6	0.7	0.0	0.0	0.0	0.0
Lower CI	82.7	50.8	28.5	11.2	6.1	3.4	2.1	2.0	1.5	0.9	0.7	0.3	0.0	0.0	0.0	0.0
Upper CI	92.6	65.4	42.2	20.4	13.0	8.1	6.0	5.9	5.2	3.8	3.5	1.9	1.7	1.7	1.7	1.7
Number	202	141	90	50	32	23	17	16	12	9	7	4	0	0	0	0

Table W1K-13 Age Group = 31-40 Gender = FEMALE Occupational group = OVERALL Total cases = 190

Threshold dB HL≥	0	5	10	15	20	25	30	35	40	45	50	55	65	75	85	95
Prevalence	87.8	61.7	34.5	19.1	13.1	10.2	7.2	6.3	3.9	2.4	2.3	2.1	1.4	0.8	0.4	0.0
Lower CI	81.0	53.3	27.4	13.9	8.8	6.5	4.5	3.7	2.0	1.3	1.2	1.1	0.6	0.3	0.1	0.0
Upper CI	92.4	69.4	42.4	25.6	19.0	15.6	11.5	10.6	7.3	4.4	4.3	4.1	3.1	2.2	1.6	2.0
Number	172	131	82	52	38	28	22	18	13	11	10	9	6	4	2	0

Table W1K-14 Age Group = 41-50 Gender = FEMALE Occupational group = OVERALL Total cases = 247

Threshold dB HL≥	0	5	10	15	20	25	30	35	40	45	50	55	65	75	85	95
Prevalence	94.6	75.5	58.2	31.8	16.7	13.4	9.3	7.1	5.5	4.4	3.9	3.5	2.0	0.9	0.9	0.6
Lower CI	90.2	68.9	51.4	26.1	12.7	10.0	6.6	4.9	3.6	2.8	2.5	2.2	1.1	0.4	0.4	0.2
Upper CI	97.0	81.1	64.7	38.2	21.6	17.8	12.8	10.3	8.2	7.0	6.2	5.5	3.5	2.2	2.2	1.5
Number	237	197	154	93	56	47	37	31	24	20	19	18	11	5	5	4

Prevalence by age group, gender and occupational group for the worse ear over 1 kHz

Table W1K-15 Age Group = 51-60 Gender = FEMALE Occupational group = OVERALL Total cases = 340

	Threshold dB HL≥															
	**0**	**5**	**10**	**15**	**20**	**25**	**30**	**35**	**40**	**45**	**50**	**55**	**65**	**75**	**85**	**95**
**Prevalence**	**97.5**	**85.2**	**65.4**	**43.3**	**27.8**	**20.6**	**15.3**	**11.2**	**9.5**	**6.5**	**5.1**	**4.0**	**2.9**	**2.2**	**1.5**	**1.3**
**Lower CI**	95.1	80.5	59.7	37.8	23.3	16.7	12.0	8.4	6.9	4.6	3.5	2.7	1.7	1.3	0.7	0.5
**Upper CI**	98.7	88.9	70.8	49.0	32.9	25.1	19.2	14.8	12.8	9.0	7.2	6.1	4.9	4.0	3.2	3.1
**Number**	333	297	242	178	130	103	82	64	56	45	39	33	22	18	11	8

Table W1K-16 Age Group = 61-70 Gender = FEMALE Occupational group = OVERALL Total cases = 242

	Threshold dB HL≥															
	**0**	**5**	**10**	**15**	**20**	**25**	**30**	**35**	**40**	**45**	**50**	**55**	**65**	**75**	**85**	**95**
**Prevalence**	**98.9**	**94.5**	**79.0**	**56.6**	**39.9**	**32.6**	**23.9**	**21.5**	**14.7**	**12.4**	**9.9**	**9.5**	**6.3**	**4.4**	**2.2**	**1.6**
**Lower CI**	95.5	90.2	72.5	49.6	33.8	27.0	18.8	16.8	10.9	8.9	6.9	6.6	4.7	2.9	1.3	0.9
**Upper CI**	99.7	96.9	84.3	63.3	46.2	38.7	29.9	27.1	19.6	16.9	14.0	13.6	8.5	6.7	3.4	2.9
**Number**	240	228	198	154	119	102	86	81	64	56	49	46	33	22	13	9

Table W1K-17 Age Group = 71-80 Gender = FEMALE Occupational group = OVERALL Total cases = 141

	Threshold dB HL≥															
	**0**	**5**	**10**	**15**	**20**	**25**	**30**	**35**	**40**	**45**	**50**	**55**	**65**	**75**	**85**	**95**
**Prevalence**	**99.4**	**94.6**	**87.0**	**81.0**	**67.5**	**51.1**	**47.0**	**42.6**	**33.8**	**27.2**	**17.9**	**15.0**	**10.4**	**7.3**	**3.2**	**1.9**
**Lower CI**	96.8	85.3	77.0	71.5	57.4	41.1	37.1	33.2	26.8	20.7	14.0	11.2	7.2	4.4	1.8	0.9
**Upper CI**	99.9	98.1	93.0	87.9	76.3	61.0	57.1	52.5	41.6	34.8	22.6	19.8	14.9	11.7	5.8	4.1
**Number**	140	138	132	126	113	100	95	85	74	62	51	44	32	20	11	7

Table W1K-18 Age Group = 18-30 Gender = MALE Occupational group = OVERALL Total cases = 177

	Threshold dB HL≥															
	**0**	**5**	**10**	**15**	**20**	**25**	**30**	**35**	**40**	**45**	**50**	**55**	**65**	**75**	**85**	**95**
**Prevalence**	**84.1**	**54.7**	**27.9**	**14.6**	**7.4**	**4.0**	**2.8**	**2.0**	**1.3**	**0.8**	**0.4**	**0.4**	**0.3**	**0.1**	**0.0**	**0.0**
**Lower CI**	76.1	45.9	21.2	9.8	4.2	2.1	1.6	1.0	0.6	0.3	0.1	0.1	0.1	0.0	0.0	0.0
**Upper CI**	89.8	63.3	35.8	21.1	12.9	7.5	5.0	3.9	2.4	1.8	1.2	1.2	1.0	0.2	0.2	0.2
**Number**	155	106	65	40	23	16	14	11	9	6	4	4	3	2	1	1

Table W1K-19 Age Group = 31-40 Gender = MALE Occupational group = OVERALL Total cases = 142

	Threshold dB HL≥															
	**0**	**5**	**10**	**15**	**20**	**25**	**30**	**35**	**40**	**45**	**50**	**55**	**65**	**75**	**85**	**95**
**Prevalence**	**95.5**	**63.8**	**40.1**	**24.6**	**18.4**	**10.4**	**6.5**	**4.7**	**2.5**	**2.4**	**2.1**	**1.5**	**1.2**	**1.2**	**1.2**	**1.2**
**Lower CI**	89.2	53.6	30.8	17.3	12.1	6.1	3.4	2.3	0.9	0.8	0.7	0.3	0.2	0.2	0.2	0.2
**Upper CI**	98.1	72.9	50.1	33.7	26.9	17.0	12.3	9.6	6.6	6.5	6.4	6.3	6.5	6.5	6.5	6.5
**Number**	135	99	62	44	35	23	15	12	6	5	4	2	1	1	1	1

Table W1K-20 Age Group = 41-50 Gender = MALE Occupational group = OVERALL Total cases = 203

	Threshold dB HL≥															
	**0**	**5**	**10**	**15**	**20**	**25**	**30**	**35**	**40**	**45**	**50**	**55**	**65**	**75**	**85**	**95**
**Prevalence**	**96.1**	**79.0**	**53.7**	**33.7**	**22.0**	**17.4**	**13.5**	**11.2**	**8.2**	**6.2**	**5.7**	**4.4**	**1.5**	**1.3**	**0.4**	**0.4**
**Lower CI**	91.3	71.9	46.1	27.3	17.3	13.2	9.8	8.0	5.4	3.8	3.5	2.5	0.8	0.6	0.1	0.1
**Upper CI**	98.3	84.7	61.1	40.7	27.6	22.6	18.1	15.6	12.1	9.8	9.3	7.6	3.0	3.0	1.6	1.6
**Number**	197	167	117	85	63	53	42	36	28	20	18	15	7	6	2	2

Table W1K-21 Age Group = 51-60 Gender = MALE Occupational group = OVERALL Total cases = 341

	Threshold dB HL≥															
	**0**	**5**	**10**	**15**	**20**	**25**	**30**	**35**	**40**	**45**	**50**	**55**	**65**	**75**	**85**	**95**
**Prevalence**	**96.2**	**85.1**	**64.2**	**45.1**	**34.8**	**21.4**	**17.1**	**13.9**	**10.5**	**9.7**	**8.3**	**7.6**	**4.6**	**3.0**	**2.0**	**1.3**
**Lower CI**	93.0	80.1	58.2	39.5	29.6	17.6	13.8	11.0	7.9	7.2	6.1	5.5	2.9	1.7	1.0	0.7
**Upper CI**	98.0	89.0	69.8	50.9	40.4	25.9	20.9	17.5	13.7	12.9	11.1	10.4	7.1	5.0	3.7	2.7
**Number**	329	300	236	183	148	106	89	75	58	53	47	43	26	18	13	10

Prevalence by age group, gender and occupational group for the worse ear over 1 kHz

Table W1K-22 Age Group = 61-70 Gender = MALE Occupational group = OVERALL Total cases = 284

	Threshold dB HL≥															
	**0**	**5**	**10**	**15**	**20**	**25**	**30**	**35**	**40**	**45**	**50**	**55**	**65**	**75**	**85**	**95**
**Prevalence**	**99.4**	**90.7**	**74.2**	**60.6**	**45.2**	**34.0**	**27.7**	**23.7**	**18.8**	**14.4**	**11.6**	**9.2**	**7.2**	**5.9**	**2.7**	**1.3**
**Lower CI**	97.5	84.2	66.4	52.8	38.2	28.4	22.8	19.2	15.0	11.2	8.6	6.7	4.8	3.7	1.6	0.6
**Upper CI**	99.8	94.7	80.7	67.9	52.4	40.0	33.2	28.9	23.4	18.3	15.5	12.7	10.7	9.4	4.4	2.6
**Number**	282	265	229	192	158	127	109	98	81	67	52	44	34	27	16	8

Table W1K-23 Age Group = 71-80 Gender = MALE Occupational group = OVERALL Total cases = 129

	Threshold dB HL≥															
	**0**	**5**	**10**	**15**	**20**	**25**	**30**	**35**	**40**	**45**	**50**	**55**	**65**	**75**	**85**	**95**
**Prevalence**	**100.0**	**93.8**	**83.2**	**70.3**	**65.7**	**52.8**	**40.6**	**28.8**	**27.8**	**24.0**	**20.9**	**15.2**	**10.1**	**6.5**	**3.5**	**2.0**
**Lower CI**	97.1	85.3	72.7	60.8	55.9	43.0	31.4	21.7	20.6	17.4	14.7	11.1	6.5	3.9	1.8	0.8
**Upper CI**	100.0	97.5	90.2	78.3	74.2	62.4	50.5	37.2	36.3	32.2	28.8	20.6	15.4	10.7	6.6	4.9
**Number**	129	124	114	102	93	81	67	55	52	46	41	34	22	15	9	5

Table W1K-24 Age Group = 18-30 Gender = OVERALL Occupational group = NON-MANUAL Total cases = 181

	Threshold dB HL≥															
	**0**	**5**	**10**	**15**	**20**	**25**	**30**	**35**	**40**	**45**	**50**	**55**	**65**	**75**	**85**	**95**
**Prevalence**	**87.2**	**50.1**	**25.6**	**9.6**	**6.3**	**3.5**	**2.5**	**1.5**	**1.3**	**1.0**	**0.7**	**0.7**	**0.1**	**0.1**	**0.0**	**0.0**
**Lower CI**	80.5	41.5	18.9	5.7	3.3	1.9	1.2	0.7	0.6	0.4	0.2	0.2	0.0	0.0	0.0	0.0
**Upper CI**	91.9	58.6	33.8	15.6	11.9	6.3	5.0	3.0	2.8	2.5	2.5	2.5	2.2	2.2	2.1	2.1
**Number**	160	97	55	24	17	13	10	8	6	4	3	3	1	1	0	0

Table W1K-25 Age Group = 31-40 Gender = OVERALL Occupational group = NON-MANUAL Total cases = 148

	Threshold dB HL≥															
	**0**	**5**	**10**	**15**	**20**	**25**	**30**	**35**	**40**	**45**	**50**	**55**	**65**	**75**	**85**	**95**
**Prevalence**	**92.4**	**59.8**	**35.4**	**17.1**	**13.4**	**9.1**	**5.5**	**4.8**	**1.6**	**0.8**	**0.6**	**0.6**	**0.3**	**0.3**	**0.3**	**0.0**
**Lower CI**	86.6	51.6	27.9	11.5	8.4	5.0	2.6	2.2	0.6	0.3	0.2	0.2	0.1	0.1	0.1	0.0
**Upper CI**	95.8	67.5	43.7	24.7	20.9	15.8	11.1	10.4	4.7	2.5	2.3	2.3	1.7	1.7	1.7	2.5
**Number**	136	97	55	30	22	15	10	9	4	3	2	2	1	1	1	0

Table W1K-26 Age Group = 41-50 Gender = OVERALL Occupational group = NON-MANUAL Total cases = 188

	Threshold dB HL≥															
	**0**	**5**	**10**	**15**	**20**	**25**	**30**	**35**	**40**	**45**	**50**	**55**	**65**	**75**	**85**	**95**
**Prevalence**	**93.9**	**73.1**	**48.5**	**28.4**	**18.0**	**15.1**	**11.4**	**8.6**	**6.3**	**2.8**	**2.8**	**1.6**	**0.9**	**0.6**	**0.5**	**0.5**
**Lower CI**	88.2	65.3	41.1	22.3	13.4	11.0	7.1	5.2	3.8	1.1	1.1	0.7	0.4	0.2	0.1	0.1
**Upper CI**	96.9	79.7	56.0	35.5	23.6	20.3	17.7	14.0	10.3	7.1	7.1	3.5	1.9	1.8	1.9	1.9
**Number**	179	142	99	57	36	29	22	18	14	7	7	6	4	3	2	2

Table W1K-27 Age Group = 51-60 Gender = OVERALL Occupational group = NON-MANUAL Total cases = 316

	Threshold dB HL≥															
	**0**	**5**	**10**	**15**	**20**	**25**	**30**	**35**	**40**	**45**	**50**	**55**	**65**	**75**	**85**	**95**
**Prevalence**	**96.7**	**83.5**	**59.7**	**36.3**	**25.0**	**15.7**	**11.5**	**9.4**	**7.5**	**6.2**	**5.6**	**4.5**	**3.1**	**2.6**	**1.7**	**1.5**
**Lower CI**	94.1	78.6	53.7	31.1	20.6	12.6	9.1	7.2	5.6	4.5	4.0	3.0	1.9	1.5	0.9	0.7
**Upper CI**	98.2	87.5	65.3	41.9	29.9	19.2	14.4	12.2	10.1	8.5	7.8	6.8	5.2	4.6	3.3	3.1
**Number**	304	264	199	143	108	80	64	55	46	40	36	28	20	17	12	10

Table W1K-28 Age Group = 61-70 Gender = OVERALL Occupational group = NON-MANUAL Total cases = 227

	Threshold dB HL≥															
	**0**	**5**	**10**	**15**	**20**	**25**	**30**	**35**	**40**	**45**	**50**	**55**	**65**	**75**	**85**	**95**
**Prevalence**	**99.1**	**92.9**	**81.7**	**57.5**	**39.0**	**30.0**	**21.4**	**19.1**	**15.3**	**11.7**	**8.1**	**6.7**	**5.8**	**5.3**	**2.7**	**1.6**
**Lower CI**	95.6	87.8	73.7	48.9	31.6	24.7	16.9	15.2	11.9	9.1	6.1	5.0	4.0	3.6	1.6	0.9
**Upper CI**	99.8	95.9	87.8	65.7	46.9	35.8	26.5	23.8	19.6	15.0	10.7	9.0	8.1	7.8	4.7	2.9
**Number**	225	210	186	138	103	85	69	65	55	46	36	32	25	22	14	10

Prevalence by age group, gender and occupational group for the worse ear over 1 kHz

Table W1K-29 Age Group = 71-80 Gender = OVERALL Occupational group = NON-MANUAL Total cases = 123

	Threshold dB HL≥															
	0	5	10	15	20	25	30	35	40	45	50	55	65	75	85	95
**Prevalence**	**99.1**	**90.6**	**78.4**	**67.0**	**61.0**	**53.6**	**43.6**	**37.2**	**25.9**	**19.9**	**14.2**	**11.6**	**9.0**	**6.0**	**2.4**	**1.0**
**Lower CI**	95.1	78.4	66.9	59.9	52.8	45.9	36.6	30.9	20.0	14.6	10.4	8.5	6.0	3.5	1.1	0.2
**Upper CI**	99.8	96.3	86.7	73.4	68.6	61.2	50.8	44.0	32.8	26.5	19.1	15.7	13.2	10.3	5.4	4.0
**Number**	122	116	105	94	84	75	63	53	44	39	31	27	22	13	6	2

Table W1K-30 Age Group = 18-30 Gender = OVERALL Occupational group = MANUAL Total cases = 218

	Threshold dB HL≥															
	0	5	10	15	20	25	30	35	40	45	50	55	65	75	85	95
**Prevalence**	**86.1**	**63.4**	**38.1**	**19.4**	**10.2**	**5.4**	**3.8**	**3.6**	**2.7**	**1.6**	**1.3**	**0.5**	**0.2**	**0.0**	**0.0**	**0.0**
**Lower CI**	78.5	55.1	30.4	14.2	6.4	3.2	2.2	2.0	1.4	0.7	0.5	0.2	0.0	0.0	0.0	0.0
**Upper CI**	91.4	70.9	46.4	26.0	16.0	8.9	6.5	6.4	5.1	3.6	3.4	1.2	0.7	0.2	0.2	0.2
**Number**	196	150	100	66	38	26	21	19	15	11	8	5	2	1	1	1

Table W1K-31 Age Group = 31-40 Gender = OVERALL Occupational group = MANUAL Total cases = 183

	Threshold dB HL≥															
	0	5	10	15	20	25	30	35	40	45	50	55	65	75	85	95
**Prevalence**	**90.1**	**64.8**	**36.9**	**24.0**	**15.6**	**10.9**	**7.6**	**5.4**	**4.1**	**3.2**	**3.0**	**2.5**	**1.8**	**1.3**	**1.0**	**0.9**
**Lower CI**	82.3	54.8	28.7	17.5	10.7	6.9	4.4	3.1	2.1	1.6	1.5	1.1	0.6	0.4	0.2	0.2
**Upper CI**	94.7	73.6	46.1	32.0	22.3	16.7	12.7	9.1	7.9	6.2	6.1	5.7	5.1	4.8	4.9	5.0
**Number**	170	132	88	65	50	35	26	20	15	13	12	9	6	4	2	1

Table W1K-32 Age Group = 41-50 Gender = OVERALL Occupational group = MANUAL Total cases = 262

	Threshold dB HL≥															
	0	5	10	15	20	25	30	35	40	45	50	55	65	75	85	95
**Prevalence**	**96.0**	**79.7**	**62.1**	**37.2**	**22.4**	**18.2**	**12.8**	**10.9**	**8.1**	**7.2**	**6.4**	**5.6**	**2.1**	**1.3**	**0.9**	**0.5**
**Lower CI**	92.0	72.9	55.0	30.9	17.6	13.9	9.6	7.9	5.6	4.8	4.3	3.6	1.3	0.7	0.5	0.2
**Upper CI**	98.1	85.2	68.8	44.1	28.1	23.5	16.9	14.8	11.6	10.6	9.5	8.6	3.2	2.2	1.6	1.4
**Number**	255	222	172	121	83	71	57	49	38	33	30	27	14	8	5	4

Table W1K-33 Age Group = 51-60 Gender = OVERALL Occupational group = MANUAL Total cases = 365

	Threshold dB HL≥															
	0	5	10	15	20	25	30	35	40	45	50	55	65	75	85	95
**Prevalence**	**97.1**	**86.1**	**69.3**	**48.0**	**34.2**	**23.7**	**18.8**	**14.2**	**11.2**	**8.7**	**6.6**	**6.0**	**3.6**	**2.0**	**1.4**	**0.9**
**Lower CI**	93.6	80.9	63.2	42.4	29.1	19.4	15.1	11.1	8.5	6.5	4.9	4.4	2.3	1.2	0.8	0.4
**Upper CI**	98.7	90.0	74.8	53.7	39.7	28.5	23.2	17.9	14.5	11.6	8.8	8.1	5.6	3.4	2.7	2.0
**Number**	358	333	279	218	170	129	107	84	68	58	50	48	28	19	12	8

Table W1K-34 Age Group = 61-70 Gender = OVERALL Occupational group = MANUAL Total cases = 298

	Threshold dB HL≥															
	0	5	10	15	20	25	30	35	40	45	50	55	65	75	85	95
**Prevalence**	**99.2**	**93.1**	**71.3**	**59.3**	**46.6**	**35.9**	**29.1**	**25.3**	**17.9**	**14.5**	**13.0**	**11.3**	**8.0**	**5.7**	**2.4**	**1.6**
**Lower CI**	96.9	87.8	63.7	51.9	39.9	29.9	24.1	20.8	13.7	10.8	9.4	7.9	5.4	3.4	1.8	1.1
**Upper CI**	99.8	96.2	77.9	66.4	53.4	42.4	34.8	30.4	23.0	19.3	17.7	15.9	11.5	9.4	3.4	2.4
**Number**	296	282	240	207	174	144	126	114	90	77	65	58	42	27	15	7

Table W1K-35 Age Group = 71-80 Gender = OVERALL Occupational group = MANUAL Total cases = 146

	Threshold dB HL≥															
	0	5	10	15	20	25	30	35	40	45	50	55	65	75	85	95
**Prevalence**	**100.0**	**97.4**	**94.0**	**88.3**	**78.1**	**51.6**	**47.6**	**38.9**	**36.3**	**28.3**	**20.2**	**16.3**	**10.3**	**6.8**	**3.5**	**2.7**
**Lower CI**	97.4	86.6	83.3	77.0	68.2	42.0	38.1	31.0	28.3	20.6	16.2	12.5	7.2	4.5	2.1	1.4
**Upper CI**	100.0	99.6	98.0	94.5	85.6	61.0	57.2	47.4	45.2	37.5	24.9	20.9	14.5	10.0	5.9	5.0
**Number**	146	145	140	133	122	106	99	87	82	69	61	51	32	22	14	10

Prevalence by age group, gender and occupational group for the worse ear over 1 kHz

Table W1K-36 Age Group = 18-80 Gender = FEMALE Occupational group = NON-MANUAL Total cases = 670

Threshold dB HL≥	0	5	10	15	20	25	30	35	40	45	50	55	65	75	85	95
Prevalence	93.8	72.0	52.2	31.5	21.8	17.4	12.9	11.3	8.4	5.9	4.2	3.9	2.8	2.1	1.2	0.8
Lower CI	91.0	67.9	48.3	28.4	19.3	15.2	11.2	9.7	7.0	4.8	3.4	3.1	2.2	1.5	0.8	0.4
Upper CI	95.7	75.8	56.1	34.7	24.6	19.7	14.9	13.1	10.0	7.2	5.3	5.0	3.6	2.9	2.0	1.5
Number	641	524	405	274	203	169	139	122	104	87	71	64	49	35	23	15

Table W1K-37 Age Group = 18-80 Gender = MALE Occupational group = NON-MANUAL Total cases = 513

Threshold dB HL≥	0	5	10	15	20	25	30	35	40	45	50	55	65	75	85	95
Prevalence	93.2	69.3	46.1	29.1	22.5	15.8	11.9	9.2	6.4	4.9	4.1	2.7	1.9	1.7	0.8	0.5
Lower CI	89.5	63.9	41.1	25.1	18.9	13.3	9.5	7.4	5.0	3.6	3.0	2.0	1.3	1.1	0.4	0.3
Upper CI	95.7	74.1	51.2	33.5	26.5	18.5	14.8	11.5	8.2	6.4	5.7	3.7	2.9	2.6	1.4	1.0
Number	485	402	294	212	167	128	99	86	65	52	44	34	24	22	12	9

Table W1K-38 Age Group = 18-80 Gender = FEMALE Occupational group = MANUAL Total cases = 712

Threshold dB HL≥	0	5	10	15	20	25	30	35	40	45	50	55	65	75	85	95
Prevalence	92.9	78.2	58.7	39.7	27.1	19.8	15.9	13.5	10.4	7.8	6.1	4.9	2.9	1.8	1.0	0.7
Lower CI	89.5	73.8	54.1	36.1	24.1	17.2	13.7	11.5	8.6	6.2	5.0	4.0	2.2	1.3	0.7	0.5
Upper CI	95.3	82.0	63.2	43.5	30.4	22.6	18.4	15.7	12.6	9.7	7.5	6.1	3.8	2.5	1.3	1.1
Number	681	606	491	377	284	233	199	172	139	116	104	90	55	34	19	13

Table W1K-39 Age Group = 18-80 Gender = MALE Occupational group = MANUAL Total cases = 760

Threshold dB HL≥	0	5	10	15	20	25	30	35	40	45	50	55	65	75	85	95
Prevalence	93.8	76.2	54.3	39.6	29.7	20.1	15.8	12.5	10.1	8.5	7.2	6.2	4.0	2.7	1.5	1.1
Lower CI	89.9	71.2	49.7	35.5	26.2	17.4	13.6	10.7	8.5	7.0	5.8	4.9	2.9	1.8	0.9	0.6
Upper CI	96.2	80.5	58.9	43.9	33.4	23.1	18.4	14.6	12.0	10.3	8.9	7.9	5.5	4.1	2.5	2.1
Number	740	658	528	433	353	278	237	201	169	145	122	108	69	47	30	18

Table W1K-40 Age Group = 18-30 Gender = FEMALE Occupational group = NON-MANUAL Total cases = 115

Threshold dB HL≥	0	5	10	15	20	25	30	35	40	45	50	55	65	75	85	95
Prevalence	88.4	51.8	27.7	10.3	7.3	5.0	3.0	2.4	2.0	1.8	1.2	1.2	0.0	0.0	0.0	0.0
Lower CI	80.5	42.1	20.2	6.2	4.2	2.7	1.6	1.1	0.8	0.7	0.3	0.3	0.0	0.0	0.0	0.0
Upper CI	93.4	61.3	36.6	16.5	12.4	9.0	5.7	5.3	4.9	4.6	4.7	4.7	3.2	3.2	3.2	3.2
Number	103	64	37	17	13	10	7	6	4	3	2	2	0	0	0	0

Table W1K-41 Age Group = 31-40 Gender = FEMALE Occupational group = NON-MANUAL Total cases = 86

Threshold dB HL≥	0	5	10	15	20	25	30	35	40	45	50	55	65	75	85	95
Prevalence	88.9	59.8	35.4	16.0	12.0	8.6	5.2	5.2	3.1	1.6	1.2	1.2	0.6	0.6	0.6	0.0
Lower CI	78.7	47.9	25.5	9.4	6.3	3.9	2.1	2.1	1.0	0.5	0.3	0.3	0.1	0.1	0.1	0.0
Upper CI	94.6	70.6	46.7	25.8	21.7	17.8	12.1	12.1	8.7	4.7	4.5	4.5	3.2	3.2	3.2	4.3
Number	78	56	33	17	12	8	6	6	4	3	2	2	1	1	1	0

Table W1K-42 Age Group = 41-50 Gender = FEMALE Occupational group = NON-MANUAL Total cases = 112

Threshold dB HL≥	0	5	10	15	20	25	30	35	40	45	50	55	65	75	85	95
Prevalence	95.5	71.7	53.3	27.6	14.9	12.1	8.2	5.6	5.4	1.6	1.6	1.6	0.6	0.0	0.0	0.0
Lower CI	88.8	61.9	43.7	20.2	10.2	8.2	4.9	2.9	2.8	0.5	0.5	0.5	0.1	0.0	0.0	0.0
Upper CI	98.3	79.8	62.6	36.6	21.2	17.6	13.5	10.4	10.2	5.3	5.3	5.3	4.3	3.3	3.3	3.3
Number	108	83	64	33	19	15	10	7	6	2	2	2	1	0	0	0

Prevalence by age group, gender and occupational group for the worse ear over 1 kHz

Table W1K-43 Age Group = 51-60 Gender = FEMALE Occupational group = NON-MANUAL Total cases = 174

Threshold dB HL≥	0	5	10	15	20	25	30	35	40	45	50	55	65	75	85	95
Prevalence	**98.6**	**86.0**	**64.1**	**38.7**	**24.3**	**16.2**	**11.7**	**9.0**	**8.7**	**6.8**	**5.9**	**4.5**	**3.7**	**3.3**	**2.4**	**2.0**
Lower CI	96.3	80.0	56.1	31.4	18.8	12.1	8.6	6.2	5.9	4.5	3.8	2.6	1.9	1.6	1.0	0.7
Upper CI	99.5	90.4	71.4	46.5	30.9	21.3	15.8	12.8	12.6	10.1	9.0	7.7	6.8	6.5	5.4	5.1
Number	171	148	116	80	58	45	36	30	28	24	21	16	13	11	8	6

Table W1K-44 Age Group = 61-70 Gender = FEMALE Occupational group = NON-MANUAL Total cases = 116

Threshold dB HL≥	0	5	10	15	20	25	30	35	40	45	50	55	65	75	85	95
Prevalence	**98.5**	**92.5**	**83.5**	**56.8**	**35.4**	**30.1**	**18.8**	**17.0**	**13.7**	**10.4**	**6.3**	**6.1**	**4.6**	**3.8**	**2.6**	**1.9**
Lower CI	92.0	84.6	75.0	46.9	27.4	22.6	12.9	11.5	9.4	7.1	5.1	4.9	3.4	2.5	1.4	0.9
Upper CI	99.7	96.5	89.6	66.2	44.3	38.7	26.7	24.3	19.6	15.0	7.8	7.6	6.2	5.7	4.6	3.9
Number	115	108	96	71	51	45	37	35	31	27	22	21	15	12	9	7

Table W1K-45 Age Group = 71-80 Gender = FEMALE Occupational group = NON-MANUAL Total cases = 67

Threshold dB HL≥	0	5	10	15	20	25	30	35	40	45	50	55	65	75	85	95
Prevalence	**98.6**	**92.5**	**79.4**	**71.1**	**64.3**	**57.4**	**50.6**	**46.9**	**29.2**	**21.6**	**14.4**	**13.8**	**12.9**	**8.6**	**3.5**	**1.7**
Lower CI	92.2	71.6	62.9	62.7	53.2	47.3	42.1	38.5	21.7	14.5	9.8	9.3	8.3	4.7	1.4	0.4
Upper CI	99.7	98.4	89.7	78.4	74.0	66.9	59.1	55.6	38.0	30.8	20.6	20.1	19.3	15.2	8.3	6.8
Number	66	65	59	56	50	46	43	38	31	28	22	21	19	11	5	2

Table W1K-46 Age Group = 18-30 Gender = MALE Occupational group = NON-MANUAL Total cases = 66

Threshold dB HL≥	0	5	10	15	20	25	30	35	40	45	50	55	65	75	85	95
Prevalence	**85.7**	**48.3**	**23.6**	**8.7**	**5.3**	**1.8**	**1.8**	**0.4**	**0.4**	**0.1**	**0.1**	**0.1**	**0.1**	**0.1**	**0.0**	**0.0**
Lower CI	73.6	34.5	13.4	3.4	1.4	0.4	0.4	0.1	0.1	0.0	0.0	0.0	0.0	0.0	0.0	0.0
Upper CI	92.9	62.3	38.1	20.7	17.8	7.5	7.5	1.3	1.3	5.7	5.7	5.7	5.7	5.7	5.5	5.5
Number	57	33	18	7	4	3	3	2	2	1	1	1	1	1	0	0

Table W1K-47 Age Group = 31-40 Gender = MALE Occupational group = NON-MANUAL Total cases = 62

Threshold dB HL≥	0	5	10	15	20	25	30	35	40	45	50	55	65	75	85	95
Prevalence	**96.4**	**59.5**	**35.5**	**18.5**	**15.1**	**9.7**	**5.7**	**4.5**	**0.0**	**0.0**	**0.0**	**0.0**	**0.0**	**0.0**	**0.0**	**0.0**
Lower CI	89.8	48.3	24.8	10.1	7.5	4.1	1.8	1.2	0.0	0.0	0.0	0.0	0.0	0.0	0.0	0.0
Upper CI	98.8	69.8	48.0	31.4	27.9	21.4	16.7	15.6	5.8	5.8	5.8	5.8	5.8	5.8	5.8	5.8
Number	58	41	22	13	10	7	4	3	0	0	0	0	0	0	0	0

Table W1K-48 Age Group = 41-50 Gender = MALE Occupational group = NON-MANUAL Total cases = 76

Threshold dB HL≥	0	5	10	15	20	25	30	35	40	45	50	55	65	75	85	95
Prevalence	**91.9**	**74.5**	**43.2**	**29.4**	**21.7**	**18.7**	**15.0**	**11.9**	**7.3**	**4.2**	**4.2**	**1.4**	**1.2**	**1.2**	**0.9**	**0.9**
Lower CI	81.0	61.6	32.1	20.0	14.1	11.8	7.5	5.9	3.4	1.2	1.2	0.5	0.4	0.4	0.2	0.2
Upper CI	96.8	84.2	55.1	40.8	31.8	28.3	28.0	22.7	15.0	13.7	13.7	3.8	3.7	3.7	3.8	3.8
Number	71	59	35	24	17	14	12	11	8	5	5	4	3	3	2	2

Table W1K-49 Age Group = 51-60 Gender = MALE Occupational group = NON-MANUAL Total cases = 142

Threshold dB HL≥	0	5	10	15	20	25	30	35	40	45	50	55	65	75	85	95
Prevalence	**94.5**	**80.7**	**54.7**	**33.7**	**25.8**	**15.1**	**11.2**	**9.8**	**6.3**	**5.5**	**5.3**	**4.5**	**2.6**	**1.9**	**1.0**	**1.0**
Lower CI	89.2	72.4	45.9	26.5	19.3	10.9	7.8	6.6	3.9	3.3	3.1	2.5	1.1	0.8	0.4	0.4
Upper CI	97.3	87.0	63.2	41.8	33.6	20.5	15.8	14.2	9.9	9.1	8.8	8.2	5.7	4.6	2.6	2.6
Number	133	116	83	63	50	35	28	25	18	16	15	12	7	6	4	4

Prevalence by age group, gender and occupational group for the worse ear over 1 kHz

Table W1K-50 Age Group = 61-70 Gender = MALE Occupational group = NON-MANUAL Total cases = 111

Threshold dB HL≥	0	5	10	15	20	25	30	35	40	45	50	55	65	75	85	95
Prevalence	99.8	93.5	80.0	58.6	43.2	29.7	24.1	21.6	17.3	13.3	9.9	7.4	7.0	7.0	2.9	1.3
Lower CI	98.9	86.5	64.6	44.0	30.4	22.9	18.2	16.4	12.3	9.5	6.4	4.5	4.1	4.1	1.2	0.5
Upper CI	100.0	97.0	89.7	71.9	56.9	37.5	31.2	28.0	23.8	18.2	15.0	12.0	11.7	11.7	7.3	3.5
Number	110	102	90	67	52	40	32	30	24	19	14	11	10	10	5	3

Table W1K-51 Age Group = 71-80 Gender = MALE Occupational group = NON-MANUAL Total cases = 56

Threshold dB HL≥	0	5	10	15	20	25	30	35	40	45	50	55	65	75	85	95
Prevalence	100.0	88.0	76.5	59.8	55.1	47.2	32.9	23.0	21.1	17.2	13.7	8.5	3.1	2.1	1.0	0.0
Lower CI	93.6	73.5	61.3	47.5	43.4	36.4	22.7	15.1	13.4	11.0	8.3	5.7	1.2	0.6	0.2	0.0
Upper CI	100.0	95.1	87.0	71.0	66.2	58.2	45.0	33.3	31.6	25.9	21.9	12.6	8.1	7.5	5.3	6.4
Number	56	51	46	38	34	29	20	15	13	11	9	6	3	2	1	0

Table W1K-52 Age Group = 18-30 Gender = FEMALE Occupational group = MANUAL Total cases = 109

Threshold dB HL≥	0	5	10	15	20	25	30	35	40	45	50	55	65	75	85	95
Prevalence	88.0	66.1	45.5	21.1	11.6	6.0	4.5	4.5	3.7	2.2	2.1	0.5	0.0	0.0	0.0	0.0
Lower CI	76.8	54.4	33.6	14.1	6.6	3.1	2.1	2.1	1.6	0.8	0.7	0.1	0.0	0.0	0.0	0.0
Upper CI	94.2	76.2	57.9	30.2	19.5	11.3	9.3	9.3	8.5	6.0	5.9	1.7	3.4	3.4	3.4	3.4
Number	99	77	53	33	19	13	10	10	8	6	5	2	0	0	0	0

Table W1K-53 Age Group = 31-40 Gender = FEMALE Occupational group = MANUAL Total cases = 103

Threshold dB HL≥	0	5	10	15	20	25	30	35	40	45	50	55	65	75	85	95
Prevalence	86.9	63.6	31.1	18.5	11.2	9.3	7.1	5.8	4.5	2.9	2.9	2.6	1.7	0.8	0.2	0.0
Lower CI	75.7	50.8	22.0	12.5	7.0	5.3	3.9	2.9	2.0	1.4	1.4	1.2	0.7	0.3	0.0	0.0
Upper CI	93.4	74.7	42.0	26.7	17.5	15.7	12.5	11.3	10.1	6.0	6.0	5.7	4.2	2.3	3.9	3.6
Number	93	74	48	34	25	19	15	11	9	8	8	7	5	3	1	0

Table W1K-54 Age Group = 41-50 Gender = FEMALE Occupational group = MANUAL Total cases = 135

Threshold dB HL≥	0	5	10	15	20	25	30	35	40	45	50	55	65	75	85	95
Prevalence	93.0	79.7	64.1	37.2	20.1	16.2	10.8	9.4	6.7	6.7	5.6	4.9	2.9	1.7	1.7	1.0
Lower CI	85.6	70.3	54.4	28.6	13.8	10.7	6.9	5.8	4.0	4.0	3.5	3.1	1.8	0.9	0.9	0.4
Upper CI	96.8	86.7	72.7	46.8	28.5	23.9	16.4	15.0	11.0	11.0	8.8	7.7	4.8	3.0	3.0	2.6
Number	129	114	90	60	37	32	27	24	18	18	17	16	10	5	5	4

Table W1K-55 Age Group = 51-60 Gender = FEMALE Occupational group = MANUAL Total cases = 166

Threshold dB HL≥	0	5	10	15	20	25	30	35	40	45	50	55	65	75	85	95
Prevalence	96.2	83.8	67.0	46.3	30.4	23.9	18.8	13.4	10.1	6.0	4.0	3.3	1.8	1.0	0.5	0.3
Lower CI	90.1	75.8	57.8	38.2	23.4	17.5	13.1	9.0	6.4	3.4	2.3	2.0	0.8	0.5	0.2	0.1
Upper CI	98.6	89.5	75.0	54.5	38.4	31.7	26.1	19.5	15.5	10.4	7.0	5.7	4.0	1.9	1.2	1.1
Number	162	149	126	98	72	58	46	34	28	21	18	17	9	7	3	2

Table W1K-56 Age Group = 61-70 Gender = FEMALE Occupational group = MANUAL Total cases = 125

Threshold dB HL≥	0	5	10	15	20	25	30	35	40	45	50	55	65	75	85	95
Prevalence	99.6	96.9	74.3	57.9	45.0	34.4	26.5	23.5	14.8	12.8	12.1	11.4	7.5	5.1	2.4	1.9
Lower CI	97.6	92.9	63.6	47.7	35.9	26.3	20.4	18.5	9.9	8.2	7.6	7.0	4.9	2.9	1.8	1.4
Upper CI	99.9	98.7	82.7	67.6	54.4	43.5	33.8	29.5	21.5	19.4	18.7	18.0	11.4	9.0	3.2	2.4
Number	124	119	101	82	68	57	49	46	33	29	27	25	18	10	4	2

Prevalence by age group, gender and occupational group for the worse ear over 1 kHz

Table W1K-57 Age Group = 71-80 Gender = FEMALE Occupational group = MANUAL Total cases = 74

Threshold dB HL≥	0	5	10	15	20	25	30	35	40	45	50	55	65	75	85	95
**Prevalence**	**100.0**	**95.7**	**95.7**	**90.8**	**76.0**	**50.3**	**48.9**	**42.7**	**38.8**	**28.1**	**16.8**	**13.0**	**6.8**	**4.4**	**2.6**	**2.3**
**Lower CI**	95.1	78.7	78.7	74.2	63.5	37.6	36.2	30.9	26.9	17.2	13.0	9.1	4.0	2.3	1.3	1.0
**Upper CI**	100.0	99.3	99.3	97.2	85.2	63.0	61.7	55.3	52.1	42.4	21.5	18.3	11.2	8.2	5.4	5.0
**Number**	74	73	73	70	63	54	52	47	43	34	29	23	13	9	6	5

Table W1K-58 Age Group = 18-30 Gender = MALE Occupational group = MANUAL Total cases = 109

Threshold dB HL≥	0	5	10	15	20	25	30	35	40	45	50	55	65	75	85	95
**Prevalence**	**84.3**	**60.3**	**30.0**	**17.6**	**8.8**	**4.5**	**2.8**	**2.5**	**1.4**	**1.0**	**0.5**	**0.5**	**0.3**	**0.1**	**0.1**	**0.1**
**Lower CI**	72.7	48.3	21.0	10.5	3.9	2.0	1.3	1.1	0.7	0.4	0.2	0.2	0.1	0.0	0.0	0.0
**Upper CI**	91.6	71.1	40.8	27.8	18.7	9.7	5.9	5.6	2.8	2.3	1.6	1.6	1.5	0.4	0.4	0.4
**Number**	97	73	47	33	19	13	11	9	7	5	3	3	2	1	1	1

Table W1K-59 Age Group = 31-40 Gender = MALE Occupational group = MANUAL Total cases = 80

Threshold dB HL≥	0	5	10	15	20	25	30	35	40	45	50	55	65	75	85	95
**Prevalence**	**93.7**	**66.1**	**43.6**	**30.2**	**21.0**	**13.0**	**8.3**	**5.0**	**3.6**	**3.4**	**3.1**	**2.3**	**1.9**	**1.9**	**1.9**	**1.9**
**Lower CI**	79.7	50.1	29.9	19.0	12.2	6.5	3.4	2.2	1.3	1.1	0.9	0.5	0.3	0.3	0.3	0.3
**Upper CI**	98.3	79.1	58.3	44.5	33.8	24.3	18.7	11.1	10.1	10.0	9.9	9.9	10.2	10.2	10.2	10.2
**Number**	77	58	40	31	25	16	11	9	6	5	4	2	1	1	1	1

Table W1K-60 Age Group = 41-50 Gender = MALE Occupational group = MANUAL Total cases = 127

Threshold dB HL≥	0	5	10	15	20	25	30	35	40	45	50	55	65	75	85	95
**Prevalence**	**99.3**	**79.7**	**60.1**	**37.2**	**24.7**	**20.3**	**14.9**	**12.3**	**9.5**	**7.6**	**7.2**	**6.2**	**1.1**	**0.9**	**0.0**	**0.0**
**Lower CI**	96.4	69.2	49.4	28.2	18.1	14.3	10.2	8.1	5.7	4.2	3.8	3.1	0.5	0.3	0.0	0.0
**Upper CI**	99.9	87.3	69.9	47.1	32.8	28.0	21.3	18.2	15.7	13.5	13.1	12.3	2.5	2.7	2.9	2.9
**Number**	126	108	82	61	46	39	30	25	20	15	13	11	4	3	0	0

Table W1K-61 Age Group = 51-60 Gender = MALE Occupational group = MANUAL Total cases = 199

Threshold dB HL≥	0	5	10	15	20	25	30	35	40	45	50	55	65	75	85	95
**Prevalence**	**98.0**	**88.5**	**71.8**	**49.6**	**38.3**	**23.4**	**18.9**	**15.1**	**12.3**	**11.6**	**9.4**	**8.9**	**5.6**	**3.2**	**2.5**	**1.6**
**Lower CI**	93.0	81.2	63.4	42.0	31.2	18.3	14.7	11.4	9.0	8.3	6.5	6.2	3.3	1.7	1.2	0.6
**Upper CI**	99.4	93.2	78.9	57.3	46.0	29.4	23.8	19.7	16.8	16.0	13.3	12.7	9.3	5.9	5.2	3.9
**Number**	196	184	153	120	98	71	61	50	40	37	32	31	19	12	9	6

Table W1K-62 Age Group = 61-70 Gender = MALE Occupational group = MANUAL Total cases = 173

Threshold dB HL≥	0	5	10	15	20	25	30	35	40	45	50	55	65	75	85	95
**Prevalence**	**98.8**	**88.7**	**67.6**	**60.8**	**48.2**	**37.3**	**31.9**	**27.2**	**21.7**	**16.8**	**14.2**	**11.1**	**8.5**	**6.5**	**2.6**	**1.3**
**Lower CI**	93.3	77.6	56.4	50.0	38.6	29.1	24.2	19.9	15.4	11.4	9.1	6.7	4.5	2.9	1.4	0.5
**Upper CI**	99.8	94.7	77.1	70.6	57.9	46.3	40.9	35.9	29.7	24.1	21.5	17.8	15.5	13.8	4.6	3.3
**Number**	172	163	139	125	106	87	77	68	57	48	38	33	24	17	11	5

Table W1K-63 Age Group = 71-80 Gender = MALE Occupational group = MANUAL Total cases = 72

Threshold dB HL≥	0	5	10	15	20	25	30	35	40	45	50	55	65	75	85	95
**Prevalence**	**100.0**	**100.0**	**91.5**	**84.4**	**81.2**	**53.8**	**46.0**	**33.4**	**32.7**	**28.6**	**25.2**	**21.2**	**15.5**	**10.3**	**4.8**	**3.3**
**Lower CI**	94.9	94.9	73.2	65.6	62.8	39.7	32.4	25.3	24.6	20.5	17.5	14.3	9.5	6.1	2.4	1.3
**Upper CI**	100.0	100.0	97.7	93.9	91.7	67.2	60.2	42.6	42.0	38.5	34.9	30.2	24.2	16.8	9.6	8.1
**Number**	72	72	67	63	59	52	47	40	39	35	32	28	19	13	8	5

Prevalence by age group, gender and occupational group for the worse ear over 2 kHz

Table W2K-1 Age Group = 18-80 Gender = OVERALL Occupational group = OVERALL Total cases = 2660

	Threshold dB HL≥															
	0	5	10	15	20	25	30	35	40	45	50	55	65	75	85	95
Prevalence	93.3	78.6	59.5	43.3	32.4	25.7	20.2	16.1	12.8	10.4	8.2	6.7	4.0	2.5	1.6	1.0
Lower CI	91.7	76.5	57.3	41.4	30.7	24.2	18.9	15.0	11.8	9.6	7.4	6.0	3.5	2.1	1.3	0.8
Upper CI	94.5	80.6	61.6	45.3	34.2	27.3	21.6	17.4	13.9	11.3	9.0	7.4	4.6	2.9	2.0	1.3
Number	2552	2276	1886	1528	1238	1049	888	763	647	550	454	387	254	164	106	71

Table W2K-2 Age Group = 18-80 Gender = FEMALE Occupational group = OVERALL Total cases = 1388

	Threshold dB HL≥															
	0	5	10	15	20	25	30	35	40	45	50	55	65	75	85	95
Prevalence	94.8	79.6	57.6	42.2	30.4	23.6	18.1	14.2	11.0	8.7	6.6	5.3	3.3	1.8	1.2	0.8
Lower CI	93.0	76.9	54.8	39.6	28.2	21.6	16.2	12.6	9.6	7.6	5.7	4.5	2.6	1.4	0.9	0.6
Upper CI	96.1	82.0	60.4	44.8	32.7	25.8	20.0	16.0	12.5	9.9	7.6	6.3	4.0	2.2	1.7	1.2
Number	1340	1182	940	754	587	482	394	334	280	239	190	163	109	67	46	31

Table W2K-3 Age Group = 18-80 Gender = MALE Occupational group = OVERALL Total cases = 1272

	Threshold dB HL≥															
	0	5	10	15	20	25	30	35	40	45	50	55	65	75	85	95
Prevalence	91.5	77.4	61.6	44.6	34.7	28.1	22.6	18.2	14.9	12.3	10.0	8.2	4.8	3.3	2.0	1.2
Lower CI	88.8	74.0	58.1	41.7	32.2	25.8	20.7	16.7	13.5	11.0	8.8	7.1	4.0	2.6	1.5	0.8
Upper CI	93.6	80.5	64.9	47.6	37.4	30.4	24.6	19.9	16.4	13.7	11.4	9.4	5.8	4.2	2.8	1.8
Number	1212	1094	946	774	651	567	494	429	367	311	264	224	145	97	60	40

Table W2K-4 Age Group = 18-30 Gender = OVERALL Occupational group = OVERALL Total cases = 401

	Threshold dB HL≥															
	0	5	10	15	20	25	30	35	40	45	50	55	65	75	85	95
Prevalence	86.2	62.1	35.7	16.6	7.8	5.0	3.7	2.6	1.6	1.2	0.5	0.3	0.2	0.0	0.0	0.0
Lower CI	81.5	56.2	30.4	13.0	5.6	3.4	2.3	1.7	1.0	0.7	0.2	0.1	0.0	0.0	0.0	0.0
Upper CI	89.8	67.6	41.3	20.9	10.7	7.4	5.9	4.1	2.7	2.2	1.1	0.8	0.6	0.1	0.1	0.1
Number	353	265	162	89	54	38	29	25	18	13	7	5	3	2	1	1

Table W2K-5 Age Group = 31-40 Gender = OVERALL Occupational group = OVERALL Total cases = 332

	Threshold dB HL≥															
	0	5	10	15	20	25	30	35	40	45	50	55	65	75	85	95
Prevalence	89.2	65.7	40.4	25.3	17.3	12.0	8.9	5.6	4.2	3.3	2.2	1.8	1.2	0.9	0.6	0.6
Lower CI	84.2	59.2	34.2	20.3	13.0	8.6	6.2	3.7	2.7	2.0	1.2	0.9	0.4	0.2	0.1	0.1
Upper CI	92.8	71.6	46.9	31.1	22.6	16.6	12.6	8.4	6.5	5.4	4.0	3.6	3.2	3.0	3.0	3.1
Number	303	233	159	113	75	58	45	34	28	24	16	13	7	4	2	1

Table W2K-6 Age Group = 41-50 Gender = OVERALL Occupational group = OVERALL Total cases = 450

	Threshold dB HL≥															
	0	5	10	15	20	25	30	35	40	45	50	55	65	75	85	95
Prevalence	95.1	81.3	59.8	40.5	27.6	18.4	14.7	11.1	8.5	6.0	4.8	3.8	2.6	1.6	1.1	0.8
Lower CI	92.3	76.8	54.8	35.7	23.6	15.3	12.0	8.7	6.5	4.4	3.4	2.6	1.7	0.9	0.6	0.4
Upper CI	96.9	85.1	64.7	45.4	32.1	22.0	17.9	13.9	11.0	8.1	6.6	5.5	4.0	2.8	1.8	1.5
Number	432	381	294	213	157	118	98	79	64	47	39	32	24	16	13	10

Table W2K-7 Age Group = 51-60 Gender = OVERALL Occupational group = OVERALL Total cases = 679

	Threshold dB HL≥															
	0	5	10	15	20	25	30	35	40	45	50	55	65	75	85	95
Prevalence	98.0	89.3	73.5	55.6	40.9	32.2	23.4	18.8	13.6	11.9	9.2	7.9	5.2	3.4	1.7	1.4
Lower CI	96.3	86.3	69.6	51.5	37.0	28.6	20.4	16.1	11.5	9.9	7.5	6.3	3.9	2.3	1.0	0.7
Upper CI	98.9	91.6	77.1	59.7	44.9	36.0	26.6	21.7	16.1	14.2	11.2	9.8	6.9	4.9	2.9	2.4
Number	669	616	529	431	339	282	226	190	152	135	110	93	65	42	22	17

Prevalence by age group, gender and occupational group for the worse ear over 2 kHz

Table W2K-8 Age Group = 61-70 Gender = OVERALL Occupational group = OVERALL Total cases = 525

Threshold dB HL≥	0	5	10	15	20	25	30	35	40	45	50	55	65	75	85	95
**Prevalence**	**99.7**	**97.5**	**87.0**	**74.9**	**61.7**	**51.0**	**39.7**	**32.3**	**26.8**	**20.2**	**16.9**	**14.3**	**8.7**	**5.3**	**3.7**	**1.8**
**Lower CI**	98.8	95.6	82.7	69.9	56.5	46.1	35.0	28.1	23.1	17.2	14.0	11.8	6.7	3.9	2.5	1.2
**Upper CI**	99.9	98.6	90.3	79.3	66.7	55.9	44.6	36.9	30.7	23.6	20.3	17.4	11.3	7.0	5.4	2.7
**Number**	523	512	476	424	371	324	278	242	216	178	150	133	84	54	36	21

Table W2K-9 Age Group = 71-80 Gender = OVERALL Occupational group = OVERALL Total cases = 273

Threshold dB HL≥	0	5	10	15	20	25	30	35	40	45	50	55	65	75	85	95
**Prevalence**	**99.6**	**97.5**	**95.8**	**88.4**	**79.9**	**73.1**	**63.7**	**54.0**	**45.4**	**39.9**	**32.1**	**24.9**	**13.2**	**7.7**	**5.4**	**3.1**
**Lower CI**	98.0	93.5	90.0	81.7	72.1	64.9	55.9	46.9	38.7	34.3	26.8	20.5	10.3	5.9	3.9	2.0
**Upper CI**	99.9	99.1	98.3	92.8	86.0	79.9	70.8	61.0	52.4	45.8	38.0	30.0	16.7	10.1	7.5	4.6
**Number**	272	269	266	258	242	229	212	193	169	153	132	111	71	46	32	21

Table W2K-10 Age Group = 18-80 Gender = OVERALL Occupational group = NON-MANUAL Total cases = 1183

Threshold dB HL≥	0	5	10	15	20	25	30	35	40	45	50	55	65	75	85	95
**Prevalence**	**91.8**	**75.5**	**55.1**	**39.7**	**29.5**	**23.8**	**19.3**	**15.4**	**12.1**	**8.7**	**6.6**	**5.0**	**2.8**	**2.0**	**1.3**	**0.9**
**Lower CI**	89.2	72.3	51.9	37.2	27.3	21.8	17.7	13.8	10.7	7.8	5.7	4.3	2.3	1.5	0.9	0.6
**Upper CI**	93.8	78.5	58.3	42.3	31.8	26.0	21.1	17.0	13.5	9.8	7.6	5.9	3.4	2.6	1.8	1.4
**Number**	1124	972	779	616	480	398	342	289	235	194	152	127	85	60	38	26

Table W2K-11 Age Group = 18-80 Gender = OVERALL Occupational group = MANUAL Total cases = 1472

Threshold dB HL≥	0	5	10	15	20	25	30	35	40	45	50	55	65	75	85	95
**Prevalence**	**94.6**	**81.1**	**63.2**	**46.1**	**34.6**	**26.9**	**21.1**	**16.6**	**13.3**	**11.1**	**9.0**	**7.4**	**4.6**	**2.7**	**1.9**	**1.1**
**Lower CI**	92.5	77.9	59.8	43.0	32.0	24.7	19.1	15.0	12.0	9.9	7.9	6.4	3.8	2.2	1.4	0.8
**Upper CI**	96.1	83.9	66.5	49.3	37.2	29.3	23.2	18.3	14.7	12.4	10.2	8.5	5.5	3.5	2.5	1.6
**Number**	1424	1301	1104	909	755	648	544	473	412	356	302	260	169	104	68	45

Table W2K-12 Age Group = 18-30 Gender = FEMALE Occupational group = OVERALL Total cases = 224

Threshold dB HL≥	0	5	10	15	20	25	30	35	40	45	50	55	65	75	85	95
**Prevalence**	**89.0**	**65.2**	**35.7**	**18.2**	**7.3**	**4.8**	**3.6**	**3.5**	**2.4**	**1.7**	**0.7**	**0.5**	**0.2**	**0.0**	**0.0**	**0.0**
**Lower CI**	83.2	57.8	29.1	13.5	4.9	3.0	2.2	2.0	1.3	0.8	0.3	0.2	0.0	0.0	0.0	0.0
**Upper CI**	92.9	72.0	42.8	24.1	10.8	7.6	6.0	5.9	4.4	3.6	1.9	1.6	1.3	1.7	1.7	1.7
**Number**	203	155	92	53	28	20	17	16	12	8	4	3	1	0	0	0

Table W2K-13 Age Group = 31-40 Gender = FEMALE Occupational group = OVERALL Total cases = 190

Threshold dB HL≥	0	5	10	15	20	25	30	35	40	45	50	55	65	75	85	95
**Prevalence**	**91.3**	**61.4**	**34.5**	**21.3**	**14.8**	**10.8**	**9.2**	**7.0**	**4.6**	**3.3**	**2.1**	**1.6**	**0.8**	**0.6**	**0.1**	**0.0**
**Lower CI**	85.9	53.2	27.4	16.0	10.7	7.4	5.9	4.3	2.7	1.8	1.1	0.8	0.3	0.2	0.0	0.0
**Upper CI**	94.8	69.1	42.3	27.8	20.2	15.7	14.0	11.4	7.8	5.8	3.8	3.2	2.2	1.9	0.5	2.0
**Number**	176	125	83	61	43	33	25	20	16	13	10	8	4	3	1	0

Table W2K-14 Age Group = 41-50 Gender = FEMALE Occupational group = OVERALL Total cases = 247

Threshold dB HL≥	0	5	10	15	20	25	30	35	40	45	50	55	65	75	85	95
**Prevalence**	**94.9**	**81.9**	**54.9**	**37.6**	**24.8**	**14.7**	**11.5**	**7.5**	**5.0**	**3.7**	**2.8**	**2.7**	**2.0**	**0.9**	**0.7**	**0.4**
**Lower CI**	90.9	75.8	48.1	31.6	19.8	11.0	8.3	5.2	3.3	2.4	1.7	1.6	1.1	0.4	0.3	0.1
**Upper CI**	97.2	86.7	61.4	44.1	30.6	19.4	15.6	10.7	7.5	5.7	4.6	4.5	3.5	2.0	1.7	1.1
**Number**	237	211	153	110	78	52	43	33	25	19	15	14	11	6	5	3

Prevalence by age group, gender and occupational group for the worse ear over 2 kHz

Table W2K-15 Age Group = 51-60 Gender = FEMALE Occupational group = OVERALL Total cases = 341

	Threshold dB HL≥															
	**0**	**5**	**10**	**15**	**20**	**25**	**30**	**35**	**40**	**45**	**50**	**55**	**65**	**75**	**85**	**95**
**Prevalence**	**99.4**	**91.1**	**72.1**	**55.4**	**36.9**	**27.6**	**18.6**	**14.3**	**8.7**	**7.4**	**4.8**	**4.0**	**3.4**	**2.0**	**1.5**	**1.3**
**Lower CI**	97.6	87.3	66.5	49.5	31.5	22.8	14.8	11.0	6.4	5.4	3.2	2.6	2.1	1.0	0.7	0.5
**Upper CI**	99.8	93.9	77.0	61.2	42.6	32.9	23.0	18.4	11.7	10.1	6.9	6.2	5.5	3.6	3.3	3.1
**Number**	339	314	261	213	156	125	95	78	56	50	37	31	26	16	10	8

Table W2K-16 Age Group = 61-70 Gender = FEMALE Occupational group = OVERALL Total cases = 242

	Threshold dB HL≥															
	**0**	**5**	**10**	**15**	**20**	**25**	**30**	**35**	**40**	**45**	**50**	**55**	**65**	**75**	**85**	**95**
**Prevalence**	**100.0**	**97.5**	**83.1**	**69.8**	**54.7**	**43.9**	**31.7**	**24.9**	**20.9**	**15.1**	**13.4**	**10.7**	**6.3**	**3.4**	**2.5**	**1.6**
**Lower CI**	98.4	94.5	76.4	62.5	47.5	37.3	25.6	19.5	15.9	11.1	9.6	7.4	3.9	2.4	1.5	0.9
**Upper CI**	100.0	98.9	88.2	76.2	61.7	50.7	38.5	31.3	27.0	20.3	18.6	15.1	10.1	4.8	4.1	2.9
**Number**	242	235	212	185	153	130	103	86	79	64	55	48	30	19	14	9

Table W2K-17 Age Group = 71-80 Gender = FEMALE Occupational group = OVERALL Total cases = 144

	Threshold dB HL≥															
	**0**	**5**	**10**	**15**	**20**	**25**	**30**	**35**	**40**	**45**	**50**	**55**	**65**	**75**	**85**	**95**
**Prevalence**	**99.4**	**97.9**	**95.0**	**83.2**	**78.2**	**70.5**	**59.9**	**48.3**	**41.6**	**35.5**	**27.5**	**21.7**	**12.2**	**6.4**	**4.6**	**2.8**
**Lower CI**	96.8	91.8	85.8	73.1	65.9	58.6	48.7	38.3	32.5	28.7	21.6	16.8	8.4	4.4	2.9	1.6
**Upper CI**	99.9	99.5	98.3	90.1	86.9	80.2	70.3	58.5	51.4	42.9	34.3	27.6	17.4	9.2	7.1	4.9
**Number**	143	142	139	132	129	122	111	101	92	85	69	59	37	23	16	11

Table W2K-18 Age Group = 18-30 Gender = MALE Occupational group = OVERALL Total cases = 177

	Threshold dB HL≥															
	**0**	**5**	**10**	**15**	**20**	**25**	**30**	**35**	**40**	**45**	**50**	**55**	**65**	**75**	**85**	**95**
**Prevalence**	**83.1**	**58.4**	**35.7**	**14.7**	**8.2**	**5.1**	**3.6**	**1.7**	**0.8**	**0.6**	**0.3**	**0.1**	**0.1**	**0.1**	**0.0**	**0.0**
**Lower CI**	75.2	49.4	27.5	9.7	4.9	2.7	1.7	0.7	0.4	0.2	0.1	0.0	0.0	0.0	0.0	0.0
**Upper CI**	88.9	66.9	44.7	21.6	13.2	9.2	7.7	3.6	1.8	1.6	1.0	0.2	0.2	0.2	0.2	0.2
**Number**	150	110	70	36	26	18	12	9	6	5	3	2	2	2	1	1

Table W2K-19 Age Group = 31-40 Gender = MALE Occupational group = OVERALL Total cases = 142

	Threshold dB HL≥															
	**0**	**5**	**10**	**15**	**20**	**25**	**30**	**35**	**40**	**45**	**50**	**55**	**65**	**75**	**85**	**95**
**Prevalence**	**86.8**	**70.5**	**47.0**	**29.9**	**20.0**	**13.4**	**8.6**	**4.1**	**3.8**	**3.5**	**2.3**	**2.0**	**1.6**	**1.2**	**1.2**	**1.2**
**Lower CI**	77.5	59.9	36.8	21.4	12.9	7.9	4.8	2.1	1.8	1.6	0.8	0.6	0.4	0.2	0.2	0.2
**Upper CI**	92.6	79.2	57.5	40.1	29.7	21.9	15.0	8.1	7.8	7.4	6.5	6.3	6.3	6.5	6.5	6.5
**Number**	127	108	76	52	32	25	20	14	12	11	6	5	3	1	1	1

Table W2K-20 Age Group = 41-50 Gender = MALE Occupational group = OVERALL Total cases = 203

	Threshold dB HL≥															
	**0**	**5**	**10**	**15**	**20**	**25**	**30**	**35**	**40**	**45**	**50**	**55**	**65**	**75**	**85**	**95**
**Prevalence**	**95.3**	**80.6**	**65.3**	**43.6**	**30.7**	**22.4**	**18.3**	**15.0**	**12.4**	**8.4**	**6.8**	**4.9**	**3.2**	**2.4**	**1.4**	**1.3**
**Lower CI**	90.9	73.6	57.7	36.2	24.5	17.5	14.0	11.0	8.9	5.6	4.4	2.9	1.8	1.2	0.7	0.6
**Upper CI**	97.6	86.0	72.2	51.3	37.7	28.2	23.4	20.0	16.9	12.5	10.5	8.2	5.8	4.8	2.8	2.7
**Number**	195	170	141	103	79	66	55	46	39	28	24	18	13	10	8	7

Table W2K-21 Age Group = 51-60 Gender = MALE Occupational group = OVERALL Total cases = 338

	Threshold dB HL≥															
	**0**	**5**	**10**	**15**	**20**	**25**	**30**	**35**	**40**	**45**	**50**	**55**	**65**	**75**	**85**	**95**
**Prevalence**	**96.4**	**87.1**	**75.0**	**55.8**	**45.4**	**37.4**	**28.7**	**23.7**	**19.1**	**16.8**	**14.1**	**12.1**	**7.2**	**5.0**	**2.0**	**1.5**
**Lower CI**	93.0	82.3	69.3	49.9	39.9	32.2	24.3	19.7	15.6	13.5	11.0	9.3	5.1	3.1	1.0	0.7
**Upper CI**	98.2	90.8	79.9	61.5	51.0	42.8	33.5	28.2	23.2	20.8	17.8	15.7	10.2	7.8	3.9	3.2
**Number**	330	302	268	218	183	157	131	112	96	85	73	62	39	26	12	9

Prevalence by age group, gender and occupational group for the worse ear over 2 kHz

Table W2K-22 Age Group = 61-70 Gender = MALE Occupational group = OVERALL Total cases = 283

Threshold dB HL≥	0	5	10	15	20	25	30	35	40	45	50	55	65	75	85	95
**Prevalence**	**99.3**	**97.6**	**91.5**	**80.8**	**69.8**	**59.1**	**49.1**	**40.9**	**33.5**	**26.0**	**21.0**	**18.7**	**11.5**	**7.5**	**5.0**	**2.0**
**Lower CI**	97.4	94.5	86.4	74.0	62.2	51.7	42.1	34.6	28.7	21.9	17.1	15.0	8.5	5.0	2.9	1.1
**Upper CI**	99.8	99.0	94.8	86.2	76.5	66.1	56.2	47.5	38.8	30.7	25.4	23.0	15.4	11.0	8.5	3.5
**Number**	281	277	264	239	218	194	175	156	137	114	95	85	54	35	22	12

Table W2K-23 Age Group = 71-80 Gender = MALE Occupational group = OVERALL Total cases = 129

Threshold dB HL≥	0	5	10	15	20	25	30	35	40	45	50	55	65	75	85	95
**Prevalence**	**100.0**	**96.7**	**96.7**	**95.9**	**81.6**	**76.2**	**68.2**	**62.1**	**50.7**	**46.1**	**39.2**	**30.0**	**14.9**	**10.0**	**6.8**	**3.6**
**Lower CI**	97.1	88.4	88.4	87.8	72.8	65.8	58.6	53.0	41.5	37.0	29.8	22.2	11.0	6.7	4.1	2.0
**Upper CI**	100.0	99.1	99.1	98.7	88.0	84.3	76.4	70.4	60.0	55.4	49.4	39.2	19.9	14.5	10.9	6.4
**Number**	129	127	127	126	113	107	101	92	77	68	63	52	34	23	16	10

Table W2K-24 Age Group = 18-30 Gender = OVERALL Occupational group = NON-MANUAL Total cases = 181

Threshold dB HL≥	0	5	10	15	20	25	30	35	40	45	50	55	65	75	85	95
**Prevalence**	**82.7**	**53.9**	**25.3**	**7.9**	**3.8**	**2.6**	**2.0**	**1.7**	**1.0**	**0.7**	**0.3**	**0.3**	**0.3**	**0.1**	**0.0**	**0.0**
**Lower CI**	74.7	45.2	18.6	5.1	2.2	1.5	1.1	0.8	0.3	0.2	0.1	0.1	0.1	0.0	0.0	0.0
**Upper CI**	88.6	62.4	33.5	11.9	6.7	4.5	3.7	3.5	2.6	2.5	1.0	1.0	1.0	2.2	2.1	2.1
**Number**	156	106	54	24	14	11	9	8	5	3	2	2	2	1	0	0

Table W2K-25 Age Group = 31-40 Gender = OVERALL Occupational group = NON-MANUAL Total cases = 148

Threshold dB HL≥	0	5	10	15	20	25	30	35	40	45	50	55	65	75	85	95
**Prevalence**	**85.3**	**62.0**	**37.8**	**22.2**	**13.4**	**10.1**	**7.8**	**5.2**	**3.4**	**2.3**	**0.7**	**0.4**	**0.3**	**0.3**	**0.0**	**0.0**
**Lower CI**	76.6	52.1	28.7	15.1	8.6	6.0	4.3	3.0	1.8	1.1	0.2	0.1	0.1	0.1	0.0	0.0
**Upper CI**	91.1	71.0	47.7	31.3	20.3	16.6	13.5	9.1	6.2	4.7	2.3	1.6	1.7	1.7	2.5	2.5
**Number**	129	95	63	40	25	20	16	13	9	7	3	2	1	1	0	0

Table W2K-26 Age Group = 41-50 Gender = OVERALL Occupational group = NON-MANUAL Total cases = 188

Threshold dB HL≥	0	5	10	15	20	25	30	35	40	45	50	55	65	75	85	95
**Prevalence**	**95.7**	**83.7**	**57.6**	**38.5**	**25.8**	**17.2**	**14.9**	**10.8**	**7.7**	**4.5**	**3.9**	**2.1**	**1.7**	**1.5**	**1.5**	**1.4**
**Lower CI**	91.7	77.6	50.1	31.7	20.1	12.8	10.9	7.0	4.4	2.4	2.0	1.3	1.0	0.8	0.8	0.7
**Upper CI**	97.8	88.4	64.9	45.9	32.6	22.7	20.1	16.2	13.3	8.3	7.5	3.3	2.9	2.6	2.6	2.5
**Number**	179	156	113	80	53	36	31	25	18	11	10	8	6	5	5	4

Table W2K-27 Age Group = 51-60 Gender = OVERALL Occupational group = NON-MANUAL Total cases = 317

Threshold dB HL≥	0	5	10	15	20	25	30	35	40	45	50	55	65	75	85	95
**Prevalence**	**98.1**	**86.1**	**70.1**	**53.0**	**35.8**	**28.9**	**22.9**	**15.7**	**11.0**	**8.5**	**5.6**	**4.7**	**4.0**	**2.6**	**1.5**	**1.2**
**Lower CI**	95.5	81.4	64.3	47.1	30.7	24.3	19.1	11.7	7.7	6.4	4.0	3.3	2.6	1.5	0.7	0.5
**Upper CI**	99.2	89.8	75.3	58.7	41.3	34.0	27.2	20.6	15.6	11.1	7.6	6.8	6.0	4.5	3.1	2.8
**Number**	312	274	227	182	134	111	94	74	56	50	37	31	26	17	10	7

Table W2K-28 Age Group = 61-70 Gender = OVERALL Occupational group = NON-MANUAL Total cases = 226

Threshold dB HL≥	0	5	10	15	20	25	30	35	40	45	50	55	65	75	85	95
**Prevalence**	**100.0**	**97.7**	**88.6**	**77.7**	**62.6**	**51.3**	**40.8**	**34.5**	**26.2**	**19.2**	**15.1**	**11.8**	**6.0**	**4.9**	**3.6**	**2.5**
**Lower CI**	98.3	93.9	82.3	70.9	53.9	43.1	33.5	27.7	21.4	15.7	11.6	8.9	4.1	3.1	1.9	1.2
**Upper CI**	100.0	99.1	92.9	83.4	70.6	59.5	48.4	42.0	31.6	23.3	19.6	15.4	8.8	7.8	6.6	5.3
**Number**	226	221	204	177	150	125	106	91	79	64	52	45	27	22	15	11

Prevalence by age group, gender and occupational group for the worse ear over 2 kHz

Table W2K-29 Age Group = 71-80 Gender = OVERALL Occupational group = NON-MANUAL Total cases = 123

	Threshold dB HL≥ 0	5	10	15	20	25	30	35	40	45	50	55	65	75	85	95
**Prevalence**	**99.1**	**96.7**	**92.8**	**87.2**	**80.4**	**72.5**	**61.2**	**52.5**	**46.8**	**34.8**	**28.4**	**22.8**	**9.3**	**5.8**	**3.4**	**1.8**
**Lower CI**	95.1	90.3	79.8	76.5	69.9	61.0	53.9	47.0	40.9	29.7	22.9	18.0	6.6	3.6	1.8	0.7
**Upper CI**	99.8	99.0	97.6	93.4	87.8	81.7	68.0	57.9	52.7	40.2	34.6	28.4	12.9	9.2	6.0	4.8
**Number**	122	120	118	113	104	95	86	78	68	59	48	39	23	14	8	4

Table W2K-30 Age Group = 18-30 Gender = OVERALL Occupational group = MANUAL Total cases = 218

	Threshold dB HL≥ 0	5	10	15	20	25	30	35	40	45	50	55	65	75	85	95
**Prevalence**	**89.3**	**67.7**	**45.7**	**24.5**	**10.1**	**6.5**	**4.9**	**3.3**	**2.1**	**1.6**	**0.5**	**0.3**	**0.0**	**0.0**	**0.0**	**0.0**
**Lower CI**	82.9	58.7	37.1	17.9	6.8	3.9	2.7	1.8	1.0	0.7	0.2	0.1	0.0	0.0	0.0	0.0
**Upper CI**	93.5	75.6	54.7	32.5	15.0	10.6	8.8	6.0	4.1	3.5	1.3	0.9	0.2	0.2	0.2	0.2
**Number**	196	159	108	65	40	27	20	17	13	10	5	3	1	1	1	1

Table W2K-31 Age Group = 31-40 Gender = OVERALL Occupational group = MANUAL Total cases = 183

	Threshold dB HL≥ 0	5	10	15	20	25	30	35	40	45	50	55	65	75	85	95
**Prevalence**	**91.6**	**68.5**	**41.8**	**27.7**	**20.6**	**12.9**	**10.0**	**5.0**	**4.9**	**4.2**	**3.0**	**2.5**	**1.6**	**1.2**	**1.0**	**0.9**
**Lower CI**	84.2	59.3	32.9	20.3	14.1	8.0	5.8	2.8	2.7	2.2	1.5	1.1	0.5	0.3	0.2	0.2
**Upper CI**	95.7	76.5	51.2	36.6	29.1	20.2	16.5	8.8	8.6	7.9	6.0	5.5	4.8	4.8	4.9	5.0
**Number**	173	137	95	72	49	37	28	20	19	17	13	11	6	3	2	1

Table W2K-32 Age Group = 41-50 Gender = OVERALL Occupational group = MANUAL Total cases = 262

	Threshold dB HL≥ 0	5	10	15	20	25	30	35	40	45	50	55	65	75	85	95
**Prevalence**	**95.5**	**82.0**	**63.7**	**43.7**	**31.7**	**21.6**	**17.3**	**13.3**	**10.3**	**7.3**	**5.9**	**5.1**	**3.2**	**2.0**	**0.9**	**0.6**
**Lower CI**	91.7	75.7	56.6	36.7	25.6	16.8	13.1	9.8	7.3	5.0	3.9	3.2	1.8	0.9	0.5	0.3
**Upper CI**	97.6	87.0	70.2	50.9	38.6	27.3	22.4	17.8	14.3	10.6	8.9	8.1	5.6	4.3	1.9	1.4
**Number**	253	225	181	133	104	82	67	54	46	36	29	24	18	11	8	6

Table W2K-33 Age Group = 51-60 Gender = OVERALL Occupational group = MANUAL Total cases = 362

	Threshold dB HL≥ 0	5	10	15	20	25	30	35	40	45	50	55	65	75	85	95
**Prevalence**	**98.2**	**92.6**	**76.3**	**56.2**	**43.5**	**34.7**	**23.2**	**20.2**	**14.6**	**12.8**	**10.7**	**8.9**	**5.5**	**3.4**	**1.5**	**1.2**
**Lower CI**	95.6	88.5	70.2	50.0	37.9	29.5	19.3	16.5	11.8	10.1	8.3	6.7	3.7	2.1	0.8	0.6
**Upper CI**	99.3	95.3	81.4	62.2	49.3	40.2	27.7	24.4	18.0	15.9	13.6	11.6	7.9	5.5	2.8	2.4
**Number**	357	342	302	249	205	171	132	116	96	85	73	62	39	25	12	10

Table W2K-34 Age Group = 61-70 Gender = OVERALL Occupational group = MANUAL Total cases = 298

	Threshold dB HL≥ 0	5	10	15	20	25	30	35	40	45	50	55	65	75	85	95
**Prevalence**	**99.3**	**96.9**	**84.2**	**72.0**	**60.3**	**50.0**	**39.3**	**31.8**	**27.1**	**21.0**	**17.5**	**15.6**	**10.4**	**5.8**	**4.4**	**1.9**
**Lower CI**	97.0	93.7	77.7	64.5	53.0	43.4	33.2	26.5	22.7	17.0	13.7	11.9	7.2	3.7	2.5	1.3
**Upper CI**	99.8	98.5	89.1	78.5	67.2	56.7	45.9	37.5	32.1	25.5	22.1	20.1	14.8	9.0	7.6	2.7
**Number**	296	290	271	246	220	198	172	151	137	114	98	88	57	32	21	10

Table W2K-35 Age Group = 71-80 Gender = OVERALL Occupational group = MANUAL Total cases = 149

	Threshold dB HL≥ 0	5	10	15	20	25	30	35	40	45	50	55	65	75	85	95
**Prevalence**	**100.0**	**97.7**	**97.4**	**89.2**	**80.9**	**71.6**	**65.2**	**54.5**	**44.7**	**41.5**	**35.1**	**25.8**	**15.6**	**8.8**	**7.1**	**4.2**
**Lower CI**	97.5	87.9	88.1	76.7	66.8	58.7	52.7	44.1	35.6	32.4	26.3	20.3	11.0	6.4	4.9	2.9
**Upper CI**	100.0	99.6	99.5	95.4	90.0	81.7	75.9	64.6	54.1	51.1	45.0	32.2	21.6	11.9	10.2	6.0
**Number**	149	148	147	144	137	133	125	115	101	94	84	72	48	32	24	17

Prevalence by age group, gender and occupational group for the worse ear over 2 kHz

Table W2K-36 Age Group = 18-80 Gender = FEMALE Occupational group = NON-MANUAL Total cases = 671

	Threshold dB HL≥															
	0	5	10	15	20	25	30	35	40	45	50	55	65	75	85	95
Prevalence	94.3	77.7	55.0	40.8	28.7	22.3	18.0	14.2	11.2	7.4	5.6	4.8	2.6	1.8	1.2	0.9
Lower CI	91.8	73.9	51.1	37.4	25.7	19.7	15.9	12.4	9.7	6.2	4.6	4.0	2.0	1.3	0.8	0.5
Upper CI	96.0	81.1	58.9	44.4	31.9	25.3	20.3	16.3	13.0	8.8	6.8	5.7	3.4	2.5	1.9	1.5
Number	643	552	428	343	255	210	181	150	126	101	77	68	46	34	22	15

Table W2K-37 Age Group = 18-80 Gender = MALE Occupational group = NON-MANUAL Total cases = 512

	Threshold dB HL≥															
	0	5	10	15	20	25	30	35	40	45	50	55	65	75	85	95
Prevalence	88.9	73.0	55.3	38.5	30.4	25.5	20.7	16.6	13.0	10.2	7.7	5.4	3.1	2.2	1.4	1.1
Lower CI	83.8	67.4	50.1	34.8	27.2	22.5	18.2	14.1	10.8	8.7	6.2	4.3	2.3	1.5	0.8	0.6
Upper CI	92.5	77.9	60.4	42.2	33.8	28.7	23.5	19.5	15.5	12.0	9.5	6.8	4.1	3.2	2.3	1.9
Number	481	420	351	273	225	188	161	139	109	93	75	59	39	26	16	11

Table W2K-38 Age Group = 18-80 Gender = FEMALE Occupational group = MANUAL Total cases = 715

	Threshold dB HL≥															
	0	5	10	15	20	25	30	35	40	45	50	55	65	75	85	95
Prevalence	95.4	81.2	60.1	43.4	31.1	24.0	17.6	13.9	10.6	9.3	7.0	5.6	3.5	1.5	1.2	0.8
Lower CI	92.3	76.9	55.6	39.3	27.8	21.1	15.1	11.8	8.9	7.7	5.7	4.5	2.6	1.2	0.9	0.6
Upper CI	97.3	84.9	64.5	47.7	34.6	27.2	20.5	16.4	12.6	11.1	8.6	6.9	4.7	2.0	1.6	1.1
Number	695	628	510	409	330	270	212	183	154	138	113	95	63	33	24	16

Table W2K-39 Age Group = 18-80 Gender = MALE Occupational group = MANUAL Total cases = 757

	Threshold dB HL≥															
	0	5	10	15	20	25	30	35	40	45	50	55	65	75	85	95
Prevalence	93.5	80.9	66.6	49.1	38.4	30.1	24.9	19.6	16.3	13.2	11.3	9.4	5.8	4.1	2.6	1.5
Lower CI	90.3	76.0	61.4	44.5	34.5	26.7	21.8	17.4	14.3	11.4	9.5	7.9	4.6	3.0	1.7	0.9
Upper CI	95.8	85.0	71.4	53.8	42.6	33.8	28.2	22.0	18.5	15.2	13.2	11.2	7.4	5.6	4.0	2.4
Number	729	673	594	500	425	378	332	290	258	218	189	165	106	71	44	29

Table W2K-40 Age Group = 18-30 Gender = FEMALE Occupational group = NON-MANUAL Total cases = 115

	Threshold dB HL≥															
	0	5	10	15	20	25	30	35	40	45	50	55	65	75	85	95
Prevalence	88.3	63.3	29.4	13.8	6.0	4.9	3.7	3.1	1.7	1.2	0.4	0.4	0.4	0.0	0.0	0.0
Lower CI	80.5	53.5	21.5	8.7	3.3	2.7	2.0	1.5	0.6	0.3	0.1	0.1	0.1	0.0	0.0	0.0
Upper CI	93.3	72.2	38.8	21.0	10.7	8.6	6.9	6.5	4.9	4.7	2.2	2.2	2.2	3.2	3.2	3.2
Number	103	75	38	21	11	10	8	7	4	2	1	1	1	0	0	0

Table W2K-41 Age Group = 31-40 Gender = FEMALE Occupational group = NON-MANUAL Total cases = 86

	Threshold dB HL≥															
	0	5	10	15	20	25	30	35	40	45	50	55	65	75	85	95
Prevalence	89.7	56.5	31.8	19.3	12.8	9.6	9.3	7.4	4.7	2.6	1.4	0.7	0.6	0.6	0.0	0.0
Lower CI	81.9	45.1	22.4	12.2	7.5	5.2	5.0	3.8	2.3	1.2	0.4	0.2	0.1	0.1	0.0	0.0
Upper CI	94.4	67.1	43.1	29.1	21.0	17.0	16.8	13.9	9.2	5.9	4.4	3.0	3.2	3.2	4.3	4.3
Number	77	49	31	21	15	12	11	9	7	5	3	2	1	1	0	0

Table W2K-42 Age Group = 41-50 Gender = FEMALE Occupational group = NON-MANUAL Total cases = 112

	Threshold dB HL≥															
	0	5	10	15	20	25	30	35	40	45	50	55	65	75	85	95
Prevalence	95.5	83.3	52.8	37.6	22.6	10.2	8.6	6.7	3.2	2.0	1.0	1.0	0.4	0.0	0.0	0.0
Lower CI	89.0	74.4	43.3	29.7	15.8	6.4	5.4	3.9	1.5	0.7	0.4	0.4	0.1	0.0	0.0	0.0
Upper CI	98.2	89.6	62.1	46.2	31.1	15.7	13.5	11.2	6.9	5.7	2.1	2.1	2.2	3.3	3.3	3.3
Number	107	94	64	48	29	16	14	11	7	3	2	2	1	0	0	0

Prevalence by age group, gender and occupational group for the worse ear over 2 kHz

Table W2K-43 Age Group = 51-60 Gender = FEMALE Occupational group = NON-MANUAL Total cases = 175

Threshold dB HL≥	0	5	10	15	20	25	30	35	40	45	50	55	65	75	85	95
Prevalence	99.4	90.4	72.8	56.6	33.4	24.8	18.3	13.3	9.3	7.9	5.3	4.5	3.6	2.9	2.2	1.8
Lower CI	96.5	84.7	65.2	48.4	26.7	18.8	13.6	9.5	6.3	5.3	3.3	2.6	1.9	1.5	0.9	0.6
Upper CI	99.9	94.1	79.3	64.3	40.9	31.9	24.2	18.4	13.4	11.6	8.4	7.6	6.7	5.8	5.3	5.0
Number	174	158	129	105	72	58	48	38	29	26	19	16	13	11	7	5

Table W2K-44 Age Group = 61-70 Gender = FEMALE Occupational group = NON-MANUAL Total cases = 116

Threshold dB HL≥	0	5	10	15	20	25	30	35	40	45	50	55	65	75	85	95
Prevalence	100.0	95.6	86.6	73.8	55.0	44.5	35.2	27.9	22.2	14.3	12.4	8.9	4.4	3.6	2.6	1.9
Lower CI	96.8	88.5	76.9	63.5	44.5	35.0	26.8	20.5	15.4	9.7	8.0	5.8	3.4	2.5	1.4	0.9
Upper CI	100.0	98.4	92.6	82.1	65.0	54.4	44.6	36.8	31.0	20.7	18.8	13.2	5.6	5.3	4.6	3.9
Number	116	111	103	89	70	60	51	41	39	31	26	23	14	12	9	7

Table W2K-45 Age Group = 71-80 Gender = FEMALE Occupational group = NON-MANUAL Total cases = 67

Threshold dB HL≥	0	5	10	15	20	25	30	35	40	45	50	55	65	75	85	95
Prevalence	98.6	95.8	89.4	80.8	80.4	72.5	60.2	48.0	44.7	27.5	22.8	21.7	10.1	6.7	4.5	2.5
Lower CI	92.2	85.0	69.5	64.6	64.2	55.1	50.7	42.0	38.5	23.4	18.5	17.3	6.6	3.9	2.2	0.8
Upper CI	99.7	98.9	96.9	90.7	90.3	84.9	69.0	54.1	51.1	32.1	27.7	26.8	15.1	11.2	8.8	7.6
Number	66	65	63	59	58	54	49	44	40	34	26	24	16	10	6	3

Table W2K-46 Age Group = 18-30 Gender = MALE Occupational group = NON-MANUAL Total cases = 66

Threshold dB HL≥	0	5	10	15	20	25	30	35	40	45	50	55	65	75	85	95
Prevalence	76.7	43.8	21.0	1.7	1.7	0.1	0.1	0.1	0.1	0.1	0.1	0.1	0.1	0.1	0.0	0.0
Lower CI	62.2	30.2	11.4	0.4	0.4	0.0	0.0	0.0	0.0	0.0	0.0	0.0	0.0	0.0	0.0	0.0
Upper CI	86.8	58.4	35.4	6.6	6.6	5.7	5.7	5.7	5.7	5.7	5.7	5.7	5.7	5.7	5.5	5.5
Number	53	31	16	3	3	1	1	1	1	1	1	1	1	1	0	0

Table W2K-47 Age Group = 31-40 Gender = MALE Occupational group = NON-MANUAL Total cases = 62

Threshold dB HL≥	0	5	10	15	20	25	30	35	40	45	50	55	65	75	85	95
Prevalence	79.9	68.0	44.5	25.5	13.9	10.6	5.9	2.8	1.9	1.9	0.0	0.0	0.0	0.0	0.0	0.0
Lower CI	63.2	50.5	29.1	13.8	6.9	4.6	1.9	1.0	0.6	0.6	0.0	0.0	0.0	0.0	0.0	0.0
Upper CI	90.3	81.6	61.0	42.1	26.3	22.8	16.7	7.2	6.3	6.3	5.8	5.8	5.8	5.8	5.8	5.8
Number	52	46	32	19	10	8	5	4	2	2	0	0	0	0	0	0

Table W2K-48 Age Group = 41-50 Gender = MALE Occupational group = NON-MANUAL Total cases = 76

Threshold dB HL≥	0	5	10	15	20	25	30	35	40	45	50	55	65	75	85	95
Prevalence	96.1	84.3	63.1	39.8	29.6	25.2	22.1	15.4	12.9	7.3	7.3	3.3	3.0	3.0	3.0	2.8
Lower CI	90.2	75.2	50.9	28.7	20.5	17.2	14.7	8.4	6.3	3.4	3.4	1.9	1.7	1.7	1.7	1.5
Upper CI	98.5	90.5	73.8	52.0	40.6	35.3	31.8	26.4	24.5	15.0	15.0	5.5	5.3	5.3	5.3	5.1
Number	72	62	49	32	24	20	17	14	11	8	8	6	5	5	5	4

Table W2K-49 Age Group = 51-60 Gender = MALE Occupational group = NON-MANUAL Total cases = 142

Threshold dB HL≥	0	5	10	15	20	25	30	35	40	45	50	55	65	75	85	95
Prevalence	96.6	81.2	66.8	48.8	38.6	33.5	28.0	18.3	12.9	9.0	5.9	5.0	4.4	2.3	0.7	0.5
Lower CI	91.3	73.0	57.9	40.5	31.1	26.6	22.4	11.6	7.2	6.1	3.8	3.1	2.5	0.9	0.2	0.1
Upper CI	98.7	87.3	74.7	57.1	46.6	41.1	34.5	27.5	22.0	13.2	8.9	8.1	7.6	5.5	2.1	2.0
Number	138	116	98	77	62	53	46	36	27	24	18	15	13	6	3	2

Prevalence by age group, gender and occupational group for the worse ear over 2 kHz

Table W2K-50 Age Group = 61-70 Gender = MALE Occupational group = NON-MANUAL Total cases = 110

	Threshold dB HL≥															
	**0**	**5**	**10**	**15**	**20**	**25**	**30**	**35**	**40**	**45**	**50**	**55**	**65**	**75**	**85**	**95**
**Prevalence**	**100.0**	**100.0**	**91.1**	**82.5**	**71.3**	**58.9**	**47.1**	**41.9**	**30.4**	**24.3**	**18.1**	**15.2**	**7.9**	**6.3**	**4.6**	**3.0**
**Lower CI**	96.6	96.6	82.3	73.5	56.1	44.6	34.8	30.0	24.5	19.7	13.1	10.6	4.4	3.1	1.8	1.0
**Upper CI**	100.0	100.0	95.8	89.0	82.9	71.8	59.8	54.8	37.1	29.5	24.5	21.3	13.9	12.4	11.2	9.0
**Number**	110	110	101	88	80	65	55	50	40	33	26	22	13	10	6	4

Table W2K-51 Age Group = 71-80 Gender = MALE Occupational group = NON-MANUAL Total cases = 56

	Threshold dB HL≥															
	**0**	**5**	**10**	**15**	**20**	**25**	**30**	**35**	**40**	**45**	**50**	**55**	**65**	**75**	**85**	**95**
**Prevalence**	**100.0**	**97.9**	**97.9**	**96.3**	**79.1**	**71.8**	**61.5**	**58.5**	**49.6**	**45.0**	**36.6**	**24.6**	**8.2**	**4.7**	**1.9**	**1.0**
**Lower CI**	93.6	89.0	89.0	87.2	67.4	59.7	50.8	48.3	38.6	34.3	25.4	15.8	4.7	1.9	0.7	0.2
**Upper CI**	100.0	99.6	99.6	99.0	87.4	81.4	71.2	68.0	60.7	56.2	49.6	36.2	14.0	11.4	5.6	5.3
**Number**	56	55	55	54	46	41	37	34	28	25	22	15	7	4	2	1

Table W2K-52 Age Group = 18-30 Gender = FEMALE Occupational group = MANUAL Total cases = 109

	Threshold dB HL≥															
	**0**	**5**	**10**	**15**	**20**	**25**	**30**	**35**	**40**	**45**	**50**	**55**	**65**	**75**	**85**	**95**
**Prevalence**	**90.2**	**65.5**	**43.7**	**24.9**	**8.5**	**5.1**	**4.0**	**4.0**	**2.9**	**2.2**	**0.7**	**0.5**	**0.0**	**0.0**	**0.0**	**0.0**
**Lower CI**	79.3	52.6	32.1	16.1	4.9	2.4	1.8	1.8	1.2	0.8	0.2	0.1	0.0	0.0	0.0	0.0
**Upper CI**	95.7	76.5	56.1	36.4	14.3	10.3	8.7	8.7	6.7	6.0	2.0	1.7	3.4	3.4	3.4	3.4
**Number**	100	80	54	32	17	10	9	9	8	6	3	2	0	0	0	0

Table W2K-53 Age Group = 31-40 Gender = FEMALE Occupational group = MANUAL Total cases = 103

	Threshold dB HL≥															
	**0**	**5**	**10**	**15**	**20**	**25**	**30**	**35**	**40**	**45**	**50**	**55**	**65**	**75**	**85**	**95**
**Prevalence**	**92.4**	**66.6**	**35.3**	**22.1**	**15.6**	**10.3**	**7.7**	**5.1**	**4.8**	**4.2**	**2.6**	**2.0**	**0.8**	**0.5**	**0.2**	**0.0**
**Lower CI**	83.2	55.0	25.2	14.7	10.0	6.2	4.0	2.4	2.2	1.8	1.2	0.9	0.3	0.1	0.0	0.0
**Upper CI**	96.7	76.6	47.0	31.7	23.5	16.6	14.3	10.7	10.4	9.7	5.4	4.5	2.3	1.6	3.9	3.6
**Number**	98	75	51	39	27	20	13	10	9	8	7	6	3	2	1	0

Table W2K-54 Age Group = 41-50 Gender = FEMALE Occupational group = MANUAL Total cases = 135

	Threshold dB HL≥															
	**0**	**5**	**10**	**15**	**20**	**25**	**30**	**35**	**40**	**45**	**50**	**55**	**65**	**75**	**85**	**95**
**Prevalence**	**95.1**	**82.0**	**58.8**	**39.0**	**27.1**	**18.7**	**14.4**	**8.8**	**6.6**	**4.9**	**4.1**	**3.9**	**2.9**	**1.4**	**1.2**	**0.6**
**Lower CI**	89.5	73.1	49.0	30.0	19.7	12.8	9.3	5.3	3.8	3.1	2.4	2.3	1.4	0.6	0.5	0.2
**Upper CI**	97.8	88.5	68.0	48.7	36.0	26.6	21.7	14.1	11.2	7.7	6.7	6.6	5.6	3.1	2.8	1.7
**Number**	130	117	89	62	49	36	29	22	18	16	13	12	10	6	5	3

Table W2K-55 Age Group = 51-60 Gender = FEMALE Occupational group = MANUAL Total cases = 166

	Threshold dB HL≥															
	**0**	**5**	**10**	**15**	**20**	**25**	**30**	**35**	**40**	**45**	**50**	**55**	**65**	**75**	**85**	**95**
**Prevalence**	**99.7**	**92.6**	**71.7**	**53.1**	**38.2**	**29.6**	**17.5**	**14.9**	**7.0**	**6.1**	**3.9**	**3.0**	**2.7**	**0.8**	**0.5**	**0.5**
**Lower CI**	98.3	86.3	62.5	43.8	30.0	22.3	12.4	10.1	4.5	3.7	2.3	1.7	1.4	0.4	0.2	0.2
**Upper CI**	99.9	96.2	79.4	62.1	47.1	38.1	24.0	21.3	10.8	9.7	6.5	5.4	5.2	1.4	1.2	1.2
**Number**	165	156	132	108	84	67	47	40	27	24	18	15	13	5	3	3

Table W2K-56 Age Group = 61-70 Gender = FEMALE Occupational group = MANUAL Total cases = 125

	Threshold dB HL≥															
	**0**	**5**	**10**	**15**	**20**	**25**	**30**	**35**	**40**	**45**	**50**	**55**	**65**	**75**	**85**	**95**
**Prevalence**	**100.0**	**99.1**	**78.6**	**65.5**	**53.4**	**40.6**	**26.6**	**20.9**	**17.5**	**14.3**	**12.7**	**11.2**	**7.3**	**3.2**	**2.7**	**1.9**
**Lower CI**	97.0	96.9	68.0	54.4	43.1	31.9	19.4	14.9	12.6	9.8	8.4	7.1	4.0	2.4	1.9	1.4
**Upper CI**	100.0	99.7	86.4	75.1	63.5	49.9	35.4	28.5	23.9	20.4	18.7	17.2	13.0	4.4	4.0	2.4
**Number**	125	123	108	95	82	69	52	45	40	33	29	25	16	7	5	2

Prevalence by age group, gender and occupational group for the worse ear over 2 kHz

Table W2K-57 Age Group = 71-80 Gender = FEMALE Occupational group = MANUAL Total cases = 77

Threshold dB HL≥	0	5	10	15	20	25	30	35	40	45	50	55	65	75	85	95
**Prevalence**	**100.0**	**100.0**	**99.6**	**85.8**	**78.0**	**70.9**	**63.7**	**51.2**	**43.1**	**41.7**	**32.4**	**22.8**	**13.5**	**5.6**	**4.4**	**3.1**
Lower CI	95.2	95.2	97.6	68.1	57.6	52.1	45.8	36.6	31.0	29.7	21.1	15.6	7.4	3.5	2.5	1.8
Upper CI	100.0	100.0	99.9	94.5	90.3	84.5	78.5	65.6	56.0	54.8	46.2	31.9	23.5	8.9	7.9	5.2
Number	77	77	76	73	71	68	62	57	52	51	43	35	21	13	10	8

Table W2K-58 Age Group = 18-30 Gender = MALE Occupational group = MANUAL Total cases = 109

Threshold dB HL≥	0	5	10	15	20	25	30	35	40	45	50	55	65	75	85	95
**Prevalence**	**88.4**	**69.6**	**47.9**	**23.8**	**11.6**	**7.7**	**5.7**	**2.4**	**1.1**	**0.8**	**0.3**	**0.1**	**0.1**	**0.1**	**0.1**	**0.1**
Lower CI	80.1	56.8	35.4	15.0	6.5	3.9	2.5	1.0	0.5	0.3	0.1	0.0	0.0	0.0	0.0	0.0
Upper CI	93.5	80.0	60.6	35.4	19.7	14.6	12.7	5.8	2.5	2.2	1.5	0.4	0.4	0.4	0.4	0.4
Number	96	79	54	33	23	17	11	8	5	4	2	1	1	1	1	1

Table W2K-59 Age Group = 31-40 Gender = MALE Occupational group = MANUAL Total cases = 80

Threshold dB HL≥	0	5	10	15	20	25	30	35	40	45	50	55	65	75	85	95
**Prevalence**	**90.7**	**70.8**	**49.0**	**34.2**	**26.2**	**15.9**	**12.5**	**5.0**	**5.0**	**4.2**	**3.5**	**3.1**	**2.5**	**1.9**	**1.9**	**1.9**
Lower CI	76.8	55.6	34.4	21.6	14.9	7.6	5.8	2.1	2.1	1.7	1.2	1.0	0.6	0.3	0.3	0.3
Upper CI	96.6	82.4	63.9	49.5	42.0	30.2	25.0	11.2	11.2	10.3	9.9	9.8	9.8	10.2	10.2	10.2
Number	75	62	44	33	22	17	15	10	10	9	6	5	3	1	1	1

Table W2K-60 Age Group = 41-50 Gender = MALE Occupational group = MANUAL Total cases = 127

Threshold dB HL≥	0	5	10	15	20	25	30	35	40	45	50	55	65	75	85	95
**Prevalence**	**95.9**	**81.9**	**69.0**	**48.9**	**36.6**	**24.5**	**20.2**	**18.1**	**14.3**	**9.9**	**7.8**	**6.3**	**3.6**	**2.5**	**0.7**	**0.7**
Lower CI	89.5	72.2	58.6	38.3	27.1	17.4	14.2	12.2	9.3	5.9	4.3	3.1	1.5	0.8	0.2	0.2
Upper CI	98.5	88.8	77.7	59.6	47.2	33.4	27.9	25.8	21.2	16.2	13.7	12.4	8.4	7.8	1.9	1.9
Number	123	108	92	71	55	46	38	32	28	20	16	12	8	5	3	3

Table W2K-61 Age Group = 51-60 Gender = MALE Occupational group = MANUAL Total cases = 196

Threshold dB HL≥	0	5	10	15	20	25	30	35	40	45	50	55	65	75	85	95
**Prevalence**	**96.6**	**92.5**	**81.2**	**59.6**	**49.5**	**40.4**	**29.6**	**26.0**	**23.0**	**20.1**	**18.2**	**15.2**	**8.4**	**6.3**	**2.6**	**2.0**
Lower CI	91.2	86.3	73.0	51.5	42.1	33.4	23.9	20.8	18.0	15.5	13.7	11.2	5.3	3.6	1.2	0.9
Upper CI	98.7	96.0	87.4	67.2	56.8	47.8	36.0	32.1	28.8	25.7	23.8	20.4	13.0	10.7	5.3	4.6
Number	192	186	170	141	121	104	85	76	69	61	55	47	26	20	9	7

Table W2K-62 Age Group = 61-70 Gender = MALE Occupational group = MANUAL Total cases = 173

Threshold dB HL≥	0	5	10	15	20	25	30	35	40	45	50	55	65	75	85	95
**Prevalence**	**98.5**	**94.3**	**91.1**	**79.7**	**68.2**	**60.9**	**54.4**	**44.4**	**38.5**	**28.9**	**23.2**	**20.7**	**14.0**	**8.9**	**6.2**	**1.9**
Lower CI	93.5	87.6	83.9	69.4	58.0	50.8	44.3	36.2	31.3	22.7	17.2	15.1	9.0	4.8	2.7	0.9
Upper CI	99.7	97.5	95.2	87.2	76.9	70.2	64.0	52.9	46.2	36.0	30.4	27.8	21.1	16.0	13.7	3.9
Number	171	167	163	151	138	129	120	106	97	81	69	63	41	25	16	8

Table W2K-63 Age Group = 71-80 Gender = MALE Occupational group = MANUAL Total cases = 72

Threshold dB HL≥	0	5	10	15	20	25	30	35	40	45	50	55	65	75	85	95
**Prevalence**	**100.0**	**94.2**	**94.2**	**94.2**	**85.2**	**72.9**	**67.5**	**59.7**	**47.2**	**41.3**	**39.4**	**30.6**	**18.8**	**13.5**	**11.1**	**5.8**
Lower CI	94.9	72.4	72.4	72.4	65.3	56.3	51.5	46.1	34.2	28.5	26.7	22.5	13.3	9.0	6.8	3.7
Upper CI	100.0	99.0	99.0	99.0	94.6	84.8	80.3	72.0	60.7	55.3	53.7	40.1	25.9	19.9	17.5	9.1
Number	72	71	71	71	66	65	63	58	49	43	41	37	27	19	14	9

Prevalence by age group, gender and occupational group for the worse ear over 3 kHz

Table W3K-1 Age Group = 18-80 Gender = OVERALL Occupational group = OVERALL Total cases = 2625

	Threshold dB HL≥															
	0	5	10	15	20	25	30	35	40	45	50	55	65	75	85	95
Prevalence	92.9	81.7	67.1	52.9	40.7	33.6	28.1	23.2	19.8	16.1	13.2	10.8	6.5	3.7	2.1	1.1
Lower CI	91.4	79.6	65.0	50.8	38.8	31.9	26.5	21.8	18.4	15.0	12.2	9.9	5.8	3.2	1.7	0.8
Upper CI	94.2	83.5	69.3	55.0	42.7	35.5	29.7	24.7	21.2	17.3	14.2	11.8	7.3	4.3	2.6	1.4
Number	2518	2326	2038	1728	1450	1251	1106	967	849	734	636	549	355	218	136	76

Table W3K-2 Age Group = 18-80 Gender = FEMALE Occupational group = OVERALL Total cases = 1370

	Threshold dB HL≥															
	0	5	10	15	20	25	30	35	40	45	50	55	65	75	85	95
Prevalence	92.7	80.4	63.5	47.9	33.8	26.8	21.7	17.6	14.7	11.0	8.6	6.9	4.2	2.1	1.3	0.7
Lower CI	90.8	77.8	60.7	45.3	31.4	24.5	19.7	15.7	13.0	9.6	7.5	5.9	3.4	1.7	1.0	0.4
Upper CI	94.2	82.9	66.2	50.5	36.4	29.1	23.9	19.5	16.5	12.6	9.8	8.1	5.1	2.6	1.7	1.0
Number	1301	1179	994	805	630	520	445	378	322	266	223	191	122	74	51	26

Table W3K-3 Age Group = 18-80 Gender = MALE Occupational group = OVERALL Total cases = 1255

	Threshold dB HL≥															
	0	5	10	15	20	25	30	35	40	45	50	55	65	75	85	95
Prevalence	93.2	83.1	71.2	58.5	48.4	41.3	35.2	29.6	25.4	21.7	18.3	15.2	9.2	5.5	3.0	1.5
Lower CI	90.6	79.8	67.8	55.2	45.4	38.6	32.8	27.4	23.4	19.9	16.6	13.7	8.0	4.5	2.3	1.1
Upper CI	95.2	85.9	74.4	61.7	51.4	44.1	37.7	31.9	27.6	23.6	20.1	16.9	10.5	6.7	3.9	2.1
Number	1217	1147	1044	923	820	731	661	589	527	468	413	358	233	144	85	50

Table W3K-4 Age Group = 18-30 Gender = OVERALL Occupational group = OVERALL Total cases = 401

	Threshold dB HL≥															
	0	5	10	15	20	25	30	35	40	45	50	55	65	75	85	95
Prevalence	83.2	60.0	41.3	23.7	13.8	10.6	5.7	4.0	3.2	2.4	1.5	0.8	0.3	0.3	0.0	0.0
Lower CI	78.3	54.2	35.8	19.2	10.5	7.7	3.8	2.5	1.9	1.3	0.7	0.3	0.1	0.1	0.0	0.0
Upper CI	87.2	65.6	47.0	28.8	18.0	14.4	8.4	6.4	5.4	4.1	3.0	1.8	1.5	1.5	0.1	0.1
Number	344	259	187	116	71	55	38	27	22	18	12	8	3	3	2	1

Table W3K-5 Age Group = 31-40 Gender = OVERALL Occupational group = OVERALL Total cases = 327

	Threshold dB HL≥															
	0	5	10	15	20	25	30	35	40	45	50	55	65	75	85	95
Prevalence	92.2	75.0	51.2	35.6	20.4	14.3	10.4	8.0	6.4	3.9	2.9	2.9	1.4	1.0	1.0	0.6
Lower CI	88.1	68.8	44.7	29.7	15.7	10.5	7.3	5.3	4.0	2.3	1.5	1.5	0.6	0.3	0.3	0.1
Upper CI	94.9	80.4	57.7	42.0	26.0	19.0	14.7	11.9	10.1	6.6	5.3	5.3	3.4	3.1	3.1	3.0
Number	306	259	186	132	85	67	51	40	32	22	16	16	8	5	5	2

Table W3K-6 Age Group = 41-50 Gender = OVERALL Occupational group = OVERALL Total cases = 443

	Threshold dB HL≥															
	0	5	10	15	20	25	30	35	40	45	50	55	65	75	85	95
Prevalence	96.4	87.8	68.3	50.7	38.8	28.9	22.3	16.4	11.8	9.2	7.0	6.1	3.5	1.4	0.8	0.5
Lower CI	93.9	84.0	63.3	45.7	34.2	24.8	18.6	13.3	9.3	7.0	5.3	4.5	2.3	0.9	0.4	0.3
Upper CI	97.9	90.8	72.9	55.6	43.7	33.5	26.4	19.9	14.8	11.9	9.3	8.3	5.3	2.2	1.4	1.1
Number	429	396	321	252	200	153	125	100	80	65	54	47	29	16	10	7

Table W3K-7 Age Group = 51-60 Gender = OVERALL Occupational group = OVERALL Total cases = 668

	Threshold dB HL≥															
	0	5	10	15	20	25	30	35	40	45	50	55	65	75	85	95
Prevalence	97.3	92.7	83.9	70.0	53.6	42.1	36.0	28.1	24.2	18.7	15.3	12.1	6.9	4.4	2.6	1.2
Lower CI	95.3	90.1	80.4	66.0	49.5	38.2	32.4	25.0	21.3	16.2	13.1	10.1	5.5	3.3	1.7	0.7
Upper CI	98.4	94.7	86.9	73.8	57.7	46.1	39.7	31.5	27.3	21.5	17.8	14.3	8.6	5.9	3.8	2.1
Number	655	629	584	505	414	343	302	257	229	186	158	131	80	54	32	16

Prevalence by age group, gender and occupational group for the worse ear over 3 kHz

Table W3K-8 Age Group = 61-70 Gender = OVERALL Occupational group = OVERALL Total cases = 517

Threshold dB HL≥	0	5	10	15	20	25	30	35	40	45	50	55	65	75	85	95
Prevalence	**99.2**	**99.0**	**93.9**	**84.6**	**75.4**	**65.8**	**58.5**	**49.3**	**40.4**	**35.2**	**30.2**	**26.2**	**16.2**	**8.8**	**4.6**	**2.4**
Lower CI	97.0	96.9	90.6	79.8	70.2	60.6	53.2	44.4	35.7	30.9	26.2	22.4	13.2	6.9	3.5	1.7
Upper CI	99.8	99.7	96.0	88.3	80.0	70.7	63.7	54.3	45.2	39.8	34.5	30.4	19.6	11.2	6.2	3.5
Number	515	514	494	464	431	391	357	320	274	249	222	197	132	77	46	28

Table W3K-9 Age Group = 71-80 Gender = OVERALL Occupational group = OVERALL Total cases = 269

Threshold dB HL≥	0	5	10	15	20	25	30	35	40	45	50	55	65	75	85	95
Prevalence	**100.0**	**100.0**	**97.9**	**92.5**	**82.6**	**79.8**	**74.2**	**69.6**	**65.7**	**56.1**	**47.1**	**37.4**	**23.8**	**13.0**	**7.6**	**3.3**
Lower CI	98.6	98.6	93.8	86.6	73.9	71.2	65.5	61.3	57.6	49.3	40.9	31.7	19.1	9.8	5.2	2.2
Upper CI	100.0	100.0	99.3	95.9	88.8	86.3	81.3	76.8	73.0	62.7	53.5	43.5	29.2	17.2	10.9	4.8
Number	269	269	266	259	249	242	233	223	212	194	174	150	103	63	41	22

Table W3K-10 Age Group = 18-80 Gender = OVERALL Occupational group = NON-MANUAL Total cases = 1167

Threshold dB HL≥	0	5	10	15	20	25	30	35	40	45	50	55	65	75	85	95
Prevalence	**92.9**	**78.9**	**62.4**	**46.9**	**35.9**	**30.6**	**26.0**	**20.8**	**17.2**	**13.1**	**10.7**	**8.3**	**5.0**	**2.2**	**1.6**	**1.1**
Lower CI	90.5	75.7	59.2	44.0	33.3	28.2	23.6	18.9	15.4	11.8	9.6	7.4	4.2	1.7	1.2	0.7
Upper CI	94.7	81.7	65.5	49.9	38.6	33.1	28.5	22.9	19.1	14.6	11.9	9.4	5.9	2.8	2.2	1.6
Number	1114	1001	849	692	558	483	417	352	299	254	211	175	111	59	45	28

Table W3K-11 Age Group = 18-80 Gender = OVERALL Occupational group = MANUAL Total cases = 1454

Threshold dB HL≥	0	5	10	15	20	25	30	35	40	45	50	55	65	75	85	95
Prevalence	**92.8**	**84.6**	**71.5**	**58.4**	**45.5**	**36.2**	**30.1**	**25.5**	**22.0**	**18.5**	**14.8**	**12.7**	**7.8**	**4.7**	**2.5**	**1.2**
Lower CI	90.4	81.6	68.1	55.2	42.5	33.6	27.9	23.5	20.2	16.9	13.5	11.3	6.7	3.9	2.0	0.9
Upper CI	94.6	87.3	74.6	61.6	48.6	38.8	32.3	27.5	24.0	20.2	16.3	14.2	9.1	5.7	3.1	1.6
Number	1401	1322	1187	1034	890	766	687	613	549	479	424	373	243	158	91	48

Table W3K-12 Age Group = 18-30 Gender = FEMALE Occupational group = OVERALL Total cases = 224

Threshold dB HL≥	0	5	10	15	20	25	30	35	40	45	50	55	65	75	85	95
Prevalence	**85.7**	**61.2**	**39.9**	**20.7**	**9.7**	**6.9**	**3.8**	**2.9**	**2.6**	**2.0**	**0.9**	**0.3**	**0.0**	**0.0**	**0.0**	**0.0**
Lower CI	80.1	53.7	33.0	15.5	6.4	4.5	2.3	1.6	1.4	1.0	0.4	0.1	0.0	0.0	0.0	0.0
Upper CI	89.8	68.1	47.2	27.0	14.3	10.6	6.3	5.0	4.6	4.0	2.2	1.3	1.7	1.7	1.7	1.7
Number	192	142	99	58	31	25	17	13	12	9	5	2	0	0	0	0

Table W3K-13 Age Group = 31-40 Gender = FEMALE Occupational group = OVERALL Total cases = 185

Threshold dB HL≥	0	5	10	15	20	25	30	35	40	45	50	55	65	75	85	95
Prevalence	**89.2**	**71.4**	**42.2**	**27.9**	**15.2**	**11.6**	**7.6**	**5.8**	**5.1**	**3.2**	**1.5**	**1.5**	**0.6**	**0.3**	**0.3**	**0.0**
Lower CI	82.9	63.2	34.4	21.4	10.6	7.9	4.8	3.3	2.8	1.5	0.6	0.6	0.2	0.1	0.1	0.0
Upper CI	93.4	78.4	50.4	35.5	21.1	16.7	12.0	10.0	9.3	6.7	4.0	4.0	1.7	1.2	1.2	2.0
Number	170	141	94	66	40	33	23	17	14	9	6	6	3	2	2	0

Table W3K-14 Age Group = 41-50 Gender = FEMALE Occupational group = OVERALL Total cases = 245

Threshold dB HL≥	0	5	10	15	20	25	30	35	40	45	50	55	65	75	85	95
Prevalence	**95.7**	**83.8**	**60.5**	**41.4**	**29.8**	**19.9**	**15.4**	**9.0**	**5.2**	**3.7**	**3.2**	**2.9**	**1.3**	**0.8**	**0.6**	**0.3**
Lower CI	91.7	78.0	53.5	35.1	24.3	15.3	11.5	6.3	3.5	2.4	2.0	1.7	0.7	0.3	0.2	0.1
Upper CI	97.8	88.4	67.0	48.0	36.0	25.5	20.4	12.7	7.7	5.6	5.0	4.8	2.5	1.9	1.6	1.0
Number	236	212	161	118	89	61	49	34	25	19	16	14	8	5	4	2

Prevalence by age group, gender and occupational group for the worse ear over 3 kHz

Table W3K-15 Age Group = 51-60 Gender = FEMALE Occupational group = OVERALL Total cases = 334

	Threshold dB HL≥ 0	5	10	15	20	25	30	35	40	45	50	55	65	75	85	95
**Prevalence**	**95.6**	**89.6**	**79.9**	**63.4**	**42.1**	**30.3**	**22.5**	**16.0**	**12.0**	**8.1**	**5.6**	**4.5**	**3.1**	**2.2**	**1.8**	**1.0**
**Lower CI**	92.3	85.3	74.6	57.4	36.4	25.3	18.2	12.7	9.2	6.0	4.1	3.2	2.0	1.4	1.0	0.4
**Upper CI**	97.6	92.7	84.3	69.0	48.1	35.7	27.3	19.9	15.4	10.8	7.7	6.4	4.8	3.5	3.1	2.1
**Number**	323	305	279	231	170	132	105	85	70	53	43	37	24	19	14	7

Table W3K-16 Age Group = 61-70 Gender = FEMALE Occupational group = OVERALL Total cases = 239

	Threshold dB HL≥ 0	5	10	15	20	25	30	35	40	45	50	55	65	75	85	95
**Prevalence**	**98.4**	**98.2**	**89.6**	**78.1**	**64.4**	**50.2**	**44.2**	**36.9**	**28.7**	**23.0**	**19.8**	**16.0**	**10.5**	**4.7**	**2.0**	**1.3**
**Lower CI**	94.4	94.4	83.8	71.0	56.5	42.6	36.9	30.2	22.8	17.9	15.1	11.5	6.9	3.2	1.1	0.7
**Upper CI**	99.6	99.4	93.5	83.8	71.6	57.8	51.7	44.2	35.5	29.2	25.5	21.7	15.6	6.8	3.7	2.7
**Number**	237	236	219	197	173	144	130	114	92	78	69	59	40	22	11	8

Table W3K-17 Age Group = 71-80 Gender = FEMALE Occupational group = OVERALL Total cases = 143

	Threshold dB HL≥ 0	5	10	15	20	25	30	35	40	45	50	55	65	75	85	95
**Prevalence**	**100.0**	**100.0**	**98.5**	**89.8**	**74.5**	**72.7**	**66.8**	**62.4**	**59.4**	**46.4**	**37.7**	**29.8**	**17.8**	**7.7**	**5.0**	**2.3**
**Lower CI**	97.4	97.4	91.8	80.9	61.1	59.4	53.9	50.2	47.2	36.8	30.3	23.3	12.7	5.3	3.4	1.2
**Upper CI**	100.0	100.0	99.7	94.9	84.5	82.8	77.6	73.2	70.5	56.2	45.8	37.3	24.5	10.9	7.4	4.5
**Number**	143	143	142	135	127	125	121	115	109	98	84	73	47	26	20	9

Table W3K-18 Age Group = 18-30 Gender = MALE Occupational group = OVERALL Total cases = 177

	Threshold dB HL≥ 0	5	10	15	20	25	30	35	40	45	50	55	65	75	85	95
**Prevalence**	**80.7**	**58.7**	**42.3**	**26.5**	**17.9**	**14.1**	**7.4**	**5.1**	**3.7**	**2.7**	**2.0**	**1.2**	**0.6**	**0.6**	**0.1**	**0.0**
**Lower CI**	72.1	49.7	33.9	19.7	12.4	9.1	4.3	2.6	1.7	1.2	0.8	0.4	0.1	0.1	0.0	0.0
**Upper CI**	87.1	67.2	51.1	34.7	25.2	21.1	12.5	9.7	8.1	6.0	5.0	3.3	3.0	3.0	0.2	0.2
**Number**	152	117	88	58	40	30	21	14	10	9	7	6	3	3	2	1

Table W3K-19 Age Group = 31-40 Gender = MALE Occupational group = OVERALL Total cases = 142

	Threshold dB HL≥ 0	5	10	15	20	25	30	35	40	45	50	55	65	75	85	95
**Prevalence**	**95.5**	**79.2**	**61.3**	**44.2**	**26.1**	**17.0**	**13.5**	**10.3**	**7.8**	**4.7**	**4.3**	**4.3**	**2.4**	**1.8**	**1.8**	**1.3**
**Lower CI**	89.3	69.1	50.4	34.2	18.0	10.9	8.1	5.8	3.9	2.3	1.9	1.9	0.8	0.5	0.5	0.3
**Upper CI**	98.2	86.7	71.2	54.7	36.1	25.5	21.6	17.8	14.7	9.7	9.3	9.3	6.7	6.5	6.5	6.4
**Number**	136	118	92	66	45	34	28	23	18	13	10	10	5	3	3	2

Table W3K-20 Age Group = 41-50 Gender = MALE Occupational group = OVERALL Total cases = 198

	Threshold dB HL≥ 0	5	10	15	20	25	30	35	40	45	50	55	65	75	85	95
**Prevalence**	**97.2**	**92.2**	**77.0**	**60.8**	**48.7**	**38.8**	**29.7**	**24.4**	**19.0**	**15.3**	**11.3**	**9.6**	**5.9**	**1.9**	**0.9**	**0.8**
**Lower CI**	93.4	86.9	69.6	53.1	41.1	31.8	23.7	19.0	14.2	11.0	7.9	6.6	3.6	1.1	0.4	0.3
**Upper CI**	98.8	95.5	83.0	68.1	56.4	46.3	36.6	30.8	24.9	20.7	15.8	13.9	9.5	3.3	1.9	1.8
**Number**	193	184	160	134	111	92	76	66	55	46	38	33	21	11	6	5

Table W3K-21 Age Group = 51-60 Gender = MALE Occupational group = OVERALL Total cases = 334

	Threshold dB HL≥ 0	5	10	15	20	25	30	35	40	45	50	55	65	75	85	95
**Prevalence**	**99.1**	**96.4**	**88.3**	**77.3**	**66.4**	**55.3**	**51.1**	**41.7**	**37.8**	**30.5**	**26.1**	**20.4**	**11.1**	**7.0**	**3.4**	**1.5**
**Lower CI**	96.9	93.2	83.6	71.8	60.4	49.4	45.3	36.2	32.5	25.9	21.8	16.6	8.5	4.8	2.0	0.7
**Upper CI**	99.8	98.1	91.9	82.0	71.8	61.1	57.0	47.4	43.3	35.6	30.9	24.8	14.4	9.9	5.9	3.0
**Number**	332	324	305	274	244	211	197	172	159	133	115	94	56	35	18	9

Prevalence by age group, gender and occupational group for the worse ear over 3 kHz

Table W3K-22 Age Group = 61-70 Gender = MALE Occupational group = OVERALL Total cases = 278

	Threshold dB HL≥															
	0	5	10	15	20	25	30	35	40	45	50	55	65	75	85	95
Prevalence	**100.0**	**100.0**	**98.9**	**92.0**	**88.3**	**84.0**	**75.2**	**63.5**	**53.8**	**49.2**	**42.3**	**38.2**	**22.7**	**13.6**	**7.7**	**3.7**
Lower CI	98.6	98.6	96.6	85.0	81.0	76.7	67.3	56.4	46.8	42.4	36.0	32.2	18.3	10.0	5.5	2.4
Upper CI	100.0	100.0	99.6	95.9	93.0	89.4	81.6	70.2	60.6	56.1	48.9	44.7	27.7	18.3	10.8	5.7
Number	278	278	275	267	258	247	227	206	182	171	153	138	92	55	35	20

Table W3K-23 Age Group = 71-80 Gender = MALE Occupational group = OVERALL Total cases = 126

	Threshold dB HL≥															
	0	5	10	15	20	25	30	35	40	45	50	55	65	75	85	95
Prevalence	**100.0**	**100.0**	**96.7**	**96.7**	**94.7**	**90.2**	**84.9**	**79.6**	**74.2**	**69.8**	**60.7**	**48.6**	**32.8**	**21.3**	**11.4**	**4.8**
Lower CI	97.0	97.0	88.4	88.4	86.3	81.6	75.4	70.2	65.8	61.3	50.2	38.7	24.7	14.3	6.6	3.0
Upper CI	100.0	100.0	99.1	99.1	98.1	95.0	91.2	86.7	81.0	77.1	70.2	58.6	42.1	30.5	18.9	7.5
Number	126	126	124	124	122	117	112	108	103	96	90	77	56	37	21	13

Table W3K-24 Age Group = 18-30 Gender = OVERALL Occupational group = NON-MANUAL Total cases = 181

	Threshold dB HL≥															
	0	5	10	15	20	25	30	35	40	45	50	55	65	75	85	95
Prevalence	**82.7**	**53.0**	**33.5**	**16.2**	**8.9**	**6.1**	**5.0**	**2.3**	**1.9**	**1.4**	**1.0**	**0.3**	**0.1**	**0.1**	**0.1**	**0.0**
Lower CI	74.9	44.3	26.0	11.0	5.0	3.1	2.3	1.0	0.8	0.5	0.2	0.1	0.0	0.0	0.0	0.0
Upper CI	88.4	61.5	42.0	23.3	15.3	11.7	10.6	5.0	4.6	4.4	3.9	1.0	2.2	2.2	2.2	2.1
Number	153	102	65	34	20	16	12	7	6	4	3	2	1	1	1	0

Table W3K-25 Age Group = 31-40 Gender = OVERALL Occupational group = NON-MANUAL Total cases = 146

	Threshold dB HL≥															
	0	5	10	15	20	25	30	35	40	45	50	55	65	75	85	95
Prevalence	**94.8**	**73.9**	**43.0**	**28.3**	**14.8**	**14.0**	**9.6**	**7.7**	**5.7**	**2.3**	**0.4**	**0.4**	**0.0**	**0.0**	**0.0**	**0.0**
Lower CI	89.8	64.3	33.8	20.3	9.0	8.3	5.0	3.8	2.3	0.9	0.1	0.1	0.0	0.0	0.0	0.0
Upper CI	97.4	81.6	52.7	37.8	23.2	22.5	17.7	15.1	13.5	5.6	3.9	3.9	2.6	2.6	2.6	2.6
Number	139	113	69	46	26	24	16	13	9	6	2	2	0	0	0	0

Table W3K-26 Age Group = 41-50 Gender = OVERALL Occupational group = NON-MANUAL Total cases = 187

	Threshold dB HL≥															
	0	5	10	15	20	25	30	35	40	45	50	55	65	75	85	95
Prevalence	**94.6**	**84.6**	**63.1**	**44.1**	**32.3**	**24.8**	**19.8**	**15.3**	**8.9**	**7.3**	**5.5**	**2.9**	**1.5**	**1.1**	**1.1**	**1.0**
Lower CI	89.8	78.3	55.3	37.0	26.0	19.2	15.2	11.5	5.4	4.2	3.2	1.3	0.9	0.7	0.7	0.6
Upper CI	97.3	89.3	70.3	51.5	39.3	31.5	25.3	20.2	14.3	12.6	9.3	6.6	2.3	1.8	1.8	1.7
Number	177	158	123	91	69	50	41	32	21	17	13	8	6	4	4	3

Table W3K-27 Age Group = 51-60 Gender = OVERALL Occupational group = NON-MANUAL Total cases = 309

	Threshold dB HL≥															
	0	5	10	15	20	25	30	35	40	45	50	55	65	75	85	95
Prevalence	**97.1**	**92.5**	**81.8**	**63.2**	**45.3**	**35.5**	**30.7**	**23.8**	**20.1**	**15.2**	**11.3**	**9.9**	**5.0**	**2.1**	**1.4**	**0.9**
Lower CI	94.0	88.5	76.5	57.4	39.8	30.6	26.0	19.9	16.8	12.6	9.3	8.2	2.6	1.3	0.8	0.4
Upper CI	98.6	95.1	86.2	68.7	50.9	40.8	35.8	28.1	23.8	18.3	13.7	12.0	9.4	3.4	2.6	2.1
Number	302	286	258	210	159	130	111	91	80	62	50	44	24	14	10	6

Table W3K-28 Age Group = 61-70 Gender = OVERALL Occupational group = NON-MANUAL Total cases = 223

	Threshold dB HL≥															
	0	5	10	15	20	25	30	35	40	45	50	55	65	75	85	95
Prevalence	**99.2**	**99.0**	**94.7**	**83.1**	**73.4**	**65.5**	**56.8**	**44.5**	**35.6**	**30.7**	**26.7**	**21.9**	**14.3**	**6.2**	**4.4**	**3.5**
Lower CI	95.4	95.6	89.6	74.4	64.5	56.7	48.2	37.9	30.1	25.8	22.4	17.8	11.0	4.3	2.6	1.8
Upper CI	99.9	99.8	97.4	89.3	80.7	73.4	65.0	51.2	41.5	36.0	31.5	26.6	18.4	8.7	7.4	6.5
Number	222	221	215	198	176	160	140	119	99	88	77	66	46	24	17	12

Prevalence by age group, gender and occupational group for the worse ear over 3 kHz

Table W3K-29 Age Group = 71-80 Gender = OVERALL Occupational group = NON-MANUAL Total cases = 121

Threshold dB HL≥	0	5	10	15	20	25	30	35	40	45	50	55	65	75	85	95
**Prevalence**	**100.0**	**100.0**	**97.6**	**90.2**	**85.3**	**81.0**	**73.0**	**67.3**	**62.7**	**46.9**	**41.9**	**31.9**	**19.8**	**8.2**	**6.2**	**3.0**
**Lower CI**	96.9	96.9	91.2	81.3	76.1	72.3	60.6	55.9	52.0	40.1	34.8	25.9	15.6	5.1	3.4	1.5
**Upper CI**	100.0	100.0	99.4	95.1	91.4	87.4	82.6	76.9	72.2	53.8	49.3	38.5	24.9	12.8	10.9	6.1
**Number**	121	121	119	113	108	103	97	90	84	77	66	53	34	16	13	7

Table W3K-30 Age Group = 18-30 Gender = OVERALL Occupational group = MANUAL Total cases = 218

Threshold dB HL≥	0	5	10	15	20	25	30	35	40	45	50	55	65	75	85	95
**Prevalence**	**83.0**	**67.9**	**49.5**	**31.7**	**18.8**	**13.8**	**5.9**	**4.9**	**3.8**	**2.9**	**1.5**	**1.0**	**0.5**	**0.5**	**0.0**	**0.0**
**Lower CI**	75.1	58.9	40.6	24.1	13.0	9.2	3.6	2.7	2.0	1.5	0.7	0.4	0.1	0.1	0.0	0.0
**Upper CI**	88.7	75.7	58.4	40.5	26.4	20.0	9.6	8.8	7.0	5.6	3.2	2.8	2.6	2.6	0.2	0.2
**Number**	190	156	122	82	51	39	26	20	16	14	9	6	2	2	1	1

Table W3K-31 Age Group = 31-40 Gender = OVERALL Occupational group = MANUAL Total cases = 181

Threshold dB HL≥	0	5	10	15	20	25	30	35	40	45	50	55	65	75	85	95
**Prevalence**	**90.5**	**76.7**	**59.0**	**42.5**	**26.2**	**14.5**	**11.8**	**8.3**	**6.5**	**4.7**	**4.5**	**4.5**	**2.2**	**1.6**	**1.6**	**1.0**
**Lower CI**	84.0	67.7	49.4	33.7	18.9	9.6	7.3	4.9	3.9	2.5	2.3	2.3	0.9	0.5	0.5	0.2
**Upper CI**	94.5	83.8	68.0	51.8	35.0	21.2	18.5	13.8	10.8	8.9	8.7	8.7	5.3	5.0	5.0	4.9
**Number**	167	146	117	86	59	43	35	27	23	16	14	14	8	5	5	2

Table W3K-32 Age Group = 41-50 Gender = OVERALL Occupational group = MANUAL Total cases = 256

Threshold dB HL≥	0	5	10	15	20	25	30	35	40	45	50	55	65	75	85	95
**Prevalence**	**97.7**	**90.9**	**71.9**	**57.0**	**45.9**	**34.0**	**25.7**	**18.9**	**15.1**	**11.7**	**8.3**	**7.9**	**4.6**	**1.5**	**0.7**	**0.4**
**Lower CI**	94.3	85.9	65.2	50.0	38.8	27.5	20.2	14.4	11.2	8.2	5.9	5.6	2.8	0.9	0.3	0.2
**Upper CI**	99.1	94.2	77.8	63.7	53.1	41.0	32.1	24.4	20.1	16.3	11.5	11.2	7.6	2.7	1.4	1.1
**Number**	252	238	198	161	131	103	84	68	59	48	41	39	23	12	6	4

Table W3K-33 Age Group = 51-60 Gender = OVERALL Occupational group = MANUAL Total cases = 359

Threshold dB HL≥	0	5	10	15	20	25	30	35	40	45	50	55	65	75	85	95
**Prevalence**	**97.5**	**93.6**	**86.0**	**75.7**	**61.3**	**47.3**	**39.6**	**31.4**	**27.4**	**21.6**	**18.6**	**14.2**	**8.2**	**5.7**	**3.3**	**1.2**
**Lower CI**	94.5	89.8	80.7	69.9	55.2	41.6	34.7	27.0	23.3	18.1	15.4	11.4	6.2	4.0	2.0	0.7
**Upper CI**	98.9	96.0	90.1	80.8	67.0	53.2	44.8	36.1	31.9	25.5	22.3	17.5	10.8	8.0	5.5	2.3
**Number**	353	343	326	295	255	213	191	166	149	124	108	87	56	40	22	10

Table W3K-34 Age Group = 61-70 Gender = OVERALL Occupational group = MANUAL Total cases = 293

Threshold dB HL≥	0	5	10	15	20	25	30	35	40	45	50	55	65	75	85	95
**Prevalence**	**99.2**	**99.2**	**93.0**	**85.4**	**77.5**	**65.0**	**60.2**	**52.6**	**43.7**	**38.6**	**32.7**	**29.3**	**18.3**	**11.0**	**5.3**	**2.6**
**Lower CI**	95.6	95.6	87.4	78.9	70.3	58.1	53.2	46.2	37.4	32.7	27.4	23.9	13.8	7.8	3.8	1.9
**Upper CI**	99.9	99.9	96.2	90.2	83.4	71.2	66.8	58.9	50.1	44.8	38.5	35.4	24.0	15.1	7.4	3.6
**Number**	292	292	278	265	254	230	216	200	175	161	145	131	86	53	29	16

Table W3K-35 Age Group = 71-80 Gender = OVERALL Occupational group = MANUAL Total cases = 147

Threshold dB HL≥	0	5	10	15	20	25	30	35	40	45	50	55	65	75	85	95
**Prevalence**	**100.0**	**100.0**	**97.7**	**94.6**	**82.3**	**81.9**	**78.5**	**75.8**	**73.7**	**66.3**	**51.4**	**43.3**	**29.4**	**16.7**	**8.4**	**3.8**
**Lower CI**	97.5	97.5	87.9	81.5	68.7	68.4	65.1	63.0	60.9	55.0	42.0	33.6	21.4	11.5	5.9	2.6
**Upper CI**	100.0	100.0	99.6	98.6	90.7	90.4	87.8	85.2	83.4	76.0	60.8	53.5	39.0	23.7	11.7	5.4
**Number**	147	147	146	145	140	138	135	132	127	116	107	96	68	46	28	15

Prevalence by age group, gender and occupational group for the worse ear over 3 kHz

Table W3K-36 Age Group = 18-80 Gender = FEMALE Occupational group = NON-MANUAL Total cases = 662

Threshold dB HL≥	0	5	10	15	20	25	30	35	40	45	50	55	65	75	85	95
Prevalence	93.6	78.8	60.6	44.2	32.1	27.0	22.5	17.9	14.9	9.7	7.3	5.8	3.7	1.4	1.0	0.7
Lower CI	91.1	75.1	56.9	40.7	29.2	24.3	19.7	15.5	12.7	8.3	6.1	4.8	2.9	1.0	0.6	0.4
Upper CI	95.4	82.0	64.1	47.7	35.1	29.8	25.5	20.6	17.4	11.5	8.7	7.0	4.7	2.0	1.6	1.2
Number	630	555	458	359	277	236	202	168	143	116	91	79	50	27	20	13

Table W3K-37 Age Group = 18-80 Gender = MALE Occupational group = NON-MANUAL Total cases = 505

Threshold dB HL≥	0	5	10	15	20	25	30	35	40	45	50	55	65	75	85	95
Prevalence	92.0	78.9	64.3	50.0	40.0	34.6	29.9	24.0	19.6	16.9	14.4	11.2	6.4	3.1	2.4	1.6
Lower CI	87.4	73.5	58.8	45.1	35.5	30.5	26.0	21.1	16.8	14.7	12.5	9.5	5.0	2.3	1.6	1.0
Upper CI	95.0	83.5	69.4	54.9	44.6	38.9	34.1	27.3	22.7	19.3	16.5	13.0	8.1	4.1	3.5	2.5
Number	484	446	391	333	281	247	215	184	156	138	120	96	61	32	25	15

Table W3K-38 Age Group = 18-80 Gender = FEMALE Occupational group = MANUAL Total cases = 707

Threshold dB HL≥	0	5	10	15	20	25	30	35	40	45	50	55	65	75	85	95
Prevalence	91.1	82.9	67.2	52.2	36.7	26.9	21.0	17.0	14.2	11.5	9.1	7.4	4.6	2.4	1.6	0.7
Lower CI	87.5	78.6	62.5	47.8	32.6	23.6	18.2	14.5	11.9	9.5	7.6	6.0	3.4	1.8	1.2	0.5
Upper CI	93.8	86.5	71.5	56.5	40.9	30.4	24.0	19.7	16.8	13.8	10.9	9.2	6.1	3.1	2.1	1.0
Number	670	623	535	445	352	283	242	209	179	150	132	112	72	47	31	13

Table W3K-39 Age Group = 18-80 Gender = MALE Occupational group = MANUAL Total cases = 747

Threshold dB HL≥	0	5	10	15	20	25	30	35	40	45	50	55	65	75	85	95
Prevalence	94.7	86.8	76.4	65.4	55.5	46.5	40.3	35.0	30.8	26.2	21.2	18.6	11.4	7.3	3.5	1.7
Lower CI	91.1	82.3	71.4	60.5	51.1	42.6	37.0	31.9	28.0	23.7	19.0	16.3	9.5	5.7	2.5	1.1
Upper CI	96.9	90.3	80.7	70.0	59.8	50.6	43.7	38.2	33.7	28.9	23.7	21.2	13.6	9.3	4.7	2.6
Number	731	699	652	589	538	483	445	404	370	329	292	261	171	111	60	35

Table W3K-40 Age Group = 18-30 Gender = FEMALE Occupational group = NON-MANUAL Total cases = 115

Threshold dB HL≥	0	5	10	15	20	25	30	35	40	45	50	55	65	75	85	95
Prevalence	87.1	56.3	33.8	14.7	6.7	5.9	4.6	2.8	2.2	1.2	0.4	0.4	0.0	0.0	0.0	0.0
Lower CI	80.0	46.7	25.8	9.4	3.8	3.3	2.4	1.3	0.9	0.3	0.1	0.1	0.0	0.0	0.0	0.0
Upper CI	91.9	65.5	42.9	22.3	11.5	10.5	8.6	6.2	5.0	4.7	2.2	2.2	3.2	3.2	3.2	3.2
Number	100	66	42	22	12	11	8	5	4	2	1	1	0	0	0	0

Table W3K-41 Age Group = 31-40 Gender = FEMALE Occupational group = NON-MANUAL Total cases = 84

Threshold dB HL≥	0	5	10	15	20	25	30	35	40	45	50	55	65	75	85	95
Prevalence	92.0	69.3	34.3	22.7	10.8	10.1	5.9	5.9	5.1	2.9	0.2	0.2	0.0	0.0	0.0	0.0
Lower CI	84.0	57.6	24.7	14.7	5.9	5.3	2.5	2.5	1.9	0.8	0.0	0.0	0.0	0.0	0.0	0.0
Upper CI	96.2	78.9	45.2	33.4	18.8	18.3	13.1	13.1	12.7	9.7	4.7	4.7	4.4	4.4	4.4	4.4
Number	78	61	33	22	12	11	6	6	5	3	1	1	0	0	0	0

Table W3K-42 Age Group = 41-50 Gender = FEMALE Occupational group = NON-MANUAL Total cases = 112

Threshold dB HL≥	0	5	10	15	20	25	30	35	40	45	50	55	65	75	85	95
Prevalence	96.1	83.6	64.3	41.6	26.2	17.1	15.8	8.9	3.8	1.9	1.1	0.8	0.4	0.0	0.0	0.0
Lower CI	90.3	74.9	54.3	33.0	19.3	11.4	10.4	5.5	2.1	1.1	0.7	0.4	0.1	0.0	0.0	0.0
Upper CI	98.5	89.7	73.1	50.8	34.5	24.8	23.2	14.0	7.0	3.5	1.7	6.0	2.2	3.3	3.3	3.3
Number	107	94	74	52	37	23	21	14	9	6	3	2	1	0	0	0

Prevalence by age group, gender and occupational group for the worse ear over 3 kHz

Table W3K-43 Age Group = 51-60 Gender = FEMALE Occupational group = NON-MANUAL Total cases = 170

	Threshold dB HL≥															
	0	5	10	15	20	25	30	35	40	45	50	55	65	75	85	95
Prevalence	96.0	90.5	78.2	58.7	38.9	28.9	23.2	16.9	13.1	9.0	5.7	5.0	2.3	1.8	1.6	1.2
Lower CI	90.6	84.2	70.6	50.2	31.4	22.7	17.6	12.7	9.5	6.5	3.9	3.4	1.4	0.8	0.7	0.4
Upper CI	98.3	94.5	84.3	66.6	47.0	36.1	30.0	22.2	17.8	12.3	8.2	7.3	3.9	3.6	3.5	3.2
Number	165	155	136	107	77	63	51	41	34	26	21	19	9	7	6	4

Table W3K-44 Age Group = 61-70 Gender = FEMALE Occupational group = NON-MANUAL Total cases = 114

	Threshold dB HL≥															
	0	5	10	15	20	25	30	35	40	45	50	55	65	75	85	95
Prevalence	98.5	98.1	90.7	79.8	67.2	57.1	48.6	39.1	31.0	24.4	20.4	14.2	8.8	3.2	1.6	1.2
Lower CI	92.0	92.1	81.4	69.3	56.5	46.7	38.6	30.1	23.2	17.9	14.7	9.1	5.2	1.9	0.7	0.5
Upper CI	99.7	99.6	95.6	87.3	76.4	66.9	58.7	49.0	40.1	32.2	27.7	21.4	14.4	5.3	3.4	3.1
Number	113	112	107	96	82	72	63	53	43	36	31	26	19	10	6	5

Table W3K-45 Age Group = 71-80 Gender = FEMALE Occupational group = NON-MANUAL Total cases = 67

	Threshold dB HL≥															
	0	5	10	15	20	25	30	35	40	45	50	55	65	75	85	95
Prevalence	100.0	100.0	97.3	85.1	80.2	77.5	68.2	63.0	61.2	36.3	31.3	25.5	19.1	5.8	4.7	2.6
Lower CI	94.6	94.6	85.8	71.3	66.7	65.2	50.5	46.9	45.7	28.1	23.3	20.0	14.2	3.1	2.3	0.9
Upper CI	100.0	100.0	99.5	92.9	89.2	86.4	81.8	76.6	74.7	45.5	40.7	31.9	25.2	10.7	9.4	7.4
Number	67	67	66	60	57	56	53	49	48	43	34	30	21	10	8	4

Table W3K-46 Age Group = 18-30 Gender = MALE Occupational group = NON-MANUAL Total cases = 66

	Threshold dB HL≥															
	0	5	10	15	20	25	30	35	40	45	50	55	65	75	85	95
Prevalence	77.8	49.0	32.5	17.6	11.0	6.0	5.2	1.6	1.6	1.6	1.6	0.1	0.1	0.1	0.1	0.0
Lower CI	63.3	34.9	20.7	9.5	4.7	1.8	1.4	0.3	0.3	0.3	0.3	0.0	0.0	0.0	0.0	0.0
Upper CI	87.7	63.2	47.1	30.3	23.9	18.1	17.8	7.5	7.5	7.5	7.5	5.7	5.7	5.7	5.7	5.5
Number	53	36	23	12	8	5	4	2	2	2	2	1	1	1	1	0

Table W3K-47 Age Group = 31-40 Gender = MALE Occupational group = NON-MANUAL Total cases = 62

	Threshold dB HL≥															
	0	5	10	15	20	25	30	35	40	45	50	55	65	75	85	95
Prevalence	97.9	78.9	52.5	34.4	18.8	17.9	13.7	9.6	6.5	1.5	0.6	0.6	0.0	0.0	0.0	0.0
Lower CI	89.1	62.1	36.3	20.9	9.1	8.4	5.6	3.4	1.5	0.6	0.1	0.1	0.0	0.0	0.0	0.0
Upper CI	99.6	89.5	68.2	50.9	34.8	34.1	29.7	24.6	23.4	3.5	7.0	7.0	5.8	5.8	5.8	5.8
Number	61	52	36	24	14	13	10	7	4	3	1	1	0	0	0	0

Table W3K-48 Age Group = 41-50 Gender = MALE Occupational group = NON-MANUAL Total cases = 75

	Threshold dB HL≥															
	0	5	10	15	20	25	30	35	40	45	50	55	65	75	85	95
Prevalence	92.9	85.9	61.8	46.8	39.1	33.4	24.3	22.7	14.7	13.5	10.4	5.3	2.6	2.3	2.3	2.1
Lower CI	83.6	75.8	49.5	35.6	28.6	23.7	17.2	15.8	7.8	6.9	5.6	2.0	1.7	1.5	1.5	1.3
Upper CI	97.1	92.2	72.8	58.5	50.7	44.7	33.3	31.4	26.0	24.7	18.6	13.7	3.9	3.7	3.7	3.5
Number	70	64	49	39	32	27	20	18	12	11	10	6	5	4	4	3

Table W3K-49 Age Group = 51-60 Gender = MALE Occupational group = NON-MANUAL Total cases = 139

	Threshold dB HL≥															
	0	5	10	15	20	25	30	35	40	45	50	55	65	75	85	95
Prevalence	98.4	94.6	85.9	68.3	52.3	42.9	39.0	31.4	27.9	22.1	17.5	15.4	8.1	2.4	1.3	0.6
Lower CI	94.2	89.1	77.8	60.2	44.5	35.3	31.6	25.0	22.5	17.5	13.8	12.3	3.5	1.2	0.5	0.2
Upper CI	99.6	97.4	91.4	75.5	60.1	50.8	47.0	38.6	34.0	27.5	21.9	19.2	17.6	4.7	3.2	2.3
Number	137	131	122	103	82	67	60	50	46	36	29	25	15	7	4	2

Prevalence by age group, gender and occupational group for the worse ear over 3 kHz

Table W3K-50 Age Group = 61-70 Gender = MALE Occupational group = NON-MANUAL Total cases = 109

Threshold dB HL≥	0	5	10	15	20	25	30	35	40	45	50	55	65	75	85	95
Prevalence	100.0	100.0	99.2	87.2	80.6	75.0	66.1	50.1	40.4	37.4	33.4	30.3	20.6	9.3	7.4	5.7
Lower CI	96.6	96.6	95.9	70.0	64.5	59.2	51.0	40.9	33.3	30.6	27.4	24.5	15.2	6.1	3.9	2.7
Upper CI	100.0	100.0	99.9	95.2	90.5	86.2	78.5	59.2	48.0	44.6	40.0	36.7	27.3	14.0	13.4	11.9
Number	109	109	108	102	94	88	77	66	56	52	46	40	27	14	11	7

Table W3K-51 Age Group = 71-80 Gender = MALE Occupational group = NON-MANUAL Total cases = 54

Threshold dB HL≥	0	5	10	15	20	25	30	35	40	45	50	55	65	75	85	95
Prevalence	100.0	100.0	97.9	97.9	93.2	86.0	80.2	73.1	63.6	61.4	56.7	40.7	21.0	11.8	8.2	3.7
Lower CI	93.4	93.4	89.0	89.0	80.9	73.8	65.8	59.8	53.0	50.7	44.8	29.1	14.3	6.3	3.3	1.5
Upper CI	100.0	100.0	99.6	99.6	97.8	93.1	89.5	83.2	73.0	71.1	67.9	53.5	29.8	21.2	18.8	8.8
Number	54	54	53	53	51	47	44	41	36	34	32	23	13	6	5	3

Table W3K-52 Age Group = 18-30 Gender = FEMALE Occupational group = MANUAL Total cases = 109

Threshold dB HL≥	0	5	10	15	20	25	30	35	40	45	50	55	65	75	85	95
Prevalence	81.9	68.6	47.7	28.8	14.9	8.4	3.2	2.9	2.9	2.7	1.0	0.2	0.0	0.0	0.0	0.0
Lower CI	70.5	56.0	35.4	18.9	8.1	4.4	1.4	1.2	1.2	1.1	0.4	0.0	0.0	0.0	0.0	0.0
Upper CI	89.5	78.9	60.3	41.2	25.9	15.3	7.0	6.8	6.8	6.6	2.7	1.1	3.4	3.4	3.4	3.4
Number	92	76	57	36	19	14	9	8	8	7	4	1	0	0	0	0

Table W3K-53 Age Group = 31-40 Gender = FEMALE Occupational group = MANUAL Total cases = 101

Threshold dB HL≥	0	5	10	15	20	25	30	35	40	45	50	55	65	75	85	95
Prevalence	86.8	74.0	52.2	33.0	18.7	12.4	8.4	5.2	4.6	3.1	2.8	2.8	0.8	0.5	0.5	0.0
Lower CI	75.9	61.3	39.9	23.0	11.6	7.6	4.6	2.5	2.0	1.1	0.9	0.9	0.3	0.1	0.1	0.0
Upper CI	93.2	83.6	64.2	44.9	28.7	19.4	14.8	10.7	10.2	8.6	8.4	8.4	2.3	1.6	1.6	3.7
Number	92	80	61	44	28	22	17	11	6	6	5	5	3	2	2	0

Table W3K-54 Age Group = 41-50 Gender = FEMALE Occupational group = MANUAL Total cases = 133

Threshold dB HL≥	0	5	10	15	20	25	30	35	40	45	50	55	65	75	85	95
Prevalence	95.6	84.5	57.5	42.5	34.4	24.0	16.2	9.8	6.1	4.4	4.4	4.2	1.8	1.3	0.9	0.4
Lower CI	89.1	76.0	47.5	33.5	25.9	16.8	10.6	6.0	3.6	2.6	2.6	2.4	0.9	0.5	0.3	0.1
Upper CI	98.3	90.4	66.9	52.1	44.0	33.1	24.1	15.8	10.3	7.2	7.2	7.1	3.6	3.0	2.2	1.6
Number	129	118	87	66	52	38	28	20	16	13	13	12	7	5	4	2

Table W3K-55 Age Group = 51-60 Gender = FEMALE Occupational group = MANUAL Total cases = 164

Threshold dB HL≥	0	5	10	15	20	25	30	35	40	45	50	55	65	75	85	95
Prevalence	95.2	89.4	82.4	68.5	46.2	31.4	20.7	14.0	10.2	7.0	5.4	3.7	3.4	2.3	1.8	0.7
Lower CI	89.7	82.6	73.9	59.3	37.3	23.9	15.2	10.0	7.0	4.5	3.3	2.2	1.9	1.3	0.8	0.2
Upper CI	97.9	93.7	88.5	76.4	55.3	40.1	27.4	19.3	14.6	10.7	8.7	6.1	5.8	4.2	3.7	2.0
Number	158	150	143	124	93	69	54	44	36	27	22	18	15	12	8	3

Table W3K-56 Age Group = 61-70 Gender = FEMALE Occupational group = MANUAL Total cases = 124

Threshold dB HL≥	0	5	10	15	20	25	30	35	40	45	50	55	65	75	85	95
Prevalence	98.5	98.5	88.4	77.3	63.0	42.9	39.8	33.3	25.5	21.2	18.2	16.1	11.7	5.4	2.7	2.1
Lower CI	91.7	91.7	78.8	66.9	51.4	32.8	29.9	24.5	17.9	14.4	12.3	10.5	6.7	3.4	1.9	1.6
Upper CI	99.7	99.7	94.0	85.1	73.2	53.7	50.7	43.4	35.0	30.0	26.2	23.9	19.7	8.6	4.0	2.8
Number	123	123	111	100	90	71	66	60	49	42	38	33	21	12	5	3

Prevalence by age group, gender and occupational group for the worse ear over 3 kHz

Table W3K-57 Age Group = 71-80 Gender = FEMALE Occupational group = MANUAL Total cases = 76

	Threshold dB HL≥															
	0	5	10	15	20	25	30	35	40	45	50	55	65	75	85	95
**Prevalence**	**100.0**	**100.0**	**100.0**	**94.8**	**74.1**	**73.7**	**70.3**	**66.5**	**63.0**	**54.7**	**43.0**	**33.7**	**19.6**	**8.0**	**5.6**	**2.0**
**Lower CI**	95.2	95.2	95.2	74.9	54.7	54.4	51.1	48.4	45.1	39.5	30.9	22.1	10.8	5.1	3.3	0.9
**Upper CI**	100.0	100.0	100.0	99.1	87.2	86.8	84.3	80.8	77.9	69.1	56.0	47.6	32.9	12.4	9.5	4.2
**Number**	76	76	76	75	70	69	68	66	61	55	50	43	26	16	12	5

Table W3K-58 Age Group = 18-30 Gender = MALE Occupational group = MANUAL Total cases = 109

	Threshold dB HL≥															
	0	5	10	15	20	25	30	35	40	45	50	55	65	75	85	95
**Prevalence**	**84.4**	**67.3**	**51.1**	**34.3**	**22.6**	**19.1**	**8.6**	**6.8**	**4.5**	**3.0**	**1.8**	**1.8**	**1.0**	**1.0**	**0.1**	**0.1**
**Lower CI**	72.5	54.2	38.6	23.5	14.2	11.4	4.6	3.2	1.8	1.2	0.6	0.6	0.2	0.2	0.0	0.0
**Upper CI**	91.7	78.1	63.5	46.9	34.0	30.2	15.3	14.0	10.5	7.4	5.3	5.3	5.1	5.1	0.4	0.4
**Number**	98	80	65	46	32	25	17	12	8	7	5	5	2	2	1	1

Table W3K-59 Age Group = 31-40 Gender = MALE Occupational group = MANUAL Total cases = 80

	Threshold dB HL≥															
	0	5	10	15	20	25	30	35	40	45	50	55	65	75	85	95
**Prevalence**	**94.7**	**80.2**	**67.0**	**53.4**	**34.5**	**16.9**	**15.7**	**11.9**	**8.7**	**6.5**	**6.3**	**6.3**	**3.6**	**2.8**	**2.8**	**2.1**
**Lower CI**	86.6	66.5	51.7	38.7	22.1	9.0	8.0	5.9	4.5	2.9	2.8	2.8	1.2	0.8	0.8	0.4
**Upper CI**	98.0	89.2	79.4	67.5	49.5	29.3	28.3	22.7	16.2	14.1	14.0	14.0	10.4	10.1	10.1	9.9
**Number**	75	66	56	42	31	21	18	16	14	10	9	9	5	3	3	2

Table W3K-60 Age Group = 41-50 Gender = MALE Occupational group = MANUAL Total cases = 123

	Threshold dB HL≥															
	0	5	10	15	20	25	30	35	40	45	50	55	65	75	85	95
**Prevalence**	**100.0**	**97.7**	**87.6**	**72.6**	**58.2**	**44.7**	**36.0**	**28.7**	**24.7**	**19.6**	**12.5**	**12.0**	**7.7**	**1.8**	**0.4**	**0.4**
**Lower CI**	97.0	91.4	77.9	61.6	46.8	34.2	26.7	20.6	17.3	12.8	8.1	7.7	4.1	0.9	0.1	0.1
**Upper CI**	100.0	99.4	93.4	81.5	68.7	55.8	46.5	38.4	34.1	28.6	18.8	18.3	13.7	3.6	1.5	1.5
**Number**	123	120	111	95	79	65	56	48	43	35	28	27	16	7	2	2

Table W3K-61 Age Group = 51-60 Gender = MALE Occupational group = MANUAL Total cases = 195

	Threshold dB HL≥															
	0	5	10	15	20	25	30	35	40	45	50	55	65	75	85	95
**Prevalence**	**100.0**	**98.4**	**90.2**	**83.6**	**78.0**	**64.9**	**60.6**	**50.7**	**46.4**	**37.5**	**33.1**	**25.7**	**13.5**	**9.4**	**4.9**	**1.9**
**Lower CI**	98.1	94.4	83.1	76.2	69.8	56.3	52.0	42.6	38.5	30.7	26.8	20.2	9.7	6.2	2.5	0.9
**Upper CI**	100.0	99.6	94.5	89.0	84.4	72.7	68.5	58.7	54.4	44.8	40.1	32.1	18.5	14.0	9.4	3.9
**Number**	195	193	183	171	162	144	137	122	113	97	86	69	41	28	14	7

Table W3K-62 Age Group = 61-70 Gender = MALE Occupational group = MANUAL Total cases = 169

	Threshold dB HL≥															
	0	5	10	15	20	25	30	35	40	45	50	55	65	75	85	95
**Prevalence**	**100.0**	**100.0**	**98.4**	**94.8**	**94.5**	**91.1**	**84.1**	**75.1**	**65.0**	**58.9**	**49.8**	**45.0**	**25.9**	**17.6**	**8.5**	**3.2**
**Lower CI**	97.8	97.8	93.5	85.8	85.7	82.1	74.4	66.5	55.2	49.4	41.1	35.8	18.9	11.5	5.4	1.9
**Upper CI**	100.0	100.0	99.6	98.2	98.0	95.8	90.6	82.2	73.6	67.8	58.6	54.6	34.4	26.0	13.2	5.4
**Number**	169	169	167	165	164	159	150	140	126	119	107	98	65	41	24	13

Table W3K-63 Age Group = 71-80 Gender = MALE Occupational group = MANUAL Total cases = 71

	Threshold dB HL≥															
	0	5	10	15	20	25	30	35	40	45	50	55	65	75	85	95
**Prevalence**	**100.0**	**100.0**	**94.2**	**94.2**	**94.2**	**93.8**	**90.4**	**89.2**	**89.2**	**83.1**	**64.2**	**57.8**	**44.0**	**29.7**	**12.4**	**6.4**
**Lower CI**	94.9	94.9	72.4	72.4	72.4	72.6	71.1	70.8	70.8	65.8	49.3	41.5	30.6	17.6	7.9	4.3
**Upper CI**	100.0	100.0	99.0	99.0	99.0	98.8	97.3	96.6	96.6	92.6	76.7	72.6	58.4	45.5	18.9	9.5
**Number**	71	71	70	70	70	69	67	66	66	61	57	53	42	30	16	10

Prevalence by age group, gender and occupational group for the worse ear over 4 kHz

Table W4K-1 Age Group = 18-80 Gender = OVERALL Occupational group = OVERALL Total cases = 2662

	Threshold dB HL≥															
	0	5	10	15	20	25	30	35	40	45	50	55	65	75	85	95
**Prevalence**	**96.2**	**88.9**	**76.9**	**64.7**	**53.1**	**44.7**	**38.1**	**32.1**	**27.2**	**23.5**	**20.3**	**17.1**	**10.8**	**6.8**	**4.1**	**2.3**
**Lower CI**	94.9	87.1	74.9	62.7	51.2	42.9	36.3	30.5	25.6	22.1	18.9	15.8	9.9	6.1	3.5	1.9
**Upper CI**	97.1	90.5	78.9	66.7	55.1	46.6	39.8	33.8	28.8	25.0	21.8	18.3	11.9	7.7	4.7	2.8
**Number**	2605	2503	2293	2041	1795	1578	1397	1245	1103	988	882	772	545	371	239	143

Table W4K-2 Age Group = 18-80 Gender = FEMALE Occupational group = OVERALL Total cases = 1386

	Threshold dB HL≥															
	0	5	10	15	20	25	30	35	40	45	50	55	65	75	85	95
**Prevalence**	**95.0**	**87.4**	**74.2**	**59.3**	**46.8**	**37.3**	**30.6**	**23.6**	**19.5**	**15.9**	**13.5**	**10.6**	**6.7**	**4.5**	**2.4**	**1.4**
**Lower CI**	93.2	84.9	71.5	56.7	44.3	35.1	28.6	21.6	17.6	14.2	11.9	9.3	5.6	3.7	2.0	1.0
**Upper CI**	96.4	89.5	76.8	61.9	49.3	39.5	32.8	25.7	21.5	17.8	15.3	12.1	7.8	5.5	3.1	1.8
**Number**	1345	1280	1144	974	819	681	572	482	411	353	306	259	179	131	83	53

Table W4K-3 Age Group = 18-80 Gender = MALE Occupational group = OVERALL Total cases = 1276

	Threshold dB HL≥															
	0	5	10	15	20	25	30	35	40	45	50	55	65	75	85	95
**Prevalence**	**97.5**	**90.7**	**80.0**	**70.8**	**60.3**	**53.2**	**46.5**	**41.8**	**35.9**	**32.1**	**28.0**	**24.3**	**15.5**	**9.5**	**5.9**	**3.3**
**Lower CI**	95.6	87.8	76.9	67.6	57.2	50.2	43.6	39.1	33.4	29.7	25.8	22.3	13.9	8.2	4.9	2.5
**Upper CI**	98.5	92.9	82.9	73.8	63.3	56.1	49.3	44.5	38.5	34.5	30.4	26.5	17.3	11.0	7.2	4.2
**Number**	1260	1223	1149	1067	976	897	825	763	692	635	576	513	366	240	156	90

Table W4K-4 Age Group = 18-30 Gender = OVERALL Occupational group = OVERALL Total cases = 401

	Threshold dB HL≥															
	0	5	10	15	20	25	30	35	40	45	50	55	65	75	85	95
**Prevalence**	**89.8**	**72.2**	**49.0**	**30.1**	**19.5**	**13.2**	**10.4**	**7.0**	**5.0**	**3.0**	**2.6**	**2.1**	**1.5**	**0.5**	**0.3**	**0.0**
**Lower CI**	85.7	66.6	43.5	25.4	15.5	9.8	7.3	4.6	3.1	1.7	1.4	1.0	0.6	0.2	0.1	0.0
**Upper CI**	92.8	77.2	54.6	35.4	24.3	17.5	14.4	10.3	8.0	5.3	4.8	4.4	3.7	1.6	1.5	0.1
**Number**	365	312	226	150	100	67	53	39	30	19	16	11	8	5	3	1

Table W4K-5 Age Group = 31-40 Gender = OVERALL Occupational group = OVERALL Total cases = 331

	Threshold dB HL≥															
	0	5	10	15	20	25	30	35	40	45	50	55	65	75	85	95
**Prevalence**	**95.4**	**85.8**	**69.8**	**52.7**	**34.2**	**24.5**	**17.8**	**14.2**	**11.2**	**10.1**	**7.5**	**5.5**	**2.7**	**1.6**	**1.5**	**1.0**
**Lower CI**	91.8	80.6	63.5	46.4	28.6	19.8	13.7	10.5	7.9	6.9	4.8	3.4	1.5	0.7	0.6	0.3
**Upper CI**	97.4	89.8	75.5	58.9	40.3	29.9	22.7	19.0	15.7	14.6	11.6	8.8	4.8	3.6	3.5	3.0
**Number**	318	293	248	195	143	107	83	66	54	45	34	26	16	10	9	5

Table W4K-6 Age Group = 41-50 Gender = OVERALL Occupational group = OVERALL Total cases = 450

	Threshold dB HL≥															
	0	5	10	15	20	25	30	35	40	45	50	55	65	75	85	95
**Prevalence**	**98.2**	**94.0**	**82.8**	**67.6**	**53.2**	**42.0**	**32.4**	**25.4**	**19.1**	**15.2**	**12.1**	**9.6**	**6.6**	**4.1**	**2.8**	**2.1**
**Lower CI**	96.1	91.0	78.5	62.7	48.4	37.4	28.2	21.7	15.9	12.4	9.6	7.4	4.8	2.7	1.7	1.2
**Upper CI**	99.2	96.0	86.4	72.1	57.9	46.7	36.9	29.6	22.8	18.5	15.2	12.4	9.2	6.4	4.8	3.6
**Number**	444	428	386	322	267	219	177	145	115	95	79	66	45	29	21	17

Table W4K-7 Age Group = 51-60 Gender = OVERALL Occupational group = OVERALL Total cases = 681

	Threshold dB HL≥															
	0	5	10	15	20	25	30	35	40	45	50	55	65	75	85	95
**Prevalence**	**99.7**	**98.9**	**93.9**	**85.4**	**73.8**	**60.8**	**49.9**	**41.1**	**34.2**	**29.9**	**25.9**	**21.0**	**12.3**	**8.5**	**5.3**	**3.2**
**Lower CI**	98.7	97.7	91.5	82.2	70.0	56.8	45.9	37.4	30.8	26.8	23.0	18.4	10.3	6.8	4.0	2.2
**Upper CI**	99.9	99.5	95.7	88.2	77.3	64.7	53.9	44.9	37.7	33.2	29.0	23.9	14.7	10.7	7.0	4.6
**Number**	679	673	645	599	539	467	409	356	310	280	248	208	137	93	64	39

Prevalence by age group, gender and occupational group for the worse ear over 4 kHz

Table W4K-15 Age Group = 51-60 Gender = FEMALE Occupational group = OVERALL Total cases = 340

Threshold dB HL≥	0	5	10	15	20	25	30	35	40	45	50	55	65	75	85	95
Prevalence	**99.7**	**98.9**	**92.0**	**78.9**	**64.7**	**48.6**	**37.0**	**26.2**	**19.0**	**14.5**	**10.2**	**7.4**	**5.2**	**4.4**	**3.2**	**2.0**
Lower CI	98.5	97.0	88.0	73.6	58.9	42.8	31.6	21.7	15.3	11.4	7.8	5.4	3.5	2.8	1.9	1.0
Upper CI	100.0	99.6	94.7	83.4	70.0	54.4	42.7	31.4	23.3	18.2	13.3	10.1	7.6	6.7	5.2	3.7
Number	339	335	315	280	241	193	158	125	100	84	66	52	37	30	23	15

Table W4K-16 Age Group = 61-70 Gender = FEMALE Occupational group = OVERALL Total cases = 243

Threshold dB HL≥	0	5	10	15	20	25	30	35	40	45	50	55	65	75	85	95
Prevalence	**100.0**	**99.4**	**96.1**	**92.2**	**82.0**	**72.0**	**61.7**	**50.2**	**40.8**	**31.8**	**27.2**	**22.8**	**14.0**	**9.0**	**3.9**	**2.3**
Lower CI	98.4	97.8	92.3	87.4	75.5	65.1	54.6	42.7	33.8	25.5	21.3	17.4	9.8	5.9	2.6	1.4
Upper CI	100.0	99.8	98.1	95.2	87.0	78.1	68.3	57.6	48.2	38.8	34.1	29.1	19.4	13.6	5.9	3.7
Number	243	241	234	225	206	186	161	141	122	101	88	77	54	38	20	14

Table W4K-17 Age Group = 71-80 Gender = FEMALE Occupational group = OVERALL Total cases = 143

Threshold dB HL≥	0	5	10	15	20	25	30	35	40	45	50	55	65	75	85	95
Prevalence	**100.0**	**100.0**	**97.6**	**97.0**	**89.3**	**89.1**	**87.0**	**77.1**	**70.6**	**64.7**	**59.2**	**46.4**	**29.2**	**19.7**	**9.3**	**4.4**
Lower CI	97.4	97.4	87.3	87.6	78.5	78.4	76.4	63.8	57.2	51.9	47.3	37.4	22.1	13.9	6.5	2.7
Upper CI	100.0	100.0	99.6	99.3	95.0	94.8	93.3	86.5	81.1	75.7	70.1	55.7	37.4	27.4	13.1	7.2
Number	143	143	142	141	136	135	132	127	122	116	109	97	67	49	29	16

Table W4K-18 Age Group = 18-30 Gender = MALE Occupational group = OVERALL Total cases = 177

Threshold dB HL≥	0	5	10	15	20	25	30	35	40	45	50	55	65	75	85	95
Prevalence	**93.7**	**75.0**	**52.4**	**35.4**	**23.0**	**18.0**	**13.8**	**10.9**	**7.2**	**4.3**	**3.9**	**3.4**	**2.6**	**0.8**	**0.6**	**0.0**
Lower CI	87.4	66.2	43.9	27.8	16.6	12.2	8.7	6.6	3.8	2.1	1.8	1.4	1.0	0.2	0.1	0.0
Upper CI	97.0	82.1	60.7	43.8	31.1	25.7	21.1	17.5	13.3	8.6	8.2	7.8	7.0	3.1	3.0	0.2
Number	168	145	107	78	51	38	30	25	18	13	11	8	6	4	3	1

Table W4K-19 Age Group = 31-40 Gender = MALE Occupational group = OVERALL Total cases = 142

Threshold dB HL≥	0	5	10	15	20	25	30	35	40	45	50	55	65	75	85	95
Prevalence	**97.2**	**90.6**	**76.5**	**64.4**	**43.7**	**32.5**	**26.6**	**22.1**	**17.2**	**15.3**	**10.4**	**7.7**	**3.0**	**2.2**	**2.2**	**1.4**
Lower CI	91.4	82.4	66.2	53.9	34.0	24.3	19.0	14.9	11.0	9.3	5.7	4.1	1.2	0.7	0.7	0.3
Upper CI	99.2	95.2	84.4	73.6	53.9	42.0	35.9	31.4	25.9	24.1	18.4	14.3	7.2	6.6	6.6	6.2
Number	139	130	116	97	75	57	50	40	34	27	19	15	8	5	5	3

Table W4K-20 Age Group = 41-50 Gender = MALE Occupational group = OVERALL Total cases = 203

Threshold dB HL≥	0	5	10	15	20	25	30	35	40	45	50	55	65	75	85	95
Prevalence	**98.0**	**96.5**	**87.8**	**77.0**	**66.6**	**56.4**	**45.6**	**38.8**	**30.3**	**25.4**	**20.5**	**16.2**	**11.6**	**7.3**	**4.8**	**3.4**
Lower CI	94.4	92.6	81.7	69.8	59.0	48.9	38.5	32.0	24.3	19.9	15.5	11.9	8.0	4.4	2.5	1.7
Upper CI	99.3	98.4	92.1	82.8	73.5	63.7	53.0	46.0	37.0	31.8	26.6	21.6	16.7	11.9	8.9	6.6
Number	200	197	184	164	148	131	112	96	80	67	56	47	34	21	14	11

Table W4K-21 Age Group = 51-60 Gender = MALE Occupational group = OVERALL Total cases = 341

Threshold dB HL≥	0	5	10	15	20	25	30	35	40	45	50	55	65	75	85	95
Prevalence	**99.6**	**99.0**	**96.1**	**92.8**	**84.2**	**74.6**	**64.4**	**57.8**	**51.2**	**47.1**	**43.5**	**36.2**	**20.2**	**13.1**	**7.6**	**4.6**
Lower CI	97.6	96.9	93.2	89.0	79.0	68.9	58.5	51.9	45.5	41.5	38.0	31.1	16.4	10.0	5.4	3.0
Upper CI	99.9	99.7	97.8	95.3	88.2	79.6	69.9	63.5	56.9	52.8	49.1	41.6	24.6	17.0	10.6	7.0
Number	340	338	330	319	298	274	251	231	210	196	182	156	100	63	41	24

Prevalence by age group, gender and occupational group for the worse ear over 4 kHz

Table W4K-8 Age Group = 61-70 Gender = OVERALL Occupational group = OVERALL Total cases = 527

Threshold dB HL≥	0	5	10	15	20	25	30	35	40	45	50	55	65	75	85	95
Prevalence	**100.0**	**99.6**	**97.9**	**95.3**	**89.3**	**82.9**	**73.6**	**64.9**	**56.1**	**49.0**	**42.3**	**37.3**	**23.7**	**14.4**	**8.4**	**4.4**
Lower CI	99.3	98.8	95.8	92.7	85.6	78.8	68.8	59.7	50.9	44.0	37.5	32.8	20.3	11.8	6.7	3.4
Upper CI	100.0	99.9	98.9	97.0	92.1	86.3	77.8	69.7	61.2	53.9	47.2	42.0	27.4	17.6	10.6	5.8
Number	527	525	517	505	483	457	418	389	355	320	287	260	191	129	80	47

Table W4K-9 Age Group = 71-80 Gender = OVERALL Occupational group = OVERALL Total cases = 272

Threshold dB HL≥	0	5	10	15	20	25	30	35	40	45	50	55	65	75	85	95
Prevalence	**100.0**	**100.0**	**98.5**	**98.2**	**92.5**	**91.8**	**90.2**	**83.8**	**77.4**	**71.2**	**66.1**	**56.9**	**38.5**	**25.1**	**12.9**	**6.3**
Lower CI	98.6	98.6	92.0	92.2	85.7	85.1	83.5	75.4	68.7	62.4	57.6	49.8	32.4	19.9	9.5	4.1
Upper CI	100.0	100.0	99.7	99.6	96.2	95.7	94.4	89.7	84.3	78.6	73.6	63.8	44.9	31.2	17.2	9.6
Number	272	272	271	270	263	261	257	250	239	229	218	201	148	105	62	34

Table W4K-10 Age Group = 18-80 Gender = OVERALL Occupational group = NON-MANUAL Total cases = 1185

Threshold dB HL≥	0	5	10	15	20	25	30	35	40	45	50	55	65	75	85	95
Prevalence	**95.9**	**87.1**	**73.2**	**60.6**	**47.9**	**40.2**	**33.6**	**28.9**	**24.9**	**21.1**	**17.9**	**13.7**	**9.2**	**4.8**	**2.8**	**1.8**
Lower CI	94.2	84.4	70.2	57.7	45.2	37.6	31.1	26.6	22.6	19.0	16.0	12.0	7.8	4.0	2.3	1.3
Upper CI	97.2	89.4	75.9	63.4	50.7	42.8	36.2	31.4	27.3	23.5	20.1	15.6	10.9	5.9	3.5	2.4
Number	1153	1090	969	847	719	618	533	473	412	365	319	267	188	119	78	50

Table W4K-11 Age Group = 18-80 Gender = OVERALL Occupational group = MANUAL Total cases = 1473

Threshold dB HL≥	0	5	10	15	20	25	30	35	40	45	50	55	65	75	85	95
Prevalence	**96.4**	**90.8**	**81.3**	**68.8**	**58.1**	**48.5**	**42.1**	**35.1**	**29.5**	**25.7**	**21.9**	**19.4**	**11.6**	**8.2**	**5.0**	**2.4**
Lower CI	94.3	88.0	78.2	65.7	55.1	45.8	39.6	32.7	27.3	23.7	20.2	17.8	10.3	7.0	4.1	2.0
Upper CI	97.8	93.0	84.0	71.8	61.1	51.3	44.7	37.5	31.8	27.8	23.8	21.2	13.1	9.6	6.1	3.1
Number	1448	1409	1321	1192	1074	958	862	770	690	622	562	504	356	251	161	93

Table W4K-12 Age Group = 18-30 Gender = FEMALE Occupational group = OVERALL Total cases = 224

Threshold dB HL≥	0	5	10	15	20	25	30	35	40	45	50	55	65	75	85	95
Prevalence	**86.1**	**69.5**	**45.8**	**24.9**	**16.0**	**8.5**	**7.0**	**3.1**	**2.8**	**1.7**	**1.2**	**0.8**	**0.3**	**0.2**	**0.0**	**0.0**
Lower CI	79.9	62.1	38.7	19.3	11.7	5.4	4.2	1.7	1.5	0.7	0.5	0.2	0.1	0.0	0.0	0.0
Upper CI	90.6	76.0	53.1	31.3	21.6	12.9	11.3	5.4	5.1	4.1	3.2	2.9	1.3	1.3	1.7	1.7
Number	197	167	119	72	49	29	23	14	12	6	5	3	2	1	0	0

Table W4K-13 Age Group = 31-40 Gender = FEMALE Occupational group = OVERALL Total cases = 189

Threshold dB HL≥	0	5	10	15	20	25	30	35	40	45	50	55	65	75	85	95
Prevalence	**93.6**	**81.5**	**63.7**	**42.0**	**25.5**	**17.0**	**10.0**	**7.1**	**5.8**	**5.4**	**4.8**	**3.4**	**2.3**	**1.2**	**0.9**	**0.6**
Lower CI	87.9	73.8	55.4	34.3	19.6	12.6	6.8	4.6	3.5	3.1	2.7	1.7	1.1	0.5	0.3	0.2
Upper CI	96.7	87.3	71.2	50.1	32.5	22.6	14.4	10.8	9.4	9.0	8.5	6.6	4.9	2.7	2.3	2.0
Number	179	163	132	98	68	50	33	26	20	18	15	11	8	5	4	2

Table W4K-14 Age Group = 41-50 Gender = FEMALE Occupational group = OVERALL Total cases = 247

Threshold dB HL≥	0	5	10	15	20	25	30	35	40	45	50	55	65	75	85	95
Prevalence	**98.3**	**91.7**	**78.3**	**59.1**	**40.9**	**28.8**	**20.3**	**13.3**	**8.9**	**5.9**	**4.5**	**3.7**	**2.1**	**1.2**	**1.0**	**0.9**
Lower CI	95.2	86.7	71.9	52.2	34.8	23.4	15.7	9.9	6.2	4.1	3.1	2.3	1.1	0.6	0.5	0.4
Upper CI	99.4	94.9	83.5	65.7	47.3	34.8	25.8	17.6	12.6	8.5	6.5	5.7	3.8	2.4	2.1	1.9
Number	244	231	202	158	119	88	65	49	35	28	23	19	11	8	7	6

Prevalence by age group, gender and occupational group for the worse ear over 4 kHz

Table W4K-22 Age Group = 61-70 Gender = MALE Occupational group = OVERALL Total cases = 284

	Threshold dB HL≥															
	**0**	**5**	**10**	**15**	**20**	**25**	**30**	**35**	**40**	**45**	**50**	**55**	**65**	**75**	**85**	**95**
**Prevalence**	**100.0**	**100.0**	**99.9**	**99.0**	**97.8**	**95.4**	**87.1**	**81.9**	**73.9**	**68.9**	**59.8**	**54.2**	**35.1**	**20.7**	**13.8**	**6.9**
**Lower CI**	98.7	98.7	99.3	97.2	94.9	91.7	80.5	74.6	66.1	61.2	52.3	47.0	30.1	16.7	10.4	4.9
**Upper CI**	100.0	100.0	100.0	99.6	99.0	97.5	91.7	87.4	80.4	75.7	67.0	61.2	40.4	25.5	18.0	9.5
**Number**	284	284	283	280	277	271	257	248	233	219	199	183	137	91	60	33

Table W4K-23 Age Group = 71-80 Gender = MALE Occupational group = OVERALL Total cases = 129

	Threshold dB HL≥															
	**0**	**5**	**10**	**15**	**20**	**25**	**30**	**35**	**40**	**45**	**50**	**55**	**65**	**75**	**85**	**95**
**Prevalence**	**100.0**	**100.0**	**100.0**	**100.0**	**97.3**	**96.0**	**95.1**	**93.4**	**87.4**	**80.4**	**76.1**	**72.2**	**52.1**	**33.0**	**18.7**	**9.0**
**Lower CI**	97.1	97.1	97.1	97.1	91.3	90.0	88.7	87.3	78.7	69.7	64.9	61.0	41.9	24.3	11.9	4.8
**Upper CI**	100.0	100.0	100.0	100.0	99.2	98.5	97.9	96.7	92.8	87.9	84.6	81.2	62.1	43.1	28.1	16.5
**Number**	129	129	129	129	127	126	125	123	117	113	109	104	81	56	33	18

Table W4K-24 Age Group = 18-30 Gender = OVERALL Occupational group = NON-MANUAL Total cases = 181

	Threshold dB HL≥															
	**0**	**5**	**10**	**15**	**20**	**25**	**30**	**35**	**40**	**45**	**50**	**55**	**65**	**75**	**85**	**95**
**Prevalence**	**89.1**	**67.7**	**41.5**	**21.6**	**12.8**	**9.5**	**7.7**	**5.5**	**5.4**	**2.7**	**2.7**	**2.7**	**2.7**	**0.3**	**0.1**	**0.0**
**Lower CI**	83.1	59.2	34.4	15.7	8.2	5.4	4.0	2.4	2.3	0.7	0.7	0.7	0.7	0.1	0.0	0.0
**Upper CI**	93.2	75.1	49.0	29.0	19.4	16.1	14.2	12.0	12.0	9.2	9.2	9.2	9.2	1.0	2.2	2.1
**Number**	161	129	80	45	30	20	15	10	9	4	4	4	4	2	1	0

Table W4K-25 Age Group = 31-40 Gender = OVERALL Occupational group = NON-MANUAL Total cases = 148

	Threshold dB HL≥															
	**0**	**5**	**10**	**15**	**20**	**25**	**30**	**35**	**40**	**45**	**50**	**55**	**65**	**75**	**85**	**95**
**Prevalence**	**95.6**	**84.1**	**64.4**	**48.4**	**28.1**	**19.1**	**13.6**	**11.2**	**8.5**	**7.8**	**7.8**	**4.7**	**1.6**	**0.3**	**0.3**	**0.3**
**Lower CI**	91.6	76.9	54.5	39.3	20.4	12.5	8.1	6.2	4.4	3.8	3.8	2.3	0.6	0.1	0.1	0.1
**Upper CI**	97.8	89.4	73.2	57.7	37.4	27.9	22.1	19.6	15.9	15.4	15.4	9.2	3.7	1.7	1.7	1.7
**Number**	142	126	102	78	50	34	25	20	17	14	14	9	4	1	1	1

Table W4K-26 Age Group = 41-50 Gender = OVERALL Occupational group = NON-MANUAL Total cases = 188

	Threshold dB HL≥															
	**0**	**5**	**10**	**15**	**20**	**25**	**30**	**35**	**40**	**45**	**50**	**55**	**65**	**75**	**85**	**95**
**Prevalence**	**97.8**	**93.0**	**78.5**	**66.3**	**45.1**	**32.6**	**25.3**	**20.4**	**14.7**	**11.8**	**8.4**	**6.5**	**4.5**	**1.5**	**1.5**	**1.2**
**Lower CI**	94.6	88.6	71.5	58.9	38.1	26.6	19.2	14.9	10.0	7.5	5.4	3.9	2.5	0.8	0.8	0.7
**Upper CI**	99.1	95.8	84.1	72.9	52.4	39.3	32.6	27.3	21.1	18.1	12.8	10.7	7.9	2.6	2.6	2.2
**Number**	184	174	150	126	90	68	55	44	33	26	21	16	10	5	5	4

Table W4K-27 Age Group = 51-60 Gender = OVERALL Occupational group = NON-MANUAL Total cases = 317

	Threshold dB HL≥															
	**0**	**5**	**10**	**15**	**20**	**25**	**30**	**35**	**40**	**45**	**50**	**55**	**65**	**75**	**85**	**95**
**Prevalence**	**99.4**	**98.6**	**92.8**	**81.5**	**69.1**	**53.8**	**42.0**	**33.8**	**29.3**	**25.8**	**21.4**	**13.6**	**8.8**	**6.2**	**3.0**	**2.0**
**Lower CI**	97.9	96.6	89.3	76.6	63.3	47.9	36.7	29.2	24.9	21.9	17.8	9.9	5.8	3.6	1.8	1.1
**Upper CI**	99.8	99.4	95.2	85.6	74.4	59.7	47.5	38.8	34.0	30.1	25.5	18.3	13.2	10.7	4.8	3.7
**Number**	315	311	291	259	228	186	156	133	115	104	87	67	46	31	20	14

Table W4K-28 Age Group = 61-70 Gender = OVERALL Occupational group = NON-MANUAL Total cases = 228

	Threshold dB HL≥															
	**0**	**5**	**10**	**15**	**20**	**25**	**30**	**35**	**40**	**45**	**50**	**55**	**65**	**75**	**85**	**95**
**Prevalence**	**100.0**	**99.2**	**97.5**	**94.5**	**87.2**	**83.3**	**70.8**	**61.0**	**51.4**	**44.7**	**36.9**	**32.3**	**21.7**	**12.1**	**9.2**	**5.4**
**Lower CI**	98.3	95.7	93.7	90.5	81.5	77.4	62.5	54.7	44.6	38.4	31.1	27.1	17.9	9.6	7.1	3.5
**Upper CI**	100.0	99.9	99.1	96.9	91.3	88.0	77.8	67.0	58.1	51.2	43.0	38.0	26.2	15.2	12.0	8.2
**Number**	228	227	223	217	204	195	171	157	136	121	104	92	70	46	33	20

Prevalence by age group, gender and occupational group for the worse ear over 4 kHz

Table W4K-29 Age Group = 71-80 Gender = OVERALL Occupational group = NON-MANUAL Total cases = 123

Threshold dB HL≥	0	5	10	15	20	25	30	35	40	45	50	55	65	75	85	95
Prevalence	**100.0**	**100.0**	**100.0**	**99.1**	**93.5**	**92.5**	**89.6**	**87.6**	**80.1**	**70.4**	**62.3**	**47.8**	**32.3**	**18.8**	**8.2**	**4.7**
Lower CI	97.0	97.0	97.0	95.1	84.7	84.1	81.0	79.0	70.2	57.7	53.1	39.7	25.1	12.8	5.3	2.3
Upper CI	100.0	100.0	100.0	99.8	97.3	96.6	94.5	93.1	87.3	80.5	70.7	55.9	40.4	26.8	12.5	9.3
Number	123	123	123	122	117	115	111	109	102	96	89	79	54	34	18	11

Table W4K-30 Age Group = 18-30 Gender = OVERALL Occupational group = MANUAL Total cases = 218

Threshold dB HL≥	0	5	10	15	20	25	30	35	40	45	50	55	65	75	85	95
Prevalence	**90.4**	**76.7**	**58.6**	**39.4**	**26.3**	**16.1**	**12.9**	**8.4**	**5.6**	**3.4**	**2.6**	**2.0**	**0.8**	**0.7**	**0.5**	**0.0**
Lower CI	83.3	67.8	49.8	31.3	19.4	10.7	8.1	5.0	2.9	1.8	1.3	0.8	0.2	0.2	0.1	0.0
Upper CI	94.7	83.7	66.8	48.1	34.7	23.4	19.9	13.7	10.5	6.3	5.2	4.7	2.6	2.6	2.6	0.2
Number	202	181	145	105	70	47	38	29	21	15	12	7	4	3	2	1

Table W4K-31 Age Group = 31-40 Gender = OVERALL Occupational group = MANUAL Total cases = 183

Threshold dB HL≥	0	5	10	15	20	25	30	35	40	45	50	55	65	75	85	95
Prevalence	**95.2**	**87.6**	**74.6**	**56.0**	**39.7**	**29.9**	**22.4**	**18.0**	**14.5**	**13.1**	**7.3**	**5.8**	**3.1**	**2.5**	**2.2**	**1.4**
Lower CI	89.3	79.3	65.5	47.2	31.8	23.1	16.7	12.6	9.4	8.1	4.0	3.0	1.5	1.1	0.9	0.4
Upper CI	98.0	92.9	81.9	64.4	48.1	37.6	29.5	25.2	21.8	20.5	12.9	11.0	6.2	5.7	5.4	4.9
Number	176	167	146	117	93	73	58	46	37	31	20	17	12	9	8	4

Table W4K-32 Age Group = 41-50 Gender = OVERALL Occupational group = MANUAL Total cases = 262

Threshold dB HL≥	0	5	10	15	20	25	30	35	40	45	50	55	65	75	85	95
Prevalence	**98.9**	**95.9**	**87.8**	**70.1**	**61.8**	**50.3**	**38.6**	**30.4**	**23.5**	**18.8**	**14.7**	**12.0**	**8.4**	**6.0**	**3.9**	**2.7**
Lower CI	95.9	92.1	82.4	63.5	54.9	43.3	32.2	24.8	18.6	14.5	11.0	8.7	5.5	3.5	1.9	1.3
Upper CI	99.7	97.9	91.7	76.0	68.3	57.3	45.4	36.7	29.2	24.0	19.3	16.2	12.5	10.1	7.6	5.3
Number	260	254	236	196	177	151	122	101	82	69	58	50	35	24	16	13

Table W4K-33 Age Group = 51-60 Gender = OVERALL Occupational group = MANUAL Total cases = 364

Threshold dB HL≥	0	5	10	15	20	25	30	35	40	45	50	55	65	75	85	95
Prevalence	**100.0**	**99.3**	**95.7**	**89.7**	**78.9**	**67.7**	**57.7**	**47.0**	**37.4**	**32.8**	**29.1**	**25.9**	**14.5**	**10.1**	**6.2**	**3.4**
Lower CI	99.0	97.4	92.1	84.8	73.3	61.7	51.6	41.5	32.6	28.4	25.0	21.9	11.6	7.6	4.4	2.3
Upper CI	100.0	99.8	97.7	93.1	83.6	73.2	63.6	52.5	42.4	37.5	33.5	30.2	17.9	13.3	8.7	5.2
Number	364	362	354	340	311	281	253	223	195	176	161	141	91	62	44	25

Table W4K-34 Age Group = 61-70 Gender = OVERALL Occupational group = MANUAL Total cases = 298

Threshold dB HL≥	0	5	10	15	20	25	30	35	40	45	50	55	65	75	85	95
Prevalence	**100.0**	**99.7**	**98.2**	**95.6**	**90.4**	**81.0**	**75.2**	**65.7**	**58.6**	**50.7**	**45.6**	**41.1**	**25.0**	**17.0**	**8.8**	**4.3**
Lower CI	98.7	98.6	95.1	91.2	84.6	74.6	68.7	58.8	51.5	44.1	38.9	34.7	20.2	12.7	6.1	3.2
Upper CI	100.0	100.0	99.3	97.9	94.2	86.0	80.7	71.9	65.4	57.4	52.5	47.9	30.4	22.5	12.5	5.7
Number	298	297	293	287	278	261	246	231	219	199	183	168	121	83	47	27

Table W4K-35 Age Group = 71-80 Gender = OVERALL Occupational group = MANUAL Total cases = 148

Threshold dB HL≥	0	5	10	15	20	25	30	35	40	45	50	55	65	75	85	95
Prevalence	**100.0**	**100.0**	**97.4**	**97.4**	**92.9**	**92.9**	**92.9**	**85.0**	**79.2**	**77.2**	**71.3**	**65.9**	**40.9**	**29.5**	**17.3**	**6.5**
Lower CI	97.5	97.5	86.6	86.6	83.7	83.7	83.7	74.6	67.3	65.4	60.1	56.2	31.3	21.1	11.5	4.4
Upper CI	100.0	100.0	99.6	99.6	97.1	97.1	97.1	91.6	87.6	85.8	80.4	74.5	51.2	39.6	25.3	9.4
Number	148	148	147	147	145	145	145	140	136	132	128	121	93	70	44	23

Prevalence by age group, gender and occupational group for the worse ear over 4 kHz

Table W4K-36 Age Group = 18-80 Gender = FEMALE Occupational group = NON-MANUAL Total cases = 671

Threshold dB HL≥	0	5	10	15	20	25	30	35	40	45	50	55	65	75	85	95
**Prevalence**	**94.7**	**86.1**	**72.0**	**57.0**	**44.1**	**35.0**	**28.7**	**23.9**	**20.6**	**16.3**	**13.1**	**9.5**	**5.3**	**3.3**	**1.9**	**1.3**
**Lower CI**	92.3	82.8	68.4	53.5	40.8	32.1	26.0	21.6	18.4	14.0	11.3	7.9	4.2	2.5	1.4	0.9
**Upper CI**	96.4	88.8	75.3	60.3	47.4	37.9	31.4	26.3	23.0	18.9	15.2	11.3	6.8	4.5	2.6	2.0
**Number**	648	610	533	443	365	298	249	218	189	161	135	115	77	55	36	26

Table W4K-37 Age Group = 18-80 Gender = MALE Occupational group = NON-MANUAL Total cases = 514

Threshold dB HL≥	0	5	10	15	20	25	30	35	40	45	50	55	65	75	85	95
**Prevalence**	**97.3**	**88.2**	**74.4**	**64.7**	**52.3**	**46.0**	**39.1**	**34.5**	**29.6**	**26.6**	**23.3**	**18.4**	**13.5**	**6.5**	**3.9**	**2.3**
**Lower CI**	94.2	83.5	69.5	60.0	47.9	41.7	34.6	30.3	25.6	22.9	19.8	15.4	10.8	5.0	3.1	1.6
**Upper CI**	98.7	91.7	78.7	69.1	56.7	50.5	43.7	38.9	34.0	30.7	27.2	21.9	16.7	8.3	5.1	3.4
**Number**	505	480	436	404	354	320	284	255	223	204	184	152	111	64	42	24

Table W4K-38 Age Group = 18-80 Gender = FEMALE Occupational group = MANUAL Total cases = 714

Threshold dB HL≥	0	5	10	15	20	25	30	35	40	45	50	55	65	75	85	95
**Prevalence**	**95.4**	**89.2**	**78.1**	**61.8**	**49.8**	**39.3**	**32.7**	**23.3**	**18.5**	**15.2**	**13.5**	**11.0**	**6.8**	**4.9**	**2.6**	**1.3**
**Lower CI**	92.1	85.2	73.7	57.5	45.9	35.8	29.5	20.5	15.9	12.9	11.3	9.1	5.4	3.7	2.0	0.9
**Upper CI**	97.3	92.3	82.0	66.0	53.7	42.9	36.1	26.3	21.4	17.9	16.0	13.3	8.5	6.4	3.4	1.8
**Number**	696	669	610	530	453	382	322	263	222	192	171	144	102	76	47	27

Table W4K-39 Age Group = 18-80 Gender = MALE Occupational group = MANUAL Total cases = 759

Threshold dB HL≥	0	5	10	15	20	25	30	35	40	45	50	55	65	75	85	95
**Prevalence**	**97.6**	**92.5**	**84.8**	**76.6**	**67.5**	**58.9**	**52.8**	**48.3**	**41.8**	**37.5**	**31.5**	**28.9**	**17.1**	**11.9**	**7.7**	**3.8**
**Lower CI**	94.4	88.2	80.3	71.9	62.8	54.7	48.8	44.5	38.2	34.3	28.7	26.2	14.9	9.9	5.9	2.8
**Upper CI**	99.0	95.3	88.4	80.7	71.9	63.1	56.8	52.2	45.5	40.8	34.4	31.8	19.6	14.3	9.9	5.0
**Number**	752	740	711	662	621	576	540	507	468	430	391	360	254	175	114	66

Table W4K-40 Age Group = 18-30 Gender = FEMALE Occupational group = NON-MANUAL Total cases = 115

Threshold dB HL≥	0	5	10	15	20	25	30	35	40	45	50	55	65	75	85	95
**Prevalence**	**84.8**	**66.5**	**39.5**	**19.7**	**13.2**	**8.1**	**6.5**	**2.2**	**2.2**	**0.4**	**0.4**	**0.4**	**0.4**	**0.4**	**0.0**	**0.0**
**Lower CI**	76.5	57.0	31.0	13.5	8.5	4.4	3.4	1.0	1.0	0.1	0.1	0.1	0.1	0.1	0.0	0.0
**Upper CI**	90.6	74.9	48.8	27.8	20.1	14.3	12.1	5.1	5.1	2.2	2.2	2.2	2.2	2.2	3.2	3.2
**Number**	99	81	50	27	20	12	10	5	5	1	1	1	1	1	0	0

Table W4K-41 Age Group = 31-40 Gender = FEMALE Occupational group = NON-MANUAL Total cases = 86

Threshold dB HL≥	0	5	10	15	20	25	30	35	40	45	50	55	65	75	85	95
**Prevalence**	**93.5**	**79.0**	**60.2**	**40.4**	**23.4**	**11.8**	**7.7**	**6.2**	**5.6**	**4.8**	**4.8**	**4.0**	**1.8**	**0.6**	**0.6**	**0.6**
**Lower CI**	87.6	68.3	48.9	30.1	15.3	6.7	3.9	3.1	2.6	1.9	1.9	1.4	0.5	0.1	0.1	0.1
**Upper CI**	96.7	86.8	70.6	51.7	34.0	19.9	14.8	12.3	11.6	11.2	11.2	10.7	6.9	3.2	3.2	3.2
**Number**	81	71	55	37	23	14	9	8	7	6	6	4	2	1	1	1

Table W4K-42 Age Group = 41-50 Gender = FEMALE Occupational group = NON-MANUAL Total cases = 112

Threshold dB HL≥	0	5	10	15	20	25	30	35	40	45	50	55	65	75	85	95
**Prevalence**	**98.9**	**92.4**	**79.1**	**62.7**	**36.8**	**22.1**	**16.5**	**11.6**	**6.6**	**3.3**	**2.8**	**1.9**	**0.4**	**0.0**	**0.0**	**0.0**
**Lower CI**	93.9	85.4	70.0	52.9	29.1	16.0	11.3	7.6	3.9	1.6	1.2	0.6	0.1	0.0	0.0	0.0
**Upper CI**	99.8	96.2	86.0	71.6	45.3	29.5	23.5	17.4	11.0	6.6	6.3	5.9	2.2	3.3	3.3	3.3
**Number**	111	104	91	73	47	30	24	18	12	8	6	4	1	0	0	0

Prevalence by age group, gender and occupational group for the worse ear over 4 kHz

Table W4K-43 Age Group = 51-60 Gender = FEMALE Occupational group = NON-MANUAL Total cases = 175

Threshold dB HL≥

	0	5	10	15	20	25	30	35	40	45	50	55	65	75	85	95
Prevalence	**99.5**	**98.8**	**91.0**	**73.8**	**60.8**	**44.3**	**31.3**	**24.7**	**20.7**	**16.3**	**10.7**	**7.8**	**5.5**	**4.0**	**2.7**	**2.5**
Lower CI	97.5	96.4	85.2	65.9	52.6	36.6	24.8	19.1	15.7	12.3	7.4	5.3	3.5	2.4	1.3	1.1
Upper CI	99.9	99.6	94.6	80.4	68.5	52.4	38.7	31.2	26.7	21.3	15.2	11.4	8.6	6.8	5.7	5.5
Number	174	172	158	133	115	89	70	60	52	45	33	28	20	15	10	9

Table W4K-44 Age Group = 61-70 Gender = FEMALE Occupational group = NON-MANUAL Total cases = 116

Threshold dB HL≥

	0	5	10	15	20	25	30	35	40	45	50	55	65	75	85	95
Prevalence	**100.0**	**98.6**	**95.9**	**90.8**	**79.3**	**74.7**	**62.9**	**53.9**	**43.5**	**34.4**	**28.5**	**23.1**	**11.4**	**5.4**	**4.5**	**2.3**
Lower CI	96.8	92.4	88.9	83.7	69.5	64.8	52.9	44.7	34.0	26.0	20.9	16.7	7.1	3.7	2.9	1.3
Upper CI	100.0	99.8	98.5	95.1	86.5	82.6	71.8	62.9	53.5	43.9	37.6	31.1	17.9	7.9	6.9	4.3
Number	116	115	112	107	97	91	77	69	57	48	41	36	25	17	14	9

Table W4K-45 Age Group = 71-80 Gender = FEMALE Occupational group = NON-MANUAL Total cases = 67

Threshold dB HL≥

	0	5	10	15	20	25	30	35	40	45	50	55	65	75	85	95
Prevalence	**100.0**	**100.0**	**100.0**	**98.6**	**91.8**	**91.5**	**88.0**	**86.2**	**80.8**	**70.6**	**58.7**	**38.2**	**22.9**	**16.5**	**6.4**	**4.3**
Lower CI	94.6	94.6	94.6	92.2	77.7	77.6	74.5	72.3	66.5	52.0	46.9	28.7	14.9	9.2	3.6	2.0
Upper CI	100.0	100.0	100.0	99.7	97.3	97.1	94.9	93.7	90.0	84.2	69.5	48.7	33.3	27.8	11.2	9.2
Number	67	67	67	66	63	62	59	58	56	53	48	42	28	21	11	7

Table W4K-46 Age Group = 18-30 Gender = MALE Occupational group = NON-MANUAL Total cases = 66

Threshold dB HL≥

	0	5	10	15	20	25	30	35	40	45	50	55	65	75	85	95
Prevalence	**93.6**	**68.7**	**43.5**	**23.4**	**12.2**	**10.7**	**8.7**	**8.7**	**8.4**	**5.0**	**5.0**	**5.0**	**5.0**	**0.1**	**0.1**	**0.0**
Lower CI	82.7	54.4	32.4	14.1	5.5	4.5	3.1	3.1	3.0	1.3	1.3	1.3	1.3	0.0	0.0	0.0
Upper CI	97.8	80.2	55.3	36.2	24.7	23.5	21.9	21.9	21.7	17.8	17.8	17.8	17.8	5.7	5.7	5.5
Number	62	48	30	18	10	8	5	5	4	3	3	3	3	1	1	0

Table W4K-47 Age Group = 31-40 Gender = MALE Occupational group = NON-MANUAL Total cases = 62

Threshold dB HL≥

	0	5	10	15	20	25	30	35	40	45	50	55	65	75	85	95
Prevalence	**97.9**	**89.7**	**68.7**	**56.9**	**33.2**	**27.0**	**20.3**	**16.9**	**11.6**	**11.0**	**11.0**	**5.2**	**1.2**	**0.0**	**0.0**	**0.0**
Lower CI	89.1	78.3	51.5	41.3	20.4	15.1	10.0	7.5	4.5	4.1	4.1	2.1	0.6	0.0	0.0	0.0
Upper CI	99.6	95.5	82.0	71.3	49.1	43.4	36.7	33.6	26.8	26.4	26.4	12.3	9.9	5.8	5.8	5.8
Number	61	55	47	41	27	20	16	12	10	8	8	5	2	0	0	0

Table W4K-48 Age Group = 41-50 Gender = MALE Occupational group = NON-MANUAL Total cases = 76

Threshold dB HL≥

	0	5	10	15	20	25	30	35	40	45	50	55	65	75	85	95
Prevalence	**96.6**	**93.9**	**77.8**	**70.4**	**54.4**	**44.3**	**34.9**	**30.1**	**23.6**	**21.3**	**14.5**	**11.6**	**9.1**	**3.0**	**3.0**	**2.5**
Lower CI	90.6	87.3	66.4	59.0	42.3	33.5	23.7	19.6	14.3	12.4	8.6	6.5	4.9	1.7	1.7	1.4
Upper CI	98.8	97.1	86.1	79.7	65.9	55.5	48.2	43.2	36.4	34.1	23.4	19.9	16.3	5.3	5.3	4.4
Number	73	70	59	53	43	38	31	26	21	18	15	12	9	5	5	4

Table W4K-49 Age Group = 51-60 Gender = MALE Occupational group = NON-MANUAL Total cases = 142

Threshold dB HL≥

	0	5	10	15	20	25	30	35	40	45	50	55	65	75	85	95
Prevalence	**99.3**	**98.2**	**94.9**	**90.3**	**78.6**	**64.5**	**54.1**	**44.1**	**38.8**	**36.4**	**33.4**	**19.9**	**12.4**	**8.7**	**3.3**	**1.4**
Lower CI	96.3	94.3	90.4	84.1	70.0	55.4	45.7	36.8	31.7	29.7	26.8	13.1	6.8	3.9	1.8	0.6
Upper CI	99.9	99.5	97.3	94.2	85.3	72.7	62.2	51.7	46.4	43.7	40.6	29.2	21.4	18.3	6.1	3.4
Number	141	139	133	126	113	97	86	73	63	59	54	39	26	16	10	5

Prevalence by age group, gender and occupational group for the worse ear over 4 kHz

Table W4K-50 Age Group = 61-70 Gender = MALE Occupational group = NON-MANUAL Total cases = 112

Threshold dB HL≥	0	5	10	15	20	25	30	35	40	45	50	55	65	75	85	95
**Prevalence**	**100.0**	**100.0**	**99.6**	**99.0**	**96.3**	**93.3**	**79.3**	**68.7**	**60.1**	**56.1**	**45.9**	**42.3**	**33.0**	**19.4**	**14.5**	**8.4**
**Lower CI**	96.7	96.7	97.8	96.2	90.5	86.8	64.2	60.4	50.9	47.1	37.7	34.5	26.9	14.7	10.5	5.0
**Upper CI**	100.0	100.0	99.9	99.7	98.6	96.7	89.1	76.0	68.6	64.8	54.3	50.5	39.7	25.0	19.6	13.9
**Number**	112	112	111	110	107	104	94	88	79	73	63	56	45	29	19	11

Table W4K-51 Age Group = 71-80 Gender = MALE Occupational group = NON-MANUAL Total cases = 56

Threshold dB HL≥	0	5	10	15	20	25	30	35	40	45	50	55	65	75	85	95
**Prevalence**	**100.0**	**100.0**	**100.0**	**100.0**	**95.8**	**93.8**	**91.5**	**89.4**	**78.5**	**69.6**	**67.6**	**61.5**	**45.4**	**21.7**	**11.0**	**5.3**
**Lower CI**	93.6	93.6	93.6	93.6	87.2	85.9	81.8	80.8	64.9	55.4	53.2	47.9	33.2	13.7	5.9	1.6
**Upper CI**	100.0	100.0	100.0	100.0	98.7	97.4	96.3	94.5	87.9	80.9	79.3	73.6	58.2	32.6	19.5	15.8
**Number**	56	56	56	56	54	53	52	51	46	43	41	37	26	13	7	4

Table W4K-52 Age Group = 18-30 Gender = FEMALE Occupational group = MANUAL Total cases = 109

Threshold dB HL≥	0	5	10	15	20	25	30	35	40	45	50	55	65	75	85	95
**Prevalence**	**88.2**	**74.6**	**57.3**	**32.0**	**20.3**	**9.0**	**7.9**	**4.1**	**3.6**	**2.9**	**1.8**	**1.3**	**0.2**	**0.0**	**0.0**	**0.0**
**Lower CI**	77.4	61.8	45.1	21.9	12.4	4.2	3.4	1.8	1.5	1.1	0.5	0.3	0.0	0.0	0.0	0.0
**Upper CI**	94.3	84.2	68.7	44.2	31.4	18.0	17.2	8.8	8.4	7.8	5.9	5.9	1.1	3.4	3.4	3.4
**Number**	98	86	69	45	29	17	13	9	7	5	4	2	1	0	0	0

Table W4K-53 Age Group = 31-40 Gender = FEMALE Occupational group = MANUAL Total cases = 103

Threshold dB HL≥	0	5	10	15	20	25	30	35	40	45	50	55	65	75	85	95
**Prevalence**	**93.2**	**84.8**	**67.6**	**43.3**	**26.6**	**21.3**	**11.4**	**7.6**	**5.8**	**5.6**	**4.8**	**2.6**	**2.4**	**1.7**	**1.1**	**0.6**
**Lower CI**	82.6	73.0	55.0	32.2	18.8	14.5	7.2	4.4	2.9	2.8	2.2	1.2	1.1	0.6	0.3	0.1
**Upper CI**	97.6	92.0	78.1	55.1	36.0	30.1	17.7	12.9	11.3	11.1	10.4	5.4	5.1	4.3	3.4	3.3
**Number**	98	92	77	61	45	36	24	18	13	12	9	7	6	4	3	1

Table W4K-54 Age Group = 41-50 Gender = FEMALE Occupational group = MANUAL Total cases = 135

Threshold dB HL≥	0	5	10	15	20	25	30	35	40	45	50	55	65	75	85	95
**Prevalence**	**97.8**	**92.0**	**79.2**	**56.0**	**46.8**	**36.5**	**25.2**	**15.1**	**10.4**	**7.2**	**5.5**	**4.7**	**3.1**	**1.9**	**1.5**	**1.3**
**Lower CI**	92.3	84.9	70.2	46.1	37.3	27.7	17.8	9.9	6.5	4.7	3.6	2.9	1.5	1.0	0.8	0.6
**Upper CI**	99.4	95.9	86.0	65.5	56.5	46.2	34.4	22.3	16.3	11.1	8.2	7.5	6.0	3.8	3.1	2.8
**Number**	133	127	111	85	72	58	41	31	23	20	17	15	10	8	7	6

Table W4K-55 Age Group = 51-60 Gender = FEMALE Occupational group = MANUAL Total cases = 165

Threshold dB HL≥	0	5	10	15	20	25	30	35	40	45	50	55	65	75	85	95
**Prevalence**	**100.0**	**98.6**	**94.0**	**84.7**	**69.9**	**54.4**	**44.1**	**27.4**	**17.0**	**12.1**	**9.2**	**6.4**	**4.1**	**3.9**	**3.1**	**1.0**
**Lower CI**	97.7	95.1	88.2	76.5	61.0	45.2	35.4	20.5	12.0	8.3	6.1	3.9	2.4	2.2	1.7	0.5
**Upper CI**	100.0	99.6	97.1	90.4	77.5	63.3	53.2	35.5	23.5	17.2	13.4	10.2	7.0	6.8	5.5	2.3
**Number**	165	163	157	147	126	104	88	65	48	39	33	24	17	15	13	6

Table W4K-56 Age Group = 61-70 Gender = FEMALE Occupational group = MANUAL Total cases = 126

Threshold dB HL≥	0	5	10	15	20	25	30	35	40	45	50	55	65	75	85	95
**Prevalence**	**100.0**	**99.5**	**96.5**	**93.1**	**83.6**	**67.6**	**59.7**	**44.8**	**37.7**	**28.6**	**25.6**	**22.2**	**14.5**	**11.5**	**3.5**	**2.5**
**Lower CI**	97.0	97.4	90.8	85.3	73.5	57.0	49.5	34.4	28.2	20.3	17.6	14.9	9.0	6.6	1.9	1.8
**Upper CI**	100.0	99.9	98.8	96.9	90.4	76.7	69.1	55.6	48.3	38.6	35.6	31.7	22.5	19.4	6.5	3.4
**Number**	126	125	121	117	108	94	83	71	65	53	47	41	29	21	6	5

Prevalence by age group, gender and occupational group for the worse ear over 4 kHz

Table W4K-57 Age Group = 71-80 Gender = FEMALE Occupational group = MANUAL Total cases = 76

Threshold dB HL≥	0	5	10	15	20	25	30	35	40	45	50	55	65	75	85	95
**Prevalence**	**100.0**	**100.0**	**95.7**	**95.7**	**88.0**	**88.0**	**88.0**	**74.9**	**65.8**	**63.3**	**62.5**	**54.2**	**30.8**	**20.7**	**10.1**	**3.9**
Lower CI	95.2	95.2	78.7	78.7	73.3	73.3	73.3	58.6	47.7	45.4	44.7	39.1	19.5	11.7	6.4	2.1
Upper CI	100.0	100.0	99.3	99.3	95.1	95.1	95.1	86.3	80.3	78.1	77.4	68.6	44.9	33.7	15.5	7.1
Number	76	76	75	75	73	73	73	69	66	63	61	55	39	28	18	9

Table W4K-58 Age Group = 18-30 Gender = MALE Occupational group = MANUAL Total cases = 109

Threshold dB HL≥	0	5	10	15	20	25	30	35	40	45	50	55	65	75	85	95
**Prevalence**	**92.9**	**78.8**	**59.4**	**46.7**	**32.3**	**23.4**	**18.0**	**12.9**	**7.6**	**3.7**	**3.3**	**2.6**	**1.3**	**1.3**	**1.0**	**0.1**
Lower CI	81.6	65.6	47.1	34.7	21.7	14.4	10.2	6.8	3.1	1.7	1.4	0.9	0.3	0.3	0.2	0.0
Upper CI	97.5	87.9	70.7	59.1	45.1	35.7	29.8	23.2	17.5	8.0	7.7	7.2	5.0	5.0	5.1	0.4
Number	104	95	76	60	41	30	25	20	14	10	8	5	3	3	2	1

Table W4K-59 Age Group = 31-40 Gender = MALE Occupational group = MANUAL Total cases = 80

Threshold dB HL≥	0	5	10	15	20	25	30	35	40	45	50	55	65	75	85	95
**Prevalence**	**97.4**	**90.8**	**82.3**	**70.0**	**54.2**	**39.1**	**34.8**	**29.6**	**24.1**	**21.4**	**10.2**	**9.4**	**3.8**	**3.4**	**3.4**	**2.3**
Lower CI	89.8	76.9	68.1	56.2	40.1	27.6	23.8	18.9	14.2	11.8	4.5	4.0	1.3	1.1	1.1	0.5
Upper CI	99.4	96.7	91.0	81.0	67.6	51.9	47.6	43.2	38.0	35.4	21.2	20.6	10.5	10.3	10.3	9.8
Number	78	75	69	56	48	37	34	28	24	19	11	10	6	5	5	3

Table W4K-60 Age Group = 41-50 Gender = MALE Occupational group = MANUAL Total cases = 127

Threshold dB HL≥	0	5	10	15	20	25	30	35	40	45	50	55	65	75	85	95
**Prevalence**	**100.0**	**100.0**	**97.1**	**85.3**	**78.1**	**65.2**	**53.0**	**46.9**	**37.5**	**31.2**	**24.6**	**19.8**	**14.2**	**10.5**	**6.4**	**4.1**
Lower CI	97.1	97.1	88.8	76.1	67.5	54.0	42.7	36.8	28.4	22.8	17.3	13.4	8.7	5.6	2.7	1.7
Upper CI	100.0	100.0	99.3	91.4	85.9	75.0	63.1	57.2	47.5	41.1	33.6	28.3	22.5	18.8	14.4	9.7
Number	127	127	125	111	105	93	81	70	59	49	41	35	25	16	9	7

Table W4K-61 Age Group = 51-60 Gender = MALE Occupational group = MANUAL Total cases = 199

Threshold dB HL≥	0	5	10	15	20	25	30	35	40	45	50	55	65	75	85	95
**Prevalence**	**100.0**	**100.0**	**97.7**	**95.3**	**89.2**	**82.5**	**72.9**	**68.7**	**60.0**	**55.7**	**51.2**	**47.5**	**25.9**	**16.9**	**9.7**	**6.0**
Lower CI	98.1	98.1	92.3	89.6	82.1	74.7	64.4	60.0	51.7	47.4	43.2	39.6	20.2	12.2	6.5	3.8
Upper CI	100.0	100.0	99.3	98.0	93.7	88.3	80.1	76.3	67.7	63.6	59.1	55.4	32.6	22.9	14.4	9.5
Number	199	199	197	193	185	177	165	158	147	137	128	117	74	47	31	19

Table W4K-62 Age Group = 61-70 Gender = MALE Occupational group = MANUAL Total cases = 172

Threshold dB HL≥	0	5	10	15	20	25	30	35	40	45	50	55	65	75	85	95
**Prevalence**	**100.0**	**100.0**	**100.0**	**98.5**	**98.5**	**96.7**	**93.2**	**90.4**	**83.4**	**76.9**	**69.4**	**63.7**	**37.6**	**23.5**	**15.2**	**6.4**
Lower CI	97.8	97.8	97.8	93.5	93.5	91.4	84.7	82.2	72.4	66.3	58.5	53.0	30.5	17.0	9.9	4.3
Upper CI	100.0	100.0	100.0	99.7	99.7	98.7	97.1	95.1	90.6	85.0	78.5	73.3	45.3	31.5	22.6	9.5
Number	172	172	172	170	170	167	163	160	154	146	136	127	92	62	41	22

Table W4K-63 Age Group = 71-80 Gender = MALE Occupational group = MANUAL Total cases = 72

Threshold dB HL≥	0	5	10	15	20	25	30	35	40	45	50	55	65	75	85	95
**Prevalence**	**100.0**	**100.0**	**100.0**	**100.0**	**100.0**	**100.0**	**100.0**	**99.6**	**98.8**	**97.5**	**84.8**	**83.5**	**56.1**	**42.6**	**28.3**	**10.2**
Lower CI	94.9	94.9	94.9	94.9	94.9	94.9	94.9	97.8	95.3	95.0	82.7	79.9	40.1	27.7	15.4	6.2
Upper CI	100.0	100.0	100.0	100.0	100.0	100.0	100.0	99.9	99.7	98.8	86.7	86.5	71.0	59.0	46.0	16.2
Number	72	72	72	72	72	72	72	71	70	69	67	66	54	42	26	14

Prevalence by age group, gender and occupational group for the worse ear over 6 kHz

Table W6K-1 Age Group = 18-80 Gender = OVERALL Occupational group = OVERALL Total cases = 2622

	Threshold dB HL≥															
	0	5	10	15	20	25	30	35	40	45	50	55	65	75	85	95
**Prevalence**	**99.0**	**96.4**	**90.3**	**80.6**	**69.5**	**59.1**	**50.9**	**42.5**	**37.7**	**32.7**	**28.1**	**24.0**	**17.3**	**11.3**	**6.8**	**4.1**
**Lower CI**	98.3	95.2	88.6	78.6	67.4	57.1	49.0	40.7	35.9	31.0	26.6	22.6	16.1	10.2	6.0	3.5
**Upper CI**	99.4	97.3	91.7	82.5	71.5	61.2	52.8	44.3	39.5	34.4	29.7	25.5	18.7	12.4	7.7	4.8
**Number**	2604	2571	2460	2300	2094	1876	1685	1482	1346	1206	1069	951	731	525	352	213

Table W6K-2 Age Group = 18-80 Gender = FEMALE Occupational group = OVERALL Total cases = 1368

	Threshold dB HL≥															
	0	5	10	15	20	25	30	35	40	45	50	55	65	75	85	95
**Prevalence**	**99.3**	**96.5**	**89.3**	**79.6**	**66.7**	**55.5**	**46.5**	**37.7**	**32.9**	**28.1**	**23.6**	**19.3**	**13.0**	**7.3**	**4.3**	**2.4**
**Lower CI**	98.5	95.0	87.1	76.9	63.9	52.8	44.0	35.4	30.7	26.0	21.7	17.6	11.7	6.2	3.5	1.8
**Upper CI**	99.7	97.6	91.2	82.0	69.4	58.1	49.0	40.1	35.3	30.2	25.6	21.1	14.5	8.6	5.2	3.1
**Number**	1360	1338	1260	1168	1030	905	783	659	583	511	439	373	267	183	123	70

Table W6K-3 Age Group = 18-80 Gender = MALE Occupational group = OVERALL Total cases = 1254

	Threshold dB HL≥															
	0	5	10	15	20	25	30	35	40	45	50	55	65	75	85	95
**Prevalence**	**98.6**	**96.2**	**91.3**	**81.8**	**72.6**	**63.3**	**56.0**	**47.9**	**43.1**	**37.9**	**33.2**	**29.3**	**22.2**	**15.6**	**9.6**	**6.0**
**Lower CI**	97.3	94.0	88.5	78.7	69.4	60.1	53.0	45.2	40.4	35.3	30.8	27.0	20.1	13.8	8.2	4.9
**Upper CI**	99.3	97.6	93.5	84.6	75.6	66.3	58.9	50.7	45.8	40.5	35.7	31.7	24.5	17.6	11.1	7.3
**Number**	1244	1233	1200	1132	1064	971	902	823	763	695	630	578	464	342	229	143

Table W6K-4 Age Group = 18-30 Gender = OVERALL Occupational group = OVERALL Total cases = 399

	Threshold dB HL≥															
	0	5	10	15	20	25	30	35	40	45	50	55	65	75	85	95
**Prevalence**	**96.4**	**89.5**	**76.9**	**56.4**	**38.3**	**24.6**	**17.0**	**11.5**	**8.8**	**5.7**	**4.9**	**3.7**	**2.4**	**0.9**	**0.7**	**0.7**
**Lower CI**	94.1	85.2	71.6	50.8	33.1	20.1	13.3	8.5	6.2	3.7	3.0	2.1	1.1	0.3	0.2	0.2
**Upper CI**	97.9	92.7	81.5	61.8	43.8	29.7	21.4	15.3	12.4	8.9	7.9	6.4	4.9	2.5	2.4	2.4
**Number**	382	364	316	240	174	119	85	60	46	32	26	19	11	6	3	3

Table W6K-5 Age Group = 31-40 Gender = OVERALL Occupational group = OVERALL Total cases = 329

	Threshold dB HL≥															
	0	5	10	15	20	25	30	35	40	45	50	55	65	75	85	95
**Prevalence**	**99.7**	**97.3**	**89.2**	**76.9**	**61.3**	**44.0**	**34.5**	**23.1**	**20.6**	**16.4**	**12.5**	**8.5**	**5.1**	**3.8**	**1.4**	**0.8**
**Lower CI**	98.5	94.8	84.5	70.9	54.7	37.6	28.5	18.1	15.9	12.1	8.8	5.6	3.0	1.9	0.6	0.2
**Upper CI**	100.0	98.6	92.5	82.0	67.6	50.6	41.0	29.0	26.4	21.9	17.5	12.5	8.6	7.4	3.4	3.0
**Number**	328	319	295	262	221	164	128	96	84	67	56	43	27	16	9	4

Table W6K-6 Age Group = 41-50 Gender = OVERALL Occupational group = OVERALL Total cases = 444

	Threshold dB HL≥															
	0	5	10	15	20	25	30	35	40	45	50	55	65	75	85	95
**Prevalence**	**100.0**	**98.7**	**94.7**	**87.5**	**74.5**	**62.0**	**48.5**	**36.7**	**30.0**	**23.7**	**18.3**	**13.6**	**8.9**	**6.7**	**3.0**	**1.9**
**Lower CI**	99.1	96.8	92.0	83.7	69.9	57.0	43.7	32.1	25.8	20.0	15.0	10.7	6.7	4.8	2.0	1.1
**Upper CI**	100.0	99.5	96.5	90.5	78.6	66.7	53.4	41.4	34.6	27.9	22.2	17.0	11.8	9.3	4.6	3.3
**Number**	444	440	419	392	339	289	236	189	163	135	106	82	60	44	25	17

Table W6K-7 Age Group = 51-60 Gender = OVERALL Occupational group = OVERALL Total cases = 664

	Threshold dB HL≥															
	0	5	10	15	20	25	30	35	40	45	50	55	65	75	85	95
**Prevalence**	**100.0**	**99.5**	**95.8**	**92.5**	**85.8**	**77.9**	**68.0**	**56.2**	**47.4**	**39.7**	**32.7**	**27.7**	**19.0**	**12.0**	**8.0**	**4.6**
**Lower CI**	99.4	98.4	93.5	89.7	82.5	74.2	63.9	52.1	43.4	35.9	29.2	24.4	16.4	9.9	6.3	3.3
**Upper CI**	100.0	99.9	97.2	94.5	88.6	81.3	71.9	60.2	51.5	43.6	36.4	31.1	22.0	14.5	10.2	6.3
**Number**	664	662	644	627	590	547	497	424	372	324	278	241	182	121	81	45

Prevalence by age group, gender and occupational group for the worse ear over 6 kHz

Table W6K-8 Age Group = 61-70 Gender = OVERALL Occupational group = OVERALL Total cases = 519

Threshold dB HL≥

	0	5	10	15	20	25	30	35	40	45	50	55	65	75	85	95
Prevalence	**100.0**	**100.0**	**100.0**	**98.3**	**96.5**	**94.0**	**89.2**	**82.7**	**76.0**	**70.4**	**59.7**	**53.5**	**38.0**	**24.5**	**14.2**	**8.3**
Lower CI	99.3	99.3	99.3	96.2	94.0	90.9	85.1	78.1	71.1	65.4	54.6	48.6	33.8	20.8	11.7	6.7
Upper CI	100.0	100.0	100.0	99.2	98.0	96.1	92.3	86.5	80.3	75.1	64.5	58.4	42.4	28.6	17.0	10.3
Number	519	519	519	513	506	495	481	457	429	404	360	333	257	189	129	83

Table W6K-9 Age Group = 71-80 Gender = OVERALL Occupational group = OVERALL Total cases = 267

Threshold dB HL≥

	0	5	10	15	20	25	30	35	40	45	50	55	65	75	85	95
Prevalence	**100.0**	**100.0**	**100.0**	**99.6**	**97.9**	**96.1**	**94.6**	**92.3**	**89.4**	**85.6**	**85.4**	**78.7**	**63.1**	**41.4**	**27.2**	**16.3**
Lower CI	98.6	98.6	98.6	98.0	93.9	90.8	89.4	85.6	82.3	78.6	78.4	71.1	55.4	34.7	21.8	11.9
Upper CI	100.0	100.0	100.0	99.9	99.3	98.4	97.3	96.0	93.9	90.6	90.4	84.8	70.1	48.4	33.3	22.1
Number	267	267	267	266	264	262	258	256	252	244	243	233	194	149	105	61

Table W6K-10 Age Group = 18-80 Gender = OVERALL Occupational group = NON-MANUAL Total cases = 1168

Threshold dB HL≥

	0	5	10	15	20	25	30	35	40	45	50	55	65	75	85	95
Prevalence	**98.7**	**95.3**	**87.8**	**77.7**	**66.0**	**55.3**	**47.0**	**38.7**	**34.5**	**29.8**	**25.2**	**22.8**	**16.2**	**10.1**	**5.3**	**3.1**
Lower CI	97.1	93.2	85.2	74.9	63.0	52.4	44.1	35.9	31.8	27.3	22.8	20.4	14.2	8.6	4.4	2.4
Upper CI	99.4	96.8	90.0	80.3	68.8	58.2	49.9	41.6	37.4	32.5	27.8	25.3	18.5	11.9	6.5	4.0
Number	1159	1137	1073	990	880	778	682	592	532	472	406	365	264	190	116	70

Table W6K-11 Age Group = 18-80 Gender = OVERALL Occupational group = MANUAL Total cases = 1450

Threshold dB HL≥

	0	5	10	15	20	25	30	35	40	45	50	55	65	75	85	95
Prevalence	**99.1**	**97.7**	**93.4**	**84.0**	**73.3**	**62.8**	**54.8**	**45.7**	**40.3**	**35.0**	**30.9**	**24.7**	**17.8**	**11.7**	**7.5**	**4.7**
Lower CI	98.4	96.3	91.3	81.1	70.1	59.6	51.8	43.1	37.8	32.7	28.7	22.9	16.3	10.4	6.5	3.8
Upper CI	99.6	98.6	95.0	86.6	76.3	65.8	57.7	48.3	42.9	37.4	33.2	26.6	19.5	13.1	8.8	5.8
Number	1441	1430	1383	1308	1212	1096	1001	888	812	732	661	585	466	334	236	143

Table W6K-12 Age Group = 18-30 Gender = FEMALE Occupational group = OVERALL Total cases = 223

Threshold dB HL≥

	0	5	10	15	20	25	30	35	40	45	50	55	65	75	85	95
Prevalence	**97.6**	**91.4**	**78.4**	**59.2**	**38.2**	**23.2**	**16.5**	**11.0**	**8.4**	**5.2**	**3.9**	**2.3**	**1.0**	**0.1**	**0.0**	**0.0**
Lower CI	94.7	86.4	71.9	52.0	31.5	17.8	11.9	7.3	5.2	2.9	2.0	1.1	0.3	0.0	0.0	0.0
Upper CI	98.9	94.7	83.8	65.9	45.4	29.6	22.3	16.2	13.3	9.2	7.4	4.7	2.9	0.6	1.7	1.7
Number	216	205	176	136	96	65	47	32	24	18	14	9	4	1	0	0

Table W6K-13 Age Group = 31-40 Gender = FEMALE Occupational group = OVERALL Total cases = 187

Threshold dB HL≥

	0	5	10	15	20	25	30	35	40	45	50	55	65	75	85	95
Prevalence	**99.5**	**96.1**	**85.3**	**72.6**	**56.9**	**38.5**	**27.4**	**19.1**	**16.1**	**11.8**	**10.4**	**7.2**	**2.8**	**1.4**	**0.2**	**0.0**
Lower CI	97.1	91.6	78.4	64.4	48.4	30.9	20.7	13.6	11.1	7.6	6.5	4.3	1.5	0.5	0.0	0.0
Upper CI	99.9	98.2	90.2	79.5	64.9	46.8	35.3	26.1	22.9	17.9	16.3	11.9	5.1	3.9	1.2	2.0
Number	186	181	163	146	120	86	61	47	39	30	27	22	12	5	1	0

Table W6K-14 Age Group = 41-50 Gender = FEMALE Occupational group = OVERALL Total cases = 245

Threshold dB HL≥

	0	5	10	15	20	25	30	35	40	45	50	55	65	75	85	95
Prevalence	**100.0**	**97.6**	**90.9**	**81.8**	**64.5**	**52.4**	**37.9**	**26.5**	**21.6**	**16.1**	**11.6**	**5.8**	**3.6**	**3.0**	**1.1**	**0.6**
Lower CI	98.5	93.9	86.0	75.8	57.7	45.6	31.8	21.3	17.0	12.2	8.4	3.9	2.1	1.6	0.5	0.2
Upper CI	100.0	99.1	94.2	86.6	70.8	59.1	44.5	32.4	27.0	21.0	15.9	8.5	6.0	5.3	2.6	1.6
Number	245	241	223	205	166	141	108	81	69	55	41	25	16	12	6	4

Prevalence by age group, gender and occupational group for the worse ear over 6 kHz

Table W6K-15 Age Group = 51-60 Gender = FEMALE Occupational group = OVERALL Total cases = 334

Threshold dB HL≥	0	5	10	15	20	25	30	35	40	45	50	55	65	75	85	95
Prevalence	100.0	99.1	93.7	89.6	80.9	71.4	59.8	44.2	36.0	29.8	22.1	16.6	8.8	4.6	3.6	2.2
Lower CI	98.9	96.9	89.9	85.2	75.6	65.7	53.9	38.5	30.6	24.9	17.7	12.9	6.5	3.3	2.5	1.3
Upper CI	100.0	99.8	96.1	92.9	85.2	76.6	65.5	50.0	41.7	35.3	27.1	21.2	12.0	6.4	5.3	3.7
Number	334	332	319	308	282	257	225	174	146	125	99	78	52	36	28	16

Table W6K-16 Age Group = 61-70 Gender = FEMALE Occupational group = OVERALL Total cases = 239

Threshold dB HL≥	0	5	10	15	20	25	30	35	40	45	50	55	65	75	85	95
Prevalence	100.0	100.0	100.0	97.2	95.0	90.5	83.7	76.3	66.9	59.4	47.1	40.3	28.0	16.7	9.0	5.2
Lower CI	98.4	98.4	98.4	93.5	90.7	85.1	77.1	69.4	59.5	52.0	40.3	33.9	22.8	12.2	6.3	3.4
Upper CI	100.0	100.0	100.0	98.8	97.4	94.1	88.6	82.0	73.5	66.4	54.0	47.1	33.7	22.6	12.6	7.9
Number	239	239	239	234	229	220	209	194	175	156	131	116	84	58	39	25

Table W6K-17 Age Group = 71-80 Gender = FEMALE Occupational group = OVERALL Total cases = 140

Threshold dB HL≥	0	5	10	15	20	25	30	35	40	45	50	55	65	75	85	95
Prevalence	100.0	100.0	100.0	99.4	96.4	94.4	92.3	88.6	86.6	83.4	83.4	78.2	60.5	33.2	21.0	11.4
Lower CI	97.3	97.3	97.3	96.8	89.7	85.9	84.0	78.0	75.7	72.8	72.8	67.4	50.5	25.2	15.3	6.9
Upper CI	100.0	100.0	100.0	99.9	98.8	97.9	96.5	94.4	93.1	90.4	90.4	86.1	69.6	42.3	28.1	18.4
Number	140	140	140	139	137	136	133	131	130	127	127	123	99	71	49	25

Table W6K-18 Age Group = 18-30 Gender = MALE Occupational group = OVERALL Total cases = 176

Threshold dB HL≥	0	5	10	15	20	25	30	35	40	45	50	55	65	75	85	95
Prevalence	95.1	87.4	75.3	53.6	38.2	25.7	17.3	11.8	9.2	6.1	5.7	5.0	3.7	1.6	1.4	1.4
Lower CI	90.7	79.7	66.5	44.9	30.3	18.9	11.9	7.6	5.5	3.2	2.9	2.3	1.5	0.5	0.4	0.4
Upper CI	97.4	92.5	82.3	62.0	46.7	33.9	24.4	17.9	14.9	11.5	11.2	10.3	8.7	4.8	4.7	4.7
Number	166	159	140	104	78	54	38	28	22	14	12	10	7	5	3	3

Table W6K-19 Age Group = 31-40 Gender = MALE Occupational group = OVERALL Total cases = 142

Threshold dB HL≥	0	5	10	15	20	25	30	35	40	45	50	55	65	75	85	95
Prevalence	100.0	98.6	93.5	81.7	66.2	50.0	42.5	27.8	25.8	21.7	14.9	9.9	7.7	6.4	2.8	1.8
Lower CI	97.4	96.2	86.1	72.2	55.7	39.7	32.5	19.5	17.9	14.2	9.0	5.4	3.8	2.8	1.1	0.5
Upper CI	100.0	99.5	97.1	88.5	75.4	60.4	53.1	37.9	35.8	31.5	23.6	17.5	14.9	13.8	7.0	6.3
Number	142	138	132	116	101	78	67	49	45	37	29	21	15	11	8	4

Table W6K-20 Age Group = 41-50 Gender = MALE Occupational group = OVERALL Total cases = 199

Threshold dB HL≥	0	5	10	15	20	25	30	35	40	45	50	55	65	75	85	95
Prevalence	100.0	100.0	98.8	93.7	85.4	72.5	60.3	47.9	39.3	32.0	25.7	22.2	14.8	10.9	5.1	3.3
Lower CI	98.1	98.1	96.1	88.8	78.9	65.0	52.5	40.4	32.2	25.7	19.8	16.7	10.6	7.3	3.1	1.8
Upper CI	100.0	100.0	99.7	96.6	90.2	78.9	67.5	55.5	46.9	39.1	32.6	28.9	20.4	16.0	8.3	6.2
Number	199	199	196	187	173	148	128	108	94	80	65	57	44	32	19	13

Table W6K-21 Age Group = 51-60 Gender = MALE Occupational group = OVERALL Total cases = 330

Threshold dB HL≥	0	5	10	15	20	25	30	35	40	45	50	55	65	75	85	95
Prevalence	100.0	100.0	98.1	95.6	91.4	85.2	77.2	69.7	60.2	50.8	44.7	40.1	30.4	20.2	12.9	7.2
Lower CI	98.8	98.8	95.4	92.1	87.1	80.1	71.5	63.7	54.2	45.0	39.2	34.8	25.6	16.1	9.6	4.8
Upper CI	100.0	100.0	99.2	97.6	94.3	89.1	82.1	75.1	66.0	56.6	50.4	45.6	35.7	25.1	17.2	10.6
Number	330	330	325	319	308	290	272	250	226	199	179	163	130	85	53	29

Prevalence by age group, gender and occupational group for the worse ear over 6 kHz

Table W6K-22 Age Group = 61-70 Gender = MALE Occupational group = OVERALL Total cases = 280

	Threshold dB HL≥															
	0	5	10	15	20	25	30	35	40	45	50	55	65	75	85	95
**Prevalence**	**100.0**	**100.0**	**100.0**	**99.4**	**98.2**	**97.9**	**95.6**	**90.2**	**86.7**	**83.4**	**74.3**	**69.1**	**49.6**	**33.6**	**20.2**	**12.0**
**Lower CI**	98.6	98.6	98.6	96.6	94.8	94.7	89.9	83.5	79.8	76.2	66.5	61.3	42.8	28.1	16.2	9.4
**Upper CI**	100.0	100.0	100.0	99.9	99.4	99.2	98.2	94.4	91.5	88.7	80.8	75.9	56.5	39.4	24.9	15.1
**Number**	280	280	280	279	277	275	272	263	254	248	229	217	173	131	90	58

Table W6K-23 Age Group = 71-80 Gender = MALE Occupational group = OVERALL Total cases = 127

	Threshold dB HL≥															
	0	5	10	15	20	25	30	35	40	45	50	55	65	75	85	95
**Prevalence**	**100.0**	**100.0**	**100.0**	**100.0**	**100.0**	**98.7**	**97.9**	**97.9**	**93.6**	**88.6**	**88.1**	**79.7**	**67.4**	**53.3**	**36.1**	**23.4**
**Lower CI**	97.1	97.1	97.1	97.1	97.1	92.7	92.2	92.2	85.4	80.0	79.6	68.7	55.9	42.4	26.7	15.6
**Upper CI**	100.0	100.0	100.0	100.0	100.0	99.8	99.4	99.4	97.4	93.7	93.4	87.5	77.1	63.8	46.8	33.6
**Number**	127	127	127	127	127	126	125	125	122	117	116	110	95	78	56	36

Table W6K-24 Age Group = 18-30 Gender = OVERALL Occupational group = NON-MANUAL Total cases = 180

	Threshold dB HL≥															
	0	5	10	15	20	25	30	35	40	45	50	55	65	75	85	95
**Prevalence**	**95.5**	**86.5**	**73.0**	**53.8**	**34.8**	**20.7**	**14.3**	**10.1**	**8.1**	**6.2**	**5.1**	**5.0**	**4.2**	**0.8**	**0.7**	**0.7**
**Lower CI**	89.9	79.0	64.8	46.1	27.9	14.9	9.4	5.8	4.3	2.9	2.1	2.0	1.5	0.2	0.1	0.1
**Upper CI**	98.1	91.6	79.9	61.3	42.4	28.1	21.3	16.9	14.8	12.8	11.8	11.7	11.2	3.9	4.0	4.0
**Number**	172	160	137	102	64	42	27	19	15	11	8	7	4	2	1	1

Table W6K-25 Age Group = 31-40 Gender = OVERALL Occupational group = NON-MANUAL Total cases = 147

	Threshold dB HL≥															
	0	5	10	15	20	25	30	35	40	45	50	55	65	75	85	95
**Prevalence**	**99.7**	**95.5**	**83.3**	**68.9**	**54.9**	**34.9**	**26.4**	**16.9**	**16.6**	**12.8**	**9.7**	**7.9**	**5.3**	**5.3**	**0.6**	**0.3**
**Lower CI**	98.5	91.4	76.7	61.4	46.3	26.2	18.6	10.6	10.4	7.3	5.1	3.7	2.0	2.0	0.3	0.0
**Upper CI**	100.0	97.7	88.3	75.6	63.2	44.7	36.0	25.9	25.6	21.6	17.7	16.0	13.2	13.2	4.6	3.1
**Number**	146	139	122	103	84	57	40	28	27	21	16	11	7	7	2	1

Table W6K-26 Age Group = 41-50 Gender = OVERALL Occupational group = NON-MANUAL Total cases = 187

	Threshold dB HL≥															
	0	5	10	15	20	25	30	35	40	45	50	55	65	75	85	95
**Prevalence**	**100.0**	**98.8**	**93.6**	**84.2**	**69.5**	**58.8**	**44.8**	**31.0**	**28.3**	**22.4**	**17.5**	**14.2**	**7.7**	**4.2**	**2.3**	**2.0**
**Lower CI**	98.0	95.8	89.3	78.0	61.9	51.2	37.2	24.5	22.0	16.7	12.2	9.6	4.7	2.2	1.1	0.8
**Upper CI**	100.0	99.7	96.2	89.0	76.2	65.9	52.6	38.3	35.5	29.4	24.4	20.4	12.2	7.9	4.8	4.6
**Number**	187	185	173	158	133	111	86	62	56	45	35	29	17	11	7	5

Table W6K-27 Age Group = 51-60 Gender = OVERALL Occupational group = NON-MANUAL Total cases = 307

	Threshold dB HL≥															
	0	5	10	15	20	25	30	35	40	45	50	55	65	75	85	95
**Prevalence**	**100.0**	**99.7**	**94.4**	**90.9**	**82.7**	**75.1**	**63.5**	**52.9**	**41.1**	**33.3**	**26.0**	**23.1**	**16.6**	**12.3**	**6.4**	**2.3**
**Lower CI**	98.8	98.1	90.5	86.5	77.6	69.6	57.5	47.2	35.7	28.5	21.8	19.2	13.6	10.0	3.6	1.4
**Upper CI**	100.0	99.9	96.8	94.0	86.8	79.8	69.1	58.5	46.7	38.5	30.7	27.6	20.1	14.9	11.0	3.9
**Number**	307	306	294	284	261	237	209	178	145	123	100	88	66	50	27	14

Table W6K-28 Age Group = 61-70 Gender = OVERALL Occupational group = NON-MANUAL Total cases = 226

	Threshold dB HL≥															
	0	5	10	15	20	25	30	35	40	45	50	55	65	75	85	95
**Prevalence**	**100.0**	**100.0**	**100.0**	**98.3**	**96.2**	**94.2**	**88.3**	**80.3**	**74.2**	**68.7**	**56.1**	**50.6**	**34.9**	**21.5**	**13.3**	**8.2**
**Lower CI**	98.3	98.3	98.3	94.7	91.7	89.2	80.0	71.8	65.5	60.0	47.8	42.6	29.5	17.4	10.0	5.8
**Upper CI**	100.0	100.0	100.0	99.5	98.3	96.9	93.5	86.7	81.3	76.2	64.0	58.6	40.6	26.3	17.3	11.4
**Number**	226	226	226	223	219	214	207	194	181	169	144	132	95	66	45	31

Prevalence by age group, gender and occupational group for the worse ear over 6 kHz

Table W6K-29 Age Group = 71-80 Gender = OVERALL Occupational group = NON-MANUAL Total cases = 121

	Threshold dB HL≥															
	**0**	**5**	**10**	**15**	**20**	**25**	**30**	**35**	**40**	**45**	**50**	**55**	**65**	**75**	**85**	**95**
**Prevalence**	**100.0**	**100.0**	**100.0**	**99.1**	**97.5**	**94.5**	**91.9**	**87.0**	**84.2**	**78.9**	**78.9**	**75.0**	**57.5**	**35.8**	**19.2**	**10.9**
**Lower CI**	96.9	96.9	96.9	95.1	90.9	85.5	83.1	73.9	71.3	67.1	67.1	63.0	45.8	26.2	14.2	6.9
**Upper CI**	100.0	100.0	100.0	99.8	99.4	98.1	96.3	94.0	92.0	87.2	87.2	84.1	68.5	46.7	25.5	16.8
**Number**	121	121	121	120	119	117	113	111	108	103	103	98	75	54	34	18

Table W6K-30 Age Group = 18-30 Gender = OVERALL Occupational group = MANUAL Total cases = 217

	Threshold dB HL≥															
	**0**	**5**	**10**	**15**	**20**	**25**	**30**	**35**	**40**	**45**	**50**	**55**	**65**	**75**	**85**	**95**
**Prevalence**	**97.0**	**93.1**	**82.7**	**60.6**	**43.2**	**27.4**	**20.1**	**12.5**	**9.2**	**5.8**	**5.5**	**2.8**	**1.6**	**0.8**	**0.5**	**0.5**
**Lower CI**	94.3	88.3	75.9	51.8	34.8	20.4	14.2	8.2	5.6	3.0	2.7	1.3	0.6	0.2	0.1	0.1
**Upper CI**	98.4	96.0	87.9	68.8	51.9	35.7	27.7	18.5	14.8	11.0	10.7	5.9	4.1	2.6	2.6	2.6
**Number**	208	202	177	138	110	77	58	41	31	21	18	12	7	4	2	2

Table W6K-31 Age Group = 31-40 Gender = OVERALL Occupational group = MANUAL Total cases = 182

	Threshold dB HL≥															
	**0**	**5**	**10**	**15**	**20**	**25**	**30**	**35**	**40**	**45**	**50**	**55**	**65**	**75**	**85**	**95**
**Prevalence**	**100.0**	**99.7**	**95.9**	**86.7**	**69.3**	**53.1**	**43.4**	**29.9**	**25.0**	**19.7**	**14.7**	**8.0**	**4.2**	**2.2**	**1.9**	**1.2**
**Lower CI**	97.9	98.8	90.5	78.7	59.4	43.4	34.3	22.3	18.1	13.2	9.5	5.2	2.5	0.9	0.7	0.3
**Upper CI**	100.0	99.9	98.3	92.0	77.7	62.5	53.0	38.8	33.4	28.3	22.1	12.1	7.0	5.4	5.1	4.8
**Number**	182	180	173	159	137	107	88	68	57	46	40	32	20	9	7	3

Table W6K-32 Age Group = 41-50 Gender = OVERALL Occupational group = MANUAL Total cases = 257

	Threshold dB HL≥															
	**0**	**5**	**10**	**15**	**20**	**25**	**30**	**35**	**40**	**45**	**50**	**55**	**65**	**75**	**85**	**95**
**Prevalence**	**100.0**	**98.7**	**96.0**	**90.2**	**77.9**	**66.1**	**52.7**	**42.2**	**33.5**	**26.1**	**20.6**	**14.0**	**9.9**	**8.2**	**3.6**	**2.2**
**Lower CI**	98.5	95.2	92.3	84.9	71.5	59.0	45.7	35.5	27.5	20.8	15.7	10.2	6.9	5.4	2.1	1.0
**Upper CI**	100.0	99.6	98.0	93.7	83.3	72.6	59.6	49.2	40.2	32.2	26.4	18.9	14.1	12.3	6.1	4.7
**Number**	257	255	246	234	206	178	150	127	107	90	71	53	43	33	18	12

Table W6K-33 Age Group = 51-60 Gender = OVERALL Occupational group = MANUAL Total cases = 357

	Threshold dB HL≥															
	**0**	**5**	**10**	**15**	**20**	**25**	**30**	**35**	**40**	**45**	**50**	**55**	**65**	**75**	**85**	**95**
**Prevalence**	**100.0**	**99.6**	**97.4**	**94.6**	**89.6**	**82.2**	**73.0**	**59.9**	**53.2**	**45.1**	**39.1**	**32.1**	**22.2**	**12.3**	**9.1**	**5.9**
**Lower CI**	98.9	98.0	94.7	91.2	85.1	76.6	67.0	53.7	47.1	39.3	33.5	26.9	18.0	9.4	6.7	3.9
**Upper CI**	100.0	99.9	98.8	96.8	92.8	86.7	78.3	65.8	59.2	51.1	45.1	37.8	27.1	16.0	12.2	8.8
**Number**	357	356	350	343	329	310	288	246	227	201	178	153	116	71	54	31

Table W6K-34 Age Group = 61-70 Gender = OVERALL Occupational group = MANUAL Total cases = 292

	Threshold dB HL≥															
	**0**	**5**	**10**	**15**	**20**	**25**	**30**	**35**	**40**	**45**	**50**	**55**	**65**	**75**	**85**	**95**
**Prevalence**	**100.0**	**100.0**	**100.0**	**97.6**	**96.6**	**93.6**	**88.7**	**83.3**	**75.5**	**69.6**	**61.3**	**54.6**	**39.2**	**26.6**	**14.8**	**8.4**
**Lower CI**	98.7	98.7	98.7	93.8	92.7	88.7	82.4	76.9	69.0	63.8	55.0	48.1	33.4	21.3	11.5	6.5
**Upper CI**	100.0	100.0	100.0	99.1	98.4	96.5	92.9	88.2	81.1	74.9	67.3	61.0	45.3	32.7	18.7	10.8
**Number**	292	292	292	289	286	280	273	262	247	234	215	201	162	123	84	52

Table W6K-35 Age Group = 71-80 Gender = OVERALL Occupational group = MANUAL Total cases = 145

	Threshold dB HL≥															
	**0**	**5**	**10**	**15**	**20**	**25**	**30**	**35**	**40**	**45**	**50**	**55**	**65**	**75**	**85**	**95**
**Prevalence**	**100.0**	**100.0**	**100.0**	**100.0**	**98.0**	**98.0**	**98.0**	**98.0**	**94.9**	**93.4**	**93.1**	**81.5**	**63.9**	**44.1**	**32.0**	**20.7**
**Lower CI**	97.4	97.4	97.4	97.4	89.5	89.5	89.5	89.5	82.2	81.6	81.4	70.6	54.8	35.6	23.3	13.0
**Upper CI**	100.0	100.0	100.0	100.0	99.7	99.7	99.7	99.7	98.7	97.8	97.6	89.0	72.1	53.0	42.1	31.4
**Number**	145	145	145	145	144	144	144	144	143	140	139	134	118	94	71	43

Prevalence by age group, gender and occupational group for the worse ear over 6 kHz

Table W6K-36 Age Group = 18-80 Gender = FEMALE Occupational group = NON-MANUAL Total cases = 662

	Threshold dB HL≥															
	**0**	**5**	**10**	**15**	**20**	**25**	**30**	**35**	**40**	**45**	**50**	**55**	**65**	**75**	**85**	**95**
**Prevalence**	**98.9**	**95.3**	**85.8**	**75.9**	**62.2**	**51.7**	**42.9**	**34.2**	**30.8**	**25.9**	**20.3**	**17.8**	**11.4**	**6.2**	**3.0**	**1.7**
**Lower CI**	97.7	93.0	82.5	72.3	58.4	48.1	39.6	30.9	27.7	23.0	17.8	15.4	9.5	4.5	2.3	1.2
**Upper CI**	99.5	96.8	88.6	79.2	65.9	55.1	46.3	37.6	34.2	28.9	23.1	20.4	13.8	8.3	4.0	2.5
**Number**	656	640	593	545	467	405	346	287	259	227	186	163	110	76	50	31

Table W6K-37 Age Group = 18-80 Gender = MALE Occupational group = NON-MANUAL Total cases = 506

	Threshold dB HL≥															
	**0**	**5**	**10**	**15**	**20**	**25**	**30**	**35**	**40**	**45**	**50**	**55**	**65**	**75**	**85**	**95**
**Prevalence**	**98.5**	**95.4**	**90.0**	**79.7**	**70.0**	**59.4**	**51.5**	**43.8**	**38.7**	**34.2**	**30.7**	**28.4**	**21.5**	**14.5**	**7.9**	**4.5**
**Lower CI**	94.7	91.1	85.5	75.2	65.3	54.6	46.6	39.2	34.2	29.8	26.5	24.2	17.9	12.0	6.1	3.3
**Upper CI**	99.6	97.7	93.2	83.6	74.3	64.1	56.4	48.5	43.4	38.9	35.3	32.9	25.7	17.4	10.1	6.2
**Number**	503	497	480	445	413	373	336	305	273	245	220	202	154	114	66	39

Table W6K-38 Age Group = 18-80 Gender = FEMALE Occupational group = MANUAL Total cases = 705

	Threshold dB HL≥															
	**0**	**5**	**10**	**15**	**20**	**25**	**30**	**35**	**40**	**45**	**50**	**55**	**65**	**75**	**85**	**95**
**Prevalence**	**99.8**	**98.5**	**94.3**	**84.0**	**71.3**	**59.1**	**49.9**	**40.6**	**34.1**	**29.4**	**26.4**	**20.1**	**13.1**	**7.2**	**4.6**	**2.7**
**Lower CI**	99.2	96.6	91.8	79.9	66.8	54.9	45.8	37.1	30.7	26.2	23.3	17.6	11.2	5.9	3.6	1.8
**Upper CI**	99.9	99.3	96.0	87.4	75.4	63.2	54.1	44.3	37.7	32.7	29.8	22.8	15.3	8.8	5.9	4.0
**Number**	703	697	666	622	562	499	436	371	323	283	252	210	157	107	73	39

Table W6K-39 Age Group = 18-80 Gender = MALE Occupational group = MANUAL Total cases = 745

	Threshold dB HL≥															
	**0**	**5**	**10**	**15**	**20**	**25**	**30**	**35**	**40**	**45**	**50**	**55**	**65**	**75**	**85**	**95**
**Prevalence**	**98.4**	**96.9**	**92.5**	**84.2**	**75.5**	**66.8**	**60.3**	**51.5**	**47.4**	**41.5**	**36.0**	**30.0**	**23.2**	**16.7**	**10.8**	**7.0**
**Lower CI**	96.7	94.4	88.8	79.6	70.8	62.1	56.0	47.7	43.8	38.1	32.9	27.4	20.9	14.5	8.9	5.4
**Upper CI**	99.2	98.3	95.1	87.8	79.6	71.1	64.3	55.2	51.0	45.0	39.1	32.8	25.7	19.2	13.1	9.0
**Number**	738	733	717	686	650	597	565	517	489	449	409	375	309	227	163	104

Table W6K-40 Age Group = 18-30 Gender = FEMALE Occupational group = NON-MANUAL Total cases = 114

	Threshold dB HL≥															
	**0**	**5**	**10**	**15**	**20**	**25**	**30**	**35**	**40**	**45**	**50**	**55**	**65**	**75**	**85**	**95**
**Prevalence**	**96.3**	**88.4**	**73.8**	**56.8**	**33.7**	**19.9**	**13.0**	**8.1**	**6.2**	**3.8**	**1.8**	**1.5**	**0.0**	**0.0**	**0.0**	**0.0**
**Lower CI**	92.0	81.2	64.9	47.7	25.4	13.6	8.0	4.2	3.0	1.7	0.6	0.4	0.0	0.0	0.0	0.0
**Upper CI**	98.3	93.1	81.1	65.4	43.0	28.3	20.4	14.9	12.4	8.5	5.1	5.0	3.3	3.3	3.3	3.3
**Number**	109	101	86	68	42	27	18	11	9	7	4	3	0	0	0	0

Table W6K-41 Age Group = 31-40 Gender = FEMALE Occupational group = NON-MANUAL Total cases = 85

	Threshold dB HL≥															
	**0**	**5**	**10**	**15**	**20**	**25**	**30**	**35**	**40**	**45**	**50**	**55**	**65**	**75**	**85**	**95**
**Prevalence**	**99.5**	**93.1**	**75.3**	**59.2**	**47.2**	**30.0**	**18.4**	**15.3**	**14.6**	**8.7**	**6.6**	**4.2**	**1.4**	**1.4**	**0.0**	**0.0**
**Lower CI**	97.1	85.3	64.6	47.8	36.2	20.9	11.1	8.6	8.0	4.0	2.7	1.3	0.3	0.3	0.0	0.0
**Upper CI**	99.9	96.9	83.7	69.7	58.6	41.1	29.0	25.6	25.0	17.7	15.0	12.5	6.5	6.5	4.3	4.3
**Number**	84	79	66	55	44	29	16	13	12	8	6	4	2	2	0	0

Table W6K-42 Age Group = 41-50 Gender = FEMALE Occupational group = NON-MANUAL Total cases = 112

	Threshold dB HL≥															
	**0**	**5**	**10**	**15**	**20**	**25**	**30**	**35**	**40**	**45**	**50**	**55**	**65**	**75**	**85**	**95**
**Prevalence**	**100.0**	**97.8**	**88.8**	**78.8**	**60.1**	**50.0**	**36.4**	**23.0**	**20.7**	**15.1**	**8.8**	**6.7**	**2.8**	**1.3**	**0.4**	**0.0**
**Lower CI**	96.7	92.2	81.3	69.8	50.0	40.5	27.8	16.5	14.7	10.1	5.2	3.7	1.3	0.3	0.1	0.0
**Upper CI**	100.0	99.4	93.6	85.6	69.3	59.5	46.0	31.1	28.4	22.1	14.3	11.7	5.8	5.2	2.2	3.3
**Number**	112	110	99	89	70	59	45	30	27	21	14	11	5	2	1	0

Prevalence by age group, gender and occupational group for the worse ear over 6 kHz

Table W6K-43 Age Group = 51-60 Gender = FEMALE Occupational group = NON-MANUAL Total cases = 170

	Threshold dB HL≥															
	0	5	10	15	20	25	30	35	40	45	50	55	65	75	85	95
Prevalence	100.0	99.4	92.6	88.7	76.7	66.1	56.7	41.6	32.9	26.6	18.4	14.9	7.7	5.7	3.8	2.2
Lower CI	97.8	96.5	86.3	81.7	68.8	57.8	48.4	34.4	26.3	20.8	13.6	10.4	5.4	3.9	2.3	1.2
Upper CI	100.0	99.9	96.1	93.2	83.1	73.5	64.5	49.0	40.2	33.4	24.5	20.7	10.9	8.3	6.1	4.2
Number	170	169	161	155	137	120	106	84	69	59	45	37	25	20	13	8

Table W6K-44 Age Group = 61-70 Gender = FEMALE Occupational group = NON-MANUAL Total cases = 115

	Threshold dB HL≥															
	0	5	10	15	20	25	30	35	40	45	50	55	65	75	85	95
Prevalence	100.0	100.0	100.0	98.2	95.4	92.3	86.0	75.9	69.6	63.7	47.5	39.5	25.9	13.1	8.5	5.5
Lower CI	96.8	96.8	96.8	92.5	88.6	84.3	76.5	66.1	59.3	53.5	38.0	30.8	18.8	8.1	4.9	3.1
Upper CI	100.0	100.0	100.0	99.6	98.2	96.4	92.0	83.6	78.2	72.9	57.2	49.0	34.6	20.7	14.4	9.5
Number	115	115	115	113	110	107	101	91	84	76	61	53	36	24	19	15

Table W6K-45 Age Group = 71-80 Gender = FEMALE Occupational group = NON-MANUAL Total cases = 66

	Threshold dB HL≥															
	0	5	10	15	20	25	30	35	40	45	50	55	65	75	85	95
Prevalence	100.0	100.0	100.0	98.6	95.8	92.3	88.8	80.9	80.9	75.4	75.4	75.1	57.6	28.8	11.4	6.2
Lower CI	94.5	94.5	94.5	92.2	85.0	77.9	75.0	61.1	61.1	57.6	57.6	57.4	41.2	16.5	7.2	2.8
Upper CI	100.0	100.0	100.0	99.7	98.9	97.6	95.4	92.0	92.0	87.4	87.4	87.1	72.5	45.2	17.5	13.1
Number	66	66	66	65	64	63	60	58	58	56	56	55	42	28	17	8

Table W6K-46 Age Group = 18-30 Gender = MALE Occupational group = NON-MANUAL Total cases = 66

	Threshold dB HL≥															
	0	5	10	15	20	25	30	35	40	45	50	55	65	75	85	95
Prevalence	94.8	84.4	72.2	50.7	35.4	21.2	15.4	12.0	10.0	8.4	8.4	8.4	8.4	1.6	1.4	1.4
Lower CI	82.4	70.0	57.9	38.3	24.8	12.3	7.8	5.3	3.9	3.0	3.0	3.0	3.0	0.3	0.3	0.3
Upper CI	98.6	92.6	83.0	62.9	47.7	34.1	28.1	24.9	23.1	21.7	21.7	21.7	21.7	7.5	7.8	7.8
Number	63	59	51	34	22	15	9	8	6	4	4	4	4	2	1	1

Table W6K-47 Age Group = 31-40 Gender = MALE Occupational group = NON-MANUAL Total cases = 62

	Threshold dB HL≥															
	0	5	10	15	20	25	30	35	40	45	50	55	65	75	85	95
Prevalence	100.0	98.3	92.3	80.0	63.6	40.3	35.4	19.0	19.0	17.5	13.3	12.2	9.5	9.5	1.2	0.6
Lower CI	94.2	97.3	83.9	70.4	50.2	25.8	21.7	9.2	9.2	8.0	5.5	4.7	3.1	3.1	0.6	0.1
Upper CI	100.0	99.0	96.5	87.1	75.2	56.8	52.1	35.3	35.3	34.1	28.8	28.1	25.5	25.5	9.9	7.0
Number	62	60	56	48	40	28	24	15	15	13	10	7	5	5	2	1

Table W6K-48 Age Group = 41-50 Gender = MALE Occupational group = NON-MANUAL Total cases = 75

	Threshold dB HL≥															
	0	5	10	15	20	25	30	35	40	45	50	55	65	75	85	95
Prevalence	100.0	100.0	98.8	90.2	79.8	68.4	54.1	39.7	36.5	30.4	27.1	22.5	13.1	7.3	4.3	4.1
Lower CI	95.1	95.1	93.6	79.9	67.3	56.1	41.1	28.4	25.3	20.1	16.9	13.7	7.3	3.5	1.9	1.7
Upper CI	100.0	100.0	99.8	95.5	88.3	78.6	66.5	52.2	49.3	43.1	40.5	34.8	22.4	14.5	9.5	9.4
Number	75	75	74	69	63	52	41	32	29	24	21	18	12	9	6	5

Table W6K-49 Age Group = 51-60 Gender = MALE Occupational group = NON-MANUAL Total cases = 137

	Threshold dB HL≥															
	0	5	10	15	20	25	30	35	40	45	50	55	65	75	85	95
Prevalence	100.0	100.0	96.4	93.4	89.4	85.2	71.3	65.7	50.4	40.9	34.6	32.5	26.6	19.5	9.3	2.4
Lower CI	97.3	97.3	91.0	87.2	82.7	77.9	62.3	56.5	41.8	33.2	27.9	26.0	20.9	15.3	4.2	1.1
Upper CI	100.0	100.0	98.6	96.7	93.8	90.3	78.8	73.8	59.0	49.1	42.0	39.7	33.2	24.6	19.4	5.4
Number	137	137	133	129	124	117	103	94	76	64	55	51	41	30	14	6

Prevalence by age group, gender and occupational group for the worse ear over 6 kHz

Table W6K-50 | Age Group = 61-70 | Gender = MALE | Occupational group = NON-MANUAL | Total cases = 111

	Threshold dB HL≥															
	0	5	10	15	20	25	30	35	40	45	50	55	65	75	85	95
Prevalence	**100.0**	**100.0**	**100.0**	**98.5**	**97.2**	**96.6**	**91.1**	**85.6**	**79.8**	**74.7**	**65.9**	**63.2**	**44.6**	**31.0**	**18.5**	**11.1**
Lower CI	96.7	96.7	96.7	92.0	90.2	90.1	73.1	68.6	63.6	59.1	51.3	48.7	37.2	24.9	13.7	7.5
Upper CI	100.0	100.0	100.0	99.7	99.2	98.9	97.5	94.2	89.9	85.8	78.0	75.7	52.3	37.8	24.6	16.3
Number	111	111	111	110	109	107	106	103	97	93	83	79	59	42	26	16

Table W6K-51 | Age Group = 71-80 | Gender = MALE | Occupational group = NON-MANUAL | Total cases = 55

	Threshold dB HL≥															
	0	5	10	15	20	25	30	35	40	45	50	55	65	75	85	95
Prevalence	**100.0**	**100.0**	**100.0**	**100.0**	**100.0**	**97.9**	**96.3**	**96.3**	**89.1**	**83.3**	**83.3**	**74.2**	**57.0**	**45.1**	**30.3**	**17.3**
Lower CI	93.5	93.5	93.5	93.5	93.5	89.0	87.2	87.2	76.2	71.7	71.7	60.3	42.7	33.5	20.1	10.0
Upper CI	100.0	100.0	100.0	100.0	100.0	99.6	99.0	99.0	95.4	90.7	90.7	84.6	70.3	57.3	42.9	28.3
Number	55	55	55	55	55	54	53	53	50	47	47	43	33	26	17	10

Table W6K-52 | Age Group = 18-30 | Gender = FEMALE | Occupational group = MANUAL | Total cases = 109

	Threshold dB HL≥															
	0	5	10	15	20	25	30	35	40	45	50	55	65	75	85	95
Prevalence	**99.2**	**96.2**	**88.0**	**63.8**	**44.1**	**25.4**	**20.7**	**13.4**	**9.8**	**6.4**	**6.3**	**2.3**	**1.8**	**0.2**	**0.0**	**0.0**
Lower CI	97.0	89.8	80.0	51.4	32.6	16.6	12.6	7.3	4.7	2.4	2.3	0.9	0.5	0.0	0.0	0.0
Upper CI	99.8	98.7	93.1	74.5	56.3	36.8	32.2	23.2	19.3	15.7	15.7	6.1	5.9	1.1	3.4	3.4
Number	107	104	90	68	54	38	29	21	15	11	10	6	4	1	0	0

Table W6K-53 | Age Group = 31-40 | Gender = FEMALE | Occupational group = MANUAL | Total cases = 102

	Threshold dB HL≥															
	0	5	10	15	20	25	30	35	40	45	50	55	65	75	85	95
Prevalence	**100.0**	**100.0**	**96.2**	**88.2**	**66.9**	**47.6**	**36.4**	**22.4**	**16.8**	**14.1**	**13.7**	**8.9**	**2.9**	**1.0**	**0.3**	**0.0**
Lower CI	96.4	96.4	90.2	77.9	53.9	35.5	25.7	14.3	9.9	7.8	7.5	5.1	1.7	0.3	0.1	0.0
Upper CI	100.0	100.0	98.6	94.0	77.8	59.9	48.7	33.2	27.1	24.1	23.8	15.2	4.9	2.7	1.7	3.6
Number	102	102	97	91	76	57	45	34	27	22	21	18	10	3	1	0

Table W6K-54 | Age Group = 41-50 | Gender = FEMALE | Occupational group = MANUAL | Total cases = 133

	Threshold dB HL≥															
	0	5	10	15	20	25	30	35	40	45	50	55	65	75	85	95
Prevalence	**100.0**	**97.5**	**93.0**	**85.4**	**69.4**	**56.8**	**40.4**	**30.7**	**23.8**	**17.7**	**14.6**	**4.7**	**3.9**	**3.7**	**1.5**	**0.9**
Lower CI	97.2	91.0	86.0	76.8	59.6	46.9	31.4	23.0	17.0	12.2	9.5	2.8	2.2	2.1	0.9	0.3
Upper CI	100.0	99.3	96.6	91.2	77.7	66.2	50.0	39.6	32.2	24.9	21.8	7.6	6.8	6.6	2.7	2.2
Number	133	131	124	116	96	82	63	51	42	34	27	14	11	10	5	4

Table W6K-55 | Age Group = 51-60 | Gender = FEMALE | Occupational group = MANUAL | Total cases = 164

	Threshold dB HL≥															
	0	5	10	15	20	25	30	35	40	45	50	55	65	75	85	95
Prevalence	**100.0**	**99.3**	**95.5**	**91.6**	**85.7**	**78.2**	**63.6**	**47.9**	**38.9**	**33.1**	**26.8**	**18.9**	**10.3**	**2.9**	**2.8**	**1.8**
Lower CI	97.7	96.3	90.4	85.6	78.3	69.5	54.2	38.9	30.5	25.2	19.5	12.7	6.1	1.8	1.7	0.8
Upper CI	100.0	99.9	97.9	95.3	90.8	85.0	72.1	57.0	48.2	42.1	35.5	27.2	16.9	4.7	4.6	3.8
Number	164	163	158	153	145	137	119	90	77	66	54	41	27	16	15	8

Table W6K-56 | Age Group = 61-70 | Gender = FEMALE | Occupational group = MANUAL | Total cases = 123

	Threshold dB HL≥															
	0	5	10	15	20	25	30	35	40	45	50	55	65	75	85	95
Prevalence	**100.0**	**100.0**	**100.0**	**95.4**	**94.6**	**89.0**	**81.4**	**76.2**	**63.0**	**53.5**	**45.4**	**40.2**	**27.7**	**18.5**	**9.0**	**5.1**
Lower CI	97.0	97.0	97.0	88.3	87.7	80.3	70.7	65.7	52.4	44.2	36.2	31.5	21.2	12.1	6.1	3.0
Upper CI	100.0	100.0	100.0	98.3	97.7	94.2	88.8	84.2	72.5	62.6	54.9	49.5	35.3	27.2	13.1	8.5
Number	123	123	123	120	118	112	107	102	90	79	69	63	48	34	20	10

Prevalence by age group, gender and occupational group for the worse ear over 6 kHz

Table W6K-57 Age Group = 71-80 Gender = FEMALE Occupational group = MANUAL Total cases = 74

	Threshold dB HL≥ 0	5	10	15	20	25	30	35	40	45	50	55	65	75	85	95
Prevalence	**100.0**	**100.0**	**100.0**	**100.0**	**96.6**	**96.6**	**96.6**	**96.6**	**91.4**	**90.8**	**90.8**	**81.9**	**55.6**	**32.6**	**24.8**	**14.9**
Lower CI	95.1	95.1	95.1	95.1	82.6	82.6	82.6	82.6	71.8	71.7	71.7	63.2	42.0	22.7	15.3	6.9
Upper CI	100.0	100.0	100.0	100.0	99.4	99.4	99.4	99.4	97.8	97.5	97.5	92.2	68.5	44.4	37.6	29.3
Number	74	74	74	74	73	73	73	73	72	71	71	68	57	43	32	17

Table W6K-58 Age Group = 18-30 Gender = MALE Occupational group = MANUAL Total cases = 108

	Threshold dB HL≥ 0	5	10	15	20	25	30	35	40	45	50	55	65	75	85	95
Prevalence	**94.3**	**89.8**	**77.2**	**57.4**	**42.1**	**29.1**	**19.2**	**11.2**	**8.3**	**5.0**	**4.4**	**3.1**	**1.3**	**1.3**	**1.0**	**1.0**
Lower CI	88.8	81.5	65.5	44.7	30.6	19.1	11.7	6.5	4.4	2.2	1.8	1.0	0.3	0.3	0.2	0.2
Upper CI	97.2	94.6	85.8	69.2	54.6	41.6	29.8	18.8	15.2	10.9	10.5	8.8	5.0	5.0	5.1	5.1
Number	101	98	87	70	56	39	29	20	16	10	8	6	3	3	2	2

Table W6K-59 Age Group = 31-40 Gender = MALE Occupational group = MANUAL Total cases = 80

	Threshold dB HL≥ 0	5	10	15	20	25	30	35	40	45	50	55	65	75	85	95
Prevalence	**100.0**	**99.3**	**95.8**	**85.1**	**71.9**	**59.2**	**51.4**	**38.5**	**34.5**	**26.2**	**15.7**	**7.1**	**5.7**	**3.6**	**3.6**	**2.5**
Lower CI	95.4	97.4	84.3	71.4	56.3	43.8	36.6	25.8	22.7	15.2	8.3	3.8	2.7	1.2	1.2	0.6
Upper CI	100.0	99.8	99.0	92.9	83.5	72.9	65.9	53.1	48.6	41.2	27.8	13.1	11.6	10.3	10.3	9.8
Number	80	78	76	68	61	50	43	34	30	24	19	14	10	6	6	3

Table W6K-60 Age Group = 41-50 Gender = MALE Occupational group = MANUAL Total cases = 124

	Threshold dB HL≥ 0	5	10	15	20	25	30	35	40	45	50	55	65	75	85	95
Prevalence	**100.0**	**100.0**	**99.3**	**95.3**	**87.1**	**76.1**	**66.1**	**54.8**	**43.9**	**35.3**	**27.0**	**24.1**	**16.5**	**13.1**	**5.9**	**3.7**
Lower CI	97.0	97.0	97.3	87.9	78.0	65.5	55.1	43.7	33.9	26.3	19.1	16.6	10.6	7.8	3.0	1.5
Upper CI	100.0	100.0	99.8	98.3	92.8	84.3	75.6	65.4	54.4	45.4	36.7	33.7	24.7	21.2	11.2	8.8
Number	124	124	122	118	110	96	87	76	65	56	44	39	32	23	13	8

Table W6K-61 Age Group = 51-60 Gender = MALE Occupational group = MANUAL Total cases = 193

	Threshold dB HL≥ 0	5	10	15	20	25	30	35	40	45	50	55	65	75	85	95
Prevalence	**100.0**	**100.0**	**99.7**	**98.1**	**94.0**	**86.6**	**83.4**	**73.4**	**69.0**	**58.7**	**53.1**	**46.9**	**35.4**	**22.7**	**15.9**	**10.4**
Lower CI	98.0	98.0	98.3	94.0	88.4	79.3	75.9	65.0	60.3	50.2	44.8	38.9	28.2	16.7	11.2	6.5
Upper CI	100.0	100.0	99.9	99.4	97.0	91.6	89.0	80.3	76.6	66.6	61.3	55.1	43.4	30.0	22.2	16.2
Number	193	193	192	190	184	173	169	156	150	135	124	112	89	55	39	23

Table W6K-62 Age Group = 61-70 Gender = MALE Occupational group = MANUAL Total cases = 169

	Threshold dB HL≥ 0	5	10	15	20	25	30	35	40	45	50	55	65	75	85	95
Prevalence	**100.0**	**100.0**	**100.0**	**100.0**	**98.8**	**98.8**	**97.2**	**91.5**	**90.5**	**88.9**	**80.3**	**72.0**	**52.7**	**36.2**	**21.8**	**12.4**
Lower CI	97.8	97.8	97.8	97.8	93.3	93.3	91.7	85.1	84.1	82.8	71.6	62.1	42.8	28.2	15.9	9.2
Upper CI	100.0	100.0	100.0	100.0	99.8	99.8	99.1	95.3	94.5	93.0	86.9	80.1	62.4	44.9	29.1	16.6
Number	169	169	169	169	168	168	166	160	157	155	146	138	114	89	64	42

Table W6K-63 Age Group = 71-80 Gender = MALE Occupational group = MANUAL Total cases = 71

	Threshold dB HL≥ 0	5	10	15	20	25	30	35	40	45	50	55	65	75	85	95
Prevalence	**100.0**	**100.0**	**100.0**	**100.0**	**100.0**	**100.0**	**100.0**	**100.0**	**100.0**	**97.1**	**96.3**	**81.5**	**76.4**	**61.4**	**42.6**	**29.4**
Lower CI	94.9	94.9	94.9	94.9	94.9	94.9	94.9	94.9	94.9	87.2	87.0	74.4	68.5	46.6	27.5	16.3
Upper CI	100.0	100.0	100.0	100.0	100.0	100.0	100.0	100.0	100.0	99.4	99.0	86.9	82.9	74.3	59.3	47.0
Number	71	71	71	71	71	71	71	71	71	69	68	66	61	51	39	26

Prevalence by age group, gender and occupational group for the worse ear over 8 kHz

Table W8K-1 Age Group = 18-80 Gender = OVERALL Occupational group = OVERALL Total cases = 2638

Threshold dB HL≥	0	5	10	15	20	25	30	35	40	45	50	55	65	75	85	95
**Prevalence**	**95.2**	**88.6**	**78.1**	**67.5**	**58.3**	**48.9**	**43.5**	**39.0**	**35.3**	**30.9**	**27.6**	**23.7**	**17.1**	**10.9**	**6.6**	**3.2**
**Lower CI**	93.8	86.8	76.0	65.4	56.3	47.0	41.7	37.3	33.6	29.4	26.1	22.4	15.8	9.8	5.8	2.7
**Upper CI**	96.3	90.2	80.1	69.5	60.4	50.7	45.3	40.7	37.0	32.5	29.1	25.1	18.4	12.0	7.6	3.8
**Number**	2568	2460	2293	2080	1878	1675	1532	1401	1289	1173	1056	950	731	500	324	184

Table W8K-2 Age Group = 18-80 Gender = FEMALE Occupational group = OVERALL Total cases = 1378

Threshold dB HL≥	0	5	10	15	20	25	30	35	40	45	50	55	65	75	85	95
**Prevalence**	**95.1**	**89.0**	**77.4**	**65.4**	**56.1**	**46.3**	**40.9**	**36.1**	**31.6**	**27.6**	**23.9**	**19.8**	**14.0**	**8.5**	**5.3**	**2.3**
**Lower CI**	93.2	86.7	74.7	62.7	53.4	43.9	38.6	33.9	29.5	25.6	22.0	18.1	12.3	7.2	4.2	1.7
**Upper CI**	96.4	91.0	79.9	68.0	58.7	48.7	43.2	38.3	33.9	29.7	25.9	21.7	15.8	10.1	6.6	3.0
**Number**	1338	1276	1166	1029	905	794	714	633	565	512	449	391	294	195	127	69

Table W8K-3 Age Group = 18-80 Gender = MALE Occupational group = OVERALL Total cases = 1260

Threshold dB HL≥	0	5	10	15	20	25	30	35	40	45	50	55	65	75	85	95
**Prevalence**	**95.2**	**88.2**	**78.9**	**69.8**	**60.9**	**51.8**	**46.5**	**42.2**	**39.3**	**34.5**	**31.7**	**28.1**	**20.5**	**13.5**	**8.1**	**4.2**
**Lower CI**	92.9	85.1	75.5	66.5	57.8	48.9	43.7	39.7	36.8	32.2	29.4	26.0	18.6	11.9	6.8	3.4
**Upper CI**	96.8	90.7	81.9	73.0	63.9	54.6	49.3	44.8	41.9	36.9	34.0	30.3	22.6	15.2	9.6	5.3
**Number**	1230	1184	1127	1051	973	881	818	768	724	661	607	559	437	305	197	115

Table W8K-4 Age Group = 18-30 Gender = OVERALL Occupational group = OVERALL Total cases = 401

Threshold dB HL≥	0	5	10	15	20	25	30	35	40	45	50	55	65	75	85	95
**Prevalence**	**88.2**	**73.2**	**54.2**	**38.0**	**26.9**	**15.9**	**11.9**	**8.9**	**7.7**	**5.1**	**4.3**	**2.9**	**1.6**	**1.1**	**0.7**	**0.3**
**Lower CI**	83.8	67.6	48.4	32.6	22.2	12.2	8.8	6.3	5.3	3.2	2.5	1.6	0.7	0.5	0.2	0.1
**Upper CI**	91.5	78.2	59.8	43.7	32.1	20.4	15.9	12.4	11.2	8.0	7.1	5.2	3.4	2.9	2.4	1.6
**Number**	358	305	239	174	124	81	62	48	37	28	22	17	10	7	3	2

Table W8K-5 Age Group = 31-40 Gender = OVERALL Occupational group = OVERALL Total cases = 330

Threshold dB HL≥	0	5	10	15	20	25	30	35	40	45	50	55	65	75	85	95
**Prevalence**	**91.3**	**83.1**	**65.9**	**50.6**	**36.5**	**24.1**	**19.0**	**15.1**	**13.6**	**10.2**	**7.7**	**5.4**	**3.3**	**1.6**	**1.2**	**1.0**
**Lower CI**	86.6	77.6	59.3	44.0	30.5	19.1	14.5	11.1	9.8	7.0	4.9	3.2	1.6	0.7	0.4	0.3
**Upper CI**	94.5	87.4	71.9	57.1	43.0	29.9	24.5	20.1	18.6	14.5	11.8	9.1	6.6	3.5	3.1	3.1
**Number**	308	281	239	188	144	106	86	68	60	50	35	26	16	10	7	4

Table W8K-6 Age Group = 41-50 Gender = OVERALL Occupational group = OVERALL Total cases = 443

Threshold dB HL≥	0	5	10	15	20	25	30	35	40	45	50	55	65	75	85	95
**Prevalence**	**99.3**	**93.6**	**83.6**	**70.6**	**59.2**	**46.1**	**39.0**	**32.2**	**26.6**	**20.5**	**16.7**	**14.4**	**7.5**	**3.9**	**1.8**	**0.8**
**Lower CI**	97.6	90.3	79.4	65.7	54.2	41.2	34.3	27.7	22.5	16.9	13.5	11.5	5.4	2.6	1.1	0.4
**Upper CI**	99.8	95.8	87.1	75.0	64.0	51.1	44.0	36.9	31.1	24.5	20.5	17.9	10.3	5.9	3.2	1.5
**Number**	440	420	381	330	284	234	200	168	145	118	99	87	51	30	17	10

Table W8K-7 Age Group = 51-60 Gender = OVERALL Occupational group = OVERALL Total cases = 672

Threshold dB HL≥	0	5	10	15	20	25	30	35	40	45	50	55	65	75	85	95
**Prevalence**	**99.7**	**98.1**	**94.1**	**86.2**	**77.7**	**68.3**	**60.6**	**51.5**	**45.2**	**38.5**	**32.0**	**26.8**	**16.9**	**10.8**	**6.0**	**3.2**
**Lower CI**	98.6	96.5	91.6	82.9	73.8	64.2	56.4	47.3	41.1	34.6	28.4	23.5	14.4	8.8	4.6	2.3
**Upper CI**	99.9	99.0	95.9	89.1	81.1	72.2	64.6	55.7	49.4	42.5	35.8	30.3	19.8	13.2	7.8	4.5
**Number**	670	662	644	604	557	503	457	405	363	321	271	238	168	116	70	42

Prevalence by age group, gender and occupational group for the worse ear over 8 kHz

Table W8K-8 Age Group = 61-70 Gender = OVERALL Occupational group = OVERALL Total cases = 526

	Threshold dB HL≥															
	**0**	**5**	**10**	**15**	**20**	**25**	**30**	**35**	**40**	**45**	**50**	**55**	**65**	**75**	**85**	**95**
**Prevalence**	**100.0**	**100.0**	**99.5**	**97.9**	**94.0**	**89.4**	**84.3**	**81.6**	**75.2**	**70.5**	**65.8**	**57.0**	**44.6**	**26.3**	**16.1**	**5.6**
**Lower CI**	99.3	99.3	97.5	95.3	90.8	85.4	79.8	76.9	70.1	65.4	60.7	51.9	39.7	22.1	12.8	4.4
**Upper CI**	100.0	100.0	99.9	99.1	96.2	92.4	88.0	85.6	79.6	75.1	70.6	61.9	49.7	31.0	20.0	7.1
**Number**	526	526	525	520	505	491	472	459	434	409	386	348	288	189	122	62

Table W8K-9 Age Group = 71-80 Gender = OVERALL Occupational group = OVERALL Total cases = 266

	Threshold dB HL≥															
	**0**	**5**	**10**	**15**	**20**	**25**	**30**	**35**	**40**	**45**	**50**	**55**	**65**	**75**	**85**	**95**
**Prevalence**	**100.0**	**100.0**	**99.1**	**98.2**	**98.2**	**96.1**	**93.3**	**92.8**	**89.9**	**87.4**	**84.0**	**78.7**	**63.0**	**45.0**	**29.3**	**16.4**
**Lower CI**	98.6	98.6	95.1	94.2	94.2	91.9	88.0	87.5	83.3	80.3	76.4	71.2	54.0	37.0	22.8	12.1
**Upper CI**	100.0	100.0	99.8	99.5	99.5	98.1	96.3	95.9	94.1	92.1	89.4	84.6	71.1	53.3	36.9	21.9
**Number**	266	266	265	264	264	260	255	253	250	247	243	234	198	148	105	64

Table W8K-10 Age Group = 18-80 Gender = OVERALL Occupational group = NON-MANUAL Total cases = 1173

	Threshold dB HL≥															
	**0**	**5**	**10**	**15**	**20**	**25**	**30**	**35**	**40**	**45**	**50**	**55**	**65**	**75**	**85**	**95**
**Prevalence**	**94.2**	**86.8**	**75.3**	**63.0**	**54.4**	**46.2**	**41.7**	**37.6**	**34.2**	**30.3**	**27.6**	**23.1**	**16.1**	**9.6**	**5.6**	**2.3**
**Lower CI**	92.0	84.1	72.2	59.9	51.5	43.3	39.0	34.9	31.5	27.8	25.1	20.8	14.2	8.3	4.6	1.8
**Upper CI**	95.9	89.1	78.1	66.0	57.4	49.0	44.5	40.3	37.0	33.0	30.2	25.7	18.2	11.2	6.6	2.9
**Number**	1132	1077	987	871	779	691	631	573	528	476	433	380	279	174	108	63

Table W8K-11 Age Group = 18-80 Gender = OVERALL Occupational group = MANUAL Total cases = 1461

	Threshold dB HL≥															
	**0**	**5**	**10**	**15**	**20**	**25**	**30**	**35**	**40**	**45**	**50**	**55**	**65**	**75**	**85**	**95**
**Prevalence**	**96.4**	**90.8**	**80.8**	**72.0**	**61.5**	**50.7**	**45.0**	**40.3**	**36.2**	**31.4**	**27.6**	**24.2**	**17.0**	**11.5**	**7.3**	**4.0**
**Lower CI**	94.5	88.2	77.7	68.7	58.4	47.9	42.4	37.9	33.9	29.3	25.6	22.5	15.4	10.1	6.2	3.2
**Upper CI**	97.7	92.9	83.6	75.0	64.6	53.4	47.6	42.7	38.6	33.6	29.7	26.0	18.7	13.1	8.5	5.0
**Number**	1432	1380	1304	1207	1097	982	899	826	759	696	622	569	451	325	216	121

Table W8K-12 Age Group = 18-30 Gender = FEMALE Occupational group = OVERALL Total cases = 224

	Threshold dB HL≥															
	**0**	**5**	**10**	**15**	**20**	**25**	**30**	**35**	**40**	**45**	**50**	**55**	**65**	**75**	**85**	**95**
**Prevalence**	**88.1**	**75.2**	**55.9**	**35.9**	**25.9**	**13.9**	**11.1**	**8.5**	**7.0**	**5.3**	**3.8**	**2.5**	**1.6**	**0.8**	**0.0**	**0.0**
**Lower CI**	82.1	68.1	48.5	29.3	20.1	9.8	7.5	5.5	4.1	3.0	1.9	1.2	0.6	0.2	0.0	0.0
**Upper CI**	92.3	81.2	63.0	43.0	32.7	19.4	16.1	13.1	11.5	9.2	7.4	5.0	4.1	2.9	1.7	1.7
**Number**	201	172	134	94	67	42	34	26	19	16	11	9	5	3	0	0

Table W8K-13 Age Group = 31-40 Gender = FEMALE Occupational group = OVERALL Total cases = 188

	Threshold dB HL≥															
	**0**	**5**	**10**	**15**	**20**	**25**	**30**	**35**	**40**	**45**	**50**	**55**	**65**	**75**	**85**	**95**
**Prevalence**	**90.4**	**82.0**	**64.5**	**51.1**	**37.4**	**23.2**	**16.8**	**13.3**	**10.7**	**8.2**	**6.5**	**4.9**	**2.0**	**0.8**	**0.5**	**0.3**
**Lower CI**	83.9	74.7	56.1	42.9	29.8	17.3	12.0	8.8	6.7	4.9	3.6	2.5	0.8	0.3	0.1	0.1
**Upper CI**	94.4	87.5	72.0	59.2	45.7	30.3	23.0	19.4	16.6	13.3	11.3	9.5	4.5	2.3	1.9	1.7
**Number**	175	161	136	106	80	60	48	34	27	23	17	13	7	3	2	1

Table W8K-14 Age Group = 41-50 Gender = FEMALE Occupational group = OVERALL Total cases = 245

	Threshold dB HL≥															
	**0**	**5**	**10**	**15**	**20**	**25**	**30**	**35**	**40**	**45**	**50**	**55**	**65**	**75**	**85**	**95**
**Prevalence**	**99.2**	**93.4**	**77.0**	**60.9**	**49.5**	**39.6**	**32.6**	**24.2**	**17.8**	**13.5**	**9.4**	**7.7**	**3.9**	**1.7**	**0.5**	**0.3**
**Lower CI**	96.8	88.9	70.5	54.0	42.8	33.4	26.8	19.1	13.5	10.1	6.8	5.5	2.4	0.8	0.2	0.1
**Upper CI**	99.8	96.2	82.3	67.3	56.1	46.2	39.0	30.1	23.0	17.9	12.9	10.8	6.2	3.5	1.4	1.1
**Number**	243	231	197	162	134	112	93	73	58	49	38	32	18	8	3	2

Prevalence by age group, gender and occupational group for the worse ear over 8 kHz

Table W8K-15 Age Group = 51-60 Gender = FEMALE Occupational group = OVERALL Total cases = 339

Threshold dB HL≥	0	5	10	15	20	25	30	35	40	45	50	55	65	75	85	95
Prevalence	**99.3**	**96.9**	**92.2**	**83.0**	**72.8**	**63.4**	**54.2**	**44.1**	**37.8**	**29.8**	**22.7**	**16.3**	**8.2**	**6.1**	**3.6**	**1.8**
Lower CI	97.4	93.9	88.1	77.9	67.0	57.4	48.2	38.3	32.2	24.9	18.3	12.7	6.2	4.4	2.4	1.1
Upper CI	99.8	98.4	95.0	87.2	77.8	69.0	60.0	50.1	43.7	35.3	27.7	20.6	10.9	8.5	5.5	2.8
Number	337	330	319	293	262	232	205	176	155	132	106	85	58	44	29	18

Table W8K-16 Age Group = 61-70 Gender = FEMALE Occupational group = OVERALL Total cases = 243

Threshold dB HL≥	0	5	10	15	20	25	30	35	40	45	50	55	65	75	85	95
Prevalence	**100.0**	**100.0**	**99.2**	**96.2**	**89.8**	**83.4**	**78.2**	**74.7**	**67.3**	**61.9**	**56.5**	**46.8**	**36.4**	**18.8**	**11.9**	**3.3**
Lower CI	98.4	98.4	95.4	91.6	84.0	76.8	71.4	67.7	60.1	54.8	49.5	40.0	30.3	13.8	8.0	2.1
Upper CI	100.0	100.0	99.9	98.3	93.7	88.4	83.8	80.6	73.8	68.6	63.3	53.7	43.0	24.9	17.4	5.1
Number	243	243	242	237	225	214	202	193	177	164	151	130	106	64	41	18

Table W8K-17 Age Group = 71-80 Gender = FEMALE Occupational group = OVERALL Total cases = 139

Threshold dB HL≥	0	5	10	15	20	25	30	35	40	45	50	55	65	75	85	95
Prevalence	**100.0**	**100.0**	**98.5**	**97.0**	**97.0**	**94.2**	**92.0**	**91.6**	**87.9**	**85.5**	**81.6**	**74.9**	**59.0**	**40.9**	**27.5**	**13.5**
Lower CI	97.3	97.3	91.8	90.1	90.1	87.4	83.7	83.3	77.4	74.5	70.2	64.0	46.2	30.1	18.7	8.6
Upper CI	100.0	100.0	99.7	99.1	99.1	97.4	96.3	96.0	93.9	92.2	89.3	83.4	70.7	52.7	38.5	20.6
Number	139	139	138	137	137	134	132	131	129	128	126	122	100	73	52	30

Table W8K-18 Age Group = 18-30 Gender = MALE Occupational group = OVERALL Total cases = 177

Threshold dB HL≥	0	5	10	15	20	25	30	35	40	45	50	55	65	75	85	95
Prevalence	**88.3**	**71.0**	**52.0**	**40.1**	**27.6**	**17.8**	**12.5**	**9.0**	**8.3**	**4.8**	**4.7**	**3.3**	**1.5**	**1.4**	**1.4**	**0.6**
Lower CI	81.4	62.0	43.1	31.7	20.7	12.1	7.9	5.4	4.8	2.3	2.2	1.3	0.5	0.4	0.4	0.1
Upper CI	92.9	78.6	60.7	49.1	35.8	25.4	19.3	14.7	14.0	9.8	9.7	7.7	4.7	4.7	4.7	3.1
Number	157	133	105	80	57	39	28	22	18	12	11	8	5	4	3	2

Table W8K-19 Age Group = 31-40 Gender = MALE Occupational group = OVERALL Total cases = 142

Threshold dB HL≥	0	5	10	15	20	25	30	35	40	45	50	55	65	75	85	95
Prevalence	**92.2**	**84.0**	**67.3**	**49.9**	**35.8**	**25.1**	**21.5**	**17.2**	**17.0**	**12.4**	**9.1**	**6.1**	**4.7**	**2.5**	**1.9**	**1.7**
Lower CI	83.9	75.0	56.5	39.6	26.6	17.3	14.1	11.0	10.8	7.4	4.7	2.8	1.8	0.9	0.6	0.5
Upper CI	96.4	90.2	76.5	60.2	46.0	34.9	31.2	25.9	25.7	20.3	16.8	12.8	11.7	6.7	6.3	6.3
Number	133	120	103	82	64	46	38	34	33	27	18	13	9	7	5	3

Table W8K-20 Age Group = 41-50 Gender = MALE Occupational group = OVERALL Total cases = 198

Threshold dB HL≥	0	5	10	15	20	25	30	35	40	45	50	55	65	75	85	95
Prevalence	**99.3**	**93.8**	**91.0**	**81.2**	**69.8**	**53.2**	**45.9**	**40.9**	**36.3**	**28.1**	**24.7**	**21.7**	**11.5**	**6.4**	**3.4**	**1.3**
Lower CI	96.1	88.2	85.0	74.1	62.2	45.5	38.4	33.6	29.4	21.9	18.9	16.2	7.7	4.0	1.8	0.7
Upper CI	99.9	96.8	94.8	86.8	76.5	60.7	53.6	48.6	43.9	35.3	31.7	28.5	16.9	10.2	6.1	2.6
Number	197	189	184	168	150	122	107	95	87	69	61	55	33	22	14	8

Table W8K-21 Age Group = 51-60 Gender = MALE Occupational group = OVERALL Total cases = 333

Threshold dB HL≥	0	5	10	15	20	25	30	35	40	45	50	55	65	75	85	95
Prevalence	**100.0**	**99.6**	**96.3**	**89.9**	**83.2**	**73.8**	**67.8**	**59.8**	**53.5**	**48.1**	**42.3**	**38.5**	**26.5**	**16.1**	**8.6**	**4.8**
Lower CI	98.9	97.9	92.7	85.2	77.8	68.1	61.9	53.8	47.6	42.2	36.5	32.9	21.8	12.4	6.1	3.0
Upper CI	100.0	99.9	98.1	93.2	87.4	78.9	73.2	65.5	59.4	54.0	48.2	44.4	31.9	20.6	11.9	7.5
Number	333	332	325	311	295	271	252	229	208	189	165	153	110	72	41	24

Prevalence by age group, gender and occupational group for the worse ear over 8 kHz

Table W8K-22 Age Group = 61-70 Gender = MALE Occupational group = OVERALL Total cases = 283

	Threshold dB HL≥															
	0	5	10	15	20	25	30	35	40	45	50	55	65	75	85	95
Prevalence	**100.0**	**100.0**	**100.0**	**100.0**	**98.9**	**96.4**	**91.4**	**89.7**	**84.2**	**80.4**	**76.6**	**68.7**	**54.3**	**35.3**	**21.0**	**8.3**
Lower CI	98.7	98.7	98.7	98.7	96.7	90.9	84.7	82.9	76.8	73.0	69.0	61.1	46.5	28.5	16.0	6.2
Upper CI	100.0	100.0	100.0	100.0	99.7	98.6	95.4	94.0	89.6	86.2	82.8	75.4	61.9	42.7	27.1	11.0
Number	283	283	283	283	280	277	270	266	257	245	235	218	182	125	81	44

Table W8K-23 Age Group = 71-80 Gender = MALE Occupational group = OVERALL Total cases = 127

	Threshold dB HL≥															
	0	5	10	15	20	25	30	35	40	45	50	55	65	75	85	95
Prevalence	**100.0**	**100.0**	**100.0**	**100.0**	**100.0**	**98.7**	**95.1**	**94.2**	**92.9**	**90.3**	**87.9**	**84.7**	**69.3**	**50.8**	**32.0**	**20.8**
Lower CI	97.1	97.1	97.1	97.1	97.1	92.7	88.7	87.7	86.5	82.9	79.1	75.7	58.1	40.2	23.5	13.7
Upper CI	100.0	100.0	100.0	100.0	100.0	99.8	97.9	97.4	96.4	94.7	93.3	90.8	78.6	61.5	41.8	30.2
Number	127	127	127	127	127	126	123	122	121	119	117	112	98	75	53	34

Table W8K-24 Age Group = 18-30 Gender = OVERALL Occupational group = NON-MANUAL Total cases = 181

	Threshold dB HL≥															
	0	5	10	15	20	25	30	35	40	45	50	55	65	75	85	95
Prevalence	**86.0**	**70.7**	**52.3**	**30.1**	**18.7**	**11.9**	**9.8**	**7.2**	**6.8**	**6.1**	**5.6**	**3.9**	**1.4**	**1.0**	**0.7**	**0.0**
Lower CI	78.6	62.5	44.2	23.0	13.0	7.4	5.6	3.6	3.3	2.8	2.4	1.5	0.5	0.2	0.1	0.0
Upper CI	91.2	77.8	60.2	38.2	26.1	18.7	16.6	14.0	13.6	12.9	12.3	10.0	4.4	3.9	4.0	2.1
Number	158	134	103	60	38	26	20	13	10	9	8	7	4	3	1	0

Table W8K-25 Age Group = 31-40 Gender = OVERALL Occupational group = NON-MANUAL Total cases = 147

	Threshold dB HL≥															
	0	5	10	15	20	25	30	35	40	45	50	55	65	75	85	95
Prevalence	**89.1**	**77.4**	**57.3**	**44.7**	**35.2**	**23.5**	**18.5**	**14.3**	**13.8**	**11.1**	**8.6**	**5.7**	**4.3**	**0.9**	**0.3**	**0.3**
Lower CI	82.6	69.9	47.9	35.5	26.5	16.0	11.9	8.5	8.1	6.1	4.3	2.3	1.4	0.3	0.0	0.0
Upper CI	93.4	83.4	66.2	54.3	45.0	33.0	27.5	22.9	22.5	19.2	16.6	13.5	12.1	3.2	3.1	3.1
Number	131	114	92	70	53	37	30	24	23	19	14	8	6	2	1	1

Table W8K-26 Age Group = 41-50 Gender = OVERALL Occupational group = NON-MANUAL Total cases = 187

	Threshold dB HL≥															
	0	5	10	15	20	25	30	35	40	45	50	55	65	75	85	95
Prevalence	**100.0**	**94.0**	**82.8**	**70.7**	**58.0**	**44.2**	**37.8**	**33.1**	**28.3**	**19.7**	**17.8**	**16.6**	**6.7**	**3.0**	**1.4**	**1.1**
Lower CI	98.0	88.6	76.3	63.3	50.3	36.4	30.4	26.1	21.7	14.0	12.4	11.4	4.1	1.6	1.0	0.7
Upper CI	100.0	96.9	87.8	77.1	65.4	52.3	45.8	41.0	36.0	26.9	24.8	23.5	10.7	5.6	1.9	1.8
Number	187	178	156	135	112	88	74	63	54	41	36	32	16	9	6	4

Table W8K-27 Age Group = 51-60 Gender = OVERALL Occupational group = NON-MANUAL Total cases = 310

	Threshold dB HL≥															
	0	5	10	15	20	25	30	35	40	45	50	55	65	75	85	95
Prevalence	**99.3**	**97.2**	**91.0**	**81.7**	**71.7**	**62.4**	**56.7**	**47.2**	**41.7**	**35.6**	**30.5**	**23.3**	**16.1**	**9.6**	**3.4**	**2.5**
Lower CI	97.5	94.0	86.3	76.2	65.7	56.3	50.6	41.4	36.0	30.3	25.5	18.3	11.8	6.4	2.3	1.5
Upper CI	99.8	98.7	94.2	86.1	77.0	68.1	62.6	53.2	47.6	41.2	35.9	29.1	21.6	14.2	5.0	3.9
Number	308	303	290	265	239	211	192	164	146	125	107	92	65	43	24	18

Table W8K-28 Age Group = 61-70 Gender = OVERALL Occupational group = NON-MANUAL Total cases = 227

	Threshold dB HL≥															
	0	5	10	15	20	25	30	35	40	45	50	55	65	75	85	95
Prevalence	**100.0**	**100.0**	**99.2**	**96.6**	**95.1**	**89.9**	**85.1**	**83.2**	**76.9**	**71.7**	**66.8**	**56.1**	**41.9**	**24.6**	**14.5**	**5.8**
Lower CI	98.3	98.3	95.4	92.0	90.2	81.7	76.6	74.6	68.4	63.5	58.3	47.9	34.3	18.3	10.9	3.8
Upper CI	100.0	100.0	99.9	98.6	97.6	94.7	90.9	89.3	83.6	78.8	74.3	64.0	49.9	32.3	19.0	8.5
Number	227	227	226	222	218	212	203	199	188	176	164	142	107	66	44	23

Prevalence by age group, gender and occupational group for the worse ear over 8 kHz

Table W8K-29 Age Group = 71-80 Gender = OVERALL Occupational group = NON-MANUAL Total cases = 121

Threshold dB HL≥	0	5	10	15	20	25	30	35	40	45	50	55	65	75	85	95
**Prevalence**	**100.0**	**100.0**	**98.4**	**96.8**	**96.8**	**95.1**	**90.3**	**89.4**	**83.7**	**82.5**	**79.2**	**74.4**	**60.7**	**41.1**	**27.1**	**9.8**
**Lower CI**	96.9	96.9	91.5	90.4	90.4	88.5	81.6	80.8	71.2	69.8	66.4	61.7	48.7	32.8	20.3	6.3
**Upper CI**	100.0	100.0	99.7	99.0	99.0	98.0	95.1	94.5	91.5	90.6	88.0	83.9	71.5	50.0	35.2	14.9
**Number**	121	121	120	119	119	117	112	110	107	106	104	99	81	51	32	17

Table W8K-30 Age Group = 18-30 Gender = OVERALL Occupational group = MANUAL Total cases = 218

Threshold dB HL≥	0	5	10	15	20	25	30	35	40	45	50	55	65	75	85	95
**Prevalence**	**90.8**	**76.7**	**55.3**	**46.2**	**34.1**	**19.3**	**13.6**	**10.6**	**8.9**	**5.5**	**4.4**	**2.1**	**1.5**	**1.2**	**0.5**	**0.5**
**Lower CI**	84.5	68.3	46.4	37.6	26.4	13.4	8.9	6.8	5.4	2.8	1.9	0.9	0.5	0.4	0.1	0.1
**Upper CI**	94.7	83.4	63.9	54.9	42.6	27.0	20.2	16.1	14.4	10.5	9.5	4.5	4.0	3.9	2.6	2.6
**Number**	198	170	136	114	86	55	42	35	27	19	14	10	6	4	2	2

Table W8K-31 Age Group = 31-40 Gender = OVERALL Occupational group = MANUAL Total cases = 183

Threshold dB HL≥	0	5	10	15	20	25	30	35	40	45	50	55	65	75	85	95
**Prevalence**	**95.1**	**90.9**	**76.2**	**57.9**	**39.7**	**24.5**	**19.8**	**16.2**	**13.8**	**8.2**	**6.2**	**5.0**	**2.4**	**2.0**	**1.8**	**1.4**
**Lower CI**	88.7	83.8	66.5	48.0	31.0	17.8	13.6	10.7	8.7	5.4	3.7	2.9	1.0	0.8	0.6	0.4
**Upper CI**	97.9	95.1	83.7	67.2	49.1	32.7	27.9	23.7	21.1	12.3	10.4	8.5	5.4	5.2	5.0	5.0
**Number**	177	167	147	118	91	69	56	44	37	31	21	18	10	8	6	3

Table W8K-32 Age Group = 41-50 Gender = OVERALL Occupational group = MANUAL Total cases = 256

Threshold dB HL≥	0	5	10	15	20	25	30	35	40	45	50	55	65	75	85	95
**Prevalence**	**98.4**	**92.7**	**85.0**	**70.3**	**58.9**	**47.3**	**40.9**	**32.2**	**26.3**	**22.1**	**16.9**	**14.6**	**7.5**	**4.5**	**2.2**	**0.7**
**Lower CI**	94.7	87.2	78.8	63.1	51.7	40.3	34.1	26.1	20.9	17.1	12.6	10.6	5.1	2.7	1.0	0.3
**Upper CI**	99.5	95.9	89.6	76.6	65.6	54.3	48.1	38.9	32.6	28.1	22.4	19.8	11.0	7.7	4.7	1.5
**Number**	253	242	225	195	172	146	126	105	91	77	63	55	35	21	11	6

Table W8K-33 Age Group = 51-60 Gender = OVERALL Occupational group = MANUAL Total cases = 362

Threshold dB HL≥	0	5	10	15	20	25	30	35	40	45	50	55	65	75	85	95
**Prevalence**	**100.0**	**98.7**	**96.4**	**90.9**	**83.2**	**74.7**	**65.3**	**56.7**	**49.5**	**42.1**	**33.6**	**29.6**	**16.5**	**11.4**	**7.1**	**3.5**
**Lower CI**	99.0	96.0	92.8	86.5	77.9	68.7	59.1	50.6	43.4	36.3	28.4	24.6	13.2	8.7	5.2	2.1
**Upper CI**	100.0	99.6	98.2	94.0	87.4	79.9	71.1	62.7	55.6	48.1	39.2	35.1	20.4	14.8	9.8	5.5
**Number**	362	359	354	339	318	292	265	241	217	196	164	146	103	73	46	24

Table W8K-34 Age Group = 61-70 Gender = OVERALL Occupational group = MANUAL Total cases = 298

Threshold dB HL≥	0	5	10	15	20	25	30	35	40	45	50	55	65	75	85	95
**Prevalence**	**100.0**	**100.0**	**100.0**	**98.9**	**92.2**	**86.5**	**81.7**	**78.6**	**71.1**	**67.2**	**62.6**	**55.9**	**47.2**	**28.1**	**17.3**	**5.9**
**Lower CI**	98.7	98.7	98.7	94.0	86.3	80.1	74.6	71.6	63.7	59.9	55.5	49.6	41.0	22.5	13.1	4.4
**Upper CI**	100.0	100.0	100.0	99.8	95.6	91.0	87.1	84.3	77.5	73.9	69.1	62.0	53.5	34.4	22.5	7.8
**Number**	298	298	298	297	286	278	268	259	245	233	222	206	181	123	78	39

Table W8K-35 Age Group = 71-80 Gender = OVERALL Occupational group = MANUAL Total cases = 144

Threshold dB HL≥	0	5	10	15	20	25	30	35	40	45	50	55	65	75	85	95
**Prevalence**	**100.0**	**100.0**	**100.0**	**100.0**	**100.0**	**97.3**	**97.3**	**97.3**	**97.3**	**93.7**	**90.2**	**84.1**	**61.1**	**46.9**	**33.2**	**23.5**
**Lower CI**	97.4	97.4	97.4	97.4	97.4	89.5	89.5	89.5	89.5	83.2	77.2	72.9	48.6	36.0	24.5	15.7
**Upper CI**	100.0	100.0	100.0	100.0	100.0	99.3	99.3	99.3	99.3	97.8	96.2	91.2	72.2	58.1	43.3	33.7
**Number**	144	144	144	144	144	142	142	142	142	140	138	134	116	96	73	47

Prevalence by age group, gender and occupational group for the worse ear over 8 kHz

Table W8K-36 Age Group = 18-80 Gender = FEMALE Occupational group = NON-MANUAL Total cases = 667

Threshold dB HL≥	0	5	10	15	20	25	30	35	40	45	50	55	65	75	85	95
**Prevalence**	**94.3**	**87.5**	**74.9**	**59.2**	**50.9**	**43.5**	**38.7**	**33.8**	**29.9**	**26.4**	**23.3**	**19.9**	**13.2**	**7.7**	**5.4**	**1.6**
**Lower CI**	91.7	84.3	71.2	55.6	47.4	40.3	35.7	31.1	26.9	23.6	20.7	17.4	11.1	6.3	4.3	1.1
**Upper CI**	96.2	90.2	78.3	62.7	54.4	46.7	41.8	36.8	33.1	29.4	26.2	22.7	15.7	9.3	6.9	2.3
**Number**	642	609	547	462	405	358	324	284	258	231	208	181	129	79	55	31

Table W8K-37 Age Group = 18-80 Gender = MALE Occupational group = NON-MANUAL Total cases = 506

Threshold dB HL≥	0	5	10	15	20	25	30	35	40	45	50	55	65	75	85	95
**Prevalence**	**94.0**	**85.9**	**75.6**	**67.3**	**58.4**	**49.1**	**45.1**	**41.7**	**39.0**	**34.7**	**32.3**	**26.8**	**19.3**	**11.8**	**5.7**	**3.1**
**Lower CI**	89.8	81.2	70.5	62.1	53.6	44.3	40.3	37.1	34.4	30.3	28.0	22.7	16.2	9.5	4.4	2.3
**Upper CI**	96.6	89.5	80.1	72.1	63.1	53.9	49.9	46.4	43.8	39.4	37.0	31.2	22.9	14.6	7.3	4.2
**Number**	490	468	440	409	374	333	307	289	270	245	225	199	150	95	53	32

Table W8K-38 Age Group = 18-80 Gender = FEMALE Occupational group = MANUAL Total cases = 710

Threshold dB HL≥	0	5	10	15	20	25	30	35	40	45	50	55	65	75	85	95
**Prevalence**	**96.3**	**90.6**	**79.4**	**71.8**	**61.1**	**48.4**	**42.6**	**38.0**	**32.6**	**28.5**	**23.9**	**19.0**	**13.4**	**8.8**	**5.0**	**2.6**
**Lower CI**	93.2	86.8	75.1	67.5	56.8	44.7	39.0	34.5	29.4	25.4	20.9	16.7	11.3	7.1	3.8	1.8
**Upper CI**	98.0	93.4	83.1	75.8	65.3	52.2	46.2	41.6	36.1	31.8	27.1	21.6	15.8	10.9	6.4	3.8
**Number**	695	666	618	566	499	435	389	348	306	281	241	210	165	116	72	38

Table W8K-39 Age Group = 18-80 Gender = MALE Occupational group = MANUAL Total cases = 751

Threshold dB HL≥	0	5	10	15	20	25	30	35	40	45	50	55	65	75	85	95
**Prevalence**	**96.6**	**91.0**	**82.4**	**72.1**	**62.1**	**53.2**	**47.7**	**42.9**	**40.2**	**34.7**	**31.8**	**30.0**	**21.1**	**14.5**	**9.9**	**5.5**
**Lower CI**	93.9	87.0	77.5	67.1	57.5	49.1	43.9	39.7	37.0	32.1	29.3	27.6	18.8	12.3	8.1	4.1
**Upper CI**	98.2	93.9	86.4	76.6	66.4	57.2	51.5	46.1	43.5	37.4	34.4	32.6	23.6	17.0	12.1	7.2
**Number**	737	714	686	641	598	547	510	478	453	415	381	359	286	209	144	83

Table W8K-40 Age Group = 18-30 Gender = FEMALE Occupational group = NON-MANUAL Total cases = 115

Threshold dB HL≥	0	5	10	15	20	25	30	35	40	45	50	55	65	75	85	95
**Prevalence**	**87.8**	**76.9**	**57.4**	**28.0**	**19.6**	**11.6**	**8.3**	**4.5**	**3.9**	**2.6**	**1.6**	**1.6**	**1.3**	**0.4**	**0.0**	**0.0**
**Lower CI**	79.8	67.8	47.7	20.6	13.2	7.1	4.5	2.0	1.5	0.9	0.5	0.5	0.3	0.1	0.0	0.0
**Upper CI**	92.9	84.1	66.6	36.9	28.2	18.6	14.8	10.0	9.4	6.9	5.0	5.0	4.8	2.2	3.2	3.2
**Number**	102	90	69	38	26	18	13	7	5	4	3	3	2	1	0	0

Table W8K-41 Age Group = 31-40 Gender = FEMALE Occupational group = NON-MANUAL Total cases = 85

Threshold dB HL≥	0	5	10	15	20	25	30	35	40	45	50	55	65	75	85	95
**Prevalence**	**86.7**	**72.7**	**54.0**	**40.8**	**27.7**	**17.3**	**11.5**	**7.8**	**7.0**	**6.4**	**4.9**	**4.2**	**1.4**	**0.0**	**0.0**	**0.0**
**Lower CI**	76.8	61.7	43.4	30.7	18.6	10.4	6.2	3.6	3.0	2.5	1.7	1.3	0.3	0.0	0.0	0.0
**Upper CI**	92.8	81.5	64.3	51.7	39.1	27.5	20.2	16.1	15.6	15.1	13.0	12.5	6.5	4.3	4.3	4.3
**Number**	75	64	51	37	25	17	13	8	7	6	5	4	2	0	0	0

Table W8K-42 Age Group = 41-50 Gender = FEMALE Occupational group = NON-MANUAL Total cases = 112

Threshold dB HL≥	0	5	10	15	20	25	30	35	40	45	50	55	65	75	85	95
**Prevalence**	**100.0**	**94.6**	**76.3**	**60.2**	**46.7**	**38.9**	**31.2**	**25.3**	**18.6**	**12.0**	**9.8**	**9.4**	**2.4**	**1.5**	**0.0**	**0.0**
**Lower CI**	96.7	87.6	66.7	50.4	37.2	30.1	23.2	18.0	12.5	7.4	6.0	5.6	1.0	0.5	0.0	0.0
**Upper CI**	100.0	97.8	83.7	69.2	56.4	48.5	40.5	34.3	26.8	18.7	15.6	15.3	5.6	4.7	3.3	3.3
**Number**	112	107	88	72	57	48	39	32	25	19	16	14	5	2	0	0

Prevalence by age group, gender and occupational group for the worse ear over 8 kHz

Table W8K-43 Age Group = 51-60 Gender = FEMALE Occupational group = NON-MANUAL Total cases = 173

Threshold dB HL≥	0	5	10	15	20	25	30	35	40	45	50	55	65	75	85	95
Prevalence	98.7	94.7	88.8	76.0	67.0	57.7	52.4	41.4	37.6	29.5	22.8	15.1	8.4	6.3	3.7	2.3
Lower CI	95.3	89.0	81.9	68.0	58.8	49.4	44.2	33.7	30.2	22.9	17.1	10.9	5.9	4.3	2.4	1.3
Upper CI	99.7	97.6	93.3	82.5	74.3	65.6	60.5	49.6	45.8	37.2	29.7	20.5	12.0	9.0	5.8	3.9
Number	171	166	159	140	125	109	100	82	75	61	50	39	28	22	16	11

Table W8K-44 Age Group = 61-70 Gender = FEMALE Occupational group = NON-MANUAL Total cases = 116

Threshold dB HL≥	0	5	10	15	20	25	30	35	40	45	50	55	65	75	85	95
Prevalence	100.0	100.0	98.5	93.9	91.0	86.0	82.0	79.6	71.9	67.5	63.0	52.9	36.3	15.1	10.7	2.6
Lower CI	96.8	96.8	92.0	85.8	82.2	76.3	72.1	69.6	62.0	57.8	53.3	43.1	27.8	9.7	6.2	1.5
Upper CI	100.0	100.0	99.7	97.5	95.7	92.1	88.9	86.9	80.1	75.9	71.7	62.4	45.9	22.7	17.6	4.6
Number	116	116	115	111	108	103	98	95	88	83	77	66	48	26	19	10

Table W8K-45 Age Group = 71-80 Gender = FEMALE Occupational group = NON-MANUAL Total cases = 66

Threshold dB HL≥	0	5	10	15	20	25	30	35	40	45	50	55	65	75	85	95
Prevalence	100.0	100.0	97.3	94.5	94.5	93.1	89.2	88.8	80.9	80.9	77.3	72.8	57.8	41.7	32.1	8.2
Lower CI	94.5	94.5	85.8	83.9	83.9	82.3	75.3	75.0	61.1	61.1	57.9	54.0	41.1	30.6	22.5	4.5
Upper CI	100.0	100.0	99.5	98.3	98.3	97.5	95.7	95.4	92.0	92.0	89.4	86.0	72.9	53.7	43.5	14.4
Number	66	66	65	64	64	63	61	60	58	58	57	55	44	28	20	10

Table W8K-46 Age Group = 18-30 Gender = MALE Occupational group = NON-MANUAL Total cases = 66

Threshold dB HL≥	0	5	10	15	20	25	30	35	40	45	50	55	65	75	85	95
Prevalence	84.1	64.0	46.6	32.3	17.6	12.0	11.2	10.0	9.7	9.7	9.7	6.3	1.6	1.6	1.4	0.0
Lower CI	70.6	50.1	34.1	20.9	9.3	5.3	4.8	3.9	3.7	3.7	3.7	1.9	0.3	0.3	0.3	0.0
Upper CI	92.1	75.9	59.5	46.2	30.6	24.9	24.3	23.1	22.9	22.9	22.9	18.7	7.5	7.5	7.8	5.5
Number	56	44	34	22	12	8	7	6	5	5	5	4	2	2	1	0

Table W8K-47 Age Group = 31-40 Gender = MALE Occupational group = NON-MANUAL Total cases = 62

Threshold dB HL≥	0	5	10	15	20	25	30	35	40	45	50	55	65	75	85	95
Prevalence	91.4	82.3	61.1	49.2	43.8	30.5	26.4	21.5	21.5	16.4	12.9	7.5	7.5	1.9	0.6	0.6
Lower CI	81.1	71.5	44.9	33.5	28.9	17.6	14.5	11.0	11.0	7.7	5.2	2.1	2.1	0.6	0.1	0.1
Upper CI	96.3	89.6	75.1	65.1	59.9	47.5	43.2	37.7	37.7	31.6	28.5	23.7	23.7	6.3	7.0	7.0
Number	56	50	41	33	28	20	17	16	16	13	9	4	4	2	1	1

Table W8K-48 Age Group = 41-50 Gender = MALE Occupational group = NON-MANUAL Total cases = 75

Threshold dB HL≥	0	5	10	15	20	25	30	35	40	45	50	55	65	75	85	95
Prevalence	100.0	93.4	90.1	82.4	70.5	49.8	44.8	41.6	38.9	28.2	26.7	24.7	11.3	4.6	2.8	2.3
Lower CI	95.1	83.5	80.1	70.1	57.4	36.7	32.3	29.5	27.0	17.8	16.5	14.9	6.3	2.1	2.0	1.5
Upper CI	100.0	97.5	95.4	90.3	80.8	62.8	58.0	54.9	52.4	41.7	40.1	38.0	19.3	9.6	4.0	3.7
Number	75	71	68	63	55	40	35	31	29	22	20	18	11	7	6	4

Table W8K-49 Age Group = 51-60 Gender = MALE Occupational group = NON-MANUAL Total cases = 137

Threshold dB HL≥	0	5	10	15	20	25	30	35	40	45	50	55	65	75	85	95
Prevalence	100.0	100.0	93.6	88.2	77.1	67.7	61.5	53.8	46.2	42.1	39.0	32.4	24.7	13.3	3.0	2.6
Lower CI	97.3	97.3	86.2	80.0	68.1	58.8	52.5	45.0	37.8	34.1	31.0	23.2	16.3	7.3	1.5	1.3
Upper CI	100.0	100.0	97.1	93.4	84.2	75.5	69.8	62.3	54.7	50.6	47.5	43.3	35.6	23.1	5.8	5.4
Number	137	137	131	125	114	102	92	82	71	64	57	53	37	21	8	7

Prevalence by age group, gender and occupational group for the worse ear over 8 kHz

Table W8K-50 Age Group = 61-70 Gender = MALE Occupational group = NON-MANUAL Total cases = 111

Threshold dB HL≥	0	5	10	15	20	25	30	35	40	45	50	55	65	75	85	95
**Prevalence**	**100.0**	**100.0**	**100.0**	**100.0**	**99.8**	**94.3**	**88.6**	**87.3**	**82.5**	**76.9**	**71.3**	**59.7**	**48.3**	**36.1**	**19.2**	**9.3**
**Lower CI**	96.7	96.7	96.7	96.7	98.9	73.6	71.0	70.1	66.3	61.7	56.1	45.8	35.2	24.0	14.0	5.7
**Upper CI**	100.0	100.0	100.0	100.0	100.0	99.0	96.1	95.3	91.9	87.3	82.9	72.3	61.6	50.3	25.7	14.8
**Number**	111	111	111	111	110	109	105	104	100	93	87	76	59	40	25	13

Table W8K-51 Age Group = 71-80 Gender = MALE Occupational group = NON-MANUAL Total cases = 55

Threshold dB HL≥	0	5	10	15	20	25	30	35	40	45	50	55	65	75	85	95
**Prevalence**	**100.0**	**100.0**	**100.0**	**100.0**	**100.0**	**97.9**	**91.5**	**89.9**	**87.8**	**84.9**	**82.0**	**76.5**	**64.6**	**39.9**	**19.5**	**12.1**
**Lower CI**	93.5	93.5	93.5	93.5	93.5	89.0	81.8	79.6	78.4	73.3	68.7	63.0	50.0	28.4	12.2	6.5
**Upper CI**	100.0	100.0	100.0	100.0	100.0	99.6	96.3	95.3	93.5	92.0	90.4	86.2	76.9	52.6	29.7	21.5
**Number**	55	55	55	55	55	54	51	50	49	48	47	44	37	23	12	7

Table W8K-52 Age Group = 18-30 Gender = FEMALE Occupational group = MANUAL Total cases = 109

Threshold dB HL≥	0	5	10	15	20	25	30	35	40	45	50	55	65	75	85	95
**Prevalence**	**89.4**	**73.7**	**51.8**	**44.6**	**33.8**	**16.1**	**12.9**	**12.3**	**9.9**	**8.3**	**6.4**	**2.5**	**1.6**	**1.3**	**0.0**	**0.0**
**Lower CI**	78.3	61.0	39.7	33.2	23.6	9.2	7.1	6.7	4.8	3.7	2.4	1.0	0.4	0.3	0.0	0.0
**Upper CI**	95.1	83.4	63.7	56.6	45.9	26.7	22.2	21.7	19.3	17.8	16.2	6.5	5.8	5.9	3.4	3.4
**Number**	99	82	65	56	41	24	21	19	14	12	8	6	3	2	0	0

Table W8K-53 Age Group = 31-40 Gender = FEMALE Occupational group = MANUAL Total cases = 103

Threshold dB HL≥	0	5	10	15	20	25	30	35	40	45	50	55	65	75	85	95
**Prevalence**	**95.5**	**93.6**	**77.4**	**62.9**	**47.2**	**27.6**	**21.0**	**17.6**	**13.4**	**8.4**	**6.9**	**4.5**	**1.9**	**1.2**	**0.9**	**0.6**
**Lower CI**	87.6	85.6	64.6	50.1	35.2	18.9	13.5	10.5	7.3	4.6	3.4	2.2	0.8	0.4	0.2	0.1
**Upper CI**	98.4	97.3	86.5	74.1	59.5	38.4	31.2	28.1	23.4	14.7	13.4	9.1	4.5	3.8	3.5	3.3
**Number**	100	97	85	69	55	43	35	26	20	17	12	9	5	3	2	1

Table W8K-54 Age Group = 41-50 Gender = FEMALE Occupational group = MANUAL Total cases = 133

Threshold dB HL≥	0	5	10	15	20	25	30	35	40	45	50	55	65	75	85	95
**Prevalence**	**98.6**	**92.6**	**78.9**	**62.6**	**52.5**	**41.0**	**35.1**	**23.1**	**16.6**	**14.8**	**9.0**	**6.6**	**4.7**	**1.4**	**0.7**	**0.5**
**Lower CI**	94.3	85.7	69.7	52.8	43.0	32.1	26.6	16.3	11.3	9.9	5.6	4.0	2.6	0.7	0.2	0.1
**Upper CI**	99.7	96.4	85.9	71.4	61.8	50.5	44.8	31.6	23.7	21.6	14.1	10.7	8.3	3.1	2.0	1.7
**Number**	131	124	109	90	77	64	54	41	33	30	22	18	13	6	3	2

Table W8K-55 Age Group = 51-60 Gender = FEMALE Occupational group = MANUAL Total cases = 166

Threshold dB HL≥	0	5	10	15	20	25	30	35	40	45	50	55	65	75	85	95
**Prevalence**	**100.0**	**98.2**	**94.5**	**90.7**	**78.8**	**70.7**	**57.8**	**49.1**	**40.0**	**32.1**	**22.6**	**18.3**	**7.0**	**5.1**	**2.9**	**1.1**
**Lower CI**	97.7	93.2	88.1	83.9	70.4	61.7	48.6	40.2	31.6	24.5	16.3	12.5	4.6	3.1	1.6	0.7
**Upper CI**	100.0	99.5	97.5	94.8	85.3	78.3	66.5	58.0	49.1	40.9	30.5	26.1	10.6	8.5	5.4	1.6
**Number**	166	164	160	153	137	123	105	94	80	71	56	46	30	22	13	7

Table W8K-56 Age Group = 61-70 Gender = FEMALE Occupational group = MANUAL Total cases = 126

Threshold dB HL≥	0	5	10	15	20	25	30	35	40	45	50	55	65	75	85	95
**Prevalence**	**100.0**	**100.0**	**100.0**	**98.1**	**87.6**	**79.3**	**74.2**	**70.2**	**60.4**	**55.3**	**48.6**	**39.0**	**34.5**	**22.0**	**13.1**	**3.7**
**Lower CI**	97.0	97.0	97.0	89.8	77.6	68.6	63.3	59.4	49.8	44.7	38.7	30.7	27.0	15.6	8.6	2.4
**Upper CI**	100.0	100.0	100.0	99.7	93.5	87.1	82.7	79.1	70.2	65.4	58.5	48.0	42.9	30.2	19.3	5.5
**Number**	126	126	126	125	116	110	103	97	88	81	74	64	58	38	22	8

Prevalence by age group, gender and occupational group for the worse ear over 8 kHz

Table W8K-57 Age Group = 71-80 Gender = FEMALE Occupational group = MANUAL Total cases = 73

Threshold dB HL≥	0	5	10	15	20	25	30	35	40	45	50	55	65	75	85	95
**Prevalence**	**100.0**	**100.0**	**100.0**	**100.0**	**100.0**	**95.4**	**95.4**	**95.4**	**95.4**	**91.1**	**85.9**	**76.4**	**56.2**	**39.6**	**24.1**	**17.5**
Lower CI	95.0	95.0	95.0	95.0	95.0	82.5	82.5	82.5	82.5	74.1	65.3	59.0	39.1	25.9	14.6	9.0
Upper CI	100.0	100.0	100.0	100.0	100.0	98.9	98.9	98.9	98.9	97.3	95.1	87.9	71.9	55.3	37.2	31.4
Number	73	73	73	73	73	71	71	71	71	70	69	67	56	45	32	20

Table W8K-58 Age Group = 18-30 Gender = MALE Occupational group = MANUAL Total cases = 109

Threshold dB HL≥	0	5	10	15	20	25	30	35	40	45	50	55	65	75	85	95
**Prevalence**	**92.3**	**79.7**	**58.4**	**47.5**	**33.9**	**22.4**	**14.1**	**8.3**	**7.6**	**2.2**	**2.0**	**1.4**	**1.3**	**1.0**	**1.0**	**1.0**
Lower CI	85.0	68.0	45.4	35.2	23.4	13.8	7.7	4.6	4.0	0.8	0.7	0.4	0.3	0.2	0.2	0.2
Upper CI	96.2	87.9	70.3	60.0	46.2	34.3	24.4	14.6	14.0	5.5	5.4	5.0	5.0	5.1	5.1	5.1
Number	99	88	71	58	45	31	21	16	13	7	6	4	3	2	2	2

Table W8K-59 Age Group = 31-40 Gender = MALE Occupational group = MANUAL Total cases = 80

Threshold dB HL≥	0	5	10	15	20	25	30	35	40	45	50	55	65	75	85	95
**Prevalence**	**94.7**	**87.9**	**74.5**	**52.2**	**31.7**	**21.1**	**18.6**	**14.7**	**14.4**	**8.0**	**5.5**	**5.5**	**2.9**	**2.9**	**2.7**	**2.3**
Lower CI	82.3	74.3	58.8	37.3	20.0	11.9	9.8	7.5	7.3	4.5	2.5	2.5	0.8	0.8	0.7	0.5
Upper CI	98.6	94.8	85.7	66.8	46.4	34.6	32.5	26.8	26.5	13.8	11.6	11.6	9.7	9.7	9.8	9.9
Number	77	70	62	49	36	26	21	18	17	14	9	9	5	5	4	2

Table W8K-60 Age Group = 41-50 Gender = MALE Occupational group = MANUAL Total cases = 123

Threshold dB HL≥	0	5	10	15	20	25	30	35	40	45	50	55	65	75	85	95
**Prevalence**	**98.2**	**92.6**	**91.6**	**78.5**	**65.6**	**53.8**	**47.0**	**41.9**	**36.8**	**30.1**	**25.5**	**23.2**	**10.5**	**7.9**	**3.9**	**0.9**
Lower CI	90.3	82.8	81.9	67.1	54.9	43.1	36.5	31.9	27.3	21.4	17.6	15.6	6.4	4.2	1.6	0.4
Upper CI	99.7	97.0	96.3	86.7	74.9	64.1	57.7	52.6	47.4	40.5	35.5	33.1	16.9	14.3	9.2	2.4
Number	122	118	116	105	95	82	72	64	58	47	41	37	22	15	8	4

Table W8K-61 Age Group = 51-60 Gender = MALE Occupational group = MANUAL Total cases = 196

Threshold dB HL≥	0	5	10	15	20	25	30	35	40	45	50	55	65	75	85	95
**Prevalence**	**100.0**	**99.3**	**98.4**	**91.1**	**88.1**	**79.3**	**73.7**	**65.4**	**60.0**	**53.2**	**45.7**	**42.1**	**26.9**	**18.3**	**11.7**	**6.0**
Lower CI	98.1	95.9	94.4	84.5	81.1	71.0	65.3	56.8	51.4	44.8	37.6	34.2	20.7	13.4	8.0	3.4
Upper CI	100.0	99.9	99.6	95.0	92.7	85.6	80.7	73.0	68.1	61.4	54.0	50.4	34.2	24.7	16.8	10.5
Number	196	195	194	186	181	169	160	147	137	125	108	100	73	51	33	17

Table W8K-62 Age Group = 61-70 Gender = MALE Occupational group = MANUAL Total cases = 172

Threshold dB HL≥	0	5	10	15	20	25	30	35	40	45	50	55	65	75	85	95
**Prevalence**	**100.0**	**100.0**	**100.0**	**100.0**	**97.5**	**95.1**	**90.8**	**88.7**	**83.7**	**81.4**	**79.1**	**75.9**	**62.3**	**35.2**	**22.2**	**8.5**
Lower CI	97.8	97.8	97.8	97.8	91.9	89.5	81.5	79.6	73.0	70.9	69.0	66.1	52.1	26.3	15.2	5.8
Upper CI	100.0	100.0	100.0	100.0	99.3	97.8	95.7	94.0	90.7	88.7	86.6	83.5	71.5	45.2	31.2	12.3
Number	172	172	172	172	170	168	165	162	157	152	148	142	123	85	56	31

Table W8K-63 Age Group = 71-80 Gender = MALE Occupational group = MANUAL Total cases = 71

Threshold dB HL≥	0	5	10	15	20	25	30	35	40	45	50	55	65	75	85	95
**Prevalence**	**100.0**	**100.0**	**100.0**	**100.0**	**100.0**	**100.0**	**100.0**	**100.0**	**100.0**	**97.5**	**96.7**	**95.7**	**68.9**	**57.9**	**46.7**	**32.5**
Lower CI	94.9	94.9	94.9	94.9	94.9	94.9	94.9	94.9	94.9	86.9	87.0	86.7	52.1	41.5	31.4	19.5
Upper CI	100.0	100.0	100.0	100.0	100.0	100.0	100.0	100.0	100.0	99.6	99.2	98.7	81.8	72.8	62.6	48.8
Number	71	71	71	71	71	71	71	71	71	70	69	67	60	51	41	27

Prevalence by age group, gender and occupational group for the better ear over 0.5, 1, 2, and 4 kHz

Table B5124-1 Age Group = 18-40 Gender = OVERALL Occupational group = OVERALL Total cases = 732

	Threshold dB HL≥															
	**0**	**5**	**10**	**15**	**20**	**25**	**30**	**35**	**40**	**45**	**50**	**55**	**65**	**75**	**85**	**95**
**Prevalence**	**86.5**	**49.8**	**21.8**	**9.5**	**3.7**	**2.1**	**1.3**	**0.7**	**0.6**	**0.5**	**0.4**	**0.3**	**0.3**	**0.2**	**0.2**	**0.2**
**Lower CI**	83.0	45.5	18.6	7.5	2.6	1.4	0.8	0.4	0.3	0.2	0.1	0.1	0.1	0.1	0.0	0.0
**Upper CI**	89.4	54.0	25.4	12.1	5.3	3.2	2.1	1.4	1.3	1.2	1.1	1.1	1.1	1.1	1.1	1.1
**Number**	655	415	211	103	49	34	24	16	13	11	7	5	3	2	1	1

Table B5124-2 Age Group = 41-60 Gender = OVERALL Occupational group = OVERALL Total cases = 1132

	Threshold dB HL≥															
	**0**	**5**	**10**	**15**	**20**	**25**	**30**	**35**	**40**	**45**	**50**	**55**	**65**	**75**	**85**	**95**
**Prevalence**	**98.1**	**83.6**	**59.8**	**35.2**	**22.0**	**14.1**	**8.8**	**6.1**	**4.1**	**3.0**	**2.2**	**1.5**	**0.7**	**0.3**	**0.1**	**0.1**
**Lower CI**	97.0	81.0	56.6	32.5	19.9	12.4	7.5	5.1	3.3	2.3	1.7	1.1	0.4	0.1	0.0	0.0
**Upper CI**	98.8	85.9	62.8	38.1	24.3	16.0	10.3	7.3	5.1	3.8	2.8	2.1	1.0	0.7	0.3	0.2
**Number**	1114	981	756	511	364	258	177	136	100	74	58	40	21	8	4	3

Table B5124-3 Age Group = 61-80 Gender = OVERALL Occupational group = OVERALL Total cases = 799

	Threshold dB HL≥															
	**0**	**5**	**10**	**15**	**20**	**25**	**30**	**35**	**40**	**45**	**50**	**55**	**65**	**75**	**85**	**95**
**Prevalence**	**99.9**	**98.5**	**89.8**	**73.6**	**59.8**	**45.8**	**33.7**	**25.5**	**18.1**	**11.4**	**8.3**	**6.1**	**3.0**	**1.9**	**1.0**	**0.4**
**Lower CI**	99.2	97.0	86.9	69.7	55.7	42.0	30.4	22.4	15.5	9.8	7.1	5.1	2.3	1.3	0.6	0.2
**Upper CI**	100.0	99.2	92.1	77.2	63.8	49.8	37.2	28.8	21.0	13.1	9.7	7.3	3.8	2.6	1.6	1.0
**Number**	798	789	734	635	549	462	373	298	228	170	135	104	54	33	17	6

Table B5124-4 Age Group = 18-40 Gender = FEMALE Occupational group = OVERALL Total cases = 413

	Threshold dB HL≥															
	**0**	**5**	**10**	**15**	**20**	**25**	**30**	**35**	**40**	**45**	**50**	**55**	**65**	**75**	**85**	**95**
**Prevalence**	**88.3**	**48.5**	**18.6**	**7.6**	**3.3**	**2.8**	**1.5**	**0.8**	**0.6**	**0.5**	**0.3**	**0.2**	**0.1**	**0.1**	**0.0**	**0.0**
**Lower CI**	84.2	43.0	15.1	5.6	2.2	1.8	0.9	0.4	0.3	0.2	0.1	0.0	0.0	0.0	0.0	0.0
**Upper CI**	91.5	54.0	22.8	10.4	4.9	4.4	2.5	1.5	1.2	1.1	0.9	0.6	0.5	0.5	0.9	0.9
**Number**	372	227	109	54	29	24	15	10	8	6	4	2	1	1	0	0

Table B5124-5 Age Group = 41-60 Gender = FEMALE Occupational group = OVERALL Total cases = 588

	Threshold dB HL≥															
	**0**	**5**	**10**	**15**	**20**	**25**	**30**	**35**	**40**	**45**	**50**	**55**	**65**	**75**	**85**	**95**
**Prevalence**	**97.6**	**82.1**	**54.3**	**28.5**	**15.8**	**10.5**	**6.9**	**4.6**	**3.0**	**2.1**	**1.8**	**1.2**	**0.7**	**0.3**	**0.1**	**0.1**
**Lower CI**	95.7	78.4	49.9	25.0	13.3	8.5	5.3	3.5	2.2	1.5	1.2	0.7	0.4	0.1	0.0	0.0
**Upper CI**	98.7	85.2	58.6	32.3	18.6	12.9	8.9	6.1	4.2	3.1	2.7	2.1	1.3	0.9	0.4	0.4
**Number**	577	499	361	223	146	108	74	58	43	31	26	18	12	5	3	3

Table B5124-6 Age Group = 61-80 Gender = FEMALE Occupational group = OVERALL Total cases = 385

	Threshold dB HL≥															
	**0**	**5**	**10**	**15**	**20**	**25**	**30**	**35**	**40**	**45**	**50**	**55**	**65**	**75**	**85**	**95**
**Prevalence**	**99.8**	**97.7**	**86.7**	**69.9**	**54.9**	**40.3**	**30.4**	**24.0**	**18.0**	**11.2**	**7.8**	**5.8**	**3.1**	**2.0**	**1.3**	**0.5**
**Lower CI**	98.7	95.1	82.0	64.3	49.0	35.1	26.2	19.7	14.2	9.0	6.3	4.6	2.2	1.3	0.7	0.2
**Upper CI**	100.0	98.9	90.3	74.9	60.6	45.8	35.0	29.0	22.4	13.8	9.6	7.3	4.3	3.2	2.3	1.5
**Number**	384	378	342	289	243	201	169	140	111	86	68	55	30	18	10	3

Table B5124-7 Age Group = 18-40 Gender = MALE Occupational group = OVERALL Total cases = 319

	Threshold dB HL≥															
	**0**	**5**	**10**	**15**	**20**	**25**	**30**	**35**	**40**	**45**	**50**	**55**	**65**	**75**	**85**	**95**
**Prevalence**	**84.6**	**51.0**	**25.2**	**11.6**	**4.0**	**1.3**	**1.0**	**0.7**	**0.5**	**0.5**	**0.5**	**0.5**	**0.4**	**0.4**	**0.4**	**0.4**
**Lower CI**	78.5	44.5	19.9	8.1	2.3	0.6	0.4	0.2	0.1	0.1	0.1	0.1	0.1	0.1	0.1	0.1
**Upper CI**	89.1	57.5	31.4	16.3	7.1	3.1	2.5	2.2	2.2	2.2	2.2	2.2	2.3	2.3	2.3	2.3
**Number**	283	188	102	49	20	10	9	6	5	5	3	3	2	1	1	1

Prevalence by age group, gender and occupational group for the better ear over 0.5, 1, 2, and 4 kHz

Table B5124-8 Age Group = 41-60 Gender = MALE Occupational group = OVERALL Total cases = 544

	Threshold dB HL≥															
	0	5	10	15	20	25	30	35	40	45	50	55	65	75	85	95
Prevalence	98.7	85.3	65.8	42.7	28.9	18.1	10.9	7.8	5.3	3.9	2.6	1.8	0.6	0.3	0.1	0.0
Lower CI	97.2	81.4	61.2	38.4	25.4	15.4	8.9	6.2	4.0	2.8	1.8	1.1	0.3	0.1	0.0	0.0
Upper CI	99.4	88.4	70.2	47.1	32.7	21.2	13.3	9.8	7.0	5.4	3.7	2.9	1.2	0.9	0.5	0.7
Number	537	482	395	288	218	150	103	78	57	43	32	22	9	3	1	0

Table B5124-9 Age Group = 61-80 Gender = MALE Occupational group = OVERALL Total cases = 414

	Threshold dB HL≥															
	0	5	10	15	20	25	30	35	40	45	50	55	65	75	85	95
Prevalence	100.0	99.5	93.8	78.4	65.9	52.8	37.9	27.3	18.4	11.7	9.2	6.6	2.9	1.7	0.7	0.3
Lower CI	99.1	98.3	90.6	72.7	60.3	47.2	32.9	23.3	15.3	9.7	7.4	5.0	2.0	1.0	0.3	0.1
Upper CI	100.0	99.8	95.9	83.1	71.0	58.3	43.1	31.7	21.9	14.2	11.4	8.6	4.2	2.7	1.4	1.0
Number	414	411	392	346	306	261	204	158	117	84	67	49	24	15	7	3

Table B5124-10 Age Group = 18-40 Gender = OVERALL Occupational group = NON-MANUAL Total cases = 329

	Threshold dB HL≥															
	0	5	10	15	20	25	30	35	40	45	50	55	65	75	85	95
Prevalence	83.2	44.0	14.7	5.7	2.3	1.4	0.8	0.5	0.2	0.1	0.1	0.0	0.0	0.0	0.0	0.0
Lower CI	77.7	37.9	10.9	3.4	1.3	0.7	0.3	0.1	0.1	0.0	0.0	0.0	0.0	0.0	0.0	0.0
Upper CI	87.5	50.2	19.7	9.5	4.3	2.6	2.0	1.5	0.6	0.6	0.6	1.2	1.2	1.2	1.2	1.2
Number	285	161	62	26	14	10	6	4	3	2	2	1	1	0	0	0

Table B5124-11 Age Group = 41-60 Gender = OVERALL Occupational group = NON-MANUAL Total cases = 505

	Threshold dB HL≥															
	0	5	10	15	20	25	30	35	40	45	50	55	65	75	85	95
Prevalence	97.9	78.6	51.1	27.5	17.3	9.1	5.2	3.5	2.0	1.5	1.2	0.7	0.4	0.2	0.0	0.0
Lower CI	96.2	74.5	46.5	23.9	14.5	6.8	4.0	2.8	1.4	1.0	0.8	0.4	0.1	0.1	0.0	0.0
Upper CI	98.8	82.2	55.6	31.5	20.5	11.9	6.9	4.4	2.7	2.1	1.8	1.3	0.9	0.7	0.2	0.2
Number	493	404	284	174	117	77	53	41	26	20	17	10	5	3	1	1

Table B5124-12 Age Group = 61-80 Gender = OVERALL Occupational group = NON-MANUAL Total cases = 350

	Threshold dB HL≥															
	0	5	10	15	20	25	30	35	40	45	50	55	65	75	85	95
Prevalence	99.7	97.8	88.3	69.4	57.7	46.4	33.8	25.0	19.5	9.4	6.7	4.1	2.6	1.5	0.5	0.2
Lower CI	98.1	95.1	84.2	62.8	51.4	41.0	30.1	21.6	16.7	7.8	5.3	3.0	1.7	0.8	0.2	0.1
Upper CI	99.9	99.0	91.4	75.2	63.8	51.8	37.7	28.7	22.7	11.3	8.4	5.7	4.1	2.8	1.4	1.0
Number	349	343	310	256	218	183	147	110	86	60	47	34	20	11	5	2

Table B5124-13 Age Group = 18-40 Gender = OVERALL Occupational group = MANUAL Total cases = 401

	Threshold dB HL≥															
	0	5	10	15	20	25	30	35	40	45	50	55	65	75	85	95
Prevalence	89.0	56.8	27.8	12.7	5.0	2.9	1.6	0.9	0.8	0.7	0.5	0.5	0.4	0.4	0.3	0.3
Lower CI	83.8	50.3	22.6	9.5	3.1	1.7	0.9	0.4	0.3	0.3	0.1	0.1	0.1	0.1	0.1	0.1
Upper CI	92.7	63.1	33.7	16.9	8.0	4.9	3.0	2.0	1.9	1.8	1.7	1.7	1.8	1.8	1.8	1.8
Number	369	254	149	77	35	24	18	12	10	9	5	4	2	2	1	1

Table B5124-14 Age Group = 41-60 Gender = OVERALL Occupational group = MANUAL Total cases = 627

	Threshold dB HL≥															
	0	5	10	15	20	25	30	35	40	45	50	55	65	75	85	95
Prevalence	98.7	89.1	68.8	41.6	26.7	18.3	11.2	7.7	5.1	3.6	2.5	1.8	0.7	0.3	0.1	0.1
Lower CI	97.1	85.6	64.3	37.4	23.3	15.5	9.2	6.2	4.0	2.7	1.8	1.3	0.4	0.1	0.0	0.0
Upper CI	99.4	91.9	73.0	46.0	30.4	21.5	13.6	9.5	6.4	4.9	3.4	2.6	1.2	0.6	0.4	0.3
Number	621	577	472	337	247	181	124	95	74	54	41	30	16	5	3	2

Prevalence by age group, gender and occupational group for the better ear over 0.5, 1, 2, and 4 kHz

Table B5124-15 Age Group = 61-80 Gender = OVERALL Occupational group = MANUAL Total cases = 447

Threshold dB HL≥	0	5	10	15	20	25	30	35	40	45	50	55	65	75	85	95
Prevalence	100.0	98.8	91.7	77.7	62.2	45.8	34.6	25.6	18.0	12.9	9.3	7.1	3.4	2.4	1.5	0.8
Lower CI	99.1	96.2	87.4	72.3	56.5	40.4	30.3	21.8	15.0	10.1	7.7	5.8	2.6	1.7	1.0	0.5
Upper CI	100.0	99.7	94.6	82.3	67.6	51.2	39.1	29.9	21.5	16.3	11.1	8.7	4.4	3.3	2.3	1.1
Number	447	444	422	377	329	278	226	188	142	110	88	70	34	22	12	4

Table B5124-16 Age Group = 18-40 Gender = FEMALE Occupational group = NON-MANUAL Total cases = 201

Threshold dB HL≥	0	5	10	15	20	25	30	35	40	45	50	55	65	75	85	95
Prevalence	85.8	42.9	14.4	5.1	2.6	2.0	1.0	0.8	0.3	0.2	0.2	0.0	0.0	0.0	0.0	0.0
Lower CI	80.3	35.9	10.3	3.0	1.5	1.0	0.3	0.2	0.1	0.0	0.0	0.0	0.0	0.0	0.0	0.0
Upper CI	90.0	50.3	19.9	8.6	4.6	4.1	2.9	2.8	1.1	1.2	1.2	1.9	1.9	1.9	1.9	1.9
Number	174	94	37	15	10	8	4	3	2	1	1	0	0	0	0	0

Table B5124-17 Age Group = 41-60 Gender = FEMALE Occupational group = NON-MANUAL Total cases = 287

Threshold dB HL≥	0	5	10	15	20	25	30	35	40	45	50	55	65	75	85	95
Prevalence	97.7	78.6	49.3	24.5	11.9	6.8	4.1	3.2	2.3	1.7	1.5	0.9	0.7	0.4	0.1	0.1
Lower CI	94.9	73.3	43.4	20.2	9.3	5.0	3.0	2.4	1.5	1.1	0.9	0.4	0.3	0.1	0.0	0.0
Upper CI	99.0	83.1	55.1	29.4	15.1	9.1	5.7	4.2	3.3	2.5	2.5	2.1	1.7	1.4	0.4	0.4
Number	281	231	157	92	54	38	25	20	15	12	11	6	5	3	1	1

Table B5124-18 Age Group = 61-80 Gender = FEMALE Occupational group = NON-MANUAL Total cases = 182

Threshold dB HL≥	0	5	10	15	20	25	30	35	40	45	50	55	65	75	85	95
Prevalence	99.4	96.3	86.4	68.1	55.9	44.1	34.1	25.6	21.4	8.1	6.1	4.9	3.3	1.7	0.8	0.4
Lower CI	96.7	91.7	80.1	59.4	47.4	37.6	29.1	21.5	17.8	6.3	4.5	3.4	2.0	0.8	0.3	0.1
Upper CI	99.9	98.4	91.0	75.7	64.2	50.9	39.5	30.3	25.4	10.4	8.1	7.0	5.4	3.4	2.2	1.8
Number	181	177	159	130	110	94	79	63	52	37	31	25	15	8	4	2

Table B5124-19 Age Group = 18-40 Gender = MALE Occupational group = NON-MANUAL Total cases = 128

Threshold dB HL≥	0	5	10	15	20	25	30	35	40	45	50	55	65	75	85	95
Prevalence	80.2	44.9	15.0	6.4	1.9	0.6	0.6	0.1	0.1	0.1	0.1	0.1	0.1	0.0	0.0	0.0
Lower CI	70.2	35.2	8.9	2.8	0.6	0.1	0.1	0.0	0.0	0.0	0.0	0.0	0.0	0.0	0.0	0.0
Upper CI	87.5	55.1	24.1	14.1	6.2	2.6	2.6	3.1	3.1	3.1	3.1	3.1	3.1	2.9	2.9	2.9
Number	111	67	25	11	4	2	2	1	1	1	1	1	1	0	0	0

Table B5124-20 Age Group = 41-60 Gender = MALE Occupational group = NON-MANUAL Total cases = 218

Threshold dB HL≥	0	5	10	15	20	25	30	35	40	45	50	55	65	75	85	95
Prevalence	98.0	78.6	53.1	30.8	23.4	11.6	6.5	3.9	1.6	1.2	0.8	0.4	0.0	0.0	0.0	0.0
Lower CI	95.5	72.0	46.1	25.0	18.3	7.6	4.3	2.8	0.9	0.6	0.4	0.2	0.0	0.0	0.0	0.0
Upper CI	99.2	84.0	60.0	37.3	29.3	17.3	9.7	5.4	2.8	2.4	1.8	1.2	1.7	1.7	1.7	1.7
Number	212	173	127	82	63	39	28	21	11	8	6	4	0	0	0	0

Table B5124-21 Age Group = 61-80 Gender = MALE Occupational group = NON-MANUAL Total cases = 168

Threshold dB HL≥	0	5	10	15	20	25	30	35	40	45	50	55	65	75	85	95
Prevalence	100.0	99.7	90.5	71.1	60.2	49.3	33.4	24.5	17.7	11.3	7.5	3.2	1.9	1.4	0.2	0.0
Lower CI	97.8	99.2	85.1	60.9	50.7	40.2	28.3	19.4	13.8	8.7	5.3	1.6	0.7	0.4	0.0	0.0
Upper CI	100.0	99.9	94.1	79.6	68.9	58.3	38.9	30.4	22.5	14.5	10.6	6.0	4.8	4.4	1.4	2.2
Number	168	166	151	126	108	89	68	47	34	23	16	9	5	3	1	0

Prevalence by age group, gender and occupational group for the better ear over 0.5, 1, 2, and 4 kHz

Table B5124-22 Age Group = 18-40 Gender = FEMALE Occupational group = MANUAL Total cases = 212

	Threshold dB HL≥															
	0	5	10	15	20	25	30	35	40	45	50	55	65	75	85	95
**Prevalence**	**90.7**	**55.8**	**23.1**	**10.0**	**4.0**	**3.7**	**1.9**	**0.9**	**0.7**	**0.6**	**0.3**	**0.2**	**0.1**	**0.1**	**0.0**	**0.0**
**Lower CI**	83.5	46.7	17.0	6.7	2.2	1.9	0.9	0.4	0.3	0.3	0.1	0.1	0.0	0.0	0.0	0.0
**Upper CI**	94.9	64.5	30.6	14.6	7.2	6.8	3.9	1.9	1.5	1.4	0.9	0.9	0.7	0.7	1.8	1.8
**Number**	198	133	72	39	19	16	11	7	6	5	3	2	1	1	0	0

Table B5124-23 Age Group = 41-60 Gender = FEMALE Occupational group = MANUAL Total cases = 301

	Threshold dB HL≥															
	0	5	10	15	20	25	30	35	40	45	50	55	65	75	85	95
**Prevalence**	**98.1**	**87.3**	**61.7**	**32.5**	**20.0**	**14.6**	**9.6**	**6.1**	**3.2**	**2.1**	**1.7**	**1.2**	**0.5**	**0.2**	**0.2**	**0.2**
**Lower CI**	95.6	82.2	55.0	26.9	15.8	11.0	6.9	4.3	2.3	1.3	0.9	0.7	0.3	0.0	0.0	0.0
**Upper CI**	99.2	91.1	68.0	38.5	25.1	19.1	13.2	8.5	4.6	3.5	3.0	2.3	1.1	0.6	0.6	0.6
**Number**	296	268	204	131	92	70	49	38	28	19	15	12	7	2	2	2

Table B5124-24 Age Group = 61-80 Gender = FEMALE Occupational group = MANUAL Total cases = 202

	Threshold dB HL≥															
	0	5	10	15	20	25	30	35	40	45	50	55	65	75	85	95
**Prevalence**	**100.0**	**98.6**	**87.4**	**71.7**	**54.0**	**37.5**	**29.5**	**22.8**	**17.2**	**13.0**	**8.4**	**6.3**	**3.4**	**2.7**	**2.0**	**1.0**
**Lower CI**	98.1	94.0	80.4	63.6	46.4	31.0	24.9	17.9	12.9	9.0	6.5	5.0	2.4	1.8	1.2	0.3
**Upper CI**	100.0	99.7	92.1	78.6	61.4	44.6	34.6	28.6	22.4	18.4	10.8	8.0	4.7	4.0	3.2	3.5
**Number**	202	200	182	158	132	107	90	77	59	49	37	30	15	10	6	1

Table B5124-25 Age Group = 18-40 Gender = MALE Occupational group = MANUAL Total cases = 189

	Threshold dB HL≥															
	0	5	10	15	20	25	30	35	40	45	50	55	65	75	85	95
**Prevalence**	**87.3**	**58.0**	**32.8**	**15.6**	**6.0**	**1.9**	**1.3**	**1.0**	**0.9**	**0.9**	**0.7**	**0.7**	**0.7**	**0.7**	**0.7**	**0.7**
**Lower CI**	79.1	48.6	24.6	10.3	3.0	0.8	0.5	0.3	0.2	0.2	0.1	0.1	0.1	0.1	0.1	0.1
**Upper CI**	92.6	66.7	42.3	23.1	11.6	4.8	3.7	3.5	3.5	3.5	3.6	3.6	3.7	3.7	3.7	3.7
**Number**	171	121	77	38	16	8	7	5	4	4	2	2	1	1	1	1

Table B5124-26 Age Group = 41-60 Gender = MALE Occupational group = MANUAL Total cases = 326

	Threshold dB HL≥															
	0	5	10	15	20	25	30	35	40	45	50	55	65	75	85	95
**Prevalence**	**99.3**	**91.2**	**76.5**	**51.6**	**34.1**	**22.5**	**13.0**	**9.5**	**7.1**	**5.2**	**3.4**	**2.5**	**0.9**	**0.4**	**0.1**	**0.0**
**Lower CI**	96.4	86.0	70.3	45.3	28.9	18.4	10.2	7.2	5.2	3.6	2.4	1.6	0.5	0.1	0.0	0.0
**Upper CI**	99.9	94.6	81.7	57.9	39.6	27.2	16.3	12.4	9.7	7.6	4.9	3.9	1.9	1.1	0.6	1.2
**Number**	325	309	268	206	155	111	75	57	46	35	26	18	9	3	1	0

Table B5124-27 Age Group = 61-80 Gender = MALE Occupational group = MANUAL Total cases = 245

	Threshold dB HL≥															
	0	5	10	15	20	25	30	35	40	45	50	55	65	75	85	95
**Prevalence**	**100.0**	**99.2**	**97.0**	**85.4**	**72.5**	**56.2**	**41.0**	**29.3**	**19.0**	**12.8**	**10.5**	**8.2**	**3.4**	**2.0**	**0.9**	**0.6**
**Lower CI**	98.5	95.6	92.6	78.3	63.4	47.5	33.5	23.7	15.3	9.9	8.0	6.0	2.3	1.2	0.4	0.2
**Upper CI**	100.0	99.9	98.8	90.5	80.0	64.5	49.0	35.6	23.3	16.4	13.7	11.3	5.1	3.5	2.0	1.7
**Number**	245	244	240	219	197	171	136	111	83	61	51	40	19	12	6	3

Prevalence by age group, gender and occupational group for the better ear over 0.5, 1 and 2 kHz

Table B512-8 Age Group = 41-60 Gender = MALE Occupational group = OVERALL Total cases = 544

	Threshold dB HL≥															
	0	5	10	15	20	25	30	35	40	45	50	55	65	75	85	95
**Prevalence**	**95.8**	**74.0**	**46.4**	**29.2**	**16.1**	**10.3**	**6.9**	**4.9**	**3.6**	**2.5**	**1.7**	**1.4**	**0.5**	**0.2**	**0.1**	**0.0**
**Lower CI**	93.3	69.6	41.9	25.5	13.4	8.3	5.3	3.6	2.5	1.7	1.1	0.9	0.2	0.1	0.0	0.0
**Upper CI**	97.4	78.0	50.9	33.2	19.3	12.8	9.0	6.5	5.1	3.5	2.6	2.3	1.1	0.7	0.5	0.7
**Number**	524	427	297	204	133	94	66	52	39	31	21	18	7	2	1	0

Table B512-9 Age Group = 61-80 Gender = MALE Occupational group = OVERALL Total cases = 414

	Threshold dB HL≥															
	0	5	10	15	20	25	30	35	40	45	50	55	65	75	85	95
**Prevalence**	**99.6**	**93.1**	**77.2**	**59.7**	**46.2**	**31.7**	**20.4**	**13.9**	**10.3**	**8.3**	**5.7**	**4.4**	**2.1**	**1.2**	**0.5**	**0.3**
**Lower CI**	98.4	89.2	71.5	54.0	40.8	27.1	17.1	11.4	8.3	6.5	4.3	3.1	1.4	0.7	0.2	0.1
**Upper CI**	99.9	95.6	82.0	65.1	51.7	36.6	24.1	16.8	12.7	10.5	7.6	6.1	3.3	2.1	1.2	1.0
**Number**	412	390	339	277	226	171	125	92	72	60	43	33	19	11	5	3

Table B512-10 Age Group = 18-40 Gender = OVERALL Occupational group = NON-MANUAL Total cases = 329

	Threshold dB HL≥															
	0	5	10	15	20	25	30	35	40	45	50	55	65	75	85	95
**Prevalence**	**79.4**	**37.8**	**13.4**	**5.5**	**2.2**	**1.3**	**0.6**	**0.5**	**0.4**	**0.1**	**0.1**	**0.0**	**0.0**	**0.0**	**0.0**	**0.0**
**Lower CI**	73.3	32.2	9.9	3.2	1.2	0.6	0.2	0.1	0.1	0.0	0.0	0.0	0.0	0.0	0.0	0.0
**Upper CI**	84.5	43.8	17.9	9.3	4.1	2.6	1.6	1.5	1.5	0.6	0.6	1.2	1.2	1.2	1.2	1.2
**Number**	276	143	59	22	13	10	5	4	3	2	2	1	0	0	0	0

Table B512-11 Age Group = 41-60 Gender = OVERALL Occupational group = NON-MANUAL Total cases = 505

	Threshold dB HL≥															
	0	5	10	15	20	25	30	35	40	45	50	55	65	75	85	95
**Prevalence**	**94.7**	**70.7**	**36.9**	**21.6**	**10.4**	**5.2**	**3.7**	**2.4**	**1.8**	**1.1**	**0.7**	**0.5**	**0.3**	**0.2**	**0.0**	**0.0**
**Lower CI**	91.8	66.3	32.7	18.4	8.4	3.9	2.5	1.7	1.3	0.7	0.4	0.2	0.1	0.1	0.0	0.0
**Upper CI**	96.6	74.8	41.4	25.3	12.7	7.0	5.3	3.4	2.6	1.8	1.2	1.0	0.8	0.4	0.2	0.2
**Number**	479	366	217	141	88	51	35	30	24	16	11	8	4	3	1	1

Table B512-12 Age Group = 61-80 Gender = OVERALL Occupational group = NON-MANUAL Total cases = 350

	Threshold dB HL≥															
	0	5	10	15	20	25	30	35	40	45	50	55	65	75	85	95
**Prevalence**	**98.8**	**93.7**	**76.3**	**56.6**	**44.2**	**33.5**	**23.5**	**18.0**	**10.3**	**6.1**	**4.5**	**3.3**	**2.4**	**1.4**	**0.4**	**0.2**
**Lower CI**	96.6	90.2	70.6	50.2	38.7	28.7	20.2	15.2	8.6	4.7	3.3	2.2	1.5	0.7	0.1	0.1
**Upper CI**	99.6	96.0	81.1	62.8	49.8	38.7	27.2	21.3	12.3	8.0	6.0	4.8	3.9	2.9	1.2	1.0
**Number**	346	327	273	214	172	134	101	77	58	46	37	27	19	9	4	2

Table B512-13 Age Group = 18-40 Gender = OVERALL Occupational group = MANUAL Total cases = 401

	Threshold dB HL≥															
	0	5	10	15	20	25	30	35	40	45	50	55	65	75	85	95
**Prevalence**	**83.1**	**48.6**	**23.2**	**9.3**	**4.0**	**3.1**	**1.5**	**0.8**	**0.8**	**0.6**	**0.5**	**0.5**	**0.4**	**0.4**	**0.3**	**0.3**
**Lower CI**	77.8	42.3	18.5	6.5	2.4	1.8	0.7	0.4	0.3	0.2	0.1	0.1	0.1	0.1	0.1	0.1
**Upper CI**	87.3	55.0	28.7	13.0	6.5	5.4	3.0	2.0	1.9	1.8	1.7	1.7	1.8	1.8	1.8	1.8
**Number**	352	226	127	61	30	24	14	10	9	5	4	4	2	2	1	1

Table B512-14 Age Group = 41-60 Gender = OVERALL Occupational group = MANUAL Total cases = 627

	Threshold dB HL≥															
	0	5	10	15	20	25	30	35	40	45	50	55	65	75	85	95
**Prevalence**	**96.6**	**78.3**	**52.1**	**31.2**	**18.8**	**12.9**	**8.7**	**6.5**	**4.3**	**2.6**	**1.7**	**1.2**	**0.5**	**0.2**	**0.1**	**0.1**
**Lower CI**	94.2	74.0	47.5	27.4	15.8	10.5	6.9	5.0	3.2	1.9	1.2	0.8	0.3	0.1	0.0	0.0
**Upper CI**	98.1	82.0	56.7	35.3	22.1	15.7	11.0	8.3	5.8	3.5	2.4	1.8	1.0	0.5	0.4	0.3
**Number**	614	523	383	259	177	128	97	79	56	42	30	24	10	4	3	2

Prevalence by age group, gender and occupational group for the better ear over 0.5, 1 and 2 kHz

Table B512-1 Age Group = 18-40 Gender = OVERALL Occupational group = OVERALL Total cases = 733

Threshold dB HL≥	0	5	10	15	20	25	30	35	40	45	50	55	65	75	85	95
Prevalence	81.2	43.3	19.0	7.6	3.1	2.2	1.0	0.7	0.6	0.4	0.4	0.3	0.2	0.2	0.2	0.2
Lower CI	77.3	39.1	15.9	5.7	2.1	1.4	0.6	0.3	0.3	0.2	0.1	0.1	0.1	0.1	0.0	0.0
Upper CI	84.5	47.5	22.4	9.9	4.5	3.3	1.9	1.4	1.3	1.2	1.1	1.1	1.1	1.1	1.1	1.1
Number	629	370	187	84	43	34	19	14	12	7	6	5	2	2	1	1

Table B512-2 Age Group = 41-60 Gender = OVERALL Occupational group = OVERALL Total cases = 1132

Threshold dB HL≥	0	5	10	15	20	25	30	35	40	45	50	55	65	75	85	95
Prevalence	95.7	74.3	45.1	26.6	15.2	9.4	6.5	5.0	3.4	2.2	1.4	1.1	0.5	0.2	0.1	0.1
Lower CI	94.1	71.4	42.0	24.1	13.4	8.0	5.4	4.0	2.7	1.7	1.1	0.8	0.3	0.1	0.0	0.0
Upper CI	96.9	77.0	48.2	29.3	17.2	11.0	7.9	6.1	4.3	2.9	2.0	1.5	0.8	0.4	0.3	0.2
Number	1093	889	600	400	265	179	132	109	80	58	41	32	14	7	4	3

Table B512-3 Age Group = 61-80 Gender = OVERALL Occupational group = OVERALL Total cases = 799

Threshold dB HL≥	0	5	10	15	20	25	30	35	40	45	50	55	65	75	85	95
Prevalence	99.3	93.9	77.6	60.5	44.6	31.6	22.8	16.1	11.8	8.5	6.2	4.6	2.6	1.7	0.9	0.5
Lower CI	98.1	91.6	73.9	56.5	40.8	28.3	19.8	13.7	10.2	7.1	5.2	3.7	2.0	1.1	0.5	0.2
Upper CI	99.7	95.7	80.9	64.4	48.5	35.0	26.0	18.9	13.7	10.2	7.4	5.7	3.4	2.4	1.5	1.0
Number	794	756	648	538	430	338	264	204	168	132	105	78	47	28	15	7

Table B512-4 Age Group = 18-40 Gender = FEMALE Occupational group = OVERALL Total cases = 414

Threshold dB HL≥	0	5	10	15	20	25	30	35	40	45	50	55	65	75	85	95
Prevalence	85.1	44.9	16.7	7.2	3.2	2.6	1.4	0.8	0.8	0.4	0.3	0.2	0.1	0.1	0.0	0.0
Lower CI	80.6	39.6	13.4	5.2	2.1	1.6	0.8	0.4	0.4	0.2	0.1	0.0	0.0	0.0	0.0	0.0
Upper CI	88.7	50.4	20.6	10.0	4.9	4.2	2.6	1.5	1.5	1.0	0.8	0.6	0.5	0.5	0.9	0.9
Number	364	214	101	50	28	22	14	10	9	4	3	2	1	1	0	0

Table B512-5 Age Group = 41-60 Gender = FEMALE Occupational group = OVERALL Total cases = 588

Threshold dB HL≥	0	5	10	15	20	25	30	35	40	45	50	55	65	75	85	95
Prevalence	95.6	74.5	43.9	24.3	14.4	8.6	6.2	5.0	3.2	1.9	1.2	0.8	0.4	0.2	0.1	0.1
Lower CI	93.2	70.5	39.7	21.0	12.0	6.7	4.7	3.7	2.3	1.3	0.8	0.4	0.2	0.1	0.0	0.0
Upper CI	97.2	78.2	48.1	27.8	17.2	10.9	8.2	6.8	4.5	2.9	1.9	1.3	0.9	0.5	0.4	0.4
Number	569	462	303	196	132	85	66	57	41	27	20	14	7	5	3	3

Table B512-6 Age Group = 61-80 Gender = FEMALE Occupational group = OVERALL Total cases = 385

Threshold dB HL≥	0	5	10	15	20	25	30	35	40	45	50	55	65	75	85	95
Prevalence	99.0	94.5	77.8	61.1	43.3	31.3	24.6	18.0	13.2	8.8	6.7	4.8	3.0	2.1	1.3	0.6
Lower CI	96.9	91.3	72.7	55.5	38.0	26.8	20.2	14.2	10.9	6.8	5.4	3.6	2.1	1.3	0.7	0.2
Upper CI	99.7	96.6	82.1	66.4	48.7	36.2	29.6	22.5	15.9	11.4	8.3	6.3	4.3	3.2	2.3	1.5
Number	382	366	309	261	204	167	139	112	96	72	62	45	28	17	10	4

Table B512-7 Age Group = 18-40 Gender = MALE Occupational group = OVERALL Total cases = 319

Threshold dB HL≥	0	5	10	15	20	25	30	35	40	45	50	55	65	75	85	95
Prevalence	77.1	41.5	21.3	7.9	2.8	1.7	0.6	0.5	0.5	0.5	0.5	0.5	0.4	0.4	0.4	0.4
Lower CI	70.6	35.3	16.4	5.1	1.4	0.8	0.2	0.1	0.1	0.1	0.1	0.1	0.1	0.1	0.1	0.1
Upper CI	82.5	48.1	27.2	12.1	5.7	3.6	2.2	2.2	2.2	2.2	2.2	2.2	2.3	2.3	2.3	2.3
Number	265	156	86	34	15	12	5	4	3	3	3	3	1	1	1	1

Prevalence by age group, gender and occupational group for the better ear over 0.5, 1 and 2 kHz

Table B512-15 Age Group = 61-80 Gender = OVERALL Occupational group = MANUAL Total cases = 447

	Threshold dB HL≥															
	0	5	10	15	20	25	30	35	40	45	50	55	65	75	85	95
Prevalence	99.7	94.0	78.8	64.7	46.1	30.3	22.5	15.6	13.6	10.2	7.0	5.3	3.0	2.1	1.4	0.9
Lower CI	98.1	90.4	73.3	58.8	40.7	26.6	19.4	12.6	10.8	7.6	5.8	4.2	2.3	1.5	1.0	0.6
Upper CI	99.9	96.2	83.4	70.2	51.6	34.3	25.8	19.1	16.9	13.6	8.6	6.7	3.9	2.9	2.2	1.3
Number	446	427	373	322	258	204	163	127	110	86	68	51	28	19	11	5

Table B512-16 Age Group = 18-40 Gender = FEMALE Occupational group = NON-MANUAL Total cases = 201

	Threshold dB HL≥															
	0	5	10	15	20	25	30	35	40	45	50	55	65	75	85	95
Prevalence	83.4	41.2	13.4	4.5	2.4	1.7	1.0	0.8	0.7	0.2	0.2	0.0	0.0	0.0	0.0	0.0
Lower CI	77.1	34.2	9.4	2.5	1.3	0.7	0.3	0.2	0.2	0.0	0.0	0.0	0.0	0.0	0.0	0.0
Upper CI	88.3	48.6	18.7	8.1	4.3	3.9	2.9	2.8	2.9	1.2	1.2	1.9	1.9	1.9	1.9	1.9
Number	171	89	35	13	9	7	4	3	2	1	1	0	0	0	0	0

Table B512-17 Age Group = 41-60 Gender = FEMALE Occupational group = NON-MANUAL Total cases = 287

	Threshold dB HL≥															
	0	5	10	15	20	25	30	35	40	45	50	55	65	75	85	95
Prevalence	95.5	72.5	38.4	20.0	10.5	4.1	3.5	3.0	2.3	1.3	0.9	0.5	0.5	0.3	0.1	0.1
Lower CI	92.0	66.9	33.1	16.2	8.1	3.0	2.5	2.0	1.5	0.7	0.4	0.2	0.2	0.1	0.0	0.0
Upper CI	97.5	77.4	44.0	24.6	13.5	5.7	5.0	4.6	3.5	2.4	1.9	1.5	1.5	0.8	0.4	0.4
Number	275	214	130	79	49	25	20	19	16	9	7	4	4	3	1	1

Table B512-18 Age Group = 61-80 Gender = FEMALE Occupational group = NON-MANUAL Total cases = 182

	Threshold dB HL≥															
	0	5	10	15	20	25	30	35	40	45	50	55	65	75	85	95
Prevalence	98.0	93.7	75.0	57.8	45.7	33.5	26.0	22.2	12.0	6.6	5.8	4.5	3.1	1.8	0.8	0.4
Lower CI	94.1	88.3	66.7	49.2	38.8	28.3	21.6	18.2	9.8	5.0	4.2	3.0	1.8	0.8	0.3	0.1
Upper CI	99.3	96.7	81.9	66.0	52.7	39.2	31.0	26.8	14.6	8.6	7.9	6.7	5.2	3.9	2.2	1.8
Number	179	171	141	114	95	76	62	52	40	32	29	22	15	7	4	2

Table B512-19 Age Group = 18-40 Gender = MALE Occupational group = NON-MANUAL Total cases = 128

	Threshold dB HL≥															
	0	5	10	15	20	25	30	35	40	45	50	55	65	75	85	95
Prevalence	75.1	34.2	13.3	6.4	1.9	0.8	0.1	0.1	0.1	0.1	0.1	0.1	0.0	0.0	0.0	0.0
Lower CI	64.0	25.7	8.0	2.8	0.6	0.2	0.0	0.0	0.0	0.0	0.0	0.0	0.0	0.0	0.0	0.0
Upper CI	83.6	43.8	21.2	14.3	6.2	2.7	3.1	3.1	3.1	3.1	3.1	3.1	2.9	2.9	2.9	2.9
Number	105	54	24	9	4	3	1	1	1	1	1	1	0	0	0	0

Table B512-20 Age Group = 41-60 Gender = MALE Occupational group = NON-MANUAL Total cases = 218

	Threshold dB HL≥															
	0	5	10	15	20	25	30	35	40	45	50	55	65	75	85	95
Prevalence	93.7	68.7	35.2	23.4	10.2	6.5	3.8	1.7	1.2	1.0	0.4	0.4	0.0	0.0	0.0	0.0
Lower CI	88.4	61.5	28.7	18.3	7.3	4.2	2.0	1.0	0.6	0.5	0.2	0.2	0.0	0.0	0.0	0.0
Upper CI	96.7	75.2	42.3	29.6	14.2	10.0	7.2	3.2	2.4	2.0	1.2	1.2	1.7	1.7	1.7	1.7
Number	204	152	87	62	39	26	15	11	8	7	4	4	0	0	0	0

Table B512-21 Age Group = 61-80 Gender = MALE Occupational group = NON-MANUAL Total cases = 168

	Threshold dB HL≥															
	0	5	10	15	20	25	30	35	40	45	50	55	65	75	85	95
Prevalence	99.9	93.6	78.1	55.5	42.5	33.7	20.7	13.5	8.6	5.6	2.9	1.9	1.7	1.0	0.0	0.0
Lower CI	99.3	88.6	71.0	45.9	33.8	25.3	16.1	10.0	6.1	3.3	1.4	0.7	0.6	0.2	0.0	0.0
Upper CI	100.0	96.5	83.9	64.6	51.8	43.3	26.2	18.0	11.9	9.3	5.8	4.8	4.7	4.2	2.2	2.2
Number	167	156	132	100	77	58	39	25	18	14	8	5	4	2	0	0

Prevalence by age group, gender and occupational group for the better ear over 0.5, 1 and 2 kHz

Table B512-22 Age Group = 18-40 Gender = FEMALE Occupational group = MANUAL Total cases = 212

	Threshold dB HL≥															
	**0**	**5**	**10**	**15**	**20**	**25**	**30**	**35**	**40**	**45**	**50**	**55**	**65**	**75**	**85**	**95**
Prevalence	**86.7**	**47.7**	**19.0**	**9.1**	**4.2**	**3.6**	**1.8**	**0.9**	**0.9**	**0.4**	**0.2**	**0.2**	**0.1**	**0.1**	**0.0**	**0.0**
Lower CI	79.1	39.2	14.1	6.0	2.4	1.9	0.8	0.4	0.4	0.1	0.1	0.1	0.0	0.0	0.0	0.0
Upper CI	91.8	56.3	25.2	13.7	7.4	6.8	4.1	1.9	1.9	1.2	0.9	0.9	0.7	0.7	1.8	1.8
Number	192	124	65	36	19	15	10	7	7	3	2	2	1	1	0	0

Table B512-23 Age Group = 41-60 Gender = FEMALE Occupational group = MANUAL Total cases = 301

	Threshold dB HL≥															
	**0**	**5**	**10**	**15**	**20**	**25**	**30**	**35**	**40**	**45**	**50**	**55**	**65**	**75**	**85**	**95**
Prevalence	**96.3**	**78.6**	**50.1**	**28.5**	**18.1**	**13.6**	**9.0**	**6.7**	**3.8**	**2.0**	**1.3**	**0.8**	**0.2**	**0.2**	**0.2**	**0.2**
Lower CI	92.5	72.7	43.6	23.3	14.0	10.0	6.3	4.6	2.4	1.2	0.7	0.5	0.1	0.0	0.0	0.0
Upper CI	98.2	83.6	56.7	34.4	23.1	18.2	12.8	9.6	6.1	3.4	2.3	1.3	0.7	0.6	0.6	0.6
Number	294	248	173	117	83	60	46	38	25	18	13	10	3	2	2	2

Table B512-24 Age Group = 61-80 Gender = FEMALE Occupational group = MANUAL Total cases = 202

	Threshold dB HL≥															
	**0**	**5**	**10**	**15**	**20**	**25**	**30**	**35**	**40**	**45**	**50**	**55**	**65**	**75**	**85**	**95**
Prevalence	**100.0**	**95.1**	**79.0**	**64.4**	**41.7**	**31.7**	**24.0**	**16.1**	**15.1**	**10.7**	**7.0**	**4.9**	**3.4**	**2.7**	**2.0**	**1.1**
Lower CI	98.1	89.8	71.7	56.5	34.7	26.3	19.6	11.9	11.0	6.9	5.6	3.7	2.4	1.8	1.2	0.9
Upper CI	100.0	97.7	84.8	71.6	49.1	37.8	29.0	21.4	20.5	16.2	8.8	6.6	4.7	4.0	3.2	1.5
Number	202	194	167	146	109	91	77	60	56	40	33	23	13	10	6	2

Table B512-25 Age Group = 18-40 Gender = MALE Occupational group = MANUAL Total cases = 189

	Threshold dB HL≥															
	**0**	**5**	**10**	**15**	**20**	**25**	**30**	**35**	**40**	**45**	**50**	**55**	**65**	**75**	**85**	**95**
Prevalence	**79.6**	**49.6**	**27.6**	**9.4**	**3.7**	**2.6**	**1.0**	**0.8**	**0.7**	**0.7**	**0.7**	**0.7**	**0.7**	**0.7**	**0.7**	**0.7**
Lower CI	71.8	40.2	19.7	5.3	1.6	0.9	0.3	0.2	0.1	0.1	0.1	0.1	0.1	0.1	0.1	0.1
Upper CI	85.7	58.9	37.2	16.0	8.6	6.9	3.7	3.6	3.6	3.6	3.6	3.6	3.7	3.7	3.7	3.7
Number	160	102	62	25	11	9	4	3	2	2	2	2	1	1	1	1

Table B512-26 Age Group = 41-60 Gender = MALE Occupational group = MANUAL Total cases = 326

	Threshold dB HL≥															
	**0**	**5**	**10**	**15**	**20**	**25**	**30**	**35**	**40**	**45**	**50**	**55**	**65**	**75**	**85**	**95**
Prevalence	**97.0**	**77.9**	**54.2**	**34.0**	**19.5**	**12.2**	**8.5**	**6.3**	**4.8**	**3.2**	**2.2**	**1.7**	**0.8**	**0.2**	**0.1**	**0.0**
Lower CI	93.2	71.5	47.7	28.7	15.5	9.3	6.2	4.4	3.2	2.2	1.5	1.0	0.4	0.1	0.0	0.0
Upper CI	98.7	83.2	60.5	39.9	24.3	15.7	11.4	8.8	7.2	4.7	3.4	2.9	1.7	0.8	0.6	1.2
Number	320	275	210	142	94	68	51	41	31	24	17	14	7	2	1	0

Table B512-27 Age Group = 61-80 Gender = MALE Occupational group = MANUAL Total cases = 245

	Threshold dB HL≥															
	**0**	**5**	**10**	**15**	**20**	**25**	**30**	**35**	**40**	**45**	**50**	**55**	**65**	**75**	**85**	**95**
Prevalence	**99.2**	**92.5**	**78.2**	**64.8**	**51.5**	**28.2**	**20.3**	**15.0**	**11.6**	**9.7**	**7.2**	**5.8**	**2.5**	**1.3**	**0.8**	**0.6**
Lower CI	95.6	86.9	69.1	55.5	43.3	23.9	16.8	11.6	8.8	7.2	5.0	3.9	1.6	0.7	0.3	0.2
Upper CI	99.9	95.8	85.2	73.1	59.5	32.9	24.4	19.2	15.2	12.9	10.2	8.4	4.0	2.5	1.9	1.7
Number	244	233	206	176	149	113	86	67	54	46	35	28	15	9	5	3

Prevalence by age group, gender and occupational group for the better ear over 4, 6 and 8 kHz

Table B468-1 Age Group = 18-40 Gender = OVERALL Occupational group = OVERALL Total cases = 732

Threshold dB HL≥	0	5	10	15	20	25	30	35	40	45	50	55	65	75	85	95
**Prevalence**	**94.3**	**81.2**	**55.3**	**33.2**	**18.1**	**9.8**	**6.0**	**3.6**	**2.6**	**2.1**	**1.3**	**1.0**	**0.7**	**0.5**	**0.4**	**0.2**
**Lower CI**	91.8	77.4	51.0	29.4	15.1	7.7	4.3	2.4	1.7	1.3	0.8	0.5	0.3	0.2	0.1	0.0
**Upper CI**	96.1	84.5	59.6	37.2	21.4	12.4	8.2	5.2	4.1	3.6	2.4	1.9	1.6	1.4	1.4	1.1
**Number**	696	617	454	289	176	112	69	47	35	26	20	16	10	6	4	2

Table B468-2 Age Group = 41-60 Gender = OVERALL Occupational group = OVERALL Total cases = 1130

Threshold dB HL≥	0	5	10	15	20	25	30	35	40	45	50	55	65	75	85	95
**Prevalence**	**100.0**	**98.4**	**91.3**	**78.3**	**62.4**	**47.8**	**37.4**	**27.6**	**22.2**	**17.3**	**13.5**	**10.5**	**5.4**	**2.9**	**1.5**	**0.9**
**Lower CI**	99.7	97.4	89.2	75.5	59.4	44.8	34.6	25.1	20.0	15.4	11.9	9.0	4.4	2.2	1.0	0.6
**Upper CI**	100.0	99.1	93.0	80.8	65.4	50.8	40.3	30.2	24.6	19.5	15.4	12.1	6.6	3.9	2.1	1.5
**Number**	1130	1115	1049	926	783	644	534	429	353	288	240	194	112	62	36	20

Table B468-3 Age Group = 61-80 Gender = OVERALL Occupational group = OVERALL Total cases = 796

Threshold dB HL≥	0	5	10	15	20	25	30	35	40	45	50	55	65	75	85	95
**Prevalence**	**100.0**	**100.0**	**100.0**	**98.5**	**94.1**	**88.7**	**84.6**	**76.4**	**69.2**	**61.4**	**52.8**	**44.6**	**29.0**	**12.4**	**5.5**	**2.2**
**Lower CI**	99.5	99.5	99.5	96.7	91.6	85.2	81.0	72.5	64.9	57.1	48.8	40.6	25.9	10.5	4.3	1.6
**Upper CI**	100.0	100.0	100.0	99.3	95.9	91.4	87.7	80.0	73.2	65.5	56.7	48.6	32.3	14.5	7.0	3.0
**Number**	796	796	796	787	764	734	704	654	605	560	508	457	322	172	84	40

Table B468-4 Age Group = 18-40 Gender = FEMALE Occupational group = OVERALL Total cases = 413

Threshold dB HL≥	0	5	10	15	20	25	30	35	40	45	50	55	65	75	85	95
**Prevalence**	**94.8**	**80.8**	**52.9**	**27.4**	**13.3**	**7.2**	**3.8**	**2.8**	**2.3**	**1.7**	**1.0**	**0.6**	**0.5**	**0.1**	**0.0**	**0.0**
**Lower CI**	91.8	76.0	47.3	23.0	10.2	5.3	2.5	1.8	1.3	0.9	0.4	0.3	0.1	0.0	0.0	0.0
**Upper CI**	96.7	84.9	58.4	32.3	17.0	9.9	5.7	4.5	4.0	3.4	2.4	1.6	1.5	0.5	0.9	0.9
**Number**	393	345	245	144	85	54	30	24	18	12	8	7	4	1	0	0

Table B468-5 Age Group = 41-60 Gender = FEMALE Occupational group = OVERALL Total cases = 587

Threshold dB HL≥	0	5	10	15	20	25	30	35	40	45	50	55	65	75	85	95
**Prevalence**	**100.0**	**97.2**	**87.1**	**71.5**	**53.2**	**37.6**	**27.6**	**17.7**	**12.1**	**8.3**	**6.0**	**4.7**	**2.3**	**1.4**	**0.7**	**0.5**
**Lower CI**	99.4	95.1	83.7	67.4	48.9	33.7	24.0	14.9	9.9	6.5	4.6	3.4	1.5	0.9	0.4	0.2
**Upper CI**	100.0	98.3	89.9	75.2	57.4	41.7	31.4	20.9	14.8	10.4	7.8	6.3	3.3	2.3	1.3	1.0
**Number**	587	573	523	442	354	272	213	157	117	88	68	52	30	20	11	7

Table B468-6 Age Group = 61-80 Gender = FEMALE Occupational group = OVERALL Total cases = 385

Threshold dB HL≥	0	5	10	15	20	25	30	35	40	45	50	55	65	75	85	95
**Prevalence**	**100.0**	**100.0**	**100.0**	**98.0**	**90.9**	**82.9**	**77.7**	**69.2**	**60.3**	**52.0**	**45.3**	**35.6**	**20.4**	**8.2**	**3.9**	**1.4**
**Lower CI**	99.0	99.0	99.0	95.6	86.9	77.2	71.9	63.2	53.9	45.9	40.0	30.2	16.7	6.3	2.8	0.8
**Upper CI**	100.0	100.0	100.0	99.1	93.7	87.4	82.6	74.6	66.4	58.1	50.8	41.4	24.6	10.6	5.4	2.4
**Number**	385	385	385	377	356	334	314	285	255	229	207	182	116	63	34	12

Table B468-7 Age Group = 18-40 Gender = MALE Occupational group = OVERALL Total cases = 319

Threshold dB HL≥	0	5	10	15	20	25	30	35	40	45	50	55	65	75	85	95
**Prevalence**	**93.8**	**81.6**	**57.9**	**39.3**	**23.2**	**12.5**	**8.3**	**4.3**	**2.9**	**2.6**	**1.7**	**1.4**	**0.9**	**0.9**	**0.8**	**0.4**
**Lower CI**	89.4	75.6	51.3	33.2	18.3	8.9	5.4	2.4	1.5	1.2	0.8	0.6	0.3	0.3	0.2	0.1
**Upper CI**	96.5	86.5	64.3	45.8	29.0	17.4	12.5	7.4	5.8	5.4	3.5	3.1	2.8	2.8	2.8	2.3
**Number**	303	272	209	145	91	58	39	23	17	14	12	9	6	5	4	2

Prevalence by age group, gender and occupational group for the better ear over 4, 6 and 8 kHz

Table B468-8 Age Group = 41-60 Gender = MALE Occupational group = OVERALL Total cases = 543

Threshold dB HL≥	0	5	10	15	20	25	30	35	40	45	50	55	65	75	85	95
**Prevalence**	**100.0**	**99.8**	**95.9**	**85.8**	**72.7**	**59.0**	**48.3**	**38.6**	**33.4**	**27.4**	**21.9**	**17.0**	**8.9**	**4.6**	**2.4**	**1.5**
Lower CI	99.3	99.2	93.2	82.0	68.3	54.4	43.9	34.5	29.5	23.9	18.8	14.3	7.0	3.2	1.5	0.8
Upper CI	100.0	100.0	97.5	88.9	76.7	63.5	52.7	42.9	37.6	31.3	25.3	20.0	11.3	6.5	3.6	2.6
Number	543	542	526	484	429	372	321	272	236	200	172	142	82	42	25	13

Table B468-9 Age Group = 61-80 Gender = MALE Occupational group = OVERALL Total cases = 411

Threshold dB HL≥	0	5	10	15	20	25	30	35	40	45	50	55	65	75	85	95
**Prevalence**	**100.0**	**100.0**	**100.0**	**99.0**	**98.1**	**95.9**	**93.3**	**85.4**	**80.4**	**73.1**	**62.2**	**56.0**	**40.2**	**18.0**	**7.6**	**3.4**
Lower CI	99.1	99.1	99.1	94.5	94.0	91.8	89.0	80.4	74.9	67.2	56.3	50.4	35.1	14.6	5.3	2.3
Upper CI	100.0	100.0	100.0	99.8	99.4	98.0	96.0	89.3	85.0	78.3	67.7	61.5	45.5	21.9	10.8	4.9
Number	411	411	411	410	408	400	390	369	350	331	301	275	206	109	50	28

Table B468-10 Age Group = 18-40 Gender = OVERALL Occupational group = NON-MANUAL Total cases = 329

Threshold dB HL≥	0	5	10	15	20	25	30	35	40	45	50	55	65	75	85	95
**Prevalence**	**95.0**	**78.9**	**48.9**	**26.9**	**12.5**	**6.6**	**3.9**	**3.1**	**2.5**	**1.9**	**0.9**	**0.6**	**0.3**	**0.0**	**0.0**	**0.0**
Lower CI	91.3	73.3	43.1	21.7	8.9	4.3	2.1	1.5	1.1	0.7	0.4	0.2	0.1	0.0	0.0	0.0
Upper CI	97.2	83.5	54.7	32.7	17.3	10.1	7.1	6.2	5.5	5.2	2.3	1.7	1.2	1.2	1.2	1.2
Number	313	268	183	103	52	31	19	15	12	8	6	4	3	0	0	0

Table B468-11 Age Group = 41-60 Gender = OVERALL Occupational group = NON-MANUAL Total cases = 505

Threshold dB HL≥	0	5	10	15	20	25	30	35	40	45	50	55	65	75	85	95
**Prevalence**	**100.0**	**98.1**	**89.9**	**73.9**	**56.6**	**41.3**	**32.8**	**23.0**	**17.8**	**13.6**	**10.4**	**8.0**	**4.1**	**1.2**	**0.7**	**0.4**
Lower CI	99.2	96.4	86.5	69.6	52.0	37.4	29.0	19.7	15.0	11.2	8.7	5.9	2.6	0.8	0.4	0.2
Upper CI	100.0	99.0	92.4	77.7	61.1	45.3	36.7	26.6	21.1	16.5	12.5	10.7	6.5	1.9	1.4	1.0
Number	505	495	458	384	308	245	197	151	120	92	76	62	33	16	10	6

Table B468-12 Age Group = 61-80 Gender = OVERALL Occupational group = NON-MANUAL Total cases = 349

Threshold dB HL≥	0	5	10	15	20	25	30	35	40	45	50	55	65	75	85	95
**Prevalence**	**100.0**	**100.0**	**100.0**	**97.4**	**93.0**	**87.2**	**84.2**	**74.0**	**67.5**	**60.2**	**50.4**	**44.4**	**25.7**	**9.8**	**5.0**	**2.0**
Lower CI	98.9	98.9	98.9	92.1	87.9	81.4	78.4	68.0	60.8	53.8	45.6	40.0	21.8	7.6	3.7	1.2
Upper CI	100.0	100.0	100.0	99.1	96.1	91.4	88.7	79.3	73.5	66.3	55.2	49.0	30.0	12.7	6.7	3.1
Number	349	349	349	343	331	314	305	273	251	229	200	177	119	53	32	11

Table B468-13 Age Group = 18-40 Gender = OVERALL Occupational group = MANUAL Total cases = 401

Threshold dB HL≥	0	5	10	15	20	25	30	35	40	45	50	55	65	75	85	95
**Prevalence**	**93.1**	**83.0**	**60.4**	**39.1**	**23.9**	**12.2**	**7.5**	**3.7**	**2.6**	**2.2**	**1.4**	**1.2**	**0.8**	**0.8**	**0.7**	**0.3**
Lower CI	88.5	77.3	53.7	33.2	19.0	9.0	5.0	2.3	1.6	1.2	0.7	0.6	0.3	0.3	0.2	0.1
Upper CI	96.0	87.5	66.7	45.3	29.5	16.4	11.2	5.9	4.2	3.8	2.8	2.6	2.3	2.3	2.2	1.8
Number	381	347	270	186	124	81	50	32	23	18	14	12	7	6	4	2

Table B468-14 Age Group = 41-60 Gender = OVERALL Occupational group = MANUAL Total cases = 625

Threshold dB HL≥	0	5	10	15	20	25	30	35	40	45	50	55	65	75	85	95
**Prevalence**	**100.0**	**99.0**	**93.4**	**83.2**	**68.3**	**53.4**	**41.7**	**31.8**	**26.0**	**20.7**	**15.9**	**12.2**	**6.4**	**3.7**	**1.7**	**1.1**
Lower CI	99.4	97.6	90.7	79.4	63.8	48.9	37.5	28.2	22.7	17.8	13.4	10.0	4.8	2.5	1.1	0.6
Upper CI	100.0	99.6	95.4	86.4	72.5	57.9	45.9	35.7	29.7	24.0	18.7	14.7	8.3	5.3	2.5	1.9
Number	625	620	591	542	475	399	337	278	233	196	164	132	79	46	26	14

Prevalence by age group, gender and occupational group for the better ear over 4, 6 and 8 kHz

Table B468-15 Age Group = 61-80 Gender = OVERALL Occupational group = MANUAL Total cases = 445

	Threshold dB HL≥															
	0	5	10	15	20	25	30	35	40	45	50	55	65	75	85	95
**Prevalence**	**100.0**	**100.0**	**100.0**	**99.0**	**94.4**	**90.0**	**85.0**	**78.3**	**70.9**	**63.3**	**54.2**	**45.1**	**29.4**	**14.0**	**6.2**	**2.9**
**Lower CI**	99.1	99.1	99.1	96.9	90.5	85.3	80.2	72.7	64.9	57.2	48.9	40.2	25.2	11.5	4.3	2.2
**Upper CI**	100.0	100.0	100.0	99.7	96.7	93.4	88.8	83.1	76.2	69.0	59.5	50.1	34.1	16.9	8.8	4.0
**Number**	445	445	445	442	431	418	397	379	352	329	306	279	203	119	52	29

Table B468-16 Age Group = 18-40 Gender = FEMALE Occupational group = NON-MANUAL Total cases = 201

	Threshold dB HL≥															
	0	5	10	15	20	25	30	35	40	45	50	55	65	75	85	95
**Prevalence**	**94.4**	**78.6**	**48.4**	**24.5**	**8.5**	**3.9**	**2.3**	**2.3**	**2.1**	**1.1**	**1.1**	**0.5**	**0.5**	**0.0**	**0.0**	**0.0**
**Lower CI**	90.4	71.9	41.2	19.0	5.6	2.3	1.2	1.2	1.1	0.3	0.3	0.1	0.1	0.0	0.0	0.0
**Upper CI**	96.8	84.0	55.5	30.8	12.7	6.6	4.4	4.4	4.2	3.6	3.6	2.5	2.5	1.9	1.9	1.9
**Number**	189	161	106	58	25	13	8	8	7	3	3	2	2	0	0	0

Table B468-17 Age Group = 41-60 Gender = FEMALE Occupational group = NON-MANUAL Total cases = 287

	Threshold dB HL≥															
	0	5	10	15	20	25	30	35	40	45	50	55	65	75	85	95
**Prevalence**	**100.0**	**96.6**	**86.6**	**67.7**	**48.9**	**33.2**	**25.2**	**15.5**	**11.0**	**7.3**	**4.8**	**4.0**	**2.5**	**1.2**	**0.7**	**0.6**
**Lower CI**	98.7	93.5	81.7	61.7	43.0	28.2	20.6	12.2	8.4	5.2	3.3	2.6	1.5	0.6	0.3	0.2
**Upper CI**	100.0	98.2	90.3	73.1	54.8	38.6	30.5	19.6	14.4	10.2	6.9	6.0	4.0	2.2	1.7	1.6
**Number**	287	278	253	203	157	118	93	68	53	37	26	20	13	8	5	4

Table B468-18 Age Group = 61-80 Gender = FEMALE Occupational group = NON-MANUAL Total cases = 182

	Threshold dB HL≥															
	0	5	10	15	20	25	30	35	40	45	50	55	65	75	85	95
**Prevalence**	**100.0**	**100.0**	**100.0**	**98.0**	**91.5**	**83.9**	**81.0**	**71.7**	**62.7**	**54.0**	**47.1**	**42.3**	**19.2**	**6.3**	**2.9**	**0.7**
**Lower CI**	97.9	97.9	97.9	94.5	85.7	76.4	73.4	64.0	53.6	45.6	40.5	35.8	14.1	4.0	1.9	0.2
**Upper CI**	100.0	100.0	100.0	99.3	95.0	89.4	86.8	78.3	71.0	62.2	53.8	49.1	25.7	9.8	4.4	1.8
**Number**	182	182	182	177	167	156	151	134	120	106	94	85	53	26	16	4

Table B468-19 Age Group = 18-40 Gender = MALE Occupational group = NON-MANUAL Total cases = 128

	Threshold dB HL≥															
	0	5	10	15	20	25	30	35	40	45	50	55	65	75	85	95
**Prevalence**	**95.7**	**79.3**	**49.7**	**29.5**	**16.8**	**9.6**	**5.6**	**4.0**	**2.9**	**2.9**	**0.8**	**0.6**	**0.1**	**0.0**	**0.0**	**0.0**
**Lower CI**	87.6	69.8	40.5	21.0	10.3	5.4	2.4	1.4	0.8	0.8	0.3	0.1	0.0	0.0	0.0	0.0
**Upper CI**	98.6	86.4	58.8	39.7	26.0	16.7	12.5	10.8	10.0	10.0	2.5	2.6	3.1	2.9	2.9	2.9
**Number**	124	107	77	45	27	18	11	7	5	5	3	2	1	0	0	0

Table B468-20 Age Group = 41-60 Gender = MALE Occupational group = NON-MANUAL Total cases = 218

	Threshold dB HL≥															
	0	5	10	15	20	25	30	35	40	45	50	55	65	75	85	95
**Prevalence**	**100.0**	**99.8**	**93.5**	**80.7**	**65.2**	**50.2**	**41.2**	**31.3**	**25.3**	**20.5**	**16.6**	**12.4**	**5.9**	**1.2**	**0.7**	**0.3**
**Lower CI**	98.3	98.7	88.3	74.2	57.8	44.2	35.4	25.7	20.2	16.1	13.4	8.4	3.1	0.6	0.3	0.1
**Upper CI**	100.0	100.0	96.5	85.9	71.9	56.2	47.2	37.5	31.2	25.8	20.5	17.9	10.8	2.2	1.8	1.1
**Number**	218	217	205	181	151	127	104	83	67	55	50	42	20	8	5	2

Table B468-21 Age Group = 61-80 Gender = MALE Occupational group = NON-MANUAL Total cases = 167

	Threshold dB HL≥															
	0	5	10	15	20	25	30	35	40	45	50	55	65	75	85	95
**Prevalence**	**100.0**	**100.0**	**100.0**	**96.5**	**94.9**	**91.2**	**88.0**	**76.8**	**73.4**	**68.1**	**54.6**	**47.3**	**33.3**	**14.3**	**7.7**	**3.8**
**Lower CI**	97.8	97.8	97.8	82.1	82.3	80.1	77.2	66.5	63.2	58.0	47.8	41.7	28.0	10.4	5.3	2.2
**Upper CI**	100.0	100.0	100.0	99.4	98.7	96.4	94.1	84.7	81.6	76.7	61.3	52.9	39.1	19.2	11.2	6.2
**Number**	167	167	167	166	164	158	154	139	131	123	106	92	66	27	16	7

Prevalence by age group, gender and occupational group for the better ear over 4, 6 and 8 kHz

Table B468-22 Age Group = 18-40 Gender = FEMALE Occupational group = MANUAL Total cases = 212

Threshold dB HL≥	0	5	10	15	20	25	30	35	40	45	50	55	65	75	85	95
**Prevalence**	**94.6**	**82.9**	**58.5**	**31.4**	**19.5**	**10.1**	**4.7**	**2.8**	**2.2**	**1.9**	**0.6**	**0.6**	**0.2**	**0.1**	**0.0**	**0.0**
**Lower CI**	87.9	74.9	49.5	24.3	13.7	6.7	2.7	1.5	1.0	0.9	0.2	0.2	0.1	0.0	0.0	0.0
**Upper CI**	97.7	88.7	67.0	39.5	27.0	14.8	8.3	5.1	4.6	4.4	1.3	1.3	0.9	0.7	1.8	1.8
**Number**	204	184	139	86	60	41	22	16	11	9	5	5	2	1	0	0

Table B468-23 Age Group = 41-60 Gender = FEMALE Occupational group = MANUAL Total cases = 300

Threshold dB HL≥	0	5	10	15	20	25	30	35	40	45	50	55	65	75	85	95
**Prevalence**	**100.0**	**98.1**	**88.9**	**76.5**	**58.2**	**42.6**	**30.1**	**19.4**	**13.1**	**8.7**	**6.6**	**4.8**	**1.9**	**1.3**	**0.5**	**0.2**
**Lower CI**	98.7	95.3	84.1	70.7	51.5	36.4	24.6	15.1	9.7	6.2	4.6	3.2	1.1	0.7	0.2	0.1
**Upper CI**	100.0	99.2	92.3	81.5	64.5	49.1	36.2	24.5	17.5	12.0	9.5	7.3	3.1	2.3	1.0	0.7
**Number**	300	295	270	239	197	154	120	89	64	51	42	32	17	12	6	3

Table B468-24 Age Group = 61-80 Gender = FEMALE Occupational group = MANUAL Total cases = 202

Threshold dB HL≥	0	5	10	15	20	25	30	35	40	45	50	55	65	75	85	95
**Prevalence**	**100.0**	**100.0**	**100.0**	**98.1**	**89.9**	**82.9**	**75.4**	**66.9**	**58.4**	**50.6**	**43.4**	**29.9**	**17.0**	**8.8**	**4.7**	**2.5**
**Lower CI**	98.1	98.1	98.1	94.4	83.2	74.7	67.2	57.6	48.9	41.6	35.8	23.4	12.6	6.7	3.2	1.6
**Upper CI**	100.0	100.0	100.0	99.4	94.1	88.8	82.1	75.0	67.2	59.6	51.4	37.4	22.7	11.5	6.8	4.0
**Number**	202	202	202	199	188	177	162	150	134	122	112	97	63	37	18	8

Table B468-25 Age Group = 18-40 Gender = MALE Occupational group = MANUAL Total cases = 189

Threshold dB HL≥	0	5	10	15	20	25	30	35	40	45	50	55	65	75	85	95
**Prevalence**	**91.5**	**82.9**	**62.4**	**46.9**	**28.5**	**14.5**	**10.6**	**4.6**	**3.0**	**2.3**	**2.3**	**1.8**	**1.4**	**1.4**	**1.4**	**0.7**
**Lower CI**	83.9	74.1	52.4	37.7	21.0	9.2	6.1	2.4	1.6	1.0	1.0	0.7	0.4	0.4	0.4	0.1
**Upper CI**	95.7	89.2	71.4	56.4	37.3	21.9	17.7	8.5	5.5	5.0	5.0	4.8	4.5	4.5	4.5	3.6
**Number**	177	163	131	100	64	40	28	16	12	9	9	7	5	5	4	2

Table B468-26 Age Group = 41-60 Gender = MALE Occupational group = MANUAL Total cases = 325

Threshold dB HL≥	0	5	10	15	20	25	30	35	40	45	50	55	65	75	85	95
**Prevalence**	**100.0**	**100.0**	**98.3**	**90.4**	**79.4**	**65.3**	**54.4**	**45.5**	**40.3**	**33.9**	**26.1**	**20.2**	**11.3**	**6.3**	**3.0**	**1.9**
**Lower CI**	98.8	98.8	95.3	85.3	73.2	58.7	48.3	39.5	34.5	28.5	21.4	16.2	8.3	4.0	1.8	1.0
**Upper CI**	100.0	100.0	99.4	93.9	84.4	71.3	60.5	51.6	46.4	39.8	31.3	25.1	15.3	9.7	4.7	3.6
**Number**	325	325	321	303	278	245	217	189	169	145	122	100	62	34	20	11

Table B468-27 Age Group = 61-80 Gender = MALE Occupational group = MANUAL Total cases = 243

Threshold dB HL≥	0	5	10	15	20	25	30	35	40	45	50	55	65	75	85	95
**Prevalence**	**100.0**	**100.0**	**100.0**	**100.0**	**100.0**	**99.0**	**97.1**	**93.0**	**86.9**	**79.3**	**68.1**	**64.8**	**45.7**	**21.0**	**8.3**	**3.5**
**Lower CI**	98.4	98.4	98.4	98.4	98.4	95.7	93.8	88.4	81.2	71.9	61.1	57.8	38.0	15.9	4.6	2.3
**Upper CI**	100.0	100.0	100.0	100.0	100.0	99.8	98.7	95.8	91.1	85.1	74.3	71.2	53.6	27.2	14.4	5.3
**Number**	243	243	243	243	243	241	235	229	218	207	194	182	140	82	34	21

Prevalence by age group, gender and occupational group for the worse ear over 0.5, 1, 2 and 4 kHz

Table W5124-1 Age Group = 18-40 Gender = OVERALL Occupational group = OVERALL Total cases = 731

	Threshold dB HL≥															
	0	5	10	15	20	25	30	35	40	45	50	55	65	75	85	95
**Prevalence**	**95.0**	**70.1**	**36.2**	**21.1**	**11.7**	**7.4**	**5.7**	**3.7**	**2.1**	**1.8**	**1.1**	**0.9**	**0.4**	**0.3**	**0.3**	**0.3**
**Lower CI**	92.5	66.0	32.3	18.0	9.5	5.8	4.3	2.7	1.4	1.2	0.6	0.5	0.2	0.1	0.1	0.1
**Upper CI**	96.7	74.0	40.3	24.6	14.4	9.4	7.5	5.0	3.0	2.7	1.8	1.6	1.2	1.1	1.1	1.1
**Number**	705	557	328	205	137	99	78	56	38	32	21	18	8	5	5	3

Table W5124-2 Age Group = 41-60 Gender = OVERALL Occupational group = OVERALL Total cases = 1123

	Threshold dB HL≥															
	0	5	10	15	20	25	30	35	40	45	50	55	65	75	85	95
**Prevalence**	**99.7**	**94.4**	**76.0**	**53.4**	**37.2**	**27.7**	**18.3**	**13.7**	**10.7**	**8.6**	**6.8**	**5.4**	**3.3**	**2.2**	**1.2**	**1.0**
**Lower CI**	99.0	92.5	73.1	50.3	34.4	25.2	16.3	12.0	9.2	7.3	5.7	4.4	2.6	1.6	0.8	0.6
**Upper CI**	99.9	95.8	78.6	56.6	40.0	30.3	20.4	15.5	12.4	10.1	8.1	6.7	4.3	3.0	1.9	1.6
**Number**	1120	1073	904	700	538	422	314	252	203	169	140	112	76	53	31	24

Table W5124-3 Age Group = 61-80 Gender = OVERALL Occupational group = OVERALL Total cases = 792

	Threshold dB HL≥															
	0	5	10	15	20	25	30	35	40	45	50	55	65	75	85	95
**Prevalence**	**99.9**	**99.2**	**96.3**	**88.1**	**73.5**	**59.1**	**46.4**	**39.0**	**33.0**	**24.5**	**19.1**	**14.5**	**9.5**	**5.2**	**2.8**	**1.9**
**Lower CI**	99.2	98.1	94.0	85.0	69.5	54.7	42.6	35.6	29.5	21.6	16.7	12.4	7.8	4.3	2.1	1.3
**Upper CI**	100.0	99.7	97.8	90.7	77.1	63.2	50.2	42.5	36.7	27.6	21.9	16.8	11.5	6.4	3.7	2.6
**Number**	791	787	770	719	632	558	471	416	364	298	247	200	136	89	49	33

Table W5124-4 Age Group = 18-40 Gender = FEMALE Occupational group = OVERALL Total cases = 412

	Threshold dB HL≥															
	0	5	10	15	20	25	30	35	40	45	50	55	65	75	85	95
**Prevalence**	**96.4**	**68.4**	**32.4**	**16.1**	**9.5**	**6.3**	**5.3**	**3.8**	**2.5**	**2.1**	**1.2**	**0.9**	**0.3**	**0.2**	**0.2**	**0.1**
**Lower CI**	93.7	62.9	27.7	12.9	7.3	4.6	3.7	2.5	1.5	1.3	0.6	0.5	0.1	0.1	0.1	0.0
**Upper CI**	98.0	73.5	37.5	20.0	12.3	8.5	7.4	5.8	4.0	3.4	2.1	1.6	0.9	0.7	0.7	0.7
**Number**	399	307	174	102	70	50	41	29	22	19	12	10	4	3	3	1

Table W5124-5 Age Group = 41-60 Gender = FEMALE Occupational group = OVERALL Total cases = 583

	Threshold dB HL≥															
	0	5	10	15	20	25	30	35	40	45	50	55	65	75	85	95
**Prevalence**	**99.9**	**94.1**	**72.1**	**48.8**	**29.9**	**21.2**	**13.1**	**9.7**	**7.2**	**5.3**	**4.3**	**3.6**	**2.7**	**1.5**	**1.1**	**0.9**
**Lower CI**	99.2	91.5	68.1	44.6	26.4	18.2	10.8	7.8	5.7	4.1	3.2	2.6	1.8	0.9	0.5	0.4
**Upper CI**	100.0	95.9	75.8	53.2	33.6	24.5	15.8	11.9	9.2	7.0	5.7	4.9	3.9	2.5	2.0	1.9
**Number**	582	555	445	331	231	172	126	101	78	63	54	45	35	24	14	11

Table W5124-6 Age Group = 61-80 Gender = FEMALE Occupational group = OVERALL Total cases = 382

	Threshold dB HL≥															
	0	5	10	15	20	25	30	35	40	45	50	55	65	75	85	95
**Prevalence**	**99.8**	**98.9**	**94.2**	**83.6**	**69.2**	**52.4**	**40.4**	**34.9**	**30.3**	**21.9**	**17.2**	**13.6**	**9.0**	**4.9**	**2.4**	**1.7**
**Lower CI**	98.7	97.0	90.3	78.5	63.5	46.2	35.2	30.2	25.4	18.3	14.1	11.0	6.8	3.8	1.7	1.0
**Upper CI**	100.0	99.6	96.6	87.7	74.3	58.6	45.8	39.8	35.6	26.0	20.8	16.7	11.9	6.4	3.6	2.7
**Number**	381	378	364	332	286	246	204	186	167	140	116	99	67	44	22	15

Table W5124-7 Age Group = 18-40 Gender = MALE Occupational group = OVERALL Total cases = 319

	Threshold dB HL≥															
	0	5	10	15	20	25	30	35	40	45	50	55	65	75	85	95
**Prevalence**	**93.5**	**71.8**	**40.2**	**26.5**	**14.0**	**8.5**	**6.1**	**3.5**	**1.6**	**1.4**	**1.0**	**0.9**	**0.6**	**0.4**	**0.4**	**0.4**
**Lower CI**	88.8	65.4	33.9	21.2	10.2	5.9	4.0	2.1	0.8	0.7	0.4	0.3	0.1	0.1	0.1	0.1
**Upper CI**	96.4	77.4	46.8	32.7	18.9	12.1	9.3	5.6	3.0	2.8	2.4	2.4	2.2	2.3	2.3	2.3
**Number**	306	250	154	103	67	49	37	27	16	13	9	8	4	2	2	2

Prevalence by age group, gender and occupational group for the worse ear over 0.5, 1, 2 and 4 kHz

Table W5124-8 Age Group = 41-60 Gender = MALE Occupational group = OVERALL Total cases = 540

Threshold dB HL≥	0	5	10	15	20	25	30	35	40	45	50	55	65	75	85	95
**Prevalence**	**99.5**	**94.6**	**80.3**	**58.5**	**45.3**	**34.9**	**24.1**	**18.1**	**14.6**	**12.2**	**9.6**	**7.5**	**4.0**	**2.9**	**1.5**	**1.1**
**Lower CI**	98.2	91.7	76.0	53.9	41.0	31.0	20.9	15.4	12.1	9.9	7.6	5.8	2.8	1.9	0.9	0.6
**Upper CI**	99.9	96.5	83.9	63.0	49.6	39.0	27.6	21.2	17.4	14.8	12.0	9.7	5.7	4.4	2.5	2.1
**Number**	538	518	459	369	307	250	188	151	125	106	86	67	41	29	17	13

Table W5124-9 Age Group = 61-80 Gender = MALE Occupational group = OVERALL Total cases = 410

Threshold dB HL≥	0	5	10	15	20	25	30	35	40	45	50	55	65	75	85	95
**Prevalence**	**100.0**	**99.7**	**99.0**	**93.9**	**78.8**	**67.3**	**53.8**	**44.0**	**36.4**	**27.9**	**21.9**	**15.7**	**10.0**	**5.7**	**3.4**	**2.1**
**Lower CI**	99.1	98.3	97.1	90.8	73.2	61.6	48.5	39.2	31.6	23.6	18.1	12.7	7.7	4.3	2.3	1.3
**Upper CI**	100.0	99.9	99.7	96.0	83.5	72.4	59.0	49.0	41.4	32.6	26.2	19.3	12.8	7.5	5.0	3.4
**Number**	410	409	406	387	346	312	267	230	197	158	131	101	69	45	27	18

Table W5124-10 Age Group = 18-40 Gender = OVERALL Occupational group = NON-MANUAL Total cases = 329

Threshold dB HL≥	0	5	10	15	20	25	30	35	40	45	50	55	65	75	85	95
**Prevalence**	**92.5**	**64.2**	**29.5**	**14.7**	**8.7**	**6.4**	**4.0**	**3.0**	**1.6**	**1.2**	**0.4**	**0.4**	**0.1**	**0.1**	**0.1**	**0.1**
**Lower CI**	87.8	57.9	24.1	10.7	5.9	4.0	2.5	1.7	0.9	0.6	0.1	0.1	0.0	0.0	0.0	0.0
**Upper CI**	95.5	70.1	35.6	19.9	12.7	10.1	6.4	5.1	2.9	2.2	1.1	1.1	0.6	0.6	0.6	0.6
**Number**	312	230	115	58	41	32	24	18	12	10	4	4	2	1	1	1

Table W5124-11 Age Group = 41-60 Gender = OVERALL Occupational group = NON-MANUAL Total cases = 502

Threshold dB HL≥	0	5	10	15	20	25	30	35	40	45	50	55	65	75	85	95
**Prevalence**	**99.5**	**92.7**	**71.3**	**45.2**	**31.7**	**23.8**	**13.9**	**10.6**	**7.2**	**5.9**	**4.9**	**3.8**	**2.7**	**1.8**	**1.1**	**1.0**
**Lower CI**	98.4	89.7	66.8	40.8	28.0	20.7	10.9	8.2	5.6	4.4	3.5	2.6	1.9	1.2	0.6	0.5
**Upper CI**	99.8	94.9	75.4	49.7	35.6	27.3	17.5	13.6	9.4	7.8	6.7	5.6	3.9	2.8	2.0	2.0
**Number**	499	469	372	260	192	149	103	86	66	54	45	36	28	20	13	12

Table W5124-12 Age Group = 61-80 Gender = OVERALL Occupational group = NON-MANUAL Total cases = 348

Threshold dB HL≥	0	5	10	15	20	25	30	35	40	45	50	55	65	75	85	95
**Prevalence**	**99.7**	**98.7**	**96.7**	**88.7**	**68.8**	**57.8**	**44.5**	**37.7**	**32.5**	**20.3**	**14.4**	**10.0**	**6.6**	**4.3**	**2.3**	**1.4**
**Lower CI**	98.1	96.1	93.7	84.7	62.1	51.3	40.1	33.6	28.5	17.4	12.0	8.2	5.3	3.1	1.4	0.8
**Upper CI**	99.9	99.6	98.3	91.8	74.8	64.1	49.1	41.9	36.8	23.5	17.1	12.0	8.3	5.9	3.8	2.5
**Number**	347	345	337	308	252	219	182	157	136	107	85	64	47	34	18	12

Table W5124-13 Age Group = 18-40 Gender = OVERALL Occupational group = MANUAL Total cases = 400

Threshold dB HL≥	0	5	10	15	20	25	30	35	40	45	50	55	65	75	85	95
**Prevalence**	**96.5**	**77.0**	**43.7**	**27.2**	**13.8**	**8.1**	**7.0**	**4.3**	**2.3**	**2.1**	**1.4**	**1.1**	**0.6**	**0.4**	**0.4**	**0.3**
**Lower CI**	92.3	71.0	37.5	22.1	10.5	5.9	5.0	2.8	1.4	1.2	0.8	0.6	0.2	0.1	0.1	0.1
**Upper CI**	98.4	82.1	50.2	33.0	18.1	10.9	9.9	6.6	3.8	3.7	2.5	2.2	1.8	1.7	1.7	1.8
**Number**	391	327	213	147	96	67	54	38	26	22	17	14	6	4	4	2

Table W5124-14 Age Group = 41-60 Gender = OVERALL Occupational group = MANUAL Total cases = 621

Threshold dB HL≥	0	5	10	15	20	25	30	35	40	45	50	55	65	75	85	95
**Prevalence**	**100.0**	**95.9**	**81.0**	**61.0**	**42.5**	**31.6**	**22.0**	**15.9**	**12.9**	**9.9**	**8.0**	**6.3**	**3.5**	**2.3**	**1.1**	**0.8**
**Lower CI**	99.4	93.3	77.1	56.3	38.3	27.9	19.0	13.5	10.8	8.1	6.4	4.9	2.5	1.5	0.7	0.4
**Upper CI**	100.0	97.5	84.5	65.5	46.9	35.6	25.4	18.7	15.4	12.1	10.1	8.1	4.8	3.5	1.9	1.5
**Number**	621	604	532	440	346	273	211	166	137	115	95	76	48	33	18	12

Prevalence by age group, gender and occupational group for the worse ear over 0.5, 1, 2 and 4 kHz

Table W5124-22 Age Group = 18-40 Gender = FEMALE Occupational group = MANUAL Total cases = 211

Threshold dB HL≥	0	5	10	15	20	25	30	35	40	45	50	55	65	75	85	95
**Prevalence**	**97.1**	**76.6**	**40.8**	**20.7**	**10.5**	**6.9**	**6.0**	**4.2**	**2.5**	**2.4**	**1.3**	**0.9**	**0.3**	**0.2**	**0.2**	**0.0**
Lower CI	90.7	68.0	32.5	15.0	7.4	4.5	3.7	2.3	1.3	1.2	0.7	0.4	0.1	0.1	0.1	0.0
Upper CI	99.2	83.4	49.6	27.9	14.8	10.5	9.6	7.5	4.9	4.9	2.7	2.0	0.9	0.6	0.6	1.8
Number	208	172	109	68	46	31	25	17	13	12	9	7	3	2	2	0

Table W5124-23 Age Group = 41-60 Gender = FEMALE Occupational group = MANUAL Total cases = 299

Threshold dB HL≥	0	5	10	15	20	25	30	35	40	45	50	55	65	75	85	95
**Prevalence**	**100.0**	**94.4**	**74.4**	**56.0**	**33.6**	**22.8**	**15.9**	**10.8**	**8.6**	**5.5**	**4.6**	**3.7**	**2.5**	**1.4**	**0.7**	**0.5**
Lower CI	98.7	90.3	68.3	49.4	28.0	18.1	12.1	8.1	6.3	3.9	3.2	2.5	1.7	0.8	0.3	0.2
Upper CI	100.0	96.9	79.7	62.4	39.6	28.2	20.6	14.4	11.7	7.7	6.7	5.3	3.7	2.2	1.5	1.1
Number	299	287	236	190	135	97	76	60	48	38	33	28	21	14	7	5

Table W5124-24 Age Group = 61-80 Gender = FEMALE Occupational group = MANUAL Total cases = 198

Threshold dB HL≥	0	5	10	15	20	25	30	35	40	45	50	55	65	75	85	95
**Prevalence**	**100.0**	**99.7**	**93.4**	**81.8**	**74.7**	**51.2**	**38.7**	**35.5**	**29.1**	**24.7**	**19.8**	**14.6**	**8.8**	**4.6**	**2.8**	**2.1**
Lower CI	98.1	98.4	86.6	74.1	67.0	42.4	32.0	29.8	23.2	19.3	14.5	10.9	6.1	3.3	1.9	1.4
Upper CI	100.0	100.0	96.8	87.5	81.1	59.9	45.8	41.7	35.9	31.2	26.4	19.3	12.6	6.3	4.2	3.0
Number	198	197	188	173	159	134	107	102	91	78	68	56	34	20	10	7

Table W5124-25 Age Group = 18-40 Gender = MALE Occupational group = MANUAL Total cases = 189

Threshold dB HL≥	0	5	10	15	20	25	30	35	40	45	50	55	65	75	85	95
**Prevalence**	**95.8**	**77.4**	**46.7**	**34.2**	**17.4**	**9.3**	**8.1**	**4.4**	**2.0**	**1.7**	**1.4**	**1.3**	**0.8**	**0.7**	**0.7**	**0.7**
Lower CI	88.9	68.5	37.5	25.9	11.6	5.9	4.9	2.3	1.0	0.7	0.5	0.5	0.2	0.1	0.1	0.1
Upper CI	98.5	84.4	56.2	43.6	25.3	14.2	13.0	8.2	4.2	3.9	3.7	3.6	3.6	3.6	3.6	3.6
Number	183	155	104	79	50	36	29	21	13	10	8	7	3	2	2	2

Table W5124-26 Age Group = 41-60 Gender = MALE Occupational group = MANUAL Total cases = 322

Threshold dB HL≥	0	5	10	15	20	25	30	35	40	45	50	55	65	75	85	95
**Prevalence**	**100.0**	**97.5**	**88.3**	**66.5**	**52.2**	**41.4**	**28.7**	**21.4**	**17.5**	**14.7**	**11.8**	**9.2**	**4.5**	**3.3**	**1.5**	**1.1**
Lower CI	98.8	93.6	82.9	59.7	46.0	35.6	24.2	17.5	14.1	11.5	8.9	6.7	2.9	1.9	0.8	0.5
Upper CI	100.0	99.0	92.2	72.6	58.4	47.4	33.8	25.8	21.5	18.6	15.4	12.5	7.1	5.8	3.0	2.5
Number	322	317	296	250	211	176	135	106	89	77	62	48	27	19	11	7

Table W5124-27 Age Group = 61-80 Gender = MALE Occupational group = MANUAL Total cases = 244

Threshold dB HL≥	0	5	10	15	20	25	30	35	40	45	50	55	65	75	85	95
**Prevalence**	**100.0**	**99.2**	**99.2**	**95.2**	**81.8**	**73.3**	**60.1**	**49.0**	**39.7**	**32.2**	**24.9**	**19.5**	**13.3**	**7.3**	**4.6**	**3.0**
Lower CI	98.5	95.6	95.6	90.8	72.9	64.2	51.4	41.6	32.8	25.6	18.9	14.9	9.4	5.3	3.0	1.9
Upper CI	100.0	99.9	99.9	97.6	88.3	80.7	68.1	56.5	47.2	39.7	32.2	24.9	18.4	10.0	7.1	4.8
Number	244	243	243	236	219	203	181	156	137	113	94	80	55	35	21	14

Prevalence by age group, gender and occupational group for the worse ear over 0.5, 1, 2 and 4 kHz

Table W5124-15 Age Group = 61-80 Gender = OVERALL Occupational group = MANUAL Total cases = 442

	Threshold dB HL≥															
	**0**	**5**	**10**	**15**	**20**	**25**	**30**	**35**	**40**	**45**	**50**	**55**	**65**	**75**	**85**	**95**
**Prevalence**	**100.0**	**99.5**	**95.9**	**87.7**	**78.0**	**61.0**	**48.1**	**41.5**	**33.8**	**28.0**	**22.0**	**16.7**	**10.8**	**5.7**	**3.6**	**2.5**
**Lower CI**	99.1	98.0	92.0	83.1	72.3	54.7	42.7	36.8	29.1	23.6	17.8	13.7	8.3	4.5	2.7	1.8
**Upper CI**	100.0	99.9	98.0	91.2	82.7	67.0	53.5	46.3	38.8	32.8	26.8	20.1	13.8	7.2	4.8	3.4
**Number**	442	440	431	409	378	337	288	258	228	191	162	136	89	55	31	21

Table W5124-16 Age Group = 18-40 Gender = FEMALE Occupational group = NON-MANUAL Total cases = 201

	Threshold dB HL≥															
	**0**	**5**	**10**	**15**	**20**	**25**	**30**	**35**	**40**	**45**	**50**	**55**	**65**	**75**	**85**	**95**
**Prevalence**	**95.1**	**62.8**	**26.3**	**12.3**	**8.2**	**5.6**	**4.7**	**3.5**	**2.5**	**1.8**	**0.7**	**0.7**	**0.2**	**0.2**	**0.2**	**0.2**
**Lower CI**	91.2	55.3	20.7	8.6	5.4	3.6	2.8	1.8	1.2	0.9	0.2	0.2	0.0	0.0	0.0	0.0
**Upper CI**	97.3	69.7	32.9	17.3	12.4	8.8	7.6	6.4	5.0	3.7	2.1	2.1	1.2	1.2	1.2	1.2
**Number**	191	135	65	34	24	19	16	12	9	7	3	3	1	1	1	1

Table W5124-17 Age Group = 41-60 Gender = FEMALE Occupational group = NON-MANUAL Total cases = 284

	Threshold dB HL≥															
	**0**	**5**	**10**	**15**	**20**	**25**	**30**	**35**	**40**	**45**	**50**	**55**	**65**	**75**	**85**	**95**
**Prevalence**	**99.7**	**94.2**	**71.5**	**42.9**	**26.6**	**19.9**	**10.2**	**8.0**	**5.3**	**4.6**	**3.7**	**2.8**	**2.3**	**1.5**	**1.2**	**1.1**
**Lower CI**	98.6	90.8	65.9	37.2	22.2	16.1	7.7	6.0	3.7	3.1	2.3	1.8	1.3	0.7	0.5	0.4
**Upper CI**	100.0	96.4	76.5	48.9	31.4	24.4	13.3	10.6	7.4	6.8	5.7	4.5	4.2	3.1	2.9	2.8
**Number**	283	268	209	141	96	75	50	41	30	25	21	17	14	10	7	6

Table W5124-18 Age Group = 61-80 Gender = FEMALE Occupational group = NON-MANUAL Total cases = 183

	Threshold dB HL≥															
	**0**	**5**	**10**	**15**	**20**	**25**	**30**	**35**	**40**	**45**	**50**	**55**	**65**	**75**	**85**	**95**
**Prevalence**	**99.4**	**97.7**	**95.0**	**85.8**	**64.5**	**54.7**	**44.1**	**36.9**	**32.5**	**18.3**	**11.4**	**10.0**	**7.8**	**5.0**	**2.5**	**1.6**
**Lower CI**	96.7	93.4	89.9	79.3	55.5	45.9	37.7	31.3	27.2	15.3	9.2	7.8	5.7	3.5	1.3	0.8
**Upper CI**	99.9	99.2	97.6	90.5	72.7	63.1	50.6	42.8	38.3	21.8	14.0	12.9	10.7	7.1	4.5	3.5
**Number**	182	180	175	158	126	111	97	84	76	62	48	43	33	24	12	8

Table W5124-19 Age Group = 18-40 Gender = MALE Occupational group = NON-MANUAL Total cases = 128

	Threshold dB HL≥															
	**0**	**5**	**10**	**15**	**20**	**25**	**30**	**35**	**40**	**45**	**50**	**55**	**65**	**75**	**85**	**95**
**Prevalence**	**89.7**	**65.5**	**32.8**	**17.4**	**9.1**	**7.1**	**3.2**	**2.3**	**0.6**	**0.6**	**0.1**	**0.1**	**0.1**	**0.0**	**0.0**	**0.0**
**Lower CI**	80.2	54.9	23.6	10.6	4.8	3.3	1.3	0.8	0.2	0.2	0.0	0.0	0.0	0.0	0.0	0.0
**Upper CI**	95.0	74.8	43.4	27.1	16.8	14.7	7.5	6.3	1.3	1.3	3.1	3.1	3.1	2.9	2.9	2.9
**Number**	121	95	50	24	17	13	8	6	3	3	1	1	1	0	0	0

Table W5124-20 Age Group = 41-60 Gender = MALE Occupational group = NON-MANUAL Total cases = 218

	Threshold dB HL≥															
	**0**	**5**	**10**	**15**	**20**	**25**	**30**	**35**	**40**	**45**	**50**	**55**	**65**	**75**	**85**	**95**
**Prevalence**	**99.2**	**91.0**	**71.0**	**47.7**	**37.3**	**28.2**	**18.0**	**13.6**	**9.4**	**7.4**	**6.2**	**4.9**	**3.1**	**2.2**	**1.0**	**1.0**
**Lower CI**	97.0	85.3	63.7	41.1	31.4	23.3	12.6	9.3	6.5	4.9	3.9	2.7	1.9	1.3	0.4	0.4
**Upper CI**	99.8	94.6	77.4	54.5	43.6	33.7	25.2	19.4	13.6	11.0	9.7	8.6	5.0	3.7	2.3	2.3
**Number**	216	201	163	119	96	74	53	45	36	29	24	19	14	10	6	6

Table W5124-21 Age Group = 61-80 Gender = MALE Occupational group = NON-MANUAL Total cases = 165

	Threshold dB HL≥															
	**0**	**5**	**10**	**15**	**20**	**25**	**30**	**35**	**40**	**45**	**50**	**55**	**65**	**75**	**85**	**95**
**Prevalence**	**100.0**	**100.0**	**98.9**	**92.3**	**74.3**	**61.8**	**45.0**	**38.4**	**32.5**	**22.8**	**18.3**	**10.1**	**5.2**	**3.5**	**2.2**	**1.2**
**Lower CI**	97.7	97.7	96.0	87.4	64.0	51.9	39.3	32.8	27.0	18.0	14.0	7.7	4.1	1.9	1.0	0.5
**Upper CI**	100.0	100.0	99.7	95.4	82.4	70.8	50.9	44.4	38.6	28.6	23.5	13.2	6.6	6.3	5.1	2.9
**Number**	165	165	162	150	126	108	85	73	60	45	37	21	14	10	6	4

Prevalence by age group, gender and occupational group for the worse ear over 0.5,1, 2 kHz

Table W512-1 Age Group = 18-40 Gender = OVERALL Occupational group = OVERALL Total cases = 732

	Threshold dB HL≥															
	0	5	10	15	20	25	30	35	40	45	50	55	65	75	85	95
Prevalence	91.8	63.8	32.8	16.1	9.8	6.0	4.7	3.4	1.8	1.6	1.2	1.0	0.5	0.3	0.3	0.2
Lower CI	88.8	59.5	28.9	13.4	7.8	4.6	3.6	2.5	1.2	1.0	0.7	0.6	0.2	0.1	0.1	0.0
Upper CI	94.0	67.9	36.9	19.2	12.2	7.8	6.2	4.7	2.7	2.5	2.0	1.7	1.2	1.1	1.1	1.1
Number	685	512	292	172	123	85	70	53	34	27	22	18	8	5	3	2

Table W512-2 Age Group = 41-60 Gender = OVERALL Occupational group = OVERALL Total cases = 1124

	Threshold dB HL≥															
	0	5	10	15	20	25	30	35	40	45	50	55	65	75	85	95
Prevalence	99.3	86.5	63.2	40.9	26.9	19.1	14.4	11.5	8.9	6.7	5.5	4.4	2.8	1.7	1.2	0.9
Lower CI	98.5	84.0	60.1	38.0	24.5	17.1	12.6	10.0	7.6	5.6	4.5	3.5	2.1	1.2	0.7	0.5
Upper CI	99.7	88.6	66.3	44.0	29.5	21.3	16.3	13.3	10.5	8.0	6.7	5.5	3.7	2.4	1.8	1.4
Number	1117	1000	782	562	417	322	260	211	171	141	116	98	65	42	29	22

Table W512-3 Age Group = 61-80 Gender = OVERALL Occupational group = OVERALL Total cases = 793

	Threshold dB HL≥															
	0	5	10	15	20	25	30	35	40	45	50	55	65	75	85	95
Prevalence	99.9	97.5	87.0	72.3	56.0	44.1	34.9	30.3	23.3	17.7	14.4	11.6	8.5	4.3	2.3	1.7
Lower CI	99.2	95.9	83.7	68.4	51.9	40.3	31.3	27.0	20.3	15.3	12.4	9.8	6.8	3.4	1.7	1.2
Upper CI	100.0	98.4	89.7	75.9	60.1	48.0	38.7	33.9	26.7	20.4	16.8	13.8	10.4	5.3	3.1	2.4
Number	792	774	711	619	523	443	380	341	277	231	196	166	121	71	41	29

Table W512-4 Age Group = 18-40 Gender = FEMALE Occupational group = OVERALL Total cases = 413

	Threshold dB HL≥															
	0	5	10	15	20	25	30	35	40	45	50	55	65	75	85	95
Prevalence	91.9	64.1	32.5	14.2	9.2	6.1	5.3	4.2	2.2	2.0	1.4	1.2	0.3	0.2	0.1	0.0
Lower CI	88.0	58.5	27.7	11.3	7.0	4.4	3.7	2.8	1.4	1.2	0.8	0.6	0.1	0.1	0.0	0.0
Upper CI	94.6	69.3	37.7	17.8	11.9	8.5	7.5	6.2	3.5	3.4	2.4	2.1	0.9	0.7	0.7	0.9
Number	388	290	165	92	68	46	39	32	21	18	14	12	4	3	1	0

Table W512-5 Age Group = 41-60 Gender = FEMALE Occupational group = OVERALL Total cases = 584

	Threshold dB HL≥															
	0	5	10	15	20	25	30	35	40	45	50	55	65	75	85	95
Prevalence	98.9	87.5	62.9	39.0	23.8	16.7	11.8	9.3	7.0	5.3	4.2	3.3	2.3	1.2	1.0	0.8
Lower CI	97.4	84.3	58.6	35.1	20.6	14.1	9.6	7.4	5.4	4.1	3.1	2.4	1.5	0.7	0.5	0.4
Upper CI	99.5	90.2	66.9	43.1	27.2	19.7	14.5	11.7	9.0	6.9	5.7	4.7	3.5	2.2	2.0	1.8
Number	579	523	403	279	194	148	114	92	76	65	52	44	31	18	14	10

Table W512-6 Age Group = 61-80 Gender = FEMALE Occupational group = OVERALL Total cases = 382

	Threshold dB HL≥															
	0	5	10	15	20	25	30	35	40	45	50	55	65	75	85	95
Prevalence	99.8	97.4	86.5	72.4	53.6	40.8	32.1	30.4	24.2	17.7	14.4	11.8	8.6	4.3	2.0	1.7
Lower CI	98.7	95.0	81.6	67.0	47.7	35.6	27.2	25.7	19.7	14.4	11.8	9.2	6.4	3.2	1.3	1.1
Upper CI	100.0	98.6	90.2	77.2	59.4	46.2	37.3	35.7	29.3	21.5	17.4	14.9	11.4	5.7	3.0	2.8
Number	381	372	340	297	245	204	179	170	140	117	105	89	64	37	18	16

Table W512-7 Age Group = 18-40 Gender = MALE Occupational group = OVERALL Total cases = 319

	Threshold dB HL≥															
	0	5	10	15	20	25	30	35	40	45	50	55	65	75	85	95
Prevalence	91.6	63.3	33.0	18.2	10.5	5.8	4.0	2.4	1.4	1.0	1.0	0.8	0.6	0.4	0.4	0.4
Lower CI	86.7	56.5	27.1	13.8	7.3	3.8	2.5	1.4	0.7	0.4	0.4	0.3	0.2	0.1	0.1	0.1
Upper CI	94.9	69.6	39.5	23.6	14.8	8.7	6.3	4.1	2.8	2.5	2.5	2.3	2.2	2.3	2.3	2.3
Number	297	222	127	80	55	39	31	21	13	9	8	6	4	2	2	2

Prevalence by age group, gender and occupational group for the worse ear over 0.5,1, 2 kHz

Table W512-8 Age Group = 41-60 Gender = MALE Occupational group = OVERALL Total cases = 540

Threshold dB HL≥	0	5	10	15	20	25	30	35	40	45	50	55	65	75	85	95
Prevalence	**99.7**	**85.2**	**63.6**	**43.0**	**30.3**	**21.7**	**17.2**	**14.0**	**11.1**	**8.2**	**6.9**	**5.6**	**3.3**	**2.2**	**1.3**	**0.9**
Lower CI	98.7	81.3	58.8	38.6	26.7	18.7	14.6	11.6	8.9	6.4	5.2	4.1	2.2	1.4	0.7	0.5
Upper CI	99.9	88.4	68.1	47.5	34.3	25.2	20.2	16.8	13.7	10.4	9.0	7.5	4.9	3.5	2.3	1.7
Number	538	477	379	283	223	174	146	119	95	76	64	54	34	24	15	12

Table W512-9 Age Group = 61-80 Gender = MALE Occupational group = OVERALL Total cases = 411

Threshold dB HL≥	0	5	10	15	20	25	30	35	40	45	50	55	65	75	85	95
Prevalence	**100.0**	**97.6**	**87.5**	**72.1**	**59.0**	**48.0**	**38.5**	**30.1**	**22.4**	**17.7**	**14.7**	**11.5**	**8.3**	**4.3**	**2.8**	**1.6**
Lower CI	99.1	95.1	83.0	66.2	53.5	42.7	33.3	25.9	18.7	14.5	11.6	9.1	6.2	3.0	1.8	0.9
Upper CI	100.0	98.8	91.0	77.3	64.4	53.5	43.9	34.8	26.7	21.5	18.4	14.4	11.1	6.0	4.3	2.8
Number	411	402	371	322	278	239	201	171	137	114	91	77	57	34	23	13

Table W512-10 Age Group = 18-40 Gender = OVERALL Occupational group = NON-MANUAL Total cases = 329

Threshold dB HL≥	0	5	10	15	20	25	30	35	40	45	50	55	65	75	85	95
Prevalence	**87.3**	**58.8**	**24.4**	**10.1**	**6.8**	**4.4**	**3.4**	**2.9**	**1.2**	**0.9**	**0.7**	**0.4**	**0.1**	**0.1**	**0.1**	**0.0**
Lower CI	82.3	52.3	19.5	7.1	4.7	2.9	2.1	1.7	0.6	0.4	0.3	0.1	0.0	0.0	0.0	0.0
Upper CI	91.1	65.0	30.0	14.2	9.6	6.6	5.6	5.1	2.2	1.9	1.8	1.1	0.6	0.6	0.6	1.2
Number	296	210	98	47	36	27	21	18	10	7	5	4	2	1	1	0

Table W512-11 Age Group = 41-60 Gender = OVERALL Occupational group = NON-MANUAL Total cases = 502

Threshold dB HL≥	0	5	10	15	20	25	30	35	40	45	50	55	65	75	85	95
Prevalence	**99.4**	**83.7**	**57.7**	**36.3**	**23.2**	**15.2**	**11.5**	**8.2**	**6.5**	**5.2**	**4.3**	**2.8**	**2.4**	**1.5**	**1.1**	**1.0**
Lower CI	98.3	79.6	53.1	32.2	20.0	12.7	9.0	6.3	4.9	3.8	3.0	2.0	1.6	0.9	0.6	0.5
Upper CI	99.8	87.0	62.2	40.7	26.9	18.1	14.6	10.5	8.7	7.1	6.3	4.0	3.6	2.5	2.0	2.0
Number	499	429	310	209	152	113	90	68	57	47	38	29	24	18	13	11

Table W512-12 Age Group = 61-80 Gender = OVERALL Occupational group = NON-MANUAL Total cases = 348

Threshold dB HL≥	0	5	10	15	20	25	30	35	40	45	50	55	65	75	85	95
Prevalence	**99.7**	**97.7**	**86.5**	**68.6**	**54.4**	**43.0**	**34.1**	**29.1**	**21.8**	**12.5**	**9.9**	**7.8**	**5.9**	**3.5**	**2.2**	**1.4**
Lower CI	98.1	95.2	81.0	62.0	48.1	37.6	29.2	25.2	18.5	10.4	7.9	6.4	4.4	2.4	1.3	0.8
Upper CI	99.9	99.0	90.6	74.5	60.5	48.5	39.4	33.4	25.4	15.0	12.4	9.5	7.8	5.0	3.7	2.5
Number	347	340	305	248	205	170	143	127	99	79	65	57	42	26	17	12

Table W512-13 Age Group = 18-40 Gender = OVERALL Occupational group = MANUAL Total cases = 400

Threshold dB HL≥	0	5	10	15	20	25	30	35	40	45	50	55	65	75	85	95
Prevalence	**96.0**	**69.7**	**40.1**	**20.7**	**11.5**	**7.1**	**5.5**	**3.2**	**2.2**	**2.0**	**1.5**	**1.3**	**0.6**	**0.4**	**0.3**	**0.3**
Lower CI	92.4	63.4	33.9	16.4	8.4	5.0	3.8	2.1	1.3	1.1	0.8	0.7	0.2	0.1	0.1	0.1
Upper CI	98.0	75.4	46.5	25.9	15.4	9.9	8.0	5.0	3.8	3.6	2.6	2.4	1.8	1.7	1.8	1.8
Number	386	301	193	124	86	57	48	34	24	20	17	14	6	4	2	2

Table W512-14 Age Group = 41-60 Gender = OVERALL Occupational group = MANUAL Total cases = 622

Threshold dB HL≥	0	5	10	15	20	25	30	35	40	45	50	55	65	75	85	95
Prevalence	**99.3**	**88.4**	**68.5**	**45.4**	**29.7**	**22.4**	**16.9**	**13.7**	**10.5**	**7.7**	**6.3**	**5.3**	**2.9**	**1.5**	**1.0**	**0.6**
Lower CI	98.2	84.8	63.8	41.0	26.2	19.3	14.2	11.4	8.6	6.1	4.9	4.0	2.0	1.0	0.6	0.3
Upper CI	99.7	91.3	72.8	49.9	33.6	26.0	19.9	16.4	12.9	9.6	8.1	6.9	4.2	2.3	1.7	1.3
Number	618	571	472	353	265	209	170	143	114	94	78	69	41	24	16	11

Prevalence by age group, gender and occupational group for the worse ear over 0.5,1, 2 kHz

Table W512-15 Age Group = 61-80 Gender = OVERALL Occupational group = MANUAL Total cases = 443

	Threshold dB HL≥															
	0	5	10	15	20	25	30	35	40	45	50	55	65	75	85	95
Prevalence	**100.0**	**96.8**	**86.7**	**75.6**	**58.2**	**45.8**	**36.0**	**31.4**	**25.5**	**21.1**	**16.7**	**13.4**	**9.8**	**4.9**	**2.7**	**2.2**
Lower CI	99.1	94.0	81.7	69.7	52.1	40.6	31.5	27.2	21.2	17.2	13.7	10.6	7.5	3.8	2.0	1.6
Upper CI	100.0	98.4	90.5	80.6	64.1	51.1	40.8	35.9	30.3	25.4	20.2	16.9	12.8	6.4	3.6	3.1
Number	443	432	404	369	316	273	237	214	178	152	131	109	79	45	24	17

Table W512-16 Age Group = 18-40 Gender = FEMALE Occupational group = NON-MANUAL Total cases = 201

	Threshold dB HL≥															
	0	5	10	15	20	25	30	35	40	45	50	55	65	75	85	95
Prevalence	**88.3**	**59.4**	**26.5**	**10.9**	**8.0**	**5.4**	**4.3**	**4.0**	**1.9**	**1.7**	**1.2**	**0.7**	**0.2**	**0.2**	**0.2**	**0.0**
Lower CI	82.2	51.9	20.7	7.5	5.2	3.3	2.5	2.2	0.9	0.8	0.4	0.2	0.0	0.0	0.0	0.0
Upper CI	92.4	66.4	33.2	15.6	12.1	8.5	7.4	7.1	3.8	3.6	3.3	2.1	1.2	1.2	1.2	1.9
Number	181	127	62	31	24	18	15	14	8	6	4	3	1	1	1	0

Table W512-17 Age Group = 41-60 Gender = FEMALE Occupational group = NON-MANUAL Total cases = 284

	Threshold dB HL≥															
	0	5	10	15	20	25	30	35	40	45	50	55	65	75	85	95
Prevalence	**98.9**	**87.0**	**60.1**	**36.0**	**21.5**	**13.7**	**9.2**	**7.7**	**5.6**	**4.3**	**3.5**	**2.4**	**1.8**	**1.4**	**1.2**	**1.0**
Lower CI	96.7	82.6	54.4	30.6	17.5	10.8	6.8	5.6	3.9	2.9	2.1	1.4	0.9	0.6	0.5	0.3
Upper CI	99.6	90.4	65.5	41.8	26.1	17.3	12.3	10.5	7.9	6.5	5.6	4.2	3.6	3.0	2.9	2.8
Number	281	249	182	118	80	59	44	36	30	24	18	14	11	9	7	5

Table W512-18 Age Group = 61-80 Gender = FEMALE Occupational group = NON-MANUAL Total cases = 183

	Threshold dB HL≥															
	0	5	10	15	20	25	30	35	40	45	50	55	65	75	85	95
Prevalence	**99.4**	**97.0**	**84.9**	**69.1**	**55.0**	**42.7**	**32.6**	**31.4**	**25.5**	**12.0**	**10.7**	**9.4**	**7.8**	**3.7**	**2.3**	**1.8**
Lower CI	96.7	92.7	76.2	60.7	46.5	36.5	27.4	26.4	21.1	9.8	8.6	7.2	5.6	2.3	1.2	0.9
Upper CI	99.9	98.8	90.8	76.4	63.3	49.1	38.3	36.9	30.4	14.6	13.3	12.1	10.7	5.8	4.4	3.7
Number	182	178	161	133	111	93	80	76	62	51	46	42	32	17	11	9

Table W512-19 Age Group = 18-40 Gender = MALE Occupational group = NON-MANUAL Total cases = 128

	Threshold dB HL≥															
	0	5	10	15	20	25	30	35	40	45	50	55	65	75	85	95
Prevalence	**86.1**	**57.9**	**22.0**	**9.3**	**5.4**	**3.1**	**2.3**	**1.7**	**0.3**	**0.1**	**0.1**	**0.1**	**0.1**	**0.0**	**0.0**	**0.0**
Lower CI	77.2	47.1	14.7	4.9	2.8	1.4	0.8	0.5	0.1	0.0	0.0	0.0	0.0	0.0	0.0	0.0
Upper CI	91.9	68.0	31.7	16.9	10.2	6.8	6.3	5.7	1.3	3.1	3.1	3.1	3.1	2.9	2.9	2.9
Number	115	83	36	16	12	9	6	4	2	1	1	1	1	0	0	0

Table W512-20 Age Group = 41-60 Gender = MALE Occupational group = NON-MANUAL Total cases = 218

	Threshold dB HL≥															
	0	5	10	15	20	25	30	35	40	45	50	55	65	75	85	95
Prevalence	**100.0**	**79.8**	**54.9**	**36.6**	**25.3**	**16.9**	**14.1**	**8.8**	**7.5**	**6.2**	**5.3**	**3.2**	**3.0**	**1.6**	**1.0**	**1.0**
Lower CI	98.3	72.4	47.5	30.5	20.1	13.0	9.7	5.9	4.8	3.8	3.0	2.0	1.8	0.8	0.4	0.4
Upper CI	100.0	85.5	62.1	43.2	31.2	21.7	20.0	12.9	11.7	9.9	9.1	5.1	4.9	3.2	2.3	2.3
Number	218	180	128	91	72	54	46	32	27	23	20	15	13	9	6	6

Table W512-21 Age Group = 61-80 Gender = MALE Occupational group = NON-MANUAL Total cases = 165

	Threshold dB HL≥															
	0	5	10	15	20	25	30	35	40	45	50	55	65	75	85	95
Prevalence	**100.0**	**98.7**	**88.5**	**67.9**	**53.4**	**43.4**	**36.3**	**26.5**	**17.7**	**13.2**	**8.9**	**5.8**	**3.5**	**3.3**	**2.2**	**0.9**
Lower CI	97.7	95.7	82.3	57.2	44.2	34.2	27.6	20.8	13.4	9.8	5.7	4.3	1.9	1.7	1.0	0.3
Upper CI	100.0	99.6	92.7	76.9	62.4	53.0	46.1	33.1	23.1	17.7	13.7	7.7	6.3	6.1	5.1	2.2
Number	165	162	144	115	94	77	63	51	37	28	19	15	10	9	6	3

Prevalence by age group, gender and occupational group for the worse ear over 0.5,1, 2 kHz

Table W512-22 Age Group = 18-40 Gender = FEMALE Occupational group = MANUAL Total cases = 211

	Threshold dB HL≥															
	0	5	10	15	20	25	30	35	40	45	50	55	65	75	85	95
Prevalence	96.6	70.4	39.0	17.1	9.5	6.4	5.8	3.8	2.5	2.4	1.5	1.3	0.3	0.2	0.0	0.0
Lower CI	90.5	61.7	30.8	12.4	6.5	4.0	3.5	2.1	1.3	1.2	0.8	0.7	0.1	0.1	0.0	0.0
Upper CI	98.8	77.8	47.8	23.2	13.6	10.0	9.3	6.7	4.9	4.9	2.9	2.7	0.9	0.6	1.8	1.8
Number	206	162	102	60	43	27	23	17	13	12	10	9	3	2	0	0

Table W512-23 Age Group = 41-60 Gender = FEMALE Occupational group = MANUAL Total cases = 300

	Threshold dB HL≥															
	0	5	10	15	20	25	30	35	40	45	50	55	65	75	85	95
Prevalence	99.1	88.9	66.4	42.7	26.4	19.3	14.3	10.9	8.4	5.9	4.7	3.6	2.3	0.9	0.7	0.5
Lower CI	96.8	83.8	59.8	36.6	21.4	15.1	10.7	8.0	5.9	4.2	3.2	2.5	1.5	0.5	0.4	0.2
Upper CI	99.7	92.6	72.5	48.9	32.0	24.4	18.7	14.8	11.7	8.2	6.7	5.1	3.5	1.6	1.5	1.0
Number	298	274	221	161	114	89	70	56	46	41	34	30	20	9	7	5

Table W512-24 Age Group = 61-80 Gender = FEMALE Occupational group = MANUAL Total cases = 198

	Threshold dB HL≥															
	0	5	10	15	20	25	30	35	40	45	50	55	65	75	85	95
Prevalence	100.0	97.4	87.3	75.6	52.2	40.9	32.3	29.9	24.5	21.0	15.6	12.2	8.4	4.6	2.1	2.1
Lower CI	98.1	93.2	80.5	67.8	44.0	33.8	26.7	24.8	19.0	15.6	11.8	8.6	5.7	3.3	1.4	1.4
Upper CI	100.0	99.1	91.9	82.0	60.4	48.4	38.5	35.7	31.1	27.5	20.3	17.1	12.1	6.3	3.0	3.0
Number	198	193	178	163	133	111	99	94	78	66	59	47	32	20	7	7

Table W512-25 Age Group = 18-40 Gender = MALE Occupational group = MANUAL Total cases = 189

	Threshold dB HL≥															
	0	5	10	15	20	25	30	35	40	45	50	55	65	75	85	95
Prevalence	95.5	68.7	41.0	24.7	13.7	7.7	5.2	2.6	1.9	1.5	1.4	1.2	0.9	0.7	0.7	0.7
Lower CI	90.5	59.1	32.1	17.6	8.6	4.6	2.9	1.4	0.8	0.6	0.5	0.4	0.2	0.1	0.1	0.1
Upper CI	98.0	77.0	50.6	33.5	21.2	12.7	9.2	4.7	4.1	3.7	3.7	3.6	3.6	3.6	3.6	3.6
Number	180	139	91	64	43	30	25	17	11	8	7	5	3	2	2	2

Table W512-26 Age Group = 41-60 Gender = MALE Occupational group = MANUAL Total cases = 322

	Threshold dB HL≥															
	0	5	10	15	20	25	30	35	40	45	50	55	65	75	85	95
Prevalence	99.5	87.9	70.7	48.3	33.4	25.7	19.7	16.7	12.9	9.6	8.1	7.1	3.5	2.1	1.3	0.9
Lower CI	98.2	82.4	63.9	42.0	28.4	21.2	16.0	13.3	10.0	7.1	5.8	4.9	2.1	1.2	0.6	0.3
Upper CI	99.9	91.8	76.7	54.7	38.8	30.8	24.0	20.7	16.6	12.9	11.2	10.2	6.0	3.7	2.7	2.1
Number	320	297	251	192	151	120	100	87	68	53	44	39	21	15	9	6

Table W512-27 Age Group = 61-80 Gender = MALE Occupational group = MANUAL Total cases = 245

	Threshold dB HL≥															
	0	5	10	15	20	25	30	35	40	45	50	55	65	75	85	95
Prevalence	100.0	96.1	85.9	75.3	65.8	51.7	40.6	33.4	26.9	21.1	18.3	15.1	11.6	5.5	3.5	2.4
Lower CI	98.5	91.4	77.4	65.9	56.7	44.4	33.6	26.8	20.6	16.4	13.8	11.1	8.0	3.7	2.3	1.4
Upper CI	100.0	98.3	91.5	82.7	74.0	59.0	48.1	40.6	34.2	26.7	24.0	20.3	16.7	8.1	5.3	4.1
Number	245	239	226	206	183	162	138	120	100	86	72	62	47	25	17	10

Prevalence by age group, gender and occupational group for the worse ear over 4, 6 and 8 kHz

Table W468-1 Age Group = 18-40 Gender = OVERALL Occupational group = OVERALL Total cases = 728

	Threshold dB HL≥															
	0	5	10	15	20	25	30	35	40	45	50	55	65	75	85	95
Prevalence	**97.4**	**88.0**	**67.3**	**43.6**	**28.8**	**20.9**	**15.1**	**10.0**	**8.0**	**6.4**	**4.4**	**3.5**	**1.8**	**1.0**	**0.8**	**0.3**
Lower CI	95.6	84.7	63.1	39.6	25.3	17.7	12.4	7.9	6.1	4.7	3.0	2.2	1.0	0.5	0.3	0.1
Upper CI	98.5	90.7	71.2	47.7	32.7	24.5	18.4	12.7	10.5	8.7	6.4	5.4	3.3	2.1	1.9	1.1
Number	710	656	527	377	265	203	145	112	88	70	50	37	22	13	9	5

Table W468-2 Age Group = 41-60 Gender = OVERALL Occupational group = OVERALL Total cases = 1105

	Threshold dB HL≥															
	0	5	10	15	20	25	30	35	40	45	50	55	65	75	85	95
Prevalence	**100.0**	**99.4**	**94.7**	**85.8**	**73.2**	**60.4**	**48.6**	**39.0**	**32.1**	**26.6**	**20.9**	**17.0**	**11.0**	**6.5**	**3.5**	**2.3**
Lower CI	99.7	98.6	93.0	83.4	70.2	57.3	45.6	36.1	29.4	24.1	18.8	15.1	9.4	5.4	2.7	1.6
Upper CI	100.0	99.7	96.0	87.9	75.9	63.5	51.7	42.0	34.9	29.2	23.3	19.1	12.7	7.9	4.5	3.1
Number	1105	1099	1057	978	861	748	632	534	457	391	329	275	197	121	70	46

Table W468-3 Age Group = 61-80 Gender = OVERALL Occupational group = OVERALL Total cases = 782

	Threshold dB HL≥															
	0	5	10	15	20	25	30	35	40	45	50	55	65	75	85	95
Prevalence	**100.0**	**100.0**	**99.9**	**99.4**	**95.6**	**92.3**	**88.7**	**83.8**	**77.9**	**72.2**	**64.5**	**55.9**	**40.6**	**24.6**	**14.2**	**6.8**
Lower CI	99.5	99.5	99.4	98.4	93.4	89.5	85.6	80.1	74.1	68.2	60.4	51.5	36.5	21.6	11.9	5.5
Upper CI	100.0	100.0	100.0	99.8	97.1	94.4	91.2	86.9	81.3	75.8	68.4	60.3	44.9	27.9	16.8	8.5
Number	782	782	781	778	759	743	721	690	654	621	571	522	411	277	176	99

Table W468-4 Age Group = 18-40 Gender = FEMALE Occupational group = OVERALL Total cases = 410

	Threshold dB HL≥															
	0	5	10	15	20	25	30	35	40	45	50	55	65	75	85	95
Prevalence	**97.3**	**86.8**	**65.4**	**40.1**	**24.6**	**18.6**	**11.5**	**8.1**	**6.6**	**4.6**	**2.8**	**1.8**	**0.7**	**0.1**	**0.1**	**0.0**
Lower CI	94.9	82.4	59.9	35.1	20.4	14.9	8.7	5.8	4.6	3.0	1.7	1.0	0.3	0.0	0.0	0.0
Upper CI	98.6	90.2	70.5	45.3	29.3	22.9	15.0	11.2	9.4	7.0	4.6	3.2	1.6	0.5	0.5	0.9
Number	399	366	289	200	136	105	70	52	43	31	22	16	7	1	1	0

Table W468-5 Age Group = 41-60 Gender = FEMALE Occupational group = OVERALL Total cases = 579

	Threshold dB HL≥															
	0	5	10	15	20	25	30	35	40	45	50	55	65	75	85	95
Prevalence	**100.0**	**98.8**	**91.4**	**78.8**	**64.2**	**52.3**	**38.3**	**28.3**	**21.3**	**16.8**	**10.6**	**7.3**	**4.6**	**2.5**	**1.8**	**1.2**
Lower CI	99.3	97.3	88.5	74.8	59.9	48.0	34.4	24.7	18.2	14.1	8.7	5.7	3.5	1.8	1.2	0.7
Upper CI	100.0	99.5	93.7	82.2	68.4	56.5	42.5	32.1	24.8	19.9	13.0	9.2	6.0	3.6	2.6	1.9
Number	579	573	537	478	403	341	269	213	169	140	107	79	56	36	26	16

Table W468-6 Age Group = 61-80 Gender = FEMALE Occupational group = OVERALL Total cases = 377

	Threshold dB HL≥															
	0	5	10	15	20	25	30	35	40	45	50	55	65	75	85	95
Prevalence	**100.0**	**100.0**	**99.8**	**99.0**	**92.5**	**87.6**	**83.1**	**77.8**	**68.7**	**63.4**	**55.8**	**47.6**	**32.2**	**17.9**	**9.8**	**4.2**
Lower CI	99.0	99.0	98.9	97.1	88.6	82.9	78.1	72.1	62.8	57.5	50.1	41.4	26.7	14.3	7.3	3.0
Upper CI	100.0	100.0	100.0	99.7	95.1	91.2	87.1	82.5	74.0	68.8	61.4	53.9	38.4	22.3	13.0	5.9
Number	377	377	376	373	356	343	327	309	280	264	237	215	161	104	64	35

Table W468-7 Age Group = 18-40 Gender = MALE Occupational group = OVERALL Total cases = 318

	Threshold dB HL≥															
	0	5	10	15	20	25	30	35	40	45	50	55	65	75	85	95
Prevalence	**97.5**	**89.3**	**69.2**	**47.1**	**33.3**	**23.4**	**19.0**	**12.0**	**9.5**	**8.3**	**6.0**	**5.1**	**3.0**	**1.9**	**1.5**	**0.6**
Lower CI	94.3	84.0	62.7	40.9	27.5	18.3	14.3	8.6	6.4	5.4	3.6	2.8	1.4	0.9	0.6	0.2
Upper CI	98.9	93.1	75.0	53.4	39.6	29.3	24.8	16.6	13.9	12.6	9.9	9.0	6.0	4.0	3.7	2.2
Number	311	290	238	177	129	98	75	60	45	39	28	21	15	12	8	5

Prevalence by age group, gender and occupational group for the worse ear over 4, 6 and 8 kHz

Table W468-8 Age Group = 41-60 Gender = MALE Occupational group = OVERALL Total cases = 526

Threshold dB HL≥	0	5	10	15	20	25	30	35	40	45	50	55	65	75	85	95
**Prevalence**	**100.0**	**100.0**	**98.4**	**93.6**	**83.1**	**69.5**	**60.1**	**51.0**	**44.1**	**37.5**	**32.4**	**27.8**	**18.1**	**10.9**	**5.3**	**3.5**
Lower CI	99.3	99.3	96.3	90.6	78.9	64.9	55.4	46.3	39.7	33.4	28.4	24.2	15.1	8.7	3.8	2.3
Upper CI	100.0	100.0	99.3	95.8	86.5	73.8	64.6	55.6	48.6	41.9	36.5	31.8	21.5	13.7	7.4	5.2
Number	526	526	520	500	458	407	363	321	288	251	222	196	141	85	44	30

Table W468-9 Age Group = 61-80 Gender = MALE Occupational group = OVERALL Total cases = 405

Threshold dB HL≥	0	5	10	15	20	25	30	35	40	45	50	55	65	75	85	95
**Prevalence**	**100.0**	**100.0**	**100.0**	**100.0**	**99.6**	**98.3**	**95.8**	**91.5**	**89.7**	**83.5**	**75.6**	**66.6**	**51.5**	**33.0**	**19.7**	**10.2**
Lower CI	99.1	99.1	99.1	99.1	98.4	95.8	91.9	87.0	85.2	78.2	69.8	60.3	45.7	28.3	15.9	7.7
Upper CI	100.0	100.0	100.0	100.0	99.9	99.3	97.8	94.5	93.0	87.7	80.7	72.3	57.2	38.1	24.2	13.5
Number	405	405	405	405	403	400	394	381	374	357	334	307	250	173	112	64

Table W468-10 Age Group = 18-40 Gender = OVERALL Occupational group = NON-MANUAL Total cases = 327

Threshold dB HL≥	0	5	10	15	20	25	30	35	40	45	50	55	65	75	85	95
**Prevalence**	**97.3**	**85.7**	**62.4**	**35.3**	**22.4**	**14.9**	**11.3**	**9.2**	**8.4**	**6.8**	**5.1**	**4.7**	**2.2**	**0.7**	**0.6**	**0.0**
Lower CI	94.1	81.1	56.6	29.8	17.5	10.8	7.6	5.9	5.1	3.9	2.6	2.3	0.8	0.2	0.1	0.0
Upper CI	98.8	89.4	68.0	41.2	28.2	20.2	16.3	14.1	13.4	11.6	10.0	9.6	5.5	2.3	2.3	1.2
Number	320	283	216	132	85	60	44	35	28	22	13	11	8	4	2	0

Table W468-11 Age Group = 41-60 Gender = OVERALL Occupational group = NON-MANUAL Total cases = 492

Threshold dB HL≥	0	5	10	15	20	25	30	35	40	45	50	55	65	75	85	95
**Prevalence**	**100.0**	**99.7**	**93.2**	**82.8**	**68.3**	**56.2**	**42.5**	**33.4**	**27.1**	**23.5**	**19.6**	**15.6**	**8.0**	**4.9**	**1.8**	**1.3**
Lower CI	99.2	98.6	90.4	78.8	63.6	51.7	38.0	29.3	23.3	20.0	16.4	12.8	6.0	3.2	1.3	0.9
Upper CI	100.0	99.9	95.3	86.2	72.6	60.6	47.2	37.7	31.2	27.5	23.2	18.8	10.6	7.5	2.6	2.0
Number	492	490	463	415	350	300	238	193	160	140	119	99	62	40	20	14

Table W468-12 Age Group = 61-80 Gender = OVERALL Occupational group = NON-MANUAL Total cases = 347

Threshold dB HL≥	0	5	10	15	20	25	30	35	40	45	50	55	65	75	85	95
**Prevalence**	**100.0**	**100.0**	**99.5**	**98.9**	**95.9**	**91.8**	**87.9**	**83.2**	**75.7**	**70.3**	**61.6**	**54.2**	**36.8**	**19.1**	**12.0**	**5.8**
Lower CI	98.9	98.9	97.3	96.4	92.6	87.3	82.2	77.2	69.0	63.6	54.8	47.6	31.5	15.7	9.7	4.0
Upper CI	100.0	100.0	99.9	99.7	97.8	94.8	91.9	87.8	81.4	76.3	67.8	60.6	42.5	23.1	14.8	8.2
Number	347	347	346	344	336	326	315	300	280	264	234	209	148	92	61	35

Table W468-13 Age Group = 18-40 Gender = OVERALL Occupational group = MANUAL Total cases = 399

Threshold dB HL≥	0	5	10	15	20	25	30	35	40	45	50	55	65	75	85	95
**Prevalence**	**97.9**	**91.3**	**73.4**	**52.0**	**34.7**	**26.3**	**19.1**	**11.0**	**8.1**	**6.1**	**3.9**	**2.7**	**1.4**	**1.0**	**0.8**	**0.5**
Lower CI	96.0	86.8	67.1	45.8	29.3	21.3	14.8	8.1	5.6	4.1	2.6	1.6	0.7	0.4	0.3	0.1
Upper CI	98.9	94.4	78.9	58.1	40.7	32.0	24.3	14.9	11.6	8.8	5.9	4.3	2.7	2.4	2.3	1.7
Number	388	371	311	245	180	143	101	77	60	48	37	26	14	9	7	5

Table W468-14 Age Group = 41-60 Gender = OVERALL Occupational group = MANUAL Total cases = 613

Threshold dB HL≥	0	5	10	15	20	25	30	35	40	45	50	55	65	75	85	95
**Prevalence**	**100.0**	**99.0**	**96.4**	**89.3**	**78.2**	**65.1**	**54.8**	**44.5**	**36.6**	**30.1**	**22.6**	**18.7**	**12.6**	**7.6**	**4.5**	**2.9**
Lower CI	99.4	97.5	94.2	86.1	74.1	60.5	50.3	40.2	32.6	26.4	19.5	15.9	10.4	5.9	3.2	1.9
Upper CI	100.0	99.6	97.8	91.8	81.8	69.4	59.3	49.0	40.8	34.1	26.0	21.8	15.3	9.7	6.2	4.4
Number	613	609	594	563	511	448	394	341	297	251	210	176	135	81	50	32

Prevalence by age group, gender and occupational group for the worse ear over 4, 6 and 8 kHz

Table W468-15 Age Group = 61-80 Gender = OVERALL Occupational group = MANUAL Total cases = 433

	Threshold dB HL≥															
	0	5	10	15	20	25	30	35	40	45	50	55	65	75	85	95
**Prevalence**	**100.0**	**100.0**	**100.0**	**99.9**	**95.0**	**92.7**	**88.3**	**83.5**	**79.5**	**73.5**	**67.6**	**57.5**	**42.8**	**27.6**	**16.2**	**7.7**
Lower CI	99.1	99.1	99.1	99.4	91.1	88.5	84.1	78.7	74.6	67.9	62.1	51.7	37.4	23.0	12.5	5.9
Upper CI	100.0	100.0	100.0	100.0	97.3	95.4	91.5	87.4	83.6	78.4	72.8	63.0	48.4	32.7	20.9	10.0
Number	433	433	433	432	421	415	404	388	372	356	336	312	262	184	115	64

Table W468-16 Age Group = 18-40 Gender = FEMALE Occupational group = NON-MANUAL Total cases = 199

	Threshold dB HL≥															
	0	5	10	15	20	25	30	35	40	45	50	55	65	75	85	95
**Prevalence**	**97.3**	**84.4**	**60.6**	**31.7**	**19.0**	**14.4**	**8.0**	**5.1**	**4.7**	**3.4**	**1.1**	**0.5**	**0.5**	**0.0**	**0.0**	**0.0**
Lower CI	93.8	78.5	53.5	25.5	14.0	10.1	5.0	2.8	2.6	1.6	0.3	0.1	0.1	0.0	0.0	0.0
Upper CI	98.9	89.0	67.2	38.6	25.2	20.2	12.6	8.8	8.6	7.1	3.6	2.5	2.5	1.9	1.9	1.9
Number	194	170	127	72	45	34	21	14	13	9	3	2	2	0	0	0

Table W468-17 Age Group = 41-60 Gender = FEMALE Occupational group = NON-MANUAL Total cases = 282

	Threshold dB HL≥															
	0	5	10	15	20	25	30	35	40	45	50	55	65	75	85	95
**Prevalence**	**100.0**	**99.4**	**89.7**	**76.3**	**61.0**	**48.2**	**35.2**	**24.8**	**17.8**	**14.8**	**11.8**	**8.0**	**3.6**	**2.0**	**1.5**	**0.9**
Lower CI	98.7	97.4	85.0	70.5	54.7	42.4	30.0	20.4	14.3	11.6	9.1	5.9	2.5	1.3	0.8	0.4
Upper CI	100.0	99.9	93.1	81.3	67.0	54.1	40.7	29.8	22.0	18.8	15.2	10.7	5.1	3.0	2.5	1.9
Number	282	280	258	223	184	153	118	91	71	61	52	39	22	14	10	6

Table W468-18 Age Group = 61-80 Gender = FEMALE Occupational group = NON-MANUAL Total cases = 181

	Threshold dB HL≥															
	0	5	10	15	20	25	30	35	40	45	50	55	65	75	85	95
**Prevalence**	**100.0**	**100.0**	**99.2**	**98.1**	**92.8**	**87.6**	**84.4**	**80.8**	**69.2**	**65.4**	**56.0**	**51.4**	**31.9**	**13.3**	**6.6**	**3.2**
Lower CI	97.9	97.9	95.5	93.9	86.9	80.2	76.9	73.2	59.9	56.4	47.0	42.8	24.5	9.2	4.7	1.9
Upper CI	100.0	100.0	99.9	99.4	96.2	92.5	89.8	86.7	77.2	73.4	64.5	59.9	40.3	18.8	9.3	5.3
Number	181	181	180	178	171	164	157	150	133	126	110	102	66	43	27	16

Table W468-19 Age Group = 18-40 Gender = MALE Occupational group = NON-MANUAL Total cases = 128

	Threshold dB HL≥															
	0	5	10	15	20	25	30	35	40	45	50	55	65	75	85	95
**Prevalence**	**97.4**	**87.0**	**64.5**	**38.9**	**26.0**	**15.3**	**14.6**	**13.6**	**12.3**	**10.4**	**9.4**	**9.1**	**3.9**	**1.4**	**1.1**	**0.0**
Lower CI	90.0	79.0	54.8	29.9	17.9	9.0	8.4	7.6	6.4	5.1	4.3	4.1	1.3	0.5	0.3	0.0
Upper CI	99.3	92.2	73.1	48.7	36.1	24.9	24.4	23.4	22.2	20.1	19.1	18.9	10.8	4.4	4.5	2.9
Number	126	113	89	60	40	26	23	21	15	13	10	9	6	4	2	0

Table W468-20 Age Group = 41-60 Gender = MALE Occupational group = NON-MANUAL Total cases = 210

	Threshold dB HL≥															
	0	5	10	15	20	25	30	35	40	45	50	55	65	75	85	95
**Prevalence**	**100.0**	**100.0**	**97.3**	**90.1**	**76.4**	**65.1**	**50.7**	**43.0**	**37.4**	**33.2**	**28.1**	**24.1**	**12.9**	**8.1**	**2.1**	**1.8**
Lower CI	98.2	98.2	93.6	84.1	69.4	58.0	43.2	35.9	30.5	26.7	22.2	18.8	8.9	4.7	1.3	1.1
Upper CI	100.0	100.0	98.8	94.0	82.2	71.6	58.2	50.3	44.9	40.4	35.0	30.3	18.2	13.7	3.4	3.0
Number	210	210	205	192	166	147	120	102	89	79	67	60	40	26	10	8

Table W468-21 Age Group = 61-80 Gender = MALE Occupational group = NON-MANUAL Total cases = 166

	Threshold dB HL≥															
	0	5	10	15	20	25	30	35	40	45	50	55	65	75	85	95
**Prevalence**	**100.0**	**100.0**	**100.0**	**100.0**	**99.9**	**97.4**	**92.1**	**86.2**	**84.1**	**76.6**	**68.6**	**57.7**	**42.8**	**26.2**	**18.5**	**8.9**
Lower CI	97.7	97.7	97.7	97.7	99.3	92.7	81.0	75.5	73.5	66.0	58.4	47.6	36.1	20.9	14.2	5.6
Upper CI	100.0	100.0	100.0	100.0	100.0	99.1	97.0	92.7	91.0	84.7	77.3	67.3	49.9	32.2	23.7	14.0
Number	166	166	166	166	165	162	158	150	147	138	124	107	82	49	34	19

Prevalence by age group, gender and occupational group for the worse ear over 4, 6 and 8 kHz

Table W468-22 Age Group = 18-40 Gender = FEMALE Occupational group = MANUAL Total cases = 211

Threshold dB HL≥	0	5	10	15	20	25	30	35	40	45	50	55	65	75	85	95
Prevalence	**98.1**	**90.5**	**71.3**	**49.5**	**30.1**	**22.7**	**14.5**	**11.0**	**8.2**	**5.0**	**3.7**	**2.6**	**0.6**	**0.1**	**0.1**	**0.0**
Lower CI	95.2	83.3	62.2	41.2	23.2	16.6	9.8	6.9	4.8	3.0	2.1	1.3	0.3	0.0	0.0	0.0
Upper CI	99.2	94.8	79.0	57.8	37.9	30.3	20.8	17.2	13.8	8.4	6.5	4.9	1.4	0.7	0.7	1.8
Number	205	196	162	128	91	71	49	38	30	22	19	14	5	1	1	0

Table W468-23 Age Group = 41-60 Gender = FEMALE Occupational group = MANUAL Total cases = 297

Threshold dB HL≥	0	5	10	15	20	25	30	35	40	45	50	55	65	75	85	95
Prevalence	**100.0**	**98.1**	**93.3**	**82.7**	**68.4**	**57.6**	**43.0**	**32.9**	**25.1**	**19.2**	**9.5**	**6.3**	**4.6**	**2.6**	**1.7**	**1.3**
Lower CI	98.7	95.3	89.2	77.5	62.1	51.1	36.8	27.2	20.1	14.8	6.9	4.3	3.2	1.6	1.1	0.7
Upper CI	100.0	99.3	95.9	87.0	74.2	63.8	49.4	39.1	30.8	24.5	12.9	9.0	6.6	4.1	2.8	2.3
Number	297	293	279	255	219	188	151	122	98	79	55	40	34	22	16	10

Table W468-24 Age Group = 61-80 Gender = FEMALE Occupational group = MANUAL Total cases = 195

Threshold dB HL≥	0	5	10	15	20	25	30	35	40	45	50	55	65	75	85	95
Prevalence	**100.0**	**100.0**	**100.0**	**99.8**	**91.7**	**87.6**	**80.7**	**74.7**	**68.4**	**61.3**	**55.8**	**44.4**	**30.6**	**18.8**	**11.5**	**4.9**
Lower CI	98.1	98.1	98.1	98.9	84.9	80.5	73.6	66.8	60.5	52.5	47.6	36.0	23.6	13.6	7.5	3.4
Upper CI	100.0	100.0	100.0	100.0	95.6	92.4	86.2	81.3	75.4	69.3	63.7	53.1	38.6	25.5	17.4	7.1
Number	195	195	195	194	184	178	169	158	146	138	127	113	95	61	37	19

Table W468-25 Age Group = 18-40 Gender = MALE Occupational group = MANUAL Total cases = 188

Threshold dB HL≥	0	5	10	15	20	25	30	35	40	45	50	55	65	75	85	95
Prevalence	**97.7**	**92.2**	**75.6**	**54.3**	**39.7**	**30.1**	**24.2**	**10.9**	**7.9**	**7.1**	**4.1**	**2.7**	**2.1**	**1.9**	**1.6**	**1.0**
Lower CI	94.5	85.8	66.5	45.2	31.3	22.4	17.2	7.4	4.8	4.2	2.3	1.3	0.9	0.8	0.5	0.3
Upper CI	99.1	95.8	82.8	63.2	48.7	39.0	32.9	15.9	12.7	12.0	7.2	5.3	5.0	4.8	4.5	3.5
Number	183	175	149	117	89	72	52	39	30	26	18	12	9	8	6	5

Table W468-26 Age Group = 41-60 Gender = MALE Occupational group = MANUAL Total cases = 316

Threshold dB HL≥	0	5	10	15	20	25	30	35	40	45	50	55	65	75	85	95
Prevalence	**100.0**	**100.0**	**99.9**	**96.4**	**88.9**	**73.4**	**67.8**	**57.3**	**49.4**	**42.2**	**37.0**	**32.2**	**21.4**	**13.1**	**7.5**	**4.7**
Lower CI	98.8	98.8	99.3	92.3	83.4	66.8	61.1	50.7	43.2	36.2	31.3	26.9	17.0	9.7	5.1	2.8
Upper CI	100.0	100.0	100.0	98.4	92.7	79.1	73.9	63.7	55.5	48.4	43.0	38.1	26.6	17.3	11.1	7.7
Number	316	316	315	308	292	260	243	219	199	172	155	136	101	59	34	22

Table W468-27 Age Group = 61-80 Gender = MALE Occupational group = MANUAL Total cases = 238

Threshold dB HL≥	0	5	10	15	20	25	30	35	40	45	50	55	65	75	85	95
Prevalence	**100.0**	**100.0**	**100.0**	**100.0**	**99.2**	**99.2**	**98.2**	**94.7**	**93.6**	**89.3**	**82.9**	**74.5**	**58.9**	**39.0**	**22.4**	**11.5**
Lower CI	98.4	98.4	98.4	98.4	95.6	95.6	94.5	90.4	89.3	83.7	75.8	67.6	50.8	31.6	16.1	8.0
Upper CI	100.0	100.0	100.0	100.0	99.9	99.9	99.4	97.1	96.3	93.1	88.3	80.4	66.5	47.0	30.3	16.3
Number	238	238	238	238	237	237	235	230	226	218	209	199	167	123	78	45

Chapter 4

Distribution of hearing impairment

4.0 Scope and introduction

The prevalence of hearing impairment is predominantly concerned with the distribution of people whose hearing impairment is worse than a particular criterion level. This chapter presents the distributional data for the hearing threshold level measures. The percentiles, mean and SD of the distribution were calculated for each ear (better, worse, left, right and average of left and right). As well as the statistics from the whole sample which represents the typical population, the data are presented for the subset of the sample who have been screened for substantial noise immission and conductive hearing impairment.

4.1 Definitions of distribution of hearing impairment

The data presented in the tables for this chapter are in three parts per table. The first part gives the mean and SD for each frequency threshold. These have been calculated directly using the structure of the study rather than the individual case weights (see Chapter 2). Therefore there is little bias from missing data or large individual case-weights. The CI for the mean is calculated as if the mean were normally distributed. Given the large N for most of the data cells, this is a fair assumption. The bias, taking the form of a high skew towards more impaired values of hearing threshold, does not exert much influence in the region near the mean. However, the SD should not be used to derive percentiles of the population distribution because in most combinations of age, gender and SEG this skew would lead to highly conservative percentiles at the median or above. Thus some groups may be thought to be discrepant when in fact they show a distribution not unlike the typical population.

The second section presents the chosen percentiles (5 to 25 in steps of 5, 50, and 75 to 95 in steps of 5). These statistics could not be derived from the strata *per se*. Therefore, the individual case-weights had to be used. As the data were weighted, it is not possible to interpolate between values to obtain a better estimate of a particular percentile (Cochran, 1976). For individual threshold measures, it should be remembered that hearing thresholds were obtained in 5 dB steps. The calibration correction was then added to the hearing threshold to the nearest 1 dB. Thus it is possible to have thresholds that are not steps of 5 dB. This is important when interpreting the percentile statistics. The percentile statistic is thus the actual range value at which that percentile fell. As the data were not constrained to 5 dB intervals, it can be shown that the percentile estimates are less biased than they would be if there was such a constraint and no interpolation of percentiles can be used due to the design of the study. In this book percentiles are derived in the normal manner (Kendall and Stuart, 1977) e.g. the 5th percentile for a particular threshold indicates that 5% of the population are estimated to have a hearing threshold less than or equal to that percentile. Thus increasing percentiles are associated with hearing thresholds that

are higher (i.e. worse). This is contrary to the definition in some international standards of hearing (e.g. ISO 1999) where percentiles are used unconventionally (Dobie, 1992).

The third section of the tables concerns the statistics of the sample. The number of people in the sample who contributed to each column is shown together with two other design-specific statistics. The first of these is the design effect (Deft), i.e. the square root of the ratio of the variance obtained in this sample compared with that if the sample had no stratification structure (i.e. a simple random sample). The generally high efficiency of the design shows that the effect of stratification, which partitions variance within and between particular strata, has been successful. For printing reasons, the Deft is shown as a percentage in this chapter, rather than a proportion, which was the case in Chapter 3. The third statistic shown is called the Bias, but represents only one aspect of the whole statistical bias. This particular aspect of bias is the extent to which the mean calculated from the whole stratification procedure differs from that found if the individual case-weights are applied. This is given in 1/100 of a dB. Thus a Bias of 3, shown in the last column of Table 1, represents a difference of 0.03 of a dB between the estimates. These differences arise because of missing data in particular strata, or through a need to re-assign cases to composite strata, if the number of people in an individual stratum fall below a critical number (<3). Whilst the Bias that arises in these tables is usually small, when particular subsets are chosen then the Bias might increase. Thus there is a larger Bias in the sample that is screened than in the typical (or unscreened) population.

The organisation of the tables in this chapter is cyclical. The five combinations of ear - better (B), worse (W), left (L), right (R), and left and right averaged (LR) - delineate the five sections (i-v) of tables in this chapter. Within each section there are the seven age groupings (18-80, 18-30, 31-40, 41-50, 51-60, 61-70 and 71-80), the three occupational groups (overall, non-manual and manual) and the three genders (overall, female and male). The tables have been cycled in that order with gender changing first, then occupational group, and then age. For each of these demographic groups two tables are presented. The first presents the overall data for the typical population, with no exclusions other than missing data on that threshold. The second presents the data when certain exclusions have been made from the sample. The exclusions that have gone towards making this 'screened' data set were noise and a measure of middle ear status. For noise immission, any subject who had significant noise immission from occupational, gunfire or social noise exposure was excluded. The criterion used was the equivalent of 80 dB(A) Leq 40 for a 50-year duration (see Davis, 1989; Lutman and Davis, 1994 for an explanation of how the noise immission measure was derived). The indicator of middle ear status was the difference between the air- and bone-conduction threshold levels - the air bone gap. Any subject who had a positive air-bone gap was removed from the dataset (see Lutman and Davis, 1994). Overall, applying these stringent criteria removes a considerable number of people from the sample and reduces the overall number from about 2659 to 1204, c.f. Tables 1 and 2 at 0.25 kHz. In screening out the people with significant noise immission and any trace of air-bone gap, the final screened sample can make one of the best attempts at providing the distribution of hearing impairment in an 'otologically normal population' (Lutman and Davis, 1994).

Within each table there are three sets of statistics that are highlighted by shading; these are the mean, median and the Number contributing to the statistics. This enables the reader to focus initially on the central tendency measures that play a large role in determining scientific or clinical action. Whilst the approximately ten-year age band used here is quite wide, it is realistic - given the numbers in a particular age, gender and occupational group, c.f. Table 26 for example - and aligns with the post-stratification, and hence reduces undue bias from non-standard age groups. Chapter 6 examines the possibility of modelling the data at one-year age bands. The variability of hearing threshold increases non-linearly with age group and with the mean more generally. It is not possible to apply a single generally appropriate transformation when trying to analyse the hearing threshold data. Thus even using a log-normal model for the hearing thresholds some account has to be made of the increasing variance with age and other factors by adding parameters. This approach has been explored by fitting a simultaneous set of general linear models to the transformed mean and variance of the hearing thresholds (Bowater, R.J., Copas, J.B., Machado, O.A., & Davis, A.C., 1994, unpublished data). This statistical model may turn out to be more appropriate than others tried hitherto in exploring the impact of demographic and other variables on hearing thresholds. The major drawback to this method is that it is highly labour intensive.

It may be worth noting that for individual frequency thresholds, the percentiles are all integer due to the way in which they were derived. However, they are not multiples of 5 dB, due to the calibration policy that was mentioned in Chapter 2. The average hearing thresholds provide percentiles whose rounded presentation reflects the number of frequencies entering each average.

There are two sets of figures that accompany the tables. In Figures section i, there are 60 figures that give the mean and percentiles (shown in the tables) of the hearing threshold distribution plotted over each frequency in an audiogram for each ear (left, right, better, worse and both ears). For each ear, every age group and gender combination is plotted separately. The five ears, six age groups, and two genders produce 60 figures, with gender changing more rapidly than age groups. The main data in the figures are connected by lines over frequency. The mean is shown as a filled-in symbol (♦), with the percentiles shown as open symbols (Δ for 5th, 95th percentiles; + for 10th, 90th percentiles; X for 15th, 85th percentiles; ◊ for 20th, 80th percentiles; ∇ for 25th, 75th percentiles; ❍ for 50th). A right-pointing arrow, which is not connected by a line gives some estimate of variability for these data. The arrows represent 0.67 standard deviations from the mean. If the hearing of thresholds came from the normal distribution the arrows would lie on top of the 75th percentile data for that age and gender group. The data are only shown here for the typical population.

In Figures section ii, the data are replotted so that each of the twelve stratifications of age and gender are shown on each audiogram. The 30 figures show the mean plus 5, 25, 50, 75, 95th percentiles as a function of ear. Thus for each statistic the five figures are shown corresponding to better, worse, left, right, and both ears. Within each figure, the data for the females is shown as a solid line and for males as a dotted line. The span between the 5th and 95th percentile is about 20-25 dB at frequencies up to 2 kHz for aged 18-30, and this is about the same shift as there is in the mean between the ages 18-30 and

71-80 over the same frequencies. However, the difference between the 5th and 95th percentile increases steeply with age until it is about 50-60 dB at age 61-70 and even larger at age group 71-80.

4.2 Summary and conclusions

This section has systematically presented the key statistics concerning the distribution of hearing thresholds in the population as a function of age, gender and occupational group. In addition, the distribution of hearing thresholds in the sub-population who have no significant noise exposure and no air-bone gap has been derived. The latter distribution is less robust in some demographic sub-populations due to the large number of exclusions, e.g. in the male manual occupational group aged 61-70 where only 22.74% of the 'typical' population are in the 'screened' sample of no significant noise exposure or air-bone gap. However, even the data here provide a very reasonable baseline for comparison. There is no evidence of lack of generality. The shift over hearing thresholds with age is clearly represented in the figures: section ii, and is present at all percentiles of the distribution, albeit getting smaller with smaller percentiles. The different pattern between the audiograms of men and women evolves with age and is shown best at percentiles above the median.

The data shown in the tables and figures of this chapter are ideal for comparison of individual subjects and of groups once the principle behind the organisation of the tables and selection of sub-populations is understood and the purpose of such comparison is made clear.

As stated in Chapter 2, the primary aim of the NSH was to obtain a systematic overview of the severity/prevalence distribution of hearing impairment in Great Britain. A second aim was to obtain baseline reference data concerning the distribution of hearing thresholds in the population for meaningful demographic sub-groups. This chapter has gone a long way towards achieving that aim using a random sample of the population.

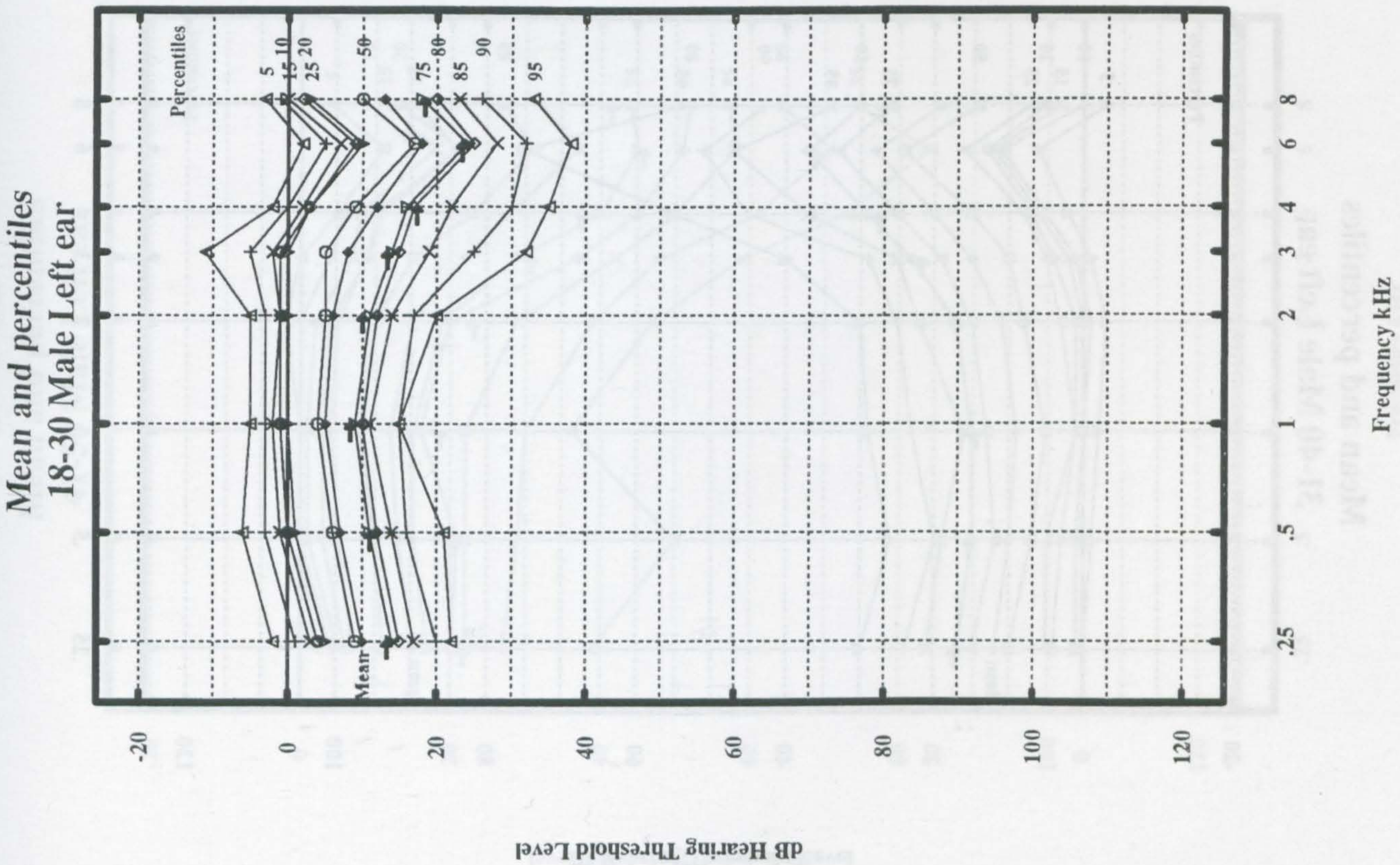
Mean and percentiles
18-30 Male Left ear
Percentiles
5
10
15
20
25
50
75
80
85
90
95
Mean
dB Hearing Threshold Level
-20
0
20
40
60
80
100
120
.25
.5
1
2
3
4
6
8
Frequency kHz

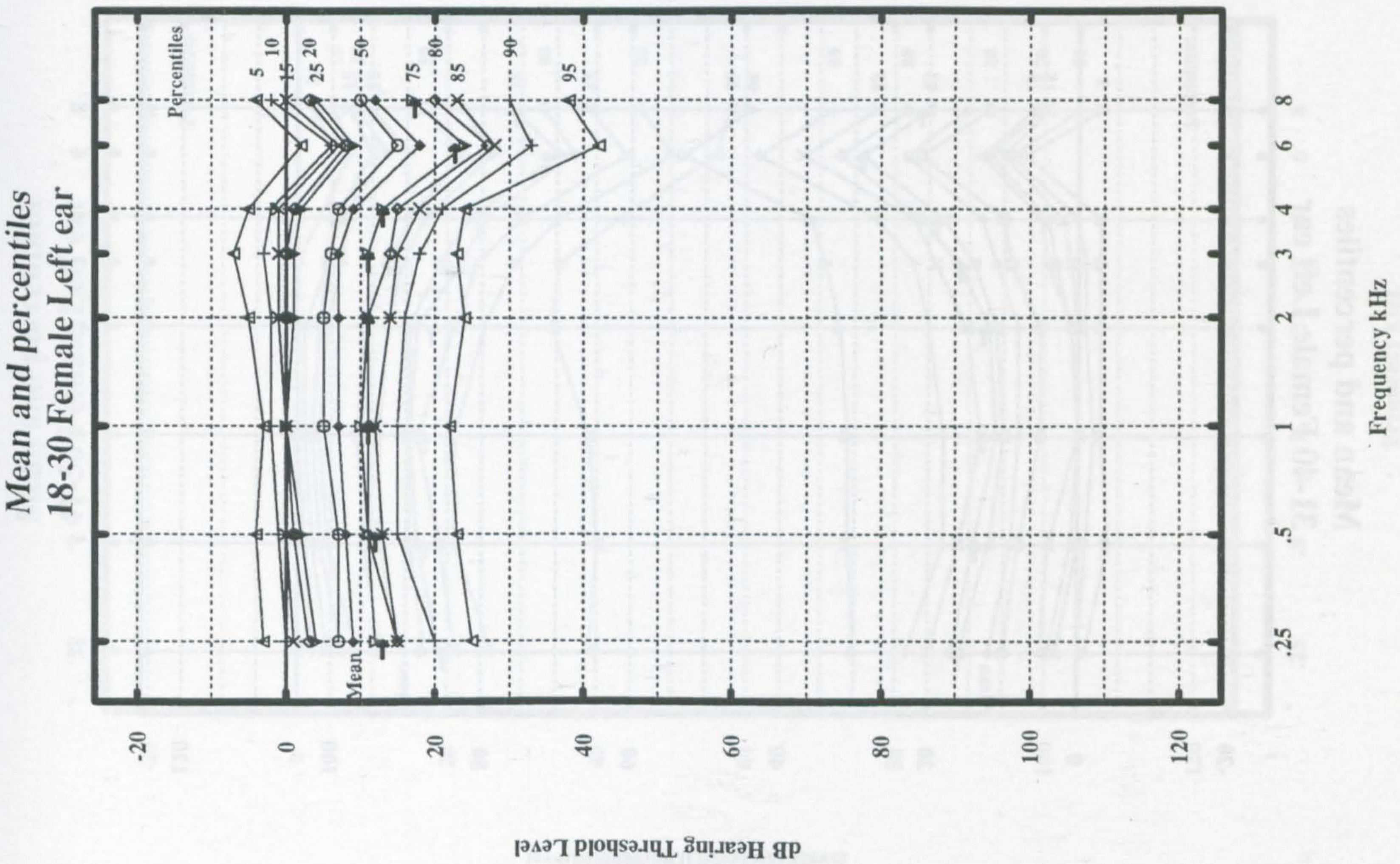
Mean and percentiles
18-30 Female Left ear
Percentiles
5
10
15
20
25
50
75
80
85
90
95
Mean
dB Hearing Threshold Level
-20
0
20
40
60
80
100
120
.25
.5
1
2
3
4
6
8
Frequency kHz

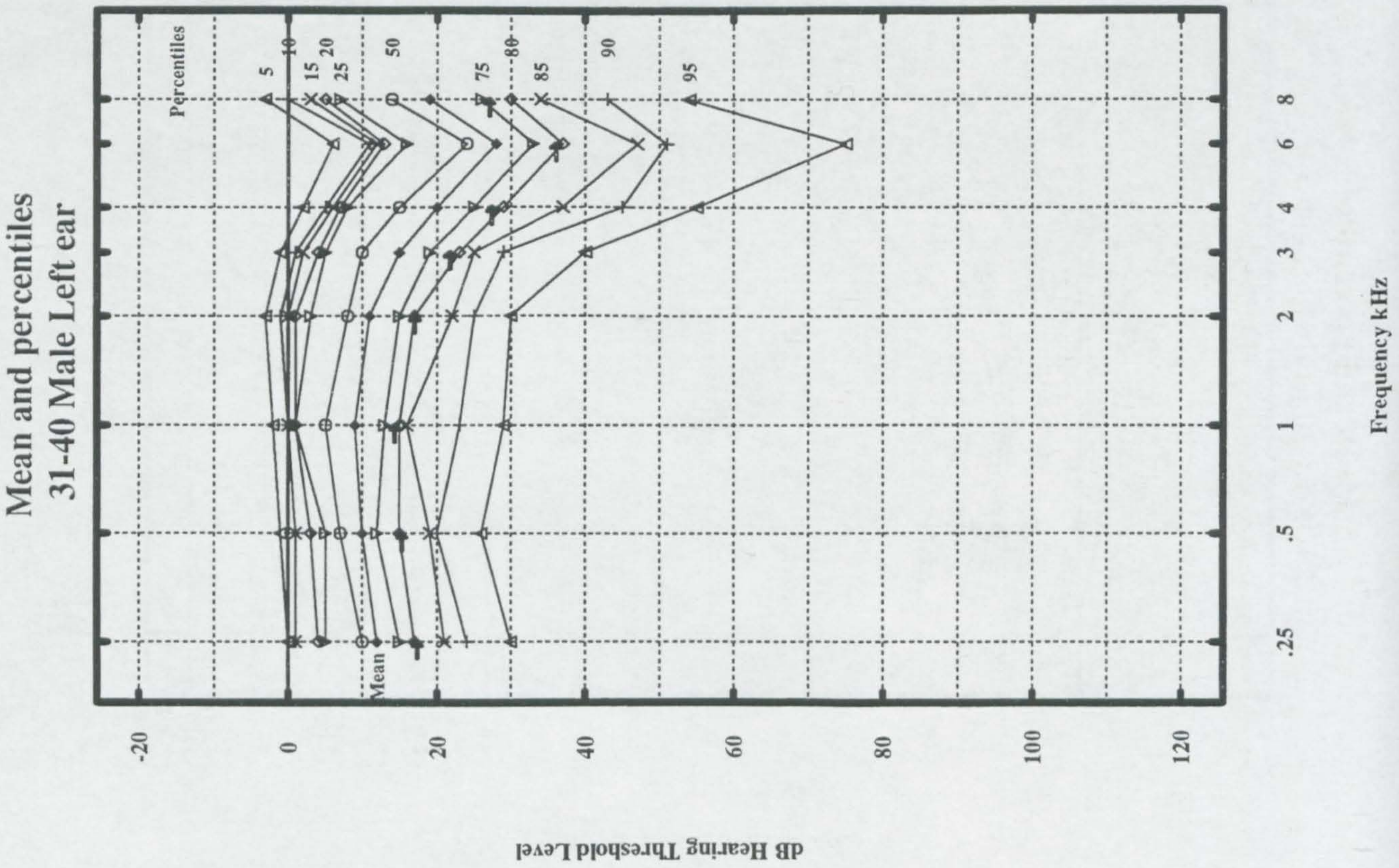
Mean and percentiles
31-40 Male Left ear
Percentiles
5
10
15
20
25
50
75
80
85
90
95
Mean
-20
0
20
40
60
80
100
120
dB Hearing Threshold Level
.25
.5
1
2
3
4
6
8
Frequency kHz

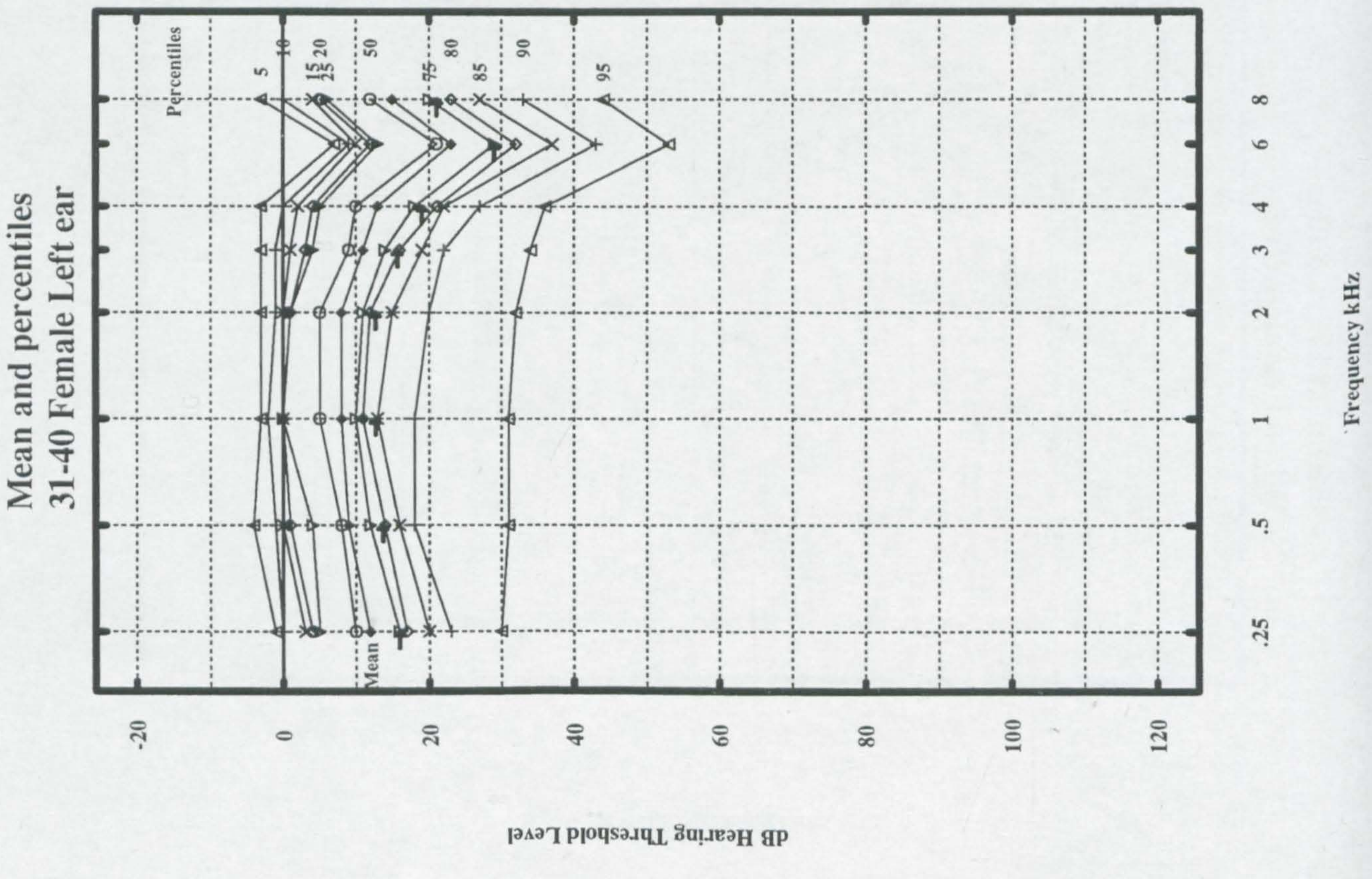
Mean and percentiles
31-40 Female Left ear
Percentiles
5
10
15
20
25
50
75
80
85
90
95
Mean
-20
0
20
40
60
80
100
120
dB Hearing Threshold Level
.25
.5
1
2
3
4
6
8
Frequency kHz

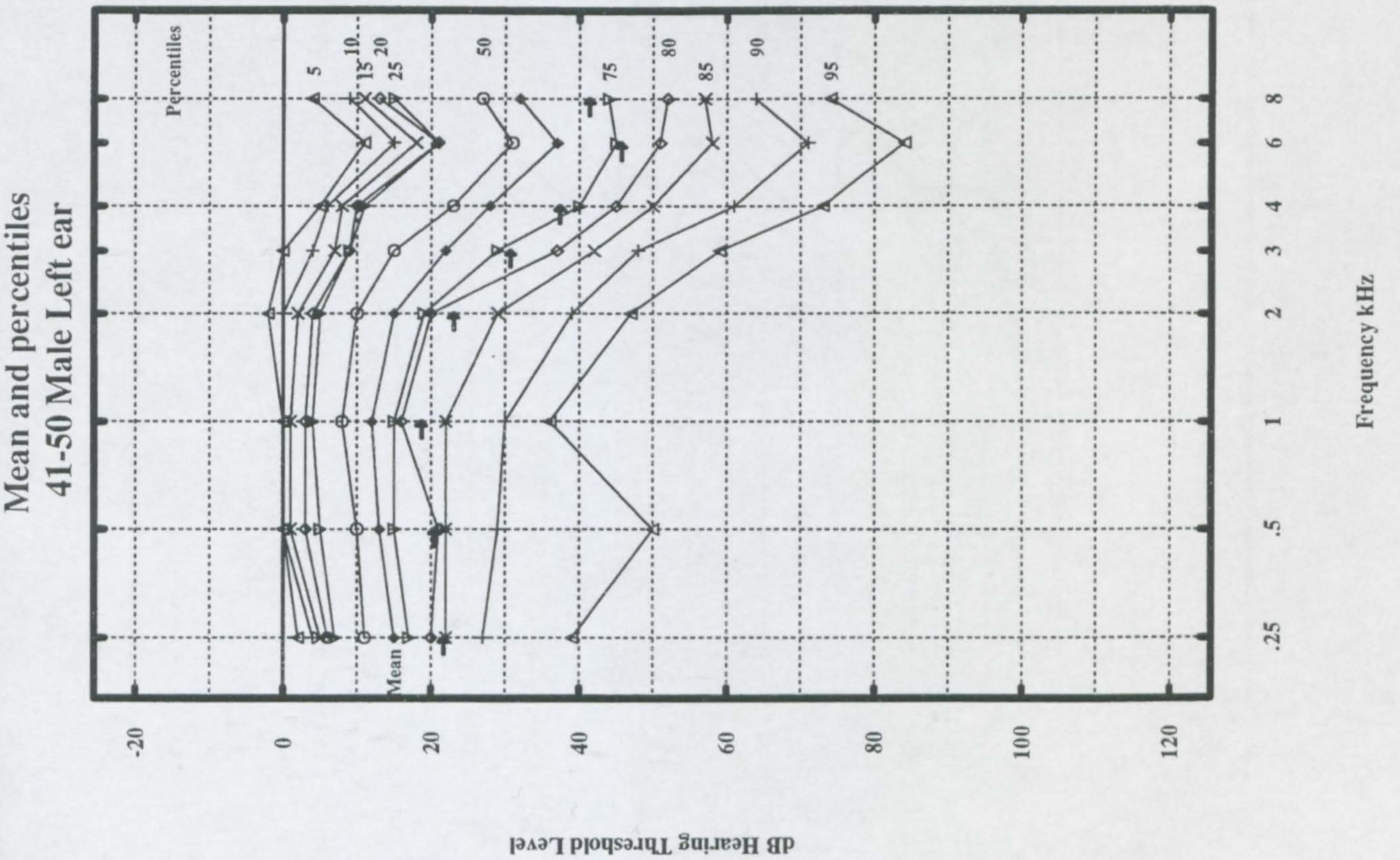
Mean and percentiles
41-50 Male Left ear
Percentiles
5
10
15
20
25
50
75
80
85
90
95
Mean
dB Hearing Threshold Level
-20
0
20
40
60
80
100
120
.25
.5
1
2
3
4
6
8
Frequency kHz

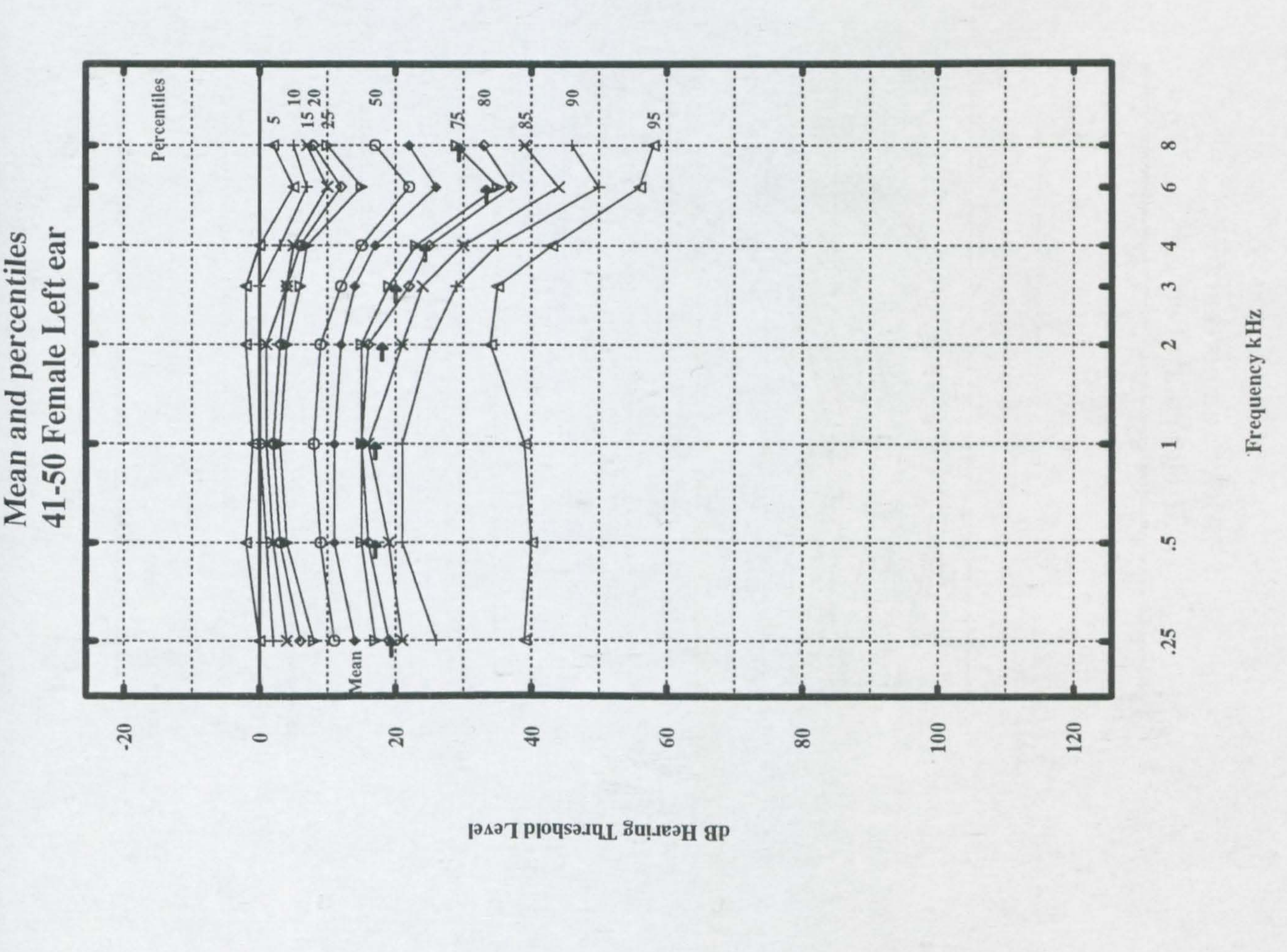
Mean and percentiles
41-50 Female Left ear
Percentiles
5
10
15
20
25
50
75
80
85
90
95
Mean
dB Hearing Threshold Level
-20
0
20
40
60
80
100
120
.25
.5
1
2
3
4
6
8
Frequency kHz

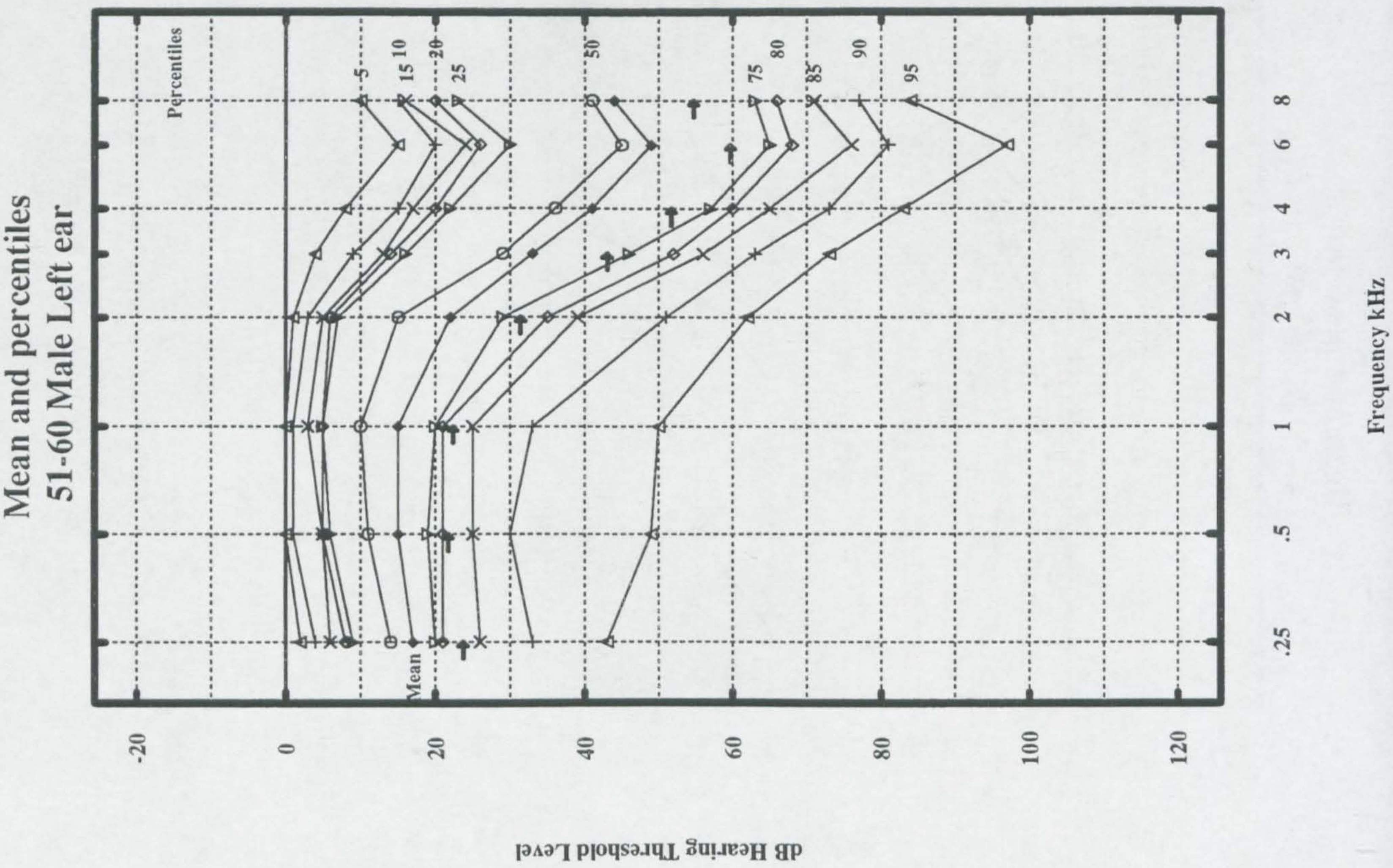
Mean and percentiles
51-60 Male Left ear
Percentiles
5
10
15
20
25
50
75
80
85
90
95
Mean
-20
0
20
40
60
80
100
120
dB Hearing Threshold Level
.25
.5
1
2
3
4
6
8
Frequency kHz

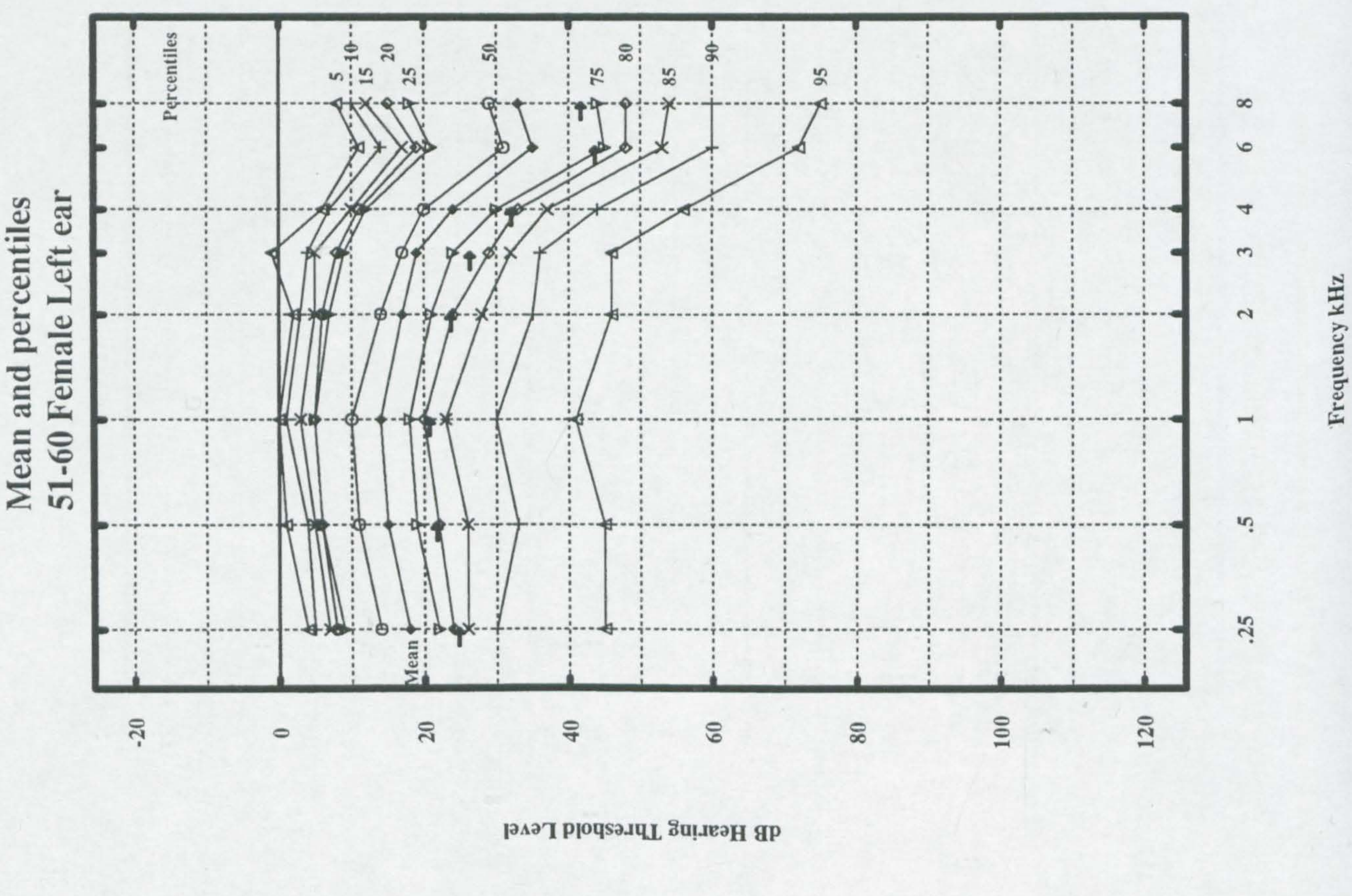
Mean and percentiles
51-60 Female Left ear
Percentiles
5
10
15
20
25
50
75
80
85
90
95
Mean
-20
0
20
40
60
80
100
120
dB Hearing Threshold Level
.25
.5
1
2
3
4
6
8
Frequency kHz

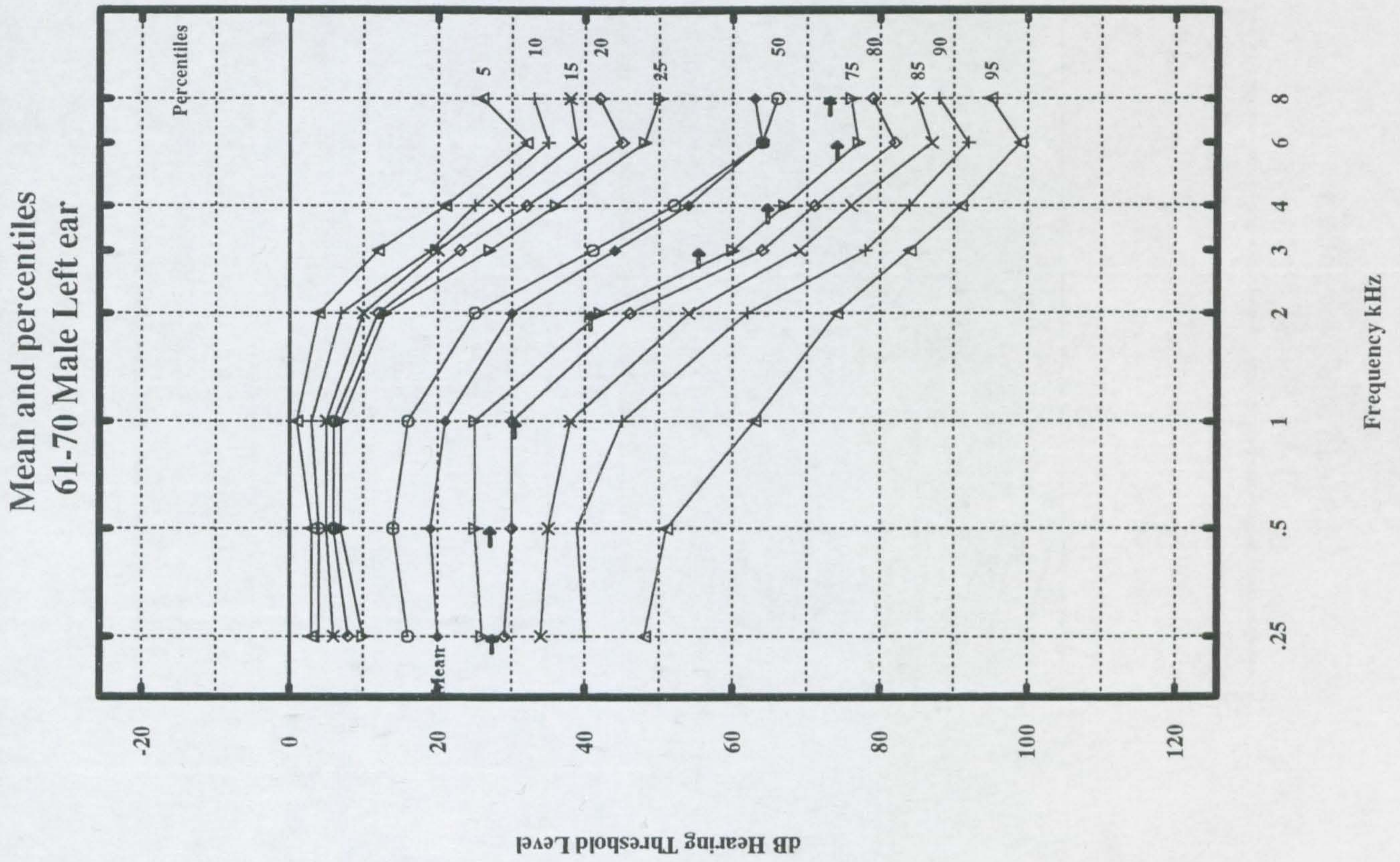
Mean and percentiles
61-70 Male Left ear
Percentiles
5
10
15
20
25
50
75
80
85
90
95
Mean
-20
0
20
40
60
80
100
120
dB Hearing Threshold Level
.25
.5
1
2
3
4
6
8
Frequency kHz

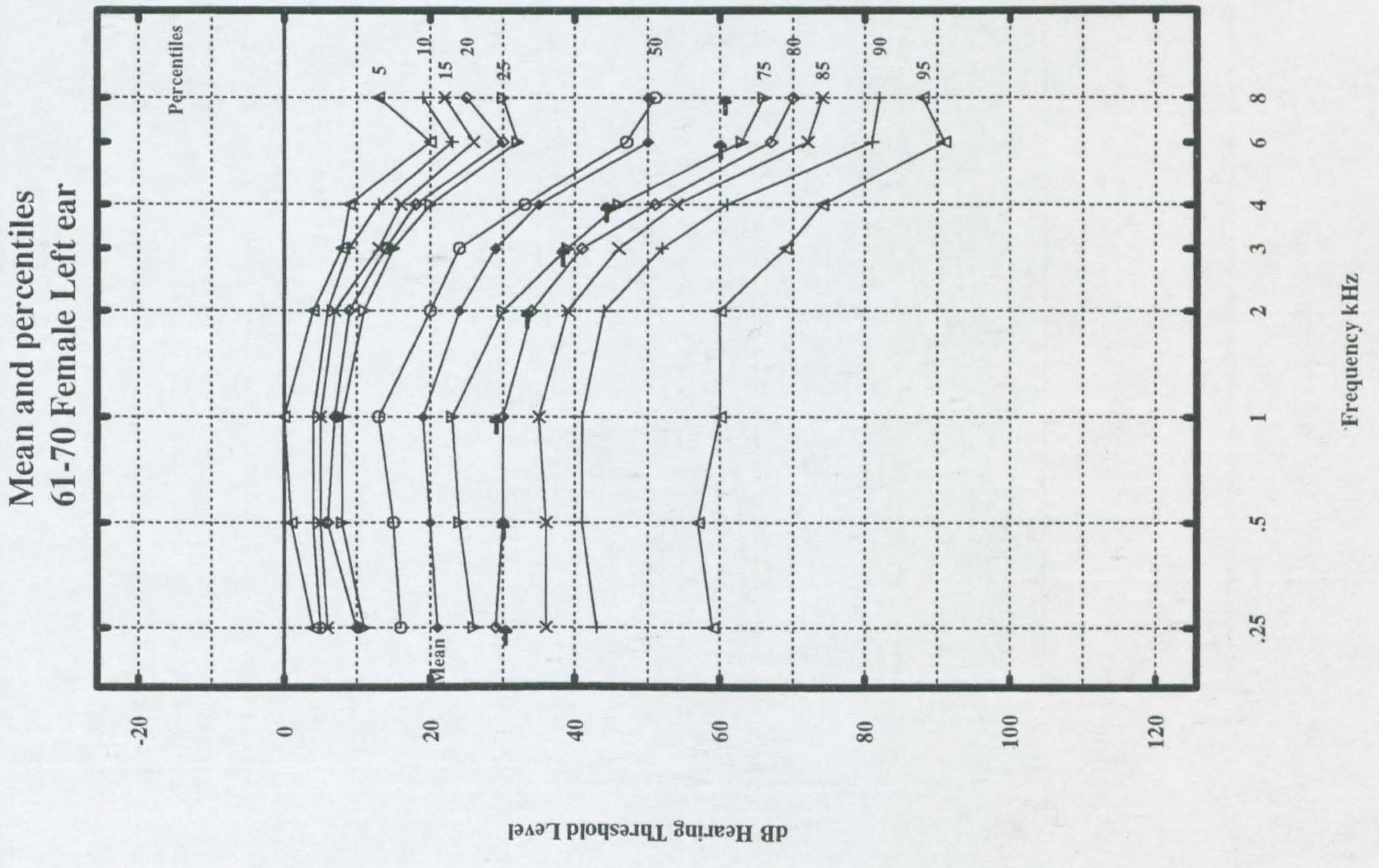
Mean and percentiles
61-70 Female Left ear
Percentiles
5
10
15
20
25
50
75
80
85
90
95
Mean
-20
0
20
40
60
80
100
120
dB Hearing Threshold Level
.25
.5
1
2
3
4
6
8
Frequency kHz

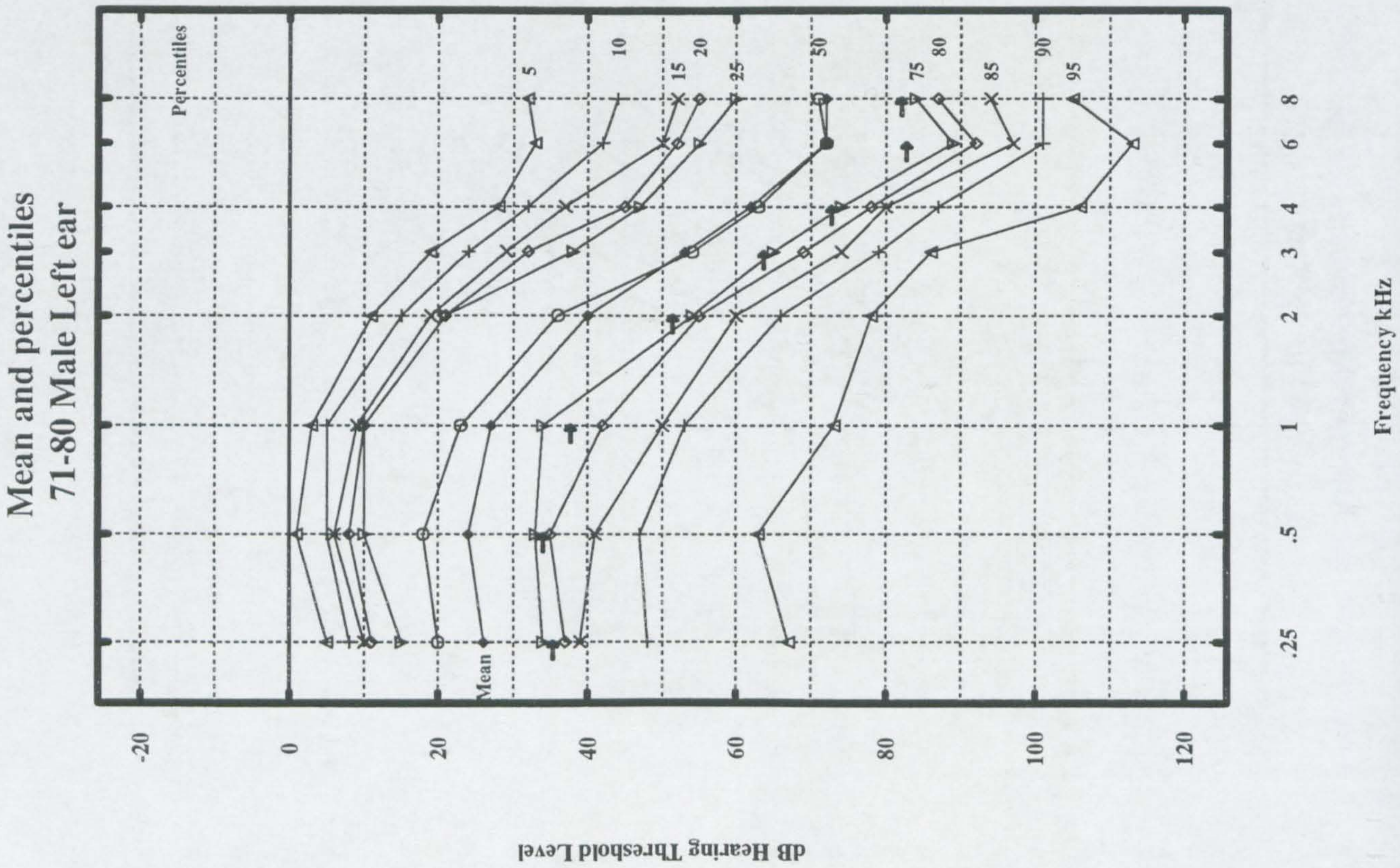
Mean and percentiles
71-80 Male Left ear
Percentiles
5
10
15
20
25
50
75
80
85
90
95
Mean
dB Hearing Threshold Level
-20
0
20
40
60
80
100
120
.25
.5
1
2
3
4
6
8
Frequency kHz

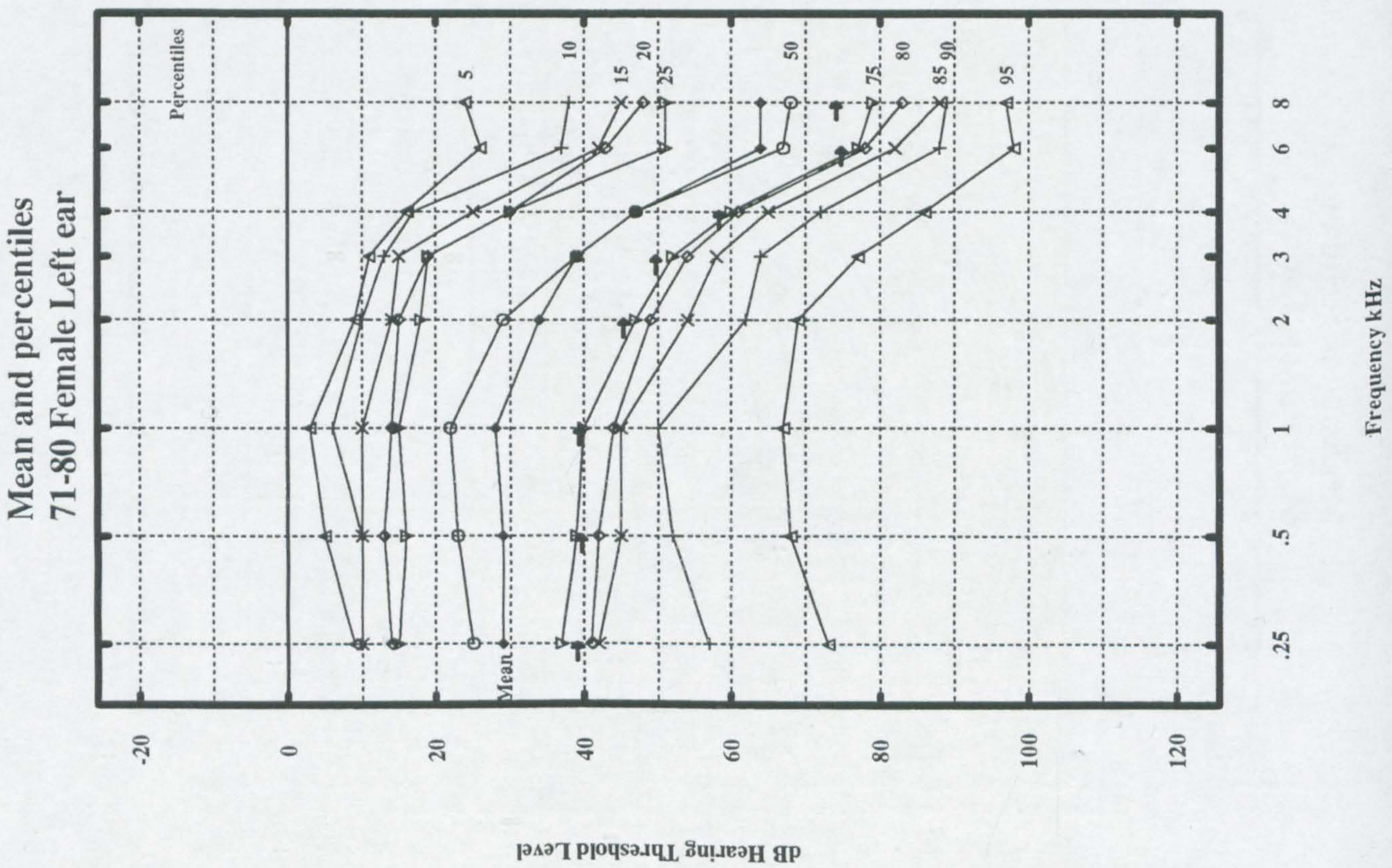
Mean and percentiles
71-80 Female Left ear
Percentiles
5
10
15
20
25
50
75
80
85
90
95
Mean
dB Hearing Threshold Level
-20
0
20
40
60
80
100
120
.25
.5
1
2
3
4
6
8
Frequency kHz

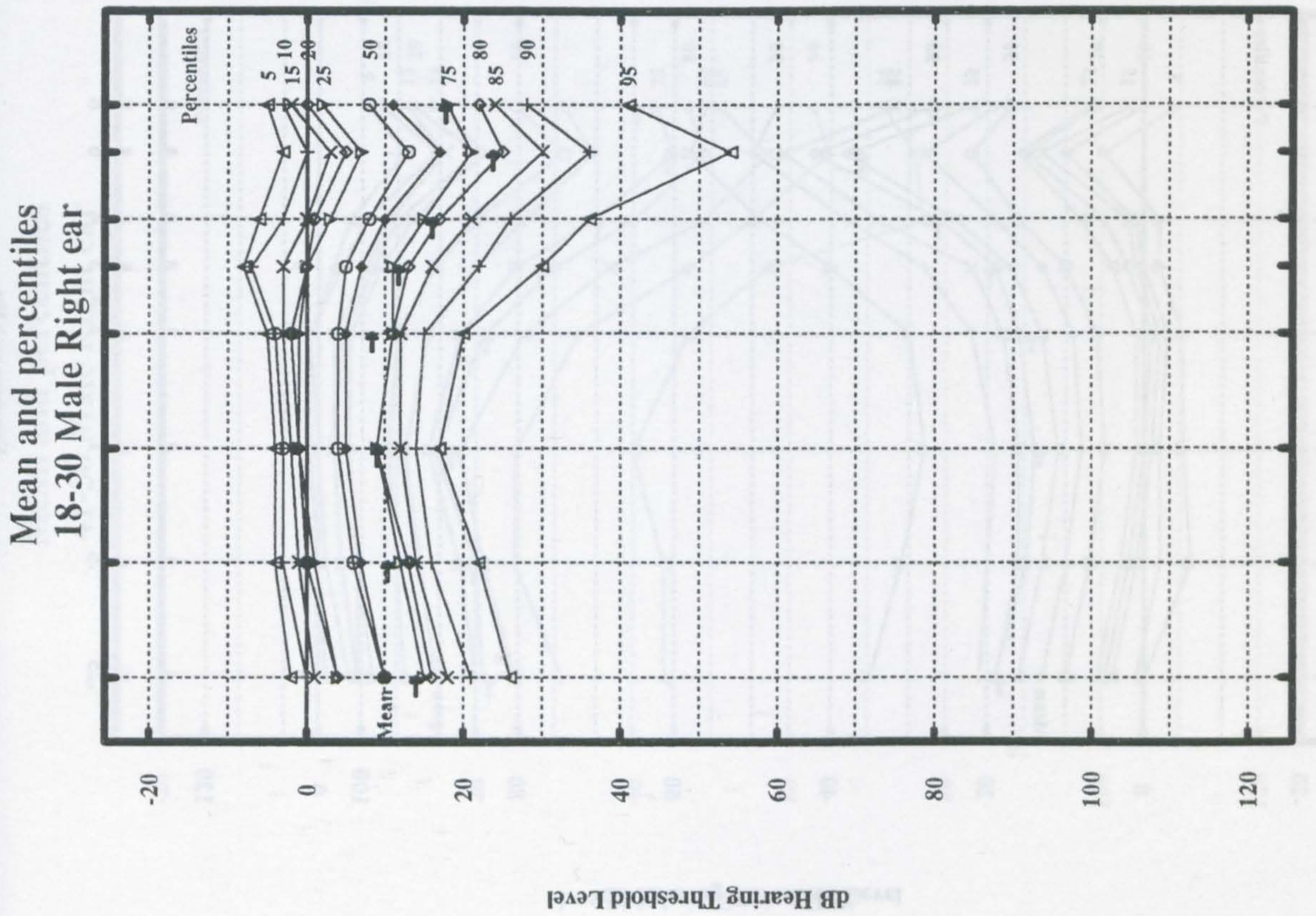
Mean and percentiles
18-30 Male Right ear
Percentiles
5
10
15
20
25
50
75
80
85
90
95
Mean
-20
0
20
40
60
80
100
120
dB Hearing Threshold Level
.25
.5
1
2
3
4
6
8
Frequency kHz

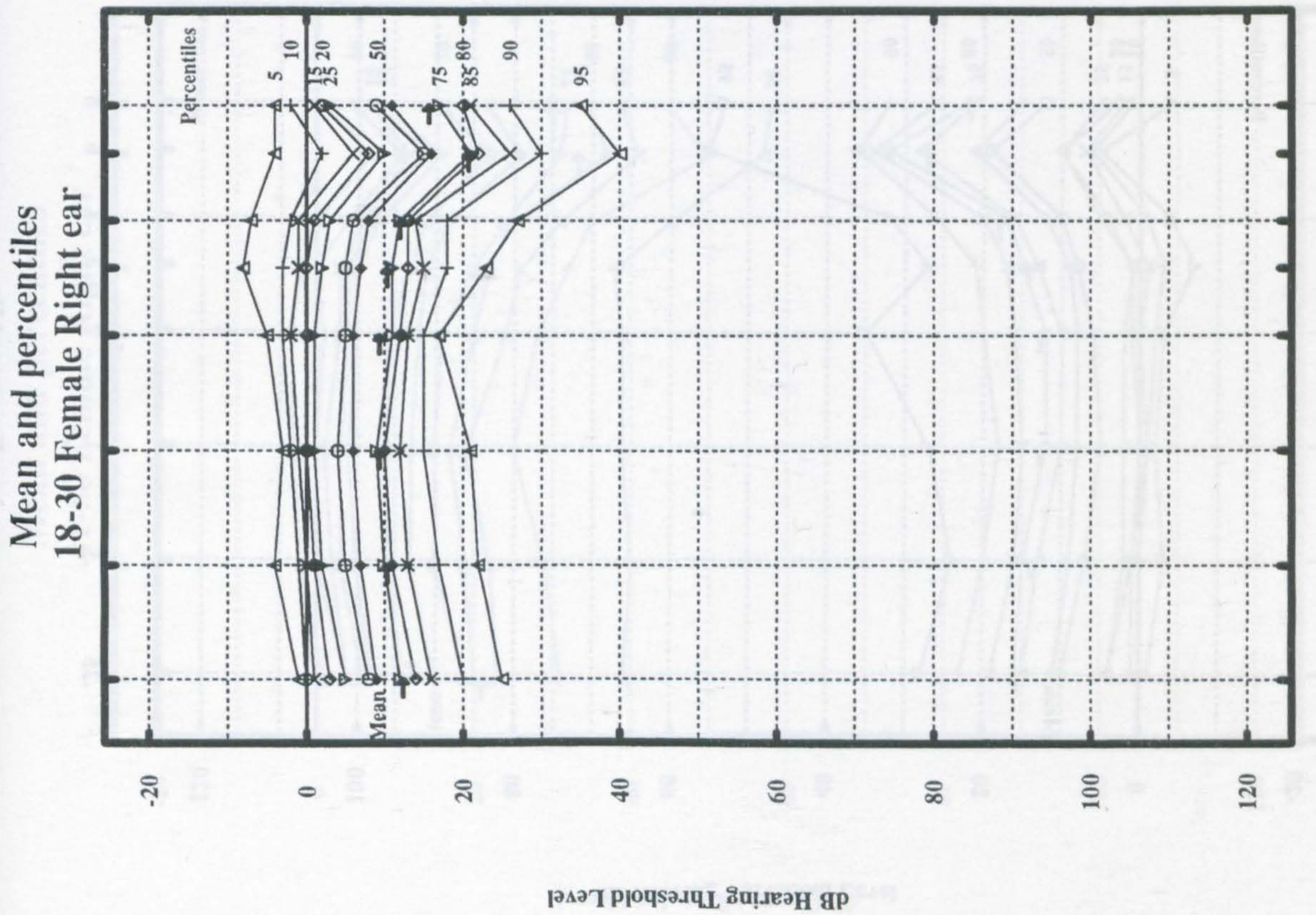
Mean and percentiles
18-30 Female Right ear
Percentiles
5
10
15
20
25
50
75
80
85
90
95
Mean
-20
0
20
40
60
80
100
120
dB Hearing Threshold Level
.25
.5
1
2
3
4
6
8
Frequency kHz

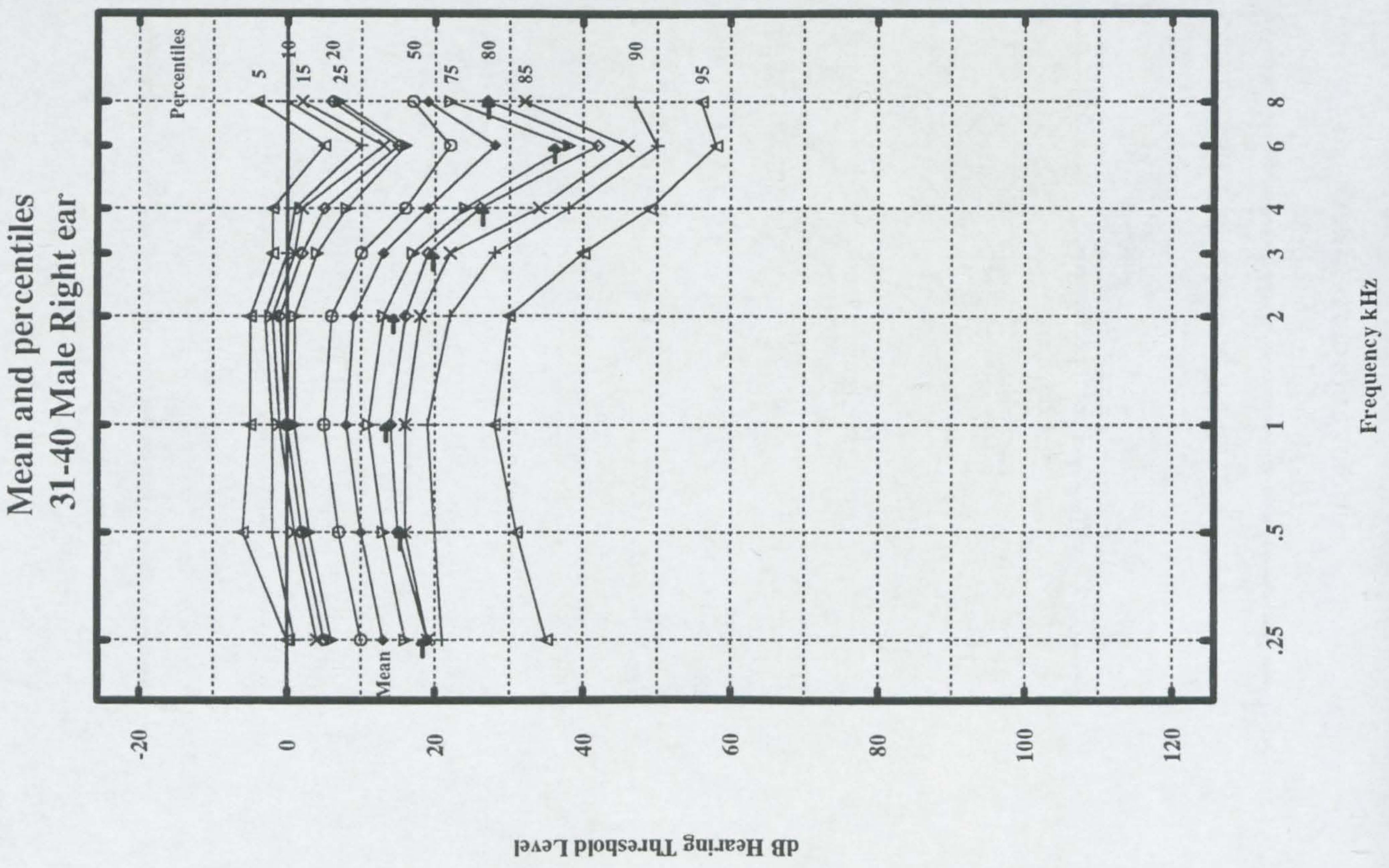
Mean and percentiles
31-40 Male Right ear
Percentiles
5
10
15
20
25
50
75
80
85
90
95
Mean
dB Hearing Threshold Level
-20
0
20
40
60
80
100
120
Frequency kHz
.25
.5
1
2
3
4
6
8

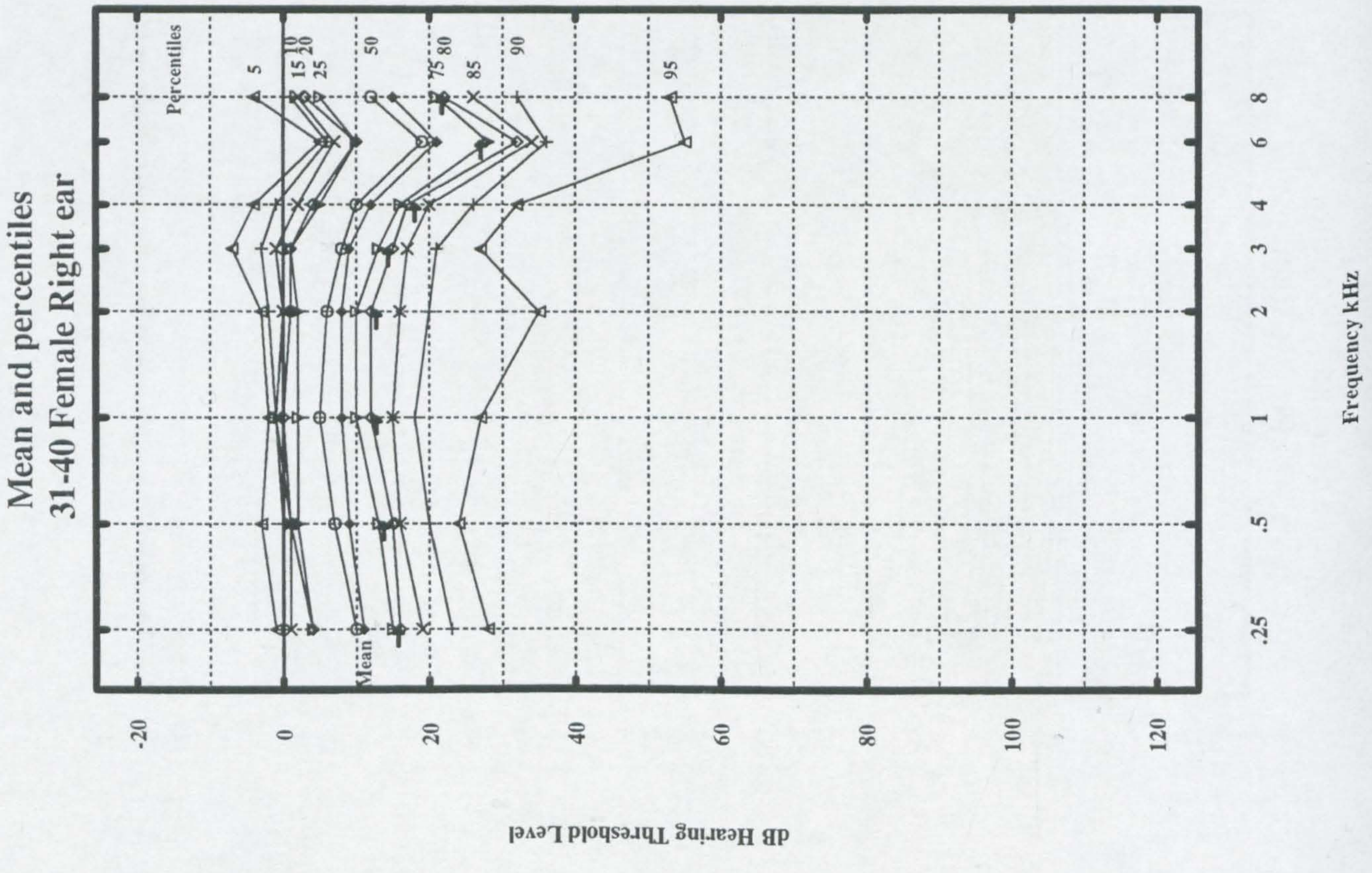
Mean and percentiles
31-40 Female Right ear
Percentiles
5
10
15
20
25
50
75
80
85
90
95
Mean
dB Hearing Threshold Level
-20
0
20
40
60
80
100
120
Frequency kHz
.25
.5
1
2
3
4
6
8

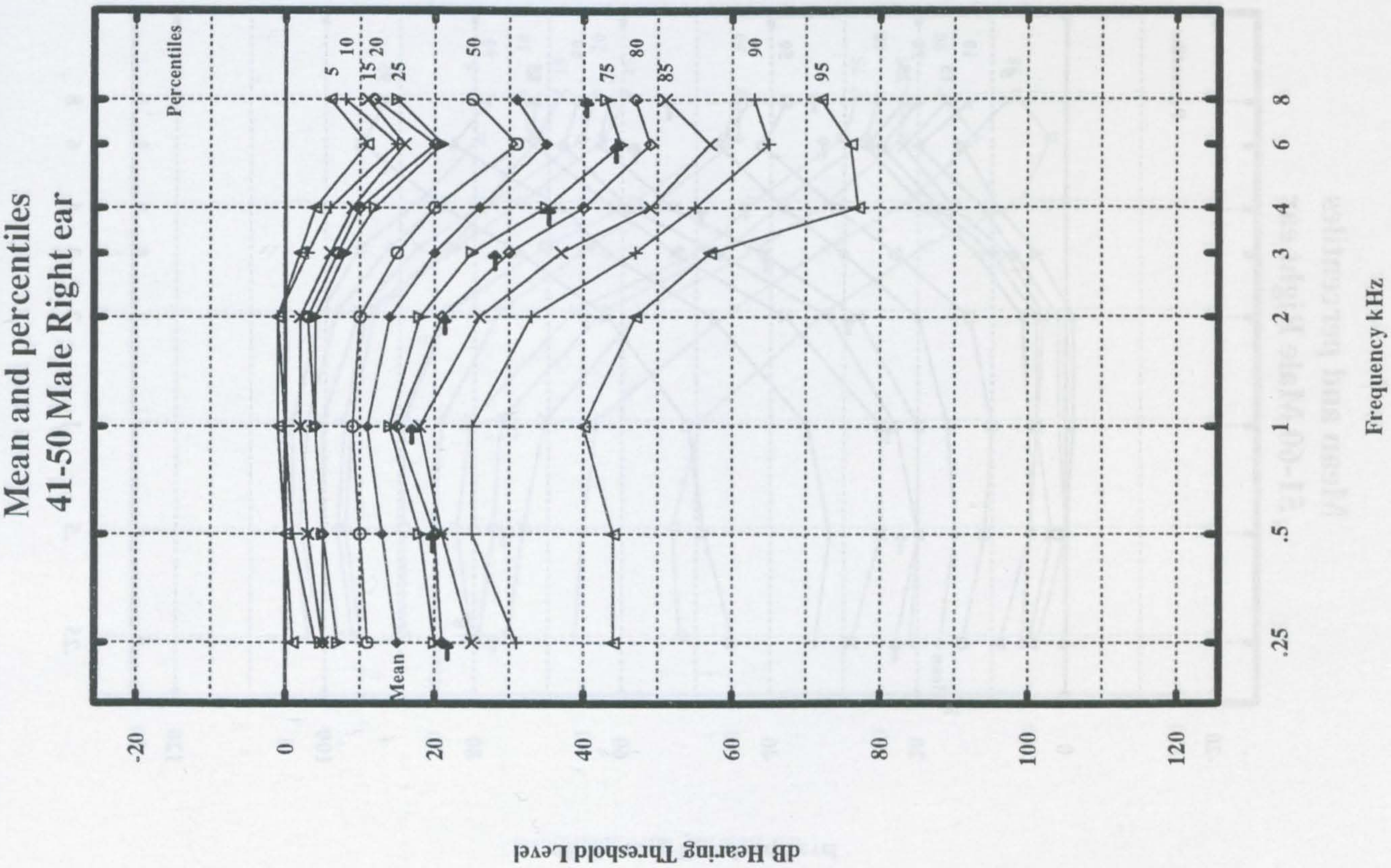
Mean and percentiles
41-50 Male Right ear
Percentiles
5
10
15 20
25
50
75
80
85
90
95
Mean
dB Hearing Threshold Level
-20
0
20
40
60
80
100
120
Frequency kHz
.25
.5
1
2
3
4
6
8

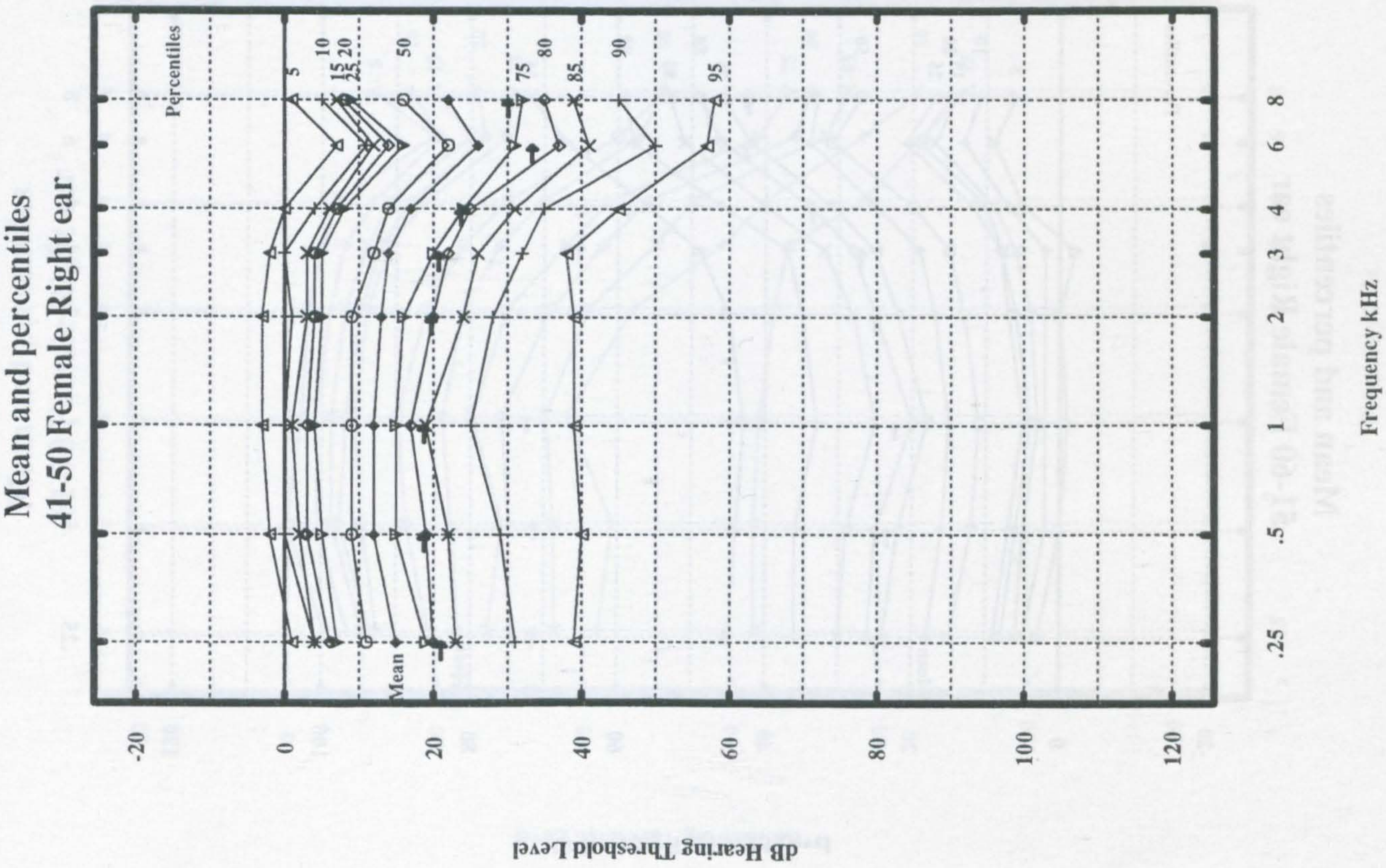
Mean and percentiles
41-50 Female Right ear
Percentiles
5
10
15 20
25
50
75
80
85
90
95
Mean
dB Hearing Threshold Level
-20
0
20
40
60
80
100
120
Frequency kHz
.25
.5
1
2
3
4
6
8

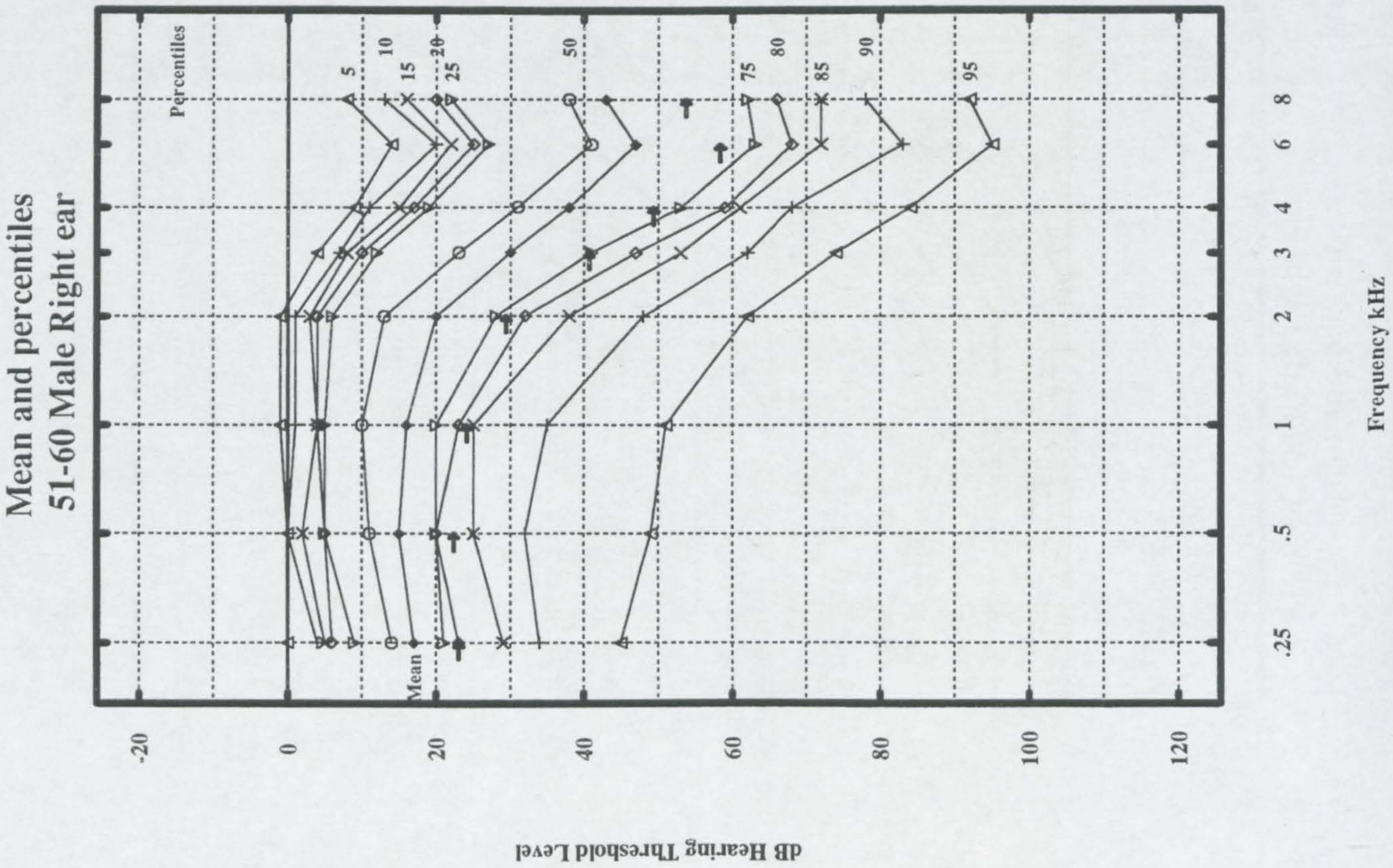
Mean and percentiles
51-60 Male Right ear
Percentiles
5
10
15
20
25
50
75
80
85
90
95
Mean
dB Hearing Threshold Level
-20
0
20
40
60
80
100
120
Frequency kHz
.25
.5
1
2
3
4
6
8

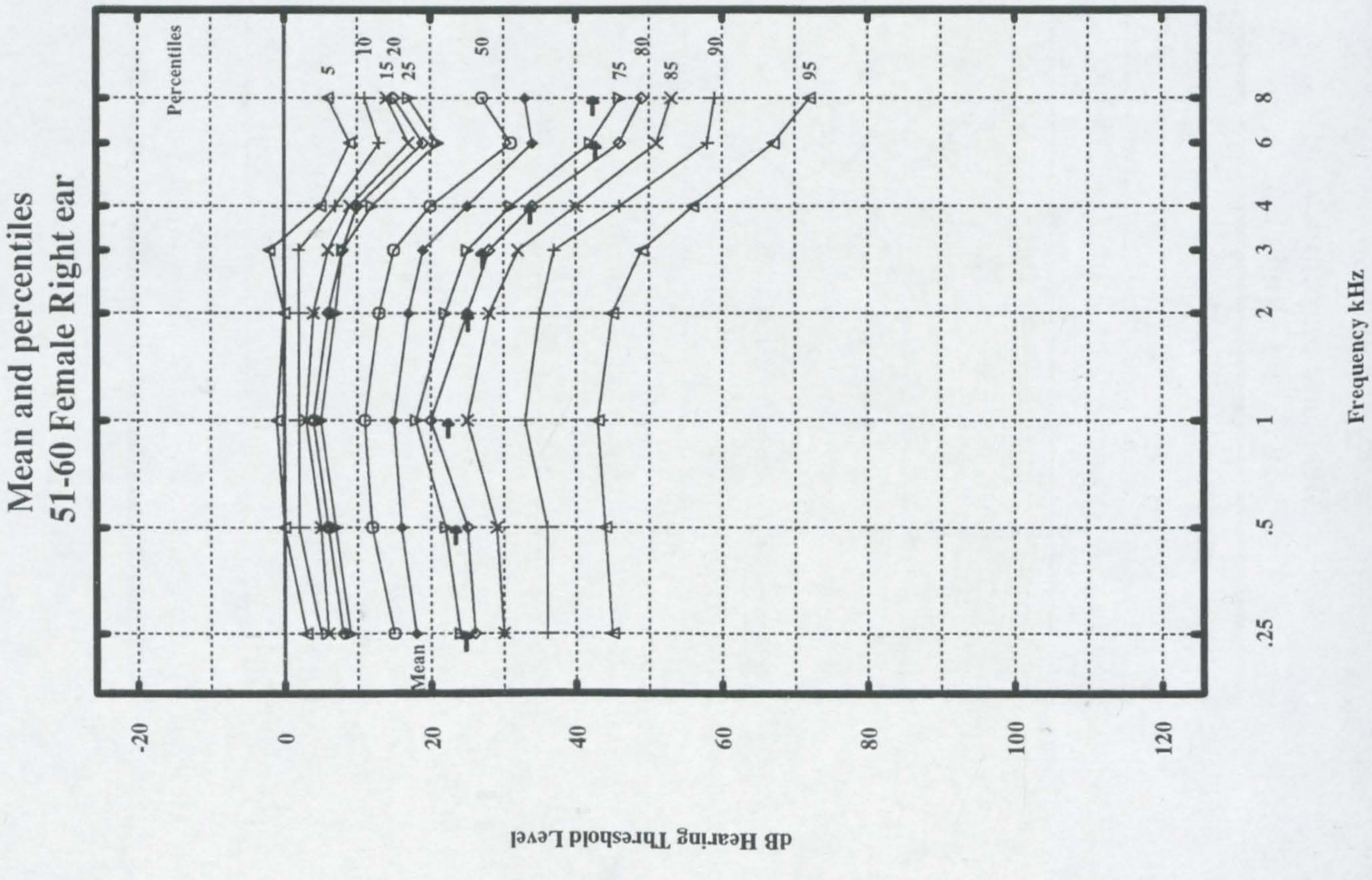
Mean and percentiles
51-60 Female Right ear
Percentiles
5
10
15
20
25
50
75
80
85
90
95
Mean
dB Hearing Threshold Level
-20
0
20
40
60
80
100
120
Frequency kHz
.25
.5
1
2
3
4
6
8

Mean and percentiles
61-70 Male Right ear

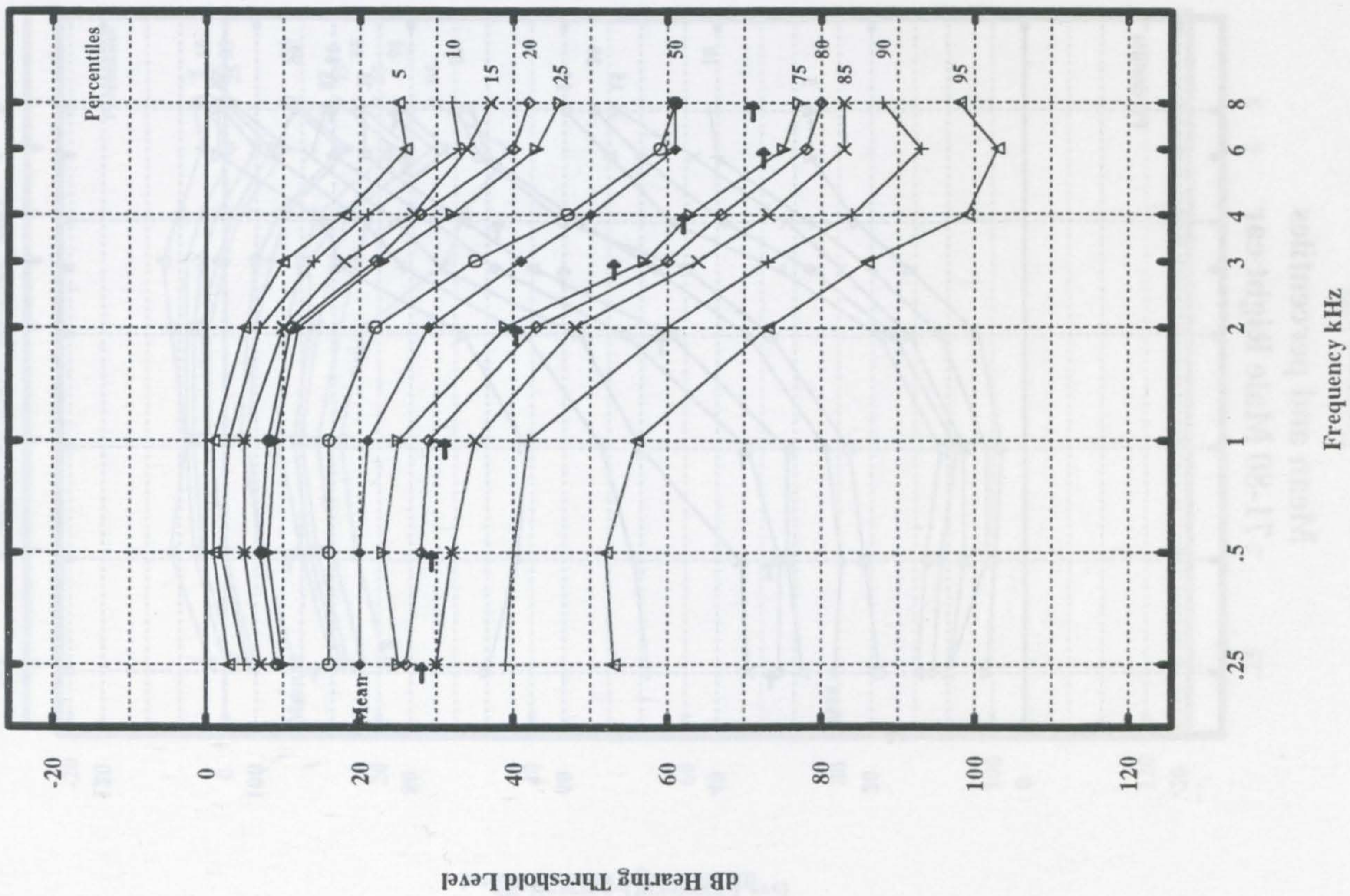

Mean and percentiles
61-70 Female Right ear

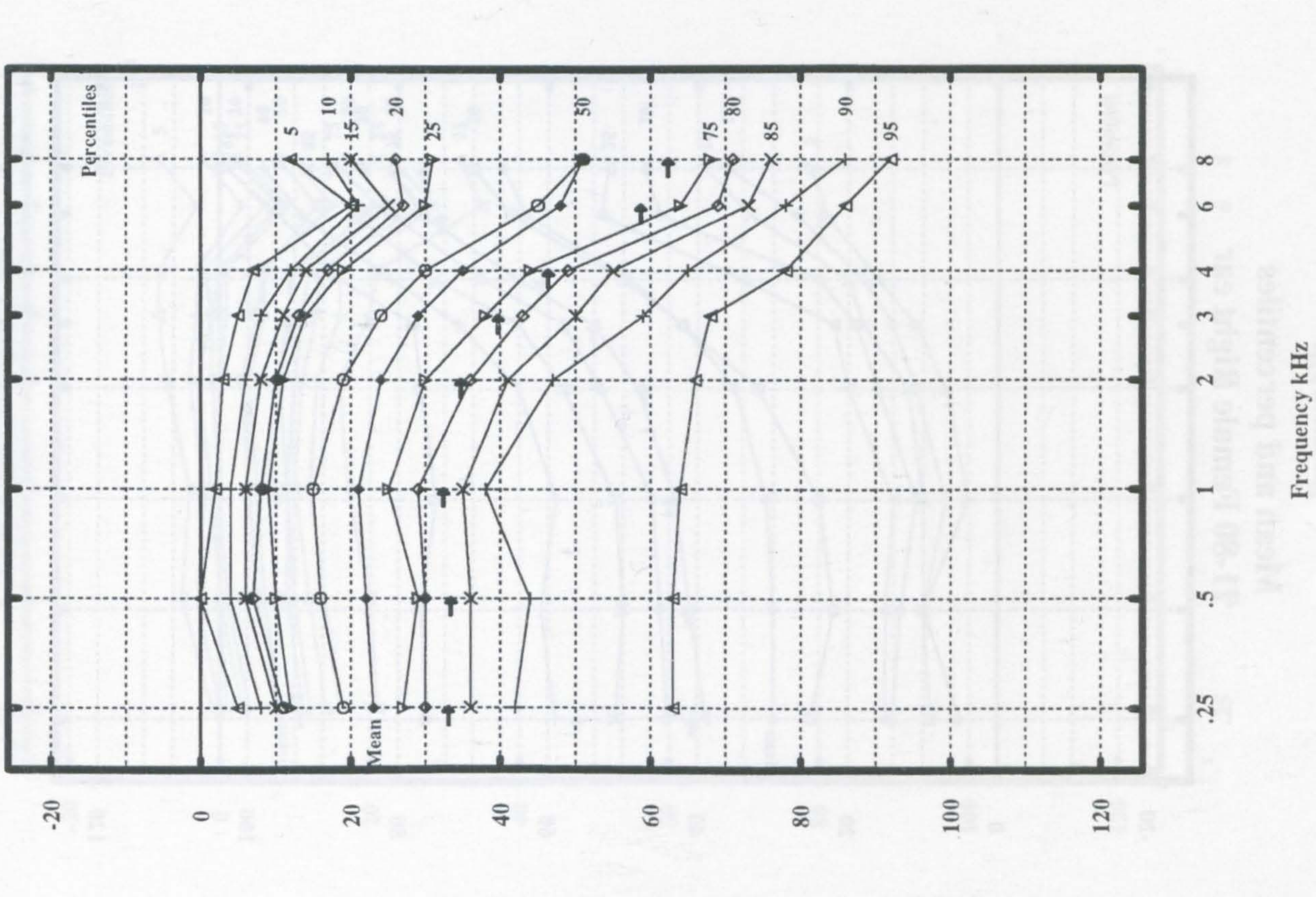

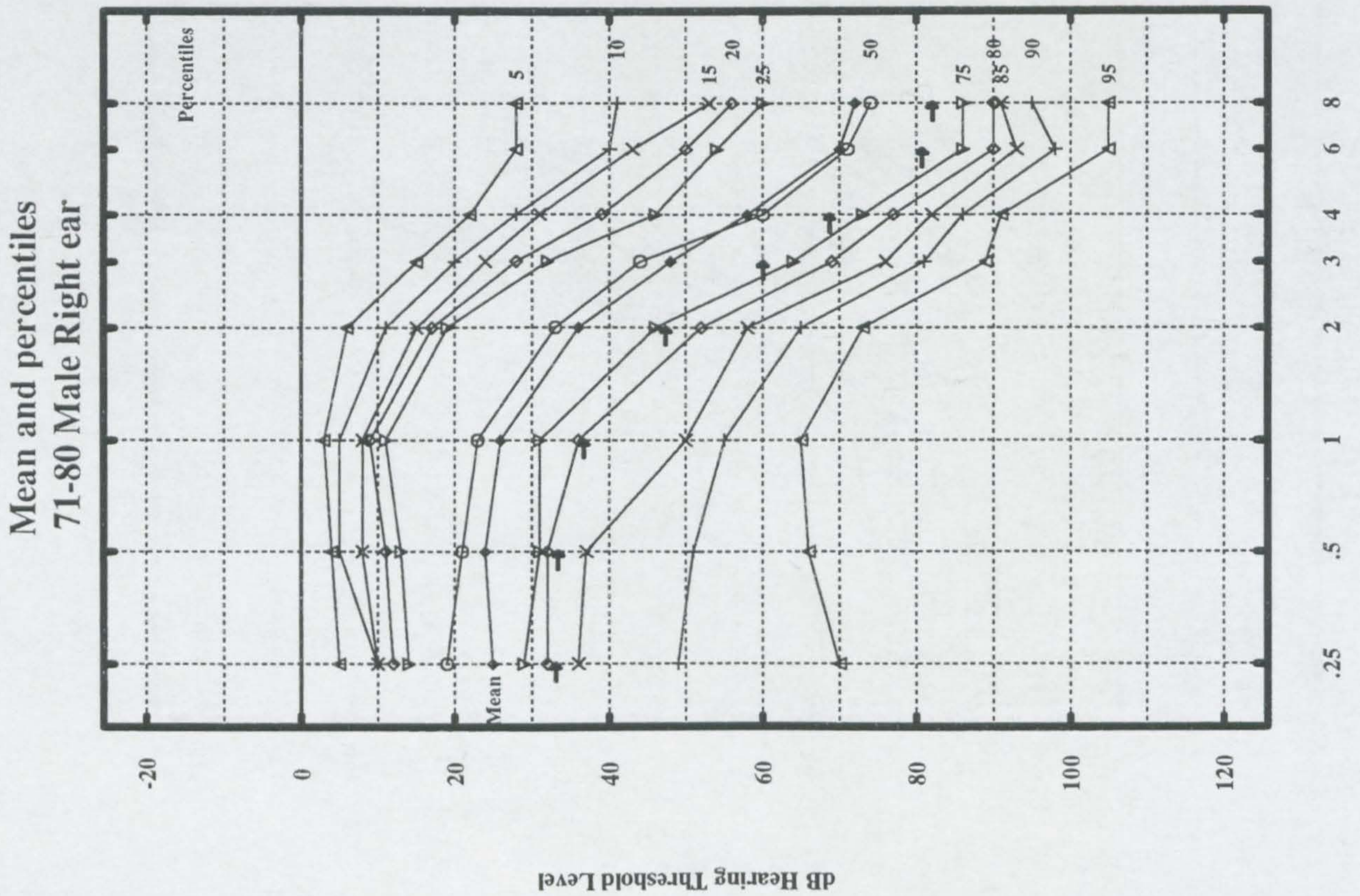
Mean and percentiles
71-80 Male Right ear
Percentiles
Mean
5
10
15
20
25
50
75
80
85
90
95
-20
0
20
40
60
80
100
120
dB Hearing Threshold Level
.25
.5
1
2
3
4
6
8
Frequency kHz

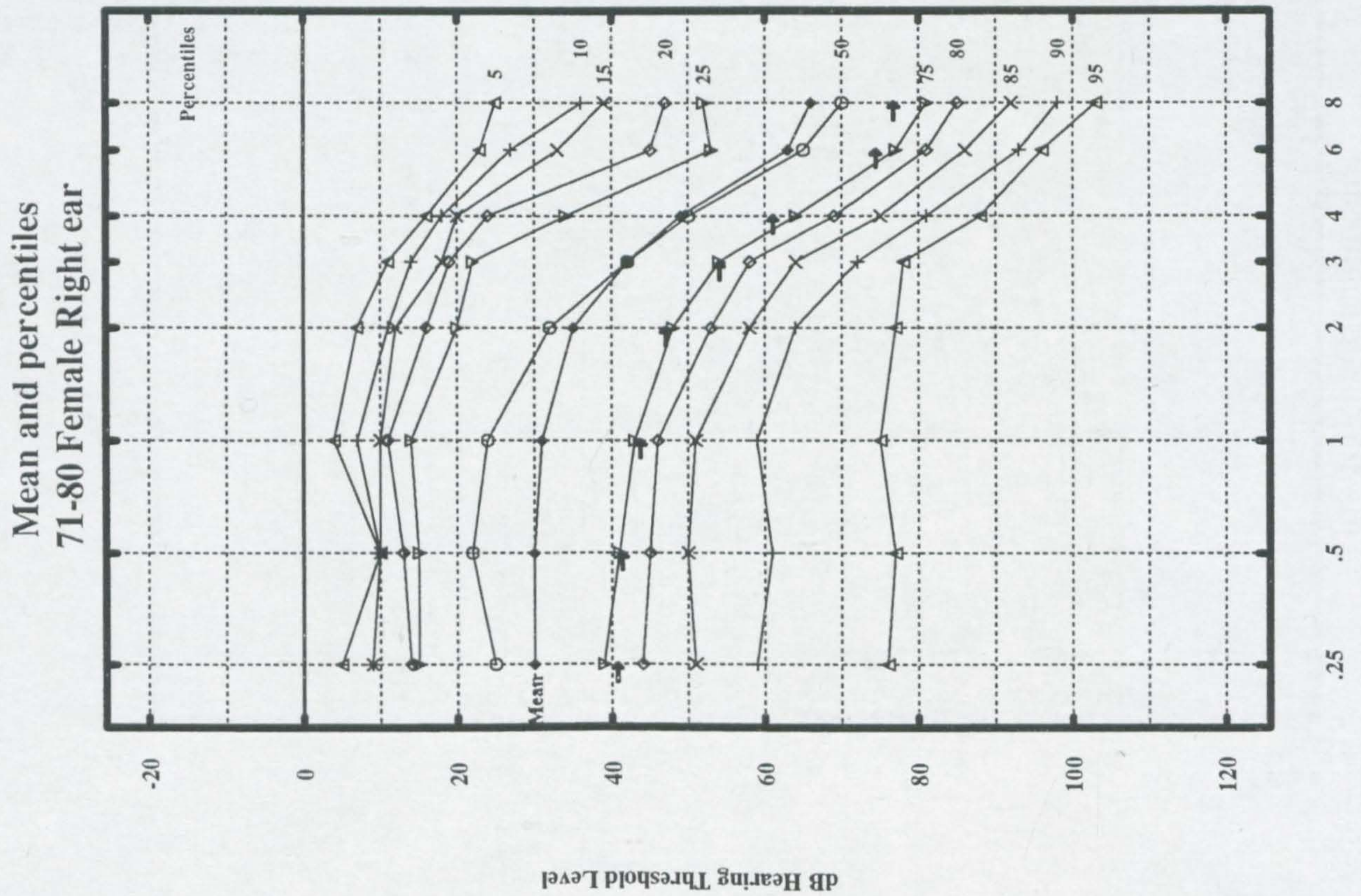
Mean and percentiles
71-80 Female Right ear
Percentiles
Mean
5
10
15
20
25
50
75
80
85
90
95
-20
0
20
40
60
80
100
120
dB Hearing Threshold Level
.25
.5
1
2
3
4
6
8
Frequency kHz

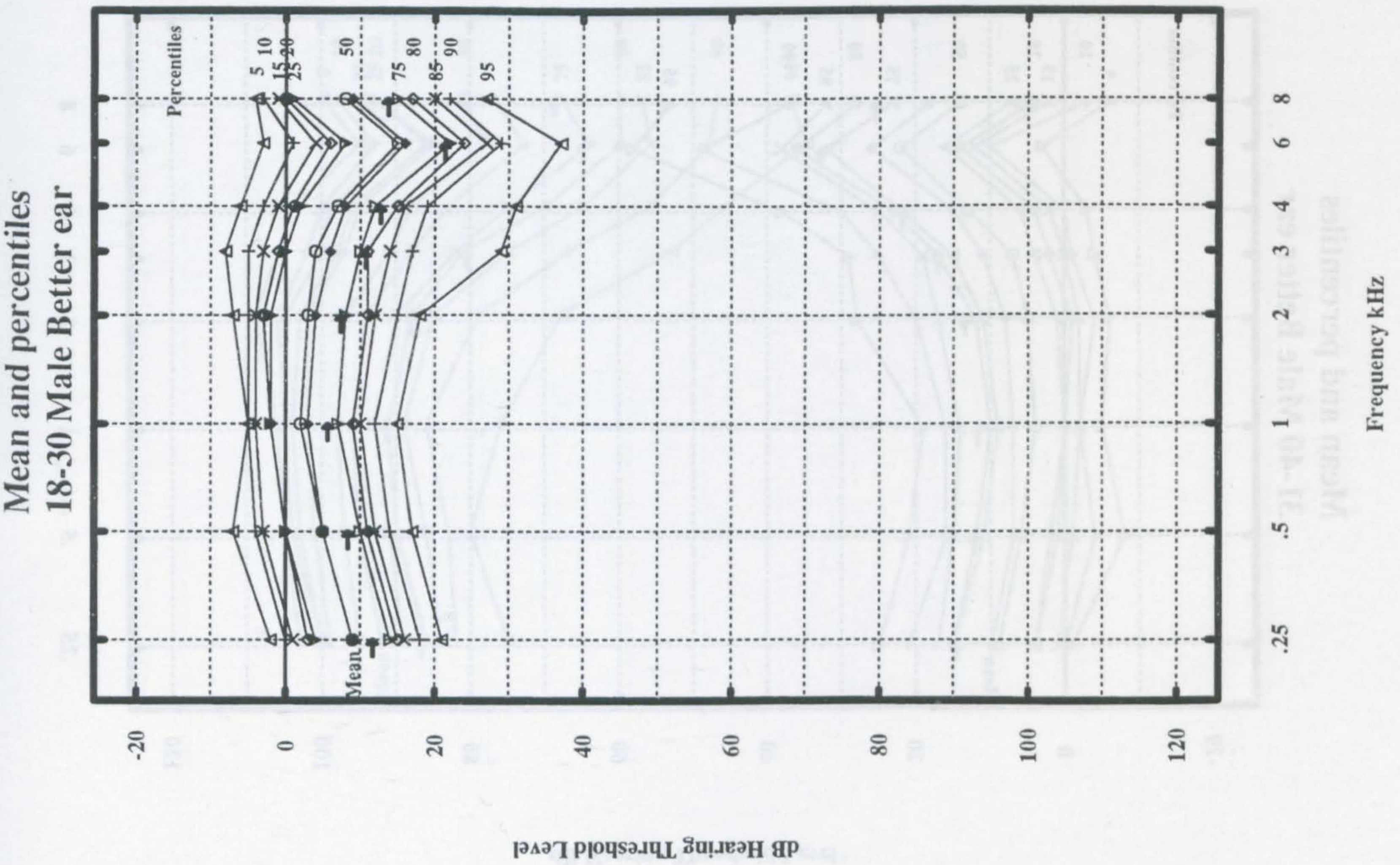
Mean and percentiles
18-30 Male Better ear
Percentiles
5 10 15 20 25 50 75 80 85 90 95
Mean
dB Hearing Threshold Level
-20
0
20
40
60
80
100
120
Frequency kHz
.25 .5 1 2 3 4 6 8

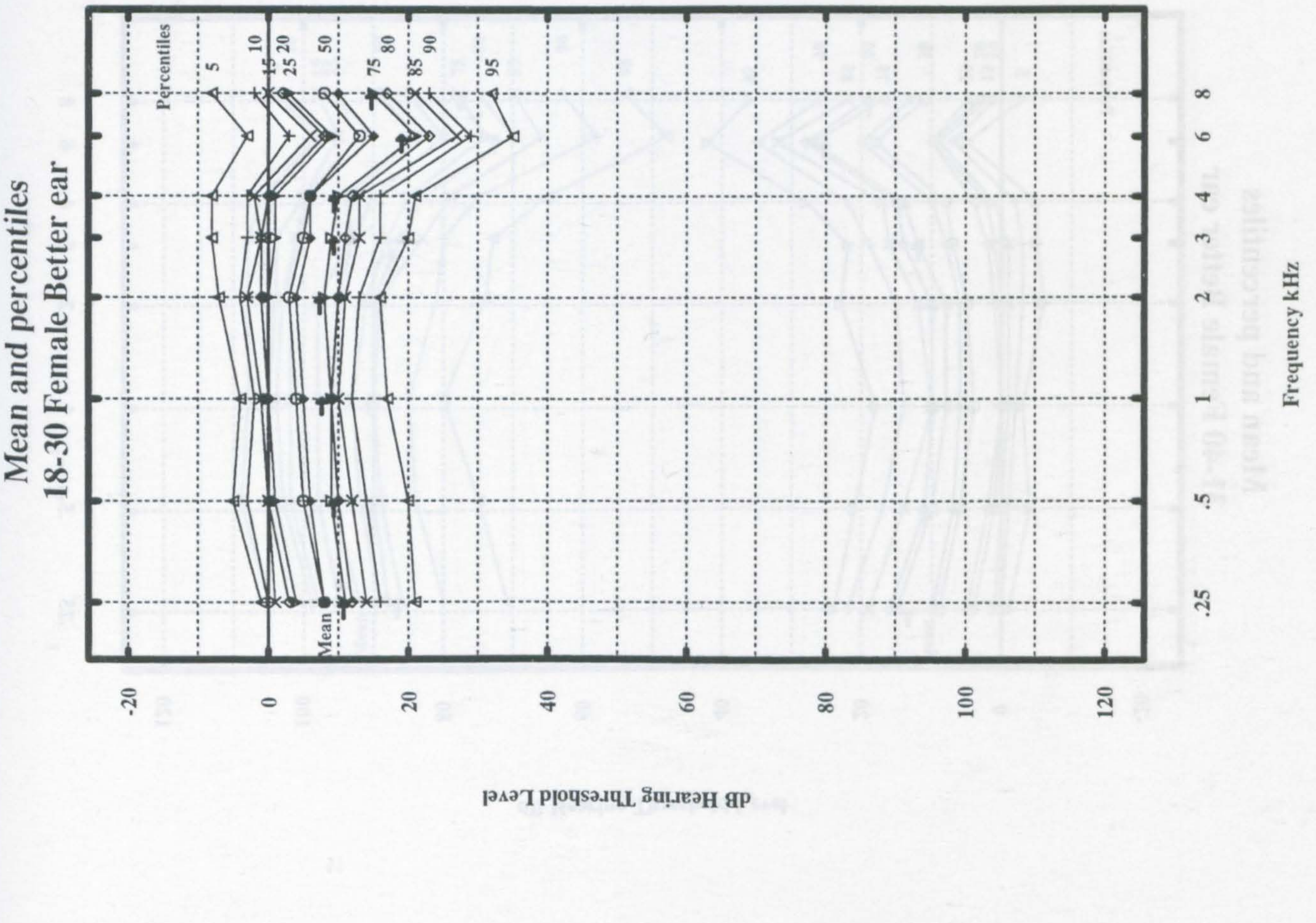
Mean and percentiles
18-30 Female Better ear
Percentiles
5 10 15 20 25 50 75 80 85 90 95
Mean
dB Hearing Threshold Level
-20
0
20
40
60
80
100
120
Frequency kHz
.25 .5 1 2 3 4 6 8

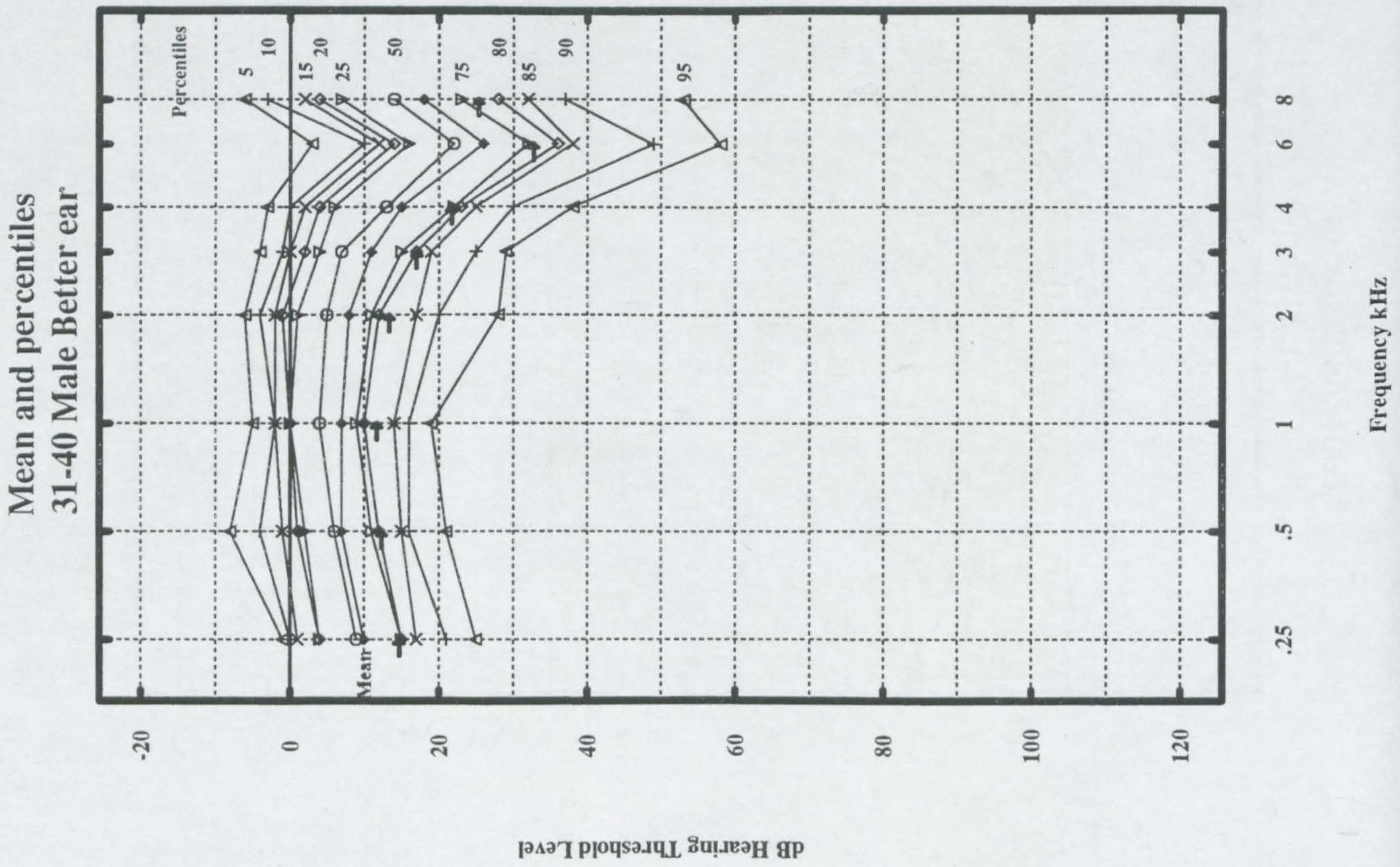
Mean and percentiles
31-40 Male Better ear
Percentiles
5
10
15
20
25
50
75
80
85
90
95
Mean
-20
0
20
40
60
80
100
120
dB Hearing Threshold Level
.25
.5
1
2
3
4
6
8
Frequency kHz

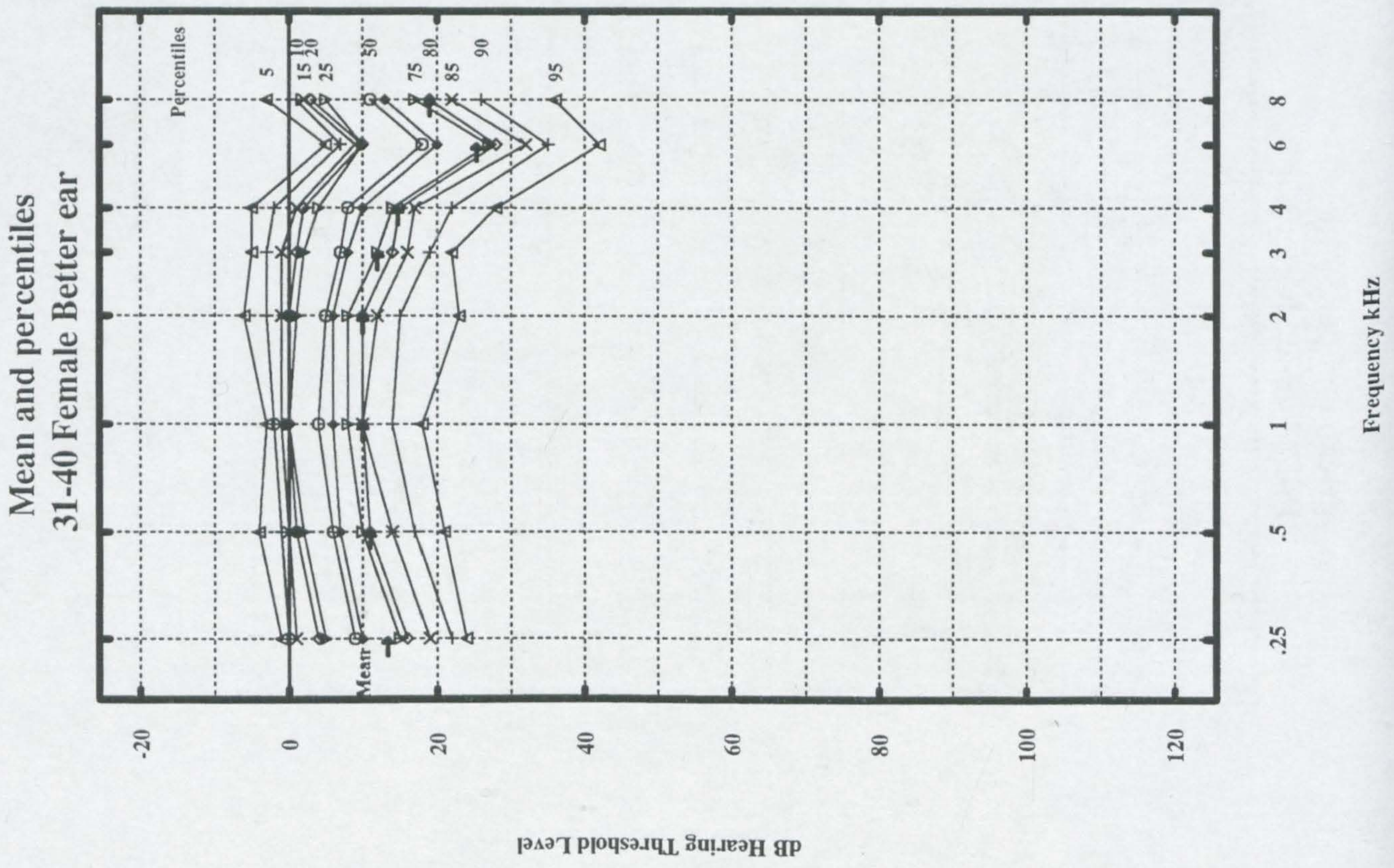
Mean and percentiles
31-40 Female Better ear
Percentiles
5
10
15
20
25
50
75
80
85
90
95
Mean
-20
0
20
40
60
80
100
120
dB Hearing Threshold Level
.25
.5
1
2
3
4
6
8
Frequency kHz

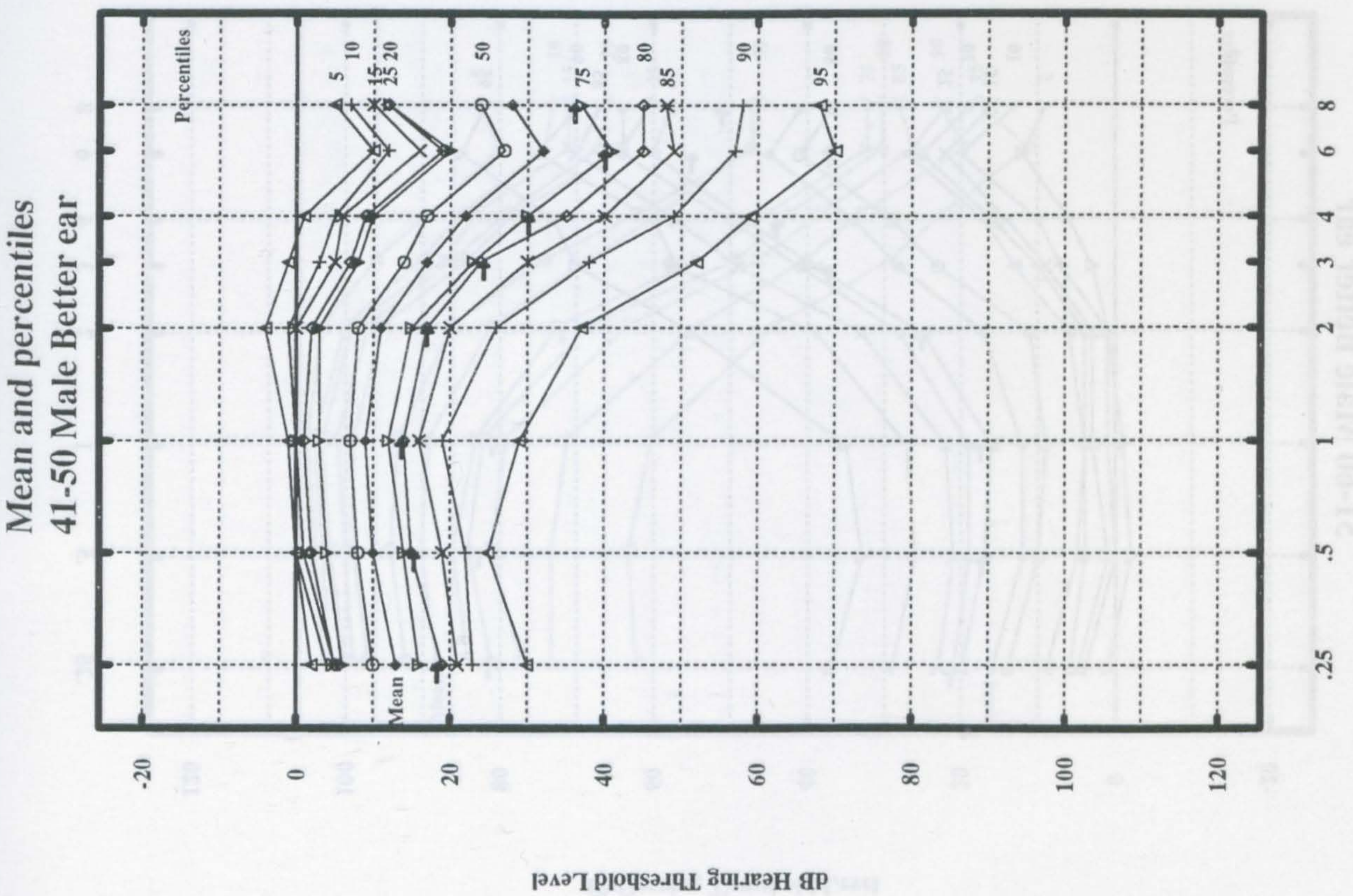
Mean and percentiles
41-50 Male Better ear
Percentiles
5 10 15 25 20 50 75 80 85 90 95
Mean
dB Hearing Threshold Level
-20 0 20 40 60 80 100 120
.25 .5 1 2 3 4 6 8
Frequency kHz

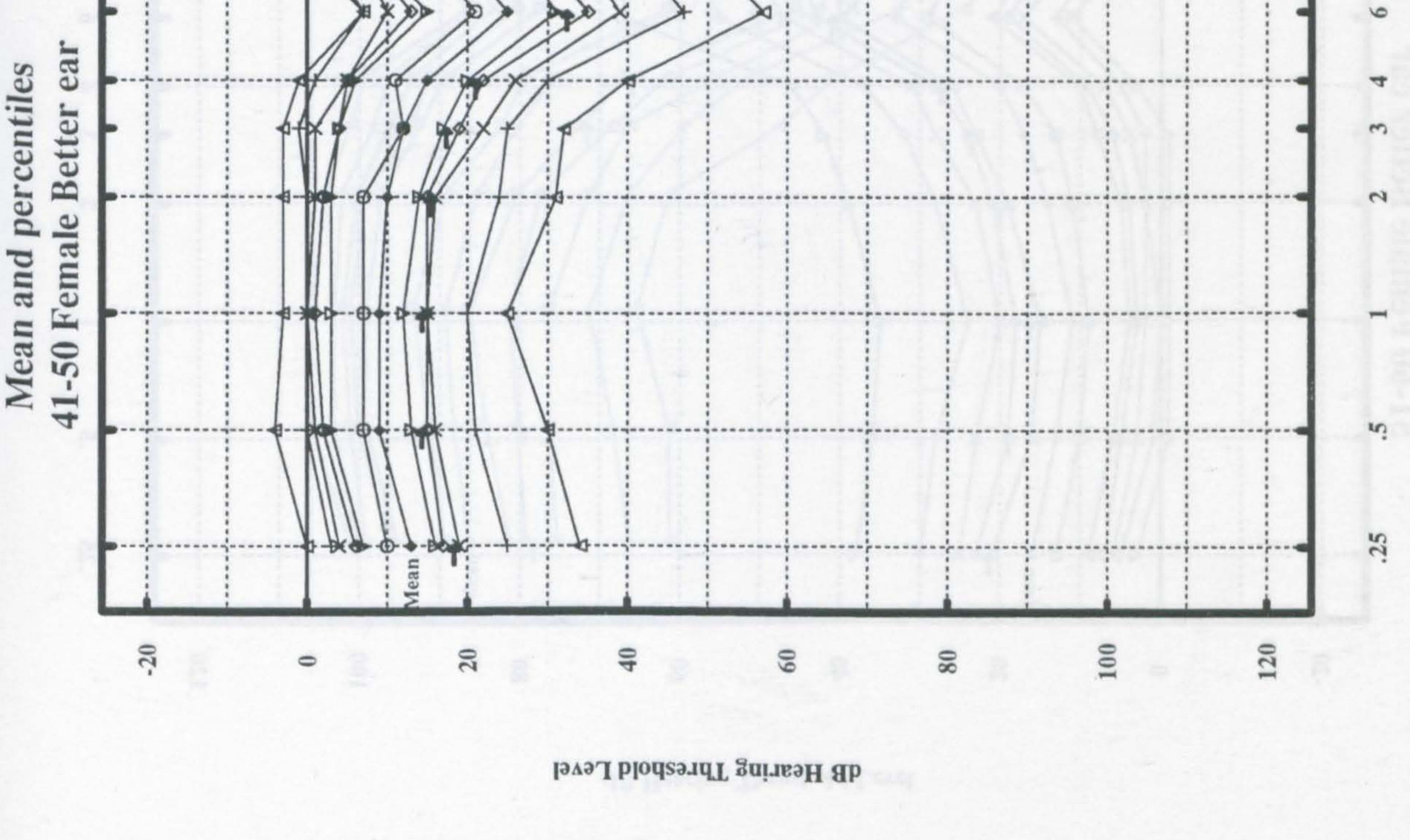
Mean and percentiles
41-50 Female Better ear
Percentiles
5 10 15 25 20 50 75 80 85 90 95
Mean
dB Hearing Threshold Level
-20 0 20 40 60 80 100 120
.25 .5 1 2 3 4 6 8
Frequency kHz

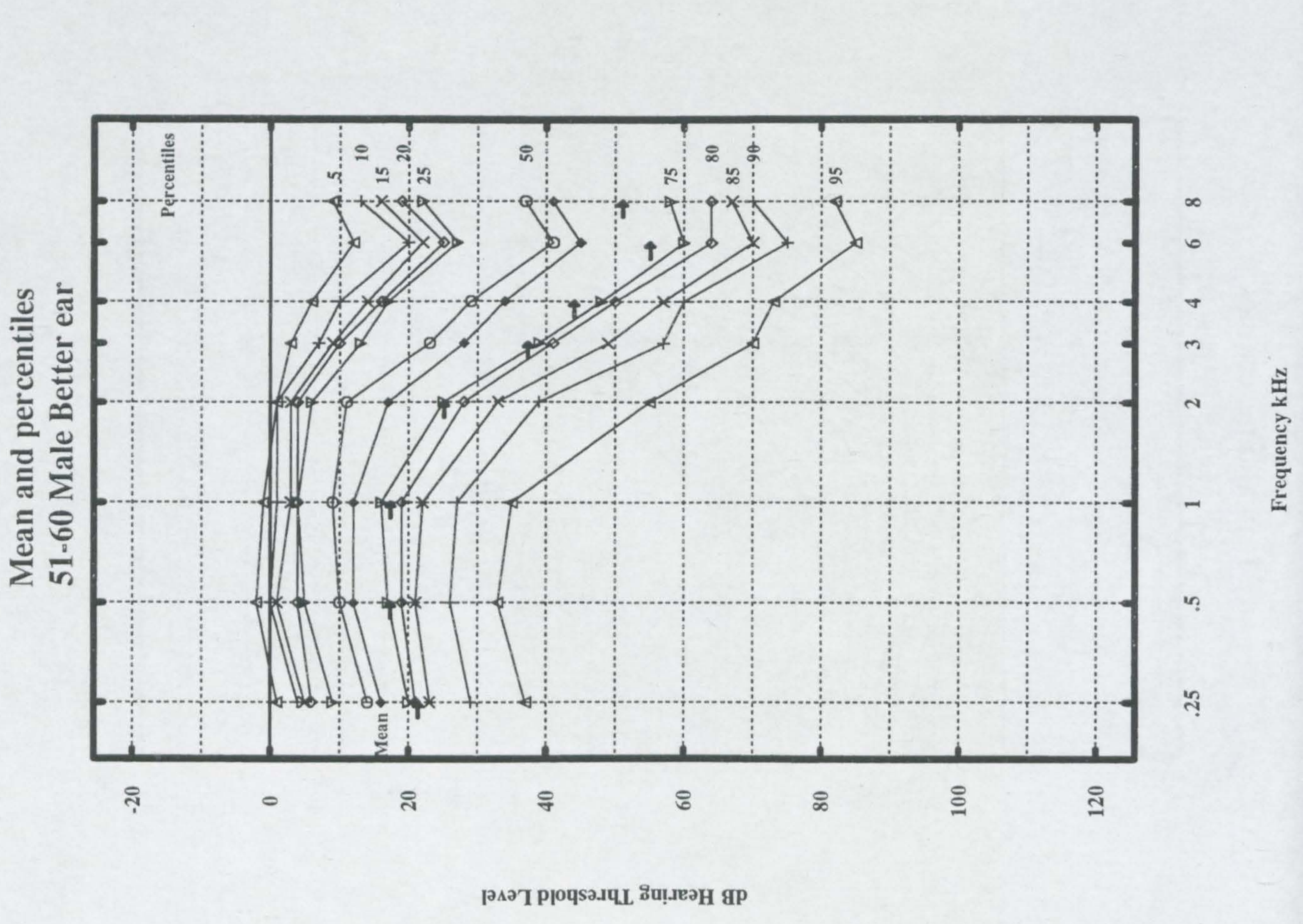
Mean and percentiles
51-60 Male Better ear
Percentiles
5
10
15
20
25
50
75
80
85
90
95
Mean
dB Hearing Threshold Level
-20
0
20
40
60
80
100
120
Frequency kHz
.25
.5
1
2
3
4
6
8

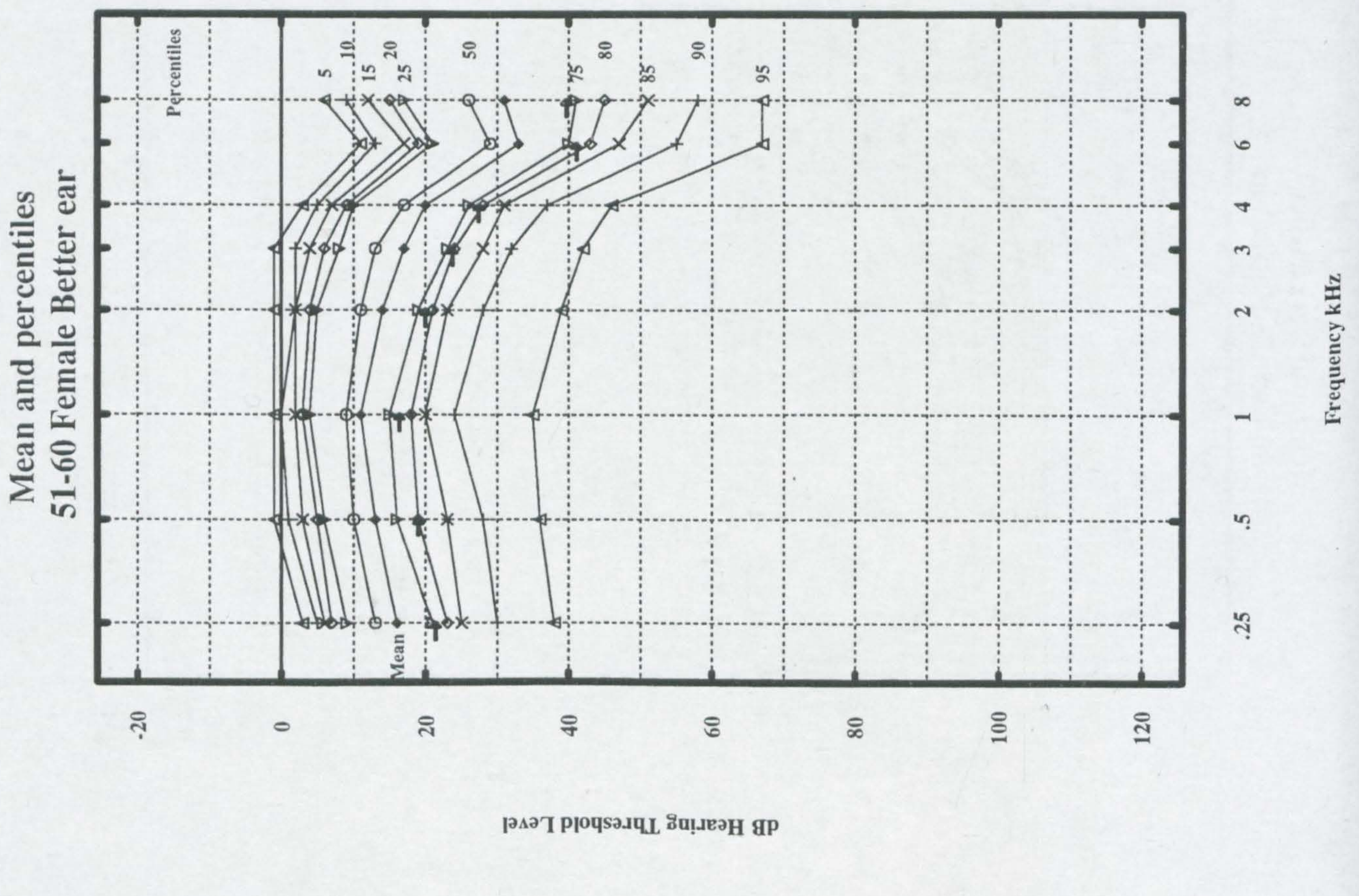
Mean and percentiles
51-60 Female Better ear
Percentiles
5
10
15
20
25
50
75
80
85
90
95
Mean
dB Hearing Threshold Level
-20
0
20
40
60
80
100
120
Frequency kHz
.25
.5
1
2
3
4
6
8

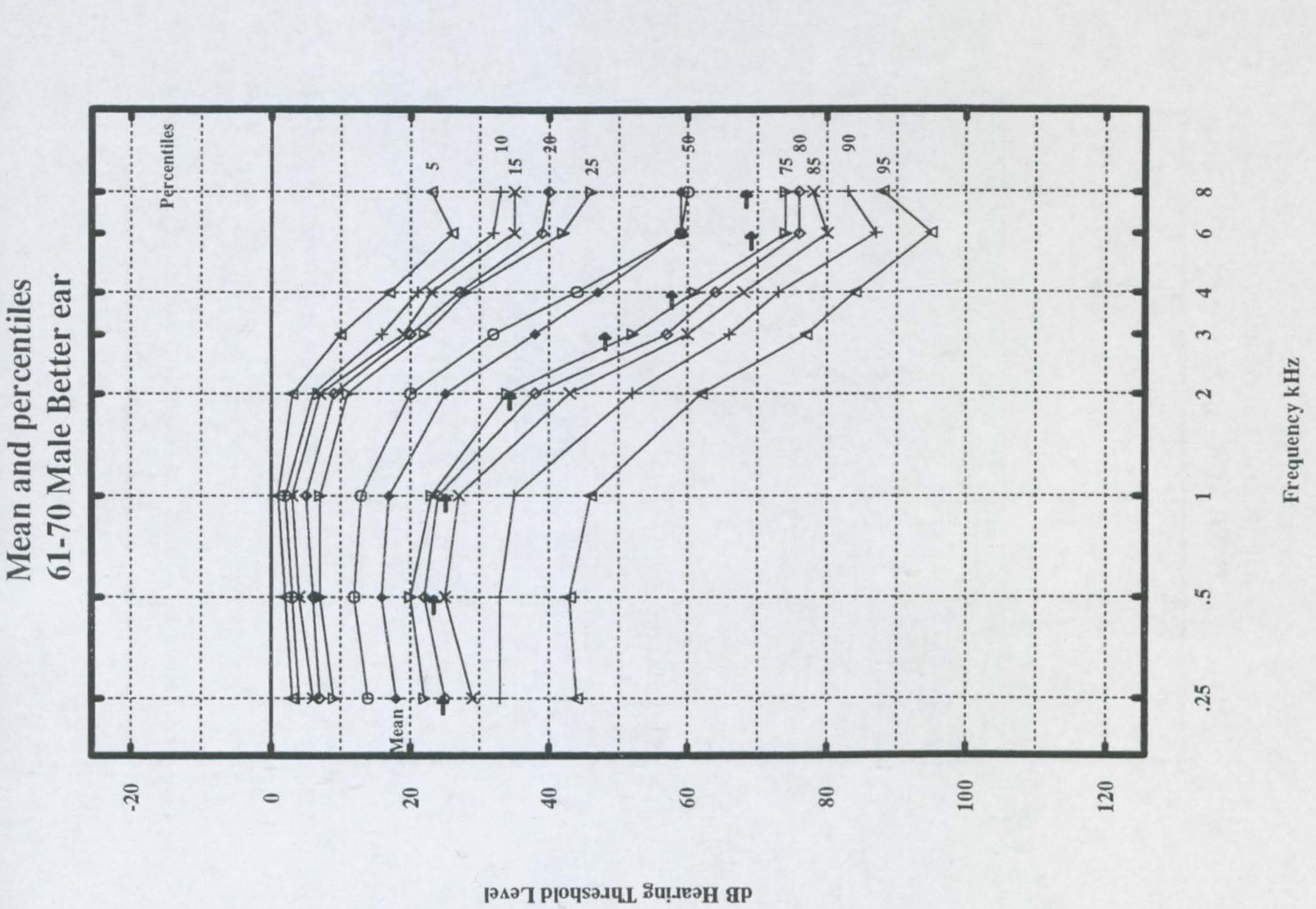
Mean and percentiles
61-70 Male Better ear
dB Hearing Threshold Level
-20
0
20
40
60
80
100
120
.25
.5
1
2
3
4
6
8
Frequency kHz
Percentiles
Mean
5
10
15
20
25
50
75
80
85
90
95

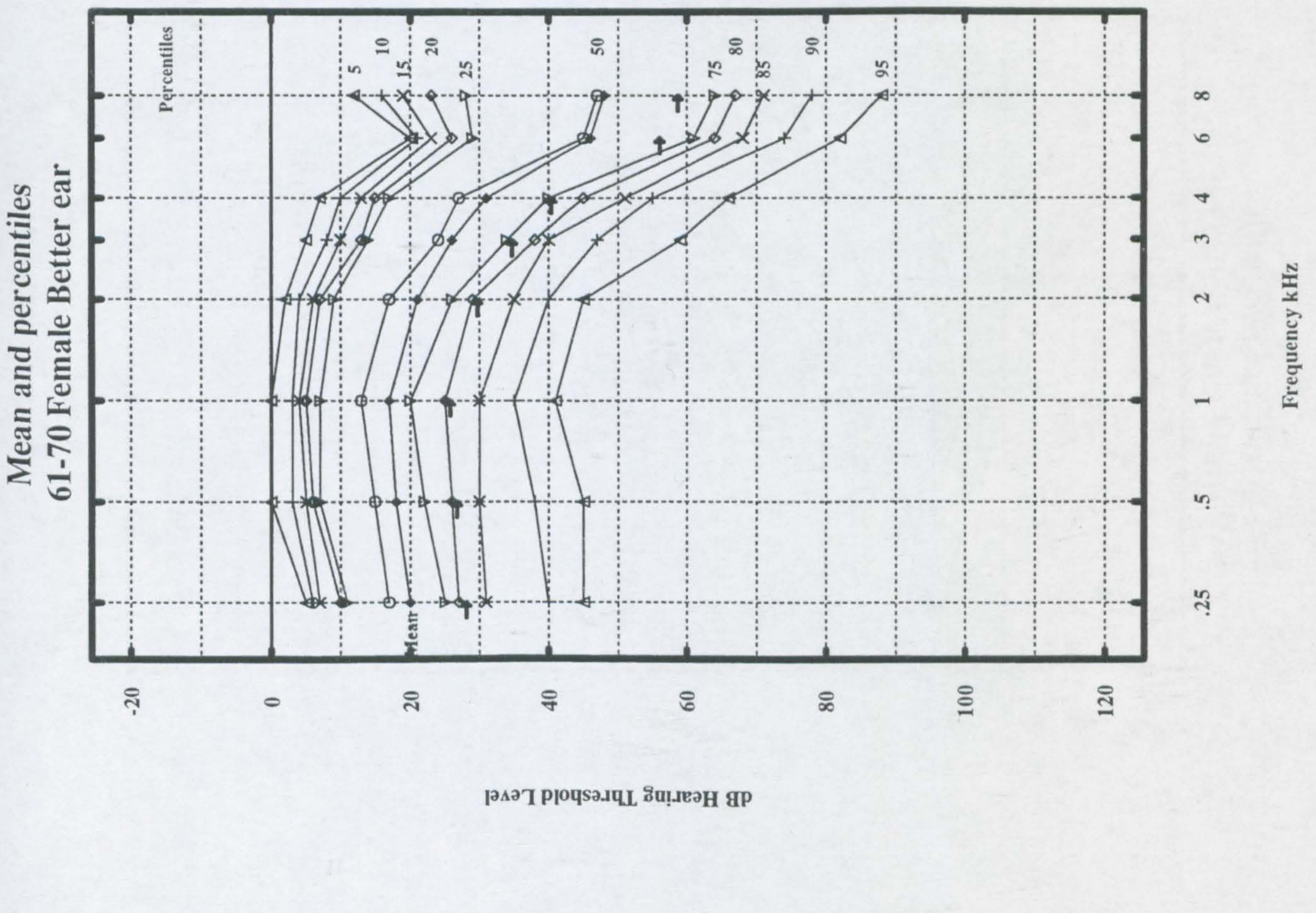
Mean and percentiles
61-70 Female Better ear
dB Hearing Threshold Level
-20
0
20
40
60
80
100
120
.25
.5
1
2
3
4
6
8
Frequency kHz
Percentiles
Mean
5
10
15
20
25
50
75
80
85
90
95

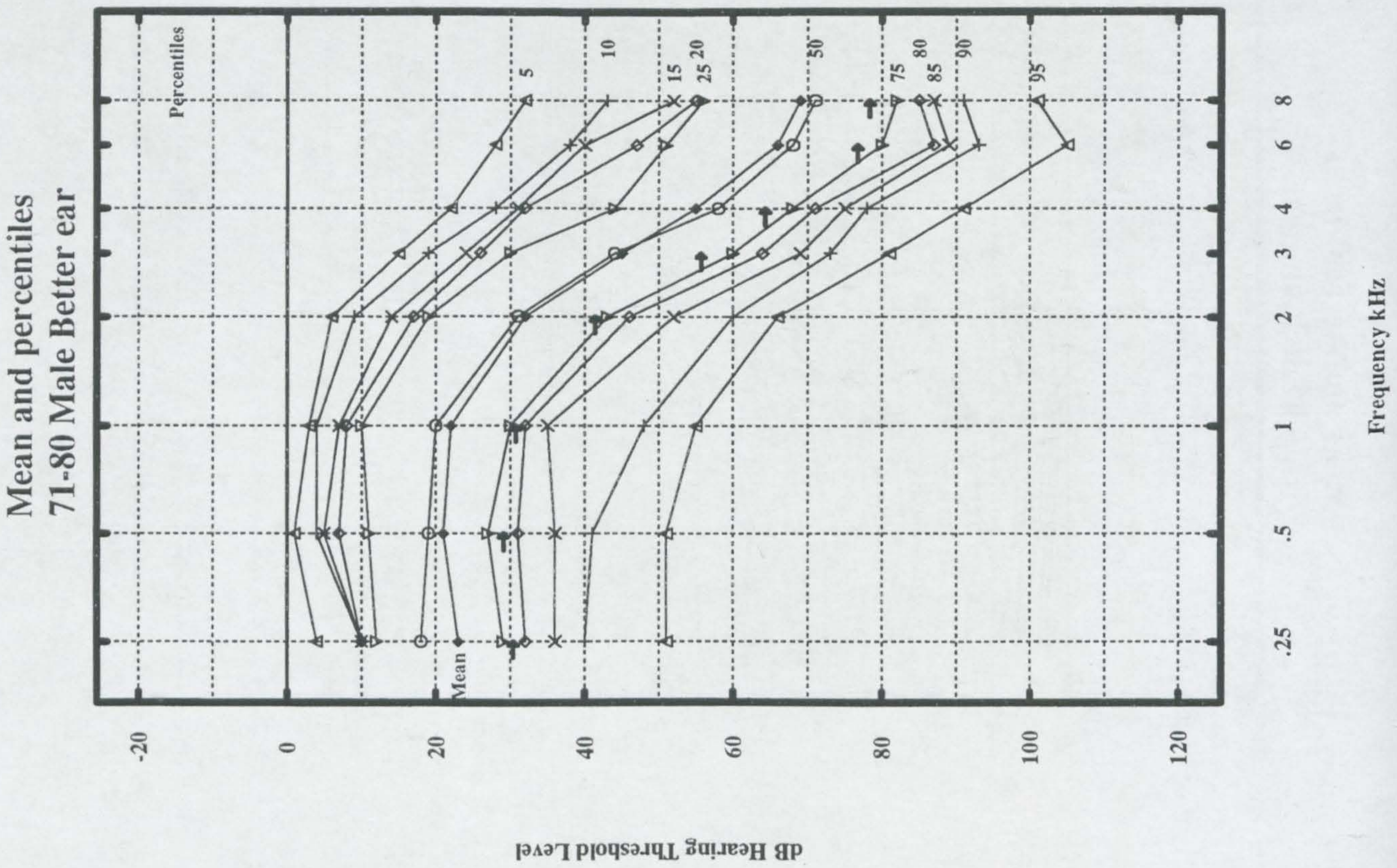
Mean and percentiles
71-80 Male Better ear
dB Hearing Threshold Level
-20
0
20
40
60
80
100
120
Frequency kHz
.25
.5
1
2
3
4
6
8
Percentiles
Mean
5
10
15
20
25
50
75
80
85
90
95

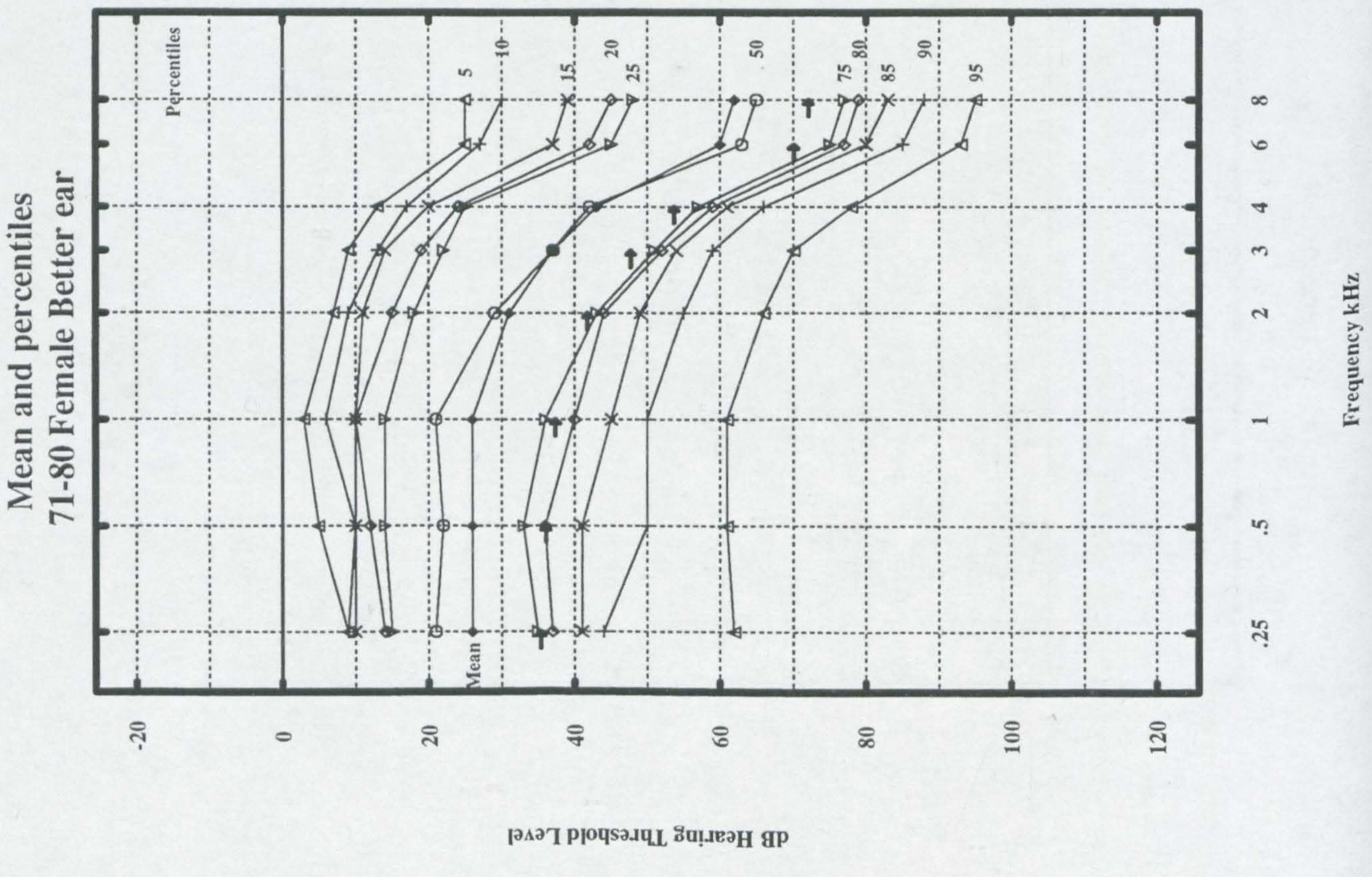
Mean and percentiles
71-80 Female Better ear
dB Hearing Threshold Level
-20
0
20
40
60
80
100
120
Frequency kHz
.25
.5
1
2
3
4
6
8
Percentiles
Mean
5
10
15
20
25
50
75
80
85
90
95

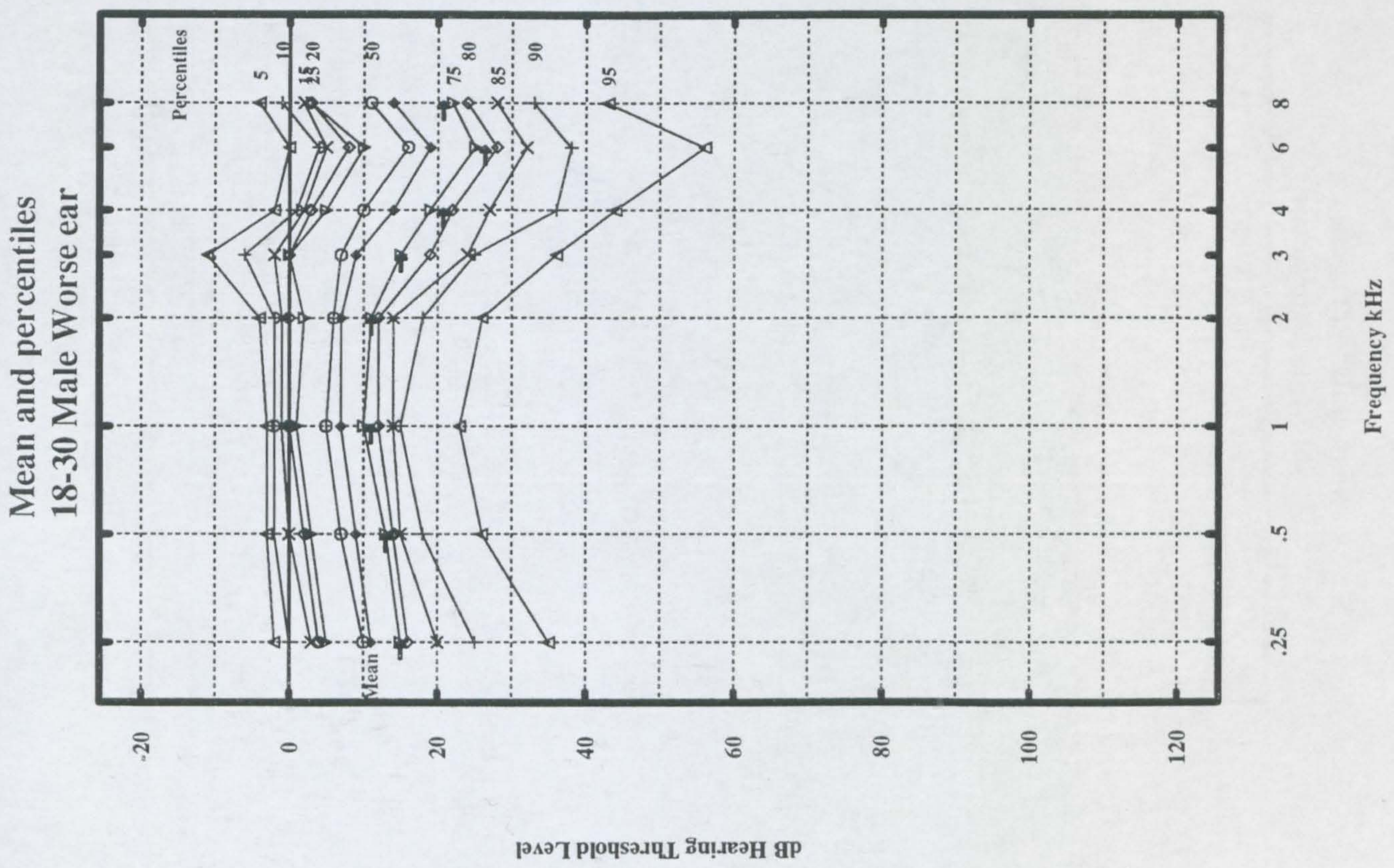
Mean and percentiles
18-30 Male Worse ear
Percentiles
5
10
15
20
50
75
80
85
90
95
Mean
-20
0
20
40
60
80
100
120
dB Hearing Threshold Level
.25
.5
1
2
3
4
6
8
Frequency kHz

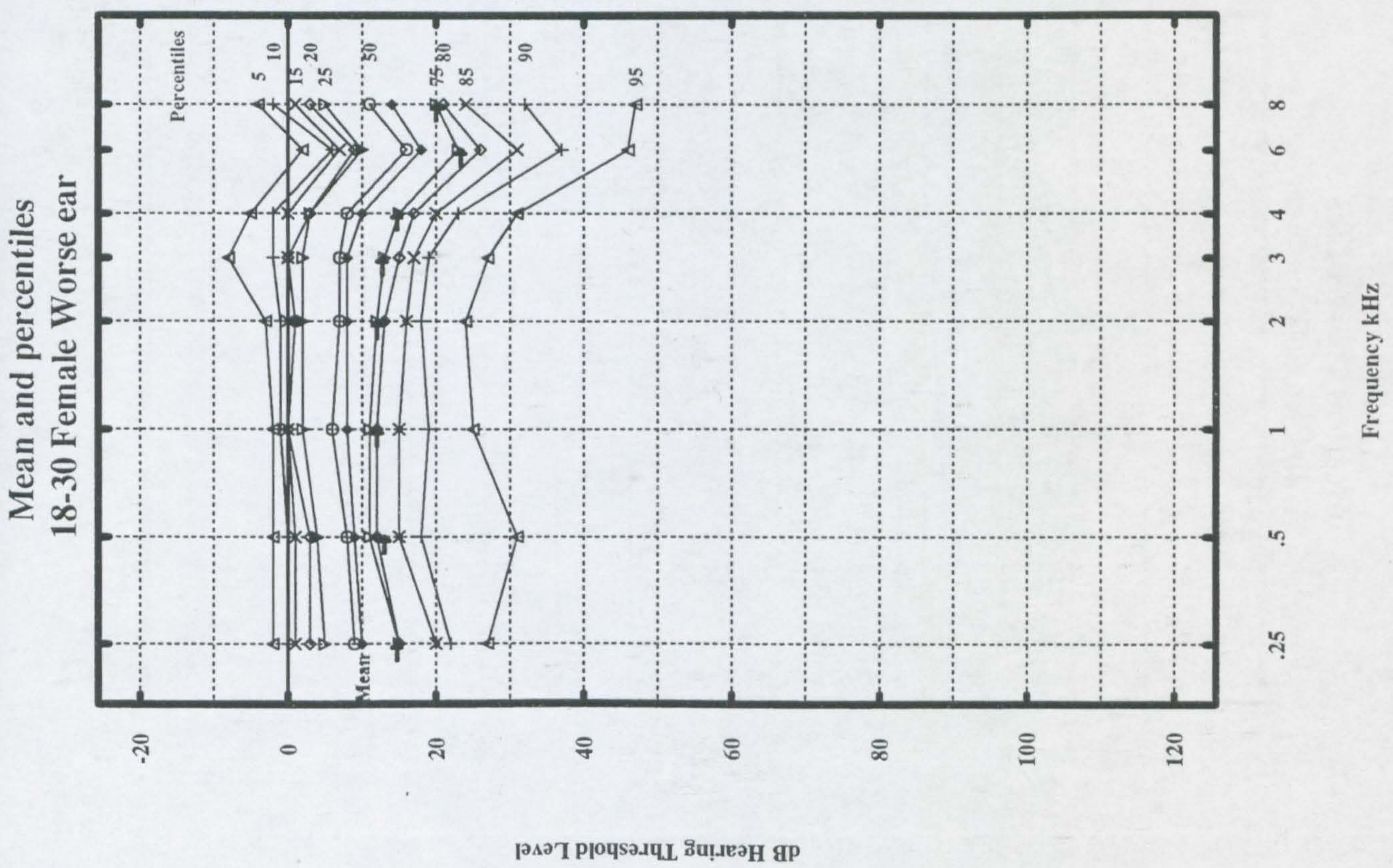
Mean and percentiles
18-30 Female Worse ear
Percentiles
5
10
15
20
25
50
75
80
85
90
95
Mean
-20
0
20
40
60
80
100
120
dB Hearing Threshold Level
.25
.5
1
2
3
4
6
8
Frequency kHz

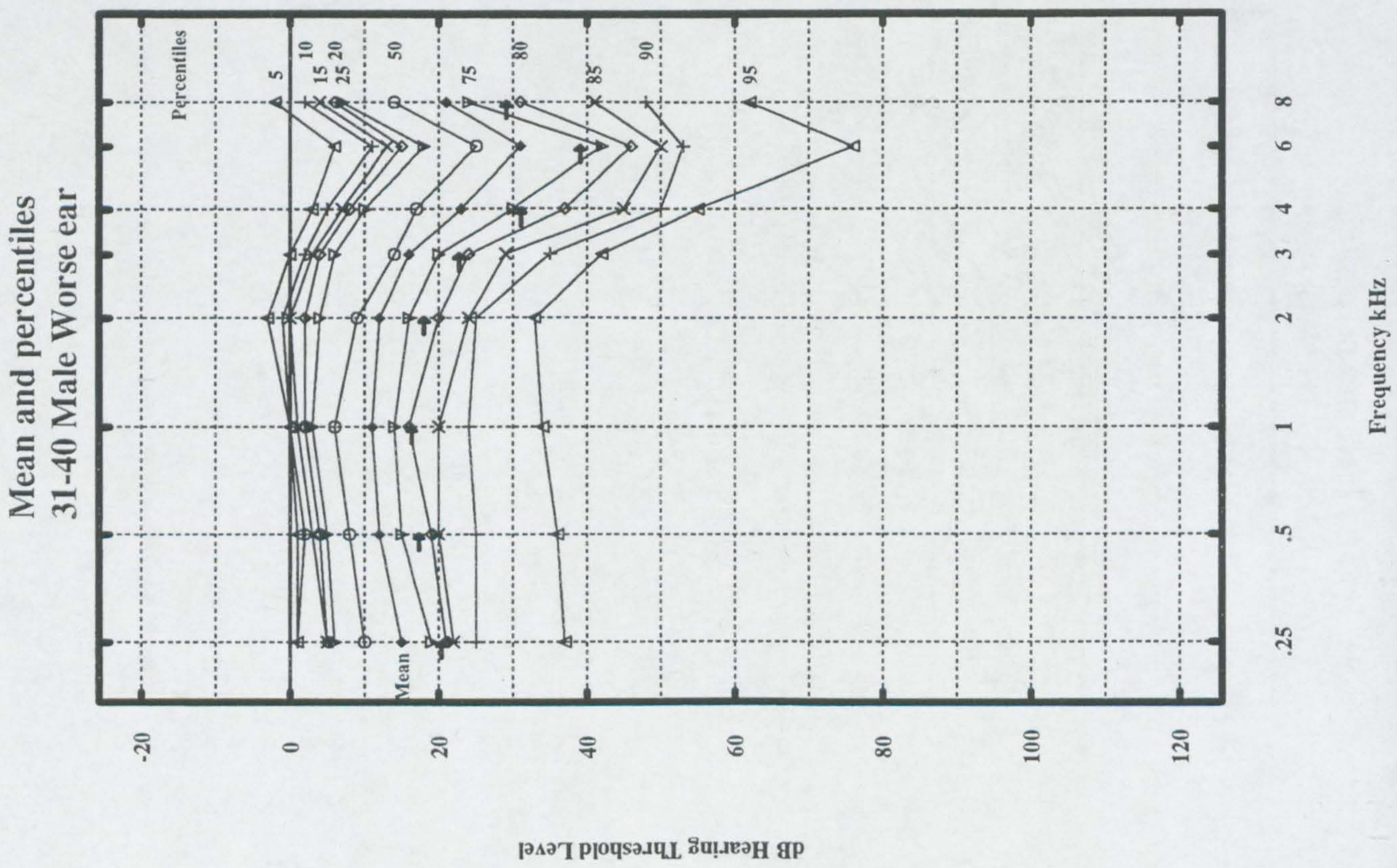
Mean and percentiles
31-40 Male Worse ear
Percentiles
5
10
15
20
25
50
75
80
85
90
95
Mean
-20
0
20
40
60
80
100
120
dB Hearing Threshold Level
.25
.5
1
2
3
4
6
8
Frequency kHz

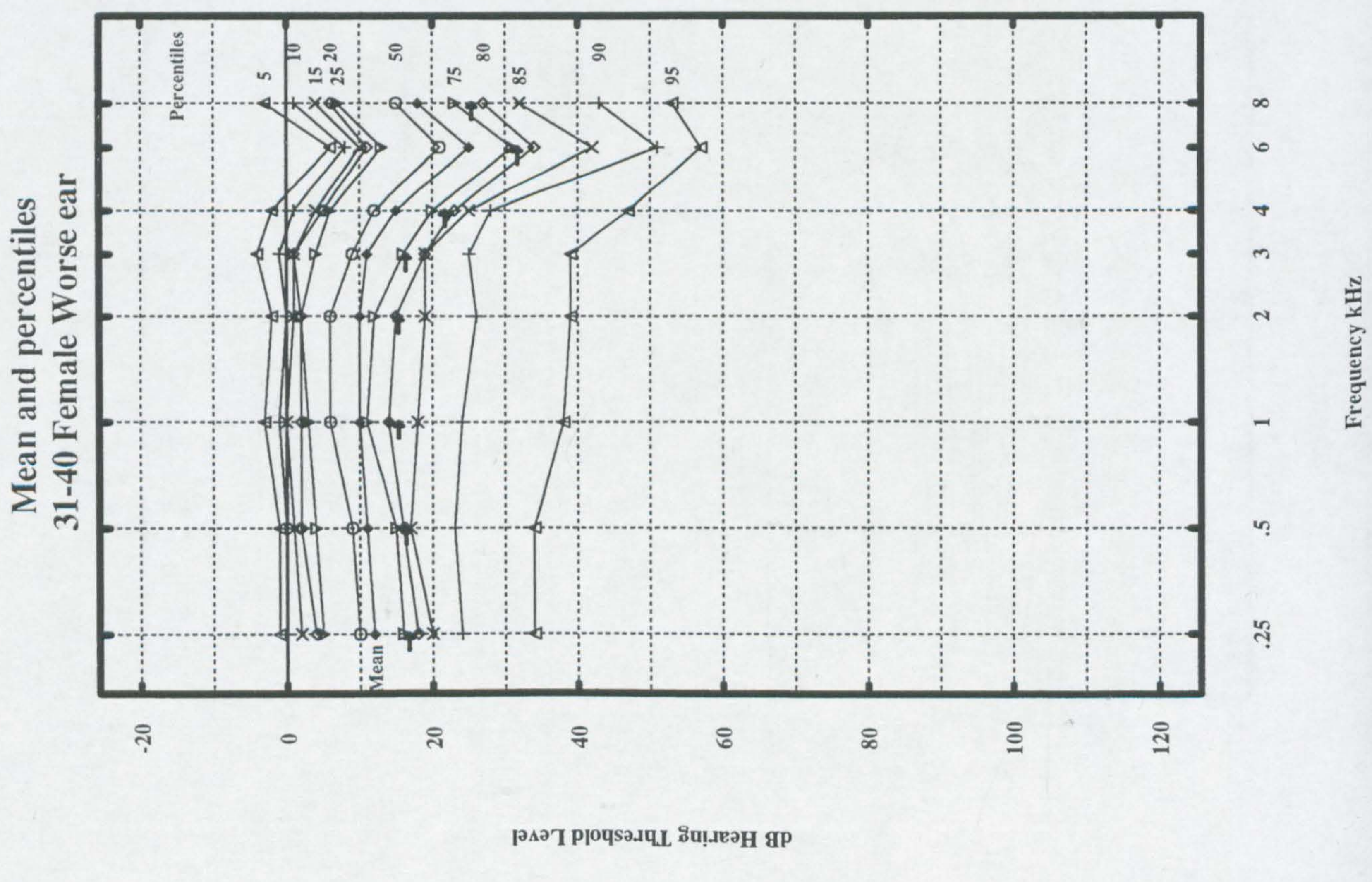
Mean and percentiles
31-40 Female Worse ear
Percentiles
5
10
15
20
25
50
75
80
85
90
95
Mean
-20
0
20
40
60
80
100
120
dB Hearing Threshold Level
.25
.5
1
2
3
4
6
8
Frequency kHz

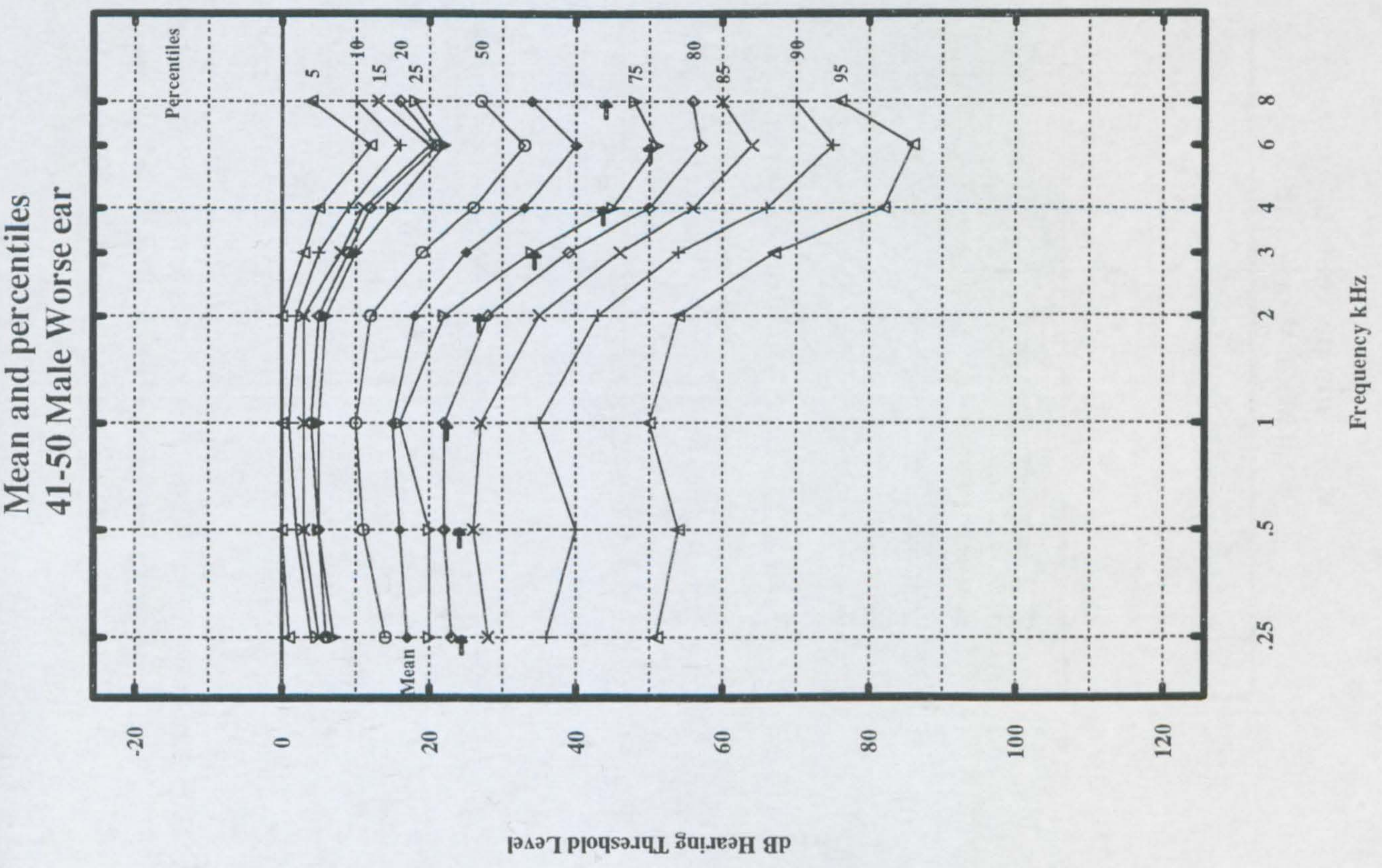
Mean and percentiles
41-50 Male Worse ear
Percentiles
5
10
15
20
25
50
75
80
85
90
95
Mean
dB Hearing Threshold Level
-20
0
20
40
60
80
100
120
.25
.5
1
2
3
4
6
8
Frequency kHz

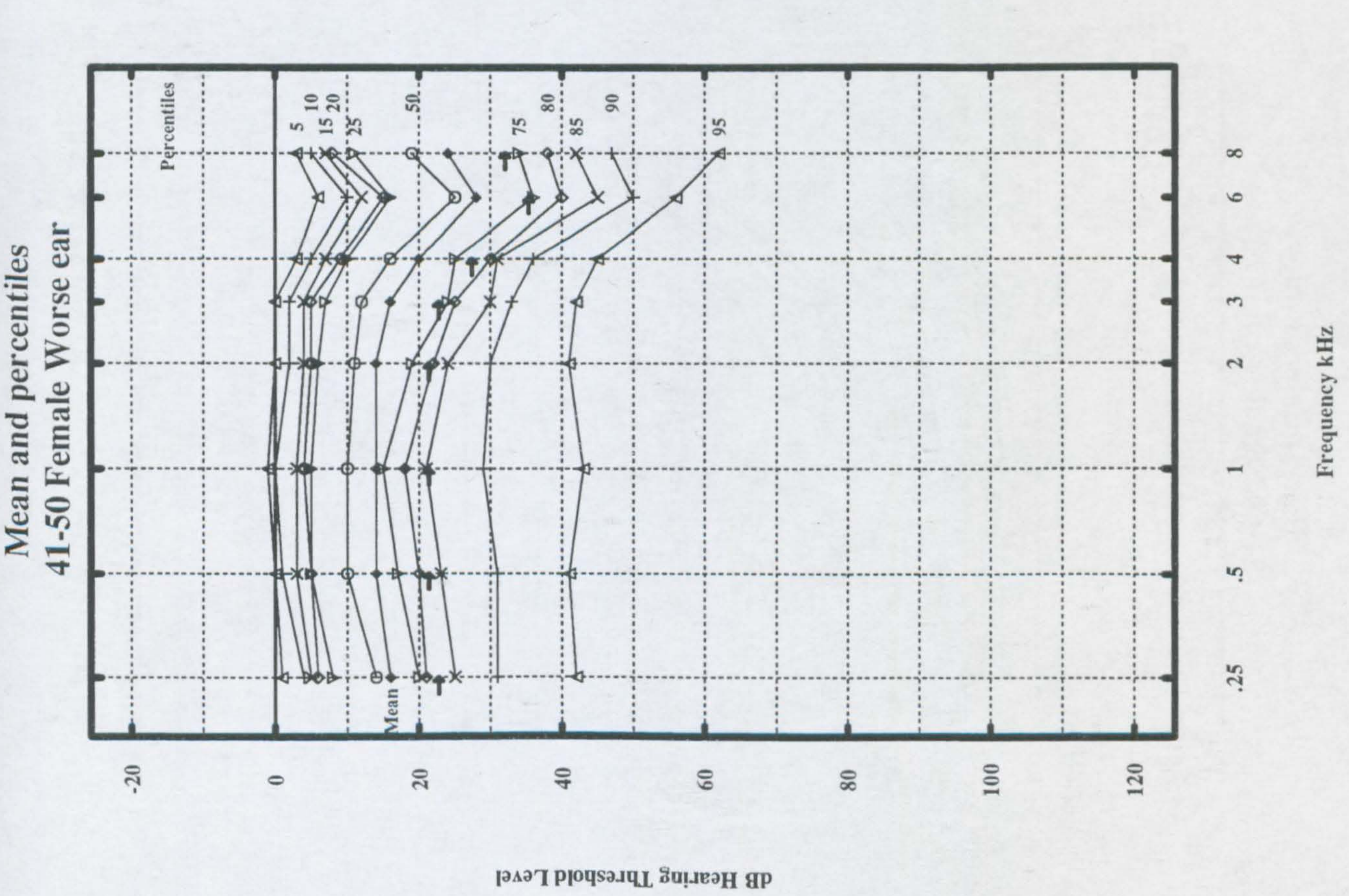
Mean and percentiles
41-50 Female Worse ear
Percentiles
5
10
15
20
25
50
75
80
85
90
95
Mean
dB Hearing Threshold Level
-20
0
20
40
60
80
100
120
.25
.5
1
2
3
4
6
8
Frequency kHz

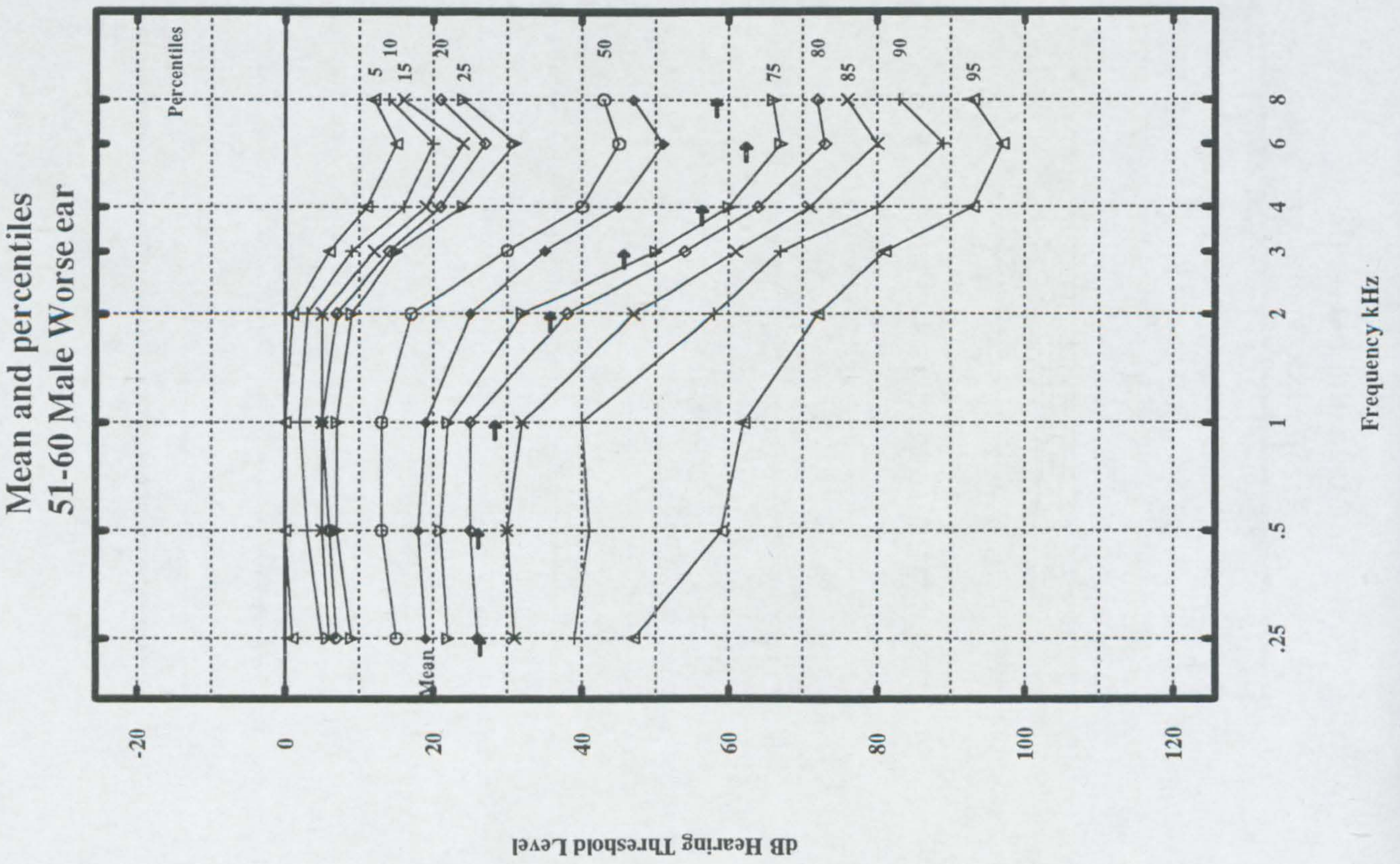
Mean and percentiles
51-60 Male Worse ear
Percentiles
5
10
15
20
25
50
75
80
85
90
95
Mean
-20
0
20
40
60
80
100
120
dB Hearing Threshold Level
.25
.5
1
2
3
4
6
8
Frequency kHz

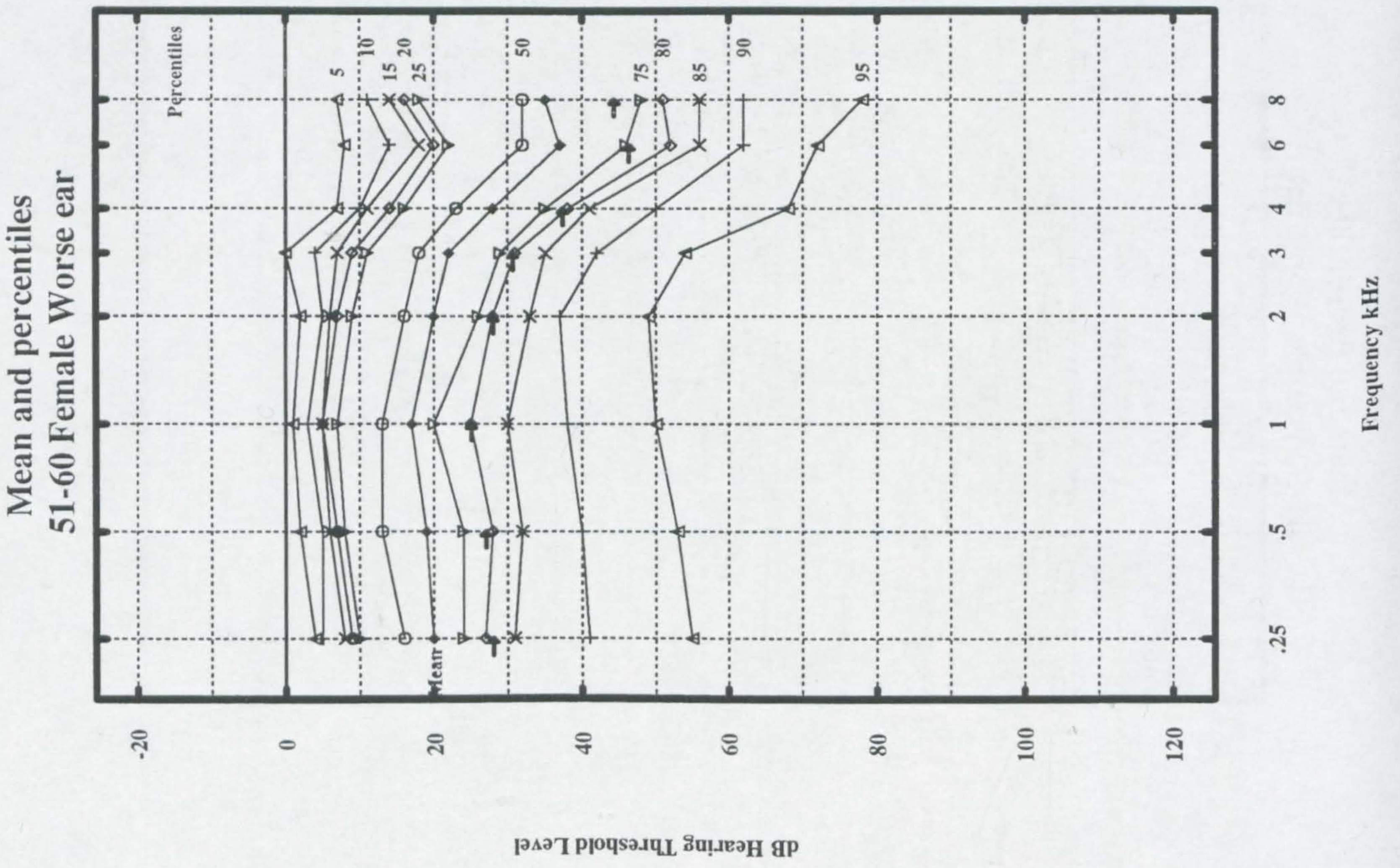
Mean and percentiles
51-60 Female Worse ear
Percentiles
5
10
15
20
25
50
75
80
85
90
95
Mean
-20
0
20
40
60
80
100
120
dB Hearing Threshold Level
.25
.5
1
2
3
4
6
8
Frequency kHz

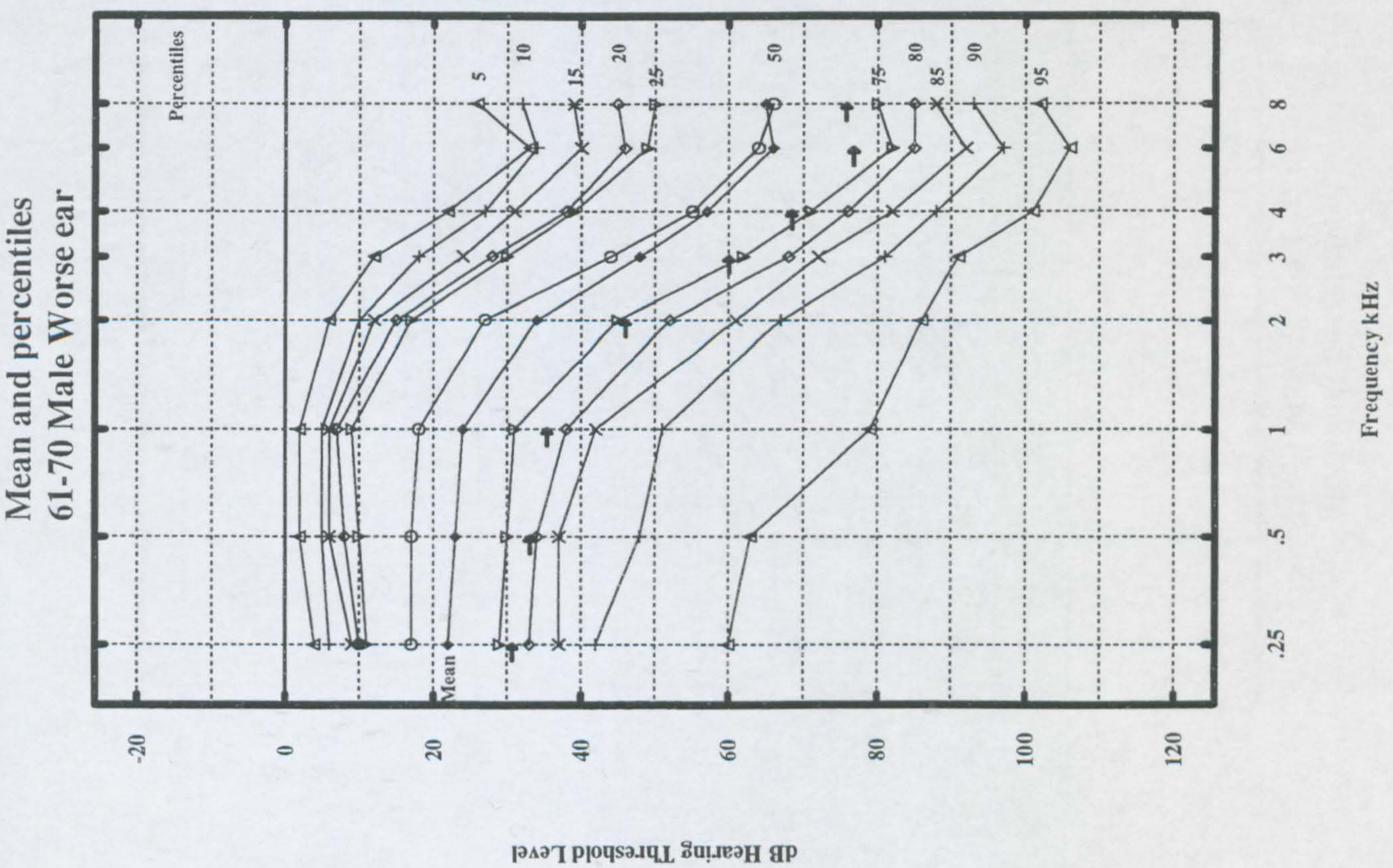
Mean and percentiles
61-70 Male Worse ear
Percentiles
5
10
15
20
25
50
75
80
85
90
95
Mean
-20
0
20
40
60
80
100
120
dB Hearing Threshold Level
.25
.5
1
2
3
4
6
8
Frequency kHz

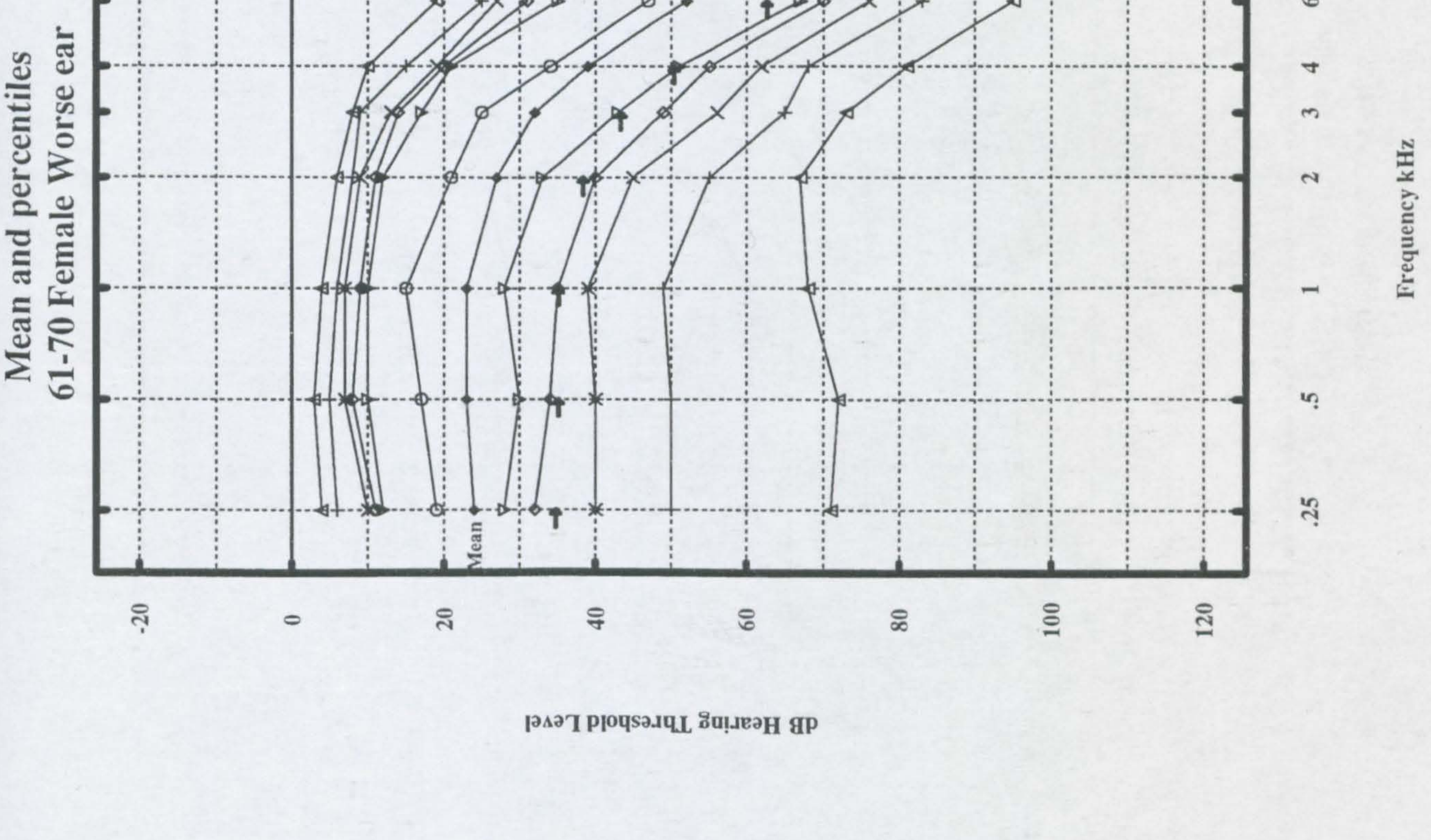
Mean and percentiles
61-70 Female Worse ear
Percentiles
5
10
15
20
25
50
75
80
85
90
95
Mean
-20
0
20
40
60
80
100
120
dB Hearing Threshold Level
.25
.5
1
2
3
4
6
8
Frequency kHz

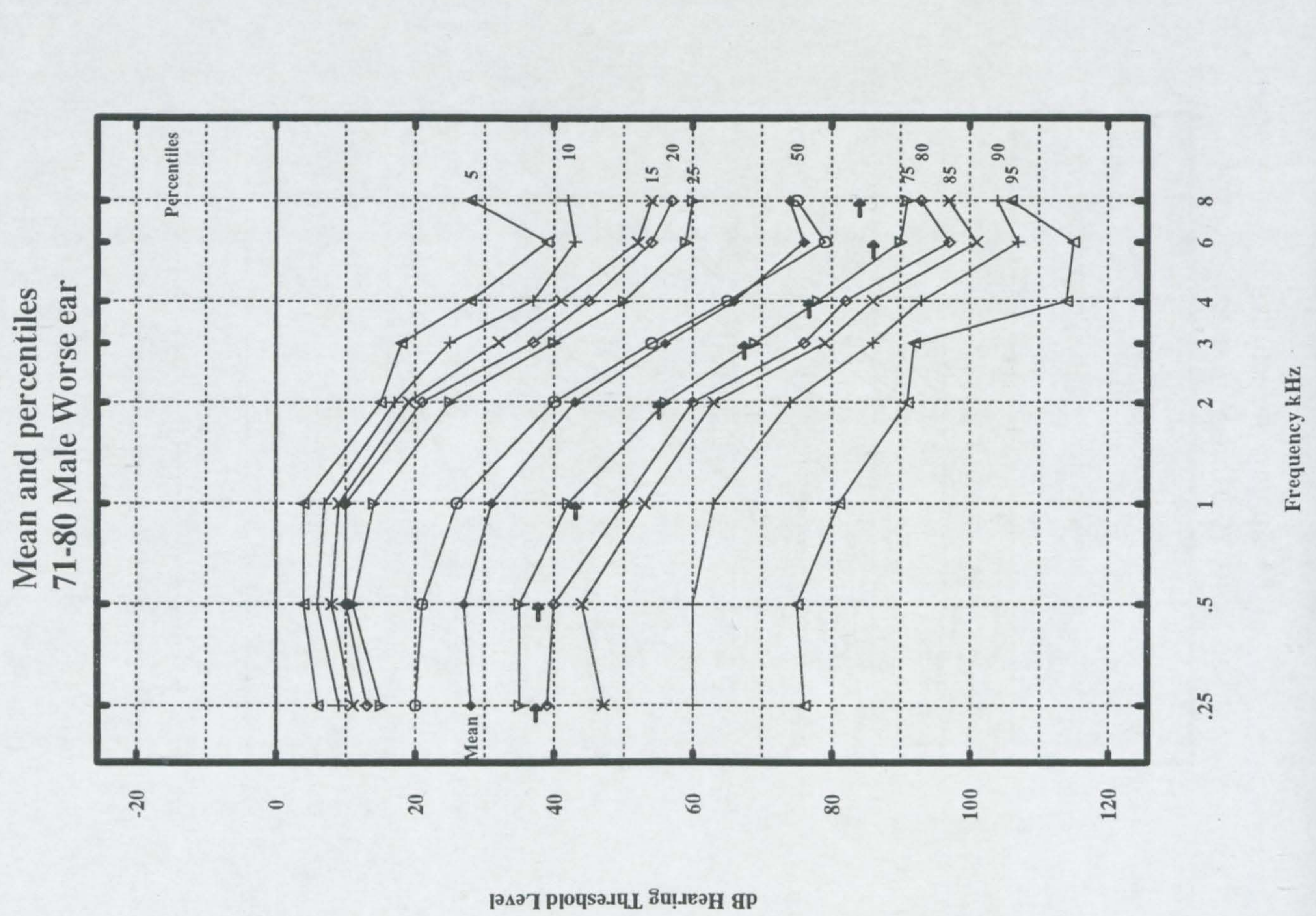
Mean and percentiles
71-80 Male Worse ear
dB Hearing Threshold Level
Frequency kHz
Percentiles
Mean
5
10
15
20
25
50
75
80
85
90
95
-20
0
20
40
60
80
100
120
.25
.5
1
2
3
4
6
8

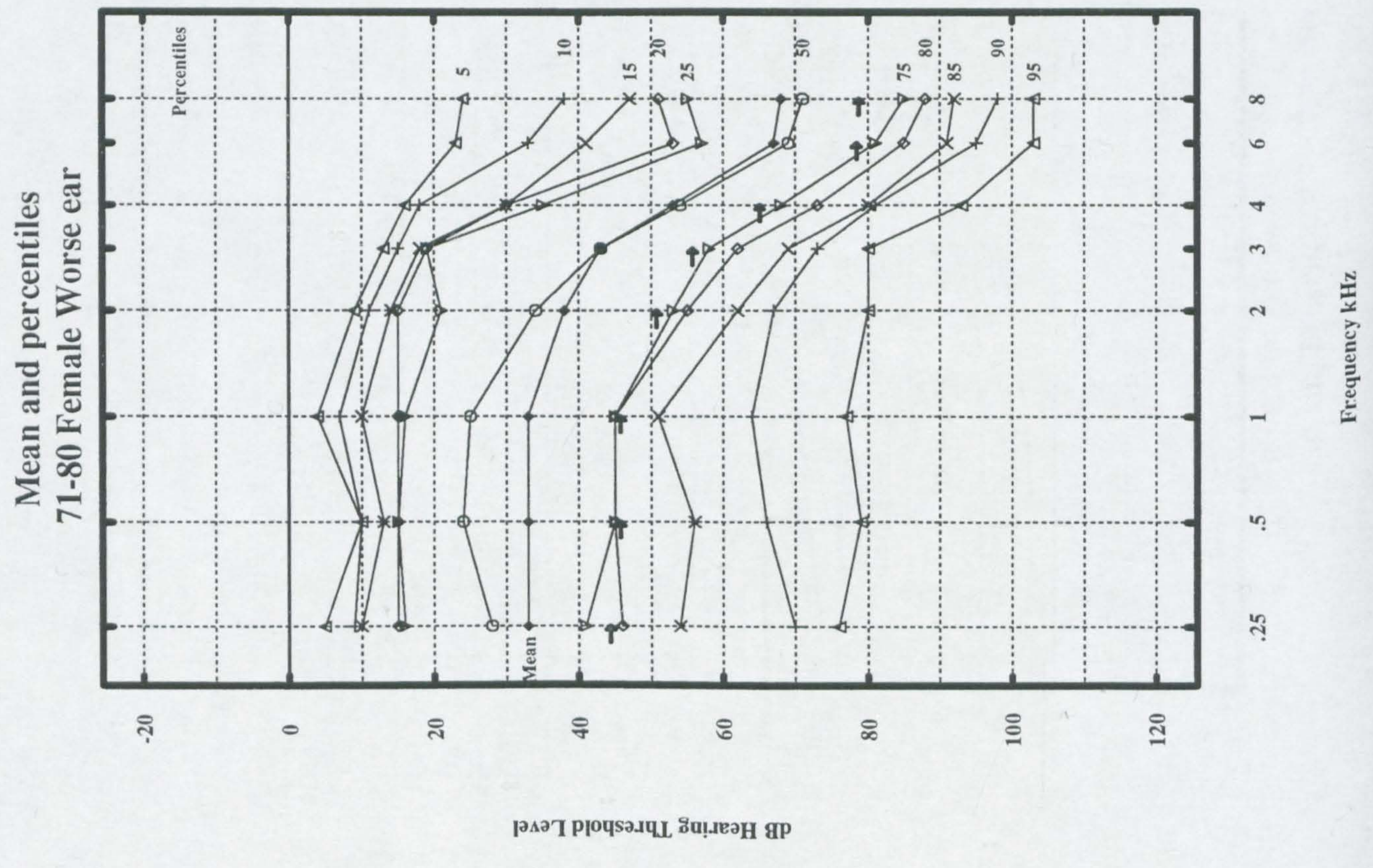
Mean and percentiles
71-80 Female Worse ear
dB Hearing Threshold Level
Frequency kHz
Percentiles
Mean
5
10
15
20
25
50
75
80
85
90
95
-20
0
20
40
60
80
100
120
.25
.5
1
2
3
4
6
8

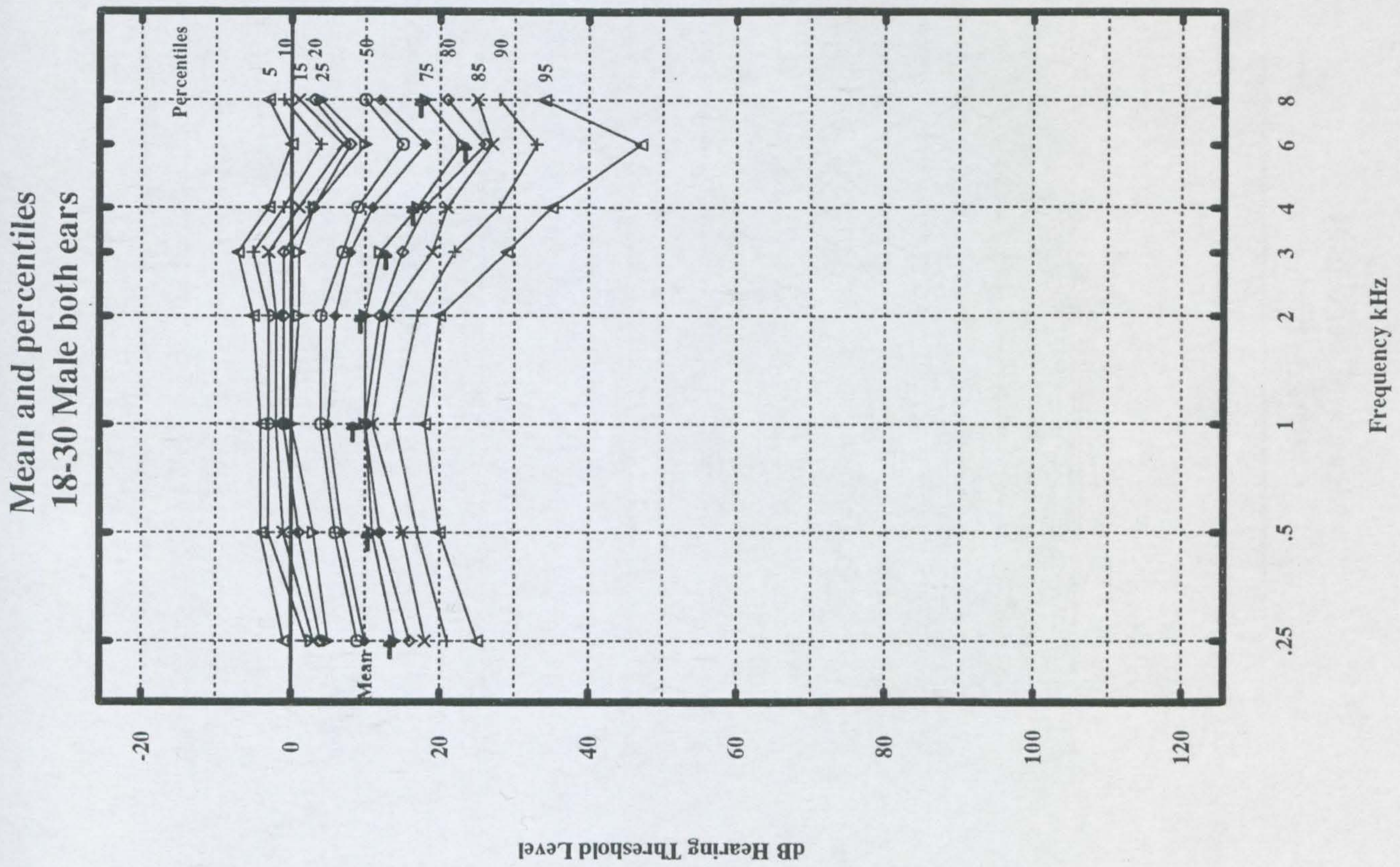
Mean and percentiles
18-30 Male both ears
Percentiles
5
10
15
25 20
50
75
80
85
90
95
Mean
dB Hearing Threshold Level
-20
0
20
40
60
80
100
120
Frequency kHz
.25
.5
1
2
3
4
6
8

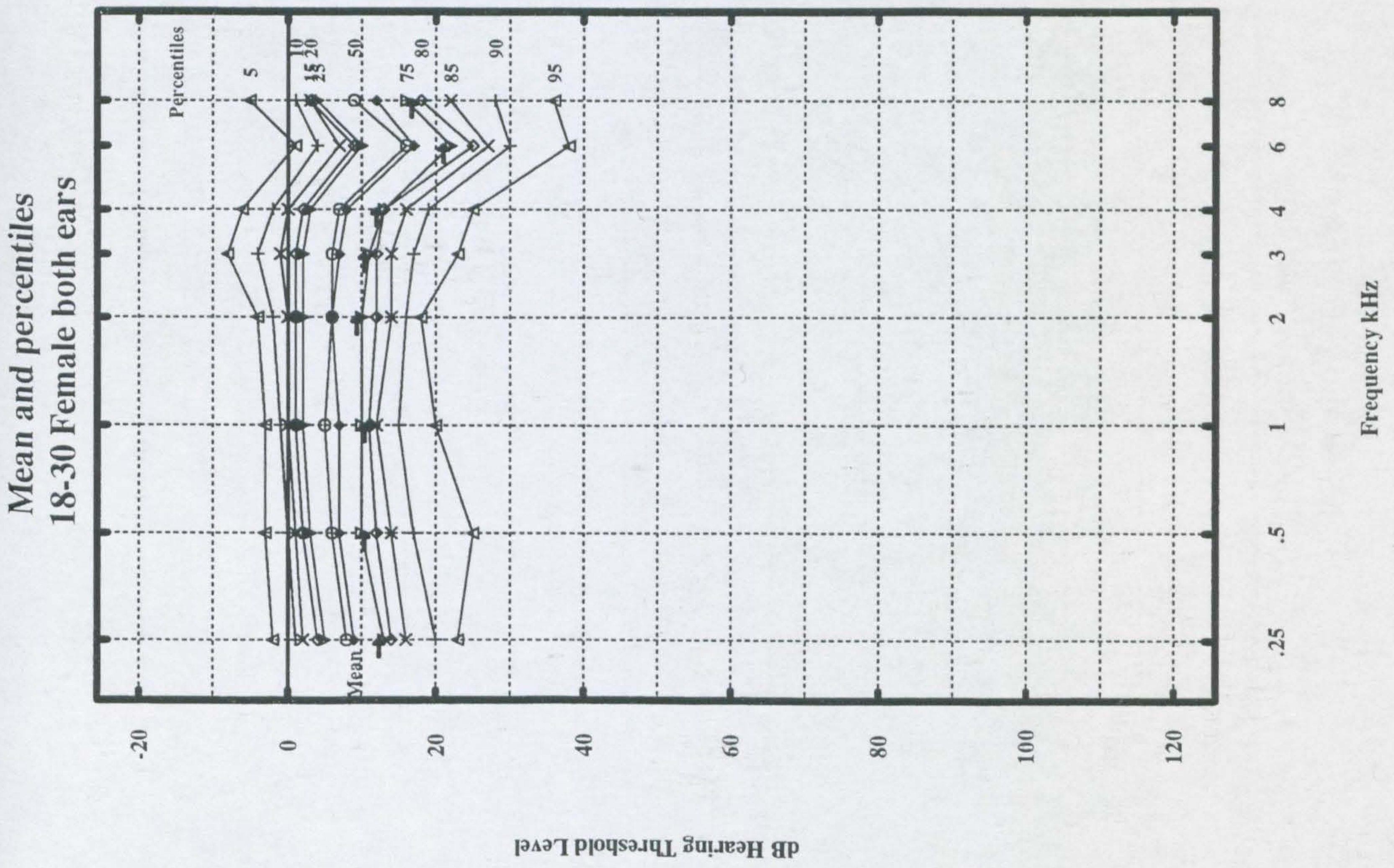
Mean and percentiles
18-30 Female both ears
Percentiles
5
10
15 20
25
50
75
80
85
90
95
Mean
dB Hearing Threshold Level
-20
0
20
40
60
80
100
120
Frequency kHz
.25
.5
1
2
3
4
6
8

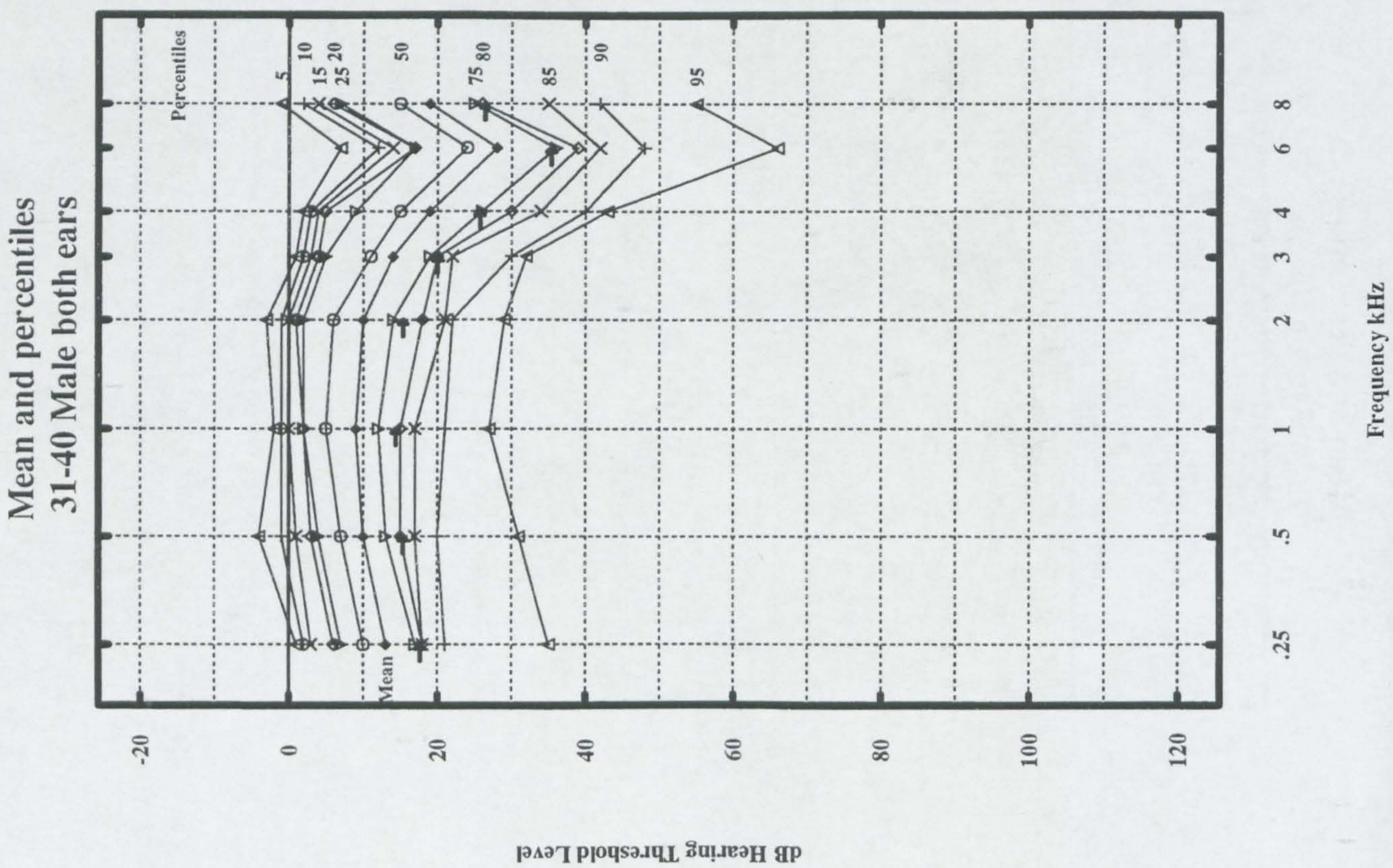
Mean and percentiles
31-40 Male both ears
Percentiles
5
10
15
25 20
50
75 80
85
90
95
Mean
-20
0
20
40
60
80
100
120
dB Hearing Threshold Level
.25
.5
1
2
3
4
6
8
Frequency kHz

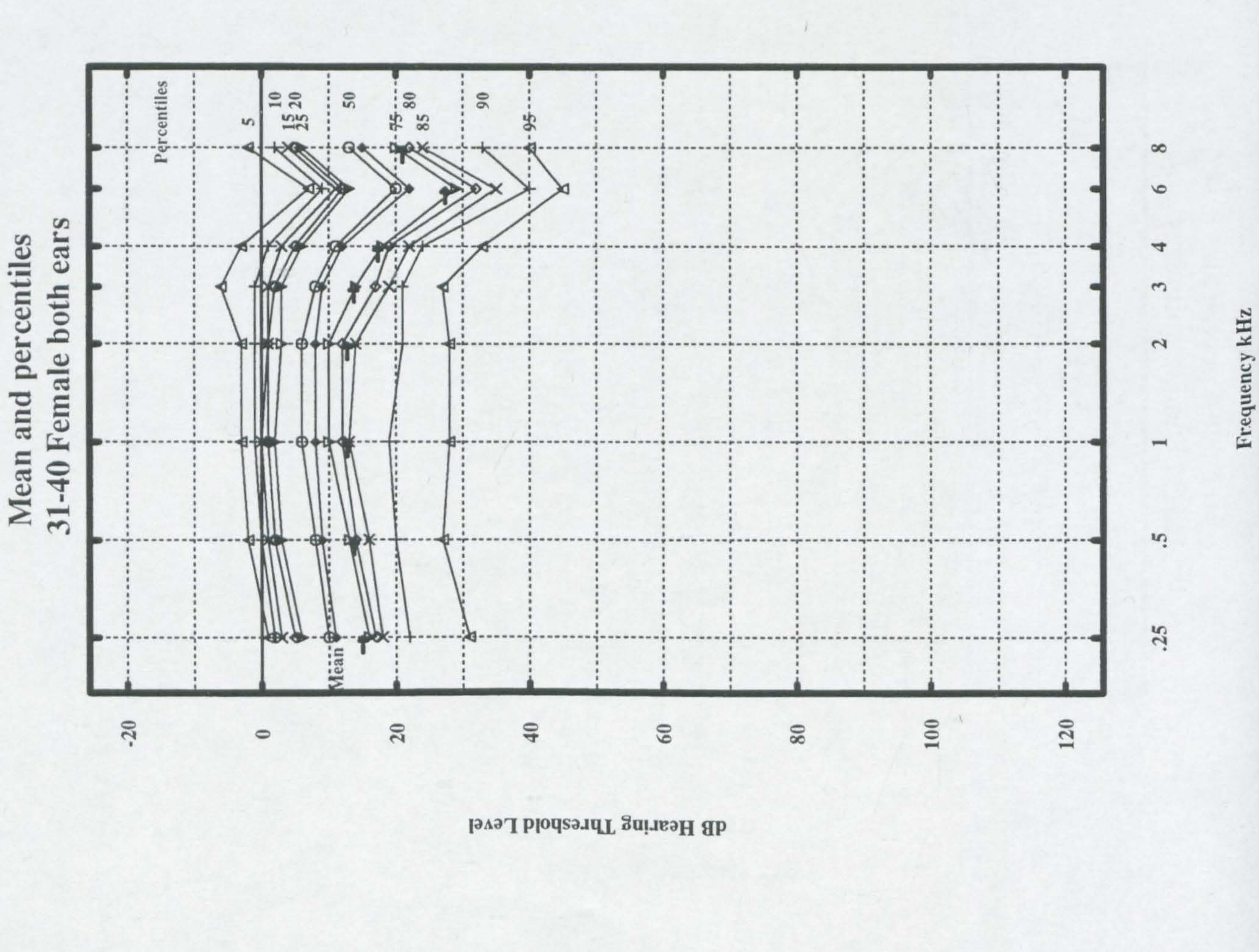
Mean and percentiles
31-40 Female both ears
Percentiles
5
10
15 20
25
50
75 80
85
90
95
Mean
-20
0
20
40
60
80
100
120
dB Hearing Threshold Level
.25
.5
1
2
3
4
6
8
Frequency kHz

Mean and percentiles
41-50 Male both ears

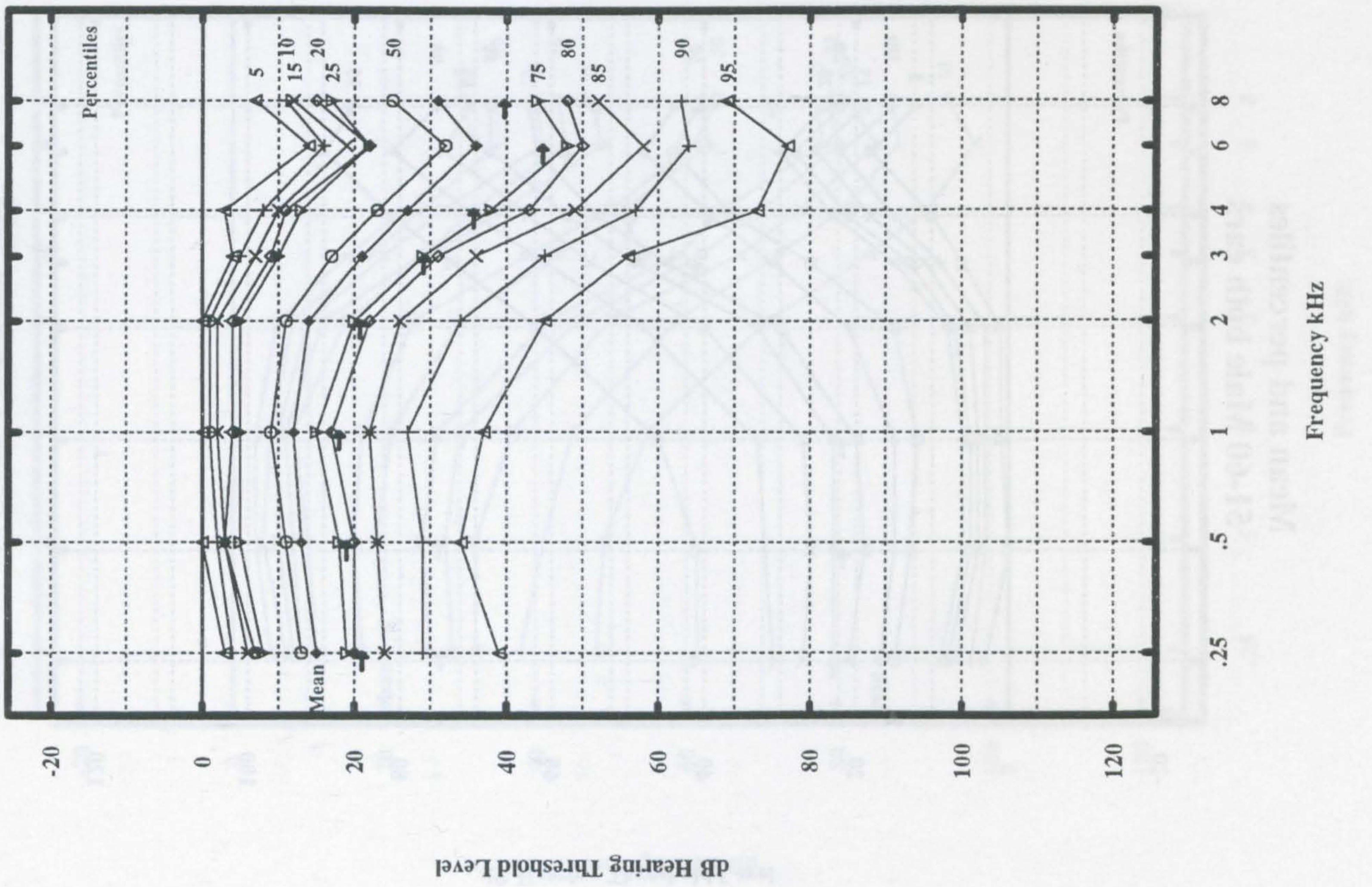

Mean and percentiles
41-50 Female both ears

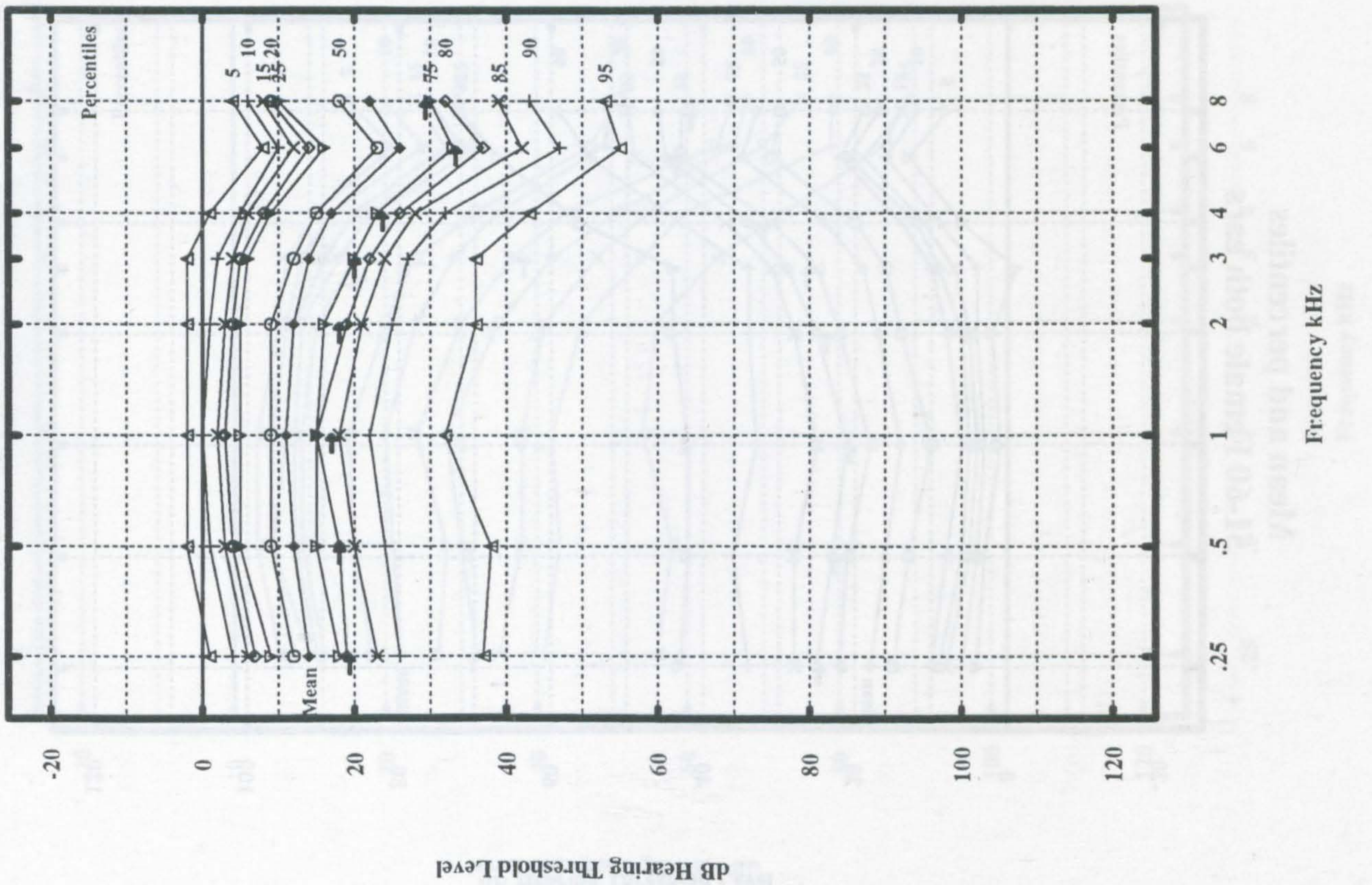

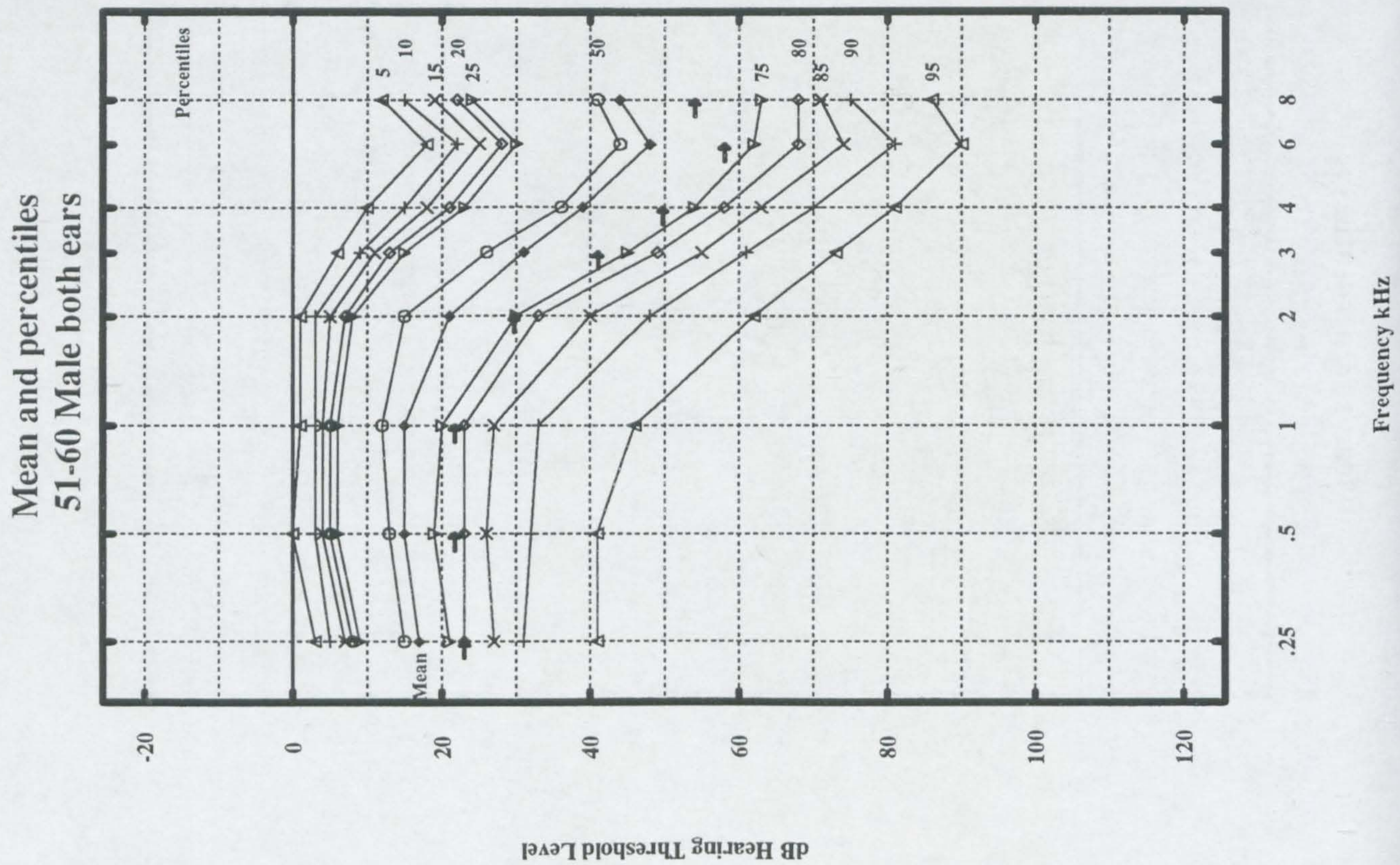
Mean and percentiles
51-60 Male both ears
Percentiles
5
10
15
20
25
50
75
80
85
90
95
Mean
dB Hearing Threshold Level
-20
0
20
40
60
80
100
120
Frequency kHz
.25
.5
1
2
3
4
6
8

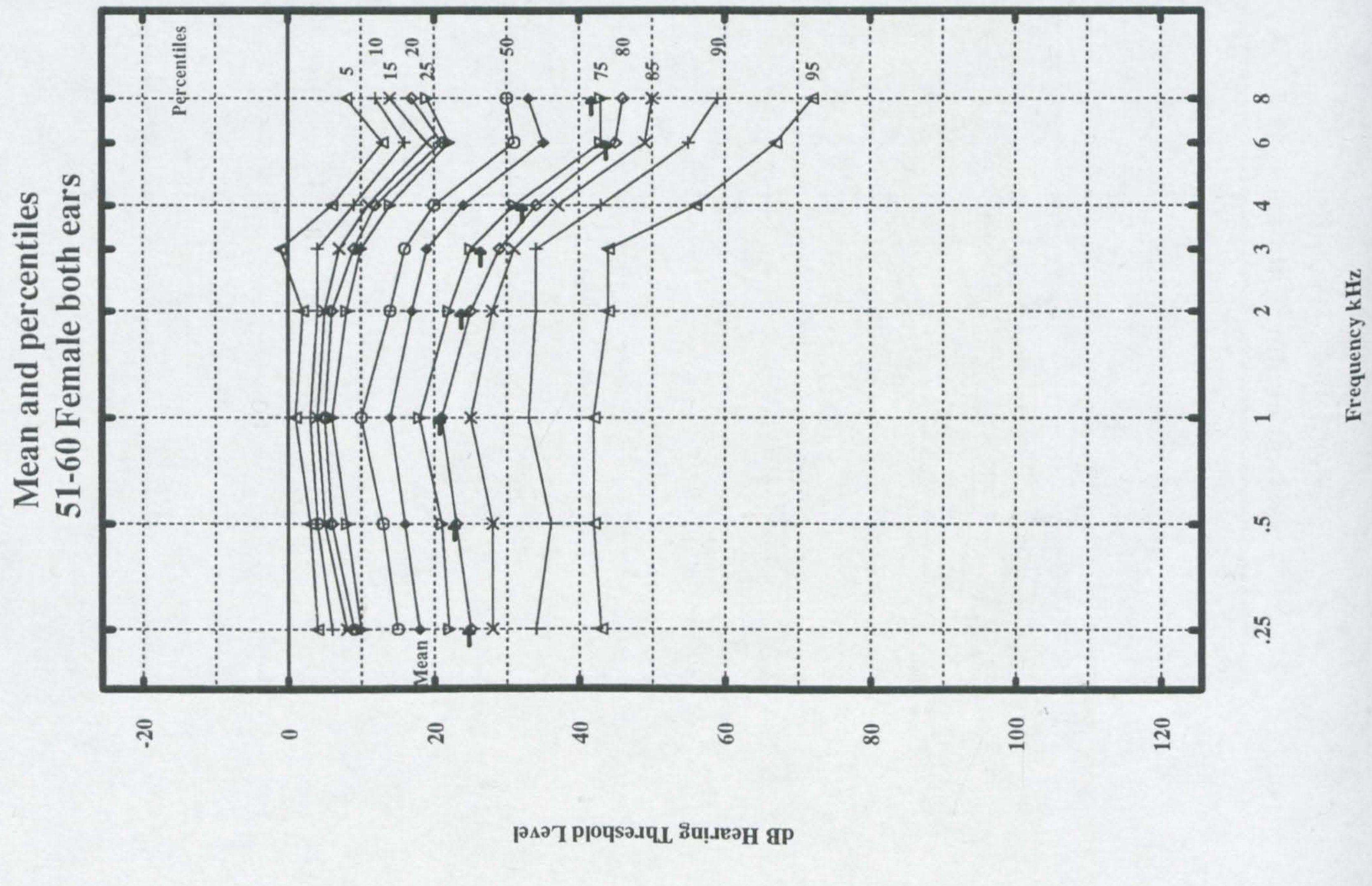
Mean and percentiles
51-60 Female both ears
Percentiles
5
10
15
20
25
50
75
80
85
90
95
Mean
dB Hearing Threshold Level
-20
0
20
40
60
80
100
120
Frequency kHz
.25
.5
1
2
3
4
6
8

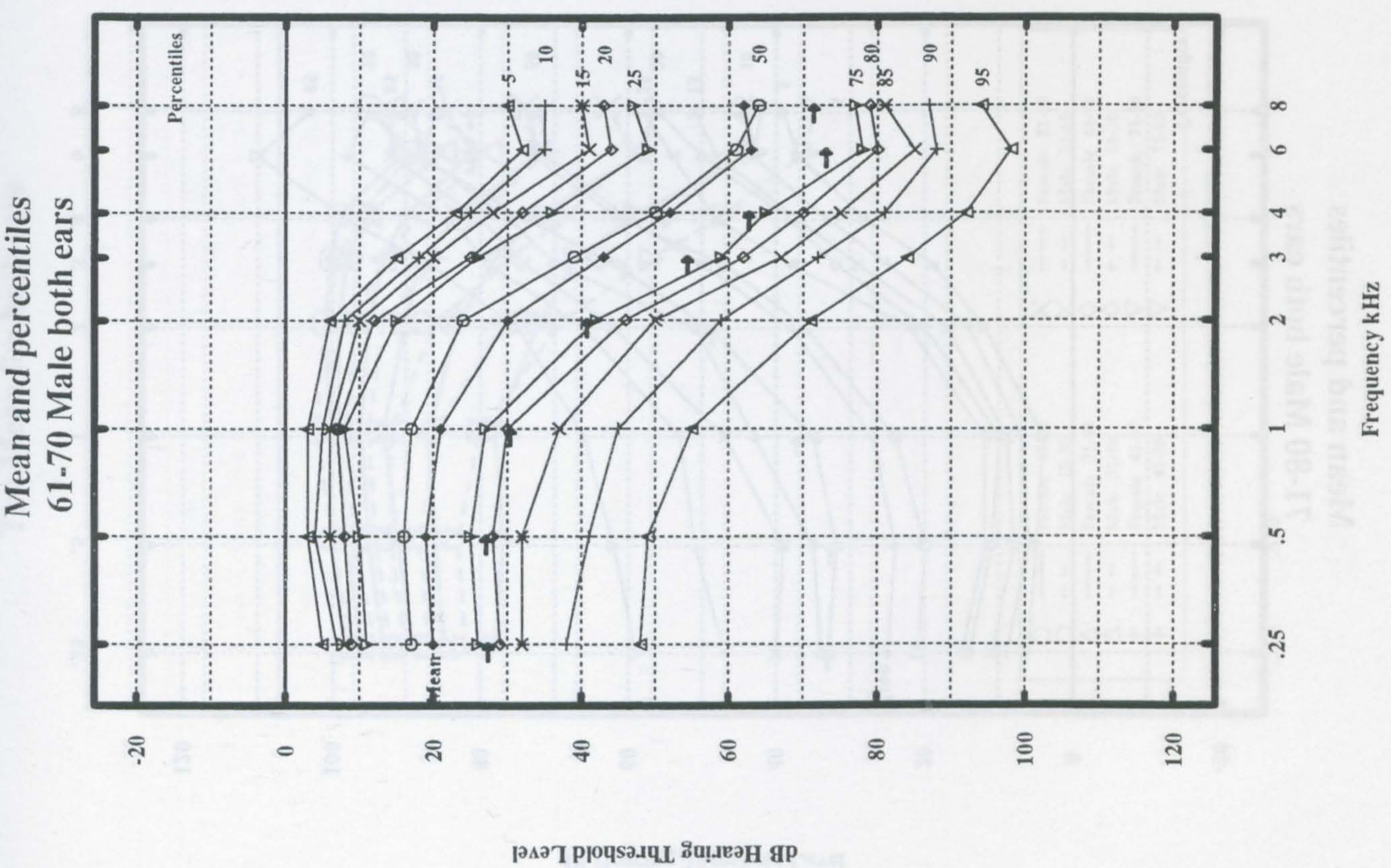
Mean and percentiles
61-70 Male both ears
Percentiles
5
10
15
20
25
50
75
80
85
90
95
Mean
-20
0
20
40
60
80
100
120
dB Hearing Threshold Level
.25
.5
1
2
3
4
6
8
Frequency kHz

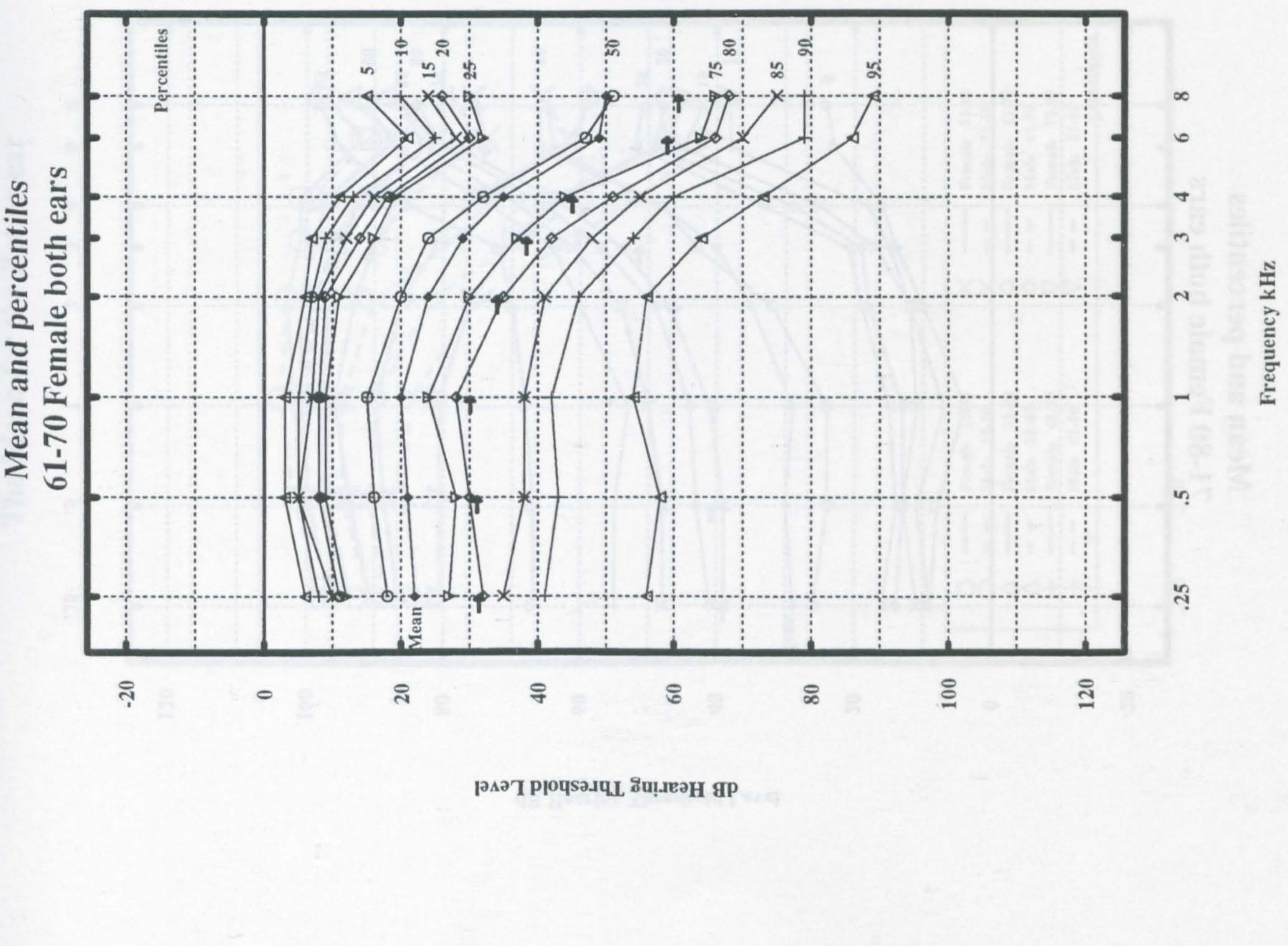
Mean and percentiles
61-70 Female both ears
Percentiles
5
10
15
20
25
50
75
80
85
90
95
Mean
-20
0
20
40
60
80
100
120
dB Hearing Threshold Level
.25
.5
1
2
3
4
6
8
Frequency kHz

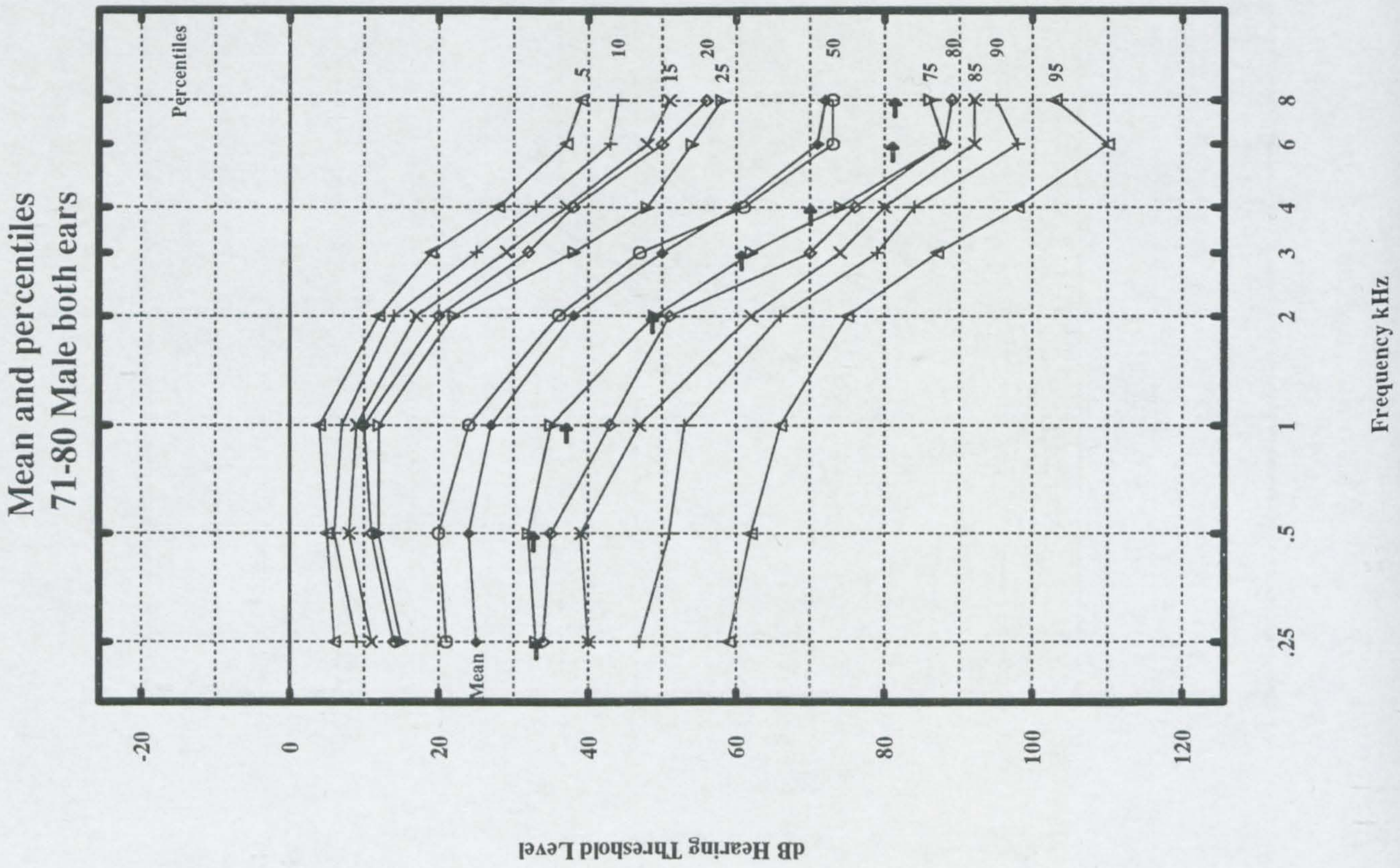
Mean and percentiles
71-80 Male both ears
Percentiles
5
10
15
20
25
50
75
80
85
90
95
Mean
-20
0
20
40
60
80
100
120
dB Hearing Threshold Level
.25
.5
1
2
3
4
6
8
Frequency kHz

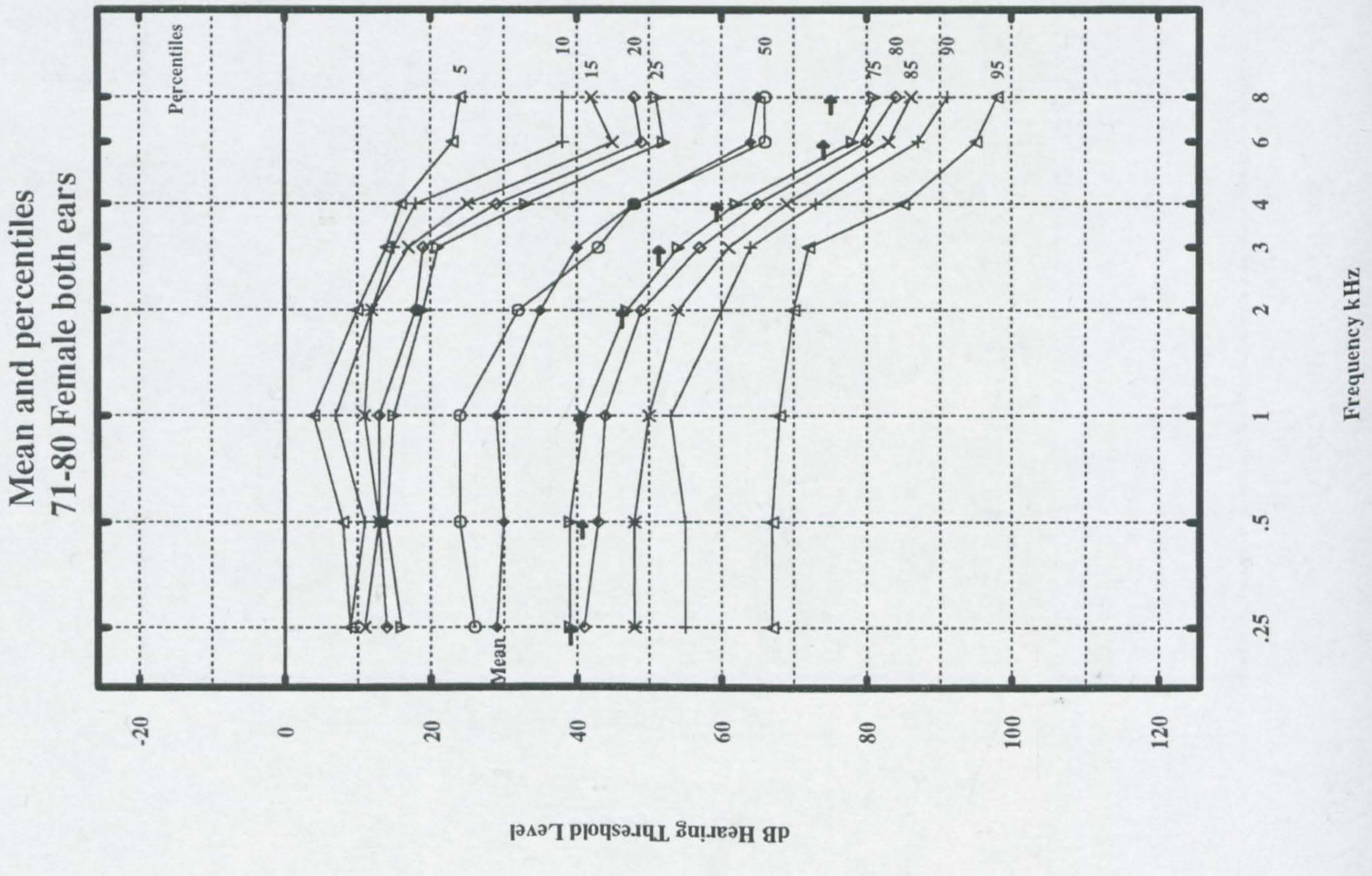
Mean and percentiles
71-80 Female both ears
Percentiles
5
10
15
20
25
50
75
80
85
90
95
Mean
-20
0
20
40
60
80
100
120
dB Hearing Threshold Level
.25
.5
1
2
3
4
6
8
Frequency kHz

The Mean for the Better ear

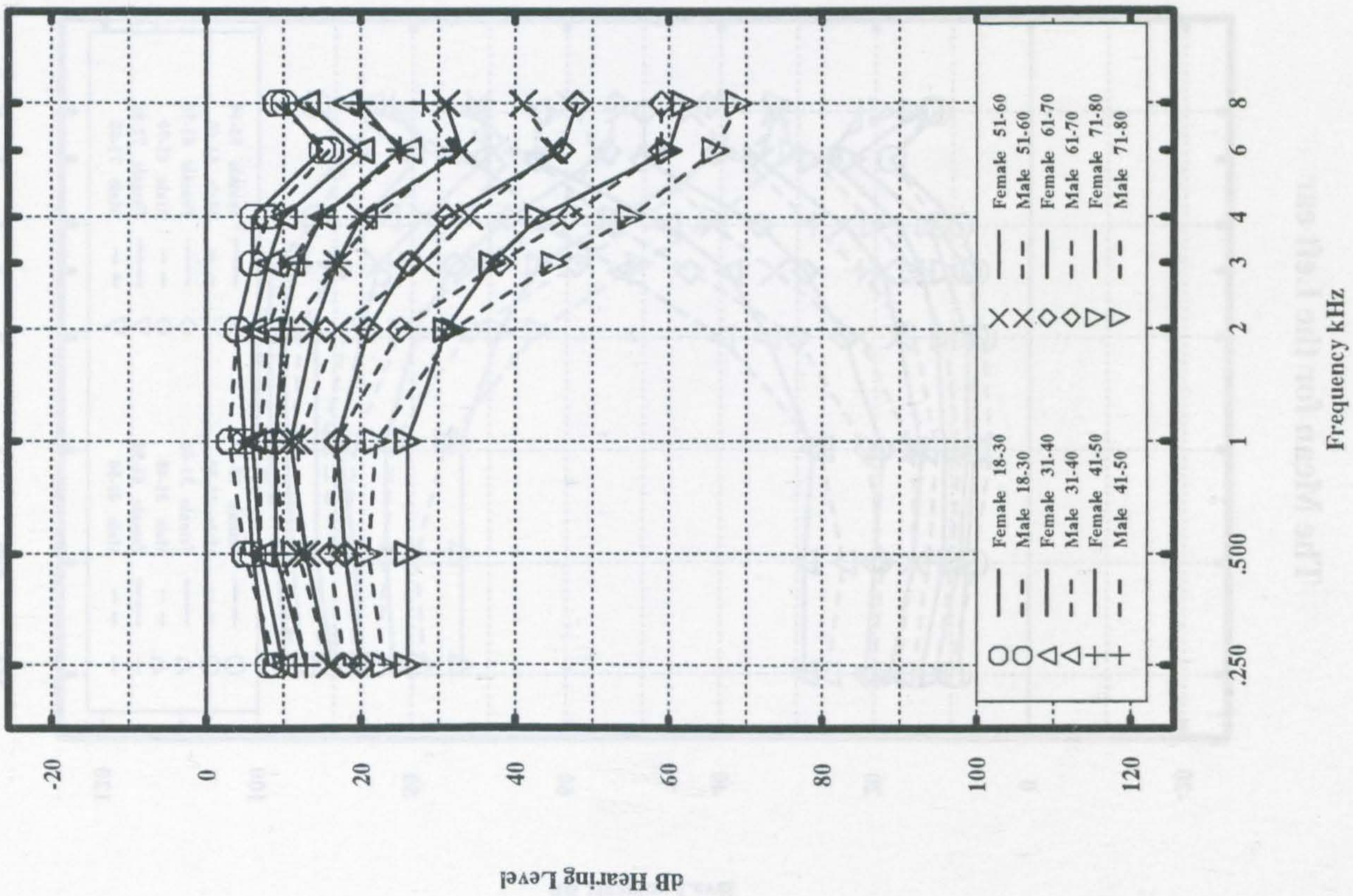

The Mean for both ears

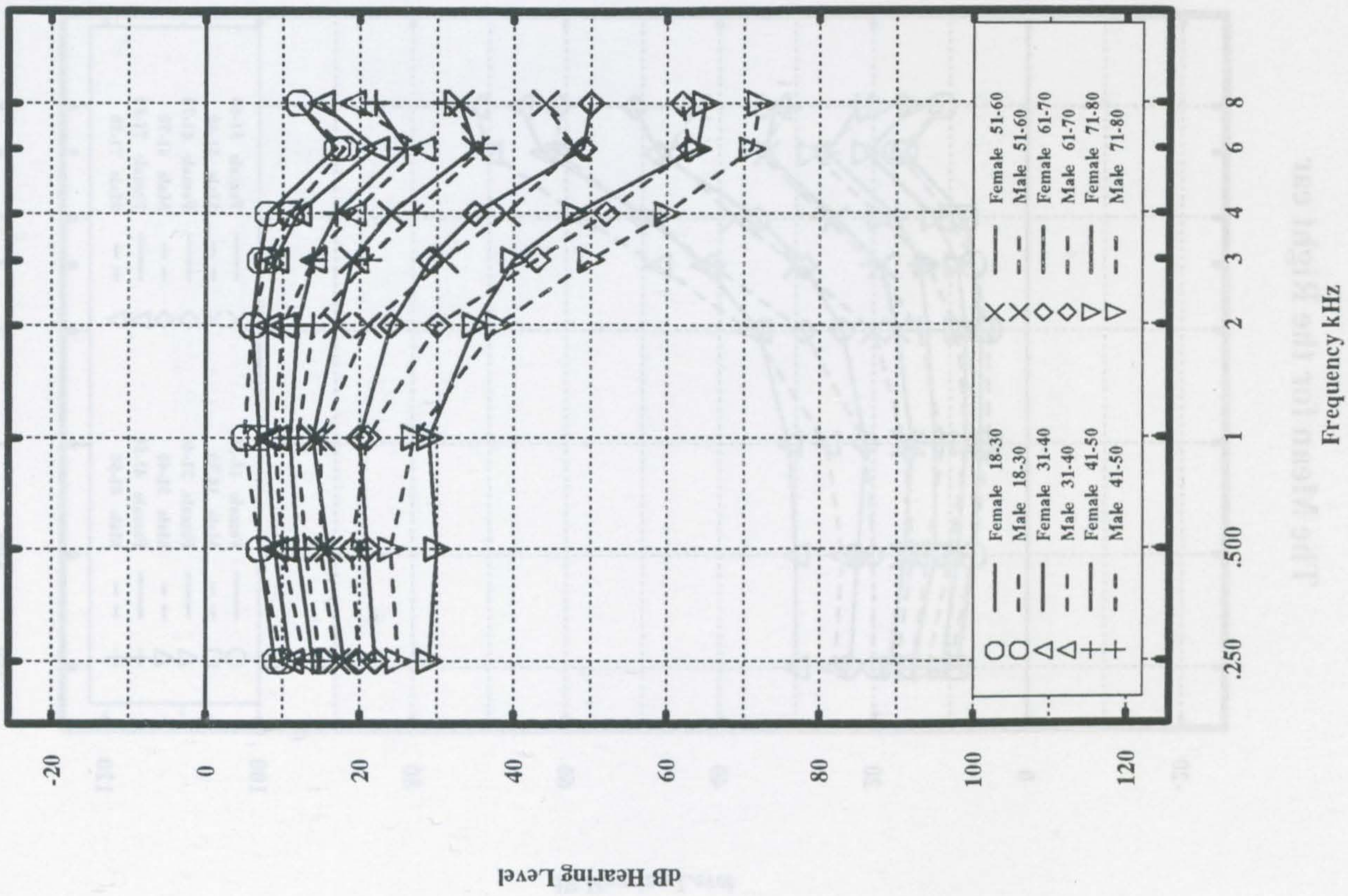

The Mean for the Right ear

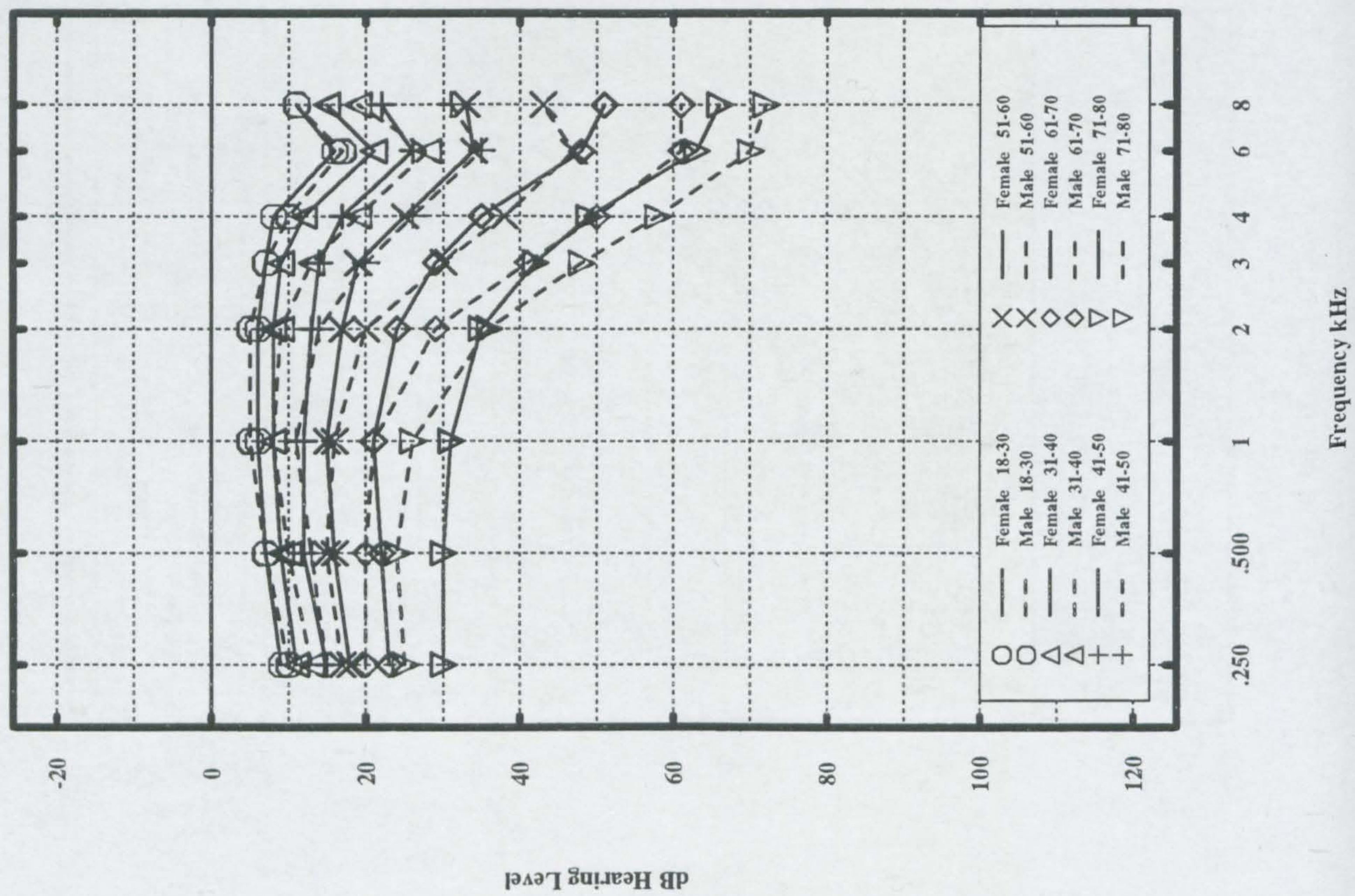

The Mean for the Left ear

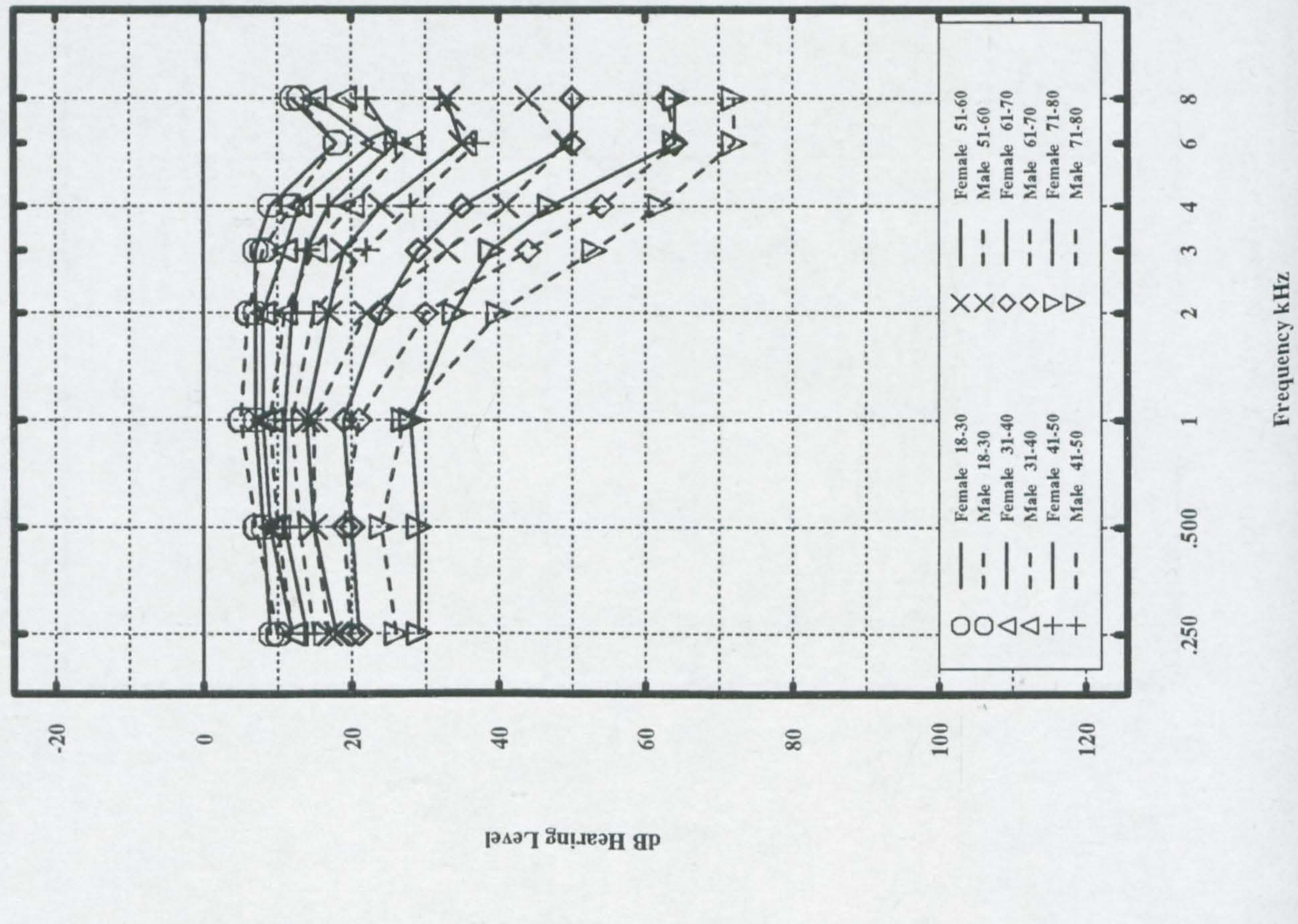

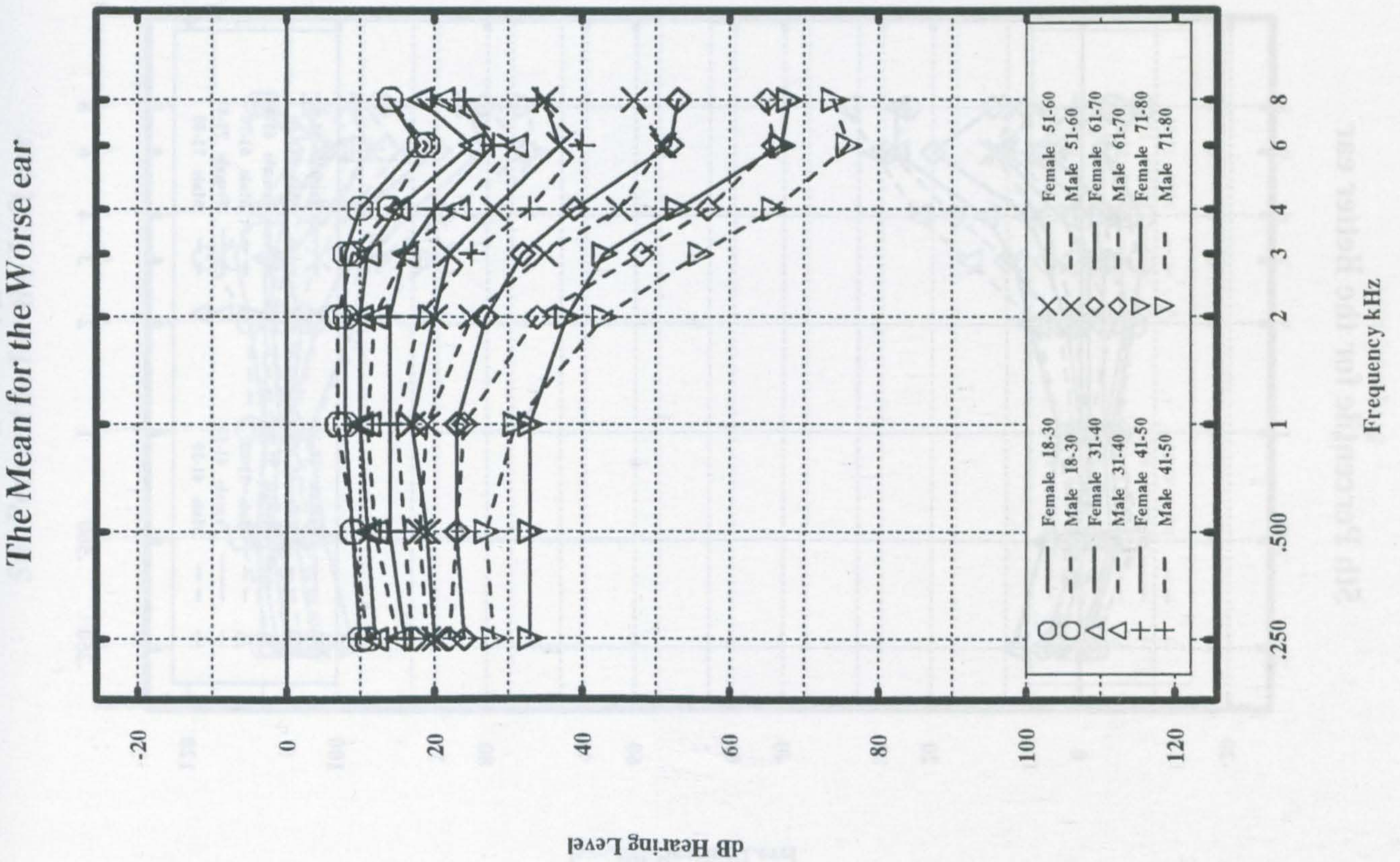
The Mean for the Worse ear
dB Hearing Level
-20
0
20
40
60
80
100
120
.250
.500
1
2
3
4
6
8
Frequency kHz
Female 18-30
Male 18-30
Female 31-40
Male 31-40
Female 41-50
Male 41-50
Female 51-60
Male 51-60
Female 61-70
Male 61-70
Female 71-80
Male 71-80

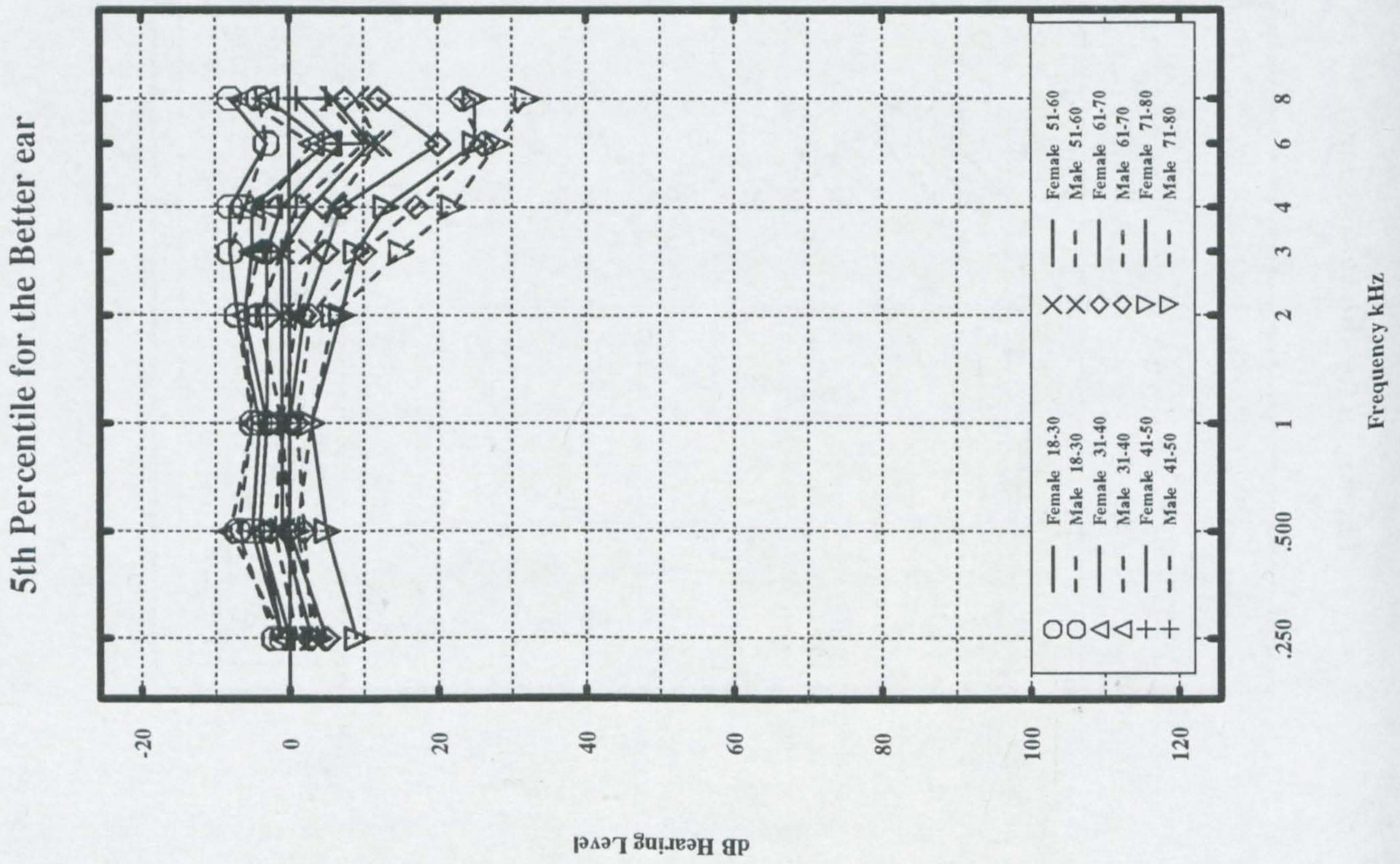
5th Percentile for the Better ear
dB Hearing Level
-20
0
20
40
60
80
100
120
Frequency kHz
.250
.500
1
2
3
4
6
8
Female 18-30
Male 18-30
Female 31-40
Male 31-40
Female 41-50
Male 41-50
Female 51-60
Male 51-60
Female 61-70
Male 61-70
Female 71-80
Male 71-80

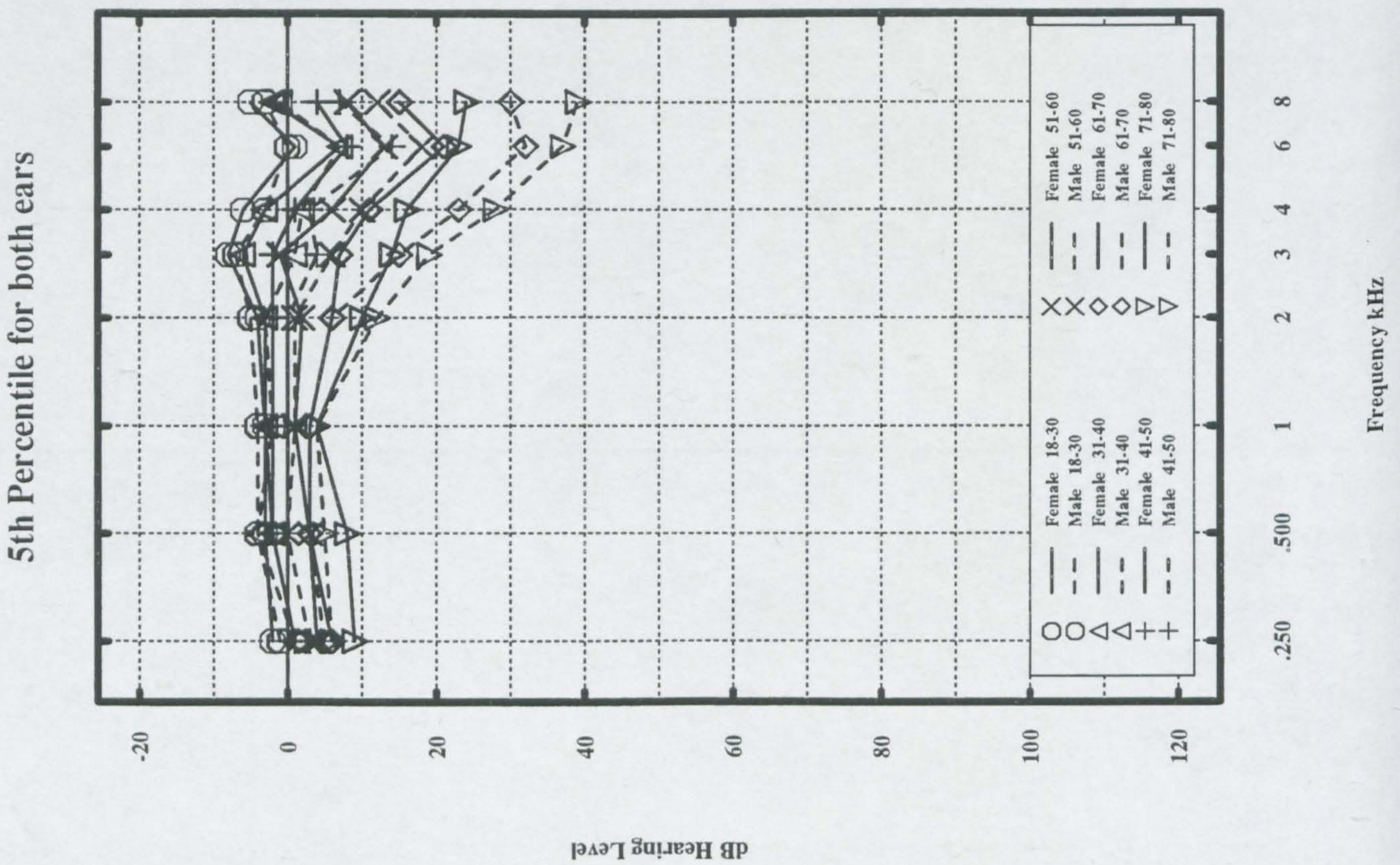
5th Percentile for both ears
dB Hearing Level
-20
0
20
40
60
80
100
120
Frequency kHz
.250
.500
1
2
3
4
6
8
Female 18-30
Male 18-30
Female 31-40
Male 31-40
Female 41-50
Male 41-50
Female 51-60
Male 51-60
Female 61-70
Male 61-70
Female 71-80
Male 71-80

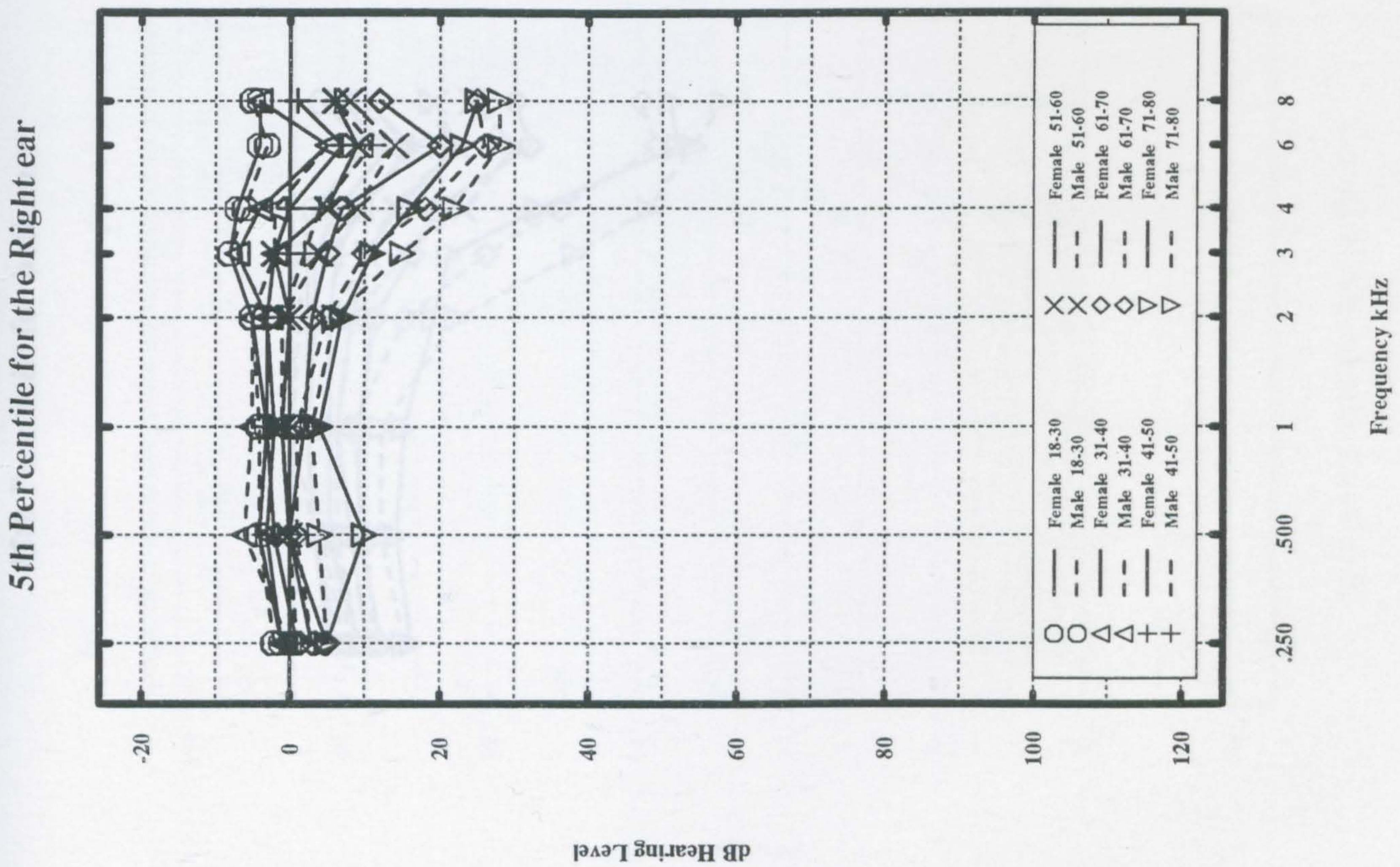
5th Percentile for the Right ear
dB Hearing Level
Frequency kHz
.250 .500 1 2 3 4 6 8
-20 0 20 40 60 80 100 120
Female 18-30
Male 18-30
Female 31-40
Male 31-40
Female 41-50
Male 41-50
Female 51-60
Male 51-60
Female 61-70
Male 61-70
Female 71-80
Male 71-80

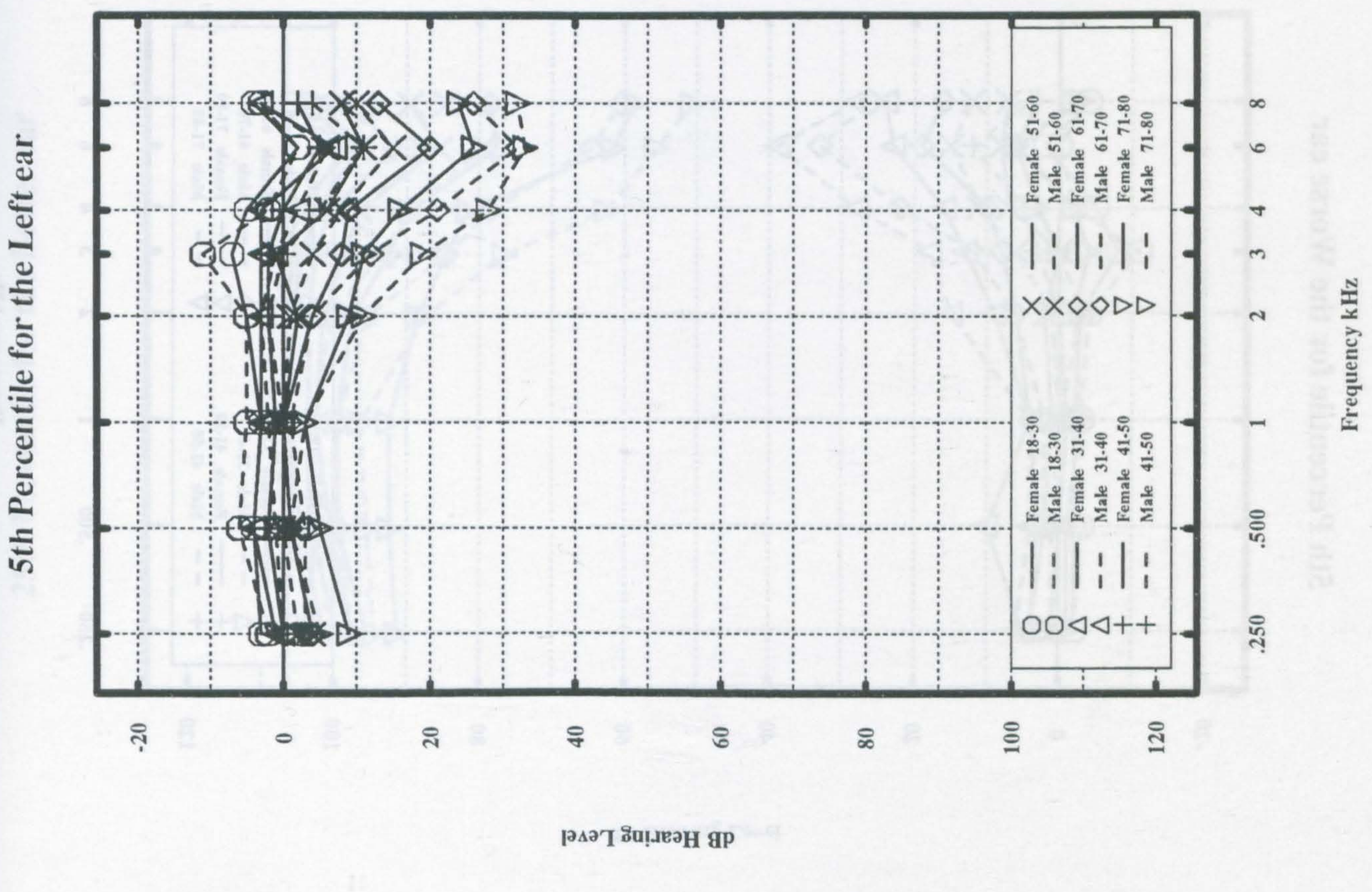
5th Percentile for the Left ear
dB Hearing Level
Frequency kHz
.250 .500 1 2 3 4 6 8
-20 0 20 40 60 80 100 120
Female 18-30
Male 18-30
Female 31-40
Male 31-40
Female 41-50
Male 41-50
Female 51-60
Male 51-60
Female 61-70
Male 61-70
Female 71-80
Male 71-80

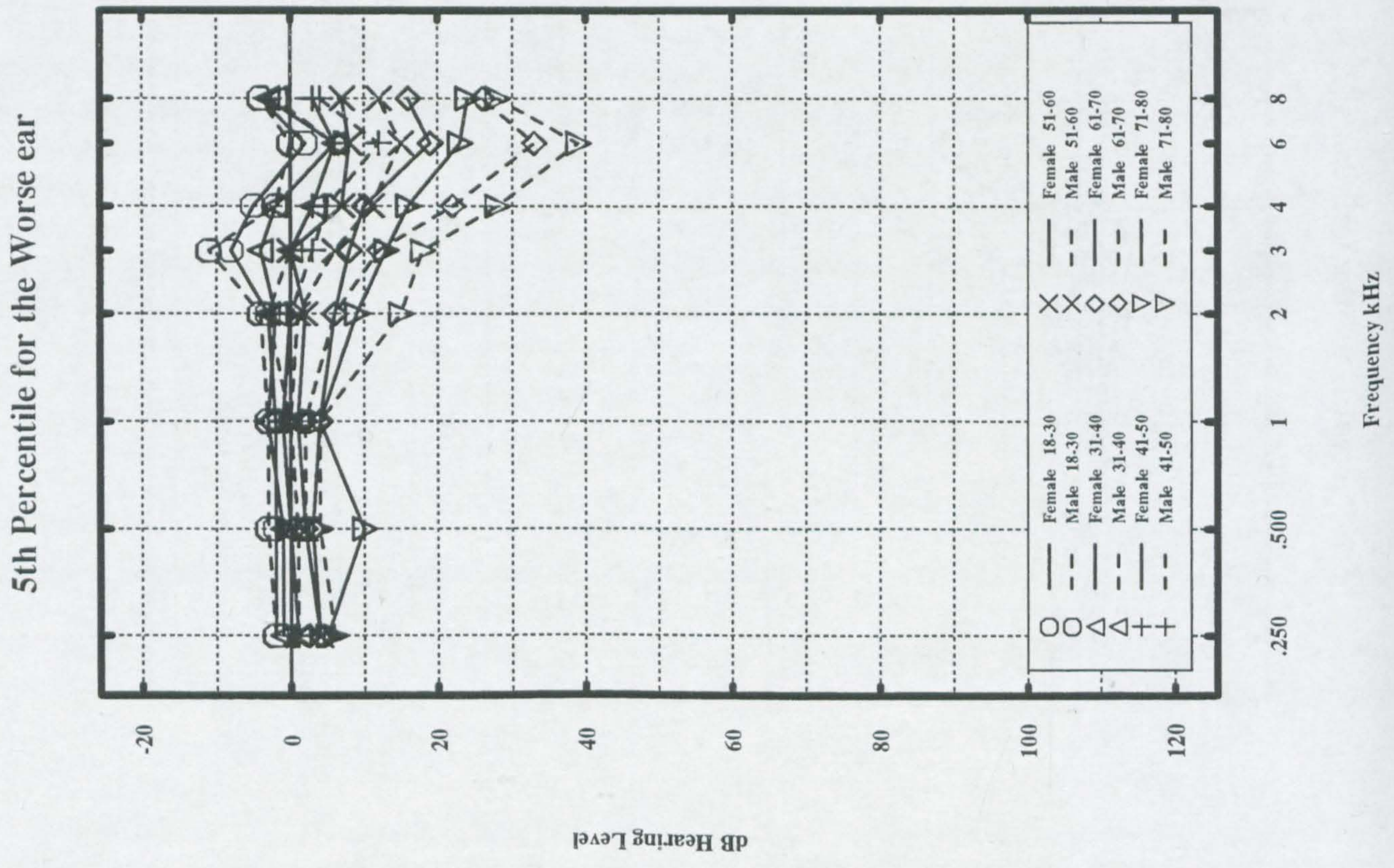
5th Percentile for the Worse ear
dB Hearing Level
-20
0
20
40
60
80
100
120
Frequency kHz
.250
.500
1
2
3
4
6
8
Female 18-30
Male 18-30
Female 31-40
Male 31-40
Female 41-50
Male 41-50
Female 51-60
Male 51-60
Female 61-70
Male 61-70
Female 71-80
Male 71-80

25th Percentile for the Better ear

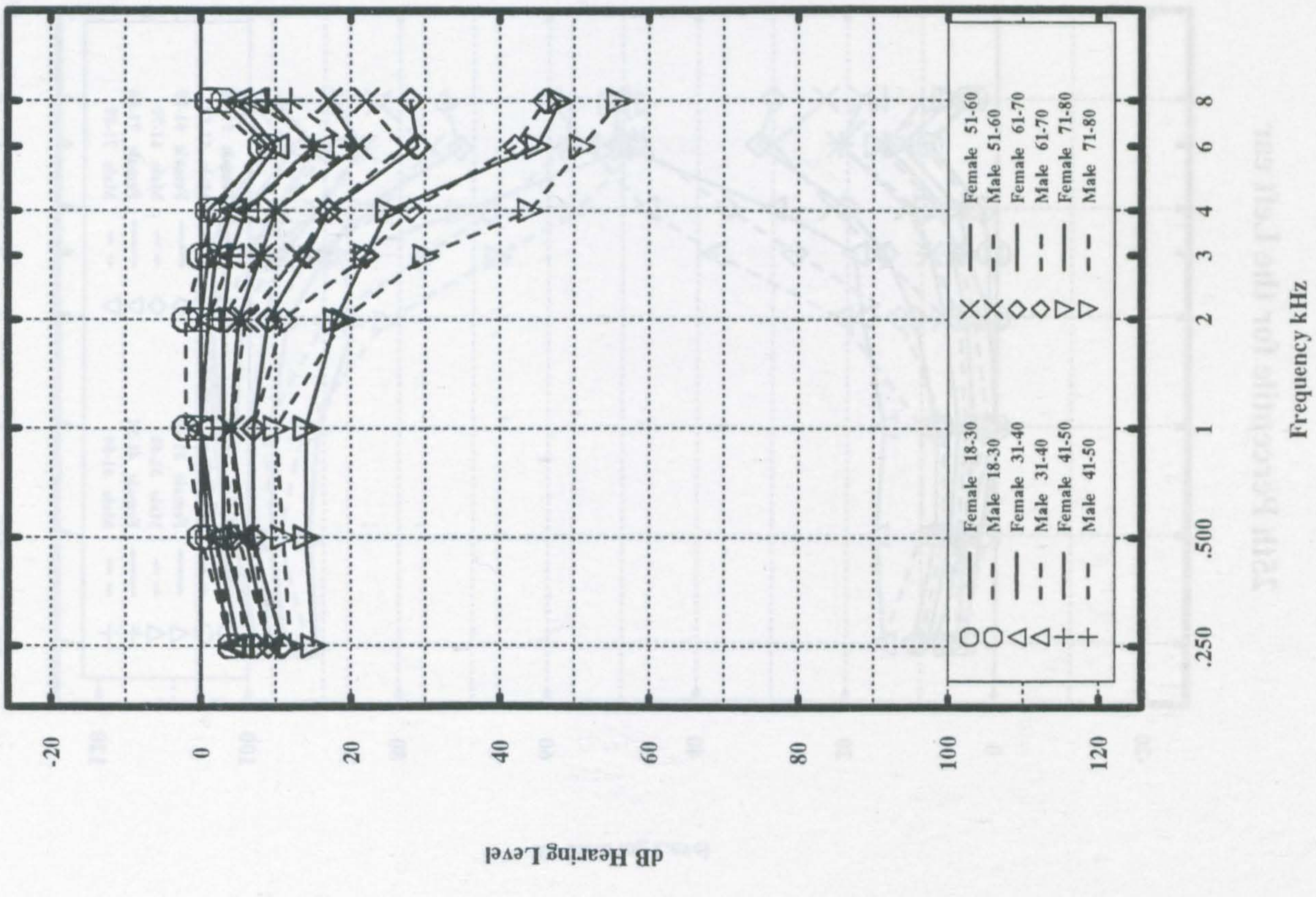

25th Percentile for both ears

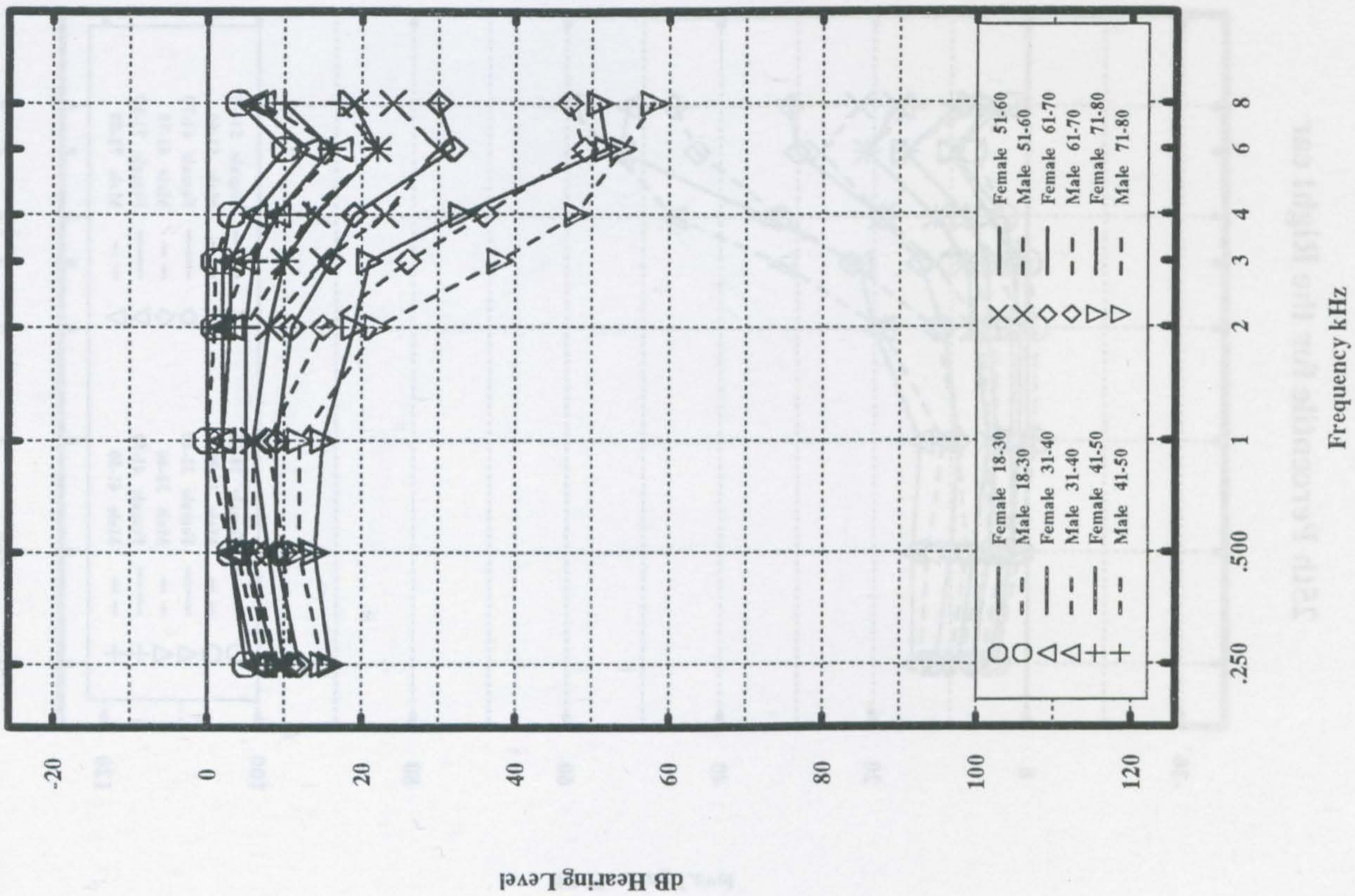

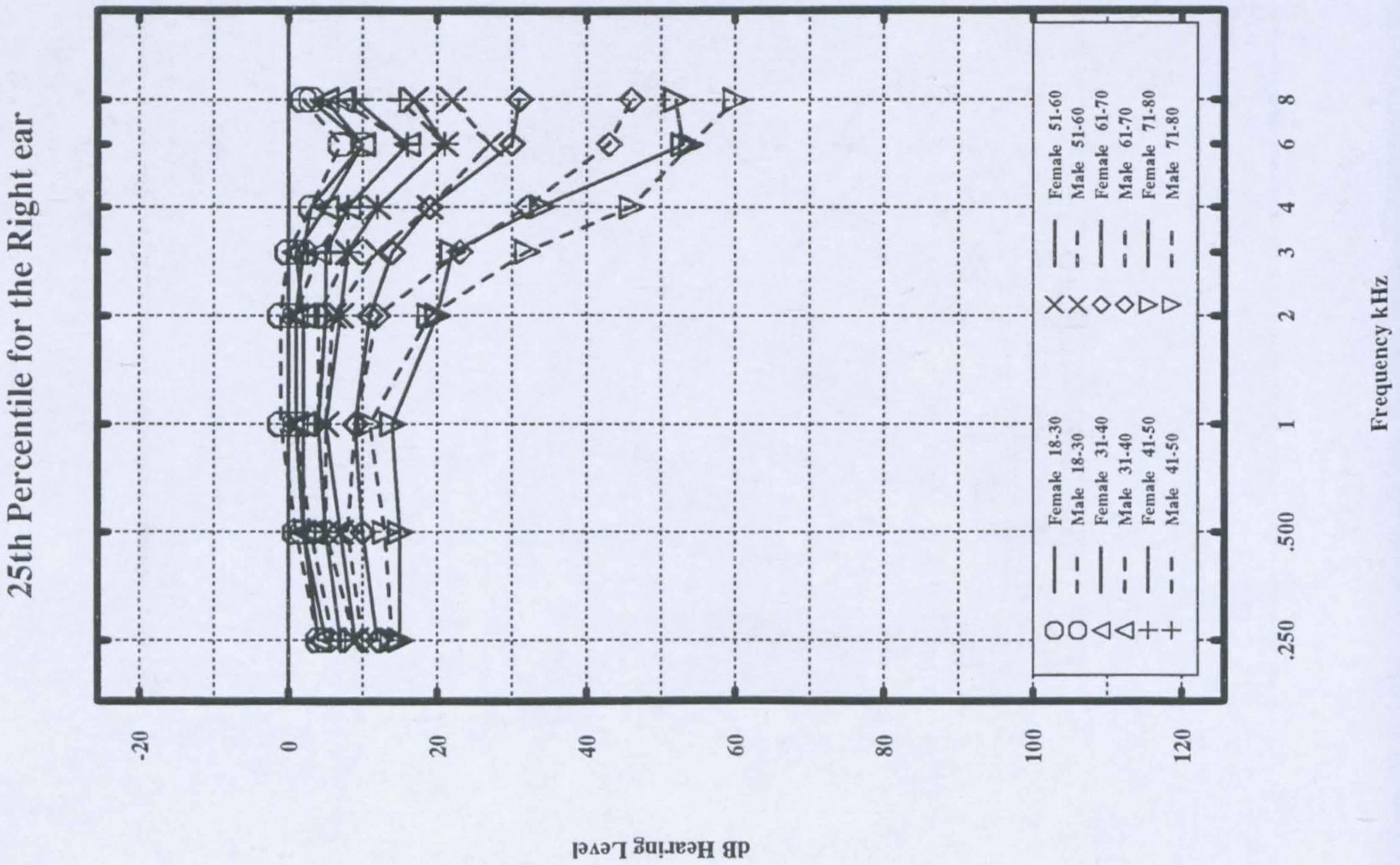
25th Percentile for the Right ear
dB Hearing Level
-20
0
20
40
60
80
100
120
Frequency kHz
.250
.500
1
2
3
4
6
8
Female 18-30
Male 18-30
Female 31-40
Male 31-40
Female 41-50
Male 41-50
Female 51-60
Male 51-60
Female 61-70
Male 61-70
Female 71-80
Male 71-80

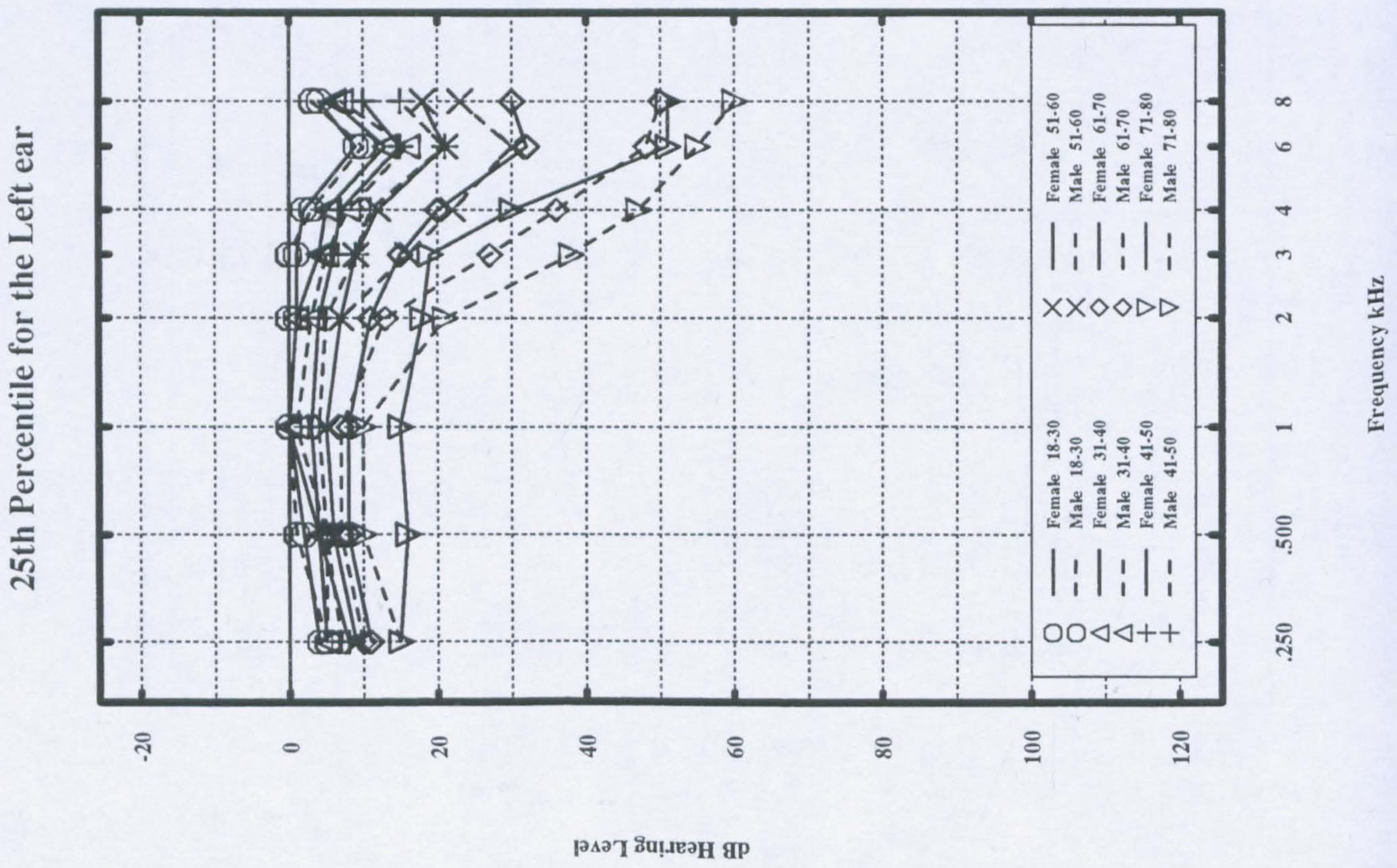
25th Percentile for the Left ear
dB Hearing Level
-20
0
20
40
60
80
100
120
Frequency kHz
.250
.500
1
2
3
4
6
8
Female 18-30
Male 18-30
Female 31-40
Male 31-40
Female 41-50
Male 41-50
Female 51-60
Male 51-60
Female 61-70
Male 61-70
Female 71-80
Male 71-80

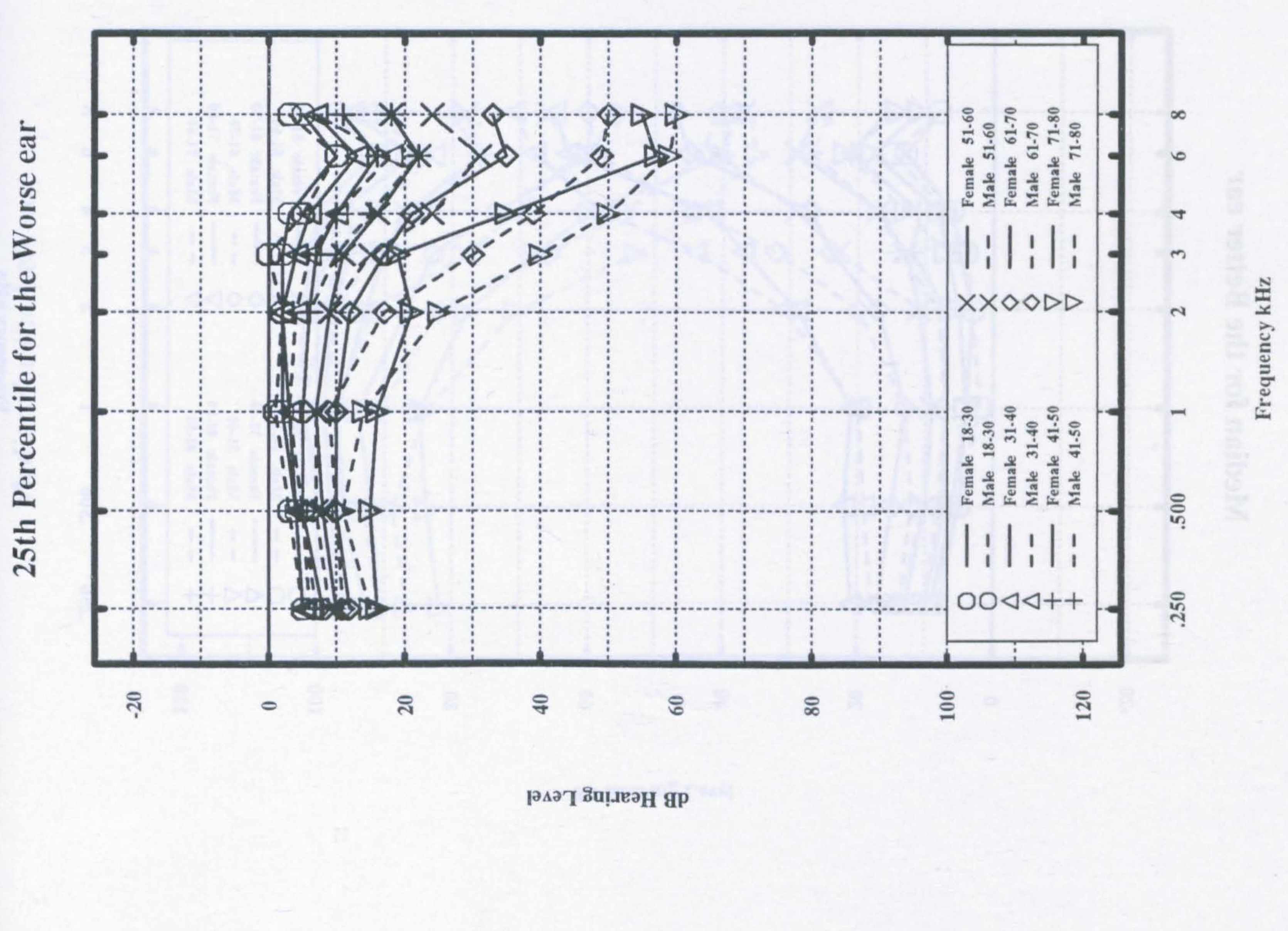
25th Percentile for the Worse ear
dB Hearing Level
-20
0
20
40
60
80
100
120
Frequency kHz
.250
.500
1
2
3
4
6
8
Female 18-30
Male 18-30
Female 31-40
Male 31-40
Female 41-50
Male 41-50
Female 51-60
Male 51-60
Female 61-70
Male 61-70
Female 71-80
Male 71-80

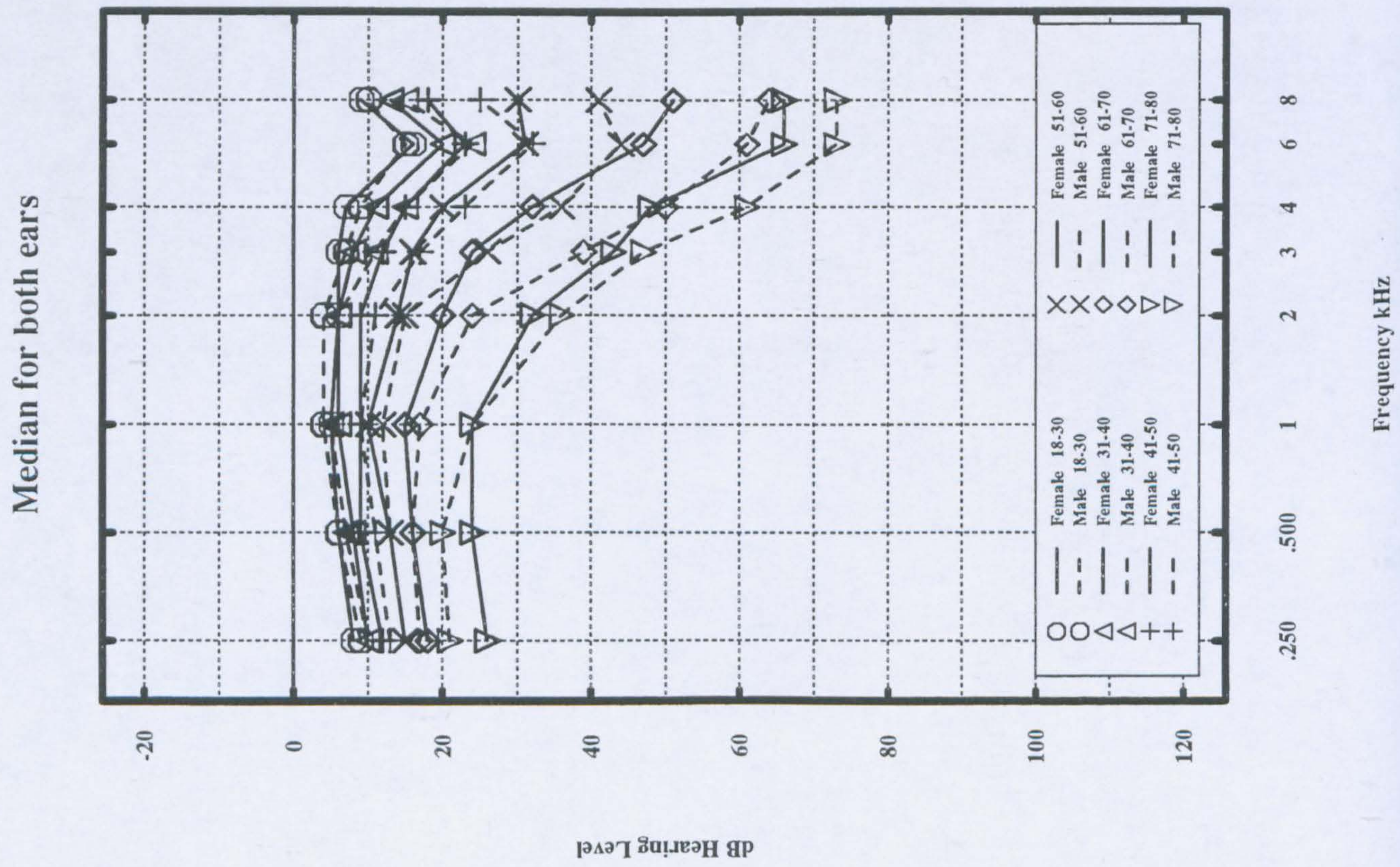
Median for both ears
dB Hearing Level
-20
0
20
40
60
80
100
120
.250
.500
1
2
3
4
6
8
Frequency kHz
Female 18-30
Male 18-30
Female 31-40
Male 31-40
Female 41-50
Male 41-50
Female 51-60
Male 51-60
Female 61-70
Male 61-70
Female 71-80
Male 71-80

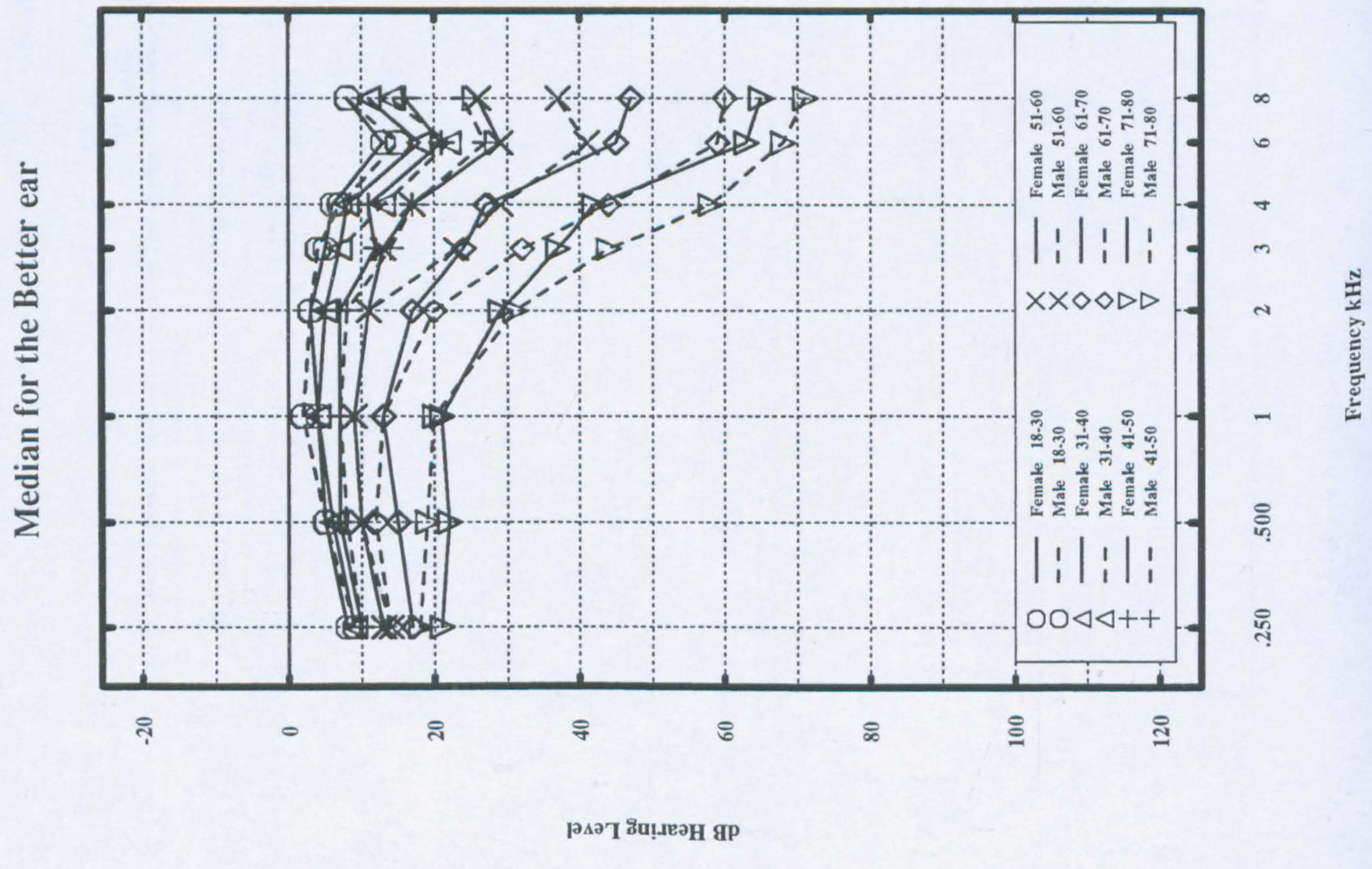
Median for the Better ear
dB Hearing Level
-20
0
20
40
60
80
100
120
.250
.500
1
2
3
4
6
8
Frequency kHz
Female 18-30
Male 18-30
Female 31-40
Male 31-40
Female 41-50
Male 41-50
Female 51-60
Male 51-60
Female 61-70
Male 61-70
Female 71-80
Male 71-80

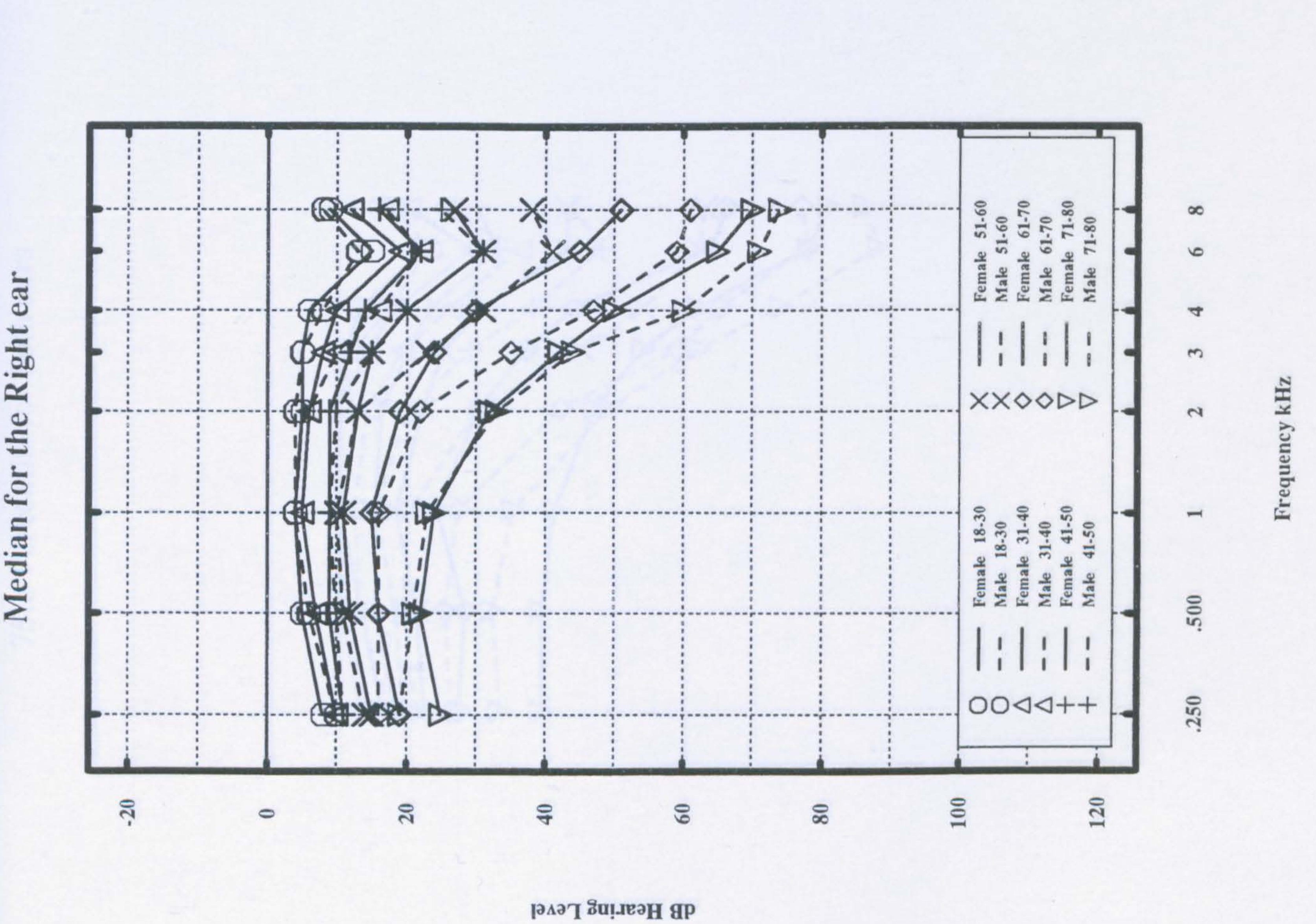
Median for the Right ear
dB Hearing Level
-20
0
20
40
60
80
100
120
Frequency kHz
.250
.500
1
2
3
4
6
8
Female 18-30
Male 18-30
Female 31-40
Male 31-40
Female 41-50
Male 41-50
Female 51-60
Male 51-60
Female 61-70
Male 61-70
Female 71-80
Male 71-80

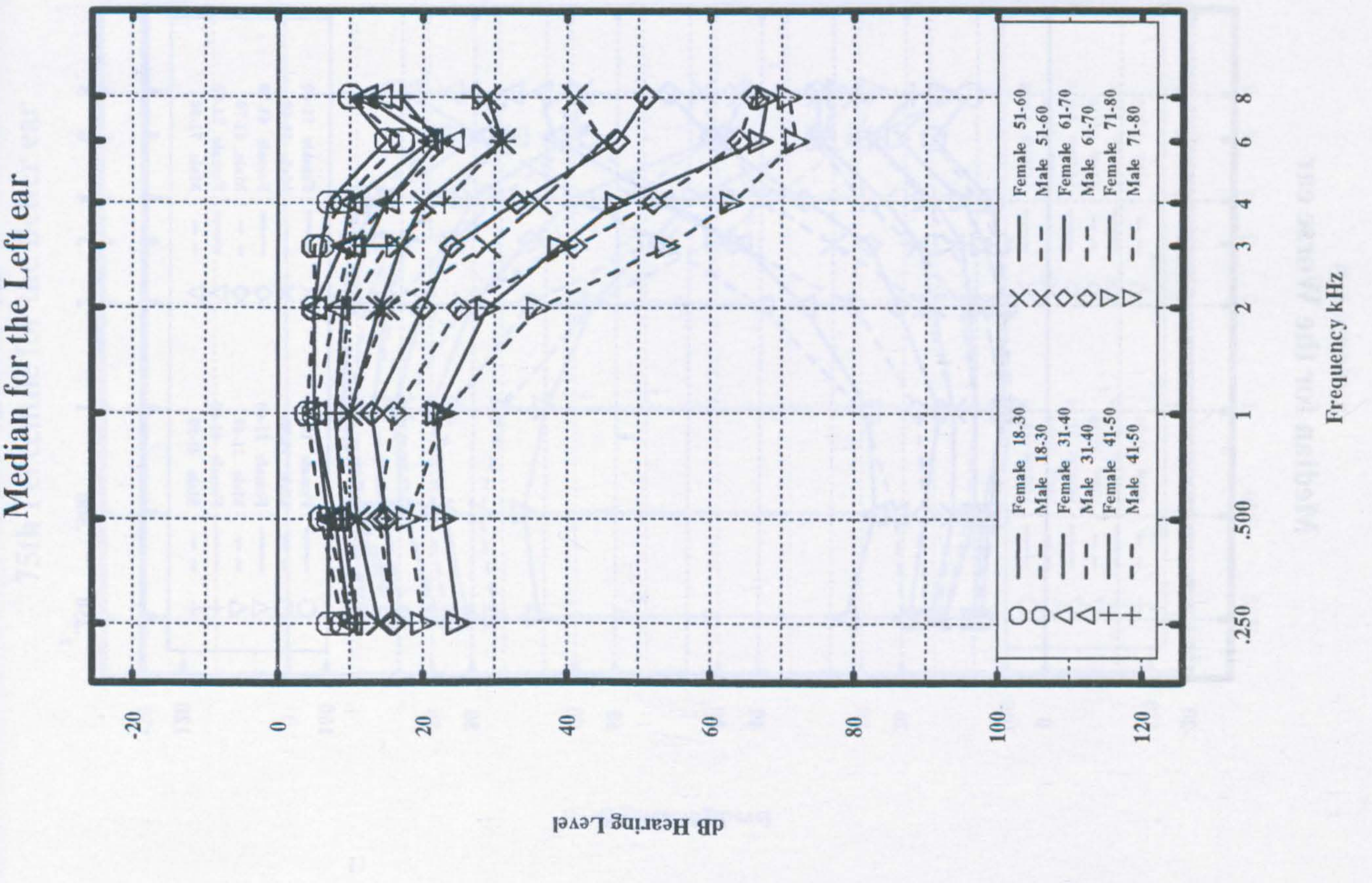
Median for the Left ear
dB Hearing Level
-20
0
20
40
60
80
100
120
Frequency kHz
.250
.500
1
2
3
4
6
8
Female 18-30
Male 18-30
Female 31-40
Male 31-40
Female 41-50
Male 41-50
Female 51-60
Male 51-60
Female 61-70
Male 61-70
Female 71-80
Male 71-80

Median for the Worse ear

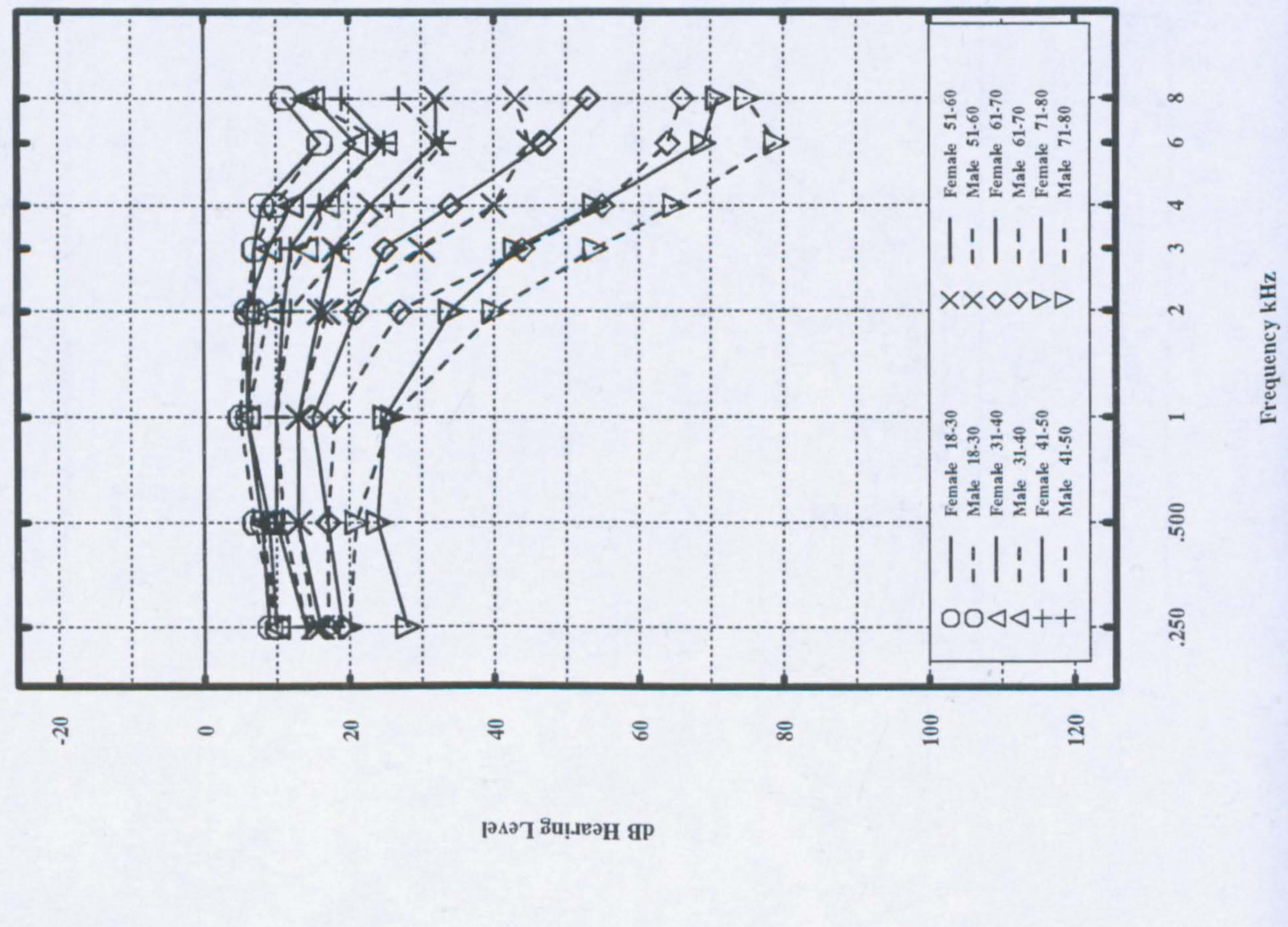

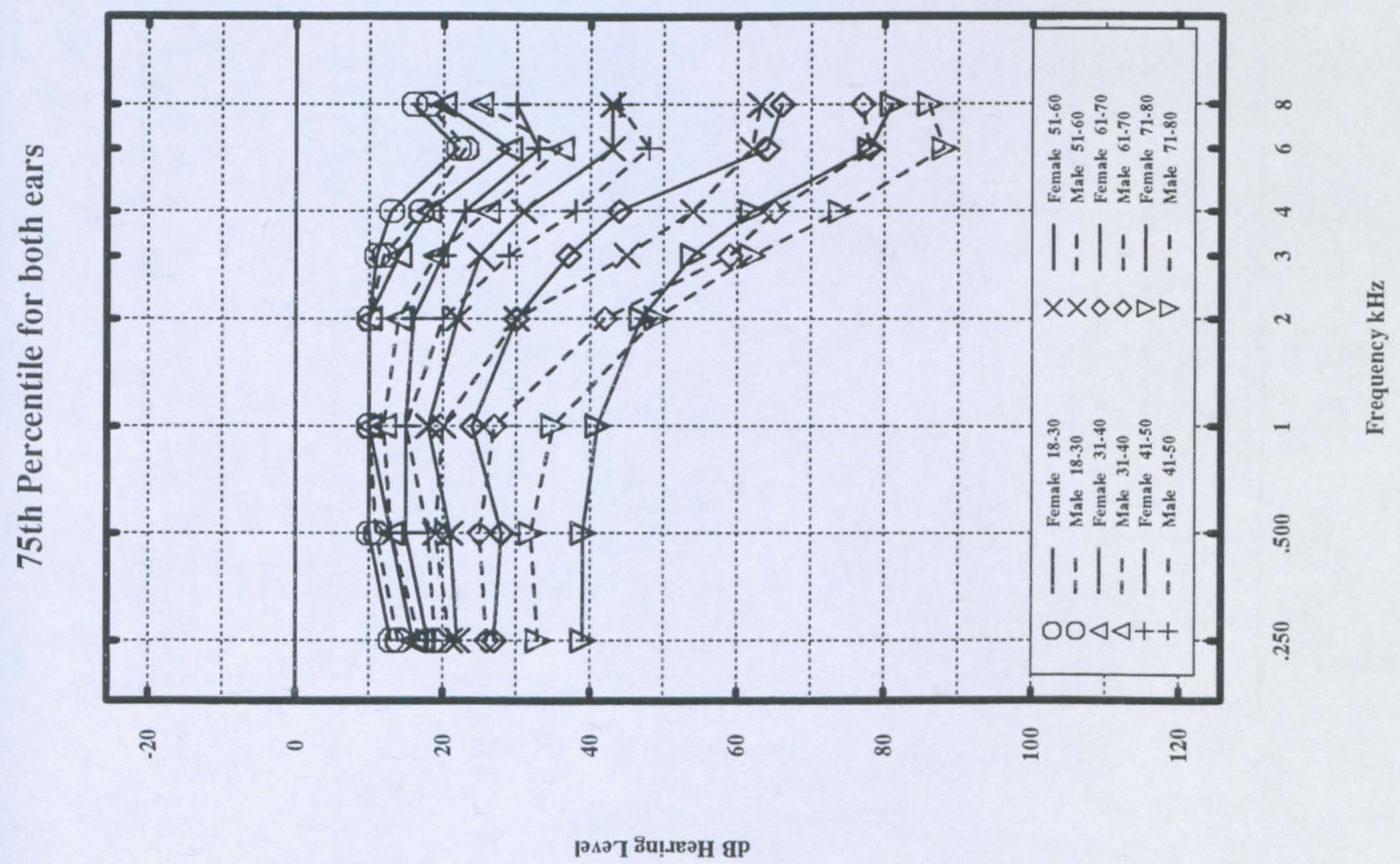
75th Percentile for both ears
dB Hearing Level
-20
0
20
40
60
80
100
120
.250
.500
1
2
3
4
6
8
Frequency kHz
Female 18-30
Male 18-30
Female 31-40
Male 31-40
Female 41-50
Male 41-50
Female 51-60
Male 51-60
Female 61-70
Male 61-70
Female 71-80
Male 71-80

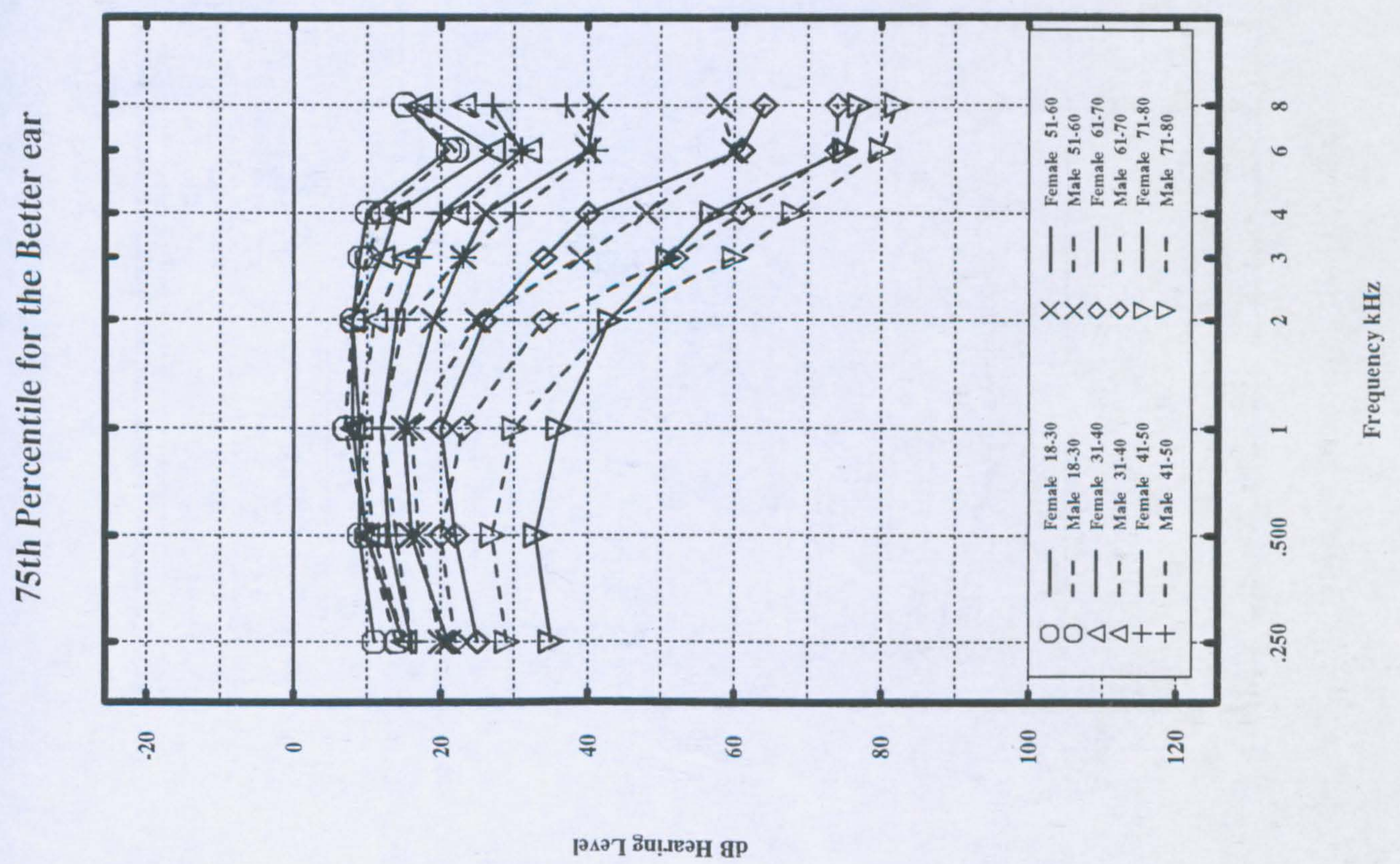
75th Percentile for the Better ear
dB Hearing Level
-20
0
20
40
60
80
100
120
.250
.500
1
2
3
4
6
8
Frequency kHz
Female 18-30
Male 18-30
Female 31-40
Male 31-40
Female 41-50
Male 41-50
Female 51-60
Male 51-60
Female 61-70
Male 61-70
Female 71-80
Male 71-80

75th Percentile for the Right ear

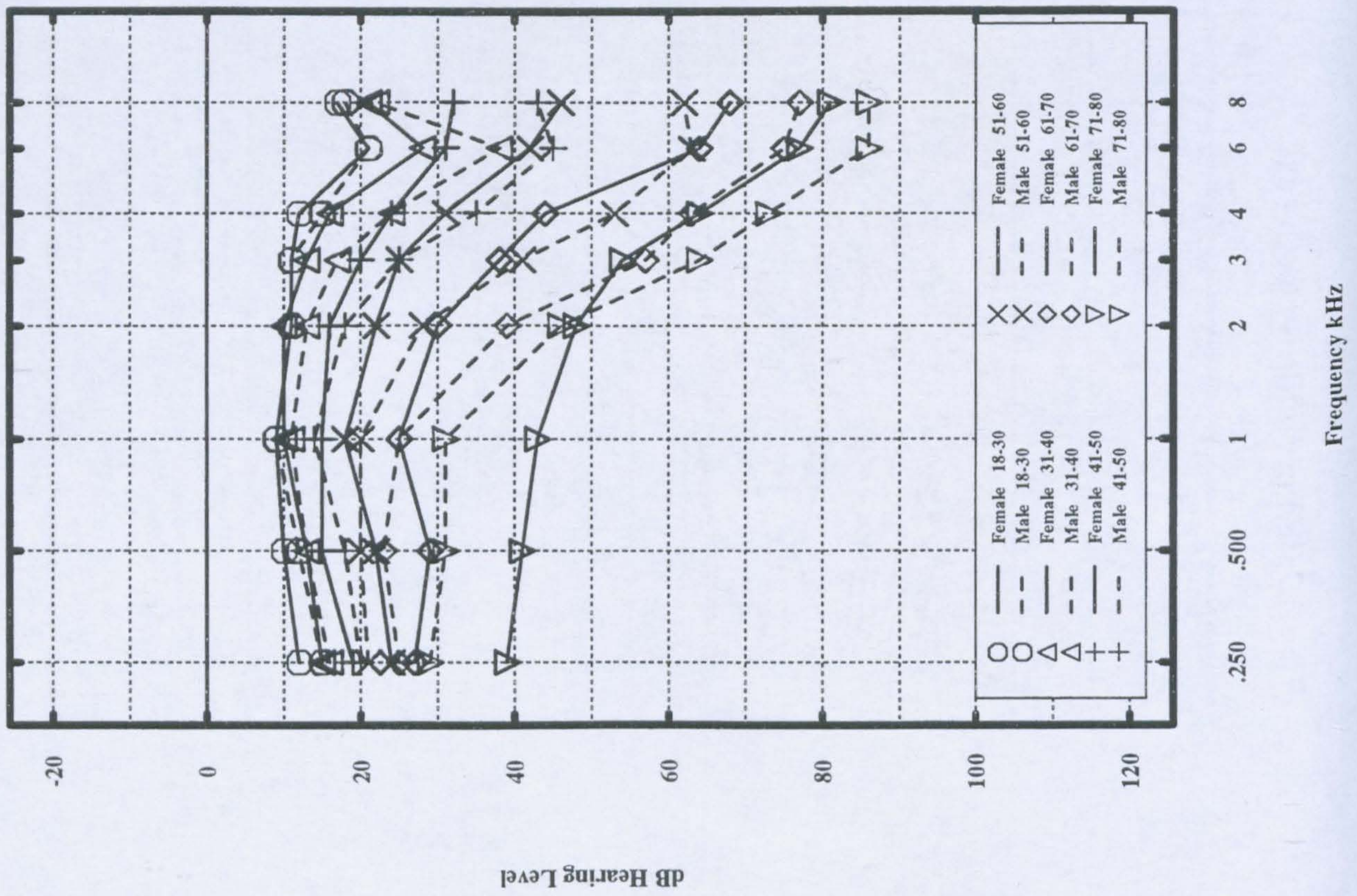

75th Percentile for the Left ear

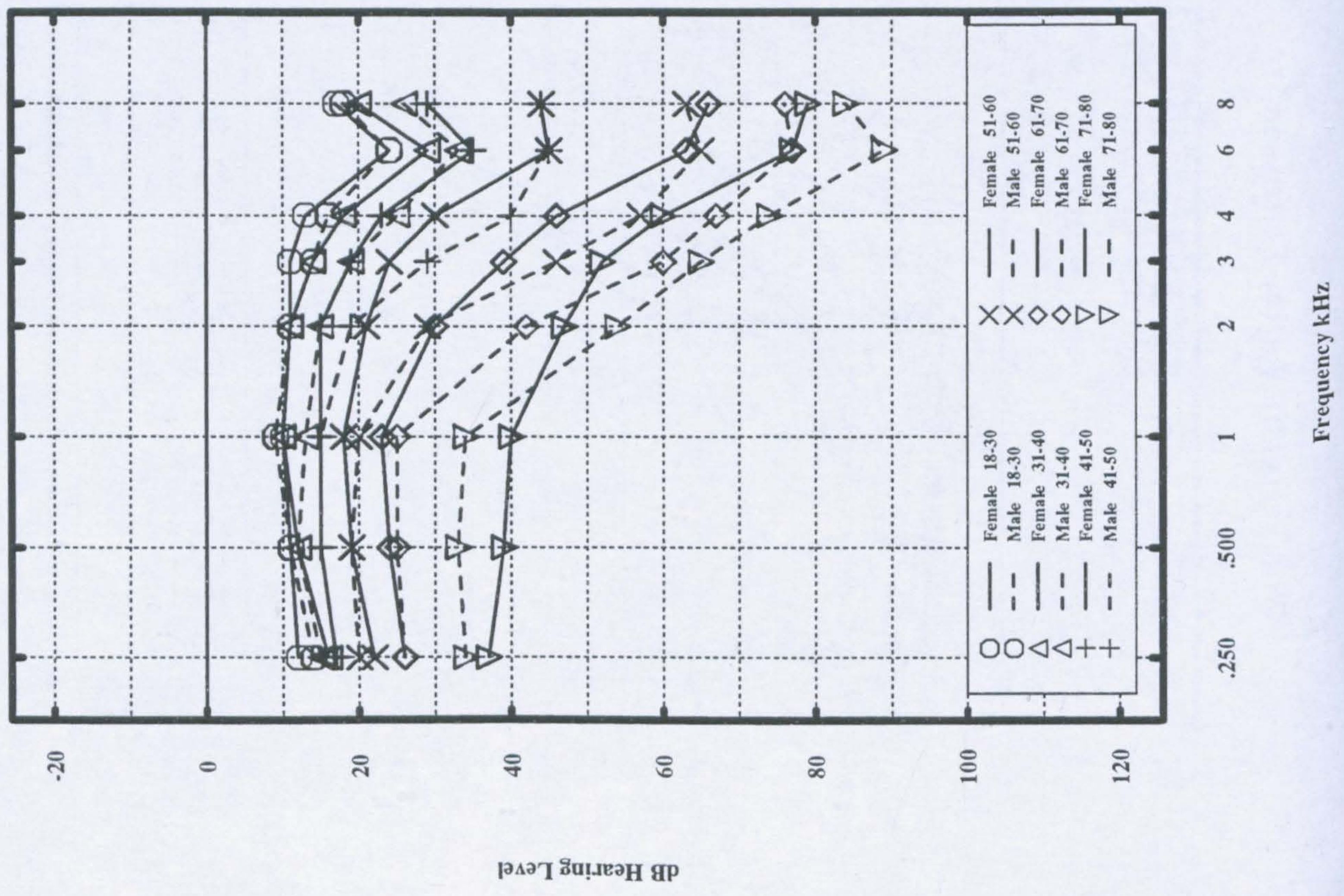

75th Percentile for the Worse ear

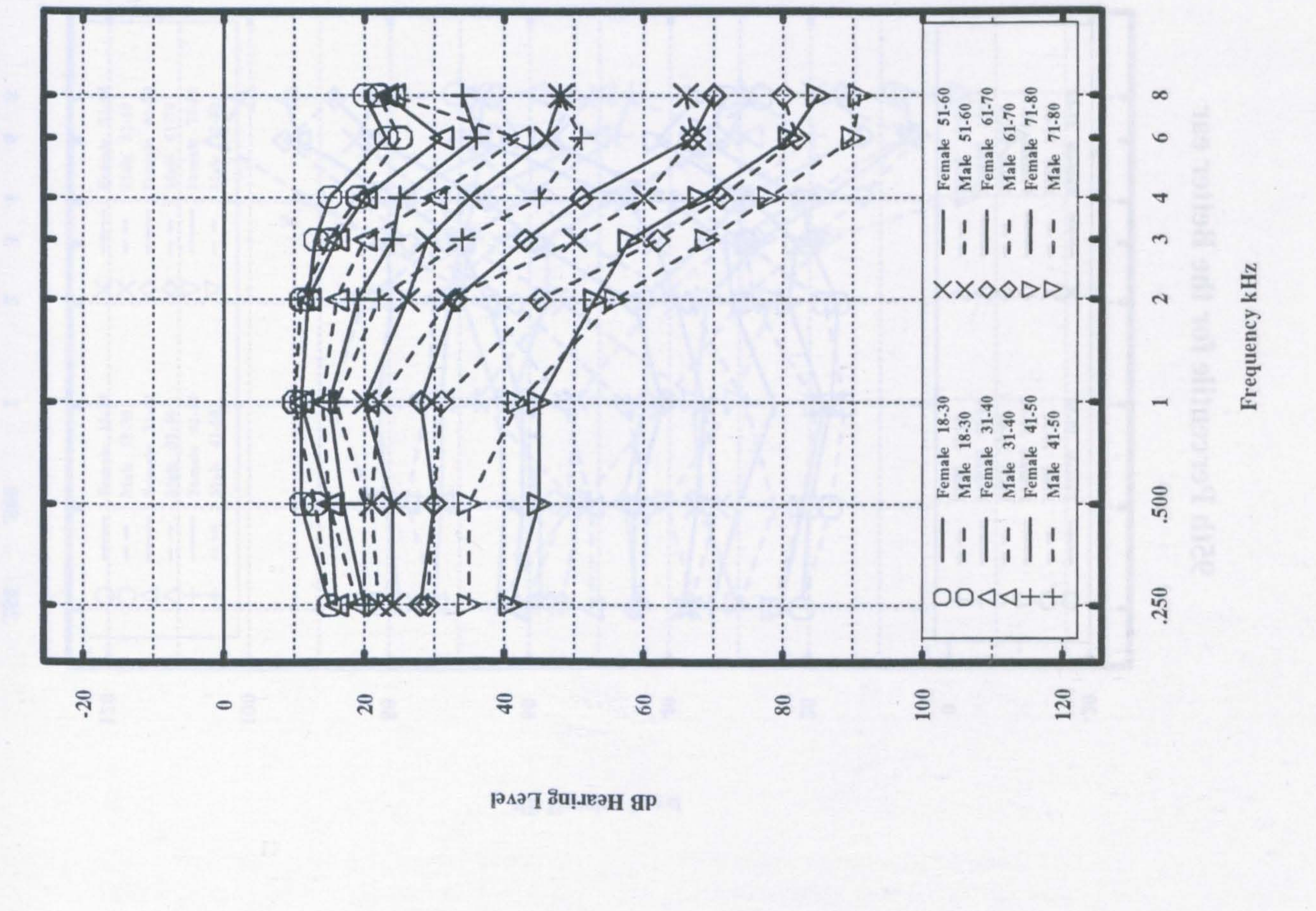

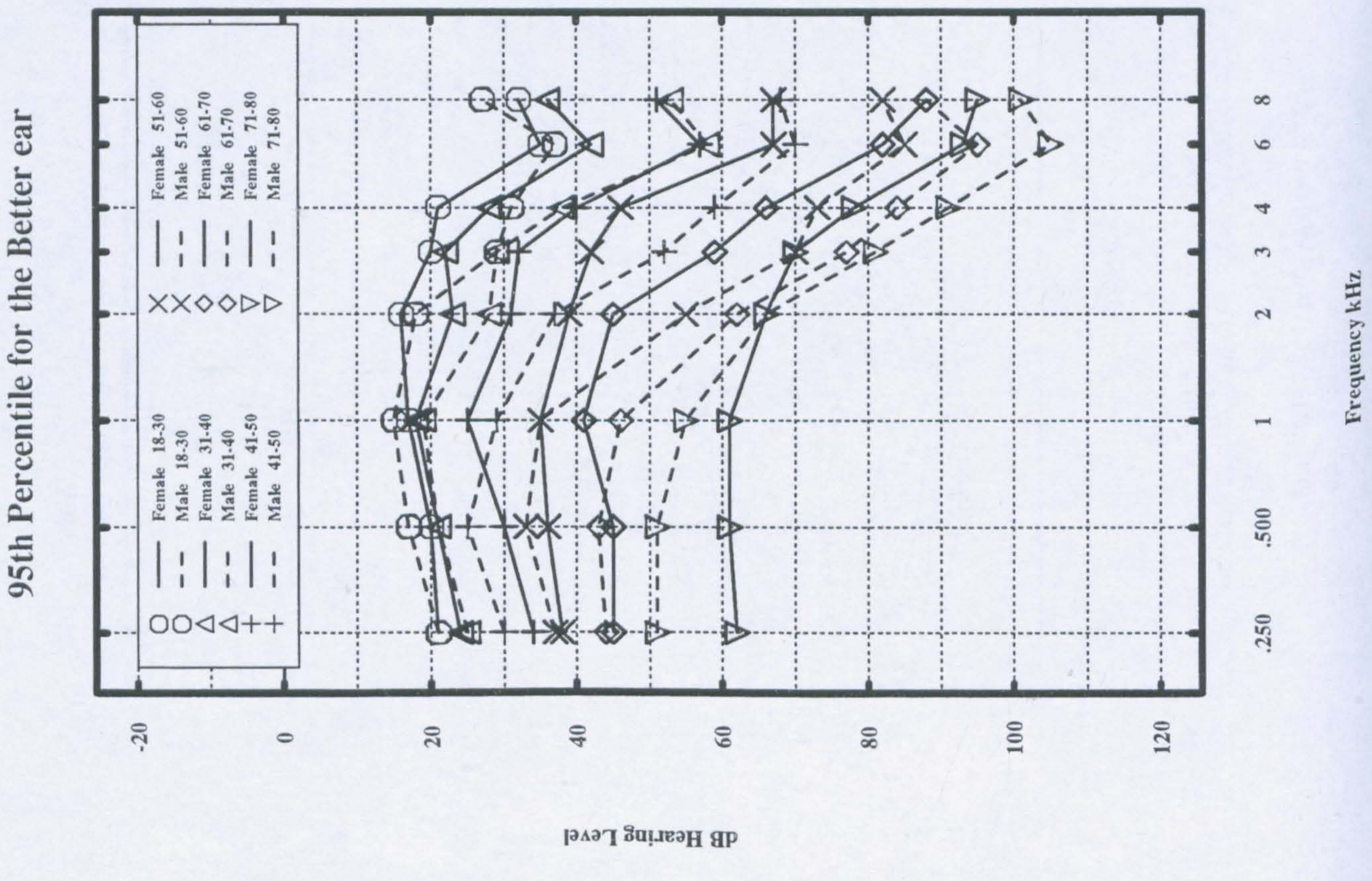
95th Percentile for the Better ear
Female 18-30
Male 18-30
Female 31-40
Male 31-40
Female 41-50
Male 41-50
Female 51-60
Male 51-60
Female 61-70
Male 61-70
Female 71-80
Male 71-80
dB Hearing Level
-20
0
20
40
60
80
100
120
Frequency kHz
.250
.500
1
2
3
4
6
8

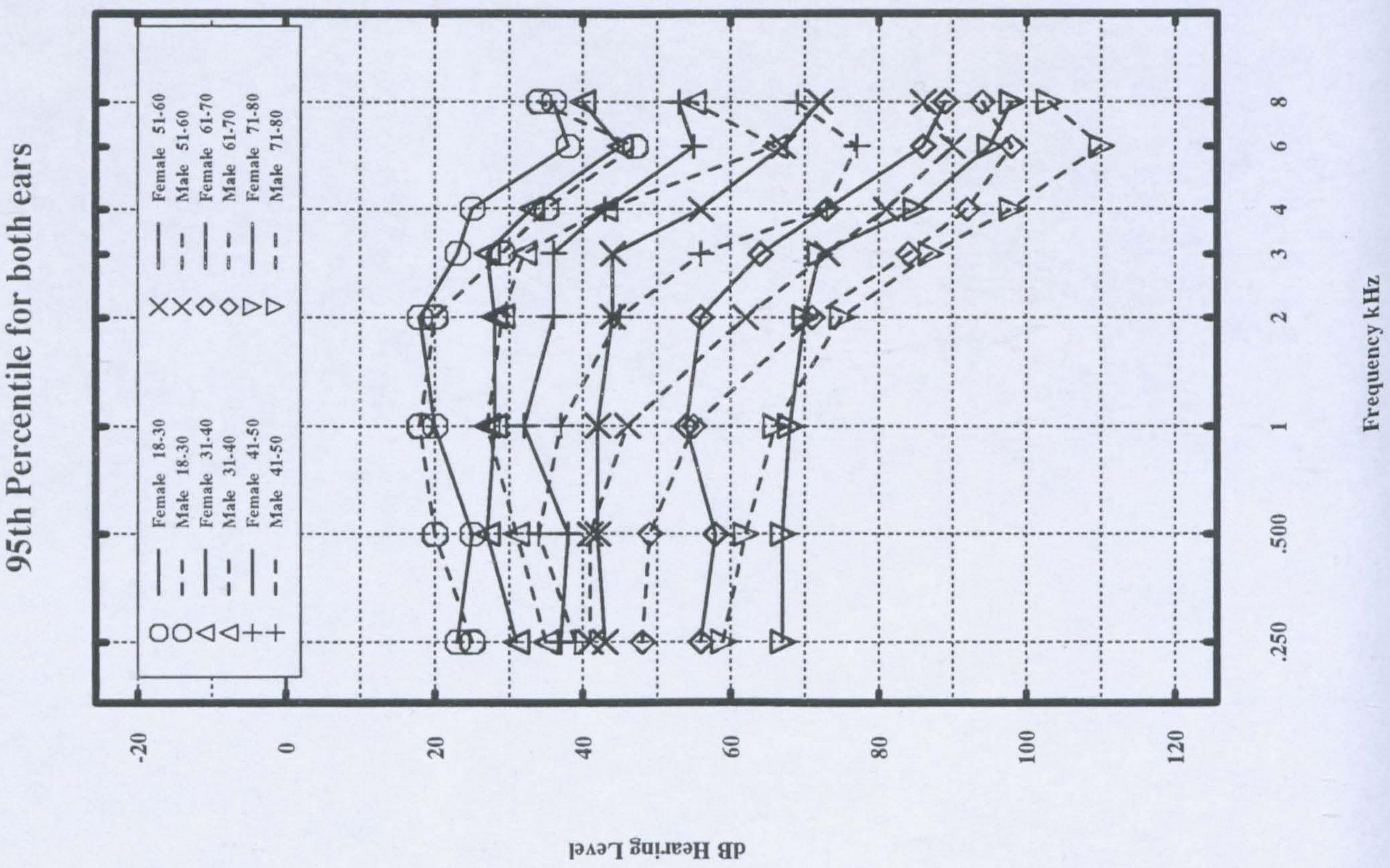
95th Percentile for both ears
Female 18-30
Male 18-30
Female 31-40
Male 31-40
Female 41-50
Male 41-50
Female 51-60
Male 51-60
Female 61-70
Male 61-70
Female 71-80
Male 71-80
dB Hearing Level
-20
0
20
40
60
80
100
120
Frequency kHz
.250
.500
1
2
3
4
6
8

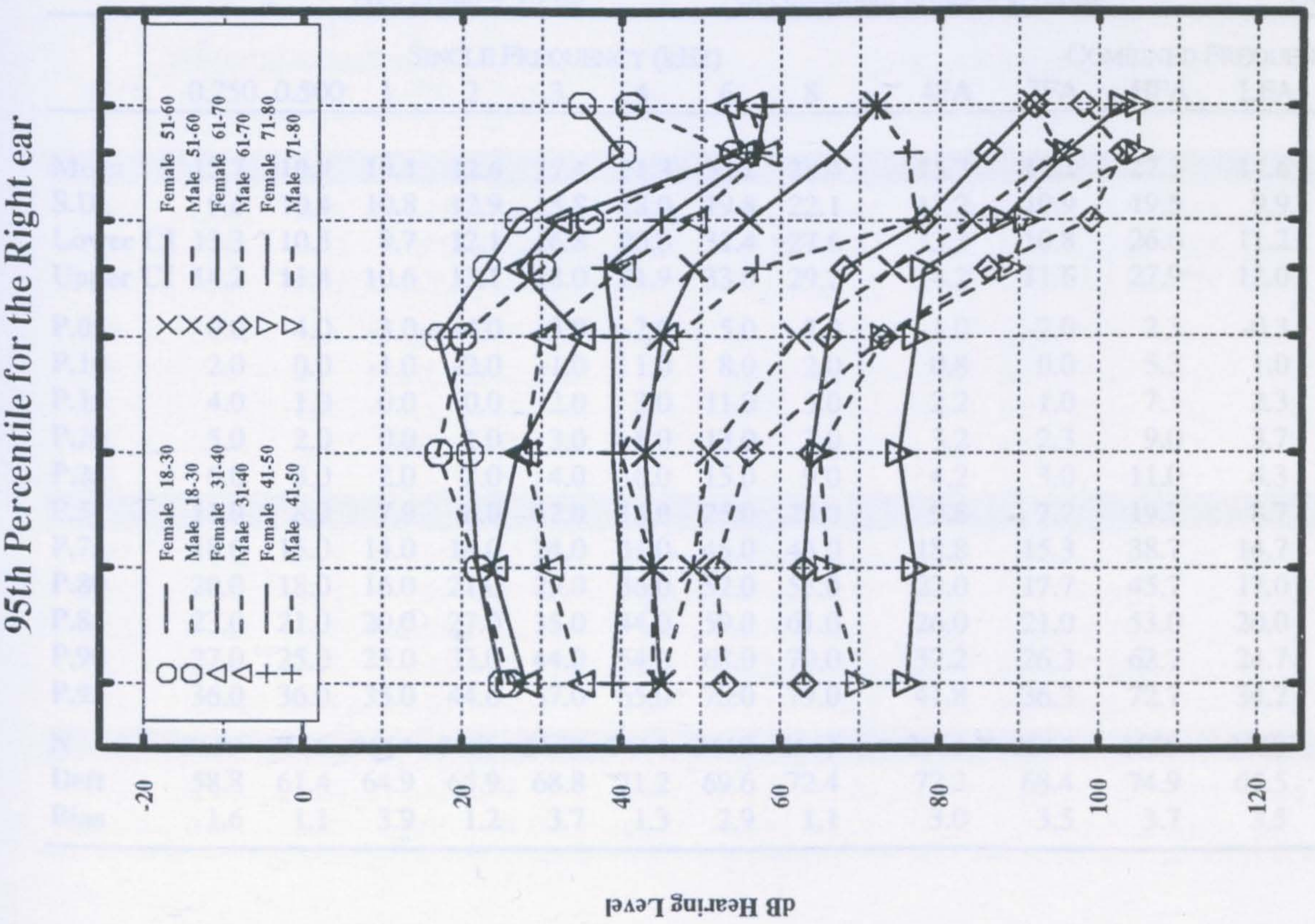
95th Percentile for the Right ear
Female 18-30
Male 18-30
Female 31-40
Male 31-40
Female 41-50
Male 41-50
Female 51-60
Male 51-60
Female 61-70
Male 61-70
Female 71-80
Male 71-80
dB Hearing Level
Frequency kHz
250 500 1 2 3 4 6 8
-20 0 20 40 60 80 100 120

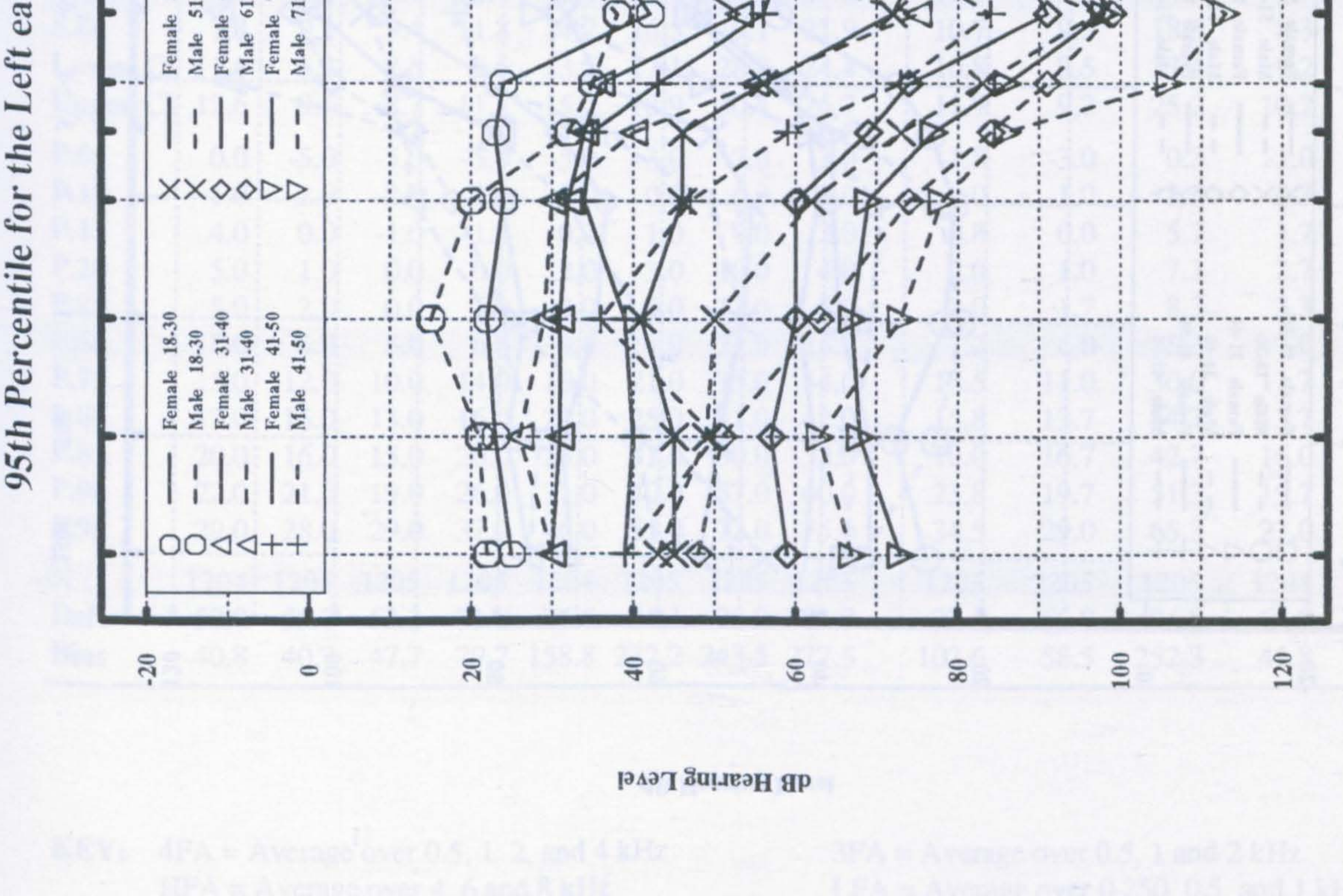
95th Percentile for the Left ear
Female 18-30
Male 18-30
Female 31-40
Male 31-40
Female 41-50
Male 41-50
Female 51-60
Male 51-60
Female 61-70
Male 61-70
Female 71-80
Male 71-80
dB Hearing Level
Frequency kHz
250 500 1 2 3 4 6 8
-20 0 20 40 60 80 100 120

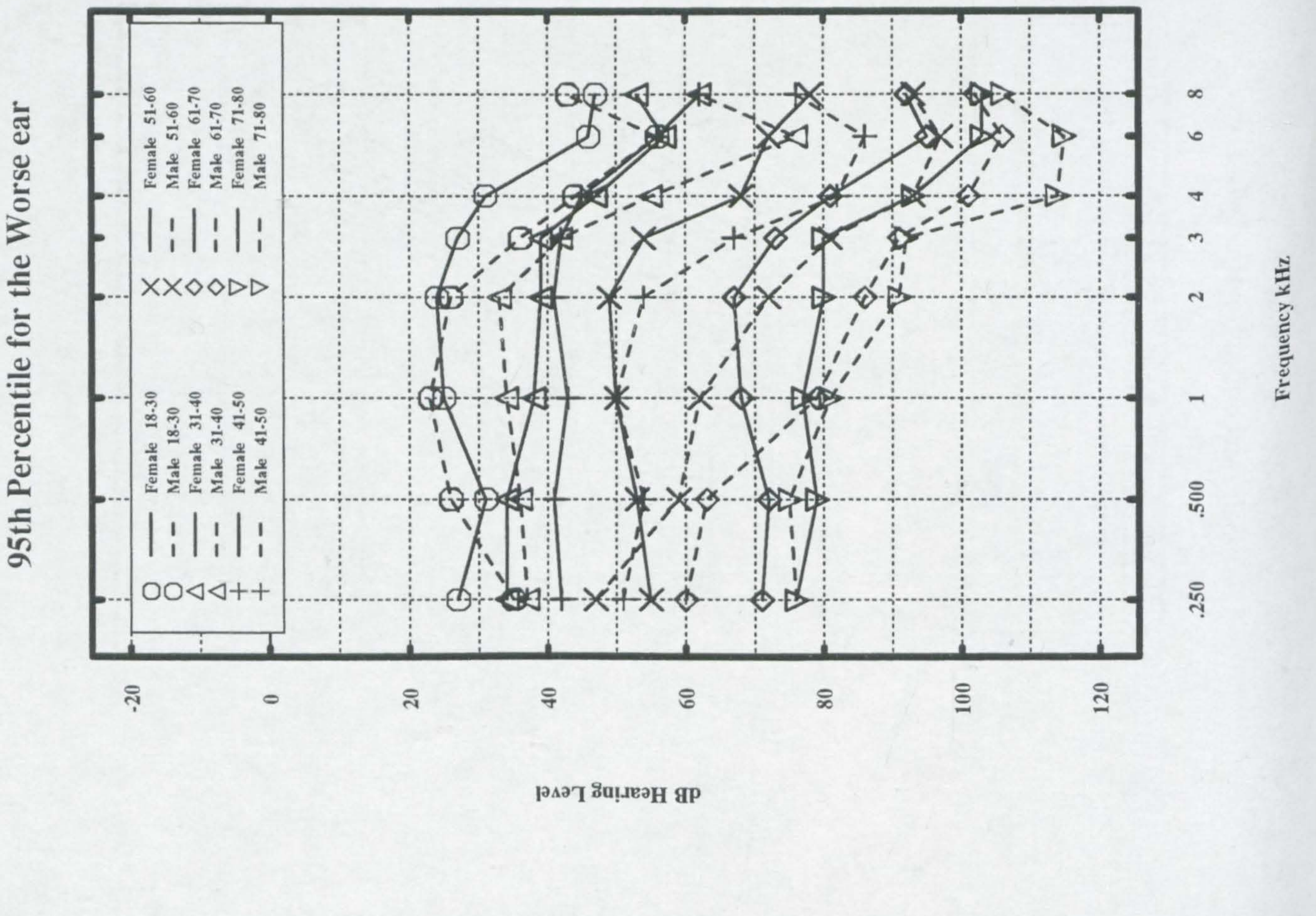
95th Percentile for the Worse ear
Female 18-30
Male 18-30
Female 31-40
Male 31-40
Female 41-50
Male 41-50
Female 51-60
Male 51-60
Female 61-70
Male 61-70
Female 71-80
Male 71-80
dB Hearing Level
-20
0
20
40
60
80
100
120
Frequency kHz
.250
.500
1
2
3
4
6
8

Mean, standard deviation, confidence intervals, and percentiles for hearing thresholds as a function of ear, age group, socioeconomic group, gender, sample and frequency

TABLE 1 SAMPLE = ALL — EAR = BETTER EAR
Age group = 18-80 — Occupational group = Overall — Gender = Overall

	Single Frequency (kHz)								Combined Frequency (kHz)					
	0.250	0.500	1	2	3	4	6	8	4FA	3FA	HFA	LFA	A123	A124
**Mean**	**13.7**	**10.9**	**10.1**	**12.6**	**17.4**	**21.3**	**32.2**	**28.4**	**13.7**	**11.2**	**27.3**	**11.6**	**13.4**	**14.7**
**S.D.**	9.6	10.4	10.8	12.9	15.8	18.0	19.8	22.1	12.2	10.9	19.5	9.9	12.6	13.2
**Lower CI**	13.3	10.5	9.7	12.1	16.8	20.6	31.4	27.6	13.3	10.8	26.6	11.2	12.9	14.2
**Upper CI**	14.2	11.4	10.6	13.1	18.0	21.9	33.0	29.2	14.2	11.6	27.9	12.0	13.8	15.1
**P.05**	0.0	-4.0	-3.0	-4.0	-3.0	-2.0	5.0	-2.0	-1.0	-2.0	2.3	-0.3	-2.3	-1.0
**P.10**	2.0	0.0	-1.0	-2.0	-1.0	1.0	8.0	2.0	0.8	0.0	5.3	1.0	-0.3	0.7
**P.15**	4.0	1.0	0.0	0.0	2.0	3.0	11.0	5.0	2.2	1.0	7.3	2.3	1.3	2.0
**P.20**	5.0	2.0	0.0	1.0	3.0	5.0	13.0	7.0	3.2	2.3	9.0	3.7	2.7	3.0
**P.25**	6.0	3.0	2.0	2.0	4.0	6.0	15.0	9.0	4.2	3.0	11.0	4.3	3.3	4.3
**P.50**	**11.0**	**8.0**	**7.0**	**8.0**	**12.0**	**15.0**	**25.0**	**20.0**	**9.8**	**7.7**	**19.7**	**8.7**	**9.3**	**10.3**
**P.75**	18.0	15.0	14.0	18.0	24.0	31.0	45.0	45.0	18.8	15.3	38.7	14.7	18.7	20.0
**P.80**	20.0	18.0	16.0	21.0	29.0	36.0	52.0	53.0	22.0	17.7	45.7	17.0	21.7	24.0
**P.85**	23.0	21.0	20.0	27.0	35.0	44.0	59.0	61.0	26.0	21.0	53.0	20.0	26.3	28.3
**P.90**	27.0	25.0	25.0	33.0	44.0	54.0	68.0	70.0	32.2	26.3	62.7	24.7	33.0	35.0
**P.95**	36.0	36.0	35.0	44.0	57.0	65.0	78.0	79.0	41.8	36.3	72.7	34.7	43.0	45.3
**N**	**2659**	**2666**	**2664**	**2666**	**2659**	**2664**	**2660**	**2661**	**2663**	**2664**	**2658**	**2658**	**2657**	**2663**
**Deft**	58.8	61.4	64.9	65.9	68.8	71.2	69.6	72.4	72.2	68.4	74.9	65.5	70.7	72.5
**Bias**	1.6	1.1	3.9	1.2	3.7	1.3	2.9	1.1	3.0	3.5	3.7	3.5	5.1	3.0

TABLE 2 SAMPLE = NO NOISE OR ABG>0 — EAR = BETTER EAR
Age group = 18-80 — Occupational group = Overall — Gender = Overall

	Single Frequency (kHz)								Combined Frequency (kHz)					
	0.250	0.500	1	2	3	4	6	8	4FA	3FA	HFA	LFA	A123	A124
**Mean**	**12.0**	**8.9**	**8.1**	**10.4**	**14.4**	**18.0**	**29.3**	**25.6**	**11.3**	**9.1**	**24.3**	**9.7**	**11.0**	**12.1**
**S.D.**	7.8	9.1	9.4	11.8	14.2	16.5	19.1	21.9	10.7	9.5	18.5	8.3	11.2	11.8
**Lower CI**	11.5	8.3	7.5	9.6	13.5	17.1	28.2	24.4	10.8	8.5	23.4	9.2	10.3	11.5
**Upper CI**	12.6	9.6	8.7	11.1	15.2	18.9	30.4	26.7	11.9	9.7	25.2	10.2	11.6	12.8
**P.05**	0.0	-5.0	-5.0	-5.0	-5.0	-3.0	2.0	-3.0	-1.8	-3.0	0.3	-1.0	-3.0	-2.0
**P.10**	1.0	-2.0	-2.0	-3.0	-2.0	0.0	6.0	0.0	0.0	-1.0	3.0	0.3	-1.3	-0.7
**P.15**	4.0	0.0	-1.0	-1.0	0.0	1.0	9.0	2.0	0.8	0.0	5.7	1.7	0.0	1.0
**P.20**	5.0	1.0	0.0	0.0	2.0	3.0	10.0	4.0	2.0	1.0	7.3	2.7	1.3	1.7
**P.25**	5.0	2.0	0.0	1.0	3.0	5.0	12.0	6.0	3.0	1.7	8.3	3.3	2.0	2.7
**P.50**	**10.0**	**6.0**	**5.0**	**6.0**	**9.0**	**11.0**	**21.0**	**15.0**	**7.2**	**6.0**	**15.3**	**7.0**	**6.7**	**7.7**
**P.75**	15.0	12.0	10.0	14.0	18.0	21.0	35.0	34.0	13.5	11.0	30.0	11.7	13.3	14.7
**P.80**	17.0	15.0	13.0	16.0	21.0	25.0	41.0	42.0	15.8	13.7	34.7	13.7	15.7	17.0
**P.85**	20.0	16.0	15.0	20.0	25.0	31.0	50.0	52.0	19.0	16.7	42.7	16.0	19.0	19.7
**P.90**	22.0	21.0	19.0	26.0	31.0	40.0	57.0	60.0	23.8	19.7	51.3	19.7	24.7	26.0
**P.95**	29.0	28.0	29.0	37.0	46.0	55.0	72.0	75.0	34.5	29.0	65.3	27.0	34.7	37.7
**N**	**1204**	**1205**	**1205**	**1205**	**1204**	**1205**	**1205**	**1205**	**1205**	**1205**	**1205**	**1204**	**1204**	**1205**
**Deft**	52.8	58.7	62.3	71.0	76.5	84.1	78.9	81.7	73.5	66.9	84.8	61.0	72.9	76.4
**Bias**	40.8	40.7	47.7	79.7	158.8	232.2	243.5	277.5	102.6	58.5	252.3	46.8	98.2	121.8

KEY:
4FA = Average over 0.5, 1, 2, and 4 kHz
3FA = Average over 0.5, 1 and 2 kHz
HFA = Average over 4, 6 and 8 kHz
LFA = Average over 0.250, 0.5, and 1 kHz
A123 = Average over 1, 2, and 3 kHz
A124 = Average over 1, 2, and 4 kHz

Mean, standard deviation, confidence intervals, and percentiles for hearing thresholds as a function of ear, age group, socioeconomic group, gender, sample and frequency

TABLE 3 SAMPLE = ALL; Age group = 18-80; Occupational group = Overall; EAR = BETTER EAR; Gender = Female

	SINGLE FREQUENCY (kHz)								COMBINED FREQUENCY (kHz)					
	0.250	0.500	1	2	3	4	6	8	4FA	3FA	HFA	LFA	A123	A124
**Mean**	**14.2**	**11.6**	**10.7**	**12.2**	**15.0**	**17.8**	**29.6**	**26.2**	**13.1**	**11.5**	**24.5**	**12.1**	**12.6**	**13.6**
**S.D.**	10.2	11.2	11.4	12.6	13.9	15.6	18.2	20.8	12.1	11.4	17.7	10.6	12.3	12.7
**Lower CI**	13.6	11.0	10.2	11.6	14.3	17.1	28.6	25.2	12.6	11.0	23.7	11.6	12.1	13.0
**Upper CI**	14.7	12.2	11.3	12.8	15.7	18.6	30.5	27.2	13.6	12.0	25.3	12.6	13.2	14.1
**P.05**	0.0	-3.0	-3.0	-4.0	-3.0	-2.0	5.0	-1.0	-0.8	-1.3	2.3	0.0	-1.7	-1.0
**P.10**	3.0	0.0	-1.0	-1.0	-1.0	0.0	8.0	2.0	0.8	0.3	5.3	1.7	0.3	1.0
**P.15**	4.0	1.0	0.0	1.0	2.0	3.0	10.0	5.0	2.5	1.7	7.0	3.0	1.3	2.0
**P.20**	5.0	2.0	1.0	2.0	3.0	5.0	12.0	7.0	3.2	2.7	8.7	4.0	2.7	3.0
**P.25**	6.0	4.0	3.0	3.0	4.0	6.0	14.0	8.0	4.2	3.3	10.0	4.3	3.7	4.0
**P.50**	**11.0**	**9.0**	**7.0**	**8.0**	**11.0**	**13.0**	**24.0**	**18.0**	**9.2**	**8.0**	**18.3**	**9.0**	**9.0**	**9.3**
**P.75**	19.0	16.0	15.0	17.0	22.0	25.0	39.0	40.0	17.2	15.0	34.3	15.0	16.7	18.3
**P.80**	21.0	19.0	17.0	20.0	24.0	29.0	47.0	47.0	20.0	17.7	39.7	18.0	19.7	21.0
**P.85**	24.0	22.0	20.0	25.0	30.0	36.0	54.0	56.0	24.0	21.0	47.7	20.7	24.0	25.3
**P.90**	29.0	28.0	26.0	31.0	38.0	44.0	63.0	66.0	30.2	27.0	56.0	27.0	30.0	32.7
**P.95**	40.0	39.0	38.0	42.0	50.0	56.0	75.0	77.0	41.2	38.0	67.3	37.0	42.0	44.3
**N**	**1384**	**1389**	**1387**	**1389**	**1387**	**1387**	**1386**	**1386**	**1386**	**1387**	**1385**	**1383**	**1385**	**1386**
**Deft**	63.7	66.0	69.5	70.2	69.6	71.7	68.7	71.6	76.1	73.4	74.5	70.7	74.2	75.8
**Bias**	-1.0	-2.6	0.2	-4.4	-5.3	-9.0	-10.1	-13.9	-1.9	0.4	-10.0	0.6	-0.9	-2.6

TABLE 4 SAMPLE = NO NOISE OR ABG>0; Age group = 18-80; Occupational group = Overall; EAR = BETTER EAR; Gender = Female

	SINGLE FREQUENCY (kHz)								COMBINED FREQUENCY (kHz)					
	0.250	0.500	1	2	3	4	6	8	4FA	3FA	HFA	LFA	A123	A124
**Mean**	**12.7**	**9.9**	**9.2**	**10.9**	**13.7**	**16.2**	**28.3**	**25.0**	**11.5**	**10.0**	**23.2**	**10.6**	**11.2**	**12.1**
**S.D.**	8.4	9.5	9.8	11.6	13.3	15.3	18.0	21.3	10.9	9.9	17.6	8.9	11.2	11.7
**Lower CI**	12.1	9.2	8.6	10.1	12.8	15.4	27.3	23.9	10.9	9.4	22.3	10.0	10.6	11.4
**Upper CI**	13.3	10.6	9.8	11.6	14.5	17.1	29.4	26.1	12.1	10.6	24.1	11.2	11.9	12.7
**P.05**	0.0	-4.0	-3.0	-4.0	-3.0	-2.0	4.0	-2.0	-0.8	-1.7	1.3	-0.3	-2.0	-1.3
**P.10**	2.0	-1.0	-1.0	-2.0	-1.0	0.0	7.0	2.0	0.2	-0.3	4.7	1.3	0.0	0.7
**P.15**	4.0	0.0	0.0	0.0	1.0	2.0	10.0	4.0	1.8	1.0	6.7	2.3	1.0	1.3
**P.20**	5.0	1.0	0.0	1.0	2.0	4.0	11.0	6.0	2.5	1.7	8.0	3.3	2.0	2.3
**P.25**	5.0	2.0	2.0	2.0	3.0	5.0	12.0	7.0	3.5	2.7	9.0	4.0	3.0	3.3
**P.50**	**10.0**	**7.0**	**6.0**	**7.0**	**9.0**	**10.0**	**22.0**	**15.0**	**7.8**	**6.7**	**15.7**	**7.3**	**7.3**	**8.0**
**P.75**	16.0	13.0	11.0	14.0	18.0	20.0	35.0	35.0	13.5	11.7	30.0	12.7	13.7	14.7
**P.80**	18.0	15.0	14.0	16.0	21.0	25.0	40.0	43.0	16.0	14.7	34.7	14.3	16.0	17.3
**P.85**	20.0	19.0	15.0	20.0	24.0	28.0	50.0	52.0	19.2	17.0	40.7	17.3	19.0	19.7
**P.90**	23.0	22.0	20.0	26.0	31.0	37.0	57.0	60.0	23.8	21.0	51.3	20.3	24.0	25.0
**P.95**	29.0	30.0	32.0	38.0	45.0	51.0	72.0	74.0	35.0	32.0	64.0	28.0	35.3	38.3
**N**	**846**	**847**	**847**	**847**	**847**	**847**	**847**	**847**	**847**	**847**	**847**	**846**	**847**	**847**
**Deft**	60.9	65.3	70.4	76.0	78.2	84.1	77.1	81.9	82.1	76.1	85.0	71.1	80.0	83.7
**Bias**	54.4	59.6	59.5	73.7	87.7	103.3	124.5	153.2	77.8	67.9	128.9	62.2	76.7	81.7

KEY:
4FA = Average over 0.5, 1, 2, and 4 kHz
3FA = Average over 0.5, 1 and 2 kHz
HFA = Average over 4, 6 and 8 kHz
LFA = Average over 0.250, 0.5, and 1 kHz
A123 = Average over 1, 2, and 3 kHz
A124 = Average over 1, 2, and 4 kHz

Mean, standard deviation, confidence intervals, and percentiles for hearing thresholds as a function of ear, age group, socioeconomic group, gender, sample and frequency

TABLE 5 SAMPLE = ALL EAR = BETTER EAR
Age group = 18-80 Occupational group = Overall Gender = Male

	Single Frequency (kHz)								Combined Frequency (kHz)					
	0.250	0.500	1	2	3	4	6	8	4FA	3FA	HFA	LFA	A123	A124
**Mean**	**13.3**	**10.2**	**9.5**	**13.1**	**20.0**	**25.1**	**35.1**	**30.8**	**14.5**	**10.9**	**30.4**	**11.0**	**14.2**	**15.9**
**S.D.**	8.7	9.4	10.1	13.3	17.3	19.7	21.1	23.3	12.2	10.4	20.9	9.1	13.0	13.6
**Lower CI**	12.6	9.5	8.8	12.2	19.0	24.0	33.9	29.6	13.8	10.3	29.3	10.4	13.4	15.1
**Upper CI**	14.0	10.9	10.2	13.9	21.0	26.3	36.4	32.1	15.2	11.6	31.5	11.6	15.0	16.7
**P.05**	0.0	-5.0	-5.0	-5.0	-4.0	-2.0	3.0	-2.0	-1.2	-2.7	2.0	-1.0	-2.7	-1.3
**P.10**	2.0	-1.0	-2.0	-3.0	0.0	1.0	8.0	1.0	0.8	-0.7	5.3	0.7	-0.7	0.3
**P.15**	4.0	0.0	-1.0	-1.0	2.0	3.0	12.0	4.0	2.0	0.3	7.7	2.0	0.7	2.0
**P.20**	5.0	1.0	0.0	1.0	4.0	6.0	14.0	7.0	3.5	1.7	9.7	3.0	2.3	3.0
**P.25**	6.0	3.0	1.0	2.0	5.0	8.0	16.0	10.0	4.2	2.7	11.7	4.0	3.3	4.3
**P.50**	**11.0**	**8.0**	**6.0**	**8.0**	**14.0**	**18.0**	**27.0**	**22.0**	**10.5**	**7.7**	**22.7**	**8.3**	**10.0**	**11.3**
**P.75**	17.0	15.0	14.0	19.0	29.0	38.0	51.0	51.0	20.0	15.3	45.7	14.3	20.0	23.0
**P.80**	20.0	17.0	15.0	22.0	35.0	45.0	57.0	58.0	23.5	17.7	51.7	16.3	23.7	27.0
**P.85**	21.0	20.0	19.0	29.0	42.0	52.0	65.0	66.0	27.8	20.7	59.3	18.7	28.7	31.7
**P.90**	25.0	23.0	24.0	34.0	52.0	60.0	72.0	73.0	33.2	26.0	66.7	22.7	34.3	37.0
**P.95**	35.0	30.0	32.0	47.0	64.0	71.0	85.0	82.0	42.2	34.3	76.0	30.3	45.0	47.3
**N**	**1275**	**1277**	**1277**	**1277**	**1272**	**1277**	**1274**	**1275**	**1277**	**1277**	**1273**	**1275**	**1272**	**1277**
**Deft**	52.7	55.2	59.3	61.9	67.1	69.2	69.3	72.6	68.3	62.6	74.0	58.8	67.3	69.1
**Bias**	6.3	5.6	8.2	7.0	14.1	13.8	18.0	16.5	8.7	6.9	18.9	7.4	11.9	9.7

TABLE 6 SAMPLE = NO NOISE OR ABG>0 EAR = BETTER EAR
Age group = 18-80 Occupational group = Overall Gender = Male

	Single Frequency (kHz)								Combined Frequency (kHz)					
	0.250	0.500	1	2	3	4	6	8	4FA	3FA	HFA	LFA	A123	A124
**Mean**	**11.3**	**7.7**	**6.8**	**9.7**	**15.2**	**20.1**	**30.4**	**26.2**	**11.1**	**8.1**	**25.6**	**8.6**	**10.6**	**12.2**
**S.D.**	6.9	8.3	8.7	11.9	15.2	17.6	20.2	22.5	10.5	8.9	19.4	7.4	11.2	11.8
**Lower CI**	10.3	6.5	5.7	8.4	13.6	18.4	28.4	24.1	10.0	7.0	23.9	7.7	9.4	11.1
**Upper CI**	12.2	8.9	7.9	11.1	16.8	21.7	32.5	28.4	12.1	9.1	27.2	9.5	11.8	13.3
**P.05**	0.0	-6.0	-5.0	-6.0	-7.0	-6.0	-1.0	-6.0	-3.2	-3.3	-2.0	-2.7	-4.0	-3.0
**P.10**	0.0	-4.0	-5.0	-4.0	-4.0	-2.0	5.0	-3.0	-1.8	-2.7	1.3	-1.0	-3.0	-1.3
**P.15**	3.0	-1.0	-3.0	-3.0	-2.0	1.0	7.0	-1.0	-0.2	-1.0	4.0	0.3	-1.7	-0.7
**P.20**	4.0	0.0	-2.0	-2.0	0.0	2.0	9.0	1.0	0.8	-0.3	5.0	1.3	-0.7	0.3
**P.25**	5.0	1.0	-1.0	0.0	1.0	4.0	11.0	2.0	2.0	0.0	7.3	2.0	0.3	1.0
**P.50**	**10.0**	**6.0**	**3.0**	**6.0**	**9.0**	**11.0**	**20.0**	**13.0**	**5.8**	**4.3**	**15.3**	**6.3**	**5.0**	**6.0**
**P.75**	14.0	10.0	9.0	12.0	19.0	22.0	37.0	30.0	13.5	10.0	29.7	10.3	12.7	14.7
**P.80**	16.0	12.0	11.0	15.0	22.0	26.0	43.0	38.0	15.0	11.3	34.0	11.7	14.7	16.7
**P.85**	18.0	14.0	14.0	18.0	26.0	33.0	51.0	51.0	17.5	15.0	45.0	14.0	18.7	20.0
**P.90**	21.0	17.0	17.0	27.0	35.0	44.0	59.0	65.0	24.2	18.7	51.7	16.7	26.0	27.7
**P.95**	24.0	22.0	23.0	36.0	49.0	59.0	72.0	76.0	32.8	25.3	68.7	21.7	33.7	36.0
**N**	**358**	**358**	**358**	**358**	**357**	**358**	**358**	**358**	**358**	**358**	**358**	**358**	**357**	**358**
**Deft**	42.8	50.0	51.2	61.8	67.6	74.4	75.4	78.6	61.4	54.7	78.3	48.0	61.8	64.8
**Bias**	65.1	80.6	109.1	131.1	242.5	340.7	404.6	480.8	165.4	106.9	408.7	87.1	162.4	193.6

KEY: 4FA = Average over 0.5, 1, 2, and 4 kHz; 3FA = Average over 0.5, 1 and 2 kHz
HFA = Average over 4, 6 and 8 kHz; LFA = Average over 0.250, 0.5, and 1 kHz
A123 = Average over 1, 2, and 3 kHz; A124 = Average over 1, 2, and 4 kHz

Mean, standard deviation, confidence intervals, and percentiles for hearing thresholds as a function of ear, age group, socioeconomic group, gender, sample and frequency

TABLE 7 SAMPLE = ALL EAR = BETTER EAR
Age group = 18-80 Occupational group = Non-Manual Gender = Overall

	SINGLE FREQUENCY (kHz)								COMBINED FREQUENCY (kHz)					
	0.250	0.500	1	2	3	4	6	8	4FA	3FA	HFA	LFA	A123	A124
**Mean**	**12.4**	**9.6**	**8.9**	**11.4**	**15.4**	**18.9**	**30.3**	**26.9**	**12.2**	**10.0**	**25.4**	**10.3**	**11.9**	**13.1**
**S.D.**	9.1	10.0	10.7	13.1	15.9	17.8	20.1	22.5	12.0	10.7	19.5	9.5	12.7	13.1
**Lower CI**	11.8	9.0	8.3	10.7	14.6	18.1	29.3	25.7	11.7	9.4	24.5	9.8	11.3	12.5
**Upper CI**	13.1	10.3	9.4	12.1	16.1	19.7	31.4	28.1	12.7	10.5	26.3	10.8	12.5	13.6
**P.05**	0.0	-5.0	-4.0	-4.0	-4.0	-2.0	4.0	-2.0	-1.5	-2.3	2.0	-0.7	-2.7	-1.3
**P.10**	1.0	-1.0	-2.0	-3.0	-1.0	0.0	8.0	1.0	0.2	-0.7	4.7	0.7	-1.0	-0.3
**P.15**	4.0	0.0	-1.0	-1.0	1.0	2.0	10.0	3.0	1.5	0.7	6.3	1.7	0.7	1.0
**P.20**	5.0	1.0	0.0	1.0	2.0	4.0	12.0	6.0	2.5	1.3	8.0	2.7	2.0	2.3
**P.25**	5.0	2.0	1.0	1.0	4.0	5.0	13.0	7.0	3.2	2.3	9.3	3.7	3.0	3.3
**P.50**	**10.0**	**7.0**	**5.0**	**7.0**	**10.0**	**12.0**	**23.0**	**17.0**	**8.0**	**6.3**	**17.3**	**7.3**	**7.7**	**8.7**
**P.75**	16.0	13.0	12.0	16.0	21.0	25.0	40.0	41.0	15.0	12.7	34.3	12.7	15.3	16.7
**P.80**	18.0	15.0	14.0	19.0	24.0	30.0	47.0	49.0	18.0	15.0	41.3	14.7	18.0	19.3
**P.85**	20.0	19.0	17.0	22.0	30.0	37.0	55.0	58.0	23.0	18.7	48.7	17.7	22.3	24.7
**P.90**	24.0	22.0	21.0	30.0	38.0	48.0	65.0	68.0	28.0	23.0	58.7	21.0	28.3	31.0
**P.95**	30.0	30.0	32.0	42.0	50.0	59.0	75.0	75.0	38.0	32.3	68.7	28.7	39.3	41.7
**N**	**1181**	**1185**	**1184**	**1185**	**1182**	**1185**	**1184**	**1183**	**1184**	**1184**	**1183**	**1180**	**1181**	**1184**
**Deft**	68.6	72.4	81.2	84.1	87.0	86.0	80.6	80.0	88.9	84.9	86.0	80.3	89.2	89.6
**Bias**	44.6	51.1	45.1	68.0	79.9	95.5	78.6	74.0	67.3	56.9	83.6	50.3	67.1	71.6

TABLE 8 SAMPLE = NO NOISE OR ABG>0 EAR = BETTER EAR
Age group = 18-80 Occupational group = Non-Manual Gender = Overall

	SINGLE FREQUENCY (kHz)								COMBINED FREQUENCY (kHz)					
	0.250	0.500	1	2	3	4	6	8	4FA	3FA	HFA	LFA	A123	A124
**Mean**	**11.1**	**7.8**	**7.1**	**9.5**	**12.7**	**16.2**	**27.2**	**23.2**	**10.2**	**8.2**	**22.2**	**8.7**	**9.8**	**11.0**
**S.D.**	7.4	8.6	9.2	11.8	13.9	15.9	18.7	21.3	10.4	9.2	17.9	7.8	11.1	11.6
**Lower CI**	10.4	7.0	6.5	8.6	11.7	15.2	25.9	21.8	9.5	7.5	21.1	8.1	9.1	10.3
**Upper CI**	11.8	8.5	7.8	10.4	13.6	17.2	28.5	24.6	10.8	8.8	23.3	9.2	10.5	11.7
**P.05**	0.0	-6.0	-5.0	-5.0	-4.0	-2.0	3.0	-3.0	-1.8	-3.3	1.0	-1.0	-3.0	-2.0
**P.10**	1.0	-3.0	-2.0	-3.0	-2.0	0.0	7.0	0.0	-0.2	-1.7	3.7	0.3	-1.3	-0.7
**P.15**	3.0	0.0	-2.0	-2.0	0.0	1.0	9.0	2.0	0.5	0.0	5.7	1.3	-0.3	0.7
**P.20**	4.0	0.0	0.0	0.0	1.0	3.0	10.0	4.0	1.5	1.0	7.0	2.0	1.0	1.3
**P.25**	5.0	2.0	0.0	1.0	2.0	4.0	12.0	6.0	2.5	1.7	8.0	3.0	2.0	2.3
**P.50**	**10.0**	**6.0**	**5.0**	**6.0**	**9.0**	**10.0**	**20.0**	**13.0**	**6.5**	**5.0**	**14.7**	**6.0**	**5.3**	**6.7**
**P.75**	15.0	11.0	10.0	13.0	16.0	20.0	34.0	32.0	12.5	10.0	26.7	10.7	12.7	13.3
**P.80**	16.0	13.0	12.0	16.0	19.0	23.0	39.0	40.0	14.8	11.7	32.7	12.7	14.3	16.0
**P.85**	19.0	15.0	14.0	19.0	24.0	28.0	48.0	51.0	17.2	15.3	41.3	15.0	17.7	18.7
**P.90**	21.0	20.0	18.0	25.0	31.0	37.0	56.0	61.0	23.5	19.7	49.3	18.3	23.7	25.7
**P.95**	25.0	23.0	25.0	36.0	42.0	51.0	72.0	74.0	31.2	26.0	65.3	24.3	31.3	34.7
**N**	**661**	**662**	**662**	**662**	**661**	**662**	**662**	**662**	**662**	**662**	**662**	**661**	**661**	**662**
**Deft**	56.6	62.4	73.0	81.0	86.1	90.3	81.8	80.3	83.1	76.0	86.9	68.7	84.3	87.0
**Bias**	39.9	43.9	40.1	63.7	106.5	161.2	133.2	127.3	81.8	53.7	142.7	47.3	74.5	91.9

KEY: 4FA = Average over 0.5, 1, 2, and 4 kHz
3FA = Average over 0.5, 1 and 2 kHz
HFA = Average over 4, 6 and 8 kHz
LFA = Average over 0.250, 0.5, and 1 kHz
A123 = Average over 1, 2, and 3 kHz
A124 = Average over 1, 2, and 4 kHz

Mean, standard deviation, confidence intervals, and percentiles for hearing thresholds as a function of ear, age group, socioeconomic group, gender, sample and frequency

TABLE 9 SAMPLE = ALL — Age group = 18-80 — Occupational group = Non-Manual — EAR = BETTER EAR — Gender = Female

	Single Frequency (kHz)								Combined Frequency (kHz)					
	0.250	0.500	1	2	3	4	6	8	4FA	3FA	HFA	LFA	A123	A124
**Mean**	**12.9**	**10.3**	**9.7**	**11.7**	**14.7**	**17.2**	**28.9**	**25.2**	**12.2**	**10.6**	**23.8**	**11.0**	**12.0**	**12.9**
**S.D.**	9.8	10.8	11.4	13.3	14.8	16.4	19.1	21.3	12.3	11.4	18.4	10.2	12.7	13.1
**Lower CI**	12.1	9.5	9.0	10.9	13.8	16.3	27.6	23.9	11.6	9.9	22.7	10.3	11.3	12.2
**Upper CI**	13.7	11.0	10.4	12.5	15.5	18.1	30.1	26.6	12.9	11.2	24.8	11.6	12.7	13.6
**P.05**	0.0	-5.0	-3.0	-4.0	-3.0	-2.0	3.0	-2.0	-1.0	-2.0	1.3	-0.3	-2.0	-1.3
**P.10**	1.0	-1.0	-1.0	-2.0	-1.0	0.0	7.0	2.0	0.2	0.0	4.7	1.0	0.0	0.0
**P.15**	4.0	0.0	0.0	0.0	1.0	2.0	9.0	4.0	1.5	1.0	6.0	2.3	1.0	1.3
**P.20**	5.0	1.0	0.0	1.0	2.0	4.0	10.0	6.0	2.5	1.7	7.7	3.0	2.0	2.3
**P.25**	5.0	2.0	2.0	2.0	3.0	5.0	12.0	7.0	3.5	2.7	9.0	4.0	3.0	3.3
**P.50**	**10.0**	**7.0**	**6.0**	**7.0**	**9.0**	**10.0**	**22.0**	**16.0**	**7.8**	**6.7**	**16.0**	**7.3**	**7.7**	**8.0**
**P.75**	17.0	14.0	12.0	15.0	20.0	22.0	37.0	38.0	14.8	12.7	31.7	13.3	15.0	16.0
**P.80**	19.0	16.0	15.0	18.0	23.0	27.0	43.0	45.0	16.8	15.0	36.3	15.7	17.0	18.3
**P.85**	22.0	20.0	18.0	22.0	27.0	33.0	53.0	54.0	21.8	19.3	44.3	19.0	21.7	22.7
**P.90**	26.0	22.0	21.0	28.0	35.0	42.0	63.0	65.0	28.2	22.7	56.0	22.3	28.0	30.3
**P.95**	31.0	31.0	34.0	42.0	47.0	56.0	75.0	75.0	40.0	35.3	67.7	31.3	39.3	44.0
**N**	**669**	**671**	**670**	**671**	**671**	**671**	**671**	**670**	**670**	**670**	**670**	**668**	**670**	**670**
**Deft**	69.1	74.0	81.6	84.1	83.0	82.9	75.8	76.2	88.0	85.5	81.5	80.8	87.5	88.3
**Bias**	29.8	59.1	54.6	71.7	86.8	93.3	82.6	72.1	73.8	65.5	83.5	52.1	74.8	76.9

TABLE 10 SAMPLE = NO NOISE OR ABG>0 — Age group = 18-80 — Occupational group = Non-Manual — EAR = BETTER EAR — Gender = Female

	Single Frequency (kHz)								Combined Frequency (kHz)					
	0.250	0.500	1	2	3	4	6	8	4FA	3FA	HFA	LFA	A123	A124
**Mean**	**11.5**	**8.5**	**8.0**	**10.0**	**12.8**	**15.3**	**26.7**	**22.9**	**10.5**	**8.9**	**21.6**	**9.3**	**10.3**	**11.1**
**S.D.**	8.3	9.6	10.0	12.2	13.7	15.4	17.9	20.2	11.1	10.1	17.2	8.8	11.5	11.9
**Lower CI**	10.7	7.7	7.3	9.1	11.9	14.2	25.3	21.5	9.7	8.1	20.5	8.6	9.5	10.3
**Upper CI**	12.3	9.3	8.8	10.9	13.8	16.3	28.1	24.4	11.2	9.6	22.7	10.0	11.1	11.9
**P.05**	0.0	-6.0	-4.0	-4.0	-2.0	-2.0	3.0	-2.0	-1.0	-2.0	1.0	-0.7	-1.7	-1.3
**P.10**	1.0	-3.0	-2.0	-3.0	-1.0	0.0	7.0	1.0	0.0	-0.7	4.7	1.0	-0.3	-0.3
**P.15**	3.0	0.0	0.0	-1.0	1.0	1.0	9.0	3.0	0.8	0.3	6.0	1.7	1.0	1.3
**P.20**	4.0	1.0	0.0	1.0	2.0	3.0	10.0	5.0	2.0	1.3	7.3	2.7	1.7	2.3
**P.25**	5.0	2.0	1.0	1.0	2.0	5.0	12.0	7.0	3.0	2.3	8.7	3.7	2.7	3.0
**P.50**	**10.0**	**6.0**	**5.0**	**6.0**	**9.0**	**10.0**	**21.0**	**14.0**	**7.2**	**6.0**	**14.7**	**6.3**	**6.7**	**7.3**
**P.75**	15.0	12.0	10.0	14.0	16.0	19.0	33.0	33.0	12.5	10.7	26.3	11.0	13.0	14.0
**P.80**	16.0	15.0	12.0	16.0	19.0	22.0	37.0	41.0	14.8	12.7	32.7	13.3	14.7	16.0
**P.85**	20.0	16.0	15.0	19.0	24.0	28.0	45.0	50.0	17.2	15.0	39.3	15.7	17.7	18.3
**P.90**	22.0	20.0	19.0	24.0	31.0	36.0	56.0	60.0	23.5	20.0	48.7	19.0	23.3	24.7
**P.95**	26.0	27.0	26.0	37.0	41.0	52.0	73.0	73.0	32.2	27.7	65.3	25.7	32.3	35.0
**N**	**457**	**458**	**458**	**458**	**458**	**458**	**458**	**458**	**458**	**458**	**458**	**457**	**458**	**458**
**Deft**	61.5	67.0	75.9	82.4	85.2	86.2	75.1	73.7	86.1	80.6	81.4	74.8	86.9	88.5
**Bias**	22.5	46.8	31.3	43.7	69.6	73.6	55.4	46.2	55.7	47.3	61.8	40.2	53.8	54.9

KEY:
4FA = Average over 0.5, 1, 2, and 4 kHz
3FA = Average over 0.5, 1 and 2 kHz
HFA = Average over 4, 6 and 8 kHz
LFA = Average over 0.250, 0.5, and 1 kHz
A123 = Average over 1, 2, and 3 kHz
A124 = Average over 1, 2, and 4 kHz

Mean, standard deviation, confidence intervals, and percentiles for hearing thresholds as a function of ear, age group, socioeconomic group, gender, sample and frequency

TABLE 11 SAMPLE = ALL EAR = BETTER EAR
Age group = 18-80 Occupational group = Non-Manual Gender = Male

	SINGLE FREQUENCY (kHz)								COMBINED FREQUENCY (kHz)					
	0.250	0.500	1	2	3	4	6	8	4FA	3FA	HFA	LFA	A123	A124
**Mean**	**12.0**	**8.9**	**7.9**	**11.1**	**16.1**	**20.8**	**32.0**	**28.8**	**12.2**	**9.3**	**27.2**	**9.6**	**11.7**	**13.3**
**S.D.**	8.2	9.0	9.8	12.9	17.1	19.1	21.0	23.6	11.7	9.9	20.6	8.5	12.6	13.1
**Lower CI**	10.9	7.8	7.1	9.9	14.8	19.5	30.4	26.7	11.4	8.4	25.8	8.7	10.8	12.4
**Upper CI**	13.0	9.9	8.8	12.3	17.4	22.2	33.6	30.9	13.0	10.2	28.6	10.4	12.7	14.2
**P.05**	0.0	-6.0	-5.0	-4.0	-5.0	-2.0	5.0	-3.0	-2.0	-3.3	3.0	-1.0	-3.0	-1.3
**P.10**	1.0	-2.0	-2.0	-3.0	-2.0	0.0	9.0	1.0	0.5	-1.0	5.0	0.3	-1.3	-0.7
**P.15**	4.0	0.0	-1.0	-1.0	0.0	3.0	10.0	3.0	1.5	0.0	7.3	1.3	-0.3	1.0
**P.20**	4.0	1.0	-1.0	0.0	3.0	4.0	13.0	7.0	2.2	1.0	9.0	2.0	1.3	2.3
**P.25**	5.0	2.0	0.0	1.0	4.0	6.0	15.0	8.0	3.0	1.7	9.7	3.0	2.7	3.0
**P.50**	**10.0**	**6.0**	**5.0**	**6.0**	**11.0**	**15.0**	**25.0**	**21.0**	**8.2**	**5.7**	**19.0**	**7.3**	**7.7**	**9.0**
**P.75**	16.0	12.0	11.0	16.0	23.0	29.0	45.0	46.0	16.0	12.3	39.7	12.3	16.0	18.0
**P.80**	17.0	15.0	13.0	19.0	28.0	35.0	51.0	55.0	19.5	15.3	46.3	13.7	19.3	22.3
**P.85**	19.0	18.0	16.0	23.0	34.0	43.0	56.0	62.0	24.0	18.3	51.7	16.7	23.7	27.0
**P.90**	22.0	21.0	20.0	31.0	41.0	52.0	65.0	69.0	28.0	23.0	60.0	19.3	29.7	32.3
**P.95**	26.0	25.0	29.0	42.0	53.0	63.0	76.0	77.0	35.8	29.0	69.3	25.3	37.7	40.7
**N**	**512**	**514**	**514**	**514**	**511**	**514**	**513**	**513**	**514**	**514**	**513**	**512**	**511**	**514**
**Deft**	71.5	73.2	83.2	84.5	89.1	86.8	84.5	82.6	91.3	86.6	89.2	83.5	91.5	91.3
**Bias**	77.2	55.4	50.0	66.4	52.5	61.1	43.2	40.9	58.2	57.3	49.4	62.9	57.9	59.2

TABLE 12 SAMPLE = NO NOISE OR ABG>0 EAR = BETTER EAR
Age group = 18-80 Occupational group = Non-Manual Gender = Male

	SINGLE FREQUENCY (kHz)								COMBINED FREQUENCY (kHz)					
	0.250	0.500	1	2	3	4	6	8	4FA	3FA	HFA	LFA	A123	A124
**Mean**	**10.7**	**6.9**	**6.0**	**8.9**	**12.4**	**17.2**	**27.8**	**23.4**	**9.8**	**7.3**	**22.8**	**7.9**	**9.1**	**10.7**
**S.D.**	5.9	7.1	8.0	11.3	14.0	16.4	19.6	22.4	9.5	7.9	18.6	6.2	10.5	11.0
**Lower CI**	9.5	5.6	5.0	7.2	10.8	15.3	25.5	20.9	8.7	6.2	20.9	6.9	7.9	9.5
**Upper CI**	11.9	8.2	7.0	10.6	14.0	19.1	30.1	26.0	10.8	8.3	24.8	8.8	10.4	12.0
**P.05**	0.0	-9.0	-5.0	-5.0	-8.0	-4.0	1.0	-4.0	-3.2	-3.3	1.0	-1.0	-4.3	-3.0
**P.10**	0.0	-4.0	-4.0	-4.0	-4.0	0.0	6.0	-1.0	-1.8	-2.3	2.3	-0.3	-3.0	-1.0
**P.15**	1.0	-1.0	-2.0	-3.0	-2.0	1.0	9.0	1.0	-0.2	-1.7	4.3	0.3	-2.3	-0.7
**P.20**	4.0	0.0	-2.0	-2.0	0.0	3.0	9.0	2.0	0.8	0.0	6.0	1.3	-1.0	0.3
**P.25**	5.0	1.0	-1.0	-1.0	1.0	3.0	12.0	4.0	1.5	0.3	7.3	2.0	-0.3	1.0
**P.50**	**10.0**	**5.0**	**3.0**	**5.0**	**8.0**	**8.0**	**20.0**	**12.0**	**5.5**	**3.3**	**14.3**	**5.7**	**3.7**	**4.7**
**P.75**	14.0	10.0	9.0	12.0	16.0	21.0	36.0	29.0	12.0	9.3	29.7	10.0	12.0	12.7
**P.80**	16.0	11.0	10.0	15.0	19.0	25.0	40.0	37.0	14.8	10.3	33.0	10.7	13.0	16.0
**P.85**	16.0	14.0	13.0	18.0	24.0	31.0	50.0	52.0	17.2	16.0	45.0	13.7	17.3	19.3
**P.90**	20.0	17.0	16.0	25.0	31.0	41.0	57.0	65.0	24.2	18.7	51.3	16.7	25.0	27.0
**P.95**	22.0	21.0	23.0	35.0	42.0	49.0	70.0	75.0	29.5	24.3	65.7	18.7	30.7	33.0
**N**	**204**	**204**	**204**	**204**	**203**	**204**	**204**	**204**	**204**	**204**	**204**	**204**	**203**	**204**
**Deft**	58.1	65.0	78.5	81.0	85.3	91.9	88.2	85.6	85.0	77.7	91.1	71.2	83.5	87.9
**Bias**	96.3	90.1	122.3	133.0	188.1	248.5	235.6	249.2	148.5	115.2	244.4	106.9	149.3	167.9

KEY: 4FA = Average over 0.5, 1, 2, and 4 kHz; 3FA = Average over 0.5, 1 and 2 kHz
HFA = Average over 4, 6 and 8 kHz; LFA = Average over 0.250, 0.5, and 1 kHz
A123 = Average over 1, 2, and 3 kHz; A124 = Average over 1, 2, and 4 kHz

Mean, standard deviation, confidence intervals, and percentiles for hearing thresholds as a function of ear, age group, socioeconomic group, gender, sample and frequency

TABLE 13 SAMPLE = ALL — EAR = BETTER EAR
Age group = 18-80 — Occupational group = Manual — Gender = Overall

	SINGLE FREQUENCY (kHz)								COMBINED FREQUENCY (kHz)					
	0.250	0.500	1	2	3	4	6	8	4FA	3FA	HFA	LFA	A123	A124
**Mean**	**14.9**	**12.1**	**11.1**	**13.5**	**18.9**	**23.2**	**33.5**	**29.2**	**15.0**	**12.2**	**28.6**	**12.7**	**14.5**	**15.9**
S.D.	10.5	11.3	11.5	13.5	16.4	18.9	20.4	22.6	12.8	11.6	20.1	10.8	13.1	13.7
Lower CI	14.2	11.4	10.5	12.7	17.9	22.2	32.3	28.0	14.3	11.6	27.6	12.1	13.8	15.2
Upper CI	15.5	12.8	11.8	14.3	19.9	24.2	34.7	30.3	15.6	12.9	29.6	13.3	15.2	16.6
P.05	0.0	-3.0	-3.0	-4.0	-3.0	-2.0	5.0	-2.0	-0.2	-1.0	2.7	-0.3	-1.3	-0.7
P.10	3.0	0.0	-1.0	-1.0	0.0	2.0	9.0	2.0	1.8	0.7	6.3	2.0	0.3	1.3
P.15	5.0	1.0	0.0	1.0	3.0	5.0	12.0	5.0	3.2	2.0	8.3	3.3	2.0	2.7
P.20	6.0	3.0	1.0	2.0	5.0	6.0	15.0	8.0	4.5	3.0	10.3	4.3	3.3	4.3
P.25	7.0	5.0	3.0	3.0	6.0	8.0	17.0	10.0	5.5	4.0	12.7	5.3	4.7	5.3
**P.50**	**13.0**	**10.0**	**8.0**	**10.0**	**14.0**	**18.0**	**27.0**	**22.0**	**11.8**	**9.0**	**22.7**	**10.0**	**11.0**	**12.3**
P.75	20.0	16.0	15.0	20.0	29.0	35.0	50.0	48.0	21.2	17.0	43.7	16.7	21.0	23.0
P.80	21.0	20.0	18.0	25.0	32.0	41.0	56.0	55.0	24.2	19.7	50.3	19.0	24.3	27.0
P.85	25.0	23.0	22.0	29.0	40.0	50.0	63.0	63.0	28.8	23.7	56.7	22.0	29.7	32.3
P.90	30.0	29.0	28.0	37.0	50.0	58.0	71.0	72.0	35.0	29.3	65.7	28.0	35.7	38.0
P.95	41.0	41.0	40.0	49.0	63.0	71.0	82.0	82.0	46.0	41.3	75.7	40.3	47.7	49.3
**N**	**1474**	**1476**	**1475**	**1476**	**1473**	**1475**	**1472**	**1474**	**1475**	**1475**	**1471**	**1474**	**1472**	**1475**
Deft	60.5	62.4	62.9	62.6	64.7	69.4	68.1	72.2	68.4	65.3	72.7	64.3	66.0	68.3
Bias	-49.8	-56.8	-56.6	-84.4	-104.6	-114.3	-108.4	-130.1	-77.4	-65.1	-115.3	-54.0	-80.0	-84.7

TABLE 14 SAMPLE = NO NOISE OR ABG>0 — EAR = BETTER EAR
Age group = 18-80 — Occupational group = Manual — Gender = Overall

	SINGLE FREQUENCY (kHz)								COMBINED FREQUENCY (kHz)					
	0.250	0.500	1	2	3	4	6	8	4FA	3FA	HFA	LFA	A123	A124
**Mean**	**12.8**	**9.6**	**8.4**	**10.2**	**15.2**	**18.5**	**29.9**	**25.8**	**11.7**	**9.4**	**24.8**	**10.3**	**11.3**	**12.4**
S.D.	8.7	9.9	9.2	11.7	14.9	16.9	19.6	22.3	10.7	9.6	18.6	8.7	11.1	11.6
Lower CI	12.0	8.7	7.5	9.1	14.1	17.2	27.9	24.4	10.8	8.6	23.4	9.6	10.4	11.4
Upper CI	13.6	10.5	9.4	11.3	16.4	19.9	31.9	27.2	12.6	10.3	26.1	11.0	12.1	13.3
P.05	0.0	-4.0	-5.0	-6.0	-6.0	-5.0	2.0	-4.0	-1.5	-1.7	-1.0	-1.3	-3.0	-2.0
P.10	2.0	-1.0	-2.0	-3.0	-2.0	-2.0	5.0	-1.0	0.5	-0.7	2.7	0.7	-0.7	-0.7
P.15	4.0	0.0	-1.0	0.0	0.0	2.0	10.0	2.0	1.8	0.0	5.3	2.3	0.3	1.0
P.20	5.0	2.0	0.0	1.0	2.0	4.0	11.0	4.0	2.5	1.3	7.7	3.3	1.3	2.3
P.25	6.0	3.0	1.0	2.0	3.0	5.0	13.0	7.0	3.8	2.7	8.7	4.0	3.0	3.0
**P.50**	**11.0**	**8.0**	**6.0**	**7.0**	**10.0**	**13.0**	**23.0**	**17.0**	**8.2**	**7.0**	**17.0**	**8.0**	**8.0**	**9.0**
P.75	16.0	13.0	12.0	14.0	19.0	23.0	37.0	35.0	15.0	12.3	33.0	12.7	14.7	15.7
P.80	18.0	15.0	14.0	17.0	23.0	26.0	45.0	43.0	18.0	15.0	36.0	14.7	16.7	19.0
P.85	21.0	19.0	16.0	20.0	27.0	33.0	53.0	52.0	20.2	17.3	43.3	17.3	20.0	21.7
P.90	23.0	23.0	20.0	27.0	34.0	40.0	58.0	60.0	25.8	20.7	51.7	20.7	26.0	28.0
P.95	34.0	35.0	32.0	39.0	50.0	56.0	71.0	77.0	38.0	33.0	66.7	30.7	38.7	41.0
**N**	**542**	**542**	**542**	**542**	**542**	**542**	**542**	**542**	**542**	**542**	**542**	**542**	**542**	**542**
Deft	55.7	59.1	49.4	61.9	70.5	76.0	76.9	80.7	60.8	56.1	78.9	54.0	59.8	62.3
Bias	0.5	-35.4	-30.6	-22.1	93.8	146.7	185.5	193.5	14.6	-29.4	175.3	-21.8	13.7	31.3

KEY:
4FA = Average over 0.5, 1, 2, and 4 kHz
3FA = Average over 0.5, 1 and 2 kHz
HFA = Average over 4, 6 and 8 kHz
LFA = Average over 0.250, 0.5, and 1 kHz
A123 = Average over 1, 2, and 3 kHz
A124 = Average over 1, 2, and 4 kHz

Mean, standard deviation, confidence intervals, and percentiles for hearing thresholds as a function of ear, age group, socioeconomic group, gender, sample and frequency

TABLE 15 SAMPLE = ALL — EAR = BETTER EAR
Age group = 18-80 — Occupational group = Manual — Gender = Female

	SINGLE FREQUENCY (kHz)								COMBINED FREQUENCY (kHz)					
	0.250	0.500	1	2	3	4	6	8	4FA	3FA	HFA	LFA	A123	A124
**Mean**	**15.2**	**12.9**	**11.6**	**12.6**	**15.6**	**18.6**	**30.1**	**26.4**	**13.9**	**12.4**	**25.0**	**13.2**	**13.3**	**14.3**
**S.D.**	11.3	12.2	12.2	13.1	14.0	15.6	17.9	20.8	12.6	12.1	17.5	11.6	12.7	13.1
**Lower CI**	14.3	12.0	10.8	11.7	14.5	17.4	28.7	24.9	13.2	11.6	23.8	12.5	12.4	13.5
**Upper CI**	16.0	13.8	12.5	13.5	16.7	19.7	31.6	27.9	14.7	13.1	26.2	14.0	14.1	15.1
**P.05**	0.0	-1.0	-2.0	-3.0	-5.0	-2.0	5.0	0.0	0.5	-0.7	3.3	0.7	-1.3	0.0
**P.10**	4.0	1.0	0.0	0.0	-1.0	1.0	10.0	3.0	2.0	1.3	6.7	2.7	0.7	1.3
**P.15**	5.0	2.0	0.0	1.0	2.0	5.0	12.0	6.0	3.2	2.3	8.3	4.0	2.3	2.7
**P.20**	6.0	4.0	2.0	3.0	4.0	6.0	14.0	8.0	4.2	3.7	9.7	4.7	3.3	4.0
**P.25**	8.0	5.0	3.0	4.0	6.0	7.0	16.0	10.0	5.2	4.3	11.7	5.7	4.7	5.0
**P.50**	**13.0**	**10.0**	**9.0**	**9.0**	**13.0**	**15.0**	**26.0**	**21.0**	**10.8**	**9.0**	**20.3**	**10.0**	**10.3**	**11.0**
**P.75**	21.0	18.0	16.0	20.0	23.0	27.0	42.0	42.0	19.2	17.0	36.0	17.3	19.3	20.7
**P.80**	22.0	21.0	20.0	23.0	27.0	32.0	50.0	51.0	22.2	19.7	41.3	20.0	21.7	24.0
**P.85**	25.0	25.0	23.0	27.0	31.0	38.0	56.0	57.0	27.2	24.7	50.0	24.0	27.0	27.7
**P.90**	34.0	32.0	30.0	34.0	39.0	45.0	63.0	66.0	33.5	31.3	56.3	30.7	33.3	34.7
**P.95**	43.0	44.0	43.0	44.0	51.0	56.0	75.0	78.0	44.2	41.7	67.0	43.0	44.0	46.0
**N**	**714**	**716**	**715**	**716**	**715**	**715**	**714**	**715**	**715**	**715**	**714**	**714**	**714**	**715**
**Deft**	68.7	68.8	70.5	71.3	68.1	70.8	67.2	70.9	75.9	74.4	72.5	72.4	73.4	75.1
**Bias**	-71.0	-81.3	-71.2	-93.4	-87.9	-105.8	-126.6	-186.1	-86.7	-80.3	-138.9	-73.6	-81.9	-89.3

TABLE 16 SAMPLE = NO NOISE OR ABG>0 — EAR = BETTER EAR
Age group = 18-80 — Occupational group = Manual — Gender = Female

	SINGLE FREQUENCY (kHz)								COMBINED FREQUENCY (kHz)					
	0.250	0.500	1	2	3	4	6	8	4FA	3FA	HFA	LFA	A123	A124
**Mean**	**13.7**	**10.8**	**9.5**	**10.6**	**13.6**	**15.9**	**28.7**	**24.8**	**11.7**	**10.3**	**23.1**	**11.3**	**11.2**	**12.0**
**S.D.**	8.8	8.9	8.5	10.1	12.2	13.7	17.4	20.9	9.6	8.7	16.4	8.2	9.8	10.2
**Lower CI**	12.8	9.8	8.5	9.5	12.2	14.5	26.9	23.0	10.8	9.4	21.7	10.5	10.2	11.0
**Upper CI**	14.7	11.8	10.4	11.8	14.9	17.3	30.5	26.5	12.6	11.2	24.5	12.2	12.3	13.0
**P.05**	0.0	-2.0	-2.0	-3.0	-6.0	-4.0	5.0	-1.0	-0.5	-1.0	1.7	-0.3	-2.7	-0.7
**P.10**	3.0	0.0	0.0	-1.0	-1.0	-1.0	9.0	2.0	1.5	0.7	4.7	2.3	0.0	1.0
**P.15**	4.0	2.0	0.0	1.0	1.0	3.0	11.0	4.0	2.5	1.3	7.7	3.0	1.7	1.7
**P.20**	5.0	3.0	2.0	2.0	3.0	5.0	12.0	7.0	3.2	3.0	8.7	4.0	3.0	2.7
**P.25**	7.0	4.0	3.0	3.0	4.0	6.0	13.0	9.0	4.2	3.7	9.7	5.0	3.7	4.0
**P.50**	**11.0**	**9.0**	**8.0**	**7.0**	**11.0**	**12.0**	**24.0**	**17.0**	**9.0**	**7.7**	**17.0**	**9.0**	**8.3**	**9.0**
**P.75**	18.0	15.0	13.0	15.0	19.0	22.0	37.0	37.0	16.0	14.0	33.0	14.0	15.3	16.3
**P.80**	19.0	16.0	15.0	19.0	22.0	26.0	43.0	45.0	18.5	16.3	36.0	16.0	17.3	19.3
**P.85**	21.0	20.0	18.0	21.0	25.0	30.0	52.0	53.0	20.8	18.0	41.3	19.7	20.0	21.7
**P.90**	24.0	25.0	24.0	27.0	30.0	40.0	57.0	60.0	25.8	22.0	51.3	23.7	24.7	25.7
**P.95**	35.0	35.0	33.0	40.0	47.0	51.0	67.0	76.0	38.0	34.3	62.3	31.0	39.7	40.0
**N**	**388**	**388**	**388**	**388**	**388**	**388**	**388**	**388**	**388**	**388**	**388**	**388**	**388**	**388**
**Deft**	64.9	58.2	51.0	58.5	62.7	67.6	73.3	78.3	61.6	57.4	75.4	58.3	59.0	61.5
**Bias**	43.0	-10.8	-19.0	-18.3	-4.1	-11.8	38.3	-5.9	-15.0	-16.0	6.9	4.4	-13.8	-16.4

KEY: 4FA = Average over 0.5, 1, 2, and 4 kHz
3FA = Average over 0.5, 1 and 2 kHz
HFA = Average over 4, 6 and 8 kHz
LFA = Average over 0.250, 0.5, and 1 kHz
A123 = Average over 1, 2, and 3 kHz
A124 = Average over 1, 2, and 4 kHz

Mean, standard deviation, confidence intervals, and percentiles for hearing thresholds as a function of ear, age group, socioeconomic group, gender, sample and frequency

TABLE 17 SAMPLE = ALL — EAR = BETTER EAR

Age group = 18-80 — Occupational group = Manual — Gender = Male

	Single Frequency (kHz)								Combined Frequency (kHz)					
	0.250	0.500	1	2	3	4	6	8	4FA	3FA	HFA	LFA	A123	A124
**Mean**	**14.5**	**11.2**	**10.6**	**14.5**	**22.6**	**28.4**	**37.3**	**32.3**	**16.2**	**12.1**	**32.7**	**12.1**	**15.9**	**17.8**
**S.D.**	9.6	10.3	10.7	14.0	18.0	20.9	22.4	24.2	12.8	11.0	22.0	9.8	13.5	14.2
**Lower CI**	13.5	10.1	9.5	13.2	21.0	26.6	35.4	30.6	15.1	11.0	31.0	11.1	14.7	16.6
**Upper CI**	15.6	12.3	11.6	15.8	24.2	30.1	39.2	34.0	17.2	13.1	34.3	13.1	17.1	19.0
**P.05**	0.0	-4.0	-4.0	-5.0	-1.0	-3.0	3.0	-2.0	-0.8	-2.3	0.3	-1.0	-1.7	-1.0
**P.10**	3.0	0.0	-1.0	-2.0	1.0	2.0	8.0	2.0	1.2	-0.3	5.7	1.0	0.3	1.0
**P.15**	4.0	1.0	0.0	0.0	3.0	6.0	12.0	5.0	3.5	1.0	8.3	3.0	2.0	2.7
**P.20**	6.0	2.0	1.0	1.0	5.0	8.0	15.0	8.0	4.5	2.3	11.0	4.0	3.3	4.3
**P.25**	7.0	4.0	3.0	3.0	7.0	10.0	18.0	11.0	5.5	4.0	14.3	5.0	4.7	5.7
**P.50**	**13.0**	**10.0**	**8.0**	**10.0**	**17.0**	**22.0**	**30.0**	**24.0**	**13.2**	**9.3**	**24.3**	**9.7**	**12.0**	**14.3**
**P.75**	20.0	16.0	15.0	21.0	33.0	45.0	56.0	54.0	23.0	17.0	50.7	16.0	22.7	26.3
**P.80**	21.0	18.0	18.0	26.0	41.0	51.0	63.0	61.0	27.2	19.7	56.7	17.7	27.0	31.0
**P.85**	23.0	21.0	20.0	31.0	49.0	59.0	70.0	67.0	29.8	23.3	64.3	20.7	32.3	35.0
**P.90**	29.0	25.0	26.0	39.0	58.0	65.0	77.0	75.0	36.8	28.3	71.7	25.7	38.3	41.3
**P.95**	38.0	36.0	35.0	56.0	71.0	77.0	88.0	84.0	47.5	40.3	79.3	35.7	50.7	52.7
**N**	**760**	**760**	**760**	**760**	**758**	**760**	**758**	**759**	**760**	**760**	**757**	**760**	**758**	**760**
**Deft**	50.8	53.9	54.4	56.8	62.7	67.9	68.9	73.2	62.1	56.5	72.8	54.6	60.7	63.4
**Bias**	-32.8	-45.4	-49.2	-62.3	-70.9	-52.1	-39.9	-35.7	-52.2	-52.3	-39.2	-42.4	-59.2	-54.5

TABLE 18 SAMPLE = NO NOISE OR ABG>0 — EAR = BETTER EAR

Age group = 18-80 — Occupational group = Manual — Gender = Male

	Single Frequency (kHz)								Combined Frequency (kHz)					
	0.250	0.500	1	2	3	4	6	8	4FA	3FA	HFA	LFA	A123	A124
**Mean**	**11.6**	**8.1**	**7.0**	**9.5**	**17.4**	**21.9**	**31.8**	**27.3**	**11.6**	**8.2**	**27.0**	**8.9**	**11.3**	**12.8**
**S.D.**	8.6	10.7	9.9	13.4	17.5	19.8	22.2	24.1	12.0	10.5	21.1	9.1	12.4	13.2
**Lower CI**	10.1	6.6	5.2	7.5	15.4	19.4	28.0	25.0	10.1	6.7	24.6	7.7	9.8	11.1
**Upper CI**	13.0	9.5	8.7	11.5	19.4	24.4	35.5	29.7	13.2	9.7	29.4	10.1	12.8	14.5
**P.05**	-1.0	-5.0	-6.0	-6.0	-7.0	-6.0	-3.0	-6.0	-3.8	-3.3	-2.3	-3.0	-3.7	-3.3
**P.10**	0.0	-4.0	-5.0	-5.0	-3.0	-3.0	3.0	-3.0	-1.8	-2.7	-1.0	-1.0	-2.0	-2.7
**P.15**	3.0	-1.0	-3.0	-4.0	-1.0	1.0	5.0	-2.0	0.2	-1.0	1.3	-0.3	-0.7	-0.7
**P.20**	4.0	0.0	-2.0	-2.0	0.0	2.0	8.0	-1.0	1.0	-0.3	5.0	1.3	-0.3	0.0
**P.25**	5.0	1.0	-1.0	0.0	2.0	5.0	11.0	2.0	2.0	0.0	6.3	2.3	0.7	2.3
**P.50**	**10.0**	**6.0**	**4.0**	**6.0**	**9.0**	**13.0**	**20.0**	**15.0**	**6.8**	**5.0**	**16.7**	**7.0**	**7.0**	**8.7**
**P.75**	15.0	11.0	10.0	11.0	22.0	26.0	37.0	32.0	14.2	10.7	33.3	11.0	13.3	15.0
**P.80**	16.0	12.0	12.0	15.0	25.0	31.0	49.0	39.0	16.0	11.7	37.0	11.7	16.0	17.3
**P.85**	18.0	14.0	15.0	18.0	30.0	35.0	54.0	48.0	18.8	14.3	45.0	14.7	20.7	21.0
**P.90**	23.0	16.0	18.0	29.0	44.0	50.0	63.0	61.0	26.2	19.3	55.0	17.0	26.7	31.7
**P.95**	33.0	31.0	23.0	37.0	64.0	72.0	77.0	81.0	37.2	28.3	76.3	30.3	34.3	41.3
**N**	**154**	**154**	**154**	**154**	**154**	**154**	**154**	**154**	**154**	**154**	**154**	**154**	**154**	**154**
**Deft**	42.4	53.0	42.2	57.2	65.0	69.1	73.2	80.0	53.5	48.5	73.5	44.1	53.6	55.7
**Bias**	-19.3	-4.7	11.3	-1.9	175.1	275.6	415.3	530.1	70.1	1.6	407.0	-4.2	61.5	95.0

KEY:
4FA = Average over 0.5, 1, 2, and 4 kHz
3FA = Average over 0.5, 1 and 2 kHz
HFA = Average over 4, 6 and 8 kHz
LFA = Average over 0.250, 0.5, and 1 kHz
A123 = Average over 1, 2, and 3 kHz
A124 = Average over 1, 2, and 4 kHz

Mean, standard deviation, confidence intervals, and percentiles for hearing thresholds as a function of ear, age group, socioeconomic group, gender, sample and frequency

TABLE 19 SAMPLE = ALL — EAR = BETTER EAR
Age group = 18-30 — Occupational group = Overall — Gender = Overall

	Single Frequency (kHz)								Combined Frequency (kHz)					
	0.250	0.500	1	2	3	4	6	8	4FA	3FA	HFA	LFA	A123	A124
**Mean**	**8.6**	**5.4**	**4.1**	**4.2**	**5.8**	**7.2**	**15.4**	**9.7**	**5.2**	**4.5**	**10.7**	**6.0**	**4.7**	**5.2**
**S.D.**	4.4	4.8	4.4	4.6	5.8	6.1	7.1	7.0	4.1	4.0	5.9	4.0	4.3	4.3
**Lower CI**	7.7	4.5	3.4	3.3	4.7	6.0	13.9	8.3	4.5	3.9	9.6	5.3	3.9	4.4
**Upper CI**	9.4	6.2	4.8	5.1	6.9	8.3	16.8	11.0	5.9	5.2	11.9	6.7	5.5	5.9
**P.05**	-2.0	-6.0	-5.0	-7.0	-8.0	-6.0	-3.0	-6.0	-3.2	-3.7	-2.0	-2.0	-4.0	-3.0
**P.10**	0.0	-3.0	-4.0	-4.0	-4.0	-3.0	2.0	-3.0	-1.5	-2.3	1.0	-0.3	-2.7	-2.0
**P.15**	1.0	-1.0	-2.0	-3.0	-2.0	-2.0	5.0	-1.0	-0.8	-1.3	2.3	0.3	-1.3	-0.7
**P.20**	3.0	0.0	-1.0	-2.0	-1.0	0.0	7.0	1.0	0.2	-0.3	4.0	1.0	-0.3	-0.3
**P.25**	4.0	1.0	-1.0	-1.0	0.0	1.0	8.0	2.0	1.0	0.3	5.0	2.0	0.3	0.7
**P.50**	**8.0**	**5.0**	**3.0**	**3.0**	**4.0**	**6.0**	**14.0**	**8.0**	**4.0**	**3.7**	**9.3**	**5.3**	**3.3**	**4.0**
**P.75**	13.0	10.0	8.0	8.0	10.0	11.0	22.0	15.0	8.0	8.0	15.0	9.3	8.0	8.3
**P.80**	14.0	10.0	9.0	11.0	11.0	13.0	23.0	17.0	9.2	8.7	16.3	10.0	9.7	9.7
**P.85**	15.0	12.0	10.0	12.0	13.0	15.0	27.0	21.0	10.8	10.3	18.0	11.3	10.7	11.0
**P.90**	17.0	14.0	12.0	14.0	16.0	17.0	29.0	22.0	13.0	11.7	21.3	13.7	12.7	13.0
**P.95**	21.0	19.0	15.0	17.0	21.0	25.0	36.0	27.0	17.5	15.0	25.7	16.0	16.7	19.3
**N**	**401**	**401**	**401**	**401**	**401**	**401**	**401**	**401**	**401**	**401**	**401**	**401**	**401**	**401**
**Deft**	36.8	39.3	39.7	33.4	35.6	37.3	33.2	34.5	38.8	39.4	35.9	40.4	36.2	37.1
**Bias**	0.9	-0.7	3.4	0.5	-2.9	-2.6	2.4	-0.9	0.2	1.1	-0.4	1.2	0.3	0.4

TABLE 20 SAMPLE = NO NOISE OR ABG>0 — EAR = BETTER EAR
Age group = 18-30 — Occupational group = Overall — Gender = Overall

	Single Frequency (kHz)								Combined Frequency (kHz)					
	0.250	0.500	1	2	3	4	6	8	4FA	3FA	HFA	LFA	A123	A124
**Mean**	**8.3**	**4.6**	**3.2**	**3.5**	**4.6**	**6.2**	**14.2**	**8.4**	**4.4**	**3.8**	**9.6**	**5.4**	**3.8**	**4.3**
**S.D.**	4.4	4.7	4.6	5.0	6.1	6.1	7.3	7.2	4.2	4.1	6.1	3.9	4.6	4.5
**Lower CI**	7.3	3.5	2.4	2.3	3.4	4.8	12.3	6.8	3.5	2.9	8.2	4.5	2.9	3.4
**Upper CI**	9.3	5.7	4.1	4.6	5.9	7.5	16.0	10.0	5.3	4.6	10.9	6.2	4.7	5.2
**P.05**	-1.0	-6.0	-5.0	-7.0	-8.0	-8.0	-4.0	-7.0	-3.2	-3.7	-2.7	-2.7	-4.7	-3.0
**P.10**	0.0	-4.0	-4.0	-5.0	-5.0	-3.0	1.0	-4.0	-1.8	-3.0	0.3	-0.7	-3.0	-2.3
**P.15**	1.0	-3.0	-3.0	-3.0	-3.0	-2.0	4.0	-2.0	-0.8	-1.7	1.3	0.3	-1.7	-1.0
**P.20**	3.0	0.0	-2.0	-3.0	-1.0	0.0	6.0	0.0	0.0	-0.7	2.3	1.0	-1.0	-0.7
**P.25**	4.0	0.0	-1.0	-1.0	0.0	1.0	8.0	1.0	0.5	0.0	4.0	1.7	0.0	0.0
**P.50**	**7.0**	**4.0**	**3.0**	**3.0**	**4.0**	**6.0**	**13.0**	**7.0**	**3.5**	**3.0**	**8.3**	**4.3**	**3.0**	**3.0**
**P.75**	11.0	8.0	7.0	8.0	9.0	10.0	20.0	13.0	7.2	7.0	14.0	7.7	7.3	7.7
**P.80**	13.0	9.0	8.0	11.0	11.0	11.0	22.0	15.0	8.2	8.3	15.0	8.7	9.3	9.0
**P.85**	14.0	10.0	9.0	11.0	12.0	13.0	25.0	18.0	9.8	9.0	16.7	9.7	10.3	10.3
**P.90**	16.0	12.0	12.0	13.0	14.0	16.0	27.0	22.0	11.0	10.7	18.3	11.7	12.3	12.0
**P.95**	21.0	16.0	14.0	17.0	19.0	21.0	32.0	27.0	15.8	14.3	24.7	15.7	15.3	15.0
**N**	**283**	**283**	**283**	**283**	**283**	**283**	**283**	**283**	**283**	**283**	**283**	**283**	**283**	**283**
**Deft**	43.8	43.3	44.1	41.9	40.9	41.4	35.6	38.5	46.9	46.1	39.3	45.8	44.2	45.6
**Bias**	27.5	9.4	-10.1	-40.3	-44.9	-6.0	-0.6	4.7	-11.8	-13.7	-0.7	8.9	-31.8	-18.8

KEY:
4FA = Average over 0.5, 1, 2, and 4 kHz
3FA = Average over 0.5, 1 and 2 kHz
HFA = Average over 4, 6 and 8 kHz
LFA = Average over 0.250, 0.5, and 1 kHz
A123 = Average over 1, 2, and 3 kHz
A124 = Average over 1, 2, and 4 kHz

Mean, standard deviation, confidence intervals, and percentiles for hearing thresholds as a function of ear, age group, socioeconomic group, gender, sample and frequency

TABLE 21 SAMPLE = ALL — EAR = BETTER EAR
Age group = 18-30 — Occupational group = Overall — Gender = Female

	SINGLE FREQUENCY (kHz)								COMBINED FREQUENCY (kHz)					
	0.250	0.500	1	2	3	4	6	8	4FA	3FA	HFA	LFA	A123	A124
**Mean**	**8.3**	**5.8**	**5.1**	**4.4**	**5.6**	**6.2**	**15.1**	**9.8**	**5.4**	**5.1**	**10.4**	**6.4**	**5.0**	**5.2**
**S.D.**	4.5	5.0	4.5	4.6	4.9	5.0	6.3	6.6	4.1	4.2	5.1	4.2	4.2	4.1
**Lower CI**	7.4	4.7	4.2	3.4	4.5	5.1	13.4	8.1	4.6	4.2	9.1	5.6	4.1	4.4
**Upper CI**	9.2	6.9	6.1	5.4	6.7	7.3	16.8	11.5	6.2	5.9	11.6	7.2	5.9	6.1
**P.05**	-1.0	-5.0	-4.0	-7.0	-8.0	-8.0	-3.0	-8.0	-1.8	-2.7	-1.7	-1.0	-3.3	-2.3
**P.10**	0.0	-3.0	-2.0	-4.0	-3.0	-3.0	3.0	-2.0	-1.0	-1.7	1.0	0.3	-1.7	-1.3
**P.15**	1.0	0.0	-1.0	-3.0	-1.0	-2.0	6.0	0.0	0.0	-0.3	2.7	1.0	-0.3	-0.3
**P.20**	3.0	0.0	0.0	-1.0	-1.0	0.0	8.0	2.0	0.5	0.7	3.3	1.7	0.3	0.7
**P.25**	4.0	1.0	0.0	0.0	1.0	1.0	9.0	3.0	1.5	1.0	5.3	2.7	1.0	1.0
**P.50**	**8.0**	**5.0**	**4.0**	**3.0**	**5.0**	**6.0**	**13.0**	**8.0**	**4.2**	**4.0**	**9.3**	**5.3**	**3.7**	**4.3**
**P.75**	11.0	9.0	9.0	8.0	9.0	10.0	21.0	15.0	7.8	8.0	14.0	8.7	8.7	8.0
**P.80**	12.0	10.0	9.0	10.0	11.0	12.0	23.0	17.0	8.5	8.3	15.0	9.3	9.7	9.3
**P.85**	14.0	12.0	10.0	11.0	13.0	13.0	27.0	21.0	10.0	10.0	16.3	10.7	10.7	10.3
**P.90**	16.0	14.0	12.0	13.0	16.0	16.0	29.0	23.0	12.0	11.7	20.0	13.3	12.0	12.0
**P.95**	21.0	20.0	17.0	16.0	20.0	21.0	35.0	32.0	15.8	15.7	24.7	17.3	15.3	16.7
**N**	**224**	**224**	**224**	**224**	**224**	**224**	**224**	**224**	**224**	**224**	**224**	**224**	**224**	**224**
**Deft**	38.7	37.5	35.8	35.9	35.1	36.5	31.7	31.8	38.9	38.4	34.1	39.4	36.4	38.0
**Bias**	-0.6	-4.1	-3.5	-1.8	1.6	1.3	4.6	-2.4	-2.0	-3.2	1.2	-2.8	-1.2	-1.3

TABLE 22 SAMPLE = NO NOISE OR ABG>0 — EAR = BETTER EAR
Age group = 18-30 — Occupational group = Overall — Gender = Female

	SINGLE FREQUENCY (kHz)								COMBINED FREQUENCY (kHz)					
	0.250	0.500	1	2	3	4	6	8	4FA	3FA	HFA	LFA	A123	A124
**Mean**	**7.8**	**4.9**	**4.7**	**4.0**	**5.5**	**5.8**	**14.5**	**8.6**	**4.9**	**4.5**	**9.6**	**5.8**	**4.7**	**4.9**
**S.D.**	4.2	4.7	4.4	4.7	5.4	5.3	6.5	6.7	4.0	4.1	5.3	3.8	4.3	4.2
**Lower CI**	6.9	3.8	3.6	2.9	4.3	4.7	12.8	7.0	4.0	3.6	8.5	5.0	3.8	4.0
**Upper CI**	8.7	6.0	5.7	5.1	6.7	7.0	16.2	10.2	5.7	5.4	10.8	6.6	5.7	5.7
**P.05**	0.0	-6.0	-4.0	-6.0	-8.0	-8.0	-4.0	-9.0	-1.5	-2.3	-2.3	-1.0	-3.7	-2.3
**P.10**	0.0	-4.0	-2.0	-4.0	-3.0	-2.0	2.0	-3.0	-0.8	-1.7	1.0	0.3	-1.3	-1.3
**P.15**	1.0	-1.0	-2.0	-3.0	-1.0	-2.0	5.0	0.0	0.0	-0.7	1.7	1.0	0.0	-0.3
**P.20**	3.0	0.0	0.0	-1.0	0.0	0.0	7.0	2.0	0.2	0.3	3.0	1.3	0.3	0.7
**P.25**	4.0	1.0	0.0	0.0	1.0	1.0	9.0	3.0	0.8	1.0	5.0	2.3	1.3	1.0
**P.50**	**7.0**	**4.0**	**4.0**	**3.0**	**5.0**	**6.0**	**13.0**	**8.0**	**3.8**	**3.7**	**9.0**	**4.7**	**3.7**	**3.7**
**P.75**	10.0	8.0	8.0	8.0	10.0	10.0	20.0	13.0	7.5	7.0	13.3	8.0	8.7	8.0
**P.80**	11.0	9.0	9.0	11.0	11.0	10.0	22.0	15.0	8.2	8.3	14.3	9.3	9.7	9.0
**P.85**	13.0	11.0	10.0	12.0	13.0	12.0	25.0	18.0	9.8	9.0	15.7	9.7	10.3	10.0
**P.90**	15.0	13.0	12.0	13.0	16.0	13.0	28.0	21.0	10.5	10.7	17.7	10.3	12.0	11.0
**P.95**	19.0	19.0	17.0	16.0	19.0	18.0	32.0	27.0	13.2	15.7	22.7	17.3	15.3	13.0
**N**	**176**	**176**	**176**	**176**	**176**	**176**	**176**	**176**	**176**	**176**	**176**	**176**	**176**	**176**
**Deft**	46.1	38.6	36.4	41.2	45.3	51.1	38.7	42.9	45.8	41.1	48.2	42.2	43.0	47.0
**Bias**	16.3	1.9	-11.1	-28.9	-19.2	11.6	32.8	2.5	-6.6	-12.7	15.6	2.4	-19.7	-9.5

KEY: 4FA = Average over 0.5, 1, 2, and 4 kHz
3FA = Average over 0.5, 1 and 2 kHz
HFA = Average over 4, 6 and 8 kHz
LFA = Average over 0.250, 0.5, and 1 kHz
A123 = Average over 1, 2, and 3 kHz
A124 = Average over 1, 2, and 4 kHz

Mean, standard deviation, confidence intervals, and percentiles for hearing thresholds as a function of ear, age group, socioeconomic group, gender, sample and frequency

TABLE 23 SAMPLE = ALL — EAR = BETTER EAR
Age group = 18-30 Occupational group = Overall Gender = Male

	Single Frequency (kHz)								Combined Frequency (kHz)					
	0.250	0.500	1	2	3	4	6	8	4FA	3FA	HFA	LFA	A123	A124
**Mean**	**8.8**	**4.9**	**3.0**	**3.9**	**6.1**	**8.2**	**15.7**	**9.5**	**5.0**	**3.9**	**11.1**	**5.6**	**4.3**	**5.1**
**S.D.**	4.3	4.6	4.1	4.6	6.6	6.9	7.7	7.3	4.1	3.7	6.5	3.7	4.3	4.4
**Lower CI**	7.4	3.6	2.0	2.4	4.1	6.2	13.3	7.4	3.9	2.9	9.2	4.5	3.1	3.8
**Upper CI**	10.2	6.1	4.1	5.4	8.0	10.2	18.1	11.5	6.2	5.0	13.0	6.6	5.6	6.3
**P.05**	-2.0	-7.0	-5.0	-7.0	-8.0	-6.0	-3.0	-4.0	-3.2	-3.7	-2.3	-2.7	-4.3	-3.7
**P.10**	0.0	-4.0	-5.0	-5.0	-5.0	-3.0	1.0	-3.0	-2.0	-3.3	0.3	-1.0	-3.0	-2.3
**P.15**	1.0	-3.0	-4.0	-4.0	-3.0	-1.0	4.0	-1.0	-1.0	-2.3	2.3	-0.3	-2.3	-1.0
**P.20**	3.0	0.0	-2.0	-3.0	-1.0	1.0	6.0	0.0	-0.2	-1.0	4.0	0.3	-1.0	-0.7
**P.25**	4.0	0.0	-2.0	-2.0	0.0	2.0	8.0	1.0	0.5	-0.3	5.0	1.3	-0.7	0.0
**P.50**	**9.0**	**5.0**	**2.0**	**3.0**	**4.0**	**7.0**	**15.0**	**8.0**	**3.8**	**2.7**	**9.3**	**5.3**	**3.0**	**3.3**
**P.75**	14.0	10.0	7.0	8.0	10.0	12.0	22.0	15.0	8.2	7.7	16.0	9.7	7.3	9.0
**P.80**	15.0	11.0	9.0	11.0	11.0	15.0	24.0	17.0	10.0	9.3	17.0	10.3	9.7	10.3
**P.85**	16.0	12.0	10.0	12.0	14.0	16.0	27.0	20.0	11.2	10.7	19.7	12.0	12.3	11.3
**P.90**	18.0	14.0	12.0	15.0	17.0	19.0	29.0	22.0	14.2	11.7	22.7	13.7	13.7	14.7
**P.95**	21.0	17.0	15.0	18.0	29.0	31.0	37.0	27.0	17.5	14.3	29.0	15.7	19.0	20.0
**N**	**177**	**177**	**177**	**177**	**177**	**177**	**177**	**177**	**177**	**177**	**177**	**177**	**177**	**177**
**Deft**	34.3	40.4	40.8	30.4	35.5	36.1	33.9	36.5	37.8	38.3	36.5	40.0	35.0	35.6
**Bias**	1.7	-1.5	5.7	0.8	-10.3	-5.2	0.6	-4.7	-0.1	1.7	-3.1	2.0	-1.3	0.4

TABLE 24 SAMPLE = NO NOISE OR ABG>0 — EAR = BETTER EAR
Age group = 18-30 Occupational group = Overall Gender = Male

	Single Frequency (kHz)								Combined Frequency (kHz)					
	0.250	0.500	1	2	3	4	6	8	4FA	3FA	HFA	LFA	A123	A124
**Mean**	**8.8**	**4.3**	**1.7**	**2.8**	**3.7**	**6.5**	**13.8**	**8.1**	**3.8**	**2.9**	**9.5**	**4.9**	**2.8**	**3.7**
**S.D.**	4.6	4.7	4.3	5.1	6.7	6.8	8.0	7.7	4.3	4.0	6.8	3.9	4.6	4.6
**Lower CI**	6.9	2.4	0.3	0.8	1.5	4.1	10.6	5.3	2.2	1.4	7.0	3.5	1.1	2.0
**Upper CI**	10.7	6.1	3.1	4.9	5.9	9.0	17.0	10.9	5.4	4.5	12.0	6.4	4.4	5.4
**P.05**	-1.0	-8.0	-6.0	-8.0	-8.0	-6.0	-5.0	-7.0	-3.5	-3.7	-2.7	-3.0	-5.3	-3.7
**P.10**	0.0	-5.0	-5.0	-5.0	-7.0	-3.0	0.0	-4.0	-3.2	-3.3	-1.0	-1.0	-4.0	-3.0
**P.15**	0.0	-3.0	-5.0	-4.0	-5.0	-2.0	3.0	-3.0	-2.0	-3.3	0.3	-0.3	-3.0	-2.3
**P.20**	3.0	0.0	-4.0	-3.0	-4.0	0.0	5.0	-2.0	-1.0	-2.3	2.0	0.0	-3.0	-1.0
**P.25**	4.0	0.0	-3.0	-3.0	-2.0	1.0	7.0	-1.0	-0.2	-1.0	3.0	0.7	-1.7	-0.7
**P.50**	**8.0**	**5.0**	**0.0**	**2.0**	**3.0**	**6.0**	**13.0**	**5.0**	**2.8**	**2.0**	**7.3**	**4.0**	**1.3**	**2.3**
**P.75**	13.0	8.0	5.0	6.0	9.0	11.0	20.0	13.0	7.0	5.7	15.3	7.7	5.3	7.0
**P.80**	14.0	10.0	7.0	10.0	9.0	13.0	21.0	17.0	8.2	8.7	16.7	8.3	7.7	9.3
**P.85**	16.0	10.0	8.0	11.0	11.0	15.0	24.0	18.0	9.8	9.3	17.3	9.3	10.0	10.7
**P.90**	18.0	12.0	10.0	13.0	13.0	17.0	27.0	22.0	13.5	10.7	20.0	11.7	12.7	14.7
**P.95**	22.0	14.0	14.0	18.0	17.0	22.0	36.0	26.0	16.0	14.0	29.3	14.7	16.0	16.0
**N**	**107**	**107**	**107**	**107**	**107**	**107**	**107**	**107**	**107**	**107**	**107**	**107**	**107**	**107**
**Deft**	39.5	50.1	51.2	40.2	34.6	33.7	31.4	33.3	45.6	51.4	32.1	49.4	41.3	41.4
**Bias**	26.9	21.6	28.3	-40.1	-59.1	-38.0	-33.7	8.0	-7.1	3.3	-21.2	25.6	-23.6	-16.6

KEY:
4FA = Average over 0.5, 1, 2, and 4 kHz
3FA = Average over 0.5, 1 and 2 kHz
HFA = Average over 4, 6 and 8 kHz
LFA = Average over 0.250, 0.5, and 1 kHz
A123 = Average over 1, 2, and 3 kHz
A124 = Average over 1, 2, and 4 kHz

Mean, standard deviation, confidence intervals, and percentiles for hearing thresholds as a function of ear, age group, socioeconomic group, gender, sample and frequency

TABLE 25 SAMPLE = ALL EAR = BETTER EAR
Age group = 18-30 Occupational group = Non-Manual Gender = Overall

	SINGLE FREQUENCY (kHz)								COMBINED FREQUENCY (kHz)					
	0.250	0.500	1	2	3	4	6	8	4FA	3FA	HFA	LFA	A123	A124
**Mean**	**7.7**	**4.4**	**2.8**	**2.9**	**3.8**	**5.2**	**13.5**	**8.3**	**3.8**	**3.4**	**9.0**	**5.0**	**3.2**	**3.6**
**S.D.**	4.7	5.1	4.8	4.8	5.1	4.9	6.4	6.7	4.1	4.3	5.0	4.2	4.4	4.2
**Lower CI**	6.4	2.9	1.8	1.7	2.5	4.0	11.8	6.6	2.8	2.3	7.8	3.9	2.2	2.7
**Upper CI**	9.0	5.8	3.8	4.2	5.1	6.4	15.3	10.0	4.8	4.4	10.3	6.0	4.2	4.6
**P.05**	-1.0	-9.0	-5.0	-5.0	-8.0	-6.0	-1.0	-7.0	-3.2	-3.7	-1.7	-2.7	-4.3	-3.0
**P.10**	0.0	-5.0	-4.0	-4.0	-6.0	-3.0	2.0	-3.0	-1.8	-3.3	1.0	-0.7	-3.0	-2.3
**P.15**	1.0	-3.0	-3.0	-3.0	-2.0	-2.0	5.0	-1.0	-1.0	-2.3	2.3	0.0	-2.3	-1.3
**P.20**	3.0	-1.0	-2.0	-3.0	-1.0	0.0	6.0	0.0	-0.5	-1.3	3.3	0.7	-1.3	-0.7
**P.25**	4.0	0.0	-1.0	-2.0	0.0	1.0	8.0	1.0	0.2	0.0	4.3	1.0	-1.0	-0.3
**P.50**	**6.0**	**4.0**	**2.0**	**2.0**	**4.0**	**5.0**	**12.0**	**7.0**	**2.8**	**2.3**	**7.7**	**4.0**	**2.7**	**2.7**
**P.75**	11.0	9.0	6.0	6.0	9.0	8.0	20.0	13.0	6.8	6.0	13.0	7.3	5.3	6.0
**P.80**	12.0	10.0	7.0	7.0	9.0	10.0	21.0	15.0	7.5	7.0	14.3	8.0	6.7	7.3
**P.85**	14.0	11.0	8.0	10.0	11.0	11.0	25.0	17.0	8.2	8.7	15.0	9.7	9.3	8.3
**P.90**	15.0	12.0	10.0	12.0	12.0	13.0	27.0	21.0	9.0	9.3	16.7	12.0	10.0	9.7
**P.95**	19.0	16.0	12.0	14.0	14.0	17.0	30.0	25.0	11.8	11.7	21.3	13.3	12.3	12.3
**N**	**181**	**181**	**181**	**181**	**181**	**181**	**181**	**181**	**181**	**181**	**181**	**181**	**181**	**181**
**Deft**	53.4	49.8	66.7	52.1	52.6	48.6	38.7	38.9	62.8	63.4	42.7	61.5	61.5	61.3
**Bias**	40.2	27.3	19.2	14.2	4.4	30.2	-3.2	2.9	22.7	20.2	10.0	28.9	12.6	21.2

TABLE 26 SAMPLE = NO NOISE OR ABG>0 EAR = BETTER EAR
Age group = 18-30 Occupational group = Non-Manual Gender = Overall

	SINGLE FREQUENCY (kHz)								COMBINED FREQUENCY (kHz)					
	0.250	0.500	1	2	3	4	6	8	4FA	3FA	HFA	LFA	A123	A124
**Mean**	**7.6**	**4.0**	**2.3**	**2.7**	**3.5**	**5.2**	**12.8**	**7.7**	**3.5**	**3.0**	**8.6**	**4.6**	**2.8**	**3.4**
**S.D.**	4.5	4.9	4.7	4.8	5.0	4.5	5.9	6.3	3.9	4.1	4.5	3.9	4.2	4.0
**Lower CI**	6.2	2.2	1.1	1.0	1.9	3.6	10.8	5.7	2.3	1.6	7.0	3.4	1.6	2.1
**Upper CI**	8.9	5.7	3.5	4.3	5.1	6.8	14.8	9.6	4.8	4.3	10.1	5.8	4.1	4.7
**P.05**	-1.0	-9.0	-5.0	-5.0	-8.0	-6.0	-3.0	-7.0	-3.2	-3.7	-2.3	-2.7	-4.3	-3.0
**P.10**	0.0	-6.0	-5.0	-5.0	-4.0	-2.0	1.0	-4.0	-1.8	-3.3	1.0	-0.7	-3.0	-2.3
**P.15**	1.0	-4.0	-4.0	-4.0	-3.0	-1.0	3.0	-2.0	-1.0	-2.3	2.0	0.0	-2.7	-1.3
**P.20**	3.0	-1.0	-2.0	-3.0	-1.0	0.0	6.0	0.0	-0.8	-1.7	2.3	0.7	-1.7	-0.7
**P.25**	4.0	0.0	-2.0	-2.0	0.0	1.0	7.0	1.0	0.0	0.0	4.0	1.0	-1.0	-0.7
**P.50**	**6.0**	**3.0**	**1.0**	**1.0**	**3.0**	**4.0**	**12.0**	**7.0**	**2.5**	**1.7**	**7.3**	**3.7**	**2.0**	**2.3**
**P.75**	10.0	7.0	5.0	6.0	9.0	8.0	20.0	12.0	6.0	5.0	12.3	6.7	4.7	5.0
**P.80**	11.0	8.0	6.0	7.0	9.0	9.0	20.0	13.0	7.0	6.0	14.0	7.3	6.0	6.7
**P.85**	12.0	9.0	7.0	9.0	10.0	10.0	22.0	16.0	7.8	8.3	14.7	7.7	8.7	8.0
**P.90**	14.0	11.0	9.0	12.0	11.0	12.0	25.0	19.0	8.8	9.0	16.0	9.3	9.7	9.0
**P.95**	20.0	15.0	12.0	14.0	13.0	14.0	28.0	22.0	10.5	10.7	16.7	12.3	11.3	11.3
**N**	**143**	**143**	**143**	**143**	**143**	**143**	**143**	**143**	**143**	**143**	**143**	**143**	**143**	**143**
**Deft**	55.5	54.3	64.7	51.3	52.4	51.0	38.9	49.6	64.9	64.2	49.4	63.7	60.9	62.3
**Bias**	63.6	54.5	27.7	11.5	12.0	69.4	26.7	76.4	40.8	31.3	57.5	48.6	17.1	36.2

KEY:
4FA = Average over 0.5, 1, 2, and 4 kHz
3FA = Average over 0.5, 1 and 2 kHz
HFA = Average over 4, 6 and 8 kHz
LFA = Average over 0.250, 0.5, and 1 kHz
A123 = Average over 1, 2, and 3 kHz
A124 = Average over 1, 2, and 4 kHz

Mean, standard deviation, confidence intervals, and percentiles for hearing thresholds as a function of ear, age group, socioeconomic group, gender, sample and frequency

TABLE 27 SAMPLE = ALL EAR = BETTER EAR
Age group = 18-30 Occupational group = Non-Manual Gender = Female

	SINGLE FREQUENCY (kHz)								COMBINED FREQUENCY (kHz)					
	0.250	0.500	1	2	3	4	6	8	4FA	3FA	HFA	LFA	A123	A124
**Mean**	7.1	4.9	4.1	3.8	4.9	5.3	14.2	9.3	4.5	4.3	9.6	5.4	4.3	4.4
**S.D.**	4.6	4.9	4.6	4.8	4.7	5.2	6.7	6.8	4.1	4.3	5.2	4.1	4.2	4.2
**Lower CI**	6.0	3.5	2.8	2.4	3.5	3.9	12.2	7.0	3.5	3.1	8.0	4.3	3.1	3.2
**Upper CI**	8.3	6.3	5.4	5.2	6.4	6.7	16.2	11.5	5.6	5.4	11.1	6.4	5.5	5.5
**P.05**	-1.0	-6.0	-4.0	-5.0	-7.0	-8.0	0.0	-8.0	-1.8	-3.0	-2.0	-1.0	-2.7	-2.3
**P.10**	0.0	-4.0	-2.0	-4.0	-3.0	-3.0	2.0	-2.0	-0.8	-2.0	1.0	0.0	-2.0	-1.3
**P.15**	1.0	-1.0	-2.0	-3.0	-1.0	-2.0	6.0	0.0	-0.5	-1.3	2.3	0.7	-1.0	-1.3
**P.20**	2.0	0.0	-1.0	-2.0	-1.0	0.0	6.0	2.0	0.0	0.0	3.0	1.0	0.0	-0.3
**P.25**	4.0	1.0	0.0	-1.0	1.0	0.0	8.0	3.0	0.2	1.0	4.7	1.3	0.7	0.3
**P.50**	6.0	4.0	4.0	2.0	4.0	5.0	12.0	7.0	3.2	3.3	8.3	4.3	3.3	3.3
**P.75**	10.0	8.0	6.0	7.0	9.0	9.0	20.0	13.0	6.8	7.0	13.3	7.3	6.7	7.3
**P.80**	10.0	9.0	8.0	9.0	9.0	10.0	22.0	16.0	7.5	8.0	14.3	7.7	8.3	8.0
**P.85**	12.0	11.0	9.0	11.0	11.0	11.0	25.0	18.0	8.2	8.3	15.7	9.3	9.3	9.0
**P.90**	14.0	12.0	10.0	12.0	13.0	13.0	27.0	21.0	9.8	10.0	16.7	10.7	10.3	10.0
**P.95**	17.0	16.0	12.0	15.0	16.0	17.0	32.0	28.0	13.0	11.7	20.0	13.3	12.3	13.0
**N**	115	115	115	115	115	115	115	115	115	115	115	115	115	115
**Deft**	60.2	50.7	59.8	50.7	45.4	50.7	39.2	35.0	62.3	61.8	40.4	64.7	57.3	59.4
**Bias**	12.4	33.9	31.3	32.1	39.3	32.7	18.2	11.1	32.5	32.4	20.7	25.9	34.2	32.1

TABLE 28 SAMPLE = NO NOISE OR ABG>0 EAR = BETTER EAR
Age group = 18-30 Occupational group = Non-Manual Gender = Female

	SINGLE FREQUENCY (kHz)								COMBINED FREQUENCY (kHz)					
	0.250	0.500	1	2	3	4	6	8	4FA	3FA	HFA	LFA	A123	A124
**Mean**	6.6	3.7	3.5	3.2	4.8	5.1	13.4	8.2	3.9	3.5	8.9	4.6	3.9	4.0
**S.D.**	3.9	4.7	4.4	4.5	4.2	4.3	5.6	5.8	3.5	3.9	4.0	3.6	3.8	3.7
**Lower CI**	5.5	2.3	2.1	1.7	3.4	3.8	11.5	6.2	2.9	2.4	7.6	3.7	2.6	2.8
**Upper CI**	7.8	5.2	5.0	4.8	6.2	6.4	15.2	10.1	4.9	4.6	10.2	5.6	5.1	5.1
**P.05**	-1.0	-6.0	-4.0	-5.0	-4.0	-8.0	0.0	-8.0	-1.5	-3.0	-2.3	-1.0	-2.7	-2.3
**P.10**	0.0	-5.0	-3.0	-4.0	-2.0	-2.0	2.0	-3.0	-0.8	-2.0	1.0	0.0	-1.7	-1.3
**P.15**	1.0	-3.0	-2.0	-3.0	-1.0	0.0	3.0	-1.0	-0.5	-1.3	1.7	0.7	-1.0	-0.7
**P.20**	3.0	0.0	-1.0	-2.0	0.0	0.0	6.0	1.0	0.0	0.0	3.0	1.0	0.3	-0.3
**P.25**	4.0	0.0	0.0	-1.0	1.0	1.0	8.0	3.0	0.2	0.7	5.0	1.3	1.0	0.7
**P.50**	6.0	3.0	3.0	3.0	4.0	5.0	12.0	7.0	3.0	2.7	8.3	4.0	3.3	3.0
**P.75**	10.0	8.0	6.0	7.0	8.0	9.0	20.0	12.0	6.2	5.7	13.0	6.0	6.0	6.3
**P.80**	10.0	8.0	7.0	9.0	9.0	10.0	20.0	13.0	7.5	6.7	14.0	7.0	8.0	7.7
**P.85**	11.0	10.0	8.0	11.0	11.0	11.0	22.0	16.0	8.2	8.3	14.7	8.7	9.3	9.0
**P.90**	12.0	11.0	9.0	11.0	13.0	12.0	27.0	19.0	8.5	8.7	15.7	9.7	9.7	9.7
**P.95**	15.0	13.0	12.0	15.0	14.0	13.0	28.0	22.0	10.0	10.7	16.7	11.7	11.3	11.0
**N**	92	92	92	92	92	92	92	92	92	92	92	92	92	92
**Deft**	56.6	55.8	58.0	47.2	42.3	46.6	37.5	44.0	60.8	61.4	43.3	64.4	56.0	58.8
**Bias**	-1.4	12.9	19.8	-2.4	22.2	40.8	36.2	61.8	17.8	10.1	46.3	10.4	13.2	19.4

KEY: 4FA = Average over 0.5, 1, 2, and 4 kHz
HFA = Average over 4, 6 and 8 kHz
A123 = Average over 1, 2, and 3 kHz
3FA = Average over 0.5, 1 and 2 kHz
LFA = Average over 0.250, 0.5, and 1 kHz
A124 = Average over 1, 2, and 4 kHz

Mean, standard deviation, confidence intervals, and percentiles for hearing thresholds as a function of ear, age group, socioeconomic group, gender, sample and frequency

TABLE 29 SAMPLE = ALL — EAR = BETTER EAR
Age group = 18-30 — Occupational group = Non-Manual — Gender = Male

	SINGLE FREQUENCY (kHz)								COMBINED FREQUENCY (kHz)					
	0.250	0.500	1	2	3	4	6	8	4FA	3FA	HFA	LFA	A123	A124
**Mean**	**8.3**	**3.8**	**1.4**	**2.0**	**2.6**	**5.1**	**12.8**	**7.4**	**3.1**	**2.4**	**8.4**	**4.5**	**2.0**	**2.8**
**S.D.**	4.8	5.1	4.6	4.5	5.3	4.4	6.0	6.4	4.0	4.2	4.7	4.2	4.1	4.0
**Lower CI**	6.0	1.3	-0.1	-0.1	0.3	3.1	9.9	4.8	1.4	0.7	6.5	2.7	0.4	1.3
**Upper CI**	10.6	6.3	2.9	4.1	4.8	7.2	15.7	9.9	4.7	4.1	10.4	6.3	3.6	4.4
**P.05**	-1.0	-9.0	-5.0	-5.0	-8.0	-6.0	-1.0	-7.0	-3.2	-3.7	-0.7	-2.7	-5.0	-3.7
**P.10**	0.0	-6.0	-5.0	-5.0	-7.0	-2.0	1.0	-4.0	-3.2	-3.3	1.0	-1.0	-4.0	-3.0
**P.15**	1.0	-5.0	-5.0	-4.0	-4.0	-1.0	3.0	-2.0	-2.0	-3.3	2.0	-1.0	-3.0	-2.3
**P.20**	3.0	-3.0	-4.0	-3.0	-3.0	0.0	6.0	0.0	-1.2	-2.7	3.3	0.0	-3.0	-1.0
**P.25**	4.0	-2.0	-2.0	-3.0	-2.0	1.0	7.0	0.0	-1.0	-2.3	4.0	0.3	-2.3	-0.7
**P.50**	**6.0**	**4.0**	**0.0**	**1.0**	**3.0**	**5.0**	**12.0**	**7.0**	**1.8**	**1.0**	**7.3**	**2.7**	**1.3**	**1.3**
**P.75**	12.0	9.0	4.0	5.0	9.0	8.0	20.0	13.0	7.0	5.3	13.0	7.7	4.3	4.7
**P.80**	14.0	10.0	5.0	6.0	9.0	10.0	21.0	14.0	7.2	5.7	14.3	8.7	5.3	6.0
**P.85**	16.0	11.0	7.0	6.0	10.0	10.0	23.0	16.0	8.2	9.0	15.0	9.7	6.3	7.3
**P.90**	16.0	12.0	9.0	12.0	11.0	12.0	27.0	22.0	9.0	9.3	16.7	12.0	10.0	9.0
**P.95**	21.0	16.0	10.0	13.0	12.0	16.0	29.0	22.0	11.8	11.7	21.3	12.7	11.3	11.7
**N**	**66**	**66**	**66**	**66**	**66**	**66**	**66**	**66**	**66**	**66**	**66**	**66**	**66**	**66**
**Deft**	44.5	46.4	71.7	50.5	55.5	46.8	37.9	46.4	60.1	61.1	47.5	55.7	61.2	61.1
**Bias**	54.6	32.4	46.1	14.5	-11.0	28.5	-6.5	23.7	30.4	31.0	15.3	44.4	16.5	29.7

TABLE 30 SAMPLE = NO NOISE OR ABG>0 — EAR = BETTER EAR
Age group = 18-30 — Occupational group = Non-Manual — Gender = Male

	SINGLE FREQUENCY (kHz)								COMBINED FREQUENCY (kHz)					
	0.250	0.500	1	2	3	4	6	8	4FA	3FA	HFA	LFA	A123	A124
**Mean**	**8.6**	**4.1**	**1.0**	**2.1**	**2.1**	**5.4**	**12.3**	**7.2**	**3.1**	**2.4**	**8.3**	**4.6**	**1.7**	**2.8**
**S.D.**	4.9	5.2	4.6	4.9	5.4	4.7	6.0	6.8	4.2	4.3	4.9	4.2	4.2	4.1
**Lower CI**	6.0	0.9	-0.9	-1.0	-0.8	2.4	8.6	3.7	0.7	-0.1	5.4	2.3	-0.6	0.5
**Upper CI**	11.1	7.4	2.9	5.1	5.1	8.3	15.9	10.7	5.5	4.9	11.2	6.8	4.0	5.1
**P.05**	-1.0	-9.0	-5.0	-7.0	-11.0	-6.0	-4.0	-7.0	-3.2	-3.7	-3.0	-2.7	-5.3	-3.7
**P.10**	0.0	-6.0	-5.0	-5.0	-7.0	-2.0	1.0	-6.0	-3.2	-3.7	1.0	-2.7	-4.3	-3.0
**P.15**	0.0	-5.0	-5.0	-4.0	-7.0	-2.0	3.0	-4.0	-2.8	-3.3	2.0	-1.0	-3.3	-2.3
**P.20**	1.0	-4.0	-4.0	-4.0	-4.0	0.0	6.0	-1.0	-2.0	-3.3	2.0	0.0	-3.0	-1.3
**P.25**	4.0	0.0	-4.0	-3.0	-3.0	0.0	6.0	0.0	-1.0	-2.3	3.0	0.3	-3.0	-1.0
**P.50**	**6.0**	**4.0**	**-1.0**	**1.0**	**2.0**	**4.0**	**10.0**	**5.0**	**1.5**	**1.0**	**6.0**	**2.7**	**0.3**	**1.0**
**P.75**	12.0	6.0	3.0	5.0	9.0	8.0	17.0	12.0	3.5	2.7	9.7	7.0	3.0	4.0
**P.80**	12.0	7.0	4.0	6.0	9.0	8.0	20.0	14.0	5.5	4.7	13.0	7.3	3.7	4.7
**P.85**	14.0	9.0	5.0	6.0	9.0	8.0	21.0	17.0	7.2	8.0	15.3	7.7	5.0	5.0
**P.90**	16.0	10.0	7.0	12.0	11.0	11.0	25.0	21.0	9.0	9.3	16.7	8.7	9.0	9.0
**P.95**	22.0	16.0	10.0	13.0	11.0	15.0	28.0	22.0	13.0	10.0	21.3	13.7	12.7	14.7
**N**	**51**	**51**	**51**	**51**	**51**	**51**	**51**	**51**	**51**	**51**	**51**	**51**	**51**	**51**
**Deft**	46.7	50.3	72.8	54.2	56.2	54.8	38.6	53.7	65.9	64.9	53.0	59.8	62.3	63.8
**Bias**	121.3	103.5	89.0	54.7	47.9	112.1	37.2	119.9	89.8	82.4	89.8	104.6	63.8	85.3

KEY:
4FA = Average over 0.5, 1, 2, and 4 kHz — 3FA = Average over 0.5, 1 and 2 kHz
HFA = Average over 4, 6 and 8 kHz — LFA = Average over 0.250, 0.5, and 1 kHz
A123 = Average over 1, 2, and 3 kHz — A124 = Average over 1, 2, and 4 kHz

Mean, standard deviation, confidence intervals, and percentiles for hearing thresholds as a function of ear, age group, socioeconomic group, gender, sample and frequency

TABLE 31 SAMPLE = ALL EAR = BETTER EAR
Age group = 18-30 Occupational group = Manual Gender = Overall

	SINGLE FREQUENCY (kHz)								COMBINED FREQUENCY (kHz)					
	0.250	0.500	1	2	3	4	6	8	4FA	3FA	HFA	LFA	A123	A124
**Mean**	**9.6**	**6.4**	**5.5**	**5.2**	**7.4**	**8.9**	**17.0**	**10.5**	**6.5**	**5.7**	**12.1**	**7.2**	**6.0**	**6.5**
**S.D.**	4.6	4.9	4.8	5.2	6.9	7.3	8.6	8.1	4.3	4.2	7.1	4.2	4.6	4.6
**Lower CI**	8.3	5.1	4.3	3.8	5.5	6.9	14.5	8.3	5.3	4.6	10.2	6.1	4.7	5.2
**Upper CI**	10.9	7.6	6.6	6.6	9.4	10.9	19.6	12.7	7.6	6.8	14.1	8.2	7.3	7.8
**P.05**	-2.0	-4.0	-5.0	-8.0	-7.0	-8.0	-7.0	-4.0	-3.2	-3.0	-2.3	-1.7	-4.0	-3.7
**P.10**	0.0	-1.0	-3.0	-5.0	-4.0	-3.0	2.0	-2.0	-0.8	-1.0	0.3	-0.3	-1.3	-1.0
**P.15**	1.0	0.0	-1.0	-3.0	-1.0	-2.0	5.0	0.0	0.5	-0.3	3.0	1.3	-0.3	0.0
**P.20**	4.0	1.0	0.0	-1.0	0.0	1.0	8.0	2.0	1.2	0.7	5.0	2.3	0.3	1.0
**P.25**	5.0	1.0	0.0	0.0	2.0	3.0	10.0	2.0	2.2	1.3	5.7	3.3	1.3	2.0
**P.50**	**10.0**	**6.0**	**5.0**	**4.0**	**6.0**	**8.0**	**16.0**	**10.0**	**5.5**	**5.0**	**11.0**	**7.0**	**4.7**	**5.3**
**P.75**	14.0	10.0	9.0	11.0	13.0	15.0	23.0	17.0	10.5	9.7	17.3	10.3	10.3	10.7
**P.80**	15.0	11.0	11.0	12.0	15.0	16.0	25.0	20.0	11.2	10.7	19.7	11.0	12.0	11.7
**P.85**	17.0	12.0	13.0	14.0	17.0	18.0	28.0	21.0	13.5	11.7	21.3	14.0	13.7	13.3
**P.90**	20.0	16.0	15.0	17.0	20.0	22.0	31.0	26.0	16.0	14.0	24.7	14.7	16.0	16.3
**P.95**	21.0	20.0	17.0	20.0	29.0	33.0	38.0	32.0	19.0	16.7	31.7	17.0	22.3	22.0
**N**	**218**	**218**	**218**	**218**	**218**	**218**	**218**	**218**	**218**	**218**	**218**	**218**	**218**	**218**
**Deft**	35.6	37.6	37.8	32.8	35.1	36.7	39.0	39.8	34.6	35.9	39.1	37.2	33.0	33.5
**Bias**	-23.4	-32.4	-19.2	-44.1	-54.4	-62.6	-5.9	-58.0	-39.6	-31.9	-42.2	-25.0	-39.2	-42.0

TABLE 32 SAMPLE = NO NOISE OR ABG>0 EAR = BETTER EAR
Age group = 18-30 Occupational group = Manual Gender = Overall

	SINGLE FREQUENCY (kHz)								COMBINED FREQUENCY (kHz)					
	0.250	0.500	1	2	3	4	6	8	4FA	3FA	HFA	LFA	A123	A124
**Mean**	**9.4**	**5.4**	**4.5**	**4.2**	**5.7**	**7.2**	**15.7**	**9.0**	**5.3**	**4.7**	**10.6**	**6.4**	**4.8**	**5.3**
**S.D.**	4.8	4.8	5.0	5.8	7.4	7.7	9.6	9.0	4.6	4.3	7.9	4.1	5.0	5.1
**Lower CI**	7.5	3.9	3.0	2.7	3.3	4.5	11.6	5.9	3.8	3.4	7.8	5.0	3.2	3.7
**Upper CI**	11.2	7.0	6.1	5.8	8.1	9.9	19.8	12.0	6.9	6.0	13.5	7.8	6.4	7.0
**P.05**	0.0	-4.0	-5.0	-8.0	-8.0	-8.0	-7.0	-7.0	-3.8	-3.0	-2.7	-2.7	-5.7	-5.0
**P.10**	0.0	-3.0	-4.0	-4.0	-6.0	-4.0	0.0	-3.0	-1.5	-1.0	-1.0	-1.0	-2.3	-2.0
**P.15**	3.0	0.0	-3.0	-3.0	-3.0	-2.0	5.0	-2.0	0.2	-0.7	0.3	1.3	-0.3	-0.7
**P.20**	4.0	0.0	-1.0	0.0	-1.0	0.0	7.0	-1.0	1.0	0.0	2.3	2.3	0.3	0.0
**P.25**	5.0	1.0	0.0	1.0	1.0	2.0	10.0	2.0	1.8	1.0	4.3	3.3	1.0	1.3
**P.50**	**10.0**	**5.0**	**4.0**	**4.0**	**6.0**	**7.0**	**15.0**	**8.0**	**5.0**	**4.3**	**9.7**	**6.7**	**4.3**	**5.0**
**P.75**	14.0	10.0	9.0	11.0	12.0	13.0	22.0	15.0	9.8	8.7	16.3	9.3	10.3	10.3
**P.80**	14.0	10.0	10.0	11.0	14.0	15.0	25.0	17.0	10.8	10.0	17.7	10.0	11.0	11.0
**P.85**	16.0	12.0	12.0	12.0	16.0	17.0	27.0	21.0	12.5	11.0	20.3	11.0	13.0	13.0
**P.90**	18.0	13.0	14.0	17.0	18.0	18.0	31.0	26.0	14.2	14.0	24.7	14.7	15.3	15.0
**P.95**	23.0	20.0	17.0	18.0	23.0	25.0	42.0	35.0	18.2	15.7	34.0	17.3	23.3	21.7
**N**	**140**	**140**	**140**	**140**	**140**	**140**	**140**	**140**	**140**	**140**	**140**	**140**	**140**	**140**
**Deft**	44.8	39.8	40.0	46.9	39.0	40.1	41.8	40.5	41.6	41.1	39.6	41.7	39.6	42.0
**Bias**	-0.7	-45.7	-46.9	-126.3	-147.1	-111.0	-45.1	-113.5	-82.4	-72.9	-89.9	-31.1	-106.7	-94.7

KEY: 4FA = Average over 0.5, 1, 2, and 4 kHz
3FA = Average over 0.5, 1 and 2 kHz
HFA = Average over 4, 6 and 8 kHz
LFA = Average over 0.250, 0.5, and 1 kHz
A123 = Average over 1, 2, and 3 kHz
A124 = Average over 1, 2, and 4 kHz

Mean, standard deviation, confidence intervals, and percentiles for hearing thresholds as a function of ear, age group, socioeconomic group, gender, sample and frequency

TABLE 33 SAMPLE = ALL EAR = BETTER EAR
Age group = 18-30 Occupational group = Manual Gender = Female

	SINGLE FREQUENCY (kHz)								COMBINED FREQUENCY (kHz)					
	0.250	0.500	1	2	3	4	6	8	4FA	3FA	HFA	LFA	A123	A124
**Mean**	**9.6**	**6.9**	**6.4**	**4.8**	**6.5**	**7.6**	**16.7**	**10.1**	**6.4**	**6.0**	**11.4**	**7.6**	**5.9**	**6.3**
**S.D.**	4.7	5.3	5.1	5.3	5.6	5.4	7.5	7.2	4.4	4.7	5.7	4.5	4.6	4.4
**Lower CI**	7.9	5.0	4.6	3.1	4.3	5.5	13.7	7.1	4.9	4.4	9.2	6.1	4.2	4.7
**Upper CI**	11.2	8.8	8.2	6.5	8.7	9.6	19.7	13.0	7.9	7.6	13.7	9.1	7.6	7.8
**P.05**	0.0	-4.0	-4.0	-8.0	-8.0	-8.0	-7.0	-10.0	-1.8	-1.7	-1.3	-1.0	-4.7	-4.0
**P.10**	0.0	-1.0	-2.0	-5.0	-6.0	-3.0	5.0	-3.0	-1.2	-0.7	1.7	2.0	-1.3	-0.3
**P.15**	3.0	0.0	0.0	-2.0	-2.0	-2.0	8.0	1.0	1.2	0.7	3.0	2.7	0.0	1.0
**P.20**	4.0	1.0	0.0	1.0	0.0	0.0	9.0	2.0	2.0	1.3	4.3	3.0	1.0	1.7
**P.25**	5.0	2.0	2.0	1.0	1.0	3.0	11.0	3.0	2.5	2.0	6.3	3.7	2.0	2.3
**P.50**	**9.0**	**6.0**	**5.0**	**5.0**	**7.0**	**7.0**	**16.0**	**9.0**	**5.8**	**5.0**	**10.7**	**6.7**	**5.3**	**6.0**
**P.75**	13.0	10.0	10.0	10.0	12.0	12.0	23.0	17.0	10.0	8.7	15.0	10.0	10.3	10.0
**P.80**	16.0	11.0	11.0	12.0	14.0	13.0	25.0	21.0	10.8	10.0	17.0	11.0	10.7	11.0
**P.85**	17.0	14.0	13.0	12.0	16.0	16.0	28.0	21.0	12.5	12.7	20.7	14.3	12.3	12.0
**P.90**	21.0	20.0	17.0	14.0	19.0	18.0	31.0	27.0	13.8	15.0	24.3	17.0	14.7	13.3
**P.95**	23.0	25.0	20.0	17.0	22.0	23.0	37.0	35.0	25.0	25.0	26.3	26.0	18.3	20.0
**N**	**109**	**109**	**109**	**109**	**109**	**109**	**109**	**109**	**109**	**109**	**109**	**109**	**109**	**109**
**Deft**	32.4	32.4	32.4	36.6	33.5	34.4	40.7	37.7	32.2	33.4	37.8	32.2	32.2	32.2
**Bias**	-44.6	-64.9	-60.6	-82.0	-39.7	-27.2	23.9	-70.8	-58.7	-69.2	-24.7	-56.7	-60.8	-56.6

TABLE 34 SAMPLE = NO NOISE OR ABG>0 EAR = BETTER EAR
Age group = 18-30 Occupational group = Manual Gender = Female

	SINGLE FREQUENCY (kHz)								COMBINED FREQUENCY (kHz)					
	0.250	0.500	1	2	3	4	6	8	4FA	3FA	HFA	LFA	A123	A124
**Mean**	**9.2**	**6.0**	**6.0**	**4.6**	**6.2**	**6.8**	**15.9**	**8.8**	**5.8**	**5.5**	**10.5**	**7.1**	**5.6**	**5.8**
**S.D.**	4.8	5.3	5.2	5.2	6.3	6.0	8.4	8.2	4.5	4.7	6.6	4.5	5.0	4.7
**Lower CI**	7.4	4.0	4.1	2.6	3.8	4.5	12.4	5.6	4.2	3.8	8.1	5.5	3.7	4.1
**Upper CI**	11.0	8.0	7.9	6.5	8.6	9.1	19.5	12.0	7.5	7.2	12.9	8.7	7.4	7.5
**P.05**	0.0	-4.0	-5.0	-7.0	-11.0	-5.0	-9.0	-10.0	-2.8	-1.7	-1.3	-2.3	-4.7	-4.0
**P.10**	0.0	-2.0	-2.0	-3.0	-3.0	-4.0	4.0	-3.0	-1.2	-0.7	0.7	1.0	-1.3	-0.3
**P.15**	3.0	0.0	0.0	0.0	-2.0	-2.0	6.0	0.0	1.2	-0.3	2.3	2.3	0.3	0.7
**P.20**	4.0	0.0	0.0	1.0	1.0	0.0	9.0	2.0	1.8	0.7	3.0	3.0	1.7	1.7
**P.25**	4.0	1.0	1.0	1.0	2.0	3.0	10.0	3.0	2.5	1.7	5.0	3.3	2.3	2.0
**P.50**	**9.0**	**5.0**	**5.0**	**5.0**	**7.0**	**7.0**	**15.0**	**9.0**	**5.8**	**5.0**	**10.3**	**6.0**	**5.3**	**6.0**
**P.75**	13.0	10.0	10.0	11.0	13.0	11.0	23.0	15.0	9.8	8.7	14.3	9.3	10.3	10.0
**P.80**	14.0	10.0	11.0	12.0	14.0	13.0	25.0	18.0	10.5	9.7	16.3	10.0	11.0	11.0
**P.85**	16.0	13.0	13.0	12.0	16.0	13.0	28.0	21.0	11.0	12.3	20.3	12.0	12.3	12.0
**P.90**	18.0	18.0	17.0	15.0	19.0	17.0	31.0	27.0	13.2	15.7	24.3	14.7	14.7	13.0
**P.95**	21.0	23.0	19.0	17.0	23.0	18.0	37.0	33.0	19.2	18.3	25.7	21.7	18.3	19.7
**N**	**84**	**84**	**84**	**84**	**84**	**84**	**84**	**84**	**84**	**84**	**84**	**84**	**84**	**84**
**Deft**	47.4	36.5	37.0	41.3	44.0	49.7	49.4	49.1	40.6	38.4	51.3	39.9	40.0	42.2
**Bias**	18.1	-55.4	-77.6	-121.4	-98.9	-33.6	18.5	-115.1	-72.0	-84.8	-43.4	-38.3	-99.3	-77.5

KEY: 4FA = Average over 0.5, 1, 2, and 4 kHz
3FA = Average over 0.5, 1 and 2 kHz
HFA = Average over 4, 6 and 8 kHz
LFA = Average over 0.250, 0.5, and 1 kHz
A123 = Average over 1, 2, and 3 kHz
A124 = Average over 1, 2, and 4 kHz

Mean, standard deviation, confidence intervals, and percentiles for hearing thresholds as a function of ear, age group, socioeconomic group, gender, sample and frequency

TABLE 35 SAMPLE = ALL — EAR = BETTER EAR
Age group = 18-30 — Occupational group = Manual — Gender = Male

	Single Frequency (kHz)								Combined Frequency (kHz)					
	0.250	0.500	1	2	3	4	6	8	4FA	3FA	HFA	LFA	A123	A124
**Mean**	**9.7**	**5.8**	**4.4**	**5.6**	**8.4**	**10.3**	**17.3**	**10.9**	**6.5**	**5.3**	**12.8**	**6.7**	**6.1**	**6.8**
**S.D.**	4.5	4.3	4.2	5.0	7.7	8.5	9.5	8.9	4.3	3.6	8.2	3.7	4.6	4.8
**Lower CI**	7.7	4.3	2.9	3.4	5.2	6.8	13.2	7.6	4.8	3.9	9.5	5.2	4.2	4.8
**Upper CI**	11.8	7.4	6.0	7.8	11.6	13.7	21.4	14.2	8.3	6.7	16.1	8.1	8.1	8.8
**P.05**	-2.0	-5.0	-5.0	-8.0	-7.0	-8.0	-7.0	-4.0	-3.2	-4.3	-2.3	-2.0	-3.7	-3.3
**P.10**	0.0	-3.0	-4.0	-4.0	-4.0	-3.0	1.0	-2.0	-0.8	-2.3	0.0	-1.0	-0.7	-2.0
**P.15**	1.0	0.0	-3.0	-3.0	-1.0	-2.0	4.0	-1.0	0.2	-0.3	2.7	0.3	-0.3	-0.7
**P.20**	3.0	0.0	-1.0	-2.0	0.0	1.0	6.0	1.0	1.0	0.0	5.0	1.3	0.3	0.3
**P.25**	5.0	1.0	0.0	-1.0	2.0	3.0	8.0	2.0	1.2	1.0	5.7	3.3	0.7	1.3
**P.50**	**10.0**	**6.0**	**4.0**	**4.0**	**6.0**	**9.0**	**16.0**	**10.0**	**5.5**	**5.0**	**11.0**	**7.0**	**4.7**	**5.0**
**P.75**	14.0	10.0	9.0	11.0	13.0	16.0	23.0	17.0	10.8	10.3	17.7	10.3	11.0	11.0
**P.80**	15.0	11.0	10.0	12.0	16.0	17.0	25.0	20.0	12.0	10.7	19.7	11.0	13.0	13.0
**P.85**	17.0	12.0	14.0	15.0	18.0	22.0	28.0	22.0	14.2	11.7	22.7	13.3	13.7	15.0
**P.90**	19.0	14.0	14.0	18.0	21.0	30.0	35.0	26.0	16.8	13.3	25.0	14.7	18.3	20.0
**P.95**	21.0	18.0	15.0	20.0	32.0	34.0	38.0	27.0	19.0	15.0	33.3	15.7	24.0	22.0
**N**	**109**	**109**	**109**	**109**	**109**	**109**	**109**	**109**	**109**	**109**	**109**	**109**	**109**	**109**
**Deft**	37.8	40.7	39.2	28.5	36.8	38.8	39.7	41.9	35.4	34.5	41.4	39.9	32.6	34.2
**Bias**	-3.5	-24.6	-16.6	-3.2	-43.7	-55.1	-24.4	-44.1	-24.9	-14.8	-41.2	-14.9	-21.2	-25.0

TABLE 36 SAMPLE = NO NOISE OR ABG>0 — EAR = BETTER EAR
Age group = 18-30 — Occupational group = Manual — Gender = Male

	Single Frequency (kHz)								Combined Frequency (kHz)					
	0.250	0.500	1	2	3	4	6	8	4FA	3FA	HFA	LFA	A123	A124
**Mean**	**9.5**	**4.7**	**2.9**	**3.9**	**5.1**	**7.7**	**15.4**	**9.0**	**4.8**	**3.8**	**10.7**	**5.7**	**4.0**	**4.9**
**S.D.**	4.7	4.0	4.1	6.3	8.2	9.0	10.7	9.6	4.5	3.6	8.9	3.6	4.9	5.3
**Lower CI**	6.3	2.4	0.4	1.6	0.9	2.7	7.8	3.7	2.2	1.8	5.4	3.4	1.3	2.0
**Upper CI**	12.8	7.0	5.4	6.3	9.4	12.8	23.1	14.4	7.4	5.9	16.1	8.1	6.6	7.7
**P.05**	0.0	-5.0	-6.0	-8.0	-8.0	-10.0	-7.0	-4.0	-3.8	-5.0	-2.7	-3.0	-5.7	-5.0
**P.10**	0.0	-3.0	-5.0	-5.0	-7.0	-6.0	-3.0	-3.0	-3.2	-3.0	-2.3	-1.0	-3.7	-3.0
**P.15**	2.0	-1.0	-4.0	-4.0	-5.0	-3.0	4.0	-2.0	-0.8	-0.7	-1.0	-0.3	-0.7	-2.0
**P.20**	3.0	0.0	-3.0	-3.0	-3.0	0.0	5.0	-2.0	0.2	0.0	0.3	1.3	-0.3	-0.7
**P.25**	5.0	0.0	-2.0	-1.0	-1.0	1.0	7.0	-1.0	0.8	0.0	2.7	2.3	0.3	-0.7
**P.50**	**10.0**	**6.0**	**2.0**	**4.0**	**6.0**	**8.0**	**15.0**	**7.0**	**4.8**	**4.3**	**8.3**	**6.7**	**3.0**	**4.7**
**P.75**	14.0	10.0	7.0	11.0	11.0	16.0	20.0	17.0	10.0	8.7	17.3	8.7	9.7	10.7
**P.80**	14.0	11.0	9.0	11.0	13.0	17.0	23.0	17.0	11.2	10.3	17.7	9.7	11.7	13.0
**P.85**	16.0	12.0	10.0	12.0	16.0	18.0	25.0	21.0	14.2	11.0	20.0	11.0	13.0	14.7
**P.90**	18.0	12.0	12.0	17.0	17.0	22.0	36.0	26.0	14.5	11.7	29.3	14.0	16.0	15.0
**P.95**	23.0	12.0	14.0	18.0	29.0	30.0	54.0	42.0	17.0	14.3	34.0	15.7	24.3	21.7
**N**	**56**	**56**	**56**	**56**	**56**	**56**	**56**	**56**	**56**	**56**	**56**	**56**	**56**	**56**
**Deft**	41.5	46.9	40.0	49.4	33.9	34.8	36.4	33.9	40.1	43.6	33.1	42.8	36.1	39.3
**Bias**	-22.9	-35.7	1.9	-123.0	-206.5	-208.8	-116.5	-120.5	-91.4	-52.2	-148.6	-18.9	-109.2	-110.0

KEY:
4FA = Average over 0.5, 1, 2, and 4 kHz
HFA = Average over 4, 6 and 8 kHz
A123 = Average over 1, 2, and 3 kHz
3FA = Average over 0.5, 1 and 2 kHz
LFA = Average over 0.250, 0.5, and 1 kHz
A124 = Average over 1, 2, and 4 kHz

Mean, standard deviation, confidence intervals, and percentiles for hearing thresholds as a function of ear, age group, socioeconomic group, gender, sample and frequency

TABLE 37 SAMPLE = ALL — EAR = BETTER EAR
Age group = 31-40 — Occupational group = Overall — Gender = Overall

	SINGLE FREQUENCY (kHz)								COMBINED FREQUENCY (kHz)					
	0.250	0.500	1	2	3	4	6	8	4FA	3FA	HFA	LFA	A123	A124
**Mean**	**10.4**	**7.2**	**6.0**	**6.8**	**9.6**	**12.4**	**22.7**	**15.1**	**8.1**	**6.7**	**16.8**	**7.8**	**7.4**	**8.4**
**S.D.**	6.2	6.6	6.5	6.9	7.7	8.7	9.7	10.0	6.3	6.1	8.7	6.0	6.5	6.6
**Lower CI**	9.0	5.7	4.5	5.2	7.7	10.5	20.5	12.8	6.6	5.2	14.8	6.5	5.9	6.8
**Upper CI**	11.8	8.7	7.5	8.5	11.4	14.4	25.0	17.4	9.5	8.1	18.7	9.2	9.0	9.9
**P.05**	-1.0	-6.0	-5.0	-6.0	-5.0	-3.0	5.0	-4.0	-1.2	-2.3	1.3	-1.3	-2.7	-2.0
**P.10**	0.0	-2.0	-2.0	-3.0	-2.0	-2.0	7.0	0.0	0.2	-1.0	4.7	0.0	-1.3	-0.7
**P.15**	1.0	0.0	-1.0	-1.0	0.0	1.0	10.0	2.0	1.0	-0.3	6.7	0.7	-0.3	1.0
**P.20**	4.0	1.0	0.0	0.0	1.0	3.0	11.0	3.0	2.2	1.0	8.0	2.0	1.0	1.7
**P.25**	5.0	2.0	0.0	1.0	2.0	5.0	13.0	5.0	3.2	1.7	8.7	2.7	2.3	3.0
**P.50**	**9.0**	**6.0**	**4.0**	**5.0**	**7.0**	**10.0**	**20.0**	**12.0**	**6.0**	**4.3**	**14.3**	**6.3**	**5.3**	**6.3**
**P.75**	15.0	10.0	9.0	10.0	14.0	17.0	28.0	21.0	10.8	8.7	20.7	10.7	9.7	11.3
**P.80**	16.0	11.0	10.0	11.0	16.0	20.0	32.0	22.0	12.5	10.7	23.3	11.7	12.0	13.0
**P.85**	19.0	15.0	12.0	14.0	19.0	22.0	35.0	27.0	14.5	13.0	26.3	14.0	13.7	14.7
**P.90**	21.0	16.0	15.0	19.0	21.0	26.0	40.0	32.0	16.2	15.3	29.7	15.7	16.3	17.7
**P.95**	25.0	21.0	19.0	24.0	27.0	35.0	50.0	46.0	21.0	19.3	42.7	20.0	21.7	21.3
**N**	**331**	**332**	**332**	**332**	**331**	**331**	**331**	**331**	**331**	**332**	**331**	**331**	**331**	**331**
**Deft**	35.2	36.5	34.9	33.8	34.3	36.6	37.1	36.6	35.3	35.0	37.4	35.8	34.1	34.9
**Bias**	2.3	5.7	8.4	4.1	-0.7	3.2	5.5	0.0	4.8	6.1	2.9	5.0	3.5	4.8

TABLE 38 SAMPLE = NO NOISE OR ABG>0 — EAR = BETTER EAR
Age group = 31-40 — Occupational group = Overall — Gender = Overall

	SINGLE FREQUENCY (kHz)								COMBINED FREQUENCY (kHz)					
	0.250	0.500	1	2	3	4	6	8	4FA	3FA	HFA	LFA	A123	A124
**Mean**	**8.6**	**4.8**	**3.4**	**5.0**	**8.5**	**10.8**	**20.0**	**11.5**	**6.0**	**4.4**	**14.1**	**5.6**	**5.6**	**6.4**
**S.D.**	4.4	4.9	4.5	5.7	7.0	7.3	8.4	8.2	4.2	4.2	6.9	3.9	4.9	4.7
**Lower CI**	7.3	3.5	2.0	3.0	6.4	8.9	17.2	9.0	4.8	3.1	12.1	4.6	4.0	5.0
**Upper CI**	9.9	6.1	4.7	7.0	10.6	12.8	22.8	14.0	7.2	5.7	16.1	6.6	7.2	7.8
**P.05**	-1.0	-11.0	-5.0	-6.0	-5.0	-6.0	2.0	-6.0	-2.0	-3.0	-0.3	-2.7	-3.0	-2.3
**P.10**	0.0	-4.0	-3.0	-4.0	-2.0	-2.0	6.0	-3.0	-0.5	-2.0	1.3	-1.0	-2.3	-0.7
**P.15**	0.0	-2.0	-2.0	-3.0	-1.0	1.0	9.0	1.0	0.2	-1.0	5.7	0.0	-1.3	0.0
**P.20**	3.0	0.0	-1.0	-1.0	0.0	2.0	10.0	2.0	0.8	-0.3	8.0	1.3	-0.3	1.0
**P.25**	4.0	1.0	0.0	1.0	2.0	4.0	11.0	3.0	2.0	0.3	8.3	1.7	1.0	1.3
**P.50**	**8.0**	**5.0**	**3.0**	**4.0**	**7.0**	**9.0**	**18.0**	**11.0**	**5.0**	**3.3**	**13.0**	**4.7**	**4.3**	**5.0**
**P.75**	14.0	9.0	7.0	7.0	12.0	16.0	26.0	17.0	7.5	6.7	18.0	9.3	8.7	9.0
**P.80**	15.0	10.0	7.0	8.0	14.0	17.0	29.0	20.0	8.8	8.0	19.3	10.3	9.7	10.3
**P.85**	16.0	11.0	9.0	10.0	18.0	21.0	33.0	21.0	11.2	9.0	20.7	10.7	11.3	12.3
**P.90**	19.0	12.0	10.0	16.0	22.0	23.0	38.0	23.0	12.2	11.0	28.3	11.3	12.7	14.7
**P.95**	21.0	16.0	15.0	23.0	29.0	34.0	51.0	37.0	18.2	17.7	37.0	15.0	20.0	19.0
**N**	**180**	**180**	**180**	**180**	**180**	**180**	**180**	**180**	**180**	**180**	**180**	**180**	**180**	**180**
**Deft**	16.9	17.7	14.8	18.5	22.4	22.2	23.9	23.4	12.6	13.5	19.7	13.0	15.1	13.9
**Bias**	-60.2	-49.3	-93.5	-80.0	-50.9	-47.1	-161.5	-145.1	-67.5	-74.2	-117.9	-67.7	-74.8	-73.5

KEY:
4FA = Average over 0.5, 1, 2, and 4 kHz — 3FA = Average over 0.5, 1 and 2 kHz
HFA = Average over 4, 6 and 8 kHz — LFA = Average over 0.250, 0.5, and 1 kHz
A123 = Average over 1, 2, and 3 kHz — A124 = Average over 1, 2, and 4 kHz

Mean, standard deviation, confidence intervals, and percentiles for hearing thresholds as a function of ear, age group, socioeconomic group, gender, sample and frequency

TABLE 39 SAMPLE = ALL EAR = BETTER EAR
Age group = 31-40 Occupational group = Overall Gender = Female

	SINGLE FREQUENCY (kHz)								COMBINED FREQUENCY (kHz)					
	0.250	0.500	1	2	3	4	6	8	4FA	3FA	HFA	LFA	A123	A124
**Mean**	**10.4**	**6.9**	**5.5**	**6.1**	**7.9**	**9.7**	**20.1**	**12.8**	**7.0**	**6.2**	**14.2**	**7.5**	**6.4**	**7.0**
**S.D.**	5.5	5.6	5.6	5.9	6.2	6.8	8.1	8.6	5.2	5.2	7.1	5.1	5.4	5.5
**Lower CI**	8.9	5.6	4.3	4.8	6.4	8.1	17.9	10.8	5.9	5.1	12.5	6.4	5.3	5.9
**Upper CI**	11.8	8.3	6.8	7.4	9.5	11.2	22.2	14.8	8.0	7.3	15.8	8.7	7.6	8.1
**P.05**	-1.0	-4.0	-3.0	-6.0	-5.0	-5.0	5.0	-3.0	-1.5	-2.0	0.7	-1.0	-3.0	-2.0
**P.10**	0.0	-1.0	-2.0	-3.0	-3.0	-2.0	7.0	1.0	0.0	-0.7	3.7	0.0	-1.3	-0.7
**P.15**	1.0	0.0	-1.0	-1.0	-1.0	1.0	9.0	2.0	0.5	0.0	5.7	1.7	-0.3	1.0
**P.20**	4.0	1.0	0.0	0.0	1.0	2.0	10.0	3.0	1.8	1.0	6.7	2.3	1.0	1.3
**P.25**	5.0	2.0	0.0	1.0	2.0	4.0	10.0	5.0	2.5	1.7	8.0	3.0	1.7	2.0
**P.50**	**9.0**	**6.0**	**4.0**	**5.0**	**7.0**	**8.0**	**18.0**	**11.0**	**5.5**	**4.7**	**12.3**	**6.3**	**4.7**	**5.3**
**P.75**	15.0	10.0	8.0	8.0	12.0	14.0	27.0	17.0	9.2	8.3	18.0	10.3	9.0	9.3
**P.80**	16.0	11.0	10.0	10.0	14.0	15.0	28.0	19.0	11.0	9.3	19.3	11.3	10.3	11.0
**P.85**	19.0	14.0	10.0	12.0	16.0	17.0	32.0	22.0	12.5	11.0	20.3	13.0	12.3	13.0
**P.90**	22.0	17.0	14.0	15.0	19.0	22.0	35.0	26.0	15.0	14.3	26.3	15.0	15.3	15.3
**P.95**	24.0	21.0	18.0	23.0	22.0	28.0	42.0	36.0	18.5	18.0	31.3	20.0	18.7	18.0
**N**	**189**	**190**	**190**	**190**	**189**	**189**	**189**	**189**	**189**	**190**	**189**	**189**	**189**	**189**
**Deft**	34.0	37.9	36.9	40.1	36.6	39.3	36.7	40.3	41.3	40.4	40.2	38.0	39.6	40.4
**Bias**	1.4	2.5	3.2	3.6	2.4	3.0	4.0	-1.9	2.5	3.1	1.7	1.8	2.7	2.9

TABLE 40 SAMPLE = NO NOISE OR ABG>0 EAR = BETTER EAR
Age group = 31-40 Occupational group = Overall Gender = Female

	SINGLE FREQUENCY (kHz)								COMBINED FREQUENCY (kHz)					
	0.250	0.500	1	2	3	4	6	8	4FA	3FA	HFA	LFA	A123	A124
**Mean**	**9.1**	**5.5**	**4.2**	**5.1**	**7.0**	**8.9**	**19.0**	**11.2**	**5.9**	**4.9**	**13.0**	**6.3**	**5.4**	**6.1**
**S.D.**	4.3	4.4	4.2	4.9	5.4	6.0	7.6	7.6	4.0	3.9	6.3	3.7	4.3	4.3
**Lower CI**	7.7	4.2	3.0	3.7	5.2	7.0	16.4	8.9	4.8	3.9	11.1	5.2	4.1	4.8
**Upper CI**	10.6	6.8	5.4	6.6	8.8	10.8	21.6	13.5	7.0	6.0	15.0	7.4	6.7	7.3
**P.05**	-1.0	-4.0	-4.0	-6.0	-5.0	-8.0	5.0	-4.0	-2.2	-2.3	0.0	-2.0	-3.0	-2.0
**P.10**	0.0	-2.0	-2.0	-3.0	-3.0	-2.0	7.0	0.0	0.0	-1.0	3.7	-0.3	-1.7	-0.7
**P.15**	1.0	0.0	-1.0	-1.0	-1.0	-1.0	9.0	2.0	0.2	-0.3	5.7	1.7	-0.3	-0.3
**P.20**	3.0	0.0	0.0	0.0	0.0	1.0	10.0	3.0	0.8	0.3	6.7	2.3	0.7	1.0
**P.25**	5.0	1.0	0.0	1.0	2.0	3.0	11.0	4.0	2.0	1.3	8.0	2.7	1.3	1.3
**P.50**	**9.0**	**6.0**	**4.0**	**4.0**	**6.0**	**8.0**	**18.0**	**11.0**	**5.0**	**4.0**	**11.7**	**5.7**	**4.3**	**5.0**
**P.75**	14.0	9.0	7.0	7.0	10.0	13.0	25.0	16.0	8.2	7.7	17.0	9.7	6.7	8.0
**P.80**	15.0	10.0	8.0	7.0	12.0	14.0	27.0	17.0	9.2	8.3	18.3	10.3	8.7	9.0
**P.85**	16.0	11.0	9.0	9.0	13.0	16.0	31.0	19.0	10.8	9.0	19.3	11.0	9.3	11.0
**P.90**	19.0	13.0	10.0	11.0	16.0	22.0	34.0	22.0	11.5	10.0	20.7	12.3	11.7	13.0
**P.95**	21.0	16.0	13.0	17.0	22.0	28.0	38.0	28.0	15.0	13.3	28.3	15.0	16.0	15.7
**N**	**129**	**129**	**129**	**129**	**129**	**129**	**129**	**129**	**129**	**129**	**129**	**129**	**129**	**129**
**Deft**	33.2	38.4	37.9	34.0	32.1	33.6	33.7	37.7	36.2	38.3	35.3	37.0	33.6	33.8
**Bias**	6.3	-5.4	15.2	27.6	20.6	25.4	-68.1	-28.7	15.7	12.5	-23.8	5.4	21.1	22.7

KEY: 4FA = Average over 0.5, 1, 2, and 4 kHz
HFA = Average over 4, 6 and 8 kHz
A123 = Average over 1, 2, and 3 kHz
3FA = Average over 0.5, 1 and 2 kHz
LFA = Average over 0.250, 0.5, and 1 kHz
A124 = Average over 1, 2, and 4 kHz

Mean, standard deviation, confidence intervals, and percentiles for hearing thresholds as a function of ear, age group, socioeconomic group, gender, sample and frequency

TABLE 41 SAMPLE = ALL EAR = BETTER EAR
Age group = 31-40 Occupational group = Overall Gender = Male

	Single Frequency (kHz) 0.250	0.500	1	2	3	4	6	8	Combined Frequency (kHz) 4FA	3FA	HFA	LFA	A123	A124
**Mean**	**10.5**	**7.4**	**6.6**	**7.6**	**11.3**	**15.5**	**25.7**	**17.6**	**9.3**	**7.2**	**19.6**	**8.2**	**8.5**	**9.9**
**S.D.**	7.0	7.6	7.4	7.8	8.7	9.6	10.5	10.8	7.1	7.0	9.4	6.9	7.4	7.4
**Lower CI**	7.9	4.6	3.7	4.4	7.9	11.7	21.7	13.4	6.4	4.4	15.9	5.6	5.5	6.9
**Upper CI**	13.1	10.3	9.4	10.9	14.8	19.3	29.8	21.9	12.1	10.0	23.3	10.8	11.5	12.9
**P.05**	-1.0	-8.0	-5.0	-6.0	-4.0	-3.0	3.0	-6.0	-1.2	-2.3	1.3	-2.3	-2.7	-1.0
**P.10**	0.0	-4.0	-2.0	-4.0	-1.0	0.0	10.0	-3.0	0.5	-1.0	5.7	-1.0	-2.0	-0.3
**P.15**	1.0	-1.0	-2.0	-2.0	0.0	2.0	12.0	2.0	2.0	-0.3	8.3	0.7	-0.7	1.0
**P.20**	4.0	1.0	0.0	-1.0	2.0	4.0	14.0	4.0	2.5	0.0	9.3	1.3	1.3	3.0
**P.25**	4.0	2.0	0.0	1.0	4.0	6.0	16.0	7.0	3.8	1.0	10.0	2.0	2.3	3.7
**P.50**	**9.0**	**6.0**	**4.0**	**5.0**	**7.0**	**13.0**	**22.0**	**14.0**	**6.2**	**4.3**	**17.0**	**6.3**	**6.0**	**7.3**
**P.75**	15.0	11.0	9.0	11.0	15.0	22.0	32.0	23.0	13.0	10.0	23.7	10.7	11.3	14.0
**P.80**	15.0	12.0	10.0	12.0	17.0	23.0	36.0	28.0	14.5	11.7	27.0	12.3	12.7	14.7
**P.85**	17.0	15.0	14.0	17.0	19.0	25.0	38.0	32.0	16.0	14.3	29.7	14.0	14.3	17.7
**P.90**	21.0	16.0	16.0	20.0	25.0	30.0	49.0	37.0	18.8	18.3	35.0	16.0	19.0	19.7
**P.95**	25.0	21.0	19.0	28.0	29.0	38.0	58.0	53.0	21.8	20.3	47.0	20.0	23.7	23.0
**N**	**142**	**142**	**142**	**142**	**142**	**142**	**142**	**142**	**142**	**142**	**142**	**142**	**142**	**142**
**Deft**	36.4	36.2	33.8	30.7	31.7	31.7	33.9	32.6	31.9	32.6	32.5	35.0	31.1	31.2
**Bias**	8.7	13.0	19.6	4.7	-3.7	6.9	14.9	1.6	11.0	12.4	7.8	13.8	6.9	10.4

TABLE 42 SAMPLE = NO NOISE OR ABG>0 EAR = BETTER EAR
Age group = 31-40 Occupational group = Overall Gender = Male

	Single Frequency (kHz) 0.250	0.500	1	2	3	4	6	8	Combined Frequency (kHz) 4FA	3FA	HFA	LFA	A123	A124
**Mean**	**8.0**	**4.1**	**2.5**	**4.7**	**10.2**	**13.0**	**21.1**	**11.8**	**6.1**	**3.8**	**15.3**	**4.9**	**5.8**	**6.7**
**S.D.**	4.5	5.4	4.7	6.5	8.0	8.1	9.2	9.0	4.5	4.4	7.3	3.8	5.4	5.1
**Lower CI**	5.7	1.8	-0.1	0.8	6.3	9.4	16.0	7.1	3.7	1.3	11.6	3.1	2.7	4.0
**Upper CI**	10.3	6.5	5.0	8.7	14.0	16.5	26.2	16.5	8.4	6.3	19.0	6.7	8.8	9.4
**P.05**	-4.0	-12.0	-7.0	-6.0	-4.0	-2.0	2.0	-7.0	-2.0	-3.3	-2.0	-3.3	-2.7	-2.7
**P.10**	0.0	-10.0	-5.0	-5.0	-2.0	1.0	5.0	-6.0	-1.2	-2.7	1.3	-2.7	-2.7	-0.7
**P.15**	0.0	-6.0	-3.0	-4.0	0.0	2.0	10.0	-3.0	0.5	-2.0	7.3	-1.0	-2.0	1.0
**P.20**	1.0	-4.0	-2.0	-3.0	0.0	4.0	10.0	1.0	0.5	-1.0	9.3	-1.0	-2.0	1.0
**P.25**	3.0	-1.0	-2.0	-2.0	2.0	5.0	14.0	2.0	2.0	-1.0	10.3	0.3	-0.7	1.0
**P.50**	**6.0**	**4.0**	**1.0**	**3.0**	**8.0**	**15.0**	**18.0**	**11.0**	**4.8**	**2.3**	**14.3**	**3.7**	**3.7**	**5.7**
**P.75**	13.0	7.0	4.0	7.0	19.0	21.0	32.0	20.0	7.0	4.7	18.7	8.7	11.3	10.3
**P.80**	14.0	9.0	5.0	10.0	22.0	22.0	36.0	21.0	8.2	6.0	23.7	9.3	12.0	12.7
**P.85**	15.0	10.0	7.0	17.0	25.0	26.0	50.0	28.0	12.0	8.0	29.7	10.3	12.0	14.7
**P.90**	19.0	12.0	15.0	19.0	27.0	34.0	51.0	30.0	15.0	14.7	37.0	10.7	20.0	18.0
**P.95**	21.0	15.0	19.0	30.0	40.0	35.0	58.0	56.0	23.2	19.7	47.0	13.3	26.0	28.0
**N**	**51**	**51**	**51**	**51**	**51**	**51**	**51**	**51**	**51**	**51**	**51**	**51**	**51**	**51**
**Deft**	8.8	9.7	7.0	11.4	15.1	14.1	16.3	15.0	6.3	6.5	11.7	5.7	8.7	7.6
**Bias**	143.3	-85.6	-237.3	-268.7	-298.5	-328.0	-405.2	-383.4	-229.9	-197.2	-372.2	-155.4	-268.1	-278.0

KEY: 4FA = Average over 0.5, 1, 2, and 4 kHz
3FA = Average over 0.5, 1 and 2 kHz
HFA = Average over 4, 6 and 8 kHz
LFA = Average over 0.250, 0.5, and 1 kHz
A123 = Average over 1, 2, and 3 kHz
A124 = Average over 1, 2, and 4 kHz

Mean, standard deviation, confidence intervals, and percentiles for hearing thresholds as a function of ear, age group, socioeconomic group, gender, sample and frequency

TABLE 43 SAMPLE = ALL — EAR = BETTER EAR
Age group = 31-40 — Occupational group = Non-Manual — Gender = Overall

	Single Frequency (kHz)								Combined Frequency (kHz)					
	0.250	0.500	1	2	3	4	6	8	4FA	3FA	HFA	LFA	A123	A124
**Mean**	**10.2**	**6.4**	**4.6**	**5.6**	**7.7**	**10.6**	**21.7**	**14.3**	**6.8**	**5.6**	**15.5**	**7.1**	**6.0**	**6.9**
**S.D.**	5.5	5.6	4.7	5.6	6.1	6.7	10.1	10.6	4.4	4.5	8.3	4.5	4.8	4.6
**Lower CI**	8.5	4.8	3.4	3.9	5.8	8.4	18.9	11.0	5.6	4.4	13.1	5.9	4.6	5.6
**Upper CI**	11.9	8.0	5.9	7.3	9.6	12.7	24.4	17.7	8.0	6.7	18.0	8.3	7.4	8.3
**P.05**	-1.0	-5.0	-5.0	-3.0	-4.0	-3.0	6.0	-3.0	-0.8	-2.0	1.0	-1.3	-2.3	-1.0
**P.10**	0.0	-1.0	-2.0	-3.0	-2.0	-1.0	8.0	1.0	0.2	-0.7	5.0	0.3	-1.3	-0.7
**P.15**	1.0	0.0	-2.0	-2.0	0.0	1.0	10.0	2.0	1.0	0.0	6.7	1.3	0.0	1.0
**P.20**	3.0	1.0	0.0	0.0	1.0	3.0	11.0	2.0	2.2	1.0	8.0	2.0	1.3	2.0
**P.25**	5.0	1.0	0.0	0.0	2.0	5.0	13.0	5.0	3.0	1.7	9.0	2.7	2.3	3.0
**P.50**	**10.0**	**6.0**	**4.0**	**4.0**	**6.0**	**9.0**	**19.0**	**11.0**	**6.2**	**4.3**	**13.0**	**5.7**	**4.3**	**6.0**
**P.75**	15.0	10.0	8.0	9.0	13.0	16.0	27.0	17.0	9.2	8.0	18.0	10.3	8.7	9.3
**P.80**	16.0	11.0	9.0	10.0	14.0	17.0	29.0	20.0	11.2	9.3	19.7	11.0	9.7	11.3
**P.85**	19.0	13.0	10.0	12.0	16.0	20.0	33.0	22.0	12.0	11.0	21.7	12.3	12.0	12.7
**P.90**	20.0	15.0	13.0	15.0	19.0	22.0	38.0	34.0	14.5	12.7	28.7	14.3	14.3	14.7
**P.95**	24.0	22.0	15.0	19.0	21.0	25.0	50.0	50.0	16.5	17.7	37.0	19.3	17.0	18.0
**N**	**148**	**148**	**148**	**148**	**148**	**148**	**148**	**148**	**148**	**148**	**148**	**148**	**148**	**148**
**Deft**	43.2	48.1	43.4	50.4	51.4	50.8	55.1	50.9	50.5	49.5	54.5	44.5	49.8	49.8
**Bias**	17.7	11.4	14.0	20.0	0.3	-1.1	36.8	45.1	11.1	15.1	26.9	14.4	11.4	11.0

TABLE 44 SAMPLE = NO NOISE OR ABG>0 — EAR = BETTER EAR
Age group = 31-40 — Occupational group = Non-Manual — Gender = Overall

	Single Frequency (kHz)								Combined Frequency (kHz)					
	0.250	0.500	1	2	3	4	6	8	4FA	3FA	HFA	LFA	A123	A124
**Mean**	**9.0**	**4.4**	**2.5**	**4.5**	**7.2**	**10.9**	**20.8**	**11.9**	**5.6**	**3.8**	**14.5**	**5.3**	**4.7**	**6.0**
**S.D.**	4.6	4.9	3.7	5.3	5.8	6.8	10.1	9.8	3.4	3.5	8.0	3.3	4.1	4.0
**Lower CI**	6.8	2.5	1.1	2.3	4.9	8.4	17.5	7.8	4.3	2.5	11.7	3.9	3.1	4.5
**Upper CI**	11.1	6.2	3.9	6.8	9.5	13.3	24.2	16.1	6.9	5.1	17.4	6.6	6.4	7.4
**P.05**	0.0	-11.0	-5.0	-3.0	-4.0	-3.0	7.0	-3.0	-2.2	-3.3	0.0	-2.7	-3.0	-1.0
**P.10**	0.0	-4.0	-2.0	-3.0	-2.0	0.0	8.0	1.0	0.0	-2.0	5.7	-1.0	-1.7	-0.7
**P.15**	0.0	-1.0	-2.0	-2.0	0.0	2.0	10.0	2.0	0.2	-0.7	7.3	0.3	-0.3	1.0
**P.20**	1.0	0.0	-2.0	0.0	1.0	3.0	10.0	3.0	0.5	0.0	8.0	1.3	1.3	1.3
**P.25**	4.0	0.0	0.0	1.0	2.0	5.0	12.0	4.0	2.5	1.0	9.3	1.7	2.0	2.3
**P.50**	**9.0**	**4.0**	**3.0**	**4.0**	**6.0**	**9.0**	**19.0**	**11.0**	**5.5**	**2.7**	**13.0**	**4.3**	**3.7**	**5.3**
**P.75**	14.0	8.0	6.0	7.0	12.0	16.0	25.0	17.0	7.2	6.0	17.3	9.3	6.7	8.0
**P.80**	15.0	9.0	7.0	7.0	13.0	17.0	27.0	17.0	8.2	6.7	18.0	10.0	8.7	9.3
**P.85**	16.0	10.0	7.0	9.0	15.0	18.0	33.0	18.0	9.8	8.0	18.7	10.3	9.7	11.3
**P.90**	19.0	12.0	9.0	16.0	19.0	22.0	38.0	21.0	11.5	9.3	24.0	11.0	11.0	12.7
**P.95**	20.0	15.0	10.0	17.0	21.0	34.0	51.0	46.0	13.2	11.0	43.7	14.3	15.0	14.7
**N**	**87**	**87**	**87**	**87**	**87**	**87**	**87**	**87**	**87**	**87**	**87**	**87**	**87**	**87**
**Deft**	42.9	48.4	49.8	52.8	57.5	52.0	56.4	49.7	46.2	47.7	52.0	41.1	52.3	50.0
**Bias**	17.8	41.4	-14.2	-2.2	17.6	39.3	-27.2	-20.9	16.1	8.4	-3.0	15.0	0.4	7.7

KEY: 4FA = Average over 0.5, 1, 2, and 4 kHz
3FA = Average over 0.5, 1 and 2 kHz
HFA = Average over 4, 6 and 8 kHz
LFA = Average over 0.250, 0.5, and 1 kHz
A123 = Average over 1, 2, and 3 kHz
A124 = Average over 1, 2, and 4 kHz

Mean, standard deviation, confidence intervals, and percentiles for hearing thresholds as a function of ear, age group, socioeconomic group, gender, sample and frequency

TABLE 45 SAMPLE = ALL EAR = BETTER EAR
Age group = 31-40 Occupational group = Non-Manual Gender = Female

	SINGLE FREQUENCY (kHz)								COMBINED FREQUENCY (kHz)					
	0.250	0.500	1	2	3	4	6	8	4FA	3FA	HFA	LFA	A123	A124
**Mean**	**10.0**	**5.9**	**4.5**	**5.1**	**6.6**	**8.7**	**18.7**	**10.6**	**6.0**	**5.2**	**12.7**	**6.8**	**5.4**	**6.1**
**S.D.**	5.5	5.4	5.1	5.8	5.9	6.5	8.6	7.8	4.8	4.7	6.9	4.6	5.0	5.0
**Lower CI**	7.8	4.0	2.9	3.7	4.9	6.8	16.0	8.0	4.7	3.8	10.6	5.2	4.1	4.8
**Upper CI**	12.2	7.9	6.2	6.5	8.3	10.5	21.4	13.3	7.4	6.6	14.8	8.5	6.7	7.4
**P.05**	-5.0	-10.0	-5.0	-3.0	-5.0	-4.0	0.0	-4.0	-3.0	-3.3	0.0	-2.7	-3.0	-2.7
**P.10**	0.0	-3.0	-2.0	-3.0	-2.0	-2.0	7.0	-1.0	-0.8	-0.7	2.7	-0.7	-1.7	-1.0
**P.15**	1.0	0.0	-1.0	-1.0	-1.0	1.0	8.0	1.0	0.0	-0.7	5.0	0.7	-0.3	-0.7
**P.20**	4.0	0.0	0.0	0.0	1.0	2.0	9.0	2.0	0.2	0.0	5.7	2.3	0.3	1.3
**P.25**	5.0	1.0	0.0	0.0	2.0	3.0	10.0	3.0	2.2	1.0	7.3	3.0	1.3	2.0
**P.50**	**9.0**	**5.0**	**4.0**	**4.0**	**5.0**	**8.0**	**17.0**	**9.0**	**5.0**	**3.3**	**11.3**	**4.7**	**4.3**	**5.3**
**P.75**	16.0	9.0	7.0	7.0	10.0	12.0	25.0	15.0	8.2	8.0	16.3	10.0	7.0	9.0
**P.80**	17.0	11.0	9.0	9.0	12.0	13.0	25.0	15.0	9.8	8.7	17.3	11.0	9.0	9.3
**P.85**	19.0	13.0	10.0	11.0	13.0	14.0	29.0	17.0	11.2	9.3	18.0	14.0	9.7	11.3
**P.90**	21.0	18.0	12.0	12.0	16.0	17.0	33.0	19.0	12.5	12.0	19.7	15.0	13.7	12.7
**P.95**	24.0	22.0	16.0	16.0	21.0	22.0	35.0	26.0	15.2	15.7	24.0	20.0	15.3	16.0
**N**	**86**	**86**	**86**	**86**	**86**	**86**	**86**	**86**	**86**	**86**	**86**	**86**	**86**	**86**
**Deft**	36.9	40.5	42.2	59.4	54.2	51.7	45.6	41.6	51.8	48.1	47.0	39.9	54.5	53.8
**Bias**	-11.2	10.8	11.8	25.1	18.4	26.3	26.8	35.0	18.5	15.9	29.4	3.8	18.4	21.1

TABLE 46 SAMPLE = NO NOISE OR ABG>0 EAR = BETTER EAR
Age group = 31-40 Occupational group = Non-Manual Gender = Female

	SINGLE FREQUENCY (kHz)								COMBINED FREQUENCY (kHz)					
	0.250	0.500	1	2	3	4	6	8	4FA	3FA	HFA	LFA	A123	A124
**Mean**	**8.9**	**4.4**	**3.1**	**4.2**	**5.9**	**8.6**	**18.9**	**10.2**	**5.1**	**3.9**	**12.5**	**5.5**	**4.4**	**5.3**
**S.D.**	4.2	4.6	3.8	4.9	5.1	5.8	7.9	8.2	3.7	3.7	6.6	3.4	3.9	4.0
**Lower CI**	6.7	2.2	1.6	2.7	4.0	6.2	15.4	6.7	3.7	2.6	9.7	3.8	3.1	3.9
**Upper CI**	11.2	6.5	4.7	5.8	7.8	11.0	22.4	13.6	6.4	5.2	15.3	7.2	5.7	6.7
**P.05**	-1.0	-10.0	-5.0	-3.0	-5.0	-4.0	2.0	-6.0	-3.2	-3.3	0.0	-2.7	-3.0	-2.3
**P.10**	0.0	-4.0	-3.0	-3.0	-2.0	-2.0	7.0	-2.0	-0.8	-2.0	1.0	-2.0	-1.7	-1.0
**P.15**	1.0	-1.0	-2.0	-1.0	-1.0	0.0	8.0	1.0	0.0	-0.7	5.7	0.3	-0.3	-0.7
**P.20**	3.0	0.0	-1.0	0.0	1.0	2.0	9.0	2.0	0.2	0.0	6.0	2.0	0.3	1.0
**P.25**	4.0	0.0	0.0	1.0	2.0	2.0	10.0	3.0	0.2	0.3	8.0	2.7	1.7	1.3
**P.50**	**9.0**	**4.0**	**4.0**	**4.0**	**4.0**	**8.0**	**19.0**	**9.0**	**4.5**	**3.3**	**11.7**	**4.3**	**4.0**	**5.3**
**P.75**	14.0	8.0	6.0	6.0	9.0	12.0	25.0	15.0	8.2	6.7	15.3	9.3	6.0	7.7
**P.80**	15.0	9.0	7.0	7.0	11.0	13.0	25.0	15.0	8.5	8.0	17.3	10.0	8.0	9.0
**P.85**	16.0	10.0	8.0	9.0	12.0	15.0	28.0	17.0	9.8	8.3	18.0	10.3	9.0	9.3
**P.90**	20.0	13.0	10.0	9.0	14.0	17.0	33.0	17.0	11.2	9.3	19.3	13.0	9.7	11.3
**P.95**	20.0	20.0	10.0	16.0	21.0	22.0	35.0	22.0	11.5	10.0	24.0	15.0	11.0	12.3
**N**	**60**	**60**	**60**	**60**	**60**	**60**	**60**	**60**	**60**	**60**	**60**	**60**	**60**	**60**
**Deft**	35.1	41.8	45.5	54.9	51.9	40.9	39.2	40.4	49.0	49.9	40.5	38.8	52.7	48.5
**Bias**	22.3	12.3	1.5	17.7	16.3	22.6	-21.5	-0.6	13.5	10.5	0.2	12.1	11.9	13.9

KEY:
4FA = Average over 0.5, 1, 2, and 4 kHz
3FA = Average over 0.5, 1 and 2 kHz
HFA = Average over 4, 6 and 8 kHz
LFA = Average over 0.250, 0.5, and 1 kHz
A123 = Average over 1, 2, and 3 kHz
A124 = Average over 1, 2, and 4 kHz

Mean, standard deviation, confidence intervals, and percentiles for hearing thresholds as a function of ear, age group, socioeconomic group, gender, sample and frequency

TABLE 47 — SAMPLE = ALL — EAR = BETTER EAR
Age group = 31-40 — Occupational group = Non-Manual — Gender = Male

	Single Frequency (kHz)								Combined Frequency (kHz)					
	0.250	0.500	1	2	3	4	6	8	4FA	3FA	HFA	LFA	A123	A124
**Mean**	**10.6**	**7.0**	**4.7**	**6.2**	**8.9**	**12.7**	**25.1**	**18.5**	**7.6**	**6.0**	**18.8**	**7.4**	**6.6**	**7.9**
**S.D.**	5.6	5.8	4.3	5.2	6.0	6.3	10.5	11.7	3.8	4.2	8.5	4.2	4.5	4.0
**Lower CI**	7.9	4.3	2.8	3.0	5.4	8.5	20.1	12.1	5.6	4.1	14.1	5.6	4.0	5.4
**Upper CI**	13.2	9.6	6.7	9.5	12.4	16.8	30.1	24.9	9.7	7.9	23.4	9.3	9.2	10.3
**P.05**	0.0	-4.0	-5.0	-3.0	-4.0	-2.0	8.0	-3.0	0.5	-2.0	5.7	-1.0	-1.3	0.3
**P.10**	0.0	-1.0	-2.0	-3.0	-2.0	1.0	12.0	2.0	1.5	0.0	9.0	0.7	-1.0	1.0
**P.15**	0.0	1.0	-2.0	-2.0	1.0	4.0	14.0	2.0	2.5	1.0	9.3	1.3	1.3	2.7
**P.20**	3.0	2.0	0.0	0.0	1.0	6.0	15.0	6.0	3.5	2.3	9.7	1.7	2.0	3.3
**P.25**	4.0	2.0	0.0	1.0	3.0	7.0	16.0	8.0	3.8	2.7	10.3	2.7	2.7	4.3
**P.50**	**10.0**	**7.0**	**3.0**	**4.0**	**8.0**	**12.0**	**20.0**	**13.0**	**6.2**	**4.3**	**14.3**	**6.3**	**4.3**	**6.7**
**P.75**	15.0	11.0	9.0	10.0	14.0	20.0	30.0	22.0	9.2	8.3	24.3	10.3	9.7	12.7
**P.80**	15.0	12.0	9.0	12.0	16.0	21.0	36.0	32.0	12.0	10.0	27.3	10.3	11.0	13.7
**P.85**	17.0	13.0	10.0	14.0	19.0	22.0	38.0	37.0	13.0	11.0	29.7	12.0	12.0	14.7
**P.90**	19.0	15.0	14.0	17.0	19.0	24.0	50.0	43.0	15.8	14.0	34.7	13.0	15.7	15.7
**P.95**	23.0	20.0	15.0	23.0	27.0	34.0	51.0	56.0	18.8	19.7	47.0	18.7	21.7	20.0
**N**	**62**	**62**	**62**	**62**	**62**	**62**	**62**	**62**	**62**	**62**	**62**	**62**	**62**	**62**
**Deft**	52.2	58.8	44.5	40.3	46.8	46.0	57.5	49.9	46.0	50.0	53.5	52.1	42.8	41.8
**Bias**	59.2	10.3	15.3	7.4	-31.9	-44.1	50.7	42.0	-2.8	11.0	16.2	28.2	-3.1	-7.1

TABLE 48 — SAMPLE = NO NOISE OR ABG>0 — EAR = BETTER EAR
Age group = 31-40 — Occupational group = Non-Manual — Gender = Male

	Single Frequency (kHz)								Combined Frequency (kHz)					
	0.250	0.500	1	2	3	4	6	8	4FA	3FA	HFA	LFA	A123	A124
**Mean**	**9.0**	**4.3**	**1.8**	**4.9**	**8.8**	**13.5**	**23.3**	**14.0**	**6.1**	**3.7**	**16.9**	**5.1**	**5.2**	**6.8**
**S.D.**	4.9	5.2	3.5	5.6	6.3	7.0	11.9	11.1	2.9	3.2	8.8	3.0	4.4	3.9
**Lower CI**	5.3	1.2	-0.7	0.3	4.2	8.9	17.3	5.9	3.8	1.2	11.6	2.9	1.7	3.9
**Upper CI**	12.7	7.5	4.3	9.6	13.3	18.1	29.2	22.2	8.5	6.2	22.2	7.2	8.6	9.6
**P.05**	0.0	-12.0	-5.0	-3.0	-4.0	1.0	10.0	2.0	0.5	-2.0	9.0	-1.0	-3.0	1.0
**P.10**	0.0	-10.0	-2.0	-3.0	-4.0	4.0	12.0	2.0	0.5	-2.0	9.3	-1.0	-1.7	1.0
**P.15**	0.0	-1.0	-2.0	-2.0	0.0	5.0	14.0	2.0	2.5	0.0	10.3	0.7	2.0	2.7
**P.20**	1.0	-1.0	-2.0	0.0	4.0	6.0	14.0	5.0	2.5	1.0	10.3	0.7	2.0	3.0
**P.25**	1.0	-1.0	-1.0	1.0	4.0	8.0	15.0	6.0	3.0	1.0	11.7	1.3	2.0	3.0
**P.50**	**8.0**	**4.0**	**1.0**	**4.0**	**8.0**	**16.0**	**18.0**	**11.0**	**6.2**	**2.7**	**14.3**	**3.7**	**3.7**	**5.7**
**P.75**	14.0	8.0	3.0	7.0	16.0	18.0	38.0	20.0	6.5	4.7	18.7	8.7	9.7	12.7
**P.80**	15.0	10.0	4.0	7.0	16.0	21.0	43.0	21.0	7.2	4.7	29.7	9.3	9.7	12.7
**P.85**	15.0	10.0	5.0	17.0	19.0	23.0	50.0	21.0	12.0	6.0	29.7	10.3	12.0	14.7
**P.90**	19.0	12.0	7.0	17.0	19.0	23.0	50.0	37.0	12.0	8.0	33.7	10.3	12.0	14.7
**P.95**	21.0	15.0	15.0	29.0	27.0	34.0	51.0	56.0	18.8	19.7	47.0	13.3	23.7	20.0
**N**	**27**	**27**	**27**	**27**	**27**	**27**	**27**	**27**	**27**	**27**	**27**	**27**	**27**	**27**
**Deft**	50.5	57.5	59.3	47.6	58.3	58.3	67.6	54.6	44.8	46.2	60.4	49.7	49.7	52.4
**Bias**	14.4	88.8	-0.2	-53.5	-56.5	-72.0	-140.3	-157.1	-9.2	11.7	-123.1	34.4	-36.7	-41.9

KEY:
4FA = Average over 0.5, 1, 2, and 4 kHz
HFA = Average over 4, 6 and 8 kHz
A123 = Average over 1, 2, and 3 kHz
3FA = Average over 0.5, 1 and 2 kHz
LFA = Average over 0.250, 0.5, and 1 kHz
A124 = Average over 1, 2, and 4 kHz

Mean, standard deviation, confidence intervals, and percentiles for hearing thresholds as a function of ear, age group, socioeconomic group, gender, sample and frequency

TABLE 49 SAMPLE = ALL — EAR = BETTER EAR

Age group = 31-40 — Occupational group = Manual — Gender = Overall

	Single Frequency (kHz)								Combined Frequency (kHz)					
	0.250	0.500	1	2	3	4	6	8	4FA	3FA	HFA	LFA	A123	A124
**Mean**	**10.5**	**7.6**	**7.0**	**7.8**	**11.2**	**13.9**	**23.5**	**15.8**	**9.1**	**7.5**	**17.7**	**8.4**	**8.7**	**9.6**
**S.D.**	7.2	7.5	7.9	8.2	9.3	10.8	11.1	10.8	7.6	7.3	10.0	7.1	7.8	8.1
**Lower CI**	8.2	5.2	4.6	5.0	8.1	10.6	20.0	12.5	6.6	5.1	14.7	6.1	6.1	7.0
**Upper CI**	12.7	10.0	9.5	10.5	14.3	17.1	27.0	19.0	11.5	9.9	20.7	10.6	11.2	12.1
**P.05**	-1.0	-6.0	-3.0	-6.0	-6.0	-5.0	2.0	-6.0	-1.2	-2.7	1.3	-2.3	-2.7	-2.0
**P.10**	0.0	-3.0	-2.0	-4.0	-2.0	-2.0	5.0	0.0	0.0	-1.0	3.7	-0.3	-2.0	-0.7
**P.15**	3.0	-1.0	-1.0	-1.0	0.0	1.0	10.0	1.0	1.2	-0.7	6.7	0.7	-0.7	1.0
**P.20**	4.0	1.0	0.0	0.0	2.0	3.0	11.0	4.0	2.0	0.3	8.0	2.0	1.0	1.3
**P.25**	4.0	2.0	0.0	1.0	3.0	5.0	13.0	6.0	3.2	1.3	8.7	2.3	2.3	2.7
**P.50**	**9.0**	**6.0**	**5.0**	**5.0**	**8.0**	**10.0**	**22.0**	**14.0**	**6.0**	**4.7**	**15.7**	**6.3**	**6.0**	**6.7**
**P.75**	14.0	10.0	10.0	11.0	16.0	19.0	30.0	22.0	13.0	10.7	23.0	10.7	12.0	13.7
**P.80**	15.0	11.0	10.0	12.0	17.0	22.0	33.0	24.0	14.5	12.0	23.7	13.0	13.7	15.0
**P.85**	18.0	15.0	15.0	17.0	20.0	26.0	36.0	30.0	15.8	14.3	28.3	14.3	16.3	17.7
**P.90**	21.0	16.0	18.0	22.0	25.0	30.0	42.0	31.0	19.0	17.3	30.7	16.7	19.0	19.3
**P.95**	28.0	21.0	22.0	28.0	37.0	41.0	58.0	42.0	23.2	22.7	43.3	20.3	26.0	28.0
**N**	**183**	**183**	**183**	**183**	**183**	**183**	**183**	**183**	**183**	**183**	**183**	**183**	**183**	**183**
**Deft**	35.0	33.5	34.1	32.1	33.2	37.1	38.3	36.8	32.2	31.8	36.9	33.3	31.8	32.8
**Bias**	-19.2	-16.6	-11.5	-15.5	-13.5	-25.6	-50.7	-53.4	-17.3	-14.5	-43.2	-15.7	-13.5	-17.5

TABLE 50 SAMPLE = NO NOISE OR ABG>0 — EAR = BETTER EAR

Age group = 31-40 — Occupational group = Manual — Gender = Overall

	Single Frequency (kHz)								Combined Frequency (kHz)					
	0.250	0.500	1	2	3	4	6	8	4FA	3FA	HFA	LFA	A123	A124
**Mean**	**8.0**	**5.0**	**4.6**	**5.5**	**10.4**	**11.3**	**19.6**	**11.0**	**6.6**	**5.0**	**13.9**	**5.9**	**6.8**	**7.1**
**S.D.**	5.8	6.0	6.3	8.4	11.1	10.8	11.9	10.7	6.6	6.1	9.8	5.3	7.5	7.5
**Lower CI**	5.4	2.2	0.9	1.3	7.2	6.6	12.2	7.9	3.4	1.8	9.9	3.5	4.2	3.7
**Upper CI**	10.6	7.8	8.2	9.7	13.5	16.0	26.9	14.0	9.8	8.3	18.0	8.2	9.5	10.6
**P.05**	-1.0	-6.0	-5.0	-6.0	-6.0	-8.0	2.0	-7.0	-2.0	-3.0	-2.0	-2.7	-3.0	-2.7
**P.10**	-1.0	-4.0	-3.0	-6.0	-2.0	-3.0	5.0	-6.0	-1.2	-2.3	1.3	-1.0	-2.7	-0.7
**P.15**	1.0	-2.0	-2.0	-4.0	-1.0	-2.0	5.0	-1.0	0.2	-1.0	4.3	-0.7	-2.0	-0.7
**P.20**	3.0	0.0	-1.0	-3.0	0.0	1.0	10.0	1.0	1.0	-1.0	6.0	0.3	-1.3	1.0
**P.25**	4.0	1.0	0.0	-1.0	0.0	3.0	10.0	2.0	2.0	-0.3	8.0	1.7	-0.3	1.0
**P.50**	**8.0**	**5.0**	**3.0**	**4.0**	**7.0**	**8.0**	**18.0**	**12.0**	**4.8**	**4.0**	**12.3**	**5.7**	**5.3**	**4.7**
**P.75**	11.0	10.0	8.0	7.0	14.0	16.0	27.0	20.0	8.2	8.0	19.3	9.7	10.3	9.7
**P.80**	14.0	10.0	9.0	9.0	17.0	21.0	32.0	22.0	11.0	9.0	20.0	10.7	12.0	11.0
**P.85**	15.0	11.0	10.0	11.0	22.0	22.0	34.0	22.0	12.2	11.0	23.7	11.0	12.7	13.0
**P.90**	18.0	13.0	15.0	19.0	29.0	26.0	35.0	30.0	15.0	13.3	28.3	12.7	16.7	15.7
**P.95**	23.0	16.0	20.0	30.0	40.0	35.0	58.0	34.0	23.2	19.3	37.0	19.3	26.0	28.0
**N**	**93**	**93**	**93**	**93**	**93**	**93**	**93**	**93**	**93**	**93**	**93**	**93**	**93**	**93**
**Deft**	18.0	15.9	15.7	23.4	32.5	28.9	33.2	29.0	16.4	15.4	26.6	13.8	20.0	19.2
**Bias**	164.2	-179.2	-157.5	-154.9	-76.3	-93.6	-253.1	-287.7	-146.3	-163.8	-211.5	-166.9	-129.5	-135.3

KEY:
4FA = Average over 0.5, 1, 2, and 4 kHz
HFA = Average over 4, 6 and 8 kHz
A123 = Average over 1, 2, and 3 kHz
3FA = Average over 0.5, 1 and 2 kHz
LFA = Average over 0.250, 0.5, and 1 kHz
A124 = Average over 1, 2, and 4 kHz

Mean, standard deviation, confidence intervals, and percentiles for hearing thresholds as a function of ear, age group, socioeconomic group, gender, sample and frequency

TABLE 51 SAMPLE = ALL — EAR = BETTER EAR
Age group = 31-40 — Occupational group = Manual — Gender = Female

	Single Frequency (kHz)								Combined Frequency (kHz)					
	0.250	0.500	1	2	3	4	6	8	4FA	3FA	HFA	LFA	A123	A124
**Mean**	**10.1**	**7.4**	**6.0**	**6.8**	**9.2**	**10.6**	**21.0**	**14.5**	**7.7**	**6.7**	**15.4**	**7.8**	**7.3**	**7.8**
**S.D.**	6.1	5.9	6.1	6.7	7.2	7.6	8.4	9.3	5.8	5.6	7.5	5.5	6.2	6.2
**Lower CI**	8.4	5.8	4.2	4.7	6.5	8.0	17.7	11.7	6.1	5.2	12.8	6.4	5.4	5.9
**Upper CI**	11.8	8.9	7.8	8.9	12.0	13.2	24.3	17.4	9.3	8.2	17.9	9.2	9.3	9.7
**P.05**	-1.0	-3.0	-2.0	-6.0	-6.0	-8.0	5.0	0.0	-0.5	-1.3	3.7	-0.3	-3.0	-0.7
**P.10**	0.0	-1.0	-1.0	-3.0	-3.0	-2.0	5.0	1.0	0.5	-0.7	4.3	0.7	-1.0	0.0
**P.15**	2.0	1.0	-1.0	-1.0	-2.0	2.0	10.0	3.0	1.8	1.3	6.7	2.0	0.7	1.0
**P.20**	4.0	2.0	0.0	1.0	1.0	4.0	11.0	5.0	2.5	1.7	8.0	2.3	1.0	1.3
**P.25**	4.0	2.0	0.0	1.0	3.0	5.0	12.0	6.0	3.2	2.3	8.7	3.7	2.3	2.7
**P.50**	**9.0**	**7.0**	**4.0**	**5.0**	**8.0**	**9.0**	**20.0**	**13.0**	**5.8**	**5.0**	**13.0**	**6.7**	**5.7**	**6.0**
**P.75**	14.0	10.0	10.0	9.0	14.0	15.0	28.0	22.0	11.0	9.0	20.0	10.7	10.3	11.0
**P.80**	15.0	11.0	10.0	11.0	16.0	17.0	32.0	22.0	12.2	11.0	23.0	11.3	12.3	13.0
**P.85**	18.0	13.0	11.0	14.0	19.0	21.0	35.0	25.0	15.0	12.0	26.3	13.0	14.7	15.0
**P.90**	22.0	16.0	15.0	20.0	21.0	27.0	36.0	31.0	15.8	14.3	29.0	16.7	16.3	17.7
**P.95**	24.0	20.0	20.0	27.0	27.0	31.0	42.0	38.0	19.2	21.3	37.0	20.0	21.7	21.3
**N**	**103**	**103**	**103**	**103**	**103**	**103**	**103**	**103**	**103**	**103**	**103**	**103**	**103**	**103**
**Deft**	39.8	38.1	35.7	40.5	36.1	38.3	36.8	41.8	38.2	38.1	38.6	37.4	38.2	38.7
**Bias**	-45.6	-44.7	-34.5	-24.6	-18.4	-28.4	-58.5	-81.4	-33.1	-34.6	-56.1	-41.6	-25.8	-29.2

TABLE 52 SAMPLE = NO NOISE OR ABG>0 — EAR = BETTER EAR
Age group = 31-40 — Occupational group = Manual — Gender = Female

	Single Frequency (kHz)								Combined Frequency (kHz)					
	0.250	0.500	1	2	3	4	6	8	4FA	3FA	HFA	LFA	A123	A124
**Mean**	**9.0**	**6.4**	**5.2**	**6.1**	**8.2**	**9.2**	**18.6**	**11.8**	**6.7**	**5.9**	**13.2**	**6.8**	**6.5**	**6.8**
**S.D.**	5.1	4.5	4.8	6.0	6.9	7.0	8.5	7.2	4.5	4.4	6.5	4.2	5.3	5.1
**Lower CI**	6.8	4.6	3.0	3.4	4.4	6.0	14.3	8.7	4.7	4.1	10.1	5.2	3.9	4.5
**Upper CI**	11.2	8.1	7.4	8.8	12.0	12.5	22.8	15.0	8.7	7.7	16.3	8.5	9.1	9.2
**P.05**	-1.0	-2.0	-3.0	-6.0	-11.0	-8.0	5.0	-1.0	-0.5	-1.3	1.3	-0.3	-3.0	-1.7
**P.10**	0.0	0.0	-2.0	-3.0	-5.0	-3.0	6.0	1.0	0.5	-1.0	4.3	0.0	-2.0	-0.7
**P.15**	2.0	1.0	-1.0	-1.0	-2.0	-2.0	10.0	2.0	0.8	1.0	6.0	2.0	-0.3	0.0
**P.20**	4.0	2.0	0.0	-1.0	0.0	1.0	10.0	4.0	1.8	1.3	7.0	2.3	0.7	1.0
**P.25**	5.0	2.0	0.0	1.0	3.0	4.0	11.0	6.0	2.5	1.3	8.3	2.7	1.3	1.7
**P.50**	**9.0**	**7.0**	**4.0**	**4.0**	**7.0**	**7.0**	**18.0**	**12.0**	**5.2**	**4.3**	**12.0**	**6.7**	**5.3**	**4.7**
**P.75**	14.0	10.0	8.0	7.0	11.0	14.0	27.0	19.0	8.2	8.0	19.3	10.3	7.7	8.0
**P.80**	15.0	10.0	8.0	8.0	12.0	15.0	30.0	21.0	10.8	9.0	19.3	10.7	9.0	11.0
**P.85**	17.0	11.0	10.0	11.0	15.0	17.0	34.0	22.0	11.5	9.3	20.3	11.3	11.7	13.0
**P.90**	18.0	13.0	11.0	14.0	19.0	22.0	35.0	23.0	13.2	13.0	26.7	11.7	15.3	15.0
**P.95**	21.0	16.0	15.0	23.0	30.0	28.0	38.0	32.0	15.5	16.0	29.0	16.7	19.0	17.7
**N**	**69**	**69**	**69**	**69**	**69**	**69**	**69**	**69**	**69**	**69**	**69**	**69**	**69**	**69**
**Deft**	43.7	43.2	36.9	36.4	35.1	36.3	38.4	37.4	34.7	37.1	36.4	40.5	34.8	34.0
**Bias**	-43.0	-49.1	12.5	42.5	33.0	27.7	-170.7	-107.2	8.4	1.9	-83.4	-26.5	29.3	27.6

KEY:
4FA = Average over 0.5, 1, 2, and 4 kHz
3FA = Average over 0.5, 1 and 2 kHz
HFA = Average over 4, 6 and 8 kHz
LFA = Average over 0.250, 0.5, and 1 kHz
A123 = Average over 1, 2, and 3 kHz
A124 = Average over 1, 2, and 4 kHz

Mean, standard deviation, confidence intervals, and percentiles for hearing thresholds as a function of ear, age group, socioeconomic group, gender, sample and frequency

TABLE 53 SAMPLE = ALL; Age group = 31-40; Occupational group = Manual; EAR = BETTER EAR; Gender = Male

	Single Frequency (kHz)								Combined Frequency (kHz)					
	0.250	0.500	1	2	3	4	6	8	4FA	3FA	HFA	LFA	A123	A124
**Mean**	**10.9**	**7.9**	**8.2**	**8.8**	**13.3**	**17.4**	**26.1**	**17.0**	**10.6**	**8.3**	**20.2**	**9.0**	**10.1**	**11.5**
**S.D.**	8.2	9.0	9.3	9.4	10.7	12.5	12.9	12.1	8.9	8.7	11.7	8.5	9.0	9.3
**Lower CI**	6.6	3.2	3.4	3.6	7.6	11.3	19.9	11.0	5.8	3.6	14.6	4.6	5.2	6.5
**Upper CI**	15.2	12.6	13.0	14.1	18.9	23.6	32.3	22.9	15.4	13.1	25.7	13.4	15.0	16.5
**P.05**	-4.0	-8.0	-5.0	-6.0	-2.0	-4.0	2.0	-7.0	-2.0	-3.0	-2.0	-3.3	-2.7	-2.7
**P.10**	0.0	-4.0	-3.0	-5.0	-1.0	-2.0	5.0	-6.0	-1.2	-2.3	2.7	-2.3	-2.0	-0.7
**P.15**	3.0	-3.0	-2.0	-4.0	0.0	1.0	10.0	-2.0	0.2	-1.0	7.3	-1.0	-2.0	-0.3
**P.20**	4.0	-1.0	0.0	-1.0	2.0	2.0	13.0	2.0	2.0	-1.0	8.3	0.7	-0.7	1.0
**P.25**	4.0	1.0	0.0	0.0	5.0	5.0	15.0	5.0	3.8	-0.3	9.7	1.3	2.3	3.3
**P.50**	**9.0**	**5.0**	**5.0**	**5.0**	**7.0**	**14.0**	**24.0**	**17.0**	**6.2**	**4.3**	**18.3**	**6.3**	**6.3**	**8.0**
**P.75**	15.0	11.0	10.0	11.0	17.0	24.0	32.0	23.0	14.5	12.0	23.7	14.0	13.0	14.3
**P.80**	15.0	15.0	13.0	17.0	19.0	26.0	35.0	27.0	16.0	14.3	24.7	14.3	13.7	17.7
**P.85**	19.0	16.0	16.0	19.0	25.0	28.0	38.0	30.0	18.2	16.3	30.0	15.3	19.0	19.3
**P.90**	21.0	18.0	19.0	22.0	29.0	35.0	49.0	31.0	20.0	19.3	37.0	17.0	19.3	21.7
**P.95**	35.0	26.0	29.0	30.0	40.0	48.0	60.0	49.0	23.2	25.0	50.7	30.0	26.0	28.0
**N**	**80**	**80**	**80**	**80**	**80**	**80**	**80**	**80**	**80**	**80**	**80**	**80**	**80**	**80**
**Deft**	33.2	32.5	33.3	28.8	30.7	33.7	37.4	34.3	29.6	29.8	34.8	32.2	28.8	29.5
**Bias**	10.1	16.5	28.2	-0.1	3.6	-1.1	-33.8	-27.8	10.9	14.9	-20.9	18.3	10.5	9.0

TABLE 54 SAMPLE = NO NOISE OR ABG>0; Age group = 31-40; Occupational group = Manual; EAR = BETTER EAR; Gender = Male

	Single Frequency (kHz)								Combined Frequency (kHz)					
	0.250	0.500	1	2	3	4	6	8	4FA	3FA	HFA	LFA	A123	A124
**Mean**	**7.4**	**4.3**	**4.0**	**4.5**	**14.4**	**14.1**	**23.3**	**10.9**	**6.7**	**4.3**	**16.1**	**5.2**	**7.6**	**7.5**
**S.D.**	7.6	8.5	8.3	12.2	15.1	16.4	16.6	16.9	10.1	8.7	15.3	7.4	10.7	11.4
**Lower CI**	0.3	-2.7	-4.4	-4.5	10.2	4.1	9.0	1.7	-0.7	-3.4	7.4	-1.3	2.3	-0.2
**Upper CI**	14.6	11.3	12.5	13.4	18.7	24.2	37.6	20.1	14.2	11.9	24.9	11.8	13.0	15.3
**P.05**	-7.0	-12.0	-7.0	-6.0	-2.0	-8.0	-1.0	-7.0	-4.2	-3.3	-5.3	-5.3	-2.7	-4.3
**P.10**	-7.0	-12.0	-7.0	-6.0	-2.0	-2.0	2.0	-7.0	-2.0	-3.3	-2.0	-5.3	-2.7	-2.7
**P.15**	-1.0	-6.0	-5.0	-6.0	-1.0	-2.0	2.0	-6.0	-2.0	-3.0	-2.0	-2.7	-2.7	-2.7
**P.20**	1.0	-6.0	-3.0	-5.0	0.0	1.0	5.0	-6.0	-1.2	-2.7	1.3	-2.7	-2.0	-0.7
**P.25**	3.0	-4.0	-3.0	-4.0	0.0	2.0	5.0	-3.0	-1.2	-2.7	1.3	-1.3	-2.0	-0.7
**P.50**	**5.0**	**3.0**	**1.0**	**3.0**	**7.0**	**9.0**	**18.0**	**9.0**	**4.5**	**-0.3**	**15.7**	**1.3**	**5.0**	**4.3**
**P.75**	10.0	7.0	6.0	8.0	25.0	22.0	32.0	20.0	7.0	7.3	23.7	6.3	12.0	10.3
**P.80**	10.0	9.0	13.0	11.0	29.0	26.0	32.0	28.0	13.0	9.0	23.7	10.7	12.7	12.0
**P.85**	11.0	9.0	19.0	13.0	29.0	26.0	36.0	30.0	15.0	14.7	27.0	10.7	17.0	17.3
**P.90**	16.0	15.0	19.0	30.0	40.0	35.0	58.0	30.0	23.2	19.3	37.0	15.7	26.0	28.0
**P.95**	90.0	99.0	103.0	108.0	117.0	126.0	126.0	122.0	109.0	103.3	124.7	97.3	109.3	112.3
**N**	**24**	**24**	**24**	**24**	**24**	**24**	**24**	**24**	**24**	**24**	**24**	**24**	**24**	**24**
**Deft**	12.9	12.3	11.0	20.9	28.1	30.1	31.2	31.3	15.3	12.3	27.8	10.3	17.0	18.3
**Bias**	263.0	-243.7	-426.6	-520.5	-313.0	-439.6	-244.2	-481.2	-407.6	-396.9	-388.3	-311.1	-420.1	-462.2

KEY:
4FA = Average over 0.5, 1, 2, and 4 kHz
3FA = Average over 0.5, 1 and 2 kHz
HFA = Average over 4, 6 and 8 kHz
LFA = Average over 0.250, 0.5, and 1 kHz
A123 = Average over 1, 2, and 3 kHz
A124 = Average over 1, 2, and 4 kHz

Mean, standard deviation, confidence intervals, and percentiles for hearing thresholds as a function of ear, age group, socioeconomic group, gender, sample and frequency

TABLE 55 SAMPLE = ALL EAR = BETTER EAR

Age group = 41-50 Occupational group = Overall Gender = Overall

	SINGLE FREQUENCY (kHz)								COMBINED FREQUENCY (kHz)					
	0.250	0.500	1	2	3	4	6	8	4FA	3FA	HFA	LFA	A123	A124
**Mean**	**12.9**	**9.7**	**8.8**	**10.2**	**14.6**	**18.1**	**28.5**	**23.7**	**11.7**	**9.5**	**23.4**	**10.4**	**11.2**	**12.4**
**S.D.**	7.9	8.1	7.6	8.1	9.7	11.0	12.1	12.3	7.7	7.4	11.0	7.6	7.8	8.0
**Lower CI**	12.0	8.8	7.9	9.2	13.4	16.6	26.9	22.0	10.8	8.7	22.0	9.6	10.3	11.4
**Upper CI**	13.7	10.6	9.7	11.2	15.8	19.7	30.1	25.3	12.6	10.4	24.8	11.2	12.1	13.3
**P.05**	0.0	-1.0	-2.0	-3.0	-2.0	0.0	7.0	2.0	1.2	-0.7	6.0	0.7	-0.7	1.0
**P.10**	4.0	0.0	0.0	-1.0	1.0	2.0	10.0	6.0	2.2	1.0	8.3	2.3	1.0	2.3
**P.15**	4.0	1.0	0.0	1.0	3.0	5.0	12.0	8.0	3.2	2.0	10.0	3.0	2.7	3.0
**P.20**	5.0	2.0	1.0	2.0	4.0	6.0	15.0	9.0	4.0	2.7	12.3	3.7	3.7	3.7
**P.25**	7.0	4.0	3.0	3.0	6.0	8.0	17.0	11.0	5.0	3.3	13.0	4.7	4.3	4.7
**P.50**	**10.0**	**7.0**	**7.0**	**7.0**	**13.0**	**15.0**	**25.0**	**19.0**	**9.5**	**7.3**	**19.3**	**8.3**	**8.7**	**9.7**
**P.75**	16.0	14.0	12.0	14.0	19.0	24.0	37.0	33.0	15.5	12.0	29.7	12.7	14.7	16.3
**P.80**	18.0	15.0	14.0	16.0	22.0	27.0	40.0	37.0	17.2	14.3	33.0	14.3	17.0	19.0
**P.85**	20.0	18.0	15.0	19.0	24.0	31.0	45.0	43.0	19.5	16.7	37.7	17.3	19.3	21.0
**P.90**	24.0	21.0	19.0	25.0	31.0	40.0	52.0	48.0	23.0	19.3	45.3	20.0	22.7	26.7
**P.95**	32.0	26.0	26.0	34.0	41.0	50.0	65.0	63.0	31.0	29.3	54.3	27.0	32.3	34.3
**N**	**450**	**450**	**450**	**450**	**450**	**450**	**450**	**450**	**450**	**450**	**450**	**450**	**450**	**450**
**Deft**	50.4	52.6	51.0	45.5	47.0	45.0	46.9	44.2	51.4	52.4	48.3	53.8	49.7	48.9
**Bias**	-9.5	-12.6	-8.2	-2.6	-4.7	-11.0	5.2	-10.4	-8.6	-7.8	-5.4	-10.1	-5.2	-7.3

TABLE 56 SAMPLE = NO NOISE OR ABG>0 EAR = BETTER EAR

Age group = 41-50 Occupational group = Overall Gender = Overall

	SINGLE FREQUENCY (kHz)								COMBINED FREQUENCY (kHz)					
	0.250	0.500	1	2	3	4	6	8	4FA	3FA	HFA	LFA	A123	A124
**Mean**	**10.7**	**6.8**	**6.7**	**7.4**	**10.9**	**12.9**	**24.8**	**20.2**	**8.5**	**7.0**	**19.3**	**8.1**	**8.3**	**9.0**
**S.D.**	6.3	6.3	6.1	6.5	8.5	9.4	10.4	11.7	5.9	5.7	9.4	5.7	6.1	6.2
**Lower CI**	9.6	5.8	5.7	6.1	9.1	10.9	22.7	17.2	7.4	6.1	17.4	7.2	7.2	7.9
**Upper CI**	11.9	7.8	7.7	8.7	12.6	14.9	26.9	23.3	9.5	7.9	21.3	8.9	9.5	10.2
**P.05**	0.0	-5.0	-3.0	-4.0	-3.0	-1.0	7.0	1.0	0.2	-1.7	5.3	0.3	-1.0	0.0
**P.10**	3.0	0.0	0.0	-2.0	-1.0	0.0	10.0	4.0	1.8	0.0	6.7	1.7	-0.3	1.7
**P.15**	4.0	0.0	0.0	0.0	1.0	2.0	11.0	6.0	2.5	1.0	8.3	2.3	1.0	2.3
**P.20**	5.0	1.0	0.0	1.0	3.0	5.0	12.0	8.0	3.0	2.0	10.0	2.7	2.7	2.7
**P.25**	6.0	2.0	1.0	2.0	4.0	5.0	14.0	9.0	3.8	2.7	11.0	3.7	3.7	3.3
**P.50**	**10.0**	**5.0**	**5.0**	**6.0**	**10.0**	**10.0**	**21.0**	**15.0**	**6.8**	**6.0**	**15.3**	**7.0**	**6.7**	**7.7**
**P.75**	15.0	10.0	10.0	11.0	14.0	17.0	30.0	25.0	11.8	9.3	22.3	10.7	12.0	12.3
**P.80**	15.0	11.0	10.0	12.0	16.0	20.0	32.0	28.0	12.5	10.7	24.0	11.7	13.0	14.0
**P.85**	16.0	13.0	12.0	14.0	19.0	21.0	35.0	32.0	13.5	11.3	26.7	12.0	13.7	14.7
**P.90**	17.0	15.0	15.0	16.0	23.0	24.0	38.0	37.0	16.0	13.7	30.7	14.0	16.0	17.0
**P.95**	21.0	16.0	18.0	23.0	24.0	31.0	50.0	43.0	18.8	17.3	36.7	17.3	20.3	19.3
**N**	**206**	**206**	**206**	**206**	**206**	**206**	**206**	**206**	**206**	**206**	**206**	**206**	**206**	**206**
**Deft**	54.2	49.7	49.0	39.4	49.5	55.9	53.9	62.9	45.3	45.7	60.1	51.9	42.6	44.1
**Bias**	26.6	11.6	19.9	-31.9	24.5	50.5	102.3	169.8	12.5	-0.1	107.5	19.4	4.2	12.8

KEY: 4FA = Average over 0.5, 1, 2, and 4 kHz
3FA = Average over 0.5, 1 and 2 kHz
HFA = Average over 4, 6 and 8 kHz
LFA = Average over 0.250, 0.5, and 1 kHz
A123 = Average over 1, 2, and 3 kHz
A124 = Average over 1, 2, and 4 kHz

Mean, standard deviation, confidence intervals, and percentiles for hearing thresholds as a function of ear, age group, socioeconomic group, gender, sample and frequency

TABLE 57 SAMPLE = ALL EAR = BETTER EAR
Age group = 41-50 Occupational group = Overall Gender = Female

	SINGLE FREQUENCY (kHz)								COMBINED FREQUENCY (kHz)					
	0.250	0.500	1	2	3	4	6	8	4FA	3FA	HFA	LFA	A123	A124
**Mean**	**12.8**	**9.5**	**9.0**	**9.8**	**12.2**	**14.6**	**24.9**	**19.5**	**10.7**	**9.4**	**19.7**	**10.4**	**10.3**	**11.1**
**S.D.**	7.6	8.2	7.7	7.8	7.9	8.6	10.7	10.7	7.4	7.5	9.2	7.5	7.3	7.4
**Lower CI**	11.6	8.2	7.7	8.5	10.7	13.0	23.0	17.6	9.6	8.3	18.1	9.3	9.1	9.9
**Upper CI**	14.0	10.7	10.2	11.1	13.6	16.2	26.8	21.4	11.8	10.5	21.2	11.5	11.5	12.3
**P.05**	0.0	-4.0	-3.0	-3.0	-3.0	-1.0	7.0	1.0	0.8	-1.0	4.7	0.3	-1.0	0.7
**P.10**	3.0	0.0	0.0	0.0	-1.0	1.0	7.0	3.0	2.0	1.0	6.3	2.0	0.3	1.7
**P.15**	4.0	1.0	0.0	1.0	1.0	5.0	10.0	7.0	2.8	2.0	8.3	2.7	2.0	2.7
**P.20**	6.0	2.0	1.0	2.0	4.0	5.0	13.0	8.0	3.8	2.7	9.7	3.7	3.7	3.7
**P.25**	7.0	3.0	3.0	3.0	4.0	6.0	15.0	9.0	4.2	3.3	10.7	4.7	4.0	4.3
**P.50**	**10.0**	**7.0**	**7.0**	**7.0**	**12.0**	**11.0**	**21.0**	**16.0**	**8.2**	**7.3**	**16.0**	**8.3**	**8.0**	**9.0**
**P.75**	16.0	13.0	12.0	14.0	17.0	20.0	31.0	27.0	13.5	11.7	24.0	12.3	13.7	14.7
**P.80**	17.0	15.0	15.0	15.0	19.0	22.0	35.0	30.0	14.8	13.7	26.7	14.0	15.0	15.7
**P.85**	19.0	16.0	15.0	16.0	22.0	26.0	39.0	35.0	18.2	16.0	31.0	16.7	17.3	18.7
**P.90**	25.0	21.0	20.0	24.0	25.0	30.0	47.0	42.0	20.0	18.7	35.0	20.0	20.3	21.3
**P.95**	34.0	30.0	25.0	31.0	32.0	40.0	57.0	51.0	30.0	28.3	45.7	30.3	31.0	33.7
**N**	**247**	**247**	**247**	**247**	**247**	**247**	**247**	**247**	**247**	**247**	**247**	**247**	**247**	**247**
**Deft**	49.2	52.0	49.9	46.7	42.9	41.6	45.0	43.1	50.5	52.3	45.7	52.7	48.3	47.9
**Bias**	-5.0	-8.0	-6.8	-0.4	-1.0	-11.5	3.4	-11.5	-6.7	-5.1	-6.5	-6.6	-2.7	-6.2

TABLE 58 SAMPLE = NO NOISE OR ABG>0 EAR = BETTER EAR
Age group = 41-50 Occupational group = Overall Gender = Female

	SINGLE FREQUENCY (kHz)								COMBINED FREQUENCY (kHz)					
	0.250	0.500	1	2	3	4	6	8	4FA	3FA	HFA	LFA	A123	A124
**Mean**	**10.5**	**6.6**	**6.6**	**8.0**	**9.8**	**11.7**	**22.7**	**16.9**	**8.2**	**7.1**	**17.1**	**7.9**	**8.1**	**8.7**
**S.D.**	4.6	5.1	4.7	5.2	5.7	6.2	8.9	8.8	4.5	4.5	6.9	4.3	4.7	4.7
**Lower CI**	9.2	5.2	5.3	6.4	8.1	9.7	20.5	14.7	6.9	5.8	15.3	6.7	6.8	7.3
**Upper CI**	11.8	8.0	7.9	9.6	11.5	13.6	24.9	19.1	9.5	8.3	18.9	9.1	9.5	10.2
**P.05**	-1.0	-5.0	-4.0	-3.0	-3.0	-1.0	7.0	1.0	-0.2	-1.7	4.7	-1.0	-1.0	0.0
**P.10**	1.0	-1.0	-2.0	-1.0	-1.0	0.0	7.0	3.0	1.8	0.3	6.0	1.3	-0.3	1.3
**P.15**	4.0	0.0	0.0	1.0	1.0	1.0	10.0	5.0	2.2	1.3	7.3	2.3	1.3	2.0
**P.20**	5.0	1.0	1.0	2.0	3.0	5.0	12.0	7.0	3.0	2.3	9.3	2.7	3.0	2.7
**P.25**	6.0	2.0	3.0	2.0	4.0	5.0	14.0	8.0	3.8	2.7	10.0	3.3	3.7	3.7
**P.50**	**10.0**	**6.0**	**5.0**	**7.0**	**10.0**	**10.0**	**21.0**	**14.0**	**7.0**	**6.0**	**14.7**	**7.0**	**6.7**	**7.7**
**P.75**	15.0	10.0	10.0	12.0	14.0	17.0	29.0	22.0	12.2	10.3	21.0	11.0	12.0	13.0
**P.80**	15.0	11.0	10.0	13.0	15.0	20.0	31.0	26.0	12.5	11.0	23.0	12.0	13.3	14.3
**P.85**	16.0	13.0	12.0	15.0	18.0	21.0	34.0	28.0	13.5	12.0	25.0	13.3	13.7	14.7
**P.90**	18.0	15.0	15.0	16.0	20.0	23.0	37.0	35.0	14.8	14.3	30.7	15.0	15.3	17.0
**P.95**	21.0	18.0	19.0	23.0	24.0	26.0	50.0	42.0	19.0	17.7	36.0	17.3	19.3	19.3
**N**	**152**	**152**	**152**	**152**	**152**	**152**	**152**	**152**	**152**	**152**	**152**	**152**	**152**	**152**
**Deft**	32.1	35.3	31.3	30.2	29.6	30.8	42.9	40.2	30.4	31.7	38.3	32.1	29.8	29.5
**Bias**	-1.5	-11.5	2.7	-6.2	-15.8	-27.2	7.2	-8.9	-10.6	-5.0	-9.6	-3.5	-6.4	-10.2

KEY:
4FA = Average over 0.5, 1, 2, and 4 kHz
3FA = Average over 0.5, 1 and 2 kHz
HFA = Average over 4, 6 and 8 kHz
LFA = Average over 0.250, 0.5, and 1 kHz
A123 = Average over 1, 2, and 3 kHz
A124 = Average over 1, 2, and 4 kHz

Mean, standard deviation, confidence intervals, and percentiles for hearing thresholds as a function of ear, age group, socioeconomic group, gender, sample and frequency

TABLE 59 SAMPLE = ALL; Age group = 41-50; Occupational group = Overall; EAR = BETTER EAR; Gender = Male

	Single Frequency (kHz)								Combined Frequency (kHz)					
	0.250	0.500	1	2	3	4	6	8	4FA	3FA	HFA	LFA	A123	A124
**Mean**	**12.9**	**9.9**	**8.6**	**10.6**	**17.3**	**22.0**	**32.5**	**28.2**	**12.8**	**9.7**	**27.6**	**10.5**	**12.2**	**13.7**
**S.D.**	8.2	8.0	7.5	8.5	10.7	12.0	12.3	12.3	7.9	7.4	11.3	7.6	8.2	8.3
**Lower CI**	11.5	8.5	7.4	9.0	15.3	19.3	29.8	25.3	11.4	8.5	25.1	9.3	10.8	12.2
**Upper CI**	14.2	11.2	9.9	12.2	19.2	24.7	35.2	31.0	14.1	10.9	30.0	11.6	13.6	15.3
**P.05**	2.0	0.0	-1.0	-4.0	-1.0	1.0	10.0	5.0	1.2	-0.7	8.7	1.0	0.3	1.3
**P.10**	4.0	0.0	0.0	-1.0	3.0	5.0	12.0	7.0	2.8	0.7	12.0	2.3	2.0	2.7
**P.15**	5.0	1.0	0.0	0.0	5.0	6.0	16.0	10.0	3.5	2.3	13.0	3.3	3.0	3.3
**P.20**	5.0	2.0	1.0	2.0	7.0	9.0	19.0	12.0	4.2	2.7	14.3	4.0	3.7	4.3
**P.25**	6.0	4.0	3.0	3.0	8.0	10.0	20.0	12.0	5.0	3.3	16.0	4.7	4.7	5.0
**P.50**	**10.0**	**8.0**	**7.0**	**8.0**	**14.0**	**17.0**	**27.0**	**24.0**	**10.5**	**7.7**	**23.0**	**8.0**	**9.7**	**11.0**
**P.75**	16.0	14.0	12.0	15.0	23.0	30.0	41.0	37.0	17.0	12.7	34.3	12.7	17.0	19.0
**P.80**	19.0	15.0	14.0	17.0	24.0	35.0	45.0	45.0	19.5	15.3	40.3	15.3	18.3	21.0
**P.85**	21.0	19.0	16.0	20.0	30.0	40.0	49.0	48.0	22.2	17.0	45.3	18.3	20.7	24.0
**P.90**	23.0	22.0	19.0	26.0	38.0	49.0	57.0	58.0	24.2	20.7	50.3	20.3	23.7	29.3
**P.95**	30.0	25.0	29.0	37.0	52.0	59.0	70.0	68.0	32.2	30.0	65.3	27.0	35.3	36.3
**N**	**203**	**203**	**203**	**203**	**203**	**203**	**203**	**203**	**203**	**203**	**203**	**203**	**203**	**203**
**Deft**	51.3	53.2	52.2	44.1	46.7	42.2	43.6	39.1	51.3	52.5	44.1	55.0	50.2	48.4
**Bias**	-15.0	-18.1	-10.5	-4.0	-6.3	-6.6	10.0	-5.5	-9.8	-10.9	-0.7	-14.5	-6.9	-7.0

TABLE 60 SAMPLE = NO NOISE OR ABG>0; Age group = 41-50; Occupational group = Overall; EAR = BETTER EAR; Gender = Male

	Single Frequency (kHz)								Combined Frequency (kHz)					
	0.250	0.500	1	2	3	4	6	8	4FA	3FA	HFA	LFA	A123	A124
**Mean**	**11.1**	**7.1**	**6.8**	**6.8**	**12.2**	**14.4**	**27.2**	**24.2**	**8.8**	**6.9**	**21.9**	**8.3**	**8.6**	**9.3**
**S.D.**	7.8	7.4	7.5	7.8	10.8	12.0	11.4	13.4	7.2	6.9	11.1	7.1	7.5	7.6
**Lower CI**	9.0	5.6	5.2	4.7	9.0	10.8	23.4	18.0	7.1	5.6	18.2	7.0	6.7	7.4
**Upper CI**	13.1	8.5	8.3	8.9	15.4	18.1	31.1	30.3	10.4	8.2	25.7	9.6	10.5	11.3
**P.05**	2.0	0.0	-1.0	-6.0	-1.0	-4.0	10.0	4.0	1.2	-0.7	8.3	1.0	-1.0	-0.3
**P.10**	4.0	0.0	0.0	-4.0	-1.0	1.0	11.0	7.0	2.5	0.0	8.3	2.3	-0.3	2.3
**P.15**	5.0	0.0	0.0	-2.0	2.0	4.0	12.0	9.0	3.0	0.0	11.0	3.3	1.0	2.7
**P.20**	5.0	1.0	0.0	-1.0	3.0	5.0	14.0	11.0	3.2	1.7	12.7	3.3	2.0	3.0
**P.25**	5.0	1.0	0.0	0.0	4.0	5.0	17.0	11.0	3.8	2.3	12.7	4.0	2.7	3.3
**P.50**	**10.0**	**5.0**	**5.0**	**6.0**	**11.0**	**10.0**	**26.0**	**20.0**	**6.0**	**5.0**	**17.0**	**6.7**	**7.0**	**6.7**
**P.75**	11.0	10.0	10.0	10.0	14.0	19.0	32.0	29.0	10.2	8.3	25.3	8.7	12.0	11.3
**P.80**	15.0	10.0	10.0	11.0	17.0	20.0	35.0	32.0	11.2	9.3	26.3	10.0	13.0	12.3
**P.85**	15.0	11.0	12.0	13.0	23.0	20.0	37.0	37.0	14.2	10.7	29.7	11.7	13.7	14.3
**P.90**	16.0	15.0	14.0	15.0	24.0	29.0	40.0	37.0	16.0	11.7	31.3	11.7	17.0	18.0
**P.95**	16.0	15.0	16.0	19.0	28.0	44.0	47.0	48.0	18.8	17.0	45.0	14.3	20.7	20.0
**N**	**54**	**54**	**54**	**54**	**54**	**54**	**54**	**54**	**54**	**54**	**54**	**54**	**54**	**54**
**Deft**	69.1	59.4	60.4	40.2	51.4	60.3	56.8	68.5	51.2	52.6	63.6	64.0	46.1	49.4
**Bias**	62.2	40.8	47.6	-29.7	1.8	89.4	93.3	215.1	37.0	19.6	132.6	50.2	6.6	35.8

KEY:
4FA = Average over 0.5, 1, 2, and 4 kHz
3FA = Average over 0.5, 1 and 2 kHz
HFA = Average over 4, 6 and 8 kHz
LFA = Average over 0.250, 0.5, and 1 kHz
A123 = Average over 1, 2, and 3 kHz
A124 = Average over 1, 2, and 4 kHz

Mean, standard deviation, confidence intervals, and percentiles for hearing thresholds as a function of ear, age group, socioeconomic group, gender, sample and frequency

TABLE 61 SAMPLE = ALL EAR = BETTER EAR

Age group = 41-50 Occupational group = Non-Manual Gender = Overall

	Single Frequency (kHz)								Combined Frequency (kHz)					
	0.250	0.500	1	2	3	4	6	8	4FA	3FA	HFA	LFA	A123	A124
**Mean**	**11.2**	**7.6**	**7.4**	**8.5**	**12.0**	**15.1**	**26.4**	**21.6**	**9.6**	**7.8**	**21.0**	**8.7**	**9.3**	**10.3**
**S.D.**	6.7	6.5	6.6	8.0	9.9	11.2	12.7	13.2	7.0	6.4	11.4	6.1	7.5	7.6
**Lower CI**	9.9	6.2	5.9	6.7	10.3	13.2	24.5	19.1	8.3	6.4	19.3	7.5	7.8	8.9
**Upper CI**	12.4	9.0	8.9	10.3	13.8	16.9	28.3	24.0	11.0	9.2	22.7	9.9	10.8	11.7
**P.05**	-1.0	-1.0	-2.0	-4.0	-2.0	-1.0	7.0	1.0	0.8	-0.7	5.0	1.0	-1.3	0.7
**P.10**	3.0	0.0	0.0	-1.0	-1.0	0.0	10.0	5.0	2.0	0.0	6.3	2.0	0.3	1.3
**P.15**	4.0	1.0	0.0	0.0	1.0	1.0	11.0	6.0	2.8	1.3	8.7	2.3	2.0	2.3
**P.20**	4.0	1.0	0.0	1.0	3.0	5.0	14.0	7.0	3.2	2.7	10.0	3.3	3.0	2.7
**P.25**	5.0	2.0	1.0	3.0	4.0	6.0	16.0	9.0	3.8	3.3	12.0	4.3	3.7	3.7
**P.50**	**10.0**	**5.0**	**5.0**	**6.0**	**10.0**	**11.0**	**22.0**	**16.0**	**7.0**	**5.7**	**17.0**	**6.7**	**6.7**	**7.7**
**P.75**	15.0	10.0	10.0	11.0	14.0	19.0	31.0	28.0	11.8	9.3	24.0	10.0	12.0	12.3
**P.80**	15.0	11.0	11.0	12.0	17.0	20.0	35.0	31.0	13.0	10.3	26.7	11.0	13.0	14.3
**P.85**	16.0	14.0	12.0	15.0	19.0	22.0	39.0	39.0	14.5	12.0	31.0	11.7	14.7	15.7
**P.90**	18.0	16.0	15.0	16.0	23.0	29.0	45.0	45.0	18.8	15.0	36.0	15.0	17.0	19.3
**P.95**	23.0	21.0	20.0	23.0	28.0	36.0	55.0	50.0	22.8	19.0	43.0	19.7	20.3	27.3
**N**	**188**	**188**	**188**	**188**	**188**	**188**	**188**	**188**	**188**	**188**	**188**	**188**	**188**	**188**
**Deft**	81.1	79.4	81.4	74.6	80.2	74.2	71.6	67.1	88.1	89.3	78.6	92.7	85.5	83.1
**Bias**	100.0	83.7	69.6	94.5	115.1	146.7	128.1	106.9	98.6	82.6	127.2	84.5	93.1	103.6

TABLE 62 SAMPLE = NO NOISE OR ABG>0 EAR = BETTER EAR

Age group = 41-50 Occupational group = Non-Manual Gender = Overall

	Single Frequency (kHz)								Combined Frequency (kHz)					
	0.250	0.500	1	2	3	4	6	8	4FA	3FA	HFA	LFA	A123	A124
**Mean**	**10.0**	**5.7**	**6.3**	**7.0**	**9.8**	**10.8**	**23.4**	**17.5**	**7.4**	**6.3**	**17.2**	**7.3**	**7.7**	**8.0**
**S.D.**	4.9	5.5	5.0	7.1	8.6	10.0	10.5	10.8	5.2	4.8	9.4	4.5	6.0	5.9
**Lower CI**	8.9	4.8	5.2	4.9	8.0	8.7	20.9	15.2	6.3	5.4	15.4	6.6	6.4	6.6
**Upper CI**	11.0	6.6	7.5	9.0	11.6	13.0	25.8	19.9	8.6	7.3	19.1	8.1	9.0	9.5
**P.05**	0.0	-4.0	-3.0	-5.0	-2.0	-1.0	7.0	1.0	0.8	-1.7	4.7	0.7	-1.7	0.0
**P.10**	2.0	0.0	0.0	-1.0	-1.0	0.0	9.0	3.0	2.0	0.0	6.3	2.0	-0.3	1.7
**P.15**	4.0	0.0	0.0	0.0	1.0	1.0	10.0	5.0	2.5	1.7	6.7	2.3	2.0	2.7
**P.20**	4.0	1.0	0.0	1.0	3.0	5.0	12.0	7.0	3.2	2.3	9.3	2.7	3.0	2.7
**P.25**	5.0	1.0	1.0	2.0	4.0	5.0	14.0	8.0	3.8	2.7	10.3	3.3	3.7	3.3
**P.50**	**9.0**	**5.0**	**5.0**	**6.0**	**9.0**	**9.0**	**21.0**	**15.0**	**6.2**	**5.0**	**14.7**	**6.0**	**6.3**	**6.7**
**P.75**	14.0	8.0	10.0	11.0	14.0	16.0	27.0	22.0	10.0	8.3	20.7	9.0	11.7	10.7
**P.80**	15.0	9.0	10.0	12.0	14.0	17.0	31.0	27.0	11.0	9.3	22.7	10.0	12.0	11.7
**P.85**	15.0	10.0	12.0	13.0	16.0	20.0	32.0	29.0	12.2	10.0	24.0	11.0	13.3	14.3
**P.90**	16.0	12.0	14.0	16.0	19.0	22.0	37.0	37.0	14.2	12.0	30.7	12.3	13.7	15.7
**P.95**	17.0	16.0	19.0	17.0	23.0	26.0	47.0	42.0	18.5	17.7	37.3	17.0	18.0	19.0
**N**	**113**	**113**	**113**	**113**	**113**	**113**	**113**	**113**	**113**	**113**	**113**	**113**	**113**	**113**
**Deft**	62.7	76.9	54.6	63.2	69.8	83.7	60.9	59.8	65.8	63.3	72.6	71.5	62.1	64.1
**Bias**	53.0	34.9	6.6	7.7	53.6	8.6	40.8	5.8	14.5	16.4	18.4	31.5	22.6	7.6

KEY:
4FA = Average over 0.5, 1, 2, and 4 kHz
3FA = Average over 0.5, 1 and 2 kHz
HFA = Average over 4, 6 and 8 kHz
LFA = Average over 0.250, 0.5, and 1 kHz
A123 = Average over 1, 2, and 3 kHz
A124 = Average over 1, 2, and 4 kHz

Mean, standard deviation, confidence intervals, and percentiles for hearing thresholds as a function of ear, age group, socioeconomic group, gender, sample and frequency

TABLE 63 SAMPLE = ALL — EAR = BETTER EAR
Age group = 41-50 — Occupational group = Non-Manual — Gender = Female

	Single Frequency (kHz)								Combined Frequency (kHz)					
	0.250	0.500	1	2	3	4	6	8	4FA	3FA	HFA	LFA	A123	A124
**Mean**	**11.0**	**7.3**	**7.3**	**8.3**	**10.6**	**12.6**	**23.0**	**18.7**	**8.9**	**7.6**	**18.1**	**8.5**	**8.7**	**9.4**
**S.D.**	6.1	6.6	6.4	6.8	6.8	7.7	10.7	11.9	6.0	6.0	9.2	5.9	6.0	6.3
**Lower CI**	9.7	6.0	6.1	7.0	9.0	10.9	20.4	16.0	7.9	6.6	16.2	7.5	7.6	8.3
**Upper CI**	12.2	8.7	8.6	9.6	12.2	14.3	25.7	21.4	9.9	8.6	20.0	9.6	9.9	10.5
**P.05**	0.0	-4.0	-3.0	-3.0	-3.0	-1.0	5.0	0.0	0.8	-1.0	4.0	0.7	-1.7	1.0
**P.10**	3.0	-1.0	-2.0	-1.0	-1.0	0.0	7.0	3.0	2.0	1.0	5.3	1.7	1.0	1.3
**P.15**	4.0	1.0	0.0	1.0	1.0	1.0	8.0	5.0	2.5	1.7	6.3	2.3	2.0	2.0
**P.20**	5.0	1.0	0.0	2.0	3.0	5.0	10.0	7.0	3.5	2.7	9.0	3.0	3.0	2.7
**P.25**	6.0	2.0	1.0	3.0	4.0	6.0	13.0	8.0	3.8	3.3	9.3	4.0	3.7	3.7
**P.50**	**10.0**	**5.0**	**6.0**	**6.0**	**10.0**	**11.0**	**19.0**	**15.0**	**7.0**	**6.0**	**15.0**	**6.7**	**7.0**	**7.7**
**P.75**	15.0	10.0	11.0	12.0	14.0	17.0	27.0	23.0	11.0	9.7	20.7	10.7	12.0	13.0
**P.80**	15.0	11.0	11.0	12.0	16.0	19.0	30.0	28.0	12.5	10.3	23.7	11.3	12.3	14.3
**P.85**	16.0	15.0	14.0	14.0	18.0	20.0	32.0	33.0	14.2	12.0	25.7	13.0	13.7	14.7
**P.90**	18.0	16.0	18.0	16.0	20.0	22.0	39.0	42.0	16.5	16.3	31.0	17.0	16.0	15.7
**P.95**	25.0	21.0	20.0	22.0	23.0	29.0	55.0	50.0	19.8	18.7	40.7	21.7	19.3	19.3
**N**	**112**	**112**	**112**	**112**	**112**	**112**	**112**	**112**	**112**	**112**	**112**	**112**	**112**	**112**
**Deft**	69.5	76.0	74.5	69.7	56.5	63.0	56.1	63.5	82.2	84.4	67.3	84.9	71.5	76.2
**Bias**	53.9	62.0	44.7	65.2	63.3	74.9	110.5	62.3	61.7	57.3	82.6	53.5	57.7	61.6

TABLE 64 SAMPLE = NO NOISE OR ABG>0 — EAR = BETTER EAR
Age group = 41-50 — Occupational group = Non-Manual — Gender = Female

	Single Frequency (kHz)								Combined Frequency (kHz)					
	0.250	0.500	1	2	3	4	6	8	4FA	3FA	HFA	LFA	A123	A124
**Mean**	**10.2**	**6.4**	**6.9**	**7.7**	**9.6**	**11.4**	**22.7**	**17.6**	**8.1**	**7.0**	**17.2**	**7.8**	**8.1**	**8.7**
**S.D.**	5.7	6.9	5.6	5.2	5.6	6.7	9.8	10.9	5.2	5.2	8.3	5.6	4.9	5.2
**Lower CI**	8.8	5.1	5.4	6.2	7.9	9.6	19.9	14.8	7.0	5.9	15.3	6.8	6.8	7.4
**Upper CI**	11.5	7.7	8.4	9.1	11.3	13.2	25.5	20.3	9.2	8.1	19.1	8.9	9.3	9.9
**P.05**	-1.0	-6.0	-3.0	-3.0	-2.0	-1.0	6.0	1.0	0.5	-2.0	4.7	0.7	-0.7	0.7
**P.10**	1.0	-1.0	-2.0	0.0	-1.0	0.0	7.0	1.0	1.8	0.0	5.7	1.3	1.0	1.7
**P.15**	3.0	0.0	0.0	1.0	2.0	1.0	9.0	4.0	2.5	1.7	6.3	2.3	2.0	2.7
**P.20**	4.0	1.0	0.0	2.0	3.0	5.0	12.0	6.0	3.2	2.3	9.0	2.3	3.7	3.3
**P.25**	5.0	2.0	1.0	3.0	4.0	5.0	13.0	7.0	3.8	2.7	10.0	2.7	3.7	3.7
**P.50**	**9.0**	**5.0**	**5.0**	**6.0**	**9.0**	**10.0**	**20.0**	**15.0**	**7.0**	**5.7**	**14.7**	**6.0**	**6.7**	**7.7**
**P.75**	14.0	9.0	10.0	12.0	14.0	16.0	27.0	22.0	11.0	9.7	20.7	10.0	11.7	11.7
**P.80**	15.0	10.0	11.0	12.0	14.0	17.0	31.0	25.0	12.2	10.3	21.0	11.0	12.3	14.0
**P.85**	15.0	11.0	14.0	14.0	16.0	20.0	32.0	30.0	13.2	11.3	24.0	12.3	13.7	14.7
**P.90**	16.0	15.0	16.0	16.0	19.0	21.0	37.0	40.0	14.5	13.7	30.7	15.0	13.7	15.7
**P.95**	18.0	16.0	20.0	20.0	23.0	24.0	56.0	43.0	19.0	18.7	38.3	17.3	18.7	19.3
**N**	**81**	**81**	**81**	**81**	**81**	**81**	**81**	**81**	**81**	**81**	**81**	**81**	**81**	**81**
**Deft**	80.7	102.8	67.3	54.6	51.7	60.2	54.3	60.1	81.5	82.1	64.7	100.5	62.0	68.3
**Bias**	63.9	77.8	41.4	24.0	29.5	46.4	59.6	32.7	47.4	47.8	46.3	61.1	31.7	37.3

KEY: 4FA = Average over 0.5, 1, 2, and 4 kHz
3FA = Average over 0.5, 1 and 2 kHz
HFA = Average over 4, 6 and 8 kHz
LFA = Average over 0.250, 0.5, and 1 kHz
A123 = Average over 1, 2, and 3 kHz
A124 = Average over 1, 2, and 4 kHz

Mean, standard deviation, confidence intervals, and percentiles for hearing thresholds as a function of ear, age group, socioeconomic group, gender, sample and frequency

TABLE 65 SAMPLE = ALL EAR = BETTER EAR
Age group = 41-50 Occupational group = Non-Manual Gender = Male

	SINGLE FREQUENCY (kHz)								COMBINED FREQUENCY (kHz)					
	0.250	0.500	1	2	3	4	6	8	4FA	3FA	HFA	LFA	A123	A124
**Mean**	**11.4**	**7.9**	**7.4**	**8.8**	**13.6**	**17.8**	**30.0**	**24.7**	**10.5**	**8.0**	**24.2**	**8.9**	**9.9**	**11.3**
**S.D.**	7.3	6.3	6.8	9.1	12.2	13.6	13.5	13.8	7.8	6.8	12.6	6.3	8.8	8.8
**Lower CI**	9.2	5.4	4.6	5.3	10.4	14.4	27.3	20.5	7.9	5.3	21.4	6.6	7.0	8.6
**Upper CI**	13.7	10.4	10.2	12.2	16.8	21.1	32.8	28.9	13.0	10.7	26.9	11.2	12.9	14.0
**P.05**	-1.0	0.0	-1.0	-5.0	-1.0	-5.0	11.0	4.0	0.5	-0.7	6.7	1.3	-0.7	-0.3
**P.10**	4.0	0.0	0.0	-3.0	-1.0	0.0	14.0	6.0	1.8	0.0	8.3	2.0	0.3	1.0
**P.15**	4.0	0.0	0.0	-1.0	2.0	1.0	17.0	6.0	2.8	0.7	11.0	3.0	2.3	2.7
**P.20**	4.0	1.0	0.0	1.0	3.0	5.0	17.0	9.0	3.2	2.7	13.0	3.3	2.7	3.0
**P.25**	5.0	3.0	1.0	1.0	4.0	6.0	20.0	11.0	3.8	3.3	14.3	4.3	3.0	3.7
**P.50**	**9.0**	**5.0**	**5.0**	**6.0**	**10.0**	**12.0**	**26.0**	**21.0**	**7.5**	**5.3**	**19.7**	**7.3**	**6.3**	**7.7**
**P.75**	15.0	8.0	10.0	11.0	14.0	21.0	37.0	32.0	12.5	8.7	28.0	9.0	12.7	12.3
**P.80**	15.0	10.0	10.0	12.0	18.0	25.0	40.0	37.0	13.0	9.3	31.3	10.0	14.3	15.0
**P.85**	16.0	11.0	11.0	16.0	23.0	31.0	42.0	45.0	18.8	12.3	33.7	11.7	17.0	19.3
**P.90**	16.0	15.0	12.0	20.0	25.0	35.0	47.0	47.0	21.0	13.3	41.0	13.7	18.3	21.7
**P.95**	21.0	21.0	19.0	23.0	38.0	50.0	51.0	51.0	23.8	19.3	49.7	17.0	22.0	28.0
**N**	**76**	**76**	**76**	**76**	**76**	**76**	**76**	**76**	**76**	**76**	**76**	**76**	**76**	**76**
**Deft**	90.4	84.4	89.1	74.6	86.6	70.9	82.7	64.7	87.3	91.9	79.4	100.5	90.0	81.8
**Bias**	159.1	106.3	101.0	132.3	156.0	194.9	83.8	107.4	133.6	113.2	128.7	122.1	129.8	142.7

TABLE 66 SAMPLE = NO NOISE OR ABG>0 EAR = BETTER EAR
Age group = 41-50 Occupational group = Non-Manual Gender = Male

	SINGLE FREQUENCY (kHz)								COMBINED FREQUENCY (kHz)					
	0.250	0.500	1	2	3	4	6	8	4FA	3FA	HFA	LFA	A123	A124
**Mean**	**9.8**	**4.7**	**5.6**	**6.1**	**10.2**	**10.4**	**24.4**	**17.7**	**6.7**	**5.5**	**17.5**	**6.7**	**7.3**	**7.4**
**S.D.**	3.7	2.8	4.0	9.1	11.7	13.3	11.6	11.0	5.4	4.2	11.1	2.5	7.4	7.0
**Lower CI**	8.2	3.6	3.8	1.7	6.7	6.2	20.3	13.6	4.6	3.8	14.1	5.6	4.7	4.6
**Upper CI**	11.4	5.9	7.4	10.5	13.8	14.6	28.5	21.7	8.9	7.2	20.9	7.9	9.9	10.2
**P.05**	1.0	0.0	0.0	-6.0	-1.0	-4.0	10.0	3.0	1.2	-0.7	6.3	2.0	-2.3	-0.3
**P.10**	4.0	0.0	0.0	-6.0	-1.0	0.0	12.0	4.0	2.5	0.0	8.3	2.3	-0.7	2.0
**P.15**	4.0	0.0	0.0	-4.0	-1.0	0.0	12.0	6.0	3.0	0.0	8.3	3.3	-0.3	2.3
**P.20**	5.0	1.0	0.0	-1.0	2.0	1.0	14.0	9.0	3.0	1.7	9.3	3.3	2.0	2.7
**P.25**	5.0	1.0	0.0	0.0	3.0	5.0	17.0	9.0	3.5	3.3	11.0	5.0	2.7	3.0
**P.50**	**9.0**	**5.0**	**5.0**	**5.0**	**9.0**	**6.0**	**22.0**	**12.0**	**5.0**	**5.0**	**14.7**	**6.0**	**5.7**	**5.0**
**P.75**	12.0	6.0	9.0	9.0	14.0	15.0	31.0	26.0	8.0	7.0	19.7	7.7	8.7	9.3
**P.80**	15.0	6.0	10.0	10.0	14.0	15.0	32.0	29.0	8.5	7.7	24.0	8.3	8.7	10.0
**P.85**	15.0	6.0	10.0	11.0	14.0	20.0	37.0	29.0	9.5	7.7	24.7	8.7	12.0	11.0
**P.90**	16.0	8.0	10.0	14.0	23.0	25.0	40.0	32.0	10.5	9.3	31.3	10.0	13.7	12.3
**P.95**	16.0	10.0	14.0	17.0	23.0	29.0	42.0	37.0	14.2	9.3	32.7	12.3	17.0	19.0
**N**	**32**	**32**	**32**	**32**	**32**	**32**	**32**	**32**	**32**	**32**	**32**	**32**	**32**	**32**
**Deft**	39.7	32.7	38.8	58.3	66.2	81.5	71.8	62.5	48.5	42.2	77.0	29.3	54.7	54.2
**Bias**	53.6	12.8	-15.9	40.8	104.0	12.1	-37.8	-29.5	12.5	12.6	-18.4	16.8	43.0	12.3

KEY: 4FA = Average over 0.5, 1, 2, and 4 kHz; 3FA = Average over 0.5, 1 and 2 kHz
HFA = Average over 4, 6 and 8 kHz; LFA = Average over 0.250, 0.5, and 1 kHz
A123 = Average over 1, 2, and 3 kHz; A124 = Average over 1, 2, and 4 kHz

Mean, standard deviation, confidence intervals, and percentiles for hearing thresholds as a function of ear, age group, socioeconomic group, gender, sample and frequency

TABLE 67 SAMPLE = ALL EAR = BETTER EAR
Age group = 41-50 Occupational group = Manual Gender = Overall

	SINGLE FREQUENCY (kHz)								COMBINED FREQUENCY (kHz)					
	0.250	0.500	1	2	3	4	6	8	4FA	3FA	HFA	LFA	A123	A124
**Mean**	**14.4**	**11.3**	**9.9**	**11.8**	**16.7**	**21.0**	**30.3**	**25.2**	**13.5**	**11.0**	**25.5**	**11.9**	**12.8**	**14.2**
**S.D.**	8.7	9.1	8.4	8.6	10.3	11.8	12.7	12.9	8.2	8.1	11.6	8.4	8.3	8.5
**Lower CI**	13.1	9.9	8.6	10.2	14.9	18.6	27.7	22.7	12.2	9.7	23.3	10.6	11.4	12.8
**Upper CI**	15.7	12.7	11.2	13.4	18.5	23.4	32.8	27.7	14.8	12.2	27.6	13.1	14.2	15.7
**P.05**	1.0	-2.0	-1.0	-2.0	-1.0	2.0	7.0	2.0	1.5	0.0	7.7	0.3	-0.3	1.7
**P.10**	4.0	0.0	0.0	0.0	3.0	5.0	11.0	7.0	2.5	1.0	10.0	2.7	1.7	2.7
**P.15**	5.0	2.0	1.0	1.0	5.0	6.0	14.0	8.0	4.5	2.3	12.0	3.3	3.7	3.7
**P.20**	6.0	3.0	2.0	3.0	7.0	9.0	16.0	11.0	5.5	3.0	13.0	4.7	4.7	5.3
**P.25**	8.0	5.0	4.0	4.0	9.0	10.0	17.0	12.0	6.2	4.7	14.7	6.0	6.0	6.3
**P.50**	**12.0**	**10.0**	**8.0**	**9.0**	**14.0**	**17.0**	**27.0**	**22.0**	**12.0**	**9.3**	**22.3**	**10.0**	**11.0**	**12.3**
**P.75**	19.0	16.0	15.0	16.0	23.0	30.0	40.0	36.0	18.0	15.7	33.3	15.7	17.7	19.3
**P.80**	21.0	18.0	15.0	19.0	25.0	32.0	45.0	38.0	19.5	16.7	38.0	18.3	20.0	21.3
**P.85**	23.0	21.0	18.0	24.0	30.0	40.0	51.0	46.0	23.0	19.3	43.7	20.0	22.0	24.7
**P.90**	28.0	25.0	23.0	30.0	36.0	45.0	58.0	55.0	29.2	24.7	50.3	24.0	30.0	31.0
**P.95**	40.0	41.0	38.0	39.0	49.0	56.0	68.0	68.0	38.2	36.7	61.7	39.7	37.3	39.7
**N**	**262**	**262**	**262**	**262**	**262**	**262**	**262**	**262**	**262**	**262**	**262**	**262**	**262**	**262**
**Deft**	44.8	48.0	46.0	40.1	43.7	43.8	43.8	42.7	45.1	45.8	45.1	47.4	43.8	43.5
**Bias**	-74.6	-91.4	-71.9	-56.0	-100.5	-90.7	-88.9	-116.6	-77.5	-73.1	-98.8	-79.3	-76.1	-72.9

TABLE 68 SAMPLE = NO NOISE OR ABG>0 EAR = BETTER EAR
Age group = 41-50 Occupational group = Manual Gender = Overall

	SINGLE FREQUENCY (kHz)								COMBINED FREQUENCY (kHz)					
	0.250	0.500	1	2	3	4	6	8	4FA	3FA	HFA	LFA	A123	A124
**Mean**	**11.5**	**7.3**	**6.0**	**7.8**	**11.3**	**13.8**	**24.9**	**22.1**	**8.7**	**7.0**	**20.3**	**8.3**	**8.4**	**9.2**
**S.D.**	7.6	7.5	6.8	6.7	8.5	8.6	9.9	13.0	6.5	6.4	9.0	6.8	6.5	6.4
**Lower CI**	10.0	5.6	4.5	5.1	8.4	11.1	21.4	19.4	7.0	5.4	17.7	7.1	6.4	7.2
**Upper CI**	13.0	9.1	7.5	10.5	14.2	16.4	28.4	24.8	10.4	8.7	22.8	9.5	10.4	11.1
**P.05**	0.0	-5.0	-5.0	-3.0	-6.0	-1.0	7.0	2.0	-0.2	-1.7	7.0	-1.3	-1.0	0.0
**P.10**	4.0	0.0	-1.0	-2.0	0.0	2.0	10.0	6.0	1.8	0.3	8.3	1.0	-0.3	1.3
**P.15**	5.0	0.0	1.0	0.0	1.0	4.0	11.0	8.0	2.2	1.0	10.0	2.7	1.0	2.0
**P.20**	6.0	2.0	1.0	1.0	3.0	5.0	12.0	9.0	2.8	1.3	11.0	2.7	1.7	2.7
**P.25**	6.0	3.0	1.0	2.0	4.0	6.0	16.0	10.0	3.8	2.3	11.7	3.7	3.7	3.3
**P.50**	**10.0**	**8.0**	**5.0**	**7.0**	**12.0**	**11.0**	**22.0**	**15.0**	**8.5**	**7.3**	**16.7**	**8.0**	**8.0**	**8.7**
**P.75**	15.0	12.0	10.0	12.0	16.0	20.0	31.0	27.0	13.5	11.0	24.7	11.7	13.0	14.3
**P.80**	16.0	13.0	10.0	14.0	19.0	21.0	35.0	29.0	13.5	11.7	26.3	12.0	13.7	14.7
**P.85**	18.0	15.0	12.0	15.0	22.0	24.0	36.0	33.0	15.5	12.3	29.7	13.3	15.0	17.0
**P.90**	20.0	16.0	15.0	17.0	24.0	26.0	40.0	37.0	17.2	16.0	30.7	14.0	18.3	18.0
**P.95**	21.0	20.0	16.0	26.0	29.0	40.0	50.0	45.0	18.8	17.0	36.7	19.3	20.7	20.0
**N**	**93**	**93**	**93**	**93**	**93**	**93**	**93**	**93**	**93**	**93**	**93**	**93**	**93**	**93**
**Deft**	51.3	45.7	38.9	31.0	37.4	36.4	43.4	68.8	35.5	36.5	45.6	43.3	33.6	32.9
**Bias**	-26.2	-109.8	-77.1	-109.1	-100.2	-71.9	6.1	216.9	-92.0	-98.6	50.4	-71.0	-95.4	-86.0

KEY:
4FA = Average over 0.5, 1, 2, and 4 kHz
3FA = Average over 0.5, 1 and 2 kHz
HFA = Average over 4, 6 and 8 kHz
LFA = Average over 0.250, 0.5, and 1 kHz
A123 = Average over 1, 2, and 3 kHz
A124 = Average over 1, 2, and 4 kHz

Mean, standard deviation, confidence intervals, and percentiles for hearing thresholds as a function of ear, age group, socioeconomic group, gender, sample and frequency

TABLE 69 SAMPLE = ALL EAR = BETTER EAR
Age group = 41-50 Occupational group = Manual Gender = Female

	SINGLE FREQUENCY (kHz)								COMBINED FREQUENCY (kHz)					
	0.250	0.500	1	2	3	4	6	8	4FA	3FA	HFA	LFA	A123	A124
**Mean**	**14.5**	**11.6**	**10.5**	**11.4**	**13.7**	**16.6**	**26.7**	**20.2**	**12.5**	**11.2**	**21.2**	**12.2**	**11.8**	**12.8**
**S.D.**	9.2	9.6	9.0	8.8	9.2	9.9	11.2	11.0	8.6	8.7	9.9	9.0	8.5	8.6
**Lower CI**	12.7	9.7	8.5	9.2	11.3	14.0	24.0	17.4	10.7	9.4	18.9	10.4	9.9	10.9
**Upper CI**	16.4	13.6	12.4	13.5	16.1	19.2	29.4	22.9	14.3	12.9	23.5	14.0	13.8	14.8
**P.05**	0.0	-2.0	-3.0	-2.0	-3.0	-1.0	7.0	2.0	1.0	-0.7	6.0	-1.3	-0.7	0.7
**P.10**	4.0	1.0	0.0	0.0	-1.0	3.0	10.0	5.0	1.8	1.0	7.7	2.7	0.3	1.7
**P.15**	5.0	2.0	1.0	1.0	1.0	5.0	12.0	8.0	3.0	2.0	9.7	3.0	1.7	3.0
**P.20**	7.0	4.0	3.0	3.0	4.0	6.0	16.0	8.0	5.0	3.0	11.0	5.0	4.0	4.3
**P.25**	8.0	5.0	4.0	4.0	5.0	7.0	16.0	9.0	5.5	4.3	12.3	6.7	4.7	6.0
**P.50**	**12.0**	**10.0**	**8.0**	**9.0**	**13.0**	**15.0**	**23.0**	**17.0**	**11.0**	**9.3**	**18.0**	**9.7**	**9.0**	**10.7**
**P.75**	18.0	15.0	15.0	15.0	19.0	25.0	35.0	28.0	15.5	14.3	28.3	14.0	15.3	17.3
**P.80**	20.0	16.0	15.0	19.0	22.0	26.0	39.0	31.0	18.5	16.0	30.7	16.7	18.0	19.3
**P.85**	24.0	20.0	19.0	25.0	25.0	30.0	47.0	35.0	20.0	17.7	33.7	19.3	20.3	21.3
**P.90**	31.0	26.0	24.0	28.0	29.0	36.0	51.0	43.0	27.2	25.0	39.3	25.3	26.3	27.3
**P.95**	45.0	45.0	40.0	35.0	35.0	45.0	60.0	57.0	38.5	40.0	52.3	46.7	36.3	38.0
**N**	**135**	**135**	**135**	**135**	**135**	**135**	**135**	**135**	**135**	**135**	**135**	**135**	**135**	**135**
**Deft**	50.6	49.7	48.5	43.2	42.5	41.0	43.5	39.9	47.3	48.6	42.3	50.7	45.7	45.3
**Bias**	-55.5	-52.6	-49.1	-47.6	-49.5	-67.5	-87.0	-87.8	-54.2	-49.8	-80.8	-52.4	-48.7	-54.8

TABLE 70 SAMPLE = NO NOISE OR ABG>0 EAR = BETTER EAR
Age group = 41-50 Occupational group = Manual Gender = Female

	SINGLE FREQUENCY (kHz)								COMBINED FREQUENCY (kHz)					
	0.250	0.500	1	2	3	4	6	8	4FA	3FA	HFA	LFA	A123	A124
**Mean**	**11.6**	**7.7**	**6.7**	**8.5**	**10.2**	**12.2**	**22.9**	**16.5**	**8.8**	**7.7**	**17.2**	**8.7**	**8.5**	**9.2**
**S.D.**	6.1	5.9	5.2	6.3	7.4	7.8	8.7	8.0	5.5	5.2	6.8	5.3	5.8	5.7
**Lower CI**	9.6	5.9	4.8	5.8	7.2	9.1	19.8	13.3	6.7	5.8	14.6	7.0	6.2	6.8
**Upper CI**	13.5	9.5	8.7	11.3	13.3	15.4	25.9	19.8	10.9	9.6	19.9	10.4	10.8	11.5
**P.05**	-1.0	-5.0	-5.0	-3.0	-7.0	-2.0	7.0	2.0	-0.2	-1.3	6.7	-2.0	-1.0	0.0
**P.10**	0.0	0.0	1.0	-1.0	-3.0	0.0	9.0	3.0	1.5	0.3	7.3	1.3	-0.3	1.3
**P.15**	4.0	1.0	1.0	0.0	1.0	3.0	10.0	6.0	1.8	1.0	8.3	2.7	0.3	1.7
**P.20**	5.0	2.0	2.0	1.0	2.0	4.0	13.0	8.0	2.5	1.7	10.0	2.7	1.7	2.3
**P.25**	6.0	3.0	3.0	2.0	4.0	5.0	16.0	8.0	3.0	2.7	10.7	3.7	3.7	3.3
**P.50**	**12.0**	**7.0**	**5.0**	**7.0**	**10.0**	**11.0**	**21.0**	**13.0**	**7.8**	**7.0**	**14.7**	**8.0**	**7.0**	**8.0**
**P.75**	15.0	11.0	10.0	12.0	15.0	21.0	29.0	23.0	12.5	11.0	23.0	12.0	13.0	13.3
**P.80**	17.0	13.0	10.0	14.0	17.0	21.0	33.0	26.0	13.5	11.0	24.0	12.7	13.7	14.7
**P.85**	18.0	15.0	10.0	15.0	19.0	24.0	35.0	28.0	13.5	12.3	25.0	13.7	15.0	16.3
**P.90**	20.0	15.0	15.0	20.0	22.0	26.0	37.0	33.0	16.8	16.0	30.7	14.0	15.3	17.3
**P.95**	21.0	21.0	15.0	25.0	25.0	31.0	47.0	40.0	19.2	17.0	35.0	18.3	19.7	20.3
**N**	**71**	**71**	**71**	**71**	**71**	**71**	**71**	**71**	**71**	**71**	**71**	**71**	**71**	**71**
**Deft**	39.0	34.0	27.2	28.3	31.3	33.1	39.1	33.7	28.6	27.9	31.9	32.0	28.5	28.0
**Bias**	-7.8	-27.1	7.1	-26.0	-44.9	-83.6	-52.8	-22.6	-32.4	-15.3	-53.0	-9.3	-21.3	-34.2

KEY: 4FA = Average over 0.5, 1, 2, and 4 kHz 3FA = Average over 0.5, 1 and 2 kHz
HFA = Average over 4, 6 and 8 kHz LFA = Average over 0.250, 0.5, and 1 kHz
A123 = Average over 1, 2, and 3 kHz A124 = Average over 1, 2, and 4 kHz

Mean, standard deviation, confidence intervals, and percentiles for hearing thresholds as a function of ear, age group, socioeconomic group, gender, sample and frequency

TABLE 71 SAMPLE = ALL — EAR = BETTER EAR
Age group = 41-50 — Occupational group = Manual — Gender = Male

	Single Frequency (kHz)								Combined Frequency (kHz)					
	0.250	0.500	1	2	3	4	6	8	4FA	3FA	HFA	LFA	A123	A124
**Mean**	**14.2**	**10.9**	**9.3**	**12.2**	**19.9**	**25.8**	**34.1**	**30.6**	**14.5**	**10.8**	**30.2**	**11.5**	**13.8**	**15.8**
**S.D.**	8.1	8.5	7.6	8.3	10.5	11.9	13.2	12.7	7.6	7.4	11.5	7.7	7.9	8.0
**Lower CI**	12.5	8.8	7.5	9.9	17.1	21.6	29.5	26.3	12.6	9.0	26.4	9.8	11.8	13.6
**Upper CI**	15.9	13.0	11.0	14.6	22.6	29.9	38.7	34.9	16.5	12.5	34.0	13.2	15.7	17.9
**P.05**	3.0	-2.0	-1.0	-2.0	5.0	6.0	10.0	7.0	2.0	0.0	11.7	0.7	1.0	2.7
**P.10**	5.0	0.0	0.0	-1.0	6.0	7.0	12.0	9.0	3.8	1.0	12.7	3.0	3.0	3.7
**P.15**	5.0	1.0	0.0	1.0	7.0	10.0	15.0	12.0	5.0	2.3	14.7	3.7	4.3	5.0
**P.20**	6.0	3.0	2.0	3.0	9.0	11.0	20.0	12.0	6.5	3.0	16.3	4.3	5.3	6.0
**P.25**	8.0	5.0	4.0	5.0	11.0	13.0	22.0	14.0	8.2	4.7	17.7	5.7	7.0	7.7
**P.50**	**11.0**	**11.0**	**9.0**	**10.0**	**15.0**	**20.0**	**30.0**	**27.0**	**13.0**	**9.3**	**25.3**	**10.3**	**12.0**	**14.0**
**P.75**	20.0	16.0	15.0	17.0	25.0	37.0	42.0	44.0	19.5	16.0	41.3	17.3	20.0	21.0
**P.80**	21.0	20.0	16.0	19.0	30.0	40.0	50.0	48.0	21.0	17.0	45.3	19.0	20.7	23.7
**P.85**	23.0	21.0	18.0	23.0	36.0	46.0	57.0	55.0	24.2	20.0	47.7	20.0	23.0	28.7
**P.90**	28.0	24.0	20.0	33.0	48.0	52.0	65.0	63.0	29.8	23.7	52.3	23.7	30.3	31.3
**P.95**	38.0	26.0	30.0	43.0	55.0	62.0	77.0	71.0	38.2	32.3	68.3	27.7	38.0	39.7
**N**	**127**	**127**	**127**	**127**	**127**	**127**	**127**	**127**	**127**	**127**	**127**	**127**	**127**	**127**
**Deft**	38.4	45.0	41.2	36.7	41.8	39.8	40.5	37.8	41.3	41.4	40.6	42.3	40.9	39.7
**Bias**	-97.5	-135.3	-103.5	-60.0	-113.7	-58.5	-46.3	-83.5	-89.3	-99.6	-62.8	-112.1	-92.4	-74.0

TABLE 72 SAMPLE = NO NOISE OR ABG>0 — EAR = BETTER EAR
Age group = 41-50 — Occupational group = Manual — Gender = Male

	Single Frequency (kHz)								Combined Frequency (kHz)					
	0.250	0.500	1	2	3	4	6	8	4FA	3FA	HFA	LFA	A123	A124
**Mean**	**11.5**	**6.7**	**4.9**	**6.8**	**12.7**	**15.5**	**27.7**	**29.3**	**8.5**	**6.1**	**24.2**	**7.7**	**8.2**	**9.1**
**S.D.**	9.2	9.2	8.3	7.1	9.6	9.2	10.6	14.6	7.7	7.6	9.8	8.3	7.4	7.4
**Lower CI**	9.1	3.5	2.5	1.7	7.5	10.9	20.4	24.7	5.6	3.3	19.2	6.1	4.8	5.7
**Upper CI**	13.8	9.8	7.4	12.0	17.9	20.1	35.0	33.8	11.4	9.0	29.2	9.3	11.5	12.5
**P.05**	5.0	-1.0	-2.0	-2.0	-2.0	2.0	5.0	12.0	1.8	-1.7	11.7	0.7	-1.0	1.3
**P.10**	5.0	0.0	-1.0	-2.0	2.0	6.0	11.0	12.0	2.5	0.0	12.7	1.0	1.0	2.7
**P.15**	6.0	0.0	-1.0	-1.0	3.0	7.0	12.0	13.0	2.5	1.0	13.0	2.7	1.0	2.7
**P.20**	6.0	1.0	0.0	0.0	7.0	9.0	12.0	13.0	3.2	1.0	13.0	3.7	2.3	3.0
**P.25**	6.0	2.0	0.0	0.0	10.0	10.0	17.0	14.0	3.8	2.3	16.0	3.7	3.0	4.7
**P.50**	**10.0**	**10.0**	**5.0**	**9.0**	**14.0**	**13.0**	**29.0**	**24.0**	**10.0**	**8.3**	**22.3**	**8.0**	**10.0**	**11.0**
**P.75**	11.0	13.0	12.0	11.0	22.0	20.0	35.0	37.0	16.0	11.3	29.3	11.7	13.3	16.3
**P.80**	15.0	15.0	12.0	13.0	23.0	20.0	37.0	37.0	16.0	11.7	29.7	11.7	14.0	17.0
**P.85**	15.0	15.0	15.0	15.0	24.0	21.0	40.0	43.0	16.2	12.7	29.7	11.7	16.0	17.0
**P.90**	16.0	16.0	16.0	15.0	24.0	40.0	46.0	48.0	17.2	16.0	45.0	12.0	20.3	18.0
**P.95**	35.0	20.0	22.0	26.0	52.0	44.0	51.0	68.0	18.8	17.0	47.7	21.7	20.7	20.0
**N**	**22**	**22**	**22**	**22**	**22**	**22**	**22**	**22**	**22**	**22**	**22**	**22**	**22**	**22**
**Deft**	52.2	47.5	40.7	31.5	40.3	35.4	38.7	67.5	38.1	38.1	42.9	44.5	36.9	35.9
**Bias**	-57.4	-280.3	-207.0	-222.3	-360.5	-243.0	-71.0	169.4	-238.2	-236.5	-48.2	-181.5	-263.3	-224.1

KEY:
4FA = Average over 0.5, 1, 2, and 4 kHz — 3FA = Average over 0.5, 1 and 2 kHz
HFA = Average over 4, 6 and 8 kHz — LFA = Average over 0.250, 0.5, and 1 kHz
A123 = Average over 1, 2, and 3 kHz — A124 = Average over 1, 2, and 4 kHz

Mean, standard deviation, confidence intervals, and percentiles for hearing thresholds as a function of ear, age group, socioeconomic group, gender, sample and frequency

TABLE 73 SAMPLE = ALL EAR = BETTER EAR
Age group = 51-60 Occupational group = Overall Gender = Overall

	SINGLE FREQUENCY (kHz)								COMBINED FREQUENCY (kHz)					
	0.250	0.500	1	2	3	4	6	8	4FA	3FA	HFA	LFA	A123	A124
**Mean**	**15.7**	**12.6**	**11.8**	**15.5**	**21.8**	**26.8**	**38.7**	**35.5**	**16.7**	**13.3**	**33.7**	**13.3**	**16.4**	**18.0**
**S.D.**	8.1	8.7	8.4	10.6	13.3	14.8	15.0	14.6	9.5	8.6	14.1	8.0	10.0	10.3
**Lower CI**	14.9	11.7	10.9	14.5	20.6	25.5	37.2	33.8	15.8	12.5	32.3	12.6	15.5	17.1
**Upper CI**	16.5	13.4	12.6	16.6	23.1	28.1	40.2	37.2	17.5	14.1	35.0	14.0	17.3	18.9
**P.05**	3.0	-2.0	-1.0	0.0	0.0	5.0	12.0	7.0	3.5	1.0	10.0	2.0	1.3	3.7
**P.10**	4.0	1.0	0.0	1.0	4.0	6.0	16.0	10.0	5.0	2.7	13.7	3.3	3.3	5.3
**P.15**	6.0	3.0	2.0	3.0	6.0	10.0	19.0	14.0	6.2	3.7	16.0	4.3	5.3	6.7
**P.20**	7.0	5.0	4.0	4.0	8.0	11.0	21.0	16.0	7.8	5.0	18.3	5.7	6.3	8.0
**P.25**	9.0	5.0	4.0	6.0	9.0	13.0	23.0	19.0	8.5	6.0	19.3	6.7	7.3	9.0
**P.50**	**14.0**	**10.0**	**9.0**	**11.0**	**17.0**	**21.0**	**34.0**	**32.0**	**13.0**	**10.0**	**29.0**	**10.7**	**12.7**	**14.0**
**P.75**	20.0	17.0	16.0	21.0	29.0	36.0	51.0	49.0	21.2	17.3	43.3	16.3	21.7	23.3
**P.80**	22.0	19.0	18.0	24.0	33.0	41.0	55.0	55.0	24.0	19.7	47.7	18.7	24.3	26.3
**P.85**	25.0	21.0	20.0	28.0	38.0	46.0	60.0	61.0	27.5	22.0	54.3	21.7	27.3	30.7
**P.90**	29.0	26.0	25.0	35.0	47.0	55.0	68.0	67.0	31.2	27.0	61.0	25.3	33.7	34.7
**P.95**	37.0	36.0	35.0	46.0	59.0	63.0	78.0	76.0	40.5	36.7	71.0	35.0	43.0	45.7
**N**	**680**	**682**	**682**	**682**	**680**	**682**	**680**	**682**	**682**	**682**	**680**	**680**	**680**	**682**
**Deft**	47.4	49.0	48.4	50.7	53.8	56.6	50.2	44.6	55.8	52.3	53.2	51.2	54.0	56.1
**Bias**	-4.0	-4.8	1.8	16.6	28.4	19.5	27.7	11.1	8.3	4.5	19.8	-2.2	15.7	12.6

TABLE 74 SAMPLE = NO NOISE OR ABG>0 EAR = BETTER EAR
Age group = 51-60 Occupational group = Overall Gender = Overall

	SINGLE FREQUENCY (kHz)								COMBINED FREQUENCY (kHz)					
	0.250	0.500	1	2	3	4	6	8	4FA	3FA	HFA	LFA	A123	A124
**Mean**	**13.9**	**10.4**	**10.3**	**13.6**	**19.4**	**24.0**	**36.4**	**34.1**	**14.6**	**11.4**	**31.5**	**11.5**	**14.4**	**16.0**
**S.D.**	6.8	8.9	8.9	11.3	13.3	14.8	15.6	15.6	9.8	9.0	14.5	7.7	10.4	10.7
**Lower CI**	12.6	8.4	8.4	11.2	16.6	21.3	33.6	30.8	12.7	9.5	28.9	10.0	12.3	13.9
**Upper CI**	15.2	12.3	12.1	15.9	22.1	26.7	39.2	37.3	16.4	13.3	34.1	13.0	16.5	18.0
**P.05**	2.0	-3.0	-2.0	-1.0	-1.0	5.0	12.0	6.0	2.2	0.0	9.3	1.3	0.3	2.3
**P.10**	4.0	0.0	0.0	1.0	2.0	5.0	13.0	9.0	4.0	2.3	11.3	3.0	3.0	4.0
**P.15**	5.0	1.0	1.0	2.0	4.0	7.0	17.0	12.0	5.5	3.0	14.0	4.0	3.7	5.3
**P.20**	6.0	3.0	3.0	3.0	6.0	9.0	19.0	14.0	6.2	4.3	15.7	5.0	4.7	6.3
**P.25**	8.0	5.0	4.0	4.0	8.0	10.0	21.0	17.0	7.8	5.0	17.7	5.7	6.3	7.3
[truncated: 2,774,017 more chars]
